# Plasma Proteome-Wide Mendelian Randomization Analysis Reveals Biomarkers and Therapeutic Targets for Different Stages of COVID-19

**DOI:** 10.1155/2024/5566180

**Published:** 2024-02-05

**Authors:** Suhas Krishnamoorthy, Ruby Lai Chong Hoo, Ching Lung Cheung

**Affiliations:** Department of Pharmacology and Pharmacy, Li Ka Shing Faculty of Medicine, The University of Hong Kong, Pokfulam, Hong Kong, China

## Abstract

**Background:**

The COVID-19 pandemic caused by the SARS-CoV-2 virus has resulted in a global health crisis with significant morbidity and mortality. While effective vaccinations have been developed, drug treatments for the disease are still required, particularly for different stages of the disease and to combat evolving variants. Identifying reliable biomarkers and potential therapeutic targets for the different stages of COVID-19 is crucial.

**Methods:**

Mendelian randomization using the largest publicly available datasets was conducted to identify potential causal plasma proteins for severe COVID-19, hospitalized COVID-19, and SARS-CoV-2 infection. Independent, and strongly associated cis- or pan-pQTLs were used as instrumental variables for each protein. The FDR *q*-value was used to correct for multiple testing followed by sensitivity analyses, reverse MR and genetic colocalization to ensure the robustness of the results.

**Results:**

We identified proteins with strong evidence of causal association with different stages of COVID-19. Some of these proteins were identified previously, such as BGAT and BCAT2, but we also identified the novel proteins, such as KLC1, MRVI1, CACO2, and PCNP.

**Conclusion:**

These proteins provide valuable insights into the underlying mechanisms of COVID-19. The identification of these proteins offers new opportunities for developing potential therapeutic targets or biomarkers for the treatment and prevention of COVID-19.

## 1. Introduction

The COVID-19 pandemic caused by the severe acute respiratory syndrome coronavirus 2 (SARS-CoV-2) has resulted in a global health crisis with significant morbidity and mortality. While effective vaccinations have been developed, the requirement for drugs to treat the disease remains, particularly for different stages of the disease and to combat evolving variants that may have increased virulence [1]. Thus, the identification of reliable biomarkers and potential therapeutic targets for different stages of COVID-19 is crucial to combat the evolving nature of the disease.

The plasma proteome represents a rich source of potential biomarkers and therapeutic targets for various diseases [2, 3]. Mendelian randomization (MR) is a powerful approach that leverages genetic variants as instrumental variables to infer causality between an exposure and an outcome. A proteome-wide MR analysis using human genetics and proteomics can identify potential causal relationships between plasma protein levels and disease outcomes in humans [4]. Furthermore, MR analysis can be coupled with genetic colocalization analysis to identify proteins that may share causal variants with the study outcomes, providing further evidence of a shared molecular basis [5]. As such, proteins identified using MR and colocalization may have a high-translational value in serving as potential biomarkers and treatment targets for disease [4, 6–8].

This study aimed to identify novel biomarkers and therapeutic targets for three COVID-19 phenotypes: severe COVID-19, hospitalized COVID-19, and SARS-CoV-2 infection using the plasma proteome-wide MR approach followed by colocalization with the latest and largest datasets available. We hypothesized that using the latest and largest GWAS datasets would provide ample power in delineating the causal relationship between plasma proteome and COVID-19.

## 2. Materials and Methods

### 2.1. GWAS of Plasma Proteins

Summary statistics from the largest plasma proteome GWAS study to date were used in this study [3]. The study included 35,559 Icelanders and measured 4,907 aptamers using the SomaScan version 4 assay (SomaLogic). The study population had a mean age of 55 years and was composed of participants from the Icelandic Cancer Project [9] and various programs at deCODE genetics. Data were downloaded from the deCODE website (https://www.decode.com/), and a subset of variants was excluded due to quality issues as recommended on the website. Aptamers were labeled using Uniprot identifiers and HUGO gene name nomenclature for the gene encoding the relevant protein, which were obtained from the SomaLogic website (https://somalogic.com/somascan-platform/).

### 2.2. GWAS for COVID-19 Phenotypes

For COVID-19, summary statistics for very severe respiratory confirmed COVID-19 (severe COVID-19), hospitalized COVID-19 (hospitalized COVID-19), and COVID-19 infection (SARS-CoV-2 infection) from the COVID-19 Host Genetics Initiatives (release 7) were used [10]. These studies included samples from the European population only, excluding samples from 23andMe.  Table 1 presents the definition of the three GWAS and the related sample size information.

### 2.3. Reference Dataset for Computing Linkage Disequilibrium (LD)

The reference panel of 503 European individuals from the 1,000 genomes project (Phase 3) was used to calculate LD estimates for the analysis [11]. PLINK files were downloaded from the IEU Open GWAS Project API [12, 13], which contains only biallelic autosomal variants with a minor allele frequency (MAF) >0.01. The variants were annotated with dbSNP153 and lifted over to hg38 using the UCSC LiftOver tool [14]. After excluding missing variants and variants on ALT contigs, 99.88% of variants were lifted over.

### 2.4. Selection of Genetic Instruments Associated with Proteins

The summary statistics of each plasma protein were filtered to include only cis-pQTLs that were located ±1 MB from the transcription start site (TSS) of the gene encoding the protein [3]. Gene coordinates were obtained using the Ensembl R package biomaRt [15, 16], using Uniprot ids and HUGO gene names. The gene start position (+strand) or gene end position (−strand) was used as the TSS depending on the gene's strand. Significant cis-pQTLs were then filtered using a threshold of *P*_pQTL_ < 5e-8. To avoid pleiotropic effects, any significant cis-pQTL associated with more than one plasma protein was excluded from the analysis. LD clumping was performed with PLINK 1.90 using a window of 10 Mb and *r*^2^ of 0.001 [8, 17, 18] to include only independent cis-pQTLs for MR analysis.

MR analysis was conducted using genome-wide (pan) pQTLs (i.e., cis-pQTLs and trans-pQTLs) for each protein as the secondary analysis. Similar to the identification of instruments for cis-analysis, significant pan-pQTLs (*P*_pQTL_ < 5e-8) were identified, pleiotropic pan-pQTLs were excluded, and the remaining pan-pQTLs were clumped to include only independent pan-pQTLs for MR analysis.

Overall, this study employed a rigorous filtering and clumping approach to identify the independent cis- and pan-pQTLs as genetic instruments for MR analysis, thereby minimizing the potential bias due to pleiotropic effects.

### 2.5. Statistical Analysis

#### 2.5.1. Mendelian Randomization

This study used MR to identify plasma proteins with a potential causal association with COVID-19 phenotypes. MR is a method to identify causal associations between an exposure and an outcome using only summary statistics from the independent studies. MR uses genetic variants as instrumental variables (IVs) to compute causal associations based on three assumptions. The IVs used should be strongly associated with the exposure, affect the outcome only through the exposure, and not be associated with any confounders. The MR analysis was conducted using the R package TwoSampleMR [13]. For the primary analysis, only cis-pQTLs were used, as these are considered to have a greater biological precedence than trans-pQTLs [8, 19]. Conversely, trans-pQTLs may affect a protein's expression through indirect mechanisms, thereby exhibiting a higher likelihood for pleiotropy, which is more prone to violation of the MR assumptions [8, 20]. However, trans-pQTLs may support causality if they are non-pleiotropic. As our approach for instrument selection included removal of pleiotropic pQTLs, we conducted a secondary analysis using pan-pQTLs in the MR analysis to evaluate the contribution of both cis- and trans-pQTLs.

For each protein (exposure) and COVID-19 phenotype (outcome), exposure and outcome datasets were harmonized using the default parameters of the harmonise_data() function in the TwoSampleMR package. Any genetic instruments from the exposure dataset that were palindromic, misaligned, or missing in the outcome dataset were excluded, and proxies were used in their place where possible. Proxies were defined as significant pQTLs (*P*_pQTL_ < 5e-8), which were in high LD (*r*^2^ > 0.8) with the excluded instrument and were identified from the 1,000 genomes reference dataset using PLINK 1.90.

The primary analysis was performed using the inverse variance weighted (IVW) method [21] or the Wald ratio [22] (in case only one instrumental variable was available), with a false discovery rate (FDR) *q*-value [23] threshold of <0.05 being used for screening potential causal proteins and accounting for the multiple testing. Proteins meeting this criterion were subjected to sensitivity analyses. Multiple sensitivity analyses were adopted to minimize the likelihood of violating the MR assumptions and being false positive findings. IVW assumes all IVs are valid. Conversely, the weighted median test [24], which assumes 50% of instruments are valid, was conducted as a sensitivity analysis. Additionally, the heterogeneity of instruments was estimated [25] using the Cochran Q test, and pleiotropy was evaluated using MR-Egger's intercept [26] and MR Pleiotropy Residual Sum and Outlier (MR-PRESSO) [27]. Leave-one-out analysis (LOO) was used to identify whether the observed association was driven by any one particular IV. Sensitivity analyses were conducted only where possible, as most methods require a minimum of three IVs, while MR-PRESSO requires four and Cochran's Q test requires two. A *P*_Egger−Intercept_ > 0.05, *P*_Q-stat_ > 0.05, *P*_Global Test_ > 0.05 and consistent *P*_LOO_ < 0.05 were required to be considered as statistically significant.

Reverse-direction MR was then conducted to eliminate spurious results that may arise due to reverse causation. Statistically significant results were those with a *P*_IVW_ or *P*_Wald_ < 0.05 and which met the criteria for all sensitivity analyses mentioned above.

For all potential causal proteins, the proportion of variance in the exposure explained by the instruments (*R*^2^) was estimated and used to evaluate the F-statistic, a measure of the strength of association between instruments and a trait. An F-statistic < 10 is indicative of potential weak instrument bias [28].

#### 2.5.2. Colocalization

Genetic colocalization is a method to identify whether two potentially related traits share common causal variants in a particular region and evidence suggests that proteins with both MR and colocalization evidence are likely to be successful drug targets [8]. In the current study, for each of the protein-outcome pairs identified from the MR analysis, genetic colocalization was evaluated using the R package COLOC (V5.1.0.1) [5]. In brief, COLOC utilizes a dense coverage of SNPs in a genomic region to calculate posterior probabilities of five hypotheses under the assumption that there is at most a single causal variant per trait. The hypotheses are: no association with either trait (PPH0), association with either Trait 1 or Trait 2 (PPH1 and PPH2), association with both traits through distinct causal variants (PPH3), and association with both traits through a single causal variant (PPH4). For the colocalization analysis, we used default priors and included all SNPs within a window of ±1 MB from the top cis-pQTL for each protein that were available in both the exposure and outcome GWAS. A high PPH0, PPH1, or PPH2 coupled with a low PPH3 and PPH4 is indicative of limited power in the colocalization analysis, we therefore retained the potential causal proteins from the MR analysis which reached PPH3 + PPH4 > 0.8 [29, 30] while PPH4 > 0.5 was considered evidence of the colocalization.

All analyses were conducted using R version 4.1.0 (https://www.r-project.org/).

## 3. Results

### 3.1. Severe COVID-19

After stringent selection and harmonization of instruments, the causal effects of 1,685 and 2,177 proteins on severe COVID-19 were evaluated in the cis- and pan-analysis, respectively.  Table 2 presents the potential causal plasma proteins for severe COVID-19. The cis-analysis identified the proteins signal transducer and activator of transcription 3 (STAT3) and kinesin light chain 1 (KLC1) as the significant (*q* < 0.05) proteins in the primary analysis. An increase of one standard deviation (SD) in plasma STAT3 and KLC1 was associated with a decreased (odds ratio: 0.584, 95% CI: 0.459–0.743) and increased (odds ratio: 2.016, 95% CI: 1.443–2.816) risk of severe COVID-19, respectively. In the pan-pQTL analysis, the result for STAT3 is the same as the cis-pQTL analysis since no trans-pQTL was identified. Conversely, the association of KLC1 with severe COVID-19 became statistically insignificant after using another IV (Table S1).

Consistent findings were observed in the sensitivity analyses (Table S2). Notably, the reverse-direction MR analysis did not identify any significant causal associations between severe COVID-19 and the identified proteins (Table S3). Both STAT3 and KLC1 were retained after colocalization analysis (Table S4), however only STAT3 colocalized with severe COVID-19.

### 3.2. Hospitalized COVID-19

Following the rigorous selection and harmonization of the instruments, the causal effects of 1,687 and 2,177 proteins on hospitalized COVID-19 were evaluated in the cis- and pan-analysis, respectively (Table S1). Of these, eight and two proteins were significant in the primary analysis (*q* < 0.05) for the cis- and pan-analysis, respectively. Consistent findings were observed in the sensitivity analysis (Table S2), and the reverse-direction MR analysis did not identify any significant causal associations between hospitalized COVID-19 and the identified proteins (Table S3). One protein, myeloperoxidase (MPO) (Table S4), was excluded after colocalization analysis. The potential causal proteins identified by the cis analysis were histo-blood group ABO system transferase (BGAT), vesicle-fusing ATPase (NSF), KLC1, PEST proteolytic signal-containing nuclear protein (PCNP), calcium-binding and coiled-coil domain-containing protein 2 (CACO2), protein MRVI1 and STAT3 (Table 3). In the cis-analysis, the most associated protein was BGAT, with an odds ratio of 1.086 (95% CI: 1.052–1.122) per SD increase. The pan-analysis identified NSF and PCNP as potential causal proteins (Table 3). The most associated protein in the pan-analysis was NSF, with an odds ratio of 0.541 (95% CI: 0.406–0.721) per SD increase. BGAT, KLC1, STAT3, and MRVI1 showed evidence of colocalization (Table S4).

### 3.3. SARS-CoV-2 Infection

The causal effects of 1,693 and 2,186 proteins on SARS-CoV-2 infection were evaluated in the cis- and pan-analysis, respectively. The cis-analysis identified BGAT, PCNP, and branched-chain-amino-acid aminotransferase, mitochondrial (BCAT2) as significant (*q* < 0.05) in the primary analysis. However, the association of BGAT was not consistent in the sensitivity analysis, as the instruments were found to be heterogenous by Cochrane's Q test (Table S2).  Table 4 presents the potential causal plasma proteins for SARS-CoV-2 infection. An increase of one standard deviation (SD) in plasma PCNP and BCAT2 was associated with a decreased (odds ratio: 0.683, 95% CI: 0.612–0.763) and increased (odds ratio: 1.151, 95% CI: 1.074–1.234) risk of SARS-CoV-2 infection, respectively. The pan-analysis identified PCNP as a potential causal protein, with the same result as the cis-analysis, as no trans-pQTLs were identified and the same IV was used in the analysis. The reverse-direction MR analysis did not identify any significant causal associations between SARS-CoV-2 infection and the identified proteins (Table S3). Colocalization analysis for both potential causal proteins yielded a PPH3 = 1 (Table S4).

### 3.4. Strength of Genetic Instruments

Table S5 provides details of the pQTLs used for MR analysis and the F-statistics for the identified potential causal proteins. All F-statistics were greater than 10 (at least 100), indicating that the instruments were strongly associated with the exposures, which minimizes the risk of weak instrument bias [28].

## 4. Discussion

Using mendelian randomization with cis-pQTLs and pan-pQTLs for plasma proteins, as well as genetic colocalization, we identified proteins with strong evidence of causal association with three COVID-19 phenotypes; severe COVID-19, hospitalized COVID-19, and SARS-CoV-2 infection. These findings improve our understanding of the mechanism underlying COVID-19 and present protein candidates for use as therapeutic targets or biomarkers for treating and preventing COVID-19.

Previous MR studies have also evaluated the effects of plasma proteins on COVID-19 phenotypes [31–35] and identified several causal candidates for the different COVID-19 phenotypes. Compared to the previous studies, our study utilized the most recent release of the HGI COVID-19 GWAS and the largest GWAS of plasma proteins to date. This allowed us to employ more stringent criteria in our instrument selection to avoid false positive findings, including a *P*-value threshold of < 5e-8 for instrument selection, a highly stringent threshold of *r*^2^ < 0.001, and a window of 10 Mb around each instrument to identify independent instruments, and the exclusion of any pQTLs significantly associated with more than one protein from the study to minimize the potential for pleiotropy. As such, we could not evaluate some known associated proteins such as OAS1, ICAM3, GCNT, CD207, FAAH2, ATP2A3, and KEL [31, 32]. Other previously found causal proteins FCRL3, SELE, SELL, ICAM5, ICAM1, C1GALT1C1, CD209, FAM3D, ENTPD5, SFTPD, TIE1, and ADGRF5 [31–33] were evaluated but were not significantly associated (*q*-value < 0.05) with any of the outcomes in our study (Table S6), potentially due to our stringent criteria limiting the number of instruments used in the analysis. Two previously identified proteins were identified in this study as well. BGAT [32–34] is encoded by the *ABO* gene and referred to as the ABO protein in many studies. In the current study, BGAT was found to increase the risk of hospitalized COVID-19. Previous studies identified similar associations in earlier releases of HGI data, including hospitalized COVID-19 [32–34], severe COVID-19 [32, 33], self-reported COVID-19 [32], and SARS-CoV-2 infection [32]. However, in this study, the association with severe COVID-19 did not pass the primary analysis threshold, and the association with SARS-CoV-2 infection did not pass the criteria for sensitivity analyses. Nonetheless, these results further show that BGAT is an important protein for hospitalized COVID-19. We also found that increased BCAT2 may be causally associated with an increased risk of SARS-CoV-2 infection, which aligns with a previous study [36].

The remaining proteins identified were found for the first-time using proteome-wide MR. However, some are already known to be associated with the disease. We found plasma levels of STAT3 to be associated with reduced risk of severe and hospitalized COVID-19. STAT3 is a transcription factor having a complex involvement with COVID-19, such as inducing inflammatory responses and suppressing antiviral responses [37, 38]. We reported that an increased plasma KLC1 level was associated with an increased risk of severe and hospitalized COVID-19. Kinesin-1 is comprised, in part, of KLC1, a molecular motor protein known to play a role in the spread of many viruses [39] and may have a role to play COVID-19's hijacking of the cytoskeleton [40]. NSF was identified as a potential causal protein decreasing the risk of hospitalized COVID-19. A previously conducted transcriptome-wide association study found that the expression of the gene *NSF* in blood was also associated with a decreased risk of hospitalized COVID-19 [41], which is consistent with our finding. Although, little is available about the relationship of NSF and COVID-19 in literature, one possible mechanism could be through *α*-SNAP, which reduces COVID-19 infection in cells and is an adaptor to NSF [42, 43]. MRVI1 (also IRAG1) and PCNP were identified as potential causal proteins affecting risk of COVID-19 phenotypes in this study for the first time. However, their role in infection is unclear. CACO2 is a novel potential causal protein associated with decreased risk of hospitalized COVID-19 involved in vesicle tethering [44]. The protein is also an autophagy receptor and may suppress the type 1 interferon response and increase virophagy [45].

In several cases, the results of the cis- and pan-analyses are identical. This is due to the fact that after expanding our analysis to include trans-pQTLs, most of the QTLs identified were significantly associated with more than one protein and were therefore considered pleiotropic and excluded, resulting in the same IVs being used in the cis- and pan- analyses. Conversely, several proteins exhibited discordant results between the cis- and pan-analyses, despite the absence of any additional trans-pQTLs detected as IVs. This discrepancy is due to the removal of pleiotropic pQTLs during instrument selection. Specifically, in the cis-analysis, we excluded any significant cis-pQTLs that were associated with more than one protein, whereas, in the pan-analysis, we excluded any significant pan-pQTLs that were associated with more than one protein. Consequently, the pQTL employed in the pan-analysis may have differed from those utilized in the cis-analysis, despite both being situated within the cis-region of the protein. For example, the protein KLC1 was significantly associated with hospitalized and severe COVID-19 in the cis-analysis, with rs12884809 being the IV. Rs12884809 is a cis-pQTL for only one protein, KLC1. However, in the pan-analysis, rs12884809 was excluded as it is also a pan-pQTL for another protein, Ankyrin Repeat, and SOCS Box protein 9. Therefore, rs55696130, the most associated pan-pQTL for KLC1 exclusively, was used as the IV for KLC1 in the pan-analysis, which explained the discordant results.

The colocalization analysis supported the association between all potential causal protein-outcome pairs identified in the MR analysis except for MPO with hospitalized COVID-19 which was subsequently excluded. However, colocalization, defined as association with both traits through a single causal variant (PPH4 > 0.5) was only observed in 5 out of the 11 pairs: BGAT, KLC1, and MRVI1 with hospitalized COVID-19 and STAT3 with severe and hospitalized COVID-19, providing strong evidence of their causal role in COVID-19 progression. For the remaining, the colocalization analysis found a significant association, but through distinct causal variants. This may be due to the variants being in high LD or due to the strict single causal variant assumption of COLOC. While colocalization aims to identify whether a single variant is associated with two traits, MR focuses on whether the protein, but not a particular genetic variant, is associated with the outcome, therefore cautious interpretation is required.

Our study's results have significant clinical implications, particularly in the context of COVID-19 management. COVID-19 has been proposed to be transitioning toward an endemic stage [46–49], indicating that it will continue to exist in the community, much like the flu, necessitating the development of effective management strategies to tackle the disease. Our findings identified potential novel biomarkers that could stratify the risk of severe and hospitalized patients and further identified proteins that are potential therapeutic targets and agents.

Our study's strengths include the utilization of the largest sample size of COVID-19 HGI data and the largest GWAS of plasma proteins to date, as well as the exclusion of pleiotropic pQTLs from the analysis to minimize the potential for confounding. We also used several sensitivity analyses, reverse MR and colocalization for robust evaluation. Furthermore, our cis- and pan-analyses allowed us to evaluate a larger number of proteins and systematically identify potential causal proteins. Nonetheless, our study has several limitations, including the potential for being confounded by the risk factors of COVID-19 and the possibility that our results may be specific to the European population. The stringent criteria employed in our study resulted in several candidate causal proteins having two or fewer instrumental variables, which may reduce the false positive rate but increase the false negative rate. Moreover, further research is necessary to validate the causal role of these proteins.

## 5. Conclusion

In conclusion, our study identified proteins with evidence of causal association with different stages of COVID-19, including novel proteins such as KLC1, MRVI1, CACO2, and PCNP. These proteins offer new insights into the underlying mechanisms of COVID-19 and may serve as potential therapeutic targets or biomarkers for treating and preventing the disease.

## Figures and Tables

**Table 1:** Phenotype definition for the COVID-19 outcomes.

Phenotype	No. of cases	No. of controls	Description
Very severe confirmed respiratory COVID-19 (severe COVID-19)	13,769	1,072,442	Hospitalized laboratory confirmed SARS-CoV-2 infection (RNA and/or serology based), AND (death OR respiratory support (intubation, CPAP, BiPAP, CNP (continue external negative pressure), Optiflow/very high flow positive end expiratory pressure oxygen^*∗*^—AND hospitalization with COVID19 as primary reason for admission

Hospitalized COVID-19	32,519	2,062,805	Hospitalized laboratory confirmed SARS-CoV-2 infection (RNA and/or serology based) AND hospitalization due to COVID-19-related symptoms

SARS-CoV-2 infection	122,616	2,475,240	Individuals with laboratory confirmation of SARS-CoV-2 infection (RNA and/or serology based) OR I/ICD coding/physician confirmed COVID-19 OR self-reported COVID-19 positive (e.g., by questionnaire)

**Table 2:** Potential causal proteins for severe COVID-19 identified in the cis- and pan-analyses.

Protein	Odds ratio	95% CI	*P* value	Cis/pan
STAT3	0.584	0.459–0.743	1.232e-05	Cis
KLC1	2.016	1.443–2.816	3.910e-05	Cis
STAT3	0.584	0.459–0.743	1.232e-05	Pan

**Table 3:** Potential causal proteins for hospitalized COVID-19 identified in the cis- and pan-analyses.

Protein	Odds ratio	95% CI	*P* value	Cis/pan
BGAT	1.086	1.052–1.122	5.147e-07	Cis
NSF	0.545	0.416–0.714	1.107e-05	Cis
KLC1	1.635	1.303–2.052	2.148e-05	Cis
Myeloperoxidase	0.881	0.830–0.935	2.712e-05	Cis
PCNP	0.620	0.493–0.779	4.237e-05	Cis
CACO2	0.637	0.507–0.800	1.041e-04	Cis
STAT3	0.738	0.631–0.864	1.497e-04	Cis
MRVI1	0.727	0.616–0.858	1.678e-04	Cis
NSF	0.541	0.406–0.721	2.642e-05	Pan
PCNP	0.620	0.493–0.779	4.237e-05	Pan

**Table 4:** Potential causal proteins for SARS-CoV-2 infection identified in the cis- and pan-analyses.

Protein	Odds ratio	95% CI	*P* value	Cis/pan
PCNP	0.683	0.612–0.763	1.221e-11	Cis
BCAT2	1.151	1.074–1.234	7.358e-05	Cis
PCNP	0.683	0.612–0.763	1.221e-11	Pan

## Data Availability

All data used in the study are freely available as stated in the manuscript. Data generated during the current study are available in the tables and supplementary tables of the manuscript. Supplementary data in alternative formats are available upon request.

## Supplementary Materials

Supplementary Materials Table S1: results of the primary analysis. Table S2: results of the sensitivity analysis. Table S3: results of the reverse-direction MR analysis. Table S4: results of the genetic colocalization analysis. Table S5: instrumental variables used for analysis after harmonization and proxies. Table S6: results of the primary analysis for previously identified protein.
