# Supplementary material for: Plasma Proteome-Wide Mendelian Randomization Analysis Reveals Biomarkers and Therapeutic Targets for Different Stages of COVID-19
Source: Transbound Emerg Dis. 2024 Feb 5;2024:5566180. doi: 10.1155/2024/5566180 (PMC12017221; doi:10.1155/2024/5566180)
Supplement: Supplementary Materials — Table S1: results of the primary analysis. Table S2: results of the sensitivity analysis. Table S3: results of the reverse-direction MR analysis. Table S4: results of the genetic colocalization analysis. Table S5: instrumental variables used for analysis after harmonization and proxies. Table S6: results of the primary analysis for previously identified protein. [file 5566180.f1.pdf]

Supplementary Table S1: Results of the primary analysis.

SomaID	Protein_short	Protein_full	UniProtID	GeneID	TSS	Method	nsnp	Beta	SE	Pval	Qval	cis/pan	Outcome
10346_5	STAT3	Signal transducer and activator of transcription 3	P40763	STAT3	chr17:42388540	Inverse variance weighted	2	-0.538	0.123	1.232E-05	0.021	cis	Severe COVID-19
12656_1	KLC1	Kinesin light chain 1	Q07866	KLC1	chr14:103561896	Wald ratio	1	0.701	0.170	3.910E-05	0.033	cis	Severe COVID-19
2580_83	Myeloperoxidase	Myeloperoxidase	P05164	MPO	chr17:58280935	Inverse variance weighted	7	-0.192	0.050	1.065E-04	0.060	cis	Severe COVID-19
4125_52	sRAGE	Advanced glycosylation end product-specific receptor, soluble	Q15109	AGER	chr6:32184322	Inverse variance weighted	2	0.227	0.068	8.277E-04	0.287	cis	Severe COVID-19
15607_56	KPYR	Pyruvate kinase PKLR	P30613	PKLR	chr1:155301438	Wald ratio	1	-0.421	0.126	8.557E-04	0.287	cis	Severe COVID-19
9253_52	BGAT	Histo-blood group ABO system transferase	P16442	ABO	chr9:133276024	Inverse variance weighted	12	0.077	0.023	1.023E-03	0.287	cis	Severe COVID-19
17769_28	PCNP	PEST proteolytic signal-containing nuclear protein	Q8WW12	PCNP	chr3:101574180	Wald ratio	1	-0.549	0.170	1.255E-03	0.302	cis	Severe COVID-19
12534_10	CACO2	Calcium-binding and coiled-coil domain-containing protein 2	Q13137	CALCOCO2	chr17:48831018	Wald ratio	1	-0.541	0.170	1.455E-03	0.306	cis	Severe COVID-19
8255_34	MRVI1	Protein MRVI1	Q9Y6F6	IRAG1	chr11:10693988	Inverse variance weighted	2	-0.380	0.124	2.121E-03	0.311	cis	Severe COVID-19
6404_20	C1QRF	C1q-related factor	O75973	C1QL1	chr17:44968303	Inverse variance weighted	3	-0.193	0.063	2.138E-03	0.311	cis	Severe COVID-19
8923_94	GLTL1	Polypeptide N-acetylgalactosaminyltransferase 16	Q8N428	GALNT16	chr14:69259277	Inverse variance weighted	4	-0.231	0.075	2.151E-03	0.311	cis	Severe COVID-19
6955_68	SNX1	Sorting nexin-1	Q13596	SNX1	chr15:64094123	Wald ratio	1	-0.348	0.114	2.214E-03	0.311	cis	Severe COVID-19
15653_9	COAA1	Collagen alpha-1(X) chain	Q03692	COL10A1	chr6:116158747	Inverse variance weighted	3	-0.221	0.073	2.492E-03	0.320	cis	Severe COVID-19
8427_118	RSPO3	R-spondin-3	Q9BXY4	RSPO3	chr6:127118671	Inverse variance weighted	4	-0.544	0.181	2.655E-03	0.320	cis	Severe COVID-19
12387_7	PDLI4	PDZ and LIM domain protein 4	P50479	PDLIM4	chr5:132257696	Wald ratio	1	-0.280	0.094	2.861E-03	0.321	cis	Severe COVID-19
9834_62	ADH1B	Alcohol dehydrogenase 1B	P00325	ADH1B	chr4:99352760	Wald ratio	1	0.395	0.135	3.365E-03	0.354	cis	Severe COVID-19
19446_1	GMPR2	GMP reductase 2	Q9P2T1	GMPR2	chr14:24232422	Inverse variance weighted	2	0.195	0.069	4.549E-03	0.439	cis	Severe COVID-19
10440_26	ACAM:ECD	CXADR-like membrane protein:Extracellular domain	Q9H6B4	CLMP	chr11:123195248	Inverse variance weighted	3	0.201	0.071	4.689E-03	0.439	cis	Severe COVID-19
3852_19	HSP 40	DnaJ homolog subfamily B member 1	P25685	DNAJB1	chr19:14560391	Wald ratio	1	1.175	0.421	5.236E-03	0.454	cis	Severe COVID-19
8464_31	RSPO4	R-spondin-4	Q2I0M5	RSPO4	chr20:1002311	Inverse variance weighted	2	0.416	0.150	5.445E-03	0.454	cis	Severe COVID-19
13434_172	PARVA	Alpha-parvin	Q9NVD7	PARVA	chr11:12377563	Wald ratio	1	-0.475	0.172	5.659E-03	0.454	cis	Severe COVID-19
9380_2	sPLA(2)-XIII	Group XIIB secretory phospholipase A2-like protein	Q9BX93	PLA2G12B	chr10:72954806	Wald ratio	1	-0.477	0.176	6.721E-03	0.515	cis	Severe COVID-19
3866_7	SBDS	Ribosome maturation protein SBDS	Q9Y3A5	SBDS	chr7:66995693	Wald ratio	1	0.318	0.119	7.396E-03	0.517	cis	Severe COVID-19
2567_5	Factor I	Complement factor I	P05156	CFI	chr4:109802150	Inverse variance weighted	2	0.217	0.081	7.635E-03	0.517	cis	Severe COVID-19
6930_95	SIA8F	Alpha-2,8-sialyltransferase 8F	P61647	ST8SIA6	chr10:17454595	Wald ratio	1	-0.808	0.303	7.670E-03	0.517	cis	Severe COVID-19
12494_99	GBRL2	Gamma-aminobutyric acid receptor-associated protein-like 2	P60520	GABARAPL2	chr16:75566375	Wald ratio	1	-0.517	0.196	8.239E-03	0.534	cis	Severe COVID-19
13943_38	DPY30	Protein dpy-30 homolog	Q9C005	DPY30	chr2:32039805	Wald ratio	1	-0.578	0.221	9.066E-03	0.554	cis	Severe COVID-19
10722_13	KSYK:Protein Kinase	Tyrosine-protein kinase SYK:Protein kinase domain	P43405	SYK	chr9:90801787	Wald ratio	1	0.751	0.288	9.213E-03	0.554	cis	Severe COVID-19
15339_32	COF2	Cofilin-2	Q9Y281	CFL2	chr14:34714823	Wald ratio	1	0.328	0.127	1.013E-02	0.575	cis	Severe COVID-19
8768_4	Bcl-10	B-cell lymphoma/leukemia 10	O95999	BCL10	chr1:85276632	Wald ratio	1	1.040	0.406	1.042E-02	0.575	cis	Severe COVID-19
9359_9	EGFL9	Protein delta homolog 2	Q6UY11	DLK2	chr6:43456632	Wald ratio	1	-0.224	0.087	1.058E-02	0.575	cis	Severe COVID-19
8100_15	ADM2	ADM2	Q7Z4H4	ADM2	chr22:50481543	Wald ratio	1	0.981	0.389	1.177E-02	0.588	cis	Severe COVID-19
14636_25	Ribonuclease UK114	Ribonuclease UK114	P52758	RIDA	chr8:98117171	Inverse variance weighted	3	-0.138	0.055	1.194E-02	0.588	cis	Severe COVID-19
5128_53	SLAF6	SLAM family member 6	Q96DU3	SLAMF6	chr1:160523262	Wald ratio	1	-0.213	0.085	1.202E-02	0.588	cis	Severe COVID-19
4541_49	CDON	Cell adhesion molecule-related/down-regulated by oncogenes	Q4KMG0	CDON	chr11:126063335	Inverse variance weighted	4	0.116	0.046	1.250E-02	0.588	cis	Severe COVID-19
8759_29	a1,4-Galactosyltransferase	Lactosylceramide 4-alpha-galactosyltransferase	Q9NPC4	A4GALT	chr22:42721298	Inverse variance weighted	4	0.107	0.043	1.270E-02	0.588	cis	Severe COVID-19
4188_1	Aflatoxin B1 aldehyde reductase	Aflatoxin B1 aldehyde reductase member 2	O43488	AKR7A2	chr1:19312144	Wald ratio	1	0.246	0.099	1.316E-02	0.588	cis	Severe COVID-19
19617_5	LTB4DH	Prostaglandin reductase 1	Q14914	PTGR1	chr9:111599893	Inverse variance weighted	8	-0.070	0.028	1.367E-02	0.588	cis	Severe COVID-19
3396_54	Renin	Renin	P00797	REN	chr1:204190324	Inverse variance weighted	3	-0.279	0.113	1.384E-02	0.588	cis	Severe COVID-19
13381_49	B4GT1	Beta-1,4-galactosyltransferase 1	P15291	B4GALT1	chr9:33167356	Inverse variance weighted	2	-0.190	0.078	1.442E-02	0.588	cis	Severe COVID-19
18435_40	UBX2B	UBX domain-containing protein 2B	Q14CS0	UBXN2B	chr8:58411359	Wald ratio	1	-0.610	0.251	1.501E-02	0.588	cis	Severe COVID-19
2731_29	NADPH-P450 Oxidoreductase	NADPH--cytochrome P450 reductase	P16435	POR	chr7:75899200	Inverse variance weighted	2	-0.306	0.126	1.530E-02	0.588	cis	Severe COVID-19
5900_11	HINT1	Histidine triad nucleotide-binding protein 1	P49773	HINT1	chr5:131224468	Wald ratio	1	0.436	0.180	1.531E-02	0.588	cis	Severe COVID-19
16918_198	TLR3	Toll-like receptor 3	O15455	TLR3	chr4:186068911	Inverse variance weighted	8	0.093	0.039	1.604E-02	0.588	cis	Severe COVID-19
19279_42	CRBP	Retinol-binding protein 1	P09455	RBP1	chr3:139539829	Wald ratio	1	0.554	0.232	1.682E-02	0.588	cis	Severe COVID-19
2658_27	TrkC	NT-3 growth factor receptor	Q16288	NTRK3	chr15:88256791	Inverse variance weighted	5	-0.153	0.064	1.688E-02	0.588	cis	Severe COVID-19
19383_131	CINP	Cyclin-dependent kinase 2-interacting protein	Q9BW66	CINP	chr14:102362916	Wald ratio	1	0.666	0.279	1.707E-02	0.588	cis	Severe COVID-19
18381_16	ALDH-E2	Aldehyde dehydrogenase, mitochondrial	P05091	ALDH2	chr12:111766887	Wald ratio	1	-0.461	0.195	1.779E-02	0.588	cis	Severe COVID-19
4249_64	NDP kinase B	Nucleoside diphosphate kinase B	P22392	NME2	chr17:51165435	Wald ratio	1	0.315	0.133	1.783E-02	0.588	cis	Severe COVID-19
8786_6	F171B:ECD	Protein FAM171B:Extracellular domain	Q6P995	FAM171B	chr2:186694060	Wald ratio	1	0.231	0.097	1.786E-02	0.588	cis	Severe COVID-19
6227_1	kallikrein 10	Kallikrein-10	O43240	KLK10	chr19:51020175	Inverse variance weighted	6	0.153	0.065	1.801E-02	0.588	cis	Severe COVID-19
4192_10	AK1A1	Alcohol dehydrogenase [NADP(+)]	P14550	AKR1A1	chr1:45550543	Inverse variance weighted	6	0.101	0.043	1.851E-02	0.588	cis	Severe COVID-19
3059_50	BAFF	Tumor necrosis factor ligand superfamily member 13B	Q9Y275	TNFSF13B	chr13:108251240	Inverse variance weighted	3	-0.388	0.165	1.884E-02	0.588	cis	Severe COVID-19
6388_21	CC126	Coiled-coil domain-containing protein 126	Q96EE4	CCDC126	chr7:23597382	Inverse variance weighted	4	0.119	0.051	1.891E-02	0.588	cis	Severe COVID-19
8288_27	b2-Glycoprotein I	Beta-2-glycoprotein 1	P02749	APOH	chr17:66256525	Inverse variance weighted	3	0.060	0.026	1.920E-02	0.588	cis	Severe COVID-19
4342_10	sICAM-1	Intercellular adhesion molecule 1	P05362	ICAM1	chr19:10271093	Inverse variance weighted	9	0.065	0.028	2.009E-02	0.604	cis	Severe COVID-19
15583_18	FCRLB	Fc receptor-like B	Q6BAA4	FCRLB	chr1:161721544	Inverse variance weighted	5	0.245	0.107	2.190E-02	0.646	cis	Severe COVID-19
6927_7	NDST1	Bifunctional heparan sulfate N-deacetylase/N-sulfotransferase 1	P52848	NDST1	chr5:150485818	Wald ratio	1	0.244	0.107	2.223E-02	0.646	cis	Severe COVID-19
16792_4	Siglec-5	Sialic acid-binding Ig-like lectin 5	O15389	SIGLEC5	chr19:51630401	Inverse variance weighted	5	0.100	0.044	2.289E-02	0.651	cis	Severe COVID-19
16892_23	ENPP2	Ectonucleotide pyrophosphatase/phosphodiesterase family member 2	Q13822	ENPP2	chr8:119673453	Inverse variance weighted	4	0.272	0.120	2.328E-02	0.651	cis	Severe COVID-19
3378_49	Kallikrein 7	Kallikrein-7	P49862	KLK7	chr19:50984099	Inverse variance weighted	2	-0.238	0.105	2.358E-02	0.651	cis	Severe COVID-19
12395_86	SYDM	Aspartate--tRNA ligase, mitochondrial	Q6PI48	DARS2	chr1:173824653	Wald ratio	1	-0.349	0.155	2.445E-02	0.665	cis	Severe COVID-19
5722_78	Prolylcarboxypeptidase	Lysosomal Pro-X carboxypeptidase	P42785	PRCP	chr11:82970584	Inverse variance weighted	3	0.233	0.104	2.494E-02	0.667	cis	Severe COVID-19
3421_54	CD30 Ligand	Tumor necrosis factor ligand superfamily member 8	P32971	TNFSF8	chr9:114930595	Inverse variance weighted	2	-0.218	0.097	2.543E-02	0.669	cis	Severe COVID-19
8356_88	NEU1	Oxytocin-neurophysin 1	P01178	OXT	chr20:3071620	Inverse variance weighted	3	0.158	0.071	2.643E-02	0.683	cis	Severe COVID-19
12428_2	LYPL1	Lysophospholipase-like protein 1	Q5VWZ2	LYPLAL1	chr1:219173869	Wald ratio	1	0.325	0.147	2.676E-02	0.683	cis	Severe COVID-19
7161_25	G6PE	GDH/6PGL endoplasmic bifunctional protein	O95479	H6PD	chr1:9234774	Inverse variance weighted	9	-0.091	0.042	2.843E-02	0.688	cis	Severe COVID-19
2637_77	Macrophage mannose receptor	Macrophage mannose receptor 1	P22897	MRC1	chr10:17809348	Inverse variance weighted	8	0.091	0.042	2.873E-02	0.688	cis	Severe COVID-19
6461_54	Apo C-III	Apolipoprotein C-III	P02656	APOC3	chr11:116829706	Inverse variance weighted	2	-0.487	0.224	2.948E-02	0.688	cis	Severe COVID-19
9962_1	MUCDL	Cadherin-related family member 5	Q9HBB8	CDHR5	chr11:626078	Wald ratio	1	-0.579	0.266	2.949E-02	0.688	cis	Severe COVID-19
12424_107	THYN1	Thymocyte nuclear protein 1	Q9P016	THYN1	chr11:134253370	Wald ratio	1	0.653	0.301	2.987E-02	0.688	cis	Severe COVID-19
2864_2	MEK1	Dual specificity mitogen-activated protein kinase kinase 1	Q02750	MAP2K1	chr15:66386837	Wald ratio	1	0.672	0.310	3.007E-02	0.688	cis	Severe COVID-19
3009_3	TGF-b R III	Transforming growth factor beta receptor type 3	Q03167	TGFBR3	chr1:91906335	Inverse variance weighted	2	-0.310	0.143	3.051E-02	0.688	cis	Severe COVID-19
14708_59	CO8G	Complement component C8 gamma chain	P07360	C8G	chr9:136945185	Inverse variance weighted	5	0.183	0.085	3.136E-02	0.688	cis	Severe COVID-19
16621_77	AIBP	NAD(P)H-hydrate epimerase	Q8NCW5	NAXE	chr1:156591756	Wald ratio	1	0.146	0.068	3.265E-02	0.688	cis	Severe COVID-19
7923_41	SEM4C	Semaphorin-4C	Q9C0C4	SEMA4C	chr2:96870757	Wald ratio	1	0.260	0.123	3.414E-02	0.688	cis	Severe COVID-19

16596_25	GLRX3	Glutaredoxin-3	O76003	GLRX3	chr10:130136391	Wald ratio	1	-0.388	0.184	3.516E-02	0.688	cis	Severe COVID-19
16605_2	C1T9A	Complement C1q and tumor necrosis factor-related protein 9A	P0C862	C1QTNF9	chr13:24307166	Wald ratio	1	0.209	0.099	3.560E-02	0.688	cis	Severe COVID-19
11547_84	MUSK	Muscle, skeletal receptor tyrosine-protein kinase	O15146	MUSK	chr9:110668779	Wald ratio	1	0.648	0.309	3.621E-02	0.688	cis	Severe COVID-19
6207_10	prosaposin	Prosaposin	P07602	PSAP	chr10:71851251	Inverse variance weighted	3	0.099	0.048	3.703E-02	0.688	cis	Severe COVID-19
9484_75	Desmoglein-2	Desmoglein-2	Q14126	DSG2	chr18:31498177	Inverse variance weighted	5	0.219	0.105	3.726E-02	0.688	cis	Severe COVID-19
15491_20	CD248	Endosialin	Q9HCU0	CD248	chr11:66317044	Inverse variance weighted	2	0.741	0.356	3.731E-02	0.688	cis	Severe COVID-19
13438_115	CHRD	Chordin	Q9H2X0	CHRD	chr3:184380054	Wald ratio	1	-0.429	0.206	3.746E-02	0.688	cis	Severe COVID-19
15603_20	Integrin alpha-2	Integrin alpha-2	P17301	ITGA2	chr5:52989340	Wald ratio	1	0.341	0.164	3.757E-02	0.688	cis	Severe COVID-19
12432_23	CYBP	Calcyclin-binding protein	Q9HB71	CACYBP	chr1:174999163	Wald ratio	1	-0.228	0.110	3.774E-02	0.688	cis	Severe COVID-19
13973_62	TTL	Tubulin--tyrosine ligase	Q8NG68	TTL	chr2:112482156	Wald ratio	1	0.554	0.267	3.782E-02	0.688	cis	Severe COVID-19
2972_57	BMP-7	Bone morphogenetic protein 7	P18075	BMP7	chr20:57266641	Wald ratio	1	0.534	0.257	3.806E-02	0.688	cis	Severe COVID-19
11102_22	REG4	Regenerating islet-derived protein 4	Q9BYZ8	REG4	chr1:119811580	Inverse variance weighted	5	-0.204	0.099	3.881E-02	0.688	cis	Severe COVID-19
13666_222	Carbonic Anhydrase X	Carbonic anhydrase-related protein 10	Q9NS85	CA10	chr17:52160017	Inverse variance weighted	4	-0.117	0.057	4.050E-02	0.688	cis	Severe COVID-19
16049_43	OLR1	Oxidized low-density lipoprotein receptor 1	P78380	OLR1	chr12:10172138	Wald ratio	1	0.524	0.256	4.091E-02	0.688	cis	Severe COVID-19
2631_50	IL-10 Rb	Interleukin-10 receptor subunit beta	Q08334	IL10RB	chr21:33266367	Wald ratio	1	0.602	0.295	4.108E-02	0.688	cis	Severe COVID-19
6925_26	SNX8	Sorting nexin-8	Q9Y5X2	SNX8	chr7:2354318	Inverse variance weighted	2	0.182	0.089	4.147E-02	0.688	cis	Severe COVID-19
11568_2	FKB1B	Peptidyl-prolyl cis-trans isomerase FKBP1B	P68106	FKBP1B	chr2:24049701	Wald ratio	1	0.273	0.134	4.163E-02	0.688	cis	Severe COVID-19
8297_8	DJC10	DnaJ homolog subfamily C member 10	Q8IXB1	DNAJC10	chr2:182716255	Inverse variance weighted	2	-0.298	0.146	4.169E-02	0.688	cis	Severe COVID-19
8989_40	SCUB1	Signal peptide, CUB and EGF-like domain-containing protein 1	Q8IWY4	SCUBE1	chr22:43343372	Inverse variance weighted	4	-0.295	0.145	4.176E-02	0.688	cis	Severe COVID-19
2741_22	Siglec-6	Sialic acid-binding Ig-like lectin 6	O43699	SIGLEC6	chr19:51531856	Inverse variance weighted	6	-0.110	0.054	4.201E-02	0.688	cis	Severe COVID-19
12558_3	UBS3B	Ubiquitin-associated and SH3 domain-containing protein B	Q8TF42	UBASH3B	chr11:122655722	Inverse variance weighted	2	-0.224	0.111	4.305E-02	0.688	cis	Severe COVID-19
11387_3	ATF6B	Cyclic AMP-dependent transcription factor ATF-6 beta	Q99941	ATF6B	chr6:32128253	Inverse variance weighted	3	-0.275	0.136	4.324E-02	0.688	cis	Severe COVID-19
15582_25	FCN1	Ficolin-1	O00602	FCN1	chr9:134917912	Inverse variance weighted	5	0.106	0.053	4.332E-02	0.688	cis	Severe COVID-19
3316_58	Heparin cofactor II	Heparin cofactor 2	P05546	SERPIND1	chr22:20774113	Wald ratio	1	0.598	0.296	4.360E-02	0.688	cis	Severe COVID-19
9263_57	SIAE	Sialate O-acetylesterase	Q9HAT2	SIAE	chr11:124695707	Wald ratio	1	0.335	0.166	4.406E-02	0.688	cis	Severe COVID-19
13590_1	ORN	Oligoribonuclease, mitochondrial	Q9Y3B8	REXO2	chr11:114439435	Wald ratio	1	-0.257	0.128	4.412E-02	0.688	cis	Severe COVID-19
2844_53	sTie-1	Tyrosine-protein kinase receptor Tie-1, soluble	P35590	TIE1	chr1:43300982	Inverse variance weighted	4	0.133	0.066	4.445E-02	0.688	cis	Severe COVID-19
3325_2	MATN2	Matrilin-2	O00339	MATN2	chr8:97868840	Inverse variance weighted	4	0.222	0.111	4.480E-02	0.688	cis	Severe COVID-19
5744_12	CA056	Protein MENT	Q9BUN1	MENT	chr1:151047751	Inverse variance weighted	2	-0.396	0.198	4.500E-02	0.688	cis	Severe COVID-19
17739_1	HCDH	Hydroxyacyl-coenzyme A dehydrogenase, mitochondrial	Q16836	HADH	chr4:107989714	Wald ratio	1	-0.432	0.216	4.525E-02	0.688	cis	Severe COVID-19
17808_37	NIT2	Omega-amidase NIT2	Q9NQR4	NIT2	chr3:100334739	Inverse variance weighted	2	0.256	0.128	4.549E-02	0.688	cis	Severe COVID-19
13940_19	IP16:HIN 1	Gamma-interferon-inducible protein 16:Isoform 2, Hematopoietic expression, interferon-inducible nature, and nuclear localization 1	Q16666	IFI16	chr1:158999968	Wald ratio	1	0.454	0.229	4.754E-02	0.688	cis	Severe COVID-19
13102_1	FAM3D	Protein FAM3D	Q96BQ1	FAM3D	chr3:58666834	Inverse variance weighted	4	-0.095	0.048	4.795E-02	0.688	cis	Severe COVID-19
9754_33	Quinone reductase 2	Ribosyldihydronicotinamide dehydrogenase [quinone]	P16083	NQO2	chr6:2987987	Inverse variance weighted	8	0.052	0.026	4.812E-02	0.688	cis	Severe COVID-19
4500_50	SCGF-alpha	Stem cell growth factor-alpha	Q9Y240	CLEC11A	chr19:50723364	Inverse variance weighted	2	-0.078	0.040	4.849E-02	0.688	cis	Severe COVID-19
8337_65	PTPRU	Receptor-type tyrosine-protein phosphatase U	Q92729	PTPRU	chr1:29236516	Inverse variance weighted	5	-0.197	0.100	4.870E-02	0.688	cis	Severe COVID-19
17836_17	S100A16	Protein S100-A16	Q96FQ6	S100A16	chr1:153613145	Wald ratio	1	-0.515	0.262	4.900E-02	0.688	cis	Severe COVID-19
15385_116	FABP2	Fatty acid-binding protein, intestinal	P12104	FABP2	chr4:119322138	Inverse variance weighted	5	0.089	0.045	4.933E-02	0.688	cis	Severe COVID-19
19233_75	ATOX1	Copper transport protein ATOX1	O00244	ATOX1	chr5:151772532	Wald ratio	1	0.558	0.284	4.942E-02	0.688	cis	Severe COVID-19
8053_16	DJB14:CD	DnaJ homolog subfamily B member 14:Cytoplasmic domain	Q8TBM8	DNAJB14	chr4:99946618	Wald ratio	1	0.491	0.250	4.958E-02	0.688	cis	Severe COVID-19
18839_24	Thyroglobulin	Thyroglobulin	P01266	TG	chr8:132866958	Wald ratio	1	-0.361	0.185	5.107E-02	0.688	cis	Severe COVID-19
12469_19	MARE1	Microtubule-associated protein RP/EB family member 1	Q15691	MAPRE1	chr20:32819954	Wald ratio	1	-0.552	0.283	5.114E-02	0.688	cis	Severe COVID-19
3431_54	EphA1	Ephrin type-A receptor 1	P21709	EPHA1	chr7:143408856	Inverse variance weighted	7	-0.076	0.039	5.196E-02	0.688	cis	Severe COVID-19
3710_49	Angiostatin	Angiostatin	P00747	PLG	chr6:160702194	Inverse variance weighted	7	0.206	0.106	5.208E-02	0.688	cis	Severe COVID-19
19347_37	Carbonic Anhydrase XII	Carbonic anhydrase 12	O43570	CA12	chr15:63381846	Wald ratio	1	-0.809	0.417	5.220E-02	0.688	cis	Severe COVID-19
18916_25	Inosine triphosphatase	Inosine triphosphate pyrophosphatase	Q9BY32	ITPA	chr20:3208868	Inverse variance weighted	8	-0.068	0.035	5.245E-02	0.688	cis	Severe COVID-19
18884_22	DNJB4	DnaJ homolog subfamily B member 4	Q9UDY4	DNAJB4	chr1:77979175	Inverse variance weighted	3	0.173	0.089	5.269E-02	0.688	cis	Severe COVID-19
4234_8	IL-1 R4	Interleukin-1 receptor-like 1	Q01638	IL1RL1	chr2:102311502	Inverse variance weighted	8	-0.068	0.035	5.283E-02	0.688	cis	Severe COVID-19
12697_30	PI42A	Phosphatidylinositol 5-phosphate 4-kinase type-2 alpha	P48426	PIP4K2A	chr10:22714578	Wald ratio	1	0.337	0.174	5.291E-02	0.688	cis	Severe COVID-19
10037_98	SIG12:Ig-like V-type 2	Sialic acid-binding Ig-like lectin 12:Ig-like V-type 2 domain, Isoform long	Q96PQ1	SIGLEC12	chr19:51501800	Inverse variance weighted	4	-0.130	0.067	5.312E-02	0.688	cis	Severe COVID-19
10554_23	BGAL	Beta-galactosidase	P16278	GLB1	chr3:33097202	Wald ratio	1	0.873	0.452	5.358E-02	0.688	cis	Severe COVID-19
3206_4	LYVE1	Lymphatic vessel endothelial hyaluronic acid receptor 1	Q9Y5Y7	LYVE1	chr11:10611689	Inverse variance weighted	6	0.144	0.075	5.396E-02	0.688	cis	Severe COVID-19
9950_229	LAG-3	Lymphocyte activation gene 3 protein	P18627	LAG3	chr12:6772512	Inverse variance weighted	2	-0.237	0.123	5.406E-02	0.688	cis	Severe COVID-19
2973_15	CD36 ANTIGEN	Platelet glycoprotein 4	P16671	CD36	chr7:80369575	Inverse variance weighted	4	0.141	0.074	5.475E-02	0.688	cis	Severe COVID-19
16558_2	MYOC	Myocilin	Q99972	MYOC	chr1:171652688	Inverse variance weighted	8	0.081	0.042	5.491E-02	0.688	cis	Severe COVID-19
6402_8	PILRA isoform FDF03-deltaTM	Paired immunoglobulin-like type 2 receptor alpha isoform FDF03-deltaTM	Q9UKJ1	PILRA	chr7:100367530	Inverse variance weighted	2	0.095	0.049	5.500E-02	0.688	cis	Severe COVID-19
5006_71	MK13	Mitogen-activated protein kinase 13	O15264	MAPK13	chr6:36127809	Wald ratio	1	-0.389	0.203	5.529E-02	0.688	cis	Severe COVID-19
15596_7	HEXI1	Protein HEXIM1	O94992	HEXIM1	chr17:45148475	Wald ratio	1	0.552	0.288	5.539E-02	0.688	cis	Severe COVID-19
9213_24	FTCD	Formimidoyltransferase-cyclodeaminase	O95954	FTCD	chr21:46155579	Inverse variance weighted	3	-0.193	0.101	5.573E-02	0.688	cis	Severe COVID-19
18933_4	TGM4	Protein-glutamine gamma-glutamyltransferase 4	P49221	TGM4	chr3:44874608	Wald ratio	1	-0.523	0.274	5.591E-02	0.688	cis	Severe COVID-19
10977_55	UCMA	Unique cartilage matrix-associated protein	Q8WVF2	UCMA	chr10:13234374	Inverse variance weighted	5	0.095	0.050	5.683E-02	0.688	cis	Severe COVID-19
6493_9	CBPZ	Carboxypeptidase Z	Q66K79	CPZ	chr4:8592660	Inverse variance weighted	2	0.329	0.173	5.705E-02	0.688	cis	Severe COVID-19
8269_327	ARSK	Arylsulfatase K	Q6UWY0	ARSK	chr5:95555101	Wald ratio	1	0.432	0.227	5.710E-02	0.688	cis	Severe COVID-19
18307_71	PPase 2	Inorganic pyrophosphatase 2, mitochondrial	Q9H2U2	PPA2	chr4:105474067	Wald ratio	1	0.442	0.233	5.716E-02	0.688	cis	Severe COVID-19
4924_32	MMP-1	Interstitial collagenase	P03956	MMP1	chr11:102798160	Inverse variance weighted	9	0.085	0.045	5.822E-02	0.696	cis	Severe COVID-19
15565_102	CA125	Mucin-16	Q8WXI7	MUC16	chr19:8981342	Inverse variance weighted	2	-0.202	0.107	5.973E-02	0.708	cis	Severe COVID-19
3327_27	NET4	Netrin-4	Q9HB63	NTN4	chr12:95791189	Inverse variance weighted	3	0.179	0.095	6.009E-02	0.708	cis	Severe COVID-19
17832_12	IDI2	Isopentenyl-diphosphate delta-isomerase 2	Q9BXS1	IDI2	chr10:1025859	Inverse variance weighted	2	0.179	0.096	6.175E-02	0.711	cis	Severe COVID-19
16828_8	Collagen a1(VI)	Collagen alpha-1(VI) chain	P12109	COL6A1	chr21:45981770	Inverse variance weighted	10	-0.047	0.025	6.231E-02	0.711	cis	Severe COVID-19
16809_1	NDKM	Nucleoside diphosphate kinase, mitochondrial	O00746	NME4	chr16:396725	Inverse variance weighted	2	0.308	0.165	6.242E-02	0.711	cis	Severe COVID-19
14123_34	GI24:ECD	V-type immunoglobulin domain-containing suppressor of T-cell activation:Extracellular domain	Q9H7M9	VSIR	chr10:71773520	Inverse variance weighted	5	-0.282	0.152	6.290E-02	0.711	cis	Severe COVID-19
17680_12	EPHB1	Ephrin type-B receptor 1	P54762	EPHB1	chr3:134795260	Inverse variance weighted	3	0.080	0.043	6.295E-02	0.711	cis	Severe COVID-19
7806_33	B4GT7	Beta-1,4-galactosyltransferase 7	Q9UBV7	B4GALT7	chr5:177600132	Inverse variance weighted	7	0.126	0.068	6.348E-02	0.711	cis	Severe COVID-19
15560_52	Apo-TC II	Transcobalamin-2	P20062	TCN2	chr22:30607003	Inverse variance weighted	4	-0.080	0.043	6.457E-02	0.711	cis	Severe COVID-19
9719_145	MMP-16	Matrix metalloproteinase-16	P51512	MMP16	chr8:88328025	Wald ratio	1	-0.611	0.331	6.468E-02	0.711	cis	Severe COVID-19
15641_20	TEFF1	Tomoregulin-1	Q8IYR6	TMEFF1	chr9:100473149	Wald ratio	1	-0.482	0.261	6.507E-02	0.711	cis	Severe COVID-19
4159_130	Factor H	Complement factor H	P08603	CFH	chr1:196651754	Wald ratio	1	0.367	0.199	6.538E-02	0.711	cis	Severe COVID-19
7891_45	UGT 1A6	UDP-glucuronosyltransferase 1-6	P19224	UGT1A6	chr2:233691607	Inverse variance weighted	4	-0.152	0.083	6.559E-02	0.711	cis	Severe COVID-19

3044_3	PARC	C-C motif chemokine 18	P55774	CCL18	chr17:36064272	Inverse variance weighted	3	0.255	0.139	6.696E-02	0.711	cis	Severe COVID-19
16613_3	CAD17	Cadherin-17	Q12864	CDH17	chr8:94217303	Inverse variance weighted	5	0.094	0.051	6.730E-02	0.711	cis	Severe COVID-19
4911_49	Glutathione S-transferase Pi	Glutathione S-transferase P	P09211	GSTP1	chr11:67583742	Inverse variance weighted	2	-0.195	0.107	6.738E-02	0.711	cis	Severe COVID-19
8925_25	RIR2B	Ribonucleoside-diphosphate reductase subunit M2 B	Q7LG56	RRM2B	chr8:102238961	Inverse variance weighted	2	0.172	0.094	6.821E-02	0.711	cis	Severe COVID-19
17821_20	NMT2	Glycylpeptide N-tetradecanoyltransferase 2	O60551	NMT2	chr10:15168693	Wald ratio	1	0.435	0.239	6.873E-02	0.711	cis	Severe COVID-19
9981_18	K1467:C-term	Protein FAM234B:C-term	A2RU67	FAM234B	chr12:13044381	Inverse variance weighted	5	-0.170	0.093	6.876E-02	0.711	cis	Severe COVID-19
2992_59	IL-17 sR	Interleukin-17 receptor A	Q96F46	IL17RA	chr22:17084954	Inverse variance weighted	7	-0.033	0.018	6.923E-02	0.711	cis	Severe COVID-19
19173_5	ZFAN1	AN1-type zinc finger protein 1	Q8TCF1	ZFAND1	chr8:81732903	Inverse variance weighted	3	0.177	0.098	6.984E-02	0.711	cis	Severe COVID-19
2480_58	TIMP-3	Metalloproteinase inhibitor 3	P35625	TIMP3	chr22:32801705	Inverse variance weighted	10	0.050	0.028	6.997E-02	0.711	cis	Severe COVID-19
5825_49	IFN-g R1	Interferon gamma receptor 1	P15260	IFNGR1	chr6:137219449	Inverse variance weighted	3	-0.408	0.226	7.021E-02	0.711	cis	Severe COVID-19
9321_400	NMB	Neuromedin-B	P08949	NMB	chr15:84658563	Inverse variance weighted	2	-0.171	0.095	7.044E-02	0.711	cis	Severe COVID-19
11126_102	TRIO	Triple functional domain protein	O75962	TRIO	chr5:14143342	Wald ratio	1	-0.601	0.333	7.121E-02	0.711	cis	Severe COVID-19
15499_11	Attractin	Attractin	O75882	ATRN	chr20:3471018	Inverse variance weighted	5	-0.107	0.059	7.125E-02	0.711	cis	Severe COVID-19
15480_2	VNN2	Vascular non-inflammatory molecule 2	O95498	VNN2	chr6:132763459	Inverse variance weighted	11	0.044	0.025	7.175E-02	0.711	cis	Severe COVID-19
9580_5	Laminin gamma-2	Laminin subunit gamma-2	Q13753	LAMC2	chr1:183186238	Inverse variance weighted	4	-0.105	0.058	7.239E-02	0.711	cis	Severe COVID-19
2635_61	Layilin	Layilin	Q6UX15	LAYN	chr11:111540280	Wald ratio	1	-0.485	0.270	7.240E-02	0.711	cis	Severe COVID-19
18875_125	Chondrocalcin	Chondrocalcin	P02458	COL2A1	chr12:48004554	Inverse variance weighted	7	-0.077	0.043	7.242E-02	0.711	cis	Severe COVID-19
10924_258	NEUFC	Neuferricin	Q8WUJ1	CYB5D2	chr17:4143168	Inverse variance weighted	3	0.186	0.103	7.253E-02	0.711	cis	Severe COVID-19
10702_1	COSA1	Collagen alpha-1(XXVIII) chain	Q2UY09	COL28A1	chr7:7535873	Wald ratio	1	0.587	0.328	7.368E-02	0.718	cis	Severe COVID-19
7808_5	GLCE	D-glucuronyl C5-epimerase	O94923	GLCE	chr15:69160584	Inverse variance weighted	7	-0.066	0.037	7.545E-02	0.720	cis	Severe COVID-19
9715_15	IGSF3	Immunoglobulin superfamily member 3	O75054	IGSF3	chr1:116667755	Inverse variance weighted	3	0.310	0.174	7.549E-02	0.720	cis	Severe COVID-19
15398_2	HERV1	FAD-linked sulfhydryl oxidase ALR	P55789	GFER	chr16:1984193	Wald ratio	1	-0.473	0.267	7.602E-02	0.720	cis	Severe COVID-19
11378_37	KSYK:SH2, 1 and 2	Tyrosine-protein kinase SYK:Src Homology domain	P43405	SYK	chr9:90801787	Wald ratio	1	0.564	0.318	7.603E-02	0.720	cis	Severe COVID-19
3376_49	IL-17 RD	Interleukin-17 receptor D	Q8NFM7	IL17RD	chr3:57170306	Inverse variance weighted	4	0.072	0.040	7.626E-02	0.720	cis	Severe COVID-19
10916_44	PLA2R	Secretory phospholipase A2 receptor	Q13018	PLA2R1	chr2:160062615	Inverse variance weighted	6	0.057	0.032	7.644E-02	0.720	cis	Severe COVID-19
2418_55	Apo E	Apolipoprotein E	P02649	APOE	chr19:44905791	Inverse variance weighted	4	0.175	0.099	7.697E-02	0.721	cis	Severe COVID-19
6527_1	TRIL:ECD	TLR4 interactor with leucine rich repeats:Extracellular domain	Q7L0X0	TRIL	chr7:28958330	Wald ratio	1	0.344	0.196	7.943E-02	0.730	cis	Severe COVID-19
3905_62	UBE2N	Ubiquitin-conjugating enzyme E2 N	P61088	UBE2N	chr12:93441947	Wald ratio	1	-0.579	0.330	7.954E-02	0.730	cis	Severe COVID-19
6998_106	HAAH	Aspartyl/asparaginyl beta-hydroxylase	Q12797	ASPH	chr8:61714640	Inverse variance weighted	2	0.277	0.158	7.979E-02	0.730	cis	Severe COVID-19
12431_13	PELO	Protein pelota homolog	Q9BRX2	PELO	chr5:52787916	Wald ratio	1	-0.568	0.325	8.034E-02	0.730	cis	Severe COVID-19
8402_22	CYTL1	Cytokine-like protein 1	Q9NRR1	CYTL1	chr4:5019458	Inverse variance weighted	3	-0.127	0.072	8.039E-02	0.730	cis	Severe COVID-19
8035_6	CA198	Uncharacterized protein C1orf198	Q9H425	C1orf198	chr1:230869589	Wald ratio	1	0.228	0.131	8.130E-02	0.730	cis	Severe COVID-19
9173_21	PGM1	Phosphoglucomutase-1	P36871	PGM1	chr1:63593411	Inverse variance weighted	2	0.247	0.142	8.149E-02	0.730	cis	Severe COVID-19
3470_1	sE-Selectin	E-selectin	P16581	SELE	chr1:169764705	Wald ratio	1	0.328	0.188	8.195E-02	0.730	cis	Severe COVID-19
17682_1	CD46	Membrane cofactor protein	P15529	CD46	chr1:207752037	Inverse variance weighted	2	0.141	0.081	8.201E-02	0.730	cis	Severe COVID-19
9005_16	PLXA1	Plexin-A1	Q9UIW2	PLXNA1	chr3:126982693	Inverse variance weighted	3	-0.202	0.116	8.229E-02	0.730	cis	Severe COVID-19
8231_122	VEGF sR1	Vascular endothelial growth factor receptor 1	P17948	FLT1	chr13:28495145	Wald ratio	1	0.501	0.291	8.474E-02	0.745	cis	Severe COVID-19
13124_20	ISLR2	Immunoglobulin superfamily containing leucine-rich repeat protein 2	Q6UXK2	ISLR2	chr15:74100311	Inverse variance weighted	4	-0.178	0.104	8.488E-02	0.745	cis	Severe COVID-19
4162_54	Transferrin	Serotransferrin	P02787	TF	chr3:133746040	Inverse variance weighted	2	0.168	0.098	8.708E-02	0.760	cis	Severe COVID-19
9765_4	NDE1	Nuclear distribution protein nudE homolog 1	Q9NXR1	NDE1	chr16:15643267	Wald ratio	1	0.365	0.214	8.769E-02	0.762	cis	Severe COVID-19
4479_14	C1-Esterase Inhibitor	Plasma protease C1 inhibitor	P05155	SERPING1	chr11:57597387	Inverse variance weighted	6	-0.058	0.034	8.939E-02	0.768	cis	Severe COVID-19
10772_21	CGAT2	Chondroitin sulfate N-acetylgalactosaminyltransferase 2	Q8N6G5	CSGALNACT2	chr10:43138445	Wald ratio	1	0.267	0.157	8.973E-02	0.768	cis	Severe COVID-19
18895_54	GSTM4	Glutathione S-transferase Mu 4	Q03013	GSTM4	chr1:109656099	Inverse variance weighted	6	0.144	0.085	8.979E-02	0.768	cis	Severe COVID-19
6605_17	IGFALS	Insulin-like growth factor-binding protein complex acid labile subunit	P35858	IGFALS	chr16:1794971	Inverse variance weighted	3	-0.164	0.097	9.059E-02	0.768	cis	Severe COVID-19
11531_24	GI24:CD	V-type immunoglobulin domain-containing suppressor of T-cell activation:Cytoplasmic domain	Q9H7M9	VSIR	chr10:71773520	Wald ratio	1	0.411	0.244	9.169E-02	0.768	cis	Severe COVID-19
17691_1	TPP1	Tripeptidyl-peptidase 1	O14773	TPP1	chr11:6619448	Inverse variance weighted	5	-0.144	0.086	9.282E-02	0.768	cis	Severe COVID-19
7185_29	GPV	Platelet glycoprotein V	P40197	GP5	chr3:194399266	Inverse variance weighted	2	-0.290	0.173	9.343E-02	0.768	cis	Severe COVID-19
12386_11	AMPB	Aminopeptidase B	Q9H4A4	RNPEP	chr1:201982372	Inverse variance weighted	5	0.075	0.045	9.348E-02	0.768	cis	Severe COVID-19
17447_52	SFRP4	Secreted frizzled-related protein 4	Q6FHJ7	SFRP4	chr7:38025695	Inverse variance weighted	2	-0.112	0.067	9.394E-02	0.768	cis	Severe COVID-19
5810_25	Cripto	Teratocarcinoma-derived growth factor 1	P13385	TDGF1	chr3:46574534	Inverse variance weighted	6	0.226	0.135	9.398E-02	0.768	cis	Severe COVID-19
5078_82	EphB6	Ephrin type-B receptor 6	O15197	EPHB6	chr7:142855061	Inverse variance weighted	3	0.155	0.093	9.424E-02	0.768	cis	Severe COVID-19
6629_3	HBD-1	Beta-defensin 1	P60022	DEFB1	chr8:6877936	Inverse variance weighted	5	-0.082	0.049	9.425E-02	0.768	cis	Severe COVID-19
8859_51	CAH11	Carbonic anhydrase-related protein 11	O75493	CA11	chr19:48646187	Wald ratio	1	0.239	0.143	9.454E-02	0.768	cis	Severe COVID-19
12449_16	PPIH	Peptidyl-prolyl cis-trans isomerase H	O43447	PPIH	chr1:42658335	Inverse variance weighted	2	0.341	0.204	9.478E-02	0.768	cis	Severe COVID-19
12016_60	CBL	E3 ubiquitin-protein ligase CBL	P22681	CBL	chr11:119206298	Wald ratio	1	0.175	0.105	9.564E-02	0.771	cis	Severe COVID-19
4920_10	Lysozyme	Lysozyme C	P61626	LYZ	chr12:69348381	Inverse variance weighted	6	-0.062	0.037	9.715E-02	0.777	cis	Severe COVID-19
13563_259	ACBD7	Acyl-CoA-binding domain-containing protein 7	Q8N6N7	ACBD7	chr10:15088776	Wald ratio	1	0.648	0.392	9.772E-02	0.777	cis	Severe COVID-19
3352_80	Carbonic anhydrase 6	Carbonic anhydrase 6	P23280	CA6	chr1:8945867	Inverse variance weighted	6	-0.046	0.028	9.829E-02	0.777	cis	Severe COVID-19
4254_6	NUDC3	NudC domain-containing protein 3	Q8IVD9	NUDCD3	chr7:44490658	Wald ratio	1	0.372	0.225	9.833E-02	0.777	cis	Severe COVID-19
16302_11	SPLC2	BPI fold-containing family A member 2	Q96DR5	BPIFA2	chr20:33161768	Wald ratio	1	-0.378	0.229	9.868E-02	0.777	cis	Severe COVID-19
13954_9	GNA1	Glucosamine 6-phosphate N-acetyltransferase	Q96EK6	GNPNAT1	chr14:52791668	Wald ratio	1	-0.428	0.260	9.973E-02	0.782	cis	Severe COVID-19
12399_194	CCD50	Coiled-coil domain-containing protein 50	Q8IVM0	CCDC50	chr3:191329085	Wald ratio	1	-0.506	0.309	1.011E-01	0.789	cis	Severe COVID-19
3003_29	NKp30	Natural cytotoxicity triggering receptor 3	O14931	NCR3	chr6:31593006	Wald ratio	1	0.332	0.203	1.027E-01	0.792	cis	Severe COVID-19
10339_48	NSE	Gamma-enolase	P09104	ENO2	chr12:6913745	Wald ratio	1	0.290	0.178	1.028E-01	0.792	cis	Severe COVID-19
15305_7	Secretagogin	Secretagogin	O76038	SCGN	chr6:25652201	Inverse variance weighted	2	0.443	0.271	1.029E-01	0.792	cis	Severe COVID-19
9266_1	sTREM-1	Triggering receptor expressed on myeloid cells 1	Q9NP99	TREM1	chr6:41286682	Inverse variance weighted	6	0.131	0.081	1.042E-01	0.795	cis	Severe COVID-19
4155_3	Tenascin	Tenascin	P24821	TNC	chr9:115118207	Inverse variance weighted	12	-0.055	0.034	1.053E-01	0.795	cis	Severe COVID-19
3461_58	PGCB	Brevican core protein	Q96GW7	BCAN	chr1:156641390	Inverse variance weighted	2	0.120	0.074	1.057E-01	0.795	cis	Severe COVID-19
2813_11	ART	Agouti-related protein	O00253	AGRP	chr16:67483547	Inverse variance weighted	2	0.130	0.080	1.066E-01	0.795	cis	Severe COVID-19
18832_65	SAA2	Serum amyloid A-2 protein	P0DJI9	SAA2	chr11:18248668	Wald ratio	1	-0.516	0.320	1.067E-01	0.795	cis	Severe COVID-19
9185_15	TFF1	Trefoil factor 1	P04155	TFF1	chr21:42366535	Inverse variance weighted	3	0.087	0.054	1.068E-01	0.795	cis	Severe COVID-19
9748_31	GSTM3-3	Glutathione S-transferase Mu 3	P21266	GSTM3	chr1:109741038	Inverse variance weighted	5	0.103	0.064	1.072E-01	0.795	cis	Severe COVID-19
8795_48	TR:ECD	Transferrin receptor protein 1:Extracellular domain	P02786	TFRC	chr3:196082153	Wald ratio	1	0.323	0.201	1.077E-01	0.795	cis	Severe COVID-19
12940_35	AL3B1	Aldehyde dehydrogenase family 3 member B1	P43353	ALDH3B1	chr11:68008578	Wald ratio	1	0.214	0.133	1.079E-01	0.795	cis	Severe COVID-19
13682_47	M-CSF R	Macrophage colony-stimulating factor 1 receptor	P07333	CSF1R	chr5:150113372	Wald ratio	1	-0.363	0.226	1.084E-01	0.795	cis	Severe COVID-19
13986_6	LANC1	LanC-like protein 1	O43813	LANCL1	chr2:210477652	Inverse variance weighted	2	-0.300	0.187	1.085E-01	0.795	cis	Severe COVID-19
18871_24	AIF1L	Allograft inflammatory factor 1-like	Q9BQI0	AIF1L	chr9:131096476	Inverse variance weighted	5	-0.079	0.049	1.093E-01	0.797	cis	Severe COVID-19
19365_11	BCAT2	Branched-chain-amino-acid aminotransferase, mitochondrial	O15382	BCAT2	chr19:48811029	Wald ratio	1	0.171	0.107	1.105E-01	0.803	cis	Severe COVID-19

18899_82	HDGR3	Hepatoma-derived growth factor-related protein 3	Q9Y3E1	HDGFL3	chr15:83207823	Wald ratio	1	-0.247	0.155	1.116E-01	0.807	cis	Severe COVID-19
5111_15	NRX3B	Neurexin-3-beta	Q9HDB5	NRXN3	chr14:78170373	Inverse variance weighted	2	-0.358	0.225	1.122E-01	0.808	cis	Severe COVID-19
18174_79	PDC6I	Programmed cell death 6-interacting protein	Q8WUM4	PDCD6IP	chr3:33798571	Wald ratio	1	-0.224	0.141	1.128E-01	0.809	cis	Severe COVID-19
7206_20	F16P1	Fructose-1,6-bisphosphatase 1	P09467	FBP1	chr9:94640249	Inverse variance weighted	2	0.247	0.156	1.136E-01	0.810	cis	Severe COVID-19
15540_6	Vimentin	Vimentin	P08670	VIM	chr10:17228241	Inverse variance weighted	2	0.288	0.183	1.145E-01	0.810	cis	Severe COVID-19
4127_75	C6	Complement component C6	P13671	C6	chr5:41261438	Inverse variance weighted	4	-0.120	0.076	1.147E-01	0.810	cis	Severe COVID-19
17697_2	OVCA2	Esterase OVCA2	Q8WZ82	OVCA2	chr17:2042022	Wald ratio	1	-0.334	0.212	1.149E-01	0.810	cis	Severe COVID-19
18337_4	GMDS	GDP-mannose 4,6 dehydratase	O60547	GMDS	chr6:2245605	Wald ratio	1	-0.454	0.290	1.170E-01	0.820	cis	Severe COVID-19
4498_62	NCAM-120	Neural cell adhesion molecule 1, 120 kDa isoform	P13591	NCAM1	chr11:112961247	Inverse variance weighted	3	0.078	0.050	1.180E-01	0.820	cis	Severe COVID-19
5091_28	ILT-4	Leukocyte immunoglobulin-like receptor subfamily B member 2	Q8N423	LILRB2	chr19:54281184	Inverse variance weighted	4	-0.224	0.144	1.186E-01	0.820	cis	Severe COVID-19
3074_6	LBP	Lipopolysaccharide-binding protein	P18428	LBP	chr20:38346482	Inverse variance weighted	5	-0.057	0.037	1.201E-01	0.820	cis	Severe COVID-19
9747_48	MARK3	MAP/microtubule affinity-regulating kinase 3	P27448	MARK3	chr14:103385377	Wald ratio	1	0.741	0.477	1.203E-01	0.820	cis	Severe COVID-19
3805_16	Endocan	Endothelial cell-specific molecule 1	Q9NQ30	ESM1	chr5:55022671	Inverse variance weighted	3	-0.150	0.097	1.217E-01	0.820	cis	Severe COVID-19
7128_9	VWA2	von Willebrand factor A domain-containing protein 2	Q5GFL6	VWA2	chr10:114239254	Inverse variance weighted	6	0.100	0.065	1.231E-01	0.820	cis	Severe COVID-19
15566_10	Calponin-1	Calponin-1	P51911	CNN1	chr19:11538767	Wald ratio	1	0.186	0.121	1.234E-01	0.820	cis	Severe COVID-19
15435_4	PNP	Purine nucleoside phosphorylase	P00491	PNP	chr14:20461992	Inverse variance weighted	2	-0.149	0.097	1.238E-01	0.820	cis	Severe COVID-19
6342_10	Nephronectin	Nephronectin	Q6UXI9	NPNT	chr4:105894775	Inverse variance weighted	3	0.383	0.249	1.244E-01	0.820	cis	Severe COVID-19
5337_64	B7-2	T-lymphocyte activation antigen CD86	P42081	CD86	chr3:122055362	Wald ratio	1	-0.397	0.258	1.245E-01	0.820	cis	Severe COVID-19
3186_2	C2	Complement C2	P06681	C2	chr6:31897785	Wald ratio	1	-0.255	0.166	1.248E-01	0.820	cis	Severe COVID-19
4430_44	Collectin Kidney 1	Collectin-11	Q9BWP8	COLEC11	chr2:3594832	Inverse variance weighted	10	0.048	0.031	1.249E-01	0.820	cis	Severe COVID-19
9312_8	AZGP1	Zinc-alpha-2-glycoprotein	P25311	AZGP1	chr7:99976042	Inverse variance weighted	3	-0.366	0.238	1.250E-01	0.820	cis	Severe COVID-19
3000_66	MBL	Mannose-binding protein C	P11226	MBL2	chr10:52772784	Inverse variance weighted	9	-0.042	0.027	1.253E-01	0.820	cis	Severe COVID-19
15308_108	VWC2	Brorin	Q2TAL6	VWC2	chr7:49773638	Inverse variance weighted	7	0.068	0.044	1.253E-01	0.820	cis	Severe COVID-19
3210_1	METAP1	Methionine aminopeptidase 1	P53582	METAP1	chr4:98995659	Wald ratio	1	0.467	0.305	1.257E-01	0.820	cis	Severe COVID-19
11646_4	Carbohydrate sulfotransferase 9	Carbohydrate sulfotransferase 9	Q7L1S5	CHST9	chr18:27185308	Inverse variance weighted	4	-0.082	0.054	1.261E-01	0.820	cis	Severe COVID-19
15503_20	Lefty-A	Left-right determination factor 2	O00292	LEFTY2	chr1:225941383	Inverse variance weighted	10	0.054	0.035	1.267E-01	0.820	cis	Severe COVID-19
2190_55	Coagulation Factor XI	Coagulation Factor XI	P03951	F11	chr4:186266189	Inverse variance weighted	4	-0.093	0.061	1.269E-01	0.820	cis	Severe COVID-19
11248_43	HEM4	Uroporphyrinogen-III synthase	P10746	UROS	chr10:125823258	Inverse variance weighted	4	0.115	0.076	1.272E-01	0.820	cis	Severe COVID-19
17776_15	PECR	Peroxisomal trans-2-enoyl-CoA reductase	Q9BY49	PECR	chr2:216082955	Wald ratio	1	0.240	0.158	1.279E-01	0.820	cis	Severe COVID-19
5475_10	PKC-B-II	Protein kinase C beta type (splice variant beta-II)	P05771	PRKCB	chr16:23835983	Wald ratio	1	-0.301	0.198	1.280E-01	0.820	cis	Severe COVID-19
8403_18	Fatty acid synthase	Fatty acid synthase	P49327	FASN	chr17:82098294	Wald ratio	1	0.161	0.106	1.283E-01	0.820	cis	Severe COVID-19
13119_26	protein Z inhibitor	Protein Z-dependent protease inhibitor	Q9UK55	SERPINA10	chr14:94293268	Inverse variance weighted	6	0.053	0.035	1.292E-01	0.820	cis	Severe COVID-19
12522_6	RD23B	UV excision repair protein RAD23 homolog B	P54727	RAD23B	chr9:107283137	Wald ratio	1	0.405	0.267	1.296E-01	0.820	cis	Severe COVID-19
6359_50	AGO61	Protein O-linked-mannose beta-1,4-N-acetylglucosaminyltransferase 2	Q8NAT1	POMGNT2	chr3:43106085	Inverse variance weighted	4	-0.127	0.084	1.305E-01	0.820	cis	Severe COVID-19
5227_60	PDK1	[Pyruvate dehydrogenase (acetyl-transferring)] kinase isozyme 1, mitochondrial	Q15118	PDK1	chr2:172555373	Inverse variance weighted	3	0.157	0.104	1.308E-01	0.820	cis	Severe COVID-19
16856_79	MARE2	Microtubule-associated protein RP/EB family member 2	Q15555	MAPRE2	chr18:34976928	Wald ratio	1	-0.150	0.099	1.309E-01	0.820	cis	Severe COVID-19
5610_32	CJ038	Protein FAM171A1	Q5VUB5	FAM171A1	chr10:15371289	Inverse variance weighted	2	-0.277	0.184	1.310E-01	0.820	cis	Severe COVID-19
8606_39	GPNMB:CD	Transmembrane glycoprotein NMB:Cytoplasmic domain	Q14956	GPNMB	chr7:23235967	Inverse variance weighted	7	-0.098	0.065	1.316E-01	0.821	cis	Severe COVID-19
3820_68	MAPK2	MAP kinase-activated protein kinase 2	P49137	MAPKAPK2	chr1:206684905	Wald ratio	1	-0.129	0.086	1.321E-01	0.821	cis	Severe COVID-19
8794_13	DPEP1	Dipeptidase 1	P16444	DPEP1	chr16:89613308	Inverse variance weighted	6	0.057	0.038	1.331E-01	0.821	cis	Severe COVID-19
5731_1	ISK6	Serine protease inhibitor Kazal-type 6	Q6UWN8	SPINK6	chr5:148202794	Inverse variance weighted	9	-0.058	0.038	1.335E-01	0.821	cis	Severe COVID-19
9838_4	SMAD1	Mothers against decapentaplegic homolog 1	Q15797	SMAD1	chr4:145481194	Wald ratio	1	-0.449	0.300	1.335E-01	0.821	cis	Severe COVID-19
9314_9	PSG5	Pregnancy-specific beta-1-glycoprotein 5	Q15238	PSG5	chr19:43186536	Inverse variance weighted	3	-0.225	0.150	1.347E-01	0.821	cis	Severe COVID-19
3283_21	BGH3	Transforming growth factor-beta-induced protein ig-h3	Q15582	TGFBI	chr5:136028988	Inverse variance weighted	5	-0.067	0.045	1.349E-01	0.821	cis	Severe COVID-19
2579_17	MMP-9	Matrix metalloproteinase-9	P14780	MMP9	chr20:46008908	Wald ratio	1	-0.299	0.200	1.352E-01	0.821	cis	Severe COVID-19
4969_2	Carbonic anhydrase I	Carbonic anhydrase 1	P00915	CA1	chr8:85379014	Inverse variance weighted	3	-0.157	0.105	1.355E-01	0.821	cis	Severe COVID-19
3169_70	IDUA	Alpha-L-iduronidase	P35475	IDUA	chr4:986997	Inverse variance weighted	7	0.090	0.061	1.375E-01	0.825	cis	Severe COVID-19
12643_4	ARRB1	Beta-arrestin-1	P49407	ARRB1	chr11:75351705	Wald ratio	1	-0.288	0.194	1.379E-01	0.825	cis	Severe COVID-19
13669_6	FGFR-3:ECD	Fibroblast growth factor receptor 3:Extracellular domain	P22607	FGFR3	chr4:1793293	Inverse variance weighted	4	-0.111	0.075	1.380E-01	0.825	cis	Severe COVID-19
15449_33	TIM-4	T-cell immunoglobulin and mucin domain-containing protein 4	Q96H15	TIMD4	chr5:156963226	Inverse variance weighted	5	-0.151	0.102	1.381E-01	0.825	cis	Severe COVID-19
5687_5	GLU2B	Glucosidase 2 subunit beta	P14314	PRKCSH	chr19:11435284	Wald ratio	1	0.406	0.274	1.390E-01	0.827	cis	Severe COVID-19
2620_4	gp130, soluble	Interleukin-6 receptor subunit beta	P40189	IL6ST	chr5:55995022	Inverse variance weighted	4	0.078	0.053	1.398E-01	0.829	cis	Severe COVID-19
15299_102	MESD2	LDLR chaperone MESD	Q14696	MESD	chr15:80989828	Wald ratio	1	0.484	0.329	1.411E-01	0.832	cis	Severe COVID-19
12370_30	Apo F	Apolipoprotein F	Q13790	APOF	chr12:56362857	Inverse variance weighted	2	-0.157	0.107	1.429E-01	0.832	cis	Severe COVID-19
17856_23	NT5M	5'(3')-deoxyribonucleotidase, mitochondrial	Q9NPB1	NT5M	chr17:17303335	Wald ratio	1	-0.184	0.126	1.431E-01	0.832	cis	Severe COVID-19
9940_35	DUS28	Dual specificity phosphatase 28	Q4G0W2	DUSP28	chr2:240560054	Inverse variance weighted	2	0.168	0.115	1.433E-01	0.832	cis	Severe COVID-19
5223_59	GCKR	Glucokinase regulatory protein	Q14397	GCKR	chr2:27496839	Wald ratio	1	-0.236	0.161	1.433E-01	0.832	cis	Severe COVID-19
13700_10	annexin II	Annexin A2	P07355	ANXA2	chr15:60402883	Inverse variance weighted	6	-0.169	0.116	1.436E-01	0.832	cis	Severe COVID-19
9638_2	TIGIT	T-cell immunoreceptor with Ig and ITIM domains	Q495A1	TIGIT	chr3:114276913	Wald ratio	1	-0.285	0.195	1.444E-01	0.832	cis	Severe COVID-19
9409_11	TRYB1	Tryptase beta-1	Q15661	TPSAB1	chr16:1240379	Inverse variance weighted	8	0.056	0.039	1.448E-01	0.832	cis	Severe COVID-19
12347_29	CCM2	Cerebral cavernous malformations 2 protein	Q9BSQ5	CCM2	chr7:44999475	Wald ratio	1	-0.359	0.247	1.453E-01	0.832	cis	Severe COVID-19
18382_109	Catechol O-methyltransferase	Catechol O-methyltransferase	P21964	COMT	chr22:19941371	Wald ratio	1	-0.246	0.169	1.457E-01	0.832	cis	Severe COVID-19
16074_12	GRB2-related adapter protein 2	GRB2-related adapter protein 2	O75791	GRAP2	chr22:39901084	Wald ratio	1	-0.675	0.464	1.458E-01	0.832	cis	Severe COVID-19
15620_4	NLGN1	Neuroligin-1	Q8N2Q7	NLGN1	chr3:173396284	Inverse variance weighted	3	0.264	0.182	1.465E-01	0.832	cis	Severe COVID-19
5483_1	RGMA	Repulsive guidance molecule A	Q96B86	RGMA	chr15:93089211	Inverse variance weighted	4	0.116	0.080	1.468E-01	0.832	cis	Severe COVID-19
3348_49	BMP-1	Bone morphogenetic protein 1	P13497	BMP1	chr8:22165140	Wald ratio	1	0.336	0.232	1.471E-01	0.832	cis	Severe COVID-19
4989_7	Fibrinogen g-chain dimer	Fibrinogen gamma chain	P02679	FGG	chr4:154612967	Wald ratio	1	-0.150	0.104	1.485E-01	0.836	cis	Severe COVID-19
18830_1	Omentin	Intelectin-1	Q8WWA0	ITLN1	chr1:160885180	Wald ratio	1	0.375	0.260	1.489E-01	0.836	cis	Severe COVID-19
13931_22	PSMD9	26S proteasome non-ATPase regulatory subunit 9	O00233	PSMD9	chr12:121888732	Wald ratio	1	0.171	0.119	1.494E-01	0.836	cis	Severe COVID-19
5238_26	PPIE	Peptidyl-prolyl cis-trans isomerase E	Q9UNP9	PPIE	chr1:39692182	Inverse variance weighted	2	0.074	0.051	1.499E-01	0.836	cis	Severe COVID-19
17419_17	TES	Testin	Q9UGI8	TES	chr7:116210506	Wald ratio	1	-0.284	0.198	1.506E-01	0.838	cis	Severe COVID-19
9744_139	DNJA4	DnaJ homolog subfamily A member 4	Q8WW22	DNAJA4	chr15:78264086	Wald ratio	1	0.451	0.315	1.519E-01	0.839	cis	Severe COVID-19
6551_94	Vaspin	Serpin A12	Q8IW75	SERPINA12	chr14:94517844	Inverse variance weighted	5	-0.060	0.042	1.520E-01	0.839	cis	Severe COVID-19
4209_60	DRG-1	Vacuolar protein sorting-associated protein VTA1 homolog	Q9NP79	VTA1	chr6:142147162	Wald ratio	1	-0.401	0.280	1.524E-01	0.839	cis	Severe COVID-19
6984_6	IGSF8	Immunoglobulin superfamily member 8	Q969P0	IGSF8	chr1:160098943	Inverse variance weighted	2	0.144	0.101	1.531E-01	0.839	cis	Severe COVID-19
17799_9	6PGL	6-phosphogluconolactonase	O95336	PGLS	chr19:17511636	Inverse variance weighted	3	-0.257	0.180	1.535E-01	0.839	cis	Severe COVID-19
3344_60	Antithrombin III	Antithrombin-III	P01008	SERPINC1	chr1:173917327	Wald ratio	1	0.337	0.236	1.539E-01	0.839	cis	Severe COVID-19
5316_54	Prothrombin	Prothrombin	P00734	F2	chr11:46719196	Wald ratio	1	0.394	0.278	1.565E-01	0.849	cis	Severe COVID-19

6393_63	Endoplasmin	Endoplasmin	P14625	HSP90B1	chr12:103930107	Inverse variance weighted	6	-0.034	0.024	1.575E-01	0.849	cis	Severe COVID-19
10042_8	SGK3	Serine/threonine-protein kinase Sgk3	Q96BR1	SGK3	chr8:66712734	Wald ratio	1	0.484	0.343	1.587E-01	0.849	cis	Severe COVID-19
10630_5	HTAI2	Oxidoreductase HTATIP2	Q9BUP3	HTATIP2	chr11:20363685	Inverse variance weighted	9	-0.056	0.039	1.588E-01	0.849	cis	Severe COVID-19
5000_52	LG3BP	Galectin-3-binding protein	Q08380	LGALS3BP	chr17:78979947	Inverse variance weighted	3	-0.148	0.105	1.590E-01	0.849	cis	Severe COVID-19
2797_56	Apo B	Apolipoprotein B	P04114	APOB	chr2:21044073	Wald ratio	1	0.290	0.206	1.590E-01	0.849	cis	Severe COVID-19
11152_46	kallikrein 13	Kallikrein-13	Q9UKR3	KLK13	chr19:51065114	Wald ratio	1	-0.257	0.183	1.593E-01	0.849	cis	Severe COVID-19
2516_57	6Ckine	C-C motif chemokine 21	O00585	CCL21	chr9:34710136	Inverse variance weighted	2	0.207	0.148	1.608E-01	0.849	cis	Severe COVID-19
17331_138	KREM1	Kremen protein 1	Q96MU8	KREMEN1	chr22:29073035	Inverse variance weighted	3	-0.124	0.089	1.613E-01	0.849	cis	Severe COVID-19
11615_16	DAPP1	Dual adapter for phosphotyrosine and 3-phosphotyrosine and 3-phosphoinositide	Q9UN19	DAPP1	chr4:99816827	Wald ratio	1	0.236	0.169	1.617E-01	0.849	cis	Severe COVID-19
11530_37	HEM3	Porphobilinogen deaminase	P08397	HMBS	chr11:119084866	Wald ratio	1	0.407	0.292	1.624E-01	0.849	cis	Severe COVID-19
19488_1	PDCD6	Programmed cell death protein 6	O75340	PDCD6	chr5:271621	Inverse variance weighted	3	0.135	0.097	1.625E-01	0.849	cis	Severe COVID-19
7784_1	Kininogen, HMW	Kininogen-1	P01042	KNG1	chr3:186717348	Inverse variance weighted	2	0.322	0.230	1.625E-01	0.849	cis	Severe COVID-19
3420_21	Carbonic anhydrase XIII	Carbonic anhydrase 13	Q8N1Q1	CA13	chr8:85220587	Inverse variance weighted	3	-0.076	0.054	1.635E-01	0.849	cis	Severe COVID-19
19254_125	GMPR1	GMP reductase 1	P36959	GMPR	chr6:16238587	Inverse variance weighted	8	-0.034	0.025	1.637E-01	0.849	cis	Severe COVID-19
8814_33	SAPL1	Proactivator polypeptide-like 1	Q6NUJ1	PSAPL1	chr4:7434930	Inverse variance weighted	8	-0.056	0.040	1.638E-01	0.849	cis	Severe COVID-19
15375_49	Carboxypeptidase B1	Carboxypeptidase B	P15086	CPB1	chr3:148791102	Inverse variance weighted	2	0.098	0.071	1.655E-01	0.855	cis	Severe COVID-19
15395_15	GST M1-1	Glutathione S-transferase Mu 1	P09488	GSTM1	chr1:109687814	Inverse variance weighted	5	-0.127	0.092	1.670E-01	0.856	cis	Severe COVID-19
7179_69	NFASC	Neurofascin	O94856	NFASC	chr1:204828651	Inverse variance weighted	4	0.057	0.041	1.671E-01	0.856	cis	Severe COVID-19
5132_71	TCCR	Interleukin-27 receptor subunit alpha	Q6UWB1	IL27RA	chr19:14031762	Inverse variance weighted	6	-0.058	0.042	1.672E-01	0.856	cis	Severe COVID-19
7921_65	FJX1	Four-jointed box protein 1	Q86VR8	FJX1	chr11:35618460	Inverse variance weighted	4	0.080	0.058	1.687E-01	0.860	cis	Severe COVID-19
11134_30	RF1ML	Peptide chain release factor 1-like, mitochondrial	Q9UGC7	MTRF1L	chr6:153002709	Wald ratio	1	0.130	0.094	1.690E-01	0.860	cis	Severe COVID-19
10974_20	ISK7	Serine protease inhibitor Kazal-type 7	P58062	SPINK7	chr5:148312419	Wald ratio	1	-0.177	0.129	1.700E-01	0.860	cis	Severe COVID-19
14054_17	IL-15 Ra	Interleukin-15 receptor subunit alpha	Q13261	IL15RA	chr10:5978187	Inverse variance weighted	5	0.052	0.038	1.701E-01	0.860	cis	Severe COVID-19
6923_1	PLOD2	Procollagen-lysine,2-oxoglutarate 5-dioxygenase 2	O00469	PLOD2	chr3:146163725	Inverse variance weighted	2	-0.192	0.140	1.705E-01	0.860	cis	Severe COVID-19
3581_53	a2-HS-Glycoprotein	Alpha-2-HS-glycoprotein	P02765	AHSG	chr3:186613060	Inverse variance weighted	4	-0.155	0.113	1.712E-01	0.861	cis	Severe COVID-19
9343_16	IL-2 sRb	Interleukin-2 receptor subunit beta	P14784	IL2RB	chr22:37175118	Wald ratio	1	-0.271	0.199	1.731E-01	0.864	cis	Severe COVID-19
13450_49	UBP8	Ubiquitin carboxyl-terminal hydrolase 8	P40818	USP8	chr15:50424380	Wald ratio	1	0.244	0.179	1.743E-01	0.864	cis	Severe COVID-19
6556_5	ENPP5	Ectonucleotide pyrophosphatase/phosphodiesterase family member 5	Q9UJA9	ENPP5	chr6:46170980	Inverse variance weighted	7	-0.045	0.033	1.748E-01	0.864	cis	Severe COVID-19
4721_54	TFF3	Trefoil factor 3	Q07654	TFF3	chr21:42315409	Inverse variance weighted	2	-0.227	0.167	1.751E-01	0.864	cis	Severe COVID-19
6223_5	GUC2B	Guanylate cyclase activator 2B	Q16661	GUCA2B	chr1:42153410	Inverse variance weighted	2	-0.294	0.217	1.756E-01	0.864	cis	Severe COVID-19
17384_110	K6PF	ATP-dependent 6-phosphofructokinase, muscle type	P08237	PFKM	chr12:48105139	Wald ratio	1	-0.205	0.151	1.757E-01	0.864	cis	Severe COVID-19
6462_12	TIMP-4	Metalloproteinase inhibitor 4	Q99727	TIMP4	chr3:12158912	Inverse variance weighted	8	-0.084	0.062	1.769E-01	0.864	cis	Severe COVID-19
2611_72	Dtk	Tyrosine-protein kinase receptor TYRO3	Q06418	TYRO3	chr15:41557675	Wald ratio	1	0.204	0.151	1.776E-01	0.864	cis	Severe COVID-19
5628_21	SEM3G	Semaphorin-3G	Q9NS98	SEMA3G	chr3:52445103	Inverse variance weighted	5	0.157	0.116	1.776E-01	0.864	cis	Severe COVID-19
11308_8	CR3L4	Cyclic AMP-responsive element-binding protein 3-like protein 4	Q8TEY5	CREB3L4	chr1:153967534	Inverse variance weighted	2	0.145	0.108	1.776E-01	0.864	cis	Severe COVID-19
2700_56	Protein S	Vitamin K-dependent protein S	P07225	PROS1	chr3:93980003	Wald ratio	1	-0.337	0.250	1.782E-01	0.864	cis	Severe COVID-19
19238_12	GLNA	Glutamine synthetase	P15104	GLUL	chr1:182392206	Wald ratio	1	-0.402	0.299	1.785E-01	0.864	cis	Severe COVID-19
5581_28	FGL1	Fibrinogen-like protein 1	Q08830	FGL1	chr8:17910365	Inverse variance weighted	7	0.045	0.034	1.785E-01	0.864	cis	Severe COVID-19
17686_27	TBCB	Tubulin-folding cofactor B	Q99426	TBCB	chr19:36114289	Wald ratio	1	0.319	0.238	1.793E-01	0.866	cis	Severe COVID-19
4464_10	Sialoadhesin	Sialoadhesin	Q9BZZ2	SIGLEC1	chr20:3712600	Wald ratio	1	0.433	0.323	1.800E-01	0.866	cis	Severe COVID-19
2737_22	NovH	Protein NOV homolog	P48745	CCN3	chr8:119416446	Wald ratio	1	0.292	0.218	1.803E-01	0.866	cis	Severe COVID-19
10605_22	APMAP	Adipocyte plasma membrane-associated protein	Q9HDC9	APMAP	chr20:24992751	Wald ratio	1	0.351	0.262	1.812E-01	0.868	cis	Severe COVID-19
8244_16	FUT8	Alpha-(1,6)-fucosyltransferase	Q9BYC5	FUT8	chr14:65410592	Inverse variance weighted	10	-0.039	0.029	1.824E-01	0.871	cis	Severe COVID-19
19448_104	UPP1	Uridine phosphorylase 1	Q16831	UPP1	chr7:48088628	Wald ratio	1	-0.280	0.211	1.829E-01	0.871	cis	Severe COVID-19
16915_153	SEM4A	Semaphorin-4A	Q9H3S1	SEMA4A	chr1:156147366	Wald ratio	1	0.116	0.087	1.844E-01	0.873	cis	Severe COVID-19
2677_1	ERBB1	Epidermal growth factor receptor	P00533	EGFR	chr7:55019017	Inverse variance weighted	3	0.139	0.105	1.844E-01	0.873	cis	Severe COVID-19
3435_53	FN1.4	Fibronectin Fragment 4	P02751	FN1	chr2:215436073	Wald ratio	1	0.775	0.585	1.853E-01	0.873	cis	Severe COVID-19
8982_65	TSP3	Thrombospondin-3	P49746	THBS3	chr1:155209051	Inverse variance weighted	3	-0.157	0.119	1.857E-01	0.873	cis	Severe COVID-19
17694_32	PSME2	Proteasome activator complex subunit 2	Q9UL46	PSME2	chr14:24147570	Inverse variance weighted	2	-0.204	0.155	1.861E-01	0.873	cis	Severe COVID-19
15576_158	ECP	Eosinophil cationic protein	P12724	RNASE3	chr14:20891385	Inverse variance weighted	5	0.051	0.039	1.881E-01	0.879	cis	Severe COVID-19
14205_6	HEXI2	Protein HEXIM2	Q96MH2	HEXIM2	chr17:45160700	Wald ratio	1	0.426	0.324	1.884E-01	0.879	cis	Severe COVID-19
2828_82	HAI-1	Kunitz-type protease inhibitor 1	O43278	SPINT1	chr15:40844018	Inverse variance weighted	2	-0.120	0.092	1.888E-01	0.879	cis	Severe COVID-19
8296_117	KDEL2	KDEL motif-containing protein 2	Q7Z4H8	POGLUT3	chr11:108498405	Inverse variance weighted	6	0.052	0.039	1.907E-01	0.885	cis	Severe COVID-19
15558_63	AMPE	Glutamyl aminopeptidase	Q07075	ENPEP	chr4:110365733	Inverse variance weighted	8	0.056	0.043	1.932E-01	0.894	cis	Severe COVID-19
6366_38	TXD15	Thioredoxin domain-containing protein 15	Q96J42	TXNDC15	chr5:134874371	Inverse variance weighted	3	0.072	0.055	1.959E-01	0.903	cis	Severe COVID-19
9249_17	TMEM9:ECD	Transmembrane protein 9:Extracellular domain	Q9P0T7	TMEM9	chr1:201171574	Wald ratio	1	-0.301	0.234	1.973E-01	0.903	cis	Severe COVID-19
7099_33	FSTL5	Follistatin-related protein 5	Q8N475	FSTL5	chr4:162164004	Wald ratio	1	0.189	0.147	1.974E-01	0.903	cis	Severe COVID-19
8866_53	QPCTL	Glutaminyl-peptide cyclotransferase-like protein	Q9NXS2	QPCTL	chr19:45692403	Inverse variance weighted	6	-0.059	0.046	1.982E-01	0.903	cis	Severe COVID-19
4867_15	VEGF121	Vascular endothelial growth factor A, isoform 121	P15692	VEGFA	chr6:43770184	Inverse variance weighted	4	0.177	0.137	1.982E-01	0.903	cis	Severe COVID-19
19557_3	KLOTB	Beta-klotho	Q86Z14	KLB	chr4:39406930	Inverse variance weighted	8	0.060	0.046	1.983E-01	0.903	cis	Severe COVID-19
17460_51	Mx1	Interferon-induced GTP-binding protein Mx1	P20591	MX1	chr21:41420020	Inverse variance weighted	4	-0.110	0.086	1.994E-01	0.904	cis	Severe COVID-19
9796_4	CEL	Bile salt-activated lipase	P19835	CEL	chr9:133061981	Inverse variance weighted	5	-0.092	0.072	1.996E-01	0.904	cis	Severe COVID-19
7856_51	F151A	Protein FAM151A	Q8WW52	FAM151A	chr1:54623556	Inverse variance weighted	5	-0.071	0.055	2.000E-01	0.904	cis	Severe COVID-19
5103_30	MO2R1:ECD	Cell surface glycoprotein CD200 receptor 1:Isoform 4, Extracellular Domain	Q8TD46	CD200R1	chr3:112975103	Wald ratio	1	0.170	0.133	2.011E-01	0.904	cis	Severe COVID-19
5463_22	GAS1	Growth arrest-specific protein 1	P54826	GAS1	chr9:86947506	Inverse variance weighted	2	-0.167	0.131	2.012E-01	0.904	cis	Severe COVID-19
12486_8	GLRX2	Glutaredoxin-2, mitochondrial	Q9NS18	GLRX2	chr1:193106114	Inverse variance weighted	3	0.121	0.095	2.037E-01	0.913	cis	Severe COVID-19
7192_37	CRF2-12	Interferon lambda receptor 1	Q8IU57	IFNLR1	chr1:24187959	Inverse variance weighted	3	-0.112	0.089	2.054E-01	0.918	cis	Severe COVID-19
3196_6	HPLN1	Hyaluronan and proteoglycan link protein 1	P10915	HAPLN1	chr5:83720855	Inverse variance weighted	3	-0.145	0.114	2.063E-01	0.919	cis	Severe COVID-19
3311_27	FCG3B	Low affinity immunoglobulin gamma Fc region receptor III-B	O75015	FCGR3B	chr1:161631963	Wald ratio	1	-0.407	0.323	2.069E-01	0.919	cis	Severe COVID-19
9002_36	SPA11	Serpin A11	Q86U17	SERPINA11	chr14:94452800	Inverse variance weighted	5	-0.127	0.101	2.081E-01	0.919	cis	Severe COVID-19
15417_3	Maspin	Serpin B5	P36952	SERPINB5	chr18:63476958	Wald ratio	1	-0.147	0.117	2.085E-01	0.919	cis	Severe COVID-19
15526_33	GSHB	Glutathione synthetase	P48637	GSS	chr20:34956027	Inverse variance weighted	2	0.134	0.106	2.089E-01	0.919	cis	Severe COVID-19
13748_4	MCP-2	C-C motif chemokine 8	P80075	CCL8	chr17:34319435	Inverse variance weighted	7	0.061	0.049	2.096E-01	0.919	cis	Severe COVID-19
8778_3	Noggin	Noggin	Q13253	NOG	chr17:56593699	Inverse variance weighted	5	0.079	0.063	2.098E-01	0.919	cis	Severe COVID-19
9385_4	GAA	Lysosomal alpha-glucosidase	P10253	GAA	chr17:80101556	Inverse variance weighted	6	-0.115	0.091	2.101E-01	0.919	cis	Severe COVID-19
12517_52	TFAR19	Programmed cell death protein 5	O14737	PDCD5	chr19:32581190	Inverse variance weighted	4	-0.090	0.072	2.110E-01	0.919	cis	Severe COVID-19
3194_36	GPVI	Platelet glycoprotein VI	Q9HCN6	GP6	chr19:55038264	Inverse variance weighted	2	0.071	0.057	2.110E-01	0.919	cis	Severe COVID-19
15441_6	SAP3	Ganglioside GM2 activator	P17900	GM2A	chr5:151212150	Inverse variance weighted	3	-0.064	0.051	2.119E-01	0.919	cis	Severe COVID-19

11098_1	PDXK	Pyridoxal kinase	O00764	PDXK	chr21:43719094	Inverse variance weighted	5	-0.140	0.112	2.124E-01	0.919	cis	Severe COVID-19
9341_1	PDGFD	Platelet-derived growth factor D	Q9GZP0	PDGFD	chr11:104164379	Inverse variance weighted	3	-0.194	0.156	2.130E-01	0.919	cis	Severe COVID-19
11649_3	SMAP1	Stromal membrane-associated protein 1	Q8IYB5	SMAP1	chr6:70667776	Inverse variance weighted	4	-0.058	0.047	2.134E-01	0.919	cis	Severe COVID-19
15636_49	SORC1	VPS10 domain-containing receptor SorCS1	Q8WY21	SORCS1	chr10:107164706	Wald ratio	1	-0.287	0.231	2.142E-01	0.921	cis	Severe COVID-19
17513_11	ANX11	Annexin A11	P50995	ANXA11	chr10:80205572	Inverse variance weighted	3	0.149	0.120	2.153E-01	0.923	cis	Severe COVID-19
15367_38	LPLC1	BPI fold-containing family B member 1	Q8TDL5	BPIFB1	chr20:33273480	Inverse variance weighted	7	0.050	0.040	2.158E-01	0.923	cis	Severe COVID-19
18280_29	DTD2	Putative D-tyrosyl-tRNA(Tyr) deacylase 2	Q96FN9	DTD2	chr14:31457506	Inverse variance weighted	2	-0.180	0.146	2.168E-01	0.925	cis	Severe COVID-19
17738_7	CRNN	Cornulin	Q9UBG3	CRNN	chr1:152414263	Wald ratio	1	0.138	0.112	2.183E-01	0.929	cis	Severe COVID-19
13704_5	HMCS2	Hydroxymethylglutaryl-CoA synthase, mitochondrial	P54868	HMGCS2	chr1:119768905	Wald ratio	1	-0.486	0.396	2.202E-01	0.930	cis	Severe COVID-19
9931_20	Keratin-1	Keratin, type II cytoskeletal 1	P04264	KRT1	chr12:52680407	Wald ratio	1	-0.564	0.461	2.205E-01	0.930	cis	Severe COVID-19
11493_169	DYL2	Dynein light chain 2, cytoplasmic	Q96FJ2	DYNLL2	chr17:58083419	Wald ratio	1	-0.357	0.292	2.212E-01	0.930	cis	Severe COVID-19
10015_119	KCAB2	Voltage-gated potassium channel subunit beta-2	Q13303	KCNAB2	chr1:5990927	Inverse variance weighted	2	-0.238	0.195	2.214E-01	0.930	cis	Severe COVID-19
12457_10	MTND	1,2-dihydroxy-3-keto-5-methylthiopentene dioxygenase	Q9BV57	ADI1	chr2:3519531	Wald ratio	1	0.321	0.263	2.218E-01	0.930	cis	Severe COVID-19
12612_37	PSB1	Proteasome subunit beta type-1	P20618	PSMB1	chr6:170553307	Inverse variance weighted	2	0.058	0.047	2.219E-01	0.930	cis	Severe COVID-19
19602_36	jun-D	Transcription factor jun-D	P17535	JUND	chr19:18281622	Wald ratio	1	-0.367	0.301	2.237E-01	0.935	cis	Severe COVID-19
5586_66	MINP1	Multiple inositol polyphosphate phosphatase 1	Q9UNW1	MINPP1	chr10:87504875	Wald ratio	1	0.296	0.243	2.241E-01	0.935	cis	Severe COVID-19
10672_75	SP-B	Pulmonary surfactant-associated protein B	P07988	SFTPB	chr2:85668741	Inverse variance weighted	4	0.166	0.137	2.265E-01	0.940	cis	Severe COVID-19
4151_6	Plasminogen	Plasminogen	P00747	PLG	chr6:160702194	Inverse variance weighted	3	-0.172	0.142	2.265E-01	0.940	cis	Severe COVID-19
5837_49	LIF sR	Leukemia inhibitory factor receptor	P42702	LIFR	chr5:38608354	Inverse variance weighted	4	0.111	0.092	2.275E-01	0.940	cis	Severe COVID-19
3600_2	Chitotriosidase-1	Chitotriosidase-1	Q13231	CHIT1	chr1:203273641	Inverse variance weighted	6	0.037	0.031	2.286E-01	0.940	cis	Severe COVID-19
6713_4	LRP11	Low-density lipoprotein receptor-related protein 11	Q86VZ4	LRP11	chr6:149864359	Inverse variance weighted	8	0.060	0.050	2.301E-01	0.940	cis	Severe COVID-19
10372_18	STAT6	Signal transducer and activator of transcription 6	P42226	STAT6	chr12:57132139	Inverse variance weighted	2	0.126	0.105	2.302E-01	0.940	cis	Severe COVID-19
9886_28	XRCC4	DNA repair protein XRCC4	Q13426	XRCC4	chr5:83077498	Wald ratio	1	0.176	0.147	2.308E-01	0.940	cis	Severe COVID-19
3324_51	LY9	T-lymphocyte surface antigen Ly-9	Q9HBG7	LY9	chr1:160796074	Inverse variance weighted	5	0.101	0.084	2.309E-01	0.940	cis	Severe COVID-19
8235_48	SCG1	Secretogranin-1	P05060	CHGB	chr20:5911510	Wald ratio	1	0.230	0.192	2.312E-01	0.940	cis	Severe COVID-19
16919_1	ACBP	Acyl-CoA-binding protein	P07108	DBI	chr2:119366924	Inverse variance weighted	2	0.101	0.084	2.315E-01	0.940	cis	Severe COVID-19
12727_7	FPRP	Prostaglandin F2 receptor negative regulator	Q9P2B2	PTGFRN	chr1:116909916	Inverse variance weighted	8	0.056	0.047	2.320E-01	0.940	cis	Severe COVID-19
12684_5	ADSV	Adseverin	Q9Y6U3	SCIN	chr7:12570577	Wald ratio	1	0.273	0.228	2.323E-01	0.940	cis	Severe COVID-19
5487_7	SLAF7	SLAM family member 7	Q9NQ25	SLAMF7	chr1:160739057	Inverse variance weighted	7	0.037	0.031	2.326E-01	0.940	cis	Severe COVID-19
12859_33	PECI	Enoyl-CoA delta isomerase 2, mitochondrial	O75521	ECI2	chr6:4135597	Inverse variance weighted	3	-0.075	0.063	2.334E-01	0.941	cis	Severe COVID-19
14273_19	PPCE	Prolyl endopeptidase	P48147	PREP	chr6:105454062	Inverse variance weighted	2	-0.108	0.091	2.348E-01	0.943	cis	Severe COVID-19
8458_111	a-Synuclein	Alpha-synuclein	P37840	SNCA	chr4:89838315	Wald ratio	1	0.476	0.402	2.357E-01	0.943	cis	Severe COVID-19
17698_15	WBP2	WW domain-binding protein 2	Q969T9	WBP2	chr17:75856507	Wald ratio	1	-0.457	0.386	2.360E-01	0.943	cis	Severe COVID-19
10561_5	PGRP-I-alpha	Peptidoglycan recognition protein 3	Q96LB9	PGLYRP3	chr1:153312952	Wald ratio	1	-0.362	0.306	2.362E-01	0.943	cis	Severe COVID-19
2742_68	Siglec-7	Sialic acid-binding Ig-like lectin 7	Q9Y286	SIGLEC7	chr19:51142299	Wald ratio	1	0.171	0.145	2.387E-01	0.948	cis	Severe COVID-19
19187_21	STABP	STAM-binding protein	O95630	STAMBP	chr2:73828916	Inverse variance weighted	2	0.241	0.204	2.394E-01	0.948	cis	Severe COVID-19
15370_5	BOLA1	BolA-like protein 1	Q9Y3E2	BOLA1	chr1:149887890	Inverse variance weighted	2	-0.061	0.052	2.395E-01	0.948	cis	Severe COVID-19
3459_49	PDGF Rb	Platelet-derived growth factor receptor beta	P09619	PDGFRB	chr5:150155872	Inverse variance weighted	11	-0.019	0.017	2.397E-01	0.948	cis	Severe COVID-19
8325_37	ADH4	Alcohol dehydrogenase 4	P08319	ADH4	chr4:99157792	Wald ratio	1	-0.218	0.186	2.405E-01	0.948	cis	Severe COVID-19
5018_68	Peroxiredoxin-6	Peroxiredoxin-6	P30041	PRDX6	chr1:173477330	Wald ratio	1	0.295	0.252	2.426E-01	0.948	cis	Severe COVID-19
3518_54	TAFI	Carboxypeptidase B2	Q96IY4	CPB2	chr13:46105033	Inverse variance weighted	5	0.052	0.045	2.439E-01	0.948	cis	Severe COVID-19
13969_24	IMA7	Importin subunit alpha-7	O60684	KPNA6	chr1:32108056	Wald ratio	1	0.302	0.260	2.443E-01	0.948	cis	Severe COVID-19
9183_7	IFN-a/b R1	Interferon alpha/beta receptor 1	P17181	IFNAR1	chr21:33324387	Inverse variance weighted	7	0.046	0.040	2.450E-01	0.948	cis	Severe COVID-19
14101_2	CNTFR alpha	Ciliary neurotrophic factor receptor subunit alpha	P26992	CNTFR	chr9:34590140	Inverse variance weighted	2	-0.127	0.110	2.455E-01	0.948	cis	Severe COVID-19
11333_82	RHG25	Rho GTPase-activating protein 25	P42331	ARHGAP25	chr2:68679601	Wald ratio	1	0.119	0.103	2.455E-01	0.948	cis	Severe COVID-19
14106_46	GDP/GTP exchange protein	Rap1 GTPase-GDP dissociation stimulator 1	P52306	RAP1GDS1	chr4:98261384	Wald ratio	1	0.347	0.299	2.457E-01	0.948	cis	Severe COVID-19
6947_4	SIA10	Type 2 lactosamine alpha-2,3-sialyltransferase	Q9Y274	ST3GAL6	chr3:98732236	Inverse variance weighted	9	-0.028	0.024	2.461E-01	0.948	cis	Severe COVID-19
6507_16	NCAM2	Neural cell adhesion molecule 2	O15394	NCAM2	chr21:20998409	Inverse variance weighted	6	0.038	0.033	2.477E-01	0.948	cis	Severe COVID-19
12975_11	Keratin 20	Keratin, type I cytoskeletal 20	P35900	KRT20	chr17:40885242	Inverse variance weighted	2	-0.244	0.211	2.479E-01	0.948	cis	Severe COVID-19
9231_23	IMPA3	Inositol monophosphatase 3	Q9NX62	BPNT2	chr8:56993867	Inverse variance weighted	6	-0.048	0.041	2.490E-01	0.948	cis	Severe COVID-19
8065_245	PATE4	Prostate and testis expressed protein 4	P0C8F1	PATE4	chr11:125833316	Wald ratio	1	-0.140	0.122	2.492E-01	0.948	cis	Severe COVID-19
4913_78	HCC-4	C-C motif chemokine 16	O15467	CCL16	chr17:35981497	Inverse variance weighted	8	-0.034	0.029	2.493E-01	0.948	cis	Severe COVID-19
2982_82	Galectin-4	Galectin-4	P56470	LGALS4	chr19:38812945	Wald ratio	1	-0.312	0.271	2.495E-01	0.948	cis	Severe COVID-19
4437_56	ENTP5	Ectonucleoside triphosphate diphosphohydrolase 5	O75356	ENTPD5	chr14:74019399	Inverse variance weighted	8	0.037	0.032	2.504E-01	0.948	cis	Severe COVID-19
6467_65	KTEL1	Protein O-glucosyltransferase 1	Q8NBL1	POGLUT1	chr3:119468963	Inverse variance weighted	3	-0.096	0.084	2.516E-01	0.948	cis	Severe COVID-19
3024_18	a2-Antiplasmin	Alpha-2-antiplasmin	P08697	SERPINF2	chr17:1742836	Inverse variance weighted	3	0.128	0.112	2.516E-01	0.948	cis	Severe COVID-19
11643_73	DTX3L	E3 ubiquitin-protein ligase DTX3L	Q8TDB6	DTX3L	chr3:122564338	Wald ratio	1	-0.401	0.349	2.517E-01	0.948	cis	Severe COVID-19
14158_17	Annexin V	Annexin A5	P08758	ANXA5	chr4:121696995	Inverse variance weighted	2	0.123	0.107	2.520E-01	0.948	cis	Severe COVID-19
16914_104	sCD14	Monocyte differentiation antigen CD14, soluble	P08571	CD14	chr5:140633700	Inverse variance weighted	4	-0.145	0.127	2.527E-01	0.948	cis	Severe COVID-19
16900_29	MDGA1	MAM domain-containing glycosylphosphatidylinositol anchor protein 1	Q8NFP4	MDGA1	chr6:37699306	Inverse variance weighted	8	-0.030	0.026	2.529E-01	0.948	cis	Severe COVID-19
6626_81	CHSTC	Carbohydrate sulfotransferase 12	Q9NRB3	CHST12	chr7:2403588	Inverse variance weighted	2	-0.093	0.081	2.544E-01	0.948	cis	Severe COVID-19
15584_9	FHR2	Complement factor H-related protein 2	P36980	CFHR2	chr1:196943738	Inverse variance weighted	4	0.049	0.043	2.547E-01	0.948	cis	Severe COVID-19
11543_84	LIMA1	LIM domain and actin-binding protein 1	Q9UHB6	LIMA1	chr12:50283520	Wald ratio	1	0.267	0.235	2.552E-01	0.948	cis	Severe COVID-19
13988_67	NMRL1	NmrA-like family domain-containing protein 1	Q9HBL8	NMRAL1	chr16:4495763	Inverse variance weighted	11	-0.045	0.039	2.554E-01	0.948	cis	Severe COVID-19
19606_28	ihh	Indian hedgehog protein	Q14623	IHH	chr2:219060921	Inverse variance weighted	3	-0.228	0.201	2.561E-01	0.948	cis	Severe COVID-19
3617_80	HGFA	Hepatocyte growth factor activator	Q04756	HGFAC	chr4:3441968	Inverse variance weighted	11	-0.035	0.031	2.571E-01	0.948	cis	Severe COVID-19
9790_28	BR serine/threonine kinase 2	Serine/threonine-protein kinase BRSK2	Q8IWQ3	BRSK2	chr11:1389899	Inverse variance weighted	2	-0.104	0.092	2.577E-01	0.948	cis	Severe COVID-19
17320_19	ADPPT	L-aminoadipate-semialdehyde dehydrogenase-phosphopantetheinyl transferase	Q9NRN7	AASDHPPT	chr11:106075501	Wald ratio	1	-0.392	0.347	2.579E-01	0.948	cis	Severe COVID-19
16818_200	CDCP1	CUB domain-containing protein 1	Q9H5V8	CDCP1	chr3:45146422	Inverse variance weighted	3	-0.116	0.103	2.598E-01	0.948	cis	Severe COVID-19
3807_1	FGF23	Fibroblast growth factor 23	Q9GZV9	FGF23	chr12:4379712	Wald ratio	1	-0.328	0.292	2.606E-01	0.948	cis	Severe COVID-19
19561_216	PLXD1	Plexin-D1	Q9Y4D7	PLXND1	chr3:129606676	Inverse variance weighted	4	0.086	0.076	2.620E-01	0.948	cis	Severe COVID-19
16753_46	CO6A2	Collagen alpha-2(VI) chain	P12110	COL6A2	chr21:46098112	Inverse variance weighted	4	-0.098	0.088	2.641E-01	0.948	cis	Severe COVID-19
4407_10	MSP	Hepatocyte growth factor-like protein	P26927	MST1	chr3:49689501	Inverse variance weighted	4	0.020	0.018	2.649E-01	0.948	cis	Severe COVID-19
17329_2	BDH2	3-hydroxybutyrate dehydrogenase type 2	Q9BUT1	BDH2	chr4:103099870	Inverse variance weighted	2	0.100	0.090	2.654E-01	0.948	cis	Severe COVID-19
13405_61	ISK2	Serine protease inhibitor Kazal-type 2	P20155	SPINK2	chr4:56821742	Inverse variance weighted	5	-0.046	0.042	2.657E-01	0.948	cis	Severe COVID-19
13044_5	TS101	Tumor susceptibility gene 101 protein	Q99816	TSG101	chr11:18526951	Inverse variance weighted	2	-0.281	0.253	2.658E-01	0.948	cis	Severe COVID-19
10512_13	IL3RB:ECD	Cytokine receptor common subunit beta:Extracellular domain	P32927	CSF2RB	chr22:36913628	Inverse variance weighted	7	-0.028	0.025	2.669E-01	0.948	cis	Severe COVID-19
4978_54	DBNL	Drebrin-like protein	Q9UJU6	DBNL	chr7:44044640	Wald ratio	1	0.264	0.238	2.672E-01	0.948	cis	Severe COVID-19

17357_33	SPCS	O-phosphoseryl-tRNA(Sec) selenium transferase	Q9HD40	SEPSECS	chr4:25160550	Wald ratio	1	-0.237	0.214	2.680E-01	0.948	cis	Severe COVID-19
10781_19	CLC4G	C-type lectin domain family 4 member G	Q6UXB4	CLEC4G	chr19:7733906	Wald ratio	1	0.542	0.490	2.690E-01	0.948	cis	Severe COVID-19
17408_2	PMM1	Phosphomannomutase 1	Q92871	PMM1	chr22:41589871	Wald ratio	1	0.320	0.290	2.692E-01	0.948	cis	Severe COVID-19
2837_3	Met	Hepatocyte growth factor receptor	P08581	MET	chr7:116672196	Inverse variance weighted	4	0.101	0.091	2.693E-01	0.948	cis	Severe COVID-19
9294_45	MFAP2	Microfibrillar-associated protein 2	P55001	MFAP2	chr1:16980632	Inverse variance weighted	2	0.269	0.244	2.704E-01	0.948	cis	Severe COVID-19
15610_72	LAP	Cytosol aminopeptidase	P28838	LAP3	chr4:17577198	Wald ratio	1	0.333	0.303	2.705E-01	0.948	cis	Severe COVID-19
6060_2	PIP	Prolactin-inducible protein	P12273	PIP	chr7:143132077	Inverse variance weighted	5	-0.063	0.057	2.712E-01	0.948	cis	Severe COVID-19
5676_54	ASIP	Agouti-signaling protein	P42127	ASIP	chr20:34194569	Wald ratio	1	0.045	0.041	2.716E-01	0.948	cis	Severe COVID-19
4874_3	Angiogenin	Angiogenin	P03950	ANG	chr14:20684177	Inverse variance weighted	5	0.069	0.063	2.723E-01	0.948	cis	Severe COVID-19
4992_49	GRN	Granulins	P28799	GRN	chr17:44345246	Inverse variance weighted	3	-0.124	0.113	2.727E-01	0.948	cis	Severe COVID-19
9870_17	SYWC	Tryptophan--tRNA ligase, cytoplasmic	P23381	WARS1	chr14:100376805	Inverse variance weighted	2	-0.084	0.076	2.731E-01	0.948	cis	Severe COVID-19
8345_27	GPX7	Glutathione peroxidase 7	Q96SL4	GPX7	chr1:52602371	Inverse variance weighted	7	0.036	0.033	2.739E-01	0.948	cis	Severe COVID-19
9384_17	Cathelicidin peptide	Cathelicidin antimicrobial peptide	P49913	CAMP	chr3:48223347	Wald ratio	1	0.229	0.209	2.742E-01	0.948	cis	Severe COVID-19
17383_4	STAR5	StAR-related lipid transfer protein 5	Q9NSY2	STARD5	chr15:81324183	Inverse variance weighted	6	-0.141	0.129	2.748E-01	0.948	cis	Severe COVID-19
3043_49	ON	SPARC	P09486	SPARC	chr5:151686975	Wald ratio	1	0.316	0.290	2.754E-01	0.948	cis	Severe COVID-19
18188_12	GATM	Glycine amidinotransferase, mitochondrial	P50440	GATM	chr15:45402327	Wald ratio	1	-0.096	0.088	2.755E-01	0.948	cis	Severe COVID-19
12382_2	DDX58	Probable ATP-dependent RNA helicase DDX58	O95786	DDX58	chr9:32526208	Inverse variance weighted	2	0.110	0.101	2.757E-01	0.948	cis	Severe COVID-19
17396_23	ADH1A	Alcohol dehydrogenase 1A	P07327	ADH1A	chr4:99291003	Wald ratio	1	-0.155	0.142	2.760E-01	0.948	cis	Severe COVID-19
6086_15	CRDL2	Chordin-like protein 2	Q6WN34	CHRDL2	chr11:74731426	Inverse variance weighted	5	0.062	0.057	2.766E-01	0.948	cis	Severe COVID-19
3216_2	PIGR	Polymeric immunoglobulin receptor	P01833	PIGR	chr1:206946466	Wald ratio	1	0.244	0.224	2.767E-01	0.948	cis	Severe COVID-19
5092_51	JAG1:ECD	Protein jagged-1:Extracellular domain	P78504	JAG1	chr20:10673999	Inverse variance weighted	2	-0.270	0.248	2.774E-01	0.948	cis	Severe COVID-19
16620_26	LY75	Lymphocyte antigen 75	O60449	LY75	chr2:159904756	Inverse variance weighted	7	-0.038	0.035	2.787E-01	0.948	cis	Severe COVID-19
9297_12	B3GN8	UDP-GlcNAc:betaGal beta-1,3-N-acetylglucosaminyltransferase 8	Q7Z7M8	B3GNT8	chr19:41428730	Inverse variance weighted	6	-0.033	0.030	2.793E-01	0.948	cis	Severe COVID-19
6415_90	CPN2	Carboxypeptidase N subunit 2	P22792	CPN2	chr3:194351328	Inverse variance weighted	6	0.062	0.058	2.802E-01	0.948	cis	Severe COVID-19
18295_102	GRHPR	Glyoxylate reductase/hydroxypyruvate reductase	Q9UBQ7	GRHPR	chr9:37422666	Inverse variance weighted	3	-0.058	0.054	2.806E-01	0.948	cis	Severe COVID-19
8245_27	sICAM-5	Intercellular adhesion molecule 5	Q9UMF0	ICAM5	chr19:10289952	Inverse variance weighted	3	0.180	0.167	2.806E-01	0.948	cis	Severe COVID-19
17224_12	MIME	Mimecan	P20774	OGN	chr9:92404696	Inverse variance weighted	6	0.084	0.078	2.807E-01	0.948	cis	Severe COVID-19
13133_73	LTBP4	Latent-transforming growth factor beta-binding protein 4	Q8N2S1	LTBP4	chr19:40592883	Inverse variance weighted	2	-0.275	0.255	2.809E-01	0.948	cis	Severe COVID-19
5621_64	THSD1	Thrombospondin type-1 domain-containing protein 1	Q9NS62	THSD1	chr13:52416373	Inverse variance weighted	4	0.070	0.065	2.809E-01	0.948	cis	Severe COVID-19
3280_49	Aggrecan	Aggrecan core protein	P16112	ACAN	chr15:88803436	Inverse variance weighted	2	-0.144	0.134	2.810E-01	0.948	cis	Severe COVID-19
5349_69	DLL1	Delta-like protein 1	O00548	DLL1	chr6:170306565	Inverse variance weighted	2	0.137	0.127	2.819E-01	0.948	cis	Severe COVID-19
4435_66	ENPP7	Ectonucleotide pyrophosphatase/phosphodiesterase family member 7	Q6UWV6	ENPP7	chr17:79730943	Inverse variance weighted	9	0.042	0.039	2.826E-01	0.948	cis	Severe COVID-19
8893_29	PARP:region 1	Poly [ADP-ribose] polymerase 1:region 1	P09874	PARP1	chr1:226408154	Wald ratio	1	0.320	0.298	2.829E-01	0.948	cis	Severe COVID-19
12498_12	TX1B3	Tax1-binding protein 3	O14907	TAX1BP3	chr17:3668679	Wald ratio	1	0.179	0.167	2.830E-01	0.948	cis	Severe COVID-19
2966_65	SCGF-beta	Stem cell growth factor-beta	Q9Y240	CLEC11A	chr19:50723364	Wald ratio	1	0.401	0.374	2.837E-01	0.948	cis	Severe COVID-19
11302_237	TENR	Tenascin-R	Q92752	TNR	chr1:175743616	Wald ratio	1	0.125	0.116	2.838E-01	0.948	cis	Severe COVID-19
14618_26	ECOP	Vesicular, overexpressed in cancer, prosurvival protein 1	Q96AW1	VOPP1	chr7:55572988	Inverse variance weighted	2	-0.141	0.131	2.846E-01	0.948	cis	Severe COVID-19
6416_8	GKN2	Gastrokine-2	Q86XP6	GKN2	chr2:68952893	Inverse variance weighted	4	0.050	0.047	2.852E-01	0.948	cis	Severe COVID-19
11278_4	COL11A2	Collagen alpha-2(XI) chain	P13942	COL11A2	chr6:33192499	Inverse variance weighted	4	0.072	0.068	2.854E-01	0.948	cis	Severe COVID-19
5400_52	sLeptin R	Leptin receptor, soluble	P48357	LEPR	chr1:65420652	Inverse variance weighted	10	0.031	0.029	2.855E-01	0.948	cis	Severe COVID-19
18401_18	ALKB3	Alpha-ketoglutarate-dependent dioxygenase alkB homolog 3	Q96Q83	ALKBH3	chr11:43880811	Inverse variance weighted	3	-0.076	0.072	2.870E-01	0.948	cis	Severe COVID-19
5963_9	Dermokine	Dermokine	Q6E0U4	DMKN	chr19:35513658	Wald ratio	1	-0.363	0.341	2.873E-01	0.948	cis	Severe COVID-19
7140_1	ELA2A	Chymotrypsin-like elastase family member 2A	P08217	CELA2A	chr1:15456728	Wald ratio	1	0.166	0.156	2.874E-01	0.948	cis	Severe COVID-19
8275_31	PEAR1:ECD	Platelet endothelial aggregation receptor 1:Extracellular domain	Q5VY43	PEAR1	chr1:156893698	Inverse variance weighted	4	-0.076	0.071	2.880E-01	0.948	cis	Severe COVID-19
3607_71	DKK3	Dickkopf-related protein 3	Q9UBP4	DKK3	chr11:12009769	Inverse variance weighted	4	-0.064	0.060	2.882E-01	0.948	cis	Severe COVID-19
5658_64	coagulation factor XIII B	Coagulation factor XIII B chain	P05160	F13B	chr1:197067260	Wald ratio	1	-0.157	0.148	2.889E-01	0.948	cis	Severe COVID-19
4968_50	CAPG	Macrophage-capping protein	P40121	CAPG	chr2:85418432	Inverse variance weighted	4	0.067	0.063	2.893E-01	0.948	cis	Severe COVID-19
19376_74	NNMT	Nicotinamide N-methyltransferase	P40261	NNMT	chr11:114257787	Wald ratio	1	0.262	0.247	2.896E-01	0.948	cis	Severe COVID-19
3605_77	MASP3:Light	Mannan-binding lectin serine protease 1:Mannan-binding lectin serine protease 1 light chain	P48740	MASP1	chr3:187291980	Wald ratio	1	0.200	0.189	2.897E-01	0.948	cis	Severe COVID-19
5491_12	Testican-2	Testican-2	Q92563	SPOCK2	chr10:72089032	Wald ratio	1	-0.081	0.077	2.933E-01	0.956	cis	Severe COVID-19
5090_49	ILT-2	Leukocyte immunoglobulin-like receptor subfamily B member 1	Q8NHL6	LILRB1	chr19:54617158	Inverse variance weighted	6	0.070	0.066	2.934E-01	0.956	cis	Severe COVID-19
5121_3	SEM6B	Semaphorin-6B	Q9H3T3	SEMA6B	chr19:4581776	Inverse variance weighted	2	0.237	0.227	2.949E-01	0.958	cis	Severe COVID-19
12396_19	HIBCH	3-hydroxyisobutyryl-CoA hydrolase, mitochondrial	Q6NVY1	HIBCH	chr2:190344193	Inverse variance weighted	5	0.043	0.042	3.000E-01	0.958	cis	Severe COVID-19
5202_4	PPID	Peptidyl-prolyl cis-trans isomerase D	Q08752	PPID	chr4:158723396	Inverse variance weighted	2	-0.060	0.058	3.007E-01	0.958	cis	Severe COVID-19
13578_98	ABLM3	Actin-binding LIM protein 3	O94929	ABLIM3	chr5:149141483	Wald ratio	1	-0.149	0.144	3.010E-01	0.958	cis	Severe COVID-19
6558_5	COL10	Collectin-10	Q9Y6Z7	COLEC10	chr8:118995452	Wald ratio	1	0.260	0.252	3.011E-01	0.958	cis	Severe COVID-19
9233_71	TFPI -2	Tissue factor pathway inhibitor 2	P48307	TFPI2	chr7:93890753	Wald ratio	1	-0.324	0.314	3.019E-01	0.958	cis	Severe COVID-19
8376_25	LSHB	Lutropin subunit beta	P01229	LHB	chr19:49017091	Inverse variance weighted	4	0.077	0.074	3.023E-01	0.958	cis	Severe COVID-19
13632_10	Zyxin	Zyxin	Q15942	ZYX	chr7:143381295	Wald ratio	1	-0.415	0.402	3.024E-01	0.958	cis	Severe COVID-19
16300_4	TREM2	Triggering receptor expressed on myeloid cells 2	Q9NZC2	TREM2	chr6:41163186	Inverse variance weighted	7	0.042	0.041	3.046E-01	0.958	cis	Severe COVID-19
9126_171	NT5D3	5'-nucleotidase domain-containing protein 3	Q86UY8	NT5DC3	chr12:103841234	Inverse variance weighted	2	0.072	0.071	3.053E-01	0.958	cis	Severe COVID-19
18321_38	MTDC	Bifunctional methylenetetrahydrofolate dehydrogenase/cyclohydrolase, mitochondrial	P13995	MTHFD2	chr2:74186172	Inverse variance weighted	2	0.117	0.114	3.055E-01	0.958	cis	Severe COVID-19
3212_30	ASAH2	Neutral ceramidase	Q9NR71	ASAH2	chr10:50279720	Inverse variance weighted	6	-0.036	0.036	3.061E-01	0.958	cis	Severe COVID-19
2890_59	CCL28	C-C motif chemokine 28	Q9NRJ3	CCL28	chr5:43412391	Wald ratio	1	-0.396	0.387	3.064E-01	0.958	cis	Severe COVID-19
3651_50	VEGF sR2	Vascular endothelial growth factor receptor 2	P35968	KDR	chr4:55125595	Inverse variance weighted	3	-0.040	0.039	3.072E-01	0.958	cis	Severe COVID-19
4546_27	EMR2	Adhesion G protein-coupled receptor E2	Q9UHX3	ADGRE2	chr19:14778560	Inverse variance weighted	5	0.053	0.052	3.080E-01	0.958	cis	Severe COVID-19
6899_37	PHS2	Pterin-4-alpha-carbinolamine dehydratase 2	Q9H0N5	PCBD2	chr5:134905120	Wald ratio	1	0.549	0.538	3.081E-01	0.958	cis	Severe COVID-19
4240_31	M2-PK	Pyruvate kinase PKM	P14618	PKM	chr15:72231819	Wald ratio	1	0.212	0.208	3.083E-01	0.958	cis	Severe COVID-19
3290_50	CD109	CD109 antigen	Q6YHK3	CD109	chr6:73695785	Inverse variance weighted	7	0.036	0.035	3.091E-01	0.958	cis	Severe COVID-19
3391_10	PK3CG	Phosphatidylinositol 4,5-bisphosphate 3-kinase catalytic subunit gamma isoform	P48736	PIK3CG	chr7:106865278	Wald ratio	1	-0.293	0.289	3.094E-01	0.958	cis	Severe COVID-19
19277_4	KAT	Thiosulfate sulfurtransferase/rhodanese-like domain-containing protein 1	Q8NFU3	TSTD1	chr1:161038977	Inverse variance weighted	2	0.098	0.096	3.097E-01	0.958	cis	Severe COVID-19
19560_23	PLXA4	Plexin-A4	Q9HCM2	PLXNA4	chr7:132648688	Wald ratio	1	-0.190	0.187	3.097E-01	0.958	cis	Severe COVID-19
11347_9	Transaldolase	Transaldolase	P37837	TALDO1	chr11:747415	Inverse variance weighted	2	-0.143	0.141	3.099E-01	0.958	cis	Severe COVID-19
9557_5	MANS1	MANSC domain-containing protein 1	Q9H8J5	MANSC1	chr12:12350242	Inverse variance weighted	3	0.081	0.079	3.104E-01	0.958	cis	Severe COVID-19
2201_17	Endostatin	Endostatin	P39060	COL18A1	chr21:45405165	Inverse variance weighted	4	-0.089	0.088	3.106E-01	0.958	cis	Severe COVID-19
11356_19	DGC14	Protein DGCR14	Q96DF8	ESS2	chr22:19144684	Wald ratio	1	0.144	0.142	3.107E-01	0.958	cis	Severe COVID-19
13132_14	SEM5A	Semaphorin-5A	Q13591	SEMA5A	chr5:9546075	Inverse variance weighted	10	-0.019	0.019	3.108E-01	0.958	cis	Severe COVID-19
4126_22	BPI	Bactericidal permeability-increasing protein	P17213	BPI	chr20:38304150	Inverse variance weighted	9	0.026	0.025	3.110E-01	0.958	cis	Severe COVID-19

9468_8	Lectin, mannose-binding 2	Vesicular integral-membrane protein VIP36	Q12907	LMAN2	chr5:177351840	Wald ratio	1	0.247	0.244	3.114E-01	0.958	cis	Severe COVID-19
8877_22	F176C:ECD	Protein eva-1 homolog C:Extracellular domain	P58658	EVA1C	chr21:32412006	Wald ratio	1	-0.243	0.241	3.117E-01	0.958	cis	Severe COVID-19
9841_197	Multifunctional protein ADE2	Multifunctional protein ADE2	P22234	PAICS	chr4:56435741	Wald ratio	1	0.178	0.176	3.120E-01	0.958	cis	Severe COVID-19
6715_63	PPBN	Alkaline phosphatase, placental-like	P10696	ALPG	chr2:232406844	Inverse variance weighted	3	0.157	0.156	3.121E-01	0.958	cis	Severe COVID-19
13488_3	ARFP1	Arfaptin-1	P53367	ARFIP1	chr4:152779937	Inverse variance weighted	2	0.091	0.090	3.129E-01	0.958	cis	Severe COVID-19
6544_33	NELL1	Protein kinase C-binding protein NELL1	Q92832	NELL1	chr11:20669551	Inverse variance weighted	8	-0.038	0.037	3.132E-01	0.958	cis	Severe COVID-19
6574_11	FAIM3	Fas apoptotic inhibitory molecule 3	O60667	FCMR	chr1:206923247	Inverse variance weighted	2	-0.220	0.218	3.134E-01	0.958	cis	Severe COVID-19
9172_69	MMP-8	Neutrophil collagenase	P22894	MMP8	chr11:102727050	Wald ratio	1	0.218	0.217	3.139E-01	0.958	cis	Severe COVID-19
3810_50	FGR	Tyrosine-protein kinase Fgr	P09769	FGR	chr1:27635185	Inverse variance weighted	2	0.135	0.134	3.144E-01	0.958	cis	Severe COVID-19
17724_3	WWOX	WW domain-containing oxidoreductase	Q9NZC7	WWOX	chr16:78099400	Wald ratio	1	0.203	0.202	3.153E-01	0.959	cis	Severe COVID-19
12820_1	GRAP	GRB2-related adapter protein	Q13588	GRAP	chr17:19047011	Wald ratio	1	-0.470	0.471	3.181E-01	0.962	cis	Severe COVID-19
13545_97	EIF1A	Probable RNA-binding protein EIF1AD	Q8N9N8	EIF1AD	chr11:66002176	Inverse variance weighted	3	0.193	0.193	3.181E-01	0.962	cis	Severe COVID-19
4771_10	ASM3A	Acid sphingomyelinase-like phosphodiesterase 3a	Q92484	SMPDL3A	chr6:122789049	Inverse variance weighted	3	0.065	0.065	3.186E-01	0.962	cis	Severe COVID-19
9565_6	PAWR	PRKC apoptosis WT1 regulator protein	Q96IZ0	PAWR	chr12:79690964	Wald ratio	1	0.332	0.333	3.187E-01	0.962	cis	Severe COVID-19
7994_41	ERO1B	ERO1-like protein beta	Q86YB8	ERO1B	chr1:236282019	Inverse variance weighted	2	-0.082	0.082	3.199E-01	0.963	cis	Severe COVID-19
10391_1	ANGL3	Angiopoietin-related protein 3	Q9Y5C1	ANGPTL3	chr1:62597520	Inverse variance weighted	7	-0.050	0.050	3.201E-01	0.963	cis	Severe COVID-19
3314_74	GFRa-1	GDNF family receptor alpha-1	P56159	GFRA1	chr10:116276803	Wald ratio	1	0.082	0.083	3.216E-01	0.964	cis	Severe COVID-19
7173_141	T132C:ECD	Transmembrane protein 132C:Extracellular domain	Q8N3T6	TMEM132C	chr12:128267170	Inverse variance weighted	5	0.047	0.047	3.216E-01	0.964	cis	Severe COVID-19
13947_371	NUD12	Peroxisomal NADH pyrophosphatase NUDT12	Q9BQG2	NUDT12	chr5:103562790	Inverse variance weighted	4	0.053	0.054	3.227E-01	0.966	cis	Severe COVID-19
2665_26	BCMA	Tumor necrosis factor receptor superfamily member 17	Q02223	TNFRSF17	chr16:11965210	Wald ratio	1	-0.112	0.114	3.248E-01	0.966	cis	Severe COVID-19
3066_12	Galectin-3	Galectin-3	P17931	LGALS3	chr14:55124110	Inverse variance weighted	4	-0.044	0.045	3.249E-01	0.966	cis	Severe COVID-19
19334_62	TXD12	Thioredoxin domain-containing protein 12	O95881	TXNDC12	chr1:52055191	Wald ratio	1	0.175	0.178	3.249E-01	0.966	cis	Severe COVID-19
2475_1	SCF sR	Mast/stem cell growth factor receptor Kit	P10721	KIT	chr4:54657267	Wald ratio	1	-0.294	0.299	3.250E-01	0.966	cis	Severe COVID-19
7244_16	TM149	IGF-like family receptor 1	Q9H665	IGFLR1	chr19:35742453	Inverse variance weighted	4	-0.030	0.031	3.264E-01	0.966	cis	Severe COVID-19
6525_17	DUSP13	Dual specificity protein phosphatase 13 isoform A	Q6B8I1	DUSP13	chr10:75109221	Inverse variance weighted	2	0.058	0.059	3.266E-01	0.966	cis	Severe COVID-19
11616_9	HSF1	Heat shock factor protein 1	Q00613	HSF1	chr8:144291591	Wald ratio	1	0.329	0.336	3.268E-01	0.966	cis	Severe COVID-19
17783_9	MMAB	Cob(I)yrinic acid a,c-diamide adenosyltransferase, mitochondrial	Q96EY8	MMAB	chr12:109573580	Inverse variance weighted	6	-0.052	0.053	3.281E-01	0.968	cis	Severe COVID-19
15515_2	SAA	Serum amyloid A-1 protein	P0DJI8	SAA1	chr11:18266260	Inverse variance weighted	4	-0.120	0.123	3.292E-01	0.970	cis	Severe COVID-19
3079_62	TIG2	Retinoic acid receptor responder protein 2	Q99969	RARRES2	chr7:150341662	Inverse variance weighted	2	0.079	0.081	3.298E-01	0.970	cis	Severe COVID-19
3296_92	CNTN2	Contactin-2	Q02246	CNTN2	chr1:205042937	Inverse variance weighted	9	0.042	0.043	3.307E-01	0.971	cis	Severe COVID-19
5618_50	FAM3B	Protein FAM3B	P58499	FAM3B	chr21:41304212	Wald ratio	1	0.194	0.200	3.314E-01	0.971	cis	Severe COVID-19
2985_35	Gro-a	Growth-regulated alpha protein	P09341	CXCL1	chr4:73869393	Inverse variance weighted	8	-0.045	0.046	3.324E-01	0.972	cis	Severe COVID-19
15522_2	GAPR1	Golgi-associated plant pathogenesis-related protein 1	Q9H4G4	GLIPR2	chr9:36136536	Inverse variance weighted	2	0.062	0.064	3.336E-01	0.973	cis	Severe COVID-19
17814_8	BPNT1	3'(2'),5'-bisphosphate nucleotidase 1	O95861	BPNT1	chr1:220090462	Wald ratio	1	0.264	0.274	3.343E-01	0.973	cis	Severe COVID-19
10445_20	ApoM	Apolipoprotein M	O95445	APOM	chr6:31652416	Inverse variance weighted	2	0.077	0.079	3.350E-01	0.973	cis	Severe COVID-19
9265_10	GLIP1	Glioma pathogenesis-related protein 1	P48060	GLIPR1	chr12:75480753	Wald ratio	1	-0.162	0.169	3.354E-01	0.973	cis	Severe COVID-19
16060_99	NID2	Nidogen-2	Q14112	NID2	chr14:52069059	Inverse variance weighted	6	0.037	0.039	3.358E-01	0.973	cis	Severe COVID-19
19289_29	DCUP	Uroporphyrinogen decarboxylase	P06132	UROD	chr1:45010950	Inverse variance weighted	3	0.057	0.059	3.366E-01	0.973	cis	Severe COVID-19
6414_8	OAF	Out at first protein homolog	Q86UD1	OAF	chr11:120211032	Inverse variance weighted	5	-0.039	0.041	3.374E-01	0.973	cis	Severe COVID-19
14271_23	RAB6B	Ras-related protein Rab-6B	Q9NRW1	RAB6B	chr3:133895882	Wald ratio	1	-0.072	0.075	3.381E-01	0.973	cis	Severe COVID-19
2851_63	C5a	C5a anaphylatoxin	P01031	C5	chr9:121075195	Inverse variance weighted	2	-0.194	0.203	3.389E-01	0.973	cis	Severe COVID-19
19504_22	KTHY	Thymidylate kinase	P23919	DTYMK	chr2:241686944	Inverse variance weighted	3	0.247	0.258	3.391E-01	0.973	cis	Severe COVID-19
10534_40	PARP:BRCT domain	Poly [ADP-ribose] polymerase 1:BRCA1 C-terminal:BRCA1 C-terminus domian	P09874	PARP1	chr1:226408154	Wald ratio	1	-0.311	0.326	3.398E-01	0.973	cis	Severe COVID-19
15427_35	LOXL3	Lysyl oxidase homolog 3	P58215	LOXL3	chr2:74555690	Wald ratio	1	-0.167	0.175	3.402E-01	0.973	cis	Severe COVID-19
4141_79	IP-10	C-X-C motif chemokine 10	P02778	CXCL10	chr4:76023497	Wald ratio	1	-0.296	0.311	3.412E-01	0.973	cis	Severe COVID-19
19581_15	IGFBP-5	Insulin-like growth factor-binding protein 5	P24593	IGFBP5	chr2:216695549	Wald ratio	1	0.274	0.289	3.436E-01	0.973	cis	Severe COVID-19
12851_5	DPOLM	DNA-directed DNA/RNA polymerase mu	Q9NP87	POLM	chr7:44082530	Inverse variance weighted	3	0.097	0.103	3.469E-01	0.973	cis	Severe COVID-19
6576_1	ART4	Ecto-ADP-ribosyltransferase 4	Q93070	ART4	chr12:14843526	Inverse variance weighted	12	0.029	0.031	3.477E-01	0.973	cis	Severe COVID-19
3060_43	C9	Complement component C9	P02748	C9	chr5:39371324	Inverse variance weighted	5	-0.070	0.074	3.488E-01	0.973	cis	Severe COVID-19
15470_11	Hexosaminidase B	Beta-hexosaminidase subunit beta	P07686	HEXB	chr5:74640023	Inverse variance weighted	4	0.072	0.077	3.492E-01	0.973	cis	Severe COVID-19
17685_9	Apo A-IV	Apolipoprotein A-IV	P06727	APOA4	chr11:116823304	Wald ratio	1	-0.351	0.376	3.501E-01	0.973	cis	Severe COVID-19
10442_1	TM190	Transmembrane protein 190	Q8WZ59	TMEM190	chr19:55376826	Inverse variance weighted	9	-0.025	0.027	3.514E-01	0.973	cis	Severe COVID-19
4961_17	annexin II	Annexin A2	P07355	ANXA2	chr15:60402883	Inverse variance weighted	2	0.179	0.192	3.518E-01	0.973	cis	Severe COVID-19
3332_57	RGM-C	Hemojuvelin	Q6ZVN8	HJV	chr1:146036746	Wald ratio	1	-0.199	0.214	3.527E-01	0.973	cis	Severe COVID-19
11659_31	EPN4	Clathrin interactor 1	Q14677	CLINT1	chr5:157859145	Wald ratio	1	0.346	0.373	3.528E-01	0.973	cis	Severe COVID-19
7019_13	Semaphorin-7A	Semaphorin-7A	O75326	SEMA7A	chr15:74433958	Wald ratio	1	0.171	0.184	3.528E-01	0.973	cis	Severe COVID-19
18181_2	TSSC4	Protein TSSC4	Q9Y5U2	TSSC4	chr11:2400488	Wald ratio	1	-0.478	0.515	3.532E-01	0.973	cis	Severe COVID-19
5355_69	LIGHT	Tumor necrosis factor ligand superfamily member 14	O43557	TNFSF14	chr19:6670588	Wald ratio	1	-0.139	0.150	3.534E-01	0.973	cis	Severe COVID-19
8773_172	EMIL3:region 1	EMILIN-3:region 1	Q9NT22	EMILIN3	chr20:41366818	Inverse variance weighted	5	0.074	0.080	3.540E-01	0.973	cis	Severe COVID-19
9348_1	C1RL1	Complement C1r subcomponent-like protein	Q9NZP8	C1RL	chr12:7109238	Wald ratio	1	0.223	0.240	3.542E-01	0.973	cis	Severe COVID-19
13580_2	Sperm-associated antigen 2	UDP-N-acetylhexosamine pyrophosphorylase	Q16222	UAP1	chr1:162561722	Wald ratio	1	-0.428	0.462	3.542E-01	0.973	cis	Severe COVID-19
16583_8	AP4A	Bis(5'-nucleosyl)-tetraphosphatase [asymmetrical]	P50583	NUDT2	chr9:34329506	Inverse variance weighted	7	0.069	0.075	3.544E-01	0.973	cis	Severe COVID-19
7871_16	T132A	Transmembrane protein 132A	Q24JP5	TMEM132A	chr11:60924460	Inverse variance weighted	6	0.038	0.041	3.551E-01	0.973	cis	Severe COVID-19
9482_110	NUDT9	ADP-ribose pyrophosphatase, mitochondrial	Q9BW91	NUDT9	chr4:87422573	Wald ratio	1	-0.293	0.317	3.553E-01	0.973	cis	Severe COVID-19
14091_42	Carbonyl reductase 3	Carbonyl reductase [NADPH] 3	O75828	CBR3	chr21:36135079	Inverse variance weighted	6	0.040	0.043	3.554E-01	0.973	cis	Severe COVID-19
14066_49	MAGI2	Membrane-associated guanylate kinase, WW and PDZ domain-containing protein 2	Q86UL8	MAGI2	chr7:79453667	Inverse variance weighted	6	-0.031	0.033	3.559E-01	0.973	cis	Severe COVID-19
14112_40	RELT	Tumor necrosis factor receptor superfamily member 19L	Q969Z4	RELT	chr11:73376399	Wald ratio	1	0.421	0.456	3.561E-01	0.973	cis	Severe COVID-19
10080_9	EI2BA	Translation initiation factor eIF-2B subunit alpha	Q14232	EIF2B1	chr12:123633766	Wald ratio	1	0.241	0.261	3.567E-01	0.973	cis	Severe COVID-19
8006_12	DJB12	DnaJ homolog subfamily B member 12	Q9NXW2	DNAJB12	chr10:72355149	Wald ratio	1	0.158	0.172	3.583E-01	0.973	cis	Severe COVID-19
8014_359	MANEA	Glycoprotein endo-alpha-1,2-mannosidase	Q5SRI9	MANEA	chr6:95577485	Inverse variance weighted	6	0.020	0.022	3.587E-01	0.973	cis	Severe COVID-19
10514_5	PGD2 synthase	Prostaglandin-H2 D-isomerase	P41222	PTGDS	chr9:136975092	Wald ratio	1	0.277	0.302	3.590E-01	0.973	cis	Severe COVID-19
4706_17	41	Protein 4.1	P11171	EPB41	chr1:28887091	Inverse variance weighted	2	-0.243	0.264	3.591E-01	0.973	cis	Severe COVID-19
14711_27	Cystatin M	Cystatin-M	Q15828	CST6	chr11:66012008	Wald ratio	1	0.176	0.192	3.603E-01	0.973	cis	Severe COVID-19
19188_21	NAP2L	nucleosome assembly protein 1-like 4	Q99733	NAP1L4	chr11:2992377	Wald ratio	1	0.090	0.098	3.603E-01	0.973	cis	Severe COVID-19
16318_12	ALK-1	Serine/threonine-protein kinase receptor R3	P37023	ACVRL1	chr12:51906908	Inverse variance weighted	3	0.069	0.076	3.607E-01	0.973	cis	Severe COVID-19
10816_150	PILRA isoform FDF03-M14	Paired immunoglobulin-like type 2 receptor alpha isoform FDF03-M14	Q9UKJ1	PILRA	chr7:100367530	Inverse variance weighted	3	0.221	0.243	3.621E-01	0.973	cis	Severe COVID-19
10088_37	APT	Adenine phosphoribosyltransferase	P07741	APRT	chr16:88811937	Inverse variance weighted	4	-0.069	0.076	3.623E-01	0.973	cis	Severe COVID-19
4990_87	GP1BA	Platelet glycoprotein Ib alpha chain	P07359	GP1BA	chr17:4932277	Inverse variance weighted	3	-0.116	0.127	3.629E-01	0.973	cis	Severe COVID-19

11656_110	EVL	Ena/VASP-like protein	Q9UI08	EVL	chr14:99971449	Inverse variance weighted	3	0.170	0.187	3.638E-01	0.973	cis	Severe COVID-19
9370_69	GGH	Gamma-glutamyl hydrolase	Q92820	GGH	chr8:63039407	Inverse variance weighted	5	-0.035	0.039	3.642E-01	0.973	cis	Severe COVID-19
17692_2	BT3A3	Butyrophilin subfamily 3 member A3	O00478	BTN3A3	chr6:26440472	Inverse variance weighted	9	-0.042	0.046	3.654E-01	0.973	cis	Severe COVID-19
14088_38	IGFBP-6	Insulin-like growth factor-binding protein 6	P24592	IGFBP6	chr12:53097436	Wald ratio	1	-0.192	0.213	3.657E-01	0.973	cis	Severe COVID-19
5350_14	GPC6	Glypican-6	Q9Y625	GPC6	chr13:93226807	Wald ratio	1	0.289	0.320	3.661E-01	0.973	cis	Severe COVID-19
8932_1	ENTP6	Ectonucleoside triphosphate diphosphohydrolase 6	O75354	ENTPD6	chr20:25195693	Wald ratio	1	-0.129	0.143	3.679E-01	0.973	cis	Severe COVID-19
13423_94	CJ058	Redox-regulatory protein FAM213A	Q9BRX8	PRXL2A	chr10:80407829	Wald ratio	1	0.275	0.306	3.680E-01	0.973	cis	Severe COVID-19
3166_92	Siglec-3	Myeloid cell surface antigen CD33	P20138	CD33	chr19:51225064	Inverse variance weighted	6	0.029	0.032	3.683E-01	0.973	cis	Severe COVID-19
14156_33	14-3-3 protein beta/alpha	14-3-3 protein beta/alpha	P31946	YWHAB	chr20:44885702	Wald ratio	1	0.242	0.269	3.685E-01	0.973	cis	Severe COVID-19
4324_33	CYTT	Cystatin-SA	P09228	CST2	chr20:23826729	Inverse variance weighted	5	-0.095	0.105	3.690E-01	0.973	cis	Severe COVID-19
3457_57	Periostin	Periostin	Q15063	POSTN	chr13:37598844	Inverse variance weighted	4	0.057	0.064	3.706E-01	0.973	cis	Severe COVID-19
6379_62	ATL2	ADAMTS-like protein 2	Q86TH1	ADAMTSL2	chr9:133532164	Inverse variance weighted	2	-0.133	0.149	3.710E-01	0.973	cis	Severe COVID-19
10708_3	GON2	Progonadoliberin-2	O43555	GNRH2	chr20:3043622	Wald ratio	1	-0.129	0.144	3.724E-01	0.973	cis	Severe COVID-19
3508_78	MDC	C-C motif chemokine 22	O00626	CCL22	chr16:57358783	Inverse variance weighted	5	-0.075	0.084	3.726E-01	0.973	cis	Severe COVID-19
7822_11	HRSL2	HRAS-like suppressor 2	Q9NWW9	PLAAT2	chr11:63563379	Wald ratio	1	-0.286	0.321	3.733E-01	0.973	cis	Severe COVID-19
4763_31	Afamin	Afamin	P43652	AFM	chr4:73481745	Inverse variance weighted	3	0.058	0.065	3.734E-01	0.973	cis	Severe COVID-19
5584_21	Holo-TC II	Holo-Transcobalamin-2	P20062	TCN2	chr22:30607003	Inverse variance weighted	4	0.055	0.062	3.735E-01	0.973	cis	Severe COVID-19
19523_215	PARK7	Protein DJ-1	Q99497	PARK7	chr1:7954291	Wald ratio	1	-0.128	0.145	3.742E-01	0.973	cis	Severe COVID-19
12437_18	ULK3	Serine/threonine-protein kinase ULK3	Q6PHR2	ULK3	chr15:74843346	Wald ratio	1	-0.305	0.343	3.743E-01	0.973	cis	Severe COVID-19
7947_19	AP4AT	AP-4 complex accessory subunit tepsin	Q96N21	TEPSIN	chr17:81239091	Wald ratio	1	0.250	0.282	3.746E-01	0.973	cis	Severe COVID-19
8097_77	LIPN	Lipase member N	Q5VXI9	LIPN	chr10:88759982	Inverse variance weighted	6	-0.029	0.033	3.759E-01	0.973	cis	Severe COVID-19
3389_7	PCI	Plasma serine protease inhibitor	P05154	SERPINA5	chr14:94561442	Inverse variance weighted	2	-0.170	0.192	3.760E-01	0.973	cis	Severe COVID-19
9793_145	IGDC4	Immunoglobulin superfamily DCC subclass member 4	Q8TDY8	IGDCC4	chr15:65422947	Inverse variance weighted	7	-0.039	0.044	3.774E-01	0.973	cis	Severe COVID-19
5749_53	COL	Colipase	P04118	CLPS	chr6:35797344	Inverse variance weighted	10	-0.036	0.041	3.776E-01	0.973	cis	Severe COVID-19
6255_74	CPXM1	Probable carboxypeptidase X1	Q96SM3	CPXM1	chr20:2800627	Inverse variance weighted	5	-0.058	0.066	3.777E-01	0.973	cis	Severe COVID-19
8885_6	CA2D3	Voltage-dependent calcium channel subunit alpha-2/delta-3	Q8IZS8	CACNA2D3	chr3:54122547	Inverse variance weighted	5	-0.069	0.078	3.780E-01	0.973	cis	Severe COVID-19
2962_50	PTHrP	Parathyroid hormone-related protein	P12272	PTHLH	chr12:27972733	Inverse variance weighted	3	0.099	0.112	3.782E-01	0.973	cis	Severe COVID-19
12356_65	Sorcin	Sorcin	P30626	SRI	chr7:88226993	Wald ratio	1	0.114	0.130	3.793E-01	0.973	cis	Severe COVID-19
2977_7	EDAR	Tumor necrosis factor receptor superfamily member EDAR	Q9UNE0	EDAR	chr2:108989372	Inverse variance weighted	7	-0.028	0.032	3.807E-01	0.973	cis	Severe COVID-19
5107_7	Notch 1	Neurogenic locus notch homolog protein 1	P46531	NOTCH1	chr9:136546048	Inverse variance weighted	2	0.112	0.127	3.811E-01	0.973	cis	Severe COVID-19
7948_129	GLTD2	Glycolipid transfer protein domain-containing protein 2	A6NH11	GLTPD2	chr17:4788964	Inverse variance weighted	3	0.067	0.076	3.818E-01	0.973	cis	Severe COVID-19
5598_3	GREM2	Gremlin-2	Q9H772	GREM2	chr1:240612155	Wald ratio	1	-0.214	0.245	3.819E-01	0.973	cis	Severe COVID-19
14131_37	EFNB2:ECD	Ephrin-B2:Extracellular domain	P52799	EFNB2	chr13:106535662	Inverse variance weighted	2	0.230	0.263	3.819E-01	0.973	cis	Severe COVID-19
7935_26	LARGE	Glycosyltransferase-like protein LARGE1	O95461	LARGE1	chr22:33922841	Inverse variance weighted	2	-0.157	0.180	3.820E-01	0.973	cis	Severe COVID-19
3438_10	FSTL3	Follistatin-related protein 3	O95633	FSTL3	chr19:676392	Inverse variance weighted	2	0.165	0.189	3.821E-01	0.973	cis	Severe COVID-19
16770_3	REG1B	Lithostathine-1-beta	P48304	REG1B	chr2:79088019	Inverse variance weighted	3	0.216	0.248	3.822E-01	0.973	cis	Severe COVID-19
6039_24	CRHBP	Corticotropin-releasing factor-binding protein	P24387	CRHBP	chr5:76953045	Inverse variance weighted	6	0.048	0.055	3.835E-01	0.973	cis	Severe COVID-19
6471_53	FHR4	Complement factor H-related protein 4	Q92496	CFHR4	chr1:196888014	Inverse variance weighted	10	0.053	0.061	3.836E-01	0.973	cis	Severe COVID-19
17325_10	KGUA	Guanylate kinase	Q16774	GUK1	chr1:228139962	Inverse variance weighted	5	-0.083	0.095	3.842E-01	0.973	cis	Severe COVID-19
3448_13	IR	Insulin receptor	P06213	INSR	chr19:7294414	Wald ratio	1	-0.185	0.213	3.852E-01	0.973	cis	Severe COVID-19
10511_10	Collagen alpha-3(VI):isoform 3	Collagen alpha-3(VI) chain:isoform 3	P12111	COL6A3	chr2:237414328	Inverse variance weighted	2	0.200	0.230	3.852E-01	0.973	cis	Severe COVID-19
13114_50	Lumican	Lumican	P51884	LUM	chr12:91111494	Inverse variance weighted	7	0.048	0.056	3.853E-01	0.973	cis	Severe COVID-19
9092_33	ANGL1:N-term	Angiopoietin-related protein 1:N-term	O95841	ANGPTL1	chr1:178871077	Wald ratio	1	0.248	0.286	3.859E-01	0.973	cis	Severe COVID-19
13242_134	SHAN3	SH3 and multiple ankyrin repeat domains protein 3	Q9BYB0	SHANK3	chr22:50674415	Inverse variance weighted	5	-0.037	0.042	3.866E-01	0.973	cis	Severe COVID-19
10948_14	PLD3	Phospholipase D3	Q8IV08	PLD3	chr19:40348456	Wald ratio	1	0.270	0.312	3.869E-01	0.973	cis	Severe COVID-19
7227_75	COCH	Cochlin	O43405	COCH	chr14:30874514	Inverse variance weighted	4	-0.056	0.065	3.871E-01	0.973	cis	Severe COVID-19
18173_11	ARK73	Aflatoxin B1 aldehyde reductase member 3	O95154	AKR7A3	chr1:19288770	Inverse variance weighted	5	0.046	0.053	3.879E-01	0.973	cis	Severe COVID-19
16851_50	SCO2	Protein SCO2 homolog, mitochondrial	O43819	SCO2	chr22:50526461	Wald ratio	1	-0.138	0.160	3.881E-01	0.973	cis	Severe COVID-19
19223_6	RAB1A	Ras-related protein Rab-1A	P62820	RAB1A	chr2:65130331	Wald ratio	1	-0.231	0.268	3.886E-01	0.973	cis	Severe COVID-19
16781_2	ENASE	Cytosolic endo-beta-N-acetylglucosaminidase	Q8NFI3	ENGASE	chr17:79074824	Inverse variance weighted	9	0.028	0.032	3.887E-01	0.973	cis	Severe COVID-19
3305_6	DLL4	Delta-like protein 4	Q9NR61	DLL4	chr15:40929340	Wald ratio	1	0.256	0.297	3.888E-01	0.973	cis	Severe COVID-19
8368_102	TNF sR-II	Tumor necrosis factor receptor superfamily member 1B	P20333	TNFRSF1B	chr1:12166991	Wald ratio	1	-0.080	0.093	3.903E-01	0.976	cis	Severe COVID-19
17515_6	STCH	Heat shock 70 kDa protein 13	P48723	HSPA13	chr21:14383484	Wald ratio	1	0.132	0.154	3.912E-01	0.977	cis	Severe COVID-19
4564_2	PLXC1	Plexin-C1	O60486	PLXNC1	chr12:94148577	Inverse variance weighted	11	0.018	0.021	3.938E-01	0.981	cis	Severe COVID-19
3366_51	ECM1	Extracellular matrix protein 1	Q16610	ECM1	chr1:150508062	Inverse variance weighted	11	-0.029	0.035	3.959E-01	0.984	cis	Severe COVID-19
12731_12	PKHA7	Pleckstrin homology domain-containing family A member 7	Q6IQ23	PLEKHA7	chr11:17014415	Inverse variance weighted	4	-0.040	0.047	3.959E-01	0.984	cis	Severe COVID-19
3580_25	a1-Antitrypsin	Alpha-1-antitrypsin	P01009	SERPINA1	chr14:94390693	Inverse variance weighted	7	0.023	0.028	3.975E-01	0.985	cis	Severe COVID-19
18922_27	CD68	Macrosialin	P34810	CD68	chr17:7579491	Inverse variance weighted	2	-0.166	0.196	3.975E-01	0.985	cis	Severe COVID-19
5116_62	ROBO2	Roundabout homolog 2	Q9HCK4	ROBO2	chr3:75906695	Wald ratio	1	-0.290	0.343	3.980E-01	0.985	cis	Severe COVID-19
15513_108	Prostasin	Prostasin	Q16651	PRSS8	chr16:31135727	Inverse variance weighted	2	-0.148	0.177	4.013E-01	0.988	cis	Severe COVID-19
5867_60	ARGI1	Arginase-1	P05089	ARG1	chr6:131470832	Inverse variance weighted	2	0.163	0.194	4.015E-01	0.988	cis	Severe COVID-19
3171_57	amyloid precursor protein	Amyloid beta A4 protein	P05067	APP	chr21:26171128	Wald ratio	1	0.170	0.203	4.022E-01	0.988	cis	Severe COVID-19
4276_10	prostatic binding protein	Phosphatidylethanolamine-binding protein 1	P30086	PEBP1	chr12:118136124	Inverse variance weighted	7	-0.039	0.046	4.022E-01	0.988	cis	Severe COVID-19
13929_27	OCTC	Peroxisomal carnitine O-octanoyltransferase	Q9UKG9	CROT	chr7:87345664	Inverse variance weighted	6	-0.044	0.053	4.022E-01	0.988	cis	Severe COVID-19
10737_96	Serpin B1	Leukocyte elastase inhibitor	P30740	SERPINB1	chr6:2841959	Wald ratio	1	0.273	0.327	4.032E-01	0.988	cis	Severe COVID-19
3054_3	Haptoglobin, Mixed Type	Haptoglobin	P00738	HP	chr16:72054505	Inverse variance weighted	4	-0.033	0.040	4.033E-01	0.988	cis	Severe COVID-19
11273_176	GSTT2	Glutathione S-transferase theta-2B	P0CG30	GSTT2B	chr22:23961195	Inverse variance weighted	4	-0.053	0.063	4.042E-01	0.988	cis	Severe COVID-19
12801_33	IRF2	Interferon regulatory factor 2	P14316	IRF2	chr4:184474558	Wald ratio	1	-0.244	0.293	4.051E-01	0.988	cis	Severe COVID-19
4908_6	Endoglin	Endoglin	P17813	ENG	chr9:127854658	Inverse variance weighted	3	0.055	0.067	4.054E-01	0.988	cis	Severe COVID-19
15472_16	LRP11	Low-density lipoprotein receptor-related protein 11	Q86VZ4	LRP11	chr6:149864359	Inverse variance weighted	6	-0.071	0.085	4.065E-01	0.988	cis	Severe COVID-19
11145_72	K154L	UPF0606 protein KIAA1549L	Q6ZVL6	KIAA1549L	chr11:33376108	Inverse variance weighted	7	-0.036	0.043	4.078E-01	0.988	cis	Severe COVID-19
19117_3	PP14A	Protein phosphatase 1 regulatory subunit 14A	Q96A00	PPP1R14A	chr19:38256532	Wald ratio	1	0.216	0.262	4.094E-01	0.988	cis	Severe COVID-19
3235_50	WFKN2	WAP, Kazal, immunoglobulin, Kunitz and NTR domain-containing protein 2	Q8TEU8	WFIKKN2	chr17:50834650	Inverse variance weighted	7	0.033	0.040	4.100E-01	0.988	cis	Severe COVID-19
6151_18	MP2K3	Dual specificity mitogen-activated protein kinase kinase 3	P46734	MAP2K3	chr17:21284672	Wald ratio	1	0.272	0.330	4.101E-01	0.988	cis	Severe COVID-19
18380_78	Albumin	Serum albumin	P02768	ALB	chr4:73397114	Wald ratio	1	0.290	0.352	4.104E-01	0.988	cis	Severe COVID-19
7916_10	S100A7	Protein S100-A7	P31151	S100A7	chr1:153460651	Inverse variance weighted	7	0.024	0.029	4.134E-01	0.988	cis	Severe COVID-19
17153_46	KI2L3	Killer cell immunoglobulin-like receptor 2DL3	P43628	KIR2DL3	chr19:54738513	Inverse variance weighted	4	-0.023	0.029	4.137E-01	0.988	cis	Severe COVID-19
5430_66	SHPS1	Tyrosine-protein phosphatase non-receptor type substrate 1	P78324	SIRPA	chr20:1894167	Inverse variance weighted	9	0.024	0.030	4.140E-01	0.988	cis	Severe COVID-19

8225_86	EPHB2	Ephrin type-B receptor 2	P29323	EPHB2	chr1:22710839	Inverse variance weighted	3	0.051	0.063	4.150E-01	0.988	cis	Severe COVID-19
18386_36	GLRX1	Glutaredoxin-1	P35754	GLRX	chr5:95822726	Wald ratio	1	-0.223	0.273	4.153E-01	0.988	cis	Severe COVID-19
8479_4	MMP-10	Stromelysin-2	P09238	MMP10	chr11:102780628	Inverse variance weighted	6	-0.059	0.073	4.163E-01	0.988	cis	Severe COVID-19
19176_27	FA49B	Protein FAM49B	Q9NUQ9	CYRIB	chr8:130017504	Wald ratio	1	0.305	0.376	4.176E-01	0.988	cis	Severe COVID-19
13473_55	IP3KA	Inositol-trisphosphate 3-kinase A	P23677	ITPKA	chr15:41493393	Wald ratio	1	0.172	0.213	4.186E-01	0.988	cis	Severe COVID-19
2819_23	Cadherin-5	Cadherin-5	P33151	CDH5	chr16:66366622	Inverse variance weighted	2	0.086	0.107	4.200E-01	0.988	cis	Severe COVID-19
4220_39	FER	Tyrosine-protein kinase Fer	P16591	FER	chr5:108747841	Wald ratio	1	-0.197	0.245	4.208E-01	0.988	cis	Severe COVID-19
18458_4	PGES2	Prostaglandin E synthase 2	Q9H7Z7	PTGES2	chr9:128128462	Wald ratio	1	-0.162	0.201	4.211E-01	0.988	cis	Severe COVID-19
19273_3	Glutathione reductase	Glutathione reductase, mitochondrial	P00390	GSR	chr8:30727846	Inverse variance weighted	3	-0.154	0.191	4.215E-01	0.988	cis	Severe COVID-19
11142_11	ANGL1:C-term	Angiopoietin-related protein 1:C-Term, Fibrinogen domian	O95841	ANGPTL1	chr1:178871077	Inverse variance weighted	4	0.039	0.048	4.216E-01	0.988	cis	Severe COVID-19
13093_6	SECTM1	Secreted and transmembrane protein 1	Q8WVN6	SECTM1	chr17:82334074	Inverse variance weighted	3	-0.082	0.102	4.216E-01	0.988	cis	Severe COVID-19
3367_8	FETUB	Fetuin-B	Q9UGM5	FETUB	chr3:186635969	Inverse variance weighted	5	0.096	0.120	4.217E-01	0.988	cis	Severe COVID-19
10620_21	PSP-94	Beta-microseminoprotein	P08118	MSMB	chr10:46048180	Inverse variance weighted	3	-0.019	0.024	4.223E-01	0.988	cis	Severe COVID-19
9964_10	MYZAP	Myocardial zonula adherens protein	P0CAP1	MYZAP	chr15:57591904	Wald ratio	1	-0.181	0.226	4.226E-01	0.988	cis	Severe COVID-19
11187_11	CL12A	C-type lectin domain family 12 member A	Q5QGZ9	CLEC12A	chr12:9951316	Inverse variance weighted	6	0.040	0.050	4.234E-01	0.988	cis	Severe COVID-19
15452_5	5'-Nucleotidase	5'-Nucleotidase	P21589	NT5E	chr6:85449584	Inverse variance weighted	4	-0.031	0.039	4.242E-01	0.988	cis	Severe COVID-19
15622_13	OBCAM	Opioid-binding protein/cell adhesion molecule	Q14982	OPCML	chr11:133532501	Wald ratio	1	-0.251	0.315	4.247E-01	0.988	cis	Severe COVID-19
2843_13	SPINT2	Kunitz-type protease inhibitor 2	O43291	SPINT2	chr19:38244035	Inverse variance weighted	7	-0.034	0.042	4.253E-01	0.988	cis	Severe COVID-19
5109_24	Nr-CAM	Neuronal cell adhesion molecule	Q92823	NRCAM	chr7:108456717	Wald ratio	1	0.252	0.317	4.257E-01	0.988	cis	Severe COVID-19
19377_14	NOE2	Noelin-2	O95897	OLFM2	chr19:9936515	Inverse variance weighted	6	-0.036	0.045	4.258E-01	0.988	cis	Severe COVID-19
7950_142	BTNL9	Butyrophilin-like protein 9	Q6UXG8	BTNL9	chr5:181040225	Wald ratio	1	0.175	0.220	4.259E-01	0.988	cis	Severe COVID-19
18214_2	GSH0	Glutamate--cysteine ligase regulatory subunit	P48507	GCLM	chr1:93909456	Inverse variance weighted	2	0.134	0.168	4.259E-01	0.988	cis	Severe COVID-19
4801_13	PERL	Lactoperoxidase	P22079	LPO	chr17:58218548	Inverse variance weighted	2	-0.054	0.067	4.260E-01	0.988	cis	Severe COVID-19
5256_86	PDE5A	cGMP-specific 3',5'-cyclic phosphodiesterase	O76074	PDE5A	chr4:119628804	Inverse variance weighted	3	-0.126	0.159	4.278E-01	0.988	cis	Severe COVID-19
3796_79	ANGL4	Angiopoietin-related protein 4	Q9BY76	ANGPTL4	chr19:8363289	Wald ratio	1	0.128	0.162	4.283E-01	0.988	cis	Severe COVID-19
2778_10	IL-22	Interleukin-22	Q9GZX6	IL22	chr12:68253604	Wald ratio	1	-0.199	0.252	4.304E-01	0.988	cis	Severe COVID-19
19153_53	MPPD2	Metallophosphoesterase MPPED2	Q15777	MPPED2	chr11:30586872	Inverse variance weighted	2	0.138	0.175	4.305E-01	0.988	cis	Severe COVID-19
4455_89	MFGM	Lactadherin	Q08431	MFGE8	chr15:88913468	Inverse variance weighted	4	-0.060	0.076	4.308E-01	0.988	cis	Severe COVID-19
7648_9	MYPC1	Myosin-binding protein C, slow-type	Q00872	MYBPC1	chr12:101568353	Wald ratio	1	0.144	0.183	4.308E-01	0.988	cis	Severe COVID-19
19635_69	FGL2	Fibroleukin	Q14314	FGL2	chr7:77199848	Wald ratio	1	0.207	0.263	4.310E-01	0.988	cis	Severe COVID-19
6986_17	HS3SB	Heparan sulfate glucosamine 3-O-sulfotransferase 3B1	Q9Y662	HS3ST3B1	chr17:14301081	Wald ratio	1	0.250	0.317	4.313E-01	0.988	cis	Severe COVID-19
6904_14	LRRT2	Leucine-rich repeat transmembrane neuronal protein 2	O43300	LRRTM2	chr5:138875368	Wald ratio	1	0.144	0.184	4.316E-01	0.988	cis	Severe COVID-19
11178_21	SVEP1:EGF-like domains 4-6	Sushi, von Willebrand factor type A, EGF and pentraxin domain-containing protein 1:EGF-like domains 4-6	Q4LDE5	SVEP1	chr9:110579880	Inverse variance weighted	4	0.079	0.100	4.318E-01	0.988	cis	Severe COVID-19
4991_12	GPC5	Glypican-5	P78333	GPC5	chr13:91398621	Inverse variance weighted	6	-0.029	0.037	4.319E-01	0.988	cis	Severe COVID-19
6521_35	NPTX2	Neuronal pentraxin-2	P47972	NPTX2	chr7:98617285	Inverse variance weighted	2	0.121	0.154	4.324E-01	0.988	cis	Severe COVID-19
13944_3	SULT 1A3	Sulfotransferase 1A3	P0DMM9	SULT1A3	chr16:30199228	Inverse variance weighted	4	0.102	0.130	4.338E-01	0.988	cis	Severe COVID-19
9884_8	PPIL1	Peptidyl-prolyl cis-trans isomerase-like 1	Q9Y3C6	PPIL1	chr6:36874803	Inverse variance weighted	4	0.025	0.032	4.338E-01	0.988	cis	Severe COVID-19
11510_31	Apo L1	Apolipoprotein L1	O14791	APOL1	chr22:36253071	Inverse variance weighted	5	-0.035	0.045	4.341E-01	0.988	cis	Severe COVID-19
16916_19	SLIK6	SLIT and NTRK-like protein 6	Q9H5Y7	SLITRK6	chr13:85806683	Inverse variance weighted	4	-0.116	0.148	4.342E-01	0.988	cis	Severe COVID-19
10833_64	HHIP	Hedgehog-interacting protein	Q96QV1	HHIP	chr4:144646156	Inverse variance weighted	2	-0.078	0.100	4.343E-01	0.988	cis	Severe COVID-19
5508_62	Cathepsin D	Cathepsin D	P07339	CTSD	chr11:1764573	Inverse variance weighted	5	0.045	0.058	4.380E-01	0.988	cis	Severe COVID-19
2831_29	Kallikrein 11	Kallikrein-11	Q9UBX7	KLK11	chr19:51028039	Inverse variance weighted	7	0.065	0.084	4.386E-01	0.988	cis	Severe COVID-19
6580_29	Pregnancy zone protein	Pregnancy zone protein	P20742	PZP	chr12:9208395	Inverse variance weighted	2	-0.097	0.126	4.419E-01	0.988	cis	Severe COVID-19
13463_1	PXDN	Peroxidasin homolog	Q92626	PXDN	chr2:1744852	Inverse variance weighted	4	0.098	0.127	4.421E-01	0.988	cis	Severe COVID-19
12008_3	CD7	T-cell antigen CD7	P09564	CD7	chr17:82317608	Inverse variance weighted	4	0.122	0.158	4.422E-01	0.988	cis	Severe COVID-19
5069_9	DAF	Complement decay-accelerating factor	P08174	CD55	chr1:207321519	Inverse variance weighted	4	-0.079	0.103	4.428E-01	0.988	cis	Severe COVID-19
3175_51	ATS13	A disintegrin and metalloproteinase with thrombospondin motifs 13	Q76LX8	ADAMTS13	chr9:133414358	Inverse variance weighted	7	0.025	0.032	4.433E-01	0.988	cis	Severe COVID-19
11360_39	RRM1	Ribonucleoside-diphosphate reductase large subunit	P23921	RRM1	chr11:4094707	Wald ratio	1	0.219	0.286	4.434E-01	0.988	cis	Severe COVID-19
13094_75	RSPO3	R-spondin-3	Q9BXY4	RSPO3	chr6:127118671	Wald ratio	1	0.190	0.248	4.445E-01	0.988	cis	Severe COVID-19
3029_52	DC-SIGN	CD209 antigen	Q9NNX6	CD209	chr19:7747564	Inverse variance weighted	4	0.074	0.096	4.445E-01	0.988	cis	Severe COVID-19
4866_59	TrkB	BDNF/NT-3 growth factors receptor	Q16620	NTRK2	chr9:84668375	Wald ratio	1	-0.186	0.243	4.446E-01	0.988	cis	Severe COVID-19
12475_48	CLIC5	Chloride intracellular channel protein 5	Q9NZA1	CLIC5	chr6:46080348	Inverse variance weighted	5	0.049	0.064	4.467E-01	0.988	cis	Severe COVID-19
4499_21	PDGF-AA	Platelet-derived growth factor subunit A	P04085	PDGFA	chr7:520296	Inverse variance weighted	3	-0.134	0.177	4.473E-01	0.988	cis	Severe COVID-19
3331_8	RGMB	RGM domain family member B	Q6NW40	RGMB	chr5:98768650	Inverse variance weighted	3	-0.093	0.122	4.483E-01	0.988	cis	Severe COVID-19
4314_12	XTP3A	dCTP pyrophosphatase 1	Q9H773	DCTPP1	chr16:30430030	Wald ratio	1	0.142	0.187	4.486E-01	0.988	cis	Severe COVID-19
17766_5	NCF-1	Neutrophil cytosol factor 1	P14598	NCF1	chr7:74774011	Inverse variance weighted	6	0.060	0.079	4.487E-01	0.988	cis	Severe COVID-19
17403_14	acyl-Coenzyme A dehydrogenase	Short/branched chain specific acyl-CoA dehydrogenase, mitochondrial	P45954	ACADSB	chr10:123008979	Inverse variance weighted	2	0.105	0.139	4.490E-01	0.988	cis	Severe COVID-19
3415_61	BSP	Bone sialoprotein 2	P21815	IBSP	chr4:87799554	Wald ratio	1	-0.110	0.146	4.499E-01	0.988	cis	Severe COVID-19
8346_9	DPP2	Dipeptidyl peptidase 2	Q9UHL4	DPP7	chr9:137118309	Inverse variance weighted	7	-0.046	0.061	4.501E-01	0.988	cis	Severe COVID-19
7875_86	PLEK	Pleckstrin	P08567	PLEK	chr2:68365282	Inverse variance weighted	2	-0.094	0.124	4.502E-01	0.988	cis	Severe COVID-19
18310_26	SELW	Selenoprotein W	P63302	SELENOW	chr19:47778677	Inverse variance weighted	3	0.079	0.104	4.503E-01	0.988	cis	Severe COVID-19
3041_55	MRC2	C-type mannose receptor 2	Q9UBG0	MRC2	chr17:62627670	Inverse variance weighted	5	-0.058	0.076	4.512E-01	0.988	cis	Severe COVID-19
5644_60	RNAS4	Ribonuclease 4	P34096	RNASE4	chr14:20684560	Inverse variance weighted	5	0.054	0.072	4.528E-01	0.988	cis	Severe COVID-19
11606_22	DNJB6	DnaJ homolog subfamily B member 6	O75190	DNAJB6	chr7:157335381	Wald ratio	1	0.218	0.291	4.535E-01	0.988	cis	Severe COVID-19
18819_21	PPIC	Peptidyl-prolyl cis-trans isomerase C	P45877	PPIC	chr5:123036725	Inverse variance weighted	3	0.033	0.044	4.553E-01	0.988	cis	Severe COVID-19
12686_15	THTM	3-mercaptopyruvate sulfurtransferase	P25325	MPST	chr22:37019635	Inverse variance weighted	2	-0.162	0.217	4.556E-01	0.988	cis	Severe COVID-19
5701_81	Tetranectin	Tetranectin	P05452	CLEC3B	chr3:45001548	Inverse variance weighted	2	-0.089	0.119	4.559E-01	0.988	cis	Severe COVID-19
7049_2	ADAM 23	Disintegrin and metalloproteinase domain-containing protein 23	O75077	ADAM23	chr2:206443532	Inverse variance weighted	6	0.018	0.025	4.559E-01	0.988	cis	Severe COVID-19
5034_79	Trypsin 2	Trypsin-2	P07478	PRSS2	chr7:142760398	Inverse variance weighted	4	-0.099	0.133	4.559E-01	0.988	cis	Severe COVID-19
18408_26	ARF4	ADP-ribosylation factor 4	P18085	ARF4	chr3:57598220	Wald ratio	1	0.153	0.205	4.560E-01	0.988	cis	Severe COVID-19
19496_1	NPL	N-acetylneuraminate lyase	Q9BXD5	NPL	chr1:182789293	Inverse variance weighted	3	-0.067	0.090	4.561E-01	0.988	cis	Severe COVID-19
13697_51	GPDA	Glycerol-3-phosphate dehydrogenase [NAD(+)], cytoplasmic	P21695	GPD1	chr12:50103982	Wald ratio	1	0.139	0.187	4.567E-01	0.988	cis	Severe COVID-19
3481_87	XPNPEP1	Xaa-Pro aminopeptidase 1	Q9NQW7	XPNPEP1	chr10:109923553	Wald ratio	1	0.174	0.234	4.569E-01	0.988	cis	Severe COVID-19
2999_6	LSAMP	Limbic system-associated membrane protein	Q13449	LSAMP	chr3:117139389	Inverse variance weighted	4	0.069	0.094	4.578E-01	0.988	cis	Severe COVID-19
6572_10	LRRT4:ECD	Leucine-rich repeat transmembrane neuronal protein 4:Isoform 2, Extracellular domain	Q86VH4	LRRTM4	chr2:77593319	Wald ratio	1	-0.233	0.315	4.590E-01	0.988	cis	Severe COVID-19
7957_2	SCG3	Secretogranin-3	Q8WXD2	SCG3	chr15:51681492	Inverse variance weighted	7	-0.028	0.038	4.591E-01	0.988	cis	Severe COVID-19
15615_8	LIRB3	Leukocyte immunoglobulin-like receptor subfamily B member 3	O75022	LILRB3	chr19:54223506	Inverse variance weighted	5	-0.048	0.064	4.593E-01	0.988	cis	Severe COVID-19
19615_213	5NT3	Cytosolic 5'-nucleotidase 3A	Q9H0P0	NT5C3A	chr7:33062796	Wald ratio	1	0.143	0.193	4.597E-01	0.988	cis	Severe COVID-19

14048_7	IL-1 R AcP	Interleukin-1 Receptor accessory protein	Q9NPH3	IL1RAP	chr3:190514051	Inverse variance weighted	6	-0.017	0.023	4.608E-01	0.988	cis	Severe COVID-19
4964_67	ARTS1	Endoplasmic reticulum aminopeptidase 1	Q9NZ08	ERAP1	chr5:96808100	Inverse variance weighted	5	-0.023	0.031	4.609E-01	0.988	cis	Severe COVID-19
9015_1	PRG3	Proteoglycan 3	Q9Y2Y8	PRG3	chr11:57381150	Wald ratio	1	-0.293	0.397	4.609E-01	0.988	cis	Severe COVID-19
15604_18	JNK2	Mitogen-activated protein kinase 9	P45984	MAPK9	chr5:180292099	Inverse variance weighted	2	-0.105	0.143	4.625E-01	0.988	cis	Severe COVID-19
17350_13	CHM2B	Charged multivesicular body protein 2b	Q9UQN3	CHMP2B	chr3:87227271	Inverse variance weighted	3	-0.056	0.076	4.625E-01	0.988	cis	Severe COVID-19
17787_1	Enoyl-CoA hydratase	Enoyl-CoA hydratase, mitochondrial	P30084	ECHS1	chr10:133373354	Wald ratio	1	-0.207	0.282	4.626E-01	0.988	cis	Severe COVID-19
3232_28	TrATPase	Tartrate-resistant acid phosphatase type 5	P13686	ACP5	chr19:11579993	Inverse variance weighted	4	-0.056	0.077	4.634E-01	0.988	cis	Severe COVID-19
3890_8	LDH-H 1	L-lactate dehydrogenase B chain	P07195	LDHB	chr12:21757857	Wald ratio	1	0.312	0.427	4.651E-01	0.988	cis	Severe COVID-19
8028_22	SPINK5	Serine protease inhibitor Kazal-type 5	Q9NQ38	SPINK5	chr5:148025683	Wald ratio	1	0.081	0.112	4.677E-01	0.988	cis	Severe COVID-19
5509_7	EGF:ECD	Epidermal growth factor:Extracellular domain	P01133	EGF	chr4:109912883	Inverse variance weighted	4	0.107	0.147	4.681E-01	0.988	cis	Severe COVID-19
2855_49	ERK-1	Mitogen-activated protein kinase 3	P27361	MAPK3	chr16:30123506	Inverse variance weighted	3	-0.062	0.086	4.691E-01	0.988	cis	Severe COVID-19
5112_73	OX2G	OX-2 membrane glycoprotein	P41217	CD200	chr3:112332347	Wald ratio	1	-0.137	0.190	4.693E-01	0.988	cis	Severe COVID-19
10908_2	GLT13	Polypeptide N-acetylgalactosaminyltransferase 13	Q8IUC8	GALNT13	chr2:153871922	Wald ratio	1	-0.245	0.340	4.697E-01	0.988	cis	Severe COVID-19
11237_49	PCOC1	Procollagen C-endopeptidase enhancer 1	Q15113	PCOLCE	chr7:100602363	Inverse variance weighted	2	0.268	0.372	4.708E-01	0.988	cis	Severe COVID-19
8229_1	GXLT1	Glucoside xylosyltransferase 1	Q4G148	GXYLT1	chr12:42144874	Inverse variance weighted	5	0.061	0.085	4.711E-01	0.988	cis	Severe COVID-19
8476_11	CgA	Chromogranin-A	P10645	CHGA	chr14:92923150	Inverse variance weighted	4	0.055	0.076	4.718E-01	0.988	cis	Severe COVID-19
13388_57	NEC1	Neuroendocrine convertase 1	P29120	PCSK1	chr5:96434143	Inverse variance weighted	7	-0.036	0.051	4.722E-01	0.988	cis	Severe COVID-19
9212_22	CATF	Cathepsin F	Q9UBX1	CTSF	chr11:66568879	Inverse variance weighted	2	-0.057	0.079	4.734E-01	0.988	cis	Severe COVID-19
12459_13	PKHA1	Pleckstrin homology domain-containing family A member 1	Q9HB21	PLEKHA1	chr10:122374696	Inverse variance weighted	4	0.035	0.048	4.734E-01	0.988	cis	Severe COVID-19
6049_64	PTPRS	Receptor-type tyrosine-protein phosphatase S	Q13332	PTPRS	chr19:5340812	Wald ratio	1	-0.123	0.172	4.737E-01	0.988	cis	Severe COVID-19
5763_67	HBD-4	Beta-defensin 104	Q8WTQ1	DEFB104A	chr8:7836436	Inverse variance weighted	3	0.191	0.266	4.738E-01	0.988	cis	Severe COVID-19
9416_77	CBPM	Carboxypeptidase M	P14384	CPM	chr12:68971570	Wald ratio	1	0.111	0.156	4.745E-01	0.988	cis	Severe COVID-19
9398_30	GALP	Galanin-like peptide	Q9UBC7	GALP	chr19:56176008	Wald ratio	1	-0.171	0.239	4.746E-01	0.988	cis	Severe COVID-19
15476_6	REG3G	Regenerating islet-derived protein 3-gamma	Q6UW15	REG3G	chr2:79025686	Inverse variance weighted	6	0.027	0.038	4.747E-01	0.988	cis	Severe COVID-19
9337_43	TKN1	Protachykinin-1	P20366	TAC1	chr7:97732084	Inverse variance weighted	3	0.054	0.075	4.754E-01	0.988	cis	Severe COVID-19
6409_57	GP116	Adhesion G protein-coupled receptor F5	Q8IZF2	ADGRF5	chr6:46954943	Inverse variance weighted	7	-0.019	0.026	4.760E-01	0.988	cis	Severe COVID-19
4355_13	DAPK2	Death-associated protein kinase 2	Q9UIK4	DAPK2	chr15:64072033	Inverse variance weighted	3	0.040	0.057	4.762E-01	0.988	cis	Severe COVID-19
16773_29	SCUB3	Signal peptide, CUB and EGF-like domain-containing protein 3	Q8IX30	SCUBE3	chr6:35213956	Inverse variance weighted	3	0.063	0.088	4.770E-01	0.988	cis	Severe COVID-19
15475_4	PLTP	Phospholipid transfer protein	P55058	PLTP	chr20:45912155	Inverse variance weighted	9	-0.035	0.049	4.771E-01	0.988	cis	Severe COVID-19
7841_84	ESAM	Endothelial cell-selective adhesion molecule	Q96AP7	ESAM	chr11:124762290	Inverse variance weighted	6	0.042	0.059	4.776E-01	0.988	cis	Severe COVID-19
13126_52	DSC2	Desmocollin-2	Q02487	DSC2	chr18:31102522	Inverse variance weighted	4	-0.064	0.090	4.777E-01	0.988	cis	Severe COVID-19
13597_20	RAB31	Ras-related protein Rab-31	Q13636	RAB31	chr18:9708275	Inverse variance weighted	3	0.082	0.115	4.785E-01	0.988	cis	Severe COVID-19
3640_14	RAP	alpha-2-macroglobulin receptor-associated protein	P30533	LRPAP1	chr4:3532446	Inverse variance weighted	4	0.056	0.080	4.793E-01	0.988	cis	Severe COVID-19
11177_16	K2C5	Keratin, type II cytoskeletal 5	P13647	KRT5	chr12:52520530	Inverse variance weighted	2	-0.127	0.180	4.806E-01	0.988	cis	Severe COVID-19
15589_1	Gc-Globulin, Mixed Type	Vitamin D-binding protein	P02774	GC	chr4:71804041	Inverse variance weighted	2	0.079	0.112	4.818E-01	0.988	cis	Severe COVID-19
5102_55	MICB	MHC class I polypeptide-related sequence B	Q29980	MICB	chr6:31494881	Inverse variance weighted	6	0.023	0.032	4.820E-01	0.988	cis	Severe COVID-19
5012_67	Myokinase, human	Adenylate kinase isoenzyme 1	P00568	AK1	chr9:127877675	Wald ratio	1	-0.249	0.355	4.822E-01	0.988	cis	Severe COVID-19
7997_118	DOC2B	Double C2-like domain-containing protein beta	Q14184	DOC2B	chr17:181650	Wald ratio	1	-0.245	0.350	4.830E-01	0.988	cis	Severe COVID-19
11219_95	FGFP3	Fibroblast growth factor-binding protein 3	Q8TAT2	FGFBP3	chr10:91909486	Inverse variance weighted	3	0.063	0.089	4.831E-01	0.988	cis	Severe COVID-19
17802_4	SIAS	Sialic acid synthase	Q9NR45	NANS	chr9:98056732	Wald ratio	1	0.122	0.174	4.833E-01	0.988	cis	Severe COVID-19
8303_102	MYG1	UPF0160 protein MYG1, mitochondrial	Q9HB07	MYG1	chr12:53299695	Wald ratio	1	0.205	0.293	4.835E-01	0.988	cis	Severe COVID-19
9288_7	FKBP7	Peptidyl-prolyl cis-trans isomerase FKBP7	Q9Y680	FKBP7	chr2:178478600	Inverse variance weighted	4	0.036	0.052	4.835E-01	0.988	cis	Severe COVID-19
9449_150	C4b-binding protein alpha chain	C4b-binding protein alpha chain	P04003	C4BPA	chr1:207104233	Inverse variance weighted	6	-0.066	0.095	4.837E-01	0.988	cis	Severe COVID-19
12682_5	KAT3	Kynurenine--oxoglutarate transaminase 3	Q6YP21	KYAT3	chr1:88992953	Inverse variance weighted	7	-0.032	0.045	4.848E-01	0.988	cis	Severe COVID-19
15556_49	Alpha-amylase 2B	Alpha-amylase 2B	P19961	AMY2B	chr1:103553815	Inverse variance weighted	3	0.122	0.175	4.850E-01	0.988	cis	Severe COVID-19
13068_139	CCS	Copper chaperone for superoxide dismutase	O14618	CCS	chr11:66593153	Wald ratio	1	0.086	0.123	4.850E-01	0.988	cis	Severe COVID-19
5742_14	PPA6	Lysophosphatidic acid phosphatase type 6	Q9NPH0	ACP6	chr1:147670524	Inverse variance weighted	13	-0.015	0.022	4.859E-01	0.988	cis	Severe COVID-19
2647_66	Rab GDP dissociation inhibitor beta	Rab GDP dissociation inhibitor beta	P50395	GDI2	chr10:5842132	Wald ratio	1	-0.107	0.154	4.889E-01	0.988	cis	Severe COVID-19
2515_14	GFRa-2	GDNF family receptor alpha-2	O00451	GFRA2	chr8:21812357	Inverse variance weighted	6	-0.038	0.055	4.891E-01	0.988	cis	Severe COVID-19
18413_24	ARL4D	ADP-ribosylation factor-like protein 4D	P49703	ARL4D	chr17:43398993	Wald ratio	1	0.193	0.279	4.899E-01	0.988	cis	Severe COVID-19
2212_69	tPA	Tissue-type plasminogen activator	P00750	PLAT	chr8:42207709	Inverse variance weighted	4	0.056	0.082	4.899E-01	0.988	cis	Severe COVID-19
3825_18	MK08	Mitogen-activated protein kinase 8	P45983	MAPK8	chr10:48306639	Wald ratio	1	0.185	0.269	4.908E-01	0.988	cis	Severe COVID-19
9322_15	RCN1	Reticulocalbin-1	Q15293	RCN1	chr11:32091074	Wald ratio	1	-0.079	0.115	4.917E-01	0.988	cis	Severe COVID-19
5451_1	ALCAM	CD166 antigen	Q13740	ALCAM	chr3:105366909	Inverse variance weighted	4	0.100	0.145	4.924E-01	0.988	cis	Severe COVID-19
12685_57	HOME2	Homer protein homolog 2	Q9NSB8	HOMER2	chr15:82986153	Wald ratio	1	0.150	0.218	4.927E-01	0.988	cis	Severe COVID-19
5656_53	PP11	Poly(U)-specific endoribonuclease	P21128	ENDOU	chr12:47725567	Wald ratio	1	-0.166	0.244	4.945E-01	0.988	cis	Severe COVID-19
12571_14	ARL3	ADP-ribosylation factor-like protein 3	P36405	ARL3	chr10:102714397	Inverse variance weighted	2	-0.068	0.100	4.950E-01	0.988	cis	Severe COVID-19
13959_7	LAP	Cytosol aminopeptidase	P28838	LAP3	chr4:17577198	Wald ratio	1	-0.309	0.453	4.951E-01	0.988	cis	Severe COVID-19
12662_82	ECH1	Delta(3,5)-Delta(2,4)-dienoyl-CoA isomerase, mitochondrial	Q13011	ECH1	chr19:38831841	Inverse variance weighted	2	-0.035	0.051	4.952E-01	0.988	cis	Severe COVID-19
2617_56	ERBB3	Receptor tyrosine-protein kinase erbB-3	P21860	ERBB3	chr12:56076799	Wald ratio	1	0.091	0.134	4.955E-01	0.988	cis	Severe COVID-19
9574_11	BIN1	Myc box-dependent-interacting protein 1	O00499	BIN1	chr2:127107288	Inverse variance weighted	2	-0.072	0.105	4.958E-01	0.988	cis	Severe COVID-19
3221_54	SARP-2	Secreted frizzled-related protein 1	Q8N474	SFRP1	chr8:41309473	Inverse variance weighted	3	0.064	0.095	4.959E-01	0.988	cis	Severe COVID-19
11708_2	LCN1	Lipocalin-1	P31025	LCN1	chr9:135521438	Wald ratio	1	-0.150	0.222	4.987E-01	0.988	cis	Severe COVID-19
10070_22	PLCG2	1-phosphatidylinositol 4,5-bisphosphate phosphodiesterase gamma-2	P16885	PLCG2	chr16:81779279	Inverse variance weighted	2	0.168	0.249	4.988E-01	0.988	cis	Severe COVID-19
3340_53	TSP4	Thrombospondin-4	P35443	THBS4	chr5:79991311	Inverse variance weighted	3	-0.049	0.073	4.993E-01	0.988	cis	Severe COVID-19
5682_13	VASN	Vasorin	Q6EMK4	VASN	chr16:4371848	Wald ratio	1	-0.139	0.206	4.996E-01	0.988	cis	Severe COVID-19
2615_60	Ephrin-A5	Ephrin-A5	P52803	EFNA5	chr5:107670937	Inverse variance weighted	2	0.087	0.129	5.009E-01	0.988	cis	Severe COVID-19
13123_3	FLRT3:ECD	Leucine-rich repeat transmembrane protein FLRT3:Extracellular domain	Q9NZU0	FLRT3	chr20:14337614	Inverse variance weighted	6	-0.017	0.026	5.012E-01	0.988	cis	Severe COVID-19
15594_47	HTRA1	Serine protease HTRA1	Q92743	HTRA1	chr10:122458551	Inverse variance weighted	2	-0.102	0.152	5.017E-01	0.988	cis	Severe COVID-19
3803_10	CYTD	Cystatin-D	P28325	CST5	chr20:23879748	Inverse variance weighted	11	0.037	0.055	5.019E-01	0.988	cis	Severe COVID-19
7928_183	TPST1	Protein-tyrosine sulfotransferase 1	O60507	TPST1	chr7:66205317	Inverse variance weighted	2	0.034	0.050	5.030E-01	0.988	cis	Severe COVID-19
6557_50	LRC15	Leucine-rich repeat-containing protein 15	Q8TF66	LRRC15	chr3:194369743	Inverse variance weighted	4	0.054	0.082	5.061E-01	0.988	cis	Severe COVID-19
15386_7	FABPA	Fatty acid-binding protein, adipocyte	P15090	FABP4	chr8:81483236	Wald ratio	1	0.124	0.187	5.066E-01	0.988	cis	Severe COVID-19
3004_67	PD-L2	Programmed cell death 1 ligand 2	Q9BQ51	PDCD1LG2	chr9:5510531	Inverse variance weighted	5	-0.030	0.045	5.079E-01	0.988	cis	Severe COVID-19
3213_65	Nidogen	Nidogen-1	P14543	NID1	chr1:236065109	Inverse variance weighted	4	0.060	0.091	5.087E-01	0.988	cis	Severe COVID-19
9223_11	ARMET	Mesencephalic astrocyte-derived neurotrophic factor	P55145	MANF	chr3:51385291	Wald ratio	1	-0.222	0.336	5.098E-01	0.988	cis	Severe COVID-19
5713_9	IFN-lambda 3	Interferon lambda-3	Q8IZI9	IFNL3	chr19:39245250	Wald ratio	1	0.210	0.319	5.107E-01	0.988	cis	Severe COVID-19
5105_2	Nogo Receptor	Reticulon-4 receptor	Q9BZR6	RTN4R	chr22:20283246	Inverse variance weighted	5	-0.034	0.051	5.108E-01	0.988	cis	Severe COVID-19

17156_72	DCAK1	Serine/threonine-protein kinase DCLK1	O15075	DCLK1	chr13:36131382	Inverse variance weighted	3	0.061	0.093	5.108E-01	0.988	cis	Severe COVID-19
19482_11	HDHD3	Haloacid dehalogenase-like hydrolase domain-containing protein 3	Q9BSH5	HDHD3	chr9:113376986	Wald ratio	1	0.053	0.081	5.114E-01	0.988	cis	Severe COVID-19
8005_1	MXRA7	Matrix-remodeling-associated protein 7	P84157	MXRA7	chr17:76711004	Inverse variance weighted	5	-0.030	0.046	5.117E-01	0.988	cis	Severe COVID-19
15614_168	LIRA2	Leukocyte immunoglobulin-like receptor subfamily A member 2	Q8N149	LILRA2	chr19:54572920	Inverse variance weighted	4	-0.033	0.051	5.119E-01	0.988	cis	Severe COVID-19
16803_4	CALB2	Calretinin	P22676	CALB2	chr16:71358713	Inverse variance weighted	2	0.151	0.230	5.120E-01	0.988	cis	Severe COVID-19
2950_57	IGFBP-4	Insulin-like growth factor-binding protein 4	P22692	IGFBP4	chr17:40443450	Wald ratio	1	-0.178	0.272	5.125E-01	0.988	cis	Severe COVID-19
4131_72	Fibronectin	Fibronectin	P02751	FN1	chr2:215436073	Inverse variance weighted	2	0.175	0.268	5.128E-01	0.988	cis	Severe COVID-19
17773_26	SNAG	Gamma-soluble NSF attachment protein	Q99747	NAPG	chr18:10525905	Wald ratio	1	-0.167	0.255	5.129E-01	0.988	cis	Severe COVID-19
2692_74	NPS-PLA2	Phospholipase A2, membrane associated	P14555	PLA2G2A	chr1:19980416	Inverse variance weighted	6	-0.022	0.033	5.135E-01	0.988	cis	Severe COVID-19
3310_62	FCG2B	Low affinity immunoglobulin gamma Fc region receptor II-b	P31994	FCGR2B	chr1:161663143	Inverse variance weighted	12	0.045	0.068	5.137E-01	0.988	cis	Severe COVID-19
9357_4	CREG1	Protein CREG1	O75629	CREG1	chr1:167553805	Inverse variance weighted	2	-0.057	0.088	5.141E-01	0.988	cis	Severe COVID-19
17786_5	GGPPS	Geranylgeranyl pyrophosphate synthase	O95749	GGPS1	chr1:235327350	Wald ratio	1	-0.110	0.169	5.146E-01	0.988	cis	Severe COVID-19
9876_20	aldolase C	Fructose-bisphosphate aldolase C	P09972	ALDOC	chr17:28576948	Inverse variance weighted	2	-0.315	0.485	5.153E-01	0.988	cis	Severe COVID-19
3835_11	TLR2	Toll-like receptor 2	O60603	TLR2	chr4:153684070	Wald ratio	1	-0.376	0.579	5.158E-01	0.988	cis	Severe COVID-19
12637_7	CACP	Carnitine O-acetyltransferase	P43155	CRAT	chr9:129111189	Wald ratio	1	0.106	0.163	5.165E-01	0.988	cis	Severe COVID-19
7779_86	CHSTB	Carbohydrate sulfotransferase 11	Q9NPF2	CHST11	chr12:104455295	Inverse variance weighted	5	-0.039	0.060	5.176E-01	0.988	cis	Severe COVID-19
2654_19	TNF sR-I	Tumor necrosis factor receptor superfamily member 1A	P19438	TNFRSF1A	chr12:6342114	Wald ratio	1	-0.084	0.130	5.194E-01	0.988	cis	Severe COVID-19
7198_197	FA20B	Glycosaminoglycan xylosylkinase	O75063	FAM20B	chr1:179025804	Inverse variance weighted	2	0.111	0.172	5.204E-01	0.988	cis	Severe COVID-19
17737_7	IVD	Isovaleryl-CoA dehydrogenase, mitochondrial	P26440	IVD	chr15:40405485	Inverse variance weighted	2	0.115	0.178	5.208E-01	0.988	cis	Severe COVID-19
18831_6	LRIG1	Leucine-rich repeats and immunoglobulin-like domains protein 1	Q96JA1	LRIG1	chr3:66501263	Inverse variance weighted	8	-0.030	0.046	5.219E-01	0.988	cis	Severe COVID-19
10666_7	GNPTG	N-acetylglucosamine-1-phosphotransferase subunit gamma	Q9UJJ9	GNPTG	chr16:1351931	Inverse variance weighted	4	0.048	0.076	5.222E-01	0.988	cis	Severe COVID-19
12408_333	RB22A	Ras-related protein Rab-22A	Q9UL26	RAB22A	chr20:58309715	Wald ratio	1	0.213	0.332	5.222E-01	0.988	cis	Severe COVID-19
15529_33	Cysteine-rich protein 1	Cysteine and glycine-rich protein 1	P21291	CSRP1	chr1:201509456	Wald ratio	1	-0.106	0.166	5.234E-01	0.988	cis	Severe COVID-19
14747_9	CRLF1	Cytokine receptor-like factor 1	O75462	CRLF1	chr19:18607741	Inverse variance weighted	2	-0.043	0.068	5.246E-01	0.988	cis	Severe COVID-19
5392_73	Fas, soluble	Tumor necrosis factor receptor superfamily member 6	P25445	FAS	chr10:88953813	Inverse variance weighted	2	0.146	0.230	5.257E-01	0.988	cis	Severe COVID-19
14076_74	Cystatin-S	Cystatin-S	P01036	CST4	chr20:23689038	Inverse variance weighted	3	-0.059	0.093	5.264E-01	0.988	cis	Severe COVID-19
19614_8	Holo-TC I	Transcobalamin-1	P20061	TCN1	chr11:59866489	Inverse variance weighted	5	0.027	0.042	5.273E-01	0.988	cis	Severe COVID-19
17370_186	ABHDA	Mycophenolic acid acyl-glucuronide esterase, mitochondrial	Q9NUJ1	ABHD10	chr3:111979010	Wald ratio	1	-0.183	0.290	5.278E-01	0.988	cis	Severe COVID-19
3554_24	Adiponectin	Adiponectin	Q15848	ADIPOQ	chr3:186842704	Inverse variance weighted	4	-0.042	0.066	5.295E-01	0.988	cis	Severe COVID-19
17758_79	DCXR	L-xylulose reductase	Q7Z4W1	DCXR	chr17:82037709	Inverse variance weighted	2	0.098	0.156	5.299E-01	0.988	cis	Severe COVID-19
4775_34	Gelsolin	Gelsolin	P06396	GSN	chr9:121207794	Inverse variance weighted	2	0.079	0.126	5.301E-01	0.988	cis	Severe COVID-19
11161_5	SPG20	Spartin	Q8N0X7	SPART	chr13:36370180	Wald ratio	1	0.200	0.320	5.308E-01	0.988	cis	Severe COVID-19
18841_1	SPB13	Serpin B13	Q9UIV8	SERPINB13	chr18:63586989	Inverse variance weighted	4	0.064	0.102	5.308E-01	0.988	cis	Severe COVID-19
3293_2	CD5L	CD5 antigen-like	O43866	CD5L	chr1:157898256	Inverse variance weighted	4	-0.053	0.084	5.312E-01	0.988	cis	Severe COVID-19
16296_43	LGR5	Leucine-rich repeat-containing G-protein coupled receptor 5	O75473	LGR5	chr12:71439798	Wald ratio	1	-0.167	0.267	5.329E-01	0.988	cis	Severe COVID-19
5601_2	PGRP-L	N-acetylmuramoyl-L-alanine amidase	Q96PD5	PGLYRP2	chr19:15498956	Inverse variance weighted	5	0.031	0.050	5.330E-01	0.988	cis	Severe COVID-19
5478_50	PSMA	Glutamate carboxypeptidase 2	Q04609	FOLH1	chr11:49208638	Inverse variance weighted	2	-0.062	0.100	5.342E-01	0.988	cis	Severe COVID-19
18397_5	AK1C4	Aldo-keto reductase family 1 member C4	P17516	AKR1C4	chr10:5195462	Inverse variance weighted	8	0.032	0.051	5.344E-01	0.988	cis	Severe COVID-19
3191_50	WFKN1	WAP, kazal, immunoglobulin, kunitz and NTR domain-containing protein 1	Q96NZ8	WFIKKN1	chr16:629239	Inverse variance weighted	2	-0.071	0.115	5.351E-01	0.988	cis	Severe COVID-19
17794_6	Phosphomannomutase 2	Phosphomannomutase 2	O15305	PMM2	chr16:8788823	Inverse variance weighted	4	0.063	0.102	5.353E-01	0.988	cis	Severe COVID-19
8248_222	SIG14	Sialic acid-binding Ig-like lectin 14	Q08ET2	SIGLEC14	chr19:51646825	Inverse variance weighted	3	0.088	0.142	5.353E-01	0.988	cis	Severe COVID-19
10815_2	HABP4	Intracellular hyaluronan-binding protein 4	Q5JVS0	HABP4	chr9:96450169	Inverse variance weighted	3	-0.035	0.057	5.358E-01	0.988	cis	Severe COVID-19
10892_8	OSMR	Oncostatin-M-specific receptor subunit beta	Q99650	OSMR	chr5:38845858	Inverse variance weighted	2	0.099	0.161	5.361E-01	0.988	cis	Severe COVID-19
7211_2	RNase 1	Ribonuclease pancreatic	P07998	RNASE1	chr14:20802855	Inverse variance weighted	4	-0.100	0.162	5.361E-01	0.988	cis	Severe COVID-19
4906_35	Coagulation Factor V	Coagulation Factor V	P12259	F5	chr1:169586588	Inverse variance weighted	5	0.030	0.049	5.365E-01	0.988	cis	Severe COVID-19
18864_7	TRY3	Trypsin-3	P35030	PRSS3	chr9:33750679	Inverse variance weighted	4	-0.023	0.037	5.366E-01	0.988	cis	Severe COVID-19
14067_6	PKP2	Plakophilin-2	Q99959	PKP2	chr12:32896777	Inverse variance weighted	2	0.095	0.154	5.369E-01	0.988	cis	Severe COVID-19
5015_15	PAFAH	Platelet-activating factor acetylhydrolase	Q13093	PLA2G7	chr6:46735693	Inverse variance weighted	2	-0.109	0.177	5.374E-01	0.988	cis	Severe COVID-19
9278_9	SDF-1	Stromal cell-derived factor 1	P48061	CXCL12	chr10:44386493	Inverse variance weighted	2	0.103	0.167	5.375E-01	0.988	cis	Severe COVID-19
17756_69	DCTD	Deoxycytidylate deaminase	P32321	DCTD	chr4:182917936	Inverse variance weighted	2	0.169	0.274	5.382E-01	0.988	cis	Severe COVID-19
18340_2	Proteasome beta chain	Proteasome subunit beta type-4	P28070	PSMB4	chr1:151399560	Inverse variance weighted	4	-0.026	0.042	5.388E-01	0.988	cis	Severe COVID-19
6367_66	fibromodulin	fibromodulin	Q06828	FMOD	chr1:203351758	Inverse variance weighted	2	-0.052	0.084	5.399E-01	0.988	cis	Severe COVID-19
2979_8	ENA-78	C-X-C motif chemokine 5	P42830	CXCL5	chr4:73998677	Wald ratio	1	-0.244	0.399	5.402E-01	0.988	cis	Severe COVID-19
5708_1	LEAP2	Liver-expressed antimicrobial peptide 2	Q969E1	LEAP2	chr5:132873444	Inverse variance weighted	5	0.028	0.046	5.402E-01	0.988	cis	Severe COVID-19
4982_54	Elafin	Elafin	P19957	PI3	chr20:45174902	Inverse variance weighted	5	0.058	0.094	5.406E-01	0.988	cis	Severe COVID-19
4145_58	Neurotrophin-3	Neurotrophin-3	P20783	NTF3	chr12:5432108	Inverse variance weighted	2	-0.159	0.260	5.412E-01	0.988	cis	Severe COVID-19
15542_19	KCRU	Creatine kinase U-type, mitochondrial	P12532	CKMT1A	chr15:43692886	Wald ratio	1	-0.092	0.151	5.427E-01	0.988	cis	Severe COVID-19
3038_9	I-TAC	C-X-C motif chemokine 11	O14625	CXCL11	chr4:76041415	Inverse variance weighted	4	-0.059	0.097	5.435E-01	0.988	cis	Severe COVID-19
8970_9	RIPK2	Receptor-interacting serine/threonine-protein kinase 2	O43353	RIPK2	chr8:89757806	Wald ratio	1	-0.157	0.259	5.439E-01	0.988	cis	Severe COVID-19
4831_4	sL-Selectin	L-Selectin	P14151	SELL	chr1:169711702	Inverse variance weighted	7	-0.022	0.036	5.439E-01	0.988	cis	Severe COVID-19
4158_54	uPA	Urokinase-type plasminogen activator	P00749	PLAU	chr10:73909177	Inverse variance weighted	4	0.037	0.060	5.447E-01	0.988	cis	Severe COVID-19
8258_22	UXS1	UDP-glucuronic acid decarboxylase 1	Q8NBZ7	UXS1	chr2:106194301	Wald ratio	1	-0.177	0.293	5.447E-01	0.988	cis	Severe COVID-19
17341_89	THIC	Acetyl-CoA acetyltransferase, cytosolic	Q9BWD1	ACAT2	chr6:159762045	Inverse variance weighted	3	0.032	0.052	5.457E-01	0.988	cis	Severe COVID-19
12333_87	RPIA	Ribose-5-phosphate isomerase	P49247	RPIA	chr2:88691673	Inverse variance weighted	2	0.090	0.149	5.462E-01	0.988	cis	Severe COVID-19
18934_50	Tissue transglutaminase	Protein-glutamine gamma-glutamyltransferase 2	P21980	TGM2	chr20:38166578	Wald ratio	1	0.048	0.080	5.473E-01	0.988	cis	Severe COVID-19
14133_93	IL-1 sRII	Interleukin-1 receptor type 2	P27930	IL1R2	chr2:101991960	Inverse variance weighted	7	-0.040	0.066	5.476E-01	0.988	cis	Severe COVID-19
13624_17	NADK	NAD kinase	O95544	NADK	chr1:1780457	Wald ratio	1	0.070	0.116	5.481E-01	0.988	cis	Severe COVID-19
3727_35	PYY	Peptide YY	P10082	PYY	chr17:44004469	Wald ratio	1	-0.121	0.202	5.498E-01	0.988	cis	Severe COVID-19
8300_82	PEX14:N-term	Peroxisomal membrane protein PEX14:N-term	O75381	PEX14	chr1:10472288	Wald ratio	1	-0.217	0.363	5.502E-01	0.988	cis	Severe COVID-19
3728_52	Secretin	Secretin	P09683	SCT	chr11:627181	Wald ratio	1	-0.140	0.236	5.523E-01	0.988	cis	Severe COVID-19
15388_24	FcRIIIa	Low affinity immunoglobulin gamma Fc region receptor III-A	P08637	FCGR3A	chr1:161550968	Inverse variance weighted	6	-0.056	0.095	5.530E-01	0.988	cis	Severe COVID-19
14655_1	DJC17	DnaJ homolog subfamily C member 17	Q9NVM6	DNAJC17	chr15:40807478	Inverse variance weighted	2	-0.087	0.146	5.543E-01	0.988	cis	Severe COVID-19
9234_8	TWSG1	Twisted gastrulation protein homolog 1	Q9GZX9	TWSG1	chr18:9334767	Inverse variance weighted	3	-0.069	0.116	5.546E-01	0.988	cis	Severe COVID-19
3007_7	Siglec-9	Sialic acid-binding Ig-like lectin 9	Q9Y336	SIGLEC9	chr19:51124906	Inverse variance weighted	6	0.032	0.054	5.547E-01	0.988	cis	Severe COVID-19
2743_5	Sonic Hedgehog	Sonic hedgehog protein	Q15465	SHH	chr7:155812463	Inverse variance weighted	2	-0.099	0.168	5.551E-01	0.988	cis	Severe COVID-19
4480_59	C3b	Complement C3b	P01024	C3	chr19:6730562	Wald ratio	1	-0.202	0.343	5.555E-01	0.988	cis	Severe COVID-19
15644_1	Biotinidase	Biotinidase	P43251	BTD	chr3:15601341	Inverse variance weighted	9	0.018	0.031	5.556E-01	0.988	cis	Severe COVID-19
18398_1	AK1D1	3-oxo-5-beta-steroid 4-dehydrogenase	P51857	AKR1D1	chr7:138002324	Wald ratio	1	0.182	0.309	5.557E-01	0.988	cis	Severe COVID-19

13107_9	LYPD3	Ly6/PLAUR domain-containing protein 3	O95274	LYPD3	chr19:43465608	Wald ratio	1	0.098	0.166	5.559E-01	0.988	cis	Severe COVID-19
2860_19	Karyopherin-a2	Importin subunit alpha-1	P52292	KPNA2	chr17:68035636	Wald ratio	1	0.175	0.298	5.568E-01	0.988	cis	Severe COVID-19
2974_61	contactin-1	Contactin-1	Q12860	CNTN1	chr12:40692439	Inverse variance weighted	4	-0.030	0.051	5.588E-01	0.988	cis	Severe COVID-19
17391_10	SPF45	Splicing factor 45	Q96I25	RBM17	chr10:6089034	Inverse variance weighted	2	0.041	0.070	5.600E-01	0.988	cis	Severe COVID-19
9329_28	TRML1:ECD	Trem-like transcript 1 protein:Extracellular domain, Ig-like V-type domain	Q86YW5	TREML1	chr6:41154347	Inverse variance weighted	2	0.138	0.237	5.613E-01	0.988	cis	Severe COVID-19
6383_90	TLL1	Tolloid-like protein 1	O43897	TLL1	chr4:165873237	Inverse variance weighted	2	-0.161	0.277	5.614E-01	0.988	cis	Severe COVID-19
8916_32	STIM1:CD	Stromal interaction molecule 1:Cytoplasmic domain	Q13586	STIM1	chr11:3854527	Wald ratio	1	0.094	0.163	5.631E-01	0.988	cis	Severe COVID-19
17140_57	PDGFD	Platelet-derived growth factor D	Q9GZP0	PDGFD	chr11:104164379	Inverse variance weighted	6	-0.090	0.156	5.632E-01	0.988	cis	Severe COVID-19
19115_13	CMBL	Carboxymethylenebutenolidase homolog	Q96DG6	CMBL	chr5:10307902	Wald ratio	1	0.056	0.098	5.633E-01	0.988	cis	Severe COVID-19
10851_77	IL27B	Interleukin-27 subunit beta	Q14213	EBI3	chr19:4229523	Inverse variance weighted	7	-0.016	0.028	5.636E-01	0.988	cis	Severe COVID-19
9177_6	FAM3B	Protein FAM3B	P58499	FAM3B	chr21:41304212	Inverse variance weighted	7	-0.034	0.059	5.637E-01	0.988	cis	Severe COVID-19
6408_2	INHBC	Inhibin beta C chain	P55103	INHBC	chr12:57434784	Inverse variance weighted	2	-0.102	0.176	5.641E-01	0.988	cis	Severe COVID-19
15495_9	FOLR3	Folate receptor gamma	P41439	FOLR3	chr11:72114869	Inverse variance weighted	9	0.021	0.036	5.645E-01	0.988	cis	Severe COVID-19
5134_52	TIMD3	Hepatitis A virus cellular receptor 2	Q8TDQ0	HAVCR2	chr5:157142869	Inverse variance weighted	7	0.025	0.044	5.646E-01	0.988	cis	Severe COVID-19
9017_58	LPH	Lactase-phlorizin hydrolase	P09848	LCT	chr2:135837184	Inverse variance weighted	4	-0.018	0.031	5.648E-01	0.988	cis	Severe COVID-19
13122_19	FLRT2	Leucine-rich repeat transmembrane protein FLRT2	O43155	FLRT2	chr14:85530144	Inverse variance weighted	5	0.031	0.053	5.652E-01	0.988	cis	Severe COVID-19
15298_199	NETO1	Neuropilin and tolloid-like protein 1	Q8TDF5	NETO1	chr18:72868146	Inverse variance weighted	2	-0.175	0.305	5.659E-01	0.988	cis	Severe COVID-19
9837_60	NAD(P)H dehydrogenase	NAD(P)H dehydrogenase [quinone] 1	P15559	NQO1	chr16:69726668	Inverse variance weighted	9	-0.014	0.024	5.676E-01	0.988	cis	Severe COVID-19
6919_3	HBAZ	Hemoglobin subunit zeta	P02008	HBZ	chr16:142728	Inverse variance weighted	6	0.021	0.037	5.684E-01	0.988	cis	Severe COVID-19
11257_1	DHPR	Dihydropteridine reductase	P09417	QDPR	chr4:17512206	Inverse variance weighted	4	0.027	0.048	5.685E-01	0.988	cis	Severe COVID-19
12659_13	GTPB9	Obg-like ATPase 1	Q9NTK5	OLA1	chr2:174248599	Wald ratio	1	-0.063	0.111	5.704E-01	0.988	cis	Severe COVID-19
7905_30	HPT	Haptoglobin isoform 2	P00738	HP	chr16:72054505	Wald ratio	1	-0.034	0.060	5.710E-01	0.988	cis	Severe COVID-19
13090_17	S100A6	Protein S100-A6	P06703	S100A6	chr1:153536244	Inverse variance weighted	2	0.137	0.242	5.713E-01	0.988	cis	Severe COVID-19
9832_33	HGD	Homogentisate 1,2-dioxygenase	Q93099	HGD	chr3:120682269	Inverse variance weighted	2	-0.071	0.126	5.726E-01	0.988	cis	Severe COVID-19
11109_56	SVEP1:Sushi 15-18	Sushi, von Willebrand factor type A, EGF and pentraxin domain-containing protein 1:Sushi 15-18	Q4LDE5	SVEP1	chr9:110579880	Inverse variance weighted	4	0.088	0.157	5.739E-01	0.988	cis	Severe COVID-19
16882_27	PHP14	14 kDa phosphohistidine phosphatase	Q9NRX4	PHPT1	chr9:136848724	Inverse variance weighted	2	0.132	0.235	5.740E-01	0.988	cis	Severe COVID-19
9916_146	LRC4B:ECD	Leucine-rich repeat-containing protein 4B:Extracellular domain	Q9NT99	LRRC4B	chr19:50568435	Inverse variance weighted	2	0.125	0.222	5.743E-01	0.988	cis	Severe COVID-19
2968_61	TNFSF15	Tumor necrosis factor ligand superfamily member 15	O95150	TNFSF15	chr9:114806039	Wald ratio	1	-0.155	0.277	5.763E-01	0.988	cis	Severe COVID-19
12401_3	STALP	AMSH-like protease	Q96FJ0	STAMBPL1	chr10:88879734	Wald ratio	1	0.089	0.159	5.764E-01	0.988	cis	Severe COVID-19
18891_98	GBP2	Guanylate-binding protein 2	P32456	GBP2	chr1:89150456	Wald ratio	1	-0.110	0.198	5.769E-01	0.988	cis	Severe COVID-19
3234_23	URB	Coiled-coil domain-containing protein 80	Q76M96	CCDC80	chr3:112649530	Inverse variance weighted	2	0.092	0.164	5.770E-01	0.988	cis	Severe COVID-19
5542_22	NRP1	Neuropilin-1	O14786	NRP1	chr10:33336262	Inverse variance weighted	5	-0.025	0.044	5.772E-01	0.988	cis	Severe COVID-19
15585_304	fibulin 5	Fibulin-5	Q9UBX5	FBLN5	chr14:91947987	Wald ratio	1	-0.102	0.183	5.772E-01	0.988	cis	Severe COVID-19
18917_53	Pancreatic alpha-amylase	Pancreatic alpha-amylase	P04746	AMY2A	chr1:103617427	Inverse variance weighted	4	-0.129	0.231	5.772E-01	0.988	cis	Severe COVID-19
14006_36	GNMT	Glycine N-methyltransferase	Q14749	GNMT	chr6:42960754	Wald ratio	1	-0.074	0.132	5.774E-01	0.988	cis	Severe COVID-19
15316_262	TXN4B	Thioredoxin-like protein 4B	Q9NX01	TXNL4B	chr16:72094431	Wald ratio	1	0.096	0.173	5.792E-01	0.988	cis	Severe COVID-19
8958_51	CHL1	Neural cell adhesion molecule L1-like protein	O00533	CHL1	chr3:196763	Inverse variance weighted	6	-0.037	0.067	5.800E-01	0.988	cis	Severe COVID-19
11265_8	Retinal dehydrogenase 1	Retinal dehydrogenase 1	P00352	ALDH1A1	chr9:73080442	Wald ratio	1	0.115	0.208	5.802E-01	0.988	cis	Severe COVID-19
9769_48	DNER:ECD	Delta and Notch-like epidermal growth factor-related receptor:Extracellular domain	Q8NFT8	DNER	chr2:229714555	Inverse variance weighted	5	-0.039	0.071	5.803E-01	0.988	cis	Severe COVID-19
3025_50	bFGF	Fibroblast growth factor 2	P09038	FGF2	chr4:122826682	Inverse variance weighted	6	-0.021	0.038	5.826E-01	0.988	cis	Severe COVID-19
8841_65	CILP2	Cartilage intermediate layer protein 2	Q8IUL8	CILP2	chr19:19538248	Wald ratio	1	-0.117	0.213	5.838E-01	0.988	cis	Severe COVID-19
17435_43	ETFA	Electron transfer flavoprotein subunit alpha, mitochondrial	P13804	ETFA	chr15:76311730	Wald ratio	1	-0.161	0.295	5.850E-01	0.988	cis	Severe COVID-19
13125_45	Vitronectin	Vitronectin	P04004	VTN	chr17:28373091	Inverse variance weighted	9	0.016	0.030	5.852E-01	0.988	cis	Severe COVID-19
3339_33	TSP2	Thrombospondin-2	P35442	THBS2	chr6:169254050	Inverse variance weighted	7	-0.027	0.049	5.859E-01	0.988	cis	Severe COVID-19
18342_2	SERC	Phosphoserine aminotransferase	Q9Y617	PSAT1	chr9:78297125	Inverse variance weighted	2	-0.153	0.280	5.864E-01	0.988	cis	Severe COVID-19
9335_28	PSG9	Pregnancy-specific beta-1-glycoprotein 9	Q00887	PSG9	chr19:43269530	Wald ratio	1	-0.167	0.308	5.865E-01	0.988	cis	Severe COVID-19
8687_26	T106B	Transmembrane protein 106B	Q9NUM4	TMEM106B	chr7:12211270	Inverse variance weighted	2	-0.075	0.139	5.866E-01	0.988	cis	Severe COVID-19
17333_20	ACADM	Medium-chain specific acyl-CoA dehydrogenase, mitochondrial	P11310	ACADM	chr1:75724431	Wald ratio	1	-0.050	0.092	5.873E-01	0.988	cis	Severe COVID-19
18241_18	HEM6	Oxygen-dependent coproporphyrinogen-III oxidase, mitochondrial	P36551	CPOX	chr3:98593648	Inverse variance weighted	4	0.044	0.081	5.883E-01	0.988	cis	Severe COVID-19
4929_55	SHBG	Sex hormone-binding globulin	P04278	SHBG	chr17:7613946	Inverse variance weighted	2	0.081	0.150	5.885E-01	0.988	cis	Severe COVID-19
9595_11	B4GT2	Beta-1,4-galactosyltransferase 2	O60909	B4GALT2	chr1:43978943	Inverse variance weighted	4	0.029	0.054	5.889E-01	0.988	cis	Severe COVID-19
18183_3	ARH	Low density lipoprotein receptor adapter protein 1	Q5SW96	LDLRAP1	chr1:25543606	Inverse variance weighted	2	0.082	0.151	5.900E-01	0.988	cis	Severe COVID-19
17151_84	IRF-3	Interferon regulatory factor 3	Q14653	IRF3	chr19:49665875	Wald ratio	1	-0.108	0.201	5.907E-01	0.988	cis	Severe COVID-19
17398_55	HO-1	Heme oxygenase 1	P09601	HMOX1	chr22:35380361	Inverse variance weighted	3	-0.072	0.134	5.911E-01	0.988	cis	Severe COVID-19
11288_26	5NTC	Cytosolic purine 5'-nucleotidase	P49902	NT5C2	chr10:103277605	Inverse variance weighted	3	-0.059	0.110	5.920E-01	0.988	cis	Severe COVID-19
15444_45	SCCA2	Serpin B4	P48594	SERPINB4	chr18:63644256	Inverse variance weighted	3	-0.106	0.198	5.925E-01	0.988	cis	Severe COVID-19
13465_5	CCP1	Calcipressin-1	P53805	RCAN1	chr21:34615113	Wald ratio	1	0.137	0.257	5.926E-01	0.988	cis	Severe COVID-19
18218_48	CB032	CB1 cannabinoid receptor-interacting protein 1	Q96F85	CNRIP1	chr2:68320051	Inverse variance weighted	7	0.025	0.046	5.935E-01	0.988	cis	Severe COVID-19
11369_23	ADHX	Alcohol dehydrogenase class-3	P11766	ADH5	chr4:99088801	Inverse variance weighted	3	-0.130	0.244	5.935E-01	0.988	cis	Severe COVID-19
16606_85	Aldose reductase	Aldose reductase	P15121	AKR1B1	chr7:134459284	Inverse variance weighted	2	-0.066	0.124	5.941E-01	0.988	cis	Severe COVID-19
2750_3	Apo A-I	Apolipoprotein A-I	P02647	APOA1	chr11:116837622	Inverse variance weighted	3	-0.068	0.127	5.943E-01	0.988	cis	Severe COVID-19
9313_27	CBLN1	Cerebellin-1	P23435	CBLN1	chr16:49281838	Inverse variance weighted	9	-0.029	0.055	5.944E-01	0.988	cis	Severe COVID-19
14100_63	C1Qc	Complement C1q subcomponent subunit C	P02747	C1QC	chr1:22643014	Inverse variance weighted	9	-0.034	0.064	5.948E-01	0.988	cis	Severe COVID-19
15544_25	kallikrein 14	Kallikrein-14	Q9P0G3	KLK14	chr19:51084245	Inverse variance weighted	2	-0.131	0.247	5.957E-01	0.988	cis	Severe COVID-19
8874_53	CLN5:LD	Ceroid-lipofuscinosis neuronal protein 5:Lumenal domain	O75503	CLN5	chr13:76990660	Wald ratio	1	-0.083	0.156	5.959E-01	0.988	cis	Severe COVID-19
8360_169	NKp46	Natural cytotoxicity triggering receptor 1	O76036	NCR1	chr19:54906148	Wald ratio	1	0.086	0.162	5.960E-01	0.988	cis	Severe COVID-19
8394_56	RNase 2	Non-secretory ribonuclease	P10153	RNASE2	chr14:20955487	Wald ratio	1	-0.136	0.257	5.969E-01	0.988	cis	Severe COVID-19
13676_46	Inhibin bB chain	Inhibin beta B chain	P09529	INHBB	chr2:120346136	Inverse variance weighted	4	0.037	0.069	5.981E-01	0.988	cis	Severe COVID-19
11313_100	PHS	Pterin-4-alpha-carbinolamine dehydratase	P61457	PCBD1	chr10:70888565	Inverse variance weighted	5	0.048	0.091	5.981E-01	0.988	cis	Severe COVID-19
15530_33	EphB4	Ephrin type-B receptor 4	P54760	EPHB4	chr7:100827523	Wald ratio	1	-0.112	0.212	5.981E-01	0.988	cis	Severe COVID-19
10451_11	NUCB1	Nucleobindin-1	Q02818	NUCB1	chr19:48900050	Inverse variance weighted	3	-0.044	0.083	5.988E-01	0.988	cis	Severe COVID-19
5803_24	C3d	Complement C3d fragment	P01024	C3	chr19:6730562	Wald ratio	1	0.143	0.273	5.996E-01	0.988	cis	Severe COVID-19
3593_72	Caspase-3	Caspase-3	P42574	CASP3	chr4:184650062	Wald ratio	1	0.082	0.157	5.997E-01	0.988	cis	Severe COVID-19
3132_1	VEGF-C	Vascular endothelial growth factor C	P49767	VEGFC	chr4:176792922	Wald ratio	1	-0.098	0.186	5.999E-01	0.988	cis	Severe COVID-19
10754_113	Prokineticin-2	Prokineticin-2	Q9HC23	PROK2	chr3:71785206	Inverse variance weighted	6	-0.020	0.038	6.006E-01	0.988	cis	Severe COVID-19
9350_3	FSTL4	Follistatin-related protein 4	Q6MZW2	FSTL4	chr5:133612541	Inverse variance weighted	2	0.100	0.190	6.006E-01	0.988	cis	Severe COVID-19
8233_2	ITIH5	Inter-alpha-trypsin inhibitor heavy chain H5	Q86UX2	ITIH5	chr10:7666998	Inverse variance weighted	7	-0.024	0.045	6.009E-01	0.988	cis	Severe COVID-19
5798_3	BID	BH3-interacting domain death agonist	P55957	BID	chr22:17774770	Wald ratio	1	0.098	0.188	6.011E-01	0.988	cis	Severe COVID-19

6392_7	WISP-2	WNT1-inducible-signaling pathway protein 2	O76076	CCN5	chr20:44714844	Inverse variance weighted	5	0.031	0.059	6.015E-01	0.988	cis	Severe COVID-19
18185_118	ALDOB	Fructose-bisphosphate aldolase B	P05062	ALDOB	chr9:101449664	Inverse variance weighted	2	0.035	0.067	6.017E-01	0.988	cis	Severe COVID-19
2687_2	MIA	Melanoma-derived growth regulatory protein	Q16674	MIA	chr19:40771648	Inverse variance weighted	7	0.025	0.049	6.024E-01	0.988	cis	Severe COVID-19
5457_5	COLEC12	Collectin-12	Q5KU26	COLEC12	chr18:500722	Inverse variance weighted	3	0.045	0.086	6.025E-01	0.988	cis	Severe COVID-19
2948_58	Growth hormone receptor	Growth hormone receptor	P10912	GHR	chr5:42423439	Inverse variance weighted	4	-0.049	0.095	6.026E-01	0.988	cis	Severe COVID-19
4534_10	BSSP4	Brain-specific serine protease 4	Q9GZN4	PRSS22	chr16:2858170	Inverse variance weighted	4	-0.033	0.064	6.028E-01	0.988	cis	Severe COVID-19
5649_83	PSG4	Pregnancy-specific beta-1-glycoprotein 4	Q00888	PSG4	chr19:43207299	Inverse variance weighted	2	-0.085	0.163	6.034E-01	0.988	cis	Severe COVID-19
12524_18	SAT2	Diamine acetyltransferase 2	Q96F10	SAT2	chr17:7627876	Inverse variance weighted	3	-0.083	0.160	6.036E-01	0.988	cis	Severe COVID-19
11351_233	NHEJ1	Non-homologous end-joining factor 1	Q9H9Q4	NHEJ1	chr2:219160869	Inverse variance weighted	3	-0.063	0.122	6.045E-01	0.988	cis	Severe COVID-19
18873_8	CEAM8	Carcinoembryonic antigen-related cell adhesion molecule 8	P31997	CEACAM8	chr19:42595055	Wald ratio	1	-0.175	0.338	6.046E-01	0.988	cis	Severe COVID-19
6897_38	B3GA3	Galactosylgalactosylxylosylprotein 3-beta-glucuronosyltransferase 3	O94766	B3GAT3	chr11:62622154	Inverse variance weighted	3	0.052	0.100	6.055E-01	0.988	cis	Severe COVID-19
13671_40	Elastase	Neutrophil elastase	P08246	ELANE	chr19:851014	Inverse variance weighted	5	-0.039	0.075	6.059E-01	0.988	cis	Severe COVID-19
8310_6	U773	Zymogen granule protein 16 homolog B	Q96DA0	ZG16B	chr16:2830253	Wald ratio	1	0.049	0.095	6.068E-01	0.988	cis	Severe COVID-19
2771_35	IGFBP-1	Insulin-like growth factor-binding protein 1	P08833	IGFBP1	chr7:45888360	Wald ratio	1	0.102	0.199	6.070E-01	0.988	cis	Severe COVID-19
16323_8	NRX3A	Neurexin-3	Q9Y4C0	NRXN3	chr14:78170373	Wald ratio	1	0.161	0.313	6.071E-01	0.988	cis	Severe COVID-19
9545_156	Granzyme K	Granzyme K	P49863	GZMK	chr5:55024256	Wald ratio	1	0.053	0.103	6.071E-01	0.988	cis	Severe COVID-19
9026_40	BTNL8	Butyrophilin-like protein 8	Q6UX41	BTNL8	chr5:180899077	Inverse variance weighted	2	-0.047	0.092	6.075E-01	0.988	cis	Severe COVID-19
2827_23	Fractalkine/CX3CL-1	Fractalkine	P78423	CX3CL1	chr16:57372477	Wald ratio	1	-0.083	0.161	6.079E-01	0.988	cis	Severe COVID-19
9772_153	NLGN2:ECD	Neuroligin-2:Extracellular domain	Q8NFZ4	NLGN2	chr17:7404874	Inverse variance weighted	2	0.060	0.117	6.090E-01	0.988	cis	Severe COVID-19
17782_23	THIK	3-ketoacyl-CoA thiolase, peroxisomal	P09110	ACAA1	chr3:38137242	Inverse variance weighted	5	-0.036	0.070	6.092E-01	0.988	cis	Severe COVID-19
15626_223	Perlecan	Basement membrane-specific heparan sulfate proteoglycan core protein	P98160	HSPG2	chr1:21937310	Inverse variance weighted	4	-0.026	0.052	6.093E-01	0.988	cis	Severe COVID-19
12786_61	GDE5	Glycerophosphocholine phosphodiesterase GPCPD1	Q9NPB8	GPCPD1	chr20:5611006	Inverse variance weighted	2	0.090	0.176	6.102E-01	0.988	cis	Severe COVID-19
12813_18	EHBP1	EH domain-binding protein 1	Q8NDI1	EHBP1	chr2:62673851	Wald ratio	1	-0.155	0.303	6.102E-01	0.988	cis	Severe COVID-19
9316_67	WFDC1	WAP four-disulfide core domain protein 1	Q9HC57	WFDC1	chr16:84294846	Inverse variance weighted	5	0.021	0.041	6.102E-01	0.988	cis	Severe COVID-19
14623_26	SUMO3	Small ubiquitin-related modifier 3	P55854	SUMO3	chr21:44818779	Wald ratio	1	0.179	0.351	6.109E-01	0.988	cis	Severe COVID-19
17231_1	L-plastin	Plastin-2	P13796	LCP1	chr13:46211871	Inverse variance weighted	4	0.047	0.093	6.110E-01	0.988	cis	Severe COVID-19
17819_30	FAHD1	Acylpyruvase FAHD1, mitochondrial	Q6P587	FAHD1	chr16:1826967	Inverse variance weighted	3	0.174	0.343	6.116E-01	0.988	cis	Severe COVID-19
3151_6	IL-2 sRa	Interleukin-2 receptor subunit alpha	P01589	IL2RA	chr10:6062370	Wald ratio	1	0.084	0.165	6.117E-01	0.988	cis	Severe COVID-19
12633_3	PTN9	Tyrosine-protein phosphatase non-receptor type 9	P43378	PTPN9	chr15:75579315	Wald ratio	1	-0.132	0.261	6.119E-01	0.988	cis	Severe COVID-19
19372_7	MDGA2	MAM domain-containing glycosylphosphatidylinositol anchor protein 2	Q7Z553	MDGA2	chr14:47675605	Inverse variance weighted	10	-0.014	0.027	6.121E-01	0.988	cis	Severe COVID-19
15466_30	CO9A1	Collagen alpha-1(IX) chain	P20849	COL9A1	chr6:70303084	Inverse variance weighted	2	0.087	0.171	6.122E-01	0.988	cis	Severe COVID-19
11211_7	TBCE	Tubulin-specific chaperone E	Q15813	TBCE	chr1:235367360	Inverse variance weighted	2	-0.163	0.322	6.128E-01	0.988	cis	Severe COVID-19
3474_19	Thrombospondin-1	Thrombospondin-1	P07996	THBS1	chr15:39581079	Wald ratio	1	0.158	0.312	6.131E-01	0.988	cis	Severe COVID-19
3322_52	LRIG3	Leucine-rich repeats and immunoglobulin-like domains protein 3	Q6UXM1	LRIG3	chr12:58920504	Inverse variance weighted	6	-0.020	0.039	6.137E-01	0.988	cis	Severe COVID-19
11287_14	Cytochrome b5	Cytochrome b5	P00167	CYB5A	chr18:74291973	Wald ratio	1	0.194	0.384	6.139E-01	0.988	cis	Severe COVID-19
10612_18	PLOD3	Procollagen-lysine,2-oxoglutarate 5-dioxygenase 3	O60568	PLOD3	chr7:101218420	Inverse variance weighted	2	-0.077	0.152	6.150E-01	0.988	cis	Severe COVID-19
9863_1	Tropomyosin 4	Tropomyosin alpha-4 chain	P67936	TPM4	chr19:16067021	Wald ratio	1	0.101	0.200	6.151E-01	0.988	cis	Severe COVID-19
15483_377	Agrin	Agrin	O00468	AGRN	chr1:1020120	Inverse variance weighted	6	-0.049	0.098	6.174E-01	0.988	cis	Severe COVID-19
8973_23	FCRL4:ECD	Fc receptor-like protein 4:Extracellular domain	Q96PJ5	FCRL4	chr1:157598085	Inverse variance weighted	13	-0.027	0.053	6.193E-01	0.988	cis	Severe COVID-19
5680_54	OBP2B	Odorant-binding protein 2b	Q9NPH6	OBP2B	chr9:133209250	Inverse variance weighted	4	0.036	0.072	6.194E-01	0.988	cis	Severe COVID-19
8480_29	FBLN3	EGF-containing fibulin-like extracellular matrix protein 1	Q12805	EFEMP1	chr2:55924139	Inverse variance weighted	2	0.053	0.106	6.196E-01	0.988	cis	Severe COVID-19
16079_2	TEC	Tyrosine-protein kinase Tec	P42680	TEC	chr4:48269838	Inverse variance weighted	2	0.060	0.122	6.200E-01	0.988	cis	Severe COVID-19
15573_110	CSPG3	Neurocan core protein	O14594	NCAN	chr19:19211958	Inverse variance weighted	2	0.063	0.129	6.217E-01	0.988	cis	Severe COVID-19
17164_15	annexin IV	Annexin A4	P09525	ANXA4	chr2:69644425	Inverse variance weighted	2	0.063	0.128	6.217E-01	0.988	cis	Severe COVID-19
19129_15	MTHFSD	Methenyltetrahydrofolate synthase domain-containing protein	Q2M296	MTHFSD	chr16:86555235	Inverse variance weighted	4	0.023	0.047	6.219E-01	0.988	cis	Severe COVID-19
10903_50	STX8	Syntaxin-8	Q9UNK0	STX8	chr17:9576591	Wald ratio	1	0.118	0.241	6.233E-01	0.988	cis	Severe COVID-19
10835_25	A4GCT	Alpha-1,4-N-acetylglucosaminyltransferase	Q9UNA3	A4GNT	chr3:138132390	Wald ratio	1	-0.114	0.232	6.238E-01	0.988	cis	Severe COVID-19
3323_37	LRP8	Low-density lipoprotein receptor-related protein 8	Q14114	LRP8	chr1:53328469	Inverse variance weighted	2	0.037	0.075	6.239E-01	0.988	cis	Severe COVID-19
13416_8	T132D	Transmembrane protein 132D	Q14C87	TMEM132D	chr12:129904025	Inverse variance weighted	6	0.030	0.061	6.249E-01	0.988	cis	Severe COVID-19
7861_9	ROR2	Tyrosine-protein kinase transmembrane receptor ROR2	Q01974	ROR2	chr9:91950228	Inverse variance weighted	2	-0.046	0.094	6.250E-01	0.988	cis	Severe COVID-19
13113_7	Osteopontin	Osteopontin	P10451	SPP1	chr4:87975667	Inverse variance weighted	2	0.046	0.095	6.251E-01	0.988	cis	Severe COVID-19
18220_141	SRA1	Steroid receptor RNA activator 1	Q9HD15	SRA1	chr5:140557677	Inverse variance weighted	2	-0.087	0.178	6.251E-01	0.988	cis	Severe COVID-19
18206_18	ADH6	Alcohol dehydrogenase 6	P28332	ADH6	chr4:99219537	Wald ratio	1	0.114	0.233	6.252E-01	0.988	cis	Severe COVID-19
4153_11	alpha-1-antichymotrypsin complex	Alpha-1-antichymotrypsin complex	P07288 | P01011	KLK3 | SERPINA3	chr14:94612384	Inverse variance weighted	3	-0.071	0.146	6.260E-01	0.988	cis	Severe COVID-19
5852_6	S100A12	Protein S100-A12	P80511	S100A12	chr1:153375621	Inverse variance weighted	2	0.059	0.123	6.276E-01	0.988	cis	Severe COVID-19
9211_19	PEDF	Pigment epithelium-derived factor	P36955	SERPINF1	chr17:1762029	Inverse variance weighted	3	0.026	0.053	6.286E-01	0.988	cis	Severe COVID-19
12575_30	C1TC	C-1-tetrahydrofolate synthase, cytoplasmic	P11586	MTHFD1	chr14:64388031	Inverse variance weighted	2	0.074	0.153	6.296E-01	0.988	cis	Severe COVID-19
12334_25	cSHMT	Serine hydroxymethyltransferase, cytosolic	P34896	SHMT1	chr17:18363563	Inverse variance weighted	6	-0.015	0.032	6.298E-01	0.988	cis	Severe COVID-19
10419_1	SCAR5	Scavenger receptor class A member 5	Q6ZMJ2	SCARA5	chr8:27992673	Inverse variance weighted	5	0.035	0.073	6.304E-01	0.988	cis	Severe COVID-19
18235_16	PGP	Glycerol-3-phosphate phosphatase	A6NDG6	PGP	chr16:2214840	Wald ratio	1	0.083	0.173	6.316E-01	0.988	cis	Severe COVID-19
16015_19	ALT	Alanine aminotransferase 1	P24298	GPT	chr8:144502973	Wald ratio	1	-0.134	0.281	6.320E-01	0.988	cis	Severe COVID-19
17170_15	CALCB	Calcitonin gene-related peptide 2	P10092	CALCB	chr11:14904997	Inverse variance weighted	2	-0.037	0.077	6.321E-01	0.988	cis	Severe COVID-19
3292_75	CD48	CD48 antigen	P09326	CD48	chr1:160711831	Inverse variance weighted	5	0.037	0.078	6.322E-01	0.988	cis	Severe COVID-19
5301_7	Eotaxin	Eotaxin	P51671	CCL11	chr17:34285742	Wald ratio	1	-0.079	0.165	6.323E-01	0.988	cis	Severe COVID-19
3299_29	Contactin-5	Contactin-5	O94779	CNTN5	chr11:99020949	Inverse variance weighted	3	-0.054	0.112	6.325E-01	0.988	cis	Severe COVID-19
6373_54	DLK1	Protein delta homolog 1	P80370	DLK1	chr14:100725705	Inverse variance weighted	2	-0.110	0.231	6.327E-01	0.988	cis	Severe COVID-19
8289_8	GPNMB:ECD	Transmembrane glycoprotein NMB:Extracellular domain	Q14956	GPNMB	chr7:23235967	Inverse variance weighted	5	-0.030	0.064	6.328E-01	0.988	cis	Severe COVID-19
16913_8	RNT2	Ribonuclease T2	O00584	RNASET2	chr6:166957191	Inverse variance weighted	6	0.016	0.035	6.345E-01	0.990	cis	Severe COVID-19
17777_31	SDSL	Serine dehydratase-like	Q96GA7	SDSL	chr12:113422380	Inverse variance weighted	6	0.024	0.052	6.367E-01	0.990	cis	Severe COVID-19
15462_28	CD8A	T-cell surface glycoprotein CD8 alpha chain	P01732	CD8A	chr2:86808396	Inverse variance weighted	2	-0.023	0.048	6.367E-01	0.990	cis	Severe COVID-19
19143_38	NB5R2	NADH-cytochrome b5 reductase 2	Q6BCY4	CYB5R2	chr11:7677222	Inverse variance weighted	6	0.039	0.082	6.375E-01	0.990	cis	Severe COVID-19
7110_2	DJB11	DnaJ homolog subfamily B member 11	Q9UBS4	DNAJB11	chr3:186567403	Inverse variance weighted	2	-0.069	0.146	6.382E-01	0.990	cis	Severe COVID-19
9829_91	SULT 2A1	Bile salt sulfotransferase	Q06520	SULT2A1	chr19:47886315	Wald ratio	1	-0.045	0.095	6.383E-01	0.990	cis	Severe COVID-19
17675_17	ACO13	Acyl-coenzyme A thioesterase 13	Q9NPJ3	ACOT13	chr6:24667035	Inverse variance weighted	2	0.079	0.168	6.387E-01	0.990	cis	Severe COVID-19
11241_8	ARLY	Argininosuccinate lyase	P04424	ASL	chr7:66075800	Inverse variance weighted	3	-0.054	0.115	6.390E-01	0.990	cis	Severe COVID-19
2888_49	C7	Complement component C7	P10643	C7	chr5:40909492	Inverse variance weighted	5	-0.015	0.033	6.395E-01	0.990	cis	Severe COVID-19
8909_77	GNPI1	Glucosamine-6-phosphate isomerase 1	P46926	GNPDA1	chr5:142013041	Wald ratio	1	0.055	0.117	6.397E-01	0.990	cis	Severe COVID-19
8956_96	SREC-II:ECD	Scavenger receptor class F member 2:Extracellular domain	Q96GP6	SCARF2	chr22:20437826	Wald ratio	1	0.067	0.143	6.414E-01	0.991	cis	Severe COVID-19

11103_24	HSP 27	Heat shock protein beta-1	P04792	HSPB1	chr7:76302673	Inverse variance weighted	4	-0.020	0.043	6.435E-01	0.991	cis	Severe COVID-19
9459_7	Fas, soluble	Tumor necrosis factor receptor superfamily member 6	P25445	FAS	chr10:88953813	Inverse variance weighted	2	-0.129	0.278	6.444E-01	0.991	cis	Severe COVID-19
5646_20	RNAS6	Ribonuclease K6	Q93091	RNASE6	chr14:20781268	Inverse variance weighted	7	-0.014	0.030	6.467E-01	0.991	cis	Severe COVID-19
7945_10	Semaphorin-6A	Semaphorin-6A	Q9H2E6	SEMA6A	chr5:116574823	Inverse variance weighted	4	-0.044	0.095	6.470E-01	0.991	cis	Severe COVID-19
10396_6	Mcl-1	Induced myeloid leukemia cell differentiation protein Mcl-1	Q07820	MCL1	chr1:150579738	Wald ratio	1	-0.053	0.115	6.472E-01	0.991	cis	Severe COVID-19
16599_38	GPN1	GPN-loop GTPase 1	Q9HCN4	GPN1	chr2:27628247	Wald ratio	1	0.090	0.197	6.474E-01	0.991	cis	Severe COVID-19
9986_14	Neuropeptide W	Neuropeptide W	Q8N729	NPW	chr16:2009926	Inverse variance weighted	3	-0.029	0.064	6.481E-01	0.991	cis	Severe COVID-19
13481_24	TCEA2	Transcription elongation factor A protein 2	Q15560	TCEA2	chr20:64049836	Wald ratio	1	-0.047	0.103	6.485E-01	0.991	cis	Severe COVID-19
5648_28	CTRB2	Chymotrypsinogen B2	Q6GPI1	CTRB2	chr16:75207161	Inverse variance weighted	8	0.010	0.021	6.487E-01	0.991	cis	Severe COVID-19
17785_11	RCL	2'-deoxynucleoside 5'-phosphate N-hydrolase 1	O43598	DNPH1	chr6:43229481	Wald ratio	1	0.120	0.263	6.490E-01	0.991	cis	Severe COVID-19
2474_54	SAP	Serum amyloid P-component	P02743	APCS	chr1:159587826	Inverse variance weighted	3	-0.037	0.081	6.492E-01	0.991	cis	Severe COVID-19
9388_18	MCEE	Methylmalonyl-CoA epimerase, mitochondrial	Q96PE7	MCEE	chr2:71130239	Wald ratio	1	0.120	0.264	6.496E-01	0.991	cis	Severe COVID-19
5089_11	IL-7 Ra	Interleukin-7 receptor subunit alpha	P16871	IL7R	chr5:35852695	Wald ratio	1	0.138	0.304	6.500E-01	0.991	cis	Severe COVID-19
16322_10	PACAP	Marginal zone B- and B1-cell-specific protein	Q8WU39	MZB1	chr5:139390081	Inverse variance weighted	3	-0.059	0.131	6.510E-01	0.991	cis	Severe COVID-19
4294_16	Sphingosine kinase 1	Sphingosine kinase 1	Q9NYA1	SPHK1	chr17:76376584	Inverse variance weighted	2	0.073	0.162	6.513E-01	0.991	cis	Severe COVID-19
10714_7	ACE	Angiotensin-converting enzyme	P12821	ACE	chr17:63477061	Inverse variance weighted	11	-0.011	0.025	6.513E-01	0.991	cis	Severe COVID-19
3519_3	TARC	C-C motif chemokine 17	Q92583	CCL17	chr16:57404767	Inverse variance weighted	2	0.036	0.079	6.519E-01	0.991	cis	Severe COVID-19
10938_13	sLFA-3	Lymphocyte function-associated antigen 3	P19256	CD58	chr1:116571039	Inverse variance weighted	2	-0.086	0.191	6.527E-01	0.991	cis	Severe COVID-19
4496_60	MMP-12	Macrophage metalloelastase	P39900	MMP12	chr11:102874982	Inverse variance weighted	7	-0.013	0.029	6.533E-01	0.991	cis	Severe COVID-19
19361_78	MATN3	Matrilin-3	O15232	MATN3	chr2:20012668	Inverse variance weighted	12	0.014	0.031	6.533E-01	0.991	cis	Severe COVID-19
6431_68	PCYOX	Prenylcysteine oxidase 1	Q9UHG3	PCYOX1	chr2:70257386	Inverse variance weighted	6	0.012	0.026	6.537E-01	0.991	cis	Severe COVID-19
19367_34	D3D2	Enoyl-CoA delta isomerase 1, mitochondrial	P42126	ECI1	chr16:2252300	Inverse variance weighted	2	0.082	0.184	6.566E-01	0.995	cis	Severe COVID-19
9175_48	DSCAM	Down syndrome cell adhesion molecule	O60469	DSCAM	chr21:40847158	Inverse variance weighted	2	-0.045	0.101	6.576E-01	0.995	cis	Severe COVID-19
2609_59	Cystatin C	Cystatin-C	P01034	CST3	chr20:23638473	Inverse variance weighted	2	0.065	0.147	6.579E-01	0.995	cis	Severe COVID-19
11681_8	NUPL	Arf-GAP domain and FG repeat-containing protein 1	P52594	AGFG1	chr2:227472152	Wald ratio	1	-0.106	0.240	6.582E-01	0.995	cis	Severe COVID-19
16292_288	GIP	Gastric inhibitory polypeptide	P09681	GIP	chr17:48968596	Wald ratio	1	-0.183	0.419	6.616E-01	0.996	cis	Severe COVID-19
16035_8	VEGF sR3	Vascular endothelial growth factor receptor 3	P35916	FLT4	chr5:180649624	Inverse variance weighted	3	-0.048	0.110	6.633E-01	0.996	cis	Severe COVID-19
12376_85	p19-INK4d	Cyclin-dependent kinase 4 inhibitor D	P55273	CDKN2D	chr19:10569059	Wald ratio	1	-0.210	0.484	6.639E-01	0.996	cis	Severe COVID-19
3313_21	FCN2	Ficolin-2	Q15485	FCN2	chr9:134880810	Inverse variance weighted	3	-0.044	0.101	6.643E-01	0.996	cis	Severe COVID-19
7015_8	LIRB5	Leukocyte immunoglobulin-like receptor subfamily B member 5	O75023	LILRB5	chr19:54257301	Inverse variance weighted	6	0.011	0.025	6.646E-01	0.996	cis	Severe COVID-19
5275_28	VAV	Proto-oncogene vav	P15498	VAV1	chr19:6772708	Wald ratio	1	0.121	0.279	6.652E-01	0.996	cis	Severe COVID-19
16908_5	OMGP	Oligodendrocyte-myelin glycoprotein	P23515	OMG	chr17:31297539	Wald ratio	1	0.121	0.281	6.658E-01	0.996	cis	Severe COVID-19
14090_23	DEFI6	Differentially expressed in FDCP 6 homolog	Q9H4E7	DEF6	chr6:35297818	Inverse variance weighted	2	-0.036	0.084	6.671E-01	0.996	cis	Severe COVID-19
3045_72	PTN	Pleiotrophin	P21246	PTN	chr7:137343774	Inverse variance weighted	3	-0.031	0.072	6.679E-01	0.996	cis	Severe COVID-19
7918_114	Amylase, alpha 1A	Alpha-amylase 1	P04745	AMY1A	chr1:103655760	Inverse variance weighted	11	0.031	0.073	6.681E-01	0.996	cis	Severe COVID-19
10907_116	NTRI	Neurotrimin	Q9P121	NTM	chr11:131370478	Inverse variance weighted	2	0.101	0.235	6.681E-01	0.996	cis	Severe COVID-19
12641_3	I5P2	Type II inositol 1,4,5-trisphosphate 5-phosphatase	P32019	INPP5B	chr1:37947057	Inverse variance weighted	2	0.046	0.107	6.692E-01	0.996	cis	Severe COVID-19
9275_2	Siglec-5	Sialic acid-binding Ig-like lectin 5	O15389	SIGLEC5	chr19:51630401	Wald ratio	1	-0.122	0.286	6.705E-01	0.996	cis	Severe COVID-19
19293_6	VP26A	Vacuolar protein sorting-associated protein 26A	O75436	VPS26A	chr10:69123512	Wald ratio	1	-0.040	0.094	6.711E-01	0.996	cis	Severe COVID-19
3858_5	PPAC	Low molecular weight phosphotyrosine protein phosphatase	P24666	ACP1	chr2:264140	Inverse variance weighted	7	-0.013	0.031	6.725E-01	0.996	cis	Severe COVID-19
9339_204	FKBP2	Peptidyl-prolyl cis-trans isomerase FKBP2	P26885	FKBP2	chr11:64241003	Wald ratio	1	0.127	0.300	6.729E-01	0.996	cis	Severe COVID-19
6990_44	SDF2L	Stromal cell-derived factor 2-like protein 1	Q9HCN8	SDF2L1	chr22:21642302	Wald ratio	1	0.112	0.267	6.732E-01	0.996	cis	Severe COVID-19
13552_7	SWP70	Switch-associated protein 70	Q9UH65	SWAP70	chr11:9664077	Inverse variance weighted	5	-0.014	0.034	6.734E-01	0.996	cis	Severe COVID-19
7156_2	FUT10	Alpha-(1,3)-fucosyltransferase 10	Q6P4F1	FUT10	chr8:33473146	Inverse variance weighted	7	0.014	0.033	6.737E-01	0.996	cis	Severe COVID-19
18896_23	H6ST3	Heparan-sulfate 6-O-sulfotransferase 3	Q8IZP7	HS6ST3	chr13:96090107	Wald ratio	1	0.065	0.156	6.742E-01	0.996	cis	Severe COVID-19
16610_13	LRP10	Low-density lipoprotein receptor-related protein 10	Q7Z4F1	LRP10	chr14:22871740	Inverse variance weighted	3	-0.056	0.133	6.742E-01	0.996	cis	Severe COVID-19
13930_3	ABC3G	DNA dC->dU-editing enzyme APOBEC-3G	Q9HC16	APOBEC3G	chr22:39077067	Inverse variance weighted	2	0.048	0.113	6.749E-01	0.996	cis	Severe COVID-19
16825_20	ATX3	Ataxin-3	P54252	ATXN3	chr14:92106621	Inverse variance weighted	4	-0.024	0.057	6.756E-01	0.996	cis	Severe COVID-19
13427_66	MA1C1	Mannosyl-oligosaccharide 1,2-alpha-mannosidase IC	Q9NR34	MAN1C1	chr1:25616791	Inverse variance weighted	3	-0.041	0.099	6.756E-01	0.996	cis	Severe COVID-19
9867_23	F16P2	Fructose-1,6-bisphosphatase isozyme 2	O00757	FBP2	chr9:94593824	Wald ratio	1	0.091	0.219	6.761E-01	0.996	cis	Severe COVID-19
4563_61	PLCG1	1-phosphatidylinositol 4,5-bisphosphate phosphodiesterase gamma-1	P19174	PLCG1	chr20:41136960	Wald ratio	1	0.110	0.262	6.762E-01	0.996	cis	Severe COVID-19
16853_5	NRK1	Nicotinamide riboside kinase 1	Q9NWW6	NMRK1	chr9:75088217	Inverse variance weighted	2	0.051	0.122	6.767E-01	0.996	cis	Severe COVID-19
3719_2	p27Kip1	Cyclin-dependent kinase inhibitor 1B	P46527	CDKN1B	chr12:12685498	Wald ratio	1	-0.105	0.252	6.770E-01	0.996	cis	Severe COVID-19
8697_38	Glypican 1	Glypican-1	P35052	GPC1	chr2:240435663	Inverse variance weighted	6	0.015	0.037	6.772E-01	0.996	cis	Severe COVID-19
13692_154	WISP-1	WNT1-inducible-signaling pathway protein 1	O95388	CCN4	chr8:133191039	Inverse variance weighted	8	0.013	0.030	6.808E-01	0.996	cis	Severe COVID-19
15494_11	FGFP1	Fibroblast growth factor-binding protein 1	Q14512	FGFBP1	chr4:15938740	Wald ratio	1	0.114	0.278	6.810E-01	0.996	cis	Severe COVID-19
3499_77	IL-17B	Interleukin-17B	Q9UHF5	IL17B	chr5:149404202	Wald ratio	1	-0.086	0.209	6.814E-01	0.996	cis	Severe COVID-19
18338_26	IDH	Isocitrate dehydrogenase [NADP] cytoplasmic	O75874	IDH1	chr2:208266074	Inverse variance weighted	3	-0.035	0.085	6.815E-01	0.996	cis	Severe COVID-19
9883_29	Glyoxalase I	Lactoylglutathione lyase	Q04760	GLO1	chr6:38703145	Inverse variance weighted	3	-0.063	0.154	6.816E-01	0.996	cis	Severe COVID-19
11117_2	SPT20	Spermatogenesis-associated protein 20	Q8TB22	SPATA20	chr17:50543058	Inverse variance weighted	4	-0.025	0.060	6.830E-01	0.996	cis	Severe COVID-19
3449_58	Kallistatin	Kallistatin	P29622	SERPINA4	chr14:94561442	Inverse variance weighted	7	-0.024	0.059	6.835E-01	0.996	cis	Severe COVID-19
3336_50	TFPI	Tissue factor pathway inhibitor	P10646	TFPI	chr2:187565760	Wald ratio	1	-0.073	0.178	6.837E-01	0.996	cis	Severe COVID-19
11390_24	Carbonic Anhydrase VIII	Carbonic anhydrase-related protein	P35219	CA8	chr8:60281400	Inverse variance weighted	6	-0.015	0.038	6.845E-01	0.996	cis	Severe COVID-19
10818_36	ASM	Sphingomyelin phosphodiesterase	P17405	SMPD1	chr11:6390440	Inverse variance weighted	5	0.017	0.043	6.847E-01	0.996	cis	Severe COVID-19
2900_53	HCC-1	C-C motif chemokine 14	Q16627	CCL14	chr17:35987004	Inverse variance weighted	4	0.045	0.111	6.854E-01	0.996	cis	Severe COVID-19
7163_26	lacritin	Extracellular glycoprotein lacritin	Q9GZZ8	LACRT	chr12:54634895	Wald ratio	1	0.153	0.377	6.854E-01	0.996	cis	Severe COVID-19
8900_28	NEO1	Neogenin	Q92859	NEO1	chr15:73051710	Inverse variance weighted	3	-0.057	0.140	6.856E-01	0.996	cis	Severe COVID-19
14009_65	TNFAIP3	Tumor necrosis factor alpha-induced protein 3	P21580	TNFAIP3	chr6:137867214	Wald ratio	1	-0.038	0.095	6.873E-01	0.996	cis	Severe COVID-19
14208_3	RET7	Retinoid-binding protein 7	Q96R05	RBP7	chr1:9997206	Inverse variance weighted	2	-0.054	0.135	6.877E-01	0.996	cis	Severe COVID-19
2946_52	Factor D	Complement factor D	P00746	CFD	chr19:859453	Inverse variance weighted	6	-0.028	0.069	6.880E-01	0.996	cis	Severe COVID-19
2836_68	Lipocalin 2	Neutrophil gelatinase-associated lipocalin	P80188	LCN2	chr9:128149071	Wald ratio	1	-0.089	0.221	6.882E-01	0.996	cis	Severe COVID-19
16594_44	FAIM1	Fas apoptotic inhibitory molecule 1	Q9NVQ4	FAIM	chr3:138608606	Inverse variance weighted	7	-0.020	0.049	6.891E-01	0.996	cis	Severe COVID-19
9077_10	MA1A2	Mannosyl-oligosaccharide 1,2-alpha-mannosidase IB	O60476	MAN1A2	chr1:117367449	Inverse variance weighted	4	-0.035	0.088	6.899E-01	0.996	cis	Severe COVID-19
12422_143	LX15B	Arachidonate 15-lipoxygenase B	O15296	ALOX15B	chr17:8039034	Wald ratio	1	0.083	0.208	6.916E-01	0.996	cis	Severe COVID-19
19194_9	Histidyl-tRNA synthetase-related	D-tyrosyl-tRNA(Tyr) deacylase 1	Q8TEA8	DTD1	chr20:18587942	Wald ratio	1	0.030	0.077	6.917E-01	0.996	cis	Severe COVID-19
6382_17	MANBA	Beta-mannosidase	O00462	MANBA	chr4:102760994	Inverse variance weighted	5	-0.031	0.079	6.921E-01	0.996	cis	Severe COVID-19
11514_196	CD59	CD59 glycoprotein	P13987	CD59	chr11:33736479	Inverse variance weighted	3	-0.058	0.148	6.940E-01	0.996	cis	Severe COVID-19
5636_10	MFAP4	Microfibril-associated glycoprotein 4	P55083	MFAP4	chr17:19387190	Inverse variance weighted	2	0.029	0.075	6.940E-01	0.996	cis	Severe COVID-19

4984_83	Esterase D	S-formylglutathione hydrolase	P10768	ESD	chr13:46797420	Inverse variance weighted	3	-0.038	0.096	6.941E-01	0.996	cis	Severe COVID-19
12020_39	PMGE	Bisphosphoglycerate mutase	P07738	BPGM	chr7:134646811	Wald ratio	1	-0.190	0.484	6.945E-01	0.996	cis	Severe COVID-19
15635_4	SMOC2	SPARC-related modular calcium-binding protein 2	Q9H3U7	SMOC2	chr6:168441151	Inverse variance weighted	5	-0.019	0.047	6.952E-01	0.996	cis	Severe COVID-19
5703_26	NOE1	Noelin	Q99784	OLFM1	chr9:135075422	Inverse variance weighted	2	0.067	0.171	6.956E-01	0.996	cis	Severe COVID-19
12563_2	TFIP8	Tumor necrosis factor alpha-induced protein 8	O95379	TNFAIP8	chr5:119268692	Inverse variance weighted	2	0.049	0.125	6.961E-01	0.996	cis	Severe COVID-19
7926_13	SPIT3	Kunitz-type protease inhibitor 3	P49223	SPINT3	chr20:45515622	Inverse variance weighted	4	0.039	0.101	6.968E-01	0.996	cis	Severe COVID-19
8992_1	TMEM2	Transmembrane protein 2	Q9UHN6	CEMIP2	chr9:71816690	Inverse variance weighted	2	0.036	0.092	6.975E-01	0.996	cis	Severe COVID-19
3853_56	MDHC	Malate dehydrogenase, cytoplasmic	P40925	MDH1	chr2:63588609	Wald ratio	1	-0.085	0.218	6.978E-01	0.996	cis	Severe COVID-19
13059_33	RIFK	Riboflavin kinase	Q969G6	RFK	chr9:76394517	Inverse variance weighted	2	-0.059	0.151	6.982E-01	0.996	cis	Severe COVID-19
2789_26	MMP-7	Matrilysin	P09237	MMP7	chr11:102530750	Inverse variance weighted	4	-0.032	0.083	6.987E-01	0.996	cis	Severe COVID-19
15363_32	Apo A-V	Apolipoprotein A-V	Q6Q788	APOA5	chr11:116792420	Inverse variance weighted	8	-0.016	0.041	7.004E-01	0.996	cis	Severe COVID-19
4328_2	BOC	Brother of CDO	Q9BWV1	BOC	chr3:113211003	Inverse variance weighted	5	-0.024	0.063	7.006E-01	0.996	cis	Severe COVID-19
18880_81	Collagen Type III	Collagen Type III	P02461	COL3A1	chr2:188974373	Inverse variance weighted	2	-0.080	0.209	7.006E-01	0.996	cis	Severe COVID-19
5095_21	KI2L4	Killer cell immunoglobulin-like receptor 2DL4	Q99706	KIR2DL4	chr19:54803610	Inverse variance weighted	2	-0.055	0.143	7.012E-01	0.996	cis	Severe COVID-19
8766_29	LIRA5	Leukocyte immunoglobulin-like receptor subfamily A member 5	A6NI73	LILRA5	chr19:54313166	Wald ratio	1	-0.099	0.257	7.014E-01	0.996	cis	Severe COVID-19
7145_1	ITIH3	Inter-alpha-trypsin inhibitor heavy chain H3	Q06033	ITIH3	chr3:52794768	Inverse variance weighted	10	0.017	0.044	7.019E-01	0.996	cis	Severe COVID-19
5704_74	Granzyme M	Granzyme M	P51124	GZMM	chr19:544034	Inverse variance weighted	2	-0.087	0.227	7.026E-01	0.996	cis	Severe COVID-19
3855_56	Peroxiredoxin-1	Peroxiredoxin-1	Q06830	PRDX1	chr1:45542732	Inverse variance weighted	2	-0.049	0.128	7.029E-01	0.996	cis	Severe COVID-19
7141_21	MGT4B	Alpha-1,3-mannosyl-glycoprotein 4-beta-N-acetylglucosaminyltransferase B	Q9UQ53	MGAT4B	chr5:179806952	Inverse variance weighted	4	-0.016	0.042	7.053E-01	0.996	cis	Severe COVID-19
6234_74	VITRN	Vitrin	Q6UXI7	VIT	chr2:36696690	Inverse variance weighted	6	0.023	0.060	7.054E-01	0.996	cis	Severe COVID-19
7655_11	N-terminal pro-BNP	N-terminal pro-BNP	P16860	NPPB	chr1:11858945	Inverse variance weighted	3	0.058	0.152	7.055E-01	0.996	cis	Severe COVID-19
19267_14	GLO2	Hydroxyacylglutathione hydrolase, mitochondrial	Q16775	HAGH	chr16:1827157	Wald ratio	1	0.038	0.101	7.062E-01	0.996	cis	Severe COVID-19
17755_5	UGDH	UDP-glucose 6-dehydrogenase	O60701	UGDH	chr4:39528311	Inverse variance weighted	3	-0.026	0.069	7.065E-01	0.996	cis	Severe COVID-19
12329_21	KS6A1	Ribosomal protein S6 kinase alpha-1	Q15418	RPS6KA1	chr1:26529761	Wald ratio	1	0.058	0.153	7.070E-01	0.996	cis	Severe COVID-19
11480_1	Aldehyde dehydrogenase, class 3	Aldehyde dehydrogenase, dimeric NADP-preferring	P30838	ALDH3A1	chr17:19748943	Wald ratio	1	-0.047	0.125	7.085E-01	0.996	cis	Severe COVID-19
16763_11	LECT2	Leukocyte cell-derived chemotaxin-2	O14960	LECT2	chr5:135954983	Inverse variance weighted	8	0.019	0.051	7.100E-01	0.996	cis	Severe COVID-19
13112_179	FSTL1	Follistatin-related protein 1	Q12841	FSTL1	chr3:120450993	Inverse variance weighted	2	0.041	0.109	7.100E-01	0.996	cis	Severe COVID-19
5124_69	sICAM-5	Intercellular adhesion molecule 5	Q9UMF0	ICAM5	chr19:10289952	Inverse variance weighted	4	-0.106	0.286	7.116E-01	0.996	cis	Severe COVID-19
5671_1	Chymotrypsin	Chymotrypsinogen B	P17538	CTRB1	chr16:75218988	Inverse variance weighted	5	-0.036	0.097	7.124E-01	0.996	cis	Severe COVID-19
9216_100	PLXB2	Plexin-B2	O15031	PLXNB2	chr22:50307646	Inverse variance weighted	4	-0.021	0.057	7.133E-01	0.996	cis	Severe COVID-19
11104_13	YKL-40	Chitinase-3-like protein 1	P36222	CHI3L1	chr1:203186704	Inverse variance weighted	7	0.011	0.031	7.145E-01	0.996	cis	Severe COVID-19
18216_22	IL-11 RA	Interleukin-11 receptor subunit alpha	Q14626	IL11RA	chr9:34652162	Inverse variance weighted	3	-0.032	0.088	7.155E-01	0.996	cis	Severe COVID-19
6485_59	IGLL1	Immunoglobulin lambda-like polypeptide 1	P15814	IGLL1	chr22:23580302	Inverse variance weighted	4	0.018	0.050	7.155E-01	0.996	cis	Severe COVID-19
18432_32	RALB	Ras-related protein Ral-B	P11234	RALB	chr2:120240064	Wald ratio	1	0.034	0.093	7.157E-01	0.996	cis	Severe COVID-19
17702_53	UGT 1A1	UDP-glucuronosyltransferase 1-1	P22309	UGT1A1	chr2:233760270	Inverse variance weighted	2	0.037	0.103	7.161E-01	0.996	cis	Severe COVID-19
15326_64	GBP1	Guanylate-binding protein 1	P32455	GBP1	chr1:89065360	Inverse variance weighted	2	0.033	0.092	7.164E-01	0.996	cis	Severe COVID-19
17345_12	ZADH2	Prostaglandin reductase 3	Q8N4Q0	PTGR3	chr18:75209139	Wald ratio	1	-0.055	0.151	7.169E-01	0.996	cis	Severe COVID-19
5632_6	CRAC1	Cartilage acidic protein 1	Q9NQ79	CRTAC1	chr10:98030828	Inverse variance weighted	9	-0.014	0.040	7.170E-01	0.996	cis	Severe COVID-19
12764_3	ELMO1	Engulfment and cell motility protein 1	Q92556	ELMO1	chr7:37449223	Inverse variance weighted	3	0.047	0.129	7.176E-01	0.996	cis	Severe COVID-19
17746_77	FIS1	Mitochondrial fission 1 protein	Q9Y3D6	FIS1	chr7:101252316	Inverse variance weighted	2	0.152	0.420	7.179E-01	0.996	cis	Severe COVID-19
11540_37	FOXO3A	Forkhead box protein O3	O43524	FOXO3	chr6:108559835	Wald ratio	1	0.106	0.294	7.185E-01	0.996	cis	Severe COVID-19
3184_25	Coagulation Factor VII	Coagulation factor VII	P08709	F7	chr13:113105788	Inverse variance weighted	7	-0.012	0.033	7.203E-01	0.996	cis	Severe COVID-19
5005_4	MK12	Mitogen-activated protein kinase 12	P53778	MAPK12	chr22:50261716	Inverse variance weighted	2	0.037	0.103	7.210E-01	0.996	cis	Severe COVID-19
8465_52	Cathepsin H	Cathepsin H	P09668	CTSH	chr15:78949574	Inverse variance weighted	10	-0.011	0.032	7.215E-01	0.996	cis	Severe COVID-19
4278_14	Protein disulfide-isomerase	Protein disulfide-isomerase	P07237	P4HB	chr17:81860856	Wald ratio	1	-0.061	0.173	7.216E-01	0.996	cis	Severe COVID-19
16057_6	IGF-II receptor	Cation-independent mannose-6-phosphate receptor	P11717	IGF2R	chr6:159969082	Inverse variance weighted	6	0.022	0.063	7.223E-01	0.996	cis	Severe COVID-19
14094_29	HB-EGF	Heparin-binding EGF-like growth factor	Q99075	HBEGF	chr5:140346603	Wald ratio	1	0.130	0.366	7.223E-01	0.996	cis	Severe COVID-19
13740_51	sFRP-3	Secreted frizzled-related protein 3	Q92765	FRZB	chr2:182866637	Inverse variance weighted	8	-0.011	0.032	7.226E-01	0.996	cis	Severe COVID-19
15468_14	FHR1	Complement factor H-related protein 1	Q03591	CFHR1	chr1:196819731	Inverse variance weighted	6	-0.023	0.064	7.229E-01	0.996	cis	Severe COVID-19
19637_9	CRH	Corticoliberin	P06850	CRH	chr8:66178464	Wald ratio	1	0.087	0.245	7.230E-01	0.996	cis	Severe COVID-19
18319_7	ODPX	Pyruvate dehydrogenase protein X component, mitochondrial	O00330	PDHX	chr11:34915829	Inverse variance weighted	2	-0.117	0.330	7.235E-01	0.996	cis	Severe COVID-19
3622_33	LGMN	Legumain	Q99538	LGMN	chr14:92748679	Inverse variance weighted	3	0.046	0.131	7.239E-01	0.996	cis	Severe COVID-19
16304_6	LGR4	Leucine-rich repeat-containing G-protein coupled receptor 4	Q9BXB1	LGR4	chr11:27472790	Inverse variance weighted	2	-0.049	0.138	7.239E-01	0.996	cis	Severe COVID-19
4568_17	SLIK5	SLIT and NTRK-like protein 5	O94991	SLITRK5	chr13:87671371	Inverse variance weighted	4	0.041	0.116	7.247E-01	0.996	cis	Severe COVID-19
4960_72	annexin I	Annexin A1	P04083	ANXA1	chr9:73151865	Wald ratio	1	-0.040	0.115	7.258E-01	0.996	cis	Severe COVID-19
12687_2	DECR	2,4-dienoyl-CoA reductase, mitochondrial	Q16698	DECR1	chr8:90001405	Inverse variance weighted	2	-0.070	0.201	7.259E-01	0.996	cis	Severe COVID-19
9021_1	TIM-1	Hepatitis A virus cellular receptor 1	Q96D42	HAVCR1	chr5:157069396	Inverse variance weighted	6	-0.016	0.047	7.262E-01	0.996	cis	Severe COVID-19
12556_7	UBE2C	Ubiquitin-conjugating enzyme E2 C	O00762	UBE2C	chr20:45812576	Inverse variance weighted	2	-0.048	0.137	7.272E-01	0.996	cis	Severe COVID-19
2436_49	CXCL16, soluble	C-X-C motif chemokine 16	Q9H2A7	CXCL16	chr17:4739928	Inverse variance weighted	3	-0.036	0.102	7.281E-01	0.996	cis	Severe COVID-19
16872_248	MAAI	Maleylacetoacetate isomerase	O43708	GSTZ1	chr14:77320996	Inverse variance weighted	8	-0.008	0.023	7.292E-01	0.996	cis	Severe COVID-19
9826_135	Fragile histidine triad protein	Bis(5'-adenosyl)-triphosphatase	P49789	FHIT	chr3:61251459	Inverse variance weighted	2	-0.103	0.297	7.295E-01	0.996	cis	Severe COVID-19
3795_6	ADAM 9	Disintegrin and metalloproteinase domain-containing protein 9	Q13443	ADAM9	chr8:38996754	Wald ratio	1	0.126	0.367	7.305E-01	0.996	cis	Severe COVID-19
6375_75	XXLT1	Xyloside xylosyltransferase 1	Q8NBI6	XXYLT1	chr3:195271159	Inverse variance weighted	2	0.023	0.068	7.306E-01	0.996	cis	Severe COVID-19
17813_21	BPHL	Valacyclovir hydrolase	Q86WA6	BPHL	chr6:3118374	Inverse variance weighted	2	-0.110	0.319	7.310E-01	0.996	cis	Severe COVID-19
7083_74	MATN4	Matrilin-4	O95460	MATN4	chr20:45308529	Inverse variance weighted	3	0.033	0.096	7.317E-01	0.996	cis	Severe COVID-19
18215_5	THG1	Probable tRNA(His) guanylyltransferase	Q9NWX6	THG1L	chr5:157731420	Inverse variance weighted	3	-0.013	0.039	7.318E-01	0.996	cis	Severe COVID-19
17377_1	Aldose reductase-like C3	Aldo-keto reductase family 1 member C3	P42330	AKR1C3	chr10:5035354	Inverse variance weighted	4	0.046	0.135	7.319E-01	0.996	cis	Severe COVID-19
8327_26	DPEP2	Dipeptidase 2	Q9H4A9	DPEP2	chr16:68000586	Inverse variance weighted	2	0.037	0.107	7.324E-01	0.996	cis	Severe COVID-19
15343_337	Kininogen, HMW, Two Chain	Kininogen, HMW, Two Chain	P01042	KNG1	chr3:186717348	Inverse variance weighted	3	-0.089	0.260	7.331E-01	0.996	cis	Severe COVID-19
18878_15	GREM1	Gremlin-1	O60565	GREM1	chr15:32718004	Inverse variance weighted	8	-0.009	0.027	7.333E-01	0.996	cis	Severe COVID-19
5108_72	Notch-3	Neurogenic locus notch homolog protein 3	Q9UM47	NOTCH3	chr19:15200995	Wald ratio	1	0.060	0.175	7.336E-01	0.996	cis	Severe COVID-19
6965_19	CNTP2	Contactin-associated protein-like 2	Q9UHC6	CNTNAP2	chr7:146116002	Inverse variance weighted	5	-0.013	0.039	7.351E-01	0.996	cis	Severe COVID-19
6620_82	LIGO1	Leucine-rich repeat and immunoglobulin-like domain-containing nogo receptor-interacting protein 1	Q96FE5	LINGO1	chr15:77820900	Inverse variance weighted	2	0.106	0.313	7.354E-01	0.996	cis	Severe COVID-19
2381_52	C5	Complement C5	P01031	C5	chr9:121075195	Inverse variance weighted	2	0.041	0.123	7.370E-01	0.996	cis	Severe COVID-19
5737_61	SEM4D	Semaphorin-4D	Q92854	SEMA4D	chr9:89498130	Inverse variance weighted	6	-0.018	0.054	7.375E-01	0.996	cis	Severe COVID-19
5691_2	CRLD2	Cysteine-rich secretory protein LCCL domain-containing 2	Q9H0B8	CRISPLD2	chr16:84819985	Inverse variance weighted	6	0.027	0.080	7.377E-01	0.996	cis	Severe COVID-19
7228_2	SIA7F	Alpha-N-acetylgalactosaminide alpha-2,6-sialyltransferase 6	Q969X2	ST6GALNAC6	chr9:127905408	Inverse variance weighted	2	-0.083	0.247	7.383E-01	0.996	cis	Severe COVID-19
6909_40	MGAT2	Alpha-1,6-mannosyl-glycoprotein 2-beta-N-acetylglucosaminyltransferase	Q10469	MGAT2	chr14:49620799	Wald ratio	1	-0.035	0.106	7.388E-01	0.996	cis	Severe COVID-19

16055_3	complement factor H-related 5	Complement factor H-related protein 5	Q9BXR6	CFHR5	chr1:196975010	Inverse variance weighted	5	-0.020	0.061	7.398E-01	0.996	cis	Severe COVID-19
11424_4	FAAA	Fumarylacetoacetase	P16930	FAH	chr15:80152490	Inverse variance weighted	5	-0.010	0.032	7.413E-01	0.996	cis	Severe COVID-19
5036_50	TSG-6	Tumor necrosis factor-inducible gene 6 protein	P98066	TNFAIP6	chr2:151357592	Inverse variance weighted	9	-0.012	0.037	7.431E-01	0.996	cis	Severe COVID-19
3343_1	Aminoacylase-1	Aminoacylase-1	Q03154	ACY1	chr3:51983340	Wald ratio	1	-0.026	0.079	7.436E-01	0.996	cis	Severe COVID-19
5631_83	MOTI	Promotilin	P12872	MLN	chr6:33804003	Inverse variance weighted	6	0.015	0.047	7.445E-01	0.996	cis	Severe COVID-19
3028_36	Ck-b-8-1	Ck-beta-8-1	P55773	CCL23	chr17:36017972	Inverse variance weighted	4	-0.049	0.151	7.447E-01	0.996	cis	Severe COVID-19
15440_57	NEC2	Neuroendocrine convertase 2	P16519	PCSK2	chr20:17226107	Wald ratio	1	-0.058	0.178	7.456E-01	0.996	cis	Severe COVID-19
3077_66	Coagulation Factor Xa	Coagulation factor Xa	P00742	F10	chr13:113122799	Inverse variance weighted	2	-0.072	0.222	7.459E-01	0.996	cis	Severe COVID-19
18343_10	DECR2	Peroxisomal 2,4-dienoyl-CoA reductase	Q9NUI1	DECR2	chr16:401858	Inverse variance weighted	5	0.015	0.046	7.465E-01	0.996	cis	Severe COVID-19
18315_38	RTP4	Receptor-transporting protein 4	Q96DX8	RTP4	chr3:187368385	Inverse variance weighted	3	0.014	0.044	7.467E-01	0.996	cis	Severe COVID-19
19563_3	SEZ6L	Seizure 6-like protein	Q9BYH1	SEZ6L	chr22:26169462	Inverse variance weighted	3	0.043	0.133	7.469E-01	0.996	cis	Severe COVID-19
9816_37	ISOC1	Isochorismatase domain-containing protein 1	Q96CN7	ISOC1	chr5:129094749	Inverse variance weighted	2	0.051	0.158	7.473E-01	0.996	cis	Severe COVID-19
5480_49	RANTES	C-C motif chemokine 5	P13501	CCL5	chr17:35880793	Inverse variance weighted	2	-0.034	0.104	7.475E-01	0.996	cis	Severe COVID-19
15602_43	IL-6 sRa	Interleukin-6 receptor subunit alpha	P08887	IL6R	chr1:154405193	Inverse variance weighted	6	-0.019	0.058	7.475E-01	0.996	cis	Severe COVID-19
15336_7	SELM	Selenoprotein M	Q8WWX9	SELENOM	chr22:31120069	Wald ratio	1	0.091	0.284	7.483E-01	0.996	cis	Severe COVID-19
4559_64	KYNU	Kynureninase	Q16719	KYNU	chr2:142877657	Inverse variance weighted	4	-0.029	0.090	7.484E-01	0.996	cis	Severe COVID-19
11441_11	PYGL	Glycogen phosphorylase, liver form	P06737	PYGL	chr14:50944483	Inverse variance weighted	2	0.039	0.123	7.490E-01	0.996	cis	Severe COVID-19
10569_28	MFAP2	Microfibrillar-associated protein 2	P55001	MFAP2	chr1:16980632	Inverse variance weighted	3	0.064	0.201	7.491E-01	0.996	cis	Severe COVID-19
9204_33	Corticotropin-lipotropin	Pro-opiomelanocortin	P01189	POMC	chr2:25168903	Inverse variance weighted	3	-0.045	0.141	7.493E-01	0.996	cis	Severe COVID-19
15324_58	Ferritin light chain	Ferritin light chain	P02792	FTL	chr19:48965309	Wald ratio	1	-0.094	0.293	7.495E-01	0.996	cis	Severe COVID-19
6920_1	GFRAL	GDNF family receptor alpha-like	Q6UXV0	GFRAL	chr6:55327469	Inverse variance weighted	3	-0.027	0.084	7.499E-01	0.996	cis	Severe COVID-19
10490_3	RPN1:CD	Dolichyl-diphosphooligosaccharide--protein glycosyltransferase subunit 1:Cytoplasmic domain	P04843	RPN1	chr3:128681075	Wald ratio	1	0.030	0.094	7.508E-01	0.996	cis	Severe COVID-19
16070_7	WIF-1	Wnt inhibitory factor 1	Q9Y5W5	WIF1	chr12:65121305	Wald ratio	1	-0.079	0.250	7.518E-01	0.996	cis	Severe COVID-19
18172_71	ASF1A	Histone chaperone ASF1A	Q9Y294	ASF1A	chr6:118894152	Wald ratio	1	0.044	0.141	7.528E-01	0.996	cis	Severe COVID-19
13998_26	PURA1	Adenylosuccinate synthetase isozyme 1	Q8N142	ADSS1	chr14:104724229	Inverse variance weighted	3	-0.026	0.084	7.528E-01	0.996	cis	Severe COVID-19
15506_34	LRP12	Low-density lipoprotein receptor-related protein 12	Q9Y561	LRP12	chr8:104589258	Inverse variance weighted	2	0.067	0.215	7.533E-01	0.996	cis	Severe COVID-19
15509_2	NAG	Alpha-N-acetylglucosaminidase	P54802	NAGLU	chr17:42536241	Inverse variance weighted	6	0.010	0.032	7.540E-01	0.996	cis	Severe COVID-19
16309_30	Soggy-1	Dickkopf-like protein 1	Q9UK85	DKKL1	chr19:49361783	Wald ratio	1	0.119	0.381	7.554E-01	0.996	cis	Severe COVID-19
10366_11	PDGFRA	Platelet-derived growth factor receptor alpha	P16234	PDGFRA	chr4:54229280	Inverse variance weighted	3	0.036	0.115	7.557E-01	0.996	cis	Severe COVID-19
14047_78	BDNF	Brain-derived neurotrophic factor	P23560	BDNF	chr11:27722058	Inverse variance weighted	2	0.091	0.293	7.560E-01	0.996	cis	Severe COVID-19
3403_1	TPSB2	Tryptase beta-2	P20231	TPSB2	chr16:1230184	Inverse variance weighted	8	-0.011	0.035	7.562E-01	0.996	cis	Severe COVID-19
15364_101	Apo C-I	Apolipoprotein C-I	P02654	APOC1	chr19:44914247	Inverse variance weighted	5	0.034	0.109	7.575E-01	0.996	cis	Severe COVID-19
13719_19	PAK4	Serine/threonine-protein kinase PAK 4	O96013	PAK4	chr19:39125770	Wald ratio	1	0.097	0.315	7.590E-01	0.996	cis	Severe COVID-19
12549_33	PTGD2	Hematopoietic prostaglandin D synthase	O60760	HPGDS	chr4:94342876	Inverse variance weighted	9	-0.011	0.035	7.591E-01	0.996	cis	Severe COVID-19
2816_50	BCAM	Basal Cell Adhesion Molecule	P50895	BCAM	chr19:44809071	Inverse variance weighted	5	-0.013	0.043	7.606E-01	0.996	cis	Severe COVID-19
15570_99	Complement receptor type 2	Complement receptor type 2	P20023	CR2	chr1:207453024	Inverse variance weighted	3	0.032	0.105	7.616E-01	0.996	cis	Severe COVID-19
5459_33	CYTN	Cystatin-SN	P01037	CST1	chr20:23751268	Inverse variance weighted	5	0.033	0.109	7.626E-01	0.996	cis	Severe COVID-19
6448_36	Sema E	Semaphorin-3C	Q99985	SEMA3C	chr7:80922359	Inverse variance weighted	2	0.040	0.133	7.644E-01	0.996	cis	Severe COVID-19
7933_75	ADA22	Disintegrin and metalloproteinase domain-containing protein 22	Q9P0K1	ADAM22	chr7:87934143	Inverse variance weighted	4	-0.014	0.047	7.648E-01	0.996	cis	Severe COVID-19
4469_78	ST4S6	Carbohydrate sulfotransferase 15	Q7LFX5	CHST15	chr10:124093598	Inverse variance weighted	2	0.039	0.131	7.654E-01	0.996	cis	Severe COVID-19
2944_66	DAN	Neuroblastoma suppressor of tumorigenicity 1	P41271	NBL1	chr1:19596979	Wald ratio	1	-0.110	0.369	7.659E-01	0.996	cis	Severe COVID-19
5638_23	GT251	Procollagen galactosyltransferase 1	Q8NBJ5	COLGALT1	chr19:17555649	Inverse variance weighted	3	-0.030	0.101	7.668E-01	0.996	cis	Severe COVID-19
6470_19	fibulin 1	Fibulin-1	P23142	FBLN1	chr22:45502238	Inverse variance weighted	3	-0.025	0.084	7.671E-01	0.996	cis	Severe COVID-19
19558_10	LRP4	Low-density lipoprotein receptor-related protein 4	O75096	LRP4	chr11:46918642	Inverse variance weighted	6	0.018	0.062	7.672E-01	0.996	cis	Severe COVID-19
17761_2	NUDT5	ADP-sugar pyrophosphatase	Q9UKK9	NUDT5	chr10:12196144	Wald ratio	1	-0.058	0.196	7.681E-01	0.996	cis	Severe COVID-19
16890_37	ATL1	ADAMTS-like protein 1	Q8N6G6	ADAMTSL1	chr9:17906563	Inverse variance weighted	6	0.019	0.063	7.694E-01	0.996	cis	Severe COVID-19
18876_77	CHST4	Carbohydrate sulfotransferase 4	Q8NCG5	CHST4	chr16:71525233	Wald ratio	1	0.120	0.410	7.700E-01	0.996	cis	Severe COVID-19
4459_68	PCSK7	Proprotein convertase subtilisin/kexin type 7	Q16549	PCSK7	chr11:117232525	Inverse variance weighted	10	0.010	0.033	7.710E-01	0.996	cis	Severe COVID-19
17456_53	GOLM1	Golgi membrane protein 1	Q8NBJ4	GOLM1	chr9:86100173	Inverse variance weighted	6	0.028	0.096	7.715E-01	0.996	cis	Severe COVID-19
11212_7	TXND5	Thioredoxin domain-containing protein 5	Q8NBS9	TXNDC5	chr6:7910788	Inverse variance weighted	6	-0.016	0.055	7.723E-01	0.996	cis	Severe COVID-19
12635_9	TRDMT	tRNA (cytosine(38)-C(5))-methyltransferase	O14717	TRDMT1	chr10:17202054	Inverse variance weighted	4	-0.022	0.076	7.726E-01	0.996	cis	Severe COVID-19
12594_5	GRAN	Grancalcin	P28676	GCA	chr2:162318840	Inverse variance weighted	2	0.018	0.061	7.733E-01	0.996	cis	Severe COVID-19
2849_49	AIF1	Allograft inflammatory factor 1	P55008	AIF1	chr6:31615217	Inverse variance weighted	2	0.108	0.377	7.734E-01	0.996	cis	Severe COVID-19
7551_33	LRC32	Leucine-rich repeat-containing protein 32	Q14392	LRRC32	chr11:76670747	Wald ratio	1	-0.067	0.233	7.743E-01	0.996	cis	Severe COVID-19
3495_15	GCP-2	C-X-C motif chemokine 6	P80162	CXCL6	chr4:73836640	Inverse variance weighted	7	-0.019	0.066	7.743E-01	0.996	cis	Severe COVID-19
9901_28	EGLN1	Egl nine homolog 1	Q9GZT9	EGLN1	chr1:231422287	Inverse variance weighted	5	0.030	0.104	7.747E-01	0.996	cis	Severe COVID-19
13686_2	IL-5 Ra	Interleukin-5 receptor subunit alpha	Q01344	IL5RA	chr3:3126613	Inverse variance weighted	6	0.011	0.038	7.759E-01	0.996	cis	Severe COVID-19
8983_7	GOLM1	Golgi membrane protein 1	Q8NBJ4	GOLM1	chr9:86100173	Wald ratio	1	-0.086	0.302	7.766E-01	0.996	cis	Severe COVID-19
13982_33	RGS18	Regulator of G-protein signaling 18	Q9NS28	RGS18	chr1:192158462	Wald ratio	1	-0.071	0.252	7.770E-01	0.996	cis	Severe COVID-19
17751_68	CRBB1	Beta-crystallin B1	P53674	CRYBB1	chr22:26618027	Wald ratio	1	-0.023	0.081	7.775E-01	0.996	cis	Severe COVID-19
8299_66	LIRA4	Leukocyte immunoglobulin-like receptor subfamily A member 4	P59901	LILRA4	chr19:54339162	Inverse variance weighted	3	0.023	0.080	7.776E-01	0.996	cis	Severe COVID-19
11428_31	PDLI1	PDZ and LIM domain protein 1	O00151	PDLIM1	chr10:95291012	Wald ratio	1	0.059	0.209	7.784E-01	0.996	cis	Severe COVID-19
9848_22	Cyclin H	Cyclin-H	P51946	CCNH	chr5:87412930	Wald ratio	1	0.050	0.178	7.786E-01	0.996	cis	Severe COVID-19
18180_58	RT4I1	Reticulon-4-interacting protein 1, mitochondrial	Q8WWV3	RTN4IP1	chr6:106629498	Wald ratio	1	0.069	0.245	7.787E-01	0.996	cis	Severe COVID-19
6472_40	GP100	Melanocyte protein PMEL	P40967	PMEL	chr12:55973317	Inverse variance weighted	2	0.027	0.097	7.788E-01	0.996	cis	Severe COVID-19
17138_8	GST A1-1	Glutathione S-transferase A1	P08263	GSTA1	chr6:52803860	Inverse variance weighted	6	0.012	0.045	7.798E-01	0.996	cis	Severe COVID-19
17148_7	BLVRB	Flavin reductase (NADPH)	P30043	BLVRB	chr19:40465764	Inverse variance weighted	3	-0.019	0.070	7.801E-01	0.996	cis	Severe COVID-19
15333_11	SDF2	Stromal cell-derived factor 2	Q99470	SDF2	chr17:28662189	Wald ratio	1	0.053	0.192	7.807E-01	0.996	cis	Severe COVID-19
12366_16	CRGD	Gamma-crystallin D	P07320	CRYGD	chr2:208124524	Wald ratio	1	-0.039	0.141	7.813E-01	0.996	cis	Severe COVID-19
3320_49	IGFBP-7	Insulin-like growth factor-binding protein 7	Q16270	IGFBP7	chr4:57110385	Inverse variance weighted	5	-0.020	0.071	7.823E-01	0.996	cis	Severe COVID-19
15394_79	UNC5B	Netrin receptor UNC5B	Q8IZJ1	UNC5B	chr10:71212570	Inverse variance weighted	3	0.041	0.148	7.827E-01	0.996	cis	Severe COVID-19
12812_25	ACYP2	Acylphosphatase-2	P14621	ACYP2	chr2:53970838	Inverse variance weighted	3	0.018	0.066	7.829E-01	0.996	cis	Severe COVID-19
8398_277	RAR-responsive protein TIG1	Retinoic acid receptor responder protein 1	P49788	RARRES1	chr3:158732489	Inverse variance weighted	6	-0.008	0.030	7.835E-01	0.996	cis	Severe COVID-19
18330_7	PGM2	Phosphoglucomutase-2	Q96G03	PGM2	chr4:37826660	Inverse variance weighted	3	0.022	0.081	7.841E-01	0.996	cis	Severe COVID-19
5688_65	CBLN4	Cerebellin-4	Q9NTU7	CBLN4	chr20:56005519	Inverse variance weighted	5	0.023	0.086	7.848E-01	0.996	cis	Severe COVID-19
4548_4	Fucosyltransferase 3	Galactoside 3(4)-L-fucosyltransferase	P21217	FUT3	chr19:5851471	Inverse variance weighted	9	-0.015	0.056	7.852E-01	0.996	cis	Severe COVID-19
13384_110	FUMH	Fumarate hydratase, mitochondrial	P07954	FH	chr1:241519799	Wald ratio	1	0.030	0.112	7.856E-01	0.996	cis	Severe COVID-19
3583_54	Arylsulfatase A	Arylsulfatase A	P15289	ARSA	chr22:50628173	Wald ratio	1	-0.050	0.183	7.856E-01	0.996	cis	Severe COVID-19

12450_42	PMVK	Phosphomevalonate kinase	Q15126	PMVK	chr1:154936719	Inverse variance weighted	2	0.173	0.639	7.860E-01	0.996	cis	Severe COVID-19
6081_52	PCOC2	Procollagen C-endopeptidase enhancer 2	Q9UKZ9	PCOLCE2	chr3:142889206	Inverse variance weighted	10	-0.014	0.052	7.871E-01	0.996	cis	Severe COVID-19
4232_19	IGF-I sR	Insulin-like growth factor 1 receptor	P08069	IGF1R	chr15:98648539	Inverse variance weighted	2	0.092	0.344	7.883E-01	0.996	cis	Severe COVID-19
18312_68	NDRG3	Protein NDRG3	Q9UGV2	NDRG3	chr20:36746090	Inverse variance weighted	3	-0.073	0.273	7.884E-01	0.996	cis	Severe COVID-19
6433_57	FA20A	Pseudokinase FAM20A	Q96MK3	FAM20A	chr17:68601367	Inverse variance weighted	3	-0.029	0.108	7.885E-01	0.996	cis	Severe COVID-19
18387_7	suppression of tumorigenicity 13	Hsc70-interacting protein	P50502	ST13	chr22:40856639	Wald ratio	1	-0.075	0.280	7.891E-01	0.996	cis	Severe COVID-19
5939_42	TWEAK	Tumor necrosis factor ligand superfamily member 12	O43508	TNFSF12	chr17:7548508	Inverse variance weighted	3	-0.062	0.233	7.896E-01	0.996	cis	Severe COVID-19
5657_28	SIA4A	CMP-N-acetylneuraminate-beta-galactosamide-alpha-2,3-sialyltransferase 1	Q11201	ST3GAL1	chr8:133571940	Inverse variance weighted	2	0.040	0.150	7.900E-01	0.996	cis	Severe COVID-19
17514_48	RAB21	Ras-related protein Rab-21	Q9UL25	RAB21	chr12:71754863	Wald ratio	1	-0.042	0.160	7.911E-01	0.996	cis	Severe COVID-19
11214_40	DNJB9	DnaJ homolog subfamily B member 9	Q9UBS3	DNAJB9	chr7:108569867	Wald ratio	1	-0.077	0.290	7.916E-01	0.996	cis	Severe COVID-19
5620_13	AMD	Peptidyl-glycine alpha-amidating monooxygenase	P19021	PAM	chr5:102753981	Inverse variance weighted	10	0.008	0.029	7.921E-01	0.996	cis	Severe COVID-19
4152_58	Prekallikrein	Plasma kallikrein	P03952	KLKB1	chr4:186226438	Inverse variance weighted	4	0.022	0.083	7.922E-01	0.996	cis	Severe COVID-19
16616_137	ENOB	Beta-enolase	P13929	ENO3	chr17:4948092	Inverse variance weighted	2	-0.013	0.049	7.932E-01	0.996	cis	Severe COVID-19
13397_88	HBD-2	Beta-defensin 4A	O15263	DEFB4A	chr8:7894677	Inverse variance weighted	3	-0.036	0.139	7.934E-01	0.996	cis	Severe COVID-19
15613_16	LIPP	Pancreatic triacylglycerol lipase	P16233	PNLIP	chr10:116545931	Wald ratio	1	-0.083	0.320	7.942E-01	0.996	cis	Severe COVID-19
10558_26	PCDH9	Protocadherin-9	Q9HC56	PCDH9	chr13:67230445	Inverse variance weighted	4	0.016	0.062	7.947E-01	0.996	cis	Severe COVID-19
6451_64	ASPN	Asporin	Q9BXN1	ASPN	chr9:92482506	Inverse variance weighted	8	-0.016	0.062	7.958E-01	0.996	cis	Severe COVID-19
13088_397	BTC	Betacellulin	P35070	BTC	chr4:74794523	Inverse variance weighted	2	-0.023	0.089	7.966E-01	0.996	cis	Severe COVID-19
9197_4	LEG9	Galectin-9	O00182	LGALS9	chr17:27629798	Wald ratio	1	-0.035	0.136	7.970E-01	0.996	cis	Severe COVID-19
2913_1	MPIF-1	C-C motif chemokine 23	P55773	CCL23	chr17:36017972	Inverse variance weighted	2	-0.047	0.182	7.971E-01	0.996	cis	Severe COVID-19
9282_12	CRIS2	Cysteine-rich secretory protein 2	P16562	CRISP2	chr6:49713590	Inverse variance weighted	6	0.014	0.056	7.978E-01	0.996	cis	Severe COVID-19
8428_102	NTRI	Neurotrimin	Q9P121	NTM	chr11:131370478	Inverse variance weighted	4	-0.032	0.126	7.982E-01	0.996	cis	Severe COVID-19
17712_7	IDI1	Isopentenyl-diphosphate Delta-isomerase 1	Q13907	IDI1	chr10:1049119	Wald ratio	1	0.055	0.214	7.983E-01	0.996	cis	Severe COVID-19
9369_174	NGL1	Leucine-rich repeat-containing protein 4C	Q9HCJ2	LRRC4C	chr11:41459773	Inverse variance weighted	3	0.038	0.150	7.983E-01	0.996	cis	Severe COVID-19
5346_24	CPNE1:C2, 1 and 2	Copine-1:Ca2+-dependent membrane-targeting module domains 1 and 2	Q99829	CPNE1	chr20:35664956	Inverse variance weighted	9	-0.008	0.031	7.998E-01	0.996	cis	Severe COVID-19
5456_59	CNDP1	Beta-Ala-His dipeptidase	Q96KN2	CNDP1	chr18:74534500	Inverse variance weighted	6	-0.013	0.051	8.007E-01	0.996	cis	Severe COVID-19
4467_49	SPARCL1	SPARC-like protein 1	Q14515	SPARCL1	chr4:87531061	Inverse variance weighted	6	0.013	0.052	8.010E-01	0.996	cis	Severe COVID-19
2780_35	Lactoferrin	Lactotransferrin	P02788	LTF	chr3:46485234	Inverse variance weighted	3	-0.030	0.119	8.013E-01	0.996	cis	Severe COVID-19
13109_82	NEGR1	Neuronal growth regulator 1	Q7Z3B1	NEGR1	chr1:72282539	Wald ratio	1	-0.058	0.232	8.019E-01	0.996	cis	Severe COVID-19
9360_33	EDIL3	EGF-like repeat and discoidin I-like domain-containing protein 3	O43854	EDIL3	chr5:84384880	Inverse variance weighted	2	-0.050	0.199	8.024E-01	0.996	cis	Severe COVID-19
5029_3	SEPR	Prolyl endopeptidase FAP	Q12884	FAP	chr2:162245151	Inverse variance weighted	5	0.048	0.191	8.027E-01	0.996	cis	Severe COVID-19
8007_19	Cathepsin B	Cathepsin B	P07858	CTSB	chr8:11869533	Inverse variance weighted	6	0.008	0.032	8.028E-01	0.996	cis	Severe COVID-19
13720_95	Proteinase-3	Myeloblastin	P24158	PRTN3	chr19:840999	Inverse variance weighted	6	0.010	0.040	8.028E-01	0.996	cis	Severe COVID-19
9599_6	PIANP	PILR alpha-associated neural protein	Q8IYJ0	PIANP	chr12:6700815	Wald ratio	1	-0.082	0.328	8.031E-01	0.996	cis	Severe COVID-19
5452_71	ASGR1	Asialoglycoprotein receptor 1	P07306	ASGR1	chr17:7179564	Inverse variance weighted	2	0.046	0.187	8.055E-01	0.996	cis	Severe COVID-19
13116_25	CD177	CD177 antigen	Q8N6Q3	CD177	chr19:43353686	Inverse variance weighted	7	-0.008	0.032	8.076E-01	0.996	cis	Severe COVID-19
3329_14	PGRP-S	Peptidoglycan recognition protein 1	O75594	PGLYRP1	chr19:46023053	Inverse variance weighted	4	-0.018	0.076	8.079E-01	0.996	cis	Severe COVID-19
13472_35	HDHD2	Haloacid dehalogenase-like hydrolase domain-containing protein 2	Q9H0R4	HDHD2	chr18:47150500	Inverse variance weighted	3	0.020	0.083	8.082E-01	0.996	cis	Severe COVID-19
8099_42	SPON2	Spondin-2	Q9BUD6	SPON2	chr4:1208962	Inverse variance weighted	6	-0.020	0.085	8.095E-01	0.996	cis	Severe COVID-19
9326_33	ITI heavy chain H2	Inter-alpha-trypsin inhibitor heavy chain H2	P19823	ITIH2	chr10:7703316	Inverse variance weighted	9	0.015	0.064	8.097E-01	0.996	cis	Severe COVID-19
4996_66	HRG	Histidine-rich glycoprotein	P04196	HRG	chr3:186660216	Inverse variance weighted	4	0.014	0.058	8.103E-01	0.996	cis	Severe COVID-19
12560_9	NT5C	5'(3')-deoxyribonucleotidase, cytosolic type	Q8TCD5	NT5C	chr17:75131757	Inverse variance weighted	5	0.009	0.037	8.105E-01	0.996	cis	Severe COVID-19
9525_1	PTK7	Inactive tyrosine-protein kinase 7	Q13308	PTK7	chr6:43076307	Inverse variance weighted	2	0.063	0.263	8.105E-01	0.996	cis	Severe COVID-19
4374_45	MIC-1	Growth/differentiation factor 15	Q99988	GDF15	chr19:18374731	Inverse variance weighted	7	-0.013	0.055	8.114E-01	0.996	cis	Severe COVID-19
8080_24	PSMP	Prostate-associated microseminoprotein	Q1L6U9	MSMP	chr9:35756613	Wald ratio	1	-0.054	0.226	8.114E-01	0.996	cis	Severe COVID-19
6252_62	Secretoglobin family 3A member 1	Secretoglobin family 3A member 1	Q96QR1	SCGB3A1	chr5:180591499	Inverse variance weighted	4	0.017	0.071	8.127E-01	0.996	cis	Severe COVID-19
15384_15	KLOTHO	Klotho	Q9UEF7	KL	chr13:33016423	Inverse variance weighted	4	0.027	0.113	8.127E-01	0.996	cis	Severe COVID-19
6444_15	PSG3	Pregnancy-specific beta-1-glycoprotein 3	Q16557	PSG3	chr19:42740481	Inverse variance weighted	6	-0.016	0.069	8.130E-01	0.996	cis	Severe COVID-19
2590_69	ROR1	Inactive tyrosine-protein kinase transmembrane receptor ROR1	Q01973	ROR1	chr1:63774017	Inverse variance weighted	3	0.015	0.065	8.131E-01	0.996	cis	Severe COVID-19
13983_27	QOR	Quinone oxidoreductase	Q08257	CRYZ	chr1:74733408	Inverse variance weighted	7	0.008	0.035	8.135E-01	0.996	cis	Severe COVID-19
15387_44	Neuropilin-2	Neuropilin-2	O60462	NRP2	chr2:205681990	Inverse variance weighted	4	-0.019	0.082	8.136E-01	0.996	cis	Severe COVID-19
14151_4	UCRP	Ubiquitin-like protein ISG15	P05161	ISG15	chr1:1001138	Inverse variance weighted	2	0.011	0.045	8.143E-01	0.996	cis	Severe COVID-19
12657_2	FCL	GDP-L-fucose synthase	Q13630	GFUS	chr8:143618048	Wald ratio	1	-0.083	0.353	8.147E-01	0.996	cis	Severe COVID-19
11969_5	S100A2	Protein S100-A2	P29034	S100A2	chr1:153567890	Wald ratio	1	-0.087	0.374	8.153E-01	0.996	cis	Severe COVID-19
9201_13	Transgelin-2	Transgelin-2	P37802	TAGLN2	chr1:159925507	Wald ratio	1	0.077	0.330	8.154E-01	0.996	cis	Severe COVID-19
8024_64	TPST2	Protein-tyrosine sulfotransferase 2	O60704	TPST2	chr22:26596717	Inverse variance weighted	2	0.030	0.129	8.159E-01	0.996	cis	Severe COVID-19
2681_23	HGF	Hepatocyte growth factor	P14210	HGF	chr7:81770438	Wald ratio	1	0.047	0.202	8.166E-01	0.996	cis	Severe COVID-19
3033_57	Galectin-2	Galectin-2	P05162	LGALS2	chr22:37582616	Inverse variance weighted	2	0.025	0.108	8.174E-01	0.996	cis	Severe COVID-19
18236_3	LEGL	Galectin-related protein	Q3ZCW2	LGALSL	chr2:64453969	Wald ratio	1	0.045	0.194	8.184E-01	0.996	cis	Severe COVID-19
7050_5	NEGR1	Neuronal growth regulator 1	Q7Z3B1	NEGR1	chr1:72282539	Wald ratio	1	0.078	0.341	8.188E-01	0.996	cis	Severe COVID-19
9713_67	PGFRL	Platelet-derived growth factor receptor-like protein	Q15198	PDGFRL	chr8:17576433	Inverse variance weighted	6	-0.010	0.043	8.193E-01	0.996	cis	Severe COVID-19
9470_15	MET24	Methyltransferase-like protein 24	Q5JXM2	METTL24	chr6:110358349	Inverse variance weighted	4	-0.027	0.120	8.209E-01	0.996	cis	Severe COVID-19
3535_84	DKK1	Dickkopf-related protein 1	O94907	DKK1	chr10:52314281	Inverse variance weighted	5	-0.019	0.083	8.222E-01	0.996	cis	Severe COVID-19
17490_4	SH3BGRL3-like protein	SH3 domain-binding glutamic acid-rich-like protein 3	Q9H299	SH3BGRL3	chr1:26280086	Inverse variance weighted	3	0.013	0.057	8.223E-01	0.996	cis	Severe COVID-19
15376_134	CATE	Cathepsin E	P14091	CTSE	chr1:206023909	Inverse variance weighted	4	-0.010	0.045	8.223E-01	0.996	cis	Severe COVID-19
8039_41	F177A	Protein FAM177A1	Q8N128	FAM177A1	chr14:35045117	Inverse variance weighted	6	0.010	0.043	8.234E-01	0.996	cis	Severe COVID-19
7208_60	MGT4C	Alpha-1,3-mannosyl-glycoprotein 4-beta-N-acetylglucosaminyltransferase C	Q9UBM8	MGAT4C	chr12:86838904	Wald ratio	1	0.055	0.247	8.235E-01	0.996	cis	Severe COVID-19
19338_3	LZIC	Protein LZIC	Q8WZA0	LZIC	chr1:9943407	Wald ratio	1	-0.098	0.442	8.242E-01	0.996	cis	Severe COVID-19
9839_148	Tirap	Toll/interleukin-1 receptor domain-containing adapter protein	P58753	TIRAP	chr11:126282497	Inverse variance weighted	3	-0.020	0.089	8.249E-01	0.996	cis	Severe COVID-19
15486_126	ABP1	Amiloride-sensitive amine oxidase [copper-containing]	P19801	AOC1	chr7:150824627	Inverse variance weighted	10	-0.006	0.026	8.253E-01	0.996	cis	Severe COVID-19
19590_46	SP-D	Pulmonary surfactant-associated protein D	P35247	SFTPD	chr10:79982614	Inverse variance weighted	6	-0.011	0.049	8.254E-01	0.996	cis	Severe COVID-19
15521_4	Alcadein alpha-1	Calsyntenin-1	O94985	CLSTN1	chr1:9823984	Inverse variance weighted	6	-0.005	0.022	8.257E-01	0.996	cis	Severe COVID-19
12381_26	CBR1	Carbonyl reductase [NADPH] 1	P16152	CBR1	chr21:36069941	Inverse variance weighted	5	-0.016	0.075	8.259E-01	0.996	cis	Severe COVID-19
4886_3	MCP-3	C-C motif chemokine 7	P80098	CCL7	chr17:34270221	Inverse variance weighted	4	-0.014	0.062	8.268E-01	0.997	cis	Severe COVID-19
19124_9	UBCP1	Ubiquitin-like domain-containing CTD phosphatase 1	Q8WVY7	UBLCP1	chr5:159263290	Inverse variance weighted	2	-0.027	0.123	8.286E-01	0.998	cis	Severe COVID-19
4471_50	TGM3	Protein-glutamine gamma-glutamyltransferase E	Q08188	TGM3	chr20:2296001	Inverse variance weighted	4	-0.009	0.040	8.300E-01	0.998	cis	Severe COVID-19
15453_3	a1-Microglobulin	Alpha-1-microglobulin	P02760	AMBP	chr9:114078328	Inverse variance weighted	2	-0.034	0.159	8.311E-01	0.998	cis	Severe COVID-19
8304_50	OPG	Tumor necrosis factor receptor superfamily member 11B	O00300	TNFRSF11B	chr8:118951885	Inverse variance weighted	4	0.017	0.080	8.343E-01	0.998	cis	Severe COVID-19

3894_15	NAGK	N-acetyl-D-glucosamine kinase	Q9UJ70	NAGK	chr2:71064344	Inverse variance weighted	3	0.017	0.080	8.347E-01	0.998	cis	Severe COVID-19
19236_24	TCP4	Activated RNA polymerase II transcriptional coactivator p15	P53999	SUB1	chr5:32531633	Wald ratio	1	0.067	0.321	8.349E-01	0.998	cis	Severe COVID-19
10521_10	MXRA8:ECD	Matrix-remodeling-associated protein 8:Extracellular domain	Q9BRK3	MXRA8	chr1:1361777	Wald ratio	1	0.033	0.159	8.353E-01	0.998	cis	Severe COVID-19
13621_31	AP-2A2	AP-2 complex subunit alpha-2	O94973	AP2A2	chr11:924881	Wald ratio	1	-0.050	0.241	8.355E-01	0.998	cis	Severe COVID-19
10075_75	ACBD6	Acyl-CoA-binding domain-containing protein 6	Q9BR61	ACBD6	chr1:180502954	Wald ratio	1	0.073	0.349	8.357E-01	0.998	cis	Severe COVID-19
3488_64	Catalase	Catalase	P04040	CAT	chr11:34438934	Inverse variance weighted	3	0.035	0.170	8.357E-01	0.998	cis	Severe COVID-19
7059_14	LIRA6	Leukocyte immunoglobulin-like receptor subfamily A member 6	Q6PI73	LILRA6	chr19:54242791	Inverse variance weighted	7	0.018	0.089	8.367E-01	0.998	cis	Severe COVID-19
9018_38	PCD10:ECD	Protocadherin-10:Extracellular domain	Q9P2E7	PCDH10	chr4:133149294	Inverse variance weighted	2	0.023	0.113	8.370E-01	0.998	cis	Severe COVID-19
8957_72	XTP3B	Endoplasmic reticulum lectin 1	Q96DZ1	ERLEC1	chr2:53787009	Wald ratio	1	0.045	0.221	8.372E-01	0.998	cis	Severe COVID-19
5727_35	B3GLT	Beta-1,3-glucosyltransferase	Q6Y288	B3GLCT	chr13:31199975	Inverse variance weighted	2	-0.018	0.089	8.386E-01	0.998	cis	Severe COVID-19
5604_30	HPSE	Heparanase	Q9Y251	HPSE	chr4:83335153	Inverse variance weighted	2	-0.030	0.149	8.387E-01	0.998	cis	Severe COVID-19
14079_14	IL-18 Ra	Interleukin-18 receptor 1	Q13478	IL18R1	chr2:102311529	Inverse variance weighted	5	-0.010	0.052	8.399E-01	0.998	cis	Severe COVID-19
8397_147	QSOX2	Sulfhydryl oxidase 2	Q6ZRP7	QSOX2	chr9:136245812	Inverse variance weighted	7	0.007	0.036	8.400E-01	0.998	cis	Severe COVID-19
6425_87	MMP19	Matrix metalloproteinase-19	Q99542	MMP19	chr12:55842966	Inverse variance weighted	3	0.023	0.114	8.418E-01	0.998	cis	Severe COVID-19
6496_60	DLK1:ECD	Protein delta homolog 1:Extracellular domain	P80370	DLK1	chr14:100725705	Inverse variance weighted	2	0.104	0.521	8.420E-01	0.998	cis	Severe COVID-19
15633_6	RBP	Retinol-binding protein 4	P02753	RBP4	chr10:93601744	Wald ratio	1	0.053	0.272	8.445E-01	0.998	cis	Severe COVID-19
7266_4	SPA9	Serpin A9	Q86WD7	SERPINA9	chr14:94479689	Inverse variance weighted	2	0.032	0.164	8.469E-01	0.998	cis	Severe COVID-19
3419_49	CAMK2D	Calcium/calmodulin-dependent protein kinase type II subunit delta	Q13557	CAMK2D	chr4:113761927	Wald ratio	1	0.064	0.334	8.484E-01	0.998	cis	Severe COVID-19
12714_38	AP1G2	AP-1 complex subunit gamma-like 2	O75843	AP1G2	chr14:23568070	Inverse variance weighted	3	0.016	0.085	8.486E-01	0.998	cis	Severe COVID-19
6617_12	FCRL6	Fc receptor-like protein 6	Q6DN72	FCRL6	chr1:159800511	Wald ratio	1	0.030	0.156	8.486E-01	0.998	cis	Severe COVID-19
19135_5	Cytosolic 5'-nucleotidase III-like protein	7-methylguanosine phosphate-specific 5'-nucleotidase	Q969T7	NT5C3B	chr17:41836260	Inverse variance weighted	9	-0.010	0.053	8.486E-01	0.998	cis	Severe COVID-19
17453_34	Ceruloplasmin	Ceruloplasmin	P00450	CP	chr3:149221829	Inverse variance weighted	3	0.020	0.107	8.487E-01	0.998	cis	Severe COVID-19
3773_15	sTie-2	Angiopoietin-1 receptor, soluble	Q02763	TEK	chr9:27109141	Inverse variance weighted	4	-0.019	0.098	8.490E-01	0.998	cis	Severe COVID-19
5462_62	Ficolin-3	Ficolin-3	O75636	FCN3	chr1:27374824	Inverse variance weighted	7	-0.005	0.026	8.498E-01	0.998	cis	Severe COVID-19
9578_263	MANS4	MANSC domain-containing protein 4	A6NHS7	MANSC4	chr12:27780236	Inverse variance weighted	7	0.007	0.035	8.505E-01	0.998	cis	Severe COVID-19
19161_1	UBP15	Ubiquitin carboxyl-terminal hydrolase 15	Q9Y4E8	USP15	chr12:62260338	Inverse variance weighted	2	-0.019	0.099	8.507E-01	0.998	cis	Severe COVID-19
4971_1	CATZ	Cathepsin Z	Q9UBR2	CTSZ	chr20:59008238	Inverse variance weighted	5	-0.009	0.048	8.511E-01	0.998	cis	Severe COVID-19
12831_21	TESC	Calcineurin B homologous protein 3	Q96BS2	TESC	chr12:117099479	Inverse variance weighted	5	0.011	0.058	8.530E-01	0.998	cis	Severe COVID-19
3181_50	Cathepsin S	Cathepsin S	P25774	CTSS	chr1:150765957	Inverse variance weighted	4	-0.009	0.048	8.542E-01	0.998	cis	Severe COVID-19
18930_28	SLIT2	Slit homolog 2 protein	O94813	SLIT2	chr4:20251905	Inverse variance weighted	2	-0.024	0.129	8.552E-01	0.998	cis	Severe COVID-19
10832_24	B4GT6	Beta-1,4-galactosyltransferase 6	Q9UBX8	B4GALT6	chr18:31685836	Inverse variance weighted	4	-0.006	0.034	8.573E-01	0.998	cis	Severe COVID-19
17843_30	PPCS	Phosphopantothenate--cysteine ligase	Q9HAB8	PPCS	chr1:42456117	Inverse variance weighted	2	0.027	0.152	8.573E-01	0.998	cis	Severe COVID-19
8274_64	Syntaxin-7	Syntaxin-7	O15400	STX7	chr6:132513198	Wald ratio	1	-0.022	0.126	8.591E-01	0.998	cis	Severe COVID-19
4930_21	Stanniocalcin-1	Stanniocalcin-1	P52823	STC1	chr8:23854806	Wald ratio	1	-0.031	0.176	8.592E-01	0.998	cis	Severe COVID-19
15514_26	Pseudocholinesterase	Cholinesterase	P06276	BCHE	chr3:165837462	Inverse variance weighted	6	0.007	0.037	8.594E-01	0.998	cis	Severe COVID-19
5129_12	SREC-I	Scavenger receptor class F member 1	Q14162	SCARF1	chr17:1645744	Inverse variance weighted	5	0.010	0.058	8.602E-01	0.998	cis	Severe COVID-19
19437_61	L-VEGF165	Isoform L-VEGF165	P15692	VEGFA	chr6:43770184	Inverse variance weighted	7	0.019	0.109	8.603E-01	0.998	cis	Severe COVID-19
19206_20	8ODP	7,8-dihydro-8-oxoguanine triphosphatase	P36639	NUDT1	chr7:2242226	Wald ratio	1	0.025	0.143	8.613E-01	0.998	cis	Severe COVID-19
11192_168	TINAL	Tubulointerstitial nephritis antigen-like	Q9GZM7	TINAGL1	chr1:31576485	Wald ratio	1	0.026	0.151	8.614E-01	0.998	cis	Severe COVID-19
17332_3	ARHL2	Poly(ADP-ribose) glycohydrolase ARH3	Q9NX46	ADPRS	chr1:36088892	Inverse variance weighted	3	-0.016	0.093	8.635E-01	0.998	cis	Severe COVID-19
7968_15	CRTAM	Cytotoxic and regulatory T-cell molecule	O95727	CRTAM	chr11:122838500	Inverse variance weighted	6	-0.009	0.055	8.636E-01	0.998	cis	Severe COVID-19
15471_29	LIPR2	Pancreatic lipase-related protein 2	P54317	PNLIPRP2	chr10:116620953	Inverse variance weighted	11	-0.004	0.024	8.646E-01	0.998	cis	Severe COVID-19
18422_41	ERG19	Diphosphomevalonate decarboxylase	P53602	MVD	chr16:88663161	Inverse variance weighted	2	-0.039	0.230	8.658E-01	0.998	cis	Severe COVID-19
13622_16	2A5A	Serine/threonine-protein phosphatase 2A 56 kDa regulatory subunit alpha isoform	Q15172	PPP2R5A	chr1:212285410	Wald ratio	1	-0.061	0.359	8.659E-01	0.998	cis	Severe COVID-19
11516_7	FABPL	Fatty acid-binding protein, liver	P07148	FABP1	chr2:88128062	Wald ratio	1	-0.023	0.135	8.669E-01	0.998	cis	Severe COVID-19
5060_62	B7-H1	Programmed cell death 1 ligand 1	Q9NZQ7	CD274	chr9:5450503	Inverse variance weighted	2	0.011	0.065	8.674E-01	0.998	cis	Severe COVID-19
8842_16	GRM1C	GRAM domain-containing protein 1C	Q8IYS0	GRAMD1C	chr3:113828182	Inverse variance weighted	5	0.017	0.100	8.677E-01	0.998	cis	Severe COVID-19
18289_16	MIP-5	C-C motif chemokine 15	Q16663	CCL15	chr17:36001553	Inverse variance weighted	5	0.011	0.068	8.680E-01	0.998	cis	Severe COVID-19
4337_49	CRP	C-reactive protein	P02741	CRP	chr1:159714589	Inverse variance weighted	2	-0.014	0.085	8.686E-01	0.998	cis	Severe COVID-19
4979_34	DERM	Dermatopontin	Q07507	DPT	chr1:168729206	Inverse variance weighted	6	-0.008	0.051	8.691E-01	0.998	cis	Severe COVID-19
18882_7	CSTN2	Calsyntenin-2	Q9H4D0	CLSTN2	chr3:139935185	Inverse variance weighted	7	0.010	0.062	8.697E-01	0.998	cis	Severe COVID-19
17748_21	QORX	Quinone oxidoreductase PIG3	Q53FA7	TP53I3	chr2:24085861	Inverse variance weighted	5	0.010	0.062	8.707E-01	0.998	cis	Severe COVID-19
12411_60	MAX	Protein max	P61244	MAX	chr14:65102695	Wald ratio	1	-0.014	0.085	8.707E-01	0.998	cis	Severe COVID-19
2705_5	TECK	C-C motif chemokine 25	O15444	CCL25	chr19:8052318	Inverse variance weighted	11	0.008	0.050	8.712E-01	0.998	cis	Severe COVID-19
5630_48	CM35H	CMRF35-like molecule 8	Q9UGN4	CD300A	chr17:74466399	Inverse variance weighted	8	-0.005	0.030	8.713E-01	0.998	cis	Severe COVID-19
8309_12	HYAL1	Hyaluronidase-1	Q12794	HYAL1	chr3:50312381	Inverse variance weighted	2	-0.047	0.290	8.718E-01	0.998	cis	Severe COVID-19
7980_72	B3GN2	N-acetyllactosaminide beta-1,3-N-acetylglucosaminyltransferase 2	Q9NY97	B3GNT2	chr2:62196115	Inverse variance weighted	3	0.015	0.091	8.725E-01	0.998	cis	Severe COVID-19
8894_80	hnRNP A/B	Heterogeneous nuclear ribonucleoprotein A/B	Q99729	HNRNPAB	chr5:178204533	Wald ratio	1	-0.034	0.214	8.739E-01	0.998	cis	Severe COVID-19
5660_51	SOD3	Extracellular superoxide dismutase [Cu-Zn]	P08294	SOD3	chr4:24789912	Inverse variance weighted	10	0.006	0.036	8.742E-01	0.998	cis	Severe COVID-19
5728_60	FCRL1	Fc receptor-like protein 1	Q96LA6	FCRL1	chr1:157820120	Inverse variance weighted	4	-0.018	0.115	8.752E-01	0.998	cis	Severe COVID-19
11696_7	RABP2	Cellular retinoic acid-binding protein 2	P29373	CRABP2	chr1:156705816	Inverse variance weighted	3	-0.013	0.083	8.756E-01	0.998	cis	Severe COVID-19
12855_16	CT032	Cas scaffolding protein family member 4	Q9NQ75	CASS4	chr20:56412112	Wald ratio	1	0.045	0.285	8.757E-01	0.998	cis	Severe COVID-19
18313_4	Asparaginase-like protein 1	Isoaspartyl peptidase/L-asparaginase	Q7L266	ASRGL1	chr11:62337448	Inverse variance weighted	2	-0.013	0.086	8.761E-01	0.998	cis	Severe COVID-19
4332_6	CLC1B	C-type lectin domain family 1 member B	Q9P126	CLEC1B	chr12:10013424	Wald ratio	1	-0.033	0.211	8.763E-01	0.998	cis	Severe COVID-19
13534_20	MYOM2	Myomesin-2	P54296	MYOM2	chr8:2045046	Inverse variance weighted	3	0.033	0.212	8.774E-01	0.998	cis	Severe COVID-19
6520_87	MGP	Matrix Gla protein	P08493	MGP	chr12:14885857	Wald ratio	1	-0.037	0.245	8.799E-01	0.998	cis	Severe COVID-19
12703_6	NEK7	Serine/threonine-protein kinase Nek7	Q8TDX7	NEK7	chr1:198156994	Wald ratio	1	-0.038	0.253	8.801E-01	0.998	cis	Severe COVID-19
17161_1	OST48	Dolichyl-diphosphooligosaccharide--protein glycosyltransferase 48 kDa subunit	P39656	DDOST	chr1:20661544	Wald ratio	1	-0.046	0.307	8.806E-01	0.998	cis	Severe COVID-19
3195_50	Granulysin	Granulysin	P22749	GNLY	chr2:85685175	Inverse variance weighted	7	-0.007	0.044	8.813E-01	0.998	cis	Severe COVID-19
9244_27	PPT1	Palmitoyl-protein thioesterase 1	P50897	PPT1	chr1:40097260	Inverse variance weighted	2	-0.011	0.073	8.818E-01	0.998	cis	Severe COVID-19
18225_13	HEBP1	Heme-binding protein 1	Q9NRV9	HEBP1	chr12:13000265	Inverse variance weighted	6	-0.004	0.025	8.825E-01	0.998	cis	Severe COVID-19
5593_11	PDIA5	Protein disulfide-isomerase A5	Q14554	PDIA5	chr3:123067025	Inverse variance weighted	8	0.004	0.024	8.830E-01	0.998	cis	Severe COVID-19
2879_9	a1-Antichymotrypsin	Alpha-1-antichymotrypsin	P01011	SERPINA3	chr14:94612384	Wald ratio	1	0.049	0.333	8.841E-01	0.998	cis	Severe COVID-19
8469_41	IGFBP-2	Insulin-like growth factor-binding protein 2	P18065	IGFBP2	chr2:216632828	Wald ratio	1	-0.043	0.296	8.855E-01	0.998	cis	Severe COVID-19
16561_9	Alpha-1B-glycoprotein	Alpha-1B-glycoprotein	P04217	A1BG	chr19:58353492	Inverse variance weighted	4	0.006	0.039	8.866E-01	0.998	cis	Severe COVID-19
18881_7	CD97	CD97 antigen	P48960	ADGRE5	chr19:14380501	Inverse variance weighted	4	-0.026	0.189	8.884E-01	0.998	cis	Severe COVID-19
4297_62	Spondin-1	Spondin-1	Q9HCB6	SPON1	chr11:13962723	Inverse variance weighted	4	-0.007	0.053	8.888E-01	0.998	cis	Severe COVID-19
3831_21	pTEN	Phosphatidylinositol 3,4,5-trisphosphate 3-phosphatase and dual-specificity protein phosphatase PTEN	P60484	PTEN	chr10:87862563	Wald ratio	1	-0.051	0.367	8.889E-01	0.998	cis	Severe COVID-19

7849_3	Glutaminyl cyclase	Glutaminyl-peptide cyclotransferase	Q16769	QPCT	chr2:37342827	Inverse variance weighted	4	-0.014	0.098	8.899E-01	0.998	cis	Severe COVID-19
6627_25	LIPR1	Inactive pancreatic lipase-related protein 1	P54315	PNLIPRP1	chr10:116590385	Inverse variance weighted	2	0.015	0.108	8.906E-01	0.998	cis	Severe COVID-19
13095_51	PSP	Lithostathine-1-alpha	P05451	REG1A	chr2:79120362	Inverse variance weighted	4	0.021	0.154	8.911E-01	0.998	cis	Severe COVID-19
9383_24	CH3L2	Chitinase-3-like protein 2	Q15782	CHI3L2	chr1:111200771	Wald ratio	1	0.034	0.254	8.924E-01	0.998	cis	Severe COVID-19
11140_56	CO1A1:C-term propeptide	Collagen alpha-1(I) chain:C-term propeptide	P02452	COL1A1	chr17:50201632	Inverse variance weighted	3	-0.012	0.091	8.926E-01	0.998	cis	Severe COVID-19
4133_54	Granzyme B	Granzyme B	P10144	GZMB	chr14:24634267	Wald ratio	1	-0.022	0.162	8.931E-01	0.998	cis	Severe COVID-19
16614_27	RSPO1	R-spondin-1	Q2MKA7	RSPO1	chr1:37634892	Inverse variance weighted	2	-0.009	0.068	8.932E-01	0.998	cis	Severe COVID-19
2644_11	PKC-A	Protein kinase C alpha type	P17252	PRKCA	chr17:66302613	Wald ratio	1	-0.045	0.334	8.938E-01	0.998	cis	Severe COVID-19
12378_71	TPSN	Tapasin	O15533	TAPBP	chr6:33314284	Inverse variance weighted	4	-0.003	0.025	8.940E-01	0.998	cis	Severe COVID-19
17764_108	RHOC	Rho-related GTP-binding protein RhoC	P08134	RHOC	chr1:112707434	Wald ratio	1	-0.018	0.138	8.943E-01	0.998	cis	Severe COVID-19
4129_72	Factor B	Complement factor B	P00751	CFB	chr6:31945650	Inverse variance weighted	2	-0.016	0.120	8.954E-01	0.998	cis	Severe COVID-19
8969_49	CD14	Monocyte differentiation antigen CD14	P08571	CD14	chr5:140633700	Wald ratio	1	0.062	0.469	8.954E-01	0.998	cis	Severe COVID-19
6364_7	TPSNR	Tapasin-related protein	Q9BX59	TAPBPL	chr12:6451690	Inverse variance weighted	7	-0.006	0.043	8.957E-01	0.998	cis	Severe COVID-19
11266_8	SELPL:ECD	P-selectin glycoprotein ligand 1:Extracellular domain	Q14242	SELPLG	chr12:108633894	Inverse variance weighted	2	0.015	0.116	8.964E-01	0.998	cis	Severe COVID-19
10462_14	INSL5	Insulin-like peptide INSL5	Q9Y5Q6	INSL5	chr1:66801276	Wald ratio	1	-0.034	0.263	8.969E-01	0.998	cis	Severe COVID-19
5131_15	TAJ	Tumor necrosis factor receptor superfamily member 19	Q9NS68	TNFRSF19	chr13:23570370	Wald ratio	1	-0.020	0.155	8.972E-01	0.998	cis	Severe COVID-19
4962_52	ARMEL	Cerebral dopamine neurotrophic factor	Q49AH0	CDNF	chr10:14838575	Inverse variance weighted	6	-0.009	0.069	8.972E-01	0.998	cis	Severe COVID-19
9076_25	PENK	Proenkephalin-A	P01210	PENK	chr8:56446671	Inverse variance weighted	5	-0.005	0.036	8.975E-01	0.998	cis	Severe COVID-19
15535_3	Marapsin	Serine protease 27	Q9BQR3	PRSS27	chr16:2720551	Wald ratio	1	0.020	0.155	8.978E-01	0.998	cis	Severe COVID-19
6247_9	SIRB1	Signal-regulatory protein beta-1	O00241	SIRPB1	chr20:1620061	Inverse variance weighted	9	-0.003	0.020	8.982E-01	0.998	cis	Severe COVID-19
8840_61	C1s	Complement C1s subcomponent	P09871	C1S	chr12:6988259	Inverse variance weighted	4	0.016	0.126	8.982E-01	0.998	cis	Severe COVID-19
7096_30	RMD1	Regulator of microtubule dynamics protein 1	Q96DB5	RMDN1	chr8:86514357	Inverse variance weighted	4	0.009	0.070	8.990E-01	0.998	cis	Severe COVID-19
15487_164	carboxylesterase, liver	Liver carboxylesterase 1	P23141	CES1	chr16:55833337	Wald ratio	1	0.039	0.311	8.999E-01	0.998	cis	Severe COVID-19
15587_20	FOLR2	Folate receptor beta	P14207	FOLR2	chr11:72216601	Wald ratio	1	-0.015	0.117	9.004E-01	0.998	cis	Severe COVID-19
11196_31	Collagen alpha-3(VI):BPTI/Kunitz inhibitor	Collagen alpha-3(VI) chain:Bovine pancreatic trypsin inhibitor/Kunitz inhibitor domain, isoform 1	P12111	COL6A3	chr2:237414328	Wald ratio	1	-0.029	0.234	9.009E-01	0.998	cis	Severe COVID-19
15539_15	SLIK1	SLIT and NTRK-like protein 1	Q96PX8	SLITRK1	chr13:83882474	Wald ratio	1	-0.028	0.225	9.018E-01	0.998	cis	Severe COVID-19
13717_15	FCN2	Ficolin-2	Q15485	FCN2	chr9:134880810	Inverse variance weighted	8	-0.007	0.055	9.019E-01	0.998	cis	Severe COVID-19
7970_315	NAR3	Ecto-ADP-ribosyltransferase 3	Q13508	ART3	chr4:76011184	Inverse variance weighted	4	0.009	0.071	9.021E-01	0.998	cis	Severe COVID-19
4811_33	ITI heavy chain H4	Inter-alpha-trypsin inhibitor heavy chain H4	Q14624	ITIH4	chr3:52830688	Inverse variance weighted	2	-0.056	0.457	9.031E-01	0.998	cis	Severe COVID-19
17765_3	SPN1	Snurportin-1	O95149	SNUPN	chr15:75626469	Wald ratio	1	0.014	0.113	9.031E-01	0.998	cis	Severe COVID-19
9251_28	MA2B2	Epididymis-specific alpha-mannosidase	Q9Y2E5	MAN2B2	chr4:6575189	Inverse variance weighted	9	-0.004	0.033	9.057E-01	0.998	cis	Severe COVID-19
5980_55	BOLA3	BolA-like protein 3	Q53S33	BOLA3	chr2:74147912	Wald ratio	1	0.015	0.126	9.058E-01	0.998	cis	Severe COVID-19
7955_195	ITI heavy chain H1	Inter-alpha-trypsin inhibitor heavy chain H1	P19827	ITIH1	chr3:52777595	Inverse variance weighted	7	0.011	0.090	9.064E-01	0.998	cis	Severe COVID-19
7628_40	CREL1	Cysteine-rich with EGF-like domain protein 1	Q96HD1	CRELD1	chr3:9933793	Inverse variance weighted	12	-0.004	0.033	9.067E-01	0.998	cis	Severe COVID-19
5066_134	CLM6	CMRF35-like molecule 6	Q08708	CD300C	chr17:74546115	Inverse variance weighted	4	0.020	0.171	9.073E-01	0.998	cis	Severe COVID-19
9256_78	NPTX1	Neuronal pentraxin-1	Q15818	NPTX1	chr17:80477843	Inverse variance weighted	6	-0.006	0.054	9.079E-01	0.998	cis	Severe COVID-19
16307_22	UNC5H4	Netrin receptor UNC5D	Q6UXZ4	UNC5D	chr8:35235475	Inverse variance weighted	3	-0.013	0.115	9.088E-01	0.998	cis	Severe COVID-19
4535_50	BST1	ADP-ribosyl cyclase/cyclic ADP-ribose hydrolase 2	Q10588	BST1	chr4:15703065	Inverse variance weighted	11	0.002	0.020	9.089E-01	0.998	cis	Severe COVID-19
3364_76	Cathepsin V	Cathepsin L2	O60911	CTSV	chr9:97156556	Inverse variance weighted	4	0.009	0.083	9.092E-01	0.998	cis	Severe COVID-19
8043_153	COMP	Cartilage oligomeric matrix protein	P49747	COMP	chr19:18791305	Inverse variance weighted	4	-0.009	0.080	9.092E-01	0.998	cis	Severe COVID-19
16785_45	HD-5	Defensin-5	Q01523	DEFA5	chr8:7056739	Wald ratio	1	0.026	0.227	9.102E-01	0.998	cis	Severe COVID-19
5634_39	OFUT1	GDP-fucose protein O-fucosyltransferase 1	Q9H488	POFUT1	chr20:32207855	Inverse variance weighted	3	0.010	0.091	9.104E-01	0.998	cis	Severe COVID-19
11208_15	NAGPA	N-acetylglucosamine-1-phosphodiester alpha-N-acetylglucosaminidase	Q9UK23	NAGPA	chr16:5034141	Inverse variance weighted	7	-0.007	0.059	9.105E-01	0.998	cis	Severe COVID-19
19262_219	ACADV	Very long-chain specific acyl-CoA dehydrogenase, mitochondrial	P49748	ACADVL	chr17:7217125	Wald ratio	1	-0.034	0.299	9.107E-01	0.998	cis	Severe COVID-19
5532_53	bFGF-R	Fibroblast growth factor receptor 1	P11362	FGFR1	chr8:38468834	Inverse variance weighted	2	-0.017	0.152	9.111E-01	0.998	cis	Severe COVID-19
18197_97	KCRS	Creatine kinase S-type, mitochondrial	P17540	CKMT2	chr5:81233320	Wald ratio	1	0.036	0.327	9.122E-01	0.998	cis	Severe COVID-19
9267_2	CBPA4	Carboxypeptidase A4	Q9UI42	CPA4	chr7:130293134	Inverse variance weighted	11	-0.002	0.018	9.135E-01	0.998	cis	Severe COVID-19
15322_35	CRADD	Death domain-containing protein CRADD	P78560	CRADD	chr12:93677375	Inverse variance weighted	2	-0.079	0.725	9.136E-01	0.998	cis	Severe COVID-19
18222_34	SHLB2	Endophilin-B2	Q9NR46	SH3GLB2	chr9:129028331	Inverse variance weighted	3	-0.006	0.054	9.146E-01	0.998	cis	Severe COVID-19
7218_87	AT1B2	Sodium/potassium-transporting ATPase subunit beta-2	P14415	ATP1B2	chr17:7646627	Inverse variance weighted	3	-0.011	0.100	9.146E-01	0.998	cis	Severe COVID-19
3173_49	ASAHL	N-acylethanolamine-hydrolyzing acid amidase	Q02083	NAAA	chr4:75941013	Inverse variance weighted	10	-0.005	0.042	9.146E-01	0.998	cis	Severe COVID-19
13536_56	POLI	DNA polymerase iota	Q9UNA4	POLI	chr18:54269517	Wald ratio	1	0.021	0.198	9.152E-01	0.998	cis	Severe COVID-19
18918_86	PDE4A	cAMP-specific 3',5'-cyclic phosphodiesterase 4A	P27815	PDE4A	chr19:10416773	Inverse variance weighted	4	-0.016	0.153	9.154E-01	0.998	cis	Severe COVID-19
5698_60	Tenascin-X	Tenascin-X	P22105	TNXB	chr6:32115334	Inverse variance weighted	5	0.005	0.050	9.156E-01	0.998	cis	Severe COVID-19
14107_1	MTHFS	5-formyltetrahydrofolate cyclo-ligase	P49914	MTHFS	chr15:79897379	Inverse variance weighted	6	-0.005	0.043	9.166E-01	0.998	cis	Severe COVID-19
2571_12	IGFBP-3	Insulin-like growth factor-binding protein 3	P17936	IGFBP3	chr7:45921874	Inverse variance weighted	4	-0.006	0.054	9.168E-01	0.998	cis	Severe COVID-19
8974_172	COFA1	Collagen alpha-1(XV) chain	P39059	COL15A1	chr9:98943179	Inverse variance weighted	4	-0.007	0.071	9.176E-01	0.998	cis	Severe COVID-19
3050_7	vWF	von Willebrand factor	P04275	VWF	chr12:6124770	Inverse variance weighted	4	0.026	0.252	9.188E-01	0.998	cis	Severe COVID-19
2991_9	IL-1 sRI	Interleukin-1 receptor type 1	P14778	IL1R1	chr2:102064544	Inverse variance weighted	2	0.025	0.247	9.195E-01	0.998	cis	Severe COVID-19
13118_5	SMOC1	SPARC-related modular calcium-binding protein 1	Q9H4F8	SMOC1	chr14:69854131	Inverse variance weighted	3	-0.007	0.072	9.220E-01	0.998	cis	Severe COVID-19
4834_61	Epithelial cell kinase	Ephrin type-A receptor 2	P29317	EPHA2	chr1:16156069	Inverse variance weighted	3	0.009	0.094	9.222E-01	0.998	cis	Severe COVID-19
8252_2	NOTUM	Palmitoleoyl-protein carboxylesterase NOTUM	Q6P988	NOTUM	chr17:81961840	Inverse variance weighted	3	-0.014	0.147	9.225E-01	0.998	cis	Severe COVID-19
5076_53	EPHAA	Ephrin type-A receptor 10	Q5JZY3	EPHA10	chr1:37765133	Wald ratio	1	0.020	0.209	9.226E-01	0.998	cis	Severe COVID-19
9478_69	KPRA	Phosphoribosyl pyrophosphate synthase-associated protein 1	Q14558	PRPSAP1	chr17:76384521	Wald ratio	1	-0.027	0.278	9.234E-01	0.998	cis	Severe COVID-19
15391_114	GAS-6	Growth arrest-specific protein 6	Q14393	GAS6	chr13:113864076	Inverse variance weighted	4	0.005	0.058	9.246E-01	0.998	cis	Severe COVID-19
18162_167	IRAK4	Interleukin-1 receptor-associated kinase 4	Q9NWZ3	IRAK4	chr12:43758944	Wald ratio	1	-0.018	0.190	9.249E-01	0.998	cis	Severe COVID-19
15511_37	NPTXR	Neuronal pentraxin receptor	O95502	NPTXR	chr22:38844028	Inverse variance weighted	7	-0.004	0.041	9.253E-01	0.998	cis	Severe COVID-19
14012_17	LGP2	Probable ATP-dependent RNA helicase DHX58	Q96C10	DHX58	chr17:42112714	Inverse variance weighted	2	0.013	0.136	9.255E-01	0.998	cis	Severe COVID-19
7251_64	C1QT3	Complement C1q tumor necrosis factor-related protein 3	Q9BXJ4	C1QTNF3	chr5:34043213	Inverse variance weighted	2	0.010	0.109	9.262E-01	0.998	cis	Severe COVID-19
15447_45	Sorbitol dehydrogenase	Sorbitol dehydrogenase	Q00796	SORD	chr15:45023147	Inverse variance weighted	4	0.006	0.066	9.263E-01	0.998	cis	Severe COVID-19
6385_63	VWA1	von Willebrand factor A domain-containing protein 1	Q6PCB0	VWA1	chr1:1434861	Inverse variance weighted	5	0.005	0.056	9.281E-01	0.998	cis	Severe COVID-19
9264_11	CATO	Cathepsin O	P43234	CTSO	chr4:155953912	Inverse variance weighted	2	-0.009	0.099	9.287E-01	0.998	cis	Severe COVID-19
4154_57	P-Selectin	P-selectin	P16109	SELP	chr1:169630193	Inverse variance weighted	3	0.011	0.126	9.295E-01	0.998	cis	Severe COVID-19
12646_2	RPE	Ribulose-phosphate 3-epimerase	Q96AT9	RPE	chr2:210002565	Wald ratio	1	-0.016	0.185	9.301E-01	0.998	cis	Severe COVID-19
6455_52	HPLN4	Hyaluronan and proteoglycan link protein 4	Q86UW8	HAPLN4	chr19:19262804	Wald ratio	1	0.019	0.212	9.302E-01	0.998	cis	Severe COVID-19
9241_40	SIRPG	Signal-regulatory protein gamma	Q9P1W8	SIRPG	chr20:1657779	Wald ratio	1	-0.021	0.241	9.305E-01	0.998	cis	Severe COVID-19
8250_2	PTPRJ	Receptor-type tyrosine-protein phosphatase eta	Q12913	PTPRJ	chr11:47980425	Inverse variance weighted	3	-0.016	0.179	9.305E-01	0.998	cis	Severe COVID-19
2278_61	TIMP-2	Metalloproteinase inhibitor 2	P16035	TIMP2	chr17:78925387	Wald ratio	1	0.019	0.214	9.311E-01	0.998	cis	Severe COVID-19

12357_41	SNP29	Synaptosomal-associated protein 29	O95721	SNAP29	chr22:20859007	Wald ratio	1	0.028	0.322	9.314E-01	0.998	cis	Severe COVID-19
3326_58	Nectin-like protein 2	Cell adhesion molecule 1	Q9BY67	CADM1	chr11:115504957	Wald ratio	1	-0.009	0.110	9.320E-01	0.998	cis	Severe COVID-19
8013_9	LMA2L	VIP36-like protein	Q9H0V9	LMAN2L	chr2:96740064	Inverse variance weighted	2	0.011	0.128	9.321E-01	0.998	cis	Severe COVID-19
8364_74	UST	Uronyl 2-sulfotransferase	Q9Y2C2	UST	chr6:148747030	Inverse variance weighted	3	0.009	0.109	9.322E-01	0.998	cis	Severe COVID-19
8660_5	OLFL3	Olfactomedin-like protein 3	Q9NRN5	OLFML3	chr1:113979391	Inverse variance weighted	2	-0.012	0.144	9.324E-01	0.998	cis	Severe COVID-19
9394_19	Aminopeptidase	Carboxypeptidase Q	Q9Y646	CPQ	chr8:96645242	Inverse variance weighted	8	0.004	0.050	9.334E-01	0.998	cis	Severe COVID-19
17726_3	SAR1A	GTP-binding protein SAR1a	Q9NR31	SAR1A	chr10:70170523	Inverse variance weighted	2	-0.011	0.133	9.336E-01	0.998	cis	Severe COVID-19
5740_17	ROBO1	Roundabout homolog 1	Q9Y6N7	ROBO1	chr3:79767998	Inverse variance weighted	3	0.011	0.127	9.339E-01	0.998	cis	Severe COVID-19
17672_184	Gastric intrinsic factor	Gastric intrinsic factor	P27352	CBLIF	chr11:59845499	Inverse variance weighted	2	-0.014	0.167	9.340E-01	0.998	cis	Severe COVID-19
18396_10	AES	Amino-terminal enhancer of split	Q08117	TLE5	chr19:3063107	Wald ratio	1	0.025	0.302	9.342E-01	0.998	cis	Severe COVID-19
2811_27	Angiopoietin-1	Angiopoietin-1	Q15389	ANGPT1	chr8:107498055	Wald ratio	1	0.018	0.224	9.345E-01	0.998	cis	Severe COVID-19
10800_15	Collagen-binding protein	Serpin H1	P50454	SERPINH1	chr11:75562056	Inverse variance weighted	2	0.014	0.171	9.351E-01	0.998	cis	Severe COVID-19
5404_53	DR6	Tumor necrosis factor receptor superfamily member 21	O75509	TNFRSF21	chr6:47309905	Inverse variance weighted	2	-0.014	0.176	9.351E-01	0.998	cis	Severe COVID-19
19197_95	THIL	Acetyl-CoA acetyltransferase, mitochondrial	P24752	ACAT1	chr11:108116695	Inverse variance weighted	2	0.017	0.208	9.355E-01	0.998	cis	Severe COVID-19
17210_2	TCL1A	T-cell leukemia/lymphoma protein 1A	P56279	TCL1A	chr14:95714196	Inverse variance weighted	3	-0.011	0.133	9.356E-01	0.998	cis	Severe COVID-19
17327_3	CNPY3	Protein canopy homolog 3	Q9BT09	CNPY3	chr6:42929480	Wald ratio	1	-0.021	0.268	9.362E-01	0.998	cis	Severe COVID-19
5231_79	PCSK9	Proprotein convertase subtilisin/kexin type 9	Q8NBP7	PCSK9	chr1:55039447	Inverse variance weighted	6	0.004	0.054	9.366E-01	0.998	cis	Severe COVID-19
3440_7	granzyme A	Granzyme A	P12544	GZMA	chr5:55102646	Inverse variance weighted	4	-0.004	0.056	9.377E-01	0.998	cis	Severe COVID-19
4160_49	MMP-2	72 kDa type IV collagenase	P08253	MMP2	chr16:55389700	Inverse variance weighted	2	0.013	0.168	9.380E-01	0.998	cis	Severe COVID-19
15579_26	ENPP6	Ectonucleotide pyrophosphatase/phosphodiesterase family member 6	Q6UWR7	ENPP6	chr4:184221230	Wald ratio	1	-0.021	0.279	9.398E-01	0.998	cis	Severe COVID-19
17706_4	PPR1A	Protein phosphatase 1 regulatory subunit 1A	Q13522	PPP1R1A	chr12:54588659	Wald ratio	1	-0.022	0.287	9.400E-01	0.998	cis	Severe COVID-19
15533_97	Macrophage scavenger receptor:ECD	Macrophage scavenger receptor types I and II:Extracellular domain	P21757	MSR1	chr8:16567490	Inverse variance weighted	5	0.003	0.040	9.417E-01	0.998	cis	Severe COVID-19
5139_32	UNC5H3	Netrin receptor UNC5C	O95185	UNC5C	chr4:95549206	Inverse variance weighted	3	0.006	0.083	9.420E-01	0.998	cis	Severe COVID-19
13544_9	HMHA1	Rho GTPase-activating protein 45	Q92619	ARHGAP45	chr19:1065923	Wald ratio	1	0.008	0.116	9.427E-01	0.998	cis	Severe COVID-19
9759_13	INDO	Indoleamine 2,3-dioxygenase 1	P14902	IDO1	chr8:39902275	Wald ratio	1	0.018	0.254	9.433E-01	0.998	cis	Severe COVID-19
15523_9	HEM2	Delta-aminolevulinic acid dehydratase	P13716	ALAD	chr9:113401290	Inverse variance weighted	7	-0.004	0.055	9.443E-01	0.998	cis	Severe COVID-19
4413_3	SLPI	Antileukoproteinase	P03973	SLPI	chr20:45254564	Wald ratio	1	-0.022	0.319	9.459E-01	0.998	cis	Severe COVID-19
19556_12	Complement receptor type 1	Complement receptor type 1	P17927	CR1	chr1:207496147	Inverse variance weighted	10	0.005	0.079	9.466E-01	0.998	cis	Severe COVID-19
5605_77	MFNG	Beta-1,3-N-acetylglucosaminyltransferase manic fringe	O00587	MFNG	chr22:37486393	Wald ratio	1	-0.014	0.212	9.466E-01	0.998	cis	Severe COVID-19
11377_19	ADH7	Alcohol dehydrogenase class 4 mu/sigma chain	P40394	ADH7	chr4:99435510	Inverse variance weighted	3	-0.004	0.052	9.468E-01	0.998	cis	Severe COVID-19
6077_63	CECR1	Adenosine deaminase CECR1	Q9NZK5	ADA2	chr22:17258235	Inverse variance weighted	5	0.003	0.040	9.480E-01	0.998	cis	Severe COVID-19
12620_3	Septin-11	Septin-11	Q9NVA2	SEPTIN11	chr4:76949751	Wald ratio	1	-0.016	0.250	9.484E-01	0.998	cis	Severe COVID-19
10603_1	HIS3	Histatin-3	P15516	HTN3	chr4:70028455	Inverse variance weighted	3	0.007	0.115	9.487E-01	0.998	cis	Severe COVID-19
2730_58	MICA	MHC class I polypeptide-related sequence A	Q29983	MICA	chr6:31399784	Inverse variance weighted	12	-0.003	0.047	9.492E-01	0.998	cis	Severe COVID-19
18213_30	UBE2F	NEDD8-conjugating enzyme UBE2F	Q969M7	UBE2F	chr2:237966827	Wald ratio	1	0.019	0.300	9.494E-01	0.998	cis	Severe COVID-19
7757_5	DQA2	HLA class II histocompatibility antigen, DQ alpha 2 chain	P01906	HLA-DQA2	chr6:32741391	Inverse variance weighted	3	-0.007	0.108	9.494E-01	0.998	cis	Severe COVID-19
19237_17	D-dopachrome decarboxylase	D-dopachrome decarboxylase	P30046	DDT	chr22:23980525	Inverse variance weighted	2	0.009	0.139	9.501E-01	0.998	cis	Severe COVID-19
3046_31	resistin	Resistin	Q9HD89	RETN	chr19:7669049	Inverse variance weighted	6	0.004	0.070	9.505E-01	0.998	cis	Severe COVID-19
3799_11	Carbonic anhydrase III	Carbonic anhydrase 3	P07451	CA3	chr8:85373436	Inverse variance weighted	4	0.006	0.090	9.508E-01	0.998	cis	Severe COVID-19
12689_56	ARC1B	Actin-related protein 2/3 complex subunit 1B	O15143	ARPC1B	chr7:99374249	Wald ratio	1	0.011	0.177	9.511E-01	0.998	cis	Severe COVID-19
16324_38	TLR1:ECD	Toll-like receptor 1:Extracellular domain	Q15399	TLR1	chr4:38856817	Wald ratio	1	-0.008	0.125	9.516E-01	0.998	cis	Severe COVID-19
5021_13	PPase	Inorganic pyrophosphatase	Q15181	PPA1	chr10:70233911	Inverse variance weighted	4	-0.006	0.106	9.523E-01	0.998	cis	Severe COVID-19
9995_6	DUT	Deoxyuridine 5'-triphosphate nucleotidohydrolase, mitochondrial	P33316	DUT	chr15:48331011	Wald ratio	1	0.014	0.239	9.527E-01	0.998	cis	Severe COVID-19
8890_9	T132B	Transmembrane protein 132B	Q14DG7	TMEM132B	chr12:125186386	Inverse variance weighted	2	0.012	0.196	9.530E-01	0.998	cis	Severe COVID-19
16288_17	EPHA4	Ephrin type-A receptor 4	P54764	EPHA4	chr2:221574202	Inverse variance weighted	3	-0.005	0.087	9.553E-01	0.998	cis	Severe COVID-19
5663_18	PF4V	Platelet factor 4 variant	P10720	PF4V1	chr4:73853296	Inverse variance weighted	2	-0.009	0.169	9.572E-01	0.998	cis	Severe COVID-19
5085_18	IL-20 Ra	Interleukin-20 receptor subunit alpha	Q9UHF4	IL20RA	chr6:137045180	Wald ratio	1	-0.020	0.379	9.579E-01	0.998	cis	Severe COVID-19
3298_52	Contactin-4	Contactin-4	Q8IWV2	CNTN4	chr3:2098813	Inverse variance weighted	8	0.002	0.046	9.581E-01	0.998	cis	Severe COVID-19
12436_84	GST omega-1	Glutathione S-transferase omega-1	P78417	GSTO1	chr10:104235356	Inverse variance weighted	9	-0.002	0.032	9.587E-01	0.998	cis	Severe COVID-19
7999_23	CD39	Ectonucleoside triphosphate diphosphohydrolase 1	P49961	ENTPD1	chr10:95711779	Inverse variance weighted	3	-0.008	0.159	9.592E-01	0.998	cis	Severe COVID-19
6649_51	NET1	Netrin-1	O95631	NTN1	chr17:9021510	Inverse variance weighted	6	0.003	0.068	9.595E-01	0.998	cis	Severe COVID-19
19251_56	Platelet proteoglycan	Serglycin	P10124	SRGN	chr10:69088103	Inverse variance weighted	5	-0.003	0.061	9.599E-01	0.998	cis	Severe COVID-19
10940_25	SRCA	Sarcalumenin	Q86TD4	SRL	chr16:4242080	Wald ratio	1	-0.014	0.277	9.601E-01	0.998	cis	Severe COVID-19
7761_125	CHKB	Choline/ethanolamine kinase	Q9Y259	CHKB	chr22:50601455	Inverse variance weighted	3	-0.007	0.143	9.609E-01	0.998	cis	Severe COVID-19
5609_92	F19A5	Protein FAM19A5	Q7Z5A7	TAFA5	chr22:48489553	Wald ratio	1	-0.015	0.326	9.631E-01	0.998	cis	Severe COVID-19
3484_60	Angiotensinogen	Angiotensinogen	P01019	AGT	chr1:230745576	Inverse variance weighted	2	0.004	0.088	9.642E-01	0.998	cis	Severe COVID-19
15559_5	ANTR2	Anthrax toxin receptor 2	P58335	ANTXR2	chr4:80125454	Inverse variance weighted	4	-0.006	0.132	9.646E-01	0.998	cis	Severe COVID-19
5353_89	IL-1Ra	Interleukin-1 receptor antagonist protein	P18510	IL1RN	chr2:113099315	Inverse variance weighted	3	0.005	0.121	9.656E-01	0.998	cis	Severe COVID-19
15516_12	SAA-4	Serum amyloid A-4 protein	P35542	SAA4	chr11:18236802	Inverse variance weighted	7	0.001	0.024	9.667E-01	0.998	cis	Severe COVID-19
17722_5	FKBP52 protein	Peptidyl-prolyl cis-trans isomerase FKBP4	Q02790	FKBP4	chr12:2794970	Inverse variance weighted	2	0.010	0.237	9.673E-01	0.998	cis	Severe COVID-19
19136_22	MMSA	Methylmalonate-semialdehyde dehydrogenase [acylating], mitochondrial	Q02252	ALDH6A1	chr14:74084492	Inverse variance weighted	2	-0.011	0.265	9.675E-01	0.998	cis	Severe COVID-19
3302_58	CYTF	Cystatin-F	O76096	CST7	chr20:24949269	Inverse variance weighted	7	0.001	0.033	9.677E-01	0.998	cis	Severe COVID-19
9906_21	TICN3	Testican-3	Q9BQ16	SPOCK3	chr4:167234796	Inverse variance weighted	11	0.001	0.037	9.681E-01	0.998	cis	Severe COVID-19
2961_1	Protein C	Vitamin K-dependent protein C	P04070	PROC	chr2:127418427	Inverse variance weighted	3	-0.005	0.123	9.698E-01	0.998	cis	Severe COVID-19
2632_5	IL-12 Rb1	Interleukin-12 receptor subunit beta-1	P42701	IL12RB1	chr19:18098944	Wald ratio	1	0.007	0.193	9.698E-01	0.998	cis	Severe COVID-19
7009_8	CD72	B-cell differentiation antigen CD72	P21854	CD72	chr9:35646810	Inverse variance weighted	2	-0.005	0.134	9.701E-01	0.998	cis	Severe COVID-19
3291_30	CD23	Low affinity immunoglobulin epsilon Fc receptor	P06734	FCER2	chr19:7702146	Inverse variance weighted	6	-0.002	0.049	9.718E-01	0.998	cis	Severe COVID-19
19213_1	ISK4	Serine protease inhibitor Kazal-type 4	O60575	SPINK4	chr9:33218365	Wald ratio	1	0.002	0.073	9.727E-01	0.998	cis	Severe COVID-19
5363_51	Semaphorin 3E	Semaphorin-3E	O15041	SEMA3E	chr7:83649139	Inverse variance weighted	10	-0.001	0.028	9.744E-01	0.998	cis	Severe COVID-19
13642_90	CIG49	Interferon-induced protein with tetratricopeptide repeats 3	O14879	IFIT3	chr10:89327307	Wald ratio	1	-0.006	0.180	9.746E-01	0.998	cis	Severe COVID-19
3172_28	ARSB	Arylsulfatase B	P15848	ARSB	chr5:78986087	Inverse variance weighted	2	0.003	0.104	9.750E-01	0.998	cis	Severe COVID-19
5087_5	IL-22BP	Interleukin-22 receptor subunit alpha-2	Q969J5	IL22RA2	chr6:137173648	Inverse variance weighted	5	-0.004	0.115	9.757E-01	0.998	cis	Severe COVID-19
10627_87	APLP2	Amyloid-like protein 2	Q06481	APLP2	chr11:130068147	Inverse variance weighted	2	-0.005	0.167	9.760E-01	0.998	cis	Severe COVID-19
9191_8	Trefoil factor 2	Trefoil factor 2	Q03403	TFF2	chr21:42350997	Inverse variance weighted	2	0.006	0.200	9.770E-01	0.998	cis	Severe COVID-19
12338_27	EGFLA	Pikachurin	Q63HQ2	EGFLAM	chr5:38258409	Inverse variance weighted	3	0.002	0.064	9.779E-01	0.998	cis	Severe COVID-19
15304_1	PAP1	Regenerating islet-derived protein 3-alpha	Q06141	REG3A	chr2:79159753	Inverse variance weighted	3	0.003	0.130	9.792E-01	0.998	cis	Severe COVID-19
7769_29	3BP2	SH3 domain-binding protein 2	P78314	SH3BP2	chr4:2793071	Inverse variance weighted	3	0.004	0.152	9.802E-01	0.998	cis	Severe COVID-19
2994_71	IL-1Rrp2	Interleukin-1 receptor-like 2	Q9HB29	IL1RL2	chr2:102187006	Wald ratio	1	-0.003	0.106	9.810E-01	0.998	cis	Severe COVID-19

15686_49	INHBC	Inhibin beta C chain	P55103	INHBC	chr12:57434784	Inverse variance weighted	14	0.001	0.028	9.810E-01	0.998	cis	Severe COVID-19
8243_55	TATI	Serine protease inhibitor Kazal-type 1	P00995	SPINK1	chr5:147831671	Inverse variance weighted	2	-0.004	0.162	9.818E-01	0.998	cis	Severe COVID-19
8664_36	PKDCC	Extracellular tyrosine-protein kinase PKDCC	Q504Y2	PKDCC	chr2:42048021	Inverse variance weighted	2	0.012	0.558	9.825E-01	0.998	cis	Severe COVID-19
11431_235	RECQ1	ATP-dependent DNA helicase Q1	P46063	RECQL	chr12:21501669	Inverse variance weighted	6	0.001	0.030	9.834E-01	0.998	cis	Severe COVID-19
18243_9	Cytidylate kinase	UMP-CMP kinase	P30085	CMPK1	chr1:47333790	Wald ratio	1	0.004	0.195	9.834E-01	0.998	cis	Severe COVID-19
2602_2	Angiopoietin-2	Angiopoietin-2	O15123	ANGPT2	chr8:6563409	Inverse variance weighted	3	0.002	0.101	9.837E-01	0.998	cis	Severe COVID-19
14688_6	PTN7	Tyrosine-protein phosphatase non-receptor type 7	P35236	PTPN7	chr1:202161588	Wald ratio	1	-0.006	0.315	9.837E-01	0.998	cis	Severe COVID-19
13657_2	PNKP	Bifunctional polynucleotide phosphatase/kinase	Q96T60	PNKP	chr19:49878351	Wald ratio	1	0.003	0.147	9.839E-01	0.998	cis	Severe COVID-19
3806_55	EphA5	Ephrin type-A receptor 5	P54756	EPHA5	chr4:65670495	Inverse variance weighted	3	-0.002	0.122	9.840E-01	0.998	cis	Severe COVID-19
5615_62	F172A	Protein FAM172A	Q8WUF8	FAM172A	chr5:94111699	Wald ratio	1	0.004	0.218	9.856E-01	0.998	cis	Severe COVID-19
4440_15	FCRL3	Fc receptor-like protein 3	Q96P31	FCRL3	chr1:157700769	Inverse variance weighted	4	0.002	0.097	9.857E-01	0.998	cis	Severe COVID-19
14684_17	CAN2	Calpain-2 catalytic subunit	P17655	CAPN2	chr1:223701593	Inverse variance weighted	3	-0.002	0.117	9.858E-01	0.998	cis	Severe COVID-19
5465_32	H6ST1	Heparan-sulfate 6-O-sulfotransferase 1	O60243	HS6ST1	chr2:128318868	Inverse variance weighted	2	-0.002	0.091	9.860E-01	0.998	cis	Severe COVID-19
19622_7	Activin A	Activin A	P08476	INHBA	chr7:41705834	Wald ratio	1	-0.005	0.274	9.860E-01	0.998	cis	Severe COVID-19
5736_1	TRML2	Trem-like transcript 2 protein	Q5T2D2	TREML2	chr6:41201149	Inverse variance weighted	5	-0.001	0.040	9.866E-01	0.998	cis	Severe COVID-19
15368_3	BMPER	BMP-binding endothelial regulator protein	Q8N8U9	BMPER	chr7:33904308	Inverse variance weighted	3	0.002	0.105	9.867E-01	0.998	cis	Severe COVID-19
3168_8	ADAMTS-5	A disintegrin and metalloproteinase with thrombospondin motifs 5	Q9UNA0	ADAMTS5	chr21:26967088	Inverse variance weighted	5	-0.001	0.052	9.872E-01	0.998	cis	Severe COVID-19
2774_10	IL-16	Interleukin-16	Q14005	IL16	chr15:81159575	Inverse variance weighted	10	0.000	0.027	9.873E-01	0.998	cis	Severe COVID-19
16609_106	KIRR2	Kin of IRRE-like protein 2	Q6UWL6	KIRREL2	chr19:35855861	Inverse variance weighted	6	-0.001	0.074	9.875E-01	0.998	cis	Severe COVID-19
10565_19	SLIK3	SLIT and NTRK-like protein 3	O94933	SLITRK3	chr3:165197109	Inverse variance weighted	3	0.001	0.059	9.877E-01	0.998	cis	Severe COVID-19
2652_15	suPAR	Urokinase plasminogen activator surface receptor	Q03405	PLAUR	chr19:43670547	Inverse variance weighted	2	0.002	0.121	9.887E-01	0.998	cis	Severe COVID-19
13934_3	MCF2L	Guanine nucleotide exchange factor DBS	O15068	MCF2L	chr13:112894378	Wald ratio	1	0.001	0.071	9.888E-01	0.998	cis	Severe COVID-19
19130_81	SPB8	Serpin B8	P50452	SERPINB8	chr18:63970029	Inverse variance weighted	3	-0.001	0.104	9.899E-01	0.998	cis	Severe COVID-19
15525_294	ADH1G	Alcohol dehydrogenase 1C	P00326	ADH1C	chr4:99352746	Wald ratio	1	-0.001	0.116	9.901E-01	0.998	cis	Severe COVID-19
19113_66	SH3L2	SH3 domain-binding glutamic acid-rich-like protein 2	Q9UJC5	SH3BGRL2	chr6:79631329	Wald ratio	1	-0.002	0.144	9.913E-01	0.998	cis	Severe COVID-19
16823_75	APOL3	Apolipoprotein L3	O95236	APOL3	chr22:36166177	Inverse variance weighted	7	0.000	0.033	9.920E-01	0.998	cis	Severe COVID-19
3309_2	FCG2A	Low affinity immunoglobulin gamma Fc region receptor II-a	P12318	FCGR2A	chr1:161505430	Inverse variance weighted	4	0.001	0.103	9.921E-01	0.998	cis	Severe COVID-19
12348_46	SYSM	Serine--tRNA ligase, mitochondrial	Q9NP81	SARS2	chr19:38930763	Wald ratio	1	0.001	0.135	9.922E-01	0.998	cis	Severe COVID-19
13130_150	HXK2	Hexokinase-2	P52789	HK2	chr2:74834127	Inverse variance weighted	2	-0.002	0.186	9.925E-01	0.998	cis	Severe COVID-19
15300_66	CC134	Coiled-coil domain-containing protein 134	Q9H6E4	CCDC134	chr22:41800679	Inverse variance weighted	2	-0.001	0.134	9.928E-01	0.998	cis	Severe COVID-19
3516_60	SDF-1	Stromal cell-derived factor 1	P48061	CXCL12	chr10:44386493	Inverse variance weighted	2	-0.001	0.125	9.932E-01	0.998	cis	Severe COVID-19
16607_78	Gelsolin	Gelsolin	P06396	GSN	chr9:121207794	Inverse variance weighted	2	-0.002	0.227	9.934E-01	0.998	cis	Severe COVID-19
4187_49	6-Phosphogluconate dehydrogenase	6-phosphogluconate dehydrogenase, decarboxylating	P52209	PGD	chr1:10398592	Wald ratio	1	0.002	0.198	9.936E-01	0.998	cis	Severe COVID-19
7810_20	C1QTNF5	Complement C1q tumor necrosis factor-related protein 5	Q9BXJ0	C1QTNF5	chr11:119340940	Inverse variance weighted	2	-0.001	0.067	9.938E-01	0.998	cis	Severe COVID-19
13460_4	CHAD	Chondroadherin	O15335	CHAD	chr17:50468906	Wald ratio	1	0.002	0.297	9.940E-01	0.998	cis	Severe COVID-19
14134_49	AMGO2	Amphoterin-induced protein 2	Q86SJ2	AMIGO2	chr12:47079959	Inverse variance weighted	3	0.001	0.139	9.940E-01	0.998	cis	Severe COVID-19
8351_17	PRS57	Serine protease 57	Q6UWY2	PRSS57	chr19:695498	Inverse variance weighted	6	0.000	0.066	9.944E-01	0.998	cis	Severe COVID-19
8960_3	LRAP	Endoplasmic reticulum aminopeptidase 2	Q6P179	ERAP2	chr5:96875986	Inverse variance weighted	4	0.000	0.040	9.946E-01	0.998	cis	Severe COVID-19
4549_78	FUT5	Alpha-(1,3)-fucosyltransferase 5	Q11128	FUT5	chr19:5870540	Inverse variance weighted	7	0.000	0.026	9.953E-01	0.998	cis	Severe COVID-19
17343_6	SNPH	Syntaphilin	O15079	SNPH	chr20:1266280	Inverse variance weighted	2	-0.001	0.174	9.959E-01	0.998	cis	Severe COVID-19
12573_80	TRIM3	Tripartite motif-containing protein 3	O75382	TRIM3	chr11:6474459	Wald ratio	1	-0.001	0.183	9.962E-01	0.998	cis	Severe COVID-19
5637_81	NTNG1	Netrin-G1	Q9Y2I2	NTNG1	chr1:107140007	Inverse variance weighted	9	0.000	0.030	9.962E-01	0.998	cis	Severe COVID-19
13733_5	IL-12 p40	Interleukin-12 subunit beta	P29460	IL12B	chr5:159330863	Inverse variance weighted	6	0.000	0.036	9.963E-01	0.998	cis	Severe COVID-19
7866_11	DJC30	DnaJ homolog subfamily C member 30	Q96LL9	DNAJC30	chr7:73683453	Inverse variance weighted	2	0.000	0.102	9.965E-01	0.998	cis	Severe COVID-19
17410_5	CETN3	Centrin-3	O15182	CETN3	chr5:90409766	Wald ratio	1	-0.001	0.212	9.967E-01	0.998	cis	Severe COVID-19
16805_5	PDE5A	cGMP-specific 3',5'-cyclic phosphodiesterase	O76074	PDE5A	chr4:119628804	Wald ratio	1	0.002	0.385	9.967E-01	0.998	cis	Severe COVID-19
19154_41	Protease nexin I	Glia-derived nexin	P07093	SERPINE2	chr2:224039318	Inverse variance weighted	8	0.000	0.034	9.967E-01	0.998	cis	Severe COVID-19
17677_47	BY55	CD160 antigen	O95971	CD160	chr1:145719471	Wald ratio	1	0.000	0.137	9.972E-01	0.998	cis	Severe COVID-19
3220_40	RET	Proto-oncogene tyrosine-protein kinase receptor Ret	P07949	RET	chr10:43077064	Inverse variance weighted	4	0.000	0.057	9.991E-01	0.999	cis	Severe COVID-19
9253_52	BGAT	Histo-blood group ABO system transferase	P16442	ABO	chr9:133276024	Inverse variance weighted	12	0.083	0.016	5.147E-07	0.001	cis	Hospitalized COVID-19
13992_12	NSF	Vesicle-fusing ATPase	P46459	NSF	chr17:46590669	Wald ratio	1	-0.607	0.138	1.107E-05	0.009	cis	Hospitalized COVID-19
12656_1	KLC1	Kinesin light chain 1	Q07866	KLC1	chr14:103561896	Wald ratio	1	0.492	0.116	2.148E-05	0.011	cis	Hospitalized COVID-19
2580_83	Myeloperoxidase	Myeloperoxidase	P05164	MPO	chr17:58280935	Inverse variance weighted	7	-0.127	0.030	2.712E-05	0.011	cis	Hospitalized COVID-19
17769_28	PCNP	PEST proteolytic signal-containing nuclear protein	Q8WW12	PCNP	chr3:101574180	Wald ratio	1	-0.479	0.117	4.237E-05	0.014	cis	Hospitalized COVID-19
12534_10	CACO2	Calcium-binding and coiled-coil domain-containing protein 2	Q13137	CALCOCO2	chr17:48831018	Wald ratio	1	-0.451	0.116	1.041E-04	0.029	cis	Hospitalized COVID-19
10346_5	STAT3	Signal transducer and activator of transcription 3	P40763	STAT3	chr17:42388540	Inverse variance weighted	2	-0.303	0.080	1.497E-04	0.035	cis	Hospitalized COVID-19
8255_34	MRVI1	Protein MRVI1	Q9Y6F6	IRAG1	chr11:10693988	Inverse variance weighted	2	-0.319	0.085	1.678E-04	0.035	cis	Hospitalized COVID-19
15607_56	KPYR	Pyruvate kinase PKLR	P30613	PKLR	chr1:155301438	Wald ratio	1	-0.303	0.085	3.601E-04	0.067	cis	Hospitalized COVID-19
13130_150	HXK2	Hexokinase-2	P52789	HK2	chr2:74834127	Inverse variance weighted	2	-0.434	0.128	7.207E-04	0.111	cis	Hospitalized COVID-19
4125_52	sRAGE	Advanced glycosylation end product-specific receptor, soluble	Q15109	AGER	chr6:32184322	Inverse variance weighted	2	0.163	0.048	7.217E-04	0.111	cis	Hospitalized COVID-19
12387_7	PDLI4	PDZ and LIM domain protein 4	P50479	PDLIM4	chr5:132257696	Wald ratio	1	-0.208	0.063	1.010E-03	0.142	cis	Hospitalized COVID-19
9638_2	TIGIT	T-cell immunoreceptor with Ig and ITIM domains	Q495A1	TIGIT	chr3:114276913	Wald ratio	1	-0.432	0.133	1.173E-03	0.152	cis	Hospitalized COVID-19
4541_49	CDON	Cell adhesion molecule-related/down-regulated by oncogenes	Q4KMG0	CDON	chr11:126063335	Inverse variance weighted	4	0.084	0.026	1.501E-03	0.181	cis	Hospitalized COVID-19
3348_49	BMP-1	Bone morphogenetic protein 1	P13497	BMP1	chr8:22165140	Wald ratio	1	0.484	0.159	2.366E-03	0.255	cis	Hospitalized COVID-19
5491_12	Testican-2	Testican-2	Q92563	SPOCK2	chr10:72089032	Wald ratio	1	-0.162	0.053	2.421E-03	0.255	cis	Hospitalized COVID-19
18884_22	DNJB4	DnaJ homolog subfamily B member 4	Q9UDY4	DNAJB4	chr1:77979175	Inverse variance weighted	3	0.177	0.060	2.864E-03	0.280	cis	Hospitalized COVID-19
11543_84	LIMA1	LIM domain and actin-binding protein 1	Q9UHB6	LIMA1	chr12:50283520	Wald ratio	1	0.476	0.161	3.057E-03	0.280	cis	Hospitalized COVID-19
5508_62	Cathepsin D	Cathepsin D	P07339	CTSD	chr11:1764573	Inverse variance weighted	5	0.118	0.040	3.195E-03	0.280	cis	Hospitalized COVID-19
15583_18	FCRLB	Fc receptor-like B	Q6BAA4	FCRLB	chr1:161721544	Inverse variance weighted	5	0.157	0.053	3.350E-03	0.280	cis	Hospitalized COVID-19
6955_68	SNX1	Sorting nexin-1	Q13596	SNX1	chr15:64094123	Wald ratio	1	-0.228	0.078	3.486E-03	0.280	cis	Hospitalized COVID-19
5586_66	MINP1	Multiple inositol polyphosphate phosphatase 1	Q9UNW1	MINPP1	chr10:87504875	Wald ratio	1	0.385	0.133	3.921E-03	0.301	cis	Hospitalized COVID-19
4155_3	Tenascin	Tenascin	P24821	TNC	chr9:115118207	Inverse variance weighted	12	-0.065	0.023	4.966E-03	0.364	cis	Hospitalized COVID-19
8866_53	QPCTL	Glutaminyl-peptide cyclotransferase-like protein	Q9NXS2	QPCTL	chr19:45692403	Inverse variance weighted	6	-0.092	0.033	5.410E-03	0.380	cis	Hospitalized COVID-19
5111_15	NRX3B	Neurexin-3-beta	Q9HDB5	NRXN3	chr14:78170373	Inverse variance weighted	2	-0.443	0.160	5.660E-03	0.382	cis	Hospitalized COVID-19
9754_33	Quinone reductase 2	Ribosyldihydronicotinamide dehydrogenase [quinone]	P16083	NQO2	chr6:2987987	Inverse variance weighted	8	0.048	0.018	6.974E-03	0.453	cis	Hospitalized COVID-19
17856_23	NT5M	5'(3')-deoxyribonucleotidase, mitochondrial	Q9NPB1	NT5M	chr17:17303335	Wald ratio	1	-0.231	0.086	7.484E-03	0.468	cis	Hospitalized COVID-19
19617_5	LTB4DH	Prostaglandin reductase 1	Q14914	PTGR1	chr9:111599893	Inverse variance weighted	8	-0.049	0.019	7.892E-03	0.475	cis	Hospitalized COVID-19
4342_10	sICAM-1	Intercellular adhesion molecule 1	P05362	ICAM1	chr19:10271093	Inverse variance weighted	9	0.038	0.015	8.903E-03	0.501	cis	Hospitalized COVID-19

5605_77	MFNG	Beta-1,3-N-acetylglucosaminyltransferase manic fringe	O00587	MFNG	chr22:37486393	Wald ratio	1	0.295	0.113	8.908E-03	0.501	cis	Hospitalized COVID-19
18899_82	HDGR3	Hepatoma-derived growth factor-related protein 3	Q9Y3E1	HDGFL3	chr15:83207823	Wald ratio	1	-0.272	0.105	9.934E-03	0.541	cis	Hospitalized COVID-19
2864_2	MEK1	Dual specificity mitogen-activated protein kinase kinase 1	Q02750	MAP2K1	chr15:66386837	Wald ratio	1	0.451	0.176	1.028E-02	0.542	cis	Hospitalized COVID-19
4188_1	Aflatoxin B1 aldehyde reductase	Aflatoxin B1 aldehyde reductase member 2	O43488	AKR7A2	chr1:19312144	Wald ratio	1	0.170	0.067	1.146E-02	0.586	cis	Hospitalized COVID-19
4549_78	FUT5	Alpha-(1,3)-fucosyltransferase 5	Q11128	FUT5	chr19:5870540	Inverse variance weighted	7	-0.044	0.017	1.181E-02	0.586	cis	Hospitalized COVID-19
19504_22	KTHY	Thymidylate kinase	P23919	DTYMK	chr2:241686944	Inverse variance weighted	3	0.331	0.133	1.284E-02	0.599	cis	Hospitalized COVID-19
2731_29	NADPH-P450 Oxidoreductase	NADPH--cytochrome P450 reductase	P16435	POR	chr7:75899200	Inverse variance weighted	2	-0.211	0.085	1.337E-02	0.599	cis	Hospitalized COVID-19
3206_4	LYVE1	Lymphatic vessel endothelial hyaluronic acid receptor 1	Q9Y5Y7	LYVE1	chr11:10611689	Inverse variance weighted	6	0.107	0.044	1.423E-02	0.599	cis	Hospitalized COVID-19
17675_17	ACO13	Acyl-coenzyme A thioesterase 13	Q9NPJ3	ACOT13	chr6:24667035	Inverse variance weighted	2	0.289	0.118	1.425E-02	0.599	cis	Hospitalized COVID-19
12801_33	IRF2	Interferon regulatory factor 2	P14316	IRF2	chr4:184474558	Wald ratio	1	-0.488	0.200	1.464E-02	0.599	cis	Hospitalized COVID-19
12386_11	AMPB	Aminopeptidase B	Q9H4A4	RNPEP	chr1:201982372	Inverse variance weighted	5	0.074	0.031	1.511E-02	0.599	cis	Hospitalized COVID-19
13988_67	NMRL1	NmrA-like family domain-containing protein 1	Q9HBL8	NMRAL1	chr16:4495763	Inverse variance weighted	10	-0.063	0.026	1.533E-02	0.599	cis	Hospitalized COVID-19
6899_37	PHS2	Pterin-4-alpha-carbinolamine dehydratase 2	Q9H0N5	PCBD2	chr5:134905120	Wald ratio	1	0.695	0.287	1.536E-02	0.599	cis	Hospitalized COVID-19
12813_18	EHBP1	EH domain-binding protein 1	Q8NDI1	EHBP1	chr2:62673851	Wald ratio	1	-0.497	0.206	1.559E-02	0.599	cis	Hospitalized COVID-19
17447_52	SFRP4	Secreted frizzled-related protein 4	Q6FHJ7	SFRP4	chr7:38025695	Inverse variance weighted	2	-0.110	0.046	1.595E-02	0.599	cis	Hospitalized COVID-19
3866_7	SBDS	Ribosome maturation protein SBDS	Q9Y3A5	SBDS	chr7:66995693	Wald ratio	1	0.202	0.084	1.610E-02	0.599	cis	Hospitalized COVID-19
9837_60	NAD(P)H dehydrogenase	NAD(P)H dehydrogenase [quinone] 1	P15559	NQO1	chr16:69726668	Inverse variance weighted	9	-0.039	0.016	1.706E-02	0.599	cis	Hospitalized COVID-19
7916_10	S100A7	Protein S100-A7	P31151	S100A7	chr1:153460651	Inverse variance weighted	7	0.043	0.018	1.748E-02	0.599	cis	Hospitalized COVID-19
4479_14	C1-Esterase Inhibitor	Plasma protease C1 inhibitor	P05155	SERPING1	chr11:57597387	Inverse variance weighted	6	-0.061	0.026	1.755E-02	0.599	cis	Hospitalized COVID-19
3003_29	NKp30	Natural cytotoxicity triggering receptor 3	O14931	NCR3	chr6:31593006	Wald ratio	1	0.327	0.138	1.768E-02	0.599	cis	Hospitalized COVID-19
10037_98	SIG12:Ig-like V-type 2	Sialic acid-binding Ig-like lectin 12:Ig-like V-type 2 domain, Isoform long	Q96PQ1	SIGLEC12	chr19:51501800	Inverse variance weighted	4	-0.094	0.040	1.774E-02	0.599	cis	Hospitalized COVID-19
10754_113	Prokineticin-2	Prokineticin-2	Q9HC23	PROK2	chr3:71785206	Inverse variance weighted	6	-0.057	0.024	1.887E-02	0.611	cis	Hospitalized COVID-19
3415_61	BSP	Bone sialoprotein 2	P21815	IBSP	chr4:87799554	Wald ratio	1	-0.234	0.100	1.891E-02	0.611	cis	Hospitalized COVID-19
6605_17	IGFALS	Insulin-like growth factor-binding protein complex acid labile subunit	P35858	IGFALS	chr16:1794971	Inverse variance weighted	3	-0.159	0.068	1.990E-02	0.611	cis	Hospitalized COVID-19
10554_23	BGAL	Beta-galactosidase	P16278	GLB1	chr3:33097202	Wald ratio	1	0.588	0.253	2.027E-02	0.611	cis	Hospitalized COVID-19
3421_54	CD30 Ligand	Tumor necrosis factor ligand superfamily member 8	P32971	TNFSF8	chr9:114930595	Inverse variance weighted	2	-0.158	0.068	2.030E-02	0.611	cis	Hospitalized COVID-19
11103_24	HSP 27	Heat shock protein beta-1	P04792	HSPB1	chr7:76302673	Inverse variance weighted	4	-0.068	0.029	2.058E-02	0.611	cis	Hospitalized COVID-19
18832_65	SAA2	Serum amyloid A-2 protein	P0DJI9	SAA2	chr11:18248668	Wald ratio	1	-0.513	0.222	2.072E-02	0.611	cis	Hospitalized COVID-19
19383_131	CINP	Cyclin-dependent kinase 2-interacting protein	Q9BW66	CINP	chr14:102362916	Wald ratio	1	0.436	0.189	2.101E-02	0.611	cis	Hospitalized COVID-19
9173_21	PGM1	Phosphoglucomutase-1	P36871	PGM1	chr1:63593411	Inverse variance weighted	2	0.221	0.096	2.165E-02	0.617	cis	Hospitalized COVID-19
2700_56	Protein S	Vitamin K-dependent protein S	P07225	PROS1	chr3:93980003	Wald ratio	1	-0.399	0.174	2.194E-02	0.617	cis	Hospitalized COVID-19
19197_95	THIL	Acetyl-CoA acetyltransferase, mitochondrial	P24752	ACAT1	chr11:108116695	Inverse variance weighted	2	0.299	0.131	2.312E-02	0.640	cis	Hospitalized COVID-19
13940_19	IP16:HIN 1	Gamma-interferon-inducible protein 16:Isoform 2, Hematopoietic expression, interferon-inducible nature, and nuclear localization 1	Q16666	IFI16	chr1:158999968	Wald ratio	1	0.349	0.155	2.399E-02	0.653	cis	Hospitalized COVID-19
11237_49	PCOC1	Procollagen C-endopeptidase enhancer 1	Q15113	PCOLCE	chr7:100602363	Wald ratio	1	0.567	0.252	2.455E-02	0.657	cis	Hospitalized COVID-19
13590_1	ORN	Oligoribonuclease, mitochondrial	Q9Y3B8	REXO2	chr11:114439435	Wald ratio	1	-0.195	0.087	2.511E-02	0.657	cis	Hospitalized COVID-19
4459_68	PCSK7	Proprotein convertase subtilisin/kexin type 7	Q16549	PCSK7	chr11:117232525	Inverse variance weighted	10	-0.058	0.026	2.530E-02	0.657	cis	Hospitalized COVID-19
12432_23	CYBP	Calcyclin-binding protein	Q9HB71	CACYBP	chr1:174999163	Wald ratio	1	-0.165	0.074	2.588E-02	0.658	cis	Hospitalized COVID-19
18188_12	GATM	Glycine amidinotransferase, mitochondrial	P50440	GATM	chr15:45402327	Wald ratio	1	-0.132	0.059	2.621E-02	0.658	cis	Hospitalized COVID-19
18382_109	Catechol O-methyltransferase	Catechol O-methyltransferase	P21964	COMT	chr22:19941371	Wald ratio	1	-0.256	0.115	2.653E-02	0.658	cis	Hospitalized COVID-19
5744_12	CA056	Protein MENT	Q9BUN1	MENT	chr1:151047751	Inverse variance weighted	2	-0.273	0.124	2.777E-02	0.669	cis	Hospitalized COVID-19
19273_3	Glutathione reductase	Glutathione reductase, mitochondrial	P00390	GSR	chr8:30727846	Inverse variance weighted	3	-0.172	0.078	2.814E-02	0.669	cis	Hospitalized COVID-19
5316_54	Prothrombin	Prothrombin	P00734	F2	chr11:46719196	Wald ratio	1	0.417	0.190	2.862E-02	0.669	cis	Hospitalized COVID-19
2843_13	SPINT2	Kunitz-type protease inhibitor 2	O43291	SPINT2	chr19:38244035	Inverse variance weighted	7	-0.060	0.027	2.923E-02	0.669	cis	Hospitalized COVID-19
9790_28	BR serine/threonine kinase 2	Serine/threonine-protein kinase BRSK2	Q8IWQ3	BRSK2	chr11:1389899	Inverse variance weighted	2	-0.136	0.063	2.964E-02	0.669	cis	Hospitalized COVID-19
9002_36	SPA11	Serpin A11	Q86U17	SERPINA11	chr14:94452800	Inverse variance weighted	5	-0.146	0.067	2.970E-02	0.669	cis	Hospitalized COVID-19
10630_5	HTAI2	Oxidoreductase HTATIP2	Q9BUP3	HTATIP2	chr11:20363685	Inverse variance weighted	9	-0.058	0.027	2.973E-02	0.669	cis	Hospitalized COVID-19
17513_11	ANX11	Annexin A11	P50995	ANXA11	chr10:80205572	Inverse variance weighted	3	0.185	0.086	3.101E-02	0.688	cis	Hospitalized COVID-19
4500_50	SCGF-alpha	Stem cell growth factor-alpha	Q9Y240	CLEC11A	chr19:50723364	Inverse variance weighted	2	-0.059	0.027	3.174E-02	0.695	cis	Hospitalized COVID-19
5825_49	IFN-g R1	Interferon gamma receptor 1	P15260	IFNGR1	chr6:137219449	Inverse variance weighted	3	-0.242	0.114	3.325E-02	0.695	cis	Hospitalized COVID-19
5337_64	B7-2	T-lymphocyte activation antigen CD86	P42081	CD86	chr3:122055362	Wald ratio	1	-0.374	0.176	3.374E-02	0.695	cis	Hospitalized COVID-19
12449_16	PPIH	Peptidyl-prolyl cis-trans isomerase H	O43447	PPIH	chr1:42658335	Inverse variance weighted	2	0.298	0.140	3.377E-02	0.695	cis	Hospitalized COVID-19
5478_50	PSMA	Glutamate carboxypeptidase 2	Q04609	FOLH1	chr11:49208638	Inverse variance weighted	2	-0.142	0.067	3.412E-02	0.695	cis	Hospitalized COVID-19
18432_32	RALB	Ras-related protein Ral-B	P11234	RALB	chr2:120240064	Wald ratio	1	-0.109	0.051	3.420E-02	0.695	cis	Hospitalized COVID-19
8337_65	PTPRU	Receptor-type tyrosine-protein phosphatase U	Q92729	PTPRU	chr1:29236516	Inverse variance weighted	5	-0.130	0.061	3.421E-02	0.695	cis	Hospitalized COVID-19
6342_10	Nephronectin	Nephronectin	Q6UXI9	NPNT	chr4:105894775	Inverse variance weighted	3	0.249	0.118	3.496E-02	0.696	cis	Hospitalized COVID-19
5636_10	MFAP4	Microfibril-associated glycoprotein 4	P55083	MFAP4	chr17:19387190	Inverse variance weighted	2	0.111	0.053	3.504E-02	0.696	cis	Hospitalized COVID-19
15339_32	COF2	Cofilin-2	Q9Y281	CFL2	chr14:34714823	Wald ratio	1	0.181	0.086	3.577E-02	0.702	cis	Hospitalized COVID-19
8464_31	RSPO4	R-spondin-4	Q2I0M5	RSPO4	chr20:1002311	Inverse variance weighted	2	0.259	0.124	3.774E-02	0.717	cis	Hospitalized COVID-19
8288_27	b2-Glycoprotein I	Beta-2-glycoprotein 1	P02749	APOH	chr17:66256525	Inverse variance weighted	3	0.057	0.028	3.776E-02	0.717	cis	Hospitalized COVID-19
7923_41	SEM4C	Semaphorin-4C	Q9C0C4	SEMA4C	chr2:96870757	Wald ratio	1	0.180	0.087	3.780E-02	0.717	cis	Hospitalized COVID-19
10440_26	ACAM:ECD	CXADR-like membrane protein:Extracellular domain	Q9H6B4	CLMP	chr11:123195248	Inverse variance weighted	3	0.096	0.047	3.901E-02	0.731	cis	Hospitalized COVID-19
15499_11	Attractin	Attractin	O75882	ATRN	chr20:3471018	Inverse variance weighted	5	-0.083	0.041	3.949E-02	0.732	cis	Hospitalized COVID-19
11126_102	TRIO	Triple functional domain protein	O75962	TRIO	chr5:14143342	Wald ratio	1	-0.471	0.231	4.132E-02	0.758	cis	Hospitalized COVID-19
8360_169	NKp46	Natural cytotoxicity triggering receptor 1	O76036	NCR1	chr19:54906148	Wald ratio	1	0.223	0.110	4.246E-02	0.766	cis	Hospitalized COVID-19
9599_6	PIANP	PILR alpha-associated neural protein	Q8IYJ0	PIANP	chr12:6700815	Wald ratio	1	-0.462	0.228	4.267E-02	0.766	cis	Hospitalized COVID-19
2611_72	Dtk	Tyrosine-protein kinase receptor TYRO3	Q06418	TYRO3	chr15:41557675	Wald ratio	1	0.210	0.104	4.379E-02	0.776	cis	Hospitalized COVID-19
3855_56	Peroxiredoxin-1	Peroxiredoxin-1	Q06830	PRDX1	chr1:45542732	Inverse variance weighted	2	-0.182	0.090	4.422E-02	0.776	cis	Hospitalized COVID-19
14205_6	HEXI2	Protein HEXIM2	Q96MH2	HEXIM2	chr17:45160700	Wald ratio	1	0.448	0.223	4.463E-02	0.776	cis	Hospitalized COVID-19
6461_54	Apo C-III	Apolipoprotein C-III	P02656	APOC3	chr11:116829706	Inverse variance weighted	2	-0.260	0.131	4.634E-02	0.792	cis	Hospitalized COVID-19
4254_6	NUDC3	NudC domain-containing protein 3	Q8IVD9	NUDCD3	chr7:44490658	Wald ratio	1	0.304	0.153	4.713E-02	0.792	cis	Hospitalized COVID-19
3074_6	LBP	Lipopolysaccharide-binding protein	P18428	LBP	chr20:38346482	Inverse variance weighted	5	-0.049	0.025	4.768E-02	0.792	cis	Hospitalized COVID-19
5091_28	ILT-4	Leukocyte immunoglobulin-like receptor subfamily B member 2	Q8N423	LILRB2	chr19:54281184	Inverse variance weighted	4	-0.148	0.075	4.771E-02	0.792	cis	Hospitalized COVID-19
18830_1	Omentin	Intelectin-1	Q8WWA0	ITLN1	chr1:160885180	Wald ratio	1	0.346	0.175	4.790E-02	0.792	cis	Hospitalized COVID-19
13438_115	CHRD	Chordin	Q9H2X0	CHRD	chr3:184380054	Wald ratio	1	-0.278	0.142	4.946E-02	0.806	cis	Hospitalized COVID-19
8768_4	Bcl-10	B-cell lymphoma/leukemia 10	O95999	BCL10	chr1:85276632	Wald ratio	1	0.438	0.224	4.999E-02	0.806	cis	Hospitalized COVID-19
19560_23	PLXA4	Plexin-A4	Q9HCM2	PLXNA4	chr7:132648688	Wald ratio	1	-0.252	0.129	5.033E-02	0.806	cis	Hospitalized COVID-19
10339_48	NSE	Gamma-enolase	P09104	ENO2	chr12:6913745	Wald ratio	1	0.186	0.096	5.204E-02	0.806	cis	Hospitalized COVID-19
4992_49	GRN	Granulins	P28799	GRN	chr17:44345246	Inverse variance weighted	3	-0.095	0.049	5.282E-02	0.806	cis	Hospitalized COVID-19

8346_9	DPP2	Dipeptidyl peptidase 2	Q9UHL4	DPP7	chr9:137118309	Inverse variance weighted	8	-0.067	0.035	5.294E-02	0.806	cis	Hospitalized COVID-19
6415_90	CPN2	Carboxypeptidase N subunit 2	P22792	CPN2	chr3:194351328	Inverse variance weighted	6	0.071	0.037	5.412E-02	0.806	cis	Hospitalized COVID-19
14711_27	Cystatin M	Cystatin-M	Q15828	CST6	chr11:66012008	Wald ratio	1	0.208	0.109	5.540E-02	0.806	cis	Hospitalized COVID-19
17785_11	RCL	2'-deoxynucleoside 5'-phosphate N-hydrolase 1	O43598	DNPH1	chr6:43229481	Wald ratio	1	-0.271	0.142	5.620E-02	0.806	cis	Hospitalized COVID-19
19267_14	GLO2	Hydroxyacylglutathione hydrolase, mitochondrial	Q16775	HAGH	chr16:1827157	Wald ratio	1	0.132	0.069	5.629E-02	0.806	cis	Hospitalized COVID-19
8248_222	SIG14	Sialic acid-binding Ig-like lectin 14	Q08ET2	SIGLEC14	chr19:51646825	Inverse variance weighted	3	0.164	0.086	5.641E-02	0.806	cis	Hospitalized COVID-19
18933_4	TGM4	Protein-glutamine gamma-glutamyltransferase 4	P49221	TGM4	chr3:44874608	Wald ratio	1	-0.354	0.186	5.721E-02	0.806	cis	Hospitalized COVID-19
3366_51	ECM1	Extracellular matrix protein 1	Q16610	ECM1	chr1:150508062	Inverse variance weighted	11	-0.046	0.024	5.732E-02	0.806	cis	Hospitalized COVID-19
8053_16	DJB14:CD	DnaJ homolog subfamily B member 14:Cytoplasmic domain	Q8TBM8	DNAJB14	chr4:99946618	Wald ratio	1	0.328	0.173	5.758E-02	0.806	cis	Hospitalized COVID-19
15466_30	CO9A1	Collagen alpha-1(IX) chain	P20849	COL9A1	chr6:70303084	Inverse variance weighted	2	0.221	0.116	5.780E-02	0.806	cis	Hospitalized COVID-19
13580_2	Sperm-associated antigen 2	UDP-N-acetylhexosamine pyrophosphorylase	Q16222	UAP1	chr1:162561722	Wald ratio	1	0.496	0.262	5.835E-02	0.806	cis	Hospitalized COVID-19
15584_9	FHR2	Complement factor H-related protein 2	P36980	CFHR2	chr1:196943738	Inverse variance weighted	4	0.061	0.032	5.837E-02	0.806	cis	Hospitalized COVID-19
11387_3	ATF6B	Cyclic AMP-dependent transcription factor ATF-6 beta	Q99941	ATF6B	chr6:32128253	Inverse variance weighted	3	-0.177	0.093	5.853E-02	0.806	cis	Hospitalized COVID-19
15558_63	AMPE	Glutamyl aminopeptidase	Q07075	ENPEP	chr4:110365733	Inverse variance weighted	8	0.054	0.028	5.862E-02	0.806	cis	Hospitalized COVID-19
15305_7	Secretagogin	Secretagogin	O76038	SCGN	chr6:25652201	Inverse variance weighted	2	0.272	0.144	5.870E-02	0.806	cis	Hospitalized COVID-19
12469_19	MARE1	Microtubule-associated protein RP/EB family member 1	Q15691	MAPRE1	chr20:32819954	Wald ratio	1	-0.365	0.193	5.879E-02	0.806	cis	Hospitalized COVID-19
12395_86	SYDM	Aspartate--tRNA ligase, mitochondrial	Q6PI48	DARS2	chr1:173824653	Wald ratio	1	-0.201	0.106	5.950E-02	0.806	cis	Hospitalized COVID-19
15449_33	TIM-4	T-cell immunoglobulin and mucin domain-containing protein 4	Q96H15	TIMD4	chr5:156963226	Inverse variance weighted	5	-0.129	0.069	5.970E-02	0.806	cis	Hospitalized COVID-19
19206_20	8ODP	7,8-dihydro-8-oxoguanine triphosphatase	P36639	NUDT1	chr7:2242226	Wald ratio	1	0.182	0.098	6.296E-02	0.843	cis	Hospitalized COVID-19
3600_2	Chitotriosidase-1	Chitotriosidase-1	Q13231	CHIT1	chr1:203273641	Inverse variance weighted	6	0.035	0.019	6.459E-02	0.858	cis	Hospitalized COVID-19
11493_169	DYL2	Dynein light chain 2, cytoplasmic	Q96FJ2	DYNLL2	chr17:58083419	Wald ratio	1	-0.368	0.201	6.668E-02	0.877	cis	Hospitalized COVID-19
12494_99	GBRL2	Gamma-aminobutyric acid receptor-associated protein-like 2	P60520	GABARAPL2	chr16:75566375	Wald ratio	1	-0.241	0.132	6.704E-02	0.877	cis	Hospitalized COVID-19
11568_2	FKB1B	Peptidyl-prolyl cis-trans isomerase FKBP1B	P68106	FKBP1B	chr2:24049701	Wald ratio	1	0.165	0.090	6.773E-02	0.879	cis	Hospitalized COVID-19
19279_42	CRBP	Retinol-binding protein 1	P09455	RBP1	chr3:139539829	Wald ratio	1	0.283	0.156	6.890E-02	0.881	cis	Hospitalized COVID-19
7926_13	SPIT3	Kunitz-type protease inhibitor 3	P49223	SPINT3	chr20:45515622	Inverse variance weighted	4	0.123	0.067	6.917E-02	0.881	cis	Hospitalized COVID-19
19635_69	FGL2	Fibroleukin	Q14314	FGL2	chr7:77199848	Wald ratio	1	0.325	0.180	7.023E-02	0.881	cis	Hospitalized COVID-19
3044_3	PARC	C-C motif chemokine 18	P55774	CCL18	chr17:36064272	Inverse variance weighted	3	0.211	0.117	7.033E-02	0.881	cis	Hospitalized COVID-19
3038_9	I-TAC	C-X-C motif chemokine 11	O14625	CXCL11	chr4:76041415	Inverse variance weighted	4	-0.107	0.060	7.189E-02	0.881	cis	Hospitalized COVID-19
5107_7	Notch 1	Neurogenic locus notch homolog protein 1	P46531	NOTCH1	chr9:136546048	Inverse variance weighted	2	0.161	0.090	7.240E-02	0.881	cis	Hospitalized COVID-19
15398_2	HERV1	FAD-linked sulfhydryl oxidase ALR	P55789	GFER	chr16:1984193	Wald ratio	1	-0.323	0.180	7.329E-02	0.881	cis	Hospitalized COVID-19
17460_51	Mx1	Interferon-induced GTP-binding protein Mx1	P20591	MX1	chr21:41420020	Inverse variance weighted	4	-0.076	0.042	7.339E-02	0.881	cis	Hospitalized COVID-19
9213_24	FTCD	Formimidoyltransferase-cyclodeaminase	O95954	FTCD	chr21:46155579	Inverse variance weighted	3	-0.123	0.069	7.434E-02	0.881	cis	Hospitalized COVID-19
2977_7	EDAR	Tumor necrosis factor receptor superfamily member EDAR	Q9UNE0	EDAR	chr2:108989372	Inverse variance weighted	7	-0.040	0.023	7.513E-02	0.881	cis	Hospitalized COVID-19
6620_82	LIGO1	Leucine-rich repeat and immunoglobulin-like domain-containing nogo receptor-interacting protein 1	Q96FE5	LINGO1	chr15:77820900	Inverse variance weighted	2	0.206	0.117	7.686E-02	0.881	cis	Hospitalized COVID-19
12940_35	AL3B1	Aldehyde dehydrogenase family 3 member B1	P43353	ALDH3B1	chr11:68008578	Wald ratio	1	0.161	0.091	7.719E-02	0.881	cis	Hospitalized COVID-19
8005_1	MXRA7	Matrix-remodeling-associated protein 7	P84157	MXRA7	chr17:76711004	Inverse variance weighted	5	-0.055	0.031	7.745E-02	0.881	cis	Hospitalized COVID-19
6930_95	SIA8F	Alpha-2,8-sialyltransferase 8F	P61647	ST8SIA6	chr10:17454595	Wald ratio	1	-0.365	0.207	7.750E-02	0.881	cis	Hospitalized COVID-19
8275_31	PEAR1:ECD	Platelet endothelial aggregation receptor 1:Extracellular domain	Q5VY43	PEAR1	chr1:156893698	Inverse variance weighted	3	-0.106	0.060	7.768E-02	0.881	cis	Hospitalized COVID-19
18875_125	Chondrocalcin	Chondrocalcin	P02458	COL2A1	chr12:48004554	Inverse variance weighted	7	-0.038	0.022	7.839E-02	0.881	cis	Hospitalized COVID-19
5238_26	PPIE	Peptidyl-prolyl cis-trans isomerase E	Q9UNP9	PPIE	chr1:39692182	Inverse variance weighted	2	0.061	0.035	7.847E-02	0.881	cis	Hospitalized COVID-19
18216_22	IL-11 RA	Interleukin-11 receptor subunit alpha	Q14626	IL11RA	chr9:34652162	Inverse variance weighted	3	-0.161	0.092	8.049E-02	0.881	cis	Hospitalized COVID-19
9359_9	EGFL9	Protein delta homolog 2	Q6UY11	DLK2	chr6:43456632	Wald ratio	1	-0.103	0.059	8.067E-02	0.881	cis	Hospitalized COVID-19
14054_17	IL-15 Ra	Interleukin-15 receptor subunit alpha	Q13261	IL15RA	chr10:5978187	Inverse variance weighted	5	0.043	0.024	8.110E-02	0.881	cis	Hospitalized COVID-19
5487_7	SLAF7	SLAM family member 7	Q9NQ25	SLAMF7	chr1:160739057	Inverse variance weighted	7	0.040	0.023	8.128E-02	0.881	cis	Hospitalized COVID-19
2637_77	Macrophage mannose receptor	Macrophage mannose receptor 1	P22897	MRC1	chr10:17809348	Inverse variance weighted	8	0.069	0.039	8.130E-02	0.881	cis	Hospitalized COVID-19
8296_117	KDEL2	KDEL motif-containing protein 2	Q7Z4H8	POGLUT3	chr11:108498405	Inverse variance weighted	6	0.048	0.028	8.174E-02	0.881	cis	Hospitalized COVID-19
3340_53	TSP4	Thrombospondin-4	P35443	THBS4	chr5:79991311	Inverse variance weighted	3	-0.076	0.044	8.335E-02	0.881	cis	Hospitalized COVID-19
7211_2	RNase 1	Ribonuclease pancreatic	P07998	RNASE1	chr14:20802855	Inverse variance weighted	4	-0.136	0.079	8.365E-02	0.881	cis	Hospitalized COVID-19
12641_3	I5P2	Type II inositol 1,4,5-trisphosphate 5-phosphatase	P32019	INPP5B	chr1:37947057	Inverse variance weighted	2	0.081	0.047	8.391E-02	0.881	cis	Hospitalized COVID-19
15503_20	Lefty-A	Left-right determination factor 2	O00292	LEFTY2	chr1:225941383	Inverse variance weighted	10	0.042	0.024	8.399E-02	0.881	cis	Hospitalized COVID-19
8035_6	CA198	Uncharacterized protein C1orf198	Q9H425	C1orf198	chr1:230869589	Wald ratio	1	0.154	0.089	8.419E-02	0.881	cis	Hospitalized COVID-19
12428_2	LYPL1	Lysophospholipase-like protein 1	Q5VWZ2	LYPLAL1	chr1:219173869	Wald ratio	1	0.172	0.100	8.432E-02	0.881	cis	Hospitalized COVID-19
19223_6	RAB1A	Ras-related protein Rab-1A	P62820	RAB1A	chr2:65130331	Wald ratio	1	-0.316	0.183	8.540E-02	0.881	cis	Hospitalized COVID-19
18916_25	Inosine triphosphatase	Inosine triphosphate pyrophosphatase	Q9BY32	ITPA	chr20:3208868	Inverse variance weighted	8	-0.038	0.022	8.569E-02	0.881	cis	Hospitalized COVID-19
15513_108	Prostasin	Prostasin	Q16651	PRSS8	chr16:31135727	Inverse variance weighted	2	-0.209	0.122	8.589E-02	0.881	cis	Hospitalized COVID-19
7849_3	Glutaminyl cyclase	Glutaminyl-peptide cyclotransferase	Q16769	QPCT	chr2:37342827	Inverse variance weighted	4	-0.110	0.064	8.608E-02	0.881	cis	Hospitalized COVID-19
9244_27	PPT1	Palmitoyl-protein thioesterase 1	P50897	PPT1	chr1:40097260	Inverse variance weighted	2	0.080	0.047	8.624E-02	0.881	cis	Hospitalized COVID-19
6715_63	PPBN	Alkaline phosphatase, placental-like	P10696	ALPG	chr2:232406844	Inverse variance weighted	3	0.153	0.089	8.668E-02	0.881	cis	Hospitalized COVID-19
18295_102	GRHPR	Glyoxylate reductase/hydroxypyruvate reductase	Q9UBQ7	GRHPR	chr9:37422666	Inverse variance weighted	3	-0.063	0.037	8.669E-02	0.881	cis	Hospitalized COVID-19
16304_6	LGR4	Leucine-rich repeat-containing G-protein coupled receptor 4	Q9BXB1	LGR4	chr11:27472790	Inverse variance weighted	2	-0.158	0.092	8.727E-02	0.882	cis	Hospitalized COVID-19
12975_11	Keratin 20	Keratin, type I cytoskeletal 20	P35900	KRT20	chr17:40885242	Inverse variance weighted	2	-0.279	0.164	8.897E-02	0.893	cis	Hospitalized COVID-19
7933_75	ADA22	Disintegrin and metalloproteinase domain-containing protein 22	Q9P0K1	ADAM22	chr7:87934143	Inverse variance weighted	4	-0.065	0.039	9.005E-02	0.899	cis	Hospitalized COVID-19
10015_119	KCAB2	Voltage-gated potassium channel subunit beta-2	Q13303	KCNAB2	chr1:5990927	Inverse variance weighted	2	-0.226	0.134	9.063E-02	0.899	cis	Hospitalized COVID-19
17691_1	TPP1	Tripeptidyl-peptidase 1	O14773	TPP1	chr11:6619448	Inverse variance weighted	5	-0.092	0.055	9.127E-02	0.899	cis	Hospitalized COVID-19
13119_26	protein Z inhibitor	Protein Z-dependent protease inhibitor	Q9UK55	SERPINA10	chr14:94293268	Inverse variance weighted	6	0.048	0.028	9.246E-02	0.899	cis	Hospitalized COVID-19
7228_2	SIA7F	Alpha-N-acetylgalactosaminide alpha-2,6-sialyltransferase 6	Q969X2	ST6GALNAC6	chr9:127905408	Inverse variance weighted	2	-0.201	0.120	9.289E-02	0.899	cis	Hospitalized COVID-19
8250_2	PTPRJ	Receptor-type tyrosine-protein phosphatase eta	Q12913	PTPRJ	chr11:47980425	Inverse variance weighted	3	-0.213	0.127	9.309E-02	0.899	cis	Hospitalized COVID-19
13943_38	DPY30	Protein dpy-30 homolog	Q9C005	DPY30	chr2:32039805	Wald ratio	1	-0.254	0.151	9.391E-02	0.899	cis	Hospitalized COVID-19
15394_79	UNC5B	Netrin receptor UNC5B	Q8IZJ1	UNC5B	chr10:71212570	Inverse variance weighted	3	0.123	0.074	9.415E-02	0.899	cis	Hospitalized COVID-19
11145_72	K154L	UPF0606 protein KIAA1549L	Q6ZVL6	KIAA1549L	chr11:33376108	Inverse variance weighted	7	-0.050	0.030	9.500E-02	0.899	cis	Hospitalized COVID-19
15322_35	CRADD	Death domain-containing protein CRADD	P78560	CRADD	chr12:93677375	Inverse variance weighted	2	0.360	0.216	9.501E-02	0.899	cis	Hospitalized COVID-19
3796_79	ANGL4	Angiopoietin-related protein 4	Q9BY76	ANGPTL4	chr19:8363289	Wald ratio	1	0.184	0.111	9.722E-02	0.899	cis	Hospitalized COVID-19
2567_5	Factor I	Complement factor I	P05156	CFI	chr4:109802150	Inverse variance weighted	2	0.084	0.051	9.770E-02	0.899	cis	Hospitalized COVID-19
15566_10	Calponin-1	Calponin-1	P51911	CNN1	chr19:11538767	Wald ratio	1	0.134	0.081	9.804E-02	0.899	cis	Hospitalized COVID-19
16818_200	CDCP1	CUB domain-containing protein 1	Q9H5V8	CDCP1	chr3:45146422	Inverse variance weighted	3	-0.064	0.039	9.822E-02	0.899	cis	Hospitalized COVID-19
8310_6	U773	Zymogen granule protein 16 homolog B	Q96DA0	ZG16B	chr16:2830253	Wald ratio	1	0.107	0.065	9.836E-02	0.899	cis	Hospitalized COVID-19
6227_1	kallikrein 10	Kallikrein-10	O43240	KLK10	chr19:51020175	Inverse variance weighted	6	0.079	0.048	9.959E-02	0.899	cis	Hospitalized COVID-19
3457_57	Periostin	Periostin	Q15063	POSTN	chr13:37598844	Inverse variance weighted	4	0.072	0.044	9.989E-02	0.899	cis	Hospitalized COVID-19

10772_21	CGAT2	Chondroitin sulfate N-acetylgalactosaminyltransferase 2	Q8N6G5	CSGALNACT2	chr10:43138445	Wald ratio	1	0.177	0.107	9.996E-02	0.899	cis	Hospitalized COVID-19
19482_11	HDHD3	Haloacid dehalogenase-like hydrolase domain-containing protein 3	Q9BSH5	HDHD3	chr9:113376986	Wald ratio	1	0.091	0.055	1.003E-01	0.899	cis	Hospitalized COVID-19
6925_26	SNX8	Sorting nexin-8	Q9Y5X2	SNX8	chr7:2354318	Inverse variance weighted	2	0.097	0.059	1.008E-01	0.899	cis	Hospitalized COVID-19
5129_12	SREC-I	Scavenger receptor class F member 1	Q14162	SCARF1	chr17:1645744	Inverse variance weighted	5	0.043	0.026	1.014E-01	0.899	cis	Hospitalized COVID-19
14618_26	ECOP	Vesicular, overexpressed in cancer, prosurvival protein 1	Q96AW1	VOPP1	chr7:55572988	Inverse variance weighted	2	-0.147	0.090	1.018E-01	0.899	cis	Hospitalized COVID-19
11428_31	PDLI1	PDZ and LIM domain protein 1	O00151	PDLIM1	chr10:95291012	Wald ratio	1	-0.242	0.148	1.020E-01	0.899	cis	Hospitalized COVID-19
9484_75	Desmoglein-2	Desmoglein-2	Q14126	DSG2	chr18:31498177	Inverse variance weighted	5	0.141	0.086	1.023E-01	0.899	cis	Hospitalized COVID-19
16809_1	NDKM	Nucleoside diphosphate kinase, mitochondrial	O00746	NME4	chr16:396725	Inverse variance weighted	2	0.185	0.113	1.030E-01	0.899	cis	Hospitalized COVID-19
9126_171	NT5D3	5'-nucleotidase domain-containing protein 3	Q86UY8	NT5DC3	chr12:103841234	Inverse variance weighted	2	0.075	0.046	1.037E-01	0.899	cis	Hospitalized COVID-19
2974_61	contactin-1	Contactin-1	Q12860	CNTN1	chr12:40692439	Inverse variance weighted	4	-0.060	0.037	1.054E-01	0.899	cis	Hospitalized COVID-19
15526_33	GSHB	Glutathione synthetase	P48637	GSS	chr20:34956027	Inverse variance weighted	2	0.120	0.074	1.064E-01	0.899	cis	Hospitalized COVID-19
17419_17	TES	Testin	Q9UGI8	TES	chr7:116210506	Wald ratio	1	-0.219	0.135	1.066E-01	0.899	cis	Hospitalized COVID-19
19561_216	PLXD1	Plexin-D1	Q9Y4D7	PLXND1	chr3:129606676	Inverse variance weighted	4	0.075	0.046	1.068E-01	0.899	cis	Hospitalized COVID-19
5722_78	Prolylcarboxypeptidase	Lysosomal Pro-X carboxypeptidase	P42785	PRCP	chr11:82970584	Inverse variance weighted	3	0.100	0.062	1.080E-01	0.899	cis	Hospitalized COVID-19
12522_6	RD23B	UV excision repair protein RAD23 homolog B	P54727	RAD23B	chr9:107283137	Wald ratio	1	0.294	0.183	1.082E-01	0.899	cis	Hospitalized COVID-19
5900_11	HINT1	Histidine triad nucleotide-binding protein 1	P49773	HINT1	chr5:131224468	Wald ratio	1	0.282	0.176	1.085E-01	0.899	cis	Hospitalized COVID-19
12396_19	HIBCH	3-hydroxyisobutyryl-CoA hydrolase, mitochondrial	Q6NVY1	HIBCH	chr2:190344193	Inverse variance weighted	5	0.046	0.029	1.085E-01	0.899	cis	Hospitalized COVID-19
13944_3	SULT 1A3	Sulfotransferase 1A3	P0DMM9	SULT1A3	chr16:30199228	Inverse variance weighted	4	0.150	0.093	1.085E-01	0.899	cis	Hospitalized COVID-19
13384_110	FUMH	Fumarate hydratase, mitochondrial	P07954	FH	chr1:241519799	Wald ratio	1	0.123	0.077	1.087E-01	0.899	cis	Hospitalized COVID-19
17138_8	GST A1-1	Glutathione S-transferase A1	P08263	GSTA1	chr6:52803860	Inverse variance weighted	6	0.054	0.034	1.126E-01	0.911	cis	Hospitalized COVID-19
11656_110	EVL	Ena/VASP-like protein	Q9UI08	EVL	chr14:99971449	Inverse variance weighted	3	0.181	0.114	1.126E-01	0.911	cis	Hospitalized COVID-19
17756_69	DCTD	Deoxycytidylate deaminase	P32321	DCTD	chr4:182917936	Inverse variance weighted	2	0.184	0.116	1.127E-01	0.911	cis	Hospitalized COVID-19
7779_86	CHSTB	Carbohydrate sulfotransferase 11	Q9NPF2	CHST11	chr12:104455295	Inverse variance weighted	5	0.057	0.036	1.127E-01	0.911	cis	Hospitalized COVID-19
8759_29	a1,4-Galactosyltransferase	Lactosylceramide 4-alpha-galactosyltransferase	Q9NPC4	A4GALT	chr22:42721298	Inverse variance weighted	4	0.046	0.029	1.134E-01	0.911	cis	Hospitalized COVID-19
12016_60	CBL	E3 ubiquitin-protein ligase CBL	P22681	CBL	chr11:119206298	Wald ratio	1	0.113	0.072	1.143E-01	0.911	cis	Hospitalized COVID-19
19254_125	GMPR1	GMP reductase 1	P36959	GMPR	chr6:16238587	Inverse variance weighted	8	-0.025	0.016	1.151E-01	0.911	cis	Hospitalized COVID-19
18343_10	DECR2	Peroxisomal 2,4-dienoyl-CoA reductase	Q9NUI1	DECR2	chr16:401858	Inverse variance weighted	5	0.051	0.033	1.156E-01	0.911	cis	Hospitalized COVID-19
9384_17	Cathelicidin peptide	Cathelicidin antimicrobial peptide	P49913	CAMP	chr3:48223347	Wald ratio	1	0.225	0.143	1.162E-01	0.911	cis	Hospitalized COVID-19
17821_20	NMT2	Glycylpeptide N-tetradecanoyltransferase 2	O60551	NMT2	chr10:15168693	Wald ratio	1	0.253	0.161	1.165E-01	0.911	cis	Hospitalized COVID-19
4978_54	DBNL	Drebrin-like protein	Q9UJU6	DBNL	chr7:44044640	Wald ratio	1	0.262	0.167	1.168E-01	0.911	cis	Hospitalized COVID-19
18896_23	H6ST3	Heparan-sulfate 6-O-sulfotransferase 3	Q8IZP7	HS6ST3	chr13:96090107	Wald ratio	1	0.170	0.108	1.172E-01	0.911	cis	Hospitalized COVID-19
2516_57	6Ckine	C-C motif chemokine 21	O00585	CCL21	chr9:34710136	Inverse variance weighted	2	0.210	0.134	1.172E-01	0.911	cis	Hospitalized COVID-19
6986_17	HS3SB	Heparan sulfate glucosamine 3-O-sulfotransferase 3B1	Q9Y662	HS3ST3B1	chr17:14301081	Wald ratio	1	0.341	0.218	1.184E-01	0.912	cis	Hospitalized COVID-19
4234_8	IL-1 R4	Interleukin-1 receptor-like 1	Q01638	IL1RL1	chr2:102311502	Inverse variance weighted	8	-0.037	0.024	1.184E-01	0.912	cis	Hospitalized COVID-19
9348_1	C1RL1	Complement C1r subcomponent-like protein	Q9NZP8	C1RL	chr12:7109238	Wald ratio	1	0.265	0.170	1.193E-01	0.915	cis	Hospitalized COVID-19
18841_1	SPB13	Serpin B13	Q9UIV8	SERPINB13	chr18:63586989	Inverse variance weighted	4	0.081	0.052	1.202E-01	0.918	cis	Hospitalized COVID-19
18313_4	Asparaginase-like protein 1	Isoaspartyl peptidase/L-asparaginase	Q7L266	ASRGL1	chr11:62337448	Inverse variance weighted	2	-0.076	0.049	1.216E-01	0.924	cis	Hospitalized COVID-19
19334_62	TXD12	Thioredoxin domain-containing protein 12	O95881	TXNDC12	chr1:52055191	Wald ratio	1	0.191	0.124	1.246E-01	0.942	cis	Hospitalized COVID-19
2741_22	Siglec-6	Sialic acid-binding Ig-like lectin 6	O43699	SIGLEC6	chr19:51531856	Inverse variance weighted	6	-0.061	0.040	1.271E-01	0.948	cis	Hospitalized COVID-19
13929_27	OCTC	Peroxisomal carnitine O-octanoyltransferase	Q9UKG9	CROT	chr7:87345664	Inverse variance weighted	6	-0.049	0.032	1.272E-01	0.948	cis	Hospitalized COVID-19
4249_64	NDP kinase B	Nucleoside diphosphate kinase B	P22392	NME2	chr17:51165435	Wald ratio	1	0.115	0.075	1.278E-01	0.948	cis	Hospitalized COVID-19
4982_54	Elafin	Elafin	P19957	PI3	chr20:45174902	Inverse variance weighted	5	0.083	0.054	1.278E-01	0.948	cis	Hospitalized COVID-19
9713_67	PGFRL	Platelet-derived growth factor receptor-like protein	Q15198	PDGFRL	chr8:17576433	Inverse variance weighted	6	-0.044	0.029	1.286E-01	0.948	cis	Hospitalized COVID-19
11540_37	FOXO3A	Forkhead box protein O3	O43524	FOXO3	chr6:108559835	Wald ratio	1	-0.308	0.203	1.287E-01	0.948	cis	Hospitalized COVID-19
11241_8	ARLY	Argininosuccinate lyase	P04424	ASL	chr7:66075800	Inverse variance weighted	3	-0.150	0.099	1.305E-01	0.948	cis	Hospitalized COVID-19
2834_54	kallikrein 8	Kallikrein-8	O60259	KLK8	chr19:51002711	Wald ratio	1	0.107	0.071	1.305E-01	0.948	cis	Hospitalized COVID-19
11969_5	S100A2	Protein S100-A2	P29034	S100A2	chr1:153567890	Wald ratio	1	-0.380	0.252	1.312E-01	0.948	cis	Hospitalized COVID-19
8427_118	RSPO3	R-spondin-3	Q9BXY4	RSPO3	chr6:127118671	Inverse variance weighted	4	-0.208	0.138	1.317E-01	0.948	cis	Hospitalized COVID-19
18185_118	ALDOB	Fructose-bisphosphate aldolase B	P05062	ALDOB	chr9:101449664	Inverse variance weighted	2	0.054	0.036	1.321E-01	0.948	cis	Hospitalized COVID-19
13463_1	PXDN	Peroxidasin homolog	Q92626	PXDN	chr2:1744852	Inverse variance weighted	4	0.111	0.074	1.330E-01	0.948	cis	Hospitalized COVID-19
16613_3	CAD17	Cadherin-17	Q12864	CDH17	chr8:94217303	Inverse variance weighted	5	0.051	0.034	1.337E-01	0.948	cis	Hospitalized COVID-19
9478_69	KPRA	Phosphoribosyl pyrophosphate synthase-associated protein 1	Q14558	PRPSAP1	chr17:76384521	Wald ratio	1	-0.232	0.155	1.344E-01	0.948	cis	Hospitalized COVID-19
9834_62	ADH1B	Alcohol dehydrogenase 1B	P00325	ADH1B	chr4:99352760	Wald ratio	1	0.136	0.091	1.349E-01	0.948	cis	Hospitalized COVID-19
9341_1	PDGFD	Platelet-derived growth factor D	Q9GZP0	PDGFD	chr11:104164379	Inverse variance weighted	3	-0.166	0.112	1.368E-01	0.948	cis	Hospitalized COVID-19
3028_36	Ck-b-8-1	Ck-beta-8-1	P55773	CCL23	chr17:36017972	Inverse variance weighted	3	-0.153	0.103	1.371E-01	0.948	cis	Hospitalized COVID-19
5078_82	EphB6	Ephrin type-B receptor 6	O15197	EPHB6	chr7:142855061	Inverse variance weighted	3	0.070	0.047	1.377E-01	0.948	cis	Hospitalized COVID-19
2890_59	CCL28	C-C motif chemokine 28	Q9NRJ3	CCL28	chr5:43412391	Wald ratio	1	-0.315	0.213	1.381E-01	0.948	cis	Hospitalized COVID-19
5202_4	PPID	Peptidyl-prolyl cis-trans isomerase D	Q08752	PPID	chr4:158723396	Inverse variance weighted	2	-0.058	0.039	1.384E-01	0.948	cis	Hospitalized COVID-19
16558_2	MYOC	Myocilin	Q99972	MYOC	chr1:171652688	Inverse variance weighted	8	0.042	0.029	1.386E-01	0.948	cis	Hospitalized COVID-19
4407_10	MSP	Hepatocyte growth factor-like protein	P26927	MST1	chr3:49689501	Inverse variance weighted	4	0.018	0.012	1.386E-01	0.948	cis	Hospitalized COVID-19
9867_23	F16P2	Fructose-1,6-bisphosphatase isozyme 2	O00757	FBP2	chr9:94593824	Wald ratio	1	0.177	0.120	1.392E-01	0.948	cis	Hospitalized COVID-19
18337_4	GMDS	GDP-mannose 4,6 dehydratase	O60547	GMDS	chr6:2245605	Wald ratio	1	-0.292	0.198	1.393E-01	0.948	cis	Hospitalized COVID-19
12475_48	CLIC5	Chloride intracellular channel protein 5	Q9NZA1	CLIC5	chr6:46080348	Inverse variance weighted	5	0.066	0.045	1.393E-01	0.948	cis	Hospitalized COVID-19
12558_3	UBS3B	Ubiquitin-associated and SH3 domain-containing protein B	Q8TF42	UBASH3B	chr11:122655722	Inverse variance weighted	2	-0.110	0.075	1.406E-01	0.948	cis	Hospitalized COVID-19
12498_12	TX1B3	Tax1-binding protein 3	O14907	TAX1BP3	chr17:3668679	Wald ratio	1	0.169	0.116	1.429E-01	0.948	cis	Hospitalized COVID-19
6039_24	CRHBP	Corticotropin-releasing factor-binding protein	P24387	CRHBP	chr5:76953045	Inverse variance weighted	6	0.035	0.024	1.434E-01	0.948	cis	Hospitalized COVID-19
2480_58	TIMP-3	Metalloproteinase inhibitor 3	P35625	TIMP3	chr22:32801705	Inverse variance weighted	10	0.040	0.028	1.438E-01	0.948	cis	Hospitalized COVID-19
14006_36	GNMT	Glycine N-methyltransferase	Q14749	GNMT	chr6:42960754	Wald ratio	1	-0.133	0.091	1.441E-01	0.948	cis	Hospitalized COVID-19
10521_10	MXRA8:ECD	Matrix-remodeling-associated protein 8:Extracellular domain	Q9BRK3	MXRA8	chr1:1361777	Wald ratio	1	-0.158	0.109	1.451E-01	0.948	cis	Hospitalized COVID-19
15603_20	Integrin alpha-2	Integrin alpha-2	P17301	ITGA2	chr5:52989340	Wald ratio	1	0.160	0.110	1.453E-01	0.948	cis	Hospitalized COVID-19
17682_1	CD46	Membrane cofactor protein	P15529	CD46	chr1:207752037	Inverse variance weighted	2	0.081	0.056	1.455E-01	0.948	cis	Hospitalized COVID-19
3431_54	EphA1	Ephrin type-A receptor 1	P21709	EPHA1	chr7:143408856	Inverse variance weighted	7	-0.029	0.020	1.459E-01	0.948	cis	Hospitalized COVID-19
5810_25	Cripto	Teratocarcinoma-derived growth factor 1	P13385	TDGF1	chr3:46574534	Inverse variance weighted	6	0.126	0.087	1.469E-01	0.948	cis	Hospitalized COVID-19
3605_77	MASP3:Light	Mannan-binding lectin serine protease 1:Mannan-binding lectin serine protease 1 light chain	P48740	MASP1	chr3:187291980	Wald ratio	1	0.189	0.130	1.471E-01	0.948	cis	Hospitalized COVID-19
8859_51	CAH11	Carbonic anhydrase-related protein 11	O75493	CA11	chr19:48646187	Wald ratio	1	0.142	0.098	1.473E-01	0.948	cis	Hospitalized COVID-19
6359_50	AGO61	Protein O-linked-mannose beta-1,4-N-acetylglucosaminyltransferase 2	Q8NAT1	POMGNT2	chr3:43106085	Inverse variance weighted	4	-0.081	0.056	1.479E-01	0.948	cis	Hospitalized COVID-19
3807_1	FGF23	Fibroblast growth factor 23	Q9GZV9	FGF23	chr12:4379712	Wald ratio	1	-0.286	0.198	1.494E-01	0.948	cis	Hospitalized COVID-19
2620_4	gp130, soluble	Interleukin-6 receptor subunit beta	P40189	IL6ST	chr5:55995022	Inverse variance weighted	4	0.052	0.036	1.496E-01	0.948	cis	Hospitalized COVID-19

19187_21	STABP	STAM-binding protein	O95630	STAMBP	chr2:73828916	Inverse variance weighted	2	0.213	0.149	1.508E-01	0.948	cis	Hospitalized COVID-19
9183_7	IFN-a/b R1	Interferon alpha/beta receptor 1	P17181	IFNAR1	chr21:33324387	Inverse variance weighted	7	0.042	0.030	1.529E-01	0.948	cis	Hospitalized COVID-19
16055_3	complement factor H-related 5	Complement factor H-related protein 5	Q9BXR6	CFHR5	chr1:196975010	Inverse variance weighted	5	-0.054	0.038	1.533E-01	0.948	cis	Hospitalized COVID-19
12571_14	ARL3	ADP-ribosylation factor-like protein 3	P36405	ARL3	chr10:102714397	Inverse variance weighted	2	0.095	0.067	1.541E-01	0.948	cis	Hospitalized COVID-19
16828_8	Collagen a1(VI)	Collagen alpha-1(VI) chain	P12109	COL6A1	chr21:45981770	Inverse variance weighted	10	-0.028	0.020	1.550E-01	0.948	cis	Hospitalized COVID-19
13954_9	GNA1	Glucosamine 6-phosphate N-acetyltransferase	Q96EK6	GNPNAT1	chr14:52791668	Wald ratio	1	-0.255	0.180	1.556E-01	0.948	cis	Hospitalized COVID-19
8794_13	DPEP1	Dipeptidase 1	P16444	DPEP1	chr16:89613308	Inverse variance weighted	6	0.046	0.032	1.563E-01	0.948	cis	Hospitalized COVID-19
17329_2	BDH2	3-hydroxybutyrate dehydrogenase type 2	Q9BUT1	BDH2	chr4:103099870	Inverse variance weighted	2	0.088	0.062	1.572E-01	0.948	cis	Hospitalized COVID-19
15298_199	NETO1	Neuropilin and tolloid-like protein 1	Q8TDF5	NETO1	chr18:72868146	Inverse variance weighted	2	-0.210	0.148	1.573E-01	0.948	cis	Hospitalized COVID-19
10702_1	COSA1	Collagen alpha-1(XXVIII) chain	Q2UY09	COL28A1	chr7:7535873	Wald ratio	1	0.317	0.224	1.580E-01	0.948	cis	Hospitalized COVID-19
9026_40	BTNL8	Butyrophilin-like protein 8	Q6UX41	BTNL8	chr5:180899077	Inverse variance weighted	2	-0.090	0.064	1.586E-01	0.948	cis	Hospitalized COVID-19
15615_8	LIRB3	Leukocyte immunoglobulin-like receptor subfamily B member 3	O75022	LILRB3	chr19:54223506	Inverse variance weighted	5	-0.060	0.043	1.587E-01	0.948	cis	Hospitalized COVID-19
18312_68	NDRG3	Protein NDRG3	Q9UGV2	NDRG3	chr20:36746090	Inverse variance weighted	3	-0.148	0.105	1.591E-01	0.948	cis	Hospitalized COVID-19
10833_64	HHIP	Hedgehog-interacting protein	Q96QV1	HHIP	chr4:144646156	Inverse variance weighted	2	-0.093	0.066	1.603E-01	0.948	cis	Hospitalized COVID-19
14012_17	LGP2	Probable ATP-dependent RNA helicase DHX58	Q96C10	DHX58	chr17:42112714	Inverse variance weighted	2	-0.107	0.076	1.608E-01	0.948	cis	Hospitalized COVID-19
15441_6	SAP3	Ganglioside GM2 activator	P17900	GM2A	chr5:151212150	Inverse variance weighted	3	-0.053	0.038	1.616E-01	0.948	cis	Hospitalized COVID-19
2631_50	IL-10 Rb	Interleukin-10 receptor subunit beta	Q08334	IL10RB	chr21:33266367	Wald ratio	1	0.232	0.166	1.619E-01	0.948	cis	Hospitalized COVID-19
13740_51	sFRP-3	Secreted frizzled-related protein 3	Q92765	FRZB	chr2:182866637	Inverse variance weighted	8	-0.032	0.023	1.628E-01	0.948	cis	Hospitalized COVID-19
2617_56	ERBB3	Receptor tyrosine-protein kinase erbB-3	P21860	ERBB3	chr12:56076799	Wald ratio	1	0.127	0.091	1.628E-01	0.948	cis	Hospitalized COVID-19
2950_57	IGFBP-4	Insulin-like growth factor-binding protein 4	P22692	IGFBP4	chr17:40443450	Wald ratio	1	-0.262	0.188	1.633E-01	0.948	cis	Hospitalized COVID-19
11649_3	SMAP1	Stromal membrane-associated protein 1	Q8IYB5	SMAP1	chr6:70667776	Inverse variance weighted	4	-0.049	0.035	1.635E-01	0.948	cis	Hospitalized COVID-19
3045_72	PTN	Pleiotrophin	P21246	PTN	chr7:137343774	Inverse variance weighted	3	-0.070	0.050	1.661E-01	0.948	cis	Hospitalized COVID-19
6151_18	MP2K3	Dual specificity mitogen-activated protein kinase kinase 3	P46734	MAP2K3	chr17:21284672	Wald ratio	1	0.316	0.228	1.662E-01	0.948	cis	Hospitalized COVID-19
5728_60	FCRL1	Fc receptor-like protein 1	Q96LA6	FCRL1	chr1:157820120	Inverse variance weighted	4	-0.109	0.079	1.665E-01	0.948	cis	Hospitalized COVID-19
10781_19	CLC4G	C-type lectin domain family 4 member G	Q6UXB4	CLEC4G	chr19:7733906	Wald ratio	1	0.380	0.275	1.668E-01	0.948	cis	Hospitalized COVID-19
7921_65	FJX1	Four-jointed box protein 1	Q86VR8	FJX1	chr11:35618460	Inverse variance weighted	4	0.055	0.040	1.680E-01	0.948	cis	Hospitalized COVID-19
4911_49	Glutathione S-transferase Pi	Glutathione S-transferase P	P09211	GSTP1	chr11:67583742	Inverse variance weighted	2	-0.192	0.139	1.692E-01	0.948	cis	Hospitalized COVID-19
9233_71	TFPI -2	Tissue factor pathway inhibitor 2	P48307	TFPI2	chr7:93890753	Wald ratio	1	-0.296	0.216	1.703E-01	0.948	cis	Hospitalized COVID-19
9995_6	DUT	Deoxyuridine 5'-triphosphate nucleotidohydrolase, mitochondrial	P33316	DUT	chr15:48331011	Wald ratio	1	0.224	0.164	1.707E-01	0.948	cis	Hospitalized COVID-19
12348_46	SYSM	Serine--tRNA ligase, mitochondrial	Q9NP81	SARS2	chr19:38930763	Wald ratio	1	0.125	0.091	1.712E-01	0.948	cis	Hospitalized COVID-19
14636_25	Ribonuclease UK114	Ribonuclease UK114	P52758	RIDA	chr8:98117171	Inverse variance weighted	3	-0.071	0.052	1.712E-01	0.948	cis	Hospitalized COVID-19
18241_18	HEM6	Oxygen-dependent coproporphyrinogen-III oxidase, mitochondrial	P36551	CPOX	chr3:98593648	Inverse variance weighted	4	0.075	0.055	1.716E-01	0.948	cis	Hospitalized COVID-19
14708_59	CO8G	Complement component C8 gamma chain	P07360	C8G	chr9:136945185	Inverse variance weighted	5	0.093	0.068	1.716E-01	0.948	cis	Hospitalized COVID-19
3232_28	TrATPase	Tartrate-resistant acid phosphatase type 5	P13686	ACP5	chr19:11579993	Inverse variance weighted	4	0.062	0.046	1.717E-01	0.948	cis	Hospitalized COVID-19
3461_58	PGCB	Brevican core protein	Q96GW7	BCAN	chr1:156641390	Inverse variance weighted	2	0.069	0.051	1.728E-01	0.948	cis	Hospitalized COVID-19
17514_48	RAB21	Ras-related protein Rab-21	Q9UL25	RAB21	chr12:71754863	Wald ratio	1	-0.155	0.114	1.729E-01	0.948	cis	Hospitalized COVID-19
13682_47	M-CSF R	Macrophage colony-stimulating factor 1 receptor	P07333	CSF1R	chr5:150113372	Wald ratio	1	-0.216	0.159	1.736E-01	0.948	cis	Hospitalized COVID-19
17739_1	HCDH	Hydroxyacyl-coenzyme A dehydrogenase, mitochondrial	Q16836	HADH	chr4:107989714	Wald ratio	1	-0.200	0.147	1.737E-01	0.948	cis	Hospitalized COVID-19
17832_12	IDI2	Isopentenyl-diphosphate delta-isomerase 2	Q9BXS1	IDI2	chr10:1025859	Inverse variance weighted	2	0.089	0.066	1.747E-01	0.948	cis	Hospitalized COVID-19
6404_20	C1QRF	C1q-related factor	O75973	C1QL1	chr17:44968303	Inverse variance weighted	3	-0.087	0.064	1.754E-01	0.948	cis	Hospitalized COVID-19
2994_71	IL-1Rrp2	Interleukin-1 receptor-like 2	Q9HB29	IL1RL2	chr2:102187006	Wald ratio	1	-0.098	0.072	1.767E-01	0.948	cis	Hospitalized COVID-19
6629_3	HBD-1	Beta-defensin 1	P60022	DEFB1	chr8:6877936	Inverse variance weighted	5	-0.040	0.030	1.771E-01	0.948	cis	Hospitalized COVID-19
16916_19	SLIK6	SLIT and NTRK-like protein 6	Q9H5Y7	SLITRK6	chr13:85806683	Inverse variance weighted	4	-0.091	0.068	1.773E-01	0.948	cis	Hospitalized COVID-19
18162_167	IRAK4	Interleukin-1 receptor-associated kinase 4	Q9NWZ3	IRAK4	chr12:43758944	Wald ratio	1	-0.173	0.129	1.781E-01	0.948	cis	Hospitalized COVID-19
18197_97	KCRS	Creatine kinase S-type, mitochondrial	P17540	CKMT2	chr5:81233320	Wald ratio	1	0.288	0.214	1.785E-01	0.948	cis	Hospitalized COVID-19
3316_58	Heparin cofactor II	Heparin cofactor 2	P05546	SERPIND1	chr22:20774113	Wald ratio	1	0.280	0.208	1.795E-01	0.948	cis	Hospitalized COVID-19
12329_21	KS6A1	Ribosomal protein S6 kinase alpha-1	Q15418	RPS6KA1	chr1:26529761	Wald ratio	1	-0.143	0.107	1.798E-01	0.948	cis	Hospitalized COVID-19
8006_12	DJB12	DnaJ homolog subfamily B member 12	Q9NXW2	DNAJB12	chr10:72355149	Wald ratio	1	0.156	0.117	1.807E-01	0.948	cis	Hospitalized COVID-19
3719_2	p27Kip1	Cyclin-dependent kinase inhibitor 1B	P46527	CDKN1B	chr12:12685498	Wald ratio	1	-0.189	0.141	1.808E-01	0.948	cis	Hospitalized COVID-19
15635_4	SMOC2	SPARC-related modular calcium-binding protein 2	Q9H3U7	SMOC2	chr6:168441151	Inverse variance weighted	5	-0.048	0.036	1.815E-01	0.948	cis	Hospitalized COVID-19
9249_17	TMEM9:ECD	Transmembrane protein 9:Extracellular domain	Q9P0T7	TMEM9	chr1:201171574	Wald ratio	1	-0.214	0.160	1.819E-01	0.948	cis	Hospitalized COVID-19
7049_2	ADAM 23	Disintegrin and metalloproteinase domain-containing protein 23	O75077	ADAM23	chr2:206443532	Inverse variance weighted	6	0.022	0.017	1.826E-01	0.948	cis	Hospitalized COVID-19
12556_7	UBE2C	Ubiquitin-conjugating enzyme E2 C	O00762	UBE2C	chr20:45812576	Inverse variance weighted	2	0.123	0.093	1.829E-01	0.948	cis	Hospitalized COVID-19
8100_15	ADM2	ADM2	Q7Z4H4	ADM2	chr22:50481543	Wald ratio	1	0.288	0.216	1.829E-01	0.948	cis	Hospitalized COVID-19
3773_15	sTie-2	Angiopoietin-1 receptor, soluble	Q02763	TEK	chr9:27109141	Inverse variance weighted	4	0.065	0.049	1.840E-01	0.948	cis	Hospitalized COVID-19
2968_61	TNFSF15	Tumor necrosis factor ligand superfamily member 15	O95150	TNFSF15	chr9:114806039	Wald ratio	1	-0.251	0.189	1.845E-01	0.948	cis	Hospitalized COVID-19
5000_52	LG3BP	Galectin-3-binding protein	Q08380	LGALS3BP	chr17:78979947	Inverse variance weighted	3	-0.102	0.077	1.846E-01	0.948	cis	Hospitalized COVID-19
18871_24	AIF1L	Allograft inflammatory factor 1-like	Q9BQI0	AIF1L	chr9:131096476	Inverse variance weighted	5	-0.045	0.034	1.854E-01	0.948	cis	Hospitalized COVID-19
15582_25	FCN1	Ficolin-1	O00602	FCN1	chr9:134917912	Inverse variance weighted	5	0.048	0.036	1.871E-01	0.948	cis	Hospitalized COVID-19
3213_65	Nidogen	Nidogen-1	P14543	NID1	chr1:236065109	Inverse variance weighted	4	0.083	0.063	1.876E-01	0.948	cis	Hospitalized COVID-19
9322_15	RCN1	Reticulocalbin-1	Q15293	RCN1	chr11:32091074	Wald ratio	1	-0.103	0.079	1.881E-01	0.948	cis	Hospitalized COVID-19
3216_2	PIGR	Polymeric immunoglobulin receptor	P01833	PIGR	chr1:206946466	Wald ratio	1	0.202	0.154	1.887E-01	0.948	cis	Hospitalized COVID-19
6626_81	CHSTC	Carbohydrate sulfotransferase 12	Q9NRB3	CHST12	chr7:2403588	Inverse variance weighted	2	-0.073	0.056	1.898E-01	0.948	cis	Hospitalized COVID-19
8840_61	C1s	Complement C1s subcomponent	P09871	C1S	chr12:6988259	Inverse variance weighted	4	0.118	0.090	1.901E-01	0.948	cis	Hospitalized COVID-19
9765_4	NDE1	Nuclear distribution protein nudE homolog 1	Q9NXR1	NDE1	chr16:15643267	Wald ratio	1	0.191	0.146	1.909E-01	0.948	cis	Hospitalized COVID-19
15427_35	LOXL3	Lysyl oxidase homolog 3	P58215	LOXL3	chr2:74555690	Wald ratio	1	-0.155	0.119	1.912E-01	0.948	cis	Hospitalized COVID-19
2190_55	Coagulation Factor XI	Coagulation Factor XI	P03951	F11	chr4:186266189	Inverse variance weighted	4	-0.053	0.041	1.915E-01	0.948	cis	Hospitalized COVID-19
9312_8	AZGP1	Zinc-alpha-2-glycoprotein	P25311	AZGP1	chr7:99976042	Inverse variance weighted	3	-0.227	0.174	1.916E-01	0.948	cis	Hospitalized COVID-19
8007_19	Cathepsin B	Cathepsin B	P07858	CTSB	chr8:11869533	Inverse variance weighted	6	0.029	0.022	1.918E-01	0.948	cis	Hospitalized COVID-19
18215_5	THG1	Probable tRNA(His) guanylyltransferase	Q9NWX6	THG1L	chr5:157731420	Inverse variance weighted	3	-0.034	0.026	1.940E-01	0.948	cis	Hospitalized COVID-19
9962_1	MUCDL	Cadherin-related family member 5	Q9HBB8	CDHR5	chr11:626078	Wald ratio	1	-0.236	0.182	1.950E-01	0.948	cis	Hospitalized COVID-19
7905_30	HPT	Haptoglobin isoform 2	P00738	HP	chr16:72054505	Wald ratio	1	0.053	0.041	1.950E-01	0.948	cis	Hospitalized COVID-19
19176_27	FA49B	Protein FAM49B	Q9NUQ9	CYRIB	chr8:130017504	Wald ratio	1	0.282	0.217	1.951E-01	0.948	cis	Hospitalized COVID-19
16856_79	MARE2	Microtubule-associated protein RP/EB family member 2	Q15555	MAPRE2	chr18:34976928	Wald ratio	1	-0.088	0.068	1.957E-01	0.948	cis	Hospitalized COVID-19
17799_9	6PGL	6-phosphogluconolactonase	O95336	PGLS	chr19:17511636	Inverse variance weighted	3	-0.130	0.100	1.959E-01	0.948	cis	Hospitalized COVID-19
13405_61	ISK2	Serine protease inhibitor Kazal-type 2	P20155	SPINK2	chr4:56821742	Inverse variance weighted	5	-0.033	0.026	1.960E-01	0.948	cis	Hospitalized COVID-19
11178_21	SVEP1:EGF-like domains 4-6	Sushi, von Willebrand factor type A, EGF and pentraxin domain-containing protein 1:EGF-like domains 4-6	Q4LDE5	SVEP1	chr9:110579880	Inverse variance weighted	4	0.089	0.069	1.970E-01	0.948	cis	Hospitalized COVID-19
9482_110	NUDT9	ADP-ribose pyrophosphatase, mitochondrial	Q9BW91	NUDT9	chr4:87422573	Wald ratio	1	-0.222	0.172	1.977E-01	0.948	cis	Hospitalized COVID-19

4568_17	SLIK5	SLIT and NTRK-like protein 5	O94991	SLITRK5	chr13:87671371	Inverse variance weighted	4	0.081	0.063	1.980E-01	0.948	cis	Hospitalized COVID-19
17726_3	SAR1A	GTP-binding protein SAR1a	Q9NR31	SAR1A	chr10:70170523	Inverse variance weighted	2	0.132	0.103	1.989E-01	0.948	cis	Hospitalized COVID-19
19622_7	Activin A	Activin A	P08476	INHBA	chr7:41705834	Wald ratio	1	0.243	0.189	1.994E-01	0.948	cis	Hospitalized COVID-19
19602_36	jun-D	Transcription factor jun-D	P17535	JUND	chr19:18281622	Wald ratio	1	-0.266	0.208	2.011E-01	0.948	cis	Hospitalized COVID-19
11192_168	TINAL	Tubulointerstitial nephritis antigen-like	Q9GZM7	TINAGL1	chr1:31576485	Wald ratio	1	-0.132	0.103	2.020E-01	0.948	cis	Hospitalized COVID-19
10372_18	STAT6	Signal transducer and activator of transcription 6	P42226	STAT6	chr12:57132139	Inverse variance weighted	2	0.091	0.071	2.028E-01	0.948	cis	Hospitalized COVID-19
7140_1	ELA2A	Chymotrypsin-like elastase family member 2A	P08217	CELA2A	chr1:15456728	Wald ratio	1	0.136	0.107	2.033E-01	0.948	cis	Hospitalized COVID-19
9197_4	LEG9	Galectin-9	O00182	LGALS9	chr17:27629798	Wald ratio	1	-0.118	0.093	2.035E-01	0.948	cis	Hospitalized COVID-19
13686_2	IL-5 Ra	Interleukin-5 receptor subunit alpha	Q01344	IL5RA	chr3:3126613	Inverse variance weighted	6	0.031	0.025	2.050E-01	0.948	cis	Hospitalized COVID-19
8923_94	GLTL1	Polypeptide N-acetylgalactosaminyltransferase 16	Q8N428	GALNT16	chr14:69259277	Inverse variance weighted	4	-0.079	0.062	2.055E-01	0.948	cis	Hospitalized COVID-19
3290_50	CD109	CD109 antigen	Q6YHK3	CD109	chr6:73695785	Inverse variance weighted	7	0.031	0.024	2.055E-01	0.948	cis	Hospitalized COVID-19
10490_3	RPN1:CD	Dolichyl-diphosphooligosaccharide--protein glycosyltransferase subunit 1:Cytoplasmic domain	P04843	RPN1	chr3:128681075	Wald ratio	1	-0.081	0.064	2.075E-01	0.948	cis	Hospitalized COVID-19
3554_24	Adiponectin	Adiponectin	Q15848	ADIPOQ	chr3:186842704	Inverse variance weighted	4	-0.058	0.046	2.077E-01	0.948	cis	Hospitalized COVID-19
5963_9	Dermokine	Dermokine	Q6E0U4	DMKN	chr19:35513658	Wald ratio	1	-0.241	0.192	2.093E-01	0.948	cis	Hospitalized COVID-19
14088_38	IGFBP-6	Insulin-like growth factor-binding protein 6	P24592	IGFBP6	chr12:53097436	Wald ratio	1	-0.180	0.143	2.097E-01	0.948	cis	Hospitalized COVID-19
5132_71	TCCR	Interleukin-27 receptor subunit alpha	Q6UWB1	IL27RA	chr19:14031762	Inverse variance weighted	7	-0.027	0.022	2.103E-01	0.948	cis	Hospitalized COVID-19
5638_23	GT251	Procollagen galactosyltransferase 1	Q8NBJ5	COLGALT1	chr19:17555649	Inverse variance weighted	3	0.086	0.069	2.105E-01	0.948	cis	Hospitalized COVID-19
15486_126	ABP1	Amiloride-sensitive amine oxidase [copper-containing]	P19801	AOC1	chr7:150824627	Inverse variance weighted	10	-0.022	0.018	2.108E-01	0.948	cis	Hospitalized COVID-19
15641_20	TEFF1	Tomoregulin-1	Q8IYR6	TMEFF1	chr9:100473149	Wald ratio	1	-0.224	0.179	2.113E-01	0.948	cis	Hospitalized COVID-19
4464_10	Sialoadhesin	Sialoadhesin	Q9BZZ2	SIGLEC1	chr20:3712600	Wald ratio	1	0.274	0.219	2.116E-01	0.948	cis	Hospitalized COVID-19
13397_88	HBD-2	Beta-defensin 4A	O15263	DEFB4A	chr8:7894677	Inverse variance weighted	3	0.099	0.079	2.122E-01	0.948	cis	Hospitalized COVID-19
14156_33	14-3-3 protein beta/alpha	14-3-3 protein beta/alpha	P31946	YWHAB	chr20:44885702	Wald ratio	1	0.229	0.184	2.122E-01	0.948	cis	Hospitalized COVID-19
2813_11	ART	Agouti-related protein	O00253	AGRP	chr16:67483547	Inverse variance weighted	2	0.068	0.054	2.130E-01	0.948	cis	Hospitalized COVID-19
15385_116	FABP2	Fatty acid-binding protein, intestinal	P12104	FABP2	chr4:119322138	Inverse variance weighted	5	0.034	0.028	2.140E-01	0.948	cis	Hospitalized COVID-19
5676_54	ASIP	Agouti-signaling protein	P42127	ASIP	chr20:34194569	Wald ratio	1	0.035	0.028	2.143E-01	0.948	cis	Hospitalized COVID-19
18235_16	PGP	Glycerol-3-phosphate phosphatase	A6NDG6	PGP	chr16:2214840	Inverse variance weighted	2	0.104	0.084	2.160E-01	0.948	cis	Hospitalized COVID-19
3009_3	TGF-b R III	Transforming growth factor beta receptor type 3	Q03167	TGFBR3	chr1:91906335	Inverse variance weighted	2	-0.138	0.111	2.163E-01	0.948	cis	Hospitalized COVID-19
2972_57	BMP-7	Bone morphogenetic protein 7	P18075	BMP7	chr20:57266641	Wald ratio	1	0.219	0.178	2.171E-01	0.948	cis	Hospitalized COVID-19
9876_20	aldolase C	Fructose-bisphosphate aldolase C	P09972	ALDOC	chr17:28576948	Inverse variance weighted	2	-0.198	0.161	2.172E-01	0.948	cis	Hospitalized COVID-19
15487_164	carboxylesterase, liver	Liver carboxylesterase 1	P23141	CES1	chr16:55833337	Wald ratio	1	0.269	0.218	2.175E-01	0.948	cis	Hospitalized COVID-19
11424_4	FAAA	Fumarylacetoacetase	P16930	FAH	chr15:80152490	Inverse variance weighted	5	-0.026	0.021	2.175E-01	0.948	cis	Hospitalized COVID-19
18917_53	Pancreatic alpha-amylase	Pancreatic alpha-amylase	P04746	AMY2A	chr1:103617427	Inverse variance weighted	4	-0.127	0.103	2.191E-01	0.948	cis	Hospitalized COVID-19
19117_3	PP14A	Protein phosphatase 1 regulatory subunit 14A	Q96A00	PPP1R14A	chr19:38256532	Wald ratio	1	0.224	0.182	2.195E-01	0.948	cis	Hospitalized COVID-19
4126_22	BPI	Bactericidal permeability-increasing protein	P17213	BPI	chr20:38304150	Inverse variance weighted	9	0.019	0.015	2.200E-01	0.948	cis	Hospitalized COVID-19
16792_4	Siglec-5	Sialic acid-binding Ig-like lectin 5	O15389	SIGLEC5	chr19:51630401	Inverse variance weighted	5	0.037	0.030	2.204E-01	0.948	cis	Hospitalized COVID-19
7096_30	RMD1	Regulator of microtubule dynamics protein 1	Q96DB5	RMDN1	chr8:86514357	Inverse variance weighted	4	0.043	0.035	2.206E-01	0.948	cis	Hospitalized COVID-19
17148_7	BLVRB	Flavin reductase (NADPH)	P30043	BLVRB	chr19:40465764	Inverse variance weighted	3	-0.056	0.046	2.211E-01	0.948	cis	Hospitalized COVID-19
16596_25	GLRX3	Glutaredoxin-3	O76003	GLRX3	chr10:130136391	Wald ratio	1	-0.156	0.127	2.215E-01	0.948	cis	Hospitalized COVID-19
2828_82	HAI-1	Kunitz-type protease inhibitor 1	O43278	SPINT1	chr15:40844018	Inverse variance weighted	2	-0.114	0.094	2.222E-01	0.948	cis	Hospitalized COVID-19
9744_139	DNJA4	DnaJ homolog subfamily A member 4	Q8WW22	DNAJA4	chr15:78264086	Wald ratio	1	0.211	0.173	2.233E-01	0.948	cis	Hospitalized COVID-19
19446_1	GMPR2	GMP reductase 2	Q9P2T1	GMPR2	chr14:24232422	Inverse variance weighted	2	0.057	0.047	2.236E-01	0.948	cis	Hospitalized COVID-19
10722_13	KSYK:Protein Kinase	Tyrosine-protein kinase SYK:Protein kinase domain	P43405	SYK	chr9:90801787	Wald ratio	1	0.254	0.209	2.238E-01	0.948	cis	Hospitalized COVID-19
9848_22	Cyclin H	Cyclin-H	P51946	CCNH	chr5:87412930	Wald ratio	1	-0.148	0.122	2.239E-01	0.948	cis	Hospitalized COVID-19
13124_20	ISLR2	Immunoglobulin superfamily containing leucine-rich repeat protein 2	Q6UXK2	ISLR2	chr15:74100311	Inverse variance weighted	4	-0.064	0.053	2.244E-01	0.948	cis	Hospitalized COVID-19
4920_10	Lysozyme	Lysozyme C	P61626	LYZ	chr12:69348381	Inverse variance weighted	6	-0.046	0.038	2.245E-01	0.948	cis	Hospitalized COVID-19
2742_68	Siglec-7	Sialic acid-binding Ig-like lectin 7	Q9Y286	SIGLEC7	chr19:51142299	Wald ratio	1	0.121	0.100	2.264E-01	0.948	cis	Hospitalized COVID-19
7009_8	CD72	B-cell differentiation antigen CD72	P21854	CD72	chr9:35646810	Inverse variance weighted	2	0.108	0.090	2.265E-01	0.948	cis	Hospitalized COVID-19
9076_25	PENK	Proenkephalin-A	P01210	PENK	chr8:56446671	Inverse variance weighted	5	0.024	0.020	2.266E-01	0.948	cis	Hospitalized COVID-19
6081_52	PCOC2	Procollagen C-endopeptidase enhancer 2	Q9UKZ9	PCOLCE2	chr3:142889206	Inverse variance weighted	10	-0.032	0.027	2.268E-01	0.948	cis	Hospitalized COVID-19
12524_18	SAT2	Diamine acetyltransferase 2	Q96F10	SAT2	chr17:7627876	Inverse variance weighted	3	-0.084	0.069	2.272E-01	0.948	cis	Hospitalized COVID-19
7015_8	LIRB5	Leukocyte immunoglobulin-like receptor subfamily B member 5	O75023	LILRB5	chr19:54257301	Inverse variance weighted	6	0.015	0.013	2.280E-01	0.948	cis	Hospitalized COVID-19
13095_51	PSP	Lithostathine-1-alpha	P05451	REG1A	chr2:79120362	Inverse variance weighted	4	0.127	0.105	2.285E-01	0.948	cis	Hospitalized COVID-19
7128_9	VWA2	von Willebrand factor A domain-containing protein 2	Q5GFL6	VWA2	chr10:114239254	Inverse variance weighted	6	0.049	0.041	2.289E-01	0.948	cis	Hospitalized COVID-19
8080_24	PSMP	Prostate-associated microseminoprotein	Q1L6U9	MSMP	chr9:35756613	Wald ratio	1	0.187	0.155	2.292E-01	0.948	cis	Hospitalized COVID-19
4162_54	Transferrin	Serotransferrin	P02787	TF	chr3:133746040	Inverse variance weighted	2	0.078	0.065	2.292E-01	0.948	cis	Hospitalized COVID-19
8795_48	TR:ECD	Transferrin receptor protein 1:Extracellular domain	P02786	TFRC	chr3:196082153	Wald ratio	1	0.163	0.136	2.305E-01	0.948	cis	Hospitalized COVID-19
8345_27	GPX7	Glutathione peroxidase 7	Q96SL4	GPX7	chr1:52602371	Inverse variance weighted	7	0.027	0.023	2.315E-01	0.948	cis	Hospitalized COVID-19
8300_82	PEX14:N-term	Peroxisomal membrane protein PEX14:N-term	O75381	PEX14	chr1:10472288	Wald ratio	1	0.252	0.211	2.322E-01	0.948	cis	Hospitalized COVID-19
4294_16	Sphingosine kinase 1	Sphingosine kinase 1	Q9NYA1	SPHK1	chr17:76376584	Inverse variance weighted	2	0.116	0.097	2.325E-01	0.948	cis	Hospitalized COVID-19
9826_135	Fragile histidine triad protein	Bis(5'-adenosyl)-triphosphatase	P49789	FHIT	chr3:61251459	Inverse variance weighted	2	0.148	0.124	2.332E-01	0.948	cis	Hospitalized COVID-19
3326_58	Nectin-like protein 2	Cell adhesion molecule 1	Q9BY67	CADM1	chr11:115504957	Wald ratio	1	0.088	0.074	2.337E-01	0.948	cis	Hospitalized COVID-19
17738_7	CRNN	Cornulin	Q9UBG3	CRNN	chr1:152414263	Wald ratio	1	0.091	0.077	2.351E-01	0.948	cis	Hospitalized COVID-19
12366_16	CRGD	Gamma-crystallin D	P07320	CRYGD	chr2:208124524	Wald ratio	1	0.113	0.096	2.366E-01	0.948	cis	Hospitalized COVID-19
18380_78	Albumin	Serum albumin	P02768	ALB	chr4:73397114	Wald ratio	1	0.228	0.193	2.370E-01	0.948	cis	Hospitalized COVID-19
16302_11	SPLC2	BPI fold-containing family A member 2	Q96DR5	BPIFA2	chr20:33161768	Wald ratio	1	-0.189	0.160	2.373E-01	0.948	cis	Hospitalized COVID-19
9278_9	SDF-1	Stromal cell-derived factor 1	P48061	CXCL12	chr10:44386493	Inverse variance weighted	2	0.136	0.115	2.386E-01	0.948	cis	Hospitalized COVID-19
4159_130	Factor H	Complement factor H	P08603	CFH	chr1:196651754	Wald ratio	1	0.166	0.141	2.388E-01	0.948	cis	Hospitalized COVID-19
2962_50	PTHrP	Parathyroid hormone-related protein	P12272	PTHLH	chr12:27972733	Inverse variance weighted	3	0.089	0.075	2.388E-01	0.948	cis	Hospitalized COVID-19
13427_66	MA1C1	Mannosyl-oligosaccharide 1,2-alpha-mannosidase IC	Q9NR34	MAN1C1	chr1:25616791	Inverse variance weighted	3	-0.060	0.051	2.388E-01	0.948	cis	Hospitalized COVID-19
4874_3	Angiogenin	Angiogenin	P03950	ANG	chr14:20684177	Inverse variance weighted	5	0.056	0.048	2.391E-01	0.948	cis	Hospitalized COVID-19
11696_7	RABP2	Cellular retinoic acid-binding protein 2	P29373	CRABP2	chr1:156705816	Inverse variance weighted	3	-0.068	0.058	2.392E-01	0.948	cis	Hospitalized COVID-19
8877_22	F176C:ECD	Protein eva-1 homolog C:Extracellular domain	P58658	EVA1C	chr21:32412006	Wald ratio	1	-0.192	0.163	2.392E-01	0.948	cis	Hospitalized COVID-19
4924_32	MMP-1	Interstitial collagenase	P03956	MMP1	chr11:102798160	Inverse variance weighted	9	0.032	0.027	2.396E-01	0.948	cis	Hospitalized COVID-19
9719_145	MMP-16	Matrix metalloproteinase-16	P51512	MMP16	chr8:88328025	Wald ratio	1	-0.219	0.186	2.406E-01	0.948	cis	Hospitalized COVID-19
11547_84	MUSK	Muscle, skeletal receptor tyrosine-protein kinase	O15146	MUSK	chr9:110668779	Wald ratio	1	0.214	0.182	2.407E-01	0.948	cis	Hospitalized COVID-19
6927_7	NDST1	Bifunctional heparan sulfate N-deacetylase/N-sulfotransferase 1	P52848	NDST1	chr5:150485818	Wald ratio	1	0.085	0.073	2.413E-01	0.948	cis	Hospitalized COVID-19
8269_327	ARSK	Arylsulfatase K	Q6UWY0	ARSK	chr5:95555101	Wald ratio	1	0.153	0.130	2.420E-01	0.948	cis	Hospitalized COVID-19
7185_29	GPV	Platelet glycoprotein V	P40197	GP5	chr3:194399266	Inverse variance weighted	2	-0.121	0.104	2.424E-01	0.948	cis	Hospitalized COVID-19

13460_4	CHAD	Chondroadherin	O15335	CHAD	chr17:50468906	Wald ratio	1	-0.235	0.201	2.428E-01	0.948	cis	Hospitalized COVID-19
14684_17	CAN2	Calpain-2 catalytic subunit	P17655	CAPN2	chr1:223701593	Inverse variance weighted	3	-0.081	0.070	2.436E-01	0.948	cis	Hospitalized COVID-19
3004_67	PD-L2	Programmed cell death 1 ligand 2	Q9BQ51	PDCD1LG2	chr9:5510531	Inverse variance weighted	5	-0.038	0.033	2.444E-01	0.948	cis	Hospitalized COVID-19
7145_1	ITIH3	Inter-alpha-trypsin inhibitor heavy chain H3	Q06033	ITIH3	chr3:52794768	Inverse variance weighted	10	0.034	0.029	2.444E-01	0.948	cis	Hospitalized COVID-19
17153_46	KI2L3	Killer cell immunoglobulin-like receptor 2DL3	P43628	KIR2DL3	chr19:54738513	Inverse variance weighted	4	-0.031	0.026	2.444E-01	0.948	cis	Hospitalized COVID-19
13472_35	HDHD2	Haloacid dehalogenase-like hydrolase domain-containing protein 2	Q9H0R4	HDHD2	chr18:47150500	Inverse variance weighted	3	0.056	0.048	2.445E-01	0.948	cis	Hospitalized COVID-19
4430_44	Collectin Kidney 1	Collectin-11	Q9BWP8	COLEC11	chr2:3594832	Inverse variance weighted	10	0.022	0.019	2.462E-01	0.948	cis	Hospitalized COVID-19
14208_3	RET7	Retinoid-binding protein 7	Q96R05	RBP7	chr1:9997206	Inverse variance weighted	2	-0.103	0.089	2.467E-01	0.948	cis	Hospitalized COVID-19
5749_53	COL	Colipase	P04118	CLPS	chr6:35797344	Inverse variance weighted	10	-0.031	0.027	2.487E-01	0.948	cis	Hospitalized COVID-19
15387_44	Neuropilin-2	Neuropilin-2	O60462	NRP2	chr2:205681990	Inverse variance weighted	4	0.061	0.053	2.500E-01	0.948	cis	Hospitalized COVID-19
13748_4	MCP-2	C-C motif chemokine 8	P80075	CCL8	chr17:34319435	Inverse variance weighted	7	0.038	0.033	2.503E-01	0.948	cis	Hospitalized COVID-19
17327_3	CNPY3	Protein canopy homolog 3	Q9BT09	CNPY3	chr6:42929480	Wald ratio	1	-0.209	0.182	2.511E-01	0.948	cis	Hospitalized COVID-19
9265_10	GLIP1	Glioma pathogenesis-related protein 1	P48060	GLIPR1	chr12:75480753	Wald ratio	1	-0.131	0.114	2.514E-01	0.948	cis	Hospitalized COVID-19
15494_11	FGFP1	Fibroblast growth factor-binding protein 1	Q14512	FGFBP1	chr4:15938740	Wald ratio	1	-0.182	0.158	2.517E-01	0.948	cis	Hospitalized COVID-19
7769_29	3BP2	SH3 domain-binding protein 2	P78314	SH3BP2	chr4:2793071	Inverse variance weighted	3	0.076	0.067	2.527E-01	0.948	cis	Hospitalized COVID-19
16770_3	REG1B	Lithostathine-1-beta	P48304	REG1B	chr2:79088019	Inverse variance weighted	3	0.243	0.215	2.569E-01	0.948	cis	Hospitalized COVID-19
7163_26	lacritin	Extracellular glycoprotein lacritin	Q9GZZ8	LACRT	chr12:54634895	Wald ratio	1	-0.247	0.219	2.577E-01	0.948	cis	Hospitalized COVID-19
15589_1	Gc-Globulin, Mixed Type	Vitamin D-binding protein	P02774	GC	chr4:71804041	Inverse variance weighted	2	0.087	0.077	2.577E-01	0.948	cis	Hospitalized COVID-19
11117_2	SPT20	Spermatogenesis-associated protein 20	Q8TB22	SPATA20	chr17:50543058	Inverse variance weighted	4	-0.044	0.039	2.585E-01	0.948	cis	Hospitalized COVID-19
7808_5	GLCE	D-glucuronyl C5-epimerase	O94923	GLCE	chr15:69160584	Inverse variance weighted	7	-0.028	0.025	2.586E-01	0.948	cis	Hospitalized COVID-19
8403_18	Fatty acid synthase	Fatty acid synthase	P49327	FASN	chr17:82098294	Wald ratio	1	0.082	0.073	2.592E-01	0.948	cis	Hospitalized COVID-19
11152_46	kallikrein 13	Kallikrein-13	Q9UKR3	KLK13	chr19:51065114	Wald ratio	1	-0.144	0.128	2.604E-01	0.948	cis	Hospitalized COVID-19
2849_49	AIF1	Allograft inflammatory factor 1	P55008	AIF1	chr6:31615217	Inverse variance weighted	2	0.195	0.174	2.611E-01	0.948	cis	Hospitalized COVID-19
6558_5	COL10	Collectin-10	Q9Y6Z7	COLEC10	chr8:118995452	Wald ratio	1	0.208	0.185	2.611E-01	0.948	cis	Hospitalized COVID-19
8606_39	GPNMB:CD	Transmembrane glycoprotein NMB:Cytoplasmic domain	Q14956	GPNMB	chr7:23235967	Inverse variance weighted	7	0.037	0.033	2.611E-01	0.948	cis	Hospitalized COVID-19
2992_59	IL-17 sR	Interleukin-17 receptor A	Q96F46	IL17RA	chr22:17084954	Inverse variance weighted	7	-0.014	0.012	2.618E-01	0.948	cis	Hospitalized COVID-19
15336_7	SELM	Selenoprotein M	Q8WWX9	SELENOM	chr22:31120069	Wald ratio	1	0.220	0.197	2.625E-01	0.948	cis	Hospitalized COVID-19
7251_64	C1QT3	Complement C1q tumor necrosis factor-related protein 3	Q9BXJ4	C1QTNF3	chr5:34043213	Inverse variance weighted	2	-0.083	0.074	2.632E-01	0.948	cis	Hospitalized COVID-19
8233_2	ITIH5	Inter-alpha-trypsin inhibitor heavy chain H5	Q86UX2	ITIH5	chr10:7666998	Inverse variance weighted	7	-0.043	0.038	2.635E-01	0.948	cis	Hospitalized COVID-19
9288_7	FKBP7	Peptidyl-prolyl cis-trans isomerase FKBP7	Q9Y680	FKBP7	chr2:178478600	Inverse variance weighted	4	0.038	0.034	2.638E-01	0.948	cis	Hospitalized COVID-19
6367_66	fibromodulin	fibromodulin	Q06828	FMOD	chr1:203351758	Inverse variance weighted	2	0.064	0.058	2.640E-01	0.948	cis	Hospitalized COVID-19
16785_45	HD-5	Defensin-5	Q01523	DEFA5	chr8:7056739	Wald ratio	1	-0.178	0.159	2.640E-01	0.948	cis	Hospitalized COVID-19
12378_71	TPSN	Tapasin	O15533	TAPBP	chr6:33314284	Inverse variance weighted	4	0.020	0.018	2.642E-01	0.948	cis	Hospitalized COVID-19
7866_11	DJC30	DnaJ homolog subfamily C member 30	Q96LL9	DNAJC30	chr7:73683453	Inverse variance weighted	2	-0.092	0.082	2.645E-01	0.948	cis	Hospitalized COVID-19
17761_2	NUDT5	ADP-sugar pyrophosphatase	Q9UKK9	NUDT5	chr10:12196144	Wald ratio	1	-0.149	0.134	2.647E-01	0.948	cis	Hospitalized COVID-19
9256_78	NPTX1	Neuronal pentraxin-1	Q15818	NPTX1	chr17:80477843	Inverse variance weighted	5	-0.048	0.043	2.653E-01	0.948	cis	Hospitalized COVID-19
19262_219	ACADV	Very long-chain specific acyl-CoA dehydrogenase, mitochondrial	P49748	ACADVL	chr17:7217125	Wald ratio	1	-0.224	0.201	2.664E-01	0.948	cis	Hospitalized COVID-19
2743_5	Sonic Hedgehog	Sonic hedgehog protein	Q15465	SHH	chr7:155812463	Inverse variance weighted	2	-0.134	0.120	2.667E-01	0.948	cis	Hospitalized COVID-19
11302_237	TENR	Tenascin-R	Q92752	TNR	chr1:175743616	Wald ratio	1	0.089	0.080	2.672E-01	0.948	cis	Hospitalized COVID-19
16070_7	WIF-1	Wnt inhibitory factor 1	Q9Y5W5	WIF1	chr12:65121305	Wald ratio	1	-0.191	0.172	2.678E-01	0.948	cis	Hospitalized COVID-19
9557_5	MANS1	MANSC domain-containing protein 1	Q9H8J5	MANSC1	chr12:12350242	Inverse variance weighted	3	0.068	0.061	2.681E-01	0.948	cis	Hospitalized COVID-19
7059_14	LIRA6	Leukocyte immunoglobulin-like receptor subfamily A member 6	Q6PI73	LILRA6	chr19:54242791	Inverse variance weighted	8	0.056	0.050	2.685E-01	0.948	cis	Hospitalized COVID-19
12686_15	THTM	3-mercaptopyruvate sulfurtransferase	P25325	MPST	chr22:37019635	Inverse variance weighted	2	-0.120	0.109	2.696E-01	0.948	cis	Hospitalized COVID-19
19606_28	ihh	Indian hedgehog protein	Q14623	IHH	chr2:219060921	Inverse variance weighted	3	-0.175	0.159	2.702E-01	0.948	cis	Hospitalized COVID-19
9077_10	MA1A2	Mannosyl-oligosaccharide 1,2-alpha-mannosidase IB	O60476	MAN1A2	chr1:117367449	Inverse variance weighted	4	-0.055	0.050	2.703E-01	0.948	cis	Hospitalized COVID-19
7950_142	BTNL9	Butyrophilin-like protein 9	Q6UXG8	BTNL9	chr5:181040225	Wald ratio	1	0.167	0.151	2.704E-01	0.948	cis	Hospitalized COVID-19
13700_10	annexin II	Annexin A2	P07355	ANXA2	chr15:60402883	Inverse variance weighted	6	-0.083	0.076	2.729E-01	0.948	cis	Hospitalized COVID-19
9266_1	sTREM-1	Triggering receptor expressed on myeloid cells 1	Q9NP99	TREM1	chr6:41286682	Inverse variance weighted	6	0.074	0.067	2.729E-01	0.948	cis	Hospitalized COVID-19
8397_147	QSOX2	Sulfhydryl oxidase 2	Q6ZRP7	QSOX2	chr9:136245812	Inverse variance weighted	7	0.026	0.024	2.731E-01	0.948	cis	Hospitalized COVID-19
4991_12	GPC5	Glypican-5	P78333	GPC5	chr13:91398621	Inverse variance weighted	6	-0.028	0.026	2.743E-01	0.948	cis	Hospitalized COVID-19
9747_48	MARK3	MAP/microtubule affinity-regulating kinase 3	P27448	MARK3	chr14:103385377	Wald ratio	1	0.292	0.268	2.752E-01	0.948	cis	Hospitalized COVID-19
5542_22	NRP1	Neuropilin-1	O14786	NRP1	chr10:33336262	Inverse variance weighted	5	-0.033	0.030	2.767E-01	0.948	cis	Hospitalized COVID-19
14623_26	SUMO3	Small ubiquitin-related modifier 3	P55854	SUMO3	chr21:44818779	Wald ratio	1	-0.214	0.197	2.771E-01	0.948	cis	Hospitalized COVID-19
2973_15	CD36 ANTIGEN	Platelet glycoprotein 4	P16671	CD36	chr7:80369575	Inverse variance weighted	4	0.055	0.051	2.790E-01	0.948	cis	Hospitalized COVID-19
10938_13	sLFA-3	Lymphocyte function-associated antigen 3	P19256	CD58	chr1:116571039	Inverse variance weighted	2	-0.071	0.066	2.803E-01	0.948	cis	Hospitalized COVID-19
13947_371	NUD12	Peroxisomal NADH pyrophosphatase NUDT12	Q9BQG2	NUDT12	chr5:103562790	Inverse variance weighted	4	0.033	0.031	2.810E-01	0.948	cis	Hospitalized COVID-19
15333_11	SDF2	Stromal cell-derived factor 2	Q99470	SDF2	chr17:28662189	Wald ratio	1	0.141	0.131	2.812E-01	0.948	cis	Hospitalized COVID-19
5128_53	SLAF6	SLAM family member 6	Q96DU3	SLAMF6	chr1:160523262	Wald ratio	1	-0.062	0.057	2.814E-01	0.948	cis	Hospitalized COVID-19
13552_7	SWP70	Switch-associated protein 70	Q9UH65	SWAP70	chr11:9664077	Inverse variance weighted	5	-0.025	0.023	2.816E-01	0.948	cis	Hospitalized COVID-19
12424_107	THYN1	Thymocyte nuclear protein 1	Q9P016	THYN1	chr11:134253370	Wald ratio	1	0.222	0.207	2.819E-01	0.948	cis	Hospitalized COVID-19
8992_1	TMEM2	Transmembrane protein 2	Q9UHN6	CEMIP2	chr9:71816690	Inverse variance weighted	2	0.094	0.087	2.821E-01	0.948	cis	Hospitalized COVID-19
17333_20	ACADM	Medium-chain specific acyl-CoA dehydrogenase, mitochondrial	P11310	ACADM	chr1:75724431	Wald ratio	1	-0.055	0.051	2.822E-01	0.948	cis	Hospitalized COVID-19
6444_15	PSG3	Pregnancy-specific beta-1-glycoprotein 3	Q16557	PSG3	chr19:42740481	Inverse variance weighted	8	0.053	0.049	2.825E-01	0.948	cis	Hospitalized COVID-19
17515_6	STCH	Heat shock 70 kDa protein 13	P48723	HSPA13	chr21:14383484	Wald ratio	1	0.111	0.104	2.848E-01	0.948	cis	Hospitalized COVID-19
17836_17	S100A16	Protein S100-A16	Q96FQ6	S100A16	chr1:153613145	Wald ratio	1	-0.157	0.147	2.857E-01	0.948	cis	Hospitalized COVID-19
10080_9	EI2BA	Translation initiation factor eIF-2B subunit alpha	Q14232	EIF2B1	chr12:123633766	Wald ratio	1	0.193	0.181	2.864E-01	0.948	cis	Hospitalized COVID-19
3059_50	BAFF	Tumor necrosis factor ligand superfamily member 13B	Q9Y275	TNFSF13B	chr13:108251240	Inverse variance weighted	3	-0.164	0.154	2.864E-01	0.948	cis	Hospitalized COVID-19
11278_4	COL11A2	Collagen alpha-2(XI) chain	P13942	COL11A2	chr6:33192499	Inverse variance weighted	4	0.041	0.038	2.866E-01	0.948	cis	Hospitalized COVID-19
16803_4	CALB2	Calretinin	P22676	CALB2	chr16:71358713	Inverse variance weighted	2	-0.170	0.159	2.867E-01	0.948	cis	Hospitalized COVID-19
7655_11	N-terminal pro-BNP	N-terminal pro-BNP	P16860	NPPB	chr1:11858945	Inverse variance weighted	3	0.056	0.052	2.874E-01	0.948	cis	Hospitalized COVID-19
15483_377	Agrin	Agrin	O00468	AGRN	chr1:1020120	Inverse variance weighted	6	-0.072	0.068	2.875E-01	0.948	cis	Hospitalized COVID-19
12347_29	CCM2	Cerebral cavernous malformations 2 protein	Q9BSQ5	CCM2	chr7:44999475	Wald ratio	1	-0.177	0.166	2.875E-01	0.948	cis	Hospitalized COVID-19
19488_1	PDCD6	Programmed cell death protein 6	O75340	PDCD6	chr5:271621	Inverse variance weighted	3	0.074	0.069	2.875E-01	0.948	cis	Hospitalized COVID-19
3364_76	Cathepsin V	Cathepsin L2	O60911	CTSV	chr9:97156556	Inverse variance weighted	4	-0.056	0.053	2.883E-01	0.948	cis	Hospitalized COVID-19
14094_29	HB-EGF	Heparin-binding EGF-like growth factor	Q99075	HBEGF	chr5:140346603	Wald ratio	1	0.226	0.213	2.894E-01	0.948	cis	Hospitalized COVID-19
6373_54	DLK1	Protein delta homolog 1	P80370	DLK1	chr14:100725705	Inverse variance weighted	2	-0.147	0.139	2.903E-01	0.948	cis	Hospitalized COVID-19
15653_9	COAA1	Collagen alpha-1(X) chain	Q03692	COL10A1	chr6:116158747	Inverse variance weighted	3	-0.076	0.072	2.907E-01	0.948	cis	Hospitalized COVID-19
13113_7	Osteopontin	Osteopontin	P10451	SPP1	chr4:87975667	Inverse variance weighted	2	0.063	0.060	2.914E-01	0.948	cis	Hospitalized COVID-19

16851_50	SCO2	Protein SCO2 homolog, mitochondrial	O43819	SCO2	chr22:50526461	Wald ratio	1	-0.113	0.107	2.933E-01	0.948	cis	Hospitalized COVID-19
15516_12	SAA-4	Serum amyloid A-4 protein	P35542	SAA4	chr11:18236802	Inverse variance weighted	7	0.017	0.016	2.939E-01	0.948	cis	Hospitalized COVID-19
11681_8	NUPL	Arf-GAP domain and FG repeat-containing protein 1	P52594	AGFG1	chr2:227472152	Wald ratio	1	-0.139	0.133	2.940E-01	0.948	cis	Hospitalized COVID-19
8786_6	F171B:ECD	Protein FAM171B:Extracellular domain	Q6P995	FAM171B	chr2:186694060	Wald ratio	1	0.069	0.066	2.941E-01	0.948	cis	Hospitalized COVID-19
14747_9	CRLF1	Cytokine receptor-like factor 1	O75462	CRLF1	chr19:18607741	Inverse variance weighted	2	-0.048	0.045	2.942E-01	0.948	cis	Hospitalized COVID-19
13090_17	S100A6	Protein S100-A6	P06703	S100A6	chr1:153536244	Inverse variance weighted	2	0.178	0.170	2.949E-01	0.948	cis	Hospitalized COVID-19
4355_13	DAPK2	Death-associated protein kinase 2	Q9UIK4	DAPK2	chr15:64072033	Inverse variance weighted	3	0.037	0.035	2.958E-01	0.948	cis	Hospitalized COVID-19
13126_52	DSC2	Desmocollin-2	Q02487	DSC2	chr18:31102522	Inverse variance weighted	4	-0.073	0.070	2.965E-01	0.948	cis	Hospitalized COVID-19
12637_7	CACP	Carnitine O-acetyltransferase	P43155	CRAT	chr9:129111189	Wald ratio	1	0.119	0.114	2.970E-01	0.948	cis	Hospitalized COVID-19
16915_153	SEM4A	Semaphorin-4A	Q9H3S1	SEMA4A	chr1:156147366	Wald ratio	1	0.062	0.059	2.983E-01	0.948	cis	Hospitalized COVID-19
12685_57	HOME2	Homer protein homolog 2	Q9NSB8	HOMER2	chr15:82986153	Wald ratio	1	0.154	0.148	2.985E-01	0.948	cis	Hospitalized COVID-19
8989_40	SCUB1	Signal peptide, CUB and EGF-like domain-containing protein 1	Q8IWY4	SCUBE1	chr22:43343372	Inverse variance weighted	4	-0.087	0.084	2.989E-01	0.948	cis	Hospitalized COVID-19
12646_2	RPE	Ribulose-phosphate 3-epimerase	Q96AT9	RPE	chr2:210002565	Wald ratio	1	0.132	0.127	2.994E-01	0.948	cis	Hospitalized COVID-19
2837_3	Met	Hepatocyte growth factor receptor	P08581	MET	chr7:116672196	Inverse variance weighted	4	0.081	0.078	3.001E-01	0.948	cis	Hospitalized COVID-19
13719_19	PAK4	Serine/threonine-protein kinase PAK 4	O96013	PAK4	chr19:39125770	Wald ratio	1	0.221	0.214	3.027E-01	0.948	cis	Hospitalized COVID-19
12411_60	MAX	Protein max	P61244	MAX	chr14:65102695	Wald ratio	1	0.059	0.057	3.031E-01	0.948	cis	Hospitalized COVID-19
5462_62	Ficolin-3	Ficolin-3	O75636	FCN3	chr1:27374824	Inverse variance weighted	7	-0.026	0.025	3.032E-01	0.948	cis	Hospitalized COVID-19
10612_18	PLOD3	Procollagen-lysine,2-oxoglutarate 5-dioxygenase 3	O60568	PLOD3	chr7:101218420	Inverse variance weighted	3	0.100	0.097	3.032E-01	0.948	cis	Hospitalized COVID-19
3172_28	ARSB	Arylsulfatase B	P15848	ARSB	chr5:78986087	Inverse variance weighted	2	0.096	0.094	3.040E-01	0.948	cis	Hospitalized COVID-19
5012_67	Myokinase, human	Adenylate kinase isoenzyme 1	P00568	AK1	chr9:127877675	Wald ratio	1	-0.248	0.242	3.046E-01	0.948	cis	Hospitalized COVID-19
5671_1	Chymotrypsin	Chymotrypsinogen B	P17538	CTRB1	chr16:75218988	Inverse variance weighted	5	-0.079	0.077	3.052E-01	0.948	cis	Hospitalized COVID-19
15417_3	Maspin	Serpin B5	P36952	SERPINB5	chr18:63476958	Wald ratio	1	-0.083	0.081	3.063E-01	0.948	cis	Hospitalized COVID-19
6627_25	LIPR1	Inactive pancreatic lipase-related protein 1	P54315	PNLIPRP1	chr10:116590385	Inverse variance weighted	2	0.069	0.068	3.069E-01	0.948	cis	Hospitalized COVID-19
6414_8	OAF	Out at first protein homolog	Q86UD1	OAF	chr11:120211032	Inverse variance weighted	5	-0.022	0.022	3.082E-01	0.948	cis	Hospitalized COVID-19
9769_48	DNER:ECD	Delta and Notch-like epidermal growth factor-related receptor:Extracellular domain	Q8NFT8	DNER	chr2:229714555	Inverse variance weighted	5	0.060	0.059	3.091E-01	0.948	cis	Hospitalized COVID-19
7192_37	CRF2-12	Interferon lambda receptor 1	Q8IU57	IFNLR1	chr1:24187959	Inverse variance weighted	3	-0.075	0.073	3.092E-01	0.948	cis	Hospitalized COVID-19
2681_23	HGF	Hepatocyte growth factor	P14210	HGF	chr7:81770438	Wald ratio	1	-0.111	0.109	3.095E-01	0.948	cis	Hospitalized COVID-19
12703_6	NEK7	Serine/threonine-protein kinase Nek7	Q8TDX7	NEK7	chr1:198156994	Wald ratio	1	0.130	0.128	3.106E-01	0.948	cis	Hospitalized COVID-19
4192_10	AK1A1	Alcohol dehydrogenase [NADP(+)]	P14550	AKR1A1	chr1:45550543	Inverse variance weighted	6	0.027	0.027	3.106E-01	0.948	cis	Hospitalized COVID-19
3858_5	PPAC	Low molecular weight phosphotyrosine protein phosphatase	P24666	ACP1	chr2:264140	Inverse variance weighted	7	-0.020	0.020	3.112E-01	0.948	cis	Hospitalized COVID-19
17783_9	MMAB	Cob(I)yrinic acid a,c-diamide adenosyltransferase, mitochondrial	Q96EY8	MMAB	chr12:109573580	Inverse variance weighted	6	0.031	0.031	3.113E-01	0.948	cis	Hospitalized COVID-19
15480_2	VNN2	Vascular non-inflammatory molecule 2	O95498	VNN2	chr6:132763459	Inverse variance weighted	11	0.017	0.017	3.115E-01	0.948	cis	Hospitalized COVID-19
6462_12	TIMP-4	Metalloproteinase inhibitor 4	Q99727	TIMP4	chr3:12158912	Inverse variance weighted	8	-0.047	0.046	3.118E-01	0.948	cis	Hospitalized COVID-19
18880_81	Collagen Type III	Collagen Type III	P02461	COL3A1	chr2:188974373	Inverse variance weighted	2	-0.162	0.160	3.129E-01	0.948	cis	Hospitalized COVID-19
15391_114	GAS-6	Growth arrest-specific protein 6	Q14393	GAS6	chr13:113864076	Inverse variance weighted	4	-0.038	0.037	3.129E-01	0.948	cis	Hospitalized COVID-19
7206_20	F16P1	Fructose-1,6-bisphosphatase 1	P09467	FBP1	chr9:94640249	Inverse variance weighted	2	0.163	0.162	3.134E-01	0.948	cis	Hospitalized COVID-19
3376_49	IL-17 RD	Interleukin-17 receptor D	Q8NFM7	IL17RD	chr3:57170306	Inverse variance weighted	4	0.027	0.027	3.136E-01	0.948	cis	Hospitalized COVID-19
8235_48	SCG1	Secretogranin-1	P05060	CHGB	chr20:5911510	Wald ratio	1	0.138	0.137	3.139E-01	0.948	cis	Hospitalized COVID-19
5392_73	Fas, soluble	Tumor necrosis factor receptor superfamily member 6	P25445	FAS	chr10:88953813	Inverse variance weighted	2	0.101	0.100	3.146E-01	0.948	cis	Hospitalized COVID-19
17776_15	PECR	Peroxisomal trans-2-enoyl-CoA reductase	Q9BY49	PECR	chr2:216082955	Wald ratio	1	0.108	0.108	3.146E-01	0.948	cis	Hospitalized COVID-19
17794_6	Phosphomannomutase 2	Phosphomannomutase 2	O15305	PMM2	chr16:8788823	Inverse variance weighted	4	0.099	0.099	3.148E-01	0.948	cis	Hospitalized COVID-19
12370_30	Apo F	Apolipoprotein F	Q13790	APOF	chr12:56362857	Inverse variance weighted	2	-0.071	0.070	3.153E-01	0.948	cis	Hospitalized COVID-19
9449_150	C4b-binding protein alpha chain	C4b-binding protein alpha chain	P04003	C4BPA	chr1:207104233	Inverse variance weighted	6	-0.057	0.057	3.160E-01	0.948	cis	Hospitalized COVID-19
13423_94	CJ058	Redox-regulatory protein FAM213A	Q9BRX8	PRXL2A	chr10:80407829	Wald ratio	1	0.211	0.210	3.164E-01	0.948	cis	Hospitalized COVID-19
18342_2	SERC	Phosphoserine aminotransferase	Q9Y617	PSAT1	chr9:78297125	Inverse variance weighted	2	-0.116	0.116	3.166E-01	0.948	cis	Hospitalized COVID-19
18831_6	LRIG1	Leucine-rich repeats and immunoglobulin-like domains protein 1	Q96JA1	LRIG1	chr3:66501263	Inverse variance weighted	8	-0.033	0.033	3.170E-01	0.948	cis	Hospitalized COVID-19
15636_49	SORC1	VPS10 domain-containing receptor SorCS1	Q8WY21	SORCS1	chr10:107164706	Wald ratio	1	-0.159	0.159	3.173E-01	0.948	cis	Hospitalized COVID-19
15367_38	LPLC1	BPI fold-containing family B member 1	Q8TDL5	BPIFB1	chr20:33273480	Inverse variance weighted	7	-0.026	0.026	3.173E-01	0.948	cis	Hospitalized COVID-19
10620_21	PSP-94	Beta-microseminoprotein	P08118	MSMB	chr10:46048180	Inverse variance weighted	3	-0.012	0.012	3.197E-01	0.948	cis	Hospitalized COVID-19
3029_52	DC-SIGN	CD209 antigen	Q9NNX6	CD209	chr19:7747564	Inverse variance weighted	4	0.041	0.042	3.206E-01	0.948	cis	Hospitalized COVID-19
4930_21	Stanniocalcin-1	Stanniocalcin-1	P52823	STC1	chr8:23854806	Wald ratio	1	-0.119	0.120	3.217E-01	0.948	cis	Hospitalized COVID-19
15596_7	HEXI1	Protein HEXIM1	O94992	HEXIM1	chr17:45148475	Wald ratio	1	0.194	0.196	3.220E-01	0.948	cis	Hospitalized COVID-19
13133_73	LTBP4	Latent-transforming growth factor beta-binding protein 4	Q8N2S1	LTBP4	chr19:40592883	Inverse variance weighted	2	-0.161	0.162	3.222E-01	0.948	cis	Hospitalized COVID-19
13416_8	T132D	Transmembrane protein 132D	Q14C87	TMEM132D	chr12:129904025	Inverse variance weighted	6	0.033	0.034	3.225E-01	0.948	cis	Hospitalized COVID-19
17357_33	SPCS	O-phosphoseryl-tRNA(Sec) selenium transferase	Q9HD40	SEPSECS	chr4:25160550	Wald ratio	1	-0.145	0.147	3.228E-01	0.948	cis	Hospitalized COVID-19
4455_89	MFGM	Lactadherin	Q08431	MFGE8	chr15:88913468	Inverse variance weighted	4	-0.041	0.041	3.245E-01	0.948	cis	Hospitalized COVID-19
4867_15	VEGF121	Vascular endothelial growth factor A, isoform 121	P15692	VEGFA	chr6:43770184	Inverse variance weighted	4	0.102	0.104	3.259E-01	0.948	cis	Hospitalized COVID-19
12697_30	PI42A	Phosphatidylinositol 5-phosphate 4-kinase type-2 alpha	P48426	PIP4K2A	chr10:22714578	Wald ratio	1	0.096	0.098	3.259E-01	0.948	cis	Hospitalized COVID-19
4763_31	Afamin	Afamin	P43652	AFM	chr4:73481745	Inverse variance weighted	3	0.039	0.040	3.260E-01	0.948	cis	Hospitalized COVID-19
3046_31	resistin	Resistin	Q9HD89	RETN	chr19:7669049	Inverse variance weighted	6	0.043	0.044	3.271E-01	0.948	cis	Hospitalized COVID-19
18174_79	PDC6I	Programmed cell death 6-interacting protein	Q8WUM4	PDCD6IP	chr3:33798571	Wald ratio	1	-0.094	0.096	3.276E-01	0.948	cis	Hospitalized COVID-19
13998_26	PURA1	Adenylosuccinate synthetase isozyme 1	Q8N142	ADSS1	chr14:104724229	Inverse variance weighted	3	0.064	0.066	3.279E-01	0.948	cis	Hospitalized COVID-19
12689_56	ARC1B	Actin-related protein 2/3 complex subunit 1B	O15143	ARPC1B	chr7:99374249	Wald ratio	1	0.115	0.118	3.292E-01	0.948	cis	Hospitalized COVID-19
5604_30	HPSE	Heparanase	Q9Y251	HPSE	chr4:83335153	Inverse variance weighted	2	-0.069	0.070	3.293E-01	0.948	cis	Hospitalized COVID-19
18839_24	Thyroglobulin	Thyroglobulin	P01266	TG	chr8:132866958	Wald ratio	1	-0.123	0.126	3.293E-01	0.948	cis	Hospitalized COVID-19
4127_75	C6	Complement component C6	P13671	C6	chr5:41261438	Inverse variance weighted	4	-0.067	0.069	3.308E-01	0.948	cis	Hospitalized COVID-19
6574_11	FAIM3	Fas apoptotic inhibitory molecule 3	O60667	FCMR	chr1:206923247	Inverse variance weighted	2	-0.146	0.151	3.312E-01	0.948	cis	Hospitalized COVID-19
8982_65	TSP3	Thrombospondin-3	P49746	THBS3	chr1:155209051	Inverse variance weighted	3	-0.191	0.197	3.315E-01	0.948	cis	Hospitalized COVID-19
5451_1	ALCAM	CD166 antigen	Q13740	ALCAM	chr3:105366909	Inverse variance weighted	4	0.080	0.082	3.342E-01	0.948	cis	Hospitalized COVID-19
3344_60	Antithrombin III	Antithrombin-III	P01008	SERPINC1	chr1:173917327	Wald ratio	1	0.167	0.173	3.343E-01	0.948	cis	Hospitalized COVID-19
15472_16	LRP11	Low-density lipoprotein receptor-related protein 11	Q86VZ4	LRP11	chr6:149864359	Inverse variance weighted	6	-0.036	0.037	3.343E-01	0.948	cis	Hospitalized COVID-19
17383_4	STAR5	StAR-related lipid transfer protein 5	Q9NSY2	STARD5	chr15:81324183	Inverse variance weighted	6	0.059	0.061	3.364E-01	0.948	cis	Hospitalized COVID-19
5459_33	CYTN	Cystatin-SN	P01037	CST1	chr20:23751268	Inverse variance weighted	5	0.073	0.077	3.384E-01	0.948	cis	Hospitalized COVID-19
3220_40	RET	Proto-oncogene tyrosine-protein kinase receptor Ret	P07949	RET	chr10:43077064	Inverse variance weighted	4	0.032	0.033	3.399E-01	0.948	cis	Hospitalized COVID-19
9369_174	NGL1	Leucine-rich repeat-containing protein 4C	Q9HCJ2	LRRC4C	chr11:41459773	Inverse variance weighted	3	0.096	0.101	3.407E-01	0.948	cis	Hospitalized COVID-19
15544_25	kallikrein 14	Kallikrein-14	Q9P0G3	KLK14	chr19:51084245	Inverse variance weighted	2	-0.150	0.157	3.411E-01	0.948	cis	Hospitalized COVID-19
11646_4	Carbohydrate sulfotransferase 9	Carbohydrate sulfotransferase 9	Q7L1S5	CHST9	chr18:27185308	Inverse variance weighted	4	-0.034	0.036	3.414E-01	0.948	cis	Hospitalized COVID-19
15614_168	LIRA2	Leukocyte immunoglobulin-like receptor subfamily A member 2	Q8N149	LILRA2	chr19:54572920	Inverse variance weighted	4	-0.034	0.036	3.421E-01	0.948	cis	Hospitalized COVID-19

16323_8	NRX3A	Neurexin-3	Q9Y4C0	NRXN3	chr14:78170373	Wald ratio	1	0.201	0.211	3.425E-01	0.948	cis	Hospitalized COVID-19
6455_52	HPLN4	Hyaluronan and proteoglycan link protein 4	Q86UW8	HAPLN4	chr19:19262804	Wald ratio	1	0.136	0.144	3.427E-01	0.948	cis	Hospitalized COVID-19
3367_8	FETUB	Fetuin-B	Q9UGM5	FETUB	chr3:186635969	Inverse variance weighted	5	0.053	0.056	3.429E-01	0.948	cis	Hospitalized COVID-19
13969_24	IMA7	Importin subunit alpha-7	O60684	KPNA6	chr1:32108056	Wald ratio	1	0.166	0.175	3.434E-01	0.948	cis	Hospitalized COVID-19
13973_62	TTL	Tubulin--tyrosine ligase	Q8NG68	TTL	chr2:112482156	Wald ratio	1	0.173	0.183	3.441E-01	0.948	cis	Hospitalized COVID-19
12408_333	RB22A	Ras-related protein Rab-22A	Q9UL26	RAB22A	chr20:58309715	Wald ratio	1	0.175	0.185	3.444E-01	0.948	cis	Hospitalized COVID-19
5131_15	TAJ	Tumor necrosis factor receptor superfamily member 19	Q9NS68	TNFRSF19	chr13:23570370	Wald ratio	1	-0.101	0.106	3.446E-01	0.948	cis	Hospitalized COVID-19
19194_9	Histidyl-tRNA synthetase-related	D-tyrosyl-tRNA(Tyr) deacylase 1	Q8TEA8	DTD1	chr20:18587942	Wald ratio	1	0.049	0.052	3.455E-01	0.948	cis	Hospitalized COVID-19
17692_2	BT3A3	Butyrophilin subfamily 3 member A3	O00478	BTN3A3	chr6:26440472	Inverse variance weighted	9	-0.020	0.022	3.458E-01	0.948	cis	Hospitalized COVID-19
14688_6	PTN7	Tyrosine-protein phosphatase non-receptor type 7	P35236	PTPN7	chr1:202161588	Wald ratio	1	-0.218	0.232	3.467E-01	0.948	cis	Hospitalized COVID-19
13986_6	LANC1	LanC-like protein 1	O43813	LANCL1	chr2:210477652	Inverse variance weighted	2	-0.105	0.112	3.468E-01	0.948	cis	Hospitalized COVID-19
4989_7	Fibrinogen g-chain dimer	Fibrinogen gamma chain	P02679	FGG	chr4:154612967	Wald ratio	1	-0.066	0.070	3.468E-01	0.948	cis	Hospitalized COVID-19
14107_1	MTHFS	5-formyltetrahydrofolate cyclo-ligase	P49914	MTHFS	chr15:79897379	Inverse variance weighted	6	0.028	0.030	3.473E-01	0.948	cis	Hospitalized COVID-19
17786_5	GGPPS	Geranylgeranyl pyrophosphate synthase	O95749	GGPS1	chr1:235327350	Wald ratio	1	-0.107	0.114	3.479E-01	0.948	cis	Hospitalized COVID-19
10892_8	OSMR	Oncostatin-M-specific receptor subunit beta	Q99650	OSMR	chr5:38845858	Inverse variance weighted	2	0.127	0.135	3.483E-01	0.948	cis	Hospitalized COVID-19
17396_23	ADH1A	Alcohol dehydrogenase 1A	P07327	ADH1A	chr4:99291003	Wald ratio	1	-0.092	0.098	3.485E-01	0.948	cis	Hospitalized COVID-19
15470_11	Hexosaminidase B	Beta-hexosaminidase subunit beta	P07686	HEXB	chr5:74640023	Inverse variance weighted	4	0.049	0.053	3.492E-01	0.948	cis	Hospitalized COVID-19
3066_12	Galectin-3	Galectin-3	P17931	LGALS3	chr14:55124110	Inverse variance weighted	4	-0.029	0.031	3.492E-01	0.948	cis	Hospitalized COVID-19
4906_35	Coagulation Factor V	Coagulation Factor V	P12259	F5	chr1:169586588	Inverse variance weighted	5	0.031	0.034	3.496E-01	0.948	cis	Hospitalized COVID-19
17156_72	DCAK1	Serine/threonine-protein kinase DCLK1	O15075	DCLK1	chr13:36131382	Inverse variance weighted	3	0.046	0.049	3.499E-01	0.948	cis	Hospitalized COVID-19
19251_56	Platelet proteoglycan	Serglycin	P10124	SRGN	chr10:69088103	Inverse variance weighted	5	-0.037	0.040	3.507E-01	0.948	cis	Hospitalized COVID-19
8894_80	hnRNP A/B	Heterogeneous nuclear ribonucleoprotein A/B	Q99729	HNRNPAB	chr5:178204533	Wald ratio	1	-0.136	0.145	3.507E-01	0.948	cis	Hospitalized COVID-19
18895_54	GSTM4	Glutathione S-transferase Mu 4	Q03013	GSTM4	chr1:109656099	Inverse variance weighted	7	0.059	0.064	3.513E-01	0.948	cis	Hospitalized COVID-19
5349_69	DLL1	Delta-like protein 1	O00548	DLL1	chr6:170306565	Inverse variance weighted	2	0.081	0.087	3.519E-01	0.948	cis	Hospitalized COVID-19
9715_15	IGSF3	Immunoglobulin superfamily member 3	O75054	IGSF3	chr1:116667755	Inverse variance weighted	3	0.090	0.097	3.519E-01	0.948	cis	Hospitalized COVID-19
4154_57	P-Selectin	P-selectin	P16109	SELP	chr1:169630193	Inverse variance weighted	3	-0.065	0.070	3.529E-01	0.948	cis	Hospitalized COVID-19
11102_22	REG4	Regenerating islet-derived protein 4	Q9BYZ8	REG4	chr1:119811580	Inverse variance weighted	5	-0.058	0.063	3.530E-01	0.948	cis	Hospitalized COVID-19
17808_37	NIT2	Omega-amidase NIT2	Q9NQR4	NIT2	chr3:100334739	Inverse variance weighted	2	0.125	0.135	3.533E-01	0.948	cis	Hospitalized COVID-19
15539_15	SLIK1	SLIT and NTRK-like protein 1	Q96PX8	SLITRK1	chr13:83882474	Wald ratio	1	-0.142	0.153	3.536E-01	0.948	cis	Hospitalized COVID-19
15491_20	CD248	Endosialin	Q9HCU0	CD248	chr11:66317044	Inverse variance weighted	2	0.391	0.423	3.550E-01	0.948	cis	Hospitalized COVID-19
7968_15	CRTAM	Cytotoxic and regulatory T-cell molecule	O95727	CRTAM	chr11:122838500	Inverse variance weighted	6	0.035	0.038	3.556E-01	0.948	cis	Hospitalized COVID-19
10511_10	Collagen alpha-3(VI):isoform 3	Collagen alpha-3(VI) chain:isoform 3	P12111	COL6A3	chr2:237414328	Inverse variance weighted	2	0.085	0.092	3.558E-01	0.948	cis	Hospitalized COVID-19
2961_1	Protein C	Vitamin K-dependent protein C	P04070	PROC	chr2:127418427	Inverse variance weighted	3	0.067	0.073	3.559E-01	0.948	cis	Hospitalized COVID-19
2609_59	Cystatin C	Cystatin-C	P01034	CST3	chr20:23638473	Inverse variance weighted	2	0.094	0.102	3.572E-01	0.948	cis	Hospitalized COVID-19
6965_19	CNTP2	Contactin-associated protein-like 2	Q9UHC6	CNTNAP2	chr7:146116002	Inverse variance weighted	5	0.024	0.027	3.582E-01	0.948	cis	Hospitalized COVID-19
9796_4	CEL	Bile salt-activated lipase	P19835	CEL	chr9:133061981	Inverse variance weighted	5	-0.052	0.057	3.588E-01	0.948	cis	Hospitalized COVID-19
9175_48	DSCAM	Down syndrome cell adhesion molecule	O60469	DSCAM	chr21:40847158	Inverse variance weighted	2	-0.066	0.072	3.592E-01	0.948	cis	Hospitalized COVID-19
4220_39	FER	Tyrosine-protein kinase Fer	P16591	FER	chr5:108747841	Wald ratio	1	0.130	0.142	3.597E-01	0.948	cis	Hospitalized COVID-19
9241_40	SIRPG	Signal-regulatory protein gamma	Q9P1W8	SIRPG	chr20:1657779	Wald ratio	1	-0.122	0.133	3.603E-01	0.948	cis	Hospitalized COVID-19
11378_37	KSYK:SH2, 1 and 2	Tyrosine-protein kinase SYK:Src Homology domain	P43405	SYK	chr9:90801787	Wald ratio	1	0.202	0.221	3.604E-01	0.948	cis	Hospitalized COVID-19
9459_7	Fas, soluble	Tumor necrosis factor receptor superfamily member 6	P25445	FAS	chr10:88953813	Inverse variance weighted	2	-0.160	0.175	3.604E-01	0.948	cis	Hospitalized COVID-19
11134_30	RF1ML	Peptide chain release factor 1-like, mitochondrial	Q9UGC7	MTRF1L	chr6:153002709	Wald ratio	1	0.059	0.064	3.607E-01	0.948	cis	Hospitalized COVID-19
7266_4	SPA9	Serpin A9	Q86WD7	SERPINA9	chr14:94479689	Inverse variance weighted	2	0.104	0.114	3.621E-01	0.948	cis	Hospitalized COVID-19
13093_6	SECTM1	Secreted and transmembrane protein 1	Q8WVN6	SECTM1	chr17:82334074	Inverse variance weighted	3	-0.068	0.075	3.626E-01	0.948	cis	Hospitalized COVID-19
4534_10	BSSP4	Brain-specific serine protease 4	Q9GZN4	PRSS22	chr16:2858170	Inverse variance weighted	4	-0.045	0.050	3.632E-01	0.948	cis	Hospitalized COVID-19
5742_14	PPA6	Lysophosphatidic acid phosphatase type 6	Q9NPH0	ACP6	chr1:147670524	Inverse variance weighted	14	-0.015	0.016	3.642E-01	0.948	cis	Hospitalized COVID-19
17766_5	NCF-1	Neutrophil cytosol factor 1	P14598	NCF1	chr7:74774011	Inverse variance weighted	6	0.043	0.048	3.667E-01	0.948	cis	Hospitalized COVID-19
14067_6	PKP2	Plakophilin-2	Q99959	PKP2	chr12:32896777	Inverse variance weighted	2	0.182	0.202	3.672E-01	0.948	cis	Hospitalized COVID-19
6433_57	FA20A	Pseudokinase FAM20A	Q96MK3	FAM20A	chr17:68601367	Inverse variance weighted	3	-0.076	0.085	3.677E-01	0.948	cis	Hospitalized COVID-19
9172_69	MMP-8	Neutrophil collagenase	P22894	MMP8	chr11:102727050	Wald ratio	1	0.104	0.115	3.679E-01	0.948	cis	Hospitalized COVID-19
5598_3	GREM2	Gremlin-2	Q9H772	GREM2	chr1:240612155	Wald ratio	1	-0.155	0.172	3.684E-01	0.948	cis	Hospitalized COVID-19
8368_102	TNF sR-II	Tumor necrosis factor receptor superfamily member 1B	P20333	TNFRSF1B	chr1:12166991	Wald ratio	1	-0.057	0.063	3.702E-01	0.948	cis	Hospitalized COVID-19
2879_9	a1-Antichymotrypsin	Alpha-1-antichymotrypsin	P01011	SERPINA3	chr14:94612384	Wald ratio	1	-0.205	0.228	3.707E-01	0.948	cis	Hospitalized COVID-19
5227_60	PDK1	[Pyruvate dehydrogenase (acetyl-transferring)] kinase isozyme 1, mitochondrial	Q15118	PDK1	chr2:172555373	Inverse variance weighted	3	0.057	0.063	3.711E-01	0.948	cis	Hospitalized COVID-19
15529_33	Cysteine-rich protein 1	Cysteine and glycine-rich protein 1	P21291	CSRP1	chr1:201509456	Wald ratio	1	0.101	0.114	3.723E-01	0.948	cis	Hospitalized COVID-19
16318_12	ALK-1	Serine/threonine-protein kinase receptor R3	P37023	ACVRL1	chr12:51906908	Inverse variance weighted	3	0.046	0.051	3.727E-01	0.948	cis	Hospitalized COVID-19
10916_44	PLA2R	Secretory phospholipase A2 receptor	Q13018	PLA2R1	chr2:160062615	Inverse variance weighted	6	0.021	0.024	3.729E-01	0.948	cis	Hospitalized COVID-19
9931_20	Keratin-1	Keratin, type II cytoskeletal 1	P04264	KRT1	chr12:52680407	Wald ratio	1	0.219	0.246	3.735E-01	0.948	cis	Hospitalized COVID-19
8956_96	SREC-II:ECD	Scavenger receptor class F member 2:Extracellular domain	Q96GP6	SCARF2	chr22:20437826	Wald ratio	1	-0.087	0.098	3.739E-01	0.948	cis	Hospitalized COVID-19
19376_74	NNMT	Nicotinamide N-methyltransferase	P40261	NNMT	chr11:114257787	Wald ratio	1	0.152	0.171	3.745E-01	0.948	cis	Hospitalized COVID-19
19237_17	D-dopachrome decarboxylase	D-dopachrome decarboxylase	P30046	DDT	chr22:23980525	Inverse variance weighted	2	0.069	0.077	3.747E-01	0.948	cis	Hospitalized COVID-19
19173_5	ZFAN1	AN1-type zinc finger protein 1	Q8TCF1	ZFAND1	chr8:81732903	Inverse variance weighted	3	0.070	0.079	3.766E-01	0.948	cis	Hospitalized COVID-19
9748_31	GSTM3-3	Glutathione S-transferase Mu 3	P21266	GSTM3	chr1:109741038	Inverse variance weighted	5	-0.034	0.038	3.767E-01	0.948	cis	Hospitalized COVID-19
17722_5	FKBP52 protein	Peptidyl-prolyl cis-trans isomerase FKBP4	Q02790	FKBP4	chr12:2794970	Inverse variance weighted	2	0.130	0.147	3.768E-01	0.948	cis	Hospitalized COVID-19
15388_24	FcRIIIa	Low affinity immunoglobulin gamma Fc region receptor III-A	P08637	FCGR3A	chr1:161550968	Inverse variance weighted	6	-0.041	0.047	3.773E-01	0.948	cis	Hospitalized COVID-19
19365_11	BCAT2	Branched-chain-amino-acid aminotransferase, mitochondrial	O15382	BCAT2	chr19:48811029	Wald ratio	1	0.065	0.073	3.775E-01	0.948	cis	Hospitalized COVID-19
16322_10	PACAP	Marginal zone B- and B1-cell-specific protein	Q8WU39	MZB1	chr5:139390081	Inverse variance weighted	3	-0.096	0.109	3.777E-01	0.948	cis	Hospitalized COVID-19
13676_46	Inhibin bB chain	Inhibin beta B chain	P09529	INHBB	chr2:120346136	Inverse variance weighted	4	0.023	0.027	3.784E-01	0.948	cis	Hospitalized COVID-19
13563_259	ACBD7	Acyl-CoA-binding domain-containing protein 7	Q8N6N7	ACBD7	chr10:15088776	Wald ratio	1	0.232	0.265	3.798E-01	0.948	cis	Hospitalized COVID-19
8356_88	NEU1	Oxytocin-neurophysin 1	P01178	OXT	chr20:3071620	Inverse variance weighted	3	0.044	0.050	3.805E-01	0.948	cis	Hospitalized COVID-19
9886_28	XRCC4	DNA repair protein XRCC4	Q13426	XRCC4	chr5:83077498	Wald ratio	1	0.087	0.100	3.807E-01	0.948	cis	Hospitalized COVID-19
16763_11	LECT2	Leukocyte cell-derived chemotaxin-2	O14960	LECT2	chr5:135954983	Inverse variance weighted	8	0.026	0.029	3.815E-01	0.948	cis	Hospitalized COVID-19
19188_21	NAP2L	nucleosome assembly protein 1-like 4	Q99733	NAP1L4	chr11:2992377	Wald ratio	1	-0.058	0.066	3.815E-01	0.948	cis	Hospitalized COVID-19
19563_3	SEZ6L	Seizure 6-like protein	Q9BYH1	SEZ6L	chr22:26169462	Inverse variance weighted	3	0.077	0.088	3.819E-01	0.948	cis	Hospitalized COVID-19
11313_100	PHS	Pterin-4-alpha-carbinolamine dehydratase	P61457	PCBD1	chr10:70888565	Inverse variance weighted	5	0.055	0.063	3.826E-01	0.948	cis	Hospitalized COVID-19
2819_23	Cadherin-5	Cadherin-5	P33151	CDH5	chr16:66366622	Inverse variance weighted	2	0.078	0.090	3.829E-01	0.948	cis	Hospitalized COVID-19
16823_75	APOL3	Apolipoprotein L3	O95236	APOL3	chr22:36166177	Inverse variance weighted	7	0.026	0.029	3.829E-01	0.948	cis	Hospitalized COVID-19
8664_36	PKDCC	Extracellular tyrosine-protein kinase PKDCC	Q504Y2	PKDCC	chr2:42048021	Inverse variance weighted	2	-0.240	0.275	3.830E-01	0.948	cis	Hospitalized COVID-19

9468_8	Lectin, mannose-binding 2	Vesicular integral-membrane protein VIP36	Q12907	LMAN2	chr5:177351840	Wald ratio	1	0.119	0.137	3.834E-01	0.948	cis	Hospitalized COVID-19
15523_9	HEM2	Delta-aminolevulinic acid dehydratase	P13716	ALAD	chr9:113401290	Inverse variance weighted	7	0.047	0.054	3.834E-01	0.948	cis	Hospitalized COVID-19
4314_12	XTP3A	dCTP pyrophosphatase 1	Q9H773	DCTPP1	chr16:30430030	Wald ratio	1	0.116	0.133	3.839E-01	0.948	cis	Hospitalized COVID-19
6049_64	PTPRS	Receptor-type tyrosine-protein phosphatase S	Q13332	PTPRS	chr19:5340812	Wald ratio	1	0.108	0.124	3.841E-01	0.948	cis	Hospitalized COVID-19
5061_27	B7-H2	ICOS ligand	O75144	ICOSLG	chr21:44241446	Wald ratio	1	-0.184	0.212	3.855E-01	0.948	cis	Hospitalized COVID-19
18396_10	AES	Amino-terminal enhancer of split	Q08117	TLE5	chr19:3063107	Wald ratio	1	-0.185	0.213	3.855E-01	0.948	cis	Hospitalized COVID-19
13704_5	HMCS2	Hydroxymethylglutaryl-CoA synthase, mitochondrial	P54868	HMGCS2	chr1:119768905	Wald ratio	1	0.192	0.221	3.864E-01	0.948	cis	Hospitalized COVID-19
6572_10	LRRT4:ECD	Leucine-rich repeat transmembrane neuronal protein 4:Isoform 2, Extracellular domain	Q86VH4	LRRTM4	chr2:77593319	Wald ratio	1	-0.188	0.217	3.865E-01	0.948	cis	Hospitalized COVID-19
4153_11	alpha-1-antichymotrypsin complex	Alpha-1-antichymotrypsin complex	P07288 | P01011	KLK3 | SERPINA3	chr14:94612384	Inverse variance weighted	3	-0.087	0.101	3.871E-01	0.948	cis	Hospitalized COVID-19
3171_57	amyloid precursor protein	Amyloid beta A4 protein	P05067	APP	chr21:26171128	Wald ratio	1	0.119	0.138	3.875E-01	0.948	cis	Hospitalized COVID-19
13697_51	GPDA	Glycerol-3-phosphate dehydrogenase [NAD(+)], cytoplasmic	P21695	GPD1	chr12:50103982	Wald ratio	1	0.114	0.132	3.882E-01	0.948	cis	Hospitalized COVID-19
12635_9	TRDMT	tRNA (cytosine(38)-C(5))-methyltransferase	O14717	TRDMT1	chr10:17202054	Inverse variance weighted	4	-0.045	0.052	3.891E-01	0.948	cis	Hospitalized COVID-19
6507_16	NCAM2	Neural cell adhesion molecule 2	O15394	NCAM2	chr21:20998409	Inverse variance weighted	6	0.025	0.029	3.897E-01	0.948	cis	Hospitalized COVID-19
12517_52	TFAR19	Programmed cell death protein 5	O14737	PDCD5	chr19:32581190	Inverse variance weighted	4	-0.030	0.035	3.901E-01	0.948	cis	Hospitalized COVID-19
3329_14	PGRP-S	Peptidoglycan recognition protein 1	O75594	PGLYRP1	chr19:46023053	Inverse variance weighted	4	-0.044	0.051	3.901E-01	0.948	cis	Hospitalized COVID-19
6409_57	GP116	Adhesion G protein-coupled receptor F5	Q8IZF2	ADGRF5	chr6:46954943	Inverse variance weighted	7	-0.017	0.019	3.904E-01	0.948	cis	Hospitalized COVID-19
5649_83	PSG4	Pregnancy-specific beta-1-glycoprotein 4	Q00888	PSG4	chr19:43207299	Inverse variance weighted	3	-0.068	0.080	3.913E-01	0.948	cis	Hospitalized COVID-19
5660_51	SOD3	Extracellular superoxide dismutase [Cu-Zn]	P08294	SOD3	chr4:24789912	Inverse variance weighted	9	-0.022	0.025	3.920E-01	0.948	cis	Hospitalized COVID-19
19523_215	PARK7	Protein DJ-1	Q99497	PARK7	chr1:7954291	Wald ratio	1	-0.083	0.097	3.925E-01	0.948	cis	Hospitalized COVID-19
15462_28	CD8A	T-cell surface glycoprotein CD8 alpha chain	P01732	CD8A	chr2:86808396	Inverse variance weighted	2	-0.028	0.033	3.925E-01	0.948	cis	Hospitalized COVID-19
7161_25	G6PE	GDH/6PGL endoplasmic bifunctional protein	O95479	H6PD	chr1:9234774	Inverse variance weighted	9	-0.026	0.031	3.942E-01	0.948	cis	Hospitalized COVID-19
15509_2	NAG	Alpha-N-acetylglucosaminidase	P54802	NAGLU	chr17:42536241	Inverse variance weighted	6	0.017	0.020	3.947E-01	0.948	cis	Hospitalized COVID-19
7970_315	NAR3	Ecto-ADP-ribosyltransferase 3	Q13508	ART3	chr4:76011184	Inverse variance weighted	4	0.039	0.046	3.955E-01	0.948	cis	Hospitalized COVID-19
11369_23	ADHX	Alcohol dehydrogenase class-3	P11766	ADH5	chr4:99088801	Inverse variance weighted	3	-0.136	0.160	3.955E-01	0.948	cis	Hospitalized COVID-19
5346_24	CPNE1:C2, 1 and 2	Copine-1:Ca2+-dependent membrane-targeting module domains 1 and 2	Q99829	CPNE1	chr20:35664956	Inverse variance weighted	9	0.016	0.019	3.965E-01	0.948	cis	Hospitalized COVID-19
6617_12	FCRL6	Fc receptor-like protein 6	Q6DN72	FCRL6	chr1:159800511	Wald ratio	1	-0.074	0.088	3.979E-01	0.948	cis	Hospitalized COVID-19
17672_184	Gastric intrinsic factor	Gastric intrinsic factor	P27352	CBLIF	chr11:59845499	Inverse variance weighted	2	0.097	0.114	3.982E-01	0.948	cis	Hospitalized COVID-19
7948_129	GLTD2	Glycolipid transfer protein domain-containing protein 2	A6NH11	GLTPD2	chr17:4788964	Inverse variance weighted	3	0.041	0.048	3.983E-01	0.948	cis	Hospitalized COVID-19
4886_3	MCP-3	C-C motif chemokine 7	P80098	CCL7	chr17:34270221	Inverse variance weighted	4	-0.065	0.077	3.992E-01	0.948	cis	Hospitalized COVID-19
7648_9	MYPC1	Myosin-binding protein C, slow-type	Q00872	MYBPC1	chr12:101568353	Wald ratio	1	0.104	0.124	3.993E-01	0.948	cis	Hospitalized COVID-19
19614_8	Holo-TC I	Transcobalamin-1	P20061	TCN1	chr11:59866489	Inverse variance weighted	5	-0.035	0.041	4.006E-01	0.948	cis	Hospitalized COVID-19
17210_2	TCL1A	T-cell leukemia/lymphoma protein 1A	P56279	TCL1A	chr14:95714196	Inverse variance weighted	3	-0.043	0.052	4.006E-01	0.948	cis	Hospitalized COVID-19
17170_15	CALCB	Calcitonin gene-related peptide 2	P10092	CALCB	chr11:14904997	Inverse variance weighted	2	-0.039	0.047	4.014E-01	0.948	cis	Hospitalized COVID-19
7955_195	ITI heavy chain H1	Inter-alpha-trypsin inhibitor heavy chain H1	P19827	ITIH1	chr3:52777595	Inverse variance weighted	7	0.047	0.056	4.021E-01	0.948	cis	Hospitalized COVID-19
11643_73	DTX3L	E3 ubiquitin-protein ligase DTX3L	Q8TDB6	DTX3L	chr3:122564338	Wald ratio	1	-0.160	0.191	4.028E-01	0.948	cis	Hospitalized COVID-19
7841_84	ESAM	Endothelial cell-selective adhesion molecule	Q96AP7	ESAM	chr11:124762290	Inverse variance weighted	6	0.034	0.040	4.035E-01	0.948	cis	Hospitalized COVID-19
3212_30	ASAH2	Neutral ceramidase	Q9NR71	ASAH2	chr10:50279720	Inverse variance weighted	5	-0.017	0.021	4.045E-01	0.948	cis	Hospitalized COVID-19
6984_6	IGSF8	Immunoglobulin superfamily member 8	Q969P0	IGSF8	chr1:160098943	Inverse variance weighted	2	0.056	0.068	4.054E-01	0.948	cis	Hospitalized COVID-19
2982_82	Galectin-4	Galectin-4	P56470	LGALS4	chr19:38812945	Wald ratio	1	-0.152	0.183	4.076E-01	0.948	cis	Hospitalized COVID-19
13578_98	ABLM3	Actin-binding LIM protein 3	O94929	ABLIM3	chr5:149141483	Wald ratio	1	-0.082	0.098	4.077E-01	0.948	cis	Hospitalized COVID-19
3581_53	a2-HS-Glycoprotein	Alpha-2-HS-glycoprotein	P02765	AHSG	chr3:186613060	Inverse variance weighted	4	-0.067	0.081	4.077E-01	0.948	cis	Hospitalized COVID-19
17814_8	BPNT1	3'(2'),5'-bisphosphate nucleotidase 1	O95861	BPNT1	chr1:220090462	Wald ratio	1	0.152	0.184	4.081E-01	0.948	cis	Hospitalized COVID-19
9263_57	SIAE	Sialate O-acetylesterase	Q9HAT2	SIAE	chr11:124695707	Wald ratio	1	0.091	0.111	4.100E-01	0.948	cis	Hospitalized COVID-19
6527_1	TRIL:ECD	TLR4 interactor with leucine rich repeats:Extracellular domain	Q7L0X0	TRIL	chr7:28958330	Wald ratio	1	0.109	0.133	4.100E-01	0.948	cis	Hospitalized COVID-19
7891_45	UGT 1A6	UDP-glucuronosyltransferase 1-6	P19224	UGT1A6	chr2:233691607	Inverse variance weighted	4	-0.067	0.082	4.109E-01	0.948	cis	Hospitalized COVID-19
17677_47	BY55	CD160 antigen	O95971	CD160	chr1:145719471	Wald ratio	1	-0.080	0.097	4.117E-01	0.948	cis	Hospitalized COVID-19
15468_14	FHR1	Complement factor H-related protein 1	Q03591	CFHR1	chr1:196819731	Inverse variance weighted	6	-0.027	0.033	4.119E-01	0.948	cis	Hospitalized COVID-19
14090_23	DEFI6	Differentially expressed in FDCP 6 homolog	Q9H4E7	DEF6	chr6:35297818	Inverse variance weighted	2	-0.047	0.057	4.122E-01	0.948	cis	Hospitalized COVID-19
3079_62	TIG2	Retinoic acid receptor responder protein 2	Q99969	RARRES2	chr7:150341662	Inverse variance weighted	2	0.052	0.064	4.126E-01	0.948	cis	Hospitalized COVID-19
17813_21	BPHL	Valacyclovir hydrolase	Q86WA6	BPHL	chr6:3118374	Inverse variance weighted	2	0.098	0.120	4.128E-01	0.948	cis	Hospitalized COVID-19
19615_213	5NT3	Cytosolic 5'-nucleotidase 3A	Q9H0P0	NT5C3A	chr7:33062796	Wald ratio	1	0.108	0.132	4.134E-01	0.948	cis	Hospitalized COVID-19
7019_13	Semaphorin-7A	Semaphorin-7A	O75326	SEMA7A	chr15:74433958	Wald ratio	1	0.104	0.128	4.136E-01	0.948	cis	Hospitalized COVID-19
13666_222	Carbonic Anhydrase X	Carbonic anhydrase-related protein 10	Q9NS85	CA10	chr17:52160017	Inverse variance weighted	4	-0.040	0.050	4.146E-01	0.948	cis	Hospitalized COVID-19
9416_77	CBPM	Carboxypeptidase M	P14384	CPM	chr12:68971570	Wald ratio	1	0.086	0.106	4.151E-01	0.948	cis	Hospitalized COVID-19
11431_235	RECQ1	ATP-dependent DNA helicase Q1	P46063	RECQL	chr12:21501669	Inverse variance weighted	7	0.018	0.022	4.160E-01	0.948	cis	Hospitalized COVID-19
10534_40	PARP:BRCT domain	Poly [ADP-ribose] polymerase 1:BRCA1 C-terminal:BRCA1 C-terminus domian	P09874	PARP1	chr1:226408154	Wald ratio	1	-0.179	0.220	4.160E-01	0.948	cis	Hospitalized COVID-19
5404_53	DR6	Tumor necrosis factor receptor superfamily member 21	O75509	TNFRSF21	chr6:47309905	Inverse variance weighted	2	-0.084	0.104	4.163E-01	0.948	cis	Hospitalized COVID-19
19496_1	NPL	N-acetylneuraminate lyase	Q9BXD5	NPL	chr1:182789293	Inverse variance weighted	3	-0.050	0.061	4.165E-01	0.948	cis	Hospitalized COVID-19
3389_7	PCI	Plasma serine protease inhibitor	P05154	SERPINA5	chr14:94561442	Inverse variance weighted	2	-0.114	0.141	4.167E-01	0.948	cis	Hospitalized COVID-19
9574_11	BIN1	Myc box-dependent-interacting protein 1	O00499	BIN1	chr2:127107288	Inverse variance weighted	2	0.058	0.072	4.167E-01	0.948	cis	Hospitalized COVID-19
3151_6	IL-2 sRa	Interleukin-2 receptor subunit alpha	P01589	IL2RA	chr10:6062370	Wald ratio	1	0.093	0.114	4.168E-01	0.948	cis	Hospitalized COVID-19
18310_26	SELW	Selenoprotein W	P63302	SELENOW	chr19:47778677	Inverse variance weighted	3	0.054	0.066	4.171E-01	0.948	cis	Hospitalized COVID-19
5483_1	RGMA	Repulsive guidance molecule A	Q96B86	RGMA	chr15:93089211	Inverse variance weighted	4	0.044	0.054	4.178E-01	0.948	cis	Hospitalized COVID-19
17819_30	FAHD1	Acylpyruvase FAHD1, mitochondrial	Q6P587	FAHD1	chr16:1826967	Inverse variance weighted	3	0.092	0.113	4.179E-01	0.948	cis	Hospitalized COVID-19
5108_72	Notch-3	Neurogenic locus notch homolog protein 3	Q9UM47	NOTCH3	chr19:15200995	Wald ratio	1	-0.096	0.119	4.182E-01	0.948	cis	Hospitalized COVID-19
6990_44	SDF2L	Stromal cell-derived factor 2-like protein 1	Q9HCN8	SDF2L1	chr22:21642302	Wald ratio	1	0.147	0.183	4.207E-01	0.948	cis	Hospitalized COVID-19
10512_13	IL3RB:ECD	Cytokine receptor common subunit beta:Extracellular domain	P32927	CSF2RB	chr22:36913628	Inverse variance weighted	7	-0.016	0.019	4.208E-01	0.948	cis	Hospitalized COVID-19
3169_70	IDUA	Alpha-L-iduronidase	P35475	IDUA	chr4:986997	Inverse variance weighted	7	0.046	0.057	4.215E-01	0.948	cis	Hospitalized COVID-19
4141_79	IP-10	C-X-C motif chemokine 10	P02778	CXCL10	chr4:76023497	Wald ratio	1	-0.171	0.213	4.217E-01	0.948	cis	Hospitalized COVID-19
15515_2	SAA	Serum amyloid A-1 protein	P0DJI8	SAA1	chr11:18266260	Inverse variance weighted	4	-0.067	0.084	4.221E-01	0.948	cis	Hospitalized COVID-19
7822_11	HRSL2	HRAS-like suppressor 2	Q9NWW9	PLAAT2	chr11:63563379	Wald ratio	1	-0.178	0.222	4.228E-01	0.948	cis	Hospitalized COVID-19
15622_13	OBCAM	Opioid-binding protein/cell adhesion molecule	Q14982	OPCML	chr11:133532501	Wald ratio	1	-0.175	0.219	4.229E-01	0.948	cis	Hospitalized COVID-19
15471_29	LIPR2	Pancreatic lipase-related protein 2	P54317	PNLIPRP2	chr10:116620953	Inverse variance weighted	11	0.012	0.015	4.252E-01	0.948	cis	Hospitalized COVID-19
6448_36	Sema E	Semaphorin-3C	Q99985	SEMA3C	chr7:80922359	Inverse variance weighted	2	-0.067	0.085	4.259E-01	0.948	cis	Hospitalized COVID-19
15521_4	Alcadein alpha-1	Calsyntenin-1	O94985	CLSTN1	chr1:9823984	Inverse variance weighted	6	0.012	0.015	4.261E-01	0.948	cis	Hospitalized COVID-19
16049_43	OLR1	Oxidized low-density lipoprotein receptor 1	P78380	OLR1	chr12:10172138	Wald ratio	1	0.105	0.132	4.262E-01	0.948	cis	Hospitalized COVID-19
12851_5	DPOLM	DNA-directed DNA/RNA polymerase mu	Q9NP87	POLM	chr7:44082530	Inverse variance weighted	3	0.054	0.068	4.264E-01	0.948	cis	Hospitalized COVID-19
3470_1	sE-Selectin	E-selectin	P16581	SELE	chr1:169764705	Wald ratio	1	0.103	0.129	4.265E-01	0.948	cis	Hospitalized COVID-19

13692_154	WISP-1	WNT1-inducible-signaling pathway protein 1	O95388	CCN4	chr8:133191039	Inverse variance weighted	8	-0.017	0.021	4.265E-01	0.948	cis	Hospitalized COVID-19
6493_9	CBPZ	Carboxypeptidase Z	Q66K79	CPZ	chr4:8592660	Inverse variance weighted	2	0.088	0.111	4.266E-01	0.948	cis	Hospitalized COVID-19
13733_5	IL-12 p40	Interleukin-12 subunit beta	P29460	IL12B	chr5:159330863	Inverse variance weighted	7	-0.020	0.025	4.268E-01	0.948	cis	Hospitalized COVID-19
3831_21	pTEN	Phosphatidylinositol 3,4,5-trisphosphate 3-phosphatase and dual-specificity protein phosphatase PTEN	P60484	PTEN	chr10:87862563	Wald ratio	1	0.199	0.250	4.269E-01	0.948	cis	Hospitalized COVID-19
15452_5	5'-Nucleotidase	5'-Nucleotidase	P21589	NT5E	chr6:85449584	Inverse variance weighted	4	-0.021	0.026	4.270E-01	0.948	cis	Hospitalized COVID-19
9409_11	TRYB1	Tryptase beta-1	Q15661	TPSAB1	chr16:1240379	Inverse variance weighted	8	0.018	0.023	4.273E-01	0.948	cis	Hospitalized COVID-19
11273_176	GSTT2	Glutathione S-transferase theta-2B	P0CG30	GSTT2B	chr22:23961195	Inverse variance weighted	4	0.037	0.047	4.281E-01	0.948	cis	Hospitalized COVID-19
19277_4	KAT	Thiosulfate sulfurtransferase/rhodanese-like domain-containing protein 1	Q8NFU3	TSTD1	chr1:161038977	Inverse variance weighted	2	0.052	0.066	4.284E-01	0.948	cis	Hospitalized COVID-19
5691_2	CRLD2	Cysteine-rich secretory protein LCCL domain-containing 2	Q9H0B8	CRISPLD2	chr16:84819985	Inverse variance weighted	6	0.033	0.042	4.289E-01	0.948	cis	Hospitalized COVID-19
4866_59	TrkB	BDNF/NT-3 growth factors receptor	Q16620	NTRK2	chr9:84668375	Wald ratio	1	-0.132	0.167	4.291E-01	0.948	cis	Hospitalized COVID-19
3352_80	Carbonic anhydrase 6	Carbonic anhydrase 6	P23280	CA6	chr1:8945867	Inverse variance weighted	6	-0.019	0.024	4.296E-01	0.948	cis	Hospitalized COVID-19
14133_93	IL-1 sRII	Interleukin-1 receptor type 2	P27930	IL1R2	chr2:101991960	Inverse variance weighted	7	-0.035	0.044	4.301E-01	0.948	cis	Hospitalized COVID-19
5034_79	Trypsin 2	Trypsin-2	P07478	PRSS2	chr7:142760398	Inverse variance weighted	4	-0.060	0.077	4.312E-01	0.948	cis	Hospitalized COVID-19
10816_150	PILRA isoform FDF03-M14	Paired immunoglobulin-like type 2 receptor alpha isoform FDF03-M14	Q9UKJ1	PILRA	chr7:100367530	Inverse variance weighted	3	0.080	0.102	4.317E-01	0.948	cis	Hospitalized COVID-19
4801_13	PERL	Lactoperoxidase	P22079	LPO	chr17:58218548	Inverse variance weighted	2	0.036	0.046	4.323E-01	0.948	cis	Hospitalized COVID-19
10088_37	APT	Adenine phosphoribosyltransferase	P07741	APRT	chr16:88811937	Inverse variance weighted	4	-0.040	0.051	4.326E-01	0.948	cis	Hospitalized COVID-19
5301_7	Eotaxin	Eotaxin	P51671	CCL11	chr17:34285742	Wald ratio	1	-0.090	0.115	4.326E-01	0.948	cis	Hospitalized COVID-19
19338_3	LZIC	Protein LZIC	Q8WZA0	LZIC	chr1:9943407	Wald ratio	1	-0.210	0.267	4.327E-01	0.948	cis	Hospitalized COVID-19
9018_38	PCD10:ECD	Protocadherin-10:Extracellular domain	Q9P2E7	PCDH10	chr4:133149294	Inverse variance weighted	2	0.047	0.059	4.332E-01	0.948	cis	Hospitalized COVID-19
12338_27	EGFLA	Pikachurin	Q63HQ2	EGFLAM	chr5:38258409	Inverse variance weighted	3	0.037	0.048	4.337E-01	0.948	cis	Hospitalized COVID-19
4559_64	KYNU	Kynureninase	Q16719	KYNU	chr2:142877657	Inverse variance weighted	4	-0.036	0.045	4.341E-01	0.948	cis	Hospitalized COVID-19
17755_5	UGDH	UDP-glucose 6-dehydrogenase	O60701	UGDH	chr4:39528311	Inverse variance weighted	3	-0.037	0.047	4.345E-01	0.948	cis	Hospitalized COVID-19
6525_17	DUSP13	Dual specificity protein phosphatase 13 isoform A	Q6B8I1	DUSP13	chr10:75109221	Inverse variance weighted	2	0.030	0.039	4.345E-01	0.948	cis	Hospitalized COVID-19
5736_1	TRML2	Trem-like transcript 2 protein	Q5T2D2	TREML2	chr6:41201149	Inverse variance weighted	5	-0.026	0.033	4.348E-01	0.948	cis	Hospitalized COVID-19
2475_1	SCF sR	Mast/stem cell growth factor receptor Kit	P10721	KIT	chr4:54657267	Wald ratio	1	-0.159	0.204	4.352E-01	0.948	cis	Hospitalized COVID-19
13132_14	SEM5A	Semaphorin-5A	Q13591	SEMA5A	chr5:9546075	Inverse variance weighted	10	-0.010	0.013	4.352E-01	0.948	cis	Hospitalized COVID-19
5698_60	Tenascin-X	Tenascin-X	P22105	TNXB	chr6:32115334	Inverse variance weighted	5	0.022	0.029	4.353E-01	0.948	cis	Hospitalized COVID-19
18173_11	ARK73	Aflatoxin B1 aldehyde reductase member 3	O95154	AKR7A3	chr1:19288770	Inverse variance weighted	5	0.028	0.036	4.358E-01	0.948	cis	Hospitalized COVID-19
15364_101	Apo C-I	Apolipoprotein C-I	P02654	APOC1	chr19:44914247	Inverse variance weighted	5	-0.041	0.053	4.362E-01	0.948	cis	Hospitalized COVID-19
16621_77	AIBP	NAD(P)H-hydrate epimerase	Q8NCW5	NAXE	chr1:156591756	Wald ratio	1	0.037	0.047	4.382E-01	0.948	cis	Hospitalized COVID-19
3168_8	ADAMTS-5	A disintegrin and metalloproteinase with thrombospondin motifs 5	Q9UNA0	ADAMTS5	chr21:26967088	Inverse variance weighted	5	0.022	0.029	4.387E-01	0.948	cis	Hospitalized COVID-19
3508_78	MDC	C-C motif chemokine 22	O00626	CCL22	chr16:57358783	Inverse variance weighted	5	-0.039	0.050	4.389E-01	0.948	cis	Hospitalized COVID-19
15560_52	Apo-TC II	Transcobalamin-2	P20062	TCN2	chr22:30607003	Inverse variance weighted	4	0.020	0.026	4.396E-01	0.948	cis	Hospitalized COVID-19
11510_31	Apo L1	Apolipoprotein L1	O14791	APOL1	chr22:36253071	Inverse variance weighted	5	-0.028	0.036	4.397E-01	0.948	cis	Hospitalized COVID-19
15435_4	PNP	Purine nucleoside phosphorylase	P00491	PNP	chr14:20461992	Inverse variance weighted	2	-0.051	0.066	4.417E-01	0.948	cis	Hospitalized COVID-19
7980_72	B3GN2	N-acetyllactosaminide beta-1,3-N-acetylglucosaminyltransferase 2	Q9NY97	B3GNT2	chr2:62196115	Inverse variance weighted	3	-0.043	0.055	4.419E-01	0.948	cis	Hospitalized COVID-19
16309_30	Soggy-1	Dickkopf-like protein 1	Q9UK85	DKKL1	chr19:49361783	Wald ratio	1	0.224	0.291	4.422E-01	0.948	cis	Hospitalized COVID-19
9335_28	PSG9	Pregnancy-specific beta-1-glycoprotein 9	Q00887	PSG9	chr19:43269530	Wald ratio	1	-0.122	0.159	4.424E-01	0.948	cis	Hospitalized COVID-19
16606_85	Aldose reductase	Aldose reductase	P15121	AKR1B1	chr7:134459284	Inverse variance weighted	2	0.128	0.167	4.428E-01	0.948	cis	Hospitalized COVID-19
4278_14	Protein disulfide-isomerase	Protein disulfide-isomerase	P07237	P4HB	chr17:81860856	Wald ratio	1	-0.076	0.099	4.443E-01	0.948	cis	Hospitalized COVID-19
9251_28	MA2B2	Epididymis-specific alpha-mannosidase	Q9Y2E5	MAN2B2	chr4:6575189	Inverse variance weighted	9	0.018	0.023	4.454E-01	0.948	cis	Hospitalized COVID-19
15594_47	HTRA1	Serine protease HTRA1	Q92743	HTRA1	chr10:122458551	Inverse variance weighted	2	0.081	0.107	4.456E-01	0.948	cis	Hospitalized COVID-19
7083_74	MATN4	Matrilin-4	O95460	MATN4	chr20:45308529	Inverse variance weighted	3	0.046	0.061	4.468E-01	0.948	cis	Hospitalized COVID-19
17231_1	L-plastin	Plastin-2	P13796	LCP1	chr13:46211871	Inverse variance weighted	4	0.047	0.063	4.476E-01	0.948	cis	Hospitalized COVID-19
3853_56	MDHC	Malate dehydrogenase, cytoplasmic	P40925	MDH1	chr2:63588609	Wald ratio	1	-0.114	0.150	4.490E-01	0.948	cis	Hospitalized COVID-19
4187_49	6-Phosphogluconate dehydrogenase	6-phosphogluconate dehydrogenase, decarboxylating	P52209	PGD	chr1:10398592	Wald ratio	1	0.102	0.136	4.514E-01	0.948	cis	Hospitalized COVID-19
13114_50	Lumican	Lumican	P51884	LUM	chr12:91111494	Inverse variance weighted	7	0.029	0.038	4.520E-01	0.948	cis	Hospitalized COVID-19
2985_35	Gro-a	Growth-regulated alpha protein	P09341	CXCL1	chr4:73869393	Inverse variance weighted	8	0.024	0.032	4.521E-01	0.948	cis	Hospitalized COVID-19
9211_19	PEDF	Pigment epithelium-derived factor	P36955	SERPINF1	chr17:1762029	Inverse variance weighted	3	0.028	0.037	4.528E-01	0.948	cis	Hospitalized COVID-19
8970_9	RIPK2	Receptor-interacting serine/threonine-protein kinase 2	O43353	RIPK2	chr8:89757806	Wald ratio	1	-0.135	0.180	4.529E-01	0.948	cis	Hospitalized COVID-19
3186_2	C2	Complement C2	P06681	C2	chr6:31897785	Wald ratio	1	-0.084	0.112	4.551E-01	0.948	cis	Hospitalized COVID-19
5584_21	Holo-TC II	Holo-Transcobalamin-2	P20062	TCN2	chr22:30607003	Inverse variance weighted	4	-0.031	0.041	4.554E-01	0.948	cis	Hospitalized COVID-19
17765_3	SPN1	Snurportin-1	O95149	SNUPN	chr15:75626469	Wald ratio	1	0.057	0.077	4.559E-01	0.948	cis	Hospitalized COVID-19
11333_82	RHG25	Rho GTPase-activating protein 25	P42331	ARHGAP25	chr2:68679601	Wald ratio	1	0.053	0.071	4.566E-01	0.948	cis	Hospitalized COVID-19
15316_262	TXN4B	Thioredoxin-like protein 4B	Q9NX01	TXNL4B	chr16:72094431	Wald ratio	1	0.089	0.119	4.566E-01	0.948	cis	Hospitalized COVID-19
9398_30	GALP	Galanin-like peptide	Q9UBC7	GALP	chr19:56176008	Wald ratio	1	-0.124	0.167	4.570E-01	0.948	cis	Hospitalized COVID-19
13622_16	2A5A	Serine/threonine-protein phosphatase 2A 56 kDa regulatory subunit alpha isoform	Q15172	PPP2R5A	chr1:212285410	Wald ratio	1	-0.155	0.208	4.578E-01	0.948	cis	Hospitalized COVID-19
14134_49	AMGO2	Amphoterin-induced protein 2	Q86SJ2	AMIGO2	chr12:47079959	Inverse variance weighted	3	-0.063	0.085	4.583E-01	0.948	cis	Hospitalized COVID-19
5087_5	IL-22BP	Interleukin-22 receptor subunit alpha-2	Q969J5	IL22RA2	chr6:137173648	Inverse variance weighted	4	0.052	0.071	4.592E-01	0.948	cis	Hospitalized COVID-19
9343_16	IL-2 sRb	Interleukin-2 receptor subunit beta	P14784	IL2RB	chr22:37175118	Wald ratio	1	-0.083	0.112	4.608E-01	0.948	cis	Hospitalized COVID-19
18881_7	CD97	CD97 antigen	P48960	ADGRE5	chr19:14380501	Inverse variance weighted	4	0.060	0.082	4.611E-01	0.948	cis	Hospitalized COVID-19
17685_9	Apo A-IV	Apolipoprotein A-IV	P06727	APOA4	chr11:116823304	Wald ratio	1	-0.164	0.222	4.612E-01	0.948	cis	Hospitalized COVID-19
4990_87	GP1BA	Platelet glycoprotein Ib alpha chain	P07359	GP1BA	chr17:4932277	Inverse variance weighted	3	-0.072	0.097	4.613E-01	0.948	cis	Hospitalized COVID-19
8960_3	LRAP	Endoplasmic reticulum aminopeptidase 2	Q6P179	ERAP2	chr5:96875986	Inverse variance weighted	4	-0.017	0.023	4.615E-01	0.948	cis	Hospitalized COVID-19
16605_2	C1T9A	Complement C1q and tumor necrosis factor-related protein 9A	P0C862	C1QTNF9	chr13:24307166	Wald ratio	1	0.041	0.056	4.618E-01	0.948	cis	Hospitalized COVID-19
5610_32	CJ038	Protein FAM171A1	Q5VUB5	FAM171A1	chr10:15371289	Inverse variance weighted	2	-0.096	0.130	4.621E-01	0.948	cis	Hospitalized COVID-19
7957_2	SCG3	Secretogranin-3	Q8WXD2	SCG3	chr15:51681492	Inverse variance weighted	7	-0.019	0.026	4.624E-01	0.948	cis	Hospitalized COVID-19
8258_22	UXS1	UDP-glucuronic acid decarboxylase 1	Q8NBZ7	UXS1	chr2:106194301	Wald ratio	1	-0.109	0.148	4.628E-01	0.948	cis	Hospitalized COVID-19
13657_2	PNKP	Bifunctional polynucleotide phosphatase/kinase	Q96T60	PNKP	chr19:49878351	Wald ratio	1	0.075	0.102	4.631E-01	0.948	cis	Hospitalized COVID-19
19377_14	NOE2	Noelin-2	O95897	OLFM2	chr19:9936515	Inverse variance weighted	6	-0.020	0.028	4.632E-01	0.948	cis	Hospitalized COVID-19
10442_1	TM190	Transmembrane protein 190	Q8WZ59	TMEM190	chr19:55376826	Inverse variance weighted	9	-0.014	0.019	4.640E-01	0.948	cis	Hospitalized COVID-19
17843_30	PPCS	Phosphopantothenate--cysteine ligase	Q9HAB8	PPCS	chr1:42456117	Inverse variance weighted	2	0.063	0.086	4.641E-01	0.948	cis	Hospitalized COVID-19
3293_2	CD5L	CD5 antigen-like	O43866	CD5L	chr1:157898256	Inverse variance weighted	4	-0.045	0.062	4.647E-01	0.948	cis	Hospitalized COVID-19
19372_7	MDGA2	MAM domain-containing glycosylphosphatidylinositol anchor protein 2	Q7Z553	MDGA2	chr14:47675605	Inverse variance weighted	10	-0.012	0.016	4.652E-01	0.948	cis	Hospitalized COVID-19
9204_33	Corticotropin-lipotropin	Pro-opiomelanocortin	P01189	POMC	chr2:25168903	Inverse variance weighted	3	-0.069	0.095	4.657E-01	0.948	cis	Hospitalized COVID-19
4706_17	41	Protein 4.1	P11171	EPB41	chr1:28887091	Inverse variance weighted	2	-0.110	0.151	4.660E-01	0.948	cis	Hospitalized COVID-19
5095_21	KI2L4	Killer cell immunoglobulin-like receptor 2DL4	Q99706	KIR2DL4	chr19:54803610	Inverse variance weighted	2	-0.040	0.055	4.664E-01	0.948	cis	Hospitalized COVID-19
19233_75	ATOX1	Copper transport protein ATOX1	O00244	ATOX1	chr5:151772532	Wald ratio	1	0.144	0.198	4.665E-01	0.948	cis	Hospitalized COVID-19

9339_204	FKBP2	Peptidyl-prolyl cis-trans isomerase FKBP2	P26885	FKBP2	chr11:64241003	Wald ratio	1	-0.147	0.202	4.673E-01	0.948	cis	Hospitalized COVID-19
5634_39	OFUT1	GDP-fucose protein O-fucosyltransferase 1	Q9H488	POFUT1	chr20:32207855	Inverse variance weighted	3	-0.030	0.042	4.678E-01	0.948	cis	Hospitalized COVID-19
19113_66	SH3L2	SH3 domain-binding glutamic acid-rich-like protein 2	Q9UJC5	SH3BGRL2	chr6:79631329	Wald ratio	1	-0.072	0.100	4.692E-01	0.948	cis	Hospitalized COVID-19
9906_21	TICN3	Testican-3	Q9BQ16	SPOCK3	chr4:167234796	Inverse variance weighted	11	0.017	0.023	4.695E-01	0.948	cis	Hospitalized COVID-19
5353_89	IL-1Ra	Interleukin-1 receptor antagonist protein	P18510	IL1RN	chr2:113099315	Inverse variance weighted	3	0.034	0.047	4.701E-01	0.948	cis	Hospitalized COVID-19
12855_16	CT032	Cas scaffolding protein family member 4	Q9NQ75	CASS4	chr20:56412112	Wald ratio	1	-0.141	0.196	4.711E-01	0.948	cis	Hospitalized COVID-19
12831_21	TESC	Calcineurin B homologous protein 3	Q96BS2	TESC	chr12:117099479	Inverse variance weighted	6	0.022	0.031	4.722E-01	0.948	cis	Hospitalized COVID-19
10391_1	ANGL3	Angiopoietin-related protein 3	Q9Y5C1	ANGPTL3	chr1:62597520	Inverse variance weighted	7	-0.023	0.032	4.738E-01	0.948	cis	Hospitalized COVID-19
17341_89	THIC	Acetyl-CoA acetyltransferase, cytosolic	Q9BWD1	ACAT2	chr6:159762045	Inverse variance weighted	3	0.025	0.036	4.746E-01	0.948	cis	Hospitalized COVID-19
14158_17	Annexin V	Annexin A5	P08758	ANXA5	chr4:121696995	Inverse variance weighted	2	0.101	0.142	4.749E-01	0.948	cis	Hospitalized COVID-19
19367_34	D3D2	Enoyl-CoA delta isomerase 1, mitochondrial	P42126	ECI1	chr16:2252300	Inverse variance weighted	2	0.077	0.108	4.759E-01	0.948	cis	Hospitalized COVID-19
18401_18	ALKB3	Alpha-ketoglutarate-dependent dioxygenase alkB homolog 3	Q96Q83	ALKBH3	chr11:43880811	Inverse variance weighted	3	-0.039	0.055	4.772E-01	0.948	cis	Hospitalized COVID-19
8303_102	MYG1	UPF0160 protein MYG1, mitochondrial	Q9HB07	MYG1	chr12:53299695	Wald ratio	1	0.118	0.167	4.774E-01	0.948	cis	Hospitalized COVID-19
2635_61	Layilin	Layilin	Q6UX15	LAYN	chr11:111540280	Wald ratio	1	-0.132	0.186	4.776E-01	0.948	cis	Hospitalized COVID-19
8814_33	SAPL1	Proactivator polypeptide-like 1	Q6NUJ1	PSAPL1	chr4:7434930	Inverse variance weighted	8	-0.020	0.028	4.776E-01	0.948	cis	Hospitalized COVID-19
18918_86	PDE4A	cAMP-specific 3',5'-cyclic phosphodiesterase 4A	P27815	PDE4A	chr19:10416773	Inverse variance weighted	4	-0.054	0.076	4.784E-01	0.948	cis	Hospitalized COVID-19
13720_95	Proteinase-3	Myeloblastin	P24158	PRTN3	chr19:840999	Inverse variance weighted	7	-0.017	0.025	4.794E-01	0.948	cis	Hospitalized COVID-19
5663_18	PF4V	Platelet factor 4 variant	P10720	PF4V1	chr4:73853296	Inverse variance weighted	2	0.113	0.160	4.798E-01	0.948	cis	Hospitalized COVID-19
12687_2	DECR	2,4-dienoyl-CoA reductase, mitochondrial	Q16698	DECR1	chr8:90001405	Inverse variance weighted	3	-0.138	0.195	4.803E-01	0.948	cis	Hospitalized COVID-19
2750_3	Apo A-I	Apolipoprotein A-I	P02647	APOA1	chr11:116837622	Inverse variance weighted	3	-0.049	0.069	4.805E-01	0.948	cis	Hospitalized COVID-19
13473_55	IP3KA	Inositol-trisphosphate 3-kinase A	P23677	ITPKA	chr15:41493393	Wald ratio	1	0.104	0.147	4.805E-01	0.948	cis	Hospitalized COVID-19
5688_65	CBLN4	Cerebellin-4	Q9NTU7	CBLN4	chr20:56005519	Inverse variance weighted	5	-0.031	0.044	4.809E-01	0.948	cis	Hospitalized COVID-19
3184_25	Coagulation Factor VII	Coagulation factor VII	P08709	F7	chr13:113105788	Inverse variance weighted	7	0.017	0.024	4.813E-01	0.948	cis	Hospitalized COVID-19
3324_51	LY9	T-lymphocyte surface antigen Ly-9	Q9HBG7	LY9	chr1:160796074	Inverse variance weighted	5	0.041	0.059	4.828E-01	0.948	cis	Hospitalized COVID-19
17787_1	Enoyl-CoA hydratase	Enoyl-CoA hydratase, mitochondrial	P30084	ECHS1	chr10:133373354	Wald ratio	1	0.135	0.193	4.829E-01	0.948	cis	Hospitalized COVID-19
18934_50	Tissue transglutaminase	Protein-glutamine gamma-glutamyltransferase 2	P21980	TGM2	chr20:38166578	Wald ratio	1	0.038	0.055	4.833E-01	0.948	cis	Hospitalized COVID-19
8097_77	LIPN	Lipase member N	Q5VXI9	LIPN	chr10:88759982	Inverse variance weighted	6	-0.019	0.028	4.837E-01	0.948	cis	Hospitalized COVID-19
10714_7	ACE	Angiotensin-converting enzyme	P12821	ACE	chr17:63477061	Inverse variance weighted	11	-0.012	0.017	4.838E-01	0.948	cis	Hospitalized COVID-19
3327_27	NET4	Netrin-4	Q9HB63	NTN4	chr12:95791189	Inverse variance weighted	3	0.049	0.071	4.840E-01	0.948	cis	Hospitalized COVID-19
12633_3	PTN9	Tyrosine-protein phosphatase non-receptor type 9	P43378	PTPN9	chr15:75579315	Wald ratio	1	-0.125	0.178	4.840E-01	0.948	cis	Hospitalized COVID-19
9841_197	Multifunctional protein ADE2	Multifunctional protein ADE2	P22234	PAICS	chr4:56435741	Wald ratio	1	0.091	0.131	4.842E-01	0.948	cis	Hospitalized COVID-19
11177_16	K2C5	Keratin, type II cytoskeletal 5	P13647	KRT5	chr12:52520530	Inverse variance weighted	2	-0.105	0.151	4.846E-01	0.948	cis	Hospitalized COVID-19
18218_48	CB032	CB1 cannabinoid receptor-interacting protein 1	Q96F85	CNRIP1	chr2:68320051	Inverse variance weighted	7	0.020	0.028	4.846E-01	0.948	cis	Hospitalized COVID-19
17746_77	FIS1	Mitochondrial fission 1 protein	Q9Y3D6	FIS1	chr7:101252316	Inverse variance weighted	2	0.106	0.152	4.856E-01	0.948	cis	Hospitalized COVID-19
6919_3	HBAZ	Hemoglobin subunit zeta	P02008	HBZ	chr16:142728	Inverse variance weighted	6	-0.015	0.021	4.863E-01	0.948	cis	Hospitalized COVID-19
15308_108	VWC2	Brorin	Q2TAL6	VWC2	chr7:49773638	Inverse variance weighted	7	0.024	0.034	4.863E-01	0.948	cis	Hospitalized COVID-19
5069_9	DAF	Complement decay-accelerating factor	P08174	CD55	chr1:207321519	Inverse variance weighted	4	0.033	0.048	4.865E-01	0.948	cis	Hospitalized COVID-19
19153_53	MPPD2	Metallophosphoesterase MPPED2	Q15777	MPPED2	chr11:30586872	Inverse variance weighted	2	0.068	0.099	4.873E-01	0.948	cis	Hospitalized COVID-19
4276_10	prostatic binding protein	Phosphatidylethanolamine-binding protein 1	P30086	PEBP1	chr12:118136124	Inverse variance weighted	7	-0.022	0.032	4.885E-01	0.948	cis	Hospitalized COVID-19
17224_12	MIME	Mimecan	P20774	OGN	chr9:92404696	Inverse variance weighted	6	0.036	0.052	4.886E-01	0.948	cis	Hospitalized COVID-19
11516_7	FABPL	Fatty acid-binding protein, liver	P07148	FABP1	chr2:88128062	Wald ratio	1	-0.063	0.091	4.889E-01	0.948	cis	Hospitalized COVID-19
5089_11	IL-7 Ra	Interleukin-7 receptor subunit alpha	P16871	IL7R	chr5:35852695	Wald ratio	1	-0.119	0.172	4.890E-01	0.948	cis	Hospitalized COVID-19
5066_134	CLM6	CMRF35-like molecule 6	Q08708	CD300C	chr17:74546115	Inverse variance weighted	4	-0.058	0.083	4.895E-01	0.948	cis	Hospitalized COVID-19
12437_18	ULK3	Serine/threonine-protein kinase ULK3	Q6PHR2	ULK3	chr15:74843346	Wald ratio	1	0.137	0.198	4.895E-01	0.948	cis	Hospitalized COVID-19
10042_8	SGK3	Serine/threonine-protein kinase Sgk3	Q96BR1	SGK3	chr8:66712734	Wald ratio	1	-0.141	0.205	4.912E-01	0.948	cis	Hospitalized COVID-19
3580_25	a1-Antitrypsin	Alpha-1-antitrypsin	P01009	SERPINA1	chr14:94390693	Inverse variance weighted	7	-0.013	0.019	4.912E-01	0.948	cis	Hospitalized COVID-19
13434_172	PARVA	Alpha-parvin	Q9NVD7	PARVA	chr11:12377563	Wald ratio	1	-0.081	0.118	4.913E-01	0.948	cis	Hospitalized COVID-19
13671_40	Elastase	Neutrophil elastase	P08246	ELANE	chr19:851014	Inverse variance weighted	5	-0.030	0.044	4.914E-01	0.948	cis	Hospitalized COVID-19
5400_52	sLeptin R	Leptin receptor, soluble	P48357	LEPR	chr1:65420652	Inverse variance weighted	10	0.012	0.018	4.921E-01	0.948	cis	Hospitalized COVID-19
7244_16	TM149	IGF-like family receptor 1	Q9H665	IGFLR1	chr19:35742453	Inverse variance weighted	4	0.014	0.021	4.929E-01	0.948	cis	Hospitalized COVID-19
6551_94	Vaspin	Serpin A12	Q8IW75	SERPINA12	chr14:94517844	Inverse variance weighted	5	-0.019	0.028	4.931E-01	0.948	cis	Hospitalized COVID-19
2590_69	ROR1	Inactive tyrosine-protein kinase transmembrane receptor ROR1	Q01973	ROR1	chr1:63774017	Inverse variance weighted	3	-0.028	0.040	4.932E-01	0.948	cis	Hospitalized COVID-19
4480_59	C3b	Complement C3b	P01024	C3	chr19:6730562	Wald ratio	1	-0.162	0.238	4.947E-01	0.948	cis	Hospitalized COVID-19
12727_7	FPRP	Prostaglandin F2 receptor negative regulator	Q9P2B2	PTGFRN	chr1:116909916	Inverse variance weighted	8	0.018	0.026	4.955E-01	0.948	cis	Hospitalized COVID-19
5687_5	GLU2B	Glucosidase 2 subunit beta	P14314	PRKCSH	chr19:11435284	Wald ratio	1	-0.128	0.188	4.957E-01	0.948	cis	Hospitalized COVID-19
13597_20	RAB31	Ras-related protein Rab-31	Q13636	RAB31	chr18:9708275	Inverse variance weighted	3	0.054	0.080	4.959E-01	0.948	cis	Hospitalized COVID-19
4908_6	Endoglin	Endoglin	P17813	ENG	chr9:127854658	Inverse variance weighted	3	-0.043	0.063	4.961E-01	0.948	cis	Hospitalized COVID-19
7208_60	MGT4C	Alpha-1,3-mannosyl-glycoprotein 4-beta-N-acetylglucosaminyltransferase C	Q9UBM8	MGAT4C	chr12:86838904	Wald ratio	1	0.114	0.167	4.961E-01	0.948	cis	Hospitalized COVID-19
4775_34	Gelsolin	Gelsolin	P06396	GSN	chr9:121207794	Inverse variance weighted	2	-0.058	0.086	4.969E-01	0.948	cis	Hospitalized COVID-19
5006_71	MK13	Mitogen-activated protein kinase 13	O15264	MAPK13	chr6:36127809	Wald ratio	1	-0.077	0.114	4.970E-01	0.948	cis	Hospitalized COVID-19
3617_80	HGFA	Hepatocyte growth factor activator	Q04756	HGFAC	chr4:3441968	Inverse variance weighted	12	-0.016	0.024	4.973E-01	0.948	cis	Hospitalized COVID-19
4979_34	DERM	Dermatopontin	Q07507	DPT	chr1:168729206	Inverse variance weighted	6	-0.020	0.030	4.974E-01	0.948	cis	Hospitalized COVID-19
10907_116	NTRI	Neurotrimin	Q9P121	NTM	chr11:131370478	Inverse variance weighted	2	0.090	0.132	4.974E-01	0.948	cis	Hospitalized COVID-19
3292_75	CD48	CD48 antigen	P09326	CD48	chr1:160711831	Inverse variance weighted	4	0.037	0.055	4.976E-01	0.948	cis	Hospitalized COVID-19
3195_50	Granulysin	Granulysin	P22749	GNLY	chr2:85685175	Inverse variance weighted	7	-0.020	0.029	4.977E-01	0.948	cis	Hospitalized COVID-19
3710_49	Angiostatin	Angiostatin	P00747	PLG	chr6:160702194	Inverse variance weighted	7	0.038	0.056	4.981E-01	0.948	cis	Hospitalized COVID-19
13242_134	SHAN3	SH3 and multiple ankyrin repeat domains protein 3	Q9BYB0	SHANK3	chr22:50674415	Inverse variance weighted	5	-0.036	0.054	4.984E-01	0.948	cis	Hospitalized COVID-19
6393_63	Endoplasmin	Endoplasmin	P14625	HSP90B1	chr12:103930107	Inverse variance weighted	6	-0.011	0.016	4.993E-01	0.948	cis	Hospitalized COVID-19
5121_3	SEM6B	Semaphorin-6B	Q9H3T3	SEMA6B	chr19:4581776	Inverse variance weighted	2	0.112	0.166	4.997E-01	0.948	cis	Hospitalized COVID-19
9321_400	NMB	Neuromedin-B	P08949	NMB	chr15:84658563	Inverse variance weighted	2	-0.043	0.064	5.000E-01	0.948	cis	Hospitalized COVID-19
10672_75	SP-B	Pulmonary surfactant-associated protein B	P07988	SFTPB	chr2:85668741	Inverse variance weighted	4	0.055	0.082	5.002E-01	0.948	cis	Hospitalized COVID-19
5648_28	CTRB2	Chymotrypsinogen B2	Q6GPI1	CTRB2	chr16:75207161	Inverse variance weighted	8	-0.009	0.014	5.003E-01	0.948	cis	Hospitalized COVID-19
9234_8	TWSG1	Twisted gastrulation protein homolog 1	Q9GZX9	TWSG1	chr18:9334767	Inverse variance weighted	3	-0.055	0.081	5.010E-01	0.948	cis	Hospitalized COVID-19
12436_84	GST omega-1	Glutathione S-transferase omega-1	P78417	GSTO1	chr10:104235356	Inverse variance weighted	9	-0.014	0.021	5.029E-01	0.948	cis	Hospitalized COVID-19
7928_183	TPST1	Protein-tyrosine sulfotransferase 1	O60507	TPST1	chr7:66205317	Inverse variance weighted	2	0.027	0.040	5.029E-01	0.948	cis	Hospitalized COVID-19
17748_21	QORX	Quinone oxidoreductase PIG3	Q53FA7	TP53I3	chr2:24085861	Inverse variance weighted	5	-0.030	0.045	5.038E-01	0.948	cis	Hospitalized COVID-19
17737_7	IVD	Isovaleryl-CoA dehydrogenase, mitochondrial	P26440	IVD	chr15:40405485	Inverse variance weighted	2	0.048	0.073	5.041E-01	0.948	cis	Hospitalized COVID-19
2658_27	TrkC	NT-3 growth factor receptor	Q16288	NTRK3	chr15:88256791	Inverse variance weighted	5	-0.029	0.044	5.042E-01	0.948	cis	Hospitalized COVID-19

5646_20	RNAS6	Ribonuclease K6	Q93091	RNASE6	chr14:20781268	Inverse variance weighted	8	0.013	0.020	5.050E-01	0.948	cis	Hospitalized COVID-19
19558_10	LRP4	Low-density lipoprotein receptor-related protein 4	O75096	LRP4	chr11:46918642	Inverse variance weighted	6	0.033	0.050	5.052E-01	0.948	cis	Hospitalized COVID-19
3905_62	UBE2N	Ubiquitin-conjugating enzyme E2 N	P61088	UBE2N	chr12:93441947	Wald ratio	1	-0.150	0.226	5.062E-01	0.948	cis	Hospitalized COVID-19
11356_19	DGC14	Protein DGCR14	Q96DF8	ESS2	chr22:19144684	Wald ratio	1	0.065	0.098	5.072E-01	0.948	cis	Hospitalized COVID-19
6521_35	NPTX2	Neuronal pentraxin-2	P47972	NPTX2	chr7:98617285	Inverse variance weighted	2	-0.062	0.094	5.091E-01	0.948	cis	Hospitalized COVID-19
8376_25	LSHB	Lutropin subunit beta	P01229	LHB	chr19:49017091	Inverse variance weighted	4	0.033	0.050	5.093E-01	0.948	cis	Hospitalized COVID-19
17410_5	CETN3	Centrin-3	O15182	CETN3	chr5:90409766	Wald ratio	1	-0.096	0.146	5.095E-01	0.948	cis	Hospitalized COVID-19
18422_41	ERG19	Diphosphomevalonate decarboxylase	P53602	MVD	chr16:88663161	Inverse variance weighted	2	0.094	0.142	5.102E-01	0.948	cis	Hospitalized COVID-19
19556_12	Complement receptor type 1	Complement receptor type 1	P17927	CR1	chr1:207496147	Inverse variance weighted	10	-0.026	0.039	5.103E-01	0.948	cis	Hospitalized COVID-19
4964_67	ARTS1	Endoplasmic reticulum aminopeptidase 1	Q9NZ08	ERAP1	chr5:96808100	Inverse variance weighted	5	-0.014	0.021	5.115E-01	0.948	cis	Hospitalized COVID-19
2212_69	tPA	Tissue-type plasminogen activator	P00750	PLAT	chr8:42207709	Inverse variance weighted	4	0.037	0.056	5.116E-01	0.948	cis	Hospitalized COVID-19
10708_3	GON2	Progonadoliberin-2	O43555	GNRH2	chr20:3043622	Wald ratio	1	-0.065	0.099	5.122E-01	0.948	cis	Hospitalized COVID-19
17758_79	DCXR	L-xylulose reductase	Q7Z4W1	DCXR	chr17:82037709	Inverse variance weighted	2	0.070	0.106	5.127E-01	0.948	cis	Hospitalized COVID-19
14106_46	GDP/GTP exchange protein	Rap1 GTPase-GDP dissociation stimulator 1	P52306	RAP1GDS1	chr4:98261384	Wald ratio	1	-0.132	0.202	5.136E-01	0.948	cis	Hospitalized COVID-19
17697_2	OVCA2	Esterase OVCA2	Q8WZ82	OVCA2	chr17:2042022	Wald ratio	1	-0.073	0.113	5.160E-01	0.948	cis	Hospitalized COVID-19
16890_37	ATL1	ADAMTS-like protein 1	Q8N6G6	ADAMTSL1	chr9:17906563	Inverse variance weighted	6	0.030	0.047	5.163E-01	0.948	cis	Hospitalized COVID-19
8778_3	Noggin	Noggin	Q13253	NOG	chr17:56593699	Inverse variance weighted	5	0.034	0.052	5.171E-01	0.948	cis	Hospitalized COVID-19
2579_17	MMP-9	Matrix metalloproteinase-9	P14780	MMP9	chr20:46008908	Wald ratio	1	-0.087	0.135	5.186E-01	0.948	cis	Hospitalized COVID-19
2816_50	BCAM	Basal Cell Adhesion Molecule	P50895	BCAM	chr19:44809071	Inverse variance weighted	5	0.026	0.041	5.188E-01	0.948	cis	Hospitalized COVID-19
3196_6	HPLN1	Hyaluronan and proteoglycan link protein 1	P10915	HAPLN1	chr5:83720855	Inverse variance weighted	3	-0.049	0.076	5.198E-01	0.948	cis	Hospitalized COVID-19
9525_1	PTK7	Inactive tyrosine-protein kinase 7	Q13308	PTK7	chr6:43076307	Inverse variance weighted	2	0.165	0.257	5.199E-01	0.948	cis	Hospitalized COVID-19
9185_15	TFF1	Trefoil factor 1	P04155	TFF1	chr21:42366535	Inverse variance weighted	3	0.024	0.037	5.208E-01	0.948	cis	Hospitalized COVID-19
8299_66	LIRA4	Leukocyte immunoglobulin-like receptor subfamily A member 4	P59901	LILRA4	chr19:54339162	Inverse variance weighted	3	-0.035	0.055	5.211E-01	0.948	cis	Hospitalized COVID-19
16599_38	GPN1	GPN-loop GTPase 1	Q9HCN4	GPN1	chr2:27628247	Wald ratio	1	0.085	0.133	5.212E-01	0.948	cis	Hospitalized COVID-19
11347_9	Transaldolase	Transaldolase	P37837	TALDO1	chr11:747415	Inverse variance weighted	2	-0.064	0.099	5.215E-01	0.948	cis	Hospitalized COVID-19
4133_54	Granzyme B	Granzyme B	P10144	GZMB	chr14:24634267	Wald ratio	1	0.071	0.110	5.221E-01	0.948	cis	Hospitalized COVID-19
9092_33	ANGL1:N-term	Angiopoietin-related protein 1:N-term	O95841	ANGPTL1	chr1:178871077	Wald ratio	1	0.125	0.195	5.225E-01	0.948	cis	Hospitalized COVID-19
13450_49	UBP8	Ubiquitin carboxyl-terminal hydrolase 8	P40818	USP8	chr15:50424380	Wald ratio	1	0.080	0.125	5.226E-01	0.948	cis	Hospitalized COVID-19
9838_4	SMAD1	Mothers against decapentaplegic homolog 1	Q15797	SMAD1	chr4:145481194	Wald ratio	1	-0.132	0.206	5.232E-01	0.948	cis	Hospitalized COVID-19
15540_6	Vimentin	Vimentin	P08670	VIM	chr10:17228241	Inverse variance weighted	2	0.079	0.125	5.245E-01	0.948	cis	Hospitalized COVID-19
15620_4	NLGN1	Neuroligin-1	Q8N2Q7	NLGN1	chr3:173396284	Inverse variance weighted	3	0.097	0.153	5.253E-01	0.948	cis	Hospitalized COVID-19
8773_172	EMIL3:region 1	EMILIN-3:region 1	Q9NT22	EMILIN3	chr20:41366818	Inverse variance weighted	6	-0.039	0.061	5.254E-01	0.948	cis	Hospitalized COVID-19
13632_10	Zyxin	Zyxin	Q15942	ZYX	chr7:143381295	Wald ratio	1	-0.143	0.225	5.257E-01	0.948	cis	Hospitalized COVID-19
9986_14	Neuropeptide W	Neuropeptide W	Q8N729	NPW	chr16:2009926	Inverse variance weighted	3	-0.027	0.042	5.258E-01	0.948	cis	Hospitalized COVID-19
3481_87	XPNPEP1	Xaa-Pro aminopeptidase 1	Q9NQW7	XPNPEP1	chr10:109923553	Wald ratio	1	0.102	0.160	5.258E-01	0.948	cis	Hospitalized COVID-19
10603_1	HIS3	Histatin-3	P15516	HTN3	chr4:70028455	Inverse variance weighted	3	-0.051	0.081	5.258E-01	0.948	cis	Hospitalized COVID-19
19115_13	CMBL	Carboxymethylenebutenolidase homolog	Q96DG6	CMBL	chr5:10307902	Wald ratio	1	0.042	0.067	5.258E-01	0.948	cis	Hospitalized COVID-19
8890_9	T132B	Transmembrane protein 132B	Q14DG7	TMEM132B	chr12:125186386	Inverse variance weighted	2	0.040	0.064	5.265E-01	0.948	cis	Hospitalized COVID-19
6383_90	TLL1	Tolloid-like protein 1	O43897	TLL1	chr4:165873237	Inverse variance weighted	2	-0.129	0.203	5.267E-01	0.948	cis	Hospitalized COVID-19
8327_26	DPEP2	Dipeptidase 2	Q9H4A9	DPEP2	chr16:68000586	Inverse variance weighted	2	0.066	0.104	5.270E-01	0.948	cis	Hospitalized COVID-19
5355_69	LIGHT	Tumor necrosis factor ligand superfamily member 14	O43557	TNFSF14	chr19:6670588	Wald ratio	1	-0.064	0.100	5.273E-01	0.948	cis	Hospitalized COVID-19
4332_6	CLC1B	C-type lectin domain family 1 member B	Q9P126	CLEC1B	chr12:10013424	Wald ratio	1	-0.091	0.143	5.274E-01	0.948	cis	Hospitalized COVID-19
13931_22	PSMD9	26S proteasome non-ATPase regulatory subunit 9	O00233	PSMD9	chr12:121888732	Wald ratio	1	0.052	0.082	5.281E-01	0.948	cis	Hospitalized COVID-19
12486_8	GLRX2	Glutaredoxin-2, mitochondrial	Q9NS18	GLRX2	chr1:193106114	Inverse variance weighted	3	0.033	0.053	5.297E-01	0.948	cis	Hospitalized COVID-19
16074_12	GRB2-related adapter protein 2	GRB2-related adapter protein 2	O75791	GRAP2	chr22:39901084	Wald ratio	1	-0.202	0.322	5.299E-01	0.948	cis	Hospitalized COVID-19
9267_2	CBPA4	Carboxypeptidase A4	Q9UI42	CPA4	chr7:130293134	Inverse variance weighted	12	-0.008	0.012	5.299E-01	0.948	cis	Hospitalized COVID-19
13481_24	TCEA2	Transcription elongation factor A protein 2	Q15560	TCEA2	chr20:64049836	Wald ratio	1	0.044	0.071	5.300E-01	0.948	cis	Hospitalized COVID-19
6077_63	CECR1	Adenosine deaminase CECR1	Q9NZK5	ADA2	chr22:17258235	Inverse variance weighted	5	0.016	0.025	5.303E-01	0.948	cis	Hospitalized COVID-19
15514_26	Pseudocholinesterase	Cholinesterase	P06276	BCHE	chr3:165837462	Inverse variance weighted	6	0.016	0.026	5.303E-01	0.948	cis	Hospitalized COVID-19
17686_27	TBCB	Tubulin-folding cofactor B	Q99426	TBCB	chr19:36114289	Wald ratio	1	0.104	0.166	5.309E-01	0.948	cis	Hospitalized COVID-19
5620_13	AMD	Peptidyl-glycine alpha-amidating monooxygenase	P19021	PAM	chr5:102753981	Inverse variance weighted	10	-0.012	0.020	5.317E-01	0.948	cis	Hospitalized COVID-19
13642_90	CIG49	Interferon-induced protein with tetratricopeptide repeats 3	O14879	IFIT3	chr10:89327307	Wald ratio	1	0.076	0.122	5.319E-01	0.948	cis	Hospitalized COVID-19
12573_80	TRIM3	Tripartite motif-containing protein 3	O75382	TRIM3	chr11:6474459	Wald ratio	1	0.064	0.103	5.332E-01	0.948	cis	Hospitalized COVID-19
11265_8	Retinal dehydrogenase 1	Retinal dehydrogenase 1	P00352	ALDH1A1	chr9:73080442	Wald ratio	1	-0.073	0.118	5.338E-01	0.948	cis	Hospitalized COVID-19
4771_10	ASM3A	Acid sphingomyelinase-like phosphodiesterase 3a	Q92484	SMPDL3A	chr6:122789049	Inverse variance weighted	3	0.028	0.045	5.341E-01	0.948	cis	Hospitalized COVID-19
3298_52	Contactin-4	Contactin-4	Q8IWV2	CNTN4	chr3:2098813	Inverse variance weighted	8	0.018	0.029	5.346E-01	0.948	cis	Hospitalized COVID-19
5593_11	PDIA5	Protein disulfide-isomerase A5	Q14554	PDIA5	chr3:123067025	Inverse variance weighted	8	-0.012	0.019	5.347E-01	0.948	cis	Hospitalized COVID-19
4152_58	Prekallikrein	Plasma kallikrein	P03952	KLKB1	chr4:186226438	Inverse variance weighted	4	0.027	0.043	5.355E-01	0.948	cis	Hospitalized COVID-19
12684_5	ADSV	Adseverin	Q9Y6U3	SCIN	chr7:12570577	Wald ratio	1	-0.080	0.130	5.357E-01	0.948	cis	Hospitalized COVID-19
9212_22	CATF	Cathepsin F	Q9UBX1	CTSF	chr11:66568879	Inverse variance weighted	2	-0.039	0.063	5.362E-01	0.948	cis	Hospitalized COVID-19
2652_15	suPAR	Urokinase plasminogen activator surface receptor	Q03405	PLAUR	chr19:43670547	Inverse variance weighted	2	-0.083	0.134	5.363E-01	0.948	cis	Hospitalized COVID-19
5103_30	MO2R1:ECD	Cell surface glycoprotein CD200 receptor 1:Isoform 4, Extracellular Domain	Q8TD46	CD200R1	chr3:112975103	Wald ratio	1	0.057	0.092	5.363E-01	0.948	cis	Hospitalized COVID-19
15556_49	Alpha-amylase 2B	Alpha-amylase 2B	P19961	AMY2B	chr1:103553815	Inverse variance weighted	4	0.073	0.118	5.371E-01	0.948	cis	Hospitalized COVID-19
2979_8	ENA-78	C-X-C motif chemokine 5	P42830	CXCL5	chr4:73998677	Wald ratio	1	-0.176	0.286	5.376E-01	0.948	cis	Hospitalized COVID-19
5465_32	H6ST1	Heparan-sulfate 6-O-sulfotransferase 1	O60243	HS6ST1	chr2:128318868	Inverse variance weighted	2	0.039	0.063	5.383E-01	0.948	cis	Hospitalized COVID-19
5601_2	PGRP-L	N-acetylmuramoyl-L-alanine amidase	Q96PD5	PGLYRP2	chr19:15498956	Inverse variance weighted	5	-0.024	0.038	5.389E-01	0.948	cis	Hospitalized COVID-19
3825_18	MK08	Mitogen-activated protein kinase 8	P45983	MAPK8	chr10:48306639	Wald ratio	1	-0.112	0.183	5.399E-01	0.948	cis	Hospitalized COVID-19
18435_40	UBX2B	UBX domain-containing protein 2B	Q14CS0	UBXN2B	chr8:58411359	Wald ratio	1	-0.106	0.174	5.404E-01	0.948	cis	Hospitalized COVID-19
8231_122	VEGF sR1	Vascular endothelial growth factor receptor 1	P17948	FLT1	chr13:28495145	Wald ratio	1	0.120	0.196	5.405E-01	0.948	cis	Hospitalized COVID-19
15506_34	LRP12	Low-density lipoprotein receptor-related protein 12	Q9Y561	LRP12	chr8:104589258	Inverse variance weighted	2	0.100	0.163	5.407E-01	0.948	cis	Hospitalized COVID-19
16035_8	VEGF sR3	Vascular endothelial growth factor receptor 3	P35916	FLT4	chr5:180649624	Inverse variance weighted	3	0.045	0.074	5.412E-01	0.948	cis	Hospitalized COVID-19
15476_6	REG3G	Regenerating islet-derived protein 3-gamma	Q6UW15	REG3G	chr2:79025686	Inverse variance weighted	6	0.016	0.026	5.419E-01	0.948	cis	Hospitalized COVID-19
5837_49	LIF sR	Leukemia inhibitory factor receptor	P42702	LIFR	chr5:38608354	Inverse variance weighted	4	0.038	0.062	5.421E-01	0.948	cis	Hospitalized COVID-19
8245_27	sICAM-5	Intercellular adhesion molecule 5	Q9UMF0	ICAM5	chr19:10289952	Inverse variance weighted	3	0.068	0.111	5.424E-01	0.948	cis	Hospitalized COVID-19
14079_14	IL-18 Ra	Interleukin-18 receptor 1	Q13478	IL18R1	chr2:102311529	Inverse variance weighted	5	-0.038	0.062	5.431E-01	0.948	cis	Hospitalized COVID-19
2278_61	TIMP-2	Metalloproteinase inhibitor 2	P16035	TIMP2	chr17:78925387	Wald ratio	1	-0.089	0.147	5.433E-01	0.948	cis	Hospitalized COVID-19
18864_7	TRY3	Trypsin-3	P35030	PRSS3	chr9:33750679	Inverse variance weighted	4	0.016	0.026	5.439E-01	0.948	cis	Hospitalized COVID-19
3799_11	Carbonic anhydrase III	Carbonic anhydrase 3	P07451	CA3	chr8:85373436	Inverse variance weighted	4	0.051	0.084	5.457E-01	0.948	cis	Hospitalized COVID-19

11161_5	SPG20	Spartin	Q8N0X7	SPART	chr13:36370180	Wald ratio	1	0.132	0.219	5.467E-01	0.948	cis	Hospitalized COVID-19
3459_49	PDGF Rb	Platelet-derived growth factor receptor beta	P09619	PDGFRB	chr5:150155872	Inverse variance weighted	11	-0.007	0.011	5.476E-01	0.948	cis	Hospitalized COVID-19
2944_66	DAN	Neuroblastoma suppressor of tumorigenicity 1	P41271	NBL1	chr1:19596979	Wald ratio	1	0.128	0.212	5.482E-01	0.948	cis	Hospitalized COVID-19
2644_11	PKC-A	Protein kinase C alpha type	P17252	PRKCA	chr17:66302613	Wald ratio	1	-0.139	0.232	5.488E-01	0.948	cis	Hospitalized COVID-19
16918_198	TLR3	Toll-like receptor 3	O15455	TLR3	chr4:186068911	Inverse variance weighted	8	0.015	0.025	5.492E-01	0.948	cis	Hospitalized COVID-19
15375_49	Carboxypeptidase B1	Carboxypeptidase B	P15086	CPB1	chr3:148791102	Inverse variance weighted	2	0.028	0.048	5.512E-01	0.948	cis	Hospitalized COVID-19
13118_5	SMOC1	SPARC-related modular calcium-binding protein 1	Q9H4F8	SMOC1	chr14:69854131	Inverse variance weighted	3	0.030	0.050	5.515E-01	0.948	cis	Hospitalized COVID-19
17435_43	ETFA	Electron transfer flavoprotein subunit alpha, mitochondrial	P13804	ETFA	chr15:76311730	Wald ratio	1	-0.118	0.198	5.517E-01	0.948	cis	Hospitalized COVID-19
10974_20	ISK7	Serine protease inhibitor Kazal-type 7	P58062	SPINK7	chr5:148312419	Wald ratio	1	-0.052	0.087	5.517E-01	0.948	cis	Hospitalized COVID-19
11208_15	NAGPA	N-acetylglucosamine-1-phosphodiester alpha-N-acetylglucosaminidase	Q9UK23	NAGPA	chr16:5034141	Inverse variance weighted	7	0.015	0.025	5.534E-01	0.948	cis	Hospitalized COVID-19
11219_95	FGFP3	Fibroblast growth factor-binding protein 3	Q8TAT2	FGFBP3	chr10:91909486	Inverse variance weighted	3	0.025	0.041	5.535E-01	0.948	cis	Hospitalized COVID-19
5463_22	GAS1	Growth arrest-specific protein 1	P54826	GAS1	chr9:86947506	Inverse variance weighted	2	0.054	0.091	5.549E-01	0.948	cis	Hospitalized COVID-19
5112_73	OX2G	OX-2 membrane glycoprotein	P41217	CD200	chr3:112332347	Wald ratio	1	-0.076	0.129	5.554E-01	0.948	cis	Hospitalized COVID-19
5708_1	LEAP2	Liver-expressed antimicrobial peptide 2	Q969E1	LEAP2	chr5:132873444	Inverse variance weighted	5	0.018	0.031	5.557E-01	0.948	cis	Hospitalized COVID-19
12422_143	LX15B	Arachidonate 15-lipoxygenase B	O15296	ALOX15B	chr17:8039034	Wald ratio	1	0.083	0.140	5.560E-01	0.948	cis	Hospitalized COVID-19
3311_27	FCG3B	Low affinity immunoglobulin gamma Fc region receptor III-B	O75015	FCGR3B	chr1:161631963	Wald ratio	1	-0.133	0.226	5.564E-01	0.948	cis	Hospitalized COVID-19
11377_19	ADH7	Alcohol dehydrogenase class 4 mu/sigma chain	P40394	ADH7	chr4:99435510	Inverse variance weighted	3	-0.021	0.035	5.568E-01	0.948	cis	Hospitalized COVID-19
17777_31	SDSL	Serine dehydratase-like	Q96GA7	SDSL	chr12:113422380	Inverse variance weighted	6	0.022	0.037	5.569E-01	0.948	cis	Hospitalized COVID-19
5618_50	FAM3B	Protein FAM3B	P58499	FAM3B	chr21:41304212	Wald ratio	1	0.080	0.136	5.578E-01	0.948	cis	Hospitalized COVID-19
2474_54	SAP	Serum amyloid P-component	P02743	APCS	chr1:159587826	Inverse variance weighted	3	0.030	0.051	5.587E-01	0.948	cis	Hospitalized COVID-19
9360_33	EDIL3	EGF-like repeat and discoidin I-like domain-containing protein 3	O43854	EDIL3	chr5:84384880	Inverse variance weighted	2	0.075	0.127	5.588E-01	0.948	cis	Hospitalized COVID-19
3305_6	DLL4	Delta-like protein 4	Q9NR61	DLL4	chr15:40929340	Wald ratio	1	0.114	0.195	5.609E-01	0.948	cis	Hospitalized COVID-19
13122_19	FLRT2	Leucine-rich repeat transmembrane protein FLRT2	O43155	FLRT2	chr14:85530144	Inverse variance weighted	5	0.021	0.035	5.619E-01	0.948	cis	Hospitalized COVID-19
13109_82	NEGR1	Neuronal growth regulator 1	Q7Z3B1	NEGR1	chr1:72282539	Wald ratio	1	-0.093	0.160	5.621E-01	0.948	cis	Hospitalized COVID-19
3175_51	ATS13	A disintegrin and metalloproteinase with thrombospondin motifs 13	Q76LX8	ADAMTS13	chr9:133414358	Inverse variance weighted	7	-0.013	0.023	5.623E-01	0.948	cis	Hospitalized COVID-19
13068_139	CCS	Copper chaperone for superoxide dismutase	O14618	CCS	chr11:66593153	Wald ratio	1	0.049	0.084	5.638E-01	0.948	cis	Hospitalized COVID-19
10451_11	NUCB1	Nucleobindin-1	Q02818	NUCB1	chr19:48900050	Inverse variance weighted	3	-0.033	0.057	5.639E-01	0.948	cis	Hospitalized COVID-19
9015_1	PRG3	Proteoglycan 3	Q9Y2Y8	PRG3	chr11:57381150	Wald ratio	1	-0.165	0.286	5.640E-01	0.948	cis	Hospitalized COVID-19
3332_57	RGM-C	Hemojuvelin	Q6ZVN8	HJV	chr1:146036746	Wald ratio	1	-0.096	0.167	5.643E-01	0.948	cis	Hospitalized COVID-19
2946_52	Factor D	Complement factor D	P00746	CFD	chr19:859453	Inverse variance weighted	6	0.028	0.048	5.649E-01	0.948	cis	Hospitalized COVID-19
17370_186	ABHDA	Mycophenolic acid acyl-glucuronide esterase, mitochondrial	Q9NUJ1	ABHD10	chr3:111979010	Wald ratio	1	-0.114	0.198	5.649E-01	0.948	cis	Hospitalized COVID-19
16307_22	UNC5H4	Netrin receptor UNC5D	Q6UXZ4	UNC5D	chr8:35235475	Inverse variance weighted	3	0.043	0.075	5.650E-01	0.948	cis	Hospitalized COVID-19
7997_118	DOC2B	Double C2-like domain-containing protein beta	Q14184	DOC2B	chr17:181650	Wald ratio	1	0.139	0.241	5.655E-01	0.948	cis	Hospitalized COVID-19
17782_23	THIK	3-ketoacyl-CoA thiolase, peroxisomal	P09110	ACAA1	chr3:38137242	Inverse variance weighted	5	0.025	0.043	5.664E-01	0.948	cis	Hospitalized COVID-19
18386_36	GLRX1	Glutaredoxin-1	P35754	GLRX	chr5:95822726	Wald ratio	1	-0.108	0.189	5.665E-01	0.948	cis	Hospitalized COVID-19
10940_25	SRCA	Sarcalumenin	Q86TD4	SRL	chr16:4242080	Wald ratio	1	0.088	0.153	5.665E-01	0.948	cis	Hospitalized COVID-19
13123_3	FLRT3:ECD	Leucine-rich repeat transmembrane protein FLRT3:Extracellular domain	Q9NZU0	FLRT3	chr20:14337614	Inverse variance weighted	6	-0.010	0.018	5.669E-01	0.948	cis	Hospitalized COVID-19
15447_45	Sorbitol dehydrogenase	Sorbitol dehydrogenase	Q00796	SORD	chr15:45023147	Inverse variance weighted	4	0.027	0.047	5.671E-01	0.948	cis	Hospitalized COVID-19
3622_33	LGMN	Legumain	Q99538	LGMN	chr14:92748679	Inverse variance weighted	3	0.036	0.064	5.674E-01	0.948	cis	Hospitalized COVID-19
19136_22	MMSA	Methylmalonate-semialdehyde dehydrogenase [acylating], mitochondrial	Q02252	ALDH6A1	chr14:74084492	Inverse variance weighted	2	0.074	0.130	5.683E-01	0.948	cis	Hospitalized COVID-19
18398_1	AK1D1	3-oxo-5-beta-steroid 4-dehydrogenase	P51857	AKR1D1	chr7:138002324	Wald ratio	1	-0.120	0.211	5.690E-01	0.948	cis	Hospitalized COVID-19
11098_1	PDXK	Pyridoxal kinase	O00764	PDXK	chr21:43719094	Inverse variance weighted	6	-0.035	0.061	5.698E-01	0.948	cis	Hospitalized COVID-19
19347_37	Carbonic Anhydrase XII	Carbonic anhydrase 12	O43570	CA12	chr15:63381846	Wald ratio	1	0.135	0.238	5.703E-01	0.948	cis	Hospitalized COVID-19
7050_5	NEGR1	Neuronal growth regulator 1	Q7Z3B1	NEGR1	chr1:72282539	Wald ratio	1	0.130	0.230	5.709E-01	0.948	cis	Hospitalized COVID-19
3283_21	BGH3	Transforming growth factor-beta-induced protein ig-h3	Q15582	TGFBI	chr5:136028988	Inverse variance weighted	5	-0.016	0.027	5.712E-01	0.948	cis	Hospitalized COVID-19
18397_5	AK1C4	Aldo-keto reductase family 1 member C4	P17516	AKR1C4	chr10:5195462	Inverse variance weighted	8	0.018	0.032	5.721E-01	0.948	cis	Hospitalized COVID-19
3495_15	GCP-2	C-X-C motif chemokine 6	P80162	CXCL6	chr4:73836640	Inverse variance weighted	6	-0.034	0.061	5.725E-01	0.948	cis	Hospitalized COVID-19
3310_62	FCG2B	Low affinity immunoglobulin gamma Fc region receptor II-b	P31994	FCGR2B	chr1:161663143	Inverse variance weighted	12	0.026	0.046	5.725E-01	0.948	cis	Hospitalized COVID-19
4437_56	ENTP5	Ectonucleoside triphosphate diphosphohydrolase 5	O75356	ENTPD5	chr14:74019399	Inverse variance weighted	8	-0.014	0.025	5.736E-01	0.948	cis	Hospitalized COVID-19
6472_40	GP100	Melanocyte protein PMEL	P40967	PMEL	chr12:55973317	Inverse variance weighted	2	0.027	0.048	5.741E-01	0.948	cis	Hospitalized COVID-19
15565_102	CA125	Mucin-16	Q8WXI7	MUC16	chr19:8981342	Inverse variance weighted	2	-0.041	0.074	5.746E-01	0.948	cis	Hospitalized COVID-19
3519_3	TARC	C-C motif chemokine 17	Q92583	CCL17	chr16:57404767	Inverse variance weighted	2	-0.031	0.055	5.749E-01	0.948	cis	Hospitalized COVID-19
9231_23	IMPA3	Inositol monophosphatase 3	Q9NX62	BPNT2	chr8:56993867	Inverse variance weighted	6	0.016	0.029	5.751E-01	0.948	cis	Hospitalized COVID-19
3396_54	Renin	Renin	P00797	REN	chr1:204190324	Inverse variance weighted	3	-0.065	0.116	5.766E-01	0.948	cis	Hospitalized COVID-19
5727_35	B3GLT	Beta-1,3-glucosyltransferase	Q6Y288	B3GLCT	chr13:31199975	Inverse variance weighted	2	0.029	0.053	5.768E-01	0.948	cis	Hospitalized COVID-19
8874_53	CLN5:LD	Ceroid-lipofuscinosis neuronal protein 5:Lumenal domain	O75503	CLN5	chr13:76990660	Wald ratio	1	-0.059	0.106	5.769E-01	0.948	cis	Hospitalized COVID-19
12399_194	CCD50	Coiled-coil domain-containing protein 50	Q8IVM0	CCDC50	chr3:191329085	Wald ratio	1	-0.118	0.212	5.770E-01	0.948	cis	Hospitalized COVID-19
9017_58	LPH	Lactase-phlorizin hydrolase	P09848	LCT	chr2:135837184	Inverse variance weighted	4	-0.013	0.024	5.777E-01	0.948	cis	Hospitalized COVID-19
3235_50	WFKN2	WAP, Kazal, immunoglobulin, Kunitz and NTR domain-containing protein 2	Q8TEU8	WFIKKN2	chr17:50834650	Inverse variance weighted	7	-0.015	0.027	5.787E-01	0.948	cis	Hospitalized COVID-19
18315_38	RTP4	Receptor-transporting protein 4	Q96DX8	RTP4	chr3:187368385	Inverse variance weighted	3	0.017	0.030	5.798E-01	0.948	cis	Hospitalized COVID-19
5036_50	TSG-6	Tumor necrosis factor-inducible gene 6 protein	P98066	TNFAIP6	chr2:151357592	Inverse variance weighted	9	-0.013	0.023	5.799E-01	0.948	cis	Hospitalized COVID-19
3484_60	Angiotensinogen	Angiotensinogen	P01019	AGT	chr1:230745576	Inverse variance weighted	2	-0.039	0.071	5.815E-01	0.948	cis	Hospitalized COVID-19
8909_77	GNPI1	Glucosamine-6-phosphate isomerase 1	P46926	GNPDA1	chr5:142013041	Wald ratio	1	0.045	0.081	5.816E-01	0.948	cis	Hospitalized COVID-19
19129_15	MTHFSD	Methenyltetrahydrofolate synthase domain-containing protein	Q2M296	MTHFSD	chr16:86555235	Inverse variance weighted	4	-0.026	0.047	5.826E-01	0.948	cis	Hospitalized COVID-19
6496_60	DLK1:ECD	Protein delta homolog 1:Extracellular domain	P80370	DLK1	chr14:100725705	Inverse variance weighted	2	0.148	0.269	5.827E-01	0.948	cis	Hospitalized COVID-19
2687_2	MIA	Melanoma-derived growth regulatory protein	Q16674	MIA	chr19:40771648	Inverse variance weighted	7	-0.015	0.027	5.833E-01	0.948	cis	Hospitalized COVID-19
6580_29	Pregnancy zone protein	Pregnancy zone protein	P20742	PZP	chr12:9208395	Inverse variance weighted	2	0.045	0.082	5.842E-01	0.948	cis	Hospitalized COVID-19
5475_10	PKC-B-II	Protein kinase C beta type (splice variant beta-II)	P05771	PRKCB	chr16:23835983	Wald ratio	1	-0.074	0.135	5.846E-01	0.948	cis	Hospitalized COVID-19
11480_1	Aldehyde dehydrogenase, class 3	Aldehyde dehydrogenase, dimeric NADP-preferring	P30838	ALDH3A1	chr17:19748943	Wald ratio	1	-0.047	0.085	5.852E-01	0.948	cis	Hospitalized COVID-19
3420_21	Carbonic anhydrase XIII	Carbonic anhydrase 13	Q8N1Q1	CA13	chr8:85220587	Inverse variance weighted	3	-0.018	0.033	5.860E-01	0.948	cis	Hospitalized COVID-19
4145_58	Neurotrophin-3	Neurotrophin-3	P20783	NTF3	chr12:5432108	Inverse variance weighted	2	-0.158	0.291	5.861E-01	0.948	cis	Hospitalized COVID-19
15368_3	BMPER	BMP-binding endothelial regulator protein	Q8N8U9	BMPER	chr7:33904308	Inverse variance weighted	3	-0.037	0.068	5.869E-01	0.948	cis	Hospitalized COVID-19
18222_34	SHLB2	Endophilin-B2	Q9NR46	SH3GLB2	chr9:129028331	Inverse variance weighted	3	0.017	0.031	5.870E-01	0.948	cis	Hospitalized COVID-19
6402_8	PILRA isoform FDF03-deltaTM	Paired immunoglobulin-like type 2 receptor alpha isoform FDF03-deltaTM	Q9UKJ1	PILRA	chr7:100367530	Inverse variance weighted	2	0.019	0.035	5.871E-01	0.948	cis	Hospitalized COVID-19
9816_37	ISOC1	Isochorismatase domain-containing protein 1	Q96CN7	ISOC1	chr5:129094749	Inverse variance weighted	2	-0.032	0.059	5.873E-01	0.948	cis	Hospitalized COVID-19
3890_8	LDH-H 1	L-lactate dehydrogenase B chain	P07195	LDHB	chr12:21757857	Wald ratio	1	0.153	0.282	5.874E-01	0.948	cis	Hospitalized COVID-19
7628_40	CREL1	Cysteine-rich with EGF-like domain protein 1	Q96HD1	CRELD1	chr3:9933793	Inverse variance weighted	12	0.012	0.022	5.875E-01	0.948	cis	Hospitalized COVID-19
11308_8	CR3L4	Cyclic AMP-responsive element-binding protein 3-like protein 4	Q8TEY5	CREB3L4	chr1:153967534	Inverse variance weighted	2	0.058	0.108	5.877E-01	0.948	cis	Hospitalized COVID-19

11214_40	DNJB9	DnaJ homolog subfamily B member 9	Q9UBS3	DNAJB9	chr7:108569867	Wald ratio	1	0.119	0.219	5.878E-01	0.948	cis	Hospitalized COVID-19
5644_60	RNAS4	Ribonuclease 4	P34096	RNASE4	chr14:20684560	Inverse variance weighted	5	-0.024	0.045	5.879E-01	0.948	cis	Hospitalized COVID-19
10075_75	ACBD6	Acyl-CoA-binding domain-containing protein 6	Q9BR61	ACBD6	chr1:180502954	Wald ratio	1	0.109	0.201	5.884E-01	0.948	cis	Hospitalized COVID-19
8065_245	PATE4	Prostate and testis expressed protein 4	P0C8F1	PATE4	chr11:125833316	Wald ratio	1	-0.044	0.082	5.887E-01	0.948	cis	Hospitalized COVID-19
11140_56	CO1A1:C-term propeptide	Collagen alpha-1(I) chain:C-term propeptide	P02452	COL1A1	chr17:50201632	Inverse variance weighted	3	-0.033	0.062	5.906E-01	0.949	cis	Hospitalized COVID-19
13102_1	FAM3D	Protein FAM3D	Q96BQ1	FAM3D	chr3:58666834	Inverse variance weighted	4	-0.020	0.037	5.916E-01	0.949	cis	Hospitalized COVID-19
5581_28	FGL1	Fibrinogen-like protein 1	Q08830	FGL1	chr8:17910365	Inverse variance weighted	7	0.012	0.022	5.918E-01	0.949	cis	Hospitalized COVID-19
12333_87	RPIA	Ribose-5-phosphate isomerase	P49247	RPIA	chr2:88691673	Inverse variance weighted	2	-0.039	0.073	5.919E-01	0.949	cis	Hospitalized COVID-19
16914_104	sCD14	Monocyte differentiation antigen CD14, soluble	P08571	CD14	chr5:140633700	Inverse variance weighted	4	-0.046	0.087	5.923E-01	0.949	cis	Hospitalized COVID-19
8900_28	NEO1	Neogenin	Q92859	NEO1	chr15:73051710	Inverse variance weighted	3	-0.047	0.088	5.926E-01	0.949	cis	Hospitalized COVID-19
6467_65	KTEL1	Protein O-glucosyltransferase 1	Q8NBL1	POGLUT1	chr3:119468963	Inverse variance weighted	3	-0.031	0.058	5.934E-01	0.949	cis	Hospitalized COVID-19
16610_13	LRP10	Low-density lipoprotein receptor-related protein 10	Q7Z4F1	LRP10	chr14:22871740	Inverse variance weighted	3	0.061	0.115	5.943E-01	0.949	cis	Hospitalized COVID-19
16296_43	LGR5	Leucine-rich repeat-containing G-protein coupled receptor 5	O75473	LGR5	chr12:71439798	Wald ratio	1	-0.097	0.183	5.945E-01	0.949	cis	Hospitalized COVID-19
9950_229	LAG-3	Lymphocyte activation gene 3 protein	P18627	LAG3	chr12:6772512	Inverse variance weighted	2	-0.053	0.100	5.949E-01	0.949	cis	Hospitalized COVID-19
6223_5	GUC2B	Guanylate cyclase activator 2B	Q16661	GUCA2B	chr1:42153410	Inverse variance weighted	2	-0.056	0.105	5.961E-01	0.949	cis	Hospitalized COVID-19
9297_12	B3GN8	UDP-GlcNAc:betaGal beta-1,3-N-acetylglucosaminyltransferase 8	Q7Z7M8	B3GNT8	chr19:41428730	Inverse variance weighted	6	-0.011	0.021	5.969E-01	0.949	cis	Hospitalized COVID-19
11248_43	HEM4	Uroporphyrinogen-III synthase	P10746	UROS	chr10:125823258	Inverse variance weighted	4	0.027	0.052	5.982E-01	0.949	cis	Hospitalized COVID-19
16900_29	MDGA1	MAM domain-containing glycosylphosphatidylinositol anchor protein 1	Q8NFP4	MDGA1	chr6:37699306	Inverse variance weighted	8	-0.009	0.016	5.987E-01	0.949	cis	Hospitalized COVID-19
19361_78	MATN3	Matrilin-3	O15232	MATN3	chr2:20012668	Inverse variance weighted	12	0.014	0.027	5.989E-01	0.949	cis	Hospitalized COVID-19
11708_2	LCN1	Lipocalin-1	P31025	LCN1	chr9:135521438	Wald ratio	1	-0.079	0.150	5.990E-01	0.949	cis	Hospitalized COVID-19
4535_50	BST1	ADP-ribosyl cyclase/cyclic ADP-ribose hydrolase 2	Q10588	BST1	chr4:15703065	Inverse variance weighted	11	0.007	0.013	5.991E-01	0.949	cis	Hospitalized COVID-19
8304_50	OPG	Tumor necrosis factor receptor superfamily member 11B	O00300	TNFRSF11B	chr8:118951885	Inverse variance weighted	4	0.030	0.057	5.999E-01	0.949	cis	Hospitalized COVID-19
13545_97	EIF1A	Probable RNA-binding protein EIF1AD	Q8N9N8	EIF1AD	chr11:66002176	Inverse variance weighted	3	0.058	0.112	6.004E-01	0.949	cis	Hospitalized COVID-19
6234_74	VITRN	Vitrin	Q6UXI7	VIT	chr2:36696690	Inverse variance weighted	6	-0.017	0.032	6.008E-01	0.949	cis	Hospitalized COVID-19
12594_5	GRAN	Grancalcin	P28676	GCA	chr2:162318840	Inverse variance weighted	2	-0.018	0.035	6.017E-01	0.949	cis	Hospitalized COVID-19
3299_29	Contactin-5	Contactin-5	O94779	CNTN5	chr11:99020949	Inverse variance weighted	3	0.040	0.076	6.032E-01	0.949	cis	Hospitalized COVID-19
5628_21	SEM3G	Semaphorin-3G	Q9NS98	SEMA3G	chr3:52445103	Inverse variance weighted	5	0.036	0.069	6.033E-01	0.949	cis	Hospitalized COVID-19
8325_37	ADH4	Alcohol dehydrogenase 4	P08319	ADH4	chr4:99157792	Wald ratio	1	-0.065	0.125	6.034E-01	0.949	cis	Hospitalized COVID-19
15299_102	MESD2	LDLR chaperone MESD	Q14696	MESD	chr15:80989828	Wald ratio	1	0.117	0.225	6.039E-01	0.949	cis	Hospitalized COVID-19
5021_13	PPase	Inorganic pyrophosphatase	Q15181	PPA1	chr10:70233911	Inverse variance weighted	4	-0.034	0.065	6.042E-01	0.949	cis	Hospitalized COVID-19
13669_6	FGFR-3:ECD	Fibroblast growth factor receptor 3:Extracellular domain	P22607	FGFR3	chr4:1793293	Inverse variance weighted	4	-0.027	0.051	6.052E-01	0.949	cis	Hospitalized COVID-19
11615_16	DAPP1	Dual adapter for phosphotyrosine and 3-phosphotyrosine and 3-phosphoinositide	Q9UN19	DAPP1	chr4:99816827	Wald ratio	1	0.060	0.116	6.064E-01	0.949	cis	Hospitalized COVID-19
7935_26	LARGE	Glycosyltransferase-like protein LARGE1	O95461	LARGE1	chr22:33922841	Inverse variance weighted	2	-0.049	0.096	6.066E-01	0.949	cis	Hospitalized COVID-19
13930_3	ABC3G	DNA dC->dU-editing enzyme APOBEC-3G	Q9HC16	APOBEC3G	chr22:39077067	Inverse variance weighted	2	0.040	0.077	6.071E-01	0.949	cis	Hospitalized COVID-19
15525_294	ADH1G	Alcohol dehydrogenase 1C	P00326	ADH1C	chr4:99352746	Wald ratio	1	0.041	0.079	6.083E-01	0.949	cis	Hospitalized COVID-19
6431_68	PCYOX	Prenylcysteine oxidase 1	Q9UHG3	PCYOX1	chr2:70257386	Inverse variance weighted	6	0.009	0.018	6.095E-01	0.949	cis	Hospitalized COVID-19
3000_66	MBL	Mannose-binding protein C	P11226	MBL2	chr10:52772784	Inverse variance weighted	9	-0.009	0.018	6.106E-01	0.949	cis	Hospitalized COVID-19
12431_13	PELO	Protein pelota homolog	Q9BRX2	PELO	chr5:52787916	Wald ratio	1	-0.111	0.219	6.124E-01	0.949	cis	Hospitalized COVID-19
8697_38	Glypican 1	Glypican-1	P35052	GPC1	chr2:240435663	Inverse variance weighted	5	-0.012	0.024	6.125E-01	0.949	cis	Hospitalized COVID-19
10737_96	Serpin B1	Leukocyte elastase inhibitor	P30740	SERPINB1	chr6:2841959	Wald ratio	1	0.092	0.182	6.130E-01	0.949	cis	Hospitalized COVID-19
3583_54	Arylsulfatase A	Arylsulfatase A	P15289	ARSA	chr22:50628173	Wald ratio	1	0.063	0.125	6.132E-01	0.949	cis	Hospitalized COVID-19
19293_6	VP26A	Vacuolar protein sorting-associated protein 26A	O75436	VPS26A	chr10:69123512	Wald ratio	1	-0.032	0.063	6.142E-01	0.949	cis	Hospitalized COVID-19
3077_66	Coagulation Factor Xa	Coagulation factor Xa	P00742	F10	chr13:113122799	Inverse variance weighted	2	-0.076	0.152	6.155E-01	0.949	cis	Hospitalized COVID-19
17680_12	EPHB1	Ephrin type-B receptor 1	P54762	EPHB1	chr3:134795260	Inverse variance weighted	3	0.027	0.055	6.156E-01	0.949	cis	Hospitalized COVID-19
6060_2	PIP	Prolactin-inducible protein	P12273	PIP	chr7:143132077	Inverse variance weighted	5	-0.012	0.024	6.168E-01	0.949	cis	Hospitalized COVID-19
9201_13	Transgelin-2	Transgelin-2	P37802	TAGLN2	chr1:159925507	Wald ratio	1	0.113	0.227	6.169E-01	0.949	cis	Hospitalized COVID-19
4969_2	Carbonic anhydrase I	Carbonic anhydrase 1	P00915	CA1	chr8:85379014	Inverse variance weighted	3	0.036	0.071	6.181E-01	0.949	cis	Hospitalized COVID-19
17773_26	SNAG	Gamma-soluble NSF attachment protein	Q99747	NAPG	chr18:10525905	Wald ratio	1	0.073	0.146	6.183E-01	0.949	cis	Hospitalized COVID-19
3894_15	NAGK	N-acetyl-D-glucosamine kinase	Q9UJ70	NAGK	chr2:71064344	Inverse variance weighted	3	-0.027	0.054	6.186E-01	0.949	cis	Hospitalized COVID-19
9916_146	LRC4B:ECD	Leucine-rich repeat-containing protein 4B:Extracellular domain	Q9NT99	LRRC4B	chr19:50568435	Inverse variance weighted	2	-0.101	0.203	6.191E-01	0.949	cis	Hospitalized COVID-19
8479_4	MMP-10	Stromelysin-2	P09238	MMP10	chr11:102780628	Inverse variance weighted	6	-0.023	0.047	6.198E-01	0.949	cis	Hospitalized COVID-19
3810_50	FGR	Tyrosine-protein kinase Fgr	P09769	FGR	chr1:27635185	Inverse variance weighted	2	0.074	0.150	6.212E-01	0.949	cis	Hospitalized COVID-19
5763_67	HBD-4	Beta-defensin 104	Q8WTQ1	DEFB104A	chr8:7836436	Inverse variance weighted	2	-0.117	0.239	6.225E-01	0.949	cis	Hospitalized COVID-19
7173_141	T132C:ECD	Transmembrane protein 132C:Extracellular domain	Q8N3T6	TMEM132C	chr12:128267170	Inverse variance weighted	5	0.023	0.047	6.226E-01	0.949	cis	Hospitalized COVID-19
18321_38	MTDC	Bifunctional methylenetetrahydrofolate dehydrogenase/cyclohydrolase, mitochondrial	P13995	MTHFD2	chr2:74186172	Inverse variance weighted	2	0.051	0.105	6.226E-01	0.949	cis	Hospitalized COVID-19
4961_17	annexin II	Annexin A2	P07355	ANXA2	chr15:60402883	Inverse variance weighted	2	0.065	0.133	6.238E-01	0.949	cis	Hospitalized COVID-19
2855_49	ERK-1	Mitogen-activated protein kinase 3	P27361	MAPK3	chr16:30123506	Inverse variance weighted	3	0.028	0.057	6.238E-01	0.949	cis	Hospitalized COVID-19
3805_16	Endocan	Endothelial cell-specific molecule 1	Q9NQ30	ESM1	chr5:55022671	Inverse variance weighted	3	-0.033	0.067	6.240E-01	0.949	cis	Hospitalized COVID-19
7918_114	Amylase, alpha 1A	Alpha-amylase 1	P04745	AMY1A	chr1:103655760	Inverse variance weighted	11	0.021	0.043	6.240E-01	0.949	cis	Hospitalized COVID-19
13534_20	MYOM2	Myomesin-2	P54296	MYOM2	chr8:2045046	Inverse variance weighted	3	-0.066	0.135	6.246E-01	0.949	cis	Hospitalized COVID-19
2692_74	NPS-PLA2	Phospholipase A2, membrane associated	P14555	PLA2G2A	chr1:19980416	Inverse variance weighted	6	-0.009	0.019	6.248E-01	0.949	cis	Hospitalized COVID-19
5452_71	ASGR1	Asialoglycoprotein receptor 1	P07306	ASGR1	chr17:7179564	Inverse variance weighted	2	-0.054	0.110	6.253E-01	0.949	cis	Hospitalized COVID-19
11531_24	GI24:CD	V-type immunoglobulin domain-containing suppressor of T-cell activation:Cytoplasmic domain	Q9H7M9	VSIR	chr10:71773520	Wald ratio	1	0.081	0.166	6.259E-01	0.949	cis	Hospitalized COVID-19
4467_49	SPARCL1	SPARC-like protein 1	Q14515	SPARCL1	chr4:87531061	Inverse variance weighted	6	0.012	0.024	6.263E-01	0.949	cis	Hospitalized COVID-19
13621_31	AP2A2	AP-2 complex subunit alpha-2	O94973	AP2A2	chr11:924881	Wald ratio	1	0.079	0.163	6.269E-01	0.949	cis	Hospitalized COVID-19
3378_49	Kallikrein 7	Kallikrein-7	P49862	KLK7	chr19:50984099	Inverse variance weighted	2	-0.056	0.116	6.269E-01	0.949	cis	Hospitalized COVID-19
15533_97	Macrophage scavenger receptor:ECD	Macrophage scavenger receptor types I and II:Extracellular domain	P21757	MSR1	chr8:16567490	Inverse variance weighted	5	-0.018	0.036	6.270E-01	0.949	cis	Hospitalized COVID-19
13934_3	MCF2L	Guanine nucleotide exchange factor DBS	O15068	MCF2L	chr13:112894378	Wald ratio	1	-0.024	0.050	6.274E-01	0.949	cis	Hospitalized COVID-19
3593_72	Caspase-3	Caspase-3	P42574	CASP3	chr4:184650062	Wald ratio	1	0.042	0.087	6.279E-01	0.949	cis	Hospitalized COVID-19
5632_6	CRAC1	Cartilage acidic protein 1	Q9NQ79	CRTAC1	chr10:98030828	Inverse variance weighted	9	-0.017	0.034	6.282E-01	0.949	cis	Hospitalized COVID-19
13388_57	NEC1	Neuroendocrine convertase 1	P29120	PCSK1	chr5:96434143	Inverse variance weighted	7	-0.021	0.043	6.287E-01	0.949	cis	Hospitalized COVID-19
11142_11	ANGL1:C-term	Angiopoietin-related protein 1:C-Term, Fibrinogen domian	O95841	ANGPTL1	chr1:178871077	Inverse variance weighted	4	0.016	0.033	6.290E-01	0.949	cis	Hospitalized COVID-19
10800_15	Collagen-binding protein	Serpin H1	P50454	SERPINH1	chr11:75562056	Inverse variance weighted	2	-0.039	0.081	6.293E-01	0.949	cis	Hospitalized COVID-19
2602_2	Angiopoietin-2	Angiopoietin-2	O15123	ANGPT2	chr8:6563409	Inverse variance weighted	3	0.033	0.069	6.296E-01	0.949	cis	Hospitalized COVID-19
2571_12	IGFBP-3	Insulin-like growth factor-binding protein 3	P17936	IGFBP3	chr7:45921874	Inverse variance weighted	4	-0.019	0.040	6.297E-01	0.949	cis	Hospitalized COVID-19
15573_110	CSPG3	Neurocan core protein	O14594	NCAN	chr19:19211958	Inverse variance weighted	2	-0.037	0.078	6.302E-01	0.949	cis	Hospitalized COVID-19
3440_7	granzyme A	Granzyme A	P12544	GZMA	chr5:55102646	Inverse variance weighted	4	0.020	0.041	6.310E-01	0.949	cis	Hospitalized COVID-19
10903_50	STX8	Syntaxin-8	Q9UNK0	STX8	chr17:9576591	Wald ratio	1	0.078	0.163	6.320E-01	0.949	cis	Hospitalized COVID-19

9370_69	GGH	Gamma-glutamyl hydrolase	Q92820	GGH	chr8:63039407	Inverse variance weighted	5	-0.012	0.026	6.321E-01	0.949	cis	Hospitalized COVID-19
19590_46	SP-D	Pulmonary surfactant-associated protein D	P35247	SFTPD	chr10:79982614	Inverse variance weighted	7	-0.020	0.041	6.322E-01	0.949	cis	Hospitalized COVID-19
3296_92	CNTN2	Contactin-2	Q02246	CNTN2	chr1:205042937	Inverse variance weighted	9	0.012	0.025	6.331E-01	0.949	cis	Hospitalized COVID-19
5631_83	MOTI	Promotilin	P12872	MLN	chr6:33804003	Inverse variance weighted	6	0.012	0.025	6.331E-01	0.949	cis	Hospitalized COVID-19
15395_15	GST M1-1	Glutathione S-transferase Mu 1	P09488	GSTM1	chr1:109687814	Inverse variance weighted	5	-0.029	0.061	6.351E-01	0.952	cis	Hospitalized COVID-19
12334_25	cSHMT	Serine hydroxymethyltransferase, cytosolic	P34896	SHMT1	chr17:18363563	Inverse variance weighted	6	-0.010	0.022	6.359E-01	0.952	cis	Hospitalized COVID-19
6366_38	TXD15	Thioredoxin domain-containing protein 15	Q96J42	TXNDC15	chr5:134874371	Inverse variance weighted	3	0.014	0.029	6.379E-01	0.954	cis	Hospitalized COVID-19
15530_33	EphB4	Ephrin type-B receptor 4	P54760	EPHB4	chr7:100827523	Wald ratio	1	-0.062	0.133	6.398E-01	0.955	cis	Hospitalized COVID-19
16300_4	TREM2	Triggering receptor expressed on myeloid cells 2	Q9NZC2	TREM2	chr6:41163186	Inverse variance weighted	7	0.017	0.036	6.399E-01	0.955	cis	Hospitalized COVID-19
5134_52	TIMD3	Hepatitis A virus cellular receptor 2	Q8TDQ0	HAVCR2	chr5:157142869	Inverse variance weighted	7	0.012	0.027	6.420E-01	0.957	cis	Hospitalized COVID-19
2827_23	Fractalkine/CX3CL-1	Fractalkine	P78423	CX3CL1	chr16:57372477	Wald ratio	1	-0.040	0.086	6.422E-01	0.957	cis	Hospitalized COVID-19
5509_7	EGF:ECD	Epidermal growth factor:Extracellular domain	P01133	EGF	chr4:109912883	Inverse variance weighted	4	-0.042	0.091	6.432E-01	0.957	cis	Hospitalized COVID-19
16620_26	LY75	Lymphocyte antigen 75	O60449	LY75	chr2:159904756	Inverse variance weighted	7	0.011	0.024	6.438E-01	0.957	cis	Hospitalized COVID-19
18206_18	ADH6	Alcohol dehydrogenase 6	P28332	ADH6	chr4:99219537	Wald ratio	1	0.077	0.166	6.444E-01	0.957	cis	Hospitalized COVID-19
8957_72	XTP3B	Endoplasmic reticulum lectin 1	Q96DZ1	ERLEC1	chr2:53787009	Wald ratio	1	-0.070	0.152	6.448E-01	0.957	cis	Hospitalized COVID-19
13088_397	BTC	Betacellulin	P35070	BTC	chr4:74794523	Inverse variance weighted	2	0.028	0.061	6.448E-01	0.957	cis	Hospitalized COVID-19
17350_13	CHM2B	Charged multivesicular body protein 2b	Q9UQN3	CHMP2B	chr3:87227271	Inverse variance weighted	3	-0.024	0.052	6.458E-01	0.957	cis	Hospitalized COVID-19
17724_3	WWOX	WW domain-containing oxidoreductase	Q9NZC7	WWOX	chr16:78099400	Wald ratio	1	0.063	0.138	6.477E-01	0.959	cis	Hospitalized COVID-19
2948_58	Growth hormone receptor	Growth hormone receptor	P10912	GHR	chr5:42423439	Inverse variance weighted	4	0.022	0.048	6.500E-01	0.961	cis	Hospitalized COVID-19
6649_51	NET1	Netrin-1	O95631	NTN1	chr17:9021510	Inverse variance weighted	6	0.016	0.036	6.502E-01	0.961	cis	Hospitalized COVID-19
13717_15	FCN2	Ficolin-2	Q15485	FCN2	chr9:134880810	Inverse variance weighted	8	-0.018	0.041	6.505E-01	0.961	cis	Hospitalized COVID-19
13536_56	POLI	DNA polymerase iota	Q9UNA4	POLI	chr18:54269517	Wald ratio	1	-0.052	0.116	6.514E-01	0.961	cis	Hospitalized COVID-19
7179_69	NFASC	Neurofascin	O94856	NFASC	chr1:204828651	Inverse variance weighted	4	-0.013	0.028	6.516E-01	0.961	cis	Hospitalized COVID-19
10070_22	PLCG2	1-phosphatidylinositol 4,5-bisphosphate phosphodiesterase gamma-2	P16885	PLCG2	chr16:81779279	Inverse variance weighted	2	0.079	0.175	6.525E-01	0.961	cis	Hospitalized COVID-19
8402_22	CYTL1	Cytokine-like protein 1	Q9NRR1	CYTL1	chr4:5019458	Inverse variance weighted	3	-0.031	0.069	6.531E-01	0.961	cis	Hospitalized COVID-19
5092_51	JAG1:ECD	Protein jagged-1:Extracellular domain	P78504	JAG1	chr20:10673999	Inverse variance weighted	2	-0.063	0.140	6.542E-01	0.961	cis	Hospitalized COVID-19
3210_1	METAP1	Methionine aminopeptidase 1	P53582	METAP1	chr4:98995659	Wald ratio	1	-0.094	0.211	6.546E-01	0.961	cis	Hospitalized COVID-19
7761_125	CHKB	Choline/ethanolamine kinase	Q9Y259	CHKB	chr22:50601455	Inverse variance weighted	3	0.044	0.099	6.552E-01	0.961	cis	Hospitalized COVID-19
17398_55	HO-1	Heme oxygenase 1	P09601	HMOX1	chr22:35380361	Inverse variance weighted	2	-0.049	0.111	6.554E-01	0.961	cis	Hospitalized COVID-19
18930_28	SLIT2	Slit homolog 2 protein	O94813	SLIT2	chr4:20251905	Inverse variance weighted	2	0.039	0.088	6.568E-01	0.963	cis	Hospitalized COVID-19
10832_24	B4GT6	Beta-1,4-galactosyltransferase 6	Q9UBX8	B4GALT6	chr18:31685836	Inverse variance weighted	4	-0.010	0.023	6.580E-01	0.964	cis	Hospitalized COVID-19
9793_145	IGDC4	Immunoglobulin superfamily DCC subclass member 4	Q8TDY8	IGDCC4	chr15:65422947	Inverse variance weighted	7	0.015	0.033	6.591E-01	0.964	cis	Hospitalized COVID-19
3339_33	TSP2	Thrombospondin-2	P35442	THBS2	chr6:169254050	Inverse variance weighted	7	0.011	0.026	6.594E-01	0.964	cis	Hospitalized COVID-19
9329_28	TRML1:ECD	Trem-like transcript 1 protein:Extracellular domain, Ig-like V-type domain	Q86YW5	TREML1	chr6:41154347	Inverse variance weighted	2	-0.043	0.098	6.606E-01	0.964	cis	Hospitalized COVID-19
3448_13	IR	Insulin receptor	P06213	INSR	chr19:7294414	Wald ratio	1	-0.063	0.145	6.610E-01	0.964	cis	Hospitalized COVID-19
12459_13	PKHA1	Pleckstrin homology domain-containing family A member 1	Q9HB21	PLEKHA1	chr10:122374696	Inverse variance weighted	4	0.015	0.033	6.614E-01	0.964	cis	Hospitalized COVID-19
15559_5	ANTR2	Anthrax toxin receptor 2	P58335	ANTXR2	chr4:80125454	Inverse variance weighted	4	-0.023	0.052	6.618E-01	0.964	cis	Hospitalized COVID-19
13044_5	TS101	Tumor susceptibility gene 101 protein	Q99816	TSG101	chr11:18526951	Inverse variance weighted	2	-0.049	0.113	6.629E-01	0.965	cis	Hospitalized COVID-19
9357_4	CREG1	Protein CREG1	O75629	CREG1	chr1:167553805	Inverse variance weighted	2	-0.034	0.079	6.643E-01	0.965	cis	Hospitalized COVID-19
2418_55	Apo E	Apolipoprotein E	P02649	APOE	chr19:44905791	Inverse variance weighted	4	0.040	0.092	6.646E-01	0.965	cis	Hospitalized COVID-19
2774_10	IL-16	Interleukin-16	Q14005	IL16	chr15:81159575	Inverse variance weighted	10	-0.010	0.024	6.650E-01	0.965	cis	Hospitalized COVID-19
11287_14	Cytochrome b5	Cytochrome b5	P00167	CYB5A	chr18:74291973	Wald ratio	1	0.113	0.262	6.653E-01	0.965	cis	Hospitalized COVID-19
3322_52	LRIG3	Leucine-rich repeats and immunoglobulin-like domains protein 3	Q6UXM1	LRIG3	chr12:58920504	Inverse variance weighted	6	0.014	0.033	6.667E-01	0.965	cis	Hospitalized COVID-19
17345_12	ZADH2	Prostaglandin reductase 3	Q8N4Q0	PTGR3	chr18:75209139	Wald ratio	1	-0.044	0.103	6.668E-01	0.965	cis	Hospitalized COVID-19
12575_30	C1TC	C-1-tetrahydrofolate synthase, cytoplasmic	P11586	MTHFD1	chr14:64388031	Inverse variance weighted	2	0.105	0.245	6.686E-01	0.965	cis	Hospitalized COVID-19
10948_14	PLD3	Phospholipase D3	Q8IV08	PLD3	chr19:40348456	Wald ratio	1	-0.091	0.212	6.687E-01	0.965	cis	Hospitalized COVID-19
14009_65	TNFAIP3	Tumor necrosis factor alpha-induced protein 3	P21580	TNFAIP3	chr6:137867214	Wald ratio	1	-0.028	0.065	6.696E-01	0.965	cis	Hospitalized COVID-19
4158_54	uPA	Urokinase-type plasminogen activator	P00749	PLAU	chr10:73909177	Inverse variance weighted	4	-0.015	0.036	6.697E-01	0.965	cis	Hospitalized COVID-19
3234_23	URB	Coiled-coil domain-containing protein 80	Q76M96	CCDC80	chr3:112649530	Inverse variance weighted	2	-0.043	0.102	6.702E-01	0.965	cis	Hospitalized COVID-19
12820_1	GRAP	GRB2-related adapter protein	Q13588	GRAP	chr17:19047011	Wald ratio	1	0.122	0.288	6.716E-01	0.965	cis	Hospitalized COVID-19
8766_29	LIRA5	Leukocyte immunoglobulin-like receptor subfamily A member 5	A6NI73	LILRA5	chr19:54313166	Wald ratio	1	-0.074	0.175	6.719E-01	0.965	cis	Hospitalized COVID-19
5615_62	F172A	Protein FAM172A	Q8WUF8	FAM172A	chr5:94111699	Wald ratio	1	-0.063	0.148	6.720E-01	0.965	cis	Hospitalized COVID-19
6897_38	B3GA3	Galactosylgalactosylxylosylprotein 3-beta-glucuronosyltransferase 3	O94766	B3GAT3	chr11:62622154	Inverse variance weighted	3	-0.019	0.046	6.722E-01	0.965	cis	Hospitalized COVID-19
5630_48	CM35H	CMRF35-like molecule 8	Q9UGN4	CD300A	chr17:74466399	Inverse variance weighted	8	0.010	0.023	6.729E-01	0.965	cis	Hospitalized COVID-19
14151_4	UCRP	Ubiquitin-like protein ISG15	P05161	ISG15	chr1:1001138	Inverse variance weighted	2	-0.013	0.032	6.729E-01	0.965	cis	Hospitalized COVID-19
11212_7	TXND5	Thioredoxin domain-containing protein 5	Q8NBS9	TXNDC5	chr6:7910788	Inverse variance weighted	6	-0.018	0.042	6.733E-01	0.965	cis	Hospitalized COVID-19
14091_42	Carbonyl reductase 3	Carbonyl reductase [NADPH] 3	O75828	CBR3	chr21:36135079	Inverse variance weighted	6	0.012	0.029	6.740E-01	0.965	cis	Hospitalized COVID-19
13094_75	RSPO3	R-spondin-3	Q9BXY4	RSPO3	chr6:127118671	Wald ratio	1	0.070	0.167	6.753E-01	0.965	cis	Hospitalized COVID-19
8893_29	PARP:region 1	Poly [ADP-ribose] polymerase 1:region 1	P09874	PARP1	chr1:226408154	Wald ratio	1	-0.085	0.204	6.759E-01	0.965	cis	Hospitalized COVID-19
14131_37	EFNB2:ECD	Ephrin-B2:Extracellular domain	P52799	EFNB2	chr13:106535662	Inverse variance weighted	2	-0.062	0.148	6.760E-01	0.965	cis	Hospitalized COVID-19
11211_7	TBCE	Tubulin-specific chaperone E	Q15813	TBCE	chr1:235367360	Inverse variance weighted	2	-0.066	0.158	6.766E-01	0.965	cis	Hospitalized COVID-19
13107_9	LYPD3	Ly6/PLAUR domain-containing protein 3	O95274	LYPD3	chr19:43465608	Wald ratio	1	-0.048	0.116	6.772E-01	0.965	cis	Hospitalized COVID-19
15535_3	Marapsin	Serine protease 27	Q9BQR3	PRSS27	chr16:2720551	Wald ratio	1	-0.033	0.080	6.774E-01	0.965	cis	Hospitalized COVID-19
12612_37	PSB1	Proteasome subunit beta type-1	P20618	PSMB1	chr6:170553307	Inverse variance weighted	2	0.024	0.057	6.777E-01	0.965	cis	Hospitalized COVID-19
2677_1	ERBB1	Epidermal growth factor receptor	P00533	EGFR	chr7:55019017	Inverse variance weighted	3	0.030	0.071	6.785E-01	0.965	cis	Hospitalized COVID-19
9223_11	ARMET	Mesencephalic astrocyte-derived neurotrophic factor	P55145	MANF	chr3:51385291	Wald ratio	1	-0.077	0.187	6.800E-01	0.965	cis	Hospitalized COVID-19
9981_18	K1467:C-term	Protein FAM234B:C-term	A2RU67	FAM234B	chr12:13044381	Inverse variance weighted	5	-0.025	0.061	6.802E-01	0.965	cis	Hospitalized COVID-19
3516_60	SDF-1	Stromal cell-derived factor 1	P48061	CXCL12	chr10:44386493	Inverse variance weighted	2	0.048	0.116	6.816E-01	0.965	cis	Hospitalized COVID-19
2913_1	MPIF-1	C-C motif chemokine 23	P55773	CCL23	chr17:36017972	Inverse variance weighted	2	-0.047	0.114	6.821E-01	0.965	cis	Hospitalized COVID-19
16781_2	ENASE	Cytosolic endo-beta-N-acetylglucosaminidase	Q8NFI3	ENGASE	chr17:79074824	Inverse variance weighted	9	0.010	0.025	6.840E-01	0.965	cis	Hospitalized COVID-19
3640_14	RAP	alpha-2-macroglobulin receptor-associated protein	P30533	LRPAP1	chr4:3532446	Inverse variance weighted	4	0.033	0.081	6.843E-01	0.965	cis	Hospitalized COVID-19
4968_50	CAPG	Macrophage-capping protein	P40121	CAPG	chr2:85418432	Inverse variance weighted	4	0.015	0.037	6.844E-01	0.965	cis	Hospitalized COVID-19
3025_50	bFGF	Fibroblast growth factor 2	P09038	FGF2	chr4:122826682	Inverse variance weighted	6	-0.012	0.029	6.845E-01	0.965	cis	Hospitalized COVID-19
9839_148	Tirap	Toll/interleukin-1 receptor domain-containing adapter protein	P58753	TIRAP	chr11:126282497	Inverse variance weighted	3	0.029	0.071	6.848E-01	0.965	cis	Hospitalized COVID-19
16324_38	TLR1:ECD	Toll-like receptor 1:Extracellular domain	Q15399	TLR1	chr4:38856817	Wald ratio	1	0.035	0.086	6.850E-01	0.965	cis	Hospitalized COVID-19
15570_99	Complement receptor type 2	Complement receptor type 2	P20023	CR2	chr1:207453024	Inverse variance weighted	3	0.028	0.068	6.862E-01	0.965	cis	Hospitalized COVID-19
12357_41	SNP29	Synaptosomal-associated protein 29	O95721	SNAP29	chr22:20859007	Wald ratio	1	0.067	0.166	6.868E-01	0.965	cis	Hospitalized COVID-19
18458_4	PGES2	Prostaglandin E synthase 2	Q9H7Z7	PTGES2	chr9:128128462	Wald ratio	1	-0.055	0.137	6.872E-01	0.965	cis	Hospitalized COVID-19

11196_31	Collagen alpha-3(VI):BPTI/Kunitz inhibitor	Collagen alpha-3(VI) chain:Bovine pancreatic trypsin inhibitor/Kunitz inhibitor domain, isoform 1	P12111	COL6A3	chr2:237414328	Wald ratio	1	-0.064	0.159	6.872E-01	0.965	cis	Hospitalized COVID-19
9264_11	CATO	Cathepsin O	P43234	CTSO	chr4:155953912	Inverse variance weighted	2	-0.028	0.069	6.872E-01	0.965	cis	Hospitalized COVID-19
4328_2	BOC	Brother of CDO	Q9BWV1	BOC	chr3:113211003	Inverse variance weighted	5	0.017	0.043	6.880E-01	0.966	cis	Hospitalized COVID-19
10924_258	NEUFC	Neuferricin	Q8WUJ1	CYB5D2	chr17:4143168	Inverse variance weighted	3	-0.023	0.057	6.893E-01	0.967	cis	Hospitalized COVID-19
12682_5	KAT3	Kynurenine--oxoglutarate transaminase 3	Q6YP21	KYAT3	chr1:88992953	Inverse variance weighted	7	-0.015	0.038	6.901E-01	0.967	cis	Hospitalized COVID-19
2615_60	Ephrin-A5	Ephrin-A5	P52803	EFNA5	chr5:107670937	Inverse variance weighted	2	-0.028	0.070	6.925E-01	0.968	cis	Hospitalized COVID-19
17702_53	UGT 1A1	UDP-glucuronosyltransferase 1-1	P22309	UGT1A1	chr2:233760270	Inverse variance weighted	2	-0.028	0.070	6.927E-01	0.968	cis	Hospitalized COVID-19
3221_54	SARP-2	Secreted frizzled-related protein 1	Q8N474	SFRP1	chr8:41309473	Inverse variance weighted	3	0.026	0.067	6.939E-01	0.968	cis	Hospitalized COVID-19
9759_13	INDO	Indoleamine 2,3-dioxygenase 1	P14902	IDO1	chr8:39902275	Wald ratio	1	-0.068	0.173	6.963E-01	0.968	cis	Hospitalized COVID-19
8229_1	GXLT1	Glucoside xylosyltransferase 1	Q4G148	GXYLT1	chr12:42144874	Inverse variance weighted	5	0.014	0.037	6.976E-01	0.968	cis	Hospitalized COVID-19
15585_304	fibulin 5	Fibulin-5	Q9UBX5	FBLN5	chr14:91947987	Wald ratio	1	0.049	0.126	6.980E-01	0.968	cis	Hospitalized COVID-19
9901_28	EGLN1	Egl nine homolog 1	Q9GZT9	EGLN1	chr1:231422287	Inverse variance weighted	5	0.025	0.063	6.983E-01	0.968	cis	Hospitalized COVID-19
14076_74	Cystatin-S	Cystatin-S	P01036	CST4	chr20:23689038	Inverse variance weighted	3	0.024	0.063	6.991E-01	0.968	cis	Hospitalized COVID-19
2647_66	Rab GDP dissociation inhibitor beta	Rab GDP dissociation inhibitor beta	P50395	GDI2	chr10:5842132	Wald ratio	1	-0.040	0.104	6.996E-01	0.968	cis	Hospitalized COVID-19
5109_24	Nr-CAM	Neuronal cell adhesion molecule	Q92823	NRCAM	chr7:108456717	Wald ratio	1	0.070	0.180	6.998E-01	0.968	cis	Hospitalized COVID-19
14112_40	RELT	Tumor necrosis factor receptor superfamily member 19L	Q969Z4	RELT	chr11:73376399	Wald ratio	1	-0.094	0.244	6.999E-01	0.968	cis	Hospitalized COVID-19
12450_42	PMVK	Phosphomevalonate kinase	Q15126	PMVK	chr1:154936719	Inverse variance weighted	2	0.206	0.536	7.003E-01	0.968	cis	Hospitalized COVID-19
2665_26	BCMA	Tumor necrosis factor receptor superfamily member 17	Q02223	TNFRSF17	chr16:11965210	Wald ratio	1	-0.030	0.078	7.010E-01	0.968	cis	Hospitalized COVID-19
5015_15	PAFAH	Platelet-activating factor acetylhydrolase	Q13093	PLA2G7	chr6:46735693	Inverse variance weighted	2	-0.057	0.148	7.011E-01	0.968	cis	Hospitalized COVID-19
4471_50	TGM3	Protein-glutamine gamma-glutamyltransferase E	Q08188	TGM3	chr20:2296001	Inverse variance weighted	4	0.010	0.027	7.012E-01	0.968	cis	Hospitalized COVID-19
6364_7	TPSNR	Tapasin-related protein	Q9BX59	TAPBPL	chr12:6451690	Inverse variance weighted	7	-0.008	0.021	7.012E-01	0.968	cis	Hospitalized COVID-19
14271_23	RAB6B	Ras-related protein Rab-6B	Q9NRW1	RAB6B	chr3:133895882	Wald ratio	1	0.020	0.052	7.013E-01	0.968	cis	Hospitalized COVID-19
12376_85	p19-INK4d	Cyclin-dependent kinase 4 inhibitor D	P55273	CDKN2D	chr19:10569059	Wald ratio	1	-0.098	0.256	7.015E-01	0.968	cis	Hospitalized COVID-19
19437_61	L-VEGF165	Isoform L-VEGF165	P15692	VEGFA	chr6:43770184	Inverse variance weighted	7	-0.027	0.071	7.027E-01	0.968	cis	Hospitalized COVID-19
6557_50	LRC15	Leucine-rich repeat-containing protein 15	Q8TF66	LRRC15	chr3:194369743	Inverse variance weighted	4	0.014	0.038	7.028E-01	0.968	cis	Hospitalized COVID-19
4913_78	HCC-4	C-C motif chemokine 16	O15467	CCL16	chr17:35981497	Inverse variance weighted	8	-0.010	0.028	7.031E-01	0.968	cis	Hospitalized COVID-19
19161_1	UBP15	Ubiquitin carboxyl-terminal hydrolase 15	Q9Y4E8	USP15	chr12:62260338	Inverse variance weighted	2	-0.027	0.070	7.037E-01	0.968	cis	Hospitalized COVID-19
19557_3	KLOTB	Beta-klotho	Q86Z14	KLB	chr4:39406930	Inverse variance weighted	8	0.011	0.028	7.042E-01	0.968	cis	Hospitalized COVID-19
15633_6	RBP	Retinol-binding protein 4	P02753	RBP4	chr10:93601744	Wald ratio	1	-0.057	0.150	7.048E-01	0.968	cis	Hospitalized COVID-19
2201_17	Endostatin	Endostatin	P39060	COL18A1	chr21:45405165	Inverse variance weighted	4	-0.022	0.058	7.050E-01	0.968	cis	Hospitalized COVID-19
8043_153	COMP	Cartilage oligomeric matrix protein	P49747	COMP	chr19:18791305	Inverse variance weighted	4	0.041	0.109	7.065E-01	0.968	cis	Hospitalized COVID-19
18319_7	ODPX	Pyruvate dehydrogenase protein X component, mitochondrial	O00330	PDHX	chr11:34915829	Inverse variance weighted	2	0.035	0.094	7.073E-01	0.968	cis	Hospitalized COVID-19
16583_8	AP4A	Bis(5'-nucleosyl)-tetraphosphatase [asymmetrical]	P50583	NUDT2	chr9:34329506	Inverse variance weighted	7	0.020	0.055	7.074E-01	0.968	cis	Hospitalized COVID-19
7784_1	Kininogen, HMW	Kininogen-1	P01042	KNG1	chr3:186717348	Inverse variance weighted	2	0.052	0.140	7.080E-01	0.968	cis	Hospitalized COVID-19
11257_1	DHPR	Dihydropteridine reductase	P09417	QDPR	chr4:17512206	Inverse variance weighted	4	0.016	0.044	7.083E-01	0.968	cis	Hospitalized COVID-19
15440_57	NEC2	Neuroendocrine convertase 2	P16519	PCSK2	chr20:17226107	Wald ratio	1	-0.046	0.124	7.083E-01	0.968	cis	Hospitalized COVID-19
8252_2	NOTUM	Palmitoleoyl-protein carboxylesterase NOTUM	Q6P988	NOTUM	chr17:81961840	Inverse variance weighted	3	0.034	0.092	7.094E-01	0.968	cis	Hospitalized COVID-19
9884_8	PPIL1	Peptidyl-prolyl cis-trans isomerase-like 1	Q9Y3C6	PPIL1	chr6:36874803	Inverse variance weighted	5	0.008	0.022	7.098E-01	0.968	cis	Hospitalized COVID-19
12620_3	Septin-11	Septin-11	Q9NVA2	SEPTIN11	chr4:76949751	Wald ratio	1	0.064	0.173	7.119E-01	0.969	cis	Hospitalized COVID-19
10569_28	MFAP2	Microfibrillar-associated protein 2	P55001	MFAP2	chr1:16980632	Inverse variance weighted	3	-0.050	0.137	7.126E-01	0.969	cis	Hospitalized COVID-19
6520_87	MGP	Matrix Gla protein	P08493	MGP	chr12:14885857	Wald ratio	1	-0.062	0.167	7.129E-01	0.969	cis	Hospitalized COVID-19
11616_9	HSF1	Heat shock factor protein 1	Q00613	HSF1	chr8:144291591	Wald ratio	1	0.069	0.188	7.136E-01	0.969	cis	Hospitalized COVID-19
8014_359	MANEA	Glycoprotein endo-alpha-1,2-mannosidase	Q5SRI9	MANEA	chr6:95577485	Inverse variance weighted	6	0.006	0.017	7.137E-01	0.969	cis	Hospitalized COVID-19
4563_61	PLCG1	1-phosphatidylinositol 4,5-bisphosphate phosphodiesterase gamma-1	P19174	PLCG1	chr20:41136960	Wald ratio	1	-0.066	0.179	7.139E-01	0.969	cis	Hospitalized COVID-19
5005_4	MK12	Mitogen-activated protein kinase 12	P53778	MAPK12	chr22:50261716	Inverse variance weighted	2	0.030	0.083	7.148E-01	0.969	cis	Hospitalized COVID-19
4498_62	NCAM-120	Neural cell adhesion molecule 1, 120 kDa isoform	P13591	NCAM1	chr11:112961247	Inverse variance weighted	3	-0.013	0.037	7.201E-01	0.974	cis	Hospitalized COVID-19
4129_72	Factor B	Complement factor B	P00751	CFB	chr6:31945650	Inverse variance weighted	3	0.034	0.095	7.202E-01	0.974	cis	Hospitalized COVID-19
9394_19	Aminopeptidase	Carboxypeptidase Q	Q9Y646	CPQ	chr8:96645242	Inverse variance weighted	8	-0.011	0.031	7.214E-01	0.974	cis	Hospitalized COVID-19
2515_14	GFRa-2	GDNF family receptor alpha-2	O00451	GFRA2	chr8:21812357	Inverse variance weighted	6	0.010	0.028	7.226E-01	0.974	cis	Hospitalized COVID-19
9883_29	Glyoxalase I	Lactoylglutathione lyase	Q04760	GLO1	chr6:38703145	Inverse variance weighted	3	-0.030	0.085	7.231E-01	0.974	cis	Hospitalized COVID-19
12643_4	ARRB1	Beta-arrestin-1	P49407	ARRB1	chr11:75351705	Wald ratio	1	-0.047	0.132	7.239E-01	0.974	cis	Hospitalized COVID-19
5350_14	GPC6	Glypican-6	Q9Y625	GPC6	chr13:93226807	Wald ratio	1	0.078	0.220	7.247E-01	0.974	cis	Hospitalized COVID-19
18236_3	LEGL	Galectin-related protein	Q3ZCW2	LGALSL	chr2:64453969	Wald ratio	1	-0.047	0.133	7.253E-01	0.974	cis	Hospitalized COVID-19
16015_19	ALT	Alanine aminotransferase 1	P24298	GPT	chr8:144502973	Wald ratio	1	-0.067	0.192	7.262E-01	0.974	cis	Hospitalized COVID-19
17140_57	PDGFD	Platelet-derived growth factor D	Q9GZP0	PDGFD	chr11:104164379	Inverse variance weighted	6	0.034	0.097	7.268E-01	0.974	cis	Hospitalized COVID-19
3325_2	MATN2	Matrilin-2	O00339	MATN2	chr8:97868840	Inverse variance weighted	4	0.023	0.067	7.269E-01	0.974	cis	Hospitalized COVID-19
17320_19	ADPPT	L-aminoadipate-semialdehyde dehydrogenase-phosphopantetheinyl transferase	Q9NRN7	AASDHPPT	chr11:106075501	Wald ratio	1	0.082	0.237	7.286E-01	0.974	cis	Hospitalized COVID-19
16614_27	RSPO1	R-spondin-1	Q2MKA7	RSPO1	chr1:37634892	Inverse variance weighted	2	-0.016	0.045	7.289E-01	0.974	cis	Hospitalized COVID-19
17490_4	SH3BGRL3-like protein	SH3 domain-binding glutamic acid-rich-like protein 3	Q9H299	SH3BGRL3	chr1:26280086	Inverse variance weighted	3	0.014	0.040	7.290E-01	0.974	cis	Hospitalized COVID-19
6471_53	FHR4	Complement factor H-related protein 4	Q92496	CFHR4	chr1:196888014	Inverse variance weighted	10	0.010	0.030	7.293E-01	0.974	cis	Hospitalized COVID-19
3054_3	Haptoglobin, Mixed Type	Haptoglobin	P00738	HP	chr16:72054505	Inverse variance weighted	4	0.009	0.026	7.302E-01	0.974	cis	Hospitalized COVID-19
4960_72	annexin I	Annexin A1	P04083	ANXA1	chr9:73151865	Wald ratio	1	-0.029	0.084	7.309E-01	0.974	cis	Hospitalized COVID-19
15604_18	JNK2	Mitogen-activated protein kinase 9	P45984	MAPK9	chr5:180292099	Inverse variance weighted	2	-0.036	0.106	7.310E-01	0.974	cis	Hospitalized COVID-19
14655_1	DJC17	DnaJ homolog subfamily C member 17	Q9NVM6	DNAJC17	chr15:40807478	Inverse variance weighted	2	0.034	0.098	7.321E-01	0.974	cis	Hospitalized COVID-19
8841_65	CILP2	Cartilage intermediate layer protein 2	Q8IUL8	CILP2	chr19:19538248	Wald ratio	1	-0.051	0.148	7.324E-01	0.974	cis	Hospitalized COVID-19
5102_55	MICB	MHC class I polypeptide-related sequence B	Q29980	MICB	chr6:31494881	Inverse variance weighted	6	0.013	0.038	7.340E-01	0.974	cis	Hospitalized COVID-19
5980_55	BOLA3	BolA-like protein 3	Q53S33	BOLA3	chr2:74147912	Wald ratio	1	0.029	0.086	7.342E-01	0.974	cis	Hospitalized COVID-19
9316_67	WFDC1	WAP four-disulfide core domain protein 1	Q9HC57	WFDC1	chr16:84294846	Inverse variance weighted	5	0.013	0.039	7.345E-01	0.974	cis	Hospitalized COVID-19
12714_38	AP1G2	AP-1 complex subunit gamma-like 2	O75843	AP1G2	chr14:23568070	Inverse variance weighted	3	0.019	0.057	7.345E-01	0.974	cis	Hospitalized COVID-19
12764_3	ELMO1	Engulfment and cell motility protein 1	Q92556	ELMO1	chr7:37449223	Inverse variance weighted	3	-0.030	0.088	7.348E-01	0.974	cis	Hospitalized COVID-19
14048_7	IL-1 R AcP	Interleukin-1 Receptor accessory protein	Q9NPH3	IL1RAP	chr3:190514051	Inverse variance weighted	6	-0.005	0.016	7.353E-01	0.974	cis	Hospitalized COVID-19
12356_65	Sorcin	Sorcin	P30626	SRI	chr7:88226993	Wald ratio	1	0.030	0.088	7.356E-01	0.974	cis	Hospitalized COVID-19
9964_10	MYZAP	Myocardial zonula adherens protein	P0CAP1	MYZAP	chr15:57591904	Wald ratio	1	-0.052	0.155	7.365E-01	0.974	cis	Hospitalized COVID-19
10627_87	APLP2	Amyloid-like protein 2	Q06481	APLP2	chr11:130068147	Inverse variance weighted	2	0.049	0.145	7.366E-01	0.974	cis	Hospitalized COVID-19
13059_33	RIFK	Riboflavin kinase	Q969G6	RFK	chr9:76394517	Inverse variance weighted	2	-0.034	0.102	7.374E-01	0.974	cis	Hospitalized COVID-19
2900_53	HCC-1	C-C motif chemokine 14	Q16627	CCL14	chr17:35987004	Inverse variance weighted	4	0.026	0.079	7.376E-01	0.974	cis	Hospitalized COVID-19
11659_31	EPN4	Clathrin interactor 1	Q14677	CLINT1	chr5:157859145	Wald ratio	1	-0.070	0.210	7.385E-01	0.974	cis	Hospitalized COVID-19
3449_58	Kallistatin	Kallistatin	P29622	SERPINA4	chr14:94561442	Inverse variance weighted	7	0.013	0.040	7.391E-01	0.974	cis	Hospitalized COVID-19

17453_34	Ceruloplasmin	Ceruloplasmin	P00450	CP	chr3:149221829	Inverse variance weighted	3	-0.029	0.086	7.391E-01	0.974	cis	Hospitalized COVID-19
13982_33	RGS18	Regulator of G-protein signaling 18	Q9NS28	RGS18	chr1:192158462	Wald ratio	1	-0.057	0.173	7.394E-01	0.974	cis	Hospitalized COVID-19
3132_1	VEGF-C	Vascular endothelial growth factor C	P49767	VEGFC	chr4:176792922	Wald ratio	1	-0.042	0.126	7.397E-01	0.974	cis	Hospitalized COVID-19
7156_2	FUT10	Alpha-(1,3)-fucosyltransferase 10	Q6P4F1	FUT10	chr8:33473146	Inverse variance weighted	7	0.007	0.023	7.400E-01	0.974	cis	Hospitalized COVID-19
9545_156	Granzyme K	Granzyme K	P49863	GZMK	chr5:55024256	Wald ratio	1	0.024	0.072	7.403E-01	0.974	cis	Hospitalized COVID-19
12020_39	PMGE	Bisphosphoglycerate mutase	P07738	BPGM	chr7:134646811	Wald ratio	1	0.089	0.271	7.423E-01	0.974	cis	Hospitalized COVID-19
2991_9	IL-1 sRI	Interleukin-1 receptor type 1	P14778	IL1R1	chr2:102064544	Inverse variance weighted	2	-0.024	0.074	7.425E-01	0.974	cis	Hospitalized COVID-19
6576_1	ART4	Ecto-ADP-ribosyltransferase 4	Q93070	ART4	chr12:14843526	Inverse variance weighted	12	0.006	0.018	7.432E-01	0.974	cis	Hospitalized COVID-19
3835_11	TLR2	Toll-like receptor 2	O60603	TLR2	chr4:153684070	Wald ratio	1	0.105	0.322	7.433E-01	0.974	cis	Hospitalized COVID-19
12786_61	GDE5	Glycerophosphocholine phosphodiesterase GPCPD1	Q9NPB8	GPCPD1	chr20:5611006	Inverse variance weighted	2	-0.037	0.112	7.436E-01	0.974	cis	Hospitalized COVID-19
3060_43	C9	Complement component C9	P02748	C9	chr5:39371324	Inverse variance weighted	5	-0.016	0.050	7.438E-01	0.974	cis	Hospitalized COVID-19
10558_26	PCDH9	Protocadherin-9	Q9HC56	PCDH9	chr13:67230445	Inverse variance weighted	4	-0.013	0.039	7.448E-01	0.974	cis	Hospitalized COVID-19
6379_62	ATL2	ADAMTS-like protein 2	Q86TH1	ADAMTSL2	chr9:133532164	Inverse variance weighted	2	-0.053	0.164	7.456E-01	0.974	cis	Hospitalized COVID-19
3518_54	TAFI	Carboxypeptidase B2	Q96IY4	CPB2	chr13:46105033	Inverse variance weighted	5	0.008	0.025	7.460E-01	0.974	cis	Hospitalized COVID-19
3173_49	ASAHL	N-acylethanolamine-hydrolyzing acid amidase	Q02083	NAAA	chr4:75941013	Inverse variance weighted	10	0.009	0.029	7.460E-01	0.974	cis	Hospitalized COVID-19
18338_26	IDH	Isocitrate dehydrogenase [NADP] cytoplasmic	O75874	IDH1	chr2:208266074	Inverse variance weighted	3	-0.012	0.037	7.485E-01	0.976	cis	Hospitalized COVID-19
5658_64	coagulation factor XIII B	Coagulation factor XIII B chain	P05160	F13B	chr1:197067260	Wald ratio	1	-0.032	0.101	7.485E-01	0.976	cis	Hospitalized COVID-19
7861_9	ROR2	Tyrosine-protein kinase transmembrane receptor ROR2	Q01974	ROR2	chr9:91950228	Inverse variance weighted	2	-0.029	0.091	7.500E-01	0.976	cis	Hospitalized COVID-19
10908_2	GLT13	Polypeptide N-acetylgalactosaminyltransferase 13	Q8IUC8	GALNT13	chr2:153871922	Wald ratio	1	0.061	0.191	7.509E-01	0.976	cis	Hospitalized COVID-19
10977_55	UCMA	Unique cartilage matrix-associated protein	Q8WVF2	UCMA	chr10:13234374	Inverse variance weighted	6	0.010	0.030	7.509E-01	0.976	cis	Hospitalized COVID-19
18225_13	HEBP1	Heme-binding protein 1	Q9NRV9	HEBP1	chr12:13000265	Inverse variance weighted	6	0.006	0.018	7.525E-01	0.976	cis	Hospitalized COVID-19
4440_15	FCRL3	Fc receptor-like protein 3	Q96P31	FCRL3	chr1:157700769	Inverse variance weighted	4	0.021	0.067	7.534E-01	0.976	cis	Hospitalized COVID-19
5939_42	TWEAK	Tumor necrosis factor ligand superfamily member 12	O43508	TNFSF12	chr17:7548508	Inverse variance weighted	3	-0.027	0.088	7.543E-01	0.976	cis	Hospitalized COVID-19
3314_74	GFRa-1	GDNF family receptor alpha-1	P56159	GFRA1	chr10:116276803	Wald ratio	1	0.018	0.058	7.544E-01	0.976	cis	Hospitalized COVID-19
11530_37	HEM3	Porphobilinogen deaminase	P08397	HMBS	chr11:119084866	Wald ratio	1	-0.062	0.197	7.545E-01	0.976	cis	Hospitalized COVID-19
9772_153	NLGN2:ECD	Neuroligin-2:Extracellular domain	Q8NFZ4	NLGN2	chr17:7404874	Inverse variance weighted	2	0.026	0.083	7.546E-01	0.976	cis	Hospitalized COVID-19
6207_10	prosaposin	Prosaposin	P07602	PSAP	chr10:71851251	Inverse variance weighted	3	0.010	0.033	7.552E-01	0.976	cis	Hospitalized COVID-19
8028_22	SPINK5	Serine protease inhibitor Kazal-type 5	Q9NQ38	SPINK5	chr5:148025683	Wald ratio	1	0.023	0.075	7.577E-01	0.976	cis	Hospitalized COVID-19
5637_81	NTNG1	Netrin-G1	Q9Y2I2	NTNG1	chr1:107140007	Inverse variance weighted	9	0.006	0.020	7.592E-01	0.976	cis	Hospitalized COVID-19
2771_35	IGFBP-1	Insulin-like growth factor-binding protein 1	P08833	IGFBP1	chr7:45888360	Wald ratio	1	0.042	0.136	7.593E-01	0.976	cis	Hospitalized COVID-19
5657_28	SIA4A	CMP-N-acetylneuraminate-beta-galactosamide-alpha-2,3-sialyltransferase 1	Q11201	ST3GAL1	chr8:133571940	Inverse variance weighted	2	-0.017	0.055	7.595E-01	0.976	cis	Hospitalized COVID-19
18413_24	ARL4D	ADP-ribosylation factor-like protein 4D	P49703	ARL4D	chr17:43398993	Wald ratio	1	0.058	0.189	7.600E-01	0.976	cis	Hospitalized COVID-19
9326_33	ITI heavy chain H2	Inter-alpha-trypsin inhibitor heavy chain H2	P19823	ITIH2	chr10:7703316	Inverse variance weighted	9	0.007	0.024	7.604E-01	0.976	cis	Hospitalized COVID-19
19154_41	Protease nexin I	Glia-derived nexin	P07093	SERPINE2	chr2:224039318	Inverse variance weighted	8	-0.007	0.023	7.619E-01	0.976	cis	Hospitalized COVID-19
2780_35	Lactoferrin	Lactotransferrin	P02788	LTF	chr3:46485234	Inverse variance weighted	3	0.034	0.112	7.622E-01	0.976	cis	Hospitalized COVID-19
18340_2	Proteasome beta chain	Proteasome subunit beta type-4	P28070	PSMB4	chr1:151399560	Inverse variance weighted	4	-0.010	0.033	7.625E-01	0.976	cis	Hospitalized COVID-19
6255_74	CPXM1	Probable carboxypeptidase X1	Q96SM3	CPXM1	chr20:2800627	Inverse variance weighted	5	-0.012	0.038	7.628E-01	0.976	cis	Hospitalized COVID-19
9383_24	CH3L2	Chitinase-3-like protein 2	Q15782	CHI3L2	chr1:111200771	Wald ratio	1	0.052	0.172	7.635E-01	0.976	cis	Hospitalized COVID-19
9337_43	TKN1	Protachykinin-1	P20366	TAC1	chr7:97732084	Inverse variance weighted	3	0.015	0.049	7.638E-01	0.976	cis	Hospitalized COVID-19
16288_17	EPHA4	Ephrin type-A receptor 4	P54764	EPHA4	chr2:221574202	Inverse variance weighted	3	0.029	0.098	7.641E-01	0.976	cis	Hospitalized COVID-19
8932_1	ENTP6	Ectonucleoside triphosphate diphosphohydrolase 6	O75354	ENTPD6	chr20:25195693	Wald ratio	1	0.033	0.109	7.644E-01	0.976	cis	Hospitalized COVID-19
12560_9	NT5C	5'(3')-deoxyribonucleotidase, cytosolic type	Q8TCD5	NT5C	chr17:75131757	Inverse variance weighted	5	-0.008	0.025	7.654E-01	0.976	cis	Hospitalized COVID-19
12659_13	GTPB9	Obg-like ATPase 1	Q9NTK5	OLA1	chr2:174248599	Wald ratio	1	-0.023	0.076	7.655E-01	0.976	cis	Hospitalized COVID-19
6388_21	CC126	Coiled-coil domain-containing protein 126	Q96EE4	CCDC126	chr7:23597382	Inverse variance weighted	4	0.010	0.034	7.655E-01	0.976	cis	Hospitalized COVID-19
5532_53	bFGF-R	Fibroblast growth factor receptor 1	P11362	FGFR1	chr8:38468834	Inverse variance weighted	2	-0.030	0.101	7.656E-01	0.976	cis	Hospitalized COVID-19
15370_5	BOLA1	BolA-like protein 1	Q9Y3E2	BOLA1	chr1:149887890	Inverse variance weighted	2	0.011	0.039	7.667E-01	0.976	cis	Hospitalized COVID-19
18408_26	ARF4	ADP-ribosylation factor 4	P18085	ARF4	chr3:57598220	Wald ratio	1	0.041	0.139	7.681E-01	0.976	cis	Hospitalized COVID-19
15579_26	ENPP6	Ectonucleotide pyrophosphatase/phosphodiesterase family member 6	Q6UWR7	ENPP6	chr4:184221230	Wald ratio	1	0.056	0.190	7.684E-01	0.976	cis	Hospitalized COVID-19
5076_53	EPHAA	Ephrin type-A receptor 10	Q5JZY3	EPHA10	chr1:37765133	Wald ratio	1	-0.042	0.145	7.711E-01	0.976	cis	Hospitalized COVID-19
4929_55	SHBG	Sex hormone-binding globulin	P04278	SHBG	chr17:7613946	Inverse variance weighted	2	0.030	0.102	7.714E-01	0.976	cis	Hospitalized COVID-19
8243_55	TATI	Serine protease inhibitor Kazal-type 1	P00995	SPINK1	chr5:147831671	Inverse variance weighted	2	0.032	0.111	7.714E-01	0.976	cis	Hospitalized COVID-19
16908_5	OMGP	Oligodendrocyte-myelin glycoprotein	P23515	OMG	chr17:31297539	Wald ratio	1	-0.044	0.150	7.716E-01	0.976	cis	Hospitalized COVID-19
4435_66	ENPP7	Ectonucleotide pyrophosphatase/phosphodiesterase family member 7	Q6UWV6	ENPP7	chr17:79730943	Inverse variance weighted	9	0.007	0.026	7.716E-01	0.976	cis	Hospitalized COVID-19
17331_138	KREM1	Kremen protein 1	Q96MU8	KREMEN1	chr22:29073035	Inverse variance weighted	3	-0.016	0.054	7.717E-01	0.976	cis	Hospitalized COVID-19
8469_41	IGFBP-2	Insulin-like growth factor-binding protein 2	P18065	IGFBP2	chr2:216632828	Inverse variance weighted	2	-0.059	0.203	7.719E-01	0.976	cis	Hospitalized COVID-19
8969_49	CD14	Monocyte differentiation antigen CD14	P08571	CD14	chr5:140633700	Wald ratio	1	-0.077	0.265	7.721E-01	0.976	cis	Hospitalized COVID-19
7141_21	MGT4B	Alpha-1,3-mannosyl-glycoprotein 4-beta-N-acetylglucosaminyltransferase B	Q9UQ53	MGAT4B	chr5:179806952	Inverse variance weighted	4	-0.010	0.033	7.724E-01	0.976	cis	Hospitalized COVID-19
3391_10	PK3CG	Phosphatidylinositol 4,5-bisphosphate 3-kinase catalytic subunit gamma isoform	P48736	PIK3CG	chr7:106865278	Wald ratio	1	-0.057	0.197	7.730E-01	0.976	cis	Hospitalized COVID-19
17408_2	PMM1	Phosphomannomutase 1	Q92871	PMM1	chr22:41589871	Wald ratio	1	0.057	0.197	7.745E-01	0.976	cis	Hospitalized COVID-19
4971_1	CATZ	Cathepsin Z	Q9UBR2	CTSZ	chr20:59008238	Inverse variance weighted	5	-0.009	0.033	7.749E-01	0.976	cis	Hospitalized COVID-19
13624_17	NADK	NAD kinase	O95544	NADK	chr1:1780457	Wald ratio	1	-0.022	0.079	7.757E-01	0.976	cis	Hospitalized COVID-19
4337_49	CRP	C-reactive protein	P02741	CRP	chr1:159714589	Inverse variance weighted	2	-0.016	0.058	7.760E-01	0.976	cis	Hospitalized COVID-19
12657_2	FCL	GDP-L-fucose synthase	Q13630	GFUS	chr8:143618048	Wald ratio	1	0.069	0.243	7.761E-01	0.976	cis	Hospitalized COVID-19
10514_5	PGD2 synthase	Prostaglandin-H2 D-isomerase	P41222	PTGDS	chr9:136975092	Wald ratio	1	0.065	0.229	7.764E-01	0.976	cis	Hospitalized COVID-19
19289_29	DCUP	Uroporphyrinogen decarboxylase	P06132	UROD	chr1:45010950	Inverse variance weighted	3	0.011	0.040	7.772E-01	0.976	cis	Hospitalized COVID-19
4209_60	DRG-1	Vacuolar protein sorting-associated protein VTA1 homolog	Q9NP79	VTA1	chr6:142147162	Wald ratio	1	0.054	0.192	7.773E-01	0.976	cis	Hospitalized COVID-19
17764_108	RHOC	Rho-related GTP-binding protein RhoC	P08134	RHOC	chr1:112707434	Wald ratio	1	0.027	0.094	7.775E-01	0.976	cis	Hospitalized COVID-19
13983_27	QOR	Quinone oxidoreductase	Q08257	CRYZ	chr1:74733408	Inverse variance weighted	7	0.007	0.026	7.783E-01	0.976	cis	Hospitalized COVID-19
19130_81	SPB8	Serpin B8	P50452	SERPINB8	chr18:63970029	Inverse variance weighted	3	-0.013	0.047	7.787E-01	0.976	cis	Hospitalized COVID-19
10396_6	Mcl-1	Induced myeloid leukemia cell differentiation protein Mcl-1	Q07820	MCL1	chr1:150579738	Wald ratio	1	0.023	0.080	7.791E-01	0.976	cis	Hospitalized COVID-19
10462_14	INSL5	Insulin-like peptide INSL5	Q9Y5Q6	INSL5	chr1:66801276	Wald ratio	1	-0.051	0.181	7.797E-01	0.976	cis	Hospitalized COVID-19
16825_20	ATX3	Ataxin-3	P54252	ATXN3	chr14:92106621	Inverse variance weighted	4	0.017	0.063	7.806E-01	0.976	cis	Hospitalized COVID-19
4131_72	Fibronectin	Fibronectin	P02751	FN1	chr2:215436073	Inverse variance weighted	2	0.062	0.222	7.808E-01	0.976	cis	Hospitalized COVID-19
5713_9	IFN-lambda 3	Interferon lambda-3	Q8IZI9	IFNL3	chr19:39245250	Wald ratio	1	-0.060	0.218	7.823E-01	0.976	cis	Hospitalized COVID-19
5737_61	SEM4D	Semaphorin-4D	Q92854	SEMA4D	chr9:89498130	Inverse variance weighted	6	-0.011	0.041	7.829E-01	0.976	cis	Hospitalized COVID-19
5731_1	ISK6	Serine protease inhibitor Kazal-type 6	Q6UWN8	SPINK6	chr5:148202794	Inverse variance weighted	9	-0.008	0.030	7.837E-01	0.976	cis	Hospitalized COVID-19
8289_8	GPNMB:ECD	Transmembrane glycoprotein NMB:Extracellular domain	Q14956	GPNMB	chr7:23235967	Inverse variance weighted	5	-0.011	0.040	7.840E-01	0.976	cis	Hospitalized COVID-19
11606_22	DNJB6	DnaJ homolog subfamily B member 6	O75190	DNAJB6	chr7:157335381	Wald ratio	1	0.055	0.202	7.850E-01	0.976	cis	Hospitalized COVID-19

5682_13	VASN	Vasorin	Q6EMK4	VASN	chr16:4371848	Wald ratio	1	0.039	0.142	7.850E-01	0.976	cis	Hospitalized COVID-19
7999_23	CD39	Ectonucleoside triphosphate diphosphohydrolase 1	P49961	ENTPD1	chr10:95711779	Inverse variance weighted	3	0.023	0.085	7.854E-01	0.976	cis	Hospitalized COVID-19
7099_33	FSTL5	Follistatin-related protein 5	Q8N475	FSTL5	chr4:162164004	Wald ratio	1	0.028	0.105	7.862E-01	0.976	cis	Hospitalized COVID-19
3291_30	CD23	Low affinity immunoglobulin epsilon Fc receptor	P06734	FCER2	chr19:7702146	Inverse variance weighted	7	0.011	0.040	7.866E-01	0.976	cis	Hospitalized COVID-19
4984_83	Esterase D	S-formylglutathione hydrolase	P10768	ESD	chr13:46797420	Inverse variance weighted	3	-0.011	0.042	7.878E-01	0.977	cis	Hospitalized COVID-19
7551_33	LRC32	Leucine-rich repeat-containing protein 32	Q14392	LRRC32	chr11:76670747	Wald ratio	1	-0.043	0.162	7.886E-01	0.977	cis	Hospitalized COVID-19
2381_52	C5	Complement C5	P01031	C5	chr9:121075195	Inverse variance weighted	2	0.041	0.154	7.907E-01	0.977	cis	Hospitalized COVID-19
9294_45	MFAP2	Microfibrillar-associated protein 2	P55001	MFAP2	chr1:16980632	Inverse variance weighted	2	-0.045	0.169	7.909E-01	0.977	cis	Hospitalized COVID-19
18280_29	DTD2	Putative D-tyrosyl-tRNA(Tyr) deacylase 2	Q96FN9	DTD2	chr14:31457506	Inverse variance weighted	2	-0.023	0.088	7.916E-01	0.977	cis	Hospitalized COVID-19
17712_7	IDI1	Isopentenyl-diphosphate Delta-isomerase 1	Q13907	IDI1	chr10:1049119	Wald ratio	1	-0.039	0.147	7.921E-01	0.977	cis	Hospitalized COVID-19
8660_5	OLFL3	Olfactomedin-like protein 3	Q9NRN5	OLFML3	chr1:113979391	Inverse variance weighted	2	0.026	0.100	7.928E-01	0.977	cis	Hospitalized COVID-19
3313_21	FCN2	Ficolin-2	Q15485	FCN2	chr9:134880810	Inverse variance weighted	3	0.018	0.068	7.928E-01	0.977	cis	Hospitalized COVID-19
8476_11	CgA	Chromogranin-A	P10645	CHGA	chr14:92923150	Inverse variance weighted	4	0.018	0.069	7.936E-01	0.977	cis	Hospitalized COVID-19
6247_9	SIRB1	Signal-regulatory protein beta-1	O00241	SIRPB1	chr20:1620061	Inverse variance weighted	9	0.004	0.014	7.940E-01	0.977	cis	Hospitalized COVID-19
6408_2	INHBC	Inhibin beta C chain	P55103	INHBC	chr12:57434784	Inverse variance weighted	2	0.035	0.135	7.941E-01	0.977	cis	Hospitalized COVID-19
11104_13	YKL-40	Chitinase-3-like protein 1	P36222	CHI3L1	chr1:203186704	Inverse variance weighted	7	0.004	0.017	7.941E-01	0.977	cis	Hospitalized COVID-19
3607_71	DKK3	Dickkopf-related protein 3	Q9UBP4	DKK3	chr11:12009769	Inverse variance weighted	4	-0.011	0.042	7.949E-01	0.977	cis	Hospitalized COVID-19
6556_5	ENPP5	Ectonucleotide pyrophosphatase/phosphodiesterase family member 5	Q9UJA9	ENPP5	chr6:46170980	Inverse variance weighted	7	-0.005	0.018	7.955E-01	0.977	cis	Hospitalized COVID-19
19238_12	GLNA	Glutamine synthetase	P15104	GLUL	chr1:182392206	Wald ratio	1	0.054	0.208	7.960E-01	0.977	cis	Hospitalized COVID-19
19213_1	ISK4	Serine protease inhibitor Kazal-type 4	O60575	SPINK4	chr9:33218365	Wald ratio	1	-0.013	0.050	7.979E-01	0.979	cis	Hospitalized COVID-19
5231_79	PCSK9	Proprotein convertase subtilisin/kexin type 9	Q8NBP7	PCSK9	chr1:55039447	Inverse variance weighted	6	0.009	0.034	8.001E-01	0.981	cis	Hospitalized COVID-19
2654_19	TNF sR-I	Tumor necrosis factor receptor superfamily member 1A	P19438	TNFRSF1A	chr12:6342114	Wald ratio	1	0.023	0.090	8.013E-01	0.981	cis	Hospitalized COVID-19
3191_50	WFKN1	WAP, kazal, immunoglobulin, kunitz and NTR domain-containing protein 1	Q96NZ8	WFIKKN1	chr16:629239	Inverse variance weighted	2	-0.019	0.075	8.015E-01	0.981	cis	Hospitalized COVID-19
6947_4	SIA10	Type 2 lactosamine alpha-2,3-sialyltransferase	Q9Y274	ST3GAL6	chr3:98732236	Inverse variance weighted	9	-0.004	0.017	8.047E-01	0.983	cis	Hospitalized COVID-19
2797_56	Apo B	Apolipoprotein B	P04114	APOB	chr2:21044073	Wald ratio	1	-0.034	0.139	8.051E-01	0.983	cis	Hospitalized COVID-19
18878_15	GREM1	Gremlin-1	O60565	GREM1	chr15:32718004	Inverse variance weighted	8	-0.005	0.022	8.054E-01	0.983	cis	Hospitalized COVID-19
6392_7	WISP-2	WNT1-inducible-signaling pathway protein 2	O76076	CCN5	chr20:44714844	Inverse variance weighted	5	0.010	0.042	8.059E-01	0.983	cis	Hospitalized COVID-19
11441_11	PYGL	Glycogen phosphorylase, liver form	P06737	PYGL	chr14:50944483	Inverse variance weighted	2	-0.020	0.083	8.065E-01	0.983	cis	Hospitalized COVID-19
18882_7	CSTN2	Calsyntenin-2	Q9H4D0	CLSTN2	chr3:139935185	Inverse variance weighted	7	-0.014	0.059	8.067E-01	0.983	cis	Hospitalized COVID-19
18387_7	suppression of tumorigenicity 13	Hsc70-interacting protein	P50502	ST13	chr22:40856639	Wald ratio	1	-0.047	0.194	8.071E-01	0.983	cis	Hospitalized COVID-19
10835_25	A4GCT	Alpha-1,4-N-acetylglucosaminyltransferase	Q9UNA3	A4GNT	chr3:138132390	Wald ratio	1	-0.038	0.157	8.076E-01	0.983	cis	Hospitalized COVID-19
18289_16	MIP-5	C-C motif chemokine 15	Q16663	CCL15	chr17:36001553	Inverse variance weighted	5	0.013	0.057	8.128E-01	0.989	cis	Hospitalized COVID-19
17377_1	Aldose reductase-like C3	Aldo-keto reductase family 1 member C3	P42330	AKR1C3	chr10:5035354	Inverse variance weighted	4	-0.027	0.115	8.139E-01	0.989	cis	Hospitalized COVID-19
8013_9	LMA2L	VIP36-like protein	Q9H0V9	LMAN2L	chr2:96740064	Inverse variance weighted	2	-0.026	0.112	8.160E-01	0.991	cis	Hospitalized COVID-19
2632_5	IL-12 Rb1	Interleukin-12 receptor subunit beta-1	P42701	IL12RB1	chr19:18098944	Wald ratio	1	-0.030	0.130	8.169E-01	0.991	cis	Hospitalized COVID-19
4564_2	PLXC1	Plexin-C1	O60486	PLXNC1	chr12:94148577	Inverse variance weighted	11	-0.004	0.016	8.174E-01	0.991	cis	Hospitalized COVID-19
3803_10	CYTD	Cystatin-D	P28325	CST5	chr20:23879748	Inverse variance weighted	11	-0.010	0.044	8.198E-01	0.993	cis	Hospitalized COVID-19
9177_6	FAM3B	Protein FAM3B	P58499	FAM3B	chr21:41304212	Inverse variance weighted	7	0.009	0.040	8.199E-01	0.993	cis	Hospitalized COVID-19
3320_49	IGFBP-7	Insulin-like growth factor-binding protein 7	Q16270	IGFBP7	chr4:57110385	Inverse variance weighted	5	0.011	0.051	8.220E-01	0.993	cis	Hospitalized COVID-19
2737_22	NovH	Protein NOV homolog	P48745	CCN3	chr8:119416446	Wald ratio	1	0.033	0.147	8.229E-01	0.993	cis	Hospitalized COVID-19
18214_2	GSH0	Glutamate--cysteine ligase regulatory subunit	P48507	GCLM	chr1:93909456	Inverse variance weighted	2	0.027	0.121	8.231E-01	0.993	cis	Hospitalized COVID-19
2789_26	MMP-7	Matrilysin	P09237	MMP7	chr11:102530750	Inverse variance weighted	4	0.011	0.049	8.237E-01	0.993	cis	Hospitalized COVID-19
6998_106	HAAH	Aspartyl/asparaginyl beta-hydroxylase	Q12797	ASPH	chr8:61714640	Inverse variance weighted	2	-0.023	0.103	8.240E-01	0.993	cis	Hospitalized COVID-19
9595_11	B4GT2	Beta-1,4-galactosyltransferase 2	O60909	B4GALT2	chr1:43978943	Inverse variance weighted	4	0.008	0.037	8.252E-01	0.993	cis	Hospitalized COVID-19
5457_5	COLEC12	Collectin-12	Q5KU26	COLEC12	chr18:500722	Inverse variance weighted	3	-0.026	0.116	8.264E-01	0.993	cis	Hospitalized COVID-19
19581_15	IGFBP-5	Insulin-like growth factor-binding protein 5	P24593	IGFBP5	chr2:216695549	Wald ratio	1	-0.044	0.201	8.265E-01	0.993	cis	Hospitalized COVID-19
8842_16	GRM1C	GRAM domain-containing protein 1C	Q8IYS0	GRAMD1C	chr3:113828182	Inverse variance weighted	5	-0.014	0.062	8.267E-01	0.993	cis	Hospitalized COVID-19
16919_1	ACBP	Acyl-CoA-binding protein	P07108	DBI	chr2:119366924	Inverse variance weighted	2	0.010	0.047	8.268E-01	0.993	cis	Hospitalized COVID-19
15384_15	KLOTHO	Klotho	Q9UEF7	KL	chr13:33016423	Inverse variance weighted	4	0.013	0.058	8.270E-01	0.993	cis	Hospitalized COVID-19
8398_277	RAR-responsive protein TIG1	Retinoic acid receptor responder protein 1	P49788	RARRES1	chr3:158732489	Inverse variance weighted	6	0.007	0.033	8.278E-01	0.993	cis	Hospitalized COVID-19
17325_10	KGUA	Guanylate kinase	Q16774	GUK1	chr1:228139962	Inverse variance weighted	5	-0.018	0.084	8.298E-01	0.993	cis	Hospitalized COVID-19
12382_2	DDX58	Probable ATP-dependent RNA helicase DDX58	O95786	DDX58	chr9:32526208	Inverse variance weighted	2	-0.013	0.059	8.307E-01	0.993	cis	Hospitalized COVID-19
16060_99	NID2	Nidogen-2	Q14112	NID2	chr14:52069059	Inverse variance weighted	6	0.006	0.027	8.318E-01	0.993	cis	Hospitalized COVID-19
4834_61	Epithelial cell kinase	Ephrin type-A receptor 2	P29317	EPHA2	chr1:16156069	Inverse variance weighted	3	-0.015	0.069	8.320E-01	0.993	cis	Hospitalized COVID-19
11187_11	CL12A	C-type lectin domain family 12 member A	Q5QGZ9	CLEC12A	chr12:9951316	Inverse variance weighted	6	0.007	0.035	8.339E-01	0.993	cis	Hospitalized COVID-19
6544_33	NELL1	Protein kinase C-binding protein NELL1	Q92832	NELL1	chr11:20669551	Inverse variance weighted	8	-0.005	0.026	8.343E-01	0.993	cis	Hospitalized COVID-19
8916_32	STIM1:CD	Stromal interaction molecule 1:Cytoplasmic domain	Q13586	STIM1	chr11:3854527	Wald ratio	1	-0.023	0.110	8.346E-01	0.993	cis	Hospitalized COVID-19
16057_6	IGF-II receptor	Cation-independent mannose-6-phosphate receptor	P11717	IGF2R	chr6:159969082	Inverse variance weighted	6	-0.008	0.040	8.351E-01	0.993	cis	Hospitalized COVID-19
9832_33	HGD	Homogentisate 1,2-dioxygenase	Q93099	HGD	chr3:120682269	Inverse variance weighted	2	-0.029	0.139	8.374E-01	0.993	cis	Hospitalized COVID-19
9021_1	TIM-1	Hepatitis A virus cellular receptor 1	Q96D42	HAVCR1	chr5:157069396	Inverse variance weighted	6	0.007	0.032	8.374E-01	0.993	cis	Hospitalized COVID-19
17694_32	PSME2	Proteasome activator complex subunit 2	Q9UL46	PSME2	chr14:24147570	Inverse variance weighted	3	0.012	0.057	8.383E-01	0.993	cis	Hospitalized COVID-19
16773_29	SCUB3	Signal peptide, CUB and EGF-like domain-containing protein 3	Q8IX30	SCUBE3	chr6:35213956	Inverse variance weighted	3	0.009	0.046	8.393E-01	0.993	cis	Hospitalized COVID-19
8364_74	UST	Uronyl 2-sulfotransferase	Q9Y2C2	UST	chr6:148747030	Inverse variance weighted	3	0.022	0.107	8.399E-01	0.993	cis	Hospitalized COVID-19
9275_2	Siglec-5	Sialic acid-binding Ig-like lectin 5	O15389	SIGLEC5	chr19:51630401	Wald ratio	1	-0.039	0.195	8.404E-01	0.993	cis	Hospitalized COVID-19
16616_137	ENOB	Beta-enolase	P13929	ENO3	chr17:4948092	Inverse variance weighted	2	0.007	0.033	8.406E-01	0.993	cis	Hospitalized COVID-19
15602_43	IL-6 sRa	Interleukin-6 receptor subunit alpha	P08887	IL6R	chr1:154405193	Inverse variance weighted	6	-0.009	0.043	8.407E-01	0.993	cis	Hospitalized COVID-19
6385_63	VWA1	von Willebrand factor A domain-containing protein 1	Q6PCB0	VWA1	chr1:1434861	Inverse variance weighted	5	-0.011	0.055	8.408E-01	0.993	cis	Hospitalized COVID-19
3499_77	IL-17B	Interleukin-17B	Q9UHF5	IL17B	chr5:149404202	Wald ratio	1	0.028	0.141	8.421E-01	0.993	cis	Hospitalized COVID-19
5798_3	BID	BH3-interacting domain death agonist	P55957	BID	chr22:17774770	Wald ratio	1	0.027	0.138	8.423E-01	0.993	cis	Hospitalized COVID-19
12381_26	CBR1	Carbonyl reductase [NADPH] 1	P16152	CBR1	chr21:36069941	Inverse variance weighted	5	0.007	0.033	8.433E-01	0.993	cis	Hospitalized COVID-19
4151_6	Plasminogen	Plasminogen	P00747	PLG	chr6:160702194	Inverse variance weighted	3	-0.028	0.142	8.434E-01	0.993	cis	Hospitalized COVID-19
16753_46	CO6A2	Collagen alpha-2(VI) chain	P12110	COL6A2	chr21:46098112	Inverse variance weighted	4	-0.013	0.065	8.446E-01	0.993	cis	Hospitalized COVID-19
9470_15	MET24	Methyltransferase-like protein 24	Q5JXM2	METTL24	chr6:110358349	Inverse variance weighted	4	-0.016	0.080	8.455E-01	0.993	cis	Hospitalized COVID-19
4546_27	EMR2	Adhesion G protein-coupled receptor E2	Q9UHX3	ADGRE2	chr19:14778560	Inverse variance weighted	5	-0.008	0.041	8.457E-01	0.993	cis	Hospitalized COVID-19
5085_18	IL-20 Ra	Interleukin-20 receptor subunit alpha	Q9UHF4	IL20RA	chr6:137045180	Wald ratio	1	0.041	0.209	8.461E-01	0.993	cis	Hospitalized COVID-19
3474_19	Thrombospondin-1	Thrombospondin-1	P07996	THBS1	chr15:39581079	Wald ratio	1	0.042	0.215	8.463E-01	0.993	cis	Hospitalized COVID-19
11514_196	CD59	CD59 glycoprotein	P13987	CD59	chr11:33736479	Inverse variance weighted	3	-0.012	0.062	8.470E-01	0.993	cis	Hospitalized COVID-19
8983_7	GOLM1	Golgi membrane protein 1	Q8NBJ4	GOLM1	chr9:86100173	Wald ratio	1	-0.044	0.229	8.483E-01	0.993	cis	Hospitalized COVID-19

6713_4	LRP11	Low-density lipoprotein receptor-related protein 11	Q86VZ4	LRP11	chr6:149864359	Inverse variance weighted	8	0.006	0.029	8.488E-01	0.993	cis	Hospitalized COVID-19
8309_12	HYAL1	Hyaluronidase-1	Q12794	HYAL1	chr3:50312381	Inverse variance weighted	2	0.030	0.156	8.489E-01	0.993	cis	Hospitalized COVID-19
18243_9	Cytidylate kinase	UMP-CMP kinase	P30085	CMPK1	chr1:47333790	Wald ratio	1	0.025	0.131	8.490E-01	0.993	cis	Hospitalized COVID-19
9216_100	PLXB2	Plexin-B2	O15031	PLXNB2	chr22:50307646	Inverse variance weighted	5	-0.006	0.033	8.495E-01	0.993	cis	Hospitalized COVID-19
7227_75	COCH	Cochlin	O43405	COCH	chr14:30874514	Inverse variance weighted	4	-0.009	0.046	8.498E-01	0.993	cis	Hospitalized COVID-19
2778_10	IL-22	Interleukin-22	Q9GZX6	IL22	chr12:68253604	Wald ratio	1	0.033	0.177	8.510E-01	0.993	cis	Hospitalized COVID-19
5060_62	B7-H1	Programmed cell death 1 ligand 1	Q9NZQ7	CD274	chr9:5450503	Inverse variance weighted	2	0.018	0.096	8.512E-01	0.993	cis	Hospitalized COVID-19
18819_21	PPIC	Peptidyl-prolyl cis-trans isomerase C	P45877	PPIC	chr5:123036725	Inverse variance weighted	3	0.005	0.026	8.514E-01	0.993	cis	Hospitalized COVID-19
17706_4	PPR1A	Protein phosphatase 1 regulatory subunit 1A	Q13522	PPP1R1A	chr12:54588659	Wald ratio	1	-0.037	0.196	8.514E-01	0.993	cis	Hospitalized COVID-19
18183_3	ARH	Low density lipoprotein receptor adapter protein 1	Q5SW96	LDLRAP1	chr1:25543606	Inverse variance weighted	2	-0.021	0.112	8.516E-01	0.993	cis	Hospitalized COVID-19
6086_15	CRDL2	Chordin-like protein 2	Q6WN34	CHRDL2	chr11:74731426	Inverse variance weighted	5	0.006	0.035	8.521E-01	0.993	cis	Hospitalized COVID-19
17332_3	ARHL2	Poly(ADP-ribose) glycohydrolase ARH3	Q9NX46	ADPRS	chr1:36088892	Inverse variance weighted	3	-0.013	0.070	8.529E-01	0.993	cis	Hospitalized COVID-19
3343_1	Aminoacylase-1	Aminoacylase-1	Q03154	ACY1	chr3:51983340	Wald ratio	1	0.011	0.057	8.530E-01	0.993	cis	Hospitalized COVID-19
5704_74	Granzyme M	Granzyme M	P51124	GZMM	chr19:544034	Inverse variance weighted	2	0.035	0.191	8.533E-01	0.993	cis	Hospitalized COVID-19
3336_50	TFPI	Tissue factor pathway inhibitor	P10646	TFPI	chr2:187565760	Wald ratio	1	-0.023	0.126	8.534E-01	0.993	cis	Hospitalized COVID-19
5090_49	ILT-2	Leukocyte immunoglobulin-like receptor subfamily B member 1	Q8NHL6	LILRB1	chr19:54617158	Inverse variance weighted	7	-0.006	0.032	8.535E-01	0.993	cis	Hospitalized COVID-19
3033_57	Galectin-2	Galectin-2	P05162	LGALS2	chr22:37582616	Inverse variance weighted	2	0.037	0.199	8.539E-01	0.993	cis	Hospitalized COVID-19
2836_68	Lipocalin 2	Neutrophil gelatinase-associated lipocalin	P80188	LCN2	chr9:128149071	Wald ratio	1	0.028	0.151	8.543E-01	0.993	cis	Hospitalized COVID-19
9350_3	FSTL4	Follistatin-related protein 4	Q6MZW2	FSTL4	chr5:133612541	Inverse variance weighted	2	-0.024	0.129	8.548E-01	0.993	cis	Hospitalized COVID-19
12662_82	ECH1	Delta(3,5)-Delta(2,4)-dienoyl-CoA isomerase, mitochondrial	Q13011	ECH1	chr19:38831841	Inverse variance weighted	2	0.006	0.034	8.549E-01	0.993	cis	Hospitalized COVID-19
11351_233	NHEJ1	Non-homologous end-joining factor 1	Q9H9Q4	NHEJ1	chr2:219160869	Inverse variance weighted	3	0.016	0.086	8.563E-01	0.993	cis	Hospitalized COVID-19
13112_179	FSTL1	Follistatin-related protein 1	Q12841	FSTL1	chr3:120450993	Inverse variance weighted	2	-0.017	0.096	8.570E-01	0.993	cis	Hospitalized COVID-19
6416_8	GKN2	Gastrokine-2	Q86XP6	GKN2	chr2:68952893	Inverse variance weighted	4	0.006	0.031	8.572E-01	0.993	cis	Hospitalized COVID-19
3050_7	vWF	von Willebrand factor	P04275	VWF	chr12:6124770	Inverse variance weighted	4	0.021	0.119	8.574E-01	0.993	cis	Hospitalized COVID-19
15453_3	a1-Microglobulin	Alpha-1-microglobulin	P02760	AMBP	chr9:114078328	Inverse variance weighted	2	0.027	0.151	8.586E-01	0.993	cis	Hospitalized COVID-19
3007_7	Siglec-9	Sialic acid-binding Ig-like lectin 9	Q9Y336	SIGLEC9	chr19:51124906	Inverse variance weighted	6	-0.007	0.037	8.590E-01	0.993	cis	Hospitalized COVID-19
3166_92	Siglec-3	Myeloid cell surface antigen CD33	P20138	CD33	chr19:51225064	Inverse variance weighted	6	0.004	0.022	8.604E-01	0.994	cis	Hospitalized COVID-19
15686_49	INHBC	Inhibin beta C chain	P55103	INHBC	chr12:57434784	Inverse variance weighted	14	-0.003	0.019	8.613E-01	0.994	cis	Hospitalized COVID-19
14100_63	C1QC	Complement C1q subcomponent subunit C	P02747	C1QC	chr1:22643014	Inverse variance weighted	9	0.005	0.028	8.620E-01	0.994	cis	Hospitalized COVID-19
16079_2	TEC	Tyrosine-protein kinase Tec	P42680	TEC	chr4:48269838	Inverse variance weighted	2	0.033	0.192	8.620E-01	0.994	cis	Hospitalized COVID-19
2999_6	LSAMP	Limbic system-associated membrane protein	Q13449	LSAMP	chr3:117139389	Inverse variance weighted	4	0.011	0.062	8.631E-01	0.994	cis	Hospitalized COVID-19
15610_72	LAP	Cytosol aminopeptidase	P28838	LAP3	chr4:17577198	Wald ratio	1	0.035	0.205	8.633E-01	0.994	cis	Hospitalized COVID-19
11109_56	SVEP1:Sushi 15-18	Sushi, von Willebrand factor type A, EGF and pentraxin domain-containing protein 1:Sushi 15-18	Q4LDE5	SVEP1	chr9:110579880	Inverse variance weighted	4	0.018	0.105	8.646E-01	0.995	cis	Hospitalized COVID-19
15324_58	Ferritin light chain	Ferritin light chain	P02792	FTL	chr19:48965309	Wald ratio	1	0.034	0.203	8.656E-01	0.995	cis	Hospitalized COVID-19
13116_25	CD177	CD177 antigen	Q8N6Q3	CD177	chr19:43353686	Inverse variance weighted	8	-0.004	0.027	8.682E-01	0.997	cis	Hospitalized COVID-19
13465_5	CCP1	Calcipressin-1	P53805	RCAN1	chr21:34615113	Wald ratio	1	0.028	0.171	8.683E-01	0.997	cis	Hospitalized COVID-19
13544_9	HMHA1	Rho GTPase-activating protein 45	Q92619	ARHGAP45	chr19:1065923	Wald ratio	1	0.013	0.079	8.687E-01	0.997	cis	Hospitalized COVID-19
17384_110	K6PF	ATP-dependent 6-phosphofructokinase, muscle type	P08237	PFKM	chr12:48105139	Wald ratio	1	0.017	0.103	8.690E-01	0.997	cis	Hospitalized COVID-19
10815_2	HABP4	Intracellular hyaluronan-binding protein 4	Q5JVS0	HABP4	chr9:96450169	Inverse variance weighted	3	-0.006	0.040	8.715E-01	0.998	cis	Hospitalized COVID-19
4374_45	MIC-1	Growth/differentiation factor 15	Q99988	GDF15	chr19:18374731	Inverse variance weighted	7	-0.005	0.029	8.715E-01	0.998	cis	Hospitalized COVID-19
16609_106	KIRR2	Kin of IRRE-like protein 2	Q6UWL6	KIRREL2	chr19:35855861	Inverse variance weighted	5	-0.006	0.035	8.725E-01	0.998	cis	Hospitalized COVID-19
10818_36	ASM	Sphingomyelin phosphodiesterase	P17405	SMPD1	chr11:6390440	Inverse variance weighted	5	0.004	0.028	8.735E-01	0.998	cis	Hospitalized COVID-19
16853_5	NRK1	Nicotinamide riboside kinase 1	Q9NWW6	NMRK1	chr9:75088217	Inverse variance weighted	2	0.010	0.062	8.743E-01	0.998	cis	Hospitalized COVID-19
3419_49	CAMK2D	Calcium/calmodulin-dependent protein kinase type II subunit delta	Q13557	CAMK2D	chr4:113761927	Wald ratio	1	0.036	0.231	8.747E-01	0.998	cis	Hospitalized COVID-19
10366_11	PDGFRA	Platelet-derived growth factor receptor alpha	P16234	PDGFRA	chr4:54229280	Inverse variance weighted	3	-0.012	0.077	8.751E-01	0.998	cis	Hospitalized COVID-19
9578_263	MANS4	MANSC domain-containing protein 4	A6NHS7	MANSC4	chr12:27780236	Inverse variance weighted	7	-0.004	0.024	8.753E-01	0.998	cis	Hospitalized COVID-19
9385_4	GAA	Lysosomal alpha-glucosidase	P10253	GAA	chr17:80101556	Inverse variance weighted	7	-0.006	0.040	8.754E-01	0.998	cis	Hospitalized COVID-19
3535_84	DKK1	Dickkopf-related protein 1	O94907	DKK1	chr10:52314281	Inverse variance weighted	5	-0.008	0.049	8.765E-01	0.998	cis	Hospitalized COVID-19
8297_8	DJC10	DnaJ homolog subfamily C member 10	Q8IXB1	DNAJC10	chr2:182716255	Inverse variance weighted	2	0.015	0.100	8.773E-01	0.998	cis	Hospitalized COVID-19
18922_27	CD68	Macrosialin	P34810	CD68	chr17:7579491	Inverse variance weighted	2	0.022	0.142	8.774E-01	0.998	cis	Hospitalized COVID-19
16805_5	PDE5A	cGMP-specific 3',5'-cyclic phosphodiesterase	O76074	PDE5A	chr4:119628804	Wald ratio	1	-0.034	0.224	8.801E-01	0.999	cis	Hospitalized COVID-19
13959_7	LAP	Cytosol aminopeptidase	P28838	LAP3	chr4:17577198	Wald ratio	1	-0.037	0.254	8.831E-01	0.999	cis	Hospitalized COVID-19
9380_2	sPLA(2)-XIII	Group XIIB secretory phospholipase A2-like protein	Q9BX93	PLA2G12B	chr10:72954806	Wald ratio	1	-0.018	0.120	8.836E-01	0.999	cis	Hospitalized COVID-19
6425_87	MMP19	Matrix metalloproteinase-19	Q99542	MMP19	chr12:55842966	Inverse variance weighted	3	-0.011	0.076	8.841E-01	0.999	cis	Hospitalized COVID-19
7218_87	AT1B2	Sodium/potassium-transporting ATPase subunit beta-2	P14415	ATP1B2	chr17:7646627	Inverse variance weighted	3	-0.010	0.069	8.874E-01	0.999	cis	Hospitalized COVID-19
7110_2	DJB11	DnaJ homolog subfamily B member 11	Q9UBS4	DNAJB11	chr3:186567403	Inverse variance weighted	2	-0.014	0.101	8.885E-01	0.999	cis	Hospitalized COVID-19
5480_49	RANTES	C-C motif chemokine 5	P13501	CCL5	chr17:35880793	Inverse variance weighted	2	0.016	0.117	8.890E-01	0.999	cis	Hospitalized COVID-19
8885_6	CA2D3	Voltage-dependent calcium channel subunit alpha-2/delta-3	Q8IZS8	CACNA2D3	chr3:54122547	Inverse variance weighted	5	0.007	0.051	8.906E-01	0.999	cis	Hospitalized COVID-19
6485_59	IGLL1	Immunoglobulin lambda-like polypeptide 1	P15814	IGLL1	chr22:23580302	Inverse variance weighted	4	-0.004	0.029	8.921E-01	0.999	cis	Hospitalized COVID-19
8458_111	a-Synuclein	Alpha-synuclein	P37840	SNCA	chr4:89838315	Wald ratio	1	-0.032	0.234	8.925E-01	0.999	cis	Hospitalized COVID-19
9282_12	CRIS2	Cysteine-rich secretory protein 2	P16562	CRISP2	chr6:49713590	Inverse variance weighted	6	-0.003	0.020	8.931E-01	0.999	cis	Hospitalized COVID-19
8244_16	FUT8	Alpha-(1,6)-fucosyltransferase	Q9BYC5	FUT8	chr14:65410592	Inverse variance weighted	10	0.003	0.022	8.947E-01	0.999	cis	Hospitalized COVID-19
3043_49	ON	SPARC	P09486	SPARC	chr5:151686975	Wald ratio	1	0.027	0.203	8.955E-01	0.999	cis	Hospitalized COVID-19
9863_1	Tropomyosin 4	Tropomyosin alpha-4 chain	P67936	TPM4	chr19:16067021	Wald ratio	1	0.018	0.137	8.956E-01	0.999	cis	Hospitalized COVID-19
5621_64	THSD1	Thrombospondin type-1 domain-containing protein 1	Q9NS62	THSD1	chr13:52416373	Inverse variance weighted	4	0.007	0.051	8.962E-01	0.999	cis	Hospitalized COVID-19
4962_52	ARMEL	Cerebral dopamine neurotrophic factor	Q49AH0	CDNF	chr10:14838575	Inverse variance weighted	6	-0.005	0.037	8.978E-01	0.999	cis	Hospitalized COVID-19
7875_86	PLEK	Pleckstrin	P08567	PLEK	chr2:68365282	Inverse variance weighted	2	0.010	0.077	8.984E-01	0.999	cis	Hospitalized COVID-19
16892_23	ENPP2	Ectonucleotide pyrophosphatase/phosphodiesterase family member 2	Q13822	ENPP2	chr8:119673453	Inverse variance weighted	4	0.009	0.072	8.986E-01	0.999	cis	Hospitalized COVID-19
19236_24	TCP4	Activated RNA polymerase II transcriptional coactivator p15	P53999	SUB1	chr5:32531633	Wald ratio	1	-0.028	0.221	8.989E-01	0.999	cis	Hospitalized COVID-19
4496_60	MMP-12	Macrophage metalloelastase	P39900	MMP12	chr11:102874982	Inverse variance weighted	7	0.002	0.019	9.011E-01	0.999	cis	Hospitalized COVID-19
8687_26	T106B	Transmembrane protein 106B	Q9NUM4	TMEM106B	chr7:12211270	Inverse variance weighted	2	-0.009	0.075	9.012E-01	0.999	cis	Hospitalized COVID-19
14101_2	CNTFR alpha	Ciliary neurotrophic factor receptor subunit alpha	P26992	CNTFR	chr9:34590140	Inverse variance weighted	2	0.009	0.075	9.013E-01	0.999	cis	Hospitalized COVID-19
18330_7	PGM2	Phosphoglucomutase-2	Q96G03	PGM2	chr4:37826660	Inverse variance weighted	3	-0.007	0.056	9.016E-01	0.999	cis	Hospitalized COVID-19
7806_33	B4GT7	Beta-1,4-galactosyltransferase 7	Q9UBV7	B4GALT7	chr5:177600132	Inverse variance weighted	7	0.006	0.048	9.019E-01	0.999	cis	Hospitalized COVID-19
4811_33	ITI heavy chain H4	Inter-alpha-trypsin inhibitor heavy chain H4	Q14624	ITIH4	chr3:52830688	Inverse variance weighted	2	-0.034	0.281	9.041E-01	0.999	cis	Hospitalized COVID-19
13381_49	B4GT1	Beta-1,4-galactosyltransferase 1	P15291	B4GALT1	chr9:33167356	Inverse variance weighted	2	0.006	0.053	9.042E-01	0.999	cis	Hospitalized COVID-19
9580_5	Laminin gamma-2	Laminin subunit gamma-2	Q13753	LAMC2	chr1:183186238	Inverse variance weighted	4	-0.004	0.034	9.048E-01	0.999	cis	Hospitalized COVID-19
3727_35	PYY	Peptide YY	P10082	PYY	chr17:44004469	Wald ratio	1	-0.017	0.141	9.049E-01	0.999	cis	Hospitalized COVID-19

10851_77	IL27B	Interleukin-27 subunit beta	Q14213	EBI3	chr19:4229523	Inverse variance weighted	7	0.002	0.018	9.056E-01	0.999	cis	Hospitalized COVID-19
3181_50	Cathepsin S	Cathepsin S	P25774	CTSS	chr1:150765957	Inverse variance weighted	3	-0.003	0.028	9.060E-01	0.999	cis	Hospitalized COVID-19
4469_78	ST4S6	Carbohydrate sulfotransferase 15	Q7LFX5	CHST15	chr10:124093598	Inverse variance weighted	2	0.012	0.105	9.083E-01	0.999	cis	Hospitalized COVID-19
3488_64	Catalase	Catalase	P04040	CAT	chr11:34438934	Inverse variance weighted	3	-0.010	0.089	9.083E-01	0.999	cis	Hospitalized COVID-19
3795_6	ADAM 9	Disintegrin and metalloproteinase domain-containing protein 9	Q13443	ADAM9	chr8:38996754	Wald ratio	1	0.029	0.256	9.093E-01	0.999	cis	Hospitalized COVID-19
9388_18	MCEE	Methylmalonyl-CoA epimerase, mitochondrial	Q96PE7	MCEE	chr2:71130239	Wald ratio	1	-0.020	0.178	9.094E-01	0.999	cis	Hospitalized COVID-19
18220_141	SRA1	Steroid receptor RNA activator 1	Q9HD15	SRA1	chr5:140557677	Inverse variance weighted	2	-0.018	0.159	9.097E-01	0.999	cis	Hospitalized COVID-19
17403_14	acyl-Coenzyme A dehydrogenase	Short/branched chain specific acyl-CoA dehydrogenase, mitochondrial	P45954	ACADSB	chr10:123008979	Inverse variance weighted	2	-0.025	0.226	9.119E-01	0.999	cis	Hospitalized COVID-19
10445_20	ApoM	Apolipoprotein M	O95445	APOM	chr6:31652416	Inverse variance weighted	2	0.006	0.055	9.126E-01	0.999	cis	Hospitalized COVID-19
5656_53	PP11	Poly(U)-specific endoribonuclease	P21128	ENDOU	chr12:47725567	Wald ratio	1	-0.018	0.167	9.128E-01	0.999	cis	Hospitalized COVID-19
18891_98	GBP2	Guanylate-binding protein 2	P32456	GBP2	chr1:89150456	Wald ratio	1	-0.015	0.137	9.134E-01	0.999	cis	Hospitalized COVID-19
8394_56	RNase 2	Non-secretory ribonuclease	P10153	RNASE2	chr14:20955487	Wald ratio	1	-0.015	0.140	9.143E-01	0.999	cis	Hospitalized COVID-19
15343_337	Kininogen, HMW, Two Chain	Kininogen, HMW, Two Chain	P01042	KNG1	chr3:186717348	Inverse variance weighted	4	-0.011	0.101	9.150E-01	0.999	cis	Hospitalized COVID-19
7856_51	F151A	Protein FAM151A	Q8WW52	FAM151A	chr1:54623556	Inverse variance weighted	5	0.004	0.037	9.152E-01	0.999	cis	Hospitalized COVID-19
17751_68	CRBB1	Beta-crystallin B1	P53674	CRYBB1	chr22:26618027	Wald ratio	1	0.006	0.055	9.164E-01	0.999	cis	Hospitalized COVID-19
3403_1	TPSB2	Tryptase beta-2	P20231	TPSB2	chr16:1230184	Inverse variance weighted	8	-0.002	0.022	9.165E-01	0.999	cis	Hospitalized COVID-19
9870_17	SYWC	Tryptophan--tRNA ligase, cytoplasmic	P23381	WARS1	chr14:100376805	Inverse variance weighted	2	-0.006	0.058	9.178E-01	0.999	cis	Hospitalized COVID-19
6904_14	LRRT2	Leucine-rich repeat transmembrane neuronal protein 2	O43300	LRRTM2	chr5:138875368	Wald ratio	1	0.013	0.125	9.193E-01	0.999	cis	Hospitalized COVID-19
4160_49	MMP-2	72 kDa type IV collagenase	P08253	MMP2	chr16:55389700	Inverse variance weighted	2	-0.010	0.095	9.198E-01	0.999	cis	Hospitalized COVID-19
4721_54	TFF3	Trefoil factor 3	Q07654	TFF3	chr21:42315409	Inverse variance weighted	3	0.011	0.110	9.199E-01	0.999	cis	Hospitalized COVID-19
3435_53	FN1.4	Fibronectin Fragment 4	P02751	FN1	chr2:215436073	Wald ratio	1	-0.031	0.313	9.203E-01	0.999	cis	Hospitalized COVID-19
16607_78	Gelsolin	Gelsolin	P06396	GSN	chr9:121207794	Inverse variance weighted	2	-0.015	0.156	9.215E-01	0.999	cis	Hospitalized COVID-19
5363_51	Semaphorin 3E	Semaphorin-3E	O15041	SEMA3E	chr7:83649139	Inverse variance weighted	10	-0.002	0.018	9.217E-01	0.999	cis	Hospitalized COVID-19
5223_59	GCKR	Glucokinase regulatory protein	Q14397	GCKR	chr2:27496839	Wald ratio	1	0.011	0.110	9.224E-01	0.999	cis	Hospitalized COVID-19
7198_197	FA20B	Glycosaminoglycan xylosylkinase	O75063	FAM20B	chr1:179025804	Inverse variance weighted	2	0.010	0.106	9.227E-01	0.999	cis	Hospitalized COVID-19
6920_1	GFRAL	GDNF family receptor alpha-like	Q6UXV0	GFRAL	chr6:55327469	Inverse variance weighted	3	0.005	0.051	9.239E-01	0.999	cis	Hospitalized COVID-19
17151_84	IRF-3	Interferon regulatory factor 3	Q14653	IRF3	chr19:49665875	Wald ratio	1	0.013	0.137	9.250E-01	0.999	cis	Hospitalized COVID-19
6909_40	MGAT2	Alpha-1,6-mannosyl-glycoprotein 2-beta-N-acetylglucosaminyltransferase	Q10469	MGAT2	chr14:49620799	Wald ratio	1	-0.007	0.072	9.254E-01	0.999	cis	Hospitalized COVID-19
5740_17	ROBO1	Roundabout homolog 1	Q9Y6N7	ROBO1	chr3:79767998	Inverse variance weighted	3	-0.007	0.078	9.255E-01	0.999	cis	Hospitalized COVID-19
12859_33	PECI	Enoyl-CoA delta isomerase 2, mitochondrial	O75521	ECI2	chr6:4135597	Inverse variance weighted	3	-0.006	0.060	9.261E-01	0.999	cis	Hospitalized COVID-19
10666_7	GNPTG	N-acetylglucosamine-1-phosphotransferase subunit gamma	Q9UJJ9	GNPTG	chr16:1351931	Inverse variance weighted	4	-0.005	0.052	9.264E-01	0.999	cis	Hospitalized COVID-19
15444_45	SCCA2	Serpin B4	P48594	SERPINB4	chr18:63644256	Inverse variance weighted	3	-0.009	0.098	9.266E-01	0.999	cis	Hospitalized COVID-19
8973_23	FCRL4:ECD	Fc receptor-like protein 4:Extracellular domain	Q96PJ5	FCRL4	chr1:157598085	Inverse variance weighted	13	-0.003	0.030	9.273E-01	0.999	cis	Hospitalized COVID-19
8925_25	RIR2B	Ribonucleoside-diphosphate reductase subunit M2 B	Q7LG56	RRM2B	chr8:102238961	Inverse variance weighted	2	0.006	0.069	9.275E-01	0.999	cis	Hospitalized COVID-19
3331_8	RGMB	RGM domain family member B	Q6NW40	RGMB	chr5:98768650	Inverse variance weighted	3	-0.005	0.058	9.286E-01	0.999	cis	Hospitalized COVID-19
17698_15	WBP2	WW domain-binding protein 2	Q969T9	WBP2	chr17:75856507	Wald ratio	1	-0.020	0.222	9.289E-01	0.999	cis	Hospitalized COVID-19
15522_2	GAPR1	Golgi-associated plant pathogenesis-related protein 1	Q9H4G4	GLIPR2	chr9:36136536	Inverse variance weighted	2	-0.003	0.039	9.291E-01	0.999	cis	Hospitalized COVID-19
19124_9	UBCP1	Ubiquitin-like domain-containing CTD phosphatase 1	Q8WVY7	UBLCP1	chr5:159263290	Inverse variance weighted	2	0.008	0.089	9.296E-01	0.999	cis	Hospitalized COVID-19
3438_10	FSTL3	Follistatin-related protein 3	O95633	FSTL3	chr19:676392	Inverse variance weighted	2	0.022	0.253	9.297E-01	0.999	cis	Hospitalized COVID-19
16882_27	PHP14	14 kDa phosphohistidine phosphatase	Q9NRX4	PHPT1	chr9:136848724	Inverse variance weighted	2	0.014	0.161	9.308E-01	0.999	cis	Hospitalized COVID-19
5018_68	Peroxiredoxin-6	Peroxiredoxin-6	P30041	PRDX6	chr1:173477330	Wald ratio	1	-0.014	0.170	9.322E-01	0.999	cis	Hospitalized COVID-19
8099_42	SPON2	Spondin-2	Q9BUD6	SPON2	chr4:1208962	Inverse variance weighted	6	-0.005	0.054	9.324E-01	0.999	cis	Hospitalized COVID-19
6923_1	PLOD2	Procollagen-lysine,2-oxoglutarate 5-dioxygenase 2	O00469	PLOD2	chr3:146163725	Inverse variance weighted	2	-0.011	0.133	9.330E-01	0.999	cis	Hospitalized COVID-19
3852_19	HSP 40	DnaJ homolog subfamily B member 1	P25685	DNAJB1	chr19:14560391	Wald ratio	1	0.018	0.214	9.336E-01	0.999	cis	Hospitalized COVID-19
5703_26	NOE1	Noelin	Q99784	OLFM1	chr9:135075422	Inverse variance weighted	2	0.009	0.108	9.339E-01	0.999	cis	Hospitalized COVID-19
17391_10	SPF45	Splicing factor 45	Q96I25	RBM17	chr10:6089034	Inverse variance weighted	2	-0.004	0.048	9.341E-01	0.999	cis	Hospitalized COVID-19
6375_75	XXLT1	Xyloside xylosyltransferase 1	Q8NBI6	XXYLT1	chr3:195271159	Inverse variance weighted	2	-0.004	0.046	9.346E-01	0.999	cis	Hospitalized COVID-19
9565_6	PAWR	PRKC apoptosis WT1 regulator protein	Q96IZ0	PAWR	chr12:79690964	Wald ratio	1	0.019	0.229	9.352E-01	0.999	cis	Hospitalized COVID-19
4232_19	IGF-I sR	Insulin-like growth factor 1 receptor	P08069	IGF1R	chr15:98648539	Inverse variance weighted	2	0.008	0.097	9.354E-01	0.999	cis	Hospitalized COVID-19
8039_41	F177A	Protein FAM177A1	Q8N128	FAM177A1	chr14:35045117	Inverse variance weighted	6	0.002	0.026	9.357E-01	0.999	cis	Hospitalized COVID-19
15304_1	PAP1	Regenerating islet-derived protein 3-alpha	Q06141	REG3A	chr2:79159753	Inverse variance weighted	3	0.011	0.141	9.358E-01	0.999	cis	Hospitalized COVID-19
12563_2	TFIP8	Tumor necrosis factor alpha-induced protein 8	O95379	TNFAIP8	chr5:119268692	Inverse variance weighted	2	-0.005	0.066	9.374E-01	0.999	cis	Hospitalized COVID-19
10565_19	SLIK3	SLIT and NTRK-like protein 3	O94933	SLITRK3	chr3:165197109	Inverse variance weighted	3	-0.003	0.033	9.375E-01	0.999	cis	Hospitalized COVID-19
5680_54	OBP2B	Odorant-binding protein 2b	Q9NPH6	OBP2B	chr9:133209250	Inverse variance weighted	4	-0.004	0.047	9.379E-01	0.999	cis	Hospitalized COVID-19
17456_53	GOLM1	Golgi membrane protein 1	Q8NBJ4	GOLM1	chr9:86100173	Inverse variance weighted	6	-0.005	0.065	9.381E-01	0.999	cis	Hospitalized COVID-19
3194_36	GPVI	Platelet glycoprotein VI	Q9HCN6	GP6	chr19:55038264	Inverse variance weighted	2	-0.003	0.038	9.384E-01	0.999	cis	Hospitalized COVID-19
2436_49	CXCL16, soluble	C-X-C motif chemokine 16	Q9H2A7	CXCL16	chr17:4739928	Inverse variance weighted	3	-0.004	0.053	9.388E-01	0.999	cis	Hospitalized COVID-19
8351_17	PRS57	Serine protease 57	Q6UWY2	PRSS57	chr19:695498	Inverse variance weighted	6	-0.003	0.040	9.388E-01	0.999	cis	Hospitalized COVID-19
9829_91	SULT 2A1	Bile salt sulfotransferase	Q06520	SULT2A1	chr19:47886315	Wald ratio	1	-0.005	0.065	9.389E-01	0.999	cis	Hospitalized COVID-19
4996_66	HRG	Histidine-rich glycoprotein	P04196	HRG	chr3:186660216	Inverse variance weighted	4	0.003	0.040	9.397E-01	0.999	cis	Hospitalized COVID-19
14273_19	PPCE	Prolyl endopeptidase	P48147	PREP	chr6:105454062	Inverse variance weighted	2	0.004	0.060	9.411E-01	0.999	cis	Hospitalized COVID-19
19448_104	UPP1	Uridine phosphorylase 1	Q16831	UPP1	chr7:48088628	Wald ratio	1	-0.009	0.119	9.414E-01	0.999	cis	Hospitalized COVID-19
18213_30	UBE2F	NEDD8-conjugating enzyme UBE2F	Q969M7	UBE2F	chr2:237966827	Wald ratio	1	-0.012	0.170	9.417E-01	0.999	cis	Hospitalized COVID-19
18180_58	RT4I1	Reticulon-4-interacting protein 1, mitochondrial	Q8WWV3	RTN4IP1	chr6:106629498	Wald ratio	1	0.012	0.168	9.419E-01	0.999	cis	Hospitalized COVID-19
19637_9	CRH	Corticoliberin	P06850	CRH	chr8:66178464	Wald ratio	1	-0.012	0.168	9.421E-01	0.999	cis	Hospitalized COVID-19
7757_5	DQA2	HLA class II histocompatibility antigen, DQ alpha 2 chain	P01906	HLA-DQA2	chr6:32741391	Inverse variance weighted	3	0.005	0.071	9.428E-01	0.999	cis	Hospitalized COVID-19
15363_32	Apo A-V	Apolipoprotein A-V	Q6Q788	APOA5	chr11:116792420	Inverse variance weighted	8	-0.002	0.027	9.434E-01	0.999	cis	Hospitalized COVID-19
7871_16	T132A	Transmembrane protein 132A	Q24JP5	TMEM132A	chr11:60924460	Inverse variance weighted	6	0.002	0.025	9.436E-01	0.999	cis	Hospitalized COVID-19
5256_86	PDE5A	cGMP-specific 3',5'-cyclic phosphodiesterase	O76074	PDE5A	chr4:119628804	Inverse variance weighted	3	0.009	0.125	9.437E-01	0.999	cis	Hospitalized COVID-19
8465_52	Cathepsin H	Cathepsin H	P09668	CTSH	chr15:78949574	Inverse variance weighted	10	-0.002	0.022	9.438E-01	0.999	cis	Hospitalized COVID-19
8428_102	NTRI	Neurotrimin	Q9P121	NTM	chr11:131370478	Inverse variance weighted	4	0.006	0.090	9.438E-01	0.999	cis	Hospitalized COVID-19
3024_18	a2-Antiplasmin	Alpha-2-antiplasmin	P08697	SERPINF2	chr17:1742836	Inverse variance weighted	3	0.004	0.064	9.443E-01	0.999	cis	Hospitalized COVID-19
17802_4	SIAS	Sialic acid synthase	Q9NR45	NANS	chr9:98056732	Wald ratio	1	0.008	0.119	9.445E-01	0.999	cis	Hospitalized COVID-19
11288_26	5NTC	Cytosolic purine 5'-nucleotidase	P49902	NT5C2	chr10:103277605	Inverse variance weighted	3	0.005	0.071	9.447E-01	0.999	cis	Hospitalized COVID-19
11390_24	Carbonic Anhydrase VIII	Carbonic anhydrase-related protein	P35219	CA8	chr8:60281400	Inverse variance weighted	6	-0.002	0.031	9.450E-01	0.999	cis	Hospitalized COVID-19
5116_62	ROBO2	Roundabout homolog 2	Q9HCK4	ROBO2	chr3:75906695	Wald ratio	1	0.016	0.232	9.455E-01	0.999	cis	Hospitalized COVID-19
17161_1	OST48	Dolichyl-diphosphooligosaccharide--protein glycosyltransferase 48 kDa subunit	P39656	DDOST	chr1:20661544	Wald ratio	1	0.014	0.207	9.462E-01	0.999	cis	Hospitalized COVID-19
9191_8	Trefoil factor 2	Trefoil factor 2	Q03403	TFF2	chr21:42350997	Inverse variance weighted	2	0.007	0.107	9.471E-01	0.999	cis	Hospitalized COVID-19

16872_248	MAAI	Maleylacetoacetate isomerase	O43708	GSTZ1	chr14:77320996	Inverse variance weighted	8	0.001	0.015	9.483E-01	0.999	cis	Hospitalized COVID-19
15495_9	FOLR3	Folate receptor gamma	P41439	FOLR3	chr11:72114869	Inverse variance weighted	9	0.002	0.027	9.486E-01	0.999	cis	Hospitalized COVID-19
5609_92	F19A5	Protein FAM19A5	Q7Z5A7	TAFA5	chr22:48489553	Wald ratio	1	-0.014	0.222	9.506E-01	0.999	cis	Hospitalized COVID-19
8274_64	Syntaxin-7	Syntaxin-7	O15400	STX7	chr6:132513198	Wald ratio	1	0.005	0.087	9.510E-01	0.999	cis	Hospitalized COVID-19
3041_55	MRC2	C-type mannose receptor 2	Q9UBG0	MRC2	chr17:62627670	Inverse variance weighted	6	-0.003	0.051	9.515E-01	0.999	cis	Hospitalized COVID-19
15386_7	FABPA	Fatty acid-binding protein, adipocyte	P15090	FABP4	chr8:81483236	Wald ratio	1	0.008	0.128	9.533E-01	0.999	cis	Hospitalized COVID-19
4297_62	Spondin-1	Spondin-1	Q9HCB6	SPON1	chr11:13962723	Inverse variance weighted	4	0.002	0.035	9.534E-01	0.999	cis	Hospitalized COVID-19
8024_64	TPST2	Protein-tyrosine sulfotransferase 2	O60704	TPST2	chr22:26596717	Inverse variance weighted	2	0.007	0.117	9.537E-01	0.999	cis	Hospitalized COVID-19
10561_5	PGRP-I-alpha	Peptidoglycan recognition protein 3	Q96LB9	PGLYRP3	chr1:153312952	Wald ratio	1	0.012	0.214	9.544E-01	0.999	cis	Hospitalized COVID-19
3728_52	Secretin	Secretin	P09683	SCT	chr11:627181	Wald ratio	1	0.008	0.138	9.551E-01	0.999	cis	Hospitalized COVID-19
3806_55	EphA5	Ephrin type-A receptor 5	P54756	EPHA5	chr4:65670495	Inverse variance weighted	3	-0.005	0.083	9.553E-01	0.999	cis	Hospitalized COVID-19
19143_38	NB5R2	NADH-cytochrome b5 reductase 2	Q6BCY4	CYB5R2	chr11:7677222	Inverse variance weighted	6	0.004	0.080	9.554E-01	0.999	cis	Hospitalized COVID-19
12457_10	MTND	1,2-dihydroxy-3-keto-5-methylthiopentene dioxygenase	Q9BV57	ADI1	chr2:3519531	Wald ratio	1	-0.008	0.152	9.570E-01	0.999	cis	Hospitalized COVID-19
18172_71	ASF1A	Histone chaperone ASF1A	Q9Y294	ASF1A	chr6:118894152	Wald ratio	1	0.005	0.096	9.570E-01	0.999	cis	Hospitalized COVID-19
12008_3	CD7	T-cell antigen CD7	P09564	CD7	chr17:82317608	Inverse variance weighted	4	-0.006	0.113	9.574E-01	0.999	cis	Hospitalized COVID-19
5029_3	SEPR	Prolyl endopeptidase FAP	Q12884	FAP	chr2:162245151	Inverse variance weighted	5	0.004	0.074	9.575E-01	0.999	cis	Hospitalized COVID-19
3323_37	LRP8	Low-density lipoprotein receptor-related protein 8	Q14114	LRP8	chr1:53328469	Inverse variance weighted	2	0.003	0.051	9.579E-01	0.999	cis	Hospitalized COVID-19
2831_29	Kallikrein 11	Kallikrein-11	Q9UBX7	KLK11	chr19:51028039	Inverse variance weighted	7	-0.003	0.056	9.594E-01	0.999	cis	Hospitalized COVID-19
4548_4	Fucosyltransferase 3	Galactoside 3(4)-L-fucosyltransferase	P21217	FUT3	chr19:5851471	Inverse variance weighted	9	0.002	0.043	9.623E-01	0.999	cis	Hospitalized COVID-19
15475_4	PLTP	Phospholipid transfer protein	P55058	PLTP	chr20:45912155	Inverse variance weighted	9	0.002	0.039	9.630E-01	0.999	cis	Hospitalized COVID-19
12812_25	ACYP2	Acylphosphatase-2	P14621	ACYP2	chr2:53970838	Inverse variance weighted	3	-0.002	0.047	9.634E-01	0.999	cis	Hospitalized COVID-19
4240_31	M2-PK	Pyruvate kinase PKM	P14618	PKM	chr15:72231819	Wald ratio	1	0.007	0.154	9.639E-01	0.999	cis	Hospitalized COVID-19
18876_77	CHST4	Carbohydrate sulfotransferase 4	Q8NCG5	CHST4	chr16:71525233	Wald ratio	1	-0.010	0.235	9.651E-01	0.999	cis	Hospitalized COVID-19
18307_71	PPase 2	Inorganic pyrophosphatase 2, mitochondrial	Q9H2U2	PPA2	chr4:105474067	Wald ratio	1	0.006	0.129	9.652E-01	0.999	cis	Hospitalized COVID-19
2851_63	C5a	C5a anaphylatoxin	P01031	C5	chr9:121075195	Inverse variance weighted	2	0.008	0.194	9.659E-01	0.999	cis	Hospitalized COVID-19
6382_17	MANBA	Beta-mannosidase	O00462	MANBA	chr4:102760994	Inverse variance weighted	5	0.002	0.055	9.659E-01	0.999	cis	Hospitalized COVID-19
19135_5	Cytosolic 5'-nucleotidase III-like protein	7-methylguanosine phosphate-specific 5'-nucleotidase	Q969T7	NT5C3B	chr17:41836260	Inverse variance weighted	9	-0.001	0.027	9.663E-01	0.999	cis	Hospitalized COVID-19
7994_41	ERO1B	ERO1-like protein beta	Q86YB8	ERO1B	chr1:236282019	Inverse variance weighted	2	0.002	0.056	9.666E-01	0.999	cis	Hospitalized COVID-19
2811_27	Angiopoietin-1	Angiopoietin-1	Q15389	ANGPT1	chr8:107498055	Wald ratio	1	0.006	0.153	9.670E-01	0.999	cis	Hospitalized COVID-19
8974_172	COFA1	Collagen alpha-1(XV) chain	P39059	COL15A1	chr9:98943179	Inverse variance weighted	4	-0.002	0.045	9.672E-01	0.999	cis	Hospitalized COVID-19
15300_66	CC134	Coiled-coil domain-containing protein 134	Q9H6E4	CCDC134	chr22:41800679	Inverse variance weighted	2	-0.004	0.089	9.672E-01	0.999	cis	Hospitalized COVID-19
5139_32	UNC5H3	Netrin receptor UNC5C	O95185	UNC5C	chr4:95549206	Inverse variance weighted	3	-0.002	0.055	9.690E-01	0.999	cis	Hospitalized COVID-19
15626_223	Perlecan	Basement membrane-specific heparan sulfate proteoglycan core protein	P98160	HSPG2	chr1:21937310	Inverse variance weighted	4	-0.001	0.038	9.696E-01	0.999	cis	Hospitalized COVID-19
15326_64	GBP1	Guanylate-binding protein 1	P32455	GBP1	chr1:89065360	Inverse variance weighted	2	-0.002	0.054	9.696E-01	0.999	cis	Hospitalized COVID-19
4413_3	SLPI	Antileukoproteinase	P03973	SLPI	chr20:45254564	Wald ratio	1	-0.008	0.221	9.699E-01	0.999	cis	Hospitalized COVID-19
7945_10	Semaphorin-6A	Semaphorin-6A	Q9H2E6	SEMA6A	chr5:116574823	Inverse variance weighted	4	0.002	0.065	9.700E-01	0.999	cis	Hospitalized COVID-19
18873_8	CEAM8	Carcinoembryonic antigen-related cell adhesion molecule 8	P31997	CEACAM8	chr19:42595055	Wald ratio	1	-0.008	0.230	9.712E-01	0.999	cis	Hospitalized COVID-19
11360_39	RRM1	Ribonucleoside-diphosphate reductase large subunit	P23921	RRM1	chr11:4094707	Wald ratio	1	0.007	0.197	9.722E-01	0.999	cis	Hospitalized COVID-19
16561_9	Alpha-1B-glycoprotein	Alpha-1B-glycoprotein	P04217	A1BG	chr19:58353492	Inverse variance weighted	4	0.001	0.027	9.725E-01	0.999	cis	Hospitalized COVID-19
15613_16	LIPP	Pancreatic triacylglycerol lipase	P16233	PNLIP	chr10:116545931	Wald ratio	1	-0.006	0.183	9.729E-01	0.999	cis	Hospitalized COVID-19
4324_33	CYTT	Cystatin-SA	P09228	CST2	chr20:23826729	Inverse variance weighted	5	-0.003	0.079	9.732E-01	0.999	cis	Hospitalized COVID-19
12549_33	PTGD2	Hematopoietic prostaglandin D synthase	O60760	HPGDS	chr4:94342876	Inverse variance weighted	9	-0.001	0.021	9.739E-01	0.999	cis	Hospitalized COVID-19
8958_51	CHL1	Neural cell adhesion molecule L1-like protein	O00533	CHL1	chr3:196763	Inverse variance weighted	6	-0.002	0.048	9.740E-01	0.999	cis	Hospitalized COVID-19
2705_5	TECK	C-C motif chemokine 25	O15444	CCL25	chr19:8052318	Inverse variance weighted	11	-0.001	0.025	9.744E-01	0.999	cis	Hospitalized COVID-19
7947_19	AP4AT	AP-4 complex accessory subunit tepsin	Q96N21	TEPSIN	chr17:81239091	Wald ratio	1	0.006	0.190	9.744E-01	0.999	cis	Hospitalized COVID-19
14123_34	GI24:ECD	V-type immunoglobulin domain-containing suppressor of T-cell activation:Extracellular domain	Q9H7M9	VSIR	chr10:71773520	Inverse variance weighted	5	0.003	0.087	9.748E-01	0.999	cis	Hospitalized COVID-19
5275_28	VAV	Proto-oncogene vav	P15498	VAV1	chr19:6772708	Wald ratio	1	-0.006	0.185	9.748E-01	0.999	cis	Hospitalized COVID-19
9313_27	CBLN1	Cerebellin-1	P23435	CBLN1	chr16:49281838	Inverse variance weighted	9	0.001	0.037	9.773E-01	0.999	cis	Hospitalized COVID-19
3302_58	CYTF	Cystatin-F	O76096	CST7	chr20:24949269	Inverse variance weighted	7	0.001	0.021	9.775E-01	0.999	cis	Hospitalized COVID-19
9940_35	DUS28	Dual specificity phosphatase 28	Q4G0W2	DUSP28	chr2:240560054	Inverse variance weighted	2	-0.002	0.065	9.785E-01	0.999	cis	Hospitalized COVID-19
16292_288	GIP	Gastric inhibitory polypeptide	P09681	GIP	chr17:48968596	Wald ratio	1	-0.006	0.237	9.789E-01	0.999	cis	Hospitalized COVID-19
12401_3	STALP	AMSH-like protease	Q96FJ0	STAMBPL1	chr10:88879734	Wald ratio	1	-0.003	0.110	9.790E-01	0.999	cis	Hospitalized COVID-19
17164_15	annexin IV	Annexin A4	P09525	ANXA4	chr2:69644425	Inverse variance weighted	2	0.002	0.073	9.796E-01	0.999	cis	Hospitalized COVID-19
17343_6	SNPH	Syntaphilin	O15079	SNPH	chr20:1266280	Inverse variance weighted	2	-0.003	0.118	9.797E-01	0.999	cis	Hospitalized COVID-19
5852_6	S100A12	Protein S100-A12	P80511	S100A12	chr1:153375621	Inverse variance weighted	2	-0.002	0.093	9.801E-01	0.999	cis	Hospitalized COVID-19
5430_66	SHPS1	Tyrosine-protein phosphatase non-receptor type substrate 1	P78324	SIRPA	chr20:1894167	Inverse variance weighted	9	-0.001	0.026	9.805E-01	0.999	cis	Hospitalized COVID-19
2860_19	Karyopherin-a2	Importin subunit alpha-1	P52292	KPNA2	chr17:68035636	Wald ratio	1	0.005	0.205	9.805E-01	0.999	cis	Hospitalized COVID-19
3280_49	Aggrecan	Aggrecan core protein	P16112	ACAN	chr15:88803436	Inverse variance weighted	2	-0.002	0.076	9.806E-01	0.999	cis	Hospitalized COVID-19
6252_62	Secretoglobin family 3A member 1	Secretoglobin family 3A member 1	Q96QR1	SCGB3A1	chr5:180591499	Inverse variance weighted	4	0.001	0.049	9.813E-01	0.999	cis	Hospitalized COVID-19
9005_16	PLXA1	Plexin-A1	Q9UIW2	PLXNA1	chr3:126982693	Inverse variance weighted	3	-0.001	0.047	9.817E-01	0.999	cis	Hospitalized COVID-19
2730_58	MICA	MHC class I polypeptide-related sequence A	Q29983	MICA	chr6:31399784	Inverse variance weighted	12	0.001	0.029	9.823E-01	0.999	cis	Hospitalized COVID-19
5456_59	CNDP1	Beta-Ala-His dipeptidase	Q96KN2	CNDP1	chr18:74534500	Inverse variance weighted	6	-0.001	0.042	9.823E-01	0.999	cis	Hospitalized COVID-19
14047_78	BDNF	Brain-derived neurotrophic factor	P23560	BDNF	chr11:27722058	Inverse variance weighted	2	-0.004	0.185	9.830E-01	0.999	cis	Hospitalized COVID-19
18381_16	ALDH-E2	Aldehyde dehydrogenase, mitochondrial	P05091	ALDH2	chr12:111766887	Wald ratio	1	0.003	0.133	9.835E-01	0.999	cis	Hospitalized COVID-19
9314_9	PSG5	Pregnancy-specific beta-1-glycoprotein 5	Q15238	PSG5	chr19:43186536	Inverse variance weighted	3	-0.002	0.098	9.840E-01	0.999	cis	Hospitalized COVID-19
15587_20	FOLR2	Folate receptor beta	P14207	FOLR2	chr11:72216601	Wald ratio	1	0.002	0.087	9.843E-01	0.999	cis	Hospitalized COVID-19
5105_2	Nogo Receptor	Reticulon-4 receptor	Q9BZR6	RTN4R	chr22:20283246	Inverse variance weighted	5	-0.001	0.031	9.847E-01	0.999	cis	Hospitalized COVID-19
6470_19	fibulin 1	Fibulin-1	P23142	FBLN1	chr22:45502238	Inverse variance weighted	3	-0.001	0.068	9.854E-01	0.999	cis	Hospitalized COVID-19
2966_65	SCGF-beta	Stem cell growth factor-beta	Q9Y240	CLEC11A	chr19:50723364	Wald ratio	1	0.005	0.281	9.854E-01	0.999	cis	Hospitalized COVID-19
5867_60	ARGI1	Arginase-1	P05089	ARG1	chr6:131470832	Inverse variance weighted	2	0.002	0.097	9.854E-01	0.999	cis	Hospitalized COVID-19
5124_69	sICAM-5	Intercellular adhesion molecule 5	Q9UMF0	ICAM5	chr19:10289952	Inverse variance weighted	4	0.003	0.188	9.856E-01	0.999	cis	Hospitalized COVID-19
12731_12	PKHA7	Pleckstrin homology domain-containing family A member 7	Q6IQ23	PLEKHA7	chr11:17014415	Inverse variance weighted	4	0.000	0.027	9.873E-01	0.999	cis	Hospitalized COVID-19
5701_81	Tetranectin	Tetranectin	P05452	CLEC3B	chr3:45001548	Inverse variance weighted	2	-0.002	0.111	9.874E-01	0.999	cis	Hospitalized COVID-19
2844_53	sTie-1	Tyrosine-protein kinase receptor Tie-1, soluble	P35590	TIE1	chr1:43300982	Inverse variance weighted	4	0.001	0.045	9.880E-01	0.999	cis	Hospitalized COVID-19
10605_22	APMAP	Adipocyte plasma membrane-associated protein	Q9HDC9	APMAP	chr20:24992751	Wald ratio	1	0.003	0.179	9.885E-01	0.999	cis	Hospitalized COVID-19
8225_86	EPHB2	Ephrin type-B receptor 2	P29323	EPHB2	chr1:22710839	Inverse variance weighted	3	-0.001	0.057	9.888E-01	0.999	cis	Hospitalized COVID-19
14066_49	MAGI2	Membrane-associated guanylate kinase, WW and PDZ domain-containing protein 2	Q86UL8	MAGI2	chr7:79453667	Inverse variance weighted	6	0.000	0.023	9.894E-01	0.999	cis	Hospitalized COVID-19
11266_8	SELPL:ECD	P-selectin glycoprotein ligand 1:Extracellular domain	Q14242	SELPLG	chr12:108633894	Inverse variance weighted	2	0.001	0.041	9.895E-01	0.999	cis	Hospitalized COVID-19

6451_64	ASPN	Asporin	Q9BXN1	ASPN	chr9:92482506	Inverse variance weighted	8	0.001	0.040	9.895E-01	0.999	cis	Hospitalized COVID-19
5803_24	C3d	Complement C3d fragment	P01024	C3	chr19:6730562	Wald ratio	1	-0.002	0.188	9.896E-01	0.999	cis	Hospitalized COVID-19
15542_19	KCRU	Creatine kinase U-type, mitochondrial	P12532	CKMT1A	chr15:43692886	Wald ratio	1	-0.001	0.087	9.899E-01	0.999	cis	Hospitalized COVID-19
7810_20	C1QTNF5	Complement C1q tumor necrosis factor-related protein 5	Q9BXJ0	C1QTNF5	chr11:119340940	Inverse variance weighted	2	0.001	0.077	9.916E-01	0.999	cis	Hospitalized COVID-19
4499_21	PDGF-AA	Platelet-derived growth factor subunit A	P04085	PDGFA	chr7:520296	Inverse variance weighted	3	-0.001	0.126	9.919E-01	0.999	cis	Hospitalized COVID-19
13125_45	Vitronectin	Vitronectin	P04004	VTN	chr17:28373091	Inverse variance weighted	9	0.000	0.020	9.923E-01	0.999	cis	Hospitalized COVID-19
16913_8	RNT2	Ribonuclease T2	O00584	RNASET2	chr6:166957191	Inverse variance weighted	6	0.000	0.024	9.924E-01	0.999	cis	Hospitalized COVID-19
13488_3	ARFP1	Arfaptin-1	P53367	ARFIP1	chr4:152779937	Inverse variance weighted	2	-0.001	0.064	9.925E-01	0.999	cis	Hospitalized COVID-19
15576_158	ECP	Eosinophil cationic protein	P12724	RNASE3	chr14:20891385	Inverse variance weighted	4	0.000	0.031	9.941E-01	0.999	cis	Hospitalized COVID-19
8480_29	FBLN3	EGF-containing fibulin-like extracellular matrix protein 1	Q12805	EFEMP1	chr2:55924139	Inverse variance weighted	2	0.000	0.072	9.952E-01	0.999	cis	Hospitalized COVID-19
16594_44	FAIM1	Fas apoptotic inhibitory molecule 1	Q9NVQ4	FAIM	chr3:138608606	Inverse variance weighted	7	0.000	0.050	9.952E-01	0.999	cis	Hospitalized COVID-19
3309_2	FCG2A	Low affinity immunoglobulin gamma Fc region receptor II-a	P12318	FCGR2A	chr1:161505430	Inverse variance weighted	4	0.000	0.051	9.955E-01	0.999	cis	Hospitalized COVID-19
3820_68	MAPK2	MAP kinase-activated protein kinase 2	P49137	MAPKAPK2	chr1:206684905	Wald ratio	1	0.000	0.058	9.958E-01	0.999	cis	Hospitalized COVID-19
4831_4	sL-Selectin	L-Selectin	P14151	SELL	chr1:169711702	Inverse variance weighted	7	0.000	0.028	9.965E-01	0.999	cis	Hospitalized COVID-19
3651_50	VEGF sR2	Vascular endothelial growth factor receptor 2	P35968	KDR	chr4:55125595	Inverse variance weighted	3	0.000	0.026	9.975E-01	0.999	cis	Hospitalized COVID-19
15511_37	NPTXR	Neuronal pentraxin receptor	O95502	NPTXR	chr22:38844028	Inverse variance weighted	7	0.000	0.030	9.979E-01	0.999	cis	Hospitalized COVID-19
15644_1	Biotinidase	Biotinidase	P43251	BTD	chr3:15601341	Inverse variance weighted	9	0.000	0.024	9.979E-01	0.999	cis	Hospitalized COVID-19
2888_49	C7	Complement component C7	P10643	C7	chr5:40909492	Inverse variance weighted	5	0.000	0.020	9.991E-01	0.999	cis	Hospitalized COVID-19
15376_134	CATE	Cathepsin E	P14091	CTSE	chr1:206023909	Inverse variance weighted	4	0.000	0.037	9.991E-01	0.999	cis	Hospitalized COVID-19
10419_1	SCAR5	Scavenger receptor class A member 5	Q6ZMJ2	SCARA5	chr8:27992673	Inverse variance weighted	5	0.000	0.039	9.993E-01	0.999	cis	Hospitalized COVID-19
9253_52	BGAT	Histo-blood group ABO system transferase	P16442	ABO	chr9:133276024	Inverse variance weighted	12	0.075	0.010	5.433E-13	0.000	cis	SARS-CoV-2 infection
17769_28	PCNP	PEST proteolytic signal-containing nuclear protein	Q8WW12	PCNP	chr3:101574180	Wald ratio	1	-0.381	0.056	1.221E-11	0.000	cis	SARS-CoV-2 infection
19365_11	BCAT2	Branched-chain-amino-acid aminotransferase, mitochondrial	O15382	BCAT2	chr19:48811029	Wald ratio	1	0.141	0.035	7.358E-05	0.041	cis	SARS-CoV-2 infection
13719_19	PAK4	Serine/threonine-protein kinase PAK 4	O96013	PAK4	chr19:39125770	Wald ratio	1	0.365	0.103	3.656E-04	0.153	cis	SARS-CoV-2 infection
17777_31	SDSL	Serine dehydratase-like	Q96GA7	SDSL	chr12:113422380	Inverse variance weighted	6	0.057	0.018	1.080E-03	0.244	cis	SARS-CoV-2 infection
15526_33	GSHB	Glutathione synthetase	P48637	GSS	chr20:34956027	Inverse variance weighted	2	0.112	0.034	1.116E-03	0.244	cis	SARS-CoV-2 infection
8480_29	FBLN3	EGF-containing fibulin-like extracellular matrix protein 1	Q12805	EFEMP1	chr2:55924139	Inverse variance weighted	2	-0.114	0.035	1.144E-03	0.244	cis	SARS-CoV-2 infection
9384_17	Cathelicidin peptide	Cathelicidin antimicrobial peptide	P49913	CAMP	chr3:48223347	Wald ratio	1	0.207	0.064	1.273E-03	0.244	cis	SARS-CoV-2 infection
17697_2	OVCA2	Esterase OVCA2	Q8WZ82	OVCA2	chr17:2042022	Wald ratio	1	0.148	0.046	1.344E-03	0.244	cis	SARS-CoV-2 infection
19129_15	MTHFSD	Methenyltetrahydrofolate synthase domain-containing protein	Q2M296	MTHFSD	chr16:86555235	Inverse variance weighted	5	-0.046	0.014	1.459E-03	0.244	cis	SARS-CoV-2 infection
17761_2	NUDT5	ADP-sugar pyrophosphatase	Q9UKK9	NUDT5	chr10:12196144	Wald ratio	1	-0.192	0.064	2.762E-03	0.411	cis	SARS-CoV-2 infection
12656_1	KLC1	Kinesin light chain 1	Q07866	KLC1	chr14:103561896	Wald ratio	1	0.161	0.054	2.998E-03	0.411	cis	SARS-CoV-2 infection
12437_18	ULK3	Serine/threonine-protein kinase ULK3	Q6PHR2	ULK3	chr15:74843346	Wald ratio	1	0.257	0.087	3.311E-03	0.411	cis	SARS-CoV-2 infection
4992_49	GRN	Granulins	P28799	GRN	chr17:44345246	Inverse variance weighted	3	-0.065	0.022	3.437E-03	0.411	cis	SARS-CoV-2 infection
9916_146	LRC4B:ECD	Leucine-rich repeat-containing protein 4B:Extracellular domain	Q9NT99	LRRC4B	chr19:50568435	Inverse variance weighted	2	-0.192	0.067	4.040E-03	0.450	cis	SARS-CoV-2 infection
7757_5	DQA2	HLA class II histocompatibility antigen, DQ alpha 2 chain	P01906	HLA-DQA2	chr6:32741391	Inverse variance weighted	3	-0.075	0.026	4.303E-03	0.450	cis	SARS-CoV-2 infection
10015_119	KCAB2	Voltage-gated potassium channel subunit beta-2	Q13303	KCNAB2	chr1:5990927	Inverse variance weighted	2	-0.181	0.064	4.683E-03	0.461	cis	SARS-CoV-2 infection
19206_20	8ODP	7,8-dihydro-8-oxoguanine triphosphatase	P36639	NUDT1	chr7:2242226	Wald ratio	1	0.131	0.048	6.217E-03	0.578	cis	SARS-CoV-2 infection
6359_50	AGO61	Protein O-linked-mannose beta-1,4-N-acetylglucosaminyltransferase 2	Q8NAT1	POMGNT2	chr3:43106085	Inverse variance weighted	4	-0.049	0.018	6.585E-03	0.580	cis	SARS-CoV-2 infection
15509_2	NAG	Alpha-N-acetylglucosaminidase	P54802	NAGLU	chr17:42536241	Inverse variance weighted	6	0.024	0.009	7.679E-03	0.628	cis	SARS-CoV-2 infection
9092_33	ANGL1:N-term	Angiopoietin-related protein 1:N-term	O95841	ANGPTL1	chr1:178871077	Wald ratio	1	0.249	0.094	8.065E-03	0.628	cis	SARS-CoV-2 infection
10366_11	PDGFRA	Platelet-derived growth factor receptor alpha	P16234	PDGFRA	chr4:54229280	Inverse variance weighted	3	0.116	0.044	8.355E-03	0.628	cis	SARS-CoV-2 infection
10346_5	STAT3	Signal transducer and activator of transcription 3	P40763	STAT3	chr17:42388540	Inverse variance weighted	2	-0.099	0.038	8.908E-03	0.628	cis	SARS-CoV-2 infection
17447_52	SFRP4	Secreted frizzled-related protein 4	Q6FHJ7	SFRP4	chr7:38025695	Inverse variance weighted	2	-0.055	0.021	9.457E-03	0.628	cis	SARS-CoV-2 infection
5586_66	MINP1	Multiple inositol polyphosphate phosphatase 1	Q9UNW1	MINPP1	chr10:87504875	Wald ratio	1	0.156	0.061	1.049E-02	0.628	cis	SARS-CoV-2 infection
16915_153	SEM4A	Semaphorin-4A	Q9H3S1	SEMA4A	chr1:156147366	Wald ratio	1	-0.072	0.028	1.081E-02	0.628	cis	SARS-CoV-2 infection
9638_2	TIGIT	T-cell immunoreceptor with Ig and ITIM domains	Q495A1	TIGIT	chr3:114276913	Wald ratio	1	-0.161	0.063	1.088E-02	0.628	cis	SARS-CoV-2 infection
5532_53	bFGF-R	Fibroblast growth factor receptor 1	P11362	FGFR1	chr8:38468834	Inverse variance weighted	2	0.113	0.045	1.204E-02	0.628	cis	SARS-CoV-2 infection
3855_56	Peroxiredoxin-1	Peroxiredoxin-1	Q06830	PRDX1	chr1:45542732	Inverse variance weighted	2	-0.101	0.040	1.276E-02	0.628	cis	SARS-CoV-2 infection
2890_59	CCL28	C-C motif chemokine 28	Q9NRJ3	CCL28	chr5:43412391	Wald ratio	1	-0.217	0.087	1.293E-02	0.628	cis	SARS-CoV-2 infection
11480_1	Aldehyde dehydrogenase, class 3	Aldehyde dehydrogenase, dimeric NADP-preferring	P30838	ALDH3A1	chr17:19748943	Wald ratio	1	-0.097	0.039	1.297E-02	0.628	cis	SARS-CoV-2 infection
19376_74	NNMT	Nicotinamide N-methyltransferase	P40261	NNMT	chr11:114257787	Wald ratio	1	0.199	0.080	1.303E-02	0.628	cis	SARS-CoV-2 infection
3348_49	BMP-1	Bone morphogenetic protein 1	P13497	BMP1	chr8:22165140	Wald ratio	1	0.183	0.074	1.311E-02	0.628	cis	SARS-CoV-2 infection
4328_2	BOC	Brother of CDO	Q9BWV1	BOC	chr3:113211003	Inverse variance weighted	5	0.050	0.020	1.326E-02	0.628	cis	SARS-CoV-2 infection
7918_114	Amylase, alpha 1A	Alpha-amylase 1	P04745	AMY1A	chr1:103655760	Inverse variance weighted	11	0.041	0.017	1.332E-02	0.628	cis	SARS-CoV-2 infection
15503_20	Lefty-A	Left-right determination factor 2	O00292	LEFTY2	chr1:225941383	Inverse variance weighted	10	0.028	0.011	1.372E-02	0.628	cis	SARS-CoV-2 infection
15441_6	SAP3	Ganglioside GM2 activator	P17900	GM2A	chr5:151212150	Inverse variance weighted	3	-0.040	0.016	1.391E-02	0.628	cis	SARS-CoV-2 infection
9848_22	Cyclin H	Cyclin-H	P51946	CCNH	chr5:87412930	Wald ratio	1	-0.141	0.058	1.513E-02	0.628	cis	SARS-CoV-2 infection
18891_98	GBP2	Guanylate-binding protein 2	P32456	GBP2	chr1:89150456	Wald ratio	1	-0.163	0.067	1.520E-02	0.628	cis	SARS-CoV-2 infection
13481_24	TCEA2	Transcription elongation factor A protein 2	Q15560	TCEA2	chr20:64049836	Wald ratio	1	0.081	0.033	1.522E-02	0.628	cis	SARS-CoV-2 infection
2730_58	MICA	MHC class I polypeptide-related sequence A	Q29983	MICA	chr6:31399784	Inverse variance weighted	12	0.023	0.009	1.673E-02	0.628	cis	SARS-CoV-2 infection
12378_71	TPSN	Tapasin	O15533	TAPBP	chr6:33314284	Inverse variance weighted	5	0.023	0.009	1.720E-02	0.628	cis	SARS-CoV-2 infection
7849_3	Glutaminyl cyclase	Glutaminyl-peptide cyclotransferase	Q16769	QPCT	chr2:37342827	Inverse variance weighted	4	-0.061	0.026	1.768E-02	0.628	cis	SARS-CoV-2 infection
10534_40	PARP:BRCT domain	Poly [ADP-ribose] polymerase 1:BRCA1 C-terminal:BRCA1 C-terminus domian	P09874	PARP1	chr1:226408154	Wald ratio	1	-0.241	0.102	1.852E-02	0.628	cis	SARS-CoV-2 infection
5628_21	SEM3G	Semaphorin-3G	Q9NS98	SEMA3G	chr3:52445103	Inverse variance weighted	5	0.078	0.033	1.882E-02	0.628	cis	SARS-CoV-2 infection
4498_62	NCAM-120	Neural cell adhesion molecule 1, 120 kDa isoform	P13591	NCAM1	chr11:112961247	Inverse variance weighted	3	-0.032	0.014	1.960E-02	0.628	cis	SARS-CoV-2 infection
11302_237	TENR	Tenascin-R	Q92752	TNR	chr1:175743616	Wald ratio	1	0.085	0.036	1.987E-02	0.628	cis	SARS-CoV-2 infection
9754_33	Quinone reductase 2	Ribosyldihydronicotinamide dehydrogenase [quinone]	P16083	NQO2	chr6:2987987	Inverse variance weighted	8	0.019	0.008	2.065E-02	0.628	cis	SARS-CoV-2 infection
3617_80	HGFA	Hepatocyte growth factor activator	Q04756	HGFAC	chr4:3441968	Inverse variance weighted	12	0.017	0.007	2.070E-02	0.628	cis	SARS-CoV-2 infection
9017_58	LPH	Lactase-phlorizin hydrolase	P09848	LCT	chr2:135837184	Inverse variance weighted	4	-0.017	0.007	2.083E-02	0.628	cis	SARS-CoV-2 infection
3600_2	Chitotriosidase-1	Chitotriosidase-1	Q13231	CHIT1	chr1:203273641	Inverse variance weighted	6	0.020	0.009	2.095E-02	0.628	cis	SARS-CoV-2 infection
4568_17	SLIK5	SLIT and NTRK-like protein 5	O94991	SLITRK5	chr13:87671371	Inverse variance weighted	4	0.068	0.030	2.123E-02	0.628	cis	SARS-CoV-2 infection
8859_51	CAH11	Carbonic anhydrase-related protein 11	O75493	CA11	chr19:48646187	Wald ratio	1	0.103	0.045	2.172E-02	0.628	cis	SARS-CoV-2 infection
5542_22	NRP1	Neuropilin-1	O14786	NRP1	chr10:33336262	Inverse variance weighted	5	-0.033	0.015	2.195E-02	0.628	cis	SARS-CoV-2 infection
7140_1	ELA2A	Chymotrypsin-like elastase family member 2A	P08217	CELA2A	chr1:15456728	Wald ratio	1	0.119	0.052	2.240E-02	0.628	cis	SARS-CoV-2 infection
5111_15	NRX3B	Neurexin-3-beta	Q9HDB5	NRXN3	chr14:78170373	Inverse variance weighted	2	-0.166	0.073	2.249E-02	0.628	cis	SARS-CoV-2 infection
3234_23	URB	Coiled-coil domain-containing protein 80	Q76M96	CCDC80	chr3:112649530	Inverse variance weighted	2	-0.104	0.045	2.250E-02	0.628	cis	SARS-CoV-2 infection
8814_33	SAPL1	Proactivator polypeptide-like 1	Q6NUJ1	PSAPL1	chr4:7434930	Inverse variance weighted	8	0.029	0.013	2.293E-02	0.628	cis	SARS-CoV-2 infection

12356_65	Sorcin	Sorcin	P30626	SRI	chr7:88226993	Wald ratio	1	-0.096	0.042	2.315E-02	0.628	cis	SARS-CoV-2 infection
15480_2	VNN2	Vascular non-inflammatory molecule 2	O95498	VNN2	chr6:132763459	Inverse variance weighted	11	0.018	0.008	2.320E-02	0.628	cis	SARS-CoV-2 infection
8233_2	ITIH5	Inter-alpha-trypsin inhibitor heavy chain H5	Q86UX2	ITIH5	chr10:7666998	Inverse variance weighted	8	-0.026	0.012	2.348E-02	0.628	cis	SARS-CoV-2 infection
5128_53	SLAF6	SLAM family member 6	Q96DU3	SLAMF6	chr1:160523262	Wald ratio	1	-0.061	0.027	2.407E-02	0.628	cis	SARS-CoV-2 infection
2580_83	Myeloperoxidase	Myeloperoxidase	P05164	MPO	chr17:58280935	Inverse variance weighted	7	-0.031	0.014	2.431E-02	0.628	cis	SARS-CoV-2 infection
4479_14	C1-Esterase Inhibitor	Plasma protease C1 inhibitor	P05155	SERPING1	chr11:57597387	Inverse variance weighted	6	-0.025	0.011	2.454E-02	0.628	cis	SARS-CoV-2 infection
17680_12	EPHB1	Ephrin type-B receptor 1	P54762	EPHB1	chr3:134795260	Inverse variance weighted	3	0.031	0.014	2.473E-02	0.628	cis	SARS-CoV-2 infection
2615_60	Ephrin-A5	Ephrin-A5	P52803	EFNA5	chr5:107670937	Inverse variance weighted	2	0.073	0.033	2.482E-02	0.628	cis	SARS-CoV-2 infection
11514_196	CD59	CD59 glycoprotein	P13987	CD59	chr11:33736479	Inverse variance weighted	3	0.055	0.025	2.545E-02	0.628	cis	SARS-CoV-2 infection
3773_15	sTie-2	Angiopoietin-1 receptor, soluble	Q02763	TEK	chr9:27109141	Inverse variance weighted	4	0.049	0.022	2.587E-02	0.628	cis	SARS-CoV-2 infection
10702_1	COSA1	Collagen alpha-1(XXVIII) chain	Q2UY09	COL28A1	chr7:7535873	Wald ratio	1	0.228	0.103	2.665E-02	0.628	cis	SARS-CoV-2 infection
3339_33	TSP2	Thrombospondin-2	P35442	THBS2	chr6:169254050	Inverse variance weighted	7	-0.026	0.012	2.690E-02	0.628	cis	SARS-CoV-2 infection
8956_96	SREC-II:ECD	Scavenger receptor class F member 2:Extracellular domain	Q96GP6	SCARF2	chr22:20437826	Wald ratio	1	-0.099	0.045	2.693E-02	0.628	cis	SARS-CoV-2 infection
12449_16	PPIH	Peptidyl-prolyl cis-trans isomerase H	O43447	PPIH	chr1:42658335	Inverse variance weighted	2	0.142	0.064	2.705E-02	0.628	cis	SARS-CoV-2 infection
13545_97	EIF1A	Probable RNA-binding protein EIF1AD	Q8N9N8	EIF1AD	chr11:66002176	Inverse variance weighted	3	0.105	0.048	2.785E-02	0.638	cis	SARS-CoV-2 infection
19448_104	UPP1	Uridine phosphorylase 1	Q16831	UPP1	chr7:48088628	Wald ratio	1	0.115	0.053	2.906E-02	0.649	cis	SARS-CoV-2 infection
2828_82	HAI-1	Kunitz-type protease inhibitor 1	O43278	SPINT1	chr15:40844018	Inverse variance weighted	2	-0.052	0.024	2.912E-02	0.649	cis	SARS-CoV-2 infection
3593_72	Caspase-3	Caspase-3	P42574	CASP3	chr4:184650062	Wald ratio	1	0.089	0.041	2.953E-02	0.650	cis	SARS-CoV-2 infection
7769_29	3BP2	SH3 domain-binding protein 2	P78314	SH3BP2	chr4:2793071	Inverse variance weighted	3	0.064	0.029	3.012E-02	0.654	cis	SARS-CoV-2 infection
12558_3	UBS3B	Ubiquitin-associated and SH3 domain-containing protein B	Q8TF42	UBASH3B	chr11:122655722	Inverse variance weighted	2	-0.097	0.046	3.259E-02	0.696	cis	SARS-CoV-2 infection
9962_1	MUCDL	Cadherin-related family member 5	Q9HBB8	CDHR5	chr11:626078	Wald ratio	1	-0.184	0.086	3.286E-02	0.696	cis	SARS-CoV-2 infection
19207_119	ADK	Adenosine kinase	P55263	ADK	chr10:74151202	Wald ratio	1	0.131	0.062	3.348E-02	0.697	cis	SARS-CoV-2 infection
9478_69	KPRA	Phosphoribosyl pyrophosphate synthase-associated protein 1	Q14558	PRPSAP1	chr17:76384521	Wald ratio	1	-0.135	0.064	3.385E-02	0.697	cis	SARS-CoV-2 infection
9715_15	IGSF3	Immunoglobulin superfamily member 3	O75054	IGSF3	chr1:116667755	Inverse variance weighted	3	0.090	0.043	3.419E-02	0.697	cis	SARS-CoV-2 infection
5636_10	MFAP4	Microfibril-associated glycoprotein 4	P55083	MFAP4	chr17:19387190	Inverse variance weighted	2	0.053	0.025	3.575E-02	0.714	cis	SARS-CoV-2 infection
5457_5	COLEC12	Collectin-12	Q5KU26	COLEC12	chr18:500722	Inverse variance weighted	3	0.070	0.034	3.665E-02	0.714	cis	SARS-CoV-2 infection
16818_200	CDCP1	CUB domain-containing protein 1	Q9H5V8	CDCP1	chr3:45146422	Inverse variance weighted	3	-0.054	0.026	3.680E-02	0.714	cis	SARS-CoV-2 infection
6904_14	LRRT2	Leucine-rich repeat transmembrane neuronal protein 2	O43300	LRRTM2	chr5:138875368	Wald ratio	1	0.122	0.059	3.704E-02	0.714	cis	SARS-CoV-2 infection
4294_16	Sphingosine kinase 1	Sphingosine kinase 1	Q9NYA1	SPHK1	chr17:76376584	Inverse variance weighted	2	-0.097	0.047	3.715E-02	0.714	cis	SARS-CoV-2 infection
13381_49	B4GT1	Beta-1,4-galactosyltransferase 1	P15291	B4GALT1	chr9:33167356	Inverse variance weighted	2	-0.051	0.025	4.024E-02	0.736	cis	SARS-CoV-2 infection
16918_198	TLR3	Toll-like receptor 3	O15455	TLR3	chr4:186068911	Inverse variance weighted	8	0.023	0.011	4.033E-02	0.736	cis	SARS-CoV-2 infection
11211_7	TBCE	Tubulin-specific chaperone E	Q15813	TBCE	chr1:235367360	Inverse variance weighted	2	-0.085	0.041	4.065E-02	0.736	cis	SARS-CoV-2 infection
15324_58	Ferritin light chain	Ferritin light chain	P02792	FTL	chr19:48965309	Wald ratio	1	-0.207	0.101	4.082E-02	0.736	cis	SARS-CoV-2 infection
2985_35	Gro-a	Growth-regulated alpha protein	P09341	CXCL1	chr4:73869393	Inverse variance weighted	8	0.030	0.015	4.140E-02	0.736	cis	SARS-CoV-2 infection
5202_4	PPID	Peptidyl-prolyl cis-trans isomerase D	Q08752	PPID	chr4:158723396	Inverse variance weighted	2	-0.038	0.019	4.185E-02	0.736	cis	SARS-CoV-2 infection
5491_12	Testican-2	Testican-2	Q92563	SPOCK2	chr10:72089032	Wald ratio	1	-0.050	0.025	4.218E-02	0.736	cis	SARS-CoV-2 infection
16302_11	SPLC2	BPI fold-containing family A member 2	Q96DR5	BPIFA2	chr20:33161768	Wald ratio	1	-0.149	0.073	4.240E-02	0.736	cis	SARS-CoV-2 infection
2644_11	PKC-A	Protein kinase C alpha type	P17252	PRKCA	chr17:66302613	Wald ratio	1	-0.237	0.117	4.243E-02	0.736	cis	SARS-CoV-2 infection
13388_57	NEC1	Neuroendocrine convertase 1	P29120	PCSK1	chr5:96434143	Inverse variance weighted	7	-0.025	0.012	4.268E-02	0.736	cis	SARS-CoV-2 infection
15316_262	TXN4B	Thioredoxin-like protein 4B	Q9NX01	TXNL4B	chr16:72094431	Wald ratio	1	0.109	0.054	4.407E-02	0.738	cis	SARS-CoV-2 infection
3003_29	NKp30	Natural cytotoxicity triggering receptor 3	O14931	NCR3	chr6:31593006	Wald ratio	1	0.134	0.067	4.420E-02	0.738	cis	SARS-CoV-2 infection
13671_40	Elastase	Neutrophil elastase	P08246	ELANE	chr19:851014	Inverse variance weighted	5	0.049	0.024	4.432E-02	0.738	cis	SARS-CoV-2 infection
7198_197	FA20B	Glycosaminoglycan xylosylkinase	O75063	FAM20B	chr1:179025804	Inverse variance weighted	2	-0.095	0.047	4.481E-02	0.738	cis	SARS-CoV-2 infection
17514_48	RAB21	Ras-related protein Rab-21	Q9UL25	RAB21	chr12:71754863	Wald ratio	1	-0.126	0.063	4.549E-02	0.738	cis	SARS-CoV-2 infection
4546_27	EMR2	Adhesion G protein-coupled receptor E2	Q9UHX3	ADGRE2	chr19:14778560	Inverse variance weighted	5	0.027	0.013	4.574E-02	0.738	cis	SARS-CoV-2 infection
11356_19	DGC14	Protein DGCR14	Q96DF8	ESS2	chr22:19144684	Wald ratio	1	-0.089	0.045	4.590E-02	0.738	cis	SARS-CoV-2 infection
2844_53	sTie-1	Tyrosine-protein kinase receptor Tie-1, soluble	P35590	TIE1	chr1:43300982	Inverse variance weighted	4	0.041	0.021	4.735E-02	0.754	cis	SARS-CoV-2 infection
15515_2	SAA	Serum amyloid A-1 protein	P0DJI8	SAA1	chr11:18266260	Inverse variance weighted	4	0.079	0.040	4.897E-02	0.767	cis	SARS-CoV-2 infection
18188_12	GATM	Glycine amidinotransferase, mitochondrial	P50440	GATM	chr15:45402327	Wald ratio	1	-0.056	0.028	4.905E-02	0.767	cis	SARS-CoV-2 infection
6629_3	HBD-1	Beta-defensin 1	P60022	DEFB1	chr8:6877936	Inverse variance weighted	5	-0.025	0.013	5.075E-02	0.776	cis	SARS-CoV-2 infection
12376_85	p19-INK4d	Cyclin-dependent kinase 4 inhibitor D	P55273	CDKN2D	chr19:10569059	Wald ratio	1	-0.212	0.108	5.096E-02	0.776	cis	SARS-CoV-2 infection
15641_20	TEFF1	Tomoregulin-1	Q8IYR6	TMEFF1	chr9:100473149	Wald ratio	1	-0.168	0.087	5.157E-02	0.776	cis	SARS-CoV-2 infection
6414_8	OAF	Out at first protein homolog	Q86UD1	OAF	chr11:120211032	Inverse variance weighted	5	-0.019	0.010	5.226E-02	0.776	cis	SARS-CoV-2 infection
3890_8	LDH-H 1	L-lactate dehydrogenase B chain	P07195	LDHB	chr12:21757857	Wald ratio	1	0.226	0.117	5.295E-02	0.776	cis	SARS-CoV-2 infection
19267_14	GLO2	Hydroxyacylglutathione hydrolase, mitochondrial	Q16775	HAGH	chr16:1827157	Wald ratio	1	0.064	0.033	5.313E-02	0.776	cis	SARS-CoV-2 infection
2418_55	Apo E	Apolipoprotein E	P02649	APOE	chr19:44905791	Inverse variance weighted	4	0.060	0.031	5.351E-02	0.776	cis	SARS-CoV-2 infection
2849_49	AIF1	Allograft inflammatory factor 1	P55008	AIF1	chr6:31615217	Inverse variance weighted	2	0.148	0.077	5.407E-02	0.776	cis	SARS-CoV-2 infection
5740_17	ROBO1	Roundabout homolog 1	Q9Y6N7	ROBO1	chr3:79767998	Inverse variance weighted	3	0.070	0.036	5.433E-02	0.776	cis	SARS-CoV-2 infection
8969_49	CD14	Monocyte differentiation antigen CD14	P08571	CD14	chr5:140633700	Wald ratio	1	-0.240	0.125	5.475E-02	0.776	cis	SARS-CoV-2 infection
10754_113	Prokineticin-2	Prokineticin-2	Q9HC23	PROK2	chr3:71785206	Inverse variance weighted	6	-0.021	0.011	5.501E-02	0.776	cis	SARS-CoV-2 infection
15386_7	FABPA	Fatty acid-binding protein, adipocyte	P15090	FABP4	chr8:81483236	Wald ratio	1	-0.117	0.061	5.585E-02	0.776	cis	SARS-CoV-2 infection
17726_3	SAR1A	GTP-binding protein SAR1a	Q9NR31	SAR1A	chr10:70170523	Inverse variance weighted	2	0.099	0.052	5.647E-02	0.776	cis	SARS-CoV-2 infection
18295_102	GRHPR	Glyoxylate reductase/hydroxypyruvate reductase	Q9UBQ7	GRHPR	chr9:37422666	Inverse variance weighted	3	-0.033	0.017	5.713E-02	0.776	cis	SARS-CoV-2 infection
4464_10	Sialoadhesin	Sialoadhesin	Q9BZZ2	SIGLEC1	chr20:3712600	Wald ratio	1	0.198	0.104	5.738E-02	0.776	cis	SARS-CoV-2 infection
3280_49	Aggrecan	Aggrecan core protein	P16112	ACAN	chr15:88803436	Inverse variance weighted	2	0.069	0.037	5.789E-02	0.776	cis	SARS-CoV-2 infection
9201_13	Transgelin-2	Transgelin-2	P37802	TAGLN2	chr1:159925507	Wald ratio	1	-0.194	0.103	5.866E-02	0.776	cis	SARS-CoV-2 infection
2750_3	Apo A-I	Apolipoprotein A-I	P02647	APOA1	chr11:116837622	Inverse variance weighted	3	-0.042	0.022	5.867E-02	0.776	cis	SARS-CoV-2 infection
6965_19	CNTP2	Contactin-associated protein-like 2	Q9UHC6	CNTNAP2	chr7:146116002	Inverse variance weighted	5	0.023	0.012	5.881E-02	0.776	cis	SARS-CoV-2 infection
6955_68	SNX1	Sorting nexin-1	Q13596	SNX1	chr15:64094123	Wald ratio	1	-0.072	0.038	5.890E-02	0.776	cis	SARS-CoV-2 infection
8397_147	QSOX2	Sulfhydryl oxidase 2	Q6ZRP7	QSOX2	chr9:136245812	Inverse variance weighted	7	0.022	0.012	6.016E-02	0.783	cis	SARS-CoV-2 infection
6462_12	TIMP-4	Metalloproteinase inhibitor 4	Q99727	TIMP4	chr3:12158912	Inverse variance weighted	8	-0.032	0.017	6.046E-02	0.783	cis	SARS-CoV-2 infection
15453_3	a1-Microglobulin	Alpha-1-microglobulin	P02760	AMBP	chr9:114078328	Inverse variance weighted	2	-0.079	0.042	6.084E-02	0.783	cis	SARS-CoV-2 infection
11142_11	ANGL1:C-term	Angiopoietin-related protein 1:C-Term, Fibrinogen domian	O95841	ANGPTL1	chr1:178871077	Inverse variance weighted	4	0.029	0.016	6.139E-02	0.784	cis	SARS-CoV-2 infection
12637_7	CACP	Carnitine O-acetyltransferase	P43155	CRAT	chr9:129111189	Wald ratio	1	0.103	0.055	6.207E-02	0.787	cis	SARS-CoV-2 infection
9876_20	aldolase C	Fructose-bisphosphate aldolase C	P09972	ALDOC	chr17:28576948	Inverse variance weighted	2	-0.132	0.071	6.343E-02	0.795	cis	SARS-CoV-2 infection
19556_12	Complement receptor type 1	Complement receptor type 1	P17927	CR1	chr1:207496147	Inverse variance weighted	10	-0.034	0.018	6.366E-02	0.795	cis	SARS-CoV-2 infection
8759_29	a1,4-Galactosyltransferase	Lactosylceramide 4-alpha-galactosyltransferase	Q9NPC4	A4GALT	chr22:42721298	Inverse variance weighted	4	0.026	0.014	6.487E-02	0.800	cis	SARS-CoV-2 infection
4276_10	prostatic binding protein	Phosphatidylethanolamine-binding protein 1	P30086	PEBP1	chr12:118136124	Inverse variance weighted	7	-0.029	0.016	6.527E-02	0.800	cis	SARS-CoV-2 infection

16606_85	Aldose reductase	Aldose reductase	P15121	AKR1B1	chr7:134459284	Inverse variance weighted	2	0.080	0.044	6.568E-02	0.800	cis	SARS-CoV-2 infection
14205_6	HEXI2	Protein HEXIM2	Q96MH2	HEXIM2	chr17:45160700	Wald ratio	1	0.203	0.111	6.643E-02	0.800	cis	SARS-CoV-2 infection
15487_164	carboxylesterase, liver	Liver carboxylesterase 1	P23141	CES1	chr16:55833337	Wald ratio	1	-0.198	0.108	6.645E-02	0.800	cis	SARS-CoV-2 infection
3484_60	Angiotensinogen	Angiotensinogen	P01019	AGT	chr1:230745576	Inverse variance weighted	2	-0.046	0.025	6.762E-02	0.802	cis	SARS-CoV-2 infection
3323_37	LRP8	Low-density lipoprotein receptor-related protein 8	Q14114	LRP8	chr1:53328469	Inverse variance weighted	2	-0.045	0.025	6.764E-02	0.802	cis	SARS-CoV-2 infection
13463_1	PXDN	Peroxidasin homolog	Q92626	PXDN	chr2:1744852	Inverse variance weighted	4	0.070	0.039	6.962E-02	0.809	cis	SARS-CoV-2 infection
14131_37	EFNB2:ECD	Ephrin-B2:Extracellular domain	P52799	EFNB2	chr13:106535662	Inverse variance weighted	2	0.116	0.064	6.976E-02	0.809	cis	SARS-CoV-2 infection
12432_23	CYBP	Calcyclin-binding protein	Q9HB71	CACYBP	chr1:174999163	Wald ratio	1	-0.064	0.035	7.004E-02	0.809	cis	SARS-CoV-2 infection
8464_31	RSPO4	R-spondin-4	Q2I0M5	RSPO4	chr20:1002311	Inverse variance weighted	2	0.089	0.049	7.013E-02	0.809	cis	SARS-CoV-2 infection
15388_24	FcRIIIa	Low affinity immunoglobulin gamma Fc region receptor III-A	P08637	FCGR3A	chr1:161550968	Inverse variance weighted	6	-0.030	0.016	7.146E-02	0.813	cis	SARS-CoV-2 infection
16856_79	MARE2	Microtubule-associated protein RP/EB family member 2	Q15555	MAPRE2	chr18:34976928	Wald ratio	1	-0.057	0.031	7.176E-02	0.813	cis	SARS-CoV-2 infection
13748_4	MCP-2	C-C motif chemokine 8	P80075	CCL8	chr17:34319435	Inverse variance weighted	7	0.027	0.015	7.198E-02	0.813	cis	SARS-CoV-2 infection
4996_66	HRG	Histidine-rich glycoprotein	P04196	HRG	chr3:186660216	Inverse variance weighted	5	0.024	0.013	7.270E-02	0.814	cis	SARS-CoV-2 infection
12395_86	SYDM	Aspartate--tRNA ligase, mitochondrial	Q6PI48	DARS2	chr1:173824653	Wald ratio	1	-0.096	0.054	7.297E-02	0.814	cis	SARS-CoV-2 infection
11104_13	YKL-40	Chitinase-3-like protein 1	P36222	CHI3L1	chr1:203186704	Inverse variance weighted	7	-0.015	0.008	7.443E-02	0.824	cis	SARS-CoV-2 infection
2966_65	SCGF-beta	Stem cell growth factor-beta	Q9Y240	CLEC11A	chr19:50723364	Wald ratio	1	0.249	0.140	7.492E-02	0.824	cis	SARS-CoV-2 infection
18917_53	Pancreatic alpha-amylase	Pancreatic alpha-amylase	P04746	AMY2A	chr1:103617427	Inverse variance weighted	4	-0.053	0.030	7.647E-02	0.834	cis	SARS-CoV-2 infection
19372_7	MDGA2	MAM domain-containing glycosylphosphatidylinositol anchor protein 2	Q7Z553	MDGA2	chr14:47675605	Inverse variance weighted	11	-0.014	0.008	7.680E-02	0.834	cis	SARS-CoV-2 infection
2617_56	ERBB3	Receptor tyrosine-protein kinase erbB-3	P21860	ERBB3	chr12:56076799	Wald ratio	1	0.077	0.044	7.765E-02	0.838	cis	SARS-CoV-2 infection
10605_22	APMAP	Adipocyte plasma membrane-associated protein	Q9HDC9	APMAP	chr20:24992751	Wald ratio	1	0.147	0.084	7.838E-02	0.840	cis	SARS-CoV-2 infection
17856_23	NT5M	5'(3')-deoxyribonucleotidase, mitochondrial	Q9NPB1	NT5M	chr17:17303335	Wald ratio	1	-0.081	0.046	7.895E-02	0.841	cis	SARS-CoV-2 infection
6947_4	SIA10	Type 2 lactosamine alpha-2,3-sialyltransferase	Q9Y274	ST3GAL6	chr3:98732236	Inverse variance weighted	9	-0.013	0.007	8.012E-02	0.848	cis	SARS-CoV-2 infection
2743_5	Sonic Hedgehog	Sonic hedgehog protein	Q15465	SHH	chr7:155812463	Inverse variance weighted	2	-0.073	0.042	8.112E-02	0.852	cis	SARS-CoV-2 infection
13107_9	LYPD3	Ly6/PLAUR domain-containing protein 3	O95274	LYPD3	chr19:43465608	Wald ratio	1	0.093	0.053	8.150E-02	0.852	cis	SARS-CoV-2 infection
6574_11	FAIM3	Fas apoptotic inhibitory molecule 3	O60667	FCMR	chr1:206923247	Inverse variance weighted	2	-0.122	0.070	8.284E-02	0.852	cis	SARS-CoV-2 infection
8894_80	hnRNP A/B	Heterogeneous nuclear ribonucleoprotein A/B	Q99729	HNRNPAB	chr5:178204533	Wald ratio	1	0.118	0.068	8.321E-02	0.852	cis	SARS-CoV-2 infection
6408_2	INHBC	Inhibin beta C chain	P55103	INHBC	chr12:57434784	Inverse variance weighted	2	0.090	0.052	8.326E-02	0.852	cis	SARS-CoV-2 infection
7950_142	BTNL9	Butyrophilin-like protein 9	Q6UXG8	BTNL9	chr5:181040225	Wald ratio	1	-0.130	0.075	8.452E-02	0.852	cis	SARS-CoV-2 infection
3212_30	ASAH2	Neutral ceramidase	Q9NR71	ASAH2	chr10:50279720	Inverse variance weighted	6	-0.016	0.009	8.513E-02	0.852	cis	SARS-CoV-2 infection
14112_40	RELT	Tumor necrosis factor receptor superfamily member 19L	Q969Z4	RELT	chr11:73376399	Wald ratio	1	-0.166	0.097	8.559E-02	0.852	cis	SARS-CoV-2 infection
19347_37	Carbonic Anhydrase XII	Carbonic anhydrase 12	O43570	CA12	chr15:63381846	Wald ratio	1	0.176	0.102	8.573E-02	0.852	cis	SARS-CoV-2 infection
11441_11	PYGL	Glycogen phosphorylase, liver form	P06737	PYGL	chr14:50944483	Inverse variance weighted	2	-0.067	0.039	8.744E-02	0.852	cis	SARS-CoV-2 infection
11369_23	ADHX	Alcohol dehydrogenase class-3	P11766	ADH5	chr4:99088801	Inverse variance weighted	3	-0.109	0.064	8.808E-02	0.852	cis	SARS-CoV-2 infection
10521_10	MXRA8:ECD	Matrix-remodeling-associated protein 8:Extracellular domain	Q9BRK3	MXRA8	chr1:1361777	Wald ratio	1	-0.089	0.052	8.908E-02	0.852	cis	SARS-CoV-2 infection
3858_5	PPAC	Low molecular weight phosphotyrosine protein phosphatase	P24666	ACP1	chr2:264140	Inverse variance weighted	7	-0.016	0.009	8.993E-02	0.852	cis	SARS-CoV-2 infection
7948_129	GLTD2	Glycolipid transfer protein domain-containing protein 2	A6NH11	GLTPD2	chr17:4788964	Inverse variance weighted	3	0.035	0.021	9.114E-02	0.852	cis	SARS-CoV-2 infection
2201_17	Endostatin	Endostatin	P39060	COL18A1	chr21:45405165	Inverse variance weighted	4	-0.045	0.027	9.151E-02	0.852	cis	SARS-CoV-2 infection
17156_72	DCAK1	Serine/threonine-protein kinase DCLK1	O15075	DCLK1	chr13:36131382	Inverse variance weighted	3	0.040	0.023	9.161E-02	0.852	cis	SARS-CoV-2 infection
17408_2	PMM1	Phosphomannomutase 1	Q92871	PMM1	chr22:41589871	Wald ratio	1	0.159	0.095	9.193E-02	0.852	cis	SARS-CoV-2 infection
16035_8	VEGF sR3	Vascular endothelial growth factor receptor 3	P35916	FLT4	chr5:180649624	Inverse variance weighted	3	-0.062	0.037	9.196E-02	0.852	cis	SARS-CoV-2 infection
13473_55	IP3KA	Inositol-trisphosphate 3-kinase A	P23677	ITPKA	chr15:41493393	Wald ratio	1	0.114	0.068	9.277E-02	0.852	cis	SARS-CoV-2 infection
13692_154	WISP-1	WNT1-inducible-signaling pathway protein 1	O95388	CCN4	chr8:133191039	Inverse variance weighted	8	-0.016	0.010	9.287E-02	0.852	cis	SARS-CoV-2 infection
14208_3	RET7	Retinoid-binding protein 7	Q96R05	RBP7	chr1:9997206	Inverse variance weighted	2	0.066	0.039	9.348E-02	0.852	cis	SARS-CoV-2 infection
6342_10	Nephronectin	Nephronectin	Q6UXI9	NPNT	chr4:105894775	Inverse variance weighted	3	0.042	0.025	9.353E-02	0.852	cis	SARS-CoV-2 infection
19504_22	KTHY	Thymidylate kinase	P23919	DTYMK	chr2:241686944	Inverse variance weighted	3	0.102	0.061	9.356E-02	0.852	cis	SARS-CoV-2 infection
13130_150	HXK2	Hexokinase-2	P52789	HK2	chr2:74834127	Inverse variance weighted	2	-0.101	0.060	9.392E-02	0.852	cis	SARS-CoV-2 infection
17694_32	PSME2	Proteasome activator complex subunit 2	Q9UL46	PSME2	chr14:24147570	Inverse variance weighted	3	-0.041	0.024	9.413E-02	0.852	cis	SARS-CoV-2 infection
15486_126	ABP1	Amiloride-sensitive amine oxidase [copper-containing]	P19801	AOC1	chr7:150824627	Inverse variance weighted	10	-0.014	0.008	9.560E-02	0.852	cis	SARS-CoV-2 infection
4342_10	sICAM-1	Intercellular adhesion molecule 1	P05362	ICAM1	chr19:10271093	Inverse variance weighted	9	0.015	0.009	9.578E-02	0.852	cis	SARS-CoV-2 infection
17682_1	CD46	Membrane cofactor protein	P15529	CD46	chr1:207752037	Inverse variance weighted	2	0.044	0.027	9.605E-02	0.852	cis	SARS-CoV-2 infection
7244_16	TM149	IGF-like family receptor 1	Q9H665	IGFLR1	chr19:35742453	Inverse variance weighted	4	0.017	0.010	9.615E-02	0.852	cis	SARS-CoV-2 infection
13733_5	IL-12 p40	Interleukin-12 subunit beta	P29460	IL12B	chr5:159330863	Inverse variance weighted	7	0.020	0.012	9.642E-02	0.852	cis	SARS-CoV-2 infection
13590_1	ORN	Oligoribonuclease, mitochondrial	Q9Y3B8	REXO2	chr11:114439435	Wald ratio	1	-0.069	0.041	9.688E-02	0.852	cis	SARS-CoV-2 infection
2991_9	IL-1 sRI	Interleukin-1 receptor type 1	P14778	IL1R1	chr2:102064544	Inverse variance weighted	2	-0.069	0.042	9.719E-02	0.852	cis	SARS-CoV-2 infection
10603_1	HIS3	Histatin-3	P15516	HTN3	chr4:70028455	Inverse variance weighted	3	-0.060	0.036	9.802E-02	0.852	cis	SARS-CoV-2 infection
7059_14	LIRA6	Leukocyte immunoglobulin-like receptor subfamily A member 6	Q6PI73	LILRA6	chr19:54242791	Inverse variance weighted	7	0.030	0.018	9.849E-02	0.852	cis	SARS-CoV-2 infection
5350_14	GPC6	Glypican-6	Q9Y625	GPC6	chr13:93226807	Wald ratio	1	0.166	0.101	9.918E-02	0.852	cis	SARS-CoV-2 infection
3325_2	MATN2	Matrilin-2	O00339	MATN2	chr8:97868840	Inverse variance weighted	4	0.050	0.030	9.938E-02	0.852	cis	SARS-CoV-2 infection
10977_55	UCMA	Unique cartilage matrix-associated protein	Q8WVF2	UCMA	chr10:13234374	Inverse variance weighted	6	0.025	0.015	9.946E-02	0.852	cis	SARS-CoV-2 infection
19279_42	CRBP	Retinol-binding protein 1	P09455	RBP1	chr3:139539829	Wald ratio	1	0.118	0.072	9.982E-02	0.852	cis	SARS-CoV-2 infection
12387_7	PDLI4	PDZ and LIM domain protein 4	P50479	PDLIM4	chr5:132257696	Wald ratio	1	-0.049	0.030	1.003E-01	0.852	cis	SARS-CoV-2 infection
4278_14	Protein disulfide-isomerase	Protein disulfide-isomerase	P07237	P4HB	chr17:81860856	Wald ratio	1	-0.067	0.041	1.015E-01	0.858	cis	SARS-CoV-2 infection
5605_77	MFNG	Beta-1,3-N-acetylglucosaminyltransferase manic fringe	O00587	MFNG	chr22:37486393	Wald ratio	1	0.073	0.045	1.041E-01	0.874	cis	SARS-CoV-2 infection
7551_33	LRC32	Leucine-rich repeat-containing protein 32	Q14392	LRRC32	chr11:76670747	Wald ratio	1	-0.127	0.078	1.045E-01	0.874	cis	SARS-CoV-2 infection
6984_6	IGSF8	Immunoglobulin superfamily member 8	Q969P0	IGSF8	chr1:160098943	Inverse variance weighted	2	0.051	0.032	1.079E-01	0.879	cis	SARS-CoV-2 infection
5900_11	HINT1	Histidine triad nucleotide-binding protein 1	P49773	HINT1	chr5:131224468	Wald ratio	1	0.103	0.064	1.080E-01	0.879	cis	SARS-CoV-2 infection
4254_6	NUDC3	NudC domain-containing protein 3	Q8IVD9	NUDCD3	chr7:44490658	Wald ratio	1	0.116	0.072	1.080E-01	0.879	cis	SARS-CoV-2 infection
9312_8	AZGP1	Zinc-alpha-2-glycoprotein	P25311	AZGP1	chr7:99976042	Inverse variance weighted	3	-0.111	0.069	1.086E-01	0.879	cis	SARS-CoV-2 infection
2837_3	Met	Hepatocyte growth factor receptor	P08581	MET	chr7:116672196	Inverse variance weighted	4	0.049	0.031	1.094E-01	0.879	cis	SARS-CoV-2 infection
17331_138	KREM1	Kremen protein 1	Q96MU8	KREMEN1	chr22:29073035	Inverse variance weighted	3	-0.038	0.024	1.096E-01	0.879	cis	SARS-CoV-2 infection
17748_21	QORX	Quinone oxidoreductase PIG3	Q53FA7	TP53I3	chr2:24085861	Inverse variance weighted	5	-0.031	0.019	1.106E-01	0.879	cis	SARS-CoV-2 infection
15582_25	FCN1	Ficolin-1	O00602	FCN1	chr9:134917912	Inverse variance weighted	5	0.027	0.017	1.116E-01	0.879	cis	SARS-CoV-2 infection
12366_16	CRGD	Gamma-crystallin D	P07320	CRYGD	chr2:208124524	Wald ratio	1	0.069	0.043	1.117E-01	0.879	cis	SARS-CoV-2 infection
5451_1	ALCAM	CD166 antigen	Q13740	ALCAM	chr3:105366909	Inverse variance weighted	4	0.059	0.037	1.120E-01	0.879	cis	SARS-CoV-2 infection
5459_33	CYTN	Cystatin-SN	P01037	CST1	chr20:23751268	Inverse variance weighted	5	0.058	0.036	1.120E-01	0.879	cis	SARS-CoV-2 infection
6364_7	TPSNR	Tapasin-related protein	Q9BX59	TAPBPL	chr12:6451690	Inverse variance weighted	7	-0.015	0.009	1.123E-01	0.879	cis	SARS-CoV-2 infection
5109_24	Nr-CAM	Neuronal cell adhesion molecule	Q92823	NRCAM	chr7:108456717	Wald ratio	1	0.126	0.079	1.127E-01	0.879	cis	SARS-CoV-2 infection
17419_17	TES	Testin	Q9UGI8	TES	chr7:116210506	Wald ratio	1	-0.105	0.066	1.127E-01	0.879	cis	SARS-CoV-2 infection

8841_65	CILP2	Cartilage intermediate layer protein 2	Q8IUL8	CILP2	chr19:19538248	Wald ratio	1	0.109	0.068	1.130E-01	0.879	cis	SARS-CoV-2 infection
8768_4	Bcl-10	B-cell lymphoma/leukemia 10	O95999	BCL10	chr1:85276632	Wald ratio	1	-0.143	0.091	1.154E-01	0.888	cis	SARS-CoV-2 infection
5691_2	CRLD2	Cysteine-rich secretory protein LCCL domain-containing 2	Q9H0B8	CRISPLD2	chr16:84819985	Inverse variance weighted	6	0.043	0.028	1.157E-01	0.888	cis	SARS-CoV-2 infection
5722_78	Prolylcarboxypeptidase	Lysosomal Pro-X carboxypeptidase	P42785	PRCP	chr11:82970584	Inverse variance weighted	3	0.042	0.027	1.184E-01	0.888	cis	SARS-CoV-2 infection
18222_34	SHLB2	Endophilin-B2	Q9NR46	SH3GLB2	chr9:129028331	Inverse variance weighted	3	-0.033	0.021	1.187E-01	0.888	cis	SARS-CoV-2 infection
18183_3	ARH	Low density lipoprotein receptor adapter protein 1	Q5SW96	LDLRAP1	chr1:25543606	Inverse variance weighted	2	0.074	0.047	1.193E-01	0.888	cis	SARS-CoV-2 infection
17686_27	TBCB	Tubulin-folding cofactor B	Q99426	TBCB	chr19:36114289	Wald ratio	1	0.118	0.076	1.195E-01	0.888	cis	SARS-CoV-2 infection
3314_74	GFRa-1	GDNF family receptor alpha-1	P56159	GFRA1	chr10:116276803	Wald ratio	1	0.041	0.026	1.196E-01	0.888	cis	SARS-CoV-2 infection
2436_49	CXCL16, soluble	C-X-C motif chemokine 16	Q9H2A7	CXCL16	chr17:4739928	Inverse variance weighted	3	0.039	0.025	1.210E-01	0.888	cis	SARS-CoV-2 infection
3060_43	C9	Complement component C9	P02748	C9	chr5:39371324	Inverse variance weighted	5	-0.036	0.023	1.211E-01	0.888	cis	SARS-CoV-2 infection
3352_80	Carbonic anhydrase 6	Carbonic anhydrase 6	P23280	CA6	chr1:8945867	Inverse variance weighted	6	-0.015	0.010	1.213E-01	0.888	cis	SARS-CoV-2 infection
10916_44	PLA2R	Secretory phospholipase A2 receptor	Q13018	PLA2R1	chr2:160062615	Inverse variance weighted	6	0.014	0.009	1.216E-01	0.888	cis	SARS-CoV-2 infection
14054_17	IL-15 Ra	Interleukin-15 receptor subunit alpha	Q13261	IL15RA	chr10:5978187	Inverse variance weighted	5	0.022	0.014	1.220E-01	0.888	cis	SARS-CoV-2 infection
3175_51	ATS13	A disintegrin and metalloproteinase with thrombospondin motifs 13	Q76LX8	ADAMTS13	chr9:133414358	Inverse variance weighted	7	0.016	0.010	1.226E-01	0.888	cis	SARS-CoV-2 infection
17765_3	SPN1	Snurportin-1	O95149	SNUPN	chr15:75626469	Wald ratio	1	0.059	0.038	1.228E-01	0.888	cis	SARS-CoV-2 infection
9329_28	TRML1:ECD	Trem-like transcript 1 protein:Extracellular domain, Ig-like V-type domain	Q86YW5	TREML1	chr6:41154347	Inverse variance weighted	2	-0.067	0.044	1.249E-01	0.888	cis	SARS-CoV-2 infection
3151_6	IL-2 sRa	Interleukin-2 receptor subunit alpha	P01589	IL2RA	chr10:6062370	Wald ratio	1	-0.082	0.054	1.256E-01	0.888	cis	SARS-CoV-2 infection
3029_52	DC-SIGN	CD209 antigen	Q9NNX6	CD209	chr19:7747564	Inverse variance weighted	4	0.030	0.020	1.266E-01	0.888	cis	SARS-CoV-2 infection
4971_1	CATZ	Cathepsin Z	Q9UBR2	CTSZ	chr20:59008238	Inverse variance weighted	5	-0.032	0.021	1.275E-01	0.888	cis	SARS-CoV-2 infection
6713_4	LRP11	Low-density lipoprotein receptor-related protein 11	Q86VZ4	LRP11	chr6:149864359	Inverse variance weighted	8	-0.019	0.013	1.279E-01	0.888	cis	SARS-CoV-2 infection
16605_2	C1T9A	Complement C1q and tumor necrosis factor-related protein 9A	P0C862	C1QTNF9	chr13:24307166	Wald ratio	1	-0.037	0.025	1.280E-01	0.888	cis	SARS-CoV-2 infection
17224_12	MIME	Mimecan	P20774	OGN	chr9:92404696	Inverse variance weighted	6	0.037	0.024	1.281E-01	0.888	cis	SARS-CoV-2 infection
15585_304	fibulin 5	Fibulin-5	Q9UBX5	FBLN5	chr14:91947987	Wald ratio	1	0.094	0.062	1.292E-01	0.888	cis	SARS-CoV-2 infection
3651_50	VEGF sR2	Vascular endothelial growth factor receptor 2	P35968	KDR	chr4:55125595	Inverse variance weighted	3	0.019	0.013	1.298E-01	0.888	cis	SARS-CoV-2 infection
3866_7	SBDS	Ribosome maturation protein SBDS	Q9Y3A5	SBDS	chr7:66995693	Wald ratio	1	0.060	0.040	1.308E-01	0.888	cis	SARS-CoV-2 infection
11248_43	HEM4	Uroporphyrinogen-III synthase	P10746	UROS	chr10:125823258	Inverse variance weighted	4	-0.037	0.025	1.320E-01	0.888	cis	SARS-CoV-2 infection
13093_6	SECTM1	Secreted and transmembrane protein 1	Q8WVN6	SECTM1	chr17:82334074	Inverse variance weighted	3	-0.037	0.025	1.325E-01	0.888	cis	SARS-CoV-2 infection
17138_8	GST A1-1	Glutathione S-transferase A1	P08263	GSTA1	chr6:52803860	Inverse variance weighted	6	0.024	0.016	1.339E-01	0.888	cis	SARS-CoV-2 infection
13580_2	Sperm-associated antigen 2	UDP-N-acetylhexosamine pyrophosphorylase	Q16222	UAP1	chr1:162561722	Wald ratio	1	0.157	0.105	1.342E-01	0.888	cis	SARS-CoV-2 infection
12498_12	TX1B3	Tax1-binding protein 3	O14907	TAX1BP3	chr17:3668679	Wald ratio	1	-0.079	0.053	1.356E-01	0.888	cis	SARS-CoV-2 infection
3296_92	CNTN2	Contactin-2	Q02246	CNTN2	chr1:205042937	Inverse variance weighted	9	0.020	0.013	1.358E-01	0.888	cis	SARS-CoV-2 infection
4775_34	Gelsolin	Gelsolin	P06396	GSN	chr9:121207794	Inverse variance weighted	2	-0.062	0.042	1.358E-01	0.888	cis	SARS-CoV-2 infection
18386_36	GLRX1	Glutaredoxin-1	P35754	GLRX	chr5:95822726	Wald ratio	1	-0.128	0.086	1.360E-01	0.888	cis	SARS-CoV-2 infection
14688_6	PTN7	Tyrosine-protein phosphatase non-receptor type 7	P35236	PTPN7	chr1:202161588	Wald ratio	1	-0.154	0.103	1.366E-01	0.888	cis	SARS-CoV-2 infection
13472_35	HDHD2	Haloacid dehalogenase-like hydrolase domain-containing protein 2	Q9H0R4	HDHD2	chr18:47150500	Inverse variance weighted	3	0.030	0.020	1.366E-01	0.888	cis	SARS-CoV-2 infection
5095_21	KI2L4	Killer cell immunoglobulin-like receptor 2DL4	Q99706	KIR2DL4	chr19:54803610	Inverse variance weighted	2	-0.038	0.026	1.368E-01	0.888	cis	SARS-CoV-2 infection
11969_5	S100A2	Protein S100-A2	P29034	S100A2	chr1:153567890	Wald ratio	1	-0.168	0.113	1.370E-01	0.888	cis	SARS-CoV-2 infection
18330_7	PGM2	Phosphoglucomutase-2	Q96G03	PGM2	chr4:37826660	Inverse variance weighted	3	-0.032	0.021	1.379E-01	0.888	cis	SARS-CoV-2 infection
17343_6	SNPH	Syntaphilin	O15079	SNPH	chr20:1266280	Inverse variance weighted	2	0.082	0.056	1.383E-01	0.888	cis	SARS-CoV-2 infection
8402_22	CYTL1	Cytokine-like protein 1	Q9NRR1	CYTL1	chr4:5019458	Inverse variance weighted	4	-0.021	0.014	1.387E-01	0.888	cis	SARS-CoV-2 infection
9837_60	NAD(P)H dehydrogenase	NAD(P)H dehydrogenase [quinone] 1	P15559	NQO1	chr16:69726668	Inverse variance weighted	9	-0.014	0.010	1.388E-01	0.888	cis	SARS-CoV-2 infection
3322_52	LRIG3	Leucine-rich repeats and immunoglobulin-like domains protein 3	Q6UXM1	LRIG3	chr12:58920504	Inverse variance weighted	6	-0.020	0.014	1.397E-01	0.888	cis	SARS-CoV-2 infection
16773_29	SCUB3	Signal peptide, CUB and EGF-like domain-containing protein 3	Q8IX30	SCUBE3	chr6:35213956	Inverse variance weighted	3	-0.032	0.022	1.398E-01	0.888	cis	SARS-CoV-2 infection
13621_31	AP2A2	AP-2 complex subunit alpha-2	O94973	AP2A2	chr11:924881	Wald ratio	1	0.114	0.077	1.421E-01	0.888	cis	SARS-CoV-2 infection
9744_139	DNJA4	DnaJ homolog subfamily A member 4	Q8WW22	DNAJA4	chr15:78264086	Wald ratio	1	-0.104	0.071	1.423E-01	0.888	cis	SARS-CoV-2 infection
15471_29	LIPR2	Pancreatic lipase-related protein 2	P54317	PNLIPRP2	chr10:116620953	Inverse variance weighted	11	0.010	0.007	1.426E-01	0.888	cis	SARS-CoV-2 infection
5713_9	IFN-lambda 3	Interferon lambda-3	Q8IZI9	IFNL3	chr19:39245250	Wald ratio	1	-0.145	0.099	1.429E-01	0.888	cis	SARS-CoV-2 infection
7970_315	NAR3	Ecto-ADP-ribosyltransferase 3	Q13508	ART3	chr4:76011184	Inverse variance weighted	4	0.029	0.020	1.435E-01	0.888	cis	SARS-CoV-2 infection
3378_49	Kallikrein 7	Kallikrein-7	P49862	KLK7	chr19:50984099	Inverse variance weighted	2	0.048	0.033	1.438E-01	0.888	cis	SARS-CoV-2 infection
5256_86	PDE5A	cGMP-specific 3',5'-cyclic phosphodiesterase	O76074	PDE5A	chr4:119628804	Inverse variance weighted	3	-0.076	0.052	1.443E-01	0.888	cis	SARS-CoV-2 infection
17327_3	CNPY3	Protein canopy homolog 3	Q9BT09	CNPY3	chr6:42929480	Wald ratio	1	0.128	0.088	1.446E-01	0.888	cis	SARS-CoV-2 infection
8960_3	LRAP	Endoplasmic reticulum aminopeptidase 2	Q6P179	ERAP2	chr5:96875986	Inverse variance weighted	4	-0.015	0.011	1.446E-01	0.888	cis	SARS-CoV-2 infection
5036_50	TSG-6	Tumor necrosis factor-inducible gene 6 protein	P98066	TNFAIP6	chr2:151357592	Inverse variance weighted	9	-0.015	0.010	1.447E-01	0.888	cis	SARS-CoV-2 infection
17140_57	PDGFD	Platelet-derived growth factor D	Q9GZP0	PDGFD	chr11:104164379	Inverse variance weighted	6	0.051	0.035	1.455E-01	0.888	cis	SARS-CoV-2 infection
4968_50	CAPG	Macrophage-capping protein	P40121	CAPG	chr2:85418432	Inverse variance weighted	4	0.027	0.018	1.456E-01	0.888	cis	SARS-CoV-2 infection
12457_10	MTND	1,2-dihydroxy-3-keto-5-methylthiopentene dioxygenase	Q9BV57	ADI1	chr2:3519531	Wald ratio	1	-0.091	0.062	1.463E-01	0.888	cis	SARS-CoV-2 infection
5749_53	COL	Colipase	P04118	CLPS	chr6:35797344	Inverse variance weighted	10	-0.021	0.014	1.482E-01	0.888	cis	SARS-CoV-2 infection
18197_97	KCRS	Creatine kinase S-type, mitochondrial	P17540	CKMT2	chr5:81233320	Wald ratio	1	0.141	0.097	1.484E-01	0.888	cis	SARS-CoV-2 infection
2968_61	TNFSF15	Tumor necrosis factor ligand superfamily member 15	O95150	TNFSF15	chr9:114806039	Wald ratio	1	-0.131	0.091	1.498E-01	0.888	cis	SARS-CoV-2 infection
7228_2	SIA7F	Alpha-N-acetylgalactosaminide alpha-2,6-sialyltransferase 6	Q969X2	ST6GALNAC6	chr9:127905408	Inverse variance weighted	2	-0.083	0.058	1.504E-01	0.888	cis	SARS-CoV-2 infection
18173_11	ARK73	Aflatoxin B1 aldehyde reductase member 3	O95154	AKR7A3	chr1:19288770	Inverse variance weighted	5	0.032	0.022	1.504E-01	0.888	cis	SARS-CoV-2 infection
4920_10	Lysozyme	Lysozyme C	P61626	LYZ	chr12:69348381	Inverse variance weighted	6	-0.017	0.012	1.506E-01	0.888	cis	SARS-CoV-2 infection
15449_33	TIM-4	T-cell immunoglobulin and mucin domain-containing protein 4	Q96H15	TIMD4	chr5:156963226	Inverse variance weighted	5	-0.049	0.034	1.516E-01	0.888	cis	SARS-CoV-2 infection
3508_78	MDC	C-C motif chemokine 22	O00626	CCL22	chr16:57358783	Inverse variance weighted	5	-0.038	0.027	1.522E-01	0.888	cis	SARS-CoV-2 infection
18235_16	PGP	Glycerol-3-phosphate phosphatase	A6NDG6	PGP	chr16:2214840	Inverse variance weighted	3	0.042	0.030	1.522E-01	0.888	cis	SARS-CoV-2 infection
5107_7	Notch 1	Neurogenic locus notch homolog protein 1	P46531	NOTCH1	chr9:136546048	Inverse variance weighted	2	0.059	0.041	1.533E-01	0.888	cis	SARS-CoV-2 infection
3066_12	Galectin-3	Galectin-3	P17931	LGALS3	chr14:55124110	Inverse variance weighted	4	-0.025	0.018	1.535E-01	0.888	cis	SARS-CoV-2 infection
18832_65	SAA2	Serum amyloid A-2 protein	P0DJI9	SAA2	chr11:18248668	Wald ratio	1	-0.143	0.100	1.535E-01	0.888	cis	SARS-CoV-2 infection
18340_2	Proteasome beta chain	Proteasome subunit beta type-4	P28070	PSMB4	chr1:151399560	Inverse variance weighted	4	-0.014	0.010	1.538E-01	0.888	cis	SARS-CoV-2 infection
9275_2	Siglec-5	Sialic acid-binding Ig-like lectin 5	O15389	SIGLEC5	chr19:51630401	Wald ratio	1	-0.134	0.094	1.544E-01	0.888	cis	SARS-CoV-2 infection
7955_195	ITI heavy chain H1	Inter-alpha-trypsin inhibitor heavy chain H1	P19827	ITIH1	chr3:52777595	Inverse variance weighted	7	0.042	0.030	1.544E-01	0.888	cis	SARS-CoV-2 infection
8024_64	TPST2	Protein-tyrosine sulfotransferase 2	O60704	TPST2	chr22:26596717	Inverse variance weighted	2	0.043	0.030	1.552E-01	0.888	cis	SARS-CoV-2 infection
12820_1	GRAP	GRB2-related adapter protein	Q13588	GRAP	chr17:19047011	Wald ratio	1	0.181	0.127	1.553E-01	0.888	cis	SARS-CoV-2 infection
18241_18	HEM6	Oxygen-dependent coproporphyrinogen-III oxidase, mitochondrial	P36551	CPOX	chr3:98593648	Inverse variance weighted	4	-0.038	0.027	1.567E-01	0.888	cis	SARS-CoV-2 infection
3195_50	Granulysin	Granulysin	P22749	GNLY	chr2:85685175	Inverse variance weighted	7	-0.020	0.014	1.572E-01	0.888	cis	SARS-CoV-2 infection
9263_57	SIAE	Sialate O-acetylesterase	Q9HAT2	SIAE	chr11:124695707	Wald ratio	1	0.072	0.051	1.576E-01	0.888	cis	SARS-CoV-2 infection
2979_8	ENA-78	C-X-C motif chemokine 5	P42830	CXCL5	chr4:73998677	Wald ratio	1	0.181	0.128	1.583E-01	0.888	cis	SARS-CoV-2 infection
9244_27	PPT1	Palmitoyl-protein thioesterase 1	P50897	PPT1	chr1:40097260	Inverse variance weighted	2	0.031	0.022	1.591E-01	0.888	cis	SARS-CoV-2 infection

16792_4	Siglec-5	Sialic acid-binding Ig-like lectin 5	O15389	SIGLEC5	chr19:51630401	Inverse variance weighted	5	-0.020	0.014	1.606E-01	0.888	cis	SARS-CoV-2 infection
15516_12	SAA-4	Serum amyloid A-4 protein	P35542	SAA4	chr11:18236802	Inverse variance weighted	7	0.011	0.008	1.612E-01	0.888	cis	SARS-CoV-2 infection
12475_48	CLIC5	Chloride intracellular channel protein 5	Q9NZA1	CLIC5	chr6:46080348	Inverse variance weighted	5	0.031	0.022	1.617E-01	0.888	cis	SARS-CoV-2 infection
4982_54	Elafin	Elafin	P19957	PI3	chr20:45174902	Inverse variance weighted	5	0.037	0.026	1.619E-01	0.888	cis	SARS-CoV-2 infection
12940_35	AL3B1	Aldehyde dehydrogenase family 3 member B1	P43353	ALDH3B1	chr11:68008578	Wald ratio	1	0.057	0.041	1.619E-01	0.888	cis	SARS-CoV-2 infection
12657_2	FCL	GDP-L-fucose synthase	Q13630	GFUS	chr8:143618048	Wald ratio	1	-0.157	0.112	1.620E-01	0.888	cis	SARS-CoV-2 infection
18382_109	Catechol O-methyltransferase	Catechol O-methyltransferase	P21964	COMT	chr22:19941371	Wald ratio	1	-0.078	0.056	1.621E-01	0.888	cis	SARS-CoV-2 infection
3232_28	TrATPase	Tartrate-resistant acid phosphatase type 5	P13686	ACP5	chr19:11579993	Inverse variance weighted	4	0.028	0.020	1.625E-01	0.888	cis	SARS-CoV-2 infection
2977_7	EDAR	Tumor necrosis factor receptor superfamily member EDAR	Q9UNE0	EDAR	chr2:108989372	Inverse variance weighted	7	-0.015	0.011	1.632E-01	0.888	cis	SARS-CoV-2 infection
4125_52	sRAGE	Advanced glycosylation end product-specific receptor, soluble	Q15109	AGER	chr6:32184322	Inverse variance weighted	2	0.029	0.021	1.637E-01	0.888	cis	SARS-CoV-2 infection
19334_62	TXD12	Thioredoxin domain-containing protein 12	O95881	TXNDC12	chr1:52055191	Wald ratio	1	0.081	0.058	1.644E-01	0.888	cis	SARS-CoV-2 infection
13044_5	TS101	Tumor susceptibility gene 101 protein	Q99816	TSG101	chr11:18526951	Inverse variance weighted	2	-0.072	0.052	1.647E-01	0.888	cis	SARS-CoV-2 infection
13125_45	Vitronectin	Vitronectin	P04004	VTN	chr17:28373091	Inverse variance weighted	9	0.013	0.009	1.652E-01	0.888	cis	SARS-CoV-2 infection
5487_7	SLAF7	SLAM family member 7	Q9NQ25	SLAMF7	chr1:160379057	Inverse variance weighted	7	-0.015	0.011	1.656E-01	0.888	cis	SARS-CoV-2 infection
17231_1	L-plastin	Plastin-2	P13796	LCP1	chr13:46211871	Inverse variance weighted	4	0.038	0.028	1.667E-01	0.888	cis	SARS-CoV-2 infection
5005_4	MK12	Mitogen-activated protein kinase 12	P53778	MAPK12	chr22:50261716	Wald ratio	1	-0.104	0.075	1.671E-01	0.888	cis	SARS-CoV-2 infection
18175_65	VAT1	Synaptic vesicle membrane protein VAT-1 homolog	Q99536	VAT1	chr17:43025123	Wald ratio	1	-0.209	0.151	1.675E-01	0.888	cis	SARS-CoV-2 infection
17370_186	ABHDA	Mycophenolic acid acyl-glucuronide esterase, mitochondrial	Q9NUJ1	ABHD10	chr3:111979010	Wald ratio	1	-0.132	0.096	1.676E-01	0.888	cis	SARS-CoV-2 infection
13934_3	MCF2L	Guanine nucleotide exchange factor DBS	O15068	MCF2L	chr13:112894378	Wald ratio	1	-0.032	0.023	1.682E-01	0.888	cis	SARS-CoV-2 infection
11656_110	EVL	Ena/VASP-like protein	Q9UI08	EVL	chr14:99971449	Inverse variance weighted	4	0.055	0.040	1.684E-01	0.888	cis	SARS-CoV-2 infection
4430_44	Collectin Kidney 1	Collectin-11	Q9BWP8	COLEC11	chr2:3594832	Inverse variance weighted	10	0.013	0.009	1.708E-01	0.888	cis	SARS-CoV-2 infection
11308_8	CR3L4	Cyclic AMP-responsive element-binding protein 3-like protein 4	Q8TEY5	CREB3L4	chr1:153967534	Inverse variance weighted	2	0.040	0.029	1.711E-01	0.888	cis	SARS-CoV-2 infection
9557_5	MANS1	MANSC domain-containing protein 1	Q9H8J5	MANSC1	chr12:12350242	Inverse variance weighted	3	0.036	0.026	1.717E-01	0.888	cis	SARS-CoV-2 infection
5825_49	IFN-g R1	Interferon gamma receptor 1	P15260	IFNGR1	chr6:137219449	Inverse variance weighted	3	-0.070	0.051	1.719E-01	0.888	cis	SARS-CoV-2 infection
9266_1	sTREM-1	Triggering receptor expressed on myeloid cells 1	Q9NP99	TREM1	chr6:41286682	Inverse variance weighted	6	0.030	0.022	1.721E-01	0.888	cis	SARS-CoV-2 infection
13563_259	ACBD7	Acyl-CoA-binding domain-containing protein 7	Q8N6N7	ACBD7	chr10:15088776	Wald ratio	1	0.174	0.127	1.721E-01	0.888	cis	SARS-CoV-2 infection
9002_36	SPA11	Serpin A11	Q86U17	SERPINA11	chr14:94452800	Inverse variance weighted	5	-0.038	0.028	1.725E-01	0.888	cis	SARS-CoV-2 infection
5651_50	PSG7	Putative pregnancy-specific beta-1-glycoprotein 7	Q13046	PSG7	chr19:42937207	Wald ratio	1	0.107	0.078	1.726E-01	0.888	cis	SARS-CoV-2 infection
8664_36	PKDCC	Extracellular tyrosine-protein kinase PKDCC	Q504Y2	PKDCC	chr2:42048021	Inverse variance weighted	2	-0.174	0.128	1.732E-01	0.888	cis	SARS-CoV-2 infection
12347_29	CCM2	Cerebral cavernous malformations 2 protein	Q9BSQ5	CCM2	chr7:44999475	Wald ratio	1	-0.106	0.078	1.733E-01	0.888	cis	SARS-CoV-2 infection
5462_62	Ficolin-3	Ficolin-3	O75636	FCN3	chr1:27374824	Inverse variance weighted	7	-0.015	0.011	1.738E-01	0.888	cis	SARS-CoV-2 infection
3415_61	BSP	Bone sialoprotein 2	P21815	IBSP	chr4:87799554	Wald ratio	1	-0.065	0.048	1.749E-01	0.888	cis	SARS-CoV-2 infection
14100_63	C1QC	Complement C1q subcomponent subunit C	P02747	C1QC	chr1:22643014	Inverse variance weighted	9	0.022	0.016	1.753E-01	0.888	cis	SARS-CoV-2 infection
5078_82	EphB6	Ephrin type-B receptor 6	O15197	EPHB6	chr7:142855061	Inverse variance weighted	3	-0.031	0.023	1.754E-01	0.888	cis	SARS-CoV-2 infection
18398_1	AK1D1	3-oxo-5-beta-steroid 4-dehydrogenase	P51857	AKR1D1	chr7:138002324	Wald ratio	1	0.136	0.100	1.755E-01	0.888	cis	SARS-CoV-2 infection
2611_72	Dtk	Tyrosine-protein kinase receptor TYRO3	Q06418	TYRO3	chr15:41557675	Wald ratio	1	0.066	0.048	1.758E-01	0.888	cis	SARS-CoV-2 infection
11192_168	TINAL	Tubulointerstitial nephritis antigen-like	Q9GZM7	TINAGL1	chr1:31576485	Wald ratio	1	-0.067	0.050	1.761E-01	0.888	cis	SARS-CoV-2 infection
8289_8	GPNMB:ECD	Transmembrane glycoprotein NMB:Extracellular domain	Q14956	GPNMB	chr7:23235967	Inverse variance weighted	5	0.026	0.019	1.762E-01	0.888	cis	SARS-CoV-2 infection
18884_22	DNJB4	DnaJ homolog subfamily B member 4	Q9UDY4	DNAJB4	chr1:77979175	Inverse variance weighted	3	0.038	0.028	1.769E-01	0.888	cis	SARS-CoV-2 infection
9713_67	PGFRL	Platelet-derived growth factor receptor-like protein	Q15198	PDGFRL	chr8:17576433	Inverse variance weighted	6	-0.018	0.013	1.776E-01	0.888	cis	SARS-CoV-2 infection
17786_5	GGPPS	Geranylgeranyl pyrophosphate synthase	O95749	GGPS1	chr1:235327350	Wald ratio	1	0.074	0.055	1.781E-01	0.888	cis	SARS-CoV-2 infection
13669_6	FGFR-3:ECD	Fibroblast growth factor receptor 3:Extracellular domain	P22607	FGFR3	chr4:1793293	Inverse variance weighted	4	-0.058	0.043	1.793E-01	0.888	cis	SARS-CoV-2 infection
14151_4	UCRP	Ubiquitin-like protein ISG15	P05161	ISG15	chr1:1001138	Inverse variance weighted	2	0.020	0.015	1.794E-01	0.888	cis	SARS-CoV-2 infection
13423_94	CJ058	Redox-regulatory protein FAM213A	Q9BRX8	PRXL2A	chr10:80407829	Wald ratio	1	0.135	0.101	1.814E-01	0.888	cis	SARS-CoV-2 infection
12422_143	LX15B	Arachidonate 15-lipoxygenase B	O15296	ALOX15B	chr17:8039034	Wald ratio	1	0.088	0.066	1.822E-01	0.888	cis	SARS-CoV-2 infection
12381_26	CBR1	Carbonyl reductase [NADPH] 1	P16152	CBR1	chr21:36069941	Inverse variance weighted	5	0.022	0.016	1.826E-01	0.888	cis	SARS-CoV-2 infection
19213_1	ISK4	Serine protease inhibitor Kazal-type 4	O60575	SPINK4	chr9:33218365	Wald ratio	1	-0.031	0.023	1.826E-01	0.888	cis	SARS-CoV-2 infection
5727_35	B3GLT	Beta-1,3-glucosyltransferase	Q6Y288	B3GLCT	chr13:31199975	Inverse variance weighted	2	0.031	0.023	1.833E-01	0.888	cis	SARS-CoV-2 infection
3622_33	LGMN	Legumain	Q99538	LGMN	chr14:92748679	Inverse variance weighted	3	0.035	0.026	1.833E-01	0.888	cis	SARS-CoV-2 infection
12575_30	C1TC	C-1-tetrahydrofolate synthase, cytoplasmic	P11586	MTHFD1	chr14:64388031	Inverse variance weighted	2	0.071	0.053	1.835E-01	0.888	cis	SARS-CoV-2 infection
6227_1	kallikrein 10	Kallikrein-10	O43240	KLK10	chr19:51020175	Inverse variance weighted	6	0.038	0.029	1.836E-01	0.888	cis	SARS-CoV-2 infection
12685_57	HOME2	Homer protein homolog 2	Q9NSB8	HOMER2	chr15:82986153	Wald ratio	1	0.093	0.070	1.839E-01	0.888	cis	SARS-CoV-2 infection
3803_10	CYTD	Cystatin-D	P28325	CST5	chr20:23879748	Inverse variance weighted	11	0.025	0.019	1.841E-01	0.888	cis	SARS-CoV-2 infection
4158_54	uPA	Urokinase-type plasminogen activator	P00749	PLAU	chr10:73909177	Inverse variance weighted	4	-0.022	0.016	1.847E-01	0.888	cis	SARS-CoV-2 infection
15620_4	NLGN1	Neuroligin-1	Q8N2Q7	NLGN1	chr3:173396284	Inverse variance weighted	3	0.048	0.036	1.849E-01	0.888	cis	SARS-CoV-2 infection
3316_58	Heparin cofactor II	Heparin cofactor 2	P05546	SERPIND1	chr22:20774113	Wald ratio	1	0.136	0.103	1.853E-01	0.888	cis	SARS-CoV-2 infection
9772_153	NLGN2:ECD	Neuroligin-2:Extracellular domain	Q8NFZ4	NLGN2	chr17:7404874	Inverse variance weighted	2	0.071	0.054	1.858E-01	0.888	cis	SARS-CoV-2 infection
2831_29	Kallikrein 11	Kallikrein-11	Q9UBX7	KLK11	chr19:51028039	Inverse variance weighted	7	0.032	0.024	1.859E-01	0.888	cis	SARS-CoV-2 infection
16070_7	WIF-1	Wnt inhibitory factor 1	Q9Y5W5	WIF1	chr12:65121305	Wald ratio	1	-0.105	0.079	1.867E-01	0.890	cis	SARS-CoV-2 infection
9249_17	TMEM9:ECD	Transmembrane protein 9:Extracellular domain	Q9P0T7	TMEM9	chr1:201171574	Wald ratio	1	-0.096	0.073	1.889E-01	0.890	cis	SARS-CoV-2 infection
9173_21	PGM1	Phosphoglucomutase-1	P36871	PGM1	chr1:63593411	Inverse variance weighted	2	0.059	0.045	1.892E-01	0.890	cis	SARS-CoV-2 infection
11510_31	Apo L1	Apolipoprotein L1	O14791	APOL1	chr22:36253071	Inverse variance weighted	5	-0.017	0.013	1.894E-01	0.890	cis	SARS-CoV-2 infection
6923_1	PLOD2	Procollagen-lysine,2-oxoglutarate 5-dioxygenase 2	O00469	PLOD2	chr3:146163725	Inverse variance weighted	2	0.066	0.051	1.900E-01	0.890	cis	SARS-CoV-2 infection
4535_50	BST1	ADP-ribosyl cyclase/cyclic ADP-ribose hydrolase 2	Q10588	BST1	chr4:15703065	Inverse variance weighted	11	-0.008	0.006	1.901E-01	0.890	cis	SARS-CoV-2 infection
8014_359	MANEA	Glycoprotein endo-alpha-1,2-mannosidase	Q5SRI9	MANEA	chr6:95577485	Inverse variance weighted	6	-0.010	0.007	1.904E-01	0.890	cis	SARS-CoV-2 infection
3376_49	IL-17 RD	Interleukin-17 receptor D	Q8NFM7	IL17RD	chr3:57170306	Inverse variance weighted	4	-0.017	0.013	1.904E-01	0.890	cis	SARS-CoV-2 infection
12424_107	THYN1	Thymocyte nuclear protein 1	Q9P016	THYN1	chr11:134253370	Wald ratio	1	0.126	0.097	1.911E-01	0.890	cis	SARS-CoV-2 infection
8275_31	PEAR1:ECD	Platelet endothelial aggregation receptor 1:Extracellular domain	Q5VY43	PEAR1	chr1:156893698	Inverse variance weighted	4	-0.030	0.023	1.917E-01	0.891	cis	SARS-CoV-2 infection
15391_114	GAS-6	Growth arrest-specific protein 6	Q14393	GAS6	chr13:113864076	Inverse variance weighted	4	-0.023	0.018	1.935E-01	0.892	cis	SARS-CoV-2 infection
14079_14	IL-18 Ra	Interleukin-18 receptor 1	Q13478	IL18R1	chr2:102311529	Inverse variance weighted	5	-0.024	0.019	1.936E-01	0.892	cis	SARS-CoV-2 infection
10903_50	STX8	Syntaxin-8	Q9UNK0	STX8	chr17:9576591	Wald ratio	1	-0.101	0.078	1.948E-01	0.892	cis	SARS-CoV-2 infection
7083_74	MATN4	Matrilin-4	O95460	MATN4	chr20:45308529	Inverse variance weighted	3	0.036	0.027	1.950E-01	0.892	cis	SARS-CoV-2 infection
9580_5	Laminin gamma-2	Laminin subunit gamma-2	Q13753	LAMC2	chr1:183186238	Inverse variance weighted	5	-0.018	0.014	1.952E-01	0.892	cis	SARS-CoV-2 infection
3905_62	UBE2N	Ubiquitin-conjugating enzyme E2 N	P61088	UBE2N	chr12:93441947	Wald ratio	1	-0.131	0.101	1.955E-01	0.892	cis	SARS-CoV-2 infection
4913_78	HCC-4	C-C motif chemokine 16	O15467	CCL16	chr17:35981497	Inverse variance weighted	8	-0.014	0.011	1.962E-01	0.892	cis	SARS-CoV-2 infection
5000_52	LG3BP	Galectin-3-binding protein	Q08380	LGALS3BP	chr17:78979947	Inverse variance weighted	3	-0.048	0.037	1.963E-01	0.892	cis	SARS-CoV-2 infection
15583_18	FCRLB	Fc receptor-like B	Q6BAA4	FCRLB	chr1:161721544	Inverse variance weighted	5	0.033	0.026	1.971E-01	0.893	cis	SARS-CoV-2 infection
11241_8	ARLY	Argininosuccinate lyase	P04424	ASL	chr7:66075800	Inverse variance weighted	3	-0.044	0.034	1.982E-01	0.896	cis	SARS-CoV-2 infection

6551_94	Vaspin	Serpin A12	Q8IW75	SERPINA12	chr14:94517844	Inverse variance weighted	5	-0.017	0.013	1.997E-01	0.899	cis	SARS-CoV-2 infection
13242_134	SHAN3	SH3 and multiple ankyrin repeat domains protein 3	Q9BYB0	SHANK3	chr22:50674415	Inverse variance weighted	5	-0.020	0.015	2.000E-01	0.899	cis	SARS-CoV-2 infection
14618_26	ECOP	Vesicular, overexpressed in cancer, prosurvival protein 1	Q96AW1	VOPP1	chr7:55572988	Inverse variance weighted	2	-0.056	0.044	2.011E-01	0.900	cis	SARS-CoV-2 infection
5615_62	F172A	Protein FAM172A	Q8WUF8	FAM172A	chr5:94111699	Wald ratio	1	-0.093	0.073	2.013E-01	0.900	cis	SARS-CoV-2 infection
13552_7	SWP70	Switch-associated protein 70	Q9UH65	SWAP70	chr11:9664077	Inverse variance weighted	5	-0.014	0.011	2.025E-01	0.901	cis	SARS-CoV-2 infection
13405_61	ISK2	Serine protease inhibitor Kazal-type 2	P20155	SPINK2	chr4:56821742	Inverse variance weighted	5	-0.015	0.012	2.026E-01	0.901	cis	SARS-CoV-2 infection
15385_116	FABP2	Fatty acid-binding protein, intestinal	P12104	FABP2	chr4:119322138	Inverse variance weighted	5	0.017	0.013	2.032E-01	0.901	cis	SARS-CoV-2 infection
17329_2	BDH2	3-hydroxybutyrate dehydrogenase type 2	Q9BUT1	BDH2	chr4:103099870	Inverse variance weighted	2	0.036	0.029	2.036E-01	0.901	cis	SARS-CoV-2 infection
5660_51	SOD3	Extracellular superoxide dismutase [Cu-Zn]	P08294	SOD3	chr4:24789912	Inverse variance weighted	10	-0.014	0.011	2.052E-01	0.905	cis	SARS-CoV-2 infection
17724_3	WWOX	WW domain-containing oxidoreductase	Q9NZC7	WWOX	chr16:78099400	Wald ratio	1	-0.084	0.067	2.060E-01	0.905	cis	SARS-CoV-2 infection
8356_88	NEU1	Oxytocin-neurophysin 1	P01178	OXT	chr20:3071620	Inverse variance weighted	3	0.029	0.023	2.067E-01	0.905	cis	SARS-CoV-2 infection
18841_1	SPB13	Serpin B13	Q9UIV8	SERPINB13	chr18:63586989	Inverse variance weighted	4	0.031	0.025	2.072E-01	0.905	cis	SARS-CoV-2 infection
2992_59	IL-17 sR	Interleukin-17 receptor A	Q96F46	IL17RA	chr22:17084954	Inverse variance weighted	8	-0.007	0.006	2.078E-01	0.905	cis	SARS-CoV-2 infection
3853_56	MDHC	Malate dehydrogenase, cytoplasmic	P40925	MDH1	chr2:63588609	Wald ratio	1	-0.086	0.068	2.084E-01	0.905	cis	SARS-CoV-2 infection
12494_99	GBRL2	Gamma-aminobutyric acid receptor-associated protein-like 2	P60520	GABARAPL2	chr16:75566375	Wald ratio	1	-0.078	0.062	2.098E-01	0.905	cis	SARS-CoV-2 infection
14012_17	LGP2	Probable ATP-dependent RNA helicase DHX58	Q96C10	DHX58	chr17:42112714	Inverse variance weighted	2	-0.055	0.044	2.101E-01	0.905	cis	SARS-CoV-2 infection
8992_1	TMEM2	Transmembrane protein 2	Q9UHN6	CEMIP2	chr9:71816690	Inverse variance weighted	2	-0.038	0.030	2.106E-01	0.905	cis	SARS-CoV-2 infection
15495_9	FOLR3	Folate receptor gamma	P41439	FOLR3	chr11:72114869	Inverse variance weighted	9	0.016	0.013	2.113E-01	0.905	cis	SARS-CoV-2 infection
13416_8	T132D	Transmembrane protein 132D	Q14C87	TMEM132D	chr12:129904025	Inverse variance weighted	6	0.021	0.017	2.126E-01	0.905	cis	SARS-CoV-2 infection
14067_6	PKP2	Plakophilin-2	Q99959	PKP2	chr12:32896777	Inverse variance weighted	2	0.071	0.057	2.132E-01	0.905	cis	SARS-CoV-2 infection
11659_31	EPN4	Clathrin interactor 1	Q14677	CLINT1	chr5:157859145	Wald ratio	1	-0.115	0.093	2.146E-01	0.905	cis	SARS-CoV-2 infection
16900_29	MDGA1	MAM domain-containing glycosylphosphatidylinositol anchor protein 1	Q8NFP4	MDGA1	chr6:37699306	Inverse variance weighted	8	-0.010	0.008	2.149E-01	0.905	cis	SARS-CoV-2 infection
5632_6	CRAC1	Cartilage acidic protein 1	Q9NQ79	CRTAC1	chr10:98030828	Inverse variance weighted	9	-0.015	0.012	2.149E-01	0.905	cis	SARS-CoV-2 infection
2999_6	LSAMP	Limbic system-associated membrane protein	Q13449	LSAMP	chr3:117139389	Inverse variance weighted	4	-0.036	0.029	2.150E-01	0.905	cis	SARS-CoV-2 infection
5618_50	FAM3B	Protein FAM3B	P58499	FAM3B	chr21:41304212	Wald ratio	1	0.082	0.066	2.153E-01	0.905	cis	SARS-CoV-2 infection
2860_19	Karyopherin-a2	Importin subunit alpha-1	P52292	KPNA2	chr17:68035636	Wald ratio	1	-0.114	0.092	2.155E-01	0.905	cis	SARS-CoV-2 infection
16809_1	NDKM	Nucleoside diphosphate kinase, mitochondrial	O00746	NME4	chr16:396725	Inverse variance weighted	2	0.078	0.063	2.155E-01	0.905	cis	SARS-CoV-2 infection
16318_12	ALK-1	Serine/threonine-protein kinase receptor R3	P37023	ACVRL1	chr12:51906908	Inverse variance weighted	3	-0.030	0.024	2.163E-01	0.905	cis	SARS-CoV-2 infection
9409_11	TRYB1	Tryptase beta-1	Q15661	TPSAB1	chr16:1240379	Inverse variance weighted	8	0.013	0.011	2.177E-01	0.905	cis	SARS-CoV-2 infection
13969_24	IMA7	Importin subunit alpha-7	O60684	KPNA6	chr1:32108056	Wald ratio	1	0.099	0.080	2.181E-01	0.905	cis	SARS-CoV-2 infection
17341_89	THIC	Acetyl-CoA acetyltransferase, cytosolic	Q9BWD1	ACAT2	chr6:159762045	Inverse variance weighted	3	0.021	0.017	2.185E-01	0.905	cis	SARS-CoV-2 infection
12643_4	ARRB1	Beta-arrestin-1	P49407	ARRB1	chr11:75351705	Wald ratio	1	-0.078	0.064	2.206E-01	0.905	cis	SARS-CoV-2 infection
4834_61	Epithelial cell kinase	Ephrin type-A receptor 2	P29317	EPHA2	chr1:16156069	Inverse variance weighted	3	-0.051	0.042	2.221E-01	0.905	cis	SARS-CoV-2 infection
9256_78	NPTX1	Neuronal pentraxin-1	Q15818	NPTX1	chr17:80477843	Inverse variance weighted	6	-0.019	0.016	2.223E-01	0.905	cis	SARS-CoV-2 infection
4126_22	BPI	Bactericidal permeability-increasing protein	P17213	BPI	chr20:38304150	Inverse variance weighted	9	0.009	0.008	2.224E-01	0.905	cis	SARS-CoV-2 infection
8364_74	UST	Uronyl 2-sulfotransferase	Q9Y2C2	UST	chr6:148747030	Inverse variance weighted	3	0.043	0.036	2.235E-01	0.905	cis	SARS-CoV-2 infection
14158_17	Annexin V	Annexin A5	P08758	ANXA5	chr4:121696995	Inverse variance weighted	2	0.034	0.028	2.236E-01	0.905	cis	SARS-CoV-2 infection
15440_57	NEC2	Neuroendocrine convertase 2	P16519	PCSK2	chr20:17226107	Wald ratio	1	-0.069	0.057	2.244E-01	0.905	cis	SARS-CoV-2 infection
12813_18	EHBP1	EH domain-binding protein 1	Q8NDI1	EHBP1	chr2:62673851	Wald ratio	1	0.122	0.100	2.246E-01	0.905	cis	SARS-CoV-2 infection
13940_19	IP16:HIN 1	Gamma-interferon-inducible protein 16:Isoform 2, Hematopoietic expression, interferon-inducible nature, and nuclear localization 1	Q16666	IFI16	chr1:158999968	Wald ratio	1	0.088	0.073	2.248E-01	0.905	cis	SARS-CoV-2 infection
18213_30	UBE2F	NEDD8-conjugating enzyme UBE2F	Q969M7	UBE2F	chr2:237966827	Wald ratio	1	-0.085	0.070	2.248E-01	0.905	cis	SARS-CoV-2 infection
13930_3	ABC3G	DNA dC->dU-editing enzyme APOBEC-3G	Q9HC16	APOBEC3G	chr22:39077067	Inverse variance weighted	2	0.056	0.046	2.255E-01	0.905	cis	SARS-CoV-2 infection
3810_50	FGR	Tyrosine-protein kinase Fgr	P09769	FGR	chr1:27635185	Inverse variance weighted	2	0.044	0.037	2.279E-01	0.905	cis	SARS-CoV-2 infection
3132_1	VEGF-C	Vascular endothelial growth factor C	P49767	VEGFC	chr4:176792922	Inverse variance weighted	2	-0.056	0.047	2.280E-01	0.905	cis	SARS-CoV-2 infection
18831_6	LRIG1	Leucine-rich repeats and immunoglobulin-like domains protein 1	Q96JA1	LRIG1	chr3:66501263	Inverse variance weighted	8	-0.016	0.013	2.289E-01	0.905	cis	SARS-CoV-2 infection
8043_153	COMP	Cartilage oligomeric matrix protein	P49747	COMP	chr19:18791305	Inverse variance weighted	4	-0.031	0.026	2.292E-01	0.905	cis	SARS-CoV-2 infection
15614_168	LIRA2	Leukocyte immunoglobulin-like receptor subfamily A member 2	Q8N149	LILRA2	chr19:54572920	Inverse variance weighted	4	-0.021	0.017	2.313E-01	0.905	cis	SARS-CoV-2 infection
5116_62	ROBO2	Roundabout homolog 2	Q9HCK4	ROBO2	chr3:75906695	Wald ratio	1	-0.136	0.114	2.314E-01	0.905	cis	SARS-CoV-2 infection
9241_40	SIRPG	Signal-regulatory protein gamma	Q9P1W8	SIRPG	chr20:1657779	Wald ratio	1	-0.069	0.057	2.318E-01	0.905	cis	SARS-CoV-2 infection
17513_11	ANX11	Annexin A11	P50995	ANXA11	chr10:80205572	Inverse variance weighted	3	0.054	0.045	2.319E-01	0.905	cis	SARS-CoV-2 infection
3028_36	Ck-b-8-1	Ck-beta-8-1	P55773	CCL23	chr17:36017972	Inverse variance weighted	4	-0.055	0.046	2.321E-01	0.905	cis	SARS-CoV-2 infection
14123_34	GI24:ECD	V-type immunoglobulin domain-containing suppressor of T-cell activation:Extracellular domain	Q9H7M9	VSIR	chr10:71773520	Inverse variance weighted	5	0.046	0.039	2.329E-01	0.905	cis	SARS-CoV-2 infection
19176_27	FA49B	Protein FAM49B	Q9NUQ9	CYRIB	chr8:130017504	Wald ratio	1	0.117	0.098	2.329E-01	0.905	cis	SARS-CoV-2 infection
8687_26	T106B	Transmembrane protein 106B	Q9NUM4	TMEM106B	chr7:12211270	Inverse variance weighted	2	0.042	0.035	2.331E-01	0.905	cis	SARS-CoV-2 infection
11177_16	K2C5	Keratin, type II cytoskeletal 5	P13647	KRT5	chr12:52520530	Inverse variance weighted	2	0.064	0.054	2.333E-01	0.905	cis	SARS-CoV-2 infection
3216_2	PIGR	Polymeric immunoglobulin receptor	P01833	PIGR	chr1:206946466	Wald ratio	1	0.085	0.071	2.351E-01	0.905	cis	SARS-CoV-2 infection
13132_14	SEM5A	Semaphorin-5A	Q13591	SEMA5A	chr5:9546075	Inverse variance weighted	10	-0.006	0.005	2.354E-01	0.905	cis	SARS-CoV-2 infection
8932_1	ENTP6	Ectonucleoside triphosphate diphosphohydrolase 6	O75354	ENTPD6	chr20:25195693	Wald ratio	1	0.060	0.050	2.355E-01	0.905	cis	SARS-CoV-2 infection
7916_10	S100A7	Protein S100-A7	P31151	S100A7	chr1:153460651	Inverse variance weighted	7	0.009	0.008	2.356E-01	0.905	cis	SARS-CoV-2 infection
16785_45	HD-5	Defensin-5	Q01523	DEFA5	chr8:7056739	Wald ratio	1	-0.089	0.075	2.360E-01	0.905	cis	SARS-CoV-2 infection
13090_17	S100A6	Protein S100-A6	P06703	S100A6	chr1:153536244	Inverse variance weighted	2	0.102	0.086	2.365E-01	0.905	cis	SARS-CoV-2 infection
11134_30	RF1ML	Peptide chain release factor 1-like, mitochondrial	Q9UGC7	MTRF1L	chr6:153002709	Wald ratio	1	-0.036	0.030	2.368E-01	0.905	cis	SARS-CoV-2 infection
15565_102	CA125	Mucin-16	Q8WXI7	MUC16	chr19:8981342	Inverse variance weighted	2	-0.081	0.068	2.368E-01	0.905	cis	SARS-CoV-2 infection
9357_4	CREG1	Protein CREG1	O75629	CREG1	chr1:167553805	Inverse variance weighted	2	0.031	0.026	2.374E-01	0.905	cis	SARS-CoV-2 infection
9183_7	IFN-a/b R1	Interferon alpha/beta receptor 1	P17181	IFNAR1	chr21:33324387	Inverse variance weighted	7	0.010	0.008	2.374E-01	0.905	cis	SARS-CoV-2 infection
4564_2	PLXC1	Plexin-C1	O60486	PLXNC1	chr12:94148577	Inverse variance weighted	11	0.008	0.007	2.376E-01	0.905	cis	SARS-CoV-2 infection
5227_60	PDK1	[Pyruvate dehydrogenase (acetyl-transferring)] kinase isozyme 1, mitochondrial	Q15118	PDK1	chr2:172555373	Inverse variance weighted	3	0.030	0.026	2.376E-01	0.905	cis	SARS-CoV-2 infection
13988_67	NMRL1	NmrA-like family domain-containing protein 1	Q9HBL8	NMRAL1	chr16:4495763	Inverse variance weighted	11	0.014	0.012	2.386E-01	0.905	cis	SARS-CoV-2 infection
9316_67	WFDC1	WAP four-disulfide core domain protein 1	Q9HC57	WFDC1	chr16:84294846	Inverse variance weighted	5	0.021	0.018	2.403E-01	0.905	cis	SARS-CoV-2 infection
4549_78	FUT5	Alpha-(1,3)-fucosyltransferase 5	Q11128	FUT5	chr19:5870540	Inverse variance weighted	7	-0.010	0.009	2.404E-01	0.905	cis	SARS-CoV-2 infection
6649_51	NET1	Netrin-1	O95631	NTN1	chr17:9021510	Inverse variance weighted	6	0.014	0.012	2.408E-01	0.905	cis	SARS-CoV-2 infection
3448_13	IR	Insulin receptor	P06213	INSR	chr19:7294414	Wald ratio	1	-0.079	0.067	2.411E-01	0.905	cis	SARS-CoV-2 infection
13133_73	LTBP4	Latent-transforming growth factor beta-binding protein 4	Q8N2S1	LTBP4	chr19:40592883	Inverse variance weighted	2	-0.084	0.071	2.411E-01	0.905	cis	SARS-CoV-2 infection
6899_37	PHS2	Pterin-4-alpha-carbinolamine dehydratase 2	Q9H0N5	PCBD2	chr5:134905120	Wald ratio	1	0.129	0.110	2.415E-01	0.905	cis	SARS-CoV-2 infection
12801_33	IRF2	Interferon regulatory factor 2	P14316	IRF2	chr4:184474558	Wald ratio	1	-0.116	0.099	2.430E-01	0.905	cis	SARS-CoV-2 infection
4131_72	Fibronectin	Fibronectin	P02751	FN1	chr2:215436073	Inverse variance weighted	2	-0.083	0.071	2.433E-01	0.905	cis	SARS-CoV-2 infection
17350_13	CHM2B	Charged multivesicular body protein 2b	Q9UQN3	CHMP2B	chr3:87227271	Inverse variance weighted	3	-0.024	0.021	2.439E-01	0.905	cis	SARS-CoV-2 infection
7049_2	ADAM 23	Disintegrin and metalloproteinase domain-containing protein 23	O75077	ADAM23	chr2:206443532	Inverse variance weighted	7	0.014	0.012	2.441E-01	0.905	cis	SARS-CoV-2 infection

5400_52	sLeptin R	Leptin receptor, soluble	P48357	LEPR	chr1:65420652	Inverse variance weighted	10	0.011	0.009	2.450E-01	0.905	cis	SARS-CoV-2 infection
4155_3	Tenascin	Tenascin	P24821	TNC	chr9:115118207	Inverse variance weighted	12	-0.011	0.009	2.453E-01	0.905	cis	SARS-CoV-2 infection
10512_13	IL3RB:ECD	Cytokine receptor common subunit beta:Extracellular domain	P32927	CSF2RB	chr22:36913628	Inverse variance weighted	7	-0.014	0.012	2.455E-01	0.905	cis	SARS-CoV-2 infection
10372_18	STAT6	Signal transducer and activator of transcription 6	P42226	STAT6	chr12:57132139	Inverse variance weighted	2	0.041	0.035	2.456E-01	0.905	cis	SARS-CoV-2 infection
13998_26	PURA1	Adenylosuccinate synthetase isozyme 1	Q8N142	ADSS1	chr14:104724229	Inverse variance weighted	3	0.029	0.025	2.457E-01	0.905	cis	SARS-CoV-2 infection
2737_22	NovH	Protein NOV homolog	P48745	CCN3	chr8:119416446	Wald ratio	1	-0.083	0.071	2.465E-01	0.905	cis	SARS-CoV-2 infection
12697_30	PI42A	Phosphatidylinositol 5-phosphate 4-kinase type-2 alpha	P48426	PIP4K2A	chr10:22714578	Wald ratio	1	0.049	0.042	2.468E-01	0.905	cis	SARS-CoV-2 infection
12401_3	STALP	AMSH-like protease	Q96FJ0	STAMBPL1	chr10:88879734	Wald ratio	1	0.059	0.051	2.472E-01	0.905	cis	SARS-CoV-2 infection
16770_3	REG1B	Lithostathine-1-beta	P48304	REG1B	chr2:79088019	Inverse variance weighted	3	0.116	0.100	2.473E-01	0.905	cis	SARS-CoV-2 infection
5604_30	HPSE	Heparanase	Q9Y251	HPSE	chr4:83335153	Inverse variance weighted	2	-0.037	0.032	2.477E-01	0.905	cis	SARS-CoV-2 infection
2742_68	Siglec-7	Sialic acid-binding Ig-like lectin 7	Q9Y286	SIGLEC7	chr19:51142299	Wald ratio	1	0.052	0.045	2.485E-01	0.905	cis	SARS-CoV-2 infection
15395_15	GST M1-1	Glutathione S-transferase Mu 1	P09488	GSTM1	chr1:109687814	Inverse variance weighted	6	0.036	0.031	2.491E-01	0.905	cis	SARS-CoV-2 infection
14107_1	MTHFS	5-formyltetrahydrofolate cyclo-ligase	P49914	MTHFS	chr15:79897379	Inverse variance weighted	6	0.016	0.014	2.495E-01	0.905	cis	SARS-CoV-2 infection
2677_1	ERBB1	Epidermal growth factor receptor	P00533	EGFR	chr7:55019017	Inverse variance weighted	3	0.039	0.034	2.526E-01	0.911	cis	SARS-CoV-2 infection
18922_27	CD68	Macrosialin	P34810	CD68	chr17:7579491	Inverse variance weighted	2	-0.100	0.087	2.530E-01	0.911	cis	SARS-CoV-2 infection
6151_18	MP2K3	Dual specificity mitogen-activated protein kinase kinase 3	P46734	MAP2K3	chr17:21284672	Wald ratio	1	0.119	0.104	2.536E-01	0.911	cis	SARS-CoV-2 infection
6620_82	LIGO1	Leucine-rich repeat and immunoglobulin-like domain-containing nogo receptor-interacting protein 1	Q96FE5	LINGO1	chr15:77820900	Inverse variance weighted	2	0.057	0.050	2.548E-01	0.911	cis	SARS-CoV-2 infection
10815_2	HABP4	Intracellular hyaluronan-binding protein 4	Q5JVS0	HABP4	chr9:96450169	Inverse variance weighted	3	-0.020	0.018	2.552E-01	0.911	cis	SARS-CoV-2 infection
15544_25	kallikrein 14	Kallikrein-14	Q9P0G3	KLK14	chr19:51084245	Inverse variance weighted	2	-0.078	0.069	2.556E-01	0.911	cis	SARS-CoV-2 infection
18387_7	suppression of tumorigenicity 13	Hsc70-interacting protein	P50502	ST13	chr22:40856639	Wald ratio	1	-0.102	0.089	2.559E-01	0.911	cis	SARS-CoV-2 infection
19223_6	RAB1A	Ras-related protein Rab-1A	P62820	RAB1A	chr2:65130331	Wald ratio	1	-0.098	0.086	2.563E-01	0.911	cis	SARS-CoV-2 infection
5238_26	PPIE	Peptidyl-prolyl cis-trans isomerase E	Q9UNP9	PPIE	chr1:39692182	Inverse variance weighted	2	0.019	0.017	2.565E-01	0.911	cis	SARS-CoV-2 infection
18819_21	PPIC	Peptidyl-prolyl cis-trans isomerase C	P45877	PPIC	chr5:123036725	Inverse variance weighted	3	0.015	0.013	2.571E-01	0.911	cis	SARS-CoV-2 infection
12459_13	PKHA1	Pleckstrin homology domain-containing family A member 1	Q9HB21	PLEKHA1	chr10:122374696	Inverse variance weighted	4	0.017	0.015	2.580E-01	0.911	cis	SARS-CoV-2 infection
13544_9	HMHA1	Rho GTPase-activating protein 45	Q92619	ARHGAP45	chr19:1065923	Wald ratio	1	0.042	0.038	2.583E-01	0.911	cis	SARS-CoV-2 infection
4496_60	MMP-12	Macrophage metalloelastase	P39900	MMP12	chr11:102874982	Inverse variance weighted	7	0.015	0.013	2.583E-01	0.911	cis	SARS-CoV-2 infection
18214_2	GSH0	Glutamate--cysteine ligase regulatory subunit	P48507	GCLM	chr1:93909456	Inverse variance weighted	2	0.050	0.045	2.596E-01	0.913	cis	SARS-CoV-2 infection
4763_31	Afamin	Afamin	P43652	AFM	chr4:73481745	Inverse variance weighted	3	0.042	0.037	2.615E-01	0.913	cis	SARS-CoV-2 infection
17712_7	IDI1	Isopentenyl-diphosphate Delta-isomerase 1	Q13907	IDI1	chr10:1049119	Wald ratio	1	-0.083	0.074	2.618E-01	0.913	cis	SARS-CoV-2 infection
12686_15	THTM	3-mercaptopyruvate sulfurtransferase	P25325	MPST	chr22:37019635	Inverse variance weighted	2	-0.057	0.051	2.620E-01	0.913	cis	SARS-CoV-2 infection
5076_53	EPHAA	Ephrin type-A receptor 10	Q5JZY3	EPHA10	chr1:37765133	Wald ratio	1	0.074	0.066	2.623E-01	0.913	cis	SARS-CoV-2 infection
11273_176	GSTT2	Glutathione S-transferase theta-2B	P0CG30	GSTT2B	chr22:23961195	Inverse variance weighted	4	0.033	0.029	2.633E-01	0.913	cis	SARS-CoV-2 infection
13536_56	POLI	DNA polymerase iota	Q9UNA4	POLI	chr18:54269517	Wald ratio	1	-0.060	0.054	2.646E-01	0.913	cis	SARS-CoV-2 infection
12594_5	GRAN	Grancalcin	P28676	GCA	chr2:162318840	Inverse variance weighted	2	0.018	0.016	2.651E-01	0.913	cis	SARS-CoV-2 infection
17490_4	SH3BGRL3-like protein	SH3 domain-binding glutamic acid-rich-like protein 3	Q9H299	SH3BGRL3	chr1:26280086	Inverse variance weighted	3	0.020	0.018	2.655E-01	0.913	cis	SARS-CoV-2 infection
4541_49	CDON	Cell adhesion molecule-related/down-regulated by oncogenes	Q4KMG0	CDON	chr11:126063335	Inverse variance weighted	4	0.014	0.013	2.657E-01	0.913	cis	SARS-CoV-2 infection
16919_1	ACBP	Acyl-CoA-binding protein	P07108	DBI	chr2:119366924	Inverse variance weighted	2	0.023	0.021	2.663E-01	0.913	cis	SARS-CoV-2 infection
15603_20	Integrin alpha-2	Integrin alpha-2	P17301	ITGA2	chr5:52989340	Wald ratio	1	0.058	0.052	2.667E-01	0.913	cis	SARS-CoV-2 infection
6471_53	FHR4	Complement factor H-related protein 4	Q92496	CFHR4	chr1:196888014	Inverse variance weighted	10	0.015	0.013	2.668E-01	0.913	cis	SARS-CoV-2 infection
7015_8	LIRB5	Leukocyte immunoglobulin-like receptor subfamily B member 5	O75023	LILRB5	chr19:54257301	Inverse variance weighted	6	0.007	0.006	2.680E-01	0.913	cis	SARS-CoV-2 infection
3206_4	LYVE1	Lymphatic vessel endothelial hyaluronic acid receptor 1	Q9Y5Y7	LYVE1	chr11:10611689	Inverse variance weighted	6	-0.025	0.023	2.680E-01	0.913	cis	SARS-CoV-2 infection
8231_122	VEGF sR1	Vascular endothelial growth factor receptor 1	P17948	FLT1	chr13:28495145	Wald ratio	1	0.103	0.093	2.684E-01	0.913	cis	SARS-CoV-2 infection
8304_50	OPG	Tumor necrosis factor receptor superfamily member 11B	O00300	TNFRSF11B	chr8:118951885	Inverse variance weighted	4	0.044	0.040	2.684E-01	0.913	cis	SARS-CoV-2 infection
3831_21	pTEN	Phosphatidylinositol 3,4,5-trisphosphate 3-phosphatase and dual-specificity protein phosphatase PTEN	P60484	PTEN	chr10:87862563	Wald ratio	1	-0.131	0.118	2.686E-01	0.913	cis	SARS-CoV-2 infection
4153_11	alpha-1-antichymotrypsin complex	Alpha-1-antichymotrypsin complex	P07288 | P01011	KLK3 | SERPINA3	chr14:94612384	Inverse variance weighted	3	0.054	0.049	2.698E-01	0.915	cis	SARS-CoV-2 infection
19115_13	CMBL	Carboxymethylenebutenolidase homolog	Q96DG6	CMBL	chr5:10307902	Wald ratio	1	0.035	0.032	2.727E-01	0.917	cis	SARS-CoV-2 infection
19367_34	D3D2	Enoyl-CoA delta isomerase 1, mitochondrial	P42126	ECI1	chr16:2252300	Inverse variance weighted	2	0.045	0.041	2.730E-01	0.917	cis	SARS-CoV-2 infection
14047_78	BDNF	Brain-derived neurotrophic factor	P23560	BDNF	chr11:27722058	Inverse variance weighted	2	-0.089	0.081	2.730E-01	0.917	cis	SARS-CoV-2 infection
9578_263	MANS4	MANSC domain-containing protein 4	A6NHS7	MANSC4	chr12:27780236	Inverse variance weighted	7	-0.012	0.011	2.750E-01	0.917	cis	SARS-CoV-2 infection
2834_54	kallikrein 8	Kallikrein-8	O60259	KLK8	chr19:51002711	Wald ratio	1	0.035	0.032	2.758E-01	0.917	cis	SARS-CoV-2 infection
13460_4	CHAD	Chondroadherin	O15335	CHAD	chr17:50468906	Wald ratio	1	0.105	0.096	2.759E-01	0.917	cis	SARS-CoV-2 infection
10818_36	ASM	Sphingomyelin phosphodiesterase	P17405	SMPD1	chr11:6390440	Inverse variance weighted	5	0.014	0.013	2.760E-01	0.917	cis	SARS-CoV-2 infection
3038_9	I-TAC	C-X-C motif chemokine 11	O14625	CXCL11	chr4:76041415	Inverse variance weighted	4	-0.058	0.053	2.762E-01	0.917	cis	SARS-CoV-2 infection
6234_74	VITRN	Vitrin	Q6UXI7	VIT	chr2:36696690	Inverse variance weighted	6	-0.016	0.015	2.763E-01	0.917	cis	SARS-CoV-2 infection
18312_68	NDRG3	Protein NDRG3	Q9UGV2	NDRG3	chr20:36746090	Inverse variance weighted	3	-0.069	0.063	2.766E-01	0.917	cis	SARS-CoV-2 infection
5609_92	F19A5	Protein FAM19A5	Q7Z5A7	TAFA5	chr22:48489553	Wald ratio	1	0.110	0.101	2.767E-01	0.917	cis	SARS-CoV-2 infection
15447_45	Sorbitol dehydrogenase	Sorbitol dehydrogenase	Q00796	SORD	chr15:45023147	Inverse variance weighted	5	0.023	0.021	2.768E-01	0.917	cis	SARS-CoV-2 infection
19615_213	5NT3	Cytosolic 5'-nucleotidase 3A	Q9H0P0	NT5C3A	chr7:33062796	Wald ratio	1	0.066	0.061	2.776E-01	0.918	cis	SARS-CoV-2 infection
17403_14	acyl-Coenzyme A dehydrogenase	Short/branched chain specific acyl-CoA dehydrogenase, mitochondrial	P45954	ACADSB	chr10:123008979	Inverse variance weighted	2	0.042	0.039	2.784E-01	0.919	cis	SARS-CoV-2 infection
13944_3	SULT 1A3	Sulfotransferase 1A3	P0DMM9	SULT1A3	chr16:30199228	Inverse variance weighted	4	0.078	0.073	2.813E-01	0.926	cis	SARS-CoV-2 infection
3196_6	HPLN1	Hyaluronan and proteoglycan link protein 1	P10915	HAPLN1	chr5:83720855	Inverse variance weighted	3	0.056	0.052	2.817E-01	0.926	cis	SARS-CoV-2 infection
14094_29	HB-EGF	Heparin-binding EGF-like growth factor	Q99075	HBEGF	chr5:140346603	Wald ratio	1	-0.103	0.096	2.835E-01	0.928	cis	SARS-CoV-2 infection
10440_26	ACAM:ECD	CXADR-like membrane protein:Extracellular domain	Q9H6B4	CLMP	chr11:123195248	Inverse variance weighted	3	0.035	0.032	2.836E-01	0.928	cis	SARS-CoV-2 infection
4209_60	DRG-1	Vacuolar protein sorting-associated protein VTA1 homolog	Q9NP79	VTA1	chr6:142147162	Wald ratio	1	-0.098	0.092	2.848E-01	0.930	cis	SARS-CoV-2 infection
8916_32	STIM1:CD	Stromal interaction molecule 1:Cytoplasmic domain	Q13586	STIM1	chr11:3854527	Wald ratio	1	-0.055	0.052	2.853E-01	0.930	cis	SARS-CoV-2 infection
3710_49	Angiostatin	Angiostatin	P00747	PLG	chr6:160702194	Inverse variance weighted	7	0.027	0.025	2.857E-01	0.930	cis	SARS-CoV-2 infection
12560_9	NT5C	5'(3')-deoxyribonucleotidase, cytosolic type	Q8TCD5	NT5C	chr17:75131757	Inverse variance weighted	5	-0.013	0.012	2.878E-01	0.931	cis	SARS-CoV-2 infection
15367_38	LPLC1	BPI fold-containing family B member 1	Q8TDL5	BPIFB1	chr20:33273480	Inverse variance weighted	7	-0.013	0.013	2.879E-01	0.931	cis	SARS-CoV-2 infection
14101_2	CNTFR alpha	Ciliary neurotrophic factor receptor subunit alpha	P26992	CNTFR	chr9:34590140	Inverse variance weighted	3	-0.035	0.033	2.882E-01	0.931	cis	SARS-CoV-2 infection
10075_75	ACBD6	Acyl-CoA-binding domain-containing protein 6	Q9BR61	ACBD6	chr1:180502954	Wald ratio	1	-0.093	0.088	2.888E-01	0.931	cis	SARS-CoV-2 infection
16908_5	OMGP	Oligodendrocyte-myelin glycoprotein	P23515	OMG	chr17:31297539	Wald ratio	1	-0.066	0.062	2.891E-01	0.931	cis	SARS-CoV-2 infection
14091_42	Carbonyl reductase 3	Carbonyl reductase [NADPH] 3	O75828	CBR3	chr21:36135079	Inverse variance weighted	6	0.018	0.017	2.894E-01	0.931	cis	SARS-CoV-2 infection
9175_48	DSCAM	Down syndrome cell adhesion molecule	O60469	DSCAM	chr21:40847158	Inverse variance weighted	2	-0.035	0.033	2.901E-01	0.931	cis	SARS-CoV-2 infection
11547_84	MUSK	Muscle, skeletal receptor tyrosine-protein kinase	O15146	MUSK	chr9:110668779	Wald ratio	1	0.085	0.080	2.909E-01	0.931	cis	SARS-CoV-2 infection
10832_24	B4GT6	Beta-1,4-galactosyltransferase 6	Q9UBX8	B4GALT6	chr18:31685836	Inverse variance weighted	4	-0.012	0.011	2.915E-01	0.931	cis	SARS-CoV-2 infection
13720_95	Proteinase-3	Myeloblastin	P24158	PRTN3	chr19:840999	Inverse variance weighted	7	0.013	0.013	2.923E-01	0.931	cis	SARS-CoV-2 infection
6388_21	CC126	Coiled-coil domain-containing protein 126	Q96EE4	CCDC126	chr7:23597382	Inverse variance weighted	4	-0.018	0.017	2.929E-01	0.931	cis	SARS-CoV-2 infection
3438_10	FSTL3	Follistatin-related protein 3	O95633	FSTL3	chr19:676392	Inverse variance weighted	2	-0.092	0.087	2.935E-01	0.931	cis	SARS-CoV-2 infection

17691_1	TPP1	Tripeptidyl-peptidase 1	O14773	TPP1	chr11:6619448	Inverse variance weighted	5	-0.026	0.025	2.939E-01	0.931	cis	SARS-CoV-2 infection
12399_194	CCD50	Coiled-coil domain-containing protein 50	Q8IVM0	CCDC50	chr3:191329085	Wald ratio	1	-0.100	0.096	2.943E-01	0.931	cis	SARS-CoV-2 infection
15494_11	FGFP1	Fibroblast growth factor-binding protein 1	Q14512	FGFBP1	chr4:15938740	Wald ratio	1	-0.070	0.067	2.945E-01	0.931	cis	SARS-CoV-2 infection
4979_34	DERM	Dermatopontin	Q07507	DPT	chr1:168729206	Inverse variance weighted	6	-0.013	0.013	2.950E-01	0.931	cis	SARS-CoV-2 infection
7997_118	DOC2B	Double C2-like domain-containing protein beta	Q14184	DOC2B	chr17:181650	Wald ratio	1	-0.116	0.111	2.957E-01	0.931	cis	SARS-CoV-2 infection
15533_97	Macrophage scavenger receptor:ECD	Macrophage scavenger receptor types I and II:Extracellular domain	P21757	MSR1	chr8:16567490	Inverse variance weighted	5	0.012	0.012	2.975E-01	0.935	cis	SARS-CoV-2 infection
19124_9	UBCP1	Ubiquitin-like domain-containing CTD phosphatase 1	Q8WVY7	UBLCP1	chr5:159263290	Inverse variance weighted	2	-0.045	0.043	2.982E-01	0.935	cis	SARS-CoV-2 infection
9459_7	Fas, soluble	Tumor necrosis factor receptor superfamily member 6	P25445	FAS	chr10:88953813	Inverse variance weighted	2	-0.085	0.082	2.985E-01	0.935	cis	SARS-CoV-2 infection
17783_9	MMAB	Cob(I)yrinic acid a,c-diamide adenosyltransferase, mitochondrial	Q96EY8	MMAB	chr12:109573580	Inverse variance weighted	6	0.014	0.013	2.993E-01	0.935	cis	SARS-CoV-2 infection
17332_3	ARHL2	Poly(ADP-ribose) glycohydrolase ARH3	Q9NX46	ADPRS	chr1:36088892	Inverse variance weighted	3	-0.037	0.036	2.996E-01	0.935	cis	SARS-CoV-2 infection
8288_27	b2-Glycoprotein I	Beta-2-glycoprotein 1	P02749	APOH	chr17:66256525	Inverse variance weighted	3	0.011	0.011	3.002E-01	0.935	cis	SARS-CoV-2 infection
18875_125	Chondrocalcin	Chondrocalcin	P02458	COL2A1	chr12:48004554	Inverse variance weighted	8	-0.015	0.014	3.006E-01	0.935	cis	SARS-CoV-2 infection
18321_38	MTDC	Bifunctional methylenetetrahydrofolate dehydrogenase/cyclohydrolase, mitochondrial	P13995	MTHFD2	chr2:74186172	Inverse variance weighted	2	0.038	0.037	3.036E-01	0.937	cis	SARS-CoV-2 infection
18289_16	MIP-5	C-C motif chemokine 15	Q16663	CCL15	chr17:36001553	Inverse variance weighted	5	0.027	0.027	3.039E-01	0.937	cis	SARS-CoV-2 infection
16558_2	MYOC	Myocilin	Q99972	MYOC	chr1:171652688	Inverse variance weighted	8	0.019	0.019	3.040E-01	0.937	cis	SARS-CoV-2 infection
4355_13	DAPK2	Death-associated protein kinase 2	Q9UIK4	DAPK2	chr15:64072033	Inverse variance weighted	3	0.017	0.017	3.049E-01	0.937	cis	SARS-CoV-2 infection
8248_222	SIG14	Sialic acid-binding Ig-like lectin 14	Q08ET2	SIGLEC14	chr19:51646825	Inverse variance weighted	3	0.052	0.051	3.049E-01	0.937	cis	SARS-CoV-2 infection
4220_39	FER	Tyrosine-protein kinase Fer	P16591	FER	chr5:108747841	Wald ratio	1	0.066	0.064	3.051E-01	0.937	cis	SARS-CoV-2 infection
9077_10	MA1A2	Mannosyl-oligosaccharide 1,2-alpha-mannosidase IB	O60476	MAN1A2	chr1:117367449	Inverse variance weighted	4	-0.032	0.031	3.052E-01	0.937	cis	SARS-CoV-2 infection
14156_33	14-3-3 protein beta/alpha	14-3-3 protein beta/alpha	P31946	YWHAB	chr20:44885702	Wald ratio	1	0.085	0.083	3.058E-01	0.937	cis	SARS-CoV-2 infection
18310_26	SELW	Selenoprotein W	P63302	SELENOW	chr19:47778677	Inverse variance weighted	3	-0.030	0.029	3.067E-01	0.937	cis	SARS-CoV-2 infection
3310_62	FCG2B	Low affinity immunoglobulin gamma Fc region receptor II-b	P31994	FCGR2B	chr1:161663143	Inverse variance weighted	12	-0.019	0.019	3.082E-01	0.937	cis	SARS-CoV-2 infection
14066_49	MAGI2	Membrane-associated guanylate kinase, WW and PDZ domain-containing protein 2	Q86UL8	MAGI2	chr7:79453667	Inverse variance weighted	6	-0.011	0.011	3.084E-01	0.937	cis	SARS-CoV-2 infection
5698_60	Tenascin-X	Tenascin-X	P22105	TNXB	chr6:32115334	Inverse variance weighted	5	-0.012	0.012	3.087E-01	0.937	cis	SARS-CoV-2 infection
12329_21	KS6A1	Ribosomal protein S6 kinase alpha-1	Q15418	RPS6KA1	chr1:26529761	Wald ratio	1	-0.050	0.049	3.087E-01	0.937	cis	SARS-CoV-2 infection
19622_7	Activin A	Activin A	P08476	INHBA	chr7:41705834	Wald ratio	1	-0.088	0.087	3.094E-01	0.938	cis	SARS-CoV-2 infection
8337_65	PTPRU	Receptor-type tyrosine-protein phosphatase U	Q92729	PTPRU	chr1:29236516	Inverse variance weighted	5	-0.033	0.033	3.133E-01	0.942	cis	SARS-CoV-2 infection
19560_23	PLXA4	Plexin-A4	Q9HCM2	PLXNA4	chr7:132648688	Wald ratio	1	-0.058	0.058	3.134E-01	0.942	cis	SARS-CoV-2 infection
5620_13	AMD	Peptidyl-glycine alpha-amidating monooxygenase	P19021	PAM	chr5:102753981	Inverse variance weighted	10	0.011	0.011	3.135E-01	0.942	cis	SARS-CoV-2 infection
17456_53	GOLM1	Golgi membrane protein 1	Q8NBJ4	GOLM1	chr9:86100173	Inverse variance weighted	6	-0.030	0.029	3.137E-01	0.942	cis	SARS-CoV-2 infection
15604_18	JNK2	Mitogen-activated protein kinase 9	P45984	MAPK9	chr5:180292099	Inverse variance weighted	2	-0.052	0.051	3.145E-01	0.942	cis	SARS-CoV-2 infection
17821_20	NMT2	Glycylpeptide N-tetradecanoyltransferase 2	O60551	NMT2	chr10:15168693	Wald ratio	1	0.076	0.076	3.145E-01	0.942	cis	SARS-CoV-2 infection
5069_9	DAF	Complement decay-accelerating factor	P08174	CD55	chr1:207321519	Inverse variance weighted	4	0.023	0.023	3.158E-01	0.942	cis	SARS-CoV-2 infection
19130_81	SPB8	Serpin B8	P50452	SERPINB8	chr18:63970029	Inverse variance weighted	3	-0.021	0.021	3.158E-01	0.942	cis	SARS-CoV-2 infection
3403_1	TPSB2	Tryptase beta-2	P20231	TPSB2	chr16:1230184	Inverse variance weighted	8	0.010	0.010	3.160E-01	0.942	cis	SARS-CoV-2 infection
4337_49	CRP	C-reactive protein	P02741	CRP	chr1:159714589	Inverse variance weighted	2	0.028	0.028	3.168E-01	0.943	cis	SARS-CoV-2 infection
13682_47	M-CSF R	Macrophage colony-stimulating factor 1 receptor	P07333	CSF1R	chr5:150113372	Wald ratio	1	-0.071	0.071	3.179E-01	0.943	cis	SARS-CoV-2 infection
13119_26	protein Z inhibitor	Protein Z-dependent protease inhibitor	Q9UK55	SERPINA10	chr14:94293268	Inverse variance weighted	6	0.011	0.011	3.179E-01	0.943	cis	SARS-CoV-2 infection
8229_1	GXLT1	Glucoside xylosyltransferase 1	Q4G148	GXYLT1	chr12:42144874	Inverse variance weighted	5	0.013	0.013	3.193E-01	0.944	cis	SARS-CoV-2 infection
18180_58	RT4I1	Reticulon-4-interacting protein 1, mitochondrial	Q8WWV3	RTN4IP1	chr6:106629498	Wald ratio	1	-0.076	0.076	3.197E-01	0.944	cis	SARS-CoV-2 infection
11424_4	FAAA	Fumarylacetoacetase	P16930	FAH	chr15:80152490	Inverse variance weighted	6	-0.010	0.010	3.209E-01	0.944	cis	SARS-CoV-2 infection
3168_8	ADAMTS-5	A disintegrin and metalloproteinase with thrombospondin motifs 5	Q9UNA0	ADAMTS5	chr21:26967088	Inverse variance weighted	5	-0.012	0.012	3.220E-01	0.944	cis	SARS-CoV-2 infection
5006_71	MK13	Mitogen-activated protein kinase 13	O15264	MAPK13	chr6:36127809	Wald ratio	1	-0.049	0.050	3.221E-01	0.944	cis	SARS-CoV-2 infection
18185_118	ALDOB	Fructose-bisphosphate aldolase B	P05062	ALDOB	chr9:101449664	Inverse variance weighted	2	0.016	0.016	3.228E-01	0.944	cis	SARS-CoV-2 infection
16872_248	MAAI	Maleylacetoacetate isomerase	O43708	GSTZ1	chr14:77320996	Inverse variance weighted	8	0.008	0.008	3.243E-01	0.944	cis	SARS-CoV-2 infection
8697_38	Glypican 1	Glypican-1	P35052	GPC1	chr2:240435663	Inverse variance weighted	6	-0.013	0.013	3.243E-01	0.944	cis	SARS-CoV-2 infection
9545_156	Granzyme K	Granzyme K	P49863	GZMK	chr5:55024256	Wald ratio	1	-0.032	0.033	3.246E-01	0.944	cis	SARS-CoV-2 infection
2687_2	MIA	Melanoma-derived growth regulatory protein	Q16674	MIA	chr19:40771648	Inverse variance weighted	7	-0.009	0.009	3.247E-01	0.944	cis	SARS-CoV-2 infection
9172_69	MMP-8	Neutrophil collagenase	P22894	MMP8	chr11:102727050	Wald ratio	1	0.048	0.049	3.251E-01	0.944	cis	SARS-CoV-2 infection
17756_69	DCTD	Deoxycytidylate deaminase	P32321	DCTD	chr4:182917936	Inverse variance weighted	2	0.047	0.048	3.257E-01	0.944	cis	SARS-CoV-2 infection
8250_2	PTPRJ	Receptor-type tyrosine-protein phosphatase eta	Q12913	PTPRJ	chr11:47980425	Inverse variance weighted	3	0.060	0.061	3.258E-01	0.944	cis	SARS-CoV-2 infection
15427_35	LOXL3	Lysyl oxidase homolog 3	P58215	LOXL3	chr2:74555690	Wald ratio	1	0.054	0.055	3.261E-01	0.944	cis	SARS-CoV-2 infection
15513_108	Prostasin	Prostasin	Q16651	PRSS8	chr16:31135727	Inverse variance weighted	2	0.057	0.058	3.270E-01	0.945	cis	SARS-CoV-2 infection
13059_33	RIFK	Riboflavin kinase	Q969G6	RFK	chr9:76394517	Inverse variance weighted	2	0.038	0.039	3.301E-01	0.947	cis	SARS-CoV-2 infection
10974_20	ISK7	Serine protease inhibitor Kazal-type 7	P58062	SPINK7	chr5:148312419	Wald ratio	1	0.040	0.041	3.305E-01	0.947	cis	SARS-CoV-2 infection
2700_56	Protein S	Vitamin K-dependent protein S	P07225	PROS1	chr3:93980003	Wald ratio	1	-0.083	0.085	3.306E-01	0.947	cis	SARS-CoV-2 infection
7947_19	AP4AT	AP-4 complex accessory subunit tepsin	Q96N21	TEPSIN	chr17:81239091	Wald ratio	1	0.084	0.086	3.310E-01	0.947	cis	SARS-CoV-2 infection
18896_23	H6ST3	Heparan-sulfate 6-O-sulfotransferase 3	Q8IZP7	HS6ST3	chr13:96090107	Wald ratio	1	0.048	0.050	3.315E-01	0.947	cis	SARS-CoV-2 infection
3420_21	Carbonic anhydrase XIII	Carbonic anhydrase 13	Q8N1Q1	CA13	chr8:85220587	Inverse variance weighted	3	-0.017	0.017	3.316E-01	0.947	cis	SARS-CoV-2 infection
3835_11	TLR2	Toll-like receptor 2	O60603	TLR2	chr4:153684070	Wald ratio	1	0.123	0.127	3.317E-01	0.947	cis	SARS-CoV-2 infection
10037_98	SIG12:Ig-like V-type 2	Sialic acid-binding Ig-like lectin 12:Ig-like V-type 2 domain, Isoform long	Q96PQ1	SIGLEC12	chr19:51501800	Inverse variance weighted	4	-0.029	0.030	3.338E-01	0.951	cis	SARS-CoV-2 infection
9838_4	SMAD1	Mothers against decapentaplegic homolog 1	Q15797	SMAD1	chr4:145481194	Wald ratio	1	-0.092	0.095	3.347E-01	0.952	cis	SARS-CoV-2 infection
18934_50	Tissue transglutaminase	Protein-glutamine gamma-glutamyltransferase 2	P21980	TGM2	chr20:38166578	Wald ratio	1	0.025	0.026	3.355E-01	0.953	cis	SARS-CoV-2 infection
11208_15	NAGPA	N-acetylglucosamine-1-phosphodiester alpha-N-acetylglucosaminidase	Q9UK23	NAGPA	chr16:5034141	Inverse variance weighted	7	0.013	0.013	3.362E-01	0.953	cis	SARS-CoV-2 infection
18933_4	TGM4	Protein-glutamine gamma-glutamyltransferase 4	P49221	TGM4	chr3:44874608	Wald ratio	1	-0.086	0.090	3.383E-01	0.956	cis	SARS-CoV-2 infection
3519_3	TARC	C-C motif chemokine 17	Q92583	CCL17	chr16:57404767	Inverse variance weighted	2	0.024	0.025	3.383E-01	0.956	cis	SARS-CoV-2 infection
15539_15	SLIK1	SLIT and NTRK-like protein 1	Q96PX8	SLITRK1	chr13:83882474	Wald ratio	1	-0.070	0.074	3.399E-01	0.958	cis	SARS-CoV-2 infection
19135_5	Cytosolic 5'-nucleotidase III-like protein	7-methylguanosine phosphate-specific 5'-nucleotidase	Q969T7	NT5C3B	chr17:41836260	Inverse variance weighted	9	-0.012	0.012	3.404E-01	0.958	cis	SARS-CoV-2 infection
5737_61	SEM4D	Semaphorin-4D	Q92854	SEMA4D	chr9:89498130	Inverse variance weighted	6	0.010	0.010	3.425E-01	0.961	cis	SARS-CoV-2 infection
6927_7	NDST1	Bifunctional heparan sulfate N-deacetylase/N-sulfotransferase 1	P52848	NDST1	chr5:150485818	Wald ratio	1	-0.033	0.035	3.448E-01	0.961	cis	SARS-CoV-2 infection
11219_95	FGFP3	Fibroblast growth factor-binding protein 3	Q8TAT2	FGFBP3	chr10:91909486	Inverse variance weighted	3	0.015	0.016	3.468E-01	0.961	cis	SARS-CoV-2 infection
16596_25	GLRX3	Glutaredoxin-3	O76003	GLRX3	chr10:130136391	Wald ratio	1	-0.055	0.059	3.470E-01	0.961	cis	SARS-CoV-2 infection
9321_400	NMB	Neuromedin-B	P08949	NMB	chr15:84658563	Inverse variance weighted	2	0.029	0.031	3.473E-01	0.961	cis	SARS-CoV-2 infection
18839_24	Thyroglobulin	Thyroglobulin	P01266	TG	chr8:132866958	Wald ratio	1	0.057	0.061	3.492E-01	0.961	cis	SARS-CoV-2 infection
6558_5	COL10	Collectin-10	Q9Y6Z7	COLEC10	chr8:118995452	Wald ratio	1	0.092	0.099	3.495E-01	0.961	cis	SARS-CoV-2 infection
8006_12	DJB12	DnaJ homolog subfamily B member 12	Q9NXW2	DNAJB12	chr10:72355149	Wald ratio	1	0.052	0.056	3.502E-01	0.961	cis	SARS-CoV-2 infection
2974_61	contactin-1	Contactin-1	Q12860	CNTN1	chr12:40692439	Inverse variance weighted	4	0.015	0.016	3.507E-01	0.961	cis	SARS-CoV-2 infection
19637_9	CRH	Corticoliberin	P06850	CRH	chr8:66178464	Wald ratio	1	0.076	0.081	3.510E-01	0.961	cis	SARS-CoV-2 infection

7980_72	B3GN2	N-acetyllactosaminide beta-1,3-N-acetylglucosaminyltransferase 2	Q9NY97	B3GNT2	chr2:62196115	Inverse variance weighted	3	0.030	0.032	3.513E-01	0.961	cis	SARS-CoV-2 infection
15529_33	Cysteine-rich protein 1	Cysteine and glycine-rich protein 1	P21291	CSRP1	chr1:201509456	Wald ratio	1	0.048	0.051	3.528E-01	0.961	cis	SARS-CoV-2 infection
19194_9	Histidyl-tRNA synthetase-related	D-tyrosyl-tRNA(Tyr) deacylase 1	Q8TEA8	DTD1	chr20:18587942	Wald ratio	1	0.026	0.028	3.545E-01	0.961	cis	SARS-CoV-2 infection
16882_27	PHP14	14 kDa phosphohistidine phosphatase	Q9NRX4	PHPT1	chr9:136848724	Inverse variance weighted	2	0.069	0.075	3.554E-01	0.961	cis	SARS-CoV-2 infection
3607_71	DKK3	Dickkopf-related protein 3	Q9UBP4	DKK3	chr11:12009769	Inverse variance weighted	4	-0.016	0.018	3.555E-01	0.961	cis	SARS-CoV-2 infection
12549_33	PTGD2	Hematopoietic prostaglandin D synthase	O60760	HPGDS	chr4:94342876	Inverse variance weighted	9	0.009	0.010	3.555E-01	0.961	cis	SARS-CoV-2 infection
6715_63	PPBN	Alkaline phosphatase, placental-like	P10696	ALPG	chr2:232406844	Inverse variance weighted	3	0.027	0.029	3.558E-01	0.961	cis	SARS-CoV-2 infection
15398_2	HERV1	FAD-linked sulfhydryl oxidase ALR	P55789	GFER	chr16:1984193	Wald ratio	1	-0.079	0.086	3.561E-01	0.961	cis	SARS-CoV-2 infection
3213_65	Nidogen	Nidogen-1	P14543	NID1	chr1:236065109	Inverse variance weighted	4	0.026	0.029	3.570E-01	0.961	cis	SARS-CoV-2 infection
9322_15	RCN1	Reticulocalbin-1	Q15293	RCN1	chr11:32091074	Wald ratio	1	-0.034	0.037	3.571E-01	0.961	cis	SARS-CoV-2 infection
10938_13	sLFA-3	Lymphocyte function-associated antigen 3	P19256	CD58	chr1:116571039	Inverse variance weighted	2	-0.025	0.027	3.571E-01	0.961	cis	SARS-CoV-2 infection
3421_54	CD30 Ligand	Tumor necrosis factor ligand superfamily member 8	P32971	TNFSF8	chr9:114930595	Inverse variance weighted	2	-0.040	0.043	3.575E-01	0.961	cis	SARS-CoV-2 infection
13717_15	FCN2	Ficolin-2	Q15485	FCN2	chr9:134880810	Inverse variance weighted	8	-0.015	0.016	3.591E-01	0.961	cis	SARS-CoV-2 infection
13438_115	CHRD	Chordin	Q9H2X0	CHRD	chr3:184380054	Wald ratio	1	-0.063	0.069	3.600E-01	0.961	cis	SARS-CoV-2 infection
3719_2	p27Kip1	Cyclin-dependent kinase inhibitor 1B	P46527	CDKN1B	chr12:12685498	Wald ratio	1	-0.055	0.061	3.613E-01	0.961	cis	SARS-CoV-2 infection
2741_22	Siglec-6	Sialic acid-binding Ig-like lectin 6	O43699	SIGLEC6	chr19:51531856	Inverse variance weighted	7	-0.012	0.014	3.627E-01	0.961	cis	SARS-CoV-2 infection
18338_26	IDH	Isocitrate dehydrogenase [NADP] cytoplasmic	O75874	IDH1	chr2:208266074	Inverse variance weighted	3	-0.014	0.016	3.631E-01	0.961	cis	SARS-CoV-2 infection
3191_50	WFKN1	WAP, kazal, immunoglobulin, kunitz and NTR domain-containing protein 1	Q96NZ8	WFIKKN1	chr16:629239	Inverse variance weighted	2	0.031	0.034	3.636E-01	0.961	cis	SARS-CoV-2 infection
12659_13	GTPB9	Obg-like ATPase 1	Q9NTK5	OLA1	chr2:174248599	Wald ratio	1	0.048	0.053	3.642E-01	0.961	cis	SARS-CoV-2 infection
15472_16	LRP11	Low-density lipoprotein receptor-related protein 11	Q86VZ4	LRP11	chr6:149864359	Inverse variance weighted	6	-0.020	0.022	3.643E-01	0.961	cis	SARS-CoV-2 infection
3302_58	CYTF	Cystatin-F	O76096	CST7	chr20:24949269	Inverse variance weighted	7	-0.009	0.010	3.647E-01	0.961	cis	SARS-CoV-2 infection
9369_174	NGL1	Leucine-rich repeat-containing protein 4C	Q9HCJ2	LRRC4C	chr11:41459773	Inverse variance weighted	3	-0.042	0.046	3.650E-01	0.961	cis	SARS-CoV-2 infection
7891_45	UGT 1A6	UDP-glucuronosyltransferase 1-6	P19224	UGT1A6	chr2:233691607	Inverse variance weighted	4	-0.023	0.025	3.664E-01	0.961	cis	SARS-CoV-2 infection
12338_27	EGFLA	Pikachurin	Q63HQ2	EGFLAM	chr5:38258409	Inverse variance weighted	3	0.016	0.017	3.669E-01	0.961	cis	SARS-CoV-2 infection
19563_3	SEZ6L	Seizure 6-like protein	Q9BYH1	SEZ6L	chr22:26169462	Inverse variance weighted	3	0.041	0.046	3.673E-01	0.961	cis	SARS-CoV-2 infection
3580_25	a1-Antitrypsin	Alpha-1-antitrypsin	P01009	SERPINA1	chr14:94390693	Inverse variance weighted	7	0.008	0.009	3.674E-01	0.961	cis	SARS-CoV-2 infection
8957_72	XTP3B	Endoplasmic reticulum lectin 1	Q96DZ1	ERLEC1	chr2:53787009	Wald ratio	1	-0.065	0.073	3.694E-01	0.961	cis	SARS-CoV-2 infection
11257_1	DHPR	Dihydropteridine reductase	P09417	QDPR	chr4:17512206	Inverse variance weighted	4	0.015	0.017	3.699E-01	0.961	cis	SARS-CoV-2 infection
3194_36	GPVI	Platelet glycoprotein VI	Q9HCN6	GP6	chr19:55038264	Inverse variance weighted	2	-0.017	0.018	3.706E-01	0.961	cis	SARS-CoV-2 infection
3326_58	Nectin-like protein 2	Cell adhesion molecule 1	Q9BY67	CADM1	chr11:115504957	Wald ratio	1	0.031	0.035	3.707E-01	0.961	cis	SARS-CoV-2 infection
9574_11	BIN1	Myc box-dependent-interacting protein 1	O00499	BIN1	chr2:127107288	Inverse variance weighted	2	0.031	0.034	3.719E-01	0.961	cis	SARS-CoV-2 infection
17808_37	NIT2	Omega-amidase NIT2	Q9NQR4	NIT2	chr3:100334739	Inverse variance weighted	2	0.037	0.041	3.719E-01	0.961	cis	SARS-CoV-2 infection
9599_6	PIANP	PILR alpha-associated neural protein	Q8IYJ0	PIANP	chr12:6700815	Wald ratio	1	-0.093	0.105	3.730E-01	0.961	cis	SARS-CoV-2 infection
5465_32	H6ST1	Heparan-sulfate 6-O-sulfotransferase 1	O60243	HS6ST1	chr2:128318868	Inverse variance weighted	2	-0.026	0.029	3.734E-01	0.961	cis	SARS-CoV-2 infection
2774_10	IL-16	Interleukin-16	Q14005	IL16	chr15:81159575	Inverse variance weighted	10	-0.009	0.010	3.749E-01	0.961	cis	SARS-CoV-2 infection
9341_1	PDGFD	Platelet-derived growth factor D	Q9GZP0	PDGFD	chr11:104164379	Inverse variance weighted	3	0.052	0.059	3.761E-01	0.961	cis	SARS-CoV-2 infection
8893_29	PARP:region 1	Poly [ADP-ribose] polymerase 1:region 1	P09874	PARP1	chr1:226408154	Wald ratio	1	-0.082	0.093	3.762E-01	0.961	cis	SARS-CoV-2 infection
6605_17	IGFALS	Insulin-like growth factor-binding protein complex acid labile subunit	P35858	IGFALS	chr16:1794971	Inverse variance weighted	3	0.039	0.044	3.762E-01	0.961	cis	SARS-CoV-2 infection
15622_13	OBCAM	Opioid-binding protein/cell adhesion molecule	Q14982	OPCML	chr11:133532501	Wald ratio	1	-0.089	0.101	3.763E-01	0.961	cis	SARS-CoV-2 infection
9834_62	ADH1B	Alcohol dehydrogenase 1B	P00325	ADH1B	chr4:99352760	Wald ratio	1	0.044	0.050	3.770E-01	0.961	cis	SARS-CoV-2 infection
4499_21	PDGF-AA	Platelet-derived growth factor subunit A	P04085	PDGFA	chr7:520296	Inverse variance weighted	3	-0.049	0.055	3.771E-01	0.961	cis	SARS-CoV-2 infection
9204_33	Corticotropin-lipotropin	Pro-opiomelanocortin	P01189	POMC	chr2:25168903	Inverse variance weighted	3	-0.046	0.052	3.775E-01	0.961	cis	SARS-CoV-2 infection
7810_20	C1QTNF5	Complement C1q tumor necrosis factor-related protein 5	Q9BXJ0	C1QTNF5	chr11:119340940	Inverse variance weighted	2	-0.019	0.022	3.781E-01	0.961	cis	SARS-CoV-2 infection
2851_63	C5a	C5a anaphylatoxin	P01031	C5	chr9:121075195	Inverse variance weighted	2	0.035	0.039	3.784E-01	0.961	cis	SARS-CoV-2 infection
12370_30	Apo F	Apolipoprotein F	Q13790	APOF	chr12:56362857	Inverse variance weighted	3	-0.027	0.030	3.791E-01	0.961	cis	SARS-CoV-2 infection
6207_10	prosaposin	Prosaposin	P07602	PSAP	chr10:71851251	Inverse variance weighted	3	0.016	0.018	3.797E-01	0.961	cis	SARS-CoV-2 infection
12620_3	Septin-11	Septin-11	Q9NVA2	SEPTIN11	chr4:76949751	Wald ratio	1	-0.070	0.080	3.805E-01	0.961	cis	SARS-CoV-2 infection
17782_23	THIK	3-ketoacyl-CoA thiolase, peroxisomal	P09110	ACAA1	chr3:38137242	Inverse variance weighted	5	0.015	0.017	3.812E-01	0.961	cis	SARS-CoV-2 infection
3171_57	amyloid precursor protein	Amyloid beta A4 protein	P05067	APP	chr21:26171128	Wald ratio	1	0.055	0.063	3.822E-01	0.961	cis	SARS-CoV-2 infection
8255_34	MRVI1	Protein MRVI1	Q9Y6F6	IRAG1	chr11:10693988	Inverse variance weighted	2	-0.038	0.044	3.875E-01	0.961	cis	SARS-CoV-2 infection
11568_2	FKB1B	Peptidyl-prolyl cis-trans isomerase FKBP1B	P68106	FKBP1B	chr2:24049701	Wald ratio	1	-0.037	0.043	3.875E-01	0.961	cis	SARS-CoV-2 infection
17751_68	CRBB1	Beta-crystallin B1	P53674	CRYBB1	chr22:26618027	Wald ratio	1	-0.023	0.027	3.882E-01	0.961	cis	SARS-CoV-2 infection
5593_11	PDIA5	Protein disulfide-isomerase A5	Q14554	PDIA5	chr3:123067025	Inverse variance weighted	8	-0.007	0.008	3.890E-01	0.961	cis	SARS-CoV-2 infection
18830_1	Omentin	Intelectin-1	Q8WWA0	ITLN1	chr1:160885180	Wald ratio	1	0.069	0.080	3.891E-01	0.961	cis	SARS-CoV-2 infection
3364_76	Cathepsin V	Cathepsin L2	O60911	CTSV	chr9:97156556	Inverse variance weighted	4	-0.025	0.029	3.893E-01	0.961	cis	SARS-CoV-2 infection
18432_32	RALB	Ras-related protein Ral-B	P11234	RALB	chr2:120240064	Wald ratio	1	-0.018	0.021	3.894E-01	0.961	cis	SARS-CoV-2 infection
4886_3	MCP-3	C-C motif chemokine 7	P80098	CCL7	chr17:34270221	Inverse variance weighted	4	-0.018	0.021	3.895E-01	0.961	cis	SARS-CoV-2 infection
4413_3	SLPI	Antileukoproteinase	P03973	SLPI	chr20:45254564	Wald ratio	1	-0.082	0.095	3.899E-01	0.961	cis	SARS-CoV-2 infection
4563_61	PLCG1	1-phosphatidylinositol 4,5-bisphosphate phosphodiesterase gamma-1	P19174	PLCG1	chr20:41136960	Wald ratio	1	-0.074	0.086	3.901E-01	0.961	cis	SARS-CoV-2 infection
4960_72	annexin I	Annexin A1	P04083	ANXA1	chr9:73151865	Wald ratio	1	0.033	0.038	3.911E-01	0.961	cis	SARS-CoV-2 infection
4324_33	CYTT	Cystatin-SA	P09228	CST2	chr20:23826729	Inverse variance weighted	5	0.047	0.055	3.911E-01	0.961	cis	SARS-CoV-2 infection
15594_47	HTRA1	Serine protease HTRA1	Q92743	HTRA1	chr10:122458551	Inverse variance weighted	2	-0.042	0.049	3.913E-01	0.961	cis	SARS-CoV-2 infection
9348_1	C1RL1	Complement C1r subcomponent-like protein	Q9NZP8	C1RL	chr12:7109238	Wald ratio	1	-0.063	0.073	3.914E-01	0.961	cis	SARS-CoV-2 infection
15394_79	UNC5B	Netrin receptor UNC5B	Q8IZJ1	UNC5B	chr10:71212570	Inverse variance weighted	3	-0.030	0.035	3.929E-01	0.961	cis	SARS-CoV-2 infection
7185_29	GPV	Platelet glycoprotein V	P40197	GP5	chr3:194399266	Inverse variance weighted	2	-0.047	0.055	3.935E-01	0.961	cis	SARS-CoV-2 infection
12517_52	TFAR19	Programmed cell death protein 5	O14737	PDCD5	chr19:32581190	Inverse variance weighted	4	0.013	0.015	3.952E-01	0.961	cis	SARS-CoV-2 infection
9832_33	HGD	Homogentisate 1,2-dioxygenase	Q93099	HGD	chr3:120682269	Inverse variance weighted	2	-0.034	0.040	3.957E-01	0.961	cis	SARS-CoV-2 infection
15558_63	AMPE	Glutamyl aminopeptidase	Q07075	ENPEP	chr4:110365733	Inverse variance weighted	8	0.013	0.015	3.962E-01	0.961	cis	SARS-CoV-2 infection
18342_2	SERC	Phosphoserine aminotransferase	Q9Y617	PSAT1	chr9:78297125	Inverse variance weighted	2	-0.031	0.036	3.964E-01	0.961	cis	SARS-CoV-2 infection
8097_77	LIPN	Lipase member N	Q5VXI9	LIPN	chr10:88759982	Inverse variance weighted	6	-0.009	0.011	3.964E-01	0.961	cis	SARS-CoV-2 infection
18435_40	UBX2B	UBX domain-containing protein 2B	Q14CS0	UBXN2B	chr8:58411359	Wald ratio	1	-0.068	0.080	3.972E-01	0.961	cis	SARS-CoV-2 infection
8773_172	EMIL3:region 1	EMILIN-3:region 1	Q9NT22	EMILIN3	chr20:41366818	Inverse variance weighted	6	-0.013	0.015	3.977E-01	0.961	cis	SARS-CoV-2 infection
9759_13	INDO	Indoleamine 2,3-dioxygenase 1	P14902	IDO1	chr8:39902275	Wald ratio	1	0.070	0.083	3.979E-01	0.961	cis	SARS-CoV-2 infection
10948_14	PLD3	Phospholipase D3	Q8IV08	PLD3	chr19:40348456	Wald ratio	1	0.081	0.096	3.982E-01	0.961	cis	SARS-CoV-2 infection
3050_7	vWF	von Willebrand factor	P04275	VWF	chr12:6124770	Inverse variance weighted	4	0.034	0.040	3.987E-01	0.961	cis	SARS-CoV-2 infection
9394_19	Aminopeptidase	Carboxypeptidase Q	Q9Y646	CPQ	chr8:96645242	Inverse variance weighted	8	0.010	0.011	3.987E-01	0.961	cis	SARS-CoV-2 infection
9126_171	NT5D3	5'-nucleotidase domain-containing protein 3	Q86UY8	NT5DC3	chr12:103841234	Inverse variance weighted	2	0.025	0.029	3.990E-01	0.961	cis	SARS-CoV-2 infection
11161_5	SPG20	Spartin	Q8N0X7	SPART	chr13:36370180	Wald ratio	1	0.083	0.099	4.004E-01	0.961	cis	SARS-CoV-2 infection

17698_15	WBP2	WW domain-binding protein 2	Q969T9	WBP2	chr17:75856507	Wald ratio	1	-0.083	0.099	4.016E-01	0.961	cis	SARS-CoV-2 infection
5132_71	TCCR	Interleukin-27 receptor subunit alpha	Q6UWB1	IL27RA	chr19:14031762	Inverse variance weighted	7	-0.008	0.009	4.016E-01	0.961	cis	SARS-CoV-2 infection
17755_5	UGDH	UDP-glucose 6-dehydrogenase	O60701	UGDH	chr4:39528311	Inverse variance weighted	3	0.018	0.022	4.019E-01	0.961	cis	SARS-CoV-2 infection
11649_3	SMAP1	Stromal membrane-associated protein 1	Q8IYB5	SMAP1	chr6:70667776	Inverse variance weighted	4	-0.012	0.015	4.049E-01	0.961	cis	SARS-CoV-2 infection
5852_6	S100A12	Protein S100-A12	P80511	S100A12	chr1:153375621	Inverse variance weighted	2	0.033	0.040	4.053E-01	0.961	cis	SARS-CoV-2 infection
8080_24	PSMP	Prostate-associated microseminoprotein	Q1L6U9	MSMP	chr9:35756613	Wald ratio	1	0.062	0.075	4.066E-01	0.961	cis	SARS-CoV-2 infection
3344_60	Antithrombin III	Antithrombin-III	P01008	SERPINC1	chr1:173917327	Wald ratio	1	0.069	0.083	4.069E-01	0.961	cis	SARS-CoV-2 infection
5598_3	GREM2	Gremlin-2	Q9H772	GREM2	chr1:240612155	Wald ratio	1	-0.069	0.084	4.090E-01	0.961	cis	SARS-CoV-2 infection
17802_4	SIAS	Sialic acid synthase	Q9NR45	NANS	chr9:98056732	Wald ratio	1	-0.046	0.056	4.106E-01	0.961	cis	SARS-CoV-2 infection
3806_55	EphA5	Ephrin type-A receptor 5	P54756	EPHA5	chr4:65670495	Inverse variance weighted	3	0.034	0.041	4.109E-01	0.961	cis	SARS-CoV-2 infection
4548_4	Fucosyltransferase 3	Galactoside 3(4)-L-fucosyltransferase	P21217	FUT3	chr19:5851471	Inverse variance weighted	9	-0.015	0.018	4.123E-01	0.961	cis	SARS-CoV-2 infection
13931_22	PSMD9	26S proteasome non-ATPase regulatory subunit 9	O00233	PSMD9	chr12:121888732	Wald ratio	1	0.032	0.039	4.124E-01	0.961	cis	SARS-CoV-2 infection
16853_5	NRK1	Nicotinamide riboside kinase 1	Q9NWW6	NMRK1	chr9:75088217	Inverse variance weighted	2	0.024	0.030	4.129E-01	0.961	cis	SARS-CoV-2 infection
19523_215	PARK7	Protein DJ-1	Q99497	PARK7	chr1:7954291	Wald ratio	1	-0.037	0.045	4.130E-01	0.961	cis	SARS-CoV-2 infection
5085_18	IL-20 Ra	Interleukin-20 receptor subunit alpha	Q9UHF4	IL20RA	chr6:137045180	Wald ratio	1	0.069	0.085	4.134E-01	0.961	cis	SARS-CoV-2 infection
4314_12	XTP3A	dCTP pyrophosphatase 1	Q9H773	DCTPP1	chr16:30430030	Wald ratio	1	-0.059	0.072	4.137E-01	0.961	cis	SARS-CoV-2 infection
13114_50	Lumican	Lumican	P51884	LUM	chr12:91111494	Inverse variance weighted	7	-0.015	0.018	4.138E-01	0.961	cis	SARS-CoV-2 infection
3807_1	FGF23	Fibroblast growth factor 23	Q9GZV9	FGF23	chr12:4379712	Wald ratio	1	-0.077	0.094	4.141E-01	0.961	cis	SARS-CoV-2 infection
17333_20	ACADM	Medium-chain specific acyl-CoA dehydrogenase, mitochondrial	P11310	ACADM	chr1:75724431	Wald ratio	1	0.018	0.023	4.145E-01	0.961	cis	SARS-CoV-2 infection
11530_37	HEM3	Porphobilinogen deaminase	P08397	HMBS	chr11:119084866	Wald ratio	1	-0.077	0.094	4.156E-01	0.961	cis	SARS-CoV-2 infection
19617_5	LTB4DH	Prostaglandin reductase 1	Q14914	PTGR1	chr9:111599893	Inverse variance weighted	8	-0.007	0.009	4.156E-01	0.961	cis	SARS-CoV-2 infection
3825_18	MK08	Mitogen-activated protein kinase 8	P45983	MAPK8	chr10:48306639	Wald ratio	1	-0.073	0.090	4.159E-01	0.961	cis	SARS-CoV-2 infection
16060_99	NID2	Nidogen-2	Q14112	NID2	chr14:52069059	Inverse variance weighted	6	0.010	0.012	4.160E-01	0.961	cis	SARS-CoV-2 infection
6467_65	KTEL1	Protein O-glucosyltransferase 1	Q8NBL1	POGLUT1	chr3:119468963	Inverse variance weighted	3	0.017	0.021	4.160E-01	0.961	cis	SARS-CoV-2 infection
12573_80	TRIM3	Tripartite motif-containing protein 3	O75382	TRIM3	chr11:6474459	Wald ratio	1	0.037	0.045	4.171E-01	0.961	cis	SARS-CoV-2 infection
3894_15	NAGK	N-acetyl-D-glucosamine kinase	Q9UJ70	NAGK	chr2:71064344	Inverse variance weighted	3	0.021	0.026	4.173E-01	0.961	cis	SARS-CoV-2 infection
5066_134	CLM6	CMRF35-like molecule 6	Q08708	CD300C	chr17:74546115	Inverse variance weighted	4	-0.054	0.066	4.173E-01	0.961	cis	SARS-CoV-2 infection
7179_69	NFASC	Neurofascin	O94856	NFASC	chr1:204828651	Inverse variance weighted	4	-0.011	0.013	4.173E-01	0.961	cis	SARS-CoV-2 infection
10558_26	PCDH9	Protocadherin-9	Q9HC56	PCDH9	chr13:67230445	Inverse variance weighted	4	-0.014	0.017	4.177E-01	0.961	cis	SARS-CoV-2 infection
11237_49	PCOC1	Procollagen C-endopeptidase enhancer 1	Q15113	PCOLCE	chr7:100602363	Inverse variance weighted	2	0.058	0.072	4.180E-01	0.961	cis	SARS-CoV-2 infection
12431_13	PELO	Protein pelota homolog	Q9BRX2	PELO	chr5:52787916	Wald ratio	1	0.085	0.105	4.180E-01	0.961	cis	SARS-CoV-2 infection
6919_3	HBAZ	Hemoglobin subunit zeta	P02008	HBZ	chr16:142728	Inverse variance weighted	6	0.008	0.010	4.182E-01	0.961	cis	SARS-CoV-2 infection
2647_66	Rab GDP dissociation inhibitor beta	Rab GDP dissociation inhibitor beta	P50395	GDI2	chr10:5842132	Wald ratio	1	-0.040	0.050	4.189E-01	0.961	cis	SARS-CoV-2 infection
9841_197	Multifunctional protein ADE2	Multifunctional protein ADE2	P22234	PAICS	chr4:56435741	Wald ratio	1	0.049	0.061	4.200E-01	0.961	cis	SARS-CoV-2 infection
3044_3	PARC	C-C motif chemokine 18	P55774	CCL18	chr17:36064272	Inverse variance weighted	4	0.031	0.038	4.209E-01	0.961	cis	SARS-CoV-2 infection
11643_73	DTX3L	E3 ubiquitin-protein ligase DTX3L	Q8TDB6	DTX3L	chr3:122564338	Wald ratio	1	-0.059	0.073	4.214E-01	0.961	cis	SARS-CoV-2 infection
7933_75	ADA22	Disintegrin and metalloproteinase domain-containing protein 22	Q9P0K1	ADAM22	chr7:87934143	Inverse variance weighted	4	-0.015	0.019	4.215E-01	0.961	cis	SARS-CoV-2 infection
5034_79	Trypsin 2	Trypsin-2	P07478	PRSS2	chr7:142760398	Inverse variance weighted	5	-0.019	0.024	4.218E-01	0.961	cis	SARS-CoV-2 infection
4459_68	PCSK7	Proprotein convertase subtilisin/kexin type 7	Q16549	PCSK7	chr11:117232525	Inverse variance weighted	10	-0.009	0.012	4.226E-01	0.961	cis	SARS-CoV-2 infection
3805_16	Endocan	Endothelial cell-specific molecule 1	Q9NQ30	ESM1	chr5:55022671	Inverse variance weighted	3	0.025	0.031	4.227E-01	0.961	cis	SARS-CoV-2 infection
8973_23	FCRL4:ECD	Fc receptor-like protein 4:Extracellular domain	Q96PJ5	FCRL4	chr1:157598085	Inverse variance weighted	13	-0.011	0.014	4.227E-01	0.961	cis	SARS-CoV-2 infection
15375_49	Carboxypeptidase B1	Carboxypeptidase B	P15086	CPB1	chr3:148791102	Inverse variance weighted	2	-0.027	0.034	4.230E-01	0.961	cis	SARS-CoV-2 infection
5763_67	HBD-4	Beta-defensin 104	Q8WTQ1	DEFB104A	chr8:7836436	Inverse variance weighted	2	-0.084	0.105	4.241E-01	0.961	cis	SARS-CoV-2 infection
3290_50	CD109	CD109 antigen	Q6YHK3	CD109	chr6:73695785	Inverse variance weighted	7	-0.009	0.011	4.243E-01	0.961	cis	SARS-CoV-2 infection
3495_15	GCP-2	C-X-C motif chemokine 6	P80162	CXCL6	chr4:73836640	Inverse variance weighted	7	-0.017	0.021	4.244E-01	0.961	cis	SARS-CoV-2 infection
8310_6	U773	Zymogen granule protein 16 homolog B	Q96DA0	ZG16B	chr16:2830253	Wald ratio	1	-0.025	0.031	4.247E-01	0.961	cis	SARS-CoV-2 infection
9863_1	Tropomyosin 4	Tropomyosin alpha-4 chain	P67936	TPM4	chr19:16067021	Wald ratio	1	0.054	0.068	4.247E-01	0.961	cis	SARS-CoV-2 infection
10737_96	Serpin B1	Leukocyte elastase inhibitor	P30740	SERPINB1	chr6:2841959	Wald ratio	1	0.060	0.075	4.256E-01	0.961	cis	SARS-CoV-2 infection
9264_11	CATO	Cathepsin O	P43234	CTSO	chr4:155953912	Inverse variance weighted	2	0.025	0.032	4.258E-01	0.961	cis	SARS-CoV-2 infection
5638_23	GT251	Procollagen galactosyltransferase 1	Q8NBJ5	COLGALT1	chr19:17555649	Inverse variance weighted	3	0.026	0.033	4.259E-01	0.961	cis	SARS-CoV-2 infection
3327_27	NET4	Netrin-4	Q9HB63	NTN4	chr12:95791189	Inverse variance weighted	3	0.017	0.021	4.264E-01	0.961	cis	SARS-CoV-2 infection
4706_17	41	Protein 4.1	P11171	EPB41	chr1:28887091	Inverse variance weighted	2	-0.068	0.086	4.266E-01	0.961	cis	SARS-CoV-2 infection
9986_14	Neuropeptide W	Neuropeptide W	Q8N729	NPW	chr16:2009926	Inverse variance weighted	3	-0.020	0.025	4.276E-01	0.961	cis	SARS-CoV-2 infection
19136_22	MMSA	Methylmalonate-semialdehyde dehydrogenase [acylating], mitochondrial	Q02252	ALDH6A1	chr14:74084492	Inverse variance weighted	2	-0.046	0.058	4.278E-01	0.961	cis	SARS-CoV-2 infection
11615_16	DAPP1	Dual adapter for phosphotyrosine and 3-phosphotyrosine and 3-phosphoinositide	Q9UN19	DAPP1	chr4:99816827	Wald ratio	1	0.044	0.056	4.284E-01	0.961	cis	SARS-CoV-2 infection
8235_48	SCG1	Secretogranin-1	P05060	CHGB	chr20:5911510	Wald ratio	1	0.047	0.060	4.291E-01	0.961	cis	SARS-CoV-2 infection
17383_4	STAR5	StAR-related lipid transfer protein 5	Q9NSY2	STARD5	chr15:81324183	Inverse variance weighted	6	0.025	0.031	4.298E-01	0.961	cis	SARS-CoV-2 infection
9981_18	K1467:C-term	Protein FAM234B:C-term	A2RU67	FAM234B	chr12:13044381	Inverse variance weighted	5	-0.021	0.026	4.300E-01	0.961	cis	SARS-CoV-2 infection
4559_64	KYNU	Kynureninase	Q16719	KYNU	chr2:142877657	Inverse variance weighted	4	-0.012	0.016	4.302E-01	0.961	cis	SARS-CoV-2 infection
15368_3	BMPER	BMP-binding endothelial regulator protein	Q8N8U9	BMPER	chr7:33904308	Inverse variance weighted	3	0.025	0.032	4.305E-01	0.961	cis	SARS-CoV-2 infection
16805_5	PDE5A	cGMP-specific 3',5'-cyclic phosphodiesterase	O76074	PDE5A	chr4:119628804	Wald ratio	1	0.080	0.101	4.306E-01	0.961	cis	SARS-CoV-2 infection
5658_64	coagulation factor XIII B	Coagulation factor XIII B chain	P05160	F13B	chr1:197067260	Wald ratio	1	-0.038	0.049	4.315E-01	0.961	cis	SARS-CoV-2 infection
3311_27	FCG3B	Low affinity immunoglobulin gamma Fc region receptor III-B	O75015	FCGR3B	chr1:161631963	Wald ratio	1	-0.086	0.109	4.326E-01	0.961	cis	SARS-CoV-2 infection
5687_5	GLU2B	Glucosidase 2 subunit beta	P14314	PRKCSH	chr19:11435284	Wald ratio	1	-0.070	0.090	4.339E-01	0.961	cis	SARS-CoV-2 infection
15387_44	Neuropilin-2	Neuropilin-2	O60462	NRP2	chr2:205681990	Inverse variance weighted	4	0.019	0.024	4.339E-01	0.961	cis	SARS-CoV-2 infection
11351_233	NHEJ1	Non-homologous end-joining factor 1	Q9H9Q4	NHEJ1	chr2:219160869	Inverse variance weighted	3	-0.060	0.076	4.339E-01	0.961	cis	SARS-CoV-2 infection
17153_46	KI2L3	Killer cell immunoglobulin-like receptor 2DL3	P43628	KIR2DL3	chr19:54738513	Inverse variance weighted	5	-0.009	0.012	4.346E-01	0.961	cis	SARS-CoV-2 infection
2190_55	Coagulation Factor XI	Coagulation Factor XI	P03951	F11	chr4:186266189	Inverse variance weighted	4	-0.015	0.019	4.346E-01	0.961	cis	SARS-CoV-2 infection
18873_8	CEAM8	Carcinoembryonic antigen-related cell adhesion molecule 8	P31997	CEACAM8	chr19:42595055	Wald ratio	1	-0.082	0.105	4.349E-01	0.961	cis	SARS-CoV-2 infection
17384_110	K6PF	ATP-dependent 6-phosphofructokinase, muscle type	P08237	PFKM	chr12:48105139	Wald ratio	1	-0.039	0.050	4.349E-01	0.961	cis	SARS-CoV-2 infection
12703_6	NEK7	Serine/threonine-protein kinase Nek7	Q8TDX7	NEK7	chr1:198156994	Wald ratio	1	0.038	0.048	4.352E-01	0.961	cis	SARS-CoV-2 infection
6470_19	fibulin 1	Fibulin-1	P23142	FBLN1	chr22:45502238	Inverse variance weighted	3	-0.025	0.032	4.359E-01	0.961	cis	SARS-CoV-2 infection
18876_77	CHST4	Carbohydrate sulfotransferase 4	Q8NCG5	CHST4	chr16:71525233	Wald ratio	1	0.082	0.105	4.368E-01	0.961	cis	SARS-CoV-2 infection
9265_10	GLIP1	Glioma pathogenesis-related protein 1	P48060	GLIPR1	chr12:75480753	Wald ratio	1	0.042	0.055	4.378E-01	0.961	cis	SARS-CoV-2 infection
5939_42	TWEAK	Tumor necrosis factor ligand superfamily member 12	O43508	TNFSF12	chr17:7548508	Inverse variance weighted	3	0.032	0.041	4.380E-01	0.961	cis	SARS-CoV-2 infection
16913_8	RNT2	Ribonuclease T2	O00584	RNASET2	chr6:166957191	Inverse variance weighted	6	0.009	0.012	4.381E-01	0.961	cis	SARS-CoV-2 infection
9294_45	MFAP2	Microfibrillar-associated protein 2	P55001	MFAP2	chr1:16980632	Inverse variance weighted	2	0.096	0.124	4.384E-01	0.961	cis	SARS-CoV-2 infection
11646_4	Carbohydrate sulfotransferase 9	Carbohydrate sulfotransferase 9	Q7L1S5	CHST9	chr18:27185308	Inverse variance weighted	4	-0.021	0.027	4.387E-01	0.961	cis	SARS-CoV-2 infection

17819_30	FAHD1	Acylpyruvase FAHD1, mitochondrial	Q6P587	FAHD1	chr16:1826967	Inverse variance weighted	3	-0.038	0.049	4.389E-01	0.961	cis	SARS-CoV-2 infection
2950_57	IGFBP-4	Insulin-like growth factor-binding protein 4	P22692	IGFBP4	chr17:40443450	Wald ratio	1	-0.067	0.087	4.391E-01	0.961	cis	SARS-CoV-2 infection
5810_25	Cripto	Teratocarcinoma-derived growth factor 1	P13385	TDGF1	chr3:46574534	Inverse variance weighted	6	0.020	0.026	4.400E-01	0.961	cis	SARS-CoV-2 infection
9015_1	PRG3	Proteoglycan 3	Q9Y2Y8	PRG3	chr11:57381150	Wald ratio	1	-0.115	0.149	4.421E-01	0.961	cis	SARS-CoV-2 infection
5980_55	BOLA3	BolA-like protein 3	Q53S33	BOLA3	chr2:74147912	Wald ratio	1	0.032	0.041	4.430E-01	0.961	cis	SARS-CoV-2 infection
6404_20	C1QRF	C1q-related factor	O75973	C1QL1	chr17:44968303	Inverse variance weighted	3	-0.020	0.026	4.437E-01	0.961	cis	SARS-CoV-2 infection
15343_337	Kininogen, HMW, Two Chain	Kininogen, HMW, Two Chain	P01042	KNG1	chr3:186717348	Inverse variance weighted	4	-0.039	0.051	4.439E-01	0.961	cis	SARS-CoV-2 infection
2590_69	ROR1	Inactive tyrosine-protein kinase transmembrane receptor ROR1	Q01973	ROR1	chr1:63774017	Inverse variance weighted	3	-0.014	0.019	4.441E-01	0.961	cis	SARS-CoV-2 infection
3220_40	RET	Proto-oncogene tyrosine-protein kinase receptor Ret	P07949	RET	chr10:43077064	Inverse variance weighted	4	-0.011	0.014	4.442E-01	0.961	cis	SARS-CoV-2 infection
3332_57	RGM-C	Hemojuvelin	Q6ZVN8	HJV	chr1:146036746	Wald ratio	1	0.055	0.072	4.442E-01	0.961	cis	SARS-CoV-2 infection
2982_82	Galectin-4	Galectin-4	P56470	LGALS4	chr19:38812945	Wald ratio	1	-0.067	0.088	4.443E-01	0.961	cis	SARS-CoV-2 infection
8297_8	DJC10	DnaJ homolog subfamily C member 10	Q8IXB1	DNAJC10	chr2:182716255	Inverse variance weighted	2	-0.045	0.059	4.448E-01	0.961	cis	SARS-CoV-2 infection
11187_11	CL12A	C-type lectin domain family 12 member A	Q5QGZ9	CLEC12A	chr12:9951316	Inverse variance weighted	6	-0.012	0.016	4.456E-01	0.961	cis	SARS-CoV-2 infection
19437_61	L-VEGF165	Isoform L-VEGF165	P15692	VEGFA	chr6:43770184	Inverse variance weighted	7	0.025	0.033	4.460E-01	0.961	cis	SARS-CoV-2 infection
16594_44	FAIM1	Fas apoptotic inhibitory molecule 1	Q9NVQ4	FAIM	chr3:138608606	Inverse variance weighted	7	0.012	0.015	4.461E-01	0.961	cis	SARS-CoV-2 infection
6521_35	NPTX2	Neuronal pentraxin-2	P47972	NPTX2	chr7:98617285	Inverse variance weighted	2	-0.043	0.056	4.463E-01	0.961	cis	SARS-CoV-2 infection
8005_1	MXRA7	Matrix-remodeling-associated protein 7	P84157	MXRA7	chr17:76711004	Inverse variance weighted	5	-0.015	0.020	4.471E-01	0.961	cis	SARS-CoV-2 infection
19143_38	NB5R2	NADH-cytochrome b5 reductase 2	Q6BCY4	CYB5R2	chr11:7677222	Inverse variance weighted	6	-0.026	0.035	4.481E-01	0.961	cis	SARS-CoV-2 infection
10672_75	SP-B	Pulmonary surfactant-associated protein B	P07988	SFTPB	chr2:85668741	Inverse variance weighted	4	-0.030	0.039	4.481E-01	0.961	cis	SARS-CoV-2 infection
10445_20	ApoM	Apolipoprotein M	O95445	APOM	chr6:31652416	Inverse variance weighted	2	0.021	0.028	4.484E-01	0.961	cis	SARS-CoV-2 infection
19238_12	GLNA	Glutamine synthetase	P15104	GLUL	chr1:182392206	Wald ratio	1	-0.073	0.097	4.498E-01	0.961	cis	SARS-CoV-2 infection
5092_51	JAG1:ECD	Protein jagged-1:Extracellular domain	P78504	JAG1	chr20:10673999	Inverse variance weighted	2	-0.045	0.060	4.501E-01	0.961	cis	SARS-CoV-2 infection
11287_14	Cytochrome b5	Cytochrome b5	P00167	CYB5A	chr18:74291973	Wald ratio	1	-0.087	0.116	4.507E-01	0.961	cis	SARS-CoV-2 infection
15596_7	HEXI1	Protein HEXIM1	O94992	HEXIM1	chr17:45148475	Wald ratio	1	-0.070	0.093	4.507E-01	0.961	cis	SARS-CoV-2 infection
9076_25	PENK	Proenkephalin-A	P01210	PENK	chr8:56446671	Inverse variance weighted	5	-0.007	0.009	4.509E-01	0.961	cis	SARS-CoV-2 infection
15559_5	ANTR2	Anthrax toxin receptor 2	P58335	ANTXR2	chr4:80125454	Inverse variance weighted	4	-0.027	0.036	4.522E-01	0.961	cis	SARS-CoV-2 infection
5657_28	SIA4A	CMP-N-acetylneuraminate-beta-galactosamide-alpha-2,3-sialyltransferase 1	Q11201	ST3GAL1	chr8:133571940	Inverse variance weighted	2	0.014	0.019	4.523E-01	0.961	cis	SARS-CoV-2 infection
4866_59	TrkB	BDNF/NT-3 growth factors receptor	Q16620	NTRK2	chr9:84668375	Wald ratio	1	0.057	0.076	4.533E-01	0.962	cis	SARS-CoV-2 infection
18458_4	PGES2	Prostaglandin E synthase 2	Q9H7Z7	PTGES2	chr9:128128462	Wald ratio	1	0.048	0.064	4.545E-01	0.963	cis	SARS-CoV-2 infection
3169_70	IDUA	Alpha-L-iduronidase	P35475	IDUA	chr4:986997	Inverse variance weighted	7	0.018	0.024	4.558E-01	0.963	cis	SARS-CoV-2 infection
2632_5	IL-12 Rb1	Interleukin-12 receptor subunit beta-1	P42701	IL12RB1	chr19:18098944	Wald ratio	1	-0.046	0.062	4.563E-01	0.963	cis	SARS-CoV-2 infection
13102_1	FAM3D	Protein FAM3D	Q96BQ1	FAM3D	chr3:58666834	Inverse variance weighted	4	0.011	0.015	4.565E-01	0.963	cis	SARS-CoV-2 infection
3309_2	FCG2A	Low affinity immunoglobulin gamma Fc region receptor II-a	P12318	FCGR2A	chr1:161505430	Inverse variance weighted	4	-0.018	0.024	4.570E-01	0.963	cis	SARS-CoV-2 infection
5108_72	Notch-3	Neurogenic locus notch homolog protein 3	Q9UM47	NOTCH3	chr19:15200995	Wald ratio	1	-0.043	0.058	4.574E-01	0.963	cis	SARS-CoV-2 infection
10620_21	PSP-94	Beta-microseminoprotein	P08118	MSMB	chr10:46048180	Inverse variance weighted	5	-0.004	0.006	4.590E-01	0.963	cis	SARS-CoV-2 infection
17357_33	SPCS	O-phosphoseryl-tRNA(Sec) selenium transferase	Q9HD40	SEPSECS	chr4:25160550	Wald ratio	1	-0.052	0.071	4.592E-01	0.963	cis	SARS-CoV-2 infection
18864_7	TRY3	Trypsin-3	P35030	PRSS3	chr9:33750679	Inverse variance weighted	4	-0.009	0.013	4.595E-01	0.963	cis	SARS-CoV-2 infection
3305_6	DLL4	Delta-like protein 4	Q9NR61	DLL4	chr15:40929340	Wald ratio	1	0.064	0.087	4.595E-01	0.963	cis	SARS-CoV-2 infection
4435_66	ENPP7	Ectonucleotide pyrophosphatase/phosphodiesterase family member 7	Q6UWV6	ENPP7	chr17:79730943	Inverse variance weighted	9	0.010	0.013	4.603E-01	0.964	cis	SARS-CoV-2 infection
2819_23	Cadherin-5	Cadherin-5	P33151	CDH5	chr16:66366622	Inverse variance weighted	2	0.023	0.031	4.614E-01	0.965	cis	SARS-CoV-2 infection
5124_69	sICAM-5	Intercellular adhesion molecule 5	Q9UMF0	ICAM5	chr19:10289952	Inverse variance weighted	4	-0.044	0.060	4.639E-01	0.966	cis	SARS-CoV-2 infection
8065_245	PATE4	Prostate and testis expressed protein 4	P0C8F1	PATE4	chr11:125833316	Wald ratio	1	-0.029	0.039	4.640E-01	0.966	cis	SARS-CoV-2 infection
12689_56	ARC1B	Actin-related protein 2/3 complex subunit 1B	O15143	ARPC1B	chr7:99374249	Wald ratio	1	0.040	0.055	4.645E-01	0.966	cis	SARS-CoV-2 infection
6617_12	FCRL6	Fc receptor-like protein 6	Q6DN72	FCRL6	chr1:159800511	Wald ratio	1	-0.029	0.039	4.649E-01	0.966	cis	SARS-CoV-2 infection
15444_45	SCCA2	Serpin B4	P48594	SERPINB4	chr18:63644256	Inverse variance weighted	3	0.030	0.041	4.651E-01	0.966	cis	SARS-CoV-2 infection
3343_1	Aminoacylase-1	Aminoacylase-1	Q03154	ACY1	chr3:51983340	Wald ratio	1	-0.021	0.029	4.677E-01	0.969	cis	SARS-CoV-2 infection
2631_50	IL-10 Rb	Interleukin-10 receptor subunit beta	Q08334	IL10RB	chr21:33266367	Wald ratio	1	0.050	0.068	4.678E-01	0.969	cis	SARS-CoV-2 infection
5744_12	CA056	Protein MENT	Q9BUN1	MENT	chr1:151047751	Inverse variance weighted	2	-0.098	0.136	4.681E-01	0.969	cis	SARS-CoV-2 infection
8958_51	CHL1	Neural cell adhesion molecule L1-like protein	O00533	CHL1	chr3:196763	Inverse variance weighted	6	0.012	0.017	4.688E-01	0.969	cis	SARS-CoV-2 infection
4874_3	Angiogenin	Angiogenin	P03950	ANG	chr14:20684177	Inverse variance weighted	5	0.022	0.030	4.705E-01	0.969	cis	SARS-CoV-2 infection
2778_10	IL-22	Interleukin-22	Q9GZX6	IL22	chr12:68253604	Wald ratio	1	-0.062	0.087	4.720E-01	0.969	cis	SARS-CoV-2 infection
5648_28	CTRB2	Chymotrypsinogen B2	Q6GPI1	CTRB2	chr16:75207161	Inverse variance weighted	8	-0.006	0.008	4.724E-01	0.969	cis	SARS-CoV-2 infection
8258_22	UXS1	UDP-glucuronic acid decarboxylase 1	Q8NBZ7	UXS1	chr2:106194301	Wald ratio	1	-0.047	0.065	4.730E-01	0.969	cis	SARS-CoV-2 infection
8309_12	HYAL1	Hyaluronidase-1	Q12794	HYAL1	chr3:50312381	Inverse variance weighted	2	-0.041	0.057	4.731E-01	0.969	cis	SARS-CoV-2 infection
4989_7	Fibrinogen g-chain dimer	Fibrinogen gamma chain	P02679	FGG	chr4:154612967	Wald ratio	1	-0.024	0.033	4.732E-01	0.969	cis	SARS-CoV-2 infection
3320_49	IGFBP-7	Insulin-like growth factor-binding protein 7	Q16270	IGFBP7	chr4:57110385	Inverse variance weighted	5	-0.017	0.023	4.732E-01	0.969	cis	SARS-CoV-2 infection
15511_37	NPTXR	Neuronal pentraxin receptor	O95502	NPTXR	chr22:38844028	Inverse variance weighted	7	-0.008	0.011	4.740E-01	0.969	cis	SARS-CoV-2 infection
15364_101	Apo C-I	Apolipoprotein C-I	P02654	APOC1	chr19:44914247	Inverse variance weighted	4	0.020	0.028	4.746E-01	0.969	cis	SARS-CoV-2 infection
15525_294	ADH1G	Alcohol dehydrogenase 1C	P00326	ADH1C	chr4:99352746	Wald ratio	1	0.026	0.036	4.746E-01	0.969	cis	SARS-CoV-2 infection
10940_25	SRCA	Sarcalumenin	Q86TD4	SRL	chr16:4242080	Wald ratio	1	0.046	0.064	4.752E-01	0.969	cis	SARS-CoV-2 infection
16300_4	TREM2	Triggering receptor expressed on myeloid cells 2	Q9NZC2	TREM2	chr6:41163186	Inverse variance weighted	7	0.009	0.013	4.776E-01	0.972	cis	SARS-CoV-2 infection
2654_19	TNF sR-I	Tumor necrosis factor receptor superfamily member 1A	P19438	TNFRSF1A	chr12:6342114	Wald ratio	1	0.031	0.043	4.781E-01	0.972	cis	SARS-CoV-2 infection
6556_5	ENPP5	Ectonucleotide pyrophosphatase/phosphodiesterase family member 5	Q9UJA9	ENPP5	chr6:46170980	Inverse variance weighted	7	-0.006	0.008	4.785E-01	0.972	cis	SARS-CoV-2 infection
17843_30	PPCS	Phosphopantothenate--cysteine ligase	Q9HAB8	PPCS	chr1:42456117	Inverse variance weighted	2	0.029	0.041	4.787E-01	0.972	cis	SARS-CoV-2 infection
6986_17	HS3SB	Heparan sulfate glucosamine 3-O-sulfotransferase 3B1	Q9Y662	HS3ST3B1	chr17:14301081	Wald ratio	1	-0.071	0.100	4.798E-01	0.972	cis	SARS-CoV-2 infection
8243_55	TATI	Serine protease inhibitor Kazal-type 1	P00995	SPINK1	chr5:147831671	Inverse variance weighted	2	-0.044	0.062	4.814E-01	0.972	cis	SARS-CoV-2 infection
18381_16	ALDH-E2	Aldehyde dehydrogenase, mitochondrial	P05091	ALDH2	chr12:111766887	Wald ratio	1	-0.045	0.063	4.818E-01	0.972	cis	SARS-CoV-2 infection
3046_31	resistin	Resistin	Q9HD89	RETN	chr19:7669049	Inverse variance weighted	6	0.011	0.015	4.820E-01	0.972	cis	SARS-CoV-2 infection
8296_117	KDEL2	KDEL motif-containing protein 2	Q7Z4H8	POGLUT3	chr11:108498405	Inverse variance weighted	6	0.009	0.013	4.820E-01	0.972	cis	SARS-CoV-2 infection
13534_20	MYOM2	Myomesin-2	P54296	MYOM2	chr8:2045046	Inverse variance weighted	3	0.043	0.061	4.824E-01	0.972	cis	SARS-CoV-2 infection
3516_60	SDF-1	Stromal cell-derived factor 1	P48061	CXCL12	chr10:44386493	Inverse variance weighted	2	0.022	0.032	4.835E-01	0.972	cis	SARS-CoV-2 infection
4908_6	Endoglin	Endoglin	P17813	ENG	chr9:127854658	Inverse variance weighted	3	0.014	0.021	4.841E-01	0.972	cis	SARS-CoV-2 infection
16914_104	sCD14	Monocyte differentiation antigen CD14, soluble	P08571	CD14	chr5:140633700	Inverse variance weighted	4	-0.030	0.042	4.852E-01	0.972	cis	SARS-CoV-2 infection
10080_9	EI2BA	Translation initiation factor eIF-2B subunit alpha	Q14232	EIF2B1	chr12:123633766	Wald ratio	1	0.063	0.090	4.856E-01	0.972	cis	SARS-CoV-2 infection
19488_1	PDCD6	Programmed cell death protein 6	O75340	PDCD6	chr5:271621	Inverse variance weighted	3	-0.024	0.034	4.858E-01	0.972	cis	SARS-CoV-2 infection
5708_1	LEAP2	Liver-expressed antimicrobial peptide 2	Q969E1	LEAP2	chr5:132873444	Inverse variance weighted	5	0.010	0.015	4.861E-01	0.972	cis	SARS-CoV-2 infection
4374_45	MIC-1	Growth/differentiation factor 15	Q99988	GDF15	chr19:18374731	Inverse variance weighted	7	0.009	0.012	4.862E-01	0.972	cis	SARS-CoV-2 infection
9234_8	TWSG1	Twisted gastrulation protein homolog 1	Q9GZX9	TWSG1	chr18:9334767	Inverse variance weighted	3	0.026	0.038	4.875E-01	0.973	cis	SARS-CoV-2 infection

3820_68	MAPK2	MAP kinase-activated protein kinase 2	P49137	MAPKAPK2	chr1:206684905	Wald ratio	1	-0.019	0.028	4.909E-01	0.978	cis	SARS-CoV-2 infection
18315_38	RTP4	Receptor-transporting protein 4	Q96DX8	RTP4	chr3:187368385	Inverse variance weighted	3	-0.010	0.014	4.913E-01	0.978	cis	SARS-CoV-2 infection
7161_25	G6PE	GDH/6PGL endoplasmic bifunctional protein	O95479	H6PD	chr1:9234774	Inverse variance weighted	9	-0.008	0.012	4.933E-01	0.978	cis	SARS-CoV-2 infection
15376_134	CATE	Cathepsin E	P14091	CTSE	chr1:206023909	Inverse variance weighted	4	-0.011	0.016	4.933E-01	0.978	cis	SARS-CoV-2 infection
12855_16	CT032	Cas scaffolding protein family member 4	Q9NQ75	CASS4	chr20:56412112	Wald ratio	1	-0.061	0.089	4.940E-01	0.978	cis	SARS-CoV-2 infection
8794_13	DPEP1	Dipeptidase 1	P16444	DPEP1	chr16:89613308	Inverse variance weighted	6	0.008	0.011	4.948E-01	0.978	cis	SARS-CoV-2 infection
5018_68	Peroxiredoxin-6	Peroxiredoxin-6	P30041	PRDX6	chr1:173477330	Wald ratio	1	-0.054	0.080	4.955E-01	0.978	cis	SARS-CoV-2 infection
19558_10	LRP4	Low-density lipoprotein receptor-related protein 4	O75096	LRP4	chr11:46918642	Inverse variance weighted	6	-0.016	0.024	4.963E-01	0.978	cis	SARS-CoV-2 infection
15522_2	GAPR1	Golgi-associated plant pathogenesis-related protein 1	Q9H4G4	GLIPR2	chr9:36136536	Inverse variance weighted	2	0.012	0.018	4.966E-01	0.978	cis	SARS-CoV-2 infection
12534_10	CACO2	Calcium-binding and coiled-coil domain-containing protein 2	Q13137	CALCOCO2	chr17:48831018	Wald ratio	1	-0.037	0.055	4.971E-01	0.978	cis	SARS-CoV-2 infection
3391_10	PK3CG	Phosphatidylinositol 4,5-bisphosphate 3-kinase catalytic subunit gamma isoform	P48736	PIK3CG	chr7:106865278	Wald ratio	1	0.064	0.095	4.976E-01	0.978	cis	SARS-CoV-2 infection
12408_333	RB22A	Ras-related protein Rab-22A	Q9UL26	RAB22A	chr20:58309715	Wald ratio	1	0.054	0.079	4.991E-01	0.978	cis	SARS-CoV-2 infection
9886_28	XRCC4	DNA repair protein XRCC4	Q13426	XRCC4	chr5:83077498	Wald ratio	1	0.031	0.047	5.007E-01	0.978	cis	SARS-CoV-2 infection
4911_49	Glutathione S-transferase Pi	Glutathione S-transferase P	P09211	GSTP1	chr11:67583742	Inverse variance weighted	2	-0.050	0.075	5.009E-01	0.978	cis	SARS-CoV-2 infection
17672_184	Gastric intrinsic factor	Gastric intrinsic factor	P27352	CBLIF	chr11:59845499	Inverse variance weighted	2	0.045	0.067	5.011E-01	0.978	cis	SARS-CoV-2 infection
15363_32	Apo A-V	Apolipoprotein A-V	Q6Q788	APOA5	chr11:116792420	Inverse variance weighted	8	-0.008	0.012	5.027E-01	0.978	cis	SARS-CoV-2 infection
19614_8	Holo-TC I	Transcobalamin-1	P20061	TCN1	chr11:59866489	Inverse variance weighted	5	0.009	0.013	5.051E-01	0.978	cis	SARS-CoV-2 infection
4145_58	Neurotrophin-3	Neurotrophin-3	P20783	NTF3	chr12:5432108	Inverse variance weighted	2	-0.036	0.054	5.055E-01	0.978	cis	SARS-CoV-2 infection
10561_5	PGRP-I-alpha	Peptidoglycan recognition protein 3	Q96LB9	PGLYRP3	chr1:153312952	Wald ratio	1	0.064	0.097	5.072E-01	0.978	cis	SARS-CoV-2 infection
5581_28	FGL1	Fibrinogen-like protein 1	Q08830	FGL1	chr8:17910365	Inverse variance weighted	7	0.008	0.012	5.078E-01	0.978	cis	SARS-CoV-2 infection
7110_2	DJB11	DnaJ homolog subfamily B member 11	Q9UBS4	DNAJB11	chr3:186567403	Inverse variance weighted	2	0.032	0.048	5.081E-01	0.978	cis	SARS-CoV-2 infection
10042_8	SGK3	Serine/threonine-protein kinase Sgk3	Q96BR1	SGK3	chr8:66712734	Wald ratio	1	-0.061	0.093	5.084E-01	0.978	cis	SARS-CoV-2 infection
5610_32	CJ038	Protein FAM171A1	Q5VUB5	FAM171A1	chr10:15371289	Inverse variance weighted	2	-0.044	0.067	5.100E-01	0.978	cis	SARS-CoV-2 infection
19289_29	DCUP	Uroporphyrinogen decarboxylase	P06132	UROD	chr1:45010950	Inverse variance weighted	3	0.012	0.019	5.104E-01	0.978	cis	SARS-CoV-2 infection
6930_95	SIA8F	Alpha-2,8-sialyltransferase 8F	P61647	ST8SIA6	chr10:17454595	Wald ratio	1	-0.064	0.098	5.113E-01	0.978	cis	SARS-CoV-2 infection
4240_31	M2-PK	Pyruvate kinase PKM	P14618	PKM	chr15:72231819	Wald ratio	1	0.050	0.076	5.119E-01	0.978	cis	SARS-CoV-2 infection
18307_71	PPase 2	Inorganic pyrophosphatase 2, mitochondrial	Q9H2U2	PPA2	chr4:105474067	Wald ratio	1	-0.036	0.055	5.120E-01	0.978	cis	SARS-CoV-2 infection
5091_28	ILT-4	Leukocyte immunoglobulin-like receptor subfamily B member 2	Q8N423	LILRB2	chr19:54281184	Inverse variance weighted	4	-0.026	0.040	5.131E-01	0.978	cis	SARS-CoV-2 infection
15576_158	ECP	Eosinophil cationic protein	P12724	RNASE3	chr14:20891385	Inverse variance weighted	5	-0.009	0.013	5.137E-01	0.978	cis	SARS-CoV-2 infection
11347_9	Transaldolase	Transaldolase	P37837	TALDO1	chr11:747415	Inverse variance weighted	2	-0.032	0.049	5.141E-01	0.978	cis	SARS-CoV-2 infection
18918_86	PDE4A	cAMP-specific 3',5'-cyclic phosphodiesterase 4A	P27815	PDE4A	chr19:10416773	Inverse variance weighted	4	0.020	0.030	5.161E-01	0.978	cis	SARS-CoV-2 infection
16561_9	Alpha-1B-glycoprotein	Alpha-1B-glycoprotein	P04217	A1BG	chr19:58353492	Inverse variance weighted	4	-0.009	0.014	5.170E-01	0.978	cis	SARS-CoV-2 infection
5703_26	NOE1	Noelin	Q99784	OLFM1	chr9:135075422	Inverse variance weighted	2	-0.027	0.042	5.172E-01	0.978	cis	SARS-CoV-2 infection
5676_54	ASIP	Agouti-signaling protein	P42127	ASIP	chr20:34194569	Wald ratio	1	0.009	0.013	5.179E-01	0.978	cis	SARS-CoV-2 infection
7251_64	C1QT3	Complement C1q tumor necrosis factor-related protein 3	Q9BXJ4	C1QTNF3	chr5:34043213	Inverse variance weighted	2	-0.023	0.035	5.181E-01	0.978	cis	SARS-CoV-2 infection
15333_11	SDF2	Stromal cell-derived factor 2	Q99470	SDF2	chr17:28662189	Wald ratio	1	0.040	0.062	5.182E-01	0.978	cis	SARS-CoV-2 infection
5349_69	DLL1	Delta-like protein 1	O00548	DLL1	chr6:170306565	Inverse variance weighted	2	0.055	0.085	5.183E-01	0.978	cis	SARS-CoV-2 infection
11265_8	Retinal dehydrogenase 1	Retinal dehydrogenase 1	P00352	ALDH1A1	chr9:73080442	Wald ratio	1	0.033	0.051	5.185E-01	0.978	cis	SARS-CoV-2 infection
8795_48	TR:ECD	Transferrin receptor protein 1:Extracellular domain	P02786	TFRC	chr3:196082153	Wald ratio	1	0.042	0.065	5.189E-01	0.978	cis	SARS-CoV-2 infection
5061_27	B7-H2	ICOS ligand	O75144	ICOSLG	chr21:44241446	Wald ratio	1	-0.052	0.081	5.199E-01	0.978	cis	SARS-CoV-2 infection
15636_49	SORC1	VPS10 domain-containing receptor SorCS1	Q8WY21	SORCS1	chr10:107164706	Wald ratio	1	0.047	0.073	5.211E-01	0.978	cis	SARS-CoV-2 infection
5105_2	Nogo Receptor	Reticulon-4 receptor	Q9BZR6	RTN4R	chr22:20283246	Inverse variance weighted	5	-0.009	0.014	5.213E-01	0.978	cis	SARS-CoV-2 infection
4188_1	Aflatoxin B1 aldehyde reductase	Aflatoxin B1 aldehyde reductase member 2	O43488	AKR7A2	chr1:19312144	Wald ratio	1	0.021	0.032	5.213E-01	0.978	cis	SARS-CoV-2 infection
6433_57	FA20A	Pseudokinase FAM20A	Q96MK3	FAM20A	chr17:68601367	Inverse variance weighted	3	-0.017	0.026	5.215E-01	0.978	cis	SARS-CoV-2 infection
16613_3	CAD17	Cadherin-17	Q12864	CDH17	chr8:94217303	Inverse variance weighted	5	0.010	0.015	5.217E-01	0.978	cis	SARS-CoV-2 infection
19251_56	Platelet proteoglycan	Serglycin	P10124	SRGN	chr10:69088103	Inverse variance weighted	5	-0.011	0.017	5.222E-01	0.978	cis	SARS-CoV-2 infection
17460_51	Mx1	Interferon-induced GTP-binding protein Mx1	P20591	MX1	chr21:41420020	Inverse variance weighted	4	-0.009	0.014	5.225E-01	0.978	cis	SARS-CoV-2 infection
7926_13	SPIT3	Kunitz-type protease inhibitor 3	P49223	SPINT3	chr20:45515622	Inverse variance weighted	4	0.027	0.042	5.227E-01	0.978	cis	SARS-CoV-2 infection
6402_8	PILRA isoform FDF03-deltaTM	Paired immunoglobulin-like type 2 receptor alpha isoform FDF03-deltaTM	Q9UKJ1	PILRA	chr7:100367530	Inverse variance weighted	2	0.010	0.016	5.235E-01	0.978	cis	SARS-CoV-2 infection
9251_28	MA2B2	Epididymis-specific alpha-mannosidase	Q9Y2E5	MAN2B2	chr4:6575189	Inverse variance weighted	9	0.007	0.011	5.236E-01	0.978	cis	SARS-CoV-2 infection
6998_106	HAAH	Aspartyl/asparaginyl beta-hydroxylase	Q12797	ASPH	chr8:61714640	Inverse variance weighted	2	-0.029	0.046	5.238E-01	0.978	cis	SARS-CoV-2 infection
15560_52	Apo-TC II	Transcobalamin-2	P20062	TCN2	chr22:30607003	Inverse variance weighted	4	0.007	0.011	5.240E-01	0.978	cis	SARS-CoV-2 infection
17814_8	BPNT1	3'(2'),5'-bisphosphate nucleotidase 1	O95861	BPNT1	chr1:220090462	Wald ratio	1	0.054	0.085	5.242E-01	0.978	cis	SARS-CoV-2 infection
5663_18	PF4V	Platelet factor 4 variant	P10720	PF4V1	chr4:73853296	Inverse variance weighted	2	0.075	0.118	5.251E-01	0.978	cis	SARS-CoV-2 infection
2948_58	Growth hormone receptor	Growth hormone receptor	P10912	GHR	chr5:42423439	Inverse variance weighted	4	0.014	0.022	5.261E-01	0.978	cis	SARS-CoV-2 infection
9883_29	Glyoxalase I	Lactoylglutathione lyase	Q04760	GLO1	chr6:38703145	Inverse variance weighted	3	-0.026	0.040	5.263E-01	0.978	cis	SARS-CoV-2 infection
10708_3	GON2	Progonadoliberin-2	O43555	GNRH2	chr20:3043622	Wald ratio	1	-0.029	0.047	5.266E-01	0.978	cis	SARS-CoV-2 infection
14134_49	AMGO2	Amphoterin-induced protein 2	Q86SJ2	AMIGO2	chr12:47079959	Inverse variance weighted	3	0.024	0.039	5.273E-01	0.978	cis	SARS-CoV-2 infection
5346_24	CPNE1:C2, 1 and 2	Copine-1:Ca2+-dependent membrane-targeting module domains 1 and 2	Q99829	CPNE1	chr20:35664956	Inverse variance weighted	9	0.005	0.009	5.275E-01	0.978	cis	SARS-CoV-2 infection
3518_54	TAFI	Carboxypeptidase B2	Q96IY4	CPB2	chr13:46105033	Inverse variance weighted	5	-0.008	0.012	5.283E-01	0.978	cis	SARS-CoV-2 infection
18899_82	HDGR3	Hepatoma-derived growth factor-related protein 3	Q9Y3E1	HDGFL3	chr15:83207823	Wald ratio	1	-0.031	0.050	5.290E-01	0.978	cis	SARS-CoV-2 infection
8100_15	ADM2	ADM2	Q7Z4H4	ADM2	chr22:50481543	Wald ratio	1	0.055	0.088	5.301E-01	0.978	cis	SARS-CoV-2 infection
11196_31	Collagen alpha-3(VI):BPTI/Kunitz inhibitor	Collagen alpha-3(VI) chain:Bovine pancreatic trypsin inhibitor/Kunitz inhibitor domain, isoform 1	P12111	COL6A3	chr2:237414328	Wald ratio	1	-0.045	0.072	5.315E-01	0.978	cis	SARS-CoV-2 infection
13124_20	ISLR2	Immunoglobulin superfamily containing leucine-rich repeat protein 2	Q6UXK2	ISLR2	chr15:74100311	Inverse variance weighted	4	-0.028	0.045	5.326E-01	0.978	cis	SARS-CoV-2 infection
8476_11	CgA	Chromogranin-A	P10645	CHGA	chr14:92923150	Inverse variance weighted	4	0.012	0.018	5.327E-01	0.978	cis	SARS-CoV-2 infection
15521_4	Alcadein alpha-1	Calsyntenin-1	O94985	CLSTN1	chr1:9823984	Inverse variance weighted	6	0.005	0.008	5.335E-01	0.978	cis	SARS-CoV-2 infection
12334_25	cSHMT	Serine hydroxymethyltransferase, cytosolic	P34896	SHMT1	chr17:18363563	Inverse variance weighted	6	0.008	0.014	5.347E-01	0.978	cis	SARS-CoV-2 infection
12556_7	UBE2C	Ubiquitin-conjugating enzyme E2 C	O00762	UBE2C	chr20:45812576	Inverse variance weighted	2	0.027	0.044	5.355E-01	0.978	cis	SARS-CoV-2 infection
6367_66	fibromodulin	fibromodulin	Q06828	FMOD	chr1:203351758	Inverse variance weighted	2	0.019	0.030	5.368E-01	0.978	cis	SARS-CoV-2 infection
9816_37	ISOC1	Isochorismatase domain-containing protein 1	Q96CN7	ISOC1	chr5:129094749	Inverse variance weighted	3	0.021	0.034	5.373E-01	0.978	cis	SARS-CoV-2 infection
10908_2	GLT13	Polypeptide N-acetylgalactosaminyltransferase 13	Q8IUC8	GALNT13	chr2:153871922	Wald ratio	1	-0.052	0.084	5.373E-01	0.978	cis	SARS-CoV-2 infection
4984_83	Esterase D	S-formylglutathione hydrolase	P10768	ESD	chr13:46797420	Inverse variance weighted	3	0.013	0.020	5.373E-01	0.978	cis	SARS-CoV-2 infection
7905_30	HPT	Haptoglobin isoform 2	P00738	HP	chr16:72054505	Wald ratio	1	-0.012	0.020	5.382E-01	0.978	cis	SARS-CoV-2 infection
17836_17	S100A16	Protein S100-A16	Q96FQ6	S100A16	chr1:153613145	Wald ratio	1	-0.042	0.068	5.385E-01	0.978	cis	SARS-CoV-2 infection
5090_49	ILT-2	Leukocyte immunoglobulin-like receptor subfamily B member 1	Q8NHL6	LILRB1	chr19:54617158	Inverse variance weighted	7	-0.009	0.015	5.393E-01	0.978	cis	SARS-CoV-2 infection
4249_64	NDP kinase B	Nucleoside diphosphate kinase B	P22392	NME2	chr17:51165435	Wald ratio	1	0.020	0.033	5.415E-01	0.978	cis	SARS-CoV-2 infection
12731_12	PKHA7	Pleckstrin homology domain-containing family A member 7	Q6IQ23	PLEKHA7	chr11:17014415	Inverse variance weighted	4	-0.008	0.013	5.421E-01	0.978	cis	SARS-CoV-2 infection
12450_42	PMVK	Phosphomevalonate kinase	Q15126	PMVK	chr1:154936719	Inverse variance weighted	2	0.094	0.154	5.422E-01	0.978	cis	SARS-CoV-2 infection

17758_79	DCXR	L-xylulose reductase	Q7Z4W1	DCXR	chr17:82037709	Inverse variance weighted	2	-0.040	0.066	5.425E-01	0.978	cis	SARS-CoV-2 infection
5131_15	TAJ	Tumor necrosis factor receptor superfamily member 19	Q9NS68	TNFRSF19	chr13:23570370	Wald ratio	1	-0.029	0.048	5.426E-01	0.978	cis	SARS-CoV-2 infection
14006_36	GNMT	Glycine N-methyltransferase	Q14749	GNMT	chr6:42960754	Inverse variance weighted	2	-0.023	0.038	5.431E-01	0.978	cis	SARS-CoV-2 infection
8923_94	GLTL1	Polypeptide N-acetylgalactosaminyltransferase 16	Q8N428	GALNT16	chr14:69259277	Inverse variance weighted	4	-0.019	0.032	5.443E-01	0.978	cis	SARS-CoV-2 infection
5601_2	PGRP-L	N-acetylmuramoyl-L-alanine amidase	Q96PD5	PGLYRP2	chr19:15498956	Inverse variance weighted	5	0.011	0.018	5.444E-01	0.978	cis	SARS-CoV-2 infection
8974_172	COFA1	Collagen alpha-1(XV) chain	P39059	COL15A1	chr9:98943179	Inverse variance weighted	4	0.012	0.020	5.449E-01	0.978	cis	SARS-CoV-2 infection
16610_13	LRP10	Low-density lipoprotein receptor-related protein 10	Q7Z4F1	LRP10	chr14:22871740	Inverse variance weighted	3	0.047	0.077	5.453E-01	0.978	cis	SARS-CoV-2 infection
9482_110	NUDT9	ADP-ribose pyrophosphatase, mitochondrial	Q9BW91	NUDT9	chr4:87422573	Wald ratio	1	-0.048	0.080	5.460E-01	0.978	cis	SARS-CoV-2 infection
17677_47	BY55	CD160 antigen	O95971	CD160	chr1:145719471	Wald ratio	1	-0.029	0.048	5.462E-01	0.978	cis	SARS-CoV-2 infection
9005_16	PLXA1	Plexin-A1	Q9UIW2	PLXNA1	chr3:126982693	Inverse variance weighted	3	0.012	0.019	5.470E-01	0.978	cis	SARS-CoV-2 infection
16074_12	GRB2-related adapter protein 2	GRB2-related adapter protein 2	O75791	GRAP2	chr22:39901084	Wald ratio	1	0.088	0.147	5.477E-01	0.978	cis	SARS-CoV-2 infection
2212_69	tPA	Tissue-type plasminogen activator	P00750	PLAT	chr8:42207709	Inverse variance weighted	4	-0.018	0.030	5.479E-01	0.978	cis	SARS-CoV-2 infection
9906_21	TICN3	Testican-3	Q9BQ16	SPOCK3	chr4:167234796	Inverse variance weighted	11	-0.006	0.011	5.480E-01	0.978	cis	SARS-CoV-2 infection
4332_6	CLC1B	C-type lectin domain family 1 member B	Q9P126	CLEC1B	chr12:10013424	Wald ratio	1	-0.041	0.068	5.483E-01	0.978	cis	SARS-CoV-2 infection
9525_1	PTK7	Inactive tyrosine-protein kinase 7	Q13308	PTK7	chr6:43076307	Inverse variance weighted	2	-0.041	0.068	5.490E-01	0.978	cis	SARS-CoV-2 infection
13624_17	NADK	NAD kinase	O95544	NADK	chr1:1780457	Inverse variance weighted	2	-0.023	0.038	5.495E-01	0.978	cis	SARS-CoV-2 infection
16892_23	ENPP2	Ectonucleotide pyrophosphatase/phosphodiesterase family member 2	Q13822	ENPP2	chr8:119673453	Inverse variance weighted	4	-0.020	0.034	5.500E-01	0.978	cis	SARS-CoV-2 infection
2879_9	a1-Antichymotrypsin	Alpha-1-antichymotrypsin	P01011	SERPINA3	chr14:94612384	Wald ratio	1	0.065	0.109	5.504E-01	0.978	cis	SARS-CoV-2 infection
15584_9	FHR2	Complement factor H-related protein 2	P36980	CFHR2	chr1:196943738	Inverse variance weighted	4	-0.008	0.014	5.506E-01	0.978	cis	SARS-CoV-2 infection
16055_3	complement factor H-related 5	Complement factor H-related protein 5	Q9BXR6	CFHR5	chr1:196975010	Inverse variance weighted	5	-0.009	0.015	5.518E-01	0.978	cis	SARS-CoV-2 infection
15300_66	CC134	Coiled-coil domain-containing protein 134	Q9H6E4	CCDC134	chr22:41800679	Inverse variance weighted	2	0.055	0.093	5.521E-01	0.978	cis	SARS-CoV-2 infection
6544_33	NELL1	Protein kinase C-binding protein NELL1	Q92832	NELL1	chr11:20669551	Inverse variance weighted	8	0.007	0.012	5.524E-01	0.978	cis	SARS-CoV-2 infection
5483_1	RGMA	Repulsive guidance molecule A	Q96886	RGMA	chr15:93089211	Inverse variance weighted	4	-0.015	0.025	5.527E-01	0.978	cis	SARS-CoV-2 infection
18881_7	CD97	CD97 antigen	P48960	ADGRE5	chr19:14380501	Inverse variance weighted	4	-0.022	0.036	5.531E-01	0.978	cis	SARS-CoV-2 infection
8458_111	a-Synuclein	Alpha-synuclein	P37840	SNCA	chr4:89838315	Wald ratio	1	-0.061	0.103	5.540E-01	0.978	cis	SARS-CoV-2 infection
8028_22	SPINK5	Serine protease inhibitor Kazal-type 5	Q9NQ38	SPINK5	chr5:148025683	Wald ratio	1	-0.021	0.036	5.543E-01	0.978	cis	SARS-CoV-2 infection
4961_17	annexin II	Annexin A2	P07355	ANXA2	chr15:60402883	Inverse variance weighted	2	0.036	0.061	5.545E-01	0.978	cis	SARS-CoV-2 infection
6425_87	MMP19	Matrix metalloproteinase-19	Q99542	MMP19	chr12:55842966	Inverse variance weighted	3	0.028	0.048	5.555E-01	0.978	cis	SARS-CoV-2 infection
17515_6	STCH	Heat shock 70 kDa protein 13	P48723	HSPA13	chr21:14383484	Wald ratio	1	0.029	0.049	5.565E-01	0.978	cis	SARS-CoV-2 infection
6520_87	MGP	Matrix Gla protein	P08493	MGP	chr12:14885857	Wald ratio	1	0.045	0.076	5.566E-01	0.978	cis	SARS-CoV-2 infection
11616_9	HSF1	Heat shock factor protein 1	Q00613	HSF1	chr8:144291591	Wald ratio	1	0.045	0.076	5.571E-01	0.978	cis	SARS-CoV-2 infection
9748_31	GSTM3-3	Glutathione S-transferase Mu 3	P21266	GSTM3	chr1:109741038	Inverse variance weighted	5	-0.010	0.017	5.577E-01	0.978	cis	SARS-CoV-2 infection
16616_137	ENOB	Beta-enolase	P13929	ENO3	chr17:4948092	Inverse variance weighted	2	0.014	0.024	5.581E-01	0.978	cis	SARS-CoV-2 infection
2658_27	TrkC	NT-3 growth factor receptor	Q16288	NTRK3	chr15:88256791	Inverse variance weighted	5	-0.014	0.024	5.585E-01	0.978	cis	SARS-CoV-2 infection
3796_79	ANGL4	Angiopoietin-related protein 4	Q9BY76	ANGPTL4	chr19:8363289	Wald ratio	1	-0.030	0.051	5.595E-01	0.978	cis	SARS-CoV-2 infection
3852_19	HSP 40	DnaJ homolog subfamily B member 1	P25685	DNAJB1	chr19:14560391	Wald ratio	1	-0.054	0.093	5.614E-01	0.978	cis	SARS-CoV-2 infection
12831_21	TESC	Calcineurin B homologous protein 3	Q96BS2	TESC	chr12:117099479	Inverse variance weighted	6	0.010	0.018	5.621E-01	0.978	cis	SARS-CoV-2 infection
11278_4	COL11A2	Collagen alpha-2(XI) chain	P13942	COL11A2	chr6:33192499	Inverse variance weighted	4	0.009	0.016	5.634E-01	0.978	cis	SARS-CoV-2 infection
6627_25	LIPR1	Inactive pancreatic lipase-related protein 1	P54315	PNLIPRP1	chr10:116590385	Inverse variance weighted	2	-0.018	0.031	5.635E-01	0.978	cis	SARS-CoV-2 infection
3045_72	PTN	Pleiotrophin	P21246	PTN	chr7:137343774	Inverse variance weighted	3	-0.015	0.026	5.638E-01	0.978	cis	SARS-CoV-2 infection
13622_16	2A5A	Serine/threonine-protein phosphatase 2A 56 kDa regulatory subunit alpha isoform	Q15172	PPP2R5A	chr1:212285410	Wald ratio	1	0.058	0.100	5.651E-01	0.978	cis	SARS-CoV-2 infection
5355_69	LIGHT	Tumor necrosis factor ligand superfamily member 14	O43557	TNFSF14	chr19:6670588	Wald ratio	1	-0.028	0.048	5.664E-01	0.978	cis	SARS-CoV-2 infection
18174_79	PDC6I	Programmed cell death 6-interacting protein	Q8WUM4	PDCD6IP	chr3:33798571	Wald ratio	1	-0.026	0.046	5.678E-01	0.978	cis	SARS-CoV-2 infection
13578_98	ABLM3	Actin-binding LIM protein 3	O94929	ABLIM3	chr5:149141483	Wald ratio	1	-0.027	0.047	5.691E-01	0.978	cis	SARS-CoV-2 infection
2972_57	BMP-7	Bone morphogenetic protein 7	P18075	BMP7	chr20:57266641	Wald ratio	1	0.050	0.088	5.697E-01	0.978	cis	SARS-CoV-2 infection
3331_8	RGMB	RGM domain family member B	Q6NW40	RGMB	chr5:98768650	Inverse variance weighted	3	-0.018	0.032	5.701E-01	0.978	cis	SARS-CoV-2 infection
2827_23	Fractalkine/CX3CL-1	Fractalkine	P78423	CX3CL1	chr16:57372477	Wald ratio	1	-0.019	0.033	5.702E-01	0.978	cis	SARS-CoV-2 infection
4906_35	Coagulation Factor V	Coagulation Factor V	P12259	F5	chr1:169586588	Inverse variance weighted	5	-0.009	0.015	5.705E-01	0.978	cis	SARS-CoV-2 infection
16621_77	AIBP	NAD(P)H-hydrate epimerase	Q8NCW5	NAXE	chr1:156591756	Wald ratio	1	0.013	0.024	5.707E-01	0.978	cis	SARS-CoV-2 infection
12662_82	ECH1	Delta(3,5)-Delta(2,4)-dienoyl-CoA isomerase, mitochondrial	Q13011	ECH1	chr19:38831841	Inverse variance weighted	2	-0.011	0.020	5.710E-01	0.978	cis	SARS-CoV-2 infection
3461_58	PGCB	Brevican core protein	Q96GW7	BCAN	chr1:156641390	Wald ratio	1	0.014	0.026	5.711E-01	0.978	cis	SARS-CoV-2 infection
18319_7	ODPX	Pyruvate dehydrogenase protein X component, mitochondrial	O00330	PDHX	chr11:34915829	Inverse variance weighted	2	0.023	0.041	5.723E-01	0.978	cis	SARS-CoV-2 infection
9931_20	Keratin-1	Keratin, type II cytoskeletal 1	P04264	KRT1	chr12:52680407	Wald ratio	1	-0.067	0.119	5.726E-01	0.978	cis	SARS-CoV-2 infection
10924_258	NEUFC	Neuferricin	Q8WUJ1	CYB5D2	chr17:4143168	Inverse variance weighted	3	-0.013	0.022	5.730E-01	0.978	cis	SARS-CoV-2 infection
7141_21	MGT4B	Alpha-1,3-mannosyl-glycoprotein 4-beta-N-acetylglucosaminyltransferase B	Q9UQ53	MGAT4B	chr5:179806952	Inverse variance weighted	4	-0.013	0.022	5.735E-01	0.978	cis	SARS-CoV-2 infection
5392_73	Fas, soluble	Tumor necrosis factor receptor superfamily member 6	P25445	FAS	chr10:88953813	Inverse variance weighted	2	0.028	0.049	5.741E-01	0.978	cis	SARS-CoV-2 infection
11126_102	TRIO	Triple functional domain protein	O75962	TRIO	chr5:14143342	Wald ratio	1	-0.059	0.106	5.756E-01	0.978	cis	SARS-CoV-2 infection
6409_57	GP116	Adhesion G protein-coupled receptor F5	Q8IZF2	ADGRF5	chr6:46954943	Inverse variance weighted	7	-0.005	0.008	5.766E-01	0.978	cis	SARS-CoV-2 infection
14747_9	CRLF1	Cytokine receptor-like factor 1	O75462	CRLF1	chr19:18607741	Inverse variance weighted	2	0.012	0.021	5.769E-01	0.978	cis	SARS-CoV-2 infection
4929_55	SHBG	Sex hormone-binding globulin	P04278	SHBG	chr17:7613946	Inverse variance weighted	2	-0.027	0.048	5.778E-01	0.978	cis	SARS-CoV-2 infection
5656_53	PP11	Poly(U)-specific endoribonuclease	P21128	ENDOU	chr12:47725567	Wald ratio	1	-0.045	0.080	5.780E-01	0.978	cis	SARS-CoV-2 infection
9826_135	Fragile histidine triad protein	Bis(5'-adenosyl)-triphosphatase	P49789	FHIT	chr3:61251459	Inverse variance weighted	2	0.032	0.057	5.789E-01	0.978	cis	SARS-CoV-2 infection
10666_7	GNPTG	N-acetylglucosamine-1-phosphotransferase subunit gamma	Q9UJJ9	GNPTG	chr16:1351931	Inverse variance weighted	4	0.017	0.031	5.791E-01	0.978	cis	SARS-CoV-2 infection
12859_33	PECI	Enoyl-CoA delta isomerase 2, mitochondrial	O75521	ECI2	chr6:4135597	Inverse variance weighted	3	0.010	0.018	5.792E-01	0.978	cis	SARS-CoV-2 infection
13700_10	annexin II	Annexin A2	P07355	ANXA2	chr15:60402883	Inverse variance weighted	6	-0.019	0.034	5.807E-01	0.978	cis	SARS-CoV-2 infection
2475_1	SCF sR	Mast/stem cell growth factor receptor Kit	P10721	KIT	chr4:54657267	Wald ratio	1	-0.055	0.099	5.809E-01	0.978	cis	SARS-CoV-2 infection
17737_7	IVD	Isovaleryl-CoA dehydrogenase, mitochondrial	P26440	IVD	chr15:40405485	Inverse variance weighted	2	0.019	0.035	5.811E-01	0.978	cis	SARS-CoV-2 infection
6455_52	HPLN4	Hyaluronan and proteoglycan link protein 4	Q86UW8	HAPLN4	chr19:19262804	Wald ratio	1	-0.038	0.069	5.819E-01	0.978	cis	SARS-CoV-2 infection
16057_6	IGF-II receptor	Cation-independent mannose-6-phosphate receptor	P11717	IGF2R	chr6:159969082	Inverse variance weighted	6	0.006	0.011	5.820E-01	0.978	cis	SARS-CoV-2 infection
6897_38	B3GA3	Galactosylgalactosylxylosylprotein 3-beta-glucuronosyltransferase 3	O94766	B3GAT3	chr11:62622154	Inverse variance weighted	3	-0.012	0.022	5.821E-01	0.978	cis	SARS-CoV-2 infection
3313_21	FCN2	Ficolin-2	Q15485	FCN2	chr9:134880810	Inverse variance weighted	4	0.024	0.043	5.824E-01	0.978	cis	SARS-CoV-2 infection
6383_90	TLL1	Tolloid-like protein 1	O43897	TLL1	chr4:165873237	Inverse variance weighted	2	0.036	0.066	5.840E-01	0.978	cis	SARS-CoV-2 infection
9026_40	BTNL8	Butyrophilin-like protein 8	Q6UX41	BTNL8	chr5:180899077	Inverse variance weighted	2	0.016	0.029	5.841E-01	0.978	cis	SARS-CoV-2 infection
8244_16	FUT8	Alpha-(1,6)-fucosyltransferase	Q9BYC5	FUT8	chr14:65410592	Inverse variance weighted	10	0.006	0.012	5.843E-01	0.978	cis	SARS-CoV-2 infection
17722_5	FKBP52 protein	Peptidyl-prolyl cis-trans isomerase FKBP4	Q02790	FKBP4	chr12:2794970	Inverse variance weighted	2	0.035	0.064	5.852E-01	0.978	cis	SARS-CoV-2 infection
19262_219	ACADV	Very long-chain specific acyl-CoA dehydrogenase, mitochondrial	P49748	ACADVL	chr17:7217125	Wald ratio	1	-0.053	0.097	5.858E-01	0.978	cis	SARS-CoV-2 infection
4151_6	Plasminogen	Plasminogen	P00747	PLG	chr6:160702194	Inverse variance weighted	3	0.023	0.042	5.859E-01	0.978	cis	SARS-CoV-2 infection
18225_13	HEBP1	Heme-binding protein 1	Q9NRV9	HEBP1	chr12:13000265	Inverse variance weighted	6	-0.005	0.008	5.860E-01	0.978	cis	SARS-CoV-2 infection

9326_33	ITI heavy chain H2	Inter-alpha-trypsin inhibitor heavy chain H2	P19823	ITIH2	chr10:7703316	Inverse variance weighted	9	-0.006	0.010	5.868E-01	0.978	cis	SARS-CoV-2 infection
4297_62	Spondin-1	Spondin-1	Q9HCB6	SPON1	chr11:13962723	Inverse variance weighted	4	0.009	0.016	5.872E-01	0.978	cis	SARS-CoV-2 infection
15607_56	KPYR	Pyruvate kinase PKLR	P30613	PKLR	chr1:155301438	Wald ratio	1	-0.021	0.039	5.884E-01	0.978	cis	SARS-CoV-2 infection
17325_10	KGUA	Guanylate kinase	Q16774	GUK1	chr1:228139962	Inverse variance weighted	5	0.029	0.054	5.892E-01	0.978	cis	SARS-CoV-2 infection
11178_21	SVEP1:EGF-like domains 4-6	Sushi, von Willebrand factor type A, EGF and pentraxin domain-containing protein 1:EGF-like domains 4-6	Q4LDE5	SVEP1	chr9:110579880	Inverse variance weighted	4	0.023	0.042	5.898E-01	0.978	cis	SARS-CoV-2 infection
8300_82	PEX14:N-term	Peroxisomal membrane protein PEX14:N-term	O75381	PEX14	chr1:10472288	Wald ratio	1	0.050	0.093	5.902E-01	0.978	cis	SARS-CoV-2 infection
19197_95	THIL	Acetyl-CoA acetyltransferase, mitochondrial	P24752	ACAT1	chr11:108116695	Inverse variance weighted	2	0.032	0.059	5.910E-01	0.978	cis	SARS-CoV-2 infection
4534_10	BSSP4	Brain-specific serine protease 4	Q9GZN4	PRSS22	chr16:2858170	Inverse variance weighted	4	-0.017	0.031	5.910E-01	0.978	cis	SARS-CoV-2 infection
15483_377	Agrin	Agrin	O00468	AGRN	chr1:1020120	Inverse variance weighted	7	-0.020	0.038	5.915E-01	0.978	cis	SARS-CoV-2 infection
2864_2	MEK1	Dual specificity mitogen-activated protein kinase kinase 1	Q02750	MAP2K1	chr15:66386837	Wald ratio	1	0.041	0.077	5.922E-01	0.978	cis	SARS-CoV-2 infection
5630_48	CM35H	CMRF35-like molecule 8	Q9UGN4	CD300A	chr17:74466399	Inverse variance weighted	8	-0.007	0.014	5.930E-01	0.978	cis	SARS-CoV-2 infection
9288_7	FKBP7	Peptidyl-prolyl cis-trans isomerase FKBP7	Q9Y680	FKBP7	chr2:178478600	Inverse variance weighted	4	0.009	0.016	5.931E-01	0.978	cis	SARS-CoV-2 infection
2780_35	Lactoferrin	Lactotransferrin	P02788	LTF	chr3:46485234	Inverse variance weighted	3	-0.014	0.026	5.943E-01	0.978	cis	SARS-CoV-2 infection
11531_24	GI24:CD	V-type immunoglobulin domain-containing suppressor of T-cell activation:Cytoplasmic domain	Q9H7M9	VSIR	chr10:71773520	Wald ratio	1	-0.043	0.080	5.959E-01	0.978	cis	SARS-CoV-2 infection
10833_64	HHIP	Hedgehog-interacting protein	Q96QV1	HHIP	chr4:144646156	Inverse variance weighted	2	-0.017	0.033	5.978E-01	0.978	cis	SARS-CoV-2 infection
3795_6	ADAM 9	Disintegrin and metalloproteinase domain-containing protein 9	Q13443	ADAM9	chr8:38996754	Wald ratio	1	0.066	0.126	5.993E-01	0.978	cis	SARS-CoV-2 infection
2913_1	MPIF-1	C-C motif chemokine 23	P55773	CCL23	chr17:36017972	Inverse variance weighted	2	0.022	0.043	5.993E-01	0.978	cis	SARS-CoV-2 infection
8269_327	ARSK	Arylsulfatase K	Q6UWY0	ARSK	chr5:95555101	Wald ratio	1	0.031	0.059	5.993E-01	0.978	cis	SARS-CoV-2 infection
5644_60	RNAS4	Ribonuclease 4	P34096	RNASE4	chr14:20684560	Inverse variance weighted	5	-0.010	0.019	6.007E-01	0.978	cis	SARS-CoV-2 infection
6496_60	DLK1:ECD	Protein delta homolog 1:Extracellular domain	P80370	DLK1	chr14:100725705	Inverse variance weighted	2	0.035	0.066	6.033E-01	0.978	cis	SARS-CoV-2 infection
13450_49	UBP8	Ubiquitin carboxyl-terminal hydrolase 8	P40818	USP8	chr15:50424380	Wald ratio	1	-0.033	0.064	6.033E-01	0.978	cis	SARS-CoV-2 infection
8877_22	F176C:ECD	Protein eva-1 homolog C:Extracellular domain	P58658	EVA1C	chr21:32412006	Wald ratio	1	-0.040	0.077	6.034E-01	0.978	cis	SARS-CoV-2 infection
9595_11	B4GT2	Beta-1,4-galactosyltransferase 2	O60909	B4GALT2	chr1:43978943	Inverse variance weighted	4	0.009	0.018	6.037E-01	0.978	cis	SARS-CoV-2 infection
13068_139	CCS	Copper chaperone for superoxide dismutase	O14618	CCS	chr11:66593153	Wald ratio	1	-0.021	0.040	6.057E-01	0.978	cis	SARS-CoV-2 infection
2813_11	ART	Agouti-related protein	O00253	AGRP	chr16:67483547	Inverse variance weighted	2	0.013	0.026	6.058E-01	0.978	cis	SARS-CoV-2 infection
6255_74	CPXM1	Probable carboxypeptidase X1	Q96SM3	CPXM1	chr20:2800627	Inverse variance weighted	6	-0.008	0.016	6.059E-01	0.978	cis	SARS-CoV-2 infection
19233_75	ATOX1	Copper transport protein ATOX1	O00244	ATOX1	chr5:151772532	Wald ratio	1	-0.047	0.091	6.059E-01	0.978	cis	SARS-CoV-2 infection
12682_5	KAT3	Kynurenine--oxoglutarate transaminase 3	Q6YP21	KYAT3	chr1:88992953	Inverse variance weighted	7	0.008	0.015	6.068E-01	0.978	cis	SARS-CoV-2 infection
14711_27	Cystatin M	Cystatin-M	Q15828	CST6	chr11:66012008	Wald ratio	1	0.024	0.047	6.072E-01	0.978	cis	SARS-CoV-2 infection
2665_26	BCMA	Tumor necrosis factor receptor superfamily member 17	Q02223	TNFRSF17	chr16:11965210	Wald ratio	1	-0.019	0.037	6.078E-01	0.978	cis	SARS-CoV-2 infection
3336_50	TFPI	Tissue factor pathway inhibitor	P10646	TFPI	chr2:187565760	Wald ratio	1	-0.032	0.062	6.087E-01	0.978	cis	SARS-CoV-2 infection
6525_17	DUSP13	Dual specificity protein phosphatase 13 isoform A	Q6B8I1	DUSP13	chr10:75109221	Inverse variance weighted	2	0.011	0.022	6.087E-01	0.978	cis	SARS-CoV-2 infection
2571_12	IGFBP-3	Insulin-like growth factor-binding protein 3	P17936	IGFBP3	chr7:45921874	Inverse variance weighted	4	0.008	0.016	6.095E-01	0.978	cis	SARS-CoV-2 infection
4127_75	C6	Complement component C6	P13671	C6	chr5:41261438	Inverse variance weighted	4	-0.017	0.034	6.098E-01	0.978	cis	SARS-CoV-2 infection
13632_10	Zyxin	Zyxin	Q15942	ZYX	chr7:143381295	Wald ratio	1	-0.051	0.099	6.101E-01	0.978	cis	SARS-CoV-2 infection
8403_18	Fatty acid synthase	Fatty acid synthase	P49327	FASN	chr17:82098294	Wald ratio	1	-0.017	0.034	6.102E-01	0.978	cis	SARS-CoV-2 infection
15633_6	RBP	Retinol-binding protein 4	P02753	RBP4	chr10:93601744	Wald ratio	1	0.031	0.060	6.104E-01	0.978	cis	SARS-CoV-2 infection
6485_59	IGLL1	Immunoglobulin lambda-like polypeptide 1	P15814	IGLL1	chr22:23580302	Inverse variance weighted	4	-0.007	0.013	6.110E-01	0.978	cis	SARS-CoV-2 infection
4467_49	SPARCL1	SPARC-like protein 1	Q14515	SPARCL1	chr4:87531061	Inverse variance weighted	6	-0.009	0.018	6.122E-01	0.978	cis	SARS-CoV-2 infection
12016_60	CBL	E3 ubiquitin-protein ligase CBL	P22681	CBL	chr11:119206298	Wald ratio	1	0.018	0.036	6.130E-01	0.978	cis	SARS-CoV-2 infection
12008_3	CD7	T-cell antigen CD7	P09564	CD7	chr17:82317608	Inverse variance weighted	5	-0.037	0.074	6.133E-01	0.978	cis	SARS-CoV-2 infection
3605_77	MASP3:Light	Mannan-binding lectin serine protease 1:Mannan-binding lectin serine protease 1 light chain	P48740	MASP1	chr3:187291980	Wald ratio	1	0.030	0.059	6.153E-01	0.978	cis	SARS-CoV-2 infection
18216_22	IL-11 RA	Interleukin-11 receptor subunit alpha	Q14626	IL11RA	chr9:34652162	Inverse variance weighted	3	0.020	0.040	6.157E-01	0.978	cis	SARS-CoV-2 infection
18916_25	Inosine triphosphatase	Inosine triphosphate pyrophosphatase	Q9BY32	ITPA	chr20:3208868	Inverse variance weighted	8	-0.005	0.010	6.163E-01	0.978	cis	SARS-CoV-2 infection
5478_50	PSMA	Glutamate carboxypeptidase 2	Q04609	FOLH1	chr11:49208638	Inverse variance weighted	2	-0.014	0.029	6.168E-01	0.978	cis	SARS-CoV-2 infection
6081_52	PCOC2	Procollagen C-endopeptidase enhancer 2	Q9UKZ9	PCOLCE2	chr3:142889206	Inverse variance weighted	10	-0.005	0.010	6.180E-01	0.978	cis	SARS-CoV-2 infection
17435_43	ETFA	Electron transfer flavoprotein subunit alpha, mitochondrial	P13804	ETFA	chr15:76311730	Wald ratio	1	-0.045	0.091	6.182E-01	0.978	cis	SARS-CoV-2 infection
13112_179	FSTL1	Follistatin-related protein 1	Q12841	FSTL1	chr3:120450993	Inverse variance weighted	2	0.014	0.028	6.189E-01	0.978	cis	SARS-CoV-2 infection
3074_6	LBP	Lipopolysaccharide-binding protein	P18428	LBP	chr20:38346482	Inverse variance weighted	5	0.009	0.019	6.193E-01	0.978	cis	SARS-CoV-2 infection
19277_4	KAT	Thiosulfate sulfurtransferase/rhodanese-like domain-containing protein 1	Q8NFU3	TSTD1	chr1:161038977	Inverse variance weighted	2	0.016	0.032	6.205E-01	0.978	cis	SARS-CoV-2 infection
2637_77	Macrophage mannose receptor	Macrophage mannose receptor 1	P22897	MRC1	chr10:17809348	Inverse variance weighted	7	-0.012	0.024	6.215E-01	0.978	cis	SARS-CoV-2 infection
4154_57	P-Selectin	P-selectin	P16109	SELP	chr1:169630193	Inverse variance weighted	3	-0.015	0.030	6.215E-01	0.978	cis	SARS-CoV-2 infection
6472_40	GP100	Melanocyte protein PMEL	P40967	PMEL	chr12:55973317	Inverse variance weighted	2	-0.010	0.020	6.224E-01	0.978	cis	SARS-CoV-2 infection
12612_37	PSB1	Proteasome subunit beta type-1	P20618	PSMB1	chr6:170553307	Inverse variance weighted	2	0.007	0.015	6.228E-01	0.978	cis	SARS-CoV-2 infection
9839_148	Tirap	Toll/interleukin-1 receptor domain-containing adapter protein	P58753	TIRAP	chr11:126282497	Inverse variance weighted	3	-0.020	0.042	6.242E-01	0.978	cis	SARS-CoV-2 infection
2515_14	GFRa-2	GDNF family receptor alpha-2	O00451	GFRA2	chr8:21812357	Inverse variance weighted	6	-0.008	0.015	6.244E-01	0.978	cis	SARS-CoV-2 infection
17766_5	NCF-1	Neutrophil cytosol factor 1	P14598	NCF1	chr7:74774011	Inverse variance weighted	4	-0.010	0.020	6.246E-01	0.978	cis	SARS-CoV-2 infection
4469_78	ST4S6	Carbohydrate sulfotransferase 15	Q7LFX5	CHST15	chr10:124093598	Inverse variance weighted	2	0.020	0.041	6.251E-01	0.978	cis	SARS-CoV-2 infection
3474_19	Thrombospondin-1	Thrombospondin-1	P07996	THBS1	chr15:39581079	Wald ratio	1	0.048	0.098	6.266E-01	0.978	cis	SARS-CoV-2 infection
10462_14	INSL5	Insulin-like peptide INSL5	Q9Y5Q6	INSL5	chr1:66801276	Wald ratio	1	-0.040	0.083	6.273E-01	0.978	cis	SARS-CoV-2 infection
19635_69	FGL2	Fibroleukin	Q14314	FGL2	chr7:77199848	Wald ratio	1	0.042	0.087	6.278E-01	0.978	cis	SARS-CoV-2 infection
13983_27	QOR	Quinone oxidoreductase	Q08257	CRYZ	chr1:74733408	Inverse variance weighted	7	0.006	0.012	6.279E-01	0.978	cis	SARS-CoV-2 infection
5223_59	GCKR	Glucokinase regulatory protein	Q14397	GCKR	chr2:27496839	Wald ratio	1	-0.026	0.053	6.283E-01	0.978	cis	SARS-CoV-2 infection
7156_2	FUT10	Alpha-(1,3)-fucosyltransferase 10	Q6P4F1	FUT10	chr8:33473146	Inverse variance weighted	7	-0.005	0.010	6.283E-01	0.978	cis	SARS-CoV-2 infection
13947_371	NUD12	Peroxisomal NADH pyrophosphatase NUDT12	Q9BQG2	NUDT12	chr5:103562790	Inverse variance weighted	4	-0.010	0.020	6.287E-01	0.978	cis	SARS-CoV-2 infection
4978_54	DBNL	Drebrin-like protein	Q9UJU6	DBNL	chr7:44044640	Wald ratio	1	0.040	0.083	6.288E-01	0.978	cis	SARS-CoV-2 infection
3059_50	BAFF	Tumor necrosis factor ligand superfamily member 13B	Q9Y275	TNFSF13B	chr13:108251240	Inverse variance weighted	3	-0.025	0.051	6.316E-01	0.978	cis	SARS-CoV-2 infection
19581_15	IGFBP-5	Insulin-like growth factor-binding protein 5	P24593	IGFBP5	chr2:216695549	Wald ratio	1	-0.044	0.093	6.326E-01	0.978	cis	SARS-CoV-2 infection
17794_6	Phosphomannomutase 2	Phosphomannomutase 2	O15305	PMM2	chr16:8788823	Inverse variance weighted	4	0.013	0.027	6.328E-01	0.978	cis	SARS-CoV-2 infection
4455_89	MFGM	Lactadherin	Q08431	MFGE8	chr15:88913468	Inverse variance weighted	4	-0.009	0.018	6.339E-01	0.978	cis	SARS-CoV-2 infection
5480_49	RANTES	C-C motif chemokine 5	P13501	CCL5	chr17:35880793	Inverse variance weighted	2	0.023	0.048	6.342E-01	0.978	cis	SARS-CoV-2 infection
17210_2	TCL1A	T-cell leukemia/lymphoma protein 1A	P56279	TCL1A	chr14:95714196	Inverse variance weighted	3	-0.011	0.024	6.347E-01	0.978	cis	SARS-CoV-2 infection
5102_55	MICB	MHC class I polypeptide-related sequence B	Q29980	MICB	chr6:31494881	Inverse variance weighted	6	0.008	0.016	6.359E-01	0.978	cis	SARS-CoV-2 infection
6392_7	WISP-2	WNT1-inducible-signaling pathway protein 2	O76076	CCN5	chr20:44714844	Inverse variance weighted	5	-0.008	0.017	6.359E-01	0.978	cis	SARS-CoV-2 infection
18172_71	ASF1A	Histone chaperone ASF1A	Q9Y294	ASF1A	chr6:118894152	Wald ratio	1	0.022	0.046	6.373E-01	0.978	cis	SARS-CoV-2 infection
13986_6	LANC1	LanC-like protein 1	O43813	LANCL1	chr2:210477652	Inverse variance weighted	2	-0.021	0.045	6.374E-01	0.978	cis	SARS-CoV-2 infection
6507_16	NCAM2	Neural cell adhesion molecule 2	O15394	NCAM2	chr21:20998409	Inverse variance weighted	6	0.006	0.012	6.386E-01	0.978	cis	SARS-CoV-2 infection
9484_75	Desmoglein-2	Desmoglein-2	Q14126	DSG2	chr18:31498177	Inverse variance weighted	5	-0.015	0.032	6.390E-01	0.978	cis	SARS-CoV-2 infection

17320_19	ADPPT	L-aminoadipate-semialdehyde dehydrogenase-phosphopantetheinyl transferase	Q9NRN7	AASDHPPT	chr11:106075501	Wald ratio	1	0.051	0.109	6.393E-01	0.978	cis	SARS-CoV-2 infection
12361_102	RRAS2	Ras-related protein R-Ras2	P62070	RRAS2	chr11:14364506	Wald ratio	1	-0.018	0.039	6.419E-01	0.978	cis	SARS-CoV-2 infection
15556_49	Alpha-amylase 2B	Alpha-amylase 2B	P19961	AMY2B	chr1:103553815	Inverse variance weighted	4	0.024	0.053	6.427E-01	0.978	cis	SARS-CoV-2 infection
10772_21	CGAT2	Chondroitin sulfate N-acetylgalactosaminyltransferase 2	Q8N6G5	CSGALNACT2	chr10:43138445	Wald ratio	1	-0.023	0.051	6.433E-01	0.978	cis	SARS-CoV-2 infection
9867_23	F16P2	Fructose-1,6-bisphosphatase isozyme 2	O00757	FBP2	chr9:94593824	Wald ratio	1	-0.023	0.049	6.434E-01	0.978	cis	SARS-CoV-2 infection
3181_50	Cathepsin S	Cathepsin S	P25774	CTSS	chr1:150765957	Inverse variance weighted	3	-0.006	0.013	6.437E-01	0.978	cis	SARS-CoV-2 infection
2681_23	HGF	Hepatocyte growth factor	P14210	HGF	chr7:81770438	Wald ratio	1	-0.020	0.044	6.439E-01	0.978	cis	SARS-CoV-2 infection
12812_25	ACYP2	Acylphosphatase-2	P14621	ACYP2	chr2:53970838	Inverse variance weighted	3	0.010	0.021	6.443E-01	0.978	cis	SARS-CoV-2 infection
2797_56	Apo B	Apolipoprotein B	P04114	APOB	chr2:21044073	Wald ratio	1	0.031	0.067	6.444E-01	0.978	cis	SARS-CoV-2 infection
5012_67	Myokinase, human	Adenylate kinase isoenzyme 1	P00568	AK1	chr9:127877675	Wald ratio	1	-0.052	0.113	6.448E-01	0.978	cis	SARS-CoV-2 infection
16851_50	SCO2	Protein SCO2 homolog, mitochondrial	O43819	SCO2	chr22:50526461	Wald ratio	1	0.023	0.050	6.463E-01	0.978	cis	SARS-CoV-2 infection
15336_7	SELM	Selenoprotein M	Q8WWX9	SELENOM	chr22:31120069	Wald ratio	1	0.043	0.094	6.469E-01	0.978	cis	SARS-CoV-2 infection
17391_10	SPF45	Splicing factor 45	Q96I25	RBM17	chr10:6089034	Inverse variance weighted	2	0.011	0.023	6.473E-01	0.978	cis	SARS-CoV-2 infection
10511_10	Collagen alpha-3(VI):isoform 3	Collagen alpha-3(VI) chain:isoform 3	P12111	COL6A3	chr2:237414328	Inverse variance weighted	2	-0.018	0.040	6.481E-01	0.978	cis	SARS-CoV-2 infection
8428_102	NTRI	Neurotrimin	Q9P121	NTM	chr11:131370478	Inverse variance weighted	4	-0.019	0.042	6.483E-01	0.978	cis	SARS-CoV-2 infection
6039_24	CRHBP	Corticotropin-releasing factor-binding protein	P24387	CRHBP	chr5:76953045	Inverse variance weighted	6	-0.006	0.014	6.485E-01	0.978	cis	SARS-CoV-2 infection
10391_1	ANGL3	Angiopoietin-related protein 3	Q9Y5C1	ANGPTL3	chr1:62597520	Inverse variance weighted	7	-0.006	0.013	6.489E-01	0.978	cis	SARS-CoV-2 infection
3329_14	PGRP-S	Peptidoglycan recognition protein 1	O75594	PGLYRP1	chr19:46023053	Inverse variance weighted	4	-0.015	0.033	6.490E-01	0.978	cis	SARS-CoV-2 infection
8225_86	EPHB2	Ephrin type-B receptor 2	P29323	EPHB2	chr1:22710839	Inverse variance weighted	3	0.013	0.029	6.491E-01	0.978	cis	SARS-CoV-2 infection
18215_5	THG1	Probable tRNA(His) guanylyltransferase	Q9NWX6	THG1L	chr5:157731420	Inverse variance weighted	3	-0.009	0.019	6.494E-01	0.978	cis	SARS-CoV-2 infection
6626_81	CHSTC	Carbohydrate sulfotransferase 12	Q9NRB3	CHST12	chr7:2403588	Inverse variance weighted	2	-0.012	0.027	6.503E-01	0.978	cis	SARS-CoV-2 infection
6572_10	LRRT4:ECD	Leucine-rich repeat transmembrane neuronal protein 4:Isoform 2, Extracellular domain	Q86VH4	LRRTM4	chr2:77593319	Wald ratio	1	0.046	0.101	6.508E-01	0.978	cis	SARS-CoV-2 infection
5509_7	EGF:ECD	Epidermal growth factor:Extracellular domain	P01133	EGF	chr4:109912883	Inverse variance weighted	4	-0.018	0.040	6.512E-01	0.978	cis	SARS-CoV-2 infection
6252_62	Secretoglobin family 3A member 1	Secretoglobin family 3A member 1	Q96QR1	SCGB3A1	chr5:180591499	Inverse variance weighted	4	-0.010	0.023	6.517E-01	0.978	cis	SARS-CoV-2 infection
6385_63	VWA1	von Willebrand factor A domain-containing protein 1	Q6PCB0	VWA1	chr1:1434861	Inverse variance weighted	4	0.007	0.015	6.519E-01	0.978	cis	SARS-CoV-2 infection
4234_8	IL-1 R4	Interleukin-1 receptor-like 1	Q01638	IL1RL1	chr2:102311502	Inverse variance weighted	8	0.005	0.012	6.526E-01	0.978	cis	SARS-CoV-2 infection
18882_7	CSTN2	Calsyntenin-2	Q9H4D0	CLSTN2	chr3:139935185	Inverse variance weighted	7	-0.007	0.016	6.532E-01	0.978	cis	SARS-CoV-2 infection
11117_2	SPT20	Spermatogenesis-associated protein 20	Q8TB22	SPATA20	chr17:50543058	Inverse variance weighted	4	-0.014	0.031	6.544E-01	0.978	cis	SARS-CoV-2 infection
12486_8	GLRX2	Glutaredoxin-2, mitochondrial	Q9NS18	GLRX2	chr1:193106114	Inverse variance weighted	3	-0.014	0.031	6.548E-01	0.978	cis	SARS-CoV-2 infection
18343_10	DECR2	Peroxisomal 2,4-dienoyl-CoA reductase	Q9NUI1	DECR2	chr16:401858	Inverse variance weighted	5	-0.012	0.027	6.550E-01	0.978	cis	SARS-CoV-2 infection
17776_15	PECR	Peroxisomal trans-2-enoyl-CoA reductase	Q9BY49	PECR	chr2:216082955	Wald ratio	1	0.023	0.052	6.553E-01	0.978	cis	SARS-CoV-2 infection
16049_43	OLR1	Oxidized low-density lipoprotein receptor 1	P78380	OLR1	chr12:10172138	Wald ratio	1	0.026	0.059	6.561E-01	0.978	cis	SARS-CoV-2 infection
18871_24	AIF1L	Allograft inflammatory factor 1-like	Q9BQI0	AIF1L	chr9:131096476	Inverse variance weighted	5	0.005	0.012	6.568E-01	0.978	cis	SARS-CoV-2 infection
15462_28	CD8A	T-cell surface glycoprotein CD8 alpha chain	P01732	CD8A	chr2:86808396	Inverse variance weighted	2	-0.007	0.016	6.581E-01	0.978	cis	SARS-CoV-2 infection
8766_29	LIRA5	Leukocyte immunoglobulin-like receptor subfamily A member 5	A6NI73	LILRA5	chr19:54313166	Wald ratio	1	-0.037	0.083	6.581E-01	0.978	cis	SARS-CoV-2 infection
16309_30	Soggy-1	Dickkopf-like protein 1	Q9UK85	DKKL1	chr19:49361783	Wald ratio	1	0.047	0.106	6.585E-01	0.978	cis	SARS-CoV-2 infection
9231_23	IMPA3	Inositol monophosphatase 3	Q9NX62	BPNT2	chr8:56993867	Inverse variance weighted	6	0.006	0.013	6.590E-01	0.978	cis	SARS-CoV-2 infection
11288_26	5NTC	Cytosolic purine 5'-nucleotidase	P49902	NT5C2	chr10:103277605	Inverse variance weighted	3	-0.014	0.032	6.592E-01	0.978	cis	SARS-CoV-2 infection
2609_59	Cystatin C	Cystatin-C	P01034	CST3	chr20:23638473	Inverse variance weighted	2	0.021	0.049	6.602E-01	0.978	cis	SARS-CoV-2 infection
12786_61	GDE5	Glycerophosphocholine phosphodiesterase GPCPD1	Q9NPB8	GPCPD1	chr20:5611006	Inverse variance weighted	2	-0.027	0.061	6.609E-01	0.978	cis	SARS-CoV-2 infection
3431_54	EphA1	Ephrin type-A receptor 1	P21709	EPHA1	chr7:143408856	Inverse variance weighted	7	0.004	0.010	6.625E-01	0.978	cis	SARS-CoV-2 infection
3727_35	PYY	Peptide YY	P10082	PYY	chr17:44004469	Wald ratio	1	0.029	0.066	6.629E-01	0.978	cis	SARS-CoV-2 infection
16803_4	CALB2	Calretinin	P22676	CALB2	chr16:71358713	Inverse variance weighted	2	0.034	0.078	6.630E-01	0.978	cis	SARS-CoV-2 infection
6576_1	ART4	Ecto-ADP-ribosyltransferase 4	Q93070	ART4	chr12:14843526	Inverse variance weighted	12	-0.004	0.009	6.633E-01	0.978	cis	SARS-CoV-2 infection
5837_49	LIF sR	Leukemia inhibitory factor receptor	P42702	LIFR	chr5:38608354	Inverse variance weighted	4	-0.012	0.028	6.636E-01	0.978	cis	SARS-CoV-2 infection
12386_11	AMPB	Aminopeptidase B	Q9H4A4	RNPEP	chr1:201982372	Inverse variance weighted	5	-0.006	0.015	6.637E-01	0.978	cis	SARS-CoV-2 infection
7266_4	SPA9	Serpin A9	Q86WD7	SERPINA9	chr14:94479689	Inverse variance weighted	2	0.023	0.054	6.660E-01	0.978	cis	SARS-CoV-2 infection
3007_7	Siglec-9	Sialic acid-binding Ig-like lectin 9	Q9Y336	SIGLEC9	chr19:51124906	Inverse variance weighted	6	-0.008	0.018	6.660E-01	0.978	cis	SARS-CoV-2 infection
9416_77	CBPM	Carboxypeptidase M	P14384	CPM	chr12:68971570	Wald ratio	1	-0.022	0.050	6.662E-01	0.978	cis	SARS-CoV-2 infection
15468_14	FHR1	Complement factor H-related protein 1	Q03591	CFHR1	chr1:196819731	Inverse variance weighted	6	0.006	0.014	6.673E-01	0.978	cis	SARS-CoV-2 infection
11098_1	PDXK	Pyridoxal kinase	O00764	PDXK	chr21:43719094	Inverse variance weighted	6	-0.012	0.028	6.673E-01	0.978	cis	SARS-CoV-2 infection
11109_56	SVEP1:Sushi 15-18	Sushi, von Willebrand factor type A, EGF and pentraxin domain-containing protein 1:Sushi 15-18	Q4LDE5	SVEP1	chr9:110579880	Inverse variance weighted	4	0.022	0.052	6.698E-01	0.978	cis	SARS-CoV-2 infection
13397_88	HBD-2	Beta-defensin 4A	O15263	DEFB4A	chr8:7894677	Inverse variance weighted	3	0.015	0.036	6.708E-01	0.978	cis	SARS-CoV-2 infection
15491_20	CD248	Endosialin	Q9HCU0	CD248	chr11:66317044	Inverse variance weighted	2	0.042	0.098	6.709E-01	0.978	cis	SARS-CoV-2 infection
12975_11	Keratin 20	Keratin, type I cytoskeletal 20	P35900	KRT20	chr17:40885242	Inverse variance weighted	2	-0.029	0.068	6.709E-01	0.978	cis	SARS-CoV-2 infection
18880_81	Collagen Type III	Collagen Type III	P02461	COL3A1	chr2:188974373	Inverse variance weighted	2	-0.029	0.069	6.712E-01	0.978	cis	SARS-CoV-2 infection
17739_1	HCDH	Hydroxyacyl-coenzyme A dehydrogenase, mitochondrial	Q16836	HADH	chr4:107989714	Wald ratio	1	-0.030	0.071	6.721E-01	0.978	cis	SARS-CoV-2 infection
3554_24	Adiponectin	Adiponectin	Q15848	ADIPOQ	chr3:186842704	Inverse variance weighted	4	-0.009	0.022	6.721E-01	0.978	cis	SARS-CoV-2 infection
13642_90	CIG49	Interferon-induced protein with tetratricopeptide repeats 3	O14879	IFIT3	chr10:89327307	Wald ratio	1	-0.025	0.059	6.725E-01	0.978	cis	SARS-CoV-2 infection
3077_66	Coagulation Factor Xa	Coagulation factor Xa	P00742	F10	chr13:113122799	Inverse variance weighted	2	-0.030	0.070	6.731E-01	0.978	cis	SARS-CoV-2 infection
19273_3	Glutathione reductase	Glutathione reductase, mitochondrial	P00390	GSR	chr8:30727846	Inverse variance weighted	3	-0.030	0.070	6.732E-01	0.978	cis	SARS-CoV-2 infection
15535_3	Marapsin	Serine protease 27	Q9BQR3	PRSS27	chr16:2720551	Wald ratio	1	0.013	0.031	6.734E-01	0.978	cis	SARS-CoV-2 infection
12764_3	ELMO1	Engulfment and cell motility protein 1	Q92556	ELMO1	chr7:37449223	Inverse variance weighted	3	-0.024	0.057	6.740E-01	0.978	cis	SARS-CoV-2 infection
12851_5	DPOLM	DNA-directed DNA/RNA polymerase mu	Q9NP87	POLM	chr7:44082530	Inverse variance weighted	3	0.012	0.028	6.747E-01	0.978	cis	SARS-CoV-2 infection
13697_51	GPDA	Glycerol-3-phosphate dehydrogenase [NAD(+)], cytoplasmic	P21695	GPD1	chr12:50103982	Wald ratio	1	0.027	0.065	6.748E-01	0.978	cis	SARS-CoV-2 infection
7784_1	Kininogen, HMW	Kininogen-1	P01042	KNG1	chr3:186717348	Inverse variance weighted	2	-0.029	0.069	6.753E-01	0.978	cis	SARS-CoV-2 infection
11212_7	TXND5	Thioredoxin domain-containing protein 5	Q8NBS9	TXNDC5	chr6:7910788	Inverse variance weighted	6	0.008	0.020	6.755E-01	0.978	cis	SARS-CoV-2 infection
5798_3	BID	BH3-interacting domain death agonist	P55957	BID	chr22:17774770	Wald ratio	1	0.027	0.065	6.770E-01	0.978	cis	SARS-CoV-2 infection
9191_8	Trefoil factor 2	Trefoil factor 2	Q03403	TFF2	chr21:42350997	Inverse variance weighted	2	-0.020	0.047	6.771E-01	0.978	cis	SARS-CoV-2 infection
3283_21	BGH3	Transforming growth factor-beta-induced protein ig-h3	Q15582	TGFBI	chr5:136028988	Inverse variance weighted	5	0.008	0.019	6.797E-01	0.978	cis	SARS-CoV-2 infection
4129_72	Factor B	Complement factor B	P00751	CFB	chr6:31945650	Inverse variance weighted	3	-0.024	0.059	6.800E-01	0.978	cis	SARS-CoV-2 infection
15542_19	KCRU	Creatine kinase U-type, mitochondrial	P12532	CKMT1A	chr15:43692886	Wald ratio	1	0.023	0.056	6.822E-01	0.978	cis	SARS-CoV-2 infection
15579_26	ENPP6	Ectonucleotide pyrophosphatase/phosphodiesterase family member 6	Q6UWR7	ENPP6	chr4:184221230	Wald ratio	1	-0.037	0.091	6.828E-01	0.978	cis	SARS-CoV-2 infection
6557_50	LRC15	Leucine-rich repeat-containing protein 15	Q8TF66	LRRC15	chr3:194369743	Inverse variance weighted	4	0.006	0.015	6.853E-01	0.978	cis	SARS-CoV-2 infection
3293_2	CD5L	CD5 antigen-like	O43866	CD5L	chr1:157898256	Inverse variance weighted	4	0.011	0.028	6.857E-01	0.978	cis	SARS-CoV-2 infection
11387_3	ATF6B	Cyclic AMP-dependent transcription factor ATF-6 beta	Q99941	ATF6B	chr6:32128253	Inverse variance weighted	3	-0.019	0.046	6.890E-01	0.978	cis	SARS-CoV-2 infection
18181_2	TSSC4	Protein TSSC4	Q9Y5U2	TSSC4	chr11:2400488	Wald ratio	1	0.049	0.123	6.892E-01	0.978	cis	SARS-CoV-2 infection
8786_6	F171B:ECD	Protein FAM171B:Extracellular domain	Q6P995	FAM171B	chr2:186694060	Wald ratio	1	-0.013	0.032	6.913E-01	0.978	cis	SARS-CoV-2 infection

18413_24	ARL4D	ADP-ribosylation factor-like protein 4D	P49703	ARL4D	chr17:43398993	Wald ratio	1	-0.035	0.089	6.915E-01	0.978	cis	SARS-CoV-2 infection
3041_55	MRC2	C-type mannose receptor 2	Q9UBG0	MRC2	chr17:62627670	Inverse variance weighted	6	-0.017	0.043	6.920E-01	0.978	cis	SARS-CoV-2 infection
14106_46	GDP/GTP exchange protein	Rap1 GTPase-GDP dissociation stimulator 1	P52306	RAP1GDS1	chr4:98261384	Wald ratio	1	-0.038	0.097	6.927E-01	0.978	cis	SARS-CoV-2 infection
12641_3	I5P2	Type II inositol 1,4,5-trisphosphate 5-phosphatase	P32019	INPP5B	chr1:37947057	Inverse variance weighted	2	0.012	0.031	6.927E-01	0.978	cis	SARS-CoV-2 infection
5363_51	Semaphorin 3E	Semaphorin-3E	O15041	SEMA3E	chr7:83649139	Inverse variance weighted	10	0.004	0.010	6.934E-01	0.978	cis	SARS-CoV-2 infection
10396_6	Mcl-1	Induced myeloid leukemia cell differentiation protein Mcl-1	Q07820	MCL1	chr1:150579738	Wald ratio	1	0.015	0.039	6.940E-01	0.978	cis	SARS-CoV-2 infection
10781_19	CLC4G	C-type lectin domain family 4 member G	Q6UXB4	CLEC4G	chr19:7733906	Wald ratio	1	0.044	0.112	6.949E-01	0.978	cis	SARS-CoV-2 infection
4962_52	ARMEL	Cerebral dopamine neurotrophic factor	Q49AH0	CDNF	chr10:14838575	Inverse variance weighted	6	0.009	0.022	6.955E-01	0.978	cis	SARS-CoV-2 infection
5301_7	Eotaxin	Eotaxin	P51671	CCL11	chr17:34285742	Wald ratio	1	0.021	0.053	6.958E-01	0.978	cis	SARS-CoV-2 infection
8479_4	MMP-10	Stromelysin-2	P09238	MMP10	chr11:102780628	Inverse variance weighted	6	0.008	0.020	6.959E-01	0.978	cis	SARS-CoV-2 infection
3004_67	PD-L2	Programmed cell death 1 ligand 2	Q9BQ51	PDCD1LG2	chr9:5510531	Inverse variance weighted	5	-0.008	0.021	6.965E-01	0.978	cis	SARS-CoV-2 infection
3366_51	ECM1	Extracellular matrix protein 1	Q16610	ECM1	chr1:150508062	Inverse variance weighted	12	-0.004	0.011	6.971E-01	0.978	cis	SARS-CoV-2 infection
3459_49	PDGF Rb	Platelet-derived growth factor receptor beta	P09619	PDGFRB	chr5:150155872	Inverse variance weighted	11	-0.002	0.005	6.979E-01	0.978	cis	SARS-CoV-2 infection
11214_40	DNJB9	DnaJ homolog subfamily B member 9	Q9UBS3	DNAJB9	chr7:108569867	Wald ratio	1	-0.042	0.109	6.981E-01	0.978	cis	SARS-CoV-2 infection
7856_51	F151A	Protein FAM151A	Q8WW52	FAM151A	chr1:54623556	Inverse variance weighted	5	-0.007	0.018	7.002E-01	0.978	cis	SARS-CoV-2 infection
8874_53	CLN5:LD	Ceroid-lipofuscinosis neuronal protein 5:Lumenal domain	O75503	CLN5	chr13:76990660	Wald ratio	1	0.019	0.050	7.005E-01	0.978	cis	SARS-CoV-2 infection
19377_14	NOE2	Noelin-2	O95897	OLFM2	chr19:9936515	Inverse variance weighted	6	-0.005	0.013	7.013E-01	0.978	cis	SARS-CoV-2 infection
6444_15	PSG3	Pregnancy-specific beta-1-glycoprotein 3	Q16557	PSG3	chr19:42740481	Inverse variance weighted	8	0.008	0.020	7.018E-01	0.978	cis	SARS-CoV-2 infection
15339_32	COF2	Cofilin-2	Q9Y281	CFL2	chr14:34714823	Wald ratio	1	-0.016	0.042	7.021E-01	0.978	cis	SARS-CoV-2 infection
11431_235	RECQ1	ATP-dependent DNA helicase Q1	P46063	RECQL	chr12:21501669	Inverse variance weighted	7	0.003	0.009	7.025E-01	0.978	cis	SARS-CoV-2 infection
8303_102	MYG1	UPF0160 protein MYG1, mitochondrial	Q9HB07	MYG1	chr12:53299695	Wald ratio	1	-0.025	0.066	7.030E-01	0.978	cis	SARS-CoV-2 infection
4480_59	C3b	Complement C3b	P01024	C3	chr19:6730562	Wald ratio	1	-0.042	0.110	7.030E-01	0.978	cis	SARS-CoV-2 infection
16307_22	UNC5H4	Netrin receptor UNC5D	Q6UXZ4	UNC5D	chr8:35235475	Inverse variance weighted	3	0.013	0.035	7.032E-01	0.978	cis	SARS-CoV-2 infection
9297_12	B3GN8	UDP-GlcNAc:betaGal beta-1,3-N-acetylglucosaminyltransferase 8	Q7Z7M8	B3GNT8	chr19:41428730	Inverse variance weighted	6	0.004	0.010	7.036E-01	0.978	cis	SARS-CoV-2 infection
7999_23	CD39	Ectonucleoside triphosphate diphosphohydrolase 1	P49961	ENTPD1	chr10:95711779	Inverse variance weighted	3	-0.011	0.028	7.038E-01	0.978	cis	SARS-CoV-2 infection
10339_48	NSE	Gamma-enolase	P09104	ENO2	chr12:6913745	Wald ratio	1	0.016	0.043	7.047E-01	0.978	cis	SARS-CoV-2 infection
8327_26	DPEP2	Dipeptidase 2	Q9H4A9	DPEP2	chr16:68000586	Inverse variance weighted	2	0.012	0.032	7.048E-01	0.978	cis	SARS-CoV-2 infection
6990_44	SDF2L	Stromal cell-derived factor 2-like protein 1	Q9HCN8	SDF2L1	chr22:21642302	Wald ratio	1	-0.035	0.094	7.060E-01	0.978	cis	SARS-CoV-2 infection
11696_7	RABP2	Cellular retinoic acid-binding protein 2	P29373	CRABP2	chr1:156705816	Inverse variance weighted	3	-0.006	0.017	7.076E-01	0.978	cis	SARS-CoV-2 infection
15305_7	Secretagogin	Secretagogin	O76038	SCGN	chr6:25652201	Inverse variance weighted	2	-0.025	0.066	7.078E-01	0.978	cis	SARS-CoV-2 infection
9829_91	SULT 2A1	Bile salt sulfotransferase	Q06520	SULT2A1	chr19:47886315	Wald ratio	1	-0.011	0.030	7.080E-01	0.978	cis	SARS-CoV-2 infection
5134_52	TIMD3	Hepatitis A virus cellular receptor 2	Q8TDQ0	HAVCR2	chr5:157142869	Inverse variance weighted	7	0.005	0.013	7.093E-01	0.978	cis	SARS-CoV-2 infection
3389_7	PCI	Plasma serine protease inhibitor	P05154	SERPINA5	chr14:94561442	Inverse variance weighted	2	-0.025	0.066	7.093E-01	0.978	cis	SARS-CoV-2 infection
8866_53	QPCTL	Glutaminyl-peptide cyclotransferase-like protein	Q9NXS2	QPCTL	chr19:45692403	Inverse variance weighted	6	0.006	0.016	7.094E-01	0.978	cis	SARS-CoV-2 infection
14088_38	IGFBP-6	Insulin-like growth factor-binding protein 6	P24592	IGFBP6	chr12:53097436	Wald ratio	1	-0.037	0.099	7.095E-01	0.978	cis	SARS-CoV-2 infection
3009_3	TGF-b R III	Transforming growth factor beta receptor type 3	Q03167	TGFBR3	chr1:91906335	Inverse variance weighted	2	-0.017	0.045	7.097E-01	0.978	cis	SARS-CoV-2 infection
18878_15	GREM1	Gremlin-1	O60565	GREM1	chr15:32718004	Inverse variance weighted	8	-0.003	0.009	7.114E-01	0.978	cis	SARS-CoV-2 infection
16781_2	ENASE	Cytosolic endo-beta-N-acetylglucosaminidase	Q8NFI3	ENGASE	chr17:79074824	Inverse variance weighted	9	-0.003	0.009	7.121E-01	0.978	cis	SARS-CoV-2 infection
10892_8	OSMR	Oncostatin-M-specific receptor subunit beta	Q99650	OSMR	chr5:38845858	Inverse variance weighted	2	0.036	0.098	7.126E-01	0.978	cis	SARS-CoV-2 infection
13126_52	DSC2	Desmocollin-2	Q02487	DSC2	chr18:31102522	Inverse variance weighted	4	-0.011	0.031	7.136E-01	0.978	cis	SARS-CoV-2 infection
16609_106	KIRR2	Kin of IRRE-like protein 2	Q6UWL6	KIRREL2	chr19:35855861	Inverse variance weighted	6	0.006	0.017	7.145E-01	0.978	cis	SARS-CoV-2 infection
10490_3	RPN1:CD	Dolichyl-diphosphooligosaccharide--protein glycosyltransferase subunit 1:Cytoplasmic domain	P04843	RPN1	chr3:128681075	Wald ratio	1	-0.011	0.031	7.145E-01	0.978	cis	SARS-CoV-2 infection
9398_30	GALP	Galanin-like peptide	Q9UBC7	GALP	chr19:56176008	Wald ratio	1	-0.030	0.083	7.166E-01	0.978	cis	SARS-CoV-2 infection
13434_172	PARVA	Alpha-parvin	Q9NVD7	PARVA	chr11:12377563	Wald ratio	1	-0.022	0.060	7.167E-01	0.978	cis	SARS-CoV-2 infection
19154_41	Protease nexin I	Glia-derived nexin	P07093	SERPINE2	chr2:224039318	Inverse variance weighted	8	-0.004	0.011	7.175E-01	0.978	cis	SARS-CoV-2 infection
11313_100	PHS	Pterin-4-alpha-carbinolamine dehydratase	P61457	PCBD1	chr10:70888565	Inverse variance weighted	5	0.011	0.032	7.189E-01	0.978	cis	SARS-CoV-2 infection
5456_59	CNDP1	Beta-Ala-His dipeptidase	Q96KN2	CNDP1	chr18:74534500	Inverse variance weighted	6	-0.007	0.018	7.189E-01	0.978	cis	SARS-CoV-2 infection
8909_77	GNPI1	Glucosamine-6-phosphate isomerase 1	P46926	GNPDA1	chr5:142013041	Wald ratio	1	0.013	0.037	7.195E-01	0.978	cis	SARS-CoV-2 infection
13488_3	ARFP1	Arfaptin-1	P53367	ARFIP1	chr4:152779937	Inverse variance weighted	3	-0.014	0.039	7.202E-01	0.978	cis	SARS-CoV-2 infection
3499_77	IL-17B	Interleukin-17B	Q9UHF5	IL17B	chr5:149404202	Wald ratio	1	-0.025	0.070	7.207E-01	0.978	cis	SARS-CoV-2 infection
19173_5	ZFAN1	AN1-type zinc finger protein 1	Q8TCF1	ZFAND1	chr8:81732903	Inverse variance weighted	3	0.018	0.050	7.215E-01	0.978	cis	SARS-CoV-2 infection
19338_3	LZIC	Protein LZIC	Q8WZA0	LZIC	chr1:9943407	Wald ratio	1	-0.038	0.106	7.216E-01	0.978	cis	SARS-CoV-2 infection
10554_23	BGAL	Beta-galactosidase	P16278	GLB1	chr3:33097202	Wald ratio	1	0.039	0.109	7.224E-01	0.978	cis	SARS-CoV-2 infection
7945_10	Semaphorin-6A	Semaphorin-6A	Q9H2E6	SEMA6A	chr5:116574823	Inverse variance weighted	4	0.015	0.043	7.227E-01	0.978	cis	SARS-CoV-2 infection
4407_10	MSP	Hepatocyte growth factor-like protein	P26927	MST1	chr3:49689501	Inverse variance weighted	4	0.002	0.006	7.227E-01	0.978	cis	SARS-CoV-2 infection
11516_7	FABPL	Fatty acid-binding protein, liver	P07148	FABP1	chr2:88128062	Wald ratio	1	-0.016	0.044	7.231E-01	0.978	cis	SARS-CoV-2 infection
4990_87	GP1BA	Platelet glycoprotein Ib alpha chain	P07359	GP1BA	chr17:4932277	Inverse variance weighted	3	-0.011	0.030	7.232E-01	0.978	cis	SARS-CoV-2 infection
2962_50	PTHrP	Parathyroid hormone-related protein	P12272	PTHLH	chr12:27972733	Inverse variance weighted	3	0.012	0.035	7.243E-01	0.978	cis	SARS-CoV-2 infection
8970_9	RIPK2	Receptor-interacting serine/threonine-protein kinase 2	O43353	RIPK2	chr8:89757806	Wald ratio	1	0.029	0.083	7.251E-01	0.978	cis	SARS-CoV-2 infection
15587_20	FOLR2	Folate receptor beta	P14207	FOLR2	chr11:72216601	Wald ratio	1	-0.018	0.051	7.257E-01	0.978	cis	SARS-CoV-2 infection
18206_18	ADH6	Alcohol dehydrogenase 6	P28332	ADH6	chr4:99219537	Wald ratio	1	-0.026	0.075	7.261E-01	0.978	cis	SARS-CoV-2 infection
12436_84	GST omega-1	Glutathione S-transferase omega-1	P78417	GSTO1	chr10:104235356	Inverse variance weighted	8	-0.004	0.012	7.271E-01	0.978	cis	SARS-CoV-2 infection
15304_1	PAP1	Regenerating islet-derived protein 3-alpha	Q06141	REG3A	chr2:79159753	Inverse variance weighted	3	-0.020	0.057	7.272E-01	0.978	cis	SARS-CoV-2 infection
2944_66	DAN	Neuroblastoma suppressor of tumorigenicity 1	P41271	NBL1	chr1:19596979	Wald ratio	1	0.032	0.092	7.272E-01	0.978	cis	SARS-CoV-2 infection
3298_52	Contactin-4	Contactin-4	Q8IWV2	CNTN4	chr3:2098813	Inverse variance weighted	8	-0.005	0.013	7.273E-01	0.978	cis	SARS-CoV-2 infection
2567_5	Factor I	Complement factor I	P05156	CFI	chr4:109802150	Inverse variance weighted	2	-0.008	0.023	7.274E-01	0.978	cis	SARS-CoV-2 infection
6366_38	TXD15	Thioredoxin domain-containing protein 15	Q96J42	TXNDC15	chr5:134874371	Inverse variance weighted	3	0.005	0.014	7.279E-01	0.978	cis	SARS-CoV-2 infection
12727_7	FPRP	Prostaglandin F2 receptor negative regulator	Q9P2B2	PTGFRN	chr1:116909916	Inverse variance weighted	8	-0.005	0.014	7.287E-01	0.978	cis	SARS-CoV-2 infection
18236_3	LEGL	Galectin-related protein	Q3ZCW2	LGALSL	chr2:64453969	Wald ratio	1	-0.022	0.064	7.291E-01	0.978	cis	SARS-CoV-2 infection
3025_50	bFGF	Fibroblast growth factor 2	P09038	FGF2	chr4:122826682	Inverse variance weighted	6	0.005	0.014	7.299E-01	0.978	cis	SARS-CoV-2 infection
13094_75	RSPO3	R-spondin-3	Q9BXY4	RSPO3	chr6:127118671	Wald ratio	1	-0.026	0.076	7.305E-01	0.978	cis	SARS-CoV-2 infection
2973_15	CD36 ANTIGEN	Platelet glycoprotein 4	P16671	CD36	chr7:80369575	Inverse variance weighted	4	-0.008	0.023	7.311E-01	0.978	cis	SARS-CoV-2 infection
2888_49	C7	Complement component C7	P10643	C7	chr5:40909492	Inverse variance weighted	5	-0.003	0.008	7.321E-01	0.978	cis	SARS-CoV-2 infection
18380_78	Albumin	Serum albumin	P02768	ALB	chr4:73397114	Wald ratio	1	0.027	0.078	7.323E-01	0.978	cis	SARS-CoV-2 infection
15589_1	Gc-Globulin, Mixed Type	Vitamin D-binding protein	P02774	GC	chr4:71804041	Inverse variance weighted	2	-0.012	0.036	7.324E-01	0.978	cis	SARS-CoV-2 infection
8982_65	TSP3	Thrombospondin-3	P49746	THBS3	chr1:155209051	Inverse variance weighted	3	-0.037	0.107	7.328E-01	0.978	cis	SARS-CoV-2 infection
4969_2	Carbonic anhydrase I	Carbonic anhydrase 1	P00915	CA1	chr8:85379014	Inverse variance weighted	3	-0.015	0.045	7.338E-01	0.978	cis	SARS-CoV-2 infection
19153_53	MPPD2	Metallophosphoesterase MPPED2	Q15777	MPPED2	chr11:30586872	Inverse variance weighted	2	0.033	0.096	7.340E-01	0.978	cis	SARS-CoV-2 infection

8890_9	T132B	Transmembrane protein 132B	Q14DG7	TMEM132B	chr12:125186386	Inverse variance weighted	2	-0.010	0.030	7.342E-01	0.978	cis	SARS-CoV-2 infection
9337_43	TKN1	Protachykinin-1	P20366	TAC1	chr7:97732084	Inverse variance weighted	3	-0.008	0.023	7.345E-01	0.978	cis	SARS-CoV-2 infection
9950_229	LAG-3	Lymphocyte activation gene 3 protein	P18627	LAG3	chr12:6772512	Inverse variance weighted	2	-0.012	0.035	7.349E-01	0.978	cis	SARS-CoV-2 infection
9211_19	PEDF	Pigment epithelium-derived factor	P36955	SERPINF1	chr17:1762029	Inverse variance weighted	4	0.008	0.023	7.354E-01	0.978	cis	SARS-CoV-2 infection
5404_53	DR6	Tumor necrosis factor receptor superfamily member 21	O75509	TNFRSF21	chr6:47309905	Inverse variance weighted	2	-0.020	0.060	7.369E-01	0.978	cis	SARS-CoV-2 infection
14048_7	IL-1 R AcP	Interleukin-1 Receptor accessory protein	Q9NPH3	IL1RAP	chr3:190514051	Inverse variance weighted	6	-0.002	0.007	7.376E-01	0.978	cis	SARS-CoV-2 infection
5728_60	FCRL1	Fc receptor-like protein 1	Q96LA6	FCRL1	chr1:157820120	Inverse variance weighted	4	-0.012	0.037	7.393E-01	0.978	cis	SARS-CoV-2 infection
8900_28	NEO1	Neogenin	Q92859	NEO1	chr15:73051710	Inverse variance weighted	3	0.012	0.036	7.399E-01	0.978	cis	SARS-CoV-2 infection
2994_71	IL-1Rrp2	Interleukin-1 receptor-like 2	Q9HB29	IL1RL2	chr2:102187006	Wald ratio	1	-0.012	0.035	7.400E-01	0.978	cis	SARS-CoV-2 infection
2816_50	BCAM	Basal Cell Adhesion Molecule	P50895	BCAM	chr19:44809071	Inverse variance weighted	5	0.006	0.019	7.404E-01	0.978	cis	SARS-CoV-2 infection
18337_4	GMDS	GDP-mannose 4,6 dehydratase	O60547	GMDS	chr6:2245605	Wald ratio	1	-0.030	0.091	7.409E-01	0.978	cis	SARS-CoV-2 infection
9223_11	ARMET	Mesencephalic astrocyte-derived neurotrophic factor	P55145	MANF	chr3:51385291	Wald ratio	1	-0.037	0.112	7.412E-01	0.978	cis	SARS-CoV-2 infection
17685_9	Apo A-IV	Apolipoprotein A-IV	P06727	APOA4	chr11:116823304	Wald ratio	1	-0.033	0.099	7.413E-01	0.978	cis	SARS-CoV-2 infection
19237_17	D-dopachrome decarboxylase	D-dopachrome decarboxylase	P30046	DDT	chr22:23980525	Inverse variance weighted	2	0.012	0.037	7.420E-01	0.978	cis	SARS-CoV-2 infection
9177_6	FAM3B	Protein FAM3B	P58499	FAM3B	chr21:41304212	Inverse variance weighted	7	-0.009	0.026	7.435E-01	0.978	cis	SARS-CoV-2 infection
3449_58	Kallistatin	Kallistatin	P29622	SERPINA4	chr14:94561442	Inverse variance weighted	7	0.006	0.018	7.436E-01	0.978	cis	SARS-CoV-2 infection
13465_5	CCP1	Calcipressin-1	P53805	RCAN1	chr21:34615113	Wald ratio	1	0.026	0.079	7.440E-01	0.978	cis	SARS-CoV-2 infection
12635_9	TRDMT	tRNA (cytosine(38)-C(5))-methyltransferase	O14717	TRDMT1	chr10:17202054	Inverse variance weighted	4	-0.011	0.033	7.445E-01	0.978	cis	SARS-CoV-2 infection
15530_33	EphB4	Ephrin type-B receptor 4	P54760	EPHB4	chr7:100827523	Wald ratio	1	-0.020	0.061	7.448E-01	0.978	cis	SARS-CoV-2 infection
7968_15	CRTAM	Cytotoxic and regulatory T-cell molecule	O95727	CRTAM	chr11:122838500	Inverse variance weighted	6	-0.008	0.026	7.458E-01	0.978	cis	SARS-CoV-2 infection
3299_29	Contactin-5	Contactin-5	O94779	CNTN5	chr11:99020949	Inverse variance weighted	3	-0.009	0.029	7.460E-01	0.978	cis	SARS-CoV-2 infection
18895_54	GSTM4	Glutathione S-transferase Mu 4	Q03013	GSTM4	chr1:109656099	Inverse variance weighted	7	0.013	0.039	7.464E-01	0.978	cis	SARS-CoV-2 infection
15298_199	NETO1	Neuropilin and tolloid-like protein 1	Q8TDF5	NETO1	chr18:72868146	Inverse variance weighted	2	0.022	0.068	7.471E-01	0.978	cis	SARS-CoV-2 infection
15470_11	Hexosaminidase B	Beta-hexosaminidase subunit beta	P07686	HEXB	chr5:74640023	Inverse variance weighted	4	0.006	0.018	7.482E-01	0.978	cis	SARS-CoV-2 infection
3172_28	ARSB	Arylsulfatase B	P15848	ARSB	chr5:78986087	Inverse variance weighted	2	-0.011	0.033	7.494E-01	0.978	cis	SARS-CoV-2 infection
7806_33	B4GT7	Beta-1,4-galactosyltransferase 7	Q9UBV7	B4GALT7	chr5:177600132	Inverse variance weighted	7	-0.005	0.017	7.496E-01	0.978	cis	SARS-CoV-2 infection
6493_9	CBPZ	Carboxypeptidase Z	Q66K79	CPZ	chr4:8592660	Inverse variance weighted	2	-0.016	0.050	7.496E-01	0.978	cis	SARS-CoV-2 infection
12563_2	TFIP8	Tumor necrosis factor alpha-induced protein 8	O95379	TNFAIP8	chr5:119268692	Inverse variance weighted	2	0.009	0.030	7.496E-01	0.978	cis	SARS-CoV-2 infection
12348_46	SYSM	Serine--tRNA ligase, mitochondrial	Q9NP81	SARS2	chr19:38930763	Wald ratio	1	-0.014	0.043	7.509E-01	0.978	cis	SARS-CoV-2 infection
15613_16	LIPP	Pancreatic triacylglycerol lipase	P16233	PNLIP	chr10:116545931	Wald ratio	1	-0.026	0.083	7.509E-01	0.978	cis	SARS-CoV-2 infection
18401_18	ALKB3	Alpha-ketoglutarate-dependent dioxygenase alkB homolog 3	Q96Q83	ALKBH3	chr11:43880811	Inverse variance weighted	3	-0.007	0.022	7.527E-01	0.978	cis	SARS-CoV-2 infection
16620_26	LY75	Lymphocyte antigen 75	O60449	LY75	chr2:159904756	Inverse variance weighted	7	0.003	0.011	7.528E-01	0.978	cis	SARS-CoV-2 infection
8346_9	DPP2	Dipeptidyl peptidase 2	Q9UHL4	DPP7	chr9:137118309	Inverse variance weighted	8	0.005	0.015	7.538E-01	0.978	cis	SARS-CoV-2 infection
9359_9	EGFL9	Protein delta homolog 2	Q6UY11	DLK2	chr6:43456632	Wald ratio	1	-0.009	0.029	7.539E-01	0.978	cis	SARS-CoV-2 infection
18162_167	IRAK4	Interleukin-1 receptor-associated kinase 4	Q9NWZ3	IRAK4	chr12:43758944	Wald ratio	1	-0.020	0.063	7.557E-01	0.978	cis	SARS-CoV-2 infection
5682_13	VASN	Vasorin	Q6EMK4	VASN	chr16:4371848	Wald ratio	1	0.021	0.068	7.562E-01	0.978	cis	SARS-CoV-2 infection
7994_41	ERO1B	ERO1-like protein beta	Q86YB8	ERO1B	chr1:236282019	Inverse variance weighted	2	-0.010	0.031	7.567E-01	0.978	cis	SARS-CoV-2 infection
12333_87	RPIA	Ribose-5-phosphate isomerase	P49247	RPIA	chr2:88691673	Inverse variance weighted	2	-0.010	0.032	7.573E-01	0.978	cis	SARS-CoV-2 infection
2635_61	Layilin	Layilin	Q6UX15	LAYN	chr11:111540280	Wald ratio	1	-0.028	0.090	7.573E-01	0.978	cis	SARS-CoV-2 infection
15308_108	VWC2	Brorin	Q2TAL6	VWC2	chr7:49773638	Inverse variance weighted	7	-0.004	0.014	7.575E-01	0.978	cis	SARS-CoV-2 infection
8398_277	RAR-responsive protein TIG1	Retinoic acid receptor responder protein 1	P49788	RARRES1	chr3:158732489	Inverse variance weighted	6	-0.003	0.009	7.576E-01	0.978	cis	SARS-CoV-2 infection
10442_1	TM190	Transmembrane protein 190	Q8WZ59	TMEM190	chr19:55376826	Inverse variance weighted	9	0.004	0.014	7.577E-01	0.978	cis	SARS-CoV-2 infection
7206_20	F16P1	Fructose-1,6-bisphosphatase 1	P09467	FBP1	chr9:94640249	Inverse variance weighted	2	0.024	0.077	7.578E-01	0.978	cis	SARS-CoV-2 infection
4867_15	VEGF121	Vascular endothelial growth factor A, isoform 121	P15692	VEGFA	chr6:43770184	Inverse variance weighted	4	0.015	0.049	7.589E-01	0.978	cis	SARS-CoV-2 infection
4437_56	ENTP5	Ectonucleoside triphosphate diphosphohydrolase 5	O75356	ENTPD5	chr14:74019399	Inverse variance weighted	8	-0.004	0.012	7.595E-01	0.978	cis	SARS-CoV-2 infection
5087_5	IL-22BP	Interleukin-22 receptor subunit alpha-2	Q969J5	IL22RA2	chr6:137173648	Inverse variance weighted	5	-0.007	0.022	7.599E-01	0.978	cis	SARS-CoV-2 infection
10565_19	SLIK3	SLIT and NTRK-like protein 3	O94933	SLITRK3	chr3:165197109	Inverse variance weighted	3	-0.005	0.015	7.600E-01	0.978	cis	SARS-CoV-2 infection
4152_58	Prekallikrein	Plasma kallikrein	P03952	KLKB1	chr4:186226438	Inverse variance weighted	4	-0.006	0.019	7.603E-01	0.978	cis	SARS-CoV-2 infection
16828_8	Collagen a1(VI)	Collagen alpha-1(VI) chain	P12109	COL6A1	chr21:45981770	Inverse variance weighted	9	-0.003	0.009	7.604E-01	0.978	cis	SARS-CoV-2 infection
9380_2	sPLA(2)-XIII	Group XIIB secretory phospholipase A2-like protein	Q9BX93	PLA2G12B	chr10:72954806	Wald ratio	1	-0.017	0.055	7.617E-01	0.978	cis	SARS-CoV-2 infection
7211_2	RNase 1	Ribonuclease pancreatic	P07998	RNASE1	chr14:20802855	Inverse variance weighted	4	-0.019	0.062	7.618E-01	0.978	cis	SARS-CoV-2 infection
5103_30	MO2R1:ECD	Cell surface glycoprotein CD200 receptor 1:Isoform 4, Extracellular Domain	Q8TD46	CD200R1	chr3:112975103	Wald ratio	1	0.013	0.044	7.620E-01	0.978	cis	SARS-CoV-2 infection
8989_40	SCUB1	Signal peptide, CUB and EGF-like domain-containing protein 1	Q8IWY4	SCUBE1	chr22:43343372	Inverse variance weighted	4	-0.009	0.030	7.620E-01	0.978	cis	SARS-CoV-2 infection
5736_1	TRML2	Trem-like transcript 2 protein	Q5T2D2	TREML2	chr6:41201149	Inverse variance weighted	5	0.004	0.013	7.628E-01	0.978	cis	SARS-CoV-2 infection
4232_19	IGF-I sR	Insulin-like growth factor 1 receptor	P08069	IGF1R	chr15:98648539	Inverse variance weighted	2	0.013	0.042	7.631E-01	0.978	cis	SARS-CoV-2 infection
9383_24	CH3L2	Chitinase-3-like protein 2	Q15782	CHI3L2	chr1:111200771	Wald ratio	1	-0.025	0.082	7.633E-01	0.978	cis	SARS-CoV-2 infection
5231_79	PCSK9	Proprotein convertase subtilisin/kexin type 9	Q8NBP7	PCSK9	chr1:55039447	Inverse variance weighted	6	0.005	0.018	7.637E-01	0.978	cis	SARS-CoV-2 infection
12571_14	ARL3	ADP-ribosylation factor-like protein 3	P36405	ARL3	chr10:102714397	Inverse variance weighted	2	-0.009	0.031	7.641E-01	0.978	cis	SARS-CoV-2 infection
19590_46	SP-D	Pulmonary surfactant-associated protein D	P35247	SFTPD	chr10:79982614	Inverse variance weighted	7	-0.005	0.018	7.644E-01	0.978	cis	SARS-CoV-2 infection
5867_60	ARGI1	Arginase-1	P05089	ARG1	chr6:131470832	Inverse variance weighted	2	0.014	0.046	7.650E-01	0.978	cis	SARS-CoV-2 infection
11377_19	ADH7	Alcohol dehydrogenase class 4 mu/sigma chain	P40394	ADH7	chr4:99435510	Inverse variance weighted	3	-0.005	0.017	7.657E-01	0.978	cis	SARS-CoV-2 infection
2278_61	TIMP-2	Metalloproteinase inhibitor 2	P16035	TIMP2	chr17:78925387	Wald ratio	1	0.021	0.071	7.660E-01	0.978	cis	SARS-CoV-2 infection
10514_5	PGD2 synthase	Prostaglandin-H2 D-isomerase	P41222	PTGDS	chr9:136975092	Wald ratio	1	0.031	0.105	7.661E-01	0.978	cis	SARS-CoV-2 infection
3184_25	Coagulation Factor VII	Coagulation factor VII	P08709	F7	chr13:113105788	Inverse variance weighted	7	0.003	0.010	7.674E-01	0.978	cis	SARS-CoV-2 infection
16583_8	AP4A	Bis(5'-nucleosyl)-tetraphosphatase [asymmetrical]	P50583	NUDT2	chr9:34329506	Inverse variance weighted	7	0.010	0.033	7.682E-01	0.978	cis	SARS-CoV-2 infection
19117_3	PP14A	Protein phosphatase 1 regulatory subunit 14A	Q96A00	PPP1R14A	chr19:38256532	Wald ratio	1	0.025	0.085	7.690E-01	0.978	cis	SARS-CoV-2 infection
8394_56	RNase 2	Non-secretory ribonuclease	P10153	RNASE2	chr14:20955487	Wald ratio	1	-0.018	0.060	7.691E-01	0.978	cis	SARS-CoV-2 infection
2692_74	NPS-PLA2	Phospholipase A2, membrane associated	P14555	PLA2G2A	chr1:19980416	Inverse variance weighted	6	0.003	0.009	7.696E-01	0.978	cis	SARS-CoV-2 infection
15435_4	PNP	Purine nucleoside phosphorylase	P00491	PNP	chr14:20461992	Inverse variance weighted	2	-0.009	0.032	7.700E-01	0.978	cis	SARS-CoV-2 infection
9964_10	MYZAP	Myocardial zonula adherens protein	P0CAP1	MYZAP	chr15:57591904	Wald ratio	1	0.021	0.073	7.706E-01	0.978	cis	SARS-CoV-2 infection
15475_4	PLTP	Phospholipid transfer protein	P55058	PLTP	chr20:45912155	Inverse variance weighted	9	-0.005	0.016	7.712E-01	0.978	cis	SARS-CoV-2 infection
4162_54	Transferrin	Serotransferrin	P02787	TF	chr3:133746040	Inverse variance weighted	2	0.009	0.030	7.717E-01	0.978	cis	SARS-CoV-2 infection
11493_169	DYL2	Dynein light chain 2, cytoplasmic	Q96FJ2	DYNLL2	chr17:58083419	Wald ratio	1	-0.027	0.092	7.725E-01	0.978	cis	SARS-CoV-2 infection
2855_49	ERK-1	Mitogen-activated protein kinase 3	P27361	MAPK3	chr16:30123506	Inverse variance weighted	3	-0.020	0.070	7.726E-01	0.978	cis	SARS-CoV-2 infection
18396_10	AES	Amino-terminal enhancer of split	Q08117	TLE5	chr19:3063107	Wald ratio	1	-0.028	0.097	7.733E-01	0.978	cis	SARS-CoV-2 infection
11390_24	Carbonic Anhydrase VIII	Carbonic anhydrase-related protein	P35219	CA8	chr8:60281400	Inverse variance weighted	6	0.004	0.012	7.739E-01	0.978	cis	SARS-CoV-2 infection
6393_63	Endoplasmin	Endoplasmin	P14625	HSP90B1	chr12:103930107	Inverse variance weighted	6	0.002	0.008	7.740E-01	0.978	cis	SARS-CoV-2 infection
5680_54	OBP2B	Odorant-binding protein 2b	Q9NPH6	OBP2B	chr9:133209250	Inverse variance weighted	4	-0.007	0.026	7.741E-01	0.978	cis	SARS-CoV-2 infection

19446_1	GMPR2	GMP reductase 2	Q9P2T1	GMPR2	chr14:24232422	Inverse variance weighted	3	0.003	0.010	7.764E-01	0.978	cis	SARS-CoV-2 infection
16322_10	PACAP	Marginal zone B- and B1-cell-specific protein	Q8WU39	MZB1	chr5:139390081	Inverse variance weighted	3	-0.012	0.041	7.767E-01	0.978	cis	SARS-CoV-2 infection
17396_23	ADH1A	Alcohol dehydrogenase 1A	P07327	ADH1A	chr4:99291003	Wald ratio	1	-0.013	0.045	7.768E-01	0.978	cis	SARS-CoV-2 infection
16323_8	NRX3A	Neurexin-3	Q9Y4C0	NRXN3	chr14:78170373	Wald ratio	1	-0.029	0.101	7.768E-01	0.978	cis	SARS-CoV-2 infection
18422_41	ERG19	Diphosphomevalonate decarboxylase	P53602	MVD	chr16:88663161	Inverse variance weighted	2	0.022	0.076	7.777E-01	0.978	cis	SARS-CoV-2 infection
15452_5	5'-Nucleotidase	5'-Nucleotidase	P21589	NT5E	chr6:85449584	Inverse variance weighted	4	0.004	0.013	7.783E-01	0.978	cis	SARS-CoV-2 infection
4831_4	sL-Selectin	L-Selectin	P14151	SELL	chr1:169711702	Inverse variance weighted	7	0.003	0.012	7.786E-01	0.978	cis	SARS-CoV-2 infection
2946_52	Factor D	Complement factor D	P00746	CFD	chr19:859453	Inverse variance weighted	6	-0.007	0.023	7.793E-01	0.978	cis	SARS-CoV-2 infection
9313_27	CBLN1	Cerebellin-1	P23435	CBLN1	chr16:49281838	Inverse variance weighted	9	-0.005	0.017	7.800E-01	0.978	cis	SARS-CoV-2 infection
9765_4	NDE1	Nuclear distribution protein nudE homolog 1	Q9NXR1	NDE1	chr16:15643267	Wald ratio	1	0.019	0.069	7.803E-01	0.978	cis	SARS-CoV-2 infection
12684_5	ADSV	Adseverin	Q9Y6U3	SCIN	chr7:12570577	Wald ratio	1	0.019	0.067	7.808E-01	0.978	cis	SARS-CoV-2 infection
10627_87	APLP2	Amyloid-like protein 2	Q06481	APLP2	chr11:130068147	Inverse variance weighted	2	-0.009	0.034	7.808E-01	0.978	cis	SARS-CoV-2 infection
8465_52	Cathepsin H	Cathepsin H	P09668	CTSH	chr15:78949574	Inverse variance weighted	10	-0.002	0.008	7.808E-01	0.978	cis	SARS-CoV-2 infection
5704_74	Granzyme M	Granzyme M	P51124	GZMM	chr19:544034	Inverse variance weighted	2	0.027	0.099	7.832E-01	0.978	cis	SARS-CoV-2 infection
14684_17	CAN2	Calpain-2 catalytic subunit	P17655	CAPN2	chr1:223701593	Inverse variance weighted	3	0.008	0.029	7.833E-01	0.978	cis	SARS-CoV-2 infection
17785_11	RCL	2'-deoxynucleoside 5'-phosphate N-hydrolase 1	O43598	DNPH1	chr6:43229481	Wald ratio	1	-0.016	0.060	7.836E-01	0.978	cis	SARS-CoV-2 infection
12020_39	PMGE	Bisphosphoglycerate mutase	P07738	BPGM	chr7:134646811	Wald ratio	1	0.034	0.125	7.837E-01	0.978	cis	SARS-CoV-2 infection
4964_67	ARTS1	Endoplasmic reticulum aminopeptidase 1	Q9NZ08	ERAP1	chr5:96808100	Inverse variance weighted	5	-0.003	0.010	7.850E-01	0.978	cis	SARS-CoV-2 infection
5584_21	Holo-TC II	Holo-Transcobalamin-2	P20062	TCN2	chr22:30607003	Inverse variance weighted	4	0.005	0.019	7.855E-01	0.978	cis	SARS-CoV-2 infection
2900_53	HCC-1	C-C motif chemokine 14	Q16627	CCL14	chr17:35987004	Inverse variance weighted	4	0.010	0.037	7.863E-01	0.978	cis	SARS-CoV-2 infection
6049_64	PTPRS	Receptor-type tyrosine-protein phosphatase S	Q13332	PTPRS	chr19:5340812	Wald ratio	1	0.016	0.057	7.864E-01	0.978	cis	SARS-CoV-2 infection
9793_145	IGDC4	Immunoglobulin superfamily DCC subclass member 4	Q8TDY8	IGDCC4	chr15:65422947	Inverse variance weighted	7	-0.005	0.017	7.876E-01	0.978	cis	SARS-CoV-2 infection
19606_28	ihh	Indian hedgehog protein	Q14623	IHH	chr2:219060921	Inverse variance weighted	3	0.013	0.050	7.882E-01	0.978	cis	SARS-CoV-2 infection
2705_5	TECK	C-C motif chemokine 25	O15444	CCL25	chr19:8052318	Inverse variance weighted	12	0.003	0.011	7.884E-01	0.978	cis	SARS-CoV-2 infection
10569_28	MFAP2	Microfibrillar-associated protein 2	P55001	MFAP2	chr1:16980632	Inverse variance weighted	4	0.025	0.094	7.888E-01	0.978	cis	SARS-CoV-2 infection
8360_169	NKp46	Natural cytotoxicity triggering receptor 1	O76036	NCR1	chr19:54906148	Wald ratio	1	0.014	0.054	7.892E-01	0.978	cis	SARS-CoV-2 infection
7096_30	RMD1	Regulator of microtubule dynamics protein 1	Q96DB5	RMDN1	chr8:86514357	Inverse variance weighted	4	-0.007	0.028	7.907E-01	0.978	cis	SARS-CoV-2 infection
5121_3	SEM6B	Semaphorin-6B	Q9H3T3	SEMA6B	chr19:4581776	Inverse variance weighted	2	-0.015	0.056	7.907E-01	0.978	cis	SARS-CoV-2 infection
9018_38	PCD10:ECD	Protocadherin-10:Extracellular domain	Q9P2E7	PCDH10	chr4:133149294	Inverse variance weighted	2	0.007	0.028	7.918E-01	0.978	cis	SARS-CoV-2 infection
5275_28	VAV	Proto-oncogene vav	P15498	VAV1	chr19:6772708	Wald ratio	1	-0.022	0.084	7.924E-01	0.978	cis	SARS-CoV-2 infection
2731_29	NADPH-P450 Oxidoreductase	NADPH--cytochrome P450 reductase	P16435	POR	chr7:75899200	Inverse variance weighted	2	-0.011	0.041	7.930E-01	0.978	cis	SARS-CoV-2 infection
9468_8	Lectin, mannose-binding 2	Vesicular integral-membrane protein VIP36	Q12907	LMAN2	chr5:177351840	Wald ratio	1	0.015	0.057	7.932E-01	0.978	cis	SARS-CoV-2 infection
7861_9	ROR2	Tyrosine-protein kinase transmembrane receptor ROR2	Q01974	ROR2	chr9:91950228	Inverse variance weighted	2	0.011	0.042	7.937E-01	0.978	cis	SARS-CoV-2 infection
15570_99	Complement receptor type 2	Complement receptor type 2	P20023	CR2	chr1:207453024	Inverse variance weighted	3	-0.008	0.032	7.943E-01	0.978	cis	SARS-CoV-2 infection
12522_6	RD23B	UV excision repair protein RAD23 homolog B	P54727	RAD23B	chr9:107283137	Wald ratio	1	-0.023	0.087	7.954E-01	0.978	cis	SARS-CoV-2 infection
19161_1	UBP15	Ubiquitin carboxyl-terminal hydrolase 15	Q9Y4E8	USP15	chr12:62260338	Inverse variance weighted	2	0.009	0.034	7.965E-01	0.978	cis	SARS-CoV-2 infection
19236_24	TCP4	Activated RNA polymerase II transcriptional coactivator p15	P53999	SUB1	chr5:32531633	Wald ratio	1	-0.026	0.100	7.968E-01	0.978	cis	SARS-CoV-2 infection
8099_42	SPON2	Spondin-2	Q9BUD6	SPON2	chr4:1208962	Inverse variance weighted	6	0.005	0.019	7.984E-01	0.978	cis	SARS-CoV-2 infection
7050_5	NEGR1	Neuronal growth regulator 1	Q7Z3B1	NEGR1	chr1:72282539	Wald ratio	1	-0.027	0.107	7.985E-01	0.978	cis	SARS-CoV-2 infection
5637_81	NTNG1	Netrin-G1	Q9Y2I2	NTNG1	chr1:107140007	Inverse variance weighted	9	-0.003	0.012	7.986E-01	0.978	cis	SARS-CoV-2 infection
13992_12	NSF	Vesicle-fusing ATPase	P46459	NSF	chr17:46590669	Wald ratio	1	-0.012	0.049	7.986E-01	0.978	cis	SARS-CoV-2 infection
3340_53	TSP4	Thrombospondin-4	P35443	THBS4	chr5:79991311	Inverse variance weighted	3	-0.005	0.019	7.987E-01	0.978	cis	SARS-CoV-2 infection
15602_43	IL-6 sRa	Interleukin-6 receptor subunit alpha	P08887	IL6R	chr1:154405193	Inverse variance weighted	6	-0.002	0.007	7.987E-01	0.978	cis	SARS-CoV-2 infection
13657_2	PNKP	Bifunctional polynucleotide phosphatase/kinase	Q96T60	PNKP	chr19:49878351	Wald ratio	1	0.012	0.047	7.992E-01	0.978	cis	SARS-CoV-2 infection
4801_13	PERL	Lactoperoxidase	P22079	LPO	chr17:58218548	Inverse variance weighted	2	0.005	0.022	7.997E-01	0.978	cis	SARS-CoV-2 infection
3488_64	Catalase	Catalase	P04040	CAT	chr11:34438934	Inverse variance weighted	3	-0.014	0.057	8.000E-01	0.978	cis	SARS-CoV-2 infection
13943_38	DPY30	Protein dpy-30 homolog	Q9C005	DPY30	chr2:32039805	Wald ratio	1	0.018	0.073	8.024E-01	0.978	cis	SARS-CoV-2 infection
15322_35	CRADD	Death domain-containing protein CRADD	P78560	CRADD	chr12:93677375	Inverse variance weighted	2	0.031	0.124	8.032E-01	0.978	cis	SARS-CoV-2 infection
5430_66	SHPS1	Tyrosine-protein phosphatase non-receptor type substrate 1	P78324	SIRPA	chr20:1894167	Inverse variance weighted	9	0.003	0.012	8.041E-01	0.978	cis	SARS-CoV-2 infection
7841_84	ESAM	Endothelial cell-selective adhesion molecule	Q96AP7	ESAM	chr11:124762290	Inverse variance weighted	6	0.005	0.019	8.046E-01	0.978	cis	SARS-CoV-2 infection
5621_64	THSD1	Thrombospondin type-1 domain-containing protein 1	Q9NS62	THSD1	chr13:52416373	Inverse variance weighted	4	0.003	0.014	8.053E-01	0.978	cis	SARS-CoV-2 infection
3033_57	Galectin-2	Galectin-2	P05162	LGALS2	chr22:37582616	Inverse variance weighted	2	-0.006	0.023	8.057E-01	0.978	cis	SARS-CoV-2 infection
15417_3	Maspin	Serpin B5	P36952	SERPINB5	chr18:63476958	Wald ratio	1	-0.009	0.036	8.058E-01	0.978	cis	SARS-CoV-2 infection
12687_2	DECR	2,4-dienoyl-CoA reductase, mitochondrial	Q16698	DECR1	chr8:90001405	Inverse variance weighted	3	-0.027	0.112	8.062E-01	0.978	cis	SARS-CoV-2 infection
16614_27	RSPO1	R-spondin-1	Q2MKA7	RSPO1	chr1:37634892	Inverse variance weighted	2	0.005	0.022	8.072E-01	0.978	cis	SARS-CoV-2 infection
8840_61	C1s	Complement C1s subcomponent	P09871	C1S	chr12:6988259	Inverse variance weighted	4	0.010	0.042	8.079E-01	0.978	cis	SARS-CoV-2 infection
17398_55	HO-1	Heme oxygenase 1	P09601	HMOX1	chr22:35380361	Inverse variance weighted	3	-0.011	0.046	8.090E-01	0.978	cis	SARS-CoV-2 infection
15370_5	BOLA1	BolA-like protein 1	Q9Y3E2	BOLA1	chr1:149887890	Inverse variance weighted	2	0.004	0.018	8.092E-01	0.978	cis	SARS-CoV-2 infection
7928_183	TPST1	Protein-tyrosine sulfotransferase 1	O60507	TPST1	chr7:66205317	Inverse variance weighted	2	-0.004	0.016	8.098E-01	0.978	cis	SARS-CoV-2 infection
5701_81	Tetranectin	Tetranectin	P05452	CLEC3B	chr3:45001548	Inverse variance weighted	2	-0.006	0.026	8.100E-01	0.978	cis	SARS-CoV-2 infection
6920_1	GFRAL	GDNF family receptor alpha-like	Q6UXV0	GFRAL	chr6:55327469	Inverse variance weighted	3	0.006	0.024	8.107E-01	0.978	cis	SARS-CoV-2 infection
17791_25	HPCL1	Hippocalcin-like protein 1	P37235	HPCAL1	chr2:10302889	Wald ratio	1	-0.015	0.064	8.119E-01	0.978	cis	SARS-CoV-2 infection
4187_49	6-Phosphogluconate dehydrogenase	6-phosphogluconate dehydrogenase, decarboxylating	P52209	PGD	chr1:10398592	Wald ratio	1	0.015	0.065	8.124E-01	0.978	cis	SARS-CoV-2 infection
13704_5	HMCS2	Hydroxymethylglutaryl-CoA synthase, mitochondrial	P54868	HMGCS2	chr1:119768905	Wald ratio	1	0.022	0.091	8.125E-01	0.978	cis	SARS-CoV-2 infection
7218_87	AT1B2	Sodium/potassium-transporting ATPase subunit beta-2	P14415	ATP1B2	chr17:7646627	Inverse variance weighted	3	0.010	0.045	8.154E-01	0.978	cis	SARS-CoV-2 infection
3324_51	LY9	T-lymphocyte surface antigen Ly-9	Q9HBG7	LY9	chr1:160796074	Inverse variance weighted	5	-0.006	0.028	8.154E-01	0.978	cis	SARS-CoV-2 infection
6431_68	PCYOX	Prenylcysteine oxidase 1	Q9UHG3	PCYOX1	chr2:70257386	Inverse variance weighted	6	-0.002	0.009	8.160E-01	0.978	cis	SARS-CoV-2 infection
2811_27	Angiopoietin-1	Angiopoietin-1	Q15389	ANGPT1	chr8:107498055	Wald ratio	1	-0.016	0.071	8.162E-01	0.978	cis	SARS-CoV-2 infection
13929_27	OCTC	Peroxisomal carnitine O-octanoyltransferase	Q9UKG9	CROT	chr7:87345664	Inverse variance weighted	6	-0.003	0.014	8.173E-01	0.978	cis	SARS-CoV-2 infection
8925_25	RIR2B	Ribonucleoside-diphosphate reductase subunit M2 B	Q7LG56	RRM2B	chr8:102238961	Inverse variance weighted	2	-0.015	0.065	8.174E-01	0.978	cis	SARS-CoV-2 infection
19113_66	SH3L2	SH3 domain-binding glutamic acid-rich-like protein 2	Q9UJC5	SH3BGRL2	chr6:79631329	Wald ratio	1	0.011	0.046	8.178E-01	0.978	cis	SARS-CoV-2 infection
15523_9	HEM2	Delta-aminolevulinic acid dehydratase	P13716	ALAD	chr9:113401290	Inverse variance weighted	7	0.004	0.020	8.185E-01	0.978	cis	SARS-CoV-2 infection
19383_131	CINP	Cyclin-dependent kinase 2-interacting protein	Q9BW66	CINP	chr14:102362916	Wald ratio	1	0.021	0.091	8.193E-01	0.978	cis	SARS-CoV-2 infection
18218_48	CB032	CB1 cannabinoid receptor-interacting protein 1	Q96F85	CNRIP1	chr2:68320051	Inverse variance weighted	7	0.003	0.012	8.195E-01	0.978	cis	SARS-CoV-2 infection
13666_222	Carbonic Anhydrase X	Carbonic anhydrase-related protein 10	Q9NS85	CA10	chr17:52160017	Inverse variance weighted	4	0.004	0.017	8.196E-01	0.978	cis	SARS-CoV-2 infection
7099_33	FSTL5	Follistatin-related protein 5	Q8N475	FSTL5	chr4:162164004	Wald ratio	1	-0.013	0.055	8.200E-01	0.978	cis	SARS-CoV-2 infection
15686_49	INHBC	Inhibin beta C chain	P55103	INHBC	chr12:57434784	Inverse variance weighted	14	0.002	0.007	8.206E-01	0.978	cis	SARS-CoV-2 infection
11543_84	LIMA1	LIM domain and actin-binding protein 1	Q9UHB6	LIMA1	chr12:50283520	Wald ratio	1	-0.017	0.077	8.217E-01	0.978	cis	SARS-CoV-2 infection

9360_33	EDIL3	EGF-like repeat and discoidin I-like domain-containing protein 3	O43854	EDIL3	chr5:84384880	Inverse variance weighted	2	0.013	0.060	8.218E-01	0.978	cis	SARS-CoV-2 infection
16015_19	ALT	Alanine aminotransferase 1	P24298	GPT	chr8:144502973	Wald ratio	1	0.021	0.092	8.224E-01	0.978	cis	SARS-CoV-2 infection
12411_60	MAX	Protein max	P61244	MAX	chr14:65102695	Wald ratio	1	-0.006	0.027	8.229E-01	0.978	cis	SARS-CoV-2 infection
6375_75	XXLT1	Xyloside xylosyltransferase 1	Q8NBI6	XXYLT1	chr3:195271159	Inverse variance weighted	2	-0.005	0.022	8.241E-01	0.978	cis	SARS-CoV-2 infection
5316_54	Prothrombin	Prothrombin	P00734	F2	chr11:46719196	Wald ratio	1	-0.021	0.094	8.253E-01	0.978	cis	SARS-CoV-2 infection
11606_22	DNJB6	DnaJ homolog subfamily B member 6	O75190	DNAJB6	chr7:157335381	Wald ratio	1	-0.021	0.094	8.256E-01	0.978	cis	SARS-CoV-2 infection
13116_25	CD177	CD177 antigen	Q8N6Q3	CD177	chr19:43353686	Inverse variance weighted	8	-0.002	0.010	8.257E-01	0.978	cis	SARS-CoV-2 infection
16916_19	SLIK6	SLIT and NTRK-like protein 6	Q9H5Y7	SLITRK6	chr13:85806683	Inverse variance weighted	4	0.005	0.025	8.260E-01	0.978	cis	SARS-CoV-2 infection
12633_3	PTN9	Tyrosine-protein phosphatase non-receptor type 9	P43378	PTPN9	chr15:75579315	Wald ratio	1	0.019	0.086	8.276E-01	0.978	cis	SARS-CoV-2 infection
8252_2	NOTUM	Palmitoleoyl-protein carboxylesterase NOTUM	Q6P988	NOTUM	chr17:81961840	Inverse variance weighted	3	-0.011	0.051	8.277E-01	0.978	cis	SARS-CoV-2 infection
2789_26	MMP-7	Matrilysin	P09237	MMP7	chr11:102530750	Inverse variance weighted	4	-0.006	0.028	8.278E-01	0.978	cis	SARS-CoV-2 infection
8885_6	CA2D3	Voltage-dependent calcium channel subunit alpha-2/delta-3	Q8IZS8	CACNA2D3	chr3:54122547	Inverse variance weighted	5	0.005	0.023	8.281E-01	0.978	cis	SARS-CoV-2 infection
14623_26	SUMO3	Small ubiquitin-related modifier 3	P55854	SUMO3	chr21:44818779	Wald ratio	1	-0.019	0.086	8.283E-01	0.978	cis	SARS-CoV-2 infection
16763_11	LECT2	Leukocyte cell-derived chemotaxin-2	O14960	LECT2	chr5:135954983	Inverse variance weighted	9	0.003	0.012	8.285E-01	0.978	cis	SARS-CoV-2 infection
18930_28	SLIT2	Slit homolog 2 protein	O94813	SLIT2	chr4:20251905	Inverse variance weighted	2	-0.013	0.062	8.287E-01	0.978	cis	SARS-CoV-2 infection
6382_17	MANBA	Beta-mannosidase	O00462	MANBA	chr4:102760994	Inverse variance weighted	5	0.005	0.022	8.303E-01	0.978	cis	SARS-CoV-2 infection
13122_19	FLRT2	Leucine-rich repeat transmembrane protein FLRT2	O43155	FLRT2	chr14:85530144	Inverse variance weighted	5	0.003	0.016	8.304E-01	0.978	cis	SARS-CoV-2 infection
7871_16	T132A	Transmembrane protein 132A	Q24JP5	TMEM132A	chr11:60924460	Inverse variance weighted	6	0.002	0.012	8.308E-01	0.978	cis	SARS-CoV-2 infection
4930_21	Stanniocalcin-1	Stanniocalcin-1	P52823	STC1	chr8:23854806	Wald ratio	1	-0.012	0.058	8.309E-01	0.978	cis	SARS-CoV-2 infection
8427_118	RSPO3	R-spondin-3	Q9BXY4	RSPO3	chr6:127118671	Inverse variance weighted	4	-0.013	0.061	8.309E-01	0.978	cis	SARS-CoV-2 infection
8299_66	LIRA4	Leukocyte immunoglobulin-like receptor subfamily A member 4	P59901	LILRA4	chr19:54339162	Inverse variance weighted	3	-0.011	0.053	8.311E-01	0.978	cis	SARS-CoV-2 infection
2474_54	SAP	Serum amyloid P-component	P02743	APCS	chr1:159587826	Inverse variance weighted	3	0.005	0.023	8.311E-01	0.978	cis	SARS-CoV-2 infection
13109_82	NEGR1	Neuronal growth regulator 1	Q7Z3B1	NEGR1	chr1:72282539	Wald ratio	1	-0.016	0.077	8.317E-01	0.978	cis	SARS-CoV-2 infection
17345_12	ZADH2	Prostaglandin reductase 3	Q8N4Q0	PTGR3	chr18:75209139	Wald ratio	1	-0.010	0.049	8.319E-01	0.978	cis	SARS-CoV-2 infection
16324_38	TLR1:ECD	Toll-like receptor 1:Extracellular domain	Q15399	TLR1	chr4:38856817	Wald ratio	1	0.009	0.042	8.327E-01	0.978	cis	SARS-CoV-2 infection
15514_26	Pseudocholinesterase	Cholinesterase	P06276	BCHE	chr3:165837462	Inverse variance weighted	6	-0.003	0.014	8.330E-01	0.978	cis	SARS-CoV-2 infection
8376_25	LSHB	Lutropin subunit beta	P01229	LHB	chr19:49017091	Inverse variance weighted	4	-0.004	0.017	8.337E-01	0.978	cis	SARS-CoV-2 infection
8660_5	OLFL3	Olfactomedin-like protein 3	Q9NRN5	OLFML3	chr1:113979391	Inverse variance weighted	2	-0.010	0.047	8.345E-01	0.978	cis	SARS-CoV-2 infection
8351_17	PRS57	Serine protease 57	Q6UWY2	PRSS57	chr19:695498	Inverse variance weighted	6	-0.004	0.020	8.345E-01	0.978	cis	SARS-CoV-2 infection
17410_5	CETN3	Centrin-3	O15182	CETN3	chr5:90409766	Wald ratio	1	-0.014	0.066	8.349E-01	0.978	cis	SARS-CoV-2 infection
15384_15	KLOTHO	Klotho	Q9UEF7	KL	chr13:33016423	Inverse variance weighted	4	-0.008	0.040	8.353E-01	0.978	cis	SARS-CoV-2 infection
10612_18	PLOD3	Procollagen-lysine,2-oxoglutarate 5-dioxygenase 3	O60568	PLOD3	chr7:101218420	Inverse variance weighted	3	0.009	0.045	8.358E-01	0.978	cis	SARS-CoV-2 infection
5646_20	RNAS6	Ribonuclease K6	Q93091	RNASE6	chr14:20781268	Inverse variance weighted	8	0.002	0.009	8.373E-01	0.978	cis	SARS-CoV-2 infection
17813_21	BPHL	Valacyclovir hydrolase	Q86WA6	BPHL	chr6:3118374	Inverse variance weighted	2	0.013	0.062	8.384E-01	0.978	cis	SARS-CoV-2 infection
11145_72	K154L	UPF0606 protein KIAA1549L	Q6ZVL6	KIAA1549L	chr11:33376108	Inverse variance weighted	7	0.004	0.018	8.386E-01	0.978	cis	SARS-CoV-2 infection
13113_7	Osteopontin	Osteopontin	P10451	SPP1	chr4:87975667	Inverse variance weighted	2	0.006	0.029	8.389E-01	0.978	cis	SARS-CoV-2 infection
10800_15	Collagen-binding protein	Serpin H1	P50454	SERPINH1	chr11:75562056	Inverse variance weighted	2	0.007	0.035	8.393E-01	0.978	cis	SARS-CoV-2 infection
2579_17	MMP-9	Matrix metalloproteinase-9	P14780	MMP9	chr20:46008908	Wald ratio	1	-0.013	0.064	8.394E-01	0.978	cis	SARS-CoV-2 infection
16823_75	APOL3	Apolipoprotein L3	O95236	APOL3	chr22:36166177	Inverse variance weighted	7	-0.002	0.011	8.396E-01	0.978	cis	SARS-CoV-2 infection
9021_1	TIM-1	Hepatitis A virus cellular receptor 1	Q96D42	HAVCR1	chr5:157069396	Inverse variance weighted	6	0.003	0.017	8.404E-01	0.978	cis	SARS-CoV-2 infection
6416_8	GKN2	Gastrokine-2	Q86XP6	GKN2	chr2:68952893	Inverse variance weighted	5	0.002	0.012	8.409E-01	0.978	cis	SARS-CoV-2 infection
16292_288	GIP	Gastric inhibitory polypeptide	P09681	GIP	chr17:48968596	Wald ratio	1	-0.020	0.102	8.409E-01	0.978	cis	SARS-CoV-2 infection
9747_48	MARK3	MAP/microtubule affinity-regulating kinase 3	P27448	MARK3	chr14:103385377	Wald ratio	1	-0.023	0.116	8.412E-01	0.978	cis	SARS-CoV-2 infection
3581_53	a2-HS-Glycoprotein	Alpha-2-HS-glycoprotein	P02765	AHSG	chr3:186613060	Inverse variance weighted	4	-0.007	0.036	8.417E-01	0.978	cis	SARS-CoV-2 infection
16753_46	CO6A2	Collagen alpha-2(VI) chain	P12110	COL6A2	chr21:46098112	Inverse variance weighted	4	0.005	0.027	8.417E-01	0.978	cis	SARS-CoV-2 infection
3043_49	ON	SPARC	P09486	SPARC	chr5:151686975	Wald ratio	1	0.020	0.103	8.421E-01	0.978	cis	SARS-CoV-2 infection
9314_9	PSG5	Pregnancy-specific beta-1-glycoprotein 5	Q15238	PSG5	chr19:43186536	Inverse variance weighted	3	0.011	0.057	8.424E-01	0.978	cis	SARS-CoV-2 infection
7019_13	Semaphorin-7A	Semaphorin-7A	O75326	SEMA7A	chr15:74433958	Wald ratio	1	-0.012	0.060	8.427E-01	0.978	cis	SARS-CoV-2 infection
6247_9	SIRB1	Signal-regulatory protein beta-1	O00241	SIRPB1	chr20:1620061	Inverse variance weighted	9	-0.001	0.005	8.442E-01	0.978	cis	SARS-CoV-2 infection
3166_92	Siglec-3	Myeloid cell surface antigen CD33	P20138	CD33	chr19:51225064	Inverse variance weighted	6	-0.002	0.010	8.451E-01	0.978	cis	SARS-CoV-2 infection
9901_28	EGLN1	Egl nine homolog 1	Q9GZT9	EGLN1	chr1:231422287	Inverse variance weighted	5	0.005	0.024	8.458E-01	0.978	cis	SARS-CoV-2 infection
3079_62	TIG2	Retinoic acid receptor responder protein 2	Q99969	RARRES2	chr7:150341662	Inverse variance weighted	2	-0.005	0.026	8.460E-01	0.978	cis	SARS-CoV-2 infection
7173_141	T132C:ECD	Transmembrane protein 132C:Extracellular domain	Q8N3T6	TMEM132C	chr12:128267170	Inverse variance weighted	5	0.003	0.015	8.485E-01	0.978	cis	SARS-CoV-2 infection
15299_102	MESD2	LDLR chaperone MESD	Q14696	MESD	chr15:80989828	Wald ratio	1	0.020	0.103	8.491E-01	0.978	cis	SARS-CoV-2 infection
16825_20	ATX3	Ataxin-3	P54252	ATXN3	chr14:92106621	Inverse variance weighted	4	0.004	0.019	8.496E-01	0.978	cis	SARS-CoV-2 infection
4721_54	TFF3	Trefoil factor 3	Q07654	TFF3	chr21:42315409	Inverse variance weighted	3	-0.012	0.061	8.496E-01	0.978	cis	SARS-CoV-2 infection
3186_2	C2	Complement C2	P06681	C2	chr6:31897785	Inverse variance weighted	2	-0.024	0.124	8.496E-01	0.978	cis	SARS-CoV-2 infection
7761_125	CHKB	Choline/ethanolamine kinase	Q9Y259	CHKB	chr22:50601455	Inverse variance weighted	3	0.011	0.056	8.500E-01	0.978	cis	SARS-CoV-2 infection
13676_46	Inhibin bB chain	Inhibin beta B chain	P09529	INHBB	chr2:120346136	Inverse variance weighted	4	-0.003	0.014	8.502E-01	0.978	cis	SARS-CoV-2 infection
5803_24	C3d	Complement C3d fragment	P01024	C3	chr19:6730562	Wald ratio	1	-0.017	0.090	8.503E-01	0.978	cis	SARS-CoV-2 infection
7648_9	MYPC1	Myosin-binding protein C, slow-type	Q00872	MYBPC1	chr12:101568353	Wald ratio	1	-0.011	0.059	8.513E-01	0.978	cis	SARS-CoV-2 infection
7208_60	MGT4C	Alpha-1,3-mannosyl-glycoprotein 4-beta-N-acetylglucosaminyltransferase C	Q9UBM8	MGAT4C	chr12:86838904	Wald ratio	1	0.015	0.082	8.516E-01	0.978	cis	SARS-CoV-2 infection
2843_13	SPINT2	Kunitz-type protease inhibitor 2	O43291	SPINT2	chr19:38244035	Inverse variance weighted	7	0.003	0.015	8.516E-01	0.978	cis	SARS-CoV-2 infection
2480_58	TIMP-3	Metalloproteinase inhibitor 3	P35625	TIMP3	chr22:32801705	Inverse variance weighted	10	-0.002	0.012	8.521E-01	0.978	cis	SARS-CoV-2 infection
12382_2	DDX58	Probable ATP-dependent RNA helicase DDX58	O95786	DDX58	chr9:32526208	Inverse variance weighted	2	-0.008	0.045	8.526E-01	0.978	cis	SARS-CoV-2 infection
6580_29	Pregnancy zone protein	Pregnancy zone protein	P20742	PZP	chr12:9208395	Inverse variance weighted	2	0.009	0.049	8.526E-01	0.978	cis	SARS-CoV-2 infection
7009_8	CD72	B-cell differentiation antigen CD72	P21854	CD72	chr9:35646810	Inverse variance weighted	2	0.008	0.042	8.529E-01	0.978	cis	SARS-CoV-2 infection
14636_25	Ribonuclease UK114	Ribonuclease UK114	P52758	RIDA	chr8:98117171	Inverse variance weighted	3	-0.003	0.018	8.542E-01	0.978	cis	SARS-CoV-2 infection
6373_54	DLK1	Protein delta homolog 1	P80370	DLK1	chr14:100725705	Inverse variance weighted	2	-0.012	0.065	8.559E-01	0.978	cis	SARS-CoV-2 infection
12396_19	HIBCH	3-hydroxyisobutyryl-CoA hydrolase, mitochondrial	Q6NVY1	HIBCH	chr2:190344193	Inverse variance weighted	5	-0.002	0.014	8.560E-01	0.978	cis	SARS-CoV-2 infection
19187_21	STABP	STAM-binding protein	O95630	STAMBP	chr2:73828916	Inverse variance weighted	2	0.013	0.074	8.564E-01	0.978	cis	SARS-CoV-2 infection
8013_9	LMA2L	VIP36-like protein	Q9H0V9	LMAN2L	chr2:96740064	Inverse variance weighted	2	-0.013	0.073	8.572E-01	0.978	cis	SARS-CoV-2 infection
5452_71	ASGR1	Asialoglycoprotein receptor 1	P07306	ASGR1	chr17:7179564	Inverse variance weighted	2	-0.009	0.047	8.572E-01	0.978	cis	SARS-CoV-2 infection
9343_16	IL-2 sRb	Interleukin-2 receptor subunit beta	P14784	IL2RB	chr22:37175118	Wald ratio	1	0.009	0.049	8.576E-01	0.978	cis	SARS-CoV-2 infection
13982_33	RGS18	Regulator of G-protein signaling 18	Q9NS28	RGS18	chr1:192158462	Wald ratio	1	-0.015	0.083	8.578E-01	0.978	cis	SARS-CoV-2 infection
16599_38	GPN1	GPN-loop GTPase 1	Q9HCN4	GPN1	chr2:27628247	Wald ratio	1	0.011	0.064	8.590E-01	0.978	cis	SARS-CoV-2 infection
7227_75	COCH	Cochlin	O43405	COCH	chr14:30874514	Inverse variance weighted	4	0.003	0.018	8.595E-01	0.978	cis	SARS-CoV-2 infection
13088_397	BTC	Betacellulin	P35070	BTC	chr4:74794523	Inverse variance weighted	2	-0.007	0.037	8.596E-01	0.978	cis	SARS-CoV-2 infection

3024_18	a2-Antiplasmin	Alpha-2-antiplasmin	P08697	SERPINF2	chr17:1742836	Inverse variance weighted	3	0.005	0.030	8.597E-01	0.978	cis	SARS-CoV-2 infection
17773_26	SNAG	Gamma-soluble NSF attachment protein	Q99747	NAPG	chr18:10525905	Wald ratio	1	-0.012	0.066	8.601E-01	0.978	cis	SARS-CoV-2 infection
15615_8	LIRB3	Leukocyte immunoglobulin-like receptor subfamily B member 3	O75022	LILRB3	chr19:54223506	Inverse variance weighted	5	-0.006	0.037	8.604E-01	0.978	cis	SARS-CoV-2 infection
12524_18	SAT2	Diamine acetyltransferase 2	Q96F10	SAT2	chr17:7627876	Inverse variance weighted	3	0.006	0.033	8.610E-01	0.978	cis	SARS-CoV-2 infection
3535_84	DKK1	Dickkopf-related protein 1	O94907	DKK1	chr10:52314281	Inverse variance weighted	5	0.006	0.036	8.624E-01	0.978	cis	SARS-CoV-2 infection
8245_27	sICAM-5	Intercellular adhesion molecule 5	Q9UMF0	ICAM5	chr19:10289952	Inverse variance weighted	3	-0.009	0.052	8.625E-01	0.978	cis	SARS-CoV-2 infection
5631_83	MOTI	Promotilin	P12872	MLN	chr6:33804003	Inverse variance weighted	6	0.002	0.011	8.630E-01	0.978	cis	SARS-CoV-2 infection
13118_5	SMOC1	SPARC-related modular calcium-binding protein 1	Q9H4F8	SMOC1	chr14:69854131	Inverse variance weighted	3	0.005	0.030	8.631E-01	0.978	cis	SARS-CoV-2 infection
12714_38	AP1G2	AP-1 complex subunit gamma-like 2	O75843	AP1G2	chr14:23568070	Inverse variance weighted	3	0.005	0.028	8.634E-01	0.978	cis	SARS-CoV-2 infection
9565_6	PAWR	PRKC apoptosis WT1 regulator protein	Q96IZ0	PAWR	chr12:79690964	Wald ratio	1	-0.018	0.104	8.649E-01	0.978	cis	SARS-CoV-2 infection
11102_22	REG4	Regenerating islet-derived protein 4	Q9BYZ8	REG4	chr1:119811580	Inverse variance weighted	5	0.005	0.029	8.650E-01	0.978	cis	SARS-CoV-2 infection
8035_6	CA198	Uncharacterized protein C1orf198	Q9H425	C1orf198	chr1:230869589	Wald ratio	1	0.007	0.043	8.658E-01	0.978	cis	SARS-CoV-2 infection
12357_41	SNP29	Synaptosomal-associated protein 29	O95721	SNAP29	chr22:20859007	Wald ratio	1	0.012	0.073	8.658E-01	0.978	cis	SARS-CoV-2 infection
17746_77	FIS1	Mitochondrial fission 1 protein	Q9Y3D6	FIS1	chr7:101252316	Inverse variance weighted	2	-0.011	0.068	8.659E-01	0.978	cis	SARS-CoV-2 infection
8368_102	TNF sR-II	Tumor necrosis factor receptor superfamily member 1B	P20333	TNFRSF1B	chr1:12166991	Wald ratio	1	0.005	0.031	8.659E-01	0.978	cis	SARS-CoV-2 infection
5731_1	ISK6	Serine protease inhibitor Kazal-type 6	Q6UWN8	SPINK6	chr5:148202794	Inverse variance weighted	9	0.002	0.011	8.662E-01	0.978	cis	SARS-CoV-2 infection
5649_83	PSG4	Pregnancy-specific beta-1-glycoprotein 4	Q00888	PSG4	chr19:43207299	Inverse variance weighted	3	-0.009	0.056	8.664E-01	0.978	cis	SARS-CoV-2 infection
4471_50	TGM3	Protein-glutamine gamma-glutamyltransferase E	Q08188	TGM3	chr20:2296001	Inverse variance weighted	4	0.002	0.013	8.672E-01	0.978	cis	SARS-CoV-2 infection
5029_3	SEPR	Prolyl endopeptidase FAP	Q12884	FAP	chr2:162245151	Inverse variance weighted	5	0.004	0.024	8.674E-01	0.978	cis	SARS-CoV-2 infection
7128_9	VWA2	von Willebrand factor A domain-containing protein 2	Q5GFL6	VWA2	chr10:114239254	Inverse variance weighted	6	-0.003	0.019	8.685E-01	0.978	cis	SARS-CoV-2 infection
19496_1	NPL	N-acetylneuraminate lyase	Q9BXD5	NPL	chr1:182789293	Inverse variance weighted	3	-0.009	0.054	8.685E-01	0.978	cis	SARS-CoV-2 infection
2381_52	C5	Complement C5	P01031	C5	chr9:121075195	Inverse variance weighted	2	-0.008	0.052	8.697E-01	0.978	cis	SARS-CoV-2 infection
9796_4	CEL	Bile salt-activated lipase	P19835	CEL	chr9:133061981	Inverse variance weighted	5	-0.004	0.022	8.702E-01	0.978	cis	SARS-CoV-2 infection
3235_50	WFKN2	WAP, Kazal, immunoglobulin, Kunitz and NTR domain-containing protein 2	Q8TEU8	WFIKKN2	chr17:50834650	Inverse variance weighted	7	0.002	0.012	8.707E-01	0.978	cis	SARS-CoV-2 infection
13384_110	FUMH	Fumarate hydratase, mitochondrial	P07954	FH	chr1:241519799	Wald ratio	1	0.006	0.035	8.711E-01	0.978	cis	SARS-CoV-2 infection
9719_145	MMP-16	Matrix metalloproteinase-16	P51512	MMP16	chr8:88328025	Wald ratio	1	0.013	0.078	8.712E-01	0.978	cis	SARS-CoV-2 infection
3640_14	RAP	alpha-2-macroglobulin receptor-associated protein	P30533	LRPAP1	chr4:3532446	Inverse variance weighted	4	-0.004	0.022	8.712E-01	0.978	cis	SARS-CoV-2 infection
7935_26	LARGE	Glycosyltransferase-like protein LARGE1	O95461	LARGE1	chr22:33922841	Inverse variance weighted	2	0.007	0.040	8.714E-01	0.978	cis	SARS-CoV-2 infection
5139_32	UNC5H3	Netrin receptor UNC5C	O95185	UNC5C	chr4:95549206	Inverse variance weighted	3	0.004	0.026	8.735E-01	0.978	cis	SARS-CoV-2 infection
5353_89	IL-1Ra	Interleukin-1 receptor antagonist protein	P18510	IL1RN	chr2:113099315	Inverse variance weighted	3	0.006	0.035	8.737E-01	0.978	cis	SARS-CoV-2 infection
14655_1	DJC17	DnaJ homolog subfamily C member 17	Q9NVM6	DNAJC17	chr15:40807478	Inverse variance weighted	2	-0.012	0.079	8.739E-01	0.978	cis	SARS-CoV-2 infection
14271_23	RAB6B	Ras-related protein Rab-6B	Q9NRW1	RAB6B	chr3:133895882	Wald ratio	1	0.004	0.024	8.745E-01	0.978	cis	SARS-CoV-2 infection
5475_10	PKC-B-II	Protein kinase C beta type (splice variant beta-II)	P05771	PRKCB	chr16:23835983	Wald ratio	1	0.010	0.065	8.763E-01	0.978	cis	SARS-CoV-2 infection
9470_15	MET24	Methyltransferase-like protein 24	Q5JXM2	METTL24	chr6:110358349	Inverse variance weighted	4	-0.005	0.034	8.764E-01	0.978	cis	SARS-CoV-2 infection
8325_37	ADH4	Alcohol dehydrogenase 4	P08319	ADH4	chr4:99157792	Wald ratio	1	-0.009	0.060	8.767E-01	0.978	cis	SARS-CoV-2 infection
9388_18	MCEE	Methylmalonyl-CoA epimerase, mitochondrial	Q96PE7	MCEE	chr2:71130239	Wald ratio	1	-0.013	0.085	8.768E-01	0.978	cis	SARS-CoV-2 infection
15476_6	REG3G	Regenerating islet-derived protein 3-gamma	Q6UW15	REG3G	chr2:79025686	Inverse variance weighted	7	0.002	0.013	8.785E-01	0.978	cis	SARS-CoV-2 infection
9216_100	PLXB2	Plexin-B2	O15031	PLXNB2	chr22:50307646	Inverse variance weighted	5	-0.002	0.015	8.786E-01	0.978	cis	SARS-CoV-2 infection
4771_10	ASM3A	Acid sphingomyelinase-like phosphodiesterase 3a	Q92484	SMPDL3A	chr6:122789049	Inverse variance weighted	3	-0.003	0.022	8.786E-01	0.978	cis	SARS-CoV-2 infection
16079_2	TEC	Tyrosine-protein kinase Tec	P42680	TEC	chr4:48269838	Inverse variance weighted	2	-0.009	0.060	8.800E-01	0.978	cis	SARS-CoV-2 infection
15540_6	Vimentin	Vimentin	P08670	VIM	chr10:17228241	Inverse variance weighted	2	0.009	0.061	8.804E-01	0.978	cis	SARS-CoV-2 infection
9769_48	DNER:ECD	Delta and Notch-like epidermal growth factor-related receptor:Extracellular domain	Q8NFT8	DNER	chr2:229714555	Inverse variance weighted	5	-0.003	0.019	8.813E-01	0.978	cis	SARS-CoV-2 infection
6086_15	CRDL2	Chordin-like protein 2	Q6WN34	CHRDL2	chr11:74731426	Inverse variance weighted	5	0.002	0.015	8.820E-01	0.978	cis	SARS-CoV-2 infection
4924_32	MMP-1	Interstitial collagenase	P03956	MMP1	chr11:102798160	Inverse variance weighted	9	0.002	0.013	8.827E-01	0.978	cis	SARS-CoV-2 infection
4811_33	ITI heavy chain H4	Inter-alpha-trypsin inhibitor heavy chain H4	Q14624	ITIH4	chr3:52830688	Inverse variance weighted	2	-0.022	0.147	8.838E-01	0.978	cis	SARS-CoV-2 infection
3799_11	Carbonic anhydrase III	Carbonic anhydrase 3	P07451	CA3	chr8:85373436	Inverse variance weighted	4	-0.004	0.029	8.840E-01	0.978	cis	SARS-CoV-2 infection
6909_40	MGAT2	Alpha-1,6-mannosyl-glycoprotein 2-beta-N-acetylglucosaminyltransferase	Q10469	MGAT2	chr14:49620799	Wald ratio	1	-0.005	0.035	8.893E-01	0.978	cis	SARS-CoV-2 infection
8842_16	GRM1C	GRAM domain-containing protein 1C	Q8IYS0	GRAMD1C	chr3:113828182	Inverse variance weighted	5	0.006	0.044	8.895E-01	0.978	cis	SARS-CoV-2 infection
5634_39	OFUT1	GDP-fucose protein O-fucosyltransferase 1	Q9H488	POFUT1	chr20:32207855	Inverse variance weighted	3	0.002	0.017	8.899E-01	0.978	cis	SARS-CoV-2 infection
3435_53	FN1.4	Fibronectin Fragment 4	P02751	FN1	chr2:215436073	Wald ratio	1	-0.017	0.121	8.900E-01	0.978	cis	SARS-CoV-2 infection
2961_1	Protein C	Vitamin K-dependent protein C	P04070	PROC	chr2:127418427	Inverse variance weighted	3	0.007	0.051	8.907E-01	0.978	cis	SARS-CoV-2 infection
13427_66	MA1C1	Mannosyl-oligosaccharide 1,2-alpha-mannosidase IC	Q9NR34	MAN1C1	chr1:25616791	Inverse variance weighted	3	0.003	0.023	8.916E-01	0.978	cis	SARS-CoV-2 infection
9278_9	SDF-1	Stromal cell-derived factor 1	P48061	CXCL12	chr10:44386493	Inverse variance weighted	2	0.007	0.054	8.917E-01	0.978	cis	SARS-CoV-2 infection
9884_8	PPIL1	Peptidyl-prolyl cis-trans isomerase-like 1	Q9Y3C6	PPIL1	chr6:36874803	Inverse variance weighted	5	0.001	0.010	8.922E-01	0.978	cis	SARS-CoV-2 infection
6461_54	Apo C-III	Apolipoprotein C-III	P02656	APOC3	chr11:116829706	Inverse variance weighted	2	-0.008	0.056	8.924E-01	0.978	cis	SARS-CoV-2 infection
15499_11	Attractin	Attractin	O75882	ATRN	chr20:3471018	Inverse variance weighted	5	-0.002	0.018	8.926E-01	0.978	cis	SARS-CoV-2 infection
7163_26	lacritin	Extracellular glycoprotein lacritin	Q9GZZ8	LACRT	chr12:54634895	Wald ratio	1	0.014	0.107	8.927E-01	0.978	cis	SARS-CoV-2 infection
9197_4	LEG9	Galectin-9	O00182	LGALS9	chr17:27629798	Wald ratio	1	-0.006	0.044	8.930E-01	0.978	cis	SARS-CoV-2 infection
4160_49	MMP-2	72 kDa type IV collagenase	P08253	MMP2	chr16:55389700	Inverse variance weighted	2	-0.006	0.044	8.934E-01	0.978	cis	SARS-CoV-2 infection
8345_27	GPX7	Glutathione peroxidase 7	Q96SL4	GPX7	chr1:52602371	Inverse variance weighted	7	-0.001	0.011	8.936E-01	0.978	cis	SARS-CoV-2 infection
7875_86	PLEK	Pleckstrin	P08567	PLEK	chr2:68365282	Inverse variance weighted	2	-0.008	0.057	8.937E-01	0.978	cis	SARS-CoV-2 infection
3054_3	Haptoglobin, Mixed Type	Haptoglobin	P00738	HP	chr16:72054505	Inverse variance weighted	4	-0.002	0.016	8.949E-01	0.978	cis	SARS-CoV-2 infection
3481_87	XPNPEP1	Xaa-Pro aminopeptidase 1	Q9NQW7	XPNPEP1	chr10:109923553	Wald ratio	1	-0.010	0.074	8.957E-01	0.978	cis	SARS-CoV-2 infection
14133_93	IL-1 sRII	Interleukin-1 receptor type 2	P27930	IL1R2	chr2:101991960	Inverse variance weighted	7	0.003	0.020	8.965E-01	0.978	cis	SARS-CoV-2 infection
15506_34	LRP12	Low-density lipoprotein receptor-related protein 12	Q9Y561	LRP12	chr8:104589258	Inverse variance weighted	2	0.017	0.129	8.979E-01	0.978	cis	SARS-CoV-2 infection
9449_150	C4b-binding protein alpha chain	C4b-binding protein alpha chain	P04003	C4BPA	chr1:207104233	Inverse variance weighted	6	0.003	0.024	8.996E-01	0.978	cis	SARS-CoV-2 infection
9995_6	DUT	Deoxyuridine 5'-triphosphate nucleotidohydrolase, mitochondrial	P33316	DUT	chr15:48331011	Wald ratio	1	0.010	0.080	8.997E-01	0.978	cis	SARS-CoV-2 infection
17161_1	OST48	Dolichyl-diphosphooligosaccharide--protein glycosyltransferase 48 kDa subunit	P39656	DDOST	chr1:20661544	Inverse variance weighted	2	-0.002	0.015	9.001E-01	0.978	cis	SARS-CoV-2 infection
7779_86	CHSTB	Carbohydrate sulfotransferase 11	Q9NPF2	CHST11	chr12:104455295	Inverse variance weighted	5	0.002	0.015	9.004E-01	0.978	cis	SARS-CoV-2 infection
17787_1	Enoyl-CoA hydratase	Enoyl-CoA hydratase, mitochondrial	P30084	ECHS1	chr10:133373354	Wald ratio	1	-0.012	0.094	9.017E-01	0.978	cis	SARS-CoV-2 infection
9185_15	TFF1	Trefoil factor 1	P04155	TFF1	chr21:42366535	Inverse variance weighted	3	-0.002	0.017	9.020E-01	0.978	cis	SARS-CoV-2 infection
9870_17	SYWC	Tryptophan--tRNA ligase, cytoplasmic	P23381	WARS1	chr14:100376805	Inverse variance weighted	2	-0.005	0.037	9.020E-01	0.978	cis	SARS-CoV-2 infection
4159_130	Factor H	Complement factor H	P08603	CFH	chr1:196651754	Wald ratio	1	0.009	0.070	9.026E-01	0.978	cis	SARS-CoV-2 infection
15644_1	Biotinidase	Biotinidase	P43251	BTD	chr3:15601341	Inverse variance weighted	9	0.001	0.012	9.029E-01	0.978	cis	SARS-CoV-2 infection
19482_11	HDHD3	Haloacid dehalogenase-like hydrolase domain-containing protein 3	Q9BSH5	HDHD3	chr9:113376986	Wald ratio	1	-0.003	0.027	9.038E-01	0.978	cis	SARS-CoV-2 infection
15566_10	Calponin-1	Calponin-1	P51911	CNN1	chr19:11538767	Wald ratio	1	0.005	0.038	9.045E-01	0.978	cis	SARS-CoV-2 infection
2771_35	IGFBP-1	Insulin-like growth factor-binding protein 1	P08833	IGFBP1	chr7:45888360	Wald ratio	1	-0.007	0.062	9.050E-01	0.978	cis	SARS-CoV-2 infection
16296_43	LGR5	Leucine-rich repeat-containing G-protein coupled receptor 5	O75473	LGR5	chr12:71439798	Wald ratio	1	0.010	0.088	9.069E-01	0.978	cis	SARS-CoV-2 infection

3292_75	CD48	CD48 antigen	P09326	CD48	chr1:160711831	Inverse variance weighted	5	-0.003	0.025	9.071E-01	0.978	cis	SARS-CoV-2 infection
10419_1	SCAR5	Scavenger receptor class A member 5	Q6ZMJ2	SCARA5	chr8:27992673	Inverse variance weighted	5	-0.004	0.031	9.075E-01	0.978	cis	SARS-CoV-2 infection
3457_57	Periostin	Periostin	Q15063	POSTN	chr13:37598844	Inverse variance weighted	4	-0.003	0.028	9.077E-01	0.978	cis	SARS-CoV-2 infection
16890_37	ATL1	ADAMTS-like protein 1	Q8N6G6	ADAMTSL1	chr9:17906563	Inverse variance weighted	6	0.002	0.014	9.079E-01	0.978	cis	SARS-CoV-2 infection
5337_64	B7-2	T-lymphocyte activation antigen CD86	P42081	CD86	chr3:122055362	Wald ratio	1	0.010	0.084	9.087E-01	0.978	cis	SARS-CoV-2 infection
13959_7	LAP	Cytosol aminopeptidase	P28838	LAP3	chr4:17577198	Wald ratio	1	-0.012	0.104	9.092E-01	0.978	cis	SARS-CoV-2 infection
8053_16	DJB14:CD	DnaJ homolog subfamily B member 14:Cytoplasmic domain	Q8TBM8	DNAJB14	chr4:99946618	Wald ratio	1	0.009	0.079	9.093E-01	0.978	cis	SARS-CoV-2 infection
3470_1	sE-Selectin	E-selectin	P16581	SELE	chr1:169764705	Wald ratio	1	0.007	0.059	9.110E-01	0.978	cis	SARS-CoV-2 infection
6527_1	TRIL:ECD	TLR4 interactor with leucine rich repeats:Extracellular domain	Q7L0X0	TRIL	chr7:28958330	Wald ratio	1	0.007	0.062	9.110E-01	0.978	cis	SARS-CoV-2 infection
11540_37	FOXO3A	Forkhead box protein O3	O43524	FOXO3	chr6:108559835	Wald ratio	1	-0.010	0.093	9.119E-01	0.978	cis	SARS-CoV-2 infection
13095_51	PSP	Lithostathine-1-alpha	P05451	REG1A	chr2:79120362	Inverse variance weighted	4	-0.005	0.050	9.120E-01	0.978	cis	SARS-CoV-2 infection
5015_15	PAFAH	Platelet-activating factor acetylhydrolase	Q13093	PLA2G7	chr6:46735693	Inverse variance weighted	2	0.005	0.049	9.125E-01	0.978	cis	SARS-CoV-2 infection
19188_21	NAP2L	nucleosome assembly protein 1-like 4	Q99733	NAP1L4	chr11:2992377	Wald ratio	1	-0.003	0.031	9.126E-01	0.978	cis	SARS-CoV-2 infection
15626_223	Perlecan	Basement membrane-specific heparan sulfate proteoglycan core protein	P98160	HSPG2	chr1:21937310	Inverse variance weighted	4	-0.002	0.017	9.130E-01	0.978	cis	SARS-CoV-2 infection
17164_15	annexin IV	Annexin A4	P09525	ANXA4	chr2:69644425	Inverse variance weighted	2	-0.003	0.031	9.135E-01	0.978	cis	SARS-CoV-2 infection
17453_34	Ceruloplasmin	Ceruloplasmin	P00450	CP	chr3:149221829	Inverse variance weighted	3	0.004	0.035	9.145E-01	0.978	cis	SARS-CoV-2 infection
11333_82	RHG25	Rho GTPase-activating protein 25	P42331	ARHGAP25	chr2:68679601	Wald ratio	1	0.003	0.032	9.146E-01	0.978	cis	SARS-CoV-2 infection
7192_37	CRF2-12	Interferon lambda receptor 1	Q8IU57	IFNLR1	chr1:24187959	Inverse variance weighted	3	0.004	0.037	9.159E-01	0.978	cis	SARS-CoV-2 infection
9370_69	GGH	Gamma-glutamyl hydrolase	Q92820	GGH	chr8:63039407	Inverse variance weighted	5	0.001	0.014	9.160E-01	0.978	cis	SARS-CoV-2 infection
5021_13	PPase	Inorganic pyrophosphatase	Q15181	PPA1	chr10:70233911	Inverse variance weighted	4	0.003	0.029	9.160E-01	0.978	cis	SARS-CoV-2 infection
14076_74	Cystatin-S	Cystatin-S	P01036	CST4	chr20:23689038	Inverse variance weighted	3	-0.003	0.031	9.162E-01	0.978	cis	SARS-CoV-2 infection
9213_24	FTCD	Formimidoyltransferase-cyclodeaminase	O95954	FTCD	chr21:46155579	Inverse variance weighted	3	-0.005	0.046	9.173E-01	0.978	cis	SARS-CoV-2 infection
16607_78	Gelsolin	Gelsolin	P06396	GSN	chr9:121207794	Inverse variance weighted	2	-0.008	0.074	9.176E-01	0.978	cis	SARS-CoV-2 infection
4141_79	IP-10	C-X-C motif chemokine 10	P02778	CXCL10	chr4:76023497	Wald ratio	1	0.010	0.102	9.191E-01	0.978	cis	SARS-CoV-2 infection
13686_2	IL-5 Ra	Interleukin-5 receptor subunit alpha	Q01344	IL5RA	chr3:3126613	Inverse variance weighted	6	0.001	0.013	9.210E-01	0.978	cis	SARS-CoV-2 infection
7957_2	SCG3	Secretogranin-3	Q8WXD2	SCG3	chr15:51681492	Inverse variance weighted	7	0.001	0.013	9.213E-01	0.978	cis	SARS-CoV-2 infection
10630_5	HTAI2	Oxidoreductase HTATIP2	Q9BUP3	HTATIP2	chr11:20363685	Inverse variance weighted	9	0.001	0.014	9.234E-01	0.978	cis	SARS-CoV-2 infection
19602_36	jun-D	Transcription factor jun-D	P17535	JUND	chr19:18281622	Wald ratio	1	0.009	0.093	9.235E-01	0.978	cis	SARS-CoV-2 infection
7921_65	FJX1	Four-jointed box protein 1	Q86VR8	FJX1	chr11:35618460	Inverse variance weighted	4	-0.002	0.019	9.235E-01	0.978	cis	SARS-CoV-2 infection
8469_41	IGFBP-2	Insulin-like growth factor-binding protein 2	P18065	IGFBP2	chr2:216632828	Inverse variance weighted	2	-0.008	0.088	9.237E-01	0.978	cis	SARS-CoV-2 infection
14090_23	DEFI6	Differentially expressed in FDCP 6 homolog	Q9H4E7	DEF6	chr6:35297818	Inverse variance weighted	2	-0.003	0.028	9.238E-01	0.978	cis	SARS-CoV-2 infection
3440_7	granzyme A	Granzyme A	P12544	GZMA	chr5:55102646	Inverse variance weighted	4	-0.003	0.031	9.244E-01	0.978	cis	SARS-CoV-2 infection
2652_15	suPAR	Urokinase plasminogen activator surface receptor	Q03405	PLAUR	chr19:43670547	Inverse variance weighted	2	0.004	0.038	9.255E-01	0.978	cis	SARS-CoV-2 infection
18280_29	DTD2	Putative D-tyrosyl-tRNA(Tyr) deacylase 2	Q96FN9	DTD2	chr14:31457506	Inverse variance weighted	2	-0.004	0.039	9.260E-01	0.978	cis	SARS-CoV-2 infection
10835_25	A4GCT	Alpha-1,4-N-acetylglucosaminyltransferase	Q9UNA3	A4GNT	chr3:138132390	Wald ratio	1	0.007	0.075	9.267E-01	0.978	cis	SARS-CoV-2 infection
6077_63	CECR1	Adenosine deaminase CECR1	Q9NZK5	ADA2	chr22:17258235	Inverse variance weighted	5	-0.001	0.012	9.278E-01	0.978	cis	SARS-CoV-2 infection
10816_150	PILRA isoform FDF03-M14	Paired immunoglobulin-like type 2 receptor alpha isoform FDF03-M14	Q9UKJ1	PILRA	chr7:100367530	Inverse variance weighted	3	-0.004	0.046	9.279E-01	0.978	cis	SARS-CoV-2 infection
13123_3	FLRT3:ECD	Leucine-rich repeat transmembrane protein FLRT3:Extracellular domain	Q9NZU0	FLRT3	chr20:14337614	Inverse variance weighted	6	0.001	0.008	9.281E-01	0.978	cis	SARS-CoV-2 infection
8039_41	F177A	Protein FAM177A1	Q8N128	FAM177A1	chr14:35045117	Inverse variance weighted	6	0.001	0.012	9.282E-01	0.978	cis	SARS-CoV-2 infection
9282_12	CRIS2	Cysteine-rich secretory protein 2	P16562	CRISP2	chr6:49713590	Inverse variance weighted	6	-0.001	0.009	9.282E-01	0.978	cis	SARS-CoV-2 infection
2516_57	6Ckine	C-C motif chemokine 21	O00585	CCL21	chr9:34710136	Inverse variance weighted	2	0.004	0.040	9.283E-01	0.978	cis	SARS-CoV-2 infection
18397_5	AK1C4	Aldo-keto reductase family 1 member C4	P17516	AKR1C4	chr10:5195462	Inverse variance weighted	8	0.001	0.014	9.285E-01	0.978	cis	SARS-CoV-2 infection
19254_125	GMPR1	GMP reductase 1	P36959	GMPR	chr6:16238587	Inverse variance weighted	8	0.001	0.007	9.289E-01	0.978	cis	SARS-CoV-2 infection
8778_3	Noggin	Noggin	Q13253	NOG	chr17:56593699	Inverse variance weighted	5	-0.002	0.020	9.304E-01	0.978	cis	SARS-CoV-2 infection
7808_5	GLCE	D-glucuronyl C5-epimerase	O94923	GLCE	chr15:69160584	Inverse variance weighted	7	0.001	0.011	9.304E-01	0.978	cis	SARS-CoV-2 infection
9350_3	FSTL4	Follistatin-related protein 4	Q6MZW2	FSTL4	chr5:133612541	Inverse variance weighted	2	-0.005	0.062	9.306E-01	0.978	cis	SARS-CoV-2 infection
8606_39	GPNMB:CD	Transmembrane glycoprotein NMB:Cytoplasmic domain	Q14956	GPNMB	chr7:23235967	Inverse variance weighted	7	0.001	0.014	9.315E-01	0.978	cis	SARS-CoV-2 infection
3221_54	SARP-2	Secreted frizzled-related protein 1	Q8N474	SFRP1	chr8:41309473	Inverse variance weighted	3	-0.002	0.022	9.317E-01	0.978	cis	SARS-CoV-2 infection
11428_31	PDLI1	PDZ and LIM domain protein 1	O00151	PDLIM1	chr10:95291012	Wald ratio	1	-0.006	0.066	9.318E-01	0.978	cis	SARS-CoV-2 infection
17702_53	UGT 1A1	UDP-glucuronosyltransferase 1-1	P22309	UGT1A1	chr2:233760270	Inverse variance weighted	2	0.007	0.083	9.323E-01	0.978	cis	SARS-CoV-2 infection
8274_64	Syntaxin-7	Syntaxin-7	O15400	STX7	chr6:132513198	Wald ratio	1	-0.003	0.040	9.324E-01	0.978	cis	SARS-CoV-2 infection
4133_54	Granzyme B	Granzyme B	P10144	GZMB	chr14:24634267	Wald ratio	1	0.004	0.053	9.325E-01	0.978	cis	SARS-CoV-2 infection
11140_56	CO1A1:C-term propeptide	Collagen alpha-1(I) chain:C-term propeptide	P02452	COL1A1	chr17:50201632	Inverse variance weighted	3	0.003	0.030	9.330E-01	0.978	cis	SARS-CoV-2 infection
3367_8	FETUB	Fetuin-B	Q9UGM5	FETUB	chr3:186635969	Inverse variance weighted	5	-0.002	0.026	9.331E-01	0.978	cis	SARS-CoV-2 infection
19293_6	VP26A	Vacuolar protein sorting-associated protein 26A	O75436	VPS26A	chr10:69123512	Wald ratio	1	0.002	0.030	9.332E-01	0.978	cis	SARS-CoV-2 infection
5508_62	Cathepsin D	Cathepsin D	P07339	CTSD	chr11:1764573	Inverse variance weighted	5	0.002	0.019	9.338E-01	0.978	cis	SARS-CoV-2 infection
12646_2	RPE	Ribulose-phosphate 3-epimerase	Q96AT9	RPE	chr2:210002565	Wald ratio	1	0.005	0.061	9.341E-01	0.978	cis	SARS-CoV-2 infection
3210_1	METAP1	Methionine aminopeptidase 1	P53582	METAP1	chr4:98995659	Wald ratio	1	0.008	0.097	9.350E-01	0.978	cis	SARS-CoV-2 infection
3173_49	ASAHL	N-acylethanolamine-hydrolyzing acid amidase	Q02083	NAAA	chr4:75941013	Inverse variance weighted	10	0.002	0.019	9.350E-01	0.978	cis	SARS-CoV-2 infection
10714_7	ACE	Angiotensin-converting enzyme	P12821	ACE	chr17:63477061	Inverse variance weighted	11	-0.001	0.008	9.371E-01	0.980	cis	SARS-CoV-2 infection
11681_8	NUPL	Arf-GAP domain and FG repeat-containing protein 1	P52594	AGFG1	chr2:227472152	Wald ratio	1	0.004	0.054	9.387E-01	0.981	cis	SARS-CoV-2 infection
15635_4	SMOC2	SPARC-related modular calcium-binding protein 2	Q9H3U7	SMOC2	chr6:168441151	Inverse variance weighted	5	0.001	0.015	9.403E-01	0.982	cis	SARS-CoV-2 infection
6448_36	Sema E	Semaphorin-3C	Q99985	SEMA3C	chr7:80922359	Inverse variance weighted	2	0.003	0.040	9.411E-01	0.982	cis	SARS-CoV-2 infection
13954_9	GNA1	Glucosamine 6-phosphate N-acetyltransferase	Q96EK6	GNPNAT1	chr14:52791668	Wald ratio	1	-0.006	0.083	9.421E-01	0.982	cis	SARS-CoV-2 infection
5112_73	OX2G	OX-2 membrane glycoprotein	P41217	CD200	chr3:112332347	Wald ratio	1	0.004	0.060	9.436E-01	0.983	cis	SARS-CoV-2 infection
6925_26	SNX8	Sorting nexin-8	Q9Y5X2	SNX8	chr7:2354318	Inverse variance weighted	2	-0.004	0.058	9.453E-01	0.984	cis	SARS-CoV-2 infection
9335_28	PSG9	Pregnancy-specific beta-1-glycoprotein 9	Q00887	PSG9	chr19:43269530	Wald ratio	1	-0.004	0.064	9.461E-01	0.985	cis	SARS-CoV-2 infection
12469_19	MARE1	Microtubule-associated protein RP/EB family member 1	Q15691	MAPRE1	chr20:32819954	Wald ratio	1	-0.006	0.092	9.469E-01	0.985	cis	SARS-CoV-2 infection
5129_12	SREC-I	Scavenger receptor class F member 1	Q14162	SCARF1	chr17:1645744	Inverse variance weighted	5	0.001	0.017	9.485E-01	0.986	cis	SARS-CoV-2 infection
11378_37	KSYK:SH2, 1 and 2	Tyrosine-protein kinase SYK:Src Homology domain	P43405	SYK	chr9:90801787	Wald ratio	1	0.006	0.101	9.494E-01	0.986	cis	SARS-CoV-2 infection
6451_64	ASPN	Asporin	Q9BXN1	ASPN	chr9:92482506	Inverse variance weighted	8	-0.002	0.027	9.495E-01	0.986	cis	SARS-CoV-2 infection
17170_15	CALCB	Calcitonin gene-related peptide 2	P10092	CALCB	chr11:14904997	Inverse variance weighted	2	-0.001	0.021	9.508E-01	0.986	cis	SARS-CoV-2 infection
5963_9	Dermokine	Dermokine	Q6E0U4	DMKN	chr19:35513658	Wald ratio	1	0.005	0.082	9.529E-01	0.986	cis	SARS-CoV-2 infection
19561_216	PLXD1	Plexin-D1	Q9Y4D7	PLXND1	chr3:129606676	Inverse variance weighted	4	0.001	0.021	9.535E-01	0.986	cis	SARS-CoV-2 infection
7628_40	CREL1	Cysteine-rich with EGF-like domain protein 1	Q96HD1	CRELD1	chr3:9933793	Inverse variance weighted	12	0.001	0.010	9.546E-01	0.986	cis	SARS-CoV-2 infection
14273_19	PPCE	Prolyl endopeptidase	P48147	PREP	chr6:105454062	Inverse variance weighted	2	0.002	0.028	9.546E-01	0.986	cis	SARS-CoV-2 infection
5688_65	CBLN4	Cerebellin-4	Q9NTU7	CBLN4	chr20:56005519	Inverse variance weighted	5	-0.001	0.022	9.571E-01	0.986	cis	SARS-CoV-2 infection
17692_2	BT3A3	Butyrophilin subfamily 3 member A3	O00478	BTN3A3	chr6:26440472	Inverse variance weighted	10	0.000	0.009	9.579E-01	0.986	cis	SARS-CoV-2 infection

9339_204	FKBP2	Peptidyl-prolyl cis-trans isomerase FKBP2	P26885	FKBP2	chr11:64241003	Wald ratio	1	-0.005	0.098	9.579E-01	0.986	cis	SARS-CoV-2 infection
2602_2	Angiopoietin-2	Angiopoietin-2	O15123	ANGPT2	chr8:6563409	Inverse variance weighted	3	0.002	0.035	9.587E-01	0.986	cis	SARS-CoV-2 infection
3583_54	Arylsulfatase A	Arylsulfatase A	P15289	ARSA	chr22:50628173	Wald ratio	1	-0.003	0.061	9.591E-01	0.986	cis	SARS-CoV-2 infection
19557_3	KLOTB	Beta-klotho	Q86Z14	KLB	chr4:39406930	Inverse variance weighted	8	-0.001	0.010	9.592E-01	0.986	cis	SARS-CoV-2 infection
18408_26	ARF4	ADP-ribosylation factor 4	P18085	ARF4	chr3:57598220	Wald ratio	1	-0.003	0.067	9.594E-01	0.986	cis	SARS-CoV-2 infection
4192_10	AK1A1	Alcohol dehydrogenase [NADP(+)]	P14550	AKR1A1	chr1:45550543	Inverse variance weighted	6	0.001	0.012	9.598E-01	0.986	cis	SARS-CoV-2 infection
9940_35	DUS28	Dual specificity phosphatase 28	Q4G0W2	DUSP28	chr2:240560054	Inverse variance weighted	2	0.002	0.042	9.599E-01	0.986	cis	SARS-CoV-2 infection
5463_22	GAS1	Growth arrest-specific protein 1	P54826	GAS1	chr9:86947506	Inverse variance weighted	2	-0.002	0.042	9.600E-01	0.986	cis	SARS-CoV-2 infection
3396_54	Renin	Renin	P00797	REN	chr1:204190324	Inverse variance weighted	3	0.003	0.061	9.602E-01	0.986	cis	SARS-CoV-2 infection
17764_108	RHOC	Rho-related GTP-binding protein RhoC	P08134	RHOC	chr1:112707434	Wald ratio	1	-0.002	0.045	9.618E-01	0.986	cis	SARS-CoV-2 infection
6223_5	GUC2B	Guanylate cyclase activator 2B	Q16661	GUCA2B	chr1:42153410	Inverse variance weighted	2	-0.002	0.051	9.626E-01	0.986	cis	SARS-CoV-2 infection
9385_4	GAA	Lysosomal alpha-glucosidase	P10253	GAA	chr17:80101556	Inverse variance weighted	7	-0.001	0.015	9.630E-01	0.986	cis	SARS-CoV-2 infection
17799_9	6PGL	6-phosphogluconolactonase	O95336	PGLS	chr19:17511636	Inverse variance weighted	3	0.002	0.042	9.630E-01	0.986	cis	SARS-CoV-2 infection
18243_9	Cytidylate kinase	UMP-CMP kinase	P30085	CMPK1	chr1:47333790	Wald ratio	1	0.003	0.064	9.637E-01	0.986	cis	SARS-CoV-2 infection
13740_51	sFRP-3	Secreted frizzled-related protein 3	Q92765	FRZB	chr2:182866637	Inverse variance weighted	8	0.000	0.010	9.637E-01	0.986	cis	SARS-CoV-2 infection
3000_66	MBL	Mannose-binding protein C	P11226	MBL2	chr10:52772784	Inverse variance weighted	9	0.000	0.008	9.646E-01	0.986	cis	SARS-CoV-2 infection
3291_30	CD23	Low affinity immunoglobulin epsilon Fc receptor	P06734	FCER2	chr19:7702146	Inverse variance weighted	7	0.001	0.016	9.651E-01	0.986	cis	SARS-CoV-2 infection
10070_22	PLCG2	1-phosphatidylinositol 4,5-bisphosphate phosphodiesterase gamma-2	P16885	PLCG2	chr16:81779279	Inverse variance weighted	2	-0.002	0.050	9.659E-01	0.986	cis	SARS-CoV-2 infection
3728_52	Secretin	Secretin	P09683	SCT	chr11:627181	Wald ratio	1	-0.002	0.052	9.671E-01	0.986	cis	SARS-CoV-2 infection
7923_41	SEM4C	Semaphorin-4C	Q9C0C4	SEMA4C	chr2:96870757	Inverse variance weighted	2	0.001	0.035	9.679E-01	0.986	cis	SARS-CoV-2 infection
9233_71	TFPI -2	Tissue factor pathway inhibitor 2	P48307	TFPI2	chr7:93890753	Wald ratio	1	0.004	0.098	9.690E-01	0.986	cis	SARS-CoV-2 infection
5060_62	B7-H1	Programmed cell death 1 ligand 1	Q9NZQ7	CD274	chr9:5450503	Inverse variance weighted	2	-0.001	0.025	9.695E-01	0.986	cis	SARS-CoV-2 infection
10451_11	NUCB1	Nucleobindin-1	Q02818	NUCB1	chr19:48900050	Inverse variance weighted	3	-0.002	0.063	9.698E-01	0.986	cis	SARS-CoV-2 infection
18313_4	Asparaginase-like protein 1	Isoaspartyl peptidase/L-asparaginase	Q7L266	ASRGL1	chr11:62337448	Inverse variance weighted	2	0.001	0.023	9.701E-01	0.986	cis	SARS-CoV-2 infection
15573_110	CSPG3	Neurocan core protein	O14594	NCAN	chr19:19211958	Inverse variance weighted	2	-0.002	0.054	9.705E-01	0.986	cis	SARS-CoV-2 infection
17148_7	BLVRB	Flavin reductase (NADPH)	P30043	BLVRB	chr19:40465764	Inverse variance weighted	3	-0.001	0.023	9.714E-01	0.986	cis	SARS-CoV-2 infection
9790_28	BR serine/threonine kinase 2	Serine/threonine-protein kinase BRSK2	Q8IWQ3	BRSK2	chr11:1389899	Inverse variance weighted	2	0.001	0.029	9.732E-01	0.986	cis	SARS-CoV-2 infection
5671_1	Chymotrypsin	Chymotrypsinogen B	P17538	CTRB1	chr16:75218988	Inverse variance weighted	6	-0.001	0.030	9.734E-01	0.986	cis	SARS-CoV-2 infection
10907_116	NTRI	Neurotrimin	Q9P121	NTM	chr11:131370478	Inverse variance weighted	2	0.002	0.058	9.736E-01	0.986	cis	SARS-CoV-2 infection
7145_1	ITIH3	Inter-alpha-trypsin inhibitor heavy chain H3	Q06033	ITIH3	chr3:52794768	Inverse variance weighted	10	-0.001	0.017	9.737E-01	0.986	cis	SARS-CoV-2 infection
4500_50	SCGF-alpha	Stem cell growth factor-alpha	Q9Y240	CLEC11A	chr19:50723364	Inverse variance weighted	2	0.000	0.014	9.741E-01	0.986	cis	SARS-CoV-2 infection
17706_4	PPR1A	Protein phosphatase 1 regulatory subunit 1A	Q13522	PPP1R1A	chr12:54588659	Wald ratio	1	0.003	0.087	9.748E-01	0.986	cis	SARS-CoV-2 infection
3419_49	CAMK2D	Calcium/calmodulin-dependent protein kinase type II subunit delta	Q13557	CAMK2D	chr4:113761927	Wald ratio	1	-0.003	0.106	9.754E-01	0.986	cis	SARS-CoV-2 infection
15466_30	CO9A1	Collagen alpha-1(IX) chain	P20849	COL9A1	chr6:70303084	Inverse variance weighted	2	0.002	0.056	9.766E-01	0.986	cis	SARS-CoV-2 infection
8007_19	Cathepsin B	Cathepsin B	P07858	CTSB	chr8:11869533	Inverse variance weighted	6	0.000	0.010	9.774E-01	0.986	cis	SARS-CoV-2 infection
6415_90	CPN2	Carboxypeptidase N subunit 2	P22792	CPN2	chr3:194351328	Inverse variance weighted	6	0.000	0.017	9.786E-01	0.986	cis	SARS-CoV-2 infection
7655_11	N-terminal pro-BNP	N-terminal pro-BNP	P16860	NPPB	chr1:11858945	Inverse variance weighted	3	-0.001	0.023	9.787E-01	0.986	cis	SARS-CoV-2 infection
11360_39	RRM1	Ribonucleoside-diphosphate reductase large subunit	P23921	RRM1	chr11:4094707	Wald ratio	1	-0.002	0.091	9.787E-01	0.986	cis	SARS-CoV-2 infection
7822_11	HRSL2	HRAS-like suppressor 2	Q9NWW9	PLAAT2	chr11:63563379	Wald ratio	1	-0.003	0.100	9.790E-01	0.986	cis	SARS-CoV-2 infection
16304_6	LGR4	Leucine-rich repeat-containing G-protein coupled receptor 4	Q9BXB1	LGR4	chr11:27472790	Inverse variance weighted	2	-0.001	0.043	9.790E-01	0.986	cis	SARS-CoV-2 infection
5742_14	PPA6	Lysophosphatidic acid phosphatase type 6	Q9NPH0	ACP6	chr1:147670524	Inverse variance weighted	14	0.000	0.007	9.800E-01	0.986	cis	SARS-CoV-2 infection
17738_7	CRNN	Cornulin	Q9UBG3	CRNN	chr1:152414263	Wald ratio	1	-0.001	0.037	9.800E-01	0.986	cis	SARS-CoV-2 infection
15610_72	LAP	Cytosol aminopeptidase	P28838	LAP3	chr4:17577198	Wald ratio	1	0.002	0.097	9.814E-01	0.986	cis	SARS-CoV-2 infection
17377_1	Aldose reductase-like C3	Aldo-keto reductase family 1 member C3	P42330	AKR1C3	chr10:5035354	Inverse variance weighted	4	0.001	0.045	9.819E-01	0.986	cis	SARS-CoV-2 infection
6060_2	PIP	Prolactin-inducible protein	P12273	PIP	chr7:143132077	Inverse variance weighted	6	0.000	0.011	9.822E-01	0.986	cis	SARS-CoV-2 infection
9212_22	CATF	Cathepsin F	Q9UBX1	CTSF	chr11:66568879	Inverse variance weighted	2	0.001	0.049	9.832E-01	0.986	cis	SARS-CoV-2 infection
11103_24	HSP 27	Heat shock protein beta-1	P04792	HSPB1	chr7:76302673	Inverse variance weighted	4	0.000	0.014	9.837E-01	0.986	cis	SARS-CoV-2 infection
5089_11	IL-7 Ra	Interleukin-7 receptor subunit alpha	P16871	IL7R	chr5:35852695	Wald ratio	1	-0.002	0.081	9.839E-01	0.986	cis	SARS-CoV-2 infection
4991_12	GPC5	Glypican-5	P78333	GPC5	chr13:91398621	Inverse variance weighted	6	0.000	0.012	9.839E-01	0.986	cis	SARS-CoV-2 infection
11708_2	LCN1	Lipocalin-1	P31025	LCN1	chr9:135521438	Wald ratio	1	-0.001	0.071	9.856E-01	0.986	cis	SARS-CoV-2 infection
10722_13	KSYK:Protein Kinase	Tyrosine-protein kinase SYK:Protein kinase domain	P43405	SYK	chr9:90801787	Wald ratio	1	-0.002	0.093	9.861E-01	0.986	cis	SARS-CoV-2 infection
17151_84	IRF-3	Interferon regulatory factor 3	Q14653	IRF3	chr19:49665875	Wald ratio	1	0.001	0.066	9.867E-01	0.986	cis	SARS-CoV-2 infection
16288_17	EPHA4	Ephrin type-A receptor 4	P54764	EPHA4	chr2:221574202	Inverse variance weighted	3	0.000	0.028	9.868E-01	0.986	cis	SARS-CoV-2 infection
13973_62	TTL	Tubulin--tyrosine ligase	Q8NG68	TTL	chr2:112482156	Wald ratio	1	0.001	0.090	9.870E-01	0.986	cis	SARS-CoV-2 infection
15326_64	GBP1	Guanylate-binding protein 1	P32455	GBP1	chr1:89065360	Inverse variance weighted	3	0.000	0.029	9.874E-01	0.986	cis	SARS-CoV-2 infection
14009_65	TNFAIP3	Tumor necrosis factor alpha-induced protein 3	P21580	TNFAIP3	chr6:137867214	Wald ratio	1	0.000	0.032	9.877E-01	0.986	cis	SARS-CoV-2 infection
14708_59	CO8G	Complement component C8 gamma chain	P07360	C8G	chr9:136945185	Inverse variance weighted	5	0.000	0.029	9.883E-01	0.986	cis	SARS-CoV-2 infection
10088_37	APT	Adenine phosphoribosyltransferase	P07741	APRT	chr16:88811937	Inverse variance weighted	4	0.000	0.026	9.890E-01	0.986	cis	SARS-CoV-2 infection
17832_12	IDI2	Isopentenyl-diphosphate delta-isomerase 2	Q9BXS1	IDI2	chr10:1025859	Inverse variance weighted	2	-0.001	0.046	9.896E-01	0.986	cis	SARS-CoV-2 infection
6379_62	ATL2	ADAMTS-like protein 2	Q86TH1	ADAMTSL2	chr9:133532164	Inverse variance weighted	2	-0.001	0.046	9.910E-01	0.986	cis	SARS-CoV-2 infection
2620_4	gp130, soluble	Interleukin-6 receptor subunit beta	P40189	IL6ST	chr5:55995022	Inverse variance weighted	4	0.000	0.017	9.917E-01	0.986	cis	SARS-CoV-2 infection
11152_46	kallikrein 13	Kallikrein-13	Q9UKR3	KLK13	chr19:51065114	Wald ratio	1	0.001	0.062	9.921E-01	0.986	cis	SARS-CoV-2 infection
2836_68	Lipocalin 2	Neutrophil gelatinase-associated lipocalin	P80188	LCN2	chr9:128149071	Wald ratio	1	-0.001	0.071	9.929E-01	0.986	cis	SARS-CoV-2 infection
12428_2	LYPL1	Lysophospholipase-like protein 1	Q5VWZ2	LYPLAL1	chr1:219173869	Wald ratio	1	0.000	0.048	9.931E-01	0.986	cis	SARS-CoV-2 infection
19361_78	MATN3	Matrilin-3	O15232	MATN3	chr2:20012668	Inverse variance weighted	12	0.000	0.009	9.937E-01	0.986	cis	SARS-CoV-2 infection
8983_7	GOLM1	Golgi membrane protein 1	Q8NBJ4	GOLM1	chr9:86100173	Wald ratio	1	0.001	0.109	9.938E-01	0.986	cis	SARS-CoV-2 infection
11266_8	SELPL:ECD	P-selectin glycoprotein ligand 1:Extracellular domain	Q14242	SELPLG	chr12:108633894	Inverse variance weighted	2	0.000	0.031	9.940E-01	0.986	cis	SARS-CoV-2 infection
10851_77	IL27B	Interleukin-27 subunit beta	Q14213	EBI3	chr19:4229523	Inverse variance weighted	7	0.000	0.008	9.943E-01	0.986	cis	SARS-CoV-2 infection
7866_11	DJC30	DnaJ homolog subfamily C member 30	Q96LL9	DNAJC30	chr7:73683453	Inverse variance weighted	2	0.000	0.033	9.953E-01	0.986	cis	SARS-CoV-2 infection
17675_17	ACO13	Acyl-coenzyme A thioesterase 13	Q9NPJ3	ACOT13	chr6:24667035	Inverse variance weighted	2	0.000	0.059	9.954E-01	0.986	cis	SARS-CoV-2 infection
15653_9	COAA1	Collagen alpha-1(X) chain	Q03692	COL10A1	chr6:116158747	Inverse variance weighted	3	0.000	0.031	9.973E-01	0.987	cis	SARS-CoV-2 infection
13597_20	RAB31	Ras-related protein Rab-31	Q13636	RAB31	chr18:9708275	Inverse variance weighted	3	0.000	0.037	9.977E-01	0.987	cis	SARS-CoV-2 infection
9267_2	CBPA4	Carboxypeptidase A4	Q9UI42	CPA4	chr7:130293134	Inverse variance weighted	12	0.000	0.006	9.991E-01	0.988	cis	SARS-CoV-2 infection
4440_15	FCRL3	Fc receptor-like protein 3	Q96P31	FCRL3	chr1:157700769	Inverse variance weighted	4	0.000	0.056	9.996E-01	0.988	cis	SARS-CoV-2 infection
18220_141	SRA1	Steroid receptor RNA activator 1	Q9HD15	SRA1	chr5:140557677	Inverse variance weighted	2	0.000	0.057	1.000E+00	0.988	cis	SARS-CoV-2 infection
10346_5	STAT3	Signal transducer and activator of transcription 3	P40763	STAT3	NA	Inverse variance weighted	2	-0.538	0.123	1.232E-05	0.026	pan	Severe COVID-19
19196_73	HOP	Homeodomain-only protein	Q9BPY8	HOPX	NA	Wald ratio	1	-0.689	0.170	5.201E-05	0.056	pan	Severe COVID-19
12387_7	PDLI4	PDZ and LIM domain protein 4	P50479	PDLIM4	NA	Wald ratio	1	-0.413	0.115	3.383E-04	0.242	pan	Severe COVID-19

6404_20	C1QRF	C1q-related factor	O75973	C1QL1	NA	Inverse variance weighted	7	-0.191	0.057	7.413E-04	0.398	pan	Severe COVID-19
17769_28	PCNP	PEST proteolytic signal-containing nuclear protein	Q8WW12	PCNP	NA	Wald ratio	1	-0.549	0.170	1.255E-03	0.459	pan	Severe COVID-19
12534_10	CACO2	Calcium-binding and coiled-coil domain-containing protein 2	Q13137	CALCOCO2	NA	Wald ratio	1	-0.541	0.170	1.455E-03	0.459	pan	Severe COVID-19
10554_23	BGAL	Beta-galactosidase	P16278	GLB1	NA	Wald ratio	1	1.060	0.337	1.662E-03	0.459	pan	Severe COVID-19
2737_22	NovH	Protein NOV homolog	P48745	CCN3	NA	Wald ratio	1	1.103	0.352	1.710E-03	0.459	pan	Severe COVID-19
8255_34	MRVI1	Protein MRVI1	Q9Y6F6	IRAG1	NA	Inverse variance weighted	2	-0.380	0.124	2.121E-03	0.476	pan	Severe COVID-19
6955_68	SNX1	Sorting nexin-1	Q13596	SNX1	NA	Wald ratio	1	-0.348	0.114	2.214E-03	0.476	pan	Severe COVID-19
9017_58	LPH	Lactase-phlorizin hydrolase	P09848	LCT	NA	Inverse variance weighted	14	-0.079	0.026	2.622E-03	0.512	pan	Severe COVID-19
9834_62	ADH1B	Alcohol dehydrogenase 1B	P00325	ADH1B	NA	Wald ratio	1	0.395	0.135	3.365E-03	0.602	pan	Severe COVID-19
18380_78	Albumin	Serum albumin	P02768	ALB	NA	Inverse variance weighted	2	0.744	0.257	3.775E-03	0.624	pan	Severe COVID-19
19446_1	GMPR2	GMP reductase 2	Q9P2T1	GMPR2	NA	Inverse variance weighted	2	0.195	0.069	4.549E-03	0.630	pan	Severe COVID-19
10440_26	ACAM:ECD	CXADR-like membrane protein:Extracellular domain	Q9H6B4	CLMP	NA	Inverse variance weighted	3	0.201	0.071	4.689E-03	0.630	pan	Severe COVID-19
4541_49	CDON	Cell adhesion molecule-related/down-regulated by oncogenes	Q4KMG0	CDON	NA	Inverse variance weighted	5	0.111	0.039	5.017E-03	0.630	pan	Severe COVID-19
13434_172	PARVA	Alpha-parvin	Q9NVD7	PARVA	NA	Wald ratio	1	-0.475	0.172	5.659E-03	0.630	pan	Severe COVID-19
10560_1	ITM2C:N-term	Integral membrane protein 2C:N-term	Q9NQX7	ITM2C	NA	Wald ratio	1	1.011	0.366	5.743E-03	0.630	pan	Severe COVID-19
10008_43	GUC1A	Guanylyl cyclase-activating protein 1	P43080	GUCA1A	NA	Inverse variance weighted	2	0.907	0.332	6.328E-03	0.630	pan	Severe COVID-19
12820_1	GRAP	GRB2-related adapter protein	Q13588	GRAP	NA	Inverse variance weighted	2	-0.443	0.164	7.006E-03	0.630	pan	Severe COVID-19
6528_95	EXTL2	Exostosin-like 2	Q9UBQ6	EXTL2	NA	Wald ratio	1	0.853	0.317	7.196E-03	0.630	pan	Severe COVID-19
8464_31	RSPO4	R-spondin-4	Q2I0M5	RSPO4	NA	Inverse variance weighted	3	0.381	0.142	7.203E-03	0.630	pan	Severe COVID-19
10620_21	PSP-94	Beta-microseminoprotein	P08118	MSMB	NA	Inverse variance weighted	19	-0.062	0.023	7.554E-03	0.630	pan	Severe COVID-19
9590_10	COX7R	Cytochrome c oxidase subunit 7A-related protein, mitochondrial	O14548	COX7A2L	NA	Wald ratio	1	0.962	0.360	7.575E-03	0.630	pan	Severe COVID-19
6930_95	SIA8F	Alpha-2,8-sialyltransferase 8F	P61647	ST8SIA6	NA	Wald ratio	1	-0.808	0.303	7.670E-03	0.630	pan	Severe COVID-19
8923_94	GLTL1	Polypeptide N-acetylgalactosaminyltransferase 16	Q8N428	GALNT16	NA	Inverse variance weighted	4	-0.201	0.076	8.165E-03	0.630	pan	Severe COVID-19
3708_62	a2-Macroglobulin	Alpha-2-macroglobulin	P01023	A2M	NA	Inverse variance weighted	2	-0.488	0.185	8.271E-03	0.630	pan	Severe COVID-19
16613_3	CAD17	Cadherin-17	Q12864	CDH17	NA	Inverse variance weighted	16	0.113	0.043	8.698E-03	0.630	pan	Severe COVID-19
10722_13	KSYK:Protein Kinase	Tyrosine-protein kinase SYK:Protein kinase domain	P43405	SYK	NA	Wald ratio	1	0.751	0.288	9.213E-03	0.630	pan	Severe COVID-19
3009_3	TGF-b R III	Transforming growth factor beta receptor type 3	Q03167	TGFBR3	NA	Inverse variance weighted	6	-0.199	0.077	9.491E-03	0.630	pan	Severe COVID-19
15339_32	COF2	Cofilin-2	Q9Y281	CFL2	NA	Wald ratio	1	0.328	0.127	1.013E-02	0.630	pan	Severe COVID-19
13992_12	NSF	Vesicle-fusing ATPase	P46459	NSF	NA	Wald ratio	1	-0.683	0.266	1.031E-02	0.630	pan	Severe COVID-19
8768_4	Bcl-10	B-cell lymphoma/leukemia 10	O95999	BCL10	NA	Wald ratio	1	1.040	0.406	1.042E-02	0.630	pan	Severe COVID-19
9359_9	EGFL9	Protein delta homolog 2	Q6UY11	DLK2	NA	Wald ratio	1	-0.224	0.087	1.058E-02	0.630	pan	Severe COVID-19
16792_4	Siglec-5	Sialic acid-binding Ig-like lectin 5	O15389	SIGLEC5	NA	Inverse variance weighted	7	0.110	0.043	1.082E-02	0.630	pan	Severe COVID-19
14007_22	PAPS1	Bifunctional 3'-phosphoadenosine 5'-phosphosulfate synthase 1	O43252	PAPSS1	NA	Inverse variance weighted	2	-0.215	0.085	1.096E-02	0.630	pan	Severe COVID-19
15529_33	Cysteine-rich protein 1	Cysteine and glycine-rich protein 1	P21291	CSRP1	NA	Inverse variance weighted	2	-0.523	0.206	1.108E-02	0.630	pan	Severe COVID-19
8100_15	ADM2	ADM2	Q7Z4H4	ADM2	NA	Wald ratio	1	0.981	0.389	1.177E-02	0.630	pan	Severe COVID-19
14636_25	Ribonuclease UK114	Ribonuclease UK114	P52758	RIDA	NA	Inverse variance weighted	4	-0.137	0.055	1.202E-02	0.630	pan	Severe COVID-19
5128_53	SLAF6	SLAM family member 6	Q96DU3	SLAMF6	NA	Wald ratio	1	-0.213	0.085	1.202E-02	0.630	pan	Severe COVID-19
6462_12	TIMP-4	Metalloproteinase inhibitor 4	Q99727	TIMP4	NA	Inverse variance weighted	8	-0.108	0.043	1.269E-02	0.630	pan	Severe COVID-19
11557_3	SMUF1	E3 ubiquitin-protein ligase SMURF1	Q9HCE7	SMURF1	NA	Wald ratio	1	0.311	0.125	1.270E-02	0.630	pan	Severe COVID-19
4157_2	Thrombin	Thrombin	P00734	F2	NA	Inverse variance weighted	6	0.233	0.094	1.279E-02	0.630	pan	Severe COVID-19
4188_1	Aflatoxin B1 aldehyde reductase	Aflatoxin B1 aldehyde reductase member 2	O43488	AKR7A2	NA	Wald ratio	1	0.246	0.099	1.316E-02	0.630	pan	Severe COVID-19
13393_46	DERL1	Derlin-1	Q9BUN8	DERL1	NA	Wald ratio	1	0.773	0.314	1.381E-02	0.630	pan	Severe COVID-19
19365_11	BCAT2	Branched-chain-amino-acid aminotransferase, mitochondrial	O15382	BCAT2	NA	Inverse variance weighted	2	0.272	0.111	1.423E-02	0.630	pan	Severe COVID-19
16753_46	CO6A2	Collagen alpha-2(VI) chain	P12110	COL6A2	NA	Inverse variance weighted	5	-0.208	0.085	1.436E-02	0.630	pan	Severe COVID-19
8269_327	ARSK	Arylsulfatase K	Q6UWY0	ARSK	NA	Inverse variance weighted	2	0.507	0.208	1.469E-02	0.630	pan	Severe COVID-19
5900_11	HINT1	Histidine triad nucleotide-binding protein 1	P49773	HINT1	NA	Wald ratio	1	0.515	0.212	1.500E-02	0.630	pan	Severe COVID-19
18435_40	UBX2B	UBX domain-containing protein 2B	Q14CS0	UBXN2B	NA	Wald ratio	1	-0.610	0.251	1.501E-02	0.630	pan	Severe COVID-19
6414_8	OAF	Out at first protein homolog	Q86UD1	OAF	NA	Inverse variance weighted	6	0.119	0.049	1.511E-02	0.630	pan	Severe COVID-19
8353_15	SCN2B	Sodium channel subunit beta-2	O60939	SCN2B	NA	Inverse variance weighted	6	-0.331	0.137	1.547E-02	0.630	pan	Severe COVID-19
18909_11	EXOS8	Exosome complex component RRP43	Q96B26	EXOSC8	NA	Wald ratio	1	-0.544	0.225	1.563E-02	0.630	pan	Severe COVID-19
12656_1	KLC1	Kinesin light chain 1	Q07866	KLC1	NA	Wald ratio	1	0.897	0.372	1.584E-02	0.630	pan	Severe COVID-19
19279_42	CRBP	Retinol-binding protein 1	P09455	RBP1	NA	Wald ratio	1	0.554	0.232	1.682E-02	0.632	pan	Severe COVID-19
19383_131	CINP	Cyclin-dependent kinase 2-interacting protein	Q9BW66	CINP	NA	Wald ratio	1	0.666	0.279	1.707E-02	0.632	pan	Severe COVID-19
4249_64	NDP kinase B	Nucleoside diphosphate kinase B	P22392	NME2	NA	Wald ratio	1	0.315	0.133	1.783E-02	0.632	pan	Severe COVID-19
8786_6	F171B:ECD	Protein FAM171B:Extracellular domain	Q6P995	FAM171B	NA	Wald ratio	1	0.231	0.097	1.786E-02	0.632	pan	Severe COVID-19
19617_5	LTB4DH	Prostaglandin reductase 1	Q14914	PTGR1	NA	Inverse variance weighted	12	-0.075	0.032	1.804E-02	0.632	pan	Severe COVID-19
16620_26	LY75	Lymphocyte antigen 75	O60449	LY75	NA	Inverse variance weighted	19	-0.078	0.033	1.807E-02	0.632	pan	Severe COVID-19
8340_9	DB110	Beta-defensin 110	Q30KQ9	DEFB110	NA	Wald ratio	1	-0.711	0.301	1.812E-02	0.632	pan	Severe COVID-19
9931_20	Keratin-1	Keratin, type II cytoskeletal 1	P04264	KRT1	NA	Inverse variance weighted	2	-0.636	0.269	1.823E-02	0.632	pan	Severe COVID-19
11094_104	LPPL	Galectin-10	Q05315	CLC	NA	Wald ratio	1	0.544	0.233	1.935E-02	0.638	pan	Severe COVID-19
16919_1	ACBP	Acyl-CoA-binding protein	P07108	DBI	NA	Inverse variance weighted	4	0.306	0.131	1.946E-02	0.638	pan	Severe COVID-19
8795_48	TR:ECD	Transferrin receptor protein 1:Extracellular domain	P02786	TFRC	NA	Inverse variance weighted	5	0.221	0.095	1.962E-02	0.638	pan	Severe COVID-19
13669_6	FGFR-3:ECD	Fibroblast growth factor receptor 3:Extracellular domain	P22607	FGFR3	NA	Inverse variance weighted	3	-0.210	0.090	1.988E-02	0.638	pan	Severe COVID-19
15653_9	COAA1	Collagen alpha-1(X) chain	Q03692	COL10A1	NA	Inverse variance weighted	4	-0.148	0.063	1.989E-02	0.638	pan	Severe COVID-19
11825_27	PRGC1	Peroxisome proliferator-activated receptor gamma coactivator 1-alpha	Q9UBK2	PPARGC1A	NA	Wald ratio	1	0.755	0.326	2.059E-02	0.650	pan	Severe COVID-19
16918_198	TLR3	Toll-like receptor 3	O15455	TLR3	NA	Inverse variance weighted	9	0.097	0.042	2.163E-02	0.673	pan	Severe COVID-19
9580_5	Laminin gamma-2	Laminin subunit gamma-2	Q13753	LAMC2	NA	Inverse variance weighted	8	-0.122	0.054	2.321E-02	0.712	pan	Severe COVID-19
19127_1	HSPB6	Heat shock protein beta-6	O14558	HSPB6	NA	Wald ratio	1	0.825	0.365	2.394E-02	0.724	pan	Severe COVID-19
3342_76	ABL2	Abelson tyrosine-protein kinase 2	P42684	ABL2	NA	Wald ratio	1	-0.731	0.326	2.468E-02	0.730	pan	Severe COVID-19
4924_32	MMP-1	Interstitial collagenase	P03956	MMP1	NA	Inverse variance weighted	12	0.096	0.043	2.482E-02	0.730	pan	Severe COVID-19
5078_82	EphB6	Ephrin type-B receptor 6	O15197	EPHB6	NA	Inverse variance weighted	4	0.152	0.068	2.516E-02	0.730	pan	Severe COVID-19
4430_44	Collectin Kidney 1	Collectin-11	Q9BWP8	COLEC11	NA	Inverse variance weighted	11	0.151	0.068	2.559E-02	0.733	pan	Severe COVID-19
3181_50	Cathepsin S	Cathepsin S	P25774	CTSS	NA	Inverse variance weighted	12	-0.129	0.058	2.603E-02	0.733	pan	Severe COVID-19
14123_34	GI24:ECD	V-type immunoglobulin domain-containing suppressor of T-cell activation:Extracellular domain	Q9H7M9	VSIR	NA	Inverse variance weighted	6	-0.295	0.133	2.632E-02	0.733	pan	Severe COVID-19
12428_2	LYPL1	Lysophospholipase-like protein 1	Q5VWZ2	LYPLAL1	NA	Wald ratio	1	0.325	0.147	2.676E-02	0.733	pan	Severe COVID-19
12583_77	ARAF	Serine/threonine-protein kinase A-Raf	P10398	ARAF	NA	Wald ratio	1	-0.632	0.286	2.695E-02	0.733	pan	Severe COVID-19
19207_119	ADK	Adenosine kinase	P55263	ADK	NA	Wald ratio	1	0.627	0.286	2.842E-02	0.760	pan	Severe COVID-19
8885_6	CA2D3	Voltage-dependent calcium channel subunit alpha-2/delta-3	Q8IZS8	CACNA2D3	NA	Inverse variance weighted	9	-0.112	0.051	2.947E-02	0.760	pan	Severe COVID-19

9962_1	MUCDL	Cadherin-related family member 5	Q9HBB8	CDHR5	NA	Wald ratio	1	-0.579	0.266	2.949E-02	0.760	pan	Severe COVID-19
5440_26	Troponin I, skeletal, fast twitch	Troponin I, fast skeletal muscle	P48788	TNNI2	NA	Wald ratio	1	-0.676	0.311	2.950E-02	0.760	pan	Severe COVID-19
6416_8	GKN2	Gastrokine-2	Q86XP6	GKN2	NA	Inverse variance weighted	10	-0.153	0.071	2.996E-02	0.760	pan	Severe COVID-19
2864_2	MEK1	Dual specificity mitogen-activated protein kinase kinase 1	Q02750	MAP2K1	NA	Wald ratio	1	0.672	0.310	3.007E-02	0.760	pan	Severe COVID-19
10511_10	Collagen alpha-3(VI):isoform 3	Collagen alpha-3(VI) chain:isoform 3	P12111	COL6A3	NA	Inverse variance weighted	2	0.397	0.184	3.125E-02	0.770	pan	Severe COVID-19
17806_6	Syntenin 1	Syntenin-1	O00560	SDCBP	NA	Wald ratio	1	0.701	0.327	3.172E-02	0.770	pan	Severe COVID-19
13107_9	LYPD3	Ly6/PLAUR domain-containing protein 3	O95274	LYPD3	NA	Inverse variance weighted	3	-0.367	0.171	3.177E-02	0.770	pan	Severe COVID-19
3378_49	Kallikrein 7	Kallikrein-7	P49862	KLK7	NA	Inverse variance weighted	2	-0.224	0.105	3.197E-02	0.770	pan	Severe COVID-19
6388_21	CC126	Coiled-coil domain-containing protein 126	Q96EE4	CCDC126	NA	Inverse variance weighted	6	0.153	0.072	3.225E-02	0.770	pan	Severe COVID-19
17460_51	Mx1	Interferon-induced GTP-binding protein Mx1	P20591	MX1	NA	Inverse variance weighted	9	-0.132	0.062	3.431E-02	0.790	pan	Severe COVID-19
10608_9	HIS1	Histatin-1	P15515	HTN1	NA	Inverse variance weighted	2	0.221	0.104	3.471E-02	0.790	pan	Severe COVID-19
16596_25	GLRX3	Glutaredoxin-3	O76003	GLRX3	NA	Wald ratio	1	-0.388	0.184	3.516E-02	0.790	pan	Severe COVID-19
15513_108	Prostasin	Prostasin	Q16651	PRSS8	NA	Wald ratio	1	0.663	0.316	3.556E-02	0.790	pan	Severe COVID-19
16605_2	C1T9A	Complement C1q and tumor necrosis factor-related protein 9A	P0C862	C1QTNF9	NA	Wald ratio	1	0.209	0.099	3.560E-02	0.790	pan	Severe COVID-19
13936_24	PGK2	Phosphoglycerate kinase 2	P07205	PGK2	NA	Wald ratio	1	0.408	0.194	3.561E-02	0.790	pan	Severe COVID-19
5229_90	IMDH1	Inosine-5'-monophosphate dehydrogenase 1	P20839	IMPDH1	NA	Inverse variance weighted	9	-0.220	0.105	3.653E-02	0.790	pan	Severe COVID-19
3518_54	TAFI	Carboxypeptidase B2	Q96IY4	CPB2	NA	Inverse variance weighted	8	0.189	0.090	3.686E-02	0.790	pan	Severe COVID-19
14204_55	FOXJ2	Forkhead box protein J2	Q9P0K8	FOXJ2	NA	Inverse variance weighted	3	-0.353	0.169	3.689E-02	0.790	pan	Severe COVID-19
13438_115	CHRD	Chordin	Q9H2X0	CHRD	NA	Wald ratio	1	-0.429	0.206	3.746E-02	0.790	pan	Severe COVID-19
15375_49	Carboxypeptidase B1	Carboxypeptidase B	P15086	CPB1	NA	Inverse variance weighted	9	0.131	0.063	3.774E-02	0.790	pan	Severe COVID-19
13973_62	TTL	Tubulin--tyrosine ligase	Q8NG68	TTL	NA	Wald ratio	1	0.554	0.267	3.782E-02	0.790	pan	Severe COVID-19
6064_4	TXNDC4	Endoplasmic reticulum resident protein 44	Q9BS26	ERP44	NA	Inverse variance weighted	3	0.419	0.202	3.788E-02	0.790	pan	Severe COVID-19
5095_21	KI2L4	Killer cell immunoglobulin-like receptor 2DL4	Q99706	KIR2DL4	NA	Wald ratio	1	-0.486	0.235	3.893E-02	0.803	pan	Severe COVID-19
5867_60	ARGI1	Arginase-1	P05089	ARG1	NA	Wald ratio	1	0.398	0.193	3.926E-02	0.803	pan	Severe COVID-19
13435_31	IL-20 Rb	Interleukin-20 receptor subunit beta	Q6UXL0	IL20RB	NA	Wald ratio	1	0.610	0.298	4.080E-02	0.813	pan	Severe COVID-19
16049_43	OLR1	Oxidized low-density lipoprotein receptor 1	P78380	OLR1	NA	Wald ratio	1	0.524	0.256	4.091E-02	0.813	pan	Severe COVID-19
2631_50	IL-10 Rb	Interleukin-10 receptor subunit beta	Q08334	IL10RB	NA	Wald ratio	1	0.602	0.295	4.108E-02	0.813	pan	Severe COVID-19
6925_26	SNX8	Sorting nexin-8	Q9Y5X2	SNX8	NA	Inverse variance weighted	2	0.182	0.089	4.147E-02	0.813	pan	Severe COVID-19
11568_2	FKB1B	Peptidyl-prolyl cis-trans isomerase FKBP1B	P68106	FKBP1B	NA	Wald ratio	1	0.273	0.134	4.163E-02	0.813	pan	Severe COVID-19
12558_3	UBS3B	Ubiquitin-associated and SH3 domain-containing protein B	Q8TF42	UBASH3B	NA	Inverse variance weighted	2	-0.224	0.111	4.305E-02	0.829	pan	Severe COVID-19
9754_33	Quinone reductase 2	Ribosyldihydronicotinamide dehydrogenase [quinone]	P16083	NQO2	NA	Inverse variance weighted	9	0.054	0.027	4.358E-02	0.829	pan	Severe COVID-19
3316_58	Heparin cofactor II	Heparin cofactor 2	P05546	SERPIND1	NA	Wald ratio	1	0.598	0.296	4.360E-02	0.829	pan	Severe COVID-19
6597_24	TM157	Membrane protein FAM174A	Q8TBP5	FAM174A	NA	Inverse variance weighted	8	-0.086	0.043	4.430E-02	0.830	pan	Severe COVID-19
6022_57	NELL2	Protein kinase C-binding protein NELL2	Q99435	NELL2	NA	Wald ratio	1	-0.396	0.197	4.488E-02	0.830	pan	Severe COVID-19
17739_1	HCDH	Hydroxyacyl-coenzyme A dehydrogenase, mitochondrial	Q16836	HADH	NA	Wald ratio	1	-0.432	0.216	4.525E-02	0.830	pan	Severe COVID-19
5735_54	C1GLC	C1GALT1-specific chaperone 1	Q96EU7	C1GALT1C1	NA	Inverse variance weighted	3	0.161	0.081	4.550E-02	0.830	pan	Severe COVID-19
9950_229	LAG-3	Lymphocyte activation gene 3 protein	P18627	LAG3	NA	Inverse variance weighted	4	-0.244	0.122	4.587E-02	0.830	pan	Severe COVID-19
3030_3	DC-SIGNR	C-type lectin domain family 4 member M	Q9H2X3	CLEC4M	NA	Wald ratio	1	0.869	0.435	4.598E-02	0.830	pan	Severe COVID-19
12707_26	DPYL3	Dihydropyrimidinase-related protein 3	Q14195	DPYSL3	NA	Inverse variance weighted	2	0.353	0.178	4.714E-02	0.837	pan	Severe COVID-19
2741_22	Siglec-6	Sialic acid-binding Ig-like lectin 6	O43699	SIGLEC6	NA	Inverse variance weighted	7	-0.104	0.053	4.740E-02	0.837	pan	Severe COVID-19
13940_19	IP16:HIN 1	Gamma-interferon-inducible protein 16:Isoform 2, Hematopoietic expression, interferon-inducible nature, and nuclear localization 1	Q16666	IFI16	NA	Wald ratio	1	0.454	0.229	4.754E-02	0.837	pan	Severe COVID-19
19233_75	ATOX1	Copper transport protein ATOX1	O00244	ATOX1	NA	Wald ratio	1	0.558	0.284	4.942E-02	0.843	pan	Severe COVID-19
18931_40	SLIT3	Slit homolog 3 protein	O75094	SLIT3	NA	Inverse variance weighted	3	-0.184	0.094	4.947E-02	0.843	pan	Severe COVID-19
8053_16	DJB14:CD	DnaJ homolog subfamily B member 14:Cytoplasmic domain	Q8TBM8	DNAJB14	NA	Wald ratio	1	0.491	0.250	4.958E-02	0.843	pan	Severe COVID-19
3894_15	NAGK	N-acetyl-D-glucosamine kinase	Q9UJ70	NAGK	NA	Inverse variance weighted	5	-0.165	0.085	5.168E-02	0.843	pan	Severe COVID-19
9171_11	CSRP3	Cysteine and glycine-rich protein 3	P50461	CSRP3	NA	Inverse variance weighted	2	0.447	0.230	5.171E-02	0.843	pan	Severe COVID-19
7776_20	UNC5B	Netrin receptor UNC5B	Q8IZJ1	UNC5B	NA	Inverse variance weighted	3	-0.320	0.165	5.196E-02	0.843	pan	Severe COVID-19
2731_29	NADPH-P450 Oxidoreductase	NADPH--cytochrome P450 reductase	P16435	POR	NA	Inverse variance weighted	4	-0.225	0.116	5.220E-02	0.843	pan	Severe COVID-19
12395_86	SYDM	Aspartate--tRNA ligase, mitochondrial	Q6PI48	DARS2	NA	Wald ratio	1	0.612	0.315	5.236E-02	0.843	pan	Severe COVID-19
18884_22	DNJB4	DnaJ homolog subfamily B member 4	Q9UDY4	DNAJB4	NA	Inverse variance weighted	3	0.173	0.089	5.269E-02	0.843	pan	Severe COVID-19
9829_91	SULT 2A1	Bile salt sulfotransferase	Q06520	SULT2A1	NA	Wald ratio	1	-0.321	0.166	5.313E-02	0.843	pan	Severe COVID-19
7178_59	DEPP	Protein DEPP	Q9NTK1	DEPP1	NA	Inverse variance weighted	6	-0.163	0.085	5.473E-02	0.843	pan	Severe COVID-19
5006_71	MK13	Mitogen-activated protein kinase 13	O15264	MAPK13	NA	Wald ratio	1	-0.389	0.203	5.529E-02	0.843	pan	Severe COVID-19
15596_7	HEXI1	Protein HEXIM1	O94992	HEXIM1	NA	Wald ratio	1	0.552	0.288	5.539E-02	0.843	pan	Severe COVID-19
17455_42	FOLR1	Folate receptor alpha	P15328	FOLR1	NA	Wald ratio	1	-0.255	0.133	5.567E-02	0.843	pan	Severe COVID-19
7932_23	SYT8	Synaptotagmin-8	Q8NBV8	SYT8	NA	Wald ratio	1	0.640	0.335	5.612E-02	0.843	pan	Severe COVID-19
8235_48	SCG1	Secretogranin-1	P05060	CHGB	NA	Inverse variance weighted	7	0.232	0.122	5.618E-02	0.843	pan	Severe COVID-19
14094_29	HB-EGF	Heparin-binding EGF-like growth factor	Q99075	HBEGF	NA	Inverse variance weighted	8	-0.228	0.120	5.696E-02	0.843	pan	Severe COVID-19
18307_71	PPase 2	Inorganic pyrophosphatase 2, mitochondrial	Q9H2U2	PPA2	NA	Wald ratio	1	0.442	0.233	5.716E-02	0.843	pan	Severe COVID-19
6366_38	TXD15	Thioredoxin domain-containing protein 15	Q96J42	TXNDC15	NA	Inverse variance weighted	12	0.080	0.042	5.737E-02	0.843	pan	Severe COVID-19
6359_50	AGO61	Protein O-linked-mannose beta-1,4-N-acetylglucosaminyltransferase 2	Q8NAT1	POMGNT2	NA	Inverse variance weighted	12	-0.110	0.058	5.913E-02	0.843	pan	Severe COVID-19
5744_12	CA056	Protein MENT	Q9BUN1	MENT	NA	Inverse variance weighted	4	-0.347	0.184	5.919E-02	0.843	pan	Severe COVID-19
10606_34	TOIP1:Perinuclear	Torsin-1A-interacting protein 1:Perinuclear domain	Q5JTV8	TOR1AIP1	NA	Wald ratio	1	0.656	0.348	5.925E-02	0.843	pan	Severe COVID-19
5124_69	sICAM-5	Intercellular adhesion molecule 5	Q9UMF0	ICAM5	NA	Inverse variance weighted	7	-0.225	0.119	5.957E-02	0.843	pan	Severe COVID-19
17832_12	IDI2	Isopentenyl-diphosphate delta-isomerase 2	Q9BXS1	IDI2	NA	Inverse variance weighted	2	0.179	0.096	6.175E-02	0.843	pan	Severe COVID-19
7257_18	TIP39	Tuberoinfundibular peptide of 39 residues	Q96A98	PTH2	NA	Wald ratio	1	0.728	0.390	6.212E-02	0.843	pan	Severe COVID-19
4500_50	SCGF-alpha	Stem cell growth factor-alpha	Q9Y240	CLEC11A	NA	Inverse variance weighted	8	-0.071	0.038	6.216E-02	0.843	pan	Severe COVID-19
5621_64	THSD1	Thrombospondin type-1 domain-containing protein 1	Q9NS62	THSD1	NA	Inverse variance weighted	13	0.072	0.038	6.280E-02	0.843	pan	Severe COVID-19
17680_12	EPHB1	Ephrin type-B receptor 1	P54762	EPHB1	NA	Inverse variance weighted	3	0.080	0.043	6.295E-02	0.843	pan	Severe COVID-19
9173_21	PGM1	Phosphoglucomutase-1	P36871	PGM1	NA	Wald ratio	1	0.341	0.185	6.480E-02	0.843	pan	Severe COVID-19
3044_3	PARC	C-C motif chemokine 18	P55774	CCL18	NA	Inverse variance weighted	9	-0.157	0.085	6.481E-02	0.843	pan	Severe COVID-19
10435_2	ASPG	N(4)-(beta-N-acetylglucosaminyl)-L-asparaginase	P20933	AGA	NA	Inverse variance weighted	2	0.437	0.237	6.493E-02	0.843	pan	Severe COVID-19
16599_38	GPN1	GPN-loop GTPase 1	Q9HCN4	GPN1	NA	Inverse variance weighted	6	-0.147	0.080	6.493E-02	0.843	pan	Severe COVID-19
15641_20	TEFF1	Tomoregulin-1	Q8IYR6	TMEFF1	NA	Wald ratio	1	-0.482	0.261	6.507E-02	0.843	pan	Severe COVID-19
7891_45	UGT 1A6	UDP-glucuronosyltransferase 1-6	P19224	UGT1A6	NA	Inverse variance weighted	4	-0.152	0.083	6.559E-02	0.843	pan	Severe COVID-19
3535_84	DKK1	Dickkopf-related protein 1	O94907	DKK1	NA	Wald ratio	1	0.587	0.320	6.655E-02	0.843	pan	Severe COVID-19
7161_25	G6PE	GDH/6PGL endoplasmic bifunctional protein	O95479	H6PD	NA	Inverse variance weighted	19	-0.065	0.036	6.686E-02	0.843	pan	Severe COVID-19
12713_365	RGS19	Regulator of G-protein signaling 19	P49795	RGS19	NA	Wald ratio	1	0.555	0.304	6.771E-02	0.843	pan	Severe COVID-19

8925_25	RIR2B	Ribonucleoside-diphosphate reductase subunit M2 B	Q7LG56	RRM2B	NA	Inverse variance weighted	2	0.172	0.094	6.821E-02	0.843	pan	Severe COVID-19
16558_2	MYOC	Myocilin	Q99972	MYOC	NA	Inverse variance weighted	9	0.076	0.042	6.838E-02	0.843	pan	Severe COVID-19
8297_8	DJC10	DnaJ homolog subfamily C member 10	Q8IXB1	DNAJC10	NA	Inverse variance weighted	6	-0.178	0.098	6.856E-02	0.843	pan	Severe COVID-19
9981_18	K1467:C-term	Protein FAM234B:C-term	A2RU67	FAM234B	NA	Inverse variance weighted	4	-0.175	0.096	6.862E-02	0.843	pan	Severe COVID-19
17821_20	NMT2	Glycylpeptide N-tetradecanoyltransferase 2	O60551	NMT2	NA	Wald ratio	1	0.435	0.239	6.873E-02	0.843	pan	Severe COVID-19
2992_59	IL-17 sR	Interleukin-17 receptor A	Q96F46	IL17RA	NA	Inverse variance weighted	7	-0.033	0.018	6.923E-02	0.843	pan	Severe COVID-19
7808_5	GLCE	D-glucuronyl C5-epimerase	O94923	GLCE	NA	Inverse variance weighted	9	-0.066	0.036	6.982E-02	0.843	pan	Severe COVID-19
17447_52	SFRP4	Secreted frizzled-related protein 4	Q6FHJ7	SFRP4	NA	Inverse variance weighted	4	-0.153	0.085	7.010E-02	0.843	pan	Severe COVID-19
6923_1	PLOD2	Procollagen-lysine,2-oxoglutarate 5-dioxygenase 2	O00469	PLOD2	NA	Inverse variance weighted	3	-0.206	0.114	7.020E-02	0.843	pan	Severe COVID-19
5825_49	IFN-g R1	Interferon gamma receptor 1	P15260	IFNGR1	NA	Inverse variance weighted	3	-0.408	0.226	7.021E-02	0.843	pan	Severe COVID-19
9321_400	NMB	Neuromedin-B	P08949	NMB	NA	Inverse variance weighted	2	-0.171	0.095	7.044E-02	0.843	pan	Severe COVID-19
8606_39	GPNMB:CD	Transmembrane glycoprotein NMB:Cytoplasmic domain	Q14956	GPNMB	NA	Inverse variance weighted	9	-0.104	0.058	7.077E-02	0.843	pan	Severe COVID-19
11126_102	TRIO	Triple functional domain protein	O75962	TRIO	NA	Wald ratio	1	-0.601	0.333	7.121E-02	0.843	pan	Severe COVID-19
18840_205	SH21B	SH2 domain-containing protein 1B	O14796	SH2D1B	NA	Wald ratio	1	0.651	0.361	7.124E-02	0.843	pan	Severe COVID-19
18917_53	Pancreatic alpha-amylase	Pancreatic alpha-amylase	P04746	AMY2A	NA	Inverse variance weighted	7	-0.302	0.168	7.137E-02	0.843	pan	Severe COVID-19
15583_18	FCRLB	Fc receptor-like B	Q6BAA4	FCRLB	NA	Inverse variance weighted	4	0.212	0.118	7.225E-02	0.843	pan	Severe COVID-19
2635_61	Layilin	Layilin	Q6UX15	LAYN	NA	Wald ratio	1	-0.485	0.270	7.240E-02	0.843	pan	Severe COVID-19
5457_5	COLEC12	Collectin-12	Q5KU26	COLEC12	NA	Inverse variance weighted	12	0.179	0.100	7.246E-02	0.843	pan	Severe COVID-19
10924_258	NEUFC	Neuferricin	Q8WUJ1	CYB5D2	NA	Inverse variance weighted	3	0.186	0.103	7.253E-02	0.843	pan	Severe COVID-19
5713_9	IFN-lambda 3	Interferon lambda-3	Q8IZI9	IFNL3	NA	Inverse variance weighted	2	0.444	0.247	7.270E-02	0.843	pan	Severe COVID-19
16856_79	MARE2	Microtubule-associated protein RP/EB family member 2	Q15555	MAPRE2	NA	Inverse variance weighted	2	0.386	0.215	7.287E-02	0.843	pan	Severe COVID-19
8383_20	CK094	Uncharacterized protein C11orf94	C9JXX5	C11orf94	NA	Wald ratio	1	0.249	0.139	7.302E-02	0.843	pan	Severe COVID-19
15385_116	FABP2	Fatty acid-binding protein, intestinal	P12104	FABP2	NA	Inverse variance weighted	10	0.082	0.046	7.420E-02	0.843	pan	Severe COVID-19
4407_10	MSP	Hepatocyte growth factor-like protein	P26927	MST1	NA	Inverse variance weighted	20	-0.067	0.037	7.478E-02	0.843	pan	Severe COVID-19
18830_1	Omentin	Intelectin-1	Q8WWA0	ITLN1	NA	Inverse variance weighted	4	0.250	0.141	7.492E-02	0.843	pan	Severe COVID-19
13666_222	Carbonic Anhydrase X	Carbonic anhydrase-related protein 10	Q9NS85	CA10	NA	Inverse variance weighted	8	-0.150	0.085	7.509E-02	0.843	pan	Severe COVID-19
16305_10	Cadherin-11:ECD	Cadherin-11:Extracellular domain	P55287	CDH11	NA	Inverse variance weighted	11	-0.113	0.063	7.532E-02	0.843	pan	Severe COVID-19
15398_2	HERV1	FAD-linked sulfhydryl oxidase ALR	P55789	GFER	NA	Wald ratio	1	-0.473	0.267	7.602E-02	0.843	pan	Severe COVID-19
11378_37	KSYK:SH2, 1 and 2	Tyrosine-protein kinase SYK:Src Homology domain	P43405	SYK	NA	Wald ratio	1	0.564	0.318	7.603E-02	0.843	pan	Severe COVID-19
15545_13	Calcineurin B a	Calcineurin subunit B type 1	P63098	PPP3R1	NA	Wald ratio	1	-0.441	0.249	7.676E-02	0.843	pan	Severe COVID-19
7059_14	LIRA6	Leukocyte immunoglobulin-like receptor subfamily A member 6	Q6PI73	LILRA6	NA	Inverse variance weighted	6	0.114	0.065	7.740E-02	0.843	pan	Severe COVID-19
9409_11	TRYB1	Tryptase beta-1	Q15661	TPSAB1	NA	Inverse variance weighted	18	0.055	0.031	7.759E-02	0.843	pan	Severe COVID-19
6372_7	YBOX2	Y-box-binding protein 2	Q9Y2T7	YBX2	NA	Inverse variance weighted	2	0.420	0.238	7.795E-02	0.843	pan	Severe COVID-19
17364_8	RU2B	U2 small nuclear ribonucleoprotein B''	P08579	SNRPB2	NA	Wald ratio	1	-0.870	0.495	7.914E-02	0.843	pan	Severe COVID-19
13575_40	DVL2	Segment polarity protein dishevelled homolog DVL-2	O14641	DVL2	NA	Wald ratio	1	0.833	0.475	7.926E-02	0.843	pan	Severe COVID-19
17512_2	NMI	N-myc-interactor	Q13287	NMI	NA	Wald ratio	1	0.690	0.393	7.933E-02	0.843	pan	Severe COVID-19
6986_17	HS3SB	Heparan sulfate glucosamine 3-O-sulfotransferase 3B1	Q9Y662	HS3ST3B1	NA	Wald ratio	1	-0.616	0.351	7.938E-02	0.843	pan	Severe COVID-19
6527_1	TRIL:ECD	TLR4 interactor with leucine rich repeats:Extracellular domain	Q7L0X0	TRIL	NA	Wald ratio	1	0.344	0.196	7.943E-02	0.843	pan	Severe COVID-19
3905_62	UBE2N	Ubiquitin-conjugating enzyme E2 N	P61088	UBE2N	NA	Wald ratio	1	-0.579	0.330	7.954E-02	0.843	pan	Severe COVID-19
18376_19	Myosin light chain 1	Myosin light chain 3	P08590	MYL3	NA	Inverse variance weighted	6	-0.263	0.150	7.993E-02	0.843	pan	Severe COVID-19
12431_13	PELO	Protein pelota homolog	Q9BRX2	PELO	NA	Wald ratio	1	-0.568	0.325	8.034E-02	0.843	pan	Severe COVID-19
6321_65	PCDGA	Protocadherin gamma-A10	Q9Y5H3	PCDHGA10	NA	Inverse variance weighted	3	0.310	0.178	8.095E-02	0.843	pan	Severe COVID-19
8035_6	CA198	Uncharacterized protein C1orf198	Q9H425	C1orf198	NA	Wald ratio	1	0.228	0.131	8.130E-02	0.843	pan	Severe COVID-19
13682_47	M-CSF R	Macrophage colony-stimulating factor 1 receptor	P07333	CSF1R	NA	Inverse variance weighted	5	-0.240	0.138	8.139E-02	0.843	pan	Severe COVID-19
13590_1	ORN	Oligoribonuclease, mitochondrial	Q9Y3B8	REXO2	NA	Inverse variance weighted	2	-0.284	0.163	8.148E-02	0.843	pan	Severe COVID-19
3448_13	IR	Insulin receptor	P06213	INSR	NA	Inverse variance weighted	13	-0.143	0.082	8.169E-02	0.843	pan	Severe COVID-19
16828_8	Collagen a1(VI)	Collagen alpha-1(VI) chain	P12109	COL6A1	NA	Inverse variance weighted	14	-0.043	0.025	8.175E-02	0.843	pan	Severe COVID-19
11926_23	P34	Alpha- and gamma-adaptin-binding protein p34	Q6PD74	AAGAB	NA	Inverse variance weighted	3	0.309	0.177	8.181E-02	0.843	pan	Severe COVID-19
14112_40	RELT	Tumor necrosis factor receptor superfamily member 19L	Q969Z4	RELT	NA	Wald ratio	1	0.524	0.301	8.198E-02	0.843	pan	Severe COVID-19
5008_51	Mn SOD	Superoxide dismutase [Mn], mitochondrial	P04179	SOD2	NA	Inverse variance weighted	4	0.325	0.187	8.223E-02	0.843	pan	Severe COVID-19
13268_45	WNT5A	Protein Wnt-5a	P41221	WNT5A	NA	Inverse variance weighted	5	0.236	0.136	8.251E-02	0.843	pan	Severe COVID-19
11387_3	ATF6B	Cyclic AMP-dependent transcription factor ATF-6 beta	Q99941	ATF6B	NA	Inverse variance weighted	4	-0.239	0.138	8.283E-02	0.843	pan	Severe COVID-19
6606_61	KISS1	Metastasis-suppressor KiSS-1	Q15726	KISS1	NA	Inverse variance weighted	14	0.072	0.041	8.330E-02	0.844	pan	Severe COVID-19
11293_14	LRRN1:CD	Leucine-rich repeat neuronal protein 1:Cytoplasmic domain	Q6UXK5	LRRN1	NA	Inverse variance weighted	7	-0.196	0.113	8.443E-02	0.849	pan	Severe COVID-19
19188_21	NAP2L	nucleosome assembly protein 1-like 4	Q99733	NAP1L4	NA	Inverse variance weighted	3	0.343	0.199	8.453E-02	0.849	pan	Severe COVID-19
8993_151	RIPK2	Receptor-interacting serine/threonine-protein kinase 2	O43353	RIPK2	NA	Wald ratio	1	-0.528	0.307	8.532E-02	0.853	pan	Severe COVID-19
15381_45	Discoidin domain receptor 2	Discoidin domain-containing receptor 2	Q16832	DDR2	NA	Wald ratio	1	0.513	0.299	8.598E-02	0.855	pan	Severe COVID-19
9765_4	NDE1	Nuclear distribution protein nudE homolog 1	Q9NXR1	NDE1	NA	Wald ratio	1	0.365	0.214	8.769E-02	0.856	pan	Severe COVID-19
10396_6	Mcl-1	Induced myeloid leukemia cell differentiation protein Mcl-1	Q07820	MCL1	NA	Inverse variance weighted	2	-0.369	0.216	8.791E-02	0.856	pan	Severe COVID-19
6342_10	Nephronectin	Nephronectin	Q6UXI9	NPNT	NA	Inverse variance weighted	4	0.348	0.205	8.903E-02	0.856	pan	Severe COVID-19
4920_10	Lysozyme	Lysozyme C	P61626	LYZ	NA	Inverse variance weighted	10	-0.061	0.036	8.933E-02	0.856	pan	Severe COVID-19
19173_5	ZFAN1	AN1-type zinc finger protein 1	Q8TCF1	ZFAND1	NA	Inverse variance weighted	4	0.145	0.086	8.939E-02	0.856	pan	Severe COVID-19
6207_10	prosaposin	Prosaposin	P07602	PSAP	NA	Inverse variance weighted	5	0.290	0.171	8.946E-02	0.856	pan	Severe COVID-19
10772_21	CGAT2	Chondroitin sulfate N-acetylgalactosaminyltransferase 2	Q8N6G5	CSGALNACT2	NA	Wald ratio	1	0.267	0.157	8.973E-02	0.856	pan	Severe COVID-19
9884_8	PPIL1	Peptidyl-prolyl cis-trans isomerase-like 1	Q9Y3C6	PPIL1	NA	Inverse variance weighted	9	0.068	0.040	9.006E-02	0.856	pan	Severe COVID-19
8252_2	NOTUM	Palmitoleoyl-protein carboxylesterase NOTUM	Q6P988	NOTUM	NA	Inverse variance weighted	4	0.231	0.136	9.026E-02	0.856	pan	Severe COVID-19
10085_25	STAR	Steroidogenic acute regulatory protein, mitochondrial	P49675	STAR	NA	Wald ratio	1	-0.574	0.339	9.099E-02	0.856	pan	Severe COVID-19
12546_1	SMTN	Smoothelin	P53814	SMTN	NA	Wald ratio	1	-0.646	0.382	9.120E-02	0.856	pan	Severe COVID-19
6467_65	KTEL1	Protein O-glucosyltransferase 1	Q8NBL1	POGLUT1	NA	Inverse variance weighted	2	-0.115	0.068	9.136E-02	0.856	pan	Severe COVID-19
5480_49	RANTES	C-C motif chemokine 5	P13501	CCL5	NA	Inverse variance weighted	2	0.367	0.217	9.155E-02	0.856	pan	Severe COVID-19
11531_24	GI24:CD	V-type immunoglobulin domain-containing suppressor of T-cell activation:Cytoplasmic domain	Q9H7M9	VSIR	NA	Wald ratio	1	0.411	0.244	9.169E-02	0.856	pan	Severe COVID-19
9886_28	XRCC4	DNA repair protein XRCC4	Q13426	XRCC4	NA	Inverse variance weighted	2	0.200	0.119	9.205E-02	0.856	pan	Severe COVID-19
15308_108	VWC2	Brorin	Q2TAL6	VWC2	NA	Inverse variance weighted	9	0.073	0.043	9.303E-02	0.861	pan	Severe COVID-19
7185_29	GPV	Platelet glycoprotein V	P40197	GP5	NA	Inverse variance weighted	2	-0.290	0.173	9.343E-02	0.861	pan	Severe COVID-19
12016_60	CBL	E3 ubiquitin-protein ligase CBL	P22681	CBL	NA	Wald ratio	1	0.175	0.105	9.564E-02	0.871	pan	Severe COVID-19
17692_2	BT3A3	Butyrophilin subfamily 3 member A3	O00478	BTN3A3	NA	Inverse variance weighted	10	-0.057	0.034	9.626E-02	0.871	pan	Severe COVID-19
13563_259	ACBD7	Acyl-CoA-binding domain-containing protein 7	Q8N6N7	ACBD7	NA	Wald ratio	1	0.648	0.392	9.772E-02	0.871	pan	Severe COVID-19
9594_30	ZNT5	Zinc transporter 5	Q8TAD4	SLC30A5	NA	Wald ratio	1	-0.341	0.206	9.777E-02	0.871	pan	Severe COVID-19

17735_130	GBRAP	Gamma-aminobutyric acid receptor-associated protein	O95166	GABARAP	NA	Wald ratio	1	0.503	0.304	9.799E-02	0.871	pan	Severe COVID-19
6629_3	HBD-1	Beta-defensin 1	P60022	DEFB1	NA	Inverse variance weighted	10	-0.075	0.045	9.820E-02	0.871	pan	Severe COVID-19
4254_6	NUDC3	NudC domain-containing protein 3	Q8IVD9	NUDCD3	NA	Wald ratio	1	0.372	0.225	9.833E-02	0.871	pan	Severe COVID-19
9901_28	EGLN1	Egl nine homolog 1	Q9GZT9	EGLN1	NA	Inverse variance weighted	7	-0.142	0.086	9.842E-02	0.871	pan	Severe COVID-19
16302_11	SPLC2	BPI fold-containing family A member 2	Q96DR5	BPIFA2	NA	Wald ratio	1	-0.378	0.229	9.868E-02	0.871	pan	Severe COVID-19
15462_28	CD8A	T-cell surface glycoprotein CD8 alpha chain	P01732	CD8A	NA	Inverse variance weighted	8	-0.086	0.052	9.888E-02	0.871	pan	Severe COVID-19
11248_43	HEM4	Uroporphyrinogen-III synthase	P10746	UROS	NA	Inverse variance weighted	4	0.118	0.072	9.908E-02	0.871	pan	Severe COVID-19
13954_9	GNA1	Glucosamine 6-phosphate N-acetyltransferase	Q96EK6	GNPNAT1	NA	Wald ratio	1	-0.428	0.260	9.973E-02	0.871	pan	Severe COVID-19
13988_67	NMRL1	NmrA-like family domain-containing protein 1	Q9HBL8	NMRAL1	NA	Inverse variance weighted	13	-0.068	0.042	1.000E-01	0.871	pan	Severe COVID-19
9751_72	NSEP1	Nuclease-sensitive element-binding protein 1	P67809	YBX1	NA	Inverse variance weighted	5	-0.229	0.140	1.008E-01	0.871	pan	Severe COVID-19
13955_33	DAPK1	Death-associated protein kinase 1	P53355	DAPK1	NA	Inverse variance weighted	3	0.327	0.200	1.008E-01	0.871	pan	Severe COVID-19
6551_94	Vaspin	Serpin A12	Q8IW75	SERPINA12	NA	Inverse variance weighted	6	-0.074	0.045	1.009E-01	0.871	pan	Severe COVID-19
9312_8	AZGP1	Zinc-alpha-2-glycoprotein	P25311	AZGP1	NA	Inverse variance weighted	4	-0.317	0.194	1.017E-01	0.874	pan	Severe COVID-19
5947_90	Tpo	Thrombopoietin	P40225	THPO	NA	Inverse variance weighted	2	0.472	0.289	1.025E-01	0.875	pan	Severe COVID-19
7139_14	SLIK4	SLIT and NTRK-like protein 4	Q8IW52	SLITRK4	NA	Wald ratio	1	0.446	0.273	1.026E-01	0.875	pan	Severe COVID-19
10557_6	TEX29	Testis-expressed sequence 29 protein	Q8N6K0	TEX29	NA	Inverse variance weighted	4	0.234	0.144	1.047E-01	0.879	pan	Severe COVID-19
9343_16	IL-2 sRb	Interleukin-2 receptor subunit beta	P14784	IL2RB	NA	Inverse variance weighted	5	-0.323	0.199	1.047E-01	0.879	pan	Severe COVID-19
12616_45	NRBP	Nuclear receptor-binding protein	Q9UHY1	NRBP1	NA	Wald ratio	1	0.195	0.121	1.048E-01	0.879	pan	Severe COVID-19
7193_98	PCDC1	Protocadherin alpha-C1	Q9H158	PCDHAC1	NA	Wald ratio	1	-0.356	0.221	1.076E-01	0.879	pan	Severe COVID-19
2900_53	HCC-1	C-C motif chemokine 14	Q16627	CCL14	NA	Inverse variance weighted	7	-0.114	0.071	1.077E-01	0.879	pan	Severe COVID-19
12940_35	AL3B1	Aldehyde dehydrogenase family 3 member B1	P43353	ALDH3B1	NA	Wald ratio	1	0.214	0.133	1.079E-01	0.879	pan	Severe COVID-19
13986_6	LANC1	LanC-like protein 1	O43813	LANCL1	NA	Inverse variance weighted	2	-0.300	0.187	1.085E-01	0.879	pan	Severe COVID-19
6919_3	HBAZ	Hemoglobin subunit zeta	P02008	HBZ	NA	Inverse variance weighted	8	0.064	0.040	1.088E-01	0.879	pan	Severe COVID-19
16892_23	ENPP2	Ectonucleotide pyrophosphatase/phosphodiesterase family member 2	Q13822	ENPP2	NA	Inverse variance weighted	6	0.204	0.127	1.093E-01	0.879	pan	Severe COVID-19
3000_66	MBL	Mannose-binding protein C	P11226	MBL2	NA	Inverse variance weighted	20	-0.044	0.028	1.094E-01	0.879	pan	Severe COVID-19
2706_69	Thyroxine-Binding Globulin	Thyroxine-binding globulin	P05543	SERPINA7	NA	Inverse variance weighted	2	0.350	0.219	1.097E-01	0.879	pan	Severe COVID-19
11542_11	TM230	Transmembrane protein 230	Q96A57	TMEM230	NA	Wald ratio	1	-0.358	0.224	1.098E-01	0.879	pan	Severe COVID-19
8476_11	CgA	Chromogranin-A	P10645	CHGA	NA	Inverse variance weighted	6	0.169	0.106	1.109E-01	0.879	pan	Severe COVID-19
9484_75	Desmoglein-2	Desmoglein-2	Q14126	DSG2	NA	Inverse variance weighted	10	0.125	0.078	1.109E-01	0.879	pan	Severe COVID-19
15304_1	PAP1	Regenerating islet-derived protein 3-alpha	Q06141	REG3A	NA	Inverse variance weighted	15	0.095	0.060	1.115E-01	0.879	pan	Severe COVID-19
19293_6	VP26A	Vacuolar protein sorting-associated protein 26A	O75436	VPS26A	NA	Inverse variance weighted	3	-0.132	0.083	1.126E-01	0.879	pan	Severe COVID-19
5132_71	TCCR	Interleukin-27 receptor subunit alpha	Q6UWB1	IL27RA	NA	Inverse variance weighted	7	-0.059	0.037	1.130E-01	0.879	pan	Severe COVID-19
2871_73	RAD51	DNA repair protein RAD51 homolog 1	Q06609	RAD51	NA	Wald ratio	1	-0.504	0.318	1.132E-01	0.879	pan	Severe COVID-19
7206_20	F16P1	Fructose-1,6-bisphosphatase 1	P09467	FBP1	NA	Inverse variance weighted	2	0.247	0.156	1.136E-01	0.879	pan	Severe COVID-19
15540_6	Vimentin	Vimentin	P08670	VIM	NA	Inverse variance weighted	2	0.288	0.183	1.145E-01	0.879	pan	Severe COVID-19
17697_2	OVCA2	Esterase OVCA2	Q8WZ82	OVCA2	NA	Wald ratio	1	-0.334	0.212	1.149E-01	0.879	pan	Severe COVID-19
6555_58	Stomatin-like protein 2	Stomatin-like protein 2, mitochondrial	Q9UJZ1	STOML2	NA	Wald ratio	1	0.463	0.294	1.151E-01	0.879	pan	Severe COVID-19
3413_50	BFL1	Bcl-2-related protein A1	Q16548	BCL2A1	NA	Wald ratio	1	-0.531	0.337	1.153E-01	0.879	pan	Severe COVID-19
17377_1	Aldose reductase-like C3	Aldo-keto reductase family 1 member C3	P42330	AKR1C3	NA	Inverse variance weighted	9	-0.101	0.064	1.157E-01	0.879	pan	Severe COVID-19
3607_71	DKK3	Dickkopf-related protein 3	Q9UBP4	DKK3	NA	Inverse variance weighted	10	-0.065	0.041	1.159E-01	0.879	pan	Severe COVID-19
4911_49	Glutathione S-transferase Pi	Glutathione S-transferase P	P09211	GSTP1	NA	Wald ratio	1	-0.172	0.109	1.161E-01	0.879	pan	Severe COVID-19
2836_68	Lipocalin 2	Neutrophil gelatinase-associated lipocalin	P80188	LCN2	NA	Inverse variance weighted	7	-0.166	0.106	1.165E-01	0.879	pan	Severe COVID-19
15347_12	Hemopexin	Hemopexin	P02790	HPX	NA	Wald ratio	1	-0.532	0.339	1.169E-01	0.879	pan	Severe COVID-19
18337_4	GMDS	GDP-mannose 4,6 dehydratase	O60547	GMDS	NA	Wald ratio	1	-0.454	0.290	1.170E-01	0.879	pan	Severe COVID-19
3077_66	Coagulation Factor Xa	Coagulation factor Xa	P00742	F10	NA	Inverse variance weighted	4	-0.242	0.155	1.171E-01	0.879	pan	Severe COVID-19
16616_137	ENOB	Beta-enolase	P13929	ENO3	NA	Inverse variance weighted	5	0.189	0.121	1.177E-01	0.879	pan	Severe COVID-19
6617_12	FCRL6	Fc receptor-like protein 6	Q6DN72	FCRL6	NA	Wald ratio	1	-0.193	0.124	1.194E-01	0.879	pan	Severe COVID-19
14101_2	CNTFR alpha	Ciliary neurotrophic factor receptor subunit alpha	P26992	CNTFR	NA	Inverse variance weighted	5	-0.155	0.100	1.201E-01	0.879	pan	Severe COVID-19
11205_10	Integrin beta-7	Integrin beta-7	P26010	ITGB7	NA	Wald ratio	1	0.503	0.324	1.201E-01	0.879	pan	Severe COVID-19
9557_5	MANS1	MANSC domain-containing protein 1	Q9H8J5	MANSC1	NA	Inverse variance weighted	5	0.088	0.057	1.203E-01	0.879	pan	Severe COVID-19
9747_48	MARK3	MAP/microtubule affinity-regulating kinase 3	P27448	MARK3	NA	Wald ratio	1	0.741	0.477	1.203E-01	0.879	pan	Severe COVID-19
4831_4	sL-Selectin	L-Selectin	P14151	SELL	NA	Inverse variance weighted	8	-0.090	0.058	1.211E-01	0.879	pan	Severe COVID-19
17384_110	K6PF	ATP-dependent 6-phosphofructokinase, muscle type	P08237	PFKM	NA	Inverse variance weighted	3	-0.197	0.127	1.214E-01	0.879	pan	Severe COVID-19
8766_29	LIRA5	Leukocyte immunoglobulin-like receptor subfamily A member 5	A6NI73	LILRA5	NA	Inverse variance weighted	3	-0.385	0.249	1.217E-01	0.879	pan	Severe COVID-19
18401_18	ALKB3	Alpha-ketoglutarate-dependent dioxygenase alkB homolog 3	Q96Q83	ALKBH3	NA	Inverse variance weighted	6	-0.094	0.061	1.229E-01	0.879	pan	Severe COVID-19
8357_43	ST4A1	Sulfotransferase 4A1	Q9BR01	SULT4A1	NA	Wald ratio	1	0.425	0.276	1.231E-01	0.879	pan	Severe COVID-19
7128_9	VWA2	von Willebrand factor A domain-containing protein 2	Q5GFL6	VWA2	NA	Inverse variance weighted	7	0.133	0.086	1.235E-01	0.879	pan	Severe COVID-19
15435_4	PNP	Purine nucleoside phosphorylase	P00491	PNP	NA	Inverse variance weighted	2	-0.149	0.097	1.238E-01	0.879	pan	Severe COVID-19
16035_8	VEGF sR3	Vascular endothelial growth factor receptor 3	P35916	FLT4	NA	Inverse variance weighted	11	-0.118	0.077	1.240E-01	0.879	pan	Severe COVID-19
2585_2	PRL	Prolactin	P01236	PRL	NA	Wald ratio	1	0.341	0.222	1.242E-01	0.879	pan	Severe COVID-19
8759_29	a1,4-Galactosyltransferase	Lactosylceramide 4-alpha-galactosyltransferase	Q9NPC4	A4GALT	NA	Inverse variance weighted	16	0.054	0.035	1.243E-01	0.879	pan	Severe COVID-19
15607_56	KPYR	Pyruvate kinase PKLR	P30613	PKLR	NA	Inverse variance weighted	3	-0.282	0.184	1.243E-01	0.879	pan	Severe COVID-19
9823_2	DYR	Dihydrofolate reductase	P00374	DHFR	NA	Inverse variance weighted	5	-0.091	0.059	1.244E-01	0.879	pan	Severe COVID-19
5337_64	B7-2	T-lymphocyte activation antigen CD86	P42081	CD86	NA	Wald ratio	1	-0.397	0.258	1.245E-01	0.879	pan	Severe COVID-19
6713_4	LRP11	Low-density lipoprotein receptor-related protein 11	Q86VZ4	LRP11	NA	Inverse variance weighted	12	0.088	0.057	1.256E-01	0.879	pan	Severe COVID-19
11360_39	RRM1	Ribonucleoside-diphosphate reductase large subunit	P23921	RRM1	NA	Inverse variance weighted	16	-0.095	0.062	1.257E-01	0.879	pan	Severe COVID-19
5610_32	CJ038	Protein FAM171A1	Q5VUB5	FAM171A1	NA	Inverse variance weighted	3	-0.268	0.175	1.265E-01	0.879	pan	Severe COVID-19
13090_17	S100A6	Protein S100-A6	P06703	S100A6	NA	Inverse variance weighted	3	0.293	0.192	1.267E-01	0.879	pan	Severe COVID-19
3038_9	I-TAC	C-X-C motif chemokine 11	O14625	CXCL11	NA	Inverse variance weighted	8	-0.119	0.078	1.273E-01	0.879	pan	Severe COVID-19
18308_30	STXB6	Syntaxin-binding protein 6	Q8NFX7	STXBP6	NA	Wald ratio	1	-0.457	0.300	1.274E-01	0.879	pan	Severe COVID-19
17776_15	PECR	Peroxisomal trans-2-enoyl-CoA reductase	Q9BY49	PECR	NA	Wald ratio	1	0.240	0.158	1.279E-01	0.879	pan	Severe COVID-19
5069_9	DAF	Complement decay-accelerating factor	P08174	CD55	NA	Inverse variance weighted	5	-0.117	0.077	1.279E-01	0.879	pan	Severe COVID-19
5475_10	PKC-B-II	Protein kinase C beta type (splice variant beta-II)	P05771	PRKCB	NA	Wald ratio	1	-0.301	0.198	1.280E-01	0.879	pan	Severe COVID-19
8403_18	Fatty acid synthase	Fatty acid synthase	P49327	FASN	NA	Wald ratio	1	0.161	0.106	1.283E-01	0.879	pan	Severe COVID-19
7806_33	B4GT7	Beta-1,4-galactosyltransferase 7	Q9UBV7	B4GALT7	NA	Inverse variance weighted	8	0.075	0.049	1.294E-01	0.879	pan	Severe COVID-19
12522_6	RD23B	UV excision repair protein RAD23 homolog B	P54727	RAD23B	NA	Wald ratio	1	0.405	0.267	1.296E-01	0.879	pan	Severe COVID-19
15548_35	SERA	D-3-phosphoglycerate dehydrogenase	O43175	PHGDH	NA	Inverse variance weighted	2	0.314	0.207	1.299E-01	0.879	pan	Severe COVID-19
12008_3	CD7	T-cell antigen CD7	P09564	CD7	NA	Inverse variance weighted	4	0.250	0.165	1.299E-01	0.879	pan	Severe COVID-19

8954_30	M4K1	Mitogen-activated protein kinase kinase kinase kinase 1	Q92918	MAP4K1	NA	Inverse variance weighted	6	-0.081	0.054	1.302E-01	0.879	pan	Severe COVID-19
9815_5	RHG01:CRAL-TRIO	Rho GTPase-activating protein 1:Cellular retinaldehyde-TRIO domain	Q07960	ARHGAP1	NA	Wald ratio	1	-0.545	0.361	1.306E-01	0.879	pan	Severe COVID-19
5227_60	PDK1	[Pyruvate dehydrogenase (acetyl-transferring)] kinase isozyme 1, mitochondrial	Q15118	PDK1	NA	Inverse variance weighted	3	0.157	0.104	1.308E-01	0.879	pan	Severe COVID-19
13610_9	MAGE-10	Melanoma-associated antigen 10	P43363	MAGEA10	NA	Inverse variance weighted	2	-0.426	0.283	1.314E-01	0.879	pan	Severe COVID-19
17671_58	ING4	Inhibitor of growth protein 4	Q9UNL4	ING4	NA	Inverse variance weighted	4	0.236	0.157	1.315E-01	0.879	pan	Severe COVID-19
10451_11	NUCB1	Nucleobindin-1	Q02818	NUCB1	NA	Wald ratio	1	0.225	0.149	1.317E-01	0.879	pan	Severe COVID-19
9779_63	HERP2	Homocysteine-responsive endoplasmic reticulum-resident ubiquitin-like domain member 2 protein	Q9BSE4	HERPUD2	NA	Wald ratio	1	0.480	0.319	1.319E-01	0.879	pan	Severe COVID-19
4192_10	AK1A1	Alcohol dehydrogenase [NADP(+)]	P14550	AKR1A1	NA	Inverse variance weighted	10	0.066	0.044	1.323E-01	0.879	pan	Severe COVID-19
18183_3	ARH	Low density lipoprotein receptor adapter protein 1	Q5SW96	LDLRAP1	NA	Wald ratio	1	-0.275	0.183	1.330E-01	0.879	pan	Severe COVID-19
8289_8	GPNMB:ECD	Transmembrane glycoprotein NMB:Extracellular domain	Q14956	GPNMB	NA	Inverse variance weighted	8	-0.090	0.060	1.333E-01	0.879	pan	Severe COVID-19
18408_26	ARF4	ADP-ribosylation factor 4	P18085	ARF4	NA	Wald ratio	1	-0.516	0.344	1.334E-01	0.879	pan	Severe COVID-19
8337_65	PTPRU	Receptor-type tyrosine-protein phosphatase U	Q92729	PTPRU	NA	Inverse variance weighted	5	-0.147	0.098	1.350E-01	0.882	pan	Severe COVID-19
3461_58	PGCB	Brevican core protein	Q96GW7	BCAN	NA	Inverse variance weighted	3	-0.181	0.121	1.352E-01	0.882	pan	Severe COVID-19
2966_65	SCGF-beta	Stem cell growth factor-beta	Q9Y240	CLEC11A	NA	Inverse variance weighted	2	0.380	0.254	1.358E-01	0.882	pan	Severe COVID-19
5731_1	ISK6	Serine protease inhibitor Kazal-type 6	Q6UWN8	SPINK6	NA	Inverse variance weighted	13	-0.067	0.045	1.362E-01	0.882	pan	Severe COVID-19
3175_51	ATS13	A disintegrin and metalloproteinase with thrombospondin motifs 13	Q76LX8	ADAMTS13	NA	Inverse variance weighted	13	-0.070	0.047	1.366E-01	0.882	pan	Severe COVID-19
5649_83	PSG4	Pregnancy-specific beta-1-glycoprotein 4	Q00888	PSG4	NA	Inverse variance weighted	4	0.258	0.173	1.367E-01	0.882	pan	Severe COVID-19
12643_4	ARRB1	Beta-arrestin-1	P49407	ARRB1	NA	Wald ratio	1	-0.288	0.194	1.379E-01	0.882	pan	Severe COVID-19
10977_55	UCMA	Unique cartilage matrix-associated protein	Q8WVF2	UCMA	NA	Inverse variance weighted	8	0.074	0.050	1.381E-01	0.882	pan	Severe COVID-19
18174_79	PDC6I	Programmed cell death 6-interacting protein	Q8WUM4	PDCD6IP	NA	Inverse variance weighted	2	-0.201	0.136	1.384E-01	0.882	pan	Severe COVID-19
15388_24	FcRIIIa	Low affinity immunoglobulin gamma Fc region receptor III-A	P08637	FCGR3A	NA	Inverse variance weighted	11	-0.105	0.071	1.389E-01	0.882	pan	Severe COVID-19
5810_25	Cripto	Teratocarcinoma-derived growth factor 1	P13385	TDGF1	NA	Inverse variance weighted	20	-0.075	0.051	1.396E-01	0.882	pan	Severe COVID-19
9385_4	GAA	Lysosomal alpha-glucosidase	P10253	GAA	NA	Inverse variance weighted	6	-0.111	0.075	1.398E-01	0.882	pan	Severe COVID-19
7856_51	F151A	Protein FAM151A	Q8WW52	FAM151A	NA	Inverse variance weighted	11	-0.075	0.051	1.399E-01	0.882	pan	Severe COVID-19
9100_32	CE046	Uncharacterized protein C5orf46	Q6UWT4	C5orf46	NA	Wald ratio	1	-0.302	0.205	1.401E-01	0.882	pan	Severe COVID-19
17153_46	KI2L3	Killer cell immunoglobulin-like receptor 2DL3	P43628	KIR2DL3	NA	Inverse variance weighted	10	0.112	0.076	1.405E-01	0.882	pan	Severe COVID-19
15299_102	MESD2	LDLR chaperone MESD	Q14696	MESD	NA	Wald ratio	1	0.484	0.329	1.411E-01	0.882	pan	Severe COVID-19
12501_10	TBCA	Tubulin-specific chaperone A	O75347	TBCA	NA	Inverse variance weighted	4	0.205	0.140	1.418E-01	0.882	pan	Severe COVID-19
5358_3	OMD	Osteomodulin	Q99983	OMD	NA	Inverse variance weighted	3	0.246	0.168	1.423E-01	0.882	pan	Severe COVID-19
3324_51	LY9	T-lymphocyte surface antigen Ly-9	Q9HBG7	LY9	NA	Inverse variance weighted	6	0.119	0.081	1.428E-01	0.882	pan	Severe COVID-19
9638_2	TIGIT	T-cell immunoreceptor with Ig and ITIM domains	Q495A1	TIGIT	NA	Wald ratio	1	-0.285	0.195	1.444E-01	0.882	pan	Severe COVID-19
3420_21	Carbonic anhydrase XIII	Carbonic anhydrase 13	Q8N1Q1	CA13	NA	Inverse variance weighted	5	-0.068	0.047	1.450E-01	0.882	pan	Severe COVID-19
12347_29	CCM2	Cerebral cavernous malformations 2 protein	Q9BSQ5	CCM2	NA	Wald ratio	1	-0.359	0.247	1.453E-01	0.882	pan	Severe COVID-19
12717_65	TOX3	TOX high mobility group box family member 3	O15405	TOX3	NA	Wald ratio	1	-0.445	0.305	1.453E-01	0.882	pan	Severe COVID-19
5107_7	Notch 1	Neurogenic locus notch homolog protein 1	P46531	NOTCH1	NA	Inverse variance weighted	3	0.203	0.140	1.457E-01	0.882	pan	Severe COVID-19
18382_109	Catechol O-methyltransferase	Catechol O-methyltransferase	P21964	COMT	NA	Wald ratio	1	-0.246	0.169	1.457E-01	0.882	pan	Severe COVID-19
9094_5	CLC4C	C-type lectin domain family 4 member C	Q8WTT0	CLEC4C	NA	Inverse variance weighted	19	-0.136	0.093	1.458E-01	0.882	pan	Severe COVID-19
15620_4	NLGN1	Neuroligin-1	Q8N2Q7	NLGN1	NA	Inverse variance weighted	3	0.264	0.182	1.465E-01	0.882	pan	Severe COVID-19
4964_67	ARTS1	Endoplasmic reticulum aminopeptidase 1	Q9NZ08	ERAP1	NA	Inverse variance weighted	22	-0.050	0.034	1.467E-01	0.882	pan	Severe COVID-19
6611_8	KDEL1	KDEL motif-containing protein 1	Q6UW63	POGLUT2	NA	Wald ratio	1	0.488	0.337	1.467E-01	0.882	pan	Severe COVID-19
3348_49	BMP-1	Bone morphogenetic protein 1	P13497	BMP1	NA	Wald ratio	1	0.336	0.232	1.471E-01	0.882	pan	Severe COVID-19
13642_90	CIG49	Interferon-induced protein with tetratricopeptide repeats 3	O14879	IFIT3	NA	Inverse variance weighted	2	0.251	0.173	1.472E-01	0.882	pan	Severe COVID-19
9172_69	MMP-8	Neutrophil collagenase	P22894	MMP8	NA	Inverse variance weighted	7	-0.138	0.096	1.477E-01	0.882	pan	Severe COVID-19
5236_2	NR1D1	Nuclear receptor subfamily 1 group D member 1	P20393	NR1D1	NA	Wald ratio	1	-0.505	0.349	1.477E-01	0.882	pan	Severe COVID-19
13931_22	PSMD9	26S proteasome non-ATPase regulatory subunit 9	O00233	PSMD9	NA	Wald ratio	1	0.171	0.119	1.494E-01	0.882	pan	Severe COVID-19
11192_168	TINAL	Tubulointerstitial nephritis antigen-like	Q9GZM7	TINAGL1	NA	Inverse variance weighted	8	-0.094	0.065	1.496E-01	0.882	pan	Severe COVID-19
15383_200	Endothelin 3	Endothelin-3	P14138	EDN3	NA	Wald ratio	1	-0.641	0.445	1.498E-01	0.882	pan	Severe COVID-19
6506_54	TMEDA	Transmembrane emp24 domain-containing protein 10	P49755	TMED10	NA	Inverse variance weighted	6	0.195	0.136	1.505E-01	0.882	pan	Severe COVID-19
17419_17	TES	Testin	Q9UGI8	TES	NA	Wald ratio	1	-0.284	0.198	1.506E-01	0.882	pan	Severe COVID-19
13524_25	H6ST2	Heparan-sulfate 6-O-sulfotransferase 2	Q96MM7	HS6ST2	NA	Wald ratio	1	-0.371	0.259	1.509E-01	0.882	pan	Severe COVID-19
15514_26	Pseudocholinesterase	Cholinesterase	P06276	BCHE	NA	Inverse variance weighted	15	0.103	0.072	1.515E-01	0.882	pan	Severe COVID-19
9744_139	DNJA4	DnaJ homolog subfamily A member 4	Q8WW22	DNAJA4	NA	Wald ratio	1	0.451	0.315	1.519E-01	0.882	pan	Severe COVID-19
8248_222	SIG14	Sialic acid-binding Ig-like lectin 14	Q08ET2	SIGLEC14	NA	Inverse variance weighted	4	0.196	0.137	1.522E-01	0.882	pan	Severe COVID-19
3459_49	PDGF Rb	Platelet-derived growth factor receptor beta	P09619	PDGFRB	NA	Inverse variance weighted	12	-0.024	0.016	1.523E-01	0.882	pan	Severe COVID-19
4209_60	DRG-1	Vacuolar protein sorting-associated protein VTA1 homolog	Q9NP79	VTA1	NA	Wald ratio	1	-0.401	0.280	1.524E-01	0.882	pan	Severe COVID-19
15480_2	VNN2	Vascular non-inflammatory molecule 2	O95498	VNN2	NA	Inverse variance weighted	16	0.035	0.025	1.527E-01	0.882	pan	Severe COVID-19
6984_6	IGSF8	Immunoglobulin superfamily member 8	Q969P0	IGSF8	NA	Inverse variance weighted	2	0.144	0.101	1.531E-01	0.882	pan	Severe COVID-19
2939_10	Artemin	Artemin	Q5T4W7	ARTN	NA	Wald ratio	1	0.478	0.335	1.534E-01	0.882	pan	Severe COVID-19
3344_60	Antithrombin III	Antithrombin-III	P01008	SERPINC1	NA	Wald ratio	1	0.337	0.236	1.539E-01	0.882	pan	Severe COVID-19
12370_30	Apo F	Apolipoprotein F	Q13790	APOF	NA	Inverse variance weighted	2	-0.218	0.153	1.542E-01	0.882	pan	Severe COVID-19
3431_54	EphA1	Ephrin type-A receptor 1	P21709	EPHA1	NA	Inverse variance weighted	10	-0.049	0.035	1.550E-01	0.882	pan	Severe COVID-19
18310_26	SELW	Selenoprotein W	P63302	SELENOW	NA	Inverse variance weighted	8	0.085	0.060	1.553E-01	0.882	pan	Severe COVID-19
5102_55	MICB	MHC class I polypeptide-related sequence B	Q29980	MICB	NA	Inverse variance weighted	14	0.073	0.051	1.558E-01	0.882	pan	Severe COVID-19
8903_1	COX6C	Cytochrome c oxidase subunit 6C	P09669	COX6C	NA	Inverse variance weighted	4	0.202	0.143	1.563E-01	0.882	pan	Severe COVID-19
9838_4	SMAD1	Mothers against decapentaplegic homolog 1	Q15797	SMAD1	NA	Wald ratio	1	-0.537	0.380	1.569E-01	0.882	pan	Severe COVID-19
4498_62	NCAM-120	Neural cell adhesion molecule 1, 120 kDa isoform	P13591	NCAM1	NA	Inverse variance weighted	12	0.072	0.051	1.572E-01	0.882	pan	Severe COVID-19
5660_51	SOD3	Extracellular superoxide dismutase [Cu-Zn]	P08294	SOD3	NA	Inverse variance weighted	15	-0.050	0.035	1.572E-01	0.882	pan	Severe COVID-19
9039_47	TOIP1:Nuclear	Torsin-1A-interacting protein 1:Nuclear domain	Q5JTV8	TOR1AIP1	NA	Inverse variance weighted	2	0.307	0.217	1.573E-01	0.882	pan	Severe COVID-19
4568_17	SLIK5	SLIT and NTRK-like protein 5	O94991	SLITRK5	NA	Inverse variance weighted	4	0.150	0.107	1.582E-01	0.882	pan	Severe COVID-19
10042_8	SGK3	Serine/threonine-protein kinase Sgk3	Q96BR1	SGK3	NA	Wald ratio	1	0.484	0.343	1.587E-01	0.882	pan	Severe COVID-19
10630_5	HTAI2	Oxidoreductase HTATIP2	Q9BUP3	HTATIP2	NA	Inverse variance weighted	9	-0.056	0.039	1.588E-01	0.882	pan	Severe COVID-19
8006_12	DJB12	DnaJ homolog subfamily B member 12	Q9NXW2	DNAJB12	NA	Inverse variance weighted	2	0.171	0.122	1.588E-01	0.882	pan	Severe COVID-19
2946_52	Factor D	Complement factor D	P00746	CFD	NA	Inverse variance weighted	5	0.172	0.123	1.606E-01	0.885	pan	Severe COVID-19
17805_35	SPF30	Survival of motor neuron-related-splicing factor 30	O75940	SMNDC1	NA	Wald ratio	1	-0.461	0.329	1.619E-01	0.885	pan	Severe COVID-19
5584_21	Holo-TC II	Holo-Transcobalamin-2	P20062	TCN2	NA	Inverse variance weighted	12	0.107	0.077	1.620E-01	0.885	pan	Severe COVID-19
18873_8	CEAM8	Carcinoembryonic antigen-related cell adhesion molecule 8	P31997	CEACAM8	NA	Inverse variance weighted	12	0.113	0.081	1.621E-01	0.885	pan	Severe COVID-19
11530_37	HEM3	Porphobilinogen deaminase	P08397	HMBS	NA	Wald ratio	1	0.407	0.292	1.624E-01	0.885	pan	Severe COVID-19
19488_1	PDCD6	Programmed cell death protein 6	O75340	PDCD6	NA	Inverse variance weighted	3	0.135	0.097	1.625E-01	0.885	pan	Severe COVID-19

7784_1	Kininogen, HMW	Kininogen-1	P01042	KNG1	NA	Inverse variance weighted	2	0.322	0.230	1.625E-01	0.885	pan	Severe COVID-19
7918_114	Amylase, alpha 1A	Alpha-amylase 1	P04745	AMY1A	NA	Inverse variance weighted	17	0.078	0.056	1.628E-01	0.885	pan	Severe COVID-19
16621_77	AIBP	NAD(P)H-hydrate epimerase	Q8NCW5	NAXE	NA	Wald ratio	1	0.111	0.080	1.641E-01	0.889	pan	Severe COVID-19
9221_6	IPIL1	Inositol 1,4,5-trisphosphate receptor-interacting protein-like 1	Q6GPH6	ITPRIPL1	NA	Inverse variance weighted	5	-0.089	0.064	1.643E-01	0.889	pan	Severe COVID-19
7980_72	B3GN2	N-acetyllactosaminide beta-1,3-N-acetylglucosaminyltransferase 2	Q9NY97	B3GNT2	NA	Inverse variance weighted	5	0.170	0.122	1.652E-01	0.892	pan	Severe COVID-19
5722_78	Prolylcarboxypeptidase	Lysosomal Pro-X carboxypeptidase	P42785	PRCP	NA	Inverse variance weighted	8	0.119	0.086	1.660E-01	0.894	pan	Severe COVID-19
7179_69	NFASC	Neurofascin	O94856	NFASC	NA	Inverse variance weighted	4	0.057	0.041	1.671E-01	0.896	pan	Severe COVID-19
6409_57	GP116	Adhesion G protein-coupled receptor F5	Q8IZF2	ADGRF5	NA	Inverse variance weighted	12	-0.033	0.024	1.675E-01	0.896	pan	Severe COVID-19
2571_12	IGFBP-3	Insulin-like growth factor-binding protein 3	P17936	IGFBP3	NA	Inverse variance weighted	8	0.075	0.055	1.676E-01	0.896	pan	Severe COVID-19
11134_30	RF1ML	Peptide chain release factor 1-like, mitochondrial	Q9UGC7	MTRF1L	NA	Wald ratio	1	0.130	0.094	1.690E-01	0.900	pan	Severe COVID-19
3327_27	NET4	Netrin-4	Q9HB63	NTN4	NA	Inverse variance weighted	5	0.149	0.108	1.693E-01	0.900	pan	Severe COVID-19
7923_41	SEM4C	Semaphorin-4C	Q9C0C4	SEMA4C	NA	Inverse variance weighted	10	0.115	0.084	1.716E-01	0.910	pan	Severe COVID-19
11449_22	CAZA1	F-actin-capping protein subunit alpha-1	P52907	CAPZA1	NA	Wald ratio	1	-0.566	0.414	1.721E-01	0.910	pan	Severe COVID-19
8778_3	Noggin	Noggin	Q13253	NOG	NA	Inverse variance weighted	11	0.065	0.047	1.724E-01	0.910	pan	Severe COVID-19
6904_14	LRRT2	Leucine-rich repeat transmembrane neuronal protein 2	O43300	LRRTM2	NA	Inverse variance weighted	3	0.162	0.119	1.730E-01	0.910	pan	Severe COVID-19
15576_158	ECP	Eosinophil cationic protein	P12724	RNASE3	NA	Inverse variance weighted	9	0.051	0.038	1.732E-01	0.910	pan	Severe COVID-19
13450_49	UBP8	Ubiquitin carboxyl-terminal hydrolase 8	P40818	USP8	NA	Wald ratio	1	0.244	0.179	1.743E-01	0.913	pan	Severe COVID-19
4234_8	IL-1 R4	Interleukin-1 receptor-like 1	Q01638	IL1RL1	NA	Inverse variance weighted	24	-0.056	0.041	1.751E-01	0.915	pan	Severe COVID-19
6227_1	kallikrein 10	Kallikrein-10	O43240	KLK10	NA	Inverse variance weighted	8	0.090	0.067	1.758E-01	0.915	pan	Severe COVID-19
5620_13	AMD	Peptidyl-glycine alpha-amidating monooxygenase	P19021	PAM	NA	Inverse variance weighted	19	0.043	0.032	1.759E-01	0.915	pan	Severe COVID-19
9522_3	AIF	Apoptosis-inducing factor 1, mitochondrial	O95831	AIFM1	NA	Wald ratio	1	0.355	0.263	1.767E-01	0.917	pan	Severe COVID-19
9896_21	FOXGB	Forkhead box protein G1	P55316	FOXG1	NA	Inverse variance weighted	2	0.361	0.267	1.773E-01	0.918	pan	Severe COVID-19
17156_72	DCAK1	Serine/threonine-protein kinase DCLK1	O15075	DCLK1	NA	Inverse variance weighted	6	0.088	0.065	1.780E-01	0.919	pan	Severe COVID-19
17686_27	TBCB	Tubulin-folding cofactor B	Q99426	TBCB	NA	Wald ratio	1	0.319	0.238	1.793E-01	0.921	pan	Severe COVID-19
12859_33	PECI	Enoyl-CoA delta isomerase 2, mitochondrial	O75521	ECI2	NA	Inverse variance weighted	4	-0.071	0.053	1.795E-01	0.921	pan	Severe COVID-19
17454_15	EGFL6	Epidermal growth factor-like protein 6	Q8IUX8	EGFL6	NA	Inverse variance weighted	5	0.184	0.137	1.797E-01	0.921	pan	Severe COVID-19
4131_72	Fibronectin	Fibronectin	P02751	FN1	NA	Inverse variance weighted	2	0.413	0.309	1.811E-01	0.923	pan	Severe COVID-19
10605_22	APMAP	Adipocyte plasma membrane-associated protein	Q9HDC9	APMAP	NA	Wald ratio	1	0.351	0.262	1.812E-01	0.923	pan	Severe COVID-19
5716_49	CSAG1	Putative chondrosarcoma-associated gene 1 protein	Q6PB30	CSAG1	NA	Inverse variance weighted	3	0.210	0.157	1.813E-01	0.923	pan	Severe COVID-19
11696_7	RABP2	Cellular retinoic acid-binding protein 2	P29373	CRABP2	NA	Wald ratio	1	0.127	0.095	1.821E-01	0.923	pan	Severe COVID-19
9213_24	FTCD	Formimidoyltransferase-cyclodeaminase	O95954	FTCD	NA	Inverse variance weighted	3	-0.134	0.100	1.822E-01	0.923	pan	Severe COVID-19
19448_104	UPP1	Uridine phosphorylase 1	Q16831	UPP1	NA	Wald ratio	1	-0.280	0.211	1.829E-01	0.924	pan	Severe COVID-19
16304_6	LGR4	Leucine-rich repeat-containing G-protein coupled receptor 4	Q9BXB1	LGR4	NA	Inverse variance weighted	3	0.199	0.149	1.834E-01	0.924	pan	Severe COVID-19
11237_49	PCOC1	Procollagen C-endopeptidase enhancer 1	Q15113	PCOLCE	NA	Inverse variance weighted	4	0.257	0.194	1.848E-01	0.924	pan	Severe COVID-19
3827_22	PAK6	Serine/threonine-protein kinase PAK 6	Q9NQU5	PAK6	NA	Wald ratio	1	-0.141	0.107	1.858E-01	0.924	pan	Severe COVID-19
17694_32	PSME2	Proteasome activator complex subunit 2	Q9UL46	PSME2	NA	Inverse variance weighted	2	-0.204	0.155	1.861E-01	0.924	pan	Severe COVID-19
7624_19	ANK2	Ankyrin-2	Q01484	ANK2	NA	Inverse variance weighted	5	0.128	0.097	1.865E-01	0.924	pan	Severe COVID-19
9237_54	Lysosomal acid phosphatase	Lysosomal acid phosphatase	P11117	ACP2	NA	Inverse variance weighted	3	-0.227	0.172	1.875E-01	0.924	pan	Severe COVID-19
13381_49	B4GT1	Beta-1,4-galactosyltransferase 1	P15291	B4GALT1	NA	Inverse variance weighted	6	-0.144	0.109	1.881E-01	0.924	pan	Severe COVID-19
2211_9	TIMP-1	Metalloproteinase inhibitor 1	P01033	TIMP1	NA	Wald ratio	1	0.414	0.314	1.881E-01	0.924	pan	Severe COVID-19
13447_42	SPRN	Shadow of prion protein	Q5BIV9	SPRN	NA	Inverse variance weighted	2	-0.324	0.247	1.883E-01	0.924	pan	Severe COVID-19
14205_6	HEXI2	Protein HEXIM2	Q96MH2	HEXIM2	NA	Wald ratio	1	0.426	0.324	1.884E-01	0.924	pan	Severe COVID-19
4763_31	Afamin	Afamin	P43652	AFM	NA	Inverse variance weighted	8	0.080	0.061	1.888E-01	0.924	pan	Severe COVID-19
2658_27	TrkC	NT-3 growth factor receptor	Q16288	NTRK3	NA	Inverse variance weighted	6	-0.105	0.080	1.893E-01	0.924	pan	Severe COVID-19
10045_47	CUL3	Cullin-3	Q13618	CUL3	NA	Wald ratio	1	0.229	0.175	1.899E-01	0.924	pan	Severe COVID-19
13733_5	IL-12 p40	Interleukin-12 subunit beta	P29460	IL12B	NA	Inverse variance weighted	15	-0.068	0.052	1.901E-01	0.924	pan	Severe COVID-19
19187_21	STABP	STAM-binding protein	O95630	STAMBP	NA	Inverse variance weighted	2	0.259	0.198	1.902E-01	0.924	pan	Severe COVID-19
19601_15	ASB9	Ankyrin repeat and SOCS box protein 9	Q96DX5	ASB9	NA	Wald ratio	1	-0.114	0.087	1.902E-01	0.924	pan	Severe COVID-19
2844_53	sTie-1	Tyrosine-protein kinase receptor Tie-1, soluble	P35590	TIE1	NA	Inverse variance weighted	16	0.064	0.049	1.902E-01	0.924	pan	Severe COVID-19
14175_78	SCP2D	SCP2 sterol-binding domain-containing protein 1	Q9UJQ7	SCP2D1	NA	Inverse variance weighted	8	-0.047	0.036	1.905E-01	0.924	pan	Severe COVID-19
2700_56	Protein S	Vitamin K-dependent protein S	P07225	PROS1	NA	Inverse variance weighted	2	-0.274	0.209	1.913E-01	0.925	pan	Severe COVID-19
15333_11	SDF2	Stromal cell-derived factor 2	Q99470	SDF2	NA	Inverse variance weighted	8	0.147	0.113	1.919E-01	0.925	pan	Severe COVID-19
13125_45	Vitronectin	Vitronectin	P04004	VTN	NA	Inverse variance weighted	12	-0.133	0.102	1.920E-01	0.925	pan	Severe COVID-19
12460_18	PSA7	Proteasome subunit alpha type-7	O14818	PSMA7	NA	Inverse variance weighted	3	-0.119	0.092	1.927E-01	0.926	pan	Severe COVID-19
13724_27	FGF-19	Fibroblast growth factor 19	O95750	FGF19	NA	Inverse variance weighted	2	0.222	0.170	1.931E-01	0.926	pan	Severe COVID-19
3060_43	C9	Complement component C9	P02748	C9	NA	Inverse variance weighted	6	-0.119	0.092	1.938E-01	0.926	pan	Severe COVID-19
13422_66	ENOX2	Ecto-NOX disulfide-thiol exchanger 2	Q16206	ENOX2	NA	Wald ratio	1	0.458	0.353	1.940E-01	0.926	pan	Severe COVID-19
6603_18	KALM	Anosmin-1	P23352	ANOS1	NA	Inverse variance weighted	3	0.155	0.120	1.961E-01	0.931	pan	Severe COVID-19
5091_28	ILT-4	Leukocyte immunoglobulin-like receptor subfamily B member 2	Q8N423	LILRB2	NA	Inverse variance weighted	3	-0.365	0.283	1.968E-01	0.931	pan	Severe COVID-19
7156_2	FUT10	Alpha-(1,3)-fucosyltransferase 10	Q6P4F1	FUT10	NA	Inverse variance weighted	16	0.037	0.029	1.968E-01	0.931	pan	Severe COVID-19
9249_17	TMEM9:ECD	Transmembrane protein 9:Extracellular domain	Q9P0T7	TMEM9	NA	Wald ratio	1	-0.301	0.234	1.973E-01	0.931	pan	Severe COVID-19
7099_33	FSTL5	Follistatin-related protein 5	Q8N475	FSTL5	NA	Wald ratio	1	0.189	0.147	1.974E-01	0.931	pan	Severe COVID-19
5813_58	Epo	Erythropoietin	P01588	EPO	NA	Inverse variance weighted	5	0.129	0.100	1.976E-01	0.931	pan	Severe COVID-19
13526_5	LA:RRM	Lupus La protein:RNA recognition motif	P05455	SSB	NA	Wald ratio	1	-0.279	0.217	1.983E-01	0.932	pan	Severe COVID-19
3519_3	TARC	C-C motif chemokine 17	Q92583	CCL17	NA	Inverse variance weighted	18	-0.051	0.039	1.988E-01	0.933	pan	Severe COVID-19
3845_51	DLRB1	Dynein light chain roadblock-type 1	Q9NP97	DYNLRB1	NA	Inverse variance weighted	8	0.109	0.085	1.993E-01	0.933	pan	Severe COVID-19
6407_63	LAD1	Ladinin-1	O00515	LAD1	NA	Wald ratio	1	0.601	0.468	1.998E-01	0.933	pan	Severe COVID-19
5103_30	MO2R1:ECD	Cell surface glycoprotein CD200 receptor 1:Isoform 4, Extracellular Domain	Q8TD46	CD200R1	NA	Wald ratio	1	0.170	0.133	2.011E-01	0.936	pan	Severe COVID-19
5463_22	GAS1	Growth arrest-specific protein 1	P54826	GAS1	NA	Inverse variance weighted	2	-0.167	0.131	2.012E-01	0.936	pan	Severe COVID-19
15370_5	BOLA1	BolA-like protein 1	Q9Y3E2	BOLA1	NA	Inverse variance weighted	12	-0.047	0.037	2.018E-01	0.937	pan	Severe COVID-19
9748_31	GSTM3-3	Glutathione S-transferase Mu 3	P21266	GSTM3	NA	Inverse variance weighted	7	0.071	0.056	2.031E-01	0.940	pan	Severe COVID-19
6415_90	CPN2	Carboxypeptidase N subunit 2	P22792	CPN2	NA	Inverse variance weighted	6	0.116	0.092	2.047E-01	0.944	pan	Severe COVID-19
17691_1	TPP1	Tripeptidyl-peptidase 1	O14773	TPP1	NA	Inverse variance weighted	6	-0.088	0.070	2.049E-01	0.944	pan	Severe COVID-19
13098_93	VEGF-D	Vascular endothelial growth factor D	O43915	VEGFD	NA	Wald ratio	1	0.444	0.351	2.052E-01	0.944	pan	Severe COVID-19
4962_52	ARMEL	Cerebral dopamine neurotrophic factor	Q49AH0	CDNF	NA	Inverse variance weighted	5	-0.104	0.082	2.057E-01	0.944	pan	Severe COVID-19
3470_1	sE-Selectin	E-selectin	P16581	SELE	NA	Inverse variance weighted	8	0.113	0.090	2.077E-01	0.948	pan	Severe COVID-19
15417_3	Maspin	Serpin B5	P36952	SERPINB5	NA	Wald ratio	1	-0.147	0.117	2.085E-01	0.948	pan	Severe COVID-19
6899_37	PHS2	Pterin-4-alpha-carbinolamine dehydratase 2	Q9H0N5	PCBD2	NA	Inverse variance weighted	4	0.267	0.213	2.090E-01	0.948	pan	Severe COVID-19

8296_117	KDEL2	KDEL motif-containing protein 2	Q7Z4H8	POGLUT3	NA	Inverse variance weighted	8	0.064	0.051	2.092E-01	0.948	pan	Severe COVID-19
6586_19	ADA11	Disintegrin and metalloproteinase domain-containing protein 11	O75078	ADAM11	NA	Wald ratio	1	0.207	0.165	2.094E-01	0.948	pan	Severe COVID-19
19437_61	L-VEGF165	Isoform L-VEGF165	P15692	VEGFA	NA	Inverse variance weighted	6	-0.150	0.120	2.095E-01	0.948	pan	Severe COVID-19
19367_34	D3D2	Enoyl-CoA delta isomerase 1, mitochondrial	P42126	ECI1	NA	Inverse variance weighted	2	-0.238	0.189	2.096E-01	0.948	pan	Severe COVID-19
3284_75	BGN	Biglycan	P21810	BGN	NA	Inverse variance weighted	13	-0.065	0.052	2.106E-01	0.950	pan	Severe COVID-19
7957_2	SCG3	Secretogranin-3	Q8WXD2	SCG3	NA	Inverse variance weighted	10	-0.054	0.044	2.115E-01	0.951	pan	Severe COVID-19
9960_2	TBCD5	TBC1 domain family member 5	Q92609	TBC1D5	NA	Wald ratio	1	-0.367	0.294	2.116E-01	0.951	pan	Severe COVID-19
8814_33	SAPL1	Proactivator polypeptide-like 1	Q6NUJ1	PSAPL1	NA	Inverse variance weighted	10	-0.049	0.039	2.124E-01	0.952	pan	Severe COVID-19
9341_1	PDGFD	Platelet-derived growth factor D	Q9GZP0	PDGFD	NA	Inverse variance weighted	3	-0.194	0.156	2.130E-01	0.952	pan	Severe COVID-19
3864_5	RS7	40S ribosomal protein S7	P62081	RPS7	NA	Wald ratio	1	-0.378	0.304	2.137E-01	0.952	pan	Severe COVID-19
10758_2	KERA	Keratocan	O60938	KERA	NA	Inverse variance weighted	2	-0.279	0.225	2.139E-01	0.952	pan	Severe COVID-19
15636_49	SORC1	VPS10 domain-containing receptor SorCS1	Q8WY21	SORCS1	NA	Wald ratio	1	-0.287	0.231	2.142E-01	0.952	pan	Severe COVID-19
17513_11	ANX11	Annexin A11	P50995	ANXA11	NA	Inverse variance weighted	3	0.149	0.120	2.153E-01	0.952	pan	Severe COVID-19
6909_40	MGAT2	Alpha-1,6-mannosyl-glycoprotein 2-beta-N-acetylglucosaminyltransferase	Q10469	MGAT2	NA	Inverse variance weighted	2	-0.214	0.173	2.156E-01	0.952	pan	Severe COVID-19
11177_16	K2C5	Keratin, type II cytoskeletal 5	P13647	KRT5	NA	Inverse variance weighted	6	-0.149	0.120	2.159E-01	0.952	pan	Severe COVID-19
4355_13	DAPK2	Death-associated protein kinase 2	Q9UIK4	DAPK2	NA	Inverse variance weighted	5	0.169	0.137	2.163E-01	0.952	pan	Severe COVID-19
18216_22	IL-11 RA	Interleukin-11 receptor subunit alpha	Q14626	IL11RA	NA	Inverse variance weighted	7	-0.086	0.070	2.167E-01	0.952	pan	Severe COVID-19
18280_29	DTD2	Putative D-tyrosyl-tRNA(Tyr) deacylase 2	Q96FN9	DTD2	NA	Inverse variance weighted	2	-0.180	0.146	2.168E-01	0.952	pan	Severe COVID-19
18875_125	Chondrocalcin	Chondrocalcin	P02458	COL2A1	NA	Inverse variance weighted	18	-0.039	0.031	2.189E-01	0.957	pan	Severe COVID-19
11608_5	MLP3B	Microtubule-associated proteins 1A/1B light chain 3B	Q9GZQ8	MAP1LC3B	NA	Wald ratio	1	-0.486	0.395	2.190E-01	0.957	pan	Severe COVID-19
11493_169	DYL2	Dynein light chain 2, cytoplasmic	Q96FJ2	DYNLL2	NA	Wald ratio	1	-0.357	0.292	2.212E-01	0.957	pan	Severe COVID-19
10015_119	KCAB2	Voltage-gated potassium channel subunit beta-2	Q13303	KCNAB2	NA	Inverse variance weighted	2	-0.238	0.195	2.214E-01	0.957	pan	Severe COVID-19
16300_4	TREM2	Triggering receptor expressed on myeloid cells 2	Q9NZC2	TREM2	NA	Inverse variance weighted	20	0.042	0.034	2.215E-01	0.957	pan	Severe COVID-19
4129_72	Factor B	Complement factor B	P00751	CFB	NA	Inverse variance weighted	3	-0.168	0.138	2.215E-01	0.957	pan	Severe COVID-19
11481_25	TIMD3	Hepatitis A virus cellular receptor 2	Q8TDQ0	HAVCR2	NA	Wald ratio	1	-0.421	0.345	2.216E-01	0.957	pan	Severe COVID-19
12457_10	MTND	1,2-dihydroxy-3-keto-5-methylthiopentene dioxygenase	Q9BV57	ADI1	NA	Wald ratio	1	0.321	0.263	2.218E-01	0.957	pan	Severe COVID-19
15560_52	Apo-TC II	Transcobalamin-2	P20062	TCN2	NA	Inverse variance weighted	10	-0.043	0.035	2.222E-01	0.957	pan	Severe COVID-19
19602_36	jun-D	Transcription factor jun-D	P17535	JUND	NA	Wald ratio	1	-0.367	0.301	2.237E-01	0.957	pan	Severe COVID-19
4159_130	Factor H	Complement factor H	P08603	CFH	NA	Wald ratio	1	0.284	0.233	2.237E-01	0.957	pan	Severe COVID-19
7928_183	TPST1	Protein-tyrosine sulfotransferase 1	O60507	TPST1	NA	Inverse variance weighted	11	0.052	0.043	2.238E-01	0.957	pan	Severe COVID-19
9928_125	LECT1	Leukocyte cell-derived chemotaxin 1	O75829	CNMD	NA	Wald ratio	1	-0.312	0.257	2.241E-01	0.957	pan	Severe COVID-19
3376_49	IL-17 RD	Interleukin-17 receptor D	Q8NFM7	IL17RD	NA	Inverse variance weighted	6	0.060	0.049	2.246E-01	0.957	pan	Severe COVID-19
5654_70	TMX3	Protein disulfide-isomerase TMX3	Q96JJ7	TMX3	NA	Inverse variance weighted	10	0.082	0.068	2.247E-01	0.957	pan	Severe COVID-19
11152_46	kallikrein 13	Kallikrein-13	Q9UKR3	KLK13	NA	Inverse variance weighted	4	-0.220	0.182	2.253E-01	0.957	pan	Severe COVID-19
15449_33	TIM-4	T-cell immunoglobulin and mucin domain-containing protein 4	Q96H15	TIMD4	NA	Inverse variance weighted	19	-0.080	0.066	2.261E-01	0.957	pan	Severe COVID-19
6478_2	IGLO5	IgLON family member 5	A6NGN9	IGLON5	NA	Wald ratio	1	0.414	0.343	2.266E-01	0.957	pan	Severe COVID-19
10672_75	SP-B	Pulmonary surfactant-associated protein B	P07988	SFTPB	NA	Inverse variance weighted	6	0.138	0.114	2.269E-01	0.957	pan	Severe COVID-19
3592_4	CAMK1	Calcium/calmodulin-dependent protein kinase type 1	Q14012	CAMK1	NA	Inverse variance weighted	11	-0.074	0.062	2.278E-01	0.957	pan	Severe COVID-19
15562_24	BGLR	Beta-glucuronidase	P08236	GUSB	NA	Inverse variance weighted	13	0.061	0.051	2.280E-01	0.957	pan	Severe COVID-19
9226_6	TMUB2	Transmembrane and ubiquitin-like domain-containing protein 2	Q71RG4	TMUB2	NA	Wald ratio	1	-0.427	0.355	2.286E-01	0.957	pan	Severe COVID-19
3029_52	DC-SIGN	CD209 antigen	Q9NNX6	CD209	NA	Inverse variance weighted	9	0.074	0.061	2.288E-01	0.957	pan	Severe COVID-19
15321_8	CPLX2	Complexin-2	Q6PUV4	CPLX2	NA	Wald ratio	1	0.394	0.328	2.298E-01	0.957	pan	Severe COVID-19
11109_56	SVEP1:Sushi 15-18	Sushi, von Willebrand factor type A, EGF and pentraxin domain-containing protein 1:Sushi 15-18	Q4LDE5	SVEP1	NA	Inverse variance weighted	8	0.111	0.092	2.301E-01	0.957	pan	Severe COVID-19
4982_54	Elafin	Elafin	P19957	PI3	NA	Inverse variance weighted	8	0.089	0.074	2.304E-01	0.957	pan	Severe COVID-19
12684_5	ADSV	Adseverin	Q9Y6U3	SCIN	NA	Wald ratio	1	0.273	0.228	2.323E-01	0.957	pan	Severe COVID-19
10702_1	COSA1	Collagen alpha-1(XXVIII) chain	Q2UY09	COL28A1	NA	Inverse variance weighted	2	0.291	0.244	2.329E-01	0.957	pan	Severe COVID-19
13044_5	TS101	Tumor susceptibility gene 101 protein	Q99816	TSG101	NA	Inverse variance weighted	2	-0.184	0.154	2.331E-01	0.957	pan	Severe COVID-19
2860_19	Karyopherin-a2	Importin subunit alpha-1	P52292	KPNA2	NA	Inverse variance weighted	10	-0.114	0.096	2.334E-01	0.957	pan	Severe COVID-19
10756_34	UCN3	Urocortin-3	Q969E3	UCN3	NA	Wald ratio	1	-0.277	0.232	2.339E-01	0.957	pan	Severe COVID-19
7952_2	VTI1A	Vesicle transport through interaction with t-SNAREs homolog 1A	Q96AJ9	VTI1A	NA	Wald ratio	1	-0.331	0.279	2.347E-01	0.957	pan	Severe COVID-19
17331_138	KREM1	Kremen protein 1	Q96MU8	KREMEN1	NA	Inverse variance weighted	4	-0.103	0.087	2.348E-01	0.957	pan	Severe COVID-19
9526_3	COLL1	Colipase-like protein 1	A2RUU4	CLPSL1	NA	Inverse variance weighted	6	0.137	0.116	2.353E-01	0.957	pan	Severe COVID-19
8458_111	a-Synuclein	Alpha-synuclein	P37840	SNCA	NA	Wald ratio	1	0.476	0.402	2.357E-01	0.957	pan	Severe COVID-19
7154_92	CF058	Protein LEG1 homolog	Q6P5S2	LEG1	NA	Inverse variance weighted	5	0.182	0.153	2.358E-01	0.957	pan	Severe COVID-19
14273_19	PPCE	Prolyl endopeptidase	P48147	PREP	NA	Inverse variance weighted	3	-0.106	0.090	2.358E-01	0.957	pan	Severe COVID-19
5400_52	sLeptin R	Leptin receptor, soluble	P48357	LEPR	NA	Inverse variance weighted	18	0.034	0.029	2.360E-01	0.957	pan	Severe COVID-19
19556_12	Complement receptor type 1	Complement receptor type 1	P17927	CR1	NA	Inverse variance weighted	11	0.062	0.052	2.367E-01	0.957	pan	Severe COVID-19
19289_29	DCUP	Uroporphyrinogen decarboxylase	P06132	UROD	NA	Inverse variance weighted	5	0.068	0.058	2.369E-01	0.957	pan	Severe COVID-19
17792_158	SSDH	Succinate-semialdehyde dehydrogenase, mitochondrial	P51649	ALDH5A1	NA	Inverse variance weighted	17	-0.069	0.059	2.391E-01	0.957	pan	Severe COVID-19
11140_56	CO1A1:C-term propeptide	Collagen alpha-1(I) chain:C-term propeptide	P02452	COL1A1	NA	Inverse variance weighted	8	-0.090	0.077	2.402E-01	0.957	pan	Severe COVID-19
9015_1	PRG3	Proteoglycan 3	Q9Y2Y8	PRG3	NA	Inverse variance weighted	10	0.136	0.116	2.406E-01	0.957	pan	Severe COVID-19
11239_49	TMPS6	Transmembrane protease serine 6	Q8IU80	TMPRSS6	NA	Wald ratio	1	-0.334	0.285	2.420E-01	0.957	pan	Severe COVID-19
3046_31	resistin	Resistin	Q9HD89	RETN	NA	Inverse variance weighted	16	-0.056	0.048	2.421E-01	0.957	pan	Severe COVID-19
7551_33	LRC32	Leucine-rich repeat-containing protein 32	Q14392	LRRC32	NA	Wald ratio	1	0.298	0.255	2.422E-01	0.957	pan	Severe COVID-19
3198_4	IDS	Iduronate 2-sulfatase	P22304	IDS	NA	Wald ratio	1	-0.272	0.233	2.426E-01	0.957	pan	Severe COVID-19
5018_68	Peroxiredoxin-6	Peroxiredoxin-6	P30041	PRDX6	NA	Wald ratio	1	0.295	0.252	2.426E-01	0.957	pan	Severe COVID-19
19238_12	GLNA	Glutamine synthetase	P15104	GLUL	NA	Inverse variance weighted	2	-0.291	0.249	2.426E-01	0.957	pan	Severe COVID-19
14158_17	Annexin V	Annexin A5	P08758	ANXA5	NA	Wald ratio	1	0.129	0.111	2.426E-01	0.957	pan	Severe COVID-19
2611_72	Dtk	Tyrosine-protein kinase receptor TYRO3	Q06418	TYRO3	NA	Inverse variance weighted	2	0.170	0.145	2.433E-01	0.957	pan	Severe COVID-19
11352_42	TITIN	Titin	Q8WZ42	TTN	NA	Wald ratio	1	0.392	0.337	2.443E-01	0.957	pan	Severe COVID-19
13969_24	IMA7	Importin subunit alpha-7	O60684	KPNA6	NA	Wald ratio	1	0.302	0.260	2.443E-01	0.957	pan	Severe COVID-19
9738_7	FAF2	FAS-associated factor 2	Q96CS3	FAF2	NA	Wald ratio	1	-0.665	0.572	2.449E-01	0.957	pan	Severe COVID-19
13126_52	DSC2	Desmocollin-2	Q02487	DSC2	NA	Inverse variance weighted	3	-0.096	0.083	2.450E-01	0.957	pan	Severe COVID-19
11333_82	RHG25	Rho GTPase-activating protein 25	P42331	ARHGAP25	NA	Wald ratio	1	0.119	0.103	2.455E-01	0.957	pan	Severe COVID-19
14106_46	GDP/GTP exchange protein	Rap1 GTPase-GDP dissociation stimulator 1	P52306	RAP1GDS1	NA	Wald ratio	1	0.347	0.299	2.457E-01	0.957	pan	Severe COVID-19
4721_54	TFF3	Trefoil factor 3	Q07654	TFF3	NA	Inverse variance weighted	3	-0.181	0.156	2.472E-01	0.957	pan	Severe COVID-19
6620_82	LIGO1	Leucine-rich repeat and immunoglobulin-like domain-containing nogo receptor-interacting protein 1	Q96FE5	LINGO1	NA	Inverse variance weighted	3	0.278	0.240	2.475E-01	0.957	pan	Severe COVID-19
13614_6	CREB-binding protein	CREB-binding protein	Q92793	CREBBP	NA	Inverse variance weighted	3	0.323	0.279	2.476E-01	0.957	pan	Severe COVID-19

9002_36	SPA11	Serpin A11	Q86U17	SERPINA11	NA	Inverse variance weighted	7	-0.119	0.103	2.479E-01	0.957	pan	Severe COVID-19
9377_25	SCF	Kit ligand	P21583	KITLG	NA	Inverse variance weighted	3	0.195	0.169	2.487E-01	0.957	pan	Severe COVID-19
8065_245	PATE4	Prostate and testis expressed protein 4	P0C8F1	PATE4	NA	Wald ratio	1	-0.140	0.122	2.492E-01	0.957	pan	Severe COVID-19
3449_58	Kallistatin	Kallistatin	P29622	SERPINA4	NA	Inverse variance weighted	7	0.100	0.087	2.494E-01	0.957	pan	Severe COVID-19
2982_82	Galectin-4	Galectin-4	P56470	LGALS4	NA	Wald ratio	1	-0.312	0.271	2.495E-01	0.957	pan	Severe COVID-19
15686_49	INHBC	Inhibin beta C chain	P55103	INHBC	NA	Inverse variance weighted	17	0.036	0.031	2.495E-01	0.957	pan	Severe COVID-19
11388_75	HE4	WAP four-disulfide core domain protein 2	Q14508	WFDC2	NA	Wald ratio	1	-0.314	0.274	2.511E-01	0.957	pan	Severe COVID-19
4922_13	MIP-3b	C-C motif chemokine 19	Q99731	CCL19	NA	Inverse variance weighted	2	0.303	0.264	2.512E-01	0.957	pan	Severe COVID-19
11643_73	DTX3L	E3 ubiquitin-protein ligase DTX3L	Q8TDB6	DTX3L	NA	Wald ratio	1	-0.401	0.349	2.517E-01	0.957	pan	Severe COVID-19
2742_68	Siglec-7	Sialic acid-binding Ig-like lectin 7	Q9Y286	SIGLEC7	NA	Inverse variance weighted	3	0.122	0.107	2.519E-01	0.957	pan	Severe COVID-19
8300_82	PEX14:N-term	Peroxisomal membrane protein PEX14:N-term	O75381	PEX14	NA	Inverse variance weighted	2	-0.280	0.245	2.527E-01	0.957	pan	Severe COVID-19
3168_8	ADAMTS-5	A disintegrin and metalloproteinase with thrombospondin motifs 5	Q9UNA0	ADAMTS5	NA	Inverse variance weighted	4	0.048	0.042	2.542E-01	0.957	pan	Severe COVID-19
6626_81	CHSTC	Carbohydrate sulfotransferase 12	Q9NRB3	CHST12	NA	Inverse variance weighted	2	-0.093	0.081	2.544E-01	0.957	pan	Severe COVID-19
10940_25	SRCA	Sarcalumenin	Q86TD4	SRL	NA	Inverse variance weighted	3	0.080	0.070	2.544E-01	0.957	pan	Severe COVID-19
11543_84	LIMA1	LIM domain and actin-binding protein 1	Q9UHB6	LIMA1	NA	Wald ratio	1	0.267	0.235	2.552E-01	0.957	pan	Severe COVID-19
12895_28	DGKB	Diacylglycerol kinase beta	Q9Y6T7	DGKB	NA	Wald ratio	1	0.295	0.260	2.565E-01	0.957	pan	Severe COVID-19
15580_2	EPHA7	Ephrin type-A receptor 7	Q15375	EPHA7	NA	Wald ratio	1	-0.060	0.053	2.571E-01	0.957	pan	Severe COVID-19
19561_216	PLXD1	Plexin-D1	Q9Y4D7	PLXND1	NA	Inverse variance weighted	6	0.130	0.115	2.575E-01	0.957	pan	Severe COVID-19
7244_16	TM149	IGF-like family receptor 1	Q9H665	IGFLR1	NA	Inverse variance weighted	7	-0.035	0.031	2.579E-01	0.957	pan	Severe COVID-19
17320_19	ADPPT	L-aminoadipate-semialdehyde dehydrogenase-phosphopantetheinyl transferase	Q9NRN7	AASDHPPT	NA	Wald ratio	1	-0.392	0.347	2.579E-01	0.957	pan	Severe COVID-19
7955_195	ITI heavy chain H1	Inter-alpha-trypsin inhibitor heavy chain H1	P19827	ITIH1	NA	Inverse variance weighted	3	0.231	0.204	2.588E-01	0.957	pan	Severe COVID-19
15533_97	Macrophage scavenger receptor:ECD	Macrophage scavenger receptor types I and II:Extracellular domain	P21757	MSR1	NA	Inverse variance weighted	7	-0.108	0.096	2.588E-01	0.957	pan	Severe COVID-19
13124_20	ISLR2	Immunoglobulin superfamily containing leucine-rich repeat protein 2	Q6UXK2	ISLR2	NA	Inverse variance weighted	4	-0.141	0.125	2.604E-01	0.957	pan	Severe COVID-19
3807_1	FGF23	Fibroblast growth factor 23	Q9GZV9	FGF23	NA	Wald ratio	1	-0.328	0.292	2.606E-01	0.957	pan	Severe COVID-19
3049_61	Trypsin	Trypsin-1	P07477	PRSS1	NA	Inverse variance weighted	4	0.393	0.350	2.608E-01	0.957	pan	Severe COVID-19
5803_24	C3d	Complement C3d fragment	P01024	C3	NA	Wald ratio	1	0.294	0.262	2.615E-01	0.957	pan	Severe COVID-19
13132_14	SEM5A	Semaphorin-5A	Q13591	SEMA5A	NA	Inverse variance weighted	9	-0.019	0.017	2.620E-01	0.957	pan	Severe COVID-19
12682_5	KAT3	Kynurenine--oxoglutarate transaminase 3	Q6YP21	KYAT3	NA	Inverse variance weighted	7	-0.050	0.045	2.626E-01	0.957	pan	Severe COVID-19
8804_39	COKA1	Collagen alpha-1(XX) chain	Q9P218	COL20A1	NA	Wald ratio	1	0.241	0.215	2.627E-01	0.957	pan	Severe COVID-19
3808_76	FGFR-2	Fibroblast growth factor receptor 2	P21802	FGFR2	NA	Wald ratio	1	-0.311	0.278	2.630E-01	0.957	pan	Severe COVID-19
3508_78	MDC	C-C motif chemokine 22	O00626	CCL22	NA	Inverse variance weighted	9	-0.076	0.068	2.639E-01	0.957	pan	Severe COVID-19
5238_26	PPIE	Peptidyl-prolyl cis-trans isomerase E	Q9UNP9	PPIE	NA	Inverse variance weighted	3	0.076	0.068	2.641E-01	0.957	pan	Severe COVID-19
17329_2	BDH2	3-hydroxybutyrate dehydrogenase type 2	Q9BUT1	BDH2	NA	Inverse variance weighted	2	0.100	0.090	2.654E-01	0.957	pan	Severe COVID-19
15511_37	NPTXR	Neuronal pentraxin receptor	O95502	NPTXR	NA	Inverse variance weighted	10	-0.040	0.036	2.664E-01	0.957	pan	Severe COVID-19
11241_8	ARLY	Argininosuccinate lyase	P04424	ASL	NA	Inverse variance weighted	7	-0.152	0.137	2.666E-01	0.957	pan	Severe COVID-19
18897_31	HDAC2	Histone deacetylase 2	Q92769	HDAC2	NA	Wald ratio	1	-0.458	0.412	2.672E-01	0.957	pan	Severe COVID-19
4978_54	DBNL	Drebrin-like protein	Q9UJU6	DBNL	NA	Wald ratio	1	0.264	0.238	2.672E-01	0.957	pan	Severe COVID-19
17766_5	NCF-1	Neutrophil cytosol factor 1	P14598	NCF1	NA	Inverse variance weighted	8	0.067	0.060	2.673E-01	0.957	pan	Severe COVID-19
15678_71	DKK2	Dickkopf-related protein 2	Q9UBU2	DKK2	NA	Inverse variance weighted	2	-0.425	0.383	2.673E-01	0.957	pan	Severe COVID-19
5691_2	CRLD2	Cysteine-rich secretory protein LCCL domain-containing 2	Q9H0B8	CRISPLD2	NA	Inverse variance weighted	10	0.073	0.066	2.675E-01	0.957	pan	Severe COVID-19
5481_16	RASA1	Ras GTPase-activating protein 1	P20936	RASA1	NA	Inverse variance weighted	2	-0.376	0.339	2.677E-01	0.957	pan	Severe COVID-19
17357_33	SPCS	O-phosphoseryl-tRNA(Sec) selenium transferase	Q9HD40	SEPSECS	NA	Wald ratio	1	-0.237	0.214	2.680E-01	0.957	pan	Severe COVID-19
17408_2	PMM1	Phosphomannomutase 1	Q92871	PMM1	NA	Wald ratio	1	0.320	0.290	2.692E-01	0.957	pan	Severe COVID-19
8888_33	CX068	Small integral membrane protein 9	A6NGZ8	SMIM9	NA	Inverse variance weighted	2	0.085	0.077	2.696E-01	0.957	pan	Severe COVID-19
6525_17	DUSP13	Dual specificity protein phosphatase 13 isoform A	Q6B8I1	DUSP13	NA	Inverse variance weighted	3	0.099	0.090	2.698E-01	0.957	pan	Severe COVID-19
9231_23	IMPA3	Inositol monophosphatase 3	Q9NX62	BPNT2	NA	Inverse variance weighted	12	-0.041	0.037	2.702E-01	0.957	pan	Severe COVID-19
3280_49	Aggrecan	Aggrecan core protein	P16112	ACAN	NA	Inverse variance weighted	3	-0.116	0.105	2.703E-01	0.957	pan	Severe COVID-19
9294_45	MFAP2	Microfibrillar-associated protein 2	P55001	MFAP2	NA	Inverse variance weighted	2	0.269	0.244	2.704E-01	0.957	pan	Severe COVID-19
8427_118	RSPO3	R-spondin-3	Q9BXY4	RSPO3	NA	Inverse variance weighted	4	-0.246	0.224	2.716E-01	0.957	pan	Severe COVID-19
13943_38	DPY30	Protein dpy-30 homolog	Q9C005	DPY30	NA	Inverse variance weighted	2	-0.378	0.345	2.723E-01	0.957	pan	Severe COVID-19
9870_17	SYWC	Tryptophan--tRNA ligase, cytoplasmic	P23381	WARS1	NA	Inverse variance weighted	2	-0.084	0.076	2.731E-01	0.957	pan	Severe COVID-19
7009_8	CD72	B-cell differentiation antigen CD72	P21854	CD72	NA	Inverse variance weighted	3	0.240	0.219	2.737E-01	0.957	pan	Severe COVID-19
19237_17	D-dopachrome decarboxylase	D-dopachrome decarboxylase	P30046	DDT	NA	Inverse variance weighted	9	-0.107	0.098	2.740E-01	0.957	pan	Severe COVID-19
16583_8	AP4A	Bis(5'-nucleosyl)-tetraphosphatase [asymmetrical]	P50583	NUDT2	NA	Inverse variance weighted	8	0.085	0.078	2.744E-01	0.957	pan	Severe COVID-19
17383_4	STAR5	StAR-related lipid transfer protein 5	Q9NSY2	STARD5	NA	Inverse variance weighted	6	-0.141	0.129	2.748E-01	0.957	pan	Severe COVID-19
12612_37	PSB1	Proteasome subunit beta type-1	P20618	PSMB1	NA	Inverse variance weighted	3	0.051	0.047	2.752E-01	0.957	pan	Severe COVID-19
18188_12	GATM	Glycine amidinotransferase, mitochondrial	P50440	GATM	NA	Wald ratio	1	-0.096	0.088	2.755E-01	0.957	pan	Severe COVID-19
5459_33	CYTN	Cystatin-SN	P01037	CST1	NA	Inverse variance weighted	6	0.126	0.116	2.756E-01	0.957	pan	Severe COVID-19
5452_71	ASGR1	Asialoglycoprotein receptor 1	P07306	ASGR1	NA	Wald ratio	1	0.381	0.350	2.761E-01	0.957	pan	Severe COVID-19
18895_54	GSTM4	Glutathione S-transferase Mu 4	Q03013	GSTM4	NA	Inverse variance weighted	10	0.078	0.072	2.765E-01	0.957	pan	Severe COVID-19
19379_154	RAB3D	Ras-related protein Rab-3D	O95716	RAB3D	NA	Wald ratio	1	0.286	0.262	2.766E-01	0.957	pan	Severe COVID-19
3719_2	p27Kip1	Cyclin-dependent kinase inhibitor 1B	P46527	CDKN1B	NA	Inverse variance weighted	2	0.204	0.187	2.766E-01	0.957	pan	Severe COVID-19
9212_22	CATF	Cathepsin F	Q9UBX1	CTSF	NA	Inverse variance weighted	3	0.229	0.211	2.769E-01	0.957	pan	Severe COVID-19
8097_77	LIPN	Lipase member N	Q5VXI9	LIPN	NA	Inverse variance weighted	14	-0.055	0.050	2.776E-01	0.957	pan	Severe COVID-19
4874_3	Angiogenin	Angiogenin	P03950	ANG	NA	Inverse variance weighted	6	0.106	0.097	2.780E-01	0.957	pan	Severe COVID-19
5392_73	Fas, soluble	Tumor necrosis factor receptor superfamily member 6	P25445	FAS	NA	Inverse variance weighted	3	0.221	0.204	2.781E-01	0.957	pan	Severe COVID-19
10531_18	RASN	GTPase NRas	P01111	NRAS	NA	Wald ratio	1	-0.426	0.393	2.781E-01	0.957	pan	Severe COVID-19
12350_86	ETS2	Protein C-ets-2	P15036	ETS2	NA	Inverse variance weighted	6	0.120	0.110	2.783E-01	0.957	pan	Severe COVID-19
12993_21	NPL4	Nuclear protein localization protein 4 homolog	Q8TAT6	NPLOC4	NA	Wald ratio	1	-0.624	0.576	2.784E-01	0.957	pan	Severe COVID-19
7019_13	Semaphorin-7A	Semaphorin-7A	O75326	SEMA7A	NA	Inverse variance weighted	2	0.279	0.258	2.800E-01	0.957	pan	Severe COVID-19
18295_102	GRHPR	Glyoxylate reductase/hydroxypyruvate reductase	Q9UBQ7	GRHPR	NA	Inverse variance weighted	3	-0.058	0.054	2.806E-01	0.957	pan	Severe COVID-19
9296_15	PTPRD	Receptor-type tyrosine-protein phosphatase delta	P23468	PTPRD	NA	Wald ratio	1	0.351	0.326	2.816E-01	0.957	pan	Severe COVID-19
18381_16	ALDH-E2	Aldehyde dehydrogenase, mitochondrial	P05091	ALDH2	NA	Inverse variance weighted	4	0.121	0.112	2.816E-01	0.957	pan	Severe COVID-19
17672_184	Gastric intrinsic factor	Gastric intrinsic factor	P27352	CBLIF	NA	Inverse variance weighted	13	-0.046	0.043	2.816E-01	0.957	pan	Severe COVID-19
8893_29	PARP:region 1	Poly [ADP-ribose] polymerase 1:region 1	P09874	PARP1	NA	Wald ratio	1	0.320	0.298	2.829E-01	0.957	pan	Severe COVID-19
12498_12	TX1B3	Tax1-binding protein 3	O14907	TAX1BP3	NA	Wald ratio	1	0.179	0.167	2.830E-01	0.957	pan	Severe COVID-19
6998_106	HAAH	Aspartyl/asparaginyl beta-hydroxylase	Q12797	ASPH	NA	Inverse variance weighted	3	-0.168	0.157	2.833E-01	0.957	pan	Severe COVID-19
12396_19	HIBCH	3-hydroxyisobutyryl-CoA hydrolase, mitochondrial	Q6NVY1	HIBCH	NA	Inverse variance weighted	6	0.045	0.042	2.836E-01	0.957	pan	Severe COVID-19

14008_22	DCP1A	mRNA-decapping enzyme 1A	Q9NPI6	DCP1A	NA	Inverse variance weighted	3	-0.091	0.085	2.838E-01	0.957	pan	Severe COVID-19
17332_3	ARHL2	Poly(ADP-ribose) glycohydrolase ARH3	Q9NX46	ADPRS	NA	Inverse variance weighted	5	-0.080	0.075	2.846E-01	0.957	pan	Severe COVID-19
5601_2	PGRP-L	N-acetylmuramoyl-L-alanine amidase	Q96PD5	PGLYRP2	NA	Inverse variance weighted	6	0.081	0.076	2.856E-01	0.957	pan	Severe COVID-19
12475_48	CLIC5	Chloride intracellular channel protein 5	Q9NZA1	CLIC5	NA	Inverse variance weighted	9	0.053	0.049	2.857E-01	0.957	pan	Severe COVID-19
13947_371	NUD12	Peroxisomal NADH pyrophosphatase NUDT12	Q9BQG2	NUDT12	NA	Inverse variance weighted	5	0.045	0.042	2.870E-01	0.957	pan	Severe COVID-19
17495_141	SIR3	NAD-dependent protein deacetylase sirtuin-3, mitochondrial	Q9NTG7	SIRT3	NA	Inverse variance weighted	3	-0.139	0.130	2.871E-01	0.957	pan	Severe COVID-19
5963_9	Dermokine	Dermokine	Q6E0U4	DMKN	NA	Wald ratio	1	-0.363	0.341	2.873E-01	0.957	pan	Severe COVID-19
7738_299	STX2	Syntaxin-2	P32856	STX2	NA	Inverse variance weighted	3	0.242	0.228	2.878E-01	0.957	pan	Severe COVID-19
5090_49	ILT-2	Leukocyte immunoglobulin-like receptor subfamily B member 1	Q8NHL6	LILRB1	NA	Inverse variance weighted	9	-0.090	0.085	2.884E-01	0.957	pan	Severe COVID-19
16858_384	RCN3	Reticulocalbin-3	Q96D15	RCN3	NA	Inverse variance weighted	2	-0.271	0.255	2.886E-01	0.957	pan	Severe COVID-19
5680_54	OBP2B	Odorant-binding protein 2b	Q9NPH6	OBP2B	NA	Inverse variance weighted	6	0.048	0.046	2.887E-01	0.957	pan	Severe COVID-19
8325_37	ADH4	Alcohol dehydrogenase 4	P08319	ADH4	NA	Wald ratio	1	0.217	0.205	2.888E-01	0.957	pan	Severe COVID-19
3221_54	SARP-2	Secreted frizzled-related protein 1	Q8N474	SFRP1	NA	Inverse variance weighted	6	0.082	0.077	2.890E-01	0.957	pan	Severe COVID-19
15470_11	Hexosaminidase B	Beta-hexosaminidase subunit beta	P07686	HEXB	NA	Inverse variance weighted	12	0.055	0.052	2.893E-01	0.957	pan	Severe COVID-19
19376_74	NNMT	Nicotinamide N-methyltransferase	P40261	NNMT	NA	Wald ratio	1	0.262	0.247	2.896E-01	0.957	pan	Severe COVID-19
13481_24	TCEA2	Transcription elongation factor A protein 2	Q15560	TCEA2	NA	Wald ratio	1	-0.170	0.162	2.926E-01	0.957	pan	Severe COVID-19
3026_5	Calpastatin	Calpastatin	P20810	CAST	NA	Inverse variance weighted	4	-0.163	0.155	2.927E-01	0.957	pan	Severe COVID-19
12817_1	GEM	GTP-binding protein GEM	P55040	GEM	NA	Wald ratio	1	0.569	0.542	2.939E-01	0.957	pan	Severe COVID-19
13068_139	CCS	Copper chaperone for superoxide dismutase	O14618	CCS	NA	Wald ratio	1	0.200	0.191	2.942E-01	0.957	pan	Severe COVID-19
8000_17	KI2LA	Killer cell immunoglobulin-like receptor 2DL5A	Q8N109	KIR2DL5A	NA	Inverse variance weighted	8	0.060	0.057	2.943E-01	0.957	pan	Severe COVID-19
15515_2	SAA	Serum amyloid A-1 protein	P0DJI8	SAA1	NA	Inverse variance weighted	5	-0.119	0.113	2.955E-01	0.957	pan	Severe COVID-19
3314_74	GFRa-1	GDNF family receptor alpha-1	P56159	GFRA1	NA	Inverse variance weighted	3	0.082	0.078	2.956E-01	0.957	pan	Severe COVID-19
8345_27	GPX7	Glutathione peroxidase 7	Q96SL4	GPX7	NA	Inverse variance weighted	8	0.035	0.033	2.966E-01	0.957	pan	Severe COVID-19
17812_2	TP4A1	Protein tyrosine phosphatase type IVA 1	Q93096	PTP4A1	NA	Wald ratio	1	-0.353	0.339	2.968E-01	0.957	pan	Severe COVID-19
19273_3	Glutathione reductase	Glutathione reductase, mitochondrial	P00390	GSR	NA	Inverse variance weighted	3	0.092	0.088	2.976E-01	0.957	pan	Severe COVID-19
19170_25	DPOE3	DNA polymerase epsilon subunit 3	Q9NRF9	POLE3	NA	Wald ratio	1	0.170	0.164	2.989E-01	0.957	pan	Severe COVID-19
3191_50	WFKN2	WAP, kazal, immunoglobulin, kunitz and NTR domain-containing protein 1	Q96NZ8	WFIKKN1	NA	Inverse variance weighted	2	-0.149	0.143	2.995E-01	0.957	pan	Severe COVID-19
5879_51	Dynactin subunit 2	Dynactin subunit 2	Q13561	DCTN2	NA	Wald ratio	1	-0.300	0.290	3.001E-01	0.957	pan	Severe COVID-19
4878_3	Coagulation Factor X	Coagulation Factor X	P00742	F10	NA	Wald ratio	1	0.351	0.340	3.008E-01	0.957	pan	Severe COVID-19
7049_2	ADAM 23	Disintegrin and metalloproteinase domain-containing protein 23	O75077	ADAM23	NA	Inverse variance weighted	13	-0.033	0.031	3.008E-01	0.957	pan	Severe COVID-19
13578_98	ABLM3	Actin-binding LIM protein 3	O94929	ABLIM3	NA	Wald ratio	1	-0.149	0.144	3.010E-01	0.957	pan	Severe COVID-19
7861_9	ROR2	Tyrosine-protein kinase transmembrane receptor ROR2	Q01974	ROR2	NA	Inverse variance weighted	3	-0.093	0.090	3.012E-01	0.957	pan	Severe COVID-19
5648_28	CTRB2	Chymotrypsinogen B2	Q6GPI1	CTRB2	NA	Inverse variance weighted	11	0.035	0.034	3.012E-01	0.957	pan	Severe COVID-19
16809_1	NDKM	Nucleoside diphosphate kinase, mitochondrial	O00746	NME4	NA	Wald ratio	1	0.214	0.208	3.016E-01	0.957	pan	Severe COVID-19
13632_10	Zyxin	Zyxin	Q15942	ZYX	NA	Wald ratio	1	-0.415	0.402	3.024E-01	0.957	pan	Severe COVID-19
5029_3	SEPR	Prolyl endopeptidase FAP	Q12884	FAP	NA	Inverse variance weighted	6	-0.085	0.083	3.026E-01	0.957	pan	Severe COVID-19
3194_36	GPVI	Platelet glycoprotein VI	Q9HCN6	GP6	NA	Inverse variance weighted	2	-0.113	0.110	3.031E-01	0.957	pan	Severe COVID-19
2750_3	Apo A-I	Apolipoprotein A-I	P02647	APOA1	NA	Inverse variance weighted	8	-0.066	0.064	3.033E-01	0.957	pan	Severe COVID-19
5121_3	SEM6B	Semaphorin-6B	Q9H3T3	SEMA6B	NA	Inverse variance weighted	2	0.195	0.190	3.036E-01	0.957	pan	Severe COVID-19
5765_53	DB121	Beta-defensin 121	Q5J5C9	DEFB121	NA	Wald ratio	1	0.507	0.493	3.036E-01	0.957	pan	Severe COVID-19
15466_30	CO9A1	Collagen alpha-1(IX) chain	P20849	COL9A1	NA	Inverse variance weighted	3	0.143	0.139	3.040E-01	0.957	pan	Severe COVID-19
14260_112	ARHG8	Neuroepithelial cell-transforming gene 1 protein	Q7Z628	NET1	NA	Inverse variance weighted	3	0.263	0.256	3.058E-01	0.957	pan	Severe COVID-19
9185_15	TFF1	Trefoil factor 1	P04155	TFF1	NA	Inverse variance weighted	8	0.051	0.050	3.059E-01	0.957	pan	Severe COVID-19
6223_5	GUC2B	Guanylate cyclase activator 2B	Q16661	GUCA2B	NA	Inverse variance weighted	5	-0.178	0.174	3.066E-01	0.957	pan	Severe COVID-19
6408_2	INHBC	Inhibin beta C chain	P55103	INHBC	NA	Inverse variance weighted	4	0.080	0.078	3.067E-01	0.957	pan	Severe COVID-19
3042_7	Myoglobin	Myoglobin	P02144	MB	NA	Inverse variance weighted	2	0.357	0.349	3.072E-01	0.957	pan	Severe COVID-19
15544_25	kallikrein 14	Kallikrein-14	Q9P0G3	KLK14	NA	Inverse variance weighted	5	-0.142	0.139	3.072E-01	0.957	pan	Severe COVID-19
6451_64	ASPN	Asporin	Q9BXN1	ASPN	NA	Inverse variance weighted	8	0.104	0.102	3.073E-01	0.957	pan	Severe COVID-19
4125_52	sRAGE	Advanced glycosylation end product-specific receptor, soluble	Q15109	AGER	NA	Inverse variance weighted	46	-0.045	0.044	3.074E-01	0.957	pan	Severe COVID-19
9204_33	Corticotropin-lipotropin	Pro-opiomelanocortin	P01189	POMC	NA	Inverse variance weighted	11	-0.065	0.064	3.079E-01	0.957	pan	Severe COVID-19
13105_7	SNP25	Synaptosomal-associated protein 25	P60880	SNAP25	NA	Wald ratio	1	0.322	0.316	3.084E-01	0.957	pan	Severe COVID-19
15558_63	AMPE	Glutamyl aminopeptidase	Q07075	ENPEP	NA	Inverse variance weighted	8	0.042	0.042	3.086E-01	0.957	pan	Severe COVID-19
15472_16	LRP11	Low-density lipoprotein receptor-related protein 11	Q86VZ4	LRP11	NA	Inverse variance weighted	8	-0.075	0.074	3.093E-01	0.957	pan	Severe COVID-19
3206_4	LYVE1	Lymphatic vessel endothelial hyaluronic acid receptor 1	Q9Y5Y7	LYVE1	NA	Inverse variance weighted	21	0.065	0.064	3.095E-01	0.957	pan	Severe COVID-19
9386_42	TTC9B	Tetratricopeptide repeat protein 9B	Q8N6N2	TTC9B	NA	Inverse variance weighted	2	0.126	0.124	3.097E-01	0.957	pan	Severe COVID-19
19560_23	PLXA4	Plexin-A4	Q9HCM2	PLXNA4	NA	Wald ratio	1	-0.190	0.187	3.097E-01	0.957	pan	Severe COVID-19
11347_9	Transaldolase	Transaldolase	P37837	TALDO1	NA	Inverse variance weighted	2	-0.143	0.141	3.099E-01	0.957	pan	Severe COVID-19
18922_27	CD68	Macrosialin	P34810	CD68	NA	Inverse variance weighted	4	-0.144	0.142	3.099E-01	0.957	pan	Severe COVID-19
9837_60	NAD(P)H dehydrogenase	NAD(P)H dehydrogenase [quinone] 1	P15559	NQO1	NA	Inverse variance weighted	12	0.036	0.035	3.101E-01	0.957	pan	Severe COVID-19
2201_17	Endostatin	Endostatin	P39060	COL18A1	NA	Inverse variance weighted	4	-0.089	0.088	3.106E-01	0.957	pan	Severe COVID-19
2967_8	VCAM-1	Vascular cell adhesion protein 1	P19320	VCAM1	NA	Inverse variance weighted	3	-0.194	0.191	3.106E-01	0.957	pan	Severe COVID-19
15503_20	Lefty-A	Left-right determination factor 2	O00292	LEFTY2	NA	Inverse variance weighted	12	0.037	0.037	3.106E-01	0.957	pan	Severe COVID-19
5596_75	CD5:ECD	T-cell surface glycoprotein CD5:Extracellular domain	P06127	CD5	NA	Inverse variance weighted	17	-0.084	0.083	3.107E-01	0.957	pan	Severe COVID-19
11356_19	DGC14	Protein DGCR14	Q96DF8	ESS2	NA	Wald ratio	1	0.144	0.142	3.107E-01	0.957	pan	Severe COVID-19
9826_135	Fragile histidine triad protein	Bis(5'-adenosyl)-triphosphatase	P49789	FHIT	NA	Inverse variance weighted	3	-0.184	0.181	3.108E-01	0.957	pan	Severe COVID-19
2837_3	Met	Hepatocyte growth factor receptor	P08581	MET	NA	Inverse variance weighted	7	0.077	0.076	3.109E-01	0.957	pan	Severe COVID-19
5478_50	PSMA	Glutamate carboxypeptidase 2	Q04609	FOLH1	NA	Inverse variance weighted	4	-0.082	0.081	3.110E-01	0.957	pan	Severe COVID-19
8877_22	F176C:ECD	Protein eva-1 homolog C:Extracellular domain	P58658	EVA1C	NA	Wald ratio	1	-0.243	0.241	3.117E-01	0.957	pan	Severe COVID-19
9841_197	Multifunctional protein ADE2	Multifunctional protein ADE2	P22234	PAICS	NA	Wald ratio	1	0.178	0.176	3.120E-01	0.957	pan	Severe COVID-19
6715_63	PPBN	Alkaline phosphatase, placental-like	P10696	ALPG	NA	Inverse variance weighted	3	0.157	0.156	3.121E-01	0.957	pan	Severe COVID-19
16322_10	PACAP	Marginal zone B- and B1-cell-specific protein	Q8WU39	MZB1	NA	Inverse variance weighted	10	-0.076	0.075	3.130E-01	0.957	pan	Severe COVID-19
4271_75	PFD5	Prefoldin subunit 5	Q99471	PFDN5	NA	Wald ratio	1	0.568	0.564	3.135E-01	0.957	pan	Severe COVID-19
3810_50	FGR	Tyrosine-protein kinase Fgr	P09769	FGR	NA	Inverse variance weighted	2	0.135	0.134	3.144E-01	0.957	pan	Severe COVID-19
3600_2	Chitotriosidase-1	Chitotriosidase-1	Q13231	CHIT1	NA	Inverse variance weighted	8	0.029	0.029	3.148E-01	0.957	pan	Severe COVID-19
11260_47	SPA4L	SUN domain-containing protein 5	Q8TC36	SUN5	NA	Inverse variance weighted	4	0.097	0.097	3.152E-01	0.957	pan	Severe COVID-19
13453_2	RM33	39S ribosomal protein L33, mitochondrial	O75394	MRPL33	NA	Inverse variance weighted	4	-0.146	0.146	3.153E-01	0.957	pan	Severe COVID-19
17724_3	WWOX	WW domain-containing oxidoreductase	Q9NZC7	WWOX	NA	Wald ratio	1	0.203	0.202	3.153E-01	0.957	pan	Severe COVID-19
6039_24	CRHBP	Corticotropin-releasing factor-binding protein	P24387	CRHBP	NA	Inverse variance weighted	8	0.054	0.054	3.153E-01	0.957	pan	Severe COVID-19

7921_65	FJX1	Four-jointed box protein 1	Q86VR8	FJX1	NA	Inverse variance weighted	4	0.063	0.063	3.156E-01	0.957	pan	Severe COVID-19
18173_11	ARK73	Aflatoxin B1 aldehyde reductase member 3	O95154	AKR7A3	NA	Inverse variance weighted	6	0.053	0.052	3.157E-01	0.957	pan	Severe COVID-19
5682_13	VASN	Vasorin	Q6EMK4	VASN	NA	Inverse variance weighted	2	-0.164	0.163	3.158E-01	0.957	pan	Severe COVID-19
13119_26	protein Z inhibitor	Protein Z-dependent protease inhibitor	Q9UK55	SERPINA10	NA	Inverse variance weighted	16	0.050	0.050	3.168E-01	0.957	pan	Severe COVID-19
2692_74	NPS-PLA2	Phospholipase A2, membrane associated	P14555	PLA2G2A	NA	Inverse variance weighted	7	-0.028	0.028	3.175E-01	0.957	pan	Severe COVID-19
10816_150	PILRA isoform FDF03-M14	Paired immunoglobulin-like type 2 receptor alpha isoform FDF03-M14	Q9UKJ1	PILRA	NA	Inverse variance weighted	9	-0.113	0.113	3.181E-01	0.957	pan	Severe COVID-19
4771_10	ASM3A	Acid sphingomyelinase-like phosphodiesterase 3a	Q92484	SMPDL3A	NA	Inverse variance weighted	3	0.065	0.065	3.186E-01	0.957	pan	Severe COVID-19
9565_6	PAWR	PRKC apoptosis WT1 regulator protein	Q96IZ0	PAWR	NA	Wald ratio	1	0.332	0.333	3.187E-01	0.957	pan	Severe COVID-19
10643_16	NOE3:C-term	Noelin-3:Isoform 2, C-term	Q96PB7	OLFM3	NA	Wald ratio	1	-0.305	0.306	3.190E-01	0.957	pan	Severe COVID-19
13993_20	E41L1	Band 4.1-like protein 1	Q9H4G0	EPB41L1	NA	Inverse variance weighted	2	0.257	0.258	3.192E-01	0.957	pan	Severe COVID-19
18321_38	MTDC	Bifunctional methylenetetrahydrofolate dehydrogenase/cyclohydrolase, mitochondrial	P13995	MTHFD2	NA	Inverse variance weighted	4	0.097	0.097	3.198E-01	0.957	pan	Severe COVID-19
7994_41	ERO1B	ERO1-like protein beta	Q86YB8	ERO1B	NA	Inverse variance weighted	2	-0.082	0.082	3.199E-01	0.957	pan	Severe COVID-19
8447_11	ghrelin	Appetite-regulating hormone	Q9UBU3	GHRL	NA	Inverse variance weighted	2	-0.216	0.218	3.202E-01	0.957	pan	Severe COVID-19
3171_57	amyloid precursor protein	Amyloid beta A4 protein	P05067	APP	NA	Inverse variance weighted	2	0.177	0.178	3.218E-01	0.957	pan	Severe COVID-19
2752_62	BMP-14	Growth/differentiation factor 5	P43026	GDF5	NA	Inverse variance weighted	2	0.164	0.166	3.224E-01	0.957	pan	Severe COVID-19
9504_19	RB27A	Ras-related protein Rab-27A	P51159	RAB27A	NA	Wald ratio	1	-0.154	0.156	3.224E-01	0.957	pan	Severe COVID-19
7215_18	NADH-cytochrome b5 reductase	NADH-cytochrome b5 reductase 3	P00387	CYB5R3	NA	Inverse variance weighted	2	-0.394	0.399	3.236E-01	0.957	pan	Severe COVID-19
18935_14	TLR5	Toll-like receptor 5	O60602	TLR5	NA	Inverse variance weighted	10	-0.044	0.045	3.239E-01	0.957	pan	Severe COVID-19
19334_62	TXD12	Thioredoxin domain-containing protein 12	O95881	TXNDC12	NA	Wald ratio	1	0.175	0.178	3.249E-01	0.957	pan	Severe COVID-19
7871_16	T132A	Transmembrane protein 132A	Q24JP5	TMEM132A	NA	Inverse variance weighted	9	0.044	0.045	3.249E-01	0.957	pan	Severe COVID-19
10613_33	CASC4	Protein CASC4	Q6P4E1	GOLM2	NA	Inverse variance weighted	6	-0.128	0.130	3.253E-01	0.957	pan	Severe COVID-19
6927_7	NDST1	Bifunctional heparan sulfate N-deacetylase/N-sulfotransferase 1	P52848	NDST1	NA	Inverse variance weighted	3	0.187	0.190	3.253E-01	0.957	pan	Severe COVID-19
16773_29	SCUB3	Signal peptide, CUB and EGF-like domain-containing protein 3	Q8IX30	SCUBE3	NA	Inverse variance weighted	4	0.083	0.085	3.257E-01	0.957	pan	Severe COVID-19
16916_19	SLIK6	SLIT and NTRK-like protein 6	Q9H5Y7	SLITRK6	NA	Inverse variance weighted	6	-0.108	0.110	3.263E-01	0.957	pan	Severe COVID-19
8369_102	DAG1	Dystroglycan	Q14118	DAG1	NA	Wald ratio	1	-0.254	0.259	3.266E-01	0.957	pan	Severe COVID-19
11616_9	HSF1	Heat shock factor protein 1	Q00613	HSF1	NA	Wald ratio	1	0.329	0.336	3.268E-01	0.957	pan	Severe COVID-19
3234_23	URB	Coiled-coil domain-containing protein 80	Q76M96	CCDC80	NA	Inverse variance weighted	4	0.136	0.139	3.268E-01	0.957	pan	Severe COVID-19
12432_23	CYBP	Calcyclin-binding protein	Q9HB71	CACYBP	NA	Wald ratio	1	-0.261	0.267	3.276E-01	0.957	pan	Severe COVID-19
8597_1	TMEM9:CD	Transmembrane protein 9:Cytoplasmic domain	Q9P0T7	TMEM9	NA	Inverse variance weighted	7	-0.056	0.058	3.278E-01	0.957	pan	Severe COVID-19
17783_9	MMAB	Cob(I)yrinic acid a,c-diamide adenosyltransferase, mitochondrial	Q96EY8	MMAB	NA	Inverse variance weighted	6	-0.052	0.053	3.281E-01	0.957	pan	Severe COVID-19
12975_11	Keratin 20	Keratin, type I cytoskeletal 20	P35900	KRT20	NA	Inverse variance weighted	3	-0.188	0.193	3.284E-01	0.957	pan	Severe COVID-19
15535_3	Marapsin	Serine protease 27	Q9BQR3	PRSS27	NA	Inverse variance weighted	11	0.088	0.090	3.294E-01	0.957	pan	Severe COVID-19
14054_17	IL-15 Ra	Interleukin-15 receptor subunit alpha	Q13261	IL15RA	NA	Inverse variance weighted	8	0.041	0.042	3.294E-01	0.957	pan	Severe COVID-19
12554_10	RA51D	DNA repair protein RAD51 homolog 4	O75771	RAD51D	NA	Inverse variance weighted	3	-0.216	0.221	3.297E-01	0.957	pan	Severe COVID-19
3079_62	TIG2	Retinoic acid receptor responder protein 2	Q99969	RARRES2	NA	Inverse variance weighted	2	0.079	0.081	3.298E-01	0.957	pan	Severe COVID-19
3628_3	MP2K2	Dual specificity mitogen-activated protein kinase kinase 2	P36507	MAP2K2	NA	Inverse variance weighted	6	-0.120	0.123	3.303E-01	0.957	pan	Severe COVID-19
9263_57	SIAE	Sialate O-acetylesterase	Q9HAT2	SIAE	NA	Inverse variance weighted	3	0.177	0.182	3.312E-01	0.958	pan	Severe COVID-19
2567_5	Factor I	Complement factor I	P05156	CFI	NA	Inverse variance weighted	2	0.109	0.112	3.318E-01	0.958	pan	Severe COVID-19
17828_3	S100A14	Protein S100-A14	Q9HCY8	S100A14	NA	Wald ratio	1	0.371	0.382	3.321E-01	0.958	pan	Severe COVID-19
5451_1	ALCAM	CD166 antigen	Q13740	ALCAM	NA	Inverse variance weighted	5	0.114	0.118	3.322E-01	0.958	pan	Severe COVID-19
8043_153	COMP	Cartilage oligomeric matrix protein	P49747	COMP	NA	Inverse variance weighted	8	0.065	0.067	3.327E-01	0.958	pan	Severe COVID-19
9216_100	PLXB2	Plexin-B2	O15031	PLXNB2	NA	Inverse variance weighted	8	-0.052	0.054	3.333E-01	0.958	pan	Severe COVID-19
3195_50	Granulysin	Granulysin	P22749	GNLY	NA	Inverse variance weighted	17	0.040	0.042	3.340E-01	0.958	pan	Severe COVID-19
17814_8	BPNT1	3'(2'),5'-bisphosphate nucleotidase 1	O95861	BPNT1	NA	Wald ratio	1	0.264	0.274	3.343E-01	0.958	pan	Severe COVID-19
6392_7	WISP-2	WNT1-inducible-signaling pathway protein 2	O76076	CCN5	NA	Inverse variance weighted	10	0.046	0.048	3.344E-01	0.958	pan	Severe COVID-19
9265_10	GLIP1	Glioma pathogenesis-related protein 1	P48060	GLIPR1	NA	Wald ratio	1	-0.162	0.169	3.354E-01	0.959	pan	Severe COVID-19
3727_35	PYY	Peptide YY	P10082	PYY	NA	Inverse variance weighted	4	-0.125	0.130	3.357E-01	0.959	pan	Severe COVID-19
8048_9	FTMT	Ferritin, mitochondrial	Q8N4E7	FTMT	NA	Inverse variance weighted	8	-0.079	0.083	3.368E-01	0.959	pan	Severe COVID-19
3285_23	C1r	Complement C1r subcomponent	P00736	C1R	NA	Inverse variance weighted	2	-0.482	0.503	3.371E-01	0.959	pan	Severe COVID-19
7648_9	MYPC1	Myosin-binding protein C, slow-type	Q00872	MYBPC1	NA	Inverse variance weighted	2	-0.248	0.259	3.372E-01	0.959	pan	Severe COVID-19
3166_92	Siglec-3	Myeloid cell surface antigen CD33	P20138	CD33	NA	Inverse variance weighted	6	0.031	0.032	3.382E-01	0.961	pan	Severe COVID-19
19504_22	KTHY	Thymidylate kinase	P23919	DTYMK	NA	Inverse variance weighted	3	0.247	0.258	3.391E-01	0.961	pan	Severe COVID-19
10534_40	PARP:BRCT domain	Poly [ADP-ribose] polymerase 1:BRCA1 C-terminal:BRCA1 C-terminus domian	P09874	PARP1	NA	Wald ratio	1	-0.311	0.326	3.398E-01	0.961	pan	Severe COVID-19
19277_4	KAT	Thiosulfate sulfurtransferase/rhodanese-like domain-containing protein 1	Q8NFU3	TSTD1	NA	Inverse variance weighted	3	0.087	0.092	3.400E-01	0.961	pan	Severe COVID-19
3320_49	IGFBP-7	Insulin-like growth factor-binding protein 7	Q16270	IGFBP7	NA	Inverse variance weighted	9	-0.057	0.060	3.405E-01	0.961	pan	Severe COVID-19
6373_54	DLK1	Protein delta homolog 1	P80370	DLK1	NA	Inverse variance weighted	3	-0.263	0.276	3.408E-01	0.961	pan	Severe COVID-19
6507_16	NCAM2	Neural cell adhesion molecule 2	O15394	NCAM2	NA	Inverse variance weighted	7	0.031	0.033	3.411E-01	0.961	pan	Severe COVID-19
13109_82	NEGR1	Neuronal growth regulator 1	Q7Z3B1	NEGR1	NA	Inverse variance weighted	2	-0.181	0.191	3.412E-01	0.961	pan	Severe COVID-19
15594_47	HTRA1	Serine protease HTRA1	Q92743	HTRA1	NA	Inverse variance weighted	2	-0.206	0.217	3.429E-01	0.963	pan	Severe COVID-19
12727_7	FPRP	Prostaglandin F2 receptor negative regulator	Q9P2B2	PTGFRN	NA	Inverse variance weighted	14	0.038	0.040	3.432E-01	0.963	pan	Severe COVID-19
19581_15	IGFBP-5	Insulin-like growth factor-binding protein 5	P24593	IGFBP5	NA	Wald ratio	1	0.274	0.289	3.436E-01	0.963	pan	Severe COVID-19
6077_63	CECR1	Adenosine deaminase CECR1	Q9NZK5	ADA2	NA	Inverse variance weighted	4	-0.096	0.101	3.443E-01	0.963	pan	Severe COVID-19
17515_6	STCH	Heat shock 70 kDa protein 13	P48723	HSPA13	NA	Inverse variance weighted	2	0.138	0.146	3.458E-01	0.963	pan	Severe COVID-19
12851_5	DPOLM	DNA-directed DNA/RNA polymerase mu	Q9NP87	POLM	NA	Inverse variance weighted	3	0.097	0.103	3.469E-01	0.963	pan	Severe COVID-19
19227_18	BATF	Basic leucine zipper transcriptional factor ATF-like	Q16520	BATF	NA	Wald ratio	1	0.305	0.325	3.474E-01	0.963	pan	Severe COVID-19
8687_26	T106B	Transmembrane protein 106B	Q9NUM4	TMEM106B	NA	Inverse variance weighted	3	-0.117	0.124	3.475E-01	0.963	pan	Severe COVID-19
3710_49	Angiostatin	Angiostatin	P00747	PLG	NA	Inverse variance weighted	8	-0.108	0.115	3.484E-01	0.963	pan	Severe COVID-19
19228_11	BAG-2	BAG family molecular chaperone regulator 2	O95816	BAG2	NA	Inverse variance weighted	2	-0.371	0.396	3.485E-01	0.963	pan	Severe COVID-19
7857_22	NEUT	Neurotensin/neuromedin N	P30990	NTS	NA	Inverse variance weighted	5	0.088	0.094	3.494E-01	0.963	pan	Severe COVID-19
17685_9	Apo A-IV	Apolipoprotein A-IV	P06727	APOA4	NA	Wald ratio	1	-0.351	0.376	3.501E-01	0.963	pan	Severe COVID-19
7211_2	RNase 1	Ribonuclease pancreatic	P07998	RNASE1	NA	Inverse variance weighted	6	-0.116	0.124	3.508E-01	0.963	pan	Severe COVID-19
2677_1	ERBB1	Epidermal growth factor receptor	P00533	EGFR	NA	Inverse variance weighted	5	0.086	0.092	3.525E-01	0.963	pan	Severe COVID-19
5111_15	NRX3B	Neurexin-3-beta	Q9HDB5	NRXN3	NA	Inverse variance weighted	3	0.192	0.206	3.529E-01	0.963	pan	Severe COVID-19
3043_49	ON	SPARC	P09486	SPARC	NA	Inverse variance weighted	2	0.170	0.183	3.530E-01	0.963	pan	Severe COVID-19
18181_2	TSSC4	Protein TSSC4	Q9Y5U2	TSSC4	NA	Wald ratio	1	-0.478	0.515	3.532E-01	0.963	pan	Severe COVID-19
5355_69	LIGHT	Tumor necrosis factor ligand superfamily member 14	O43557	TNFSF14	NA	Wald ratio	1	-0.139	0.150	3.534E-01	0.963	pan	Severe COVID-19
13580_2	Sperm-associated antigen 2	UDP-N-acetylhexosamine pyrophosphorylase	Q16222	UAP1	NA	Wald ratio	1	-0.428	0.462	3.542E-01	0.963	pan	Severe COVID-19
19117_3	PP14A	Protein phosphatase 1 regulatory subunit 14A	Q96A00	PPP1R14A	NA	Inverse variance weighted	2	0.186	0.201	3.545E-01	0.963	pan	Severe COVID-19

12486_8	GLRX2	Glutaredoxin-2, mitochondrial	Q9NS18	GLRX2	NA	Inverse variance weighted	3	0.085	0.092	3.546E-01	0.963	pan	Severe COVID-19
9482_110	NUDT9	ADP-ribose pyrophosphatase, mitochondrial	Q9BW91	NUDT9	NA	Wald ratio	1	-0.293	0.317	3.553E-01	0.963	pan	Severe COVID-19
16900_29	MDGA1	MAM domain-containing glycosylphosphatidylinositol anchor protein 1	Q8NFP4	MDGA1	NA	Inverse variance weighted	12	-0.029	0.032	3.558E-01	0.963	pan	Severe COVID-19
4564_2	PLXC1	Plexin-C1	O60486	PLXNC1	NA	Inverse variance weighted	11	0.020	0.022	3.558E-01	0.963	pan	Severe COVID-19
6997_32	RAB26	Ras-related protein Rab-26	Q9ULW5	RAB26	NA	Inverse variance weighted	3	-0.205	0.222	3.560E-01	0.963	pan	Severe COVID-19
9416_77	CBPM	Carboxypeptidase M	P14384	CPM	NA	Inverse variance weighted	5	0.094	0.102	3.560E-01	0.963	pan	Severe COVID-19
10080_9	EI2BA	Translation initiation factor eIF-2B subunit alpha	Q14232	EIF2B1	NA	Wald ratio	1	0.241	0.261	3.567E-01	0.963	pan	Severe COVID-19
6538_90	K2013	Uncharacterized protein KIAA2013	Q8IYS2	KIAA2013	NA	Inverse variance weighted	2	0.193	0.210	3.570E-01	0.963	pan	Severe COVID-19
5464_52	GRB2 adapter protein	Growth factor receptor-bound protein 2	P62993	GRB2	NA	Wald ratio	1	0.371	0.403	3.573E-01	0.963	pan	Severe COVID-19
10833_64	HHIP	Hedgehog-interacting protein	Q96QV1	HHIP	NA	Inverse variance weighted	3	-0.080	0.086	3.574E-01	0.963	pan	Severe COVID-19
11229_16	ERN1	Serine/threonine-protein kinase/endoribonuclease IRE1	O75460	ERN1	NA	Wald ratio	1	0.503	0.547	3.578E-01	0.963	pan	Severe COVID-19
6455_52	HPLN4	Hyaluronan and proteoglycan link protein 4	Q86UW8	HAPLN4	NA	Wald ratio	1	0.289	0.316	3.596E-01	0.963	pan	Severe COVID-19
2620_4	gp130, soluble	Interleukin-6 receptor subunit beta	P40189	IL6ST	NA	Inverse variance weighted	10	0.041	0.045	3.600E-01	0.963	pan	Severe COVID-19
12628_31	LANC2	LanC-like protein 2	Q9NS86	LANCL2	NA	Wald ratio	1	0.293	0.321	3.600E-01	0.963	pan	Severe COVID-19
9790_28	BR serine/threonine kinase 2	Serine/threonine-protein kinase BRSK2	Q8IWQ3	BRSK2	NA	Wald ratio	1	-0.089	0.097	3.601E-01	0.963	pan	Severe COVID-19
16318_12	ALK-1	Serine/threonine-protein kinase receptor R3	P37023	ACVRL1	NA	Inverse variance weighted	3	0.069	0.076	3.607E-01	0.963	pan	Severe COVID-19
17325_10	KGUA	Guanylate kinase	Q16774	GUK1	NA	Inverse variance weighted	6	-0.062	0.068	3.613E-01	0.963	pan	Severe COVID-19
16288_17	EPHA4	Ephrin type-A receptor 4	P54764	EPHA4	NA	Inverse variance weighted	5	-0.068	0.075	3.619E-01	0.963	pan	Severe COVID-19
8014_359	MANEA	Glycoprotein endo-alpha-1,2-mannosidase	Q5SRI9	MANEA	NA	Inverse variance weighted	19	-0.020	0.022	3.620E-01	0.963	pan	Severe COVID-19
3798_71	Carbonic anhydrase 9	Carbonic anhydrase 9	Q16790	CA9	NA	Wald ratio	1	0.362	0.398	3.622E-01	0.963	pan	Severe COVID-19
10600_24	CLGN	Calmegin	O14967	CLGN	NA	Inverse variance weighted	3	-0.148	0.163	3.629E-01	0.963	pan	Severe COVID-19
9793_145	IGDC4	Immunoglobulin superfamily DCC subclass member 4	Q8TDY8	IGDCC4	NA	Inverse variance weighted	8	-0.039	0.043	3.629E-01	0.963	pan	Severe COVID-19
14048_7	IL-1 R AcP	Interleukin-1 Receptor accessory protein	Q9NPH3	IL1RAP	NA	Inverse variance weighted	6	-0.021	0.023	3.633E-01	0.963	pan	Severe COVID-19
11656_110	EVL	Ena/VASP-like protein	Q9UI08	EVL	NA	Inverse variance weighted	3	0.170	0.187	3.638E-01	0.963	pan	Severe COVID-19
13231_90	IgG4, Kappa	Ig gamma-4, Kappa	P01861	IGHG4	NA	Inverse variance weighted	2	0.206	0.227	3.643E-01	0.963	pan	Severe COVID-19
11104_13	YKL-40	Chitinase-3-like protein 1	P36222	CHI3L1	NA	Inverse variance weighted	11	-0.027	0.030	3.667E-01	0.963	pan	Severe COVID-19
15589_1	Gc-Globulin, Mixed Type	Vitamin D-binding protein	P02774	GC	NA	Inverse variance weighted	4	0.091	0.101	3.669E-01	0.963	pan	Severe COVID-19
12956_40	KBP	KIF1-binding protein	Q96EK5	KIFBP	NA	Wald ratio	1	0.317	0.351	3.673E-01	0.963	pan	Severe COVID-19
2913_1	MPIF-1	C-C motif chemokine 23	P55773	CCL23	NA	Inverse variance weighted	4	0.118	0.131	3.673E-01	0.963	pan	Severe COVID-19
6600_70	RELL2	RELT-like protein 2	Q8NC24	RELL2	NA	Wald ratio	1	-0.227	0.252	3.674E-01	0.963	pan	Severe COVID-19
13423_94	CJ058	Redox-regulatory protein FAM213A	Q9BRX8	PRXL2A	NA	Wald ratio	1	0.275	0.306	3.680E-01	0.963	pan	Severe COVID-19
14156_33	14-3-3 protein beta/alpha	14-3-3 protein beta/alpha	P31946	YWHAB	NA	Wald ratio	1	0.242	0.269	3.685E-01	0.963	pan	Severe COVID-19
9898_161	ARI1A	AT-rich interactive domain-containing protein 1A	O14497	ARID1A	NA	Wald ratio	1	0.394	0.439	3.694E-01	0.963	pan	Severe COVID-19
3457_57	Periostin	Periostin	Q15063	POSTN	NA	Inverse variance weighted	4	0.057	0.064	3.706E-01	0.963	pan	Severe COVID-19
10708_3	GON2	Progonadoliberin-2	O43555	GNRH2	NA	Wald ratio	1	-0.129	0.144	3.724E-01	0.963	pan	Severe COVID-19
17799_9	6PGL	6-phosphogluconolactonase	O95336	PGLS	NA	Inverse variance weighted	4	-0.137	0.153	3.724E-01	0.963	pan	Severe COVID-19
7822_11	HRSL2	HRAS-like suppressor 2	Q9NWW9	PLAAT2	NA	Wald ratio	1	-0.286	0.321	3.733E-01	0.963	pan	Severe COVID-19
4294_16	Sphingosine kinase 1	Sphingosine kinase 1	Q9NYA1	SPHK1	NA	Inverse variance weighted	3	0.163	0.183	3.735E-01	0.963	pan	Severe COVID-19
3415_61	BSP	Bone sialoprotein 2	P21815	IBSP	NA	Inverse variance weighted	2	-0.274	0.308	3.737E-01	0.963	pan	Severe COVID-19
13113_7	Osteopontin	Osteopontin	P10451	SPP1	NA	Inverse variance weighted	5	0.072	0.081	3.738E-01	0.963	pan	Severe COVID-19
8948_13	ADA19	Disintegrin and metalloproteinase domain-containing protein 19	Q9H013	ADAM19	NA	Wald ratio	1	-0.372	0.419	3.745E-01	0.963	pan	Severe COVID-19
7947_19	AP4AT	AP-4 complex accessory subunit tepsin	Q96N21	TEPSIN	NA	Wald ratio	1	0.250	0.282	3.746E-01	0.963	pan	Severe COVID-19
5837_49	LIF sR	Leukemia inhibitory factor receptor	P42702	LIFR	NA	Inverse variance weighted	9	0.069	0.078	3.753E-01	0.963	pan	Severe COVID-19
3331_8	RGMB	RGM domain family member B	Q6NW40	RGMB	NA	Wald ratio	1	-0.286	0.323	3.755E-01	0.963	pan	Severe COVID-19
3389_7	PCI	Plasma serine protease inhibitor	P05154	SERPINA5	NA	Inverse variance weighted	2	-0.170	0.192	3.760E-01	0.963	pan	Severe COVID-19
11302_237	TENR	Tenascin-R	Q92752	TNR	NA	Inverse variance weighted	3	0.201	0.228	3.765E-01	0.963	pan	Severe COVID-19
6049_64	PTPRS	Receptor-type tyrosine-protein phosphatase S	Q13332	PTPRS	NA	Inverse variance weighted	4	-0.114	0.130	3.777E-01	0.963	pan	Severe COVID-19
6255_74	CPXM1	Probable carboxypeptidase X1	Q96SM3	CPXM1	NA	Inverse variance weighted	5	-0.058	0.066	3.777E-01	0.963	pan	Severe COVID-19
15602_43	IL-6 sRa	Interleukin-6 receptor subunit alpha	P08887	IL6R	NA	Inverse variance weighted	15	-0.036	0.041	3.780E-01	0.963	pan	Severe COVID-19
2962_50	PTHrP	Parathyroid hormone-related protein	P12272	PTHLH	NA	Inverse variance weighted	3	0.099	0.112	3.782E-01	0.963	pan	Severe COVID-19
9796_4	CEL	Bile salt-activated lipase	P19835	CEL	NA	Inverse variance weighted	7	0.044	0.050	3.783E-01	0.963	pan	Severe COVID-19
10391_1	ANGL3	Angiopoietin-related protein 3	Q9Y5C1	ANGPTL3	NA	Inverse variance weighted	9	-0.051	0.058	3.784E-01	0.963	pan	Severe COVID-19
8042_88	ISK9	Serine protease inhibitor Kazal-type 9	Q5DT21	SPINK9	NA	Inverse variance weighted	5	-0.128	0.146	3.785E-01	0.963	pan	Severe COVID-19
12356_65	Sorcin	Sorcin	P30626	SRI	NA	Wald ratio	1	0.114	0.130	3.793E-01	0.963	pan	Severe COVID-19
13094_75	RSPO3	R-spondin-3	Q9BXY4	RSPO3	NA	Inverse variance weighted	2	0.204	0.232	3.793E-01	0.963	pan	Severe COVID-19
3283_21	BGH3	Transforming growth factor-beta-induced protein ig-h3	Q15582	TGFBI	NA	Inverse variance weighted	11	-0.041	0.047	3.798E-01	0.963	pan	Severe COVID-19
18312_68	NDRG3	Protein NDRG3	Q9UGV2	NDRG3	NA	Inverse variance weighted	3	0.196	0.223	3.798E-01	0.963	pan	Severe COVID-19
7223_60	S100A13	Protein S100-A13	Q99584	S100A13	NA	Inverse variance weighted	6	0.082	0.093	3.804E-01	0.963	pan	Severe COVID-19
19372_7	MDGA2	MAM domain-containing glycosylphosphatidylinositol anchor protein 2	Q7Z553	MDGA2	NA	Inverse variance weighted	19	-0.020	0.023	3.813E-01	0.963	pan	Severe COVID-19
5202_4	PPID	Peptidyl-prolyl cis-trans isomerase D	Q08752	PPID	NA	Inverse variance weighted	5	-0.048	0.055	3.814E-01	0.963	pan	Severe COVID-19
6289_78	RHG36	Rho GTPase-activating protein 36	Q6ZRI8	ARHGAP36	NA	Wald ratio	1	-0.283	0.323	3.815E-01	0.963	pan	Severe COVID-19
7948_129	GLTD2	Glycolipid transfer protein domain-containing protein 2	A6NH11	GLTPD2	NA	Inverse variance weighted	3	0.067	0.076	3.818E-01	0.963	pan	Severe COVID-19
5598_3	GREM2	Gremlin-2	Q9H772	GREM2	NA	Wald ratio	1	-0.214	0.245	3.819E-01	0.963	pan	Severe COVID-19
14131_37	EFNB2:ECD	Ephrin-B2:Extracellular domain	P52799	EFNB2	NA	Inverse variance weighted	2	0.230	0.263	3.819E-01	0.963	pan	Severe COVID-19
3438_10	FSTL3	Follistatin-related protein 3	O95633	FSTL3	NA	Inverse variance weighted	2	0.165	0.189	3.821E-01	0.963	pan	Severe COVID-19
3352_80	Carbonic anhydrase 6	Carbonic anhydrase 6	P23280	CA6	NA	Inverse variance weighted	17	-0.031	0.036	3.824E-01	0.963	pan	Severe COVID-19
8479_4	MMP-10	Stromelysin-2	P09238	MMP10	NA	Inverse variance weighted	9	-0.056	0.065	3.825E-01	0.963	pan	Severe COVID-19
9038_12	JTB	Protein JTB	O76095	JTB	NA	Wald ratio	1	-0.229	0.263	3.826E-01	0.963	pan	Severe COVID-19
6425_87	MMP19	Matrix metalloproteinase-19	Q99542	MMP19	NA	Inverse variance weighted	4	-0.126	0.144	3.826E-01	0.963	pan	Severe COVID-19
18880_81	Collagen Type III	Collagen Type III	P02461	COL3A1	NA	Inverse variance weighted	9	-0.100	0.114	3.830E-01	0.963	pan	Severe COVID-19
18330_7	PGM2	Phosphoglucomutase-2	Q96G03	PGM2	NA	Inverse variance weighted	7	0.065	0.075	3.830E-01	0.963	pan	Severe COVID-19
16915_153	SEM4A	Semaphorin-4A	Q9H3S1	SEMA4A	NA	Inverse variance weighted	8	0.084	0.096	3.834E-01	0.963	pan	Severe COVID-19
18338_26	IDH	Isocitrate dehydrogenase [NADP] cytoplasmic	O75874	IDH1	NA	Inverse variance weighted	3	-0.075	0.086	3.843E-01	0.963	pan	Severe COVID-19
11708_2	LCN1	Lipocalin-1	P31025	LCN1	NA	Wald ratio	1	-0.377	0.433	3.843E-01	0.963	pan	Severe COVID-19
3292_75	CD48	CD48 antigen	P09326	CD48	NA	Inverse variance weighted	9	0.167	0.192	3.849E-01	0.963	pan	Severe COVID-19
9021_1	TIM-1	Hepatitis A virus cellular receptor 1	Q96D42	HAVCR1	NA	Inverse variance weighted	7	-0.041	0.047	3.850E-01	0.963	pan	Severe COVID-19
2797_56	Apo B	Apolipoprotein B	P04114	APOB	NA	Wald ratio	1	-0.262	0.302	3.856E-01	0.963	pan	Severe COVID-19
9092_33	ANGL1:N-term	Angiopoietin-related protein 1:N-term	O95841	ANGPTL1	NA	Wald ratio	1	0.248	0.286	3.859E-01	0.963	pan	Severe COVID-19

6605_17	IGFALS	Insulin-like growth factor-binding protein complex acid labile subunit	P35858	IGFALS	NA	Inverse variance weighted	3	0.182	0.211	3.870E-01	0.964	pan	Severe COVID-19
16851_50	SCO2	Protein SCO2 homolog, mitochondrial	O43819	SCO2	NA	Wald ratio	1	-0.138	0.160	3.881E-01	0.964	pan	Severe COVID-19
19622_7	Activin A	Activin A	P08476	INHBA	NA	Inverse variance weighted	2	0.204	0.236	3.883E-01	0.964	pan	Severe COVID-19
8326_63	SIGIRR	Single Ig IL-1-related receptor	Q6IA17	SIGIRR	NA	Inverse variance weighted	15	-0.059	0.068	3.884E-01	0.964	pan	Severe COVID-19
3305_6	DLL4	Delta-like protein 4	Q9NR61	DLL4	NA	Wald ratio	1	0.256	0.297	3.888E-01	0.964	pan	Severe COVID-19
15326_64	GBP1	Guanylate-binding protein 1	P32455	GBP1	NA	Inverse variance weighted	9	-0.059	0.068	3.891E-01	0.964	pan	Severe COVID-19
5462_62	Ficolin-3	Ficolin-3	O75636	FCN3	NA	Inverse variance weighted	12	-0.031	0.036	3.894E-01	0.964	pan	Severe COVID-19
8368_102	TNF sR-II	Tumor necrosis factor receptor superfamily member 1B	P20333	TNFRSF1B	NA	Wald ratio	1	-0.080	0.093	3.903E-01	0.965	pan	Severe COVID-19
11208_15	NAGPA	N-acetylglucosamine-1-phosphodiester alpha-N-acetylglucosaminidase	Q9UK23	NAGPA	NA	Inverse variance weighted	7	0.032	0.037	3.913E-01	0.965	pan	Severe COVID-19
15467_10	CTHR1	Collagen triple helix repeat-containing protein 1	Q96CG8	CTHRC1	NA	Inverse variance weighted	2	0.401	0.468	3.915E-01	0.965	pan	Severe COVID-19
11421_10	EHD4	EH domain-containing protein 4	Q9H223	EHD4	NA	Wald ratio	1	0.126	0.147	3.924E-01	0.965	pan	Severe COVID-19
2516_57	6Ckine	C-C motif chemokine 21	O00585	CCL21	NA	Inverse variance weighted	17	-0.045	0.052	3.926E-01	0.965	pan	Severe COVID-19
7968_15	CRTAM	Cytotoxic and regulatory T-cell molecule	O95727	CRTAM	NA	Inverse variance weighted	7	-0.046	0.054	3.926E-01	0.965	pan	Severe COVID-19
13700_10	annexin II	Annexin A2	P07355	ANXA2	NA	Inverse variance weighted	8	-0.080	0.094	3.931E-01	0.965	pan	Severe COVID-19
16323_8	NRX3A	Neurexin-3	Q9Y4C0	NRXN3	NA	Inverse variance weighted	3	-0.168	0.197	3.952E-01	0.968	pan	Severe COVID-19
18823_52	H2B3B	Histone H2B type 3-B	Q8N257	H2BU1	NA	Inverse variance weighted	2	0.231	0.271	3.956E-01	0.968	pan	Severe COVID-19
18831_6	LRIG1	Leucine-rich repeats and immunoglobulin-like domains protein 1	Q96JA1	LRIG1	NA	Inverse variance weighted	8	-0.032	0.038	3.968E-01	0.968	pan	Severe COVID-19
2948_58	Growth hormone receptor	Growth hormone receptor	P10912	GHR	NA	Inverse variance weighted	4	0.080	0.095	3.991E-01	0.968	pan	Severe COVID-19
16857_2	RAB2A	Ras-related protein Rab-2A	P61019	RAB2A	NA	Wald ratio	1	-0.181	0.216	4.006E-01	0.968	pan	Severe COVID-19
9339_204	FKBP2	Peptidyl-prolyl cis-trans isomerase FKBP2	P26885	FKBP2	NA	Wald ratio	1	0.270	0.321	4.006E-01	0.968	pan	Severe COVID-19
11510_31	Apo L1	Apolipoprotein L1	O14791	APOL1	NA	Inverse variance weighted	8	-0.031	0.037	4.007E-01	0.968	pan	Severe COVID-19
19175_18	MARCKSL1	MARCKS-related protein	P49006	MARCKSL1	NA	Inverse variance weighted	7	0.144	0.171	4.007E-01	0.968	pan	Severe COVID-19
14146_92	H31	Histone H3.1	P68431	H3C1	NA	Wald ratio	1	0.212	0.252	4.012E-01	0.968	pan	Severe COVID-19
5657_28	SIA4A	CMP-N-acetylneuraminate-beta-galactosamide-alpha-2,3-sialyltransferase 1	Q11201	ST3GAL1	NA	Inverse variance weighted	3	-0.077	0.091	4.012E-01	0.968	pan	Severe COVID-19
18925_24	Proteasome subunit alpha type 5	Proteasome subunit alpha type-5	P28066	PSMA5	NA	Wald ratio	1	-0.374	0.445	4.015E-01	0.968	pan	Severe COVID-19
6380_23	ICT1	Peptidyl-tRNA hydrolase ICT1, mitochondrial	Q14197	MRPL58	NA	Wald ratio	1	-0.175	0.208	4.015E-01	0.968	pan	Severe COVID-19
14091_42	Carbonyl reductase 3	Carbonyl reductase [NADPH] 3	O75828	CBR3	NA	Inverse variance weighted	5	0.037	0.044	4.020E-01	0.968	pan	Severe COVID-19
11212_7	TXND5	Thioredoxin domain-containing protein 5	Q8NBS9	TXNDC5	NA	Inverse variance weighted	5	0.071	0.085	4.022E-01	0.968	pan	Severe COVID-19
12685_57	HOME2	Homer protein homolog 2	Q9NSB8	HOMER2	NA	Inverse variance weighted	2	-0.251	0.300	4.023E-01	0.968	pan	Severe COVID-19
17777_31	SDSL	Serine dehydratase-like	Q96GA7	SDSL	NA	Inverse variance weighted	7	0.041	0.049	4.024E-01	0.968	pan	Severe COVID-19
10737_96	Serpin B1	Leukocyte elastase inhibitor	P30740	SERPINB1	NA	Wald ratio	1	0.273	0.327	4.032E-01	0.968	pan	Severe COVID-19
12437_18	ULK3	Serine/threonine-protein kinase ULK3	Q6PHR2	ULK3	NA	Wald ratio	1	0.248	0.297	4.034E-01	0.968	pan	Severe COVID-19
4801_13	PERL	Lactoperoxidase	P22079	LPO	NA	Inverse variance weighted	9	0.052	0.062	4.045E-01	0.968	pan	Severe COVID-19
12382_2	DDX58	Probable ATP-dependent RNA helicase DDX58	O95786	DDX58	NA	Inverse variance weighted	3	0.080	0.096	4.047E-01	0.968	pan	Severe COVID-19
8098_37	THAS	Thromboxane-A synthase	P24557	TBXAS1	NA	Wald ratio	1	-0.188	0.225	4.047E-01	0.968	pan	Severe COVID-19
12801_33	IRF2	Interferon regulatory factor 2	P14316	IRF2	NA	Wald ratio	1	-0.244	0.293	4.051E-01	0.968	pan	Severe COVID-19
3504_58	LEAP-1	Hepcidin	P81172	HAMP	NA	Wald ratio	1	-0.248	0.298	4.051E-01	0.968	pan	Severe COVID-19
2644_11	PKC-A	Protein kinase C alpha type	P17252	PRKCA	NA	Wald ratio	1	0.317	0.381	4.054E-01	0.968	pan	Severe COVID-19
8890_9	T132B	Transmembrane protein 132B	Q14DG7	TMEM132B	NA	Inverse variance weighted	3	-0.044	0.053	4.058E-01	0.968	pan	Severe COVID-19
7124_18	IL-21	Interleukin-21	Q9HBE4	IL21	NA	Wald ratio	1	0.189	0.227	4.064E-01	0.968	pan	Severe COVID-19
7228_2	SIA7F	Alpha-N-acetylgalactosaminide alpha-2,6-sialyltransferase 6	Q969X2	ST6GALNAC6	NA	Inverse variance weighted	3	-0.167	0.201	4.065E-01	0.968	pan	Severe COVID-19
17752_24	Arginase	Arginase-2, mitochondrial	P78540	ARG2	NA	Inverse variance weighted	2	0.118	0.143	4.073E-01	0.969	pan	Severe COVID-19
11145_72	K154L	UPF0606 protein KIAA1549L	Q6ZVL6	KIAA1549L	NA	Inverse variance weighted	7	-0.036	0.043	4.078E-01	0.969	pan	Severe COVID-19
12634_79	BCAR3:Ras-GEF	Breast cancer anti-estrogen resistance protein 3:Guanine Nucleotide Exchange Factor Domain	O75815	BCAR3	NA	Inverse variance weighted	3	-0.092	0.111	4.081E-01	0.969	pan	Severe COVID-19
4337_49	CRP	C-reactive protein	P02741	CRP	NA	Inverse variance weighted	7	0.084	0.101	4.091E-01	0.969	pan	Severe COVID-19
5349_69	DLL1	Delta-like protein 1	O00548	DLL1	NA	Inverse variance weighted	3	0.097	0.117	4.093E-01	0.969	pan	Severe COVID-19
12659_13	GTPB9	Obg-like ATPase 1	Q9NTK5	OLA1	NA	Inverse variance weighted	2	-0.099	0.120	4.102E-01	0.970	pan	Severe COVID-19
2911_27	Midkine	Midkine	P21741	MDK	NA	Inverse variance weighted	3	0.162	0.197	4.115E-01	0.972	pan	Severe COVID-19
2515_14	GFRa-2	GDNF family receptor alpha-2	O00451	GFRA2	NA	Inverse variance weighted	7	-0.042	0.051	4.116E-01	0.972	pan	Severe COVID-19
8794_13	DPEP1	Dipeptidase 1	P16444	DPEP1	NA	Inverse variance weighted	6	0.059	0.072	4.127E-01	0.972	pan	Severe COVID-19
15441_6	SAP3	Ganglioside GM2 activator	P17900	GM2A	NA	Inverse variance weighted	6	-0.066	0.080	4.129E-01	0.972	pan	Severe COVID-19
5430_66	SHPS1	Tyrosine-protein phosphatase non-receptor type substrate 1	P78324	SIRPA	NA	Inverse variance weighted	9	0.024	0.030	4.140E-01	0.972	pan	Severe COVID-19
8225_86	EPHB2	Ephrin type-B receptor 2	P29323	EPHB2	NA	Inverse variance weighted	3	0.051	0.063	4.150E-01	0.972	pan	Severe COVID-19
4435_66	ENPP7	Ectonucleotide pyrophosphatase/phosphodiesterase family member 7	Q6UWV6	ENPP7	NA	Inverse variance weighted	10	0.036	0.044	4.152E-01	0.972	pan	Severe COVID-19
11431_235	RECQ1	ATP-dependent DNA helicase Q1	P46063	RECQL	NA	Inverse variance weighted	10	0.022	0.027	4.154E-01	0.972	pan	Severe COVID-19
13460_4	CHAD	Chondroadherin	O15335	CHAD	NA	Inverse variance weighted	2	-0.149	0.184	4.171E-01	0.972	pan	Severe COVID-19
19364_163	PCNA	Proliferating cell nuclear antigen	P12004	PCNA	NA	Inverse variance weighted	2	-0.159	0.196	4.173E-01	0.972	pan	Severe COVID-19
19176_27	FA49B	Protein FAM49B	Q9NUQ9	CYRIB	NA	Wald ratio	1	0.305	0.376	4.176E-01	0.972	pan	Severe COVID-19
17140_57	PDGFD	Platelet-derived growth factor D	Q9GZP0	PDGFD	NA	Inverse variance weighted	5	0.106	0.131	4.177E-01	0.972	pan	Severe COVID-19
13659_36	AT131	Manganese-transporting ATPase 13A1	Q9HD20	ATP13A1	NA	Inverse variance weighted	2	0.181	0.223	4.179E-01	0.972	pan	Severe COVID-19
9244_27	PPT1	Palmitoyl-protein thioesterase 1	P50897	PPT1	NA	Inverse variance weighted	2	-0.074	0.091	4.180E-01	0.972	pan	Severe COVID-19
13473_55	IP3KA	Inositol-trisphosphate 3-kinase A	P23677	ITPKA	NA	Wald ratio	1	0.172	0.213	4.186E-01	0.972	pan	Severe COVID-19
9314_9	PSG5	Pregnancy-specific beta-1-glycoprotein 5	Q15238	PSG5	NA	Inverse variance weighted	4	0.119	0.147	4.186E-01	0.972	pan	Severe COVID-19
17750_8	RIR2	Ribonucleoside-diphosphate reductase subunit M2	P31350	RRM2	NA	Inverse variance weighted	2	-0.159	0.196	4.192E-01	0.972	pan	Severe COVID-19
14011_17	S100A11	Protein S100-A11	P31949	S100A11	NA	Inverse variance weighted	3	-0.068	0.084	4.197E-01	0.972	pan	Severe COVID-19
9380_2	sPLA(2)-XIII	Group XIIB secretory phospholipase A2-like protein	Q9BX93	PLA2G12B	NA	Inverse variance weighted	2	-0.165	0.205	4.207E-01	0.972	pan	Severe COVID-19
19259_176	IF2A	Eukaryotic translation initiation factor 2 subunit 1	P05198	EIF2S1	NA	Wald ratio	1	0.267	0.331	4.208E-01	0.972	pan	Severe COVID-19
13676_46	Inhibin bB chain	Inhibin beta B chain	P09529	INHBB	NA	Inverse variance weighted	6	-0.050	0.062	4.208E-01	0.972	pan	Severe COVID-19
13405_61	ISK2	Serine protease inhibitor Kazal-type 2	P20155	SPINK2	NA	Inverse variance weighted	6	-0.034	0.042	4.210E-01	0.972	pan	Severe COVID-19
18458_4	PGES2	Prostaglandin E synthase 2	Q9H7Z7	PTGES2	NA	Wald ratio	1	-0.162	0.201	4.211E-01	0.972	pan	Severe COVID-19
4140_3	IL-7	Interleukin-7	P13232	IL7	NA	Wald ratio	1	0.265	0.332	4.236E-01	0.973	pan	Severe COVID-19
4968_50	CAPG	Macrophage-capping protein	P40121	CAPG	NA	Inverse variance weighted	8	0.040	0.050	4.242E-01	0.973	pan	Severe COVID-19
14153_8	Ephrin-A3	Ephrin-A3	P52797	EFNA3	NA	Wald ratio	1	0.209	0.262	4.246E-01	0.973	pan	Severe COVID-19
5701_81	Tetranectin	Tetranectin	P05452	CLEC3B	NA	Inverse variance weighted	3	0.180	0.226	4.253E-01	0.973	pan	Severe COVID-19
3403_1	TPSB2	Tryptase beta-2	P20231	TPSB2	NA	Inverse variance weighted	10	0.031	0.039	4.255E-01	0.973	pan	Severe COVID-19
5109_24	Nr-CAM	Neuronal cell adhesion molecule	Q92823	NRCAM	NA	Wald ratio	1	0.252	0.317	4.257E-01	0.973	pan	Severe COVID-19
7950_142	BTNL9	Butyrophilin-like protein 9	Q6UXG8	BTNL9	NA	Wald ratio	1	0.175	0.220	4.259E-01	0.973	pan	Severe COVID-19
18214_2	GSH0	Glutamate--cysteine ligase regulatory subunit	P48507	GCLM	NA	Inverse variance weighted	2	0.134	0.168	4.259E-01	0.973	pan	Severe COVID-19

5015_15	PAFAH	Platelet-activating factor acetylhydrolase	Q13093	PLA2G7	NA	Inverse variance weighted	10	-0.047	0.059	4.259E-01	0.973	pan	Severe COVID-19
11308_8	CR3L4	Cyclic AMP-responsive element-binding protein 3-like protein 4	Q8TEY5	CREB3L4	NA	Inverse variance weighted	8	0.072	0.090	4.265E-01	0.973	pan	Severe COVID-19
11424_4	FAAA	Fumarylacetoacetase	P16930	FAH	NA	Inverse variance weighted	7	0.054	0.068	4.271E-01	0.973	pan	Severe COVID-19
7810_20	C1QTNF5	Complement C1q tumor necrosis factor-related protein 5	Q9BXJ0	C1QTNF5	NA	Inverse variance weighted	10	0.048	0.061	4.273E-01	0.973	pan	Severe COVID-19
4588_1	PH	Pancreatic hormone	P01298	PPY	NA	Inverse variance weighted	9	-0.089	0.113	4.274E-01	0.973	pan	Severe COVID-19
3516_60	SDF-1	Stromal cell-derived factor 1	P48061	CXCL12	NA	Inverse variance weighted	2	-0.077	0.097	4.296E-01	0.973	pan	Severe COVID-19
5021_13	PPase	Inorganic pyrophosphatase	Q15181	PPA1	NA	Inverse variance weighted	5	-0.077	0.097	4.303E-01	0.973	pan	Severe COVID-19
2778_10	IL-22	Interleukin-22	Q9GZX6	IL22	NA	Wald ratio	1	-0.199	0.252	4.304E-01	0.973	pan	Severe COVID-19
19153_53	MPPD2	Metallophosphoesterase MPPED2	Q15777	MPPED2	NA	Inverse variance weighted	2	0.138	0.175	4.305E-01	0.973	pan	Severe COVID-19
2843_13	SPINT2	Kunitz-type protease inhibitor 2	O43291	SPINT2	NA	Inverse variance weighted	15	0.031	0.039	4.315E-01	0.973	pan	Severe COVID-19
8842_16	GRM1C	GRAM domain-containing protein 1C	Q8IYS0	GRAMD1C	NA	Inverse variance weighted	9	0.060	0.077	4.317E-01	0.973	pan	Severe COVID-19
8484_24	Leptin	Leptin	P41159	LEP	NA	Inverse variance weighted	4	0.173	0.220	4.320E-01	0.973	pan	Severe COVID-19
16760_2	IL-26	Interleukin-26	Q9NPH9	IL26	NA	Inverse variance weighted	4	-0.128	0.163	4.325E-01	0.973	pan	Severe COVID-19
13488_3	ARFP1	Arfaptin-1	P53367	ARFIP1	NA	Inverse variance weighted	2	0.082	0.104	4.326E-01	0.973	pan	Severe COVID-19
3365_7	Dkk-4	Dickkopf-related protein 4	Q9UBT3	DKK4	NA	Inverse variance weighted	4	0.134	0.171	4.327E-01	0.973	pan	Severe COVID-19
17350_13	CHM2B	Charged multivesicular body protein 2b	Q9UQN3	CHMP2B	NA	Inverse variance weighted	3	-0.059	0.075	4.330E-01	0.973	pan	Severe COVID-19
3310_62	FCG2B	Low affinity immunoglobulin gamma Fc region receptor II-b	P31994	FCGR2B	NA	Inverse variance weighted	12	0.054	0.069	4.334E-01	0.973	pan	Severe COVID-19
10693_43	KLRG2:C-term	Killer cell lectin-like receptor subfamily G member 2:C-term	A4D1S0	KLRG2	NA	Inverse variance weighted	4	0.174	0.222	4.339E-01	0.973	pan	Severe COVID-19
10851_77	IL27B	Interleukin-27 subunit beta	Q14213	EBI3	NA	Inverse variance weighted	10	-0.023	0.029	4.350E-01	0.973	pan	Severe COVID-19
8074_32	TMM70	Transmembrane protein 70, mitochondrial	Q9BUB7	TMEM70	NA	Inverse variance weighted	3	0.150	0.192	4.351E-01	0.973	pan	Severe COVID-19
11601_26	DHX8	ATP-dependent RNA helicase DHX8	Q14562	DHX8	NA	Inverse variance weighted	4	0.095	0.122	4.370E-01	0.973	pan	Severe COVID-19
17164_15	annexin IV	Annexin A4	P09525	ANXA4	NA	Inverse variance weighted	2	0.123	0.158	4.379E-01	0.973	pan	Severe COVID-19
8773_172	EMIL3:region 1	EMILIN-3:region 1	Q9NT22	EMILIN3	NA	Inverse variance weighted	6	0.047	0.061	4.385E-01	0.973	pan	Severe COVID-19
4152_58	Prekallikrein	Plasma kallikrein	P03952	KLKB1	NA	Inverse variance weighted	4	0.111	0.144	4.392E-01	0.973	pan	Severe COVID-19
5698_60	Tenascin-X	Tenascin-X	P22105	TNXB	NA	Inverse variance weighted	13	0.027	0.035	4.398E-01	0.973	pan	Severe COVID-19
8231_122	VEGF sR1	Vascular endothelial growth factor receptor 1	P17948	FLT1	NA	Inverse variance weighted	4	0.157	0.203	4.405E-01	0.973	pan	Severe COVID-19
17137_160	Beta-globin	Hemoglobin subunit beta	P68871	HBB	NA	Inverse variance weighted	2	0.166	0.215	4.408E-01	0.973	pan	Severe COVID-19
14082_56	TLN2	Talin-2	Q9Y4G6	TLN2	NA	Wald ratio	1	0.236	0.307	4.421E-01	0.973	pan	Severe COVID-19
19230_12	GSTT1	Glutathione S-transferase theta-1	P30711	GSTT1	NA	Inverse variance weighted	11	0.038	0.049	4.422E-01	0.973	pan	Severe COVID-19
7809_22	SPAT9	Spermatogenesis-associated protein 9	Q9BWV2	SPATA9	NA	Wald ratio	1	-0.194	0.252	4.429E-01	0.973	pan	Severe COVID-19
18340_2	Proteasome beta chain	Proteasome subunit beta type-4	P28070	PSMB4	NA	Inverse variance weighted	4	-0.041	0.053	4.443E-01	0.973	pan	Severe COVID-19
9971_5	CSMD2	CUB and sushi domain-containing protein 2	Q7Z408	CSMD2	NA	Wald ratio	1	0.280	0.367	4.453E-01	0.973	pan	Severe COVID-19
4145_58	Neurotrophin-3	Neurotrophin-3	P20783	NTF3	NA	Inverse variance weighted	5	0.088	0.116	4.453E-01	0.973	pan	Severe COVID-19
6383_90	TLL1	Tolloid-like protein 1	O43897	TLL1	NA	Inverse variance weighted	4	0.140	0.183	4.456E-01	0.973	pan	Severe COVID-19
12687_2	DECR	2,4-dienoyl-CoA reductase, mitochondrial	Q16698	DECR1	NA	Inverse variance weighted	5	-0.084	0.111	4.457E-01	0.973	pan	Severe COVID-19
10047_12	NCF-2	Neutrophil cytosol factor 2	P19878	NCF2	NA	Inverse variance weighted	4	0.107	0.140	4.457E-01	0.973	pan	Severe COVID-19
15367_38	LPLC1	BPI fold-containing family B member 1	Q8TDL5	BPIFB1	NA	Inverse variance weighted	15	0.035	0.046	4.459E-01	0.973	pan	Severe COVID-19
12573_80	TRIM3	Tripartite motif-containing protein 3	O75382	TRIM3	NA	Wald ratio	1	-0.187	0.246	4.460E-01	0.973	pan	Severe COVID-19
13122_19	FLRT2	Leucine-rich repeat transmembrane protein FLRT2	O43155	FLRT2	NA	Inverse variance weighted	7	0.037	0.048	4.461E-01	0.973	pan	Severe COVID-19
4158_54	uPA	Urokinase-type plasminogen activator	P00749	PLAU	NA	Inverse variance weighted	11	0.053	0.070	4.467E-01	0.973	pan	Severe COVID-19
4499_21	PDGF-AA	Platelet-derived growth factor subunit A	P04085	PDGFA	NA	Inverse variance weighted	3	-0.134	0.177	4.473E-01	0.973	pan	Severe COVID-19
16317_20	Desmoglein-3	Desmoglein-3	P32926	DSG3	NA	Inverse variance weighted	2	0.199	0.262	4.474E-01	0.973	pan	Severe COVID-19
4455_89	MFGM	Lactadherin	Q08431	MFGE8	NA	Inverse variance weighted	4	-0.010	0.013	4.479E-01	0.973	pan	Severe COVID-19
4314_12	XTP3A	dCTP pyrophosphatase 1	Q9H773	DCTPP1	NA	Wald ratio	1	0.142	0.187	4.486E-01	0.973	pan	Severe COVID-19
11440_58	SOCS-3	Suppressor of cytokine signaling 3	O14543	SOCS3	NA	Inverse variance weighted	9	0.032	0.042	4.488E-01	0.973	pan	Severe COVID-19
15468_14	FHR1	Complement factor H-related protein 1	Q03591	CFHR1	NA	Inverse variance weighted	10	0.067	0.088	4.491E-01	0.973	pan	Severe COVID-19
13499_30	Coagulation Factor VIII	Coagulation Factor VIII	P00451	F8	NA	Inverse variance weighted	6	0.089	0.117	4.492E-01	0.973	pan	Severe COVID-19
15494_11	FGFP1	Fibroblast growth factor-binding protein 1	Q14512	FGFBP1	NA	Inverse variance weighted	2	0.141	0.187	4.495E-01	0.973	pan	Severe COVID-19
7875_86	PLEK	Pleckstrin	P08567	PLEK	NA	Inverse variance weighted	2	-0.094	0.124	4.502E-01	0.973	pan	Severe COVID-19
10442_1	TM190	Transmembrane protein 190	Q8WZ59	TMEM190	NA	Inverse variance weighted	8	-0.020	0.027	4.503E-01	0.973	pan	Severe COVID-19
19553_14	STX1a	Syntaxin-1A	Q16623	STX1A	NA	Inverse variance weighted	4	0.108	0.143	4.520E-01	0.973	pan	Severe COVID-19
19254_125	GMPR1	GMP reductase 1	P36959	GMPR	NA	Inverse variance weighted	15	-0.027	0.036	4.525E-01	0.973	pan	Severe COVID-19
6544_33	NELL1	Protein kinase C-binding protein NELL1	Q92832	NELL1	NA	Inverse variance weighted	21	-0.043	0.057	4.527E-01	0.973	pan	Severe COVID-19
8013_9	LMA2L	VIP36-like protein	Q9H0V9	LMAN2L	NA	Inverse variance weighted	2	-0.120	0.160	4.528E-01	0.973	pan	Severe COVID-19
5644_60	RNAS4	Ribonuclease 4	P34096	RNASE4	NA	Inverse variance weighted	5	0.054	0.072	4.528E-01	0.973	pan	Severe COVID-19
11319_106	MRE11	Double-strand break repair protein MRE11	P49959	MRE11	NA	Wald ratio	1	-0.219	0.292	4.528E-01	0.973	pan	Severe COVID-19
5532_53	bFGF-R	Fibroblast growth factor receptor 1	P11362	FGFR1	NA	Inverse variance weighted	5	0.090	0.120	4.530E-01	0.973	pan	Severe COVID-19
8376_25	LSHB	Lutropin subunit beta	P01229	LHB	NA	Inverse variance weighted	4	0.055	0.073	4.544E-01	0.973	pan	Severe COVID-19
12686_15	THTM	3-mercaptopyruvate sulfurtransferase	P25325	MPST	NA	Inverse variance weighted	2	-0.162	0.217	4.556E-01	0.973	pan	Severe COVID-19
16781_2	ENASE	Cytosolic endo-beta-N-acetylglucosaminidase	Q8NFI3	ENGASE	NA	Inverse variance weighted	11	0.022	0.030	4.557E-01	0.973	pan	Severe COVID-19
4534_10	BSSP4	Brain-specific serine protease 4	Q9GZN4	PRSS22	NA	Inverse variance weighted	4	-0.050	0.068	4.562E-01	0.973	pan	Severe COVID-19
9126_171	NT5D3	5'-nucleotidase domain-containing protein 3	Q86UY8	NT5DC3	NA	Inverse variance weighted	3	0.051	0.068	4.575E-01	0.973	pan	Severe COVID-19
5663_18	PF4V	Platelet factor 4 variant	P10720	PF4V1	NA	Inverse variance weighted	2	-0.200	0.269	4.578E-01	0.973	pan	Severe COVID-19
6440_31	MFAP5	Microfibrillar-associated protein 5	Q13361	MFAP5	NA	Inverse variance weighted	3	-0.235	0.317	4.586E-01	0.973	pan	Severe COVID-19
4546_27	EMR2	Adhesion G protein-coupled receptor E2	Q9UHX3	ADGRE2	NA	Inverse variance weighted	6	0.040	0.054	4.589E-01	0.973	pan	Severe COVID-19
6572_10	LRRT4:ECD	Leucine-rich repeat transmembrane neuronal protein 4:Isoform 2, Extracellular domain	Q86VH4	LRRTM4	NA	Wald ratio	1	-0.233	0.315	4.590E-01	0.973	pan	Severe COVID-19
5491_12	Testican-2	Testican-2	Q92563	SPOCK2	NA	Inverse variance weighted	13	-0.039	0.053	4.597E-01	0.973	pan	Severe COVID-19
18301_10	NAIF1	Nuclear apoptosis-inducing factor 1	Q69YI7	NAIF1	NA	Inverse variance weighted	3	-0.133	0.180	4.599E-01	0.973	pan	Severe COVID-19
9805_51	SEM4B	Semaphorin-4B	Q9NPR2	SEMA4B	NA	Wald ratio	1	-0.198	0.268	4.601E-01	0.973	pan	Severe COVID-19
15506_34	LRP12	Low-density lipoprotein receptor-related protein 12	Q9Y561	LRP12	NA	Wald ratio	1	0.220	0.299	4.603E-01	0.973	pan	Severe COVID-19
14318_1	VPS29	Vacuolar protein sorting-associated protein 29	Q9UBQ0	VPS29	NA	Inverse variance weighted	2	0.188	0.255	4.608E-01	0.973	pan	Severe COVID-19
14628_72	FBX3	F-box only protein 3	Q9UK99	FBXO3	NA	Wald ratio	1	-0.472	0.641	4.608E-01	0.973	pan	Severe COVID-19
15604_18	JNK2	Mitogen-activated protein kinase 9	P45984	MAPK9	NA	Inverse variance weighted	2	-0.105	0.143	4.625E-01	0.973	pan	Severe COVID-19
17787_1	Enoyl-CoA hydratase	Enoyl-CoA hydratase, mitochondrial	P30084	ECHS1	NA	Wald ratio	1	-0.207	0.282	4.626E-01	0.973	pan	Severe COVID-19
6518_85	ghrelin	Appetite-regulating hormone	Q9UBU3	GHRL	NA	Inverse variance weighted	3	-0.089	0.121	4.632E-01	0.973	pan	Severe COVID-19
18397_5	AK1C4	Aldo-keto reductase family 1 member C4	P17516	AKR1C4	NA	Inverse variance weighted	11	0.037	0.050	4.645E-01	0.973	pan	Severe COVID-19
3890_8	LDH-H 1	L-lactate dehydrogenase B chain	P07195	LDHB	NA	Wald ratio	1	0.312	0.427	4.651E-01	0.973	pan	Severe COVID-19
18877_15	CNN2	Calponin-2	Q99439	CNN2	NA	Inverse variance weighted	2	-0.121	0.166	4.653E-01	0.973	pan	Severe COVID-19

6576_1	ART4	Ecto-ADP-ribosyltransferase 4	Q93070	ART4	NA	Inverse variance weighted	10	0.020	0.028	4.657E-01	0.973	pan	Severe COVID-19
9266_1	sTREM-1	Triggering receptor expressed on myeloid cells 1	Q9NP99	TREM1	NA	Inverse variance weighted	7	0.080	0.110	4.657E-01	0.973	pan	Severe COVID-19
12494_99	GBRL2	Gamma-aminobutyric acid receptor-associated protein-like 2	P60520	GABARAPL2	NA	Inverse variance weighted	2	0.302	0.415	4.671E-01	0.973	pan	Severe COVID-19
4437_56	ENTP5	Ectonucleoside triphosphate diphosphohydrolase 5	O75356	ENTPD5	NA	Inverse variance weighted	9	0.024	0.033	4.672E-01	0.973	pan	Severe COVID-19
3803_10	CYTD	Cystatin-D	P28325	CST5	NA	Inverse variance weighted	17	0.038	0.053	4.681E-01	0.973	pan	Severe COVID-19
10603_1	HIS3	Histatin-3	P15516	HTN3	NA	Inverse variance weighted	12	0.050	0.069	4.683E-01	0.973	pan	Severe COVID-19
5456_59	CNDP1	Beta-Ala-His dipeptidase	Q96KN2	CNDP1	NA	Inverse variance weighted	12	-0.025	0.035	4.688E-01	0.973	pan	Severe COVID-19
8352_26	SIG12:Ig-like C2-type 2	Sialic acid-binding Ig-like lectin 12:Ig-like C2-type 2 domain, Isoform short	Q96PQ1	SIGLEC12	NA	Wald ratio	1	0.257	0.354	4.689E-01	0.973	pan	Severe COVID-19
15582_25	FCN1	Ficolin-1	O00602	FCN1	NA	Inverse variance weighted	4	0.080	0.111	4.692E-01	0.973	pan	Severe COVID-19
17224_12	MIME	Mimecan	P20774	OGN	NA	Inverse variance weighted	6	-0.099	0.137	4.692E-01	0.973	pan	Severe COVID-19
5112_73	OX2G	OX-2 membrane glycoprotein	P41217	CD200	NA	Wald ratio	1	-0.137	0.190	4.693E-01	0.973	pan	Severe COVID-19
10908_2	GLT13	Polypeptide N-acetylgalactosaminyltransferase 13	Q8IUC8	GALNT13	NA	Wald ratio	1	-0.245	0.340	4.697E-01	0.973	pan	Severe COVID-19
5861_78	3HAO	3-hydroxyanthranilate 3,4-dioxygenase	P46952	HAAO	NA	Inverse variance weighted	6	-0.096	0.133	4.701E-01	0.973	pan	Severe COVID-19
3605_77	MASP3:Light	Mannan-binding lectin serine protease 1:Mannan-binding lectin serine protease 1 light chain	P48740	MASP1	NA	Wald ratio	1	-0.205	0.285	4.712E-01	0.973	pan	Severe COVID-19
9275_2	Siglec-5	Sialic acid-binding Ig-like lectin 5	O15389	SIGLEC5	NA	Inverse variance weighted	4	-0.130	0.181	4.716E-01	0.973	pan	Severe COVID-19
7959_34	CADH7	Cadherin-7	Q9ULB5	CDH7	NA	Inverse variance weighted	6	-0.095	0.132	4.722E-01	0.973	pan	Severe COVID-19
3066_12	Galectin-3	Galectin-3	P17931	LGALS3	NA	Inverse variance weighted	6	-0.031	0.044	4.723E-01	0.973	pan	Severe COVID-19
9233_71	TFPI -2	Tissue factor pathway inhibitor 2	P48307	TFPI2	NA	Inverse variance weighted	2	-0.151	0.210	4.723E-01	0.973	pan	Severe COVID-19
17398_55	HO-1	Heme oxygenase 1	P09601	HMOX1	NA	Inverse variance weighted	10	-0.058	0.081	4.732E-01	0.973	pan	Severe COVID-19
9398_30	GALP	Galanin-like peptide	Q9UBC7	GALP	NA	Wald ratio	1	-0.171	0.239	4.746E-01	0.973	pan	Severe COVID-19
5605_77	MFNG	Beta-1,3-N-acetylglucosaminyltransferase manic fringe	O00587	MFNG	NA	Inverse variance weighted	2	-0.128	0.180	4.757E-01	0.973	pan	Severe COVID-19
12630_8	ARFP2	Arfaptin-2	P53365	ARFIP2	NA	Inverse variance weighted	5	0.092	0.129	4.758E-01	0.973	pan	Severe COVID-19
11288_26	5NTC	Cytosolic purine 5'-nucleotidase	P49902	NT5C2	NA	Inverse variance weighted	3	-0.109	0.153	4.761E-01	0.973	pan	Severe COVID-19
15325_14	NEUM	Neuromodulin	P17677	GAP43	NA	Wald ratio	1	-0.153	0.215	4.771E-01	0.973	pan	Severe COVID-19
10512_13	IL3RB:ECD	Cytokine receptor common subunit beta:Extracellular domain	P32927	CSF2RB	NA	Inverse variance weighted	6	-0.022	0.031	4.781E-01	0.973	pan	Severe COVID-19
4923_79	MIS	Muellerian-inhibiting factor	P03971	AMH	NA	Inverse variance weighted	5	0.088	0.124	4.783E-01	0.973	pan	Severe COVID-19
2831_29	Kallikrein 11	Kallikrein-11	Q9UBX7	KLK11	NA	Inverse variance weighted	7	0.059	0.083	4.795E-01	0.973	pan	Severe COVID-19
3820_68	MAPK2	MAP kinase-activated protein kinase 2	P49137	MAPKAPK2	NA	Wald ratio	1	-0.101	0.143	4.796E-01	0.973	pan	Severe COVID-19
8382_47	MAPK5	MAP kinase-activated protein kinase 5	Q8IW41	MAPKAPK5	NA	Inverse variance weighted	12	-0.049	0.069	4.799E-01	0.973	pan	Severe COVID-19
19272_9	PEF1	Peflin	Q9UBV8	PEF1	NA	Inverse variance weighted	10	0.031	0.044	4.803E-01	0.973	pan	Severe COVID-19
8288_27	b2-Glycoprotein I	Beta-2-glycoprotein 1	P02749	APOH	NA	Inverse variance weighted	6	0.030	0.043	4.809E-01	0.973	pan	Severe COVID-19
9595_11	B4GT2	Beta-1,4-galactosyltransferase 2	O60909	B4GALT2	NA	Inverse variance weighted	4	-0.055	0.078	4.816E-01	0.973	pan	Severe COVID-19
5012_67	Myokinase, human	Adenylate kinase isoenzyme 1	P00568	AK1	NA	Wald ratio	1	-0.249	0.355	4.822E-01	0.973	pan	Severe COVID-19
9832_33	HGD	Homogentisate 1,2-dioxygenase	Q93099	HGD	NA	Inverse variance weighted	2	-0.092	0.131	4.826E-01	0.973	pan	Severe COVID-19
7997_118	DOC2B	Double C2-like domain-containing protein beta	Q14184	DOC2B	NA	Wald ratio	1	-0.245	0.350	4.830E-01	0.973	pan	Severe COVID-19
11219_95	FGFP3	Fibroblast growth factor-binding protein 3	Q8TAT2	FGFBP3	NA	Inverse variance weighted	3	0.063	0.089	4.831E-01	0.973	pan	Severe COVID-19
18916_25	Inosine triphosphatase	Inosine triphosphate pyrophosphatase	Q9BY32	ITPA	NA	Inverse variance weighted	11	0.021	0.030	4.832E-01	0.973	pan	Severe COVID-19
17802_4	SIAS	Sialic acid synthase	Q9NR45	NANS	NA	Wald ratio	1	0.122	0.174	4.833E-01	0.973	pan	Severe COVID-19
8303_102	MYG1	UPF0160 protein MYG1, mitochondrial	Q9HB07	MYG1	NA	Wald ratio	1	0.205	0.293	4.835E-01	0.973	pan	Severe COVID-19
8299_66	LIRA4	Leukocyte immunoglobulin-like receptor subfamily A member 4	P59901	LILRA4	NA	Inverse variance weighted	7	0.091	0.130	4.840E-01	0.973	pan	Severe COVID-19
8956_96	SREC-II:ECD	Scavenger receptor class F member 2:Extracellular domain	Q96GP6	SCARF2	NA	Inverse variance weighted	2	0.092	0.132	4.852E-01	0.973	pan	Severe COVID-19
19496_1	NPL	N-acetylneuraminate lyase	Q9BXD5	NPL	NA	Inverse variance weighted	4	-0.059	0.085	4.853E-01	0.973	pan	Severe COVID-19
8839_4	Metaxin-2	Metaxin-2	O75431	MTX2	NA	Wald ratio	1	-0.209	0.300	4.865E-01	0.973	pan	Severe COVID-19
8908_14	KCE1L:CD	Potassium voltage-gated channel subfamily E regulatory beta subunit 5:Cytoplasmic domain	Q9UJ90	KCNE5	NA	Inverse variance weighted	3	0.193	0.278	4.870E-01	0.973	pan	Severe COVID-19
5487_7	SLAF7	SLAM family member 7	Q9NQ25	SLAMF7	NA	Inverse variance weighted	14	0.027	0.039	4.870E-01	0.973	pan	Severe COVID-19
4925_54	MMP-13	Collagenase 3	P45452	MMP13	NA	Inverse variance weighted	2	0.161	0.232	4.870E-01	0.973	pan	Severe COVID-19
5957_30	Somatostatin-28	Somatostatin-28	P61278	SST	NA	Wald ratio	1	0.209	0.300	4.874E-01	0.973	pan	Severe COVID-19
18206_18	ADH6	Alcohol dehydrogenase 6	P28332	ADH6	NA	Inverse variance weighted	2	-0.182	0.262	4.876E-01	0.973	pan	Severe COVID-19
3343_1	Aminoacylase-1	Aminoacylase-1	Q03154	ACY1	NA	Inverse variance weighted	3	0.109	0.157	4.877E-01	0.973	pan	Severe COVID-19
10621_26	PRR16	Protein Largen	Q569H4	PRR16	NA	Inverse variance weighted	4	0.117	0.168	4.878E-01	0.973	pan	Severe COVID-19
8244_16	FUT8	Alpha-(1,6)-fucosyltransferase	Q9BYC5	FUT8	NA	Inverse variance weighted	19	-0.037	0.053	4.885E-01	0.973	pan	Severe COVID-19
18839_24	Thyroglobulin	Thyroglobulin	P01266	TG	NA	Inverse variance weighted	6	-0.063	0.091	4.885E-01	0.973	pan	Severe COVID-19
2647_66	Rab GDP dissociation inhibitor beta	Rab GDP dissociation inhibitor beta	P50395	GDI2	NA	Wald ratio	1	-0.107	0.154	4.889E-01	0.973	pan	Severe COVID-19
17403_14	acyl-Coenzyme A dehydrogenase	Short/branched chain specific acyl-CoA dehydrogenase, mitochondrial	P45954	ACADSB	NA	Inverse variance weighted	4	0.085	0.124	4.899E-01	0.973	pan	Severe COVID-19
18413_24	ARL4D	ADP-ribosylation factor-like protein 4D	P49703	ARL4D	NA	Wald ratio	1	0.193	0.279	4.899E-01	0.973	pan	Severe COVID-19
15305_7	Secretagogin	Secretagogin	O76038	SCGN	NA	Inverse variance weighted	2	0.201	0.292	4.912E-01	0.973	pan	Severe COVID-19
3296_92	CNTN2	Contactin-2	Q02246	CNTN2	NA	Inverse variance weighted	9	-0.020	0.029	4.913E-01	0.973	pan	Severe COVID-19
18878_15	GREM1	Gremlin-1	O60565	GREM1	NA	Inverse variance weighted	10	-0.023	0.034	4.916E-01	0.973	pan	Severe COVID-19
4154_57	P-Selectin	P-selectin	P16109	SELP	NA	Inverse variance weighted	4	0.068	0.099	4.936E-01	0.973	pan	Severe COVID-19
5092_51	JAG1:ECD	Protein jagged-1:Extracellular domain	P78504	JAG1	NA	Wald ratio	1	-0.200	0.292	4.936E-01	0.973	pan	Severe COVID-19
3235_50	WFKN2	WAP, Kazal, immunoglobulin, Kunitz and NTR domain-containing protein 2	Q8TEU8	WFIKKN2	NA	Inverse variance weighted	8	0.027	0.040	4.939E-01	0.973	pan	Severe COVID-19
18832_65	SAA2	Serum amyloid A-2 protein	P0DJI9	SAA2	NA	Inverse variance weighted	3	-0.231	0.337	4.940E-01	0.973	pan	Severe COVID-19
5656_53	PP11	Poly(U)-specific endoribonuclease	P21128	ENDOU	NA	Wald ratio	1	-0.166	0.244	4.945E-01	0.973	pan	Severe COVID-19
4906_35	Coagulation Factor V	Coagulation Factor V	P12259	F5	NA	Inverse variance weighted	3	0.116	0.170	4.947E-01	0.973	pan	Severe COVID-19
11351_233	NHEJ1	Non-homologous end-joining factor 1	Q9H9Q4	NHEJ1	NA	Inverse variance weighted	3	-0.107	0.157	4.957E-01	0.973	pan	Severe COVID-19
15471_29	LIPR2	Pancreatic lipase-related protein 2	P54317	PNLIPRP2	NA	Inverse variance weighted	14	-0.015	0.022	4.958E-01	0.973	pan	Severe COVID-19
9574_11	BIN1	Myc box-dependent-interacting protein 1	O00499	BIN1	NA	Inverse variance weighted	2	-0.072	0.105	4.958E-01	0.973	pan	Severe COVID-19
4151_6	Plasminogen	Plasminogen	P00747	PLG	NA	Inverse variance weighted	5	-0.124	0.182	4.961E-01	0.973	pan	Severe COVID-19
12560_9	NT5C	5'(3')-deoxyribonucleotidase, cytosolic type	Q8TCD5	NT5C	NA	Inverse variance weighted	5	0.026	0.038	4.973E-01	0.973	pan	Severe COVID-19
17781_191	MLP3A	Microtubule-associated proteins 1A/1B light chain 3A	Q9H492	MAP1LC3A	NA	Inverse variance weighted	8	0.088	0.130	4.979E-01	0.973	pan	Severe COVID-19
10070_22	PLCG2	1-phosphatidylinositol 4,5-bisphosphate phosphodiesterase gamma-2	P16885	PLCG2	NA	Inverse variance weighted	2	0.168	0.249	4.988E-01	0.973	pan	Severe COVID-19
4842_62	Glypican 3	Glypican-3	P51654	GPC3	NA	Inverse variance weighted	4	-0.118	0.175	4.995E-01	0.973	pan	Severe COVID-19
14133_93	IL-1 sRII	Interleukin-1 receptor type 2	P27930	IL1R2	NA	Inverse variance weighted	12	0.039	0.057	4.996E-01	0.973	pan	Severe COVID-19
3805_16	Endocan	Endothelial cell-specific molecule 1	Q9NQ30	ESM1	NA	Inverse variance weighted	5	-0.067	0.100	4.997E-01	0.973	pan	Severe COVID-19
15584_9	FHR2	Complement factor H-related protein 2	P36980	CFHR2	NA	Inverse variance weighted	16	-0.028	0.041	4.997E-01	0.973	pan	Severe COVID-19
5593_11	PDIA5	Protein disulfide-isomerase A5	Q14554	PDIA5	NA	Inverse variance weighted	11	-0.034	0.051	4.999E-01	0.973	pan	Severe COVID-19
13518_5	ASAP2	Arf-GAP with SH3 domain, ANK repeat and PH domain-containing protein 2	O43150	ASAP2	NA	Wald ratio	1	-0.151	0.223	5.000E-01	0.973	pan	Severe COVID-19
17175_5	MP2K6	Dual specificity mitogen-activated protein kinase kinase 6	P52564	MAP2K6	NA	Inverse variance weighted	2	0.157	0.233	5.007E-01	0.973	pan	Severe COVID-19

16908_5	OMGP	Oligodendrocyte-myelin glycoprotein	P23515	OMG	NA	Inverse variance weighted	5	0.085	0.126	5.008E-01	0.973	pan	Severe COVID-19
8327_26	DPEP2	Dipeptidase 2	Q9H4A9	DPEP2	NA	Inverse variance weighted	6	0.049	0.073	5.018E-01	0.973	pan	Severe COVID-19
9370_69	GGH	Gamma-glutamyl hydrolase	Q92820	GGH	NA	Inverse variance weighted	13	-0.027	0.040	5.027E-01	0.973	pan	Severe COVID-19
3495_15	GCP-2	C-X-C motif chemokine 6	P80162	CXCL6	NA	Inverse variance weighted	13	-0.026	0.038	5.028E-01	0.973	pan	Severe COVID-19
4141_79	IP-10	C-X-C motif chemokine 10	P02778	CXCL10	NA	Inverse variance weighted	2	0.190	0.284	5.032E-01	0.973	pan	Severe COVID-19
4258_15	PA2G4	Proliferation-associated protein 2G4	Q9UQ80	PA2G4	NA	Wald ratio	1	0.173	0.259	5.045E-01	0.973	pan	Severe COVID-19
13093_6	SECTM1	Secreted and transmembrane protein 1	Q8WVN6	SECTM1	NA	Inverse variance weighted	2	-0.089	0.133	5.045E-01	0.973	pan	Severe COVID-19
3302_58	CYTF	Cystatin-F	O76096	CST7	NA	Inverse variance weighted	13	0.023	0.035	5.052E-01	0.973	pan	Severe COVID-19
4969_2	Carbonic anhydrase I	Carbonic anhydrase 1	P00915	CA1	NA	Inverse variance weighted	6	-0.054	0.081	5.059E-01	0.973	pan	Severe COVID-19
8033_1	DJC18	DnaJ homolog subfamily C member 18	Q9H819	DNAJC18	NA	Wald ratio	1	-0.181	0.272	5.062E-01	0.973	pan	Severe COVID-19
13477_65	LT4R1	Leukotriene B4 receptor 1	Q15722	LTB4R	NA	Inverse variance weighted	2	0.114	0.171	5.063E-01	0.973	pan	Severe COVID-19
12676_1	PACN1	Protein kinase C and casein kinase substrate in neurons protein 1	Q9BY11	PACSIN1	NA	Inverse variance weighted	2	-0.077	0.116	5.069E-01	0.973	pan	Severe COVID-19
5631_83	MOTI	Promotilin	P12872	MLN	NA	Inverse variance weighted	26	-0.025	0.038	5.071E-01	0.973	pan	Severe COVID-19
14066_49	MAGI2	Membrane-associated guanylate kinase, WW and PDZ domain-containing protein 2	Q86UL8	MAGI2	NA	Inverse variance weighted	10	-0.021	0.032	5.073E-01	0.973	pan	Severe COVID-19
12990_39	MAP3K3	Mitogen-activated protein kinase kinase kinase 3	Q99759	MAP3K3	NA	Wald ratio	1	0.090	0.136	5.078E-01	0.973	pan	Severe COVID-19
7916_10	S100A7	Protein S100-A7	P31151	S100A7	NA	Inverse variance weighted	16	-0.020	0.030	5.097E-01	0.973	pan	Severe COVID-19
4891_50	Glucagon	Glucagon	P01275	GCG	NA	Wald ratio	1	0.202	0.306	5.099E-01	0.973	pan	Severe COVID-19
7015_8	LIRB5	Leukocyte immunoglobulin-like receptor subfamily B member 5	O75023	LILRB5	NA	Inverse variance weighted	7	0.044	0.067	5.100E-01	0.973	pan	Severe COVID-19
16818_200	CDCP1	CUB domain-containing protein 1	Q9H5V8	CDCP1	NA	Inverse variance weighted	5	-0.113	0.171	5.103E-01	0.973	pan	Severe COVID-19
5087_5	IL-22BP	Interleukin-22 receptor subunit alpha-2	Q969J5	IL22RA2	NA	Inverse variance weighted	20	-0.033	0.051	5.103E-01	0.973	pan	Severe COVID-19
5105_2	Nogo Receptor	Reticulon-4 receptor	Q9BZR6	RTN4R	NA	Inverse variance weighted	5	-0.034	0.051	5.108E-01	0.973	pan	Severe COVID-19
13740_51	sFRP-3	Secreted frizzled-related protein 3	Q92765	FRZB	NA	Inverse variance weighted	10	-0.020	0.030	5.110E-01	0.973	pan	Severe COVID-19
19482_11	HDHD3	Haloacid dehalogenase-like hydrolase domain-containing protein 3	Q9BSH5	HDHD3	NA	Wald ratio	1	0.053	0.081	5.114E-01	0.973	pan	Severe COVID-19
8005_1	MXRA7	Matrix-remodeling-associated protein 7	P84157	MXRA7	NA	Inverse variance weighted	5	-0.030	0.046	5.117E-01	0.973	pan	Severe COVID-19
2999_6	LSAMP	Limbic system-associated membrane protein	Q13449	LSAMP	NA	Inverse variance weighted	5	0.057	0.087	5.129E-01	0.973	pan	Severe COVID-19
17773_26	SNAG	Gamma-soluble NSF attachment protein	Q99747	NAPG	NA	Wald ratio	1	-0.167	0.255	5.129E-01	0.973	pan	Severe COVID-19
15614_168	LIRA2	Leukocyte immunoglobulin-like receptor subfamily A member 2	Q8N149	LILRA2	NA	Inverse variance weighted	5	-0.027	0.042	5.130E-01	0.973	pan	Severe COVID-19
3391_10	PK3CG	Phosphatidylinositol 4,5-bisphosphate 3-kinase catalytic subunit gamma isoform	P48736	PIK3CG	NA	Wald ratio	1	-0.292	0.446	5.135E-01	0.973	pan	Severe COVID-19
16913_8	RNT2	Ribonuclease T2	O00584	RNASET2	NA	Inverse variance weighted	6	0.022	0.034	5.139E-01	0.973	pan	Severe COVID-19
7905_30	HPT	Haptoglobin isoform 2	P00738	HP	NA	Wald ratio	1	-0.156	0.239	5.140E-01	0.973	pan	Severe COVID-19
19193_18	PFD3	Prefoldin subunit 3	P61758	VBP1	NA	Inverse variance weighted	12	-0.077	0.118	5.144E-01	0.973	pan	Severe COVID-19
17786_5	GGPPS	Geranylgeranyl pyrophosphate synthase	O95749	GGPS1	NA	Wald ratio	1	-0.110	0.169	5.146E-01	0.973	pan	Severe COVID-19
3835_11	TLR2	Toll-like receptor 2	O60603	TLR2	NA	Wald ratio	1	-0.376	0.579	5.158E-01	0.973	pan	Severe COVID-19
12637_7	CACP	Carnitine O-acetyltransferase	P43155	CRAT	NA	Wald ratio	1	0.106	0.163	5.165E-01	0.973	pan	Severe COVID-19
9506_10	Apo L1	Apolipoprotein L1	O14791	APOL1	NA	Inverse variance weighted	15	-0.048	0.074	5.174E-01	0.973	pan	Severe COVID-19
7779_86	CHSTB	Carbohydrate sulfotransferase 11	Q9NPF2	CHST11	NA	Inverse variance weighted	5	-0.039	0.060	5.176E-01	0.973	pan	Severe COVID-19
14088_38	IGFBP-6	Insulin-like growth factor-binding protein 6	P24592	IGFBP6	NA	Inverse variance weighted	2	-0.111	0.172	5.177E-01	0.973	pan	Severe COVID-19
17782_23	THIK	3-ketoacyl-CoA thiolase, peroxisomal	P09110	ACAA1	NA	Inverse variance weighted	8	-0.032	0.050	5.183E-01	0.973	pan	Severe COVID-19
5749_53	COL	Colipase	P04118	CLPS	NA	Inverse variance weighted	8	-0.025	0.038	5.187E-01	0.973	pan	Severe COVID-19
9102_28	ZFY27:CD region 2	Protrudin:Cytoplasmic domain, region 2, isoform 5	Q5T4F4	ZFYVE27	NA	Wald ratio	1	0.163	0.253	5.194E-01	0.973	pan	Severe COVID-19
2654_19	TNF sR-I	Tumor necrosis factor receptor superfamily member 1A	P19438	TNFRSF1A	NA	Wald ratio	1	-0.084	0.130	5.194E-01	0.973	pan	Severe COVID-19
5611_56	D108B	Beta-defensin 108B	Q8NET1	DEFB108B	NA	Wald ratio	1	-0.245	0.380	5.197E-01	0.973	pan	Severe COVID-19
5542_22	NRP1	Neuropilin-1	O14786	NRP1	NA	Inverse variance weighted	8	-0.032	0.049	5.200E-01	0.973	pan	Severe COVID-19
6382_17	MANBA	Beta-mannosidase	O00462	MANBA	NA	Inverse variance weighted	8	-0.035	0.054	5.200E-01	0.973	pan	Severe COVID-19
18285_6	SVIP	Small VCP/p97-interacting protein	Q8NHG7	SVIP	NA	Inverse variance weighted	2	0.319	0.496	5.200E-01	0.973	pan	Severe COVID-19
7198_197	FA20B	Glycosaminoglycan xylosylkinase	O75063	FAM20B	NA	Inverse variance weighted	2	0.111	0.172	5.204E-01	0.973	pan	Severe COVID-19
18235_16	PGP	Glycerol-3-phosphate phosphatase	A6NDG6	PGP	NA	Inverse variance weighted	2	0.101	0.158	5.208E-01	0.973	pan	Severe COVID-19
2813_11	ART	Agouti-related protein	O00253	AGRP	NA	Inverse variance weighted	5	0.109	0.171	5.214E-01	0.973	pan	Severe COVID-19
19492_5	KAAG1	Kidney-associated antigen 1	Q9UBP8	KAAG1	NA	Inverse variance weighted	14	-0.040	0.063	5.216E-01	0.973	pan	Severe COVID-19
5231_79	PCSK9	Proprotein convertase subtilisin/kexin type 9	Q8NBP7	PCSK9	NA	Inverse variance weighted	5	0.042	0.066	5.220E-01	0.973	pan	Severe COVID-19
8358_30	Peroxiredoxin-3	Thioredoxin-dependent peroxide reductase, mitochondrial	P30048	PRDX3	NA	Wald ratio	1	0.202	0.316	5.220E-01	0.973	pan	Severe COVID-19
10666_7	GNPTG	N-acetylglucosamine-1-phosphotransferase subunit gamma	Q9UJJ9	GNPTG	NA	Inverse variance weighted	4	0.048	0.076	5.222E-01	0.973	pan	Severe COVID-19
7898_29	MDM1	Nuclear protein MDM1	Q8TC05	MDM1	NA	Inverse variance weighted	2	-0.110	0.172	5.227E-01	0.973	pan	Severe COVID-19
13056_18	S6A16	Orphan sodium- and chloride-dependent neurotransmitter transporter NTT5	Q9GZN6	SLC6A16	NA	Wald ratio	1	-0.221	0.345	5.227E-01	0.973	pan	Severe COVID-19
8233_2	ITIH5	Inter-alpha-trypsin inhibitor heavy chain H5	Q86UX2	ITIH5	NA	Inverse variance weighted	10	0.030	0.047	5.235E-01	0.973	pan	Severe COVID-19
2888_49	C7	Complement component C7	P10643	C7	NA	Inverse variance weighted	14	-0.019	0.030	5.236E-01	0.973	pan	Severe COVID-19
12731_12	PKHA7	Pleckstrin homology domain-containing family A member 7	Q6IQ23	PLEKHA7	NA	Inverse variance weighted	8	-0.024	0.038	5.236E-01	0.973	pan	Severe COVID-19
5636_10	MFAP4	Microfibril-associated glycoprotein 4	P55083	MFAP4	NA	Inverse variance weighted	11	0.039	0.062	5.254E-01	0.973	pan	Severe COVID-19
2991_9	IL-1 sRI	Interleukin-1 receptor type 1	P14778	IL1R1	NA	Inverse variance weighted	8	-0.072	0.115	5.268E-01	0.973	pan	Severe COVID-19
6252_62	Secretoglobin family 3A member 1	Secretoglobin family 3A member 1	Q96QR1	SCGB3A1	NA	Inverse variance weighted	28	0.048	0.075	5.272E-01	0.973	pan	Severe COVID-19
17396_23	ADH1A	Alcohol dehydrogenase 1A	P07327	ADH1A	NA	Inverse variance weighted	3	-0.078	0.123	5.273E-01	0.973	pan	Severe COVID-19
13697_51	GPDA	Glycerol-3-phosphate dehydrogenase [NAD(+)], cytoplasmic	P21695	GPD1	NA	Wald ratio	1	0.206	0.326	5.275E-01	0.973	pan	Severe COVID-19
8983_7	GOLM1	Golgi membrane protein 1	Q8NBJ4	GOLM1	NA	Inverse variance weighted	5	0.102	0.162	5.276E-01	0.973	pan	Severe COVID-19
17370_186	ABHDA	Mycophenolic acid acyl-glucuronide esterase, mitochondrial	Q9NUJ1	ABHD10	NA	Wald ratio	1	-0.183	0.290	5.278E-01	0.973	pan	Severe COVID-19
17738_7	CRNN	Cornulin	Q9UBG3	CRNN	NA	Wald ratio	1	0.105	0.167	5.281E-01	0.973	pan	Severe COVID-19
3323_37	LRP8	Low-density lipoprotein receptor-related protein 8	Q14114	LRP8	NA	Inverse variance weighted	4	0.055	0.087	5.295E-01	0.973	pan	Severe COVID-19
17758_79	DCXR	L-xylulose reductase	Q7Z4W1	DCXR	NA	Inverse variance weighted	2	0.098	0.156	5.299E-01	0.973	pan	Severe COVID-19
6556_5	ENPP5	Ectonucleotide pyrophosphatase/phosphodiesterase family member 5	Q9UJA9	ENPP5	NA	Inverse variance weighted	15	-0.012	0.020	5.299E-01	0.973	pan	Severe COVID-19
11161_5	SPG20	Spartin	Q8N0X7	SPART	NA	Wald ratio	1	0.200	0.320	5.308E-01	0.973	pan	Severe COVID-19
18841_1	SPB13	Serpin B13	Q9UIV8	SERPINB13	NA	Inverse variance weighted	4	0.064	0.102	5.308E-01	0.973	pan	Severe COVID-19
15603_20	Integrin alpha-2	Integrin alpha-2	P17301	ITGA2	NA	Inverse variance weighted	2	0.117	0.187	5.317E-01	0.973	pan	Severe COVID-19
19361_78	MATN3	Matrilin-3	O15232	MATN3	NA	Inverse variance weighted	14	0.016	0.026	5.324E-01	0.973	pan	Severe COVID-19
17856_23	NT5M	5'(3')-deoxyribonucleotidase, mitochondrial	Q9NPB1	NT5M	NA	Inverse variance weighted	2	0.089	0.143	5.324E-01	0.973	pan	Severe COVID-19
16296_43	LGR5	Leucine-rich repeat-containing G-protein coupled receptor 5	O75473	LGR5	NA	Wald ratio	1	-0.167	0.267	5.329E-01	0.973	pan	Severe COVID-19
6234_74	VITRN	Vitrin	Q6UXI7	VIT	NA	Inverse variance weighted	9	0.030	0.048	5.334E-01	0.973	pan	Severe COVID-19
7935_26	LARGE	Glycosyltransferase-like protein LARGE1	O95461	LARGE1	NA	Inverse variance weighted	7	-0.069	0.112	5.352E-01	0.973	pan	Severe COVID-19
14618_26	ECOP	Vesicular, overexpressed in cancer, prosurvival protein 1	Q96AW1	VOPP1	NA	Inverse variance weighted	10	-0.043	0.069	5.354E-01	0.973	pan	Severe COVID-19
3045_72	PTN	Pleiotrophin	P21246	PTN	NA	Inverse variance weighted	4	-0.044	0.071	5.355E-01	0.973	pan	Severe COVID-19

5301_7	Eotaxin	Eotaxin	P51671	CCL11	NA	Inverse variance weighted	9	0.052	0.084	5.356E-01	0.973	pan	Severe COVID-19
10892_8	OSMR	Oncostatin-M-specific receptor subunit beta	Q99650	OSMR	NA	Inverse variance weighted	2	0.099	0.161	5.361E-01	0.973	pan	Severe COVID-19
6461_54	Apo C-III	Apolipoprotein C-III	P02656	APOC3	NA	Inverse variance weighted	6	-0.134	0.216	5.362E-01	0.973	pan	Severe COVID-19
3339_33	TSP2	Thrombospondin-2	P35442	THBS2	NA	Inverse variance weighted	8	-0.028	0.046	5.364E-01	0.973	pan	Severe COVID-19
13961_18	KIF3A	Kinesin-like protein KIF3A	Q9Y496	KIF3A	NA	Wald ratio	1	-0.180	0.291	5.366E-01	0.973	pan	Severe COVID-19
14090_23	DEFI6	Differentially expressed in FDCP 6 homolog	Q9H4E7	DEF6	NA	Inverse variance weighted	2	-0.098	0.159	5.368E-01	0.973	pan	Severe COVID-19
14067_6	PKP2	Plakophilin-2	Q99959	PKP2	NA	Inverse variance weighted	2	0.095	0.154	5.369E-01	0.973	pan	Severe COVID-19
19110_6	MEMO1	Protein MEMO1	Q9Y316	MEMO1	NA	Inverse variance weighted	2	0.086	0.140	5.372E-01	0.973	pan	Severe COVID-19
17756_69	DCTD	Deoxycytidylate deaminase	P32321	DCTD	NA	Inverse variance weighted	2	0.169	0.274	5.382E-01	0.973	pan	Severe COVID-19
3366_51	ECM1	Extracellular matrix protein 1	Q16610	ECM1	NA	Inverse variance weighted	14	-0.021	0.035	5.384E-01	0.973	pan	Severe COVID-19
11646_4	Carbohydrate sulfotransferase 9	Carbohydrate sulfotransferase 9	Q7L1S5	CHST9	NA	Inverse variance weighted	3	-0.067	0.109	5.388E-01	0.973	pan	Severe COVID-19
8099_42	SPON2	Spondin-2	Q9BUD6	SPON2	NA	Inverse variance weighted	4	-0.040	0.066	5.390E-01	0.973	pan	Severe COVID-19
2979_8	ENA-78	C-X-C motif chemokine 5	P42830	CXCL5	NA	Wald ratio	1	-0.244	0.399	5.402E-01	0.973	pan	Severe COVID-19
15667_39	BMP-4	Bone morphogenetic protein 4	P12644	BMP4	NA	Inverse variance weighted	3	-0.086	0.141	5.405E-01	0.973	pan	Severe COVID-19
3622_33	LGMN	Legumain	Q99538	LGMN	NA	Inverse variance weighted	4	0.071	0.117	5.407E-01	0.973	pan	Severe COVID-19
5509_7	EGF:ECD	Epidermal growth factor:Extracellular domain	P01133	EGF	NA	Inverse variance weighted	4	0.120	0.197	5.412E-01	0.973	pan	Severe COVID-19
8346_9	DPP2	Dipeptidyl peptidase 2	Q9UHL4	DPP7	NA	Inverse variance weighted	6	-0.041	0.066	5.419E-01	0.973	pan	Severe COVID-19
14079_14	IL-18 Ra	Interleukin-18 receptor 1	Q13478	IL18R1	NA	Inverse variance weighted	10	0.028	0.046	5.428E-01	0.973	pan	Severe COVID-19
8992_1	TMEM2	Transmembrane protein 2	Q9UHN6	CEMIP2	NA	Inverse variance weighted	4	0.052	0.086	5.429E-01	0.973	pan	Severe COVID-19
8970_9	RIPK2	Receptor-interacting serine/threonine-protein kinase 2	O43353	RIPK2	NA	Wald ratio	1	-0.157	0.259	5.439E-01	0.973	pan	Severe COVID-19
8989_40	SCUB1	Signal peptide, CUB and EGF-like domain-containing protein 1	Q8IWY4	SCUBE1	NA	Inverse variance weighted	6	-0.049	0.080	5.439E-01	0.973	pan	Severe COVID-19
8374_5	NGF R	Tumor necrosis factor receptor superfamily member 16	P08138	NGFR	NA	Inverse variance weighted	6	0.061	0.100	5.442E-01	0.973	pan	Severe COVID-19
5223_59	GCKR	Glucokinase regulatory protein	Q14397	GCKR	NA	Wald ratio	1	0.144	0.237	5.449E-01	0.973	pan	Severe COVID-19
10818_36	ASM	Sphingomyelin phosphodiesterase	P17405	SMPD1	NA	Inverse variance weighted	11	0.024	0.040	5.451E-01	0.973	pan	Severe COVID-19
4992_49	GRN	Granulins	P28799	GRN	NA	Inverse variance weighted	9	0.060	0.099	5.457E-01	0.973	pan	Severe COVID-19
17341_89	THIC	Acetyl-CoA acetyltransferase, cytosolic	Q9BWD1	ACAT2	NA	Inverse variance weighted	3	0.032	0.052	5.457E-01	0.973	pan	Severe COVID-19
3025_50	bFGF	Fibroblast growth factor 2	P09038	FGF2	NA	Inverse variance weighted	7	-0.024	0.039	5.458E-01	0.973	pan	Severe COVID-19
5634_39	OFUT1	GDP-fucose protein O-fucosyltransferase 1	Q9H488	POFUT1	NA	Inverse variance weighted	6	0.038	0.062	5.459E-01	0.973	pan	Severe COVID-19
11196_31	Collagen alpha-3(VI):BPTI/Kunitz inhibitor	Collagen alpha-3(VI) chain:Bovine pancreatic trypsin inhibitor/Kunitz inhibitor domain, isoform 1	P12111	COL6A3	NA	Wald ratio	1	-0.191	0.317	5.463E-01	0.973	pan	Severe COVID-19
18934_50	Tissue transglutaminase	Protein-glutamine gamma-glutamyltransferase 2	P21980	TGM2	NA	Wald ratio	1	0.048	0.080	5.473E-01	0.973	pan	Severe COVID-19
10710_23	ZPBP2	Zona pellucida-binding protein 2	Q6X784	ZPBP2	NA	Wald ratio	1	0.190	0.316	5.474E-01	0.973	pan	Severe COVID-19
10959_125	BET1L	BET1-like protein	Q9NYM9	BET1L	NA	Wald ratio	1	0.143	0.239	5.478E-01	0.973	pan	Severe COVID-19
4908_6	Endoglin	Endoglin	P17813	ENG	NA	Inverse variance weighted	7	0.030	0.051	5.479E-01	0.973	pan	Severe COVID-19
12800_5	TPPP2	Tubulin polymerization-promoting protein family member 2	P59282	TPPP2	NA	Inverse variance weighted	4	0.151	0.252	5.481E-01	0.973	pan	Severe COVID-19
19639_53	IAPP	Islet amyloid polypeptide	P10997	IAPP	NA	Inverse variance weighted	9	0.049	0.082	5.485E-01	0.973	pan	Severe COVID-19
7769_29	3BP2	SH3 domain-binding protein 2	P78314	SH3BP2	NA	Inverse variance weighted	2	0.072	0.120	5.487E-01	0.973	pan	Severe COVID-19
12549_33	PTGD2	Hematopoietic prostaglandin D synthase	O60760	HPGDS	NA	Inverse variance weighted	11	-0.023	0.038	5.488E-01	0.973	pan	Severe COVID-19
13101_60	SOST	Sclerostin	Q9BQB4	SOST	NA	Inverse variance weighted	3	0.082	0.137	5.496E-01	0.973	pan	Severe COVID-19
3581_53	a2-HS-Glycoprotein	Alpha-2-HS-glycoprotein	P02765	AHSG	NA	Inverse variance weighted	4	0.093	0.157	5.512E-01	0.973	pan	Severe COVID-19
6364_7	TPSNR	Tapasin-related protein	Q9BX59	TAPBPL	NA	Inverse variance weighted	14	-0.019	0.032	5.517E-01	0.973	pan	Severe COVID-19
2855_49	ERK-1	Mitogen-activated protein kinase 3	P27361	MAPK3	NA	Inverse variance weighted	2	-0.053	0.089	5.519E-01	0.973	pan	Severe COVID-19
9844_138	ACTN2	Alpha-actinin-2	P35609	ACTN2	NA	Inverse variance weighted	5	-0.095	0.159	5.520E-01	0.973	pan	Severe COVID-19
8874_53	CLN5:LD	Ceroid-lipofuscinosis neuronal protein 5:Lumenal domain	O75503	CLN5	NA	Inverse variance weighted	2	-0.083	0.140	5.521E-01	0.973	pan	Severe COVID-19
3728_52	Secretin	Secretin	P09683	SCT	NA	Wald ratio	1	-0.140	0.236	5.523E-01	0.973	pan	Severe COVID-19
15316_262	TXN4B	Thioredoxin-like protein 4B	Q9NX01	TXNL4B	NA	Inverse variance weighted	3	0.105	0.178	5.527E-01	0.973	pan	Severe COVID-19
3059_50	BAFF	Tumor necrosis factor ligand superfamily member 13B	Q9Y275	TNFSF13B	NA	Wald ratio	1	0.159	0.269	5.532E-01	0.973	pan	Severe COVID-19
11649_3	SMAP1	Stromal membrane-associated protein 1	Q8IYB5	SMAP1	NA	Inverse variance weighted	6	-0.042	0.071	5.532E-01	0.973	pan	Severe COVID-19
7173_141	T132C:ECD	Transmembrane protein 132C:Extracellular domain	Q8N3T6	TMEM132C	NA	Inverse variance weighted	10	0.025	0.042	5.540E-01	0.973	pan	Severe COVID-19
5763_67	HBD-4	Beta-defensin 104	Q8WTQ1	DEFB104A	NA	Wald ratio	1	-0.206	0.348	5.547E-01	0.973	pan	Severe COVID-19
15613_16	LIPP	Pancreatic triacylglycerol lipase	P16233	PNLIP	NA	Inverse variance weighted	5	0.130	0.221	5.548E-01	0.973	pan	Severe COVID-19
2743_5	Sonic Hedgehog	Sonic hedgehog protein	Q15465	SHH	NA	Inverse variance weighted	2	-0.099	0.168	5.551E-01	0.973	pan	Severe COVID-19
14121_24	TRAIL R4	Tumor necrosis factor receptor superfamily member 10D	Q9UBN6	TNFRSF10D	NA	Wald ratio	1	0.226	0.383	5.551E-01	0.973	pan	Severe COVID-19
18398_1	AK1D1	3-oxo-5-beta-steroid 4-dehydrogenase	P51857	AKR1D1	NA	Wald ratio	1	0.182	0.309	5.557E-01	0.973	pan	Severe COVID-19
16914_104	sCD14	Monocyte differentiation antigen CD14, soluble	P08571	CD14	NA	Inverse variance weighted	5	-0.086	0.146	5.562E-01	0.973	pan	Severe COVID-19
9391_60	PCSK1	ProSAAS	Q9UHG2	PCSK1N	NA	Inverse variance weighted	14	0.024	0.041	5.564E-01	0.973	pan	Severe COVID-19
12524_18	SAT2	Diamine acetyltransferase 2	Q96F10	SAT2	NA	Inverse variance weighted	3	-0.066	0.113	5.568E-01	0.973	pan	Severe COVID-19
19590_46	SP-D	Pulmonary surfactant-associated protein D	P35247	SFTPD	NA	Inverse variance weighted	20	0.027	0.047	5.573E-01	0.973	pan	Severe COVID-19
18881_7	CD97	CD97 antigen	P48960	ADGRE5	NA	Inverse variance weighted	7	-0.066	0.113	5.579E-01	0.973	pan	Severe COVID-19
5742_14	PPA6	Lysophosphatidic acid phosphatase type 6	Q9NPH0	ACP6	NA	Inverse variance weighted	23	-0.015	0.025	5.586E-01	0.973	pan	Severe COVID-19
11142_11	ANGL1:C-term	Angiopoietin-related protein 1:C-Term, Fibrinogen domian	O95841	ANGPTL1	NA	Inverse variance weighted	7	0.029	0.049	5.593E-01	0.973	pan	Severe COVID-19
17391_10	SPF45	Splicing factor 45	Q96I25	RBM17	NA	Inverse variance weighted	2	0.041	0.070	5.600E-01	0.973	pan	Severe COVID-19
2475_1	SCF sR	Mast/stem cell growth factor receptor Kit	P10721	KIT	NA	Inverse variance weighted	9	0.050	0.086	5.606E-01	0.973	pan	Severe COVID-19
3052_8	Fas ligand, soluble	Tumor necrosis factor ligand superfamily member 6, soluble form	P48023	FASLG	NA	Inverse variance weighted	4	-0.105	0.180	5.606E-01	0.973	pan	Severe COVID-19
9005_16	PLXA1	Plexin-A1	Q9UIW2	PLXNA1	NA	Inverse variance weighted	4	-0.055	0.094	5.609E-01	0.973	pan	Severe COVID-19
12954_71	PPARa	Peroxisome proliferator-activated receptor alpha	Q07869	PPARA	NA	Wald ratio	1	0.132	0.227	5.611E-01	0.973	pan	Severe COVID-19
18819_21	PPIC	Peptidyl-prolyl cis-trans isomerase C	P45877	PPIC	NA	Inverse variance weighted	10	0.022	0.039	5.612E-01	0.973	pan	Severe COVID-19
3291_30	CD23	Low affinity immunoglobulin epsilon Fc receptor	P06734	FCER2	NA	Inverse variance weighted	9	-0.034	0.058	5.614E-01	0.973	pan	Severe COVID-19
8916_32	STIM1:CD	Stromal interaction molecule 1:Cytoplasmic domain	Q13586	STIM1	NA	Wald ratio	1	0.094	0.163	5.631E-01	0.974	pan	Severe COVID-19
19115_13	CMBL	Carboxymethylenebutenolidase homolog	Q96DG6	CMBL	NA	Wald ratio	1	0.056	0.098	5.633E-01	0.974	pan	Severe COVID-19
9177_6	FAM3B	Protein FAM3B	P58499	FAM3B	NA	Inverse variance weighted	7	-0.034	0.059	5.637E-01	0.974	pan	Severe COVID-19
15587_20	FOLR2	Folate receptor beta	P14207	FOLR2	NA	Inverse variance weighted	3	0.104	0.180	5.639E-01	0.974	pan	Severe COVID-19
15298_199	NETO1	Neuropilin and tolloid-like protein 1	Q8TDF5	NETO1	NA	Inverse variance weighted	2	-0.175	0.305	5.659E-01	0.974	pan	Severe COVID-19
11103_24	HSP 27	Heat shock protein beta-1	P04792	HSPB1	NA	Inverse variance weighted	8	-0.024	0.041	5.662E-01	0.974	pan	Severe COVID-19
2681_23	HGF	Hepatocyte growth factor	P14210	HGF	NA	Wald ratio	1	-0.087	0.152	5.669E-01	0.974	pan	Severe COVID-19
4961_17	annexin II	Annexin A2	P07355	ANXA2	NA	Inverse variance weighted	3	0.104	0.182	5.674E-01	0.974	pan	Severe COVID-19
9215_117	UBP25	Ubiquitin carboxyl-terminal hydrolase 25	Q9UHP3	USP25	NA	Wald ratio	1	-0.261	0.458	5.679E-01	0.974	pan	Severe COVID-19
13378_80	YD286	Glutaredoxin-like protein C5orf63	A6NC05	C5orf63	NA	Wald ratio	1	-0.163	0.285	5.686E-01	0.974	pan	Severe COVID-19
13566_2	SSU72	RNA polymerase II subunit A C-terminal domain phosphatase SSU72	Q9NP77	SSU72	NA	Inverse variance weighted	6	0.035	0.062	5.689E-01	0.974	pan	Severe COVID-19

15391_114	GAS-6	Growth arrest-specific protein 6	Q14393	GAS6	NA	Inverse variance weighted	5	0.034	0.060	5.691E-01	0.974	pan	Severe COVID-19
17327_3	CNPY3	Protein canopy homolog 3	Q9BT09	CNPY3	NA	Inverse variance weighted	2	0.081	0.143	5.707E-01	0.974	pan	Severe COVID-19
18896_23	H6ST3	Heparan-sulfate 6-O-sulfotransferase 3	Q8IZP7	HS6ST3	NA	Inverse variance weighted	2	0.081	0.144	5.733E-01	0.974	pan	Severe COVID-19
11311_79	RAG-1	V(D)J recombination-activating protein 1	P15918	RAG1	NA	Wald ratio	1	-0.157	0.278	5.734E-01	0.974	pan	Severe COVID-19
17148_7	BLVRB	Flavin reductase (NADPH)	P30043	BLVRB	NA	Inverse variance weighted	2	-0.084	0.149	5.737E-01	0.974	pan	Severe COVID-19
6612_90	CP079	BRICHOS domain-containing protein 5	Q6PL45	BRICD5	NA	Inverse variance weighted	2	0.135	0.241	5.737E-01	0.974	pan	Severe COVID-19
16882_27	PHP14	14 kDa phosphohistidine phosphatase	Q9NRX4	PHPT1	NA	Inverse variance weighted	2	0.132	0.235	5.740E-01	0.974	pan	Severe COVID-19
2973_15	CD36 ANTIGEN	Platelet glycoprotein 4	P16671	CD36	NA	Inverse variance weighted	8	0.037	0.065	5.743E-01	0.974	pan	Severe COVID-19
9916_146	LRC4B:ECD	Leucine-rich repeat-containing protein 4B:Extracellular domain	Q9NT99	LRRC4B	NA	Inverse variance weighted	2	0.125	0.222	5.743E-01	0.974	pan	Severe COVID-19
2480_58	TIMP-3	Metalloproteinase inhibitor 3	P35625	TIMP3	NA	Inverse variance weighted	10	0.017	0.031	5.749E-01	0.974	pan	Severe COVID-19
18289_16	MIP-5	C-C motif chemokine 15	Q16663	CCL15	NA	Inverse variance weighted	9	-0.048	0.085	5.754E-01	0.974	pan	Severe COVID-19
9077_10	MA1A2	Mannosyl-oligosaccharide 1,2-alpha-mannosidase IB	O60476	MAN1A2	NA	Inverse variance weighted	5	-0.048	0.086	5.756E-01	0.974	pan	Severe COVID-19
3396_54	Renin	Renin	P00797	REN	NA	Inverse variance weighted	7	-0.083	0.149	5.759E-01	0.974	pan	Severe COVID-19
2968_61	TNFSF15	Tumor necrosis factor ligand superfamily member 15	O95150	TNFSF15	NA	Wald ratio	1	-0.155	0.277	5.763E-01	0.974	pan	Severe COVID-19
12401_3	STALP	AMSH-like protease	Q96FJ0	STAMBPL1	NA	Wald ratio	1	0.089	0.159	5.764E-01	0.974	pan	Severe COVID-19
18891_98	GBP2	Guanylate-binding protein 2	P32456	GBP2	NA	Wald ratio	1	-0.110	0.198	5.769E-01	0.974	pan	Severe COVID-19
4989_7	Fibrinogen g-chain dimer	Fibrinogen gamma chain	P02679	FGG	NA	Wald ratio	1	0.172	0.308	5.771E-01	0.974	pan	Severe COVID-19
9849_13	APEX1	DNA-(apurinic or apyrimidinic site) lyase	P27695	APEX1	NA	Wald ratio	1	-0.187	0.336	5.771E-01	0.974	pan	Severe COVID-19
15585_304	fibulin 5	Fibulin-5	Q9UBX5	FBLN5	NA	Wald ratio	1	-0.102	0.183	5.772E-01	0.974	pan	Severe COVID-19
11265_8	Retinal dehydrogenase 1	Retinal dehydrogenase 1	P00352	ALDH1A1	NA	Wald ratio	1	0.115	0.208	5.802E-01	0.974	pan	Severe COVID-19
9769_48	DNER:ECD	Delta and Notch-like epidermal growth factor-related receptor:Extracellular domain	Q8NFT8	DNER	NA	Inverse variance weighted	5	-0.039	0.071	5.803E-01	0.974	pan	Severe COVID-19
18921_30	PHEX	Phosphate-regulating neutral endopeptidase	P78562	PHEX	NA	Wald ratio	1	-0.130	0.234	5.804E-01	0.974	pan	Severe COVID-19
17682_1	CD46	Membrane cofactor protein	P15529	CD46	NA	Inverse variance weighted	3	0.088	0.159	5.808E-01	0.974	pan	Severe COVID-19
2811_27	Angiopoietin-1	Angiopoietin-1	Q15389	ANGPT1	NA	Inverse variance weighted	2	0.099	0.179	5.809E-01	0.974	pan	Severe COVID-19
4479_14	C1-Esterase Inhibitor	Plasma protease C1 inhibitor	P05155	SERPING1	NA	Inverse variance weighted	13	-0.027	0.049	5.817E-01	0.974	pan	Severe COVID-19
18185_118	ALDOB	Fructose-bisphosphate aldolase B	P05062	ALDOB	NA	Inverse variance weighted	3	0.043	0.078	5.818E-01	0.974	pan	Severe COVID-19
15573_110	CSPG3	Neurocan core protein	O14594	NCAN	NA	Inverse variance weighted	11	-0.041	0.074	5.824E-01	0.974	pan	Severe COVID-19
3773_15	sTie-2	Angiopoietin-1 receptor, soluble	Q02763	TEK	NA	Inverse variance weighted	5	-0.053	0.097	5.837E-01	0.974	pan	Severe COVID-19
11390_24	Carbonic Anhydrase VIII	Carbonic anhydrase-related protein	P35219	CA8	NA	Inverse variance weighted	7	-0.019	0.036	5.854E-01	0.974	pan	Severe COVID-19
8040_9	LEMD1	LEM domain-containing protein 1	Q68G75	LEMD1	NA	Wald ratio	1	-0.101	0.186	5.856E-01	0.974	pan	Severe COVID-19
18342_2	SERC	Phosphoserine aminotransferase	Q9Y617	PSAT1	NA	Inverse variance weighted	2	-0.153	0.280	5.864E-01	0.974	pan	Severe COVID-19
19296_51	MLRA	Myosin regulatory light chain 2, atrial isoform	Q01449	MYL7	NA	Inverse variance weighted	6	-0.066	0.122	5.878E-01	0.974	pan	Severe COVID-19
7194_36	NPTN	Neuroplastin	Q9Y639	NPTN	NA	Inverse variance weighted	2	0.117	0.216	5.886E-01	0.974	pan	Severe COVID-19
4156_74	TGF-b2	Transforming growth factor beta-2	P61812	TGFB2	NA	Wald ratio	1	-0.170	0.314	5.890E-01	0.974	pan	Severe COVID-19
3299_29	Contactin-5	Contactin-5	O94779	CNTN5	NA	Inverse variance weighted	4	-0.040	0.074	5.890E-01	0.974	pan	Severe COVID-19
17151_84	IRF-3	Interferon regulatory factor 3	Q14653	IRF3	NA	Wald ratio	1	-0.108	0.201	5.907E-01	0.974	pan	Severe COVID-19
8894_80	hnRNP A/B	Heterogeneous nuclear ribonucleoprotein A/B	Q99729	HNRNPAB	NA	Inverse variance weighted	2	0.142	0.265	5.911E-01	0.974	pan	Severe COVID-19
7956_11	MOS1	MICOS complex subunit MIC10	Q5TGZ0	MICOS10	NA	Inverse variance weighted	12	0.006	0.011	5.911E-01	0.974	pan	Severe COVID-19
13465_5	CCP1	Calcipressin-1	P53805	RCAN1	NA	Wald ratio	1	0.137	0.257	5.926E-01	0.974	pan	Severe COVID-19
16606_85	Aldose reductase	Aldose reductase	P15121	AKR1B1	NA	Inverse variance weighted	2	-0.066	0.124	5.941E-01	0.974	pan	Severe COVID-19
11709_29	CPT1B	Carnitine O-palmitoyltransferase 1, muscle isoform	Q92523	CPT1B	NA	Wald ratio	1	0.131	0.246	5.946E-01	0.974	pan	Severe COVID-19
8229_1	GXLT1	Glucoside xylosyltransferase 1	Q4G148	GXYLT1	NA	Inverse variance weighted	11	-0.027	0.050	5.948E-01	0.974	pan	Severe COVID-19
8394_56	RNase 2	Non-secretory ribonuclease	P10153	RNASE2	NA	Wald ratio	1	-0.136	0.257	5.969E-01	0.974	pan	Severe COVID-19
15509_2	NAG	Alpha-N-acetylglucosaminidase	P54802	NAGLU	NA	Inverse variance weighted	28	0.013	0.025	5.969E-01	0.974	pan	Severe COVID-19
19223_6	RAB1A	Ras-related protein Rab-1A	P62820	RAB1A	NA	Inverse variance weighted	2	-0.121	0.229	5.970E-01	0.974	pan	Severe COVID-19
17820_170	MLF1	Myeloid leukemia factor 1	P58340	MLF1	NA	Inverse variance weighted	4	-0.077	0.146	5.974E-01	0.974	pan	Severe COVID-19
5089_11	IL-7 Ra	Interleukin-7 receptor subunit alpha	P16871	IL7R	NA	Wald ratio	1	0.095	0.179	5.977E-01	0.974	pan	Severe COVID-19
3593_72	Caspase-3	Caspase-3	P42574	CASP3	NA	Wald ratio	1	0.082	0.157	5.997E-01	0.974	pan	Severe COVID-19
3132_1	VEGF-C	Vascular endothelial growth factor C	P49767	VEGFC	NA	Wald ratio	1	-0.098	0.186	5.999E-01	0.974	pan	Severe COVID-19
11615_16	DAPP1	Dual adapter for phosphotyrosine and 3-phosphotyrosine and 3-phosphoinositide	Q9UN19	DAPP1	NA	Inverse variance weighted	2	0.103	0.196	6.000E-01	0.974	pan	Severe COVID-19
10574_10	b2-Microglobulin	Beta-2-microglobulin	P61769	B2M	NA	Inverse variance weighted	8	-0.032	0.060	6.004E-01	0.974	pan	Severe COVID-19
9350_3	FSTL4	Follistatin-related protein 4	Q6MZW2	FSTL4	NA	Inverse variance weighted	2	0.100	0.190	6.006E-01	0.974	pan	Severe COVID-19
17698_15	WBP2	WW domain-binding protein 2	Q969T9	WBP2	NA	Wald ratio	1	-0.246	0.470	6.011E-01	0.974	pan	Severe COVID-19
5798_3	BID	BH3-interacting domain death agonist	P55957	BID	NA	Wald ratio	1	0.098	0.188	6.011E-01	0.974	pan	Severe COVID-19
14623_26	SUMO3	Small ubiquitin-related modifier 3	P55854	SUMO3	NA	Wald ratio	1	0.182	0.350	6.029E-01	0.974	pan	Severe COVID-19
3050_7	vWF	von Willebrand factor	P04275	VWF	NA	Inverse variance weighted	3	-0.111	0.215	6.034E-01	0.974	pan	Severe COVID-19
15579_26	ENPP6	Ectonucleotide pyrophosphatase/phosphodiesterase family member 6	Q6UWR7	ENPP6	NA	Inverse variance weighted	2	0.101	0.194	6.035E-01	0.974	pan	Severe COVID-19
4126_22	BPI	Bactericidal permeability-increasing protein	P17213	BPI	NA	Inverse variance weighted	13	-0.037	0.072	6.035E-01	0.974	pan	Severe COVID-19
5646_20	RNAS6	Ribonuclease K6	Q93091	RNASE6	NA	Inverse variance weighted	5	-0.016	0.030	6.053E-01	0.974	pan	Severe COVID-19
5000_52	LG3BP	Galectin-3-binding protein	Q08380	LGALS3BP	NA	Inverse variance weighted	10	-0.034	0.067	6.054E-01	0.974	pan	Severe COVID-19
3651_50	VEGF sR2	Vascular endothelial growth factor receptor 2	P35968	KDR	NA	Inverse variance weighted	9	-0.019	0.037	6.057E-01	0.974	pan	Severe COVID-19
8900_28	NEO1	Neogenin	Q92859	NEO1	NA	Inverse variance weighted	4	-0.037	0.072	6.058E-01	0.974	pan	Severe COVID-19
10082_251	NFL	Neurofilament light polypeptide	P07196	NEFL	NA	Inverse variance weighted	3	-0.210	0.407	6.064E-01	0.974	pan	Severe COVID-19
4240_31	M2-PK	Pyruvate kinase PKM	P14618	PKM	NA	Inverse variance weighted	3	-0.095	0.184	6.066E-01	0.974	pan	Severe COVID-19
8310_6	U773	Zymogen granule protein 16 homolog B	Q96DA0	ZG16B	NA	Wald ratio	1	0.049	0.095	6.068E-01	0.974	pan	Severe COVID-19
8982_65	TSP3	Thrombospondin-3	P49746	THBS3	NA	Inverse variance weighted	4	-0.092	0.179	6.069E-01	0.974	pan	Severe COVID-19
15324_58	Ferritin light chain	Ferritin light chain	P02792	FTL	NA	Inverse variance weighted	4	0.080	0.155	6.075E-01	0.974	pan	Severe COVID-19
9026_40	BTNL8	Butyrophilin-like protein 8	Q6UX41	BTNL8	NA	Inverse variance weighted	2	-0.047	0.092	6.075E-01	0.974	pan	Severe COVID-19
2827_23	Fractalkine/CX3CL-1	Fractalkine	P78423	CX3CL1	NA	Wald ratio	1	-0.083	0.161	6.079E-01	0.974	pan	Severe COVID-19
10041_3	HNF4A	Hepatocyte nuclear factor 4-alpha	P41235	HNF4A	NA	Wald ratio	1	-0.161	0.314	6.079E-01	0.974	pan	Severe COVID-19
9772_153	NLGN2:ECD	Neuroligin-2:Extracellular domain	Q8NFZ4	NLGN2	NA	Inverse variance weighted	2	0.060	0.117	6.090E-01	0.974	pan	Severe COVID-19
16060_99	NID2	Nidogen-2	Q14112	NID2	NA	Inverse variance weighted	9	0.019	0.037	6.094E-01	0.974	pan	Severe COVID-19
4979_34	DERM	Dermatopontin	Q07507	DPT	NA	Inverse variance weighted	7	0.022	0.042	6.096E-01	0.974	pan	Severe COVID-19
3169_70	IDUA	Alpha-L-iduronidase	P35475	IDUA	NA	Inverse variance weighted	6	0.025	0.049	6.102E-01	0.974	pan	Severe COVID-19
12786_61	GDE5	Glycerophosphocholine phosphodiesterase GPCPD1	Q9NPB8	GPCPD1	NA	Inverse variance weighted	2	0.090	0.176	6.102E-01	0.974	pan	Severe COVID-19
9348_1	C1RL1	Complement C1r subcomponent-like protein	Q9NZP8	C1RL	NA	Inverse variance weighted	2	0.101	0.199	6.102E-01	0.974	pan	Severe COVID-19
12813_18	EHBP1	EH domain-binding protein 1	Q8NDI1	EHBP1	NA	Wald ratio	1	-0.155	0.303	6.102E-01	0.974	pan	Severe COVID-19
9316_67	WFDC1	WAP four-disulfide core domain protein 1	Q9HC57	WFDC1	NA	Inverse variance weighted	5	0.021	0.041	6.102E-01	0.974	pan	Severe COVID-19

13374_4	DB113	Beta-defensin 113	Q30KQ7	DEFB113	NA	Wald ratio	1	0.166	0.326	6.103E-01	0.974	pan	Severe COVID-19
19523_215	PARK7	Protein DJ-1	Q99497	PARK7	NA	Wald ratio	1	0.083	0.162	6.107E-01	0.974	pan	Severe COVID-19
11098_1	PDXK	Pyridoxal kinase	O00764	PDXK	NA	Inverse variance weighted	4	0.075	0.147	6.111E-01	0.974	pan	Severe COVID-19
10916_44	PLA2R	Secretory phospholipase A2 receptor	Q13018	PLA2R1	NA	Inverse variance weighted	18	-0.018	0.036	6.116E-01	0.974	pan	Severe COVID-19
3151_6	IL-2 sRa	Interleukin-2 receptor subunit alpha	P01589	IL2RA	NA	Wald ratio	1	0.084	0.165	6.117E-01	0.974	pan	Severe COVID-19
12633_3	PTN9	Tyrosine-protein phosphatase non-receptor type 9	P43378	PTPN9	NA	Wald ratio	1	-0.132	0.261	6.119E-01	0.974	pan	Severe COVID-19
11211_7	TBCE	Tubulin-specific chaperone E	Q15813	TBCE	NA	Inverse variance weighted	2	-0.163	0.322	6.128E-01	0.974	pan	Severe COVID-19
3474_19	Thrombospondin-1	Thrombospondin-1	P07996	THBS1	NA	Wald ratio	1	0.158	0.312	6.131E-01	0.974	pan	Severe COVID-19
15475_4	PLTP	Phospholipid transfer protein	P55058	PLTP	NA	Inverse variance weighted	11	-0.039	0.077	6.131E-01	0.974	pan	Severe COVID-19
4328_2	BOC	Brother of CDO	Q9BWV1	BOC	NA	Inverse variance weighted	4	-0.033	0.065	6.133E-01	0.974	pan	Severe COVID-19
17837_5	CLIC2	Chloride intracellular channel protein 2	O15247	CLIC2	NA	Wald ratio	1	-0.158	0.313	6.134E-01	0.974	pan	Severe COVID-19
11287_14	Cytochrome b5	Cytochrome b5	P00167	CYB5A	NA	Wald ratio	1	0.194	0.384	6.139E-01	0.974	pan	Severe COVID-19
4996_66	HRG	Histidine-rich glycoprotein	P04196	HRG	NA	Inverse variance weighted	7	-0.043	0.085	6.140E-01	0.974	pan	Severe COVID-19
8250_2	PTPRJ	Receptor-type tyrosine-protein phosphatase eta	Q12913	PTPRJ	NA	Inverse variance weighted	8	-0.046	0.092	6.142E-01	0.974	pan	Severe COVID-19
19615_213	5NT3	Cytosolic 5'-nucleotidase 3A	Q9H0P0	NT5C3A	NA	Wald ratio	1	0.111	0.221	6.144E-01	0.974	pan	Severe COVID-19
9863_1	Tropomyosin 4	Tropomyosin alpha-4 chain	P67936	TPM4	NA	Wald ratio	1	0.101	0.200	6.151E-01	0.974	pan	Severe COVID-19
6521_35	NPTX2	Neuronal pentraxin-2	P47972	NPTX2	NA	Inverse variance weighted	3	0.103	0.206	6.161E-01	0.974	pan	Severe COVID-19
4929_55	SHBG	Sex hormone-binding globulin	P04278	SHBG	NA	Inverse variance weighted	5	0.065	0.131	6.181E-01	0.974	pan	Severe COVID-19
7989_5	CA226	Uncharacterized protein C1orf226	A1L170	C1orf226	NA	Wald ratio	1	0.125	0.252	6.185E-01	0.974	pan	Severe COVID-19
13516_46	TMM85	ER membrane protein complex subunit 4	Q5J8M3	EMC4	NA	Inverse variance weighted	7	-0.014	0.028	6.194E-01	0.974	pan	Severe COVID-19
16079_2	TEC	Tyrosine-protein kinase Tec	P42680	TEC	NA	Inverse variance weighted	2	0.060	0.122	6.200E-01	0.974	pan	Severe COVID-19
7788_1	CF6	ATP synthase-coupling factor 6, mitochondrial	P18859	ATP5PF	NA	Wald ratio	1	0.122	0.246	6.213E-01	0.974	pan	Severe COVID-19
9883_29	Glyoxalase I	Lactoylglutathione lyase	Q04760	GLO1	NA	Inverse variance weighted	2	0.064	0.129	6.213E-01	0.974	pan	Severe COVID-19
18882_7	CSTN2	Calsyntenin-2	Q9H4D0	CLSTN2	NA	Inverse variance weighted	10	-0.030	0.061	6.224E-01	0.974	pan	Severe COVID-19
10903_50	STX8	Syntaxin-8	Q9UNK0	STX8	NA	Wald ratio	1	0.118	0.241	6.233E-01	0.974	pan	Severe COVID-19
12743_18	BAG5	BAG family molecular chaperone regulator 5	Q9UL15	BAG5	NA	Wald ratio	1	0.193	0.393	6.236E-01	0.974	pan	Severe COVID-19
13545_97	EIF1A	Probable RNA-binding protein EIF1AD	Q8N9N8	EIF1AD	NA	Inverse variance weighted	3	0.084	0.172	6.238E-01	0.974	pan	Severe COVID-19
10835_25	A4GCT	Alpha-1,4-N-acetylglucosaminyltransferase	Q9UNA3	A4GNT	NA	Wald ratio	1	-0.114	0.232	6.238E-01	0.974	pan	Severe COVID-19
13384_110	FUMH	Fumarate hydratase, mitochondrial	P07954	FH	NA	Inverse variance weighted	2	0.173	0.353	6.242E-01	0.974	pan	Severe COVID-19
18220_141	SRA1	Steroid receptor RNA activator 1	Q9HD15	SRA1	NA	Inverse variance weighted	2	-0.087	0.178	6.251E-01	0.974	pan	Severe COVID-19
3035_80	IL-19	Interleukin-19	Q9UHD0	IL19	NA	Inverse variance weighted	5	-0.053	0.109	6.251E-01	0.974	pan	Severe COVID-19
4153_11	alpha-1-antichymotrypsin complex	Alpha-1-antichymotrypsin complex	P07288 | P01011	KLK3 | SERPINA3	NA	Inverse variance weighted	3	-0.071	0.146	6.260E-01	0.974	pan	Severe COVID-19
6442_6	BAGE3	B melanoma antigen 3	Q86Y29	BAGE3	NA	Wald ratio	1	-0.177	0.364	6.265E-01	0.974	pan	Severe COVID-19
7127_3	Apo A-II	Apolipoprotein A-II	P02652	APOA2	NA	Inverse variance weighted	11	0.038	0.078	6.268E-01	0.974	pan	Severe COVID-19
18242_8	Sperm protein 17	Sperm surface protein Sp17	Q15506	SPA17	NA	Wald ratio	1	-0.215	0.443	6.270E-01	0.974	pan	Severe COVID-19
15516_12	SAA-4	Serum amyloid A-4 protein	P35542	SAA4	NA	Inverse variance weighted	7	-0.024	0.050	6.272E-01	0.974	pan	Severe COVID-19
19323_1	RCAS1	Receptor-binding cancer antigen expressed on SiSo cells	O00559	EBAG9	NA	Inverse variance weighted	3	-0.094	0.194	6.285E-01	0.974	pan	Severe COVID-19
4459_68	PCSK7	Proprotein convertase subtilisin/kexin type 7	Q16549	PCSK7	NA	Inverse variance weighted	14	0.017	0.035	6.288E-01	0.974	pan	Severe COVID-19
9369_174	NGL1	Leucine-rich repeat-containing protein 4C	Q9HCJ2	LRRC4C	NA	Inverse variance weighted	4	0.051	0.105	6.290E-01	0.974	pan	Severe COVID-19
13692_154	WISP-1	WNT1-inducible-signaling pathway protein 1	O95388	CCN4	NA	Inverse variance weighted	12	0.014	0.030	6.294E-01	0.974	pan	Severe COVID-19
8088_56	CA210	Type III endosome membrane protein TEMP	Q8IVY1	C1orf210	NA	Wald ratio	1	-0.178	0.370	6.302E-01	0.974	pan	Severe COVID-19
8994_65	SLAMF8	SLAM family member 8	Q9P0V8	SLAMF8	NA	Inverse variance weighted	4	-0.097	0.202	6.304E-01	0.974	pan	Severe COVID-19
14747_9	CRLF1	Cytokine receptor-like factor 1	O75462	CRLF1	NA	Inverse variance weighted	2	-0.036	0.075	6.304E-01	0.974	pan	Severe COVID-19
13944_3	SULT 1A3	Sulfotransferase 1A3	P0DMM9	SULT1A3	NA	Inverse variance weighted	6	-0.063	0.131	6.305E-01	0.974	pan	Severe COVID-19
3580_25	a1-Antitrypsin	Alpha-1-antitrypsin	P01009	SERPINA1	NA	Inverse variance weighted	6	0.042	0.088	6.307E-01	0.974	pan	Severe COVID-19
9326_33	ITI heavy chain H2	Inter-alpha-trypsin inhibitor heavy chain H2	P19823	ITIH2	NA	Inverse variance weighted	10	0.029	0.061	6.308E-01	0.974	pan	Severe COVID-19
8480_29	FBLN3	EGF-containing fibulin-like extracellular matrix protein 1	Q12805	EFEMP1	NA	Inverse variance weighted	3	-0.051	0.106	6.309E-01	0.974	pan	Severe COVID-19
3293_2	CD5L	CD5 antigen-like	O43866	CD5L	NA	Inverse variance weighted	8	-0.036	0.076	6.314E-01	0.974	pan	Severe COVID-19
2972_57	BMP-7	Bone morphogenetic protein 7	P18075	BMP7	NA	Wald ratio	1	-0.153	0.320	6.320E-01	0.974	pan	Severe COVID-19
16015_19	ALT	Alanine aminotransferase 1	P24298	GPT	NA	Wald ratio	1	-0.134	0.281	6.320E-01	0.974	pan	Severe COVID-19
17170_15	CALCB	Calcitonin gene-related peptide 2	P10092	CALCB	NA	Inverse variance weighted	2	-0.037	0.077	6.321E-01	0.974	pan	Severe COVID-19
2780_35	Lactoferrin	Lactotransferrin	P02788	LTF	NA	Inverse variance weighted	2	0.098	0.204	6.324E-01	0.974	pan	Severe COVID-19
18299_13	CPNS1	Calpain small subunit 1	P04632	CAPNS1	NA	Inverse variance weighted	4	-0.048	0.100	6.325E-01	0.974	pan	Severe COVID-19
2579_17	MMP-9	Matrix metalloproteinase-9	P14780	MMP9	NA	Inverse variance weighted	6	0.070	0.146	6.338E-01	0.974	pan	Severe COVID-19
13463_1	PXDN	Peroxidasin homolog	Q92626	PXDN	NA	Inverse variance weighted	4	0.047	0.098	6.339E-01	0.974	pan	Severe COVID-19
3041_55	MRC2	C-type mannose receptor 2	Q9UBG0	MRC2	NA	Inverse variance weighted	5	-0.049	0.104	6.369E-01	0.974	pan	Severe COVID-19
13948_50	GABR2:ECD	Gamma-aminobutyric acid type B receptor subunit 2:Extracellular domain	O75899	GABBR2	NA	Wald ratio	1	0.129	0.274	6.371E-01	0.974	pan	Severe COVID-19
7953_20	SLAF1	Signaling lymphocytic activation molecule	Q13291	SLAMF1	NA	Wald ratio	1	0.125	0.266	6.379E-01	0.974	pan	Severe COVID-19
11547_84	MUSK	Muscle, skeletal receptor tyrosine-protein kinase	O15146	MUSK	NA	Inverse variance weighted	2	0.228	0.486	6.383E-01	0.974	pan	Severe COVID-19
12433_8	ARL11	ADP-ribosylation factor-like protein 11	Q969Q4	ARL11	NA	Inverse variance weighted	2	-0.114	0.242	6.389E-01	0.974	pan	Severe COVID-19
3216_2	PIGR	Polymeric immunoglobulin receptor	P01833	PIGR	NA	Inverse variance weighted	4	0.069	0.147	6.396E-01	0.974	pan	Severe COVID-19
8909_77	GNPI1	Glucosamine-6-phosphate isomerase 1	P46926	GNPDA1	NA	Wald ratio	1	0.055	0.117	6.397E-01	0.974	pan	Severe COVID-19
15427_35	LOXL3	Lysyl oxidase homolog 3	P58215	LOXL3	NA	Inverse variance weighted	11	0.018	0.039	6.398E-01	0.974	pan	Severe COVID-19
15491_20	CD248	Endosialin	Q9HCU0	CD248	NA	Wald ratio	1	0.130	0.278	6.403E-01	0.974	pan	Severe COVID-19
12436_84	GST omega-1	Glutathione S-transferase omega-1	P78417	GSTO1	NA	Inverse variance weighted	17	0.014	0.030	6.407E-01	0.974	pan	Severe COVID-19
8817_29	CENPV	Centromere protein V	Q7Z7K6	CENPV	NA	Wald ratio	1	-0.180	0.385	6.411E-01	0.974	pan	Severe COVID-19
12585_39	ERCC1	DNA excision repair protein ERCC-1	P07992	ERCC1	NA	Wald ratio	1	0.137	0.295	6.423E-01	0.974	pan	Severe COVID-19
3898_5	PSD7	26S proteasome non-ATPase regulatory subunit 7	P51665	PSMD7	NA	Wald ratio	1	-0.142	0.305	6.426E-01	0.974	pan	Severe COVID-19
5581_28	FGL1	Fibrinogen-like protein 1	Q08830	FGL1	NA	Inverse variance weighted	10	0.018	0.039	6.426E-01	0.974	pan	Severe COVID-19
6641_60	PolyUbiquitin K48	PolyUbiquitin K48-linked	P0CG47	UBB	NA	Wald ratio	1	-0.136	0.294	6.428E-01	0.974	pan	Severe COVID-19
13624_17	NADK	NAD kinase	O95544	NADK	NA	Wald ratio	1	0.058	0.125	6.429E-01	0.974	pan	Severe COVID-19
7227_75	COCH	Cochlin	O43405	COCH	NA	Inverse variance weighted	17	-0.019	0.042	6.430E-01	0.974	pan	Severe COVID-19
18918_86	PDE4A	cAMP-specific 3',5'-cyclic phosphodiesterase 4A	P27815	PDE4A	NA	Inverse variance weighted	4	0.068	0.147	6.432E-01	0.974	pan	Severe COVID-19
17751_68	CRBB1	Beta-crystallin B1	P53674	CRYBB1	NA	Inverse variance weighted	3	-0.039	0.084	6.435E-01	0.974	pan	Severe COVID-19
7655_11	N-terminal pro-BNP	N-terminal pro-BNP	P16860	NPPB	NA	Inverse variance weighted	4	-0.042	0.091	6.441E-01	0.974	pan	Severe COVID-19
9384_17	Cathelicidin peptide	Cathelicidin antimicrobial peptide	P49913	CAMP	NA	Inverse variance weighted	8	0.053	0.116	6.443E-01	0.974	pan	Severe COVID-19
9459_7	Fas, soluble	Tumor necrosis factor receptor superfamily member 6	P25445	FAS	NA	Inverse variance weighted	2	-0.129	0.278	6.444E-01	0.974	pan	Severe COVID-19
12399_194	CCD50	Coiled-coil domain-containing protein 50	Q8IVM0	CCDC50	NA	Inverse variance weighted	2	-0.167	0.361	6.445E-01	0.974	pan	Severe COVID-19

11273_176	GSTT2	Glutathione S-transferase theta-2B	P0CG30	GSTT2B	NA	Inverse variance weighted	4	0.055	0.121	6.475E-01	0.974	pan	Severe COVID-19
7192_37	CRF2-12	Interferon lambda receptor 1	Q8IU57	IFNLR1	NA	Inverse variance weighted	2	-0.097	0.213	6.476E-01	0.974	pan	Severe COVID-19
15447_45	Sorbitol dehydrogenase	Sorbitol dehydrogenase	Q00796	SORD	NA	Inverse variance weighted	5	-0.036	0.079	6.490E-01	0.974	pan	Severe COVID-19
17785_11	RCL	2'-deoxynucleoside 5'-phosphate N-hydrolase 1	O43598	DNPH1	NA	Wald ratio	1	0.120	0.263	6.490E-01	0.974	pan	Severe COVID-19
2977_7	EDAR	Tumor necrosis factor receptor superfamily member EDAR	Q9UNE0	EDAR	NA	Inverse variance weighted	15	-0.016	0.035	6.494E-01	0.974	pan	Severe COVID-19
5852_6	S100A12	Protein S100-A12	P80511	S100A12	NA	Inverse variance weighted	2	0.060	0.131	6.505E-01	0.974	pan	Severe COVID-19
2754_50	C3	Complement C3	P01024	C3	NA	Wald ratio	1	0.151	0.333	6.507E-01	0.974	pan	Severe COVID-19
15499_11	Attractin	Attractin	O75882	ATRN	NA	Inverse variance weighted	5	-0.038	0.085	6.508E-01	0.974	pan	Severe COVID-19
17161_1	OST48	Dolichyl-diphosphooligosaccharide--protein glycosyltransferase 48 kDa subunit	P39656	DDOST	NA	Inverse variance weighted	2	-0.074	0.163	6.508E-01	0.974	pan	Severe COVID-19
14006_36	GNMT	Glycine N-methyltransferase	Q14749	GNMT	NA	Inverse variance weighted	3	-0.059	0.130	6.509E-01	0.974	pan	Severe COVID-19
4559_64	KYNU	Kynureninase	Q16719	KYNU	NA	Inverse variance weighted	6	-0.031	0.069	6.509E-01	0.974	pan	Severe COVID-19
14662_6	MZF1	Myeloid zinc finger 1	P28698	MZF1	NA	Wald ratio	1	0.090	0.200	6.512E-01	0.974	pan	Severe COVID-19
15452_5	5'-Nucleotidase	5'-Nucleotidase	P21589	NT5E	NA	Inverse variance weighted	7	-0.019	0.042	6.513E-01	0.974	pan	Severe COVID-19
9360_33	EDIL3	EGF-like repeat and discoidin I-like domain-containing protein 3	O43854	EDIL3	NA	Inverse variance weighted	3	-0.075	0.165	6.514E-01	0.974	pan	Severe COVID-19
3173_49	ASAHL	N-acylethanolamine-hydrolyzing acid amidase	Q02083	NAAA	NA	Inverse variance weighted	24	-0.017	0.037	6.516E-01	0.974	pan	Severe COVID-19
8841_65	CILP2	Cartilage intermediate layer protein 2	Q8IUL8	CILP2	NA	Inverse variance weighted	2	0.201	0.445	6.517E-01	0.974	pan	Severe COVID-19
10938_13	sLFA-3	Lymphocyte function-associated antigen 3	P19256	CD58	NA	Inverse variance weighted	2	-0.086	0.191	6.527E-01	0.974	pan	Severe COVID-19
13929_27	OCTC	Peroxisomal carnitine O-octanoyltransferase	Q9UKG9	CROT	NA	Inverse variance weighted	9	-0.021	0.046	6.529E-01	0.974	pan	Severe COVID-19
17696_1	TIRP	TIR domain-containing adapter molecule 2	Q86XR7	TICAM2	NA	Wald ratio	1	0.182	0.405	6.530E-01	0.974	pan	Severe COVID-19
13717_15	FCN2	Ficolin-2	Q15485	FCN2	NA	Inverse variance weighted	11	0.027	0.060	6.531E-01	0.974	pan	Severe COVID-19
11277_23	ATF6A	Cyclic AMP-dependent transcription factor ATF-6 alpha	P18850	ATF6	NA	Inverse variance weighted	10	-0.037	0.083	6.538E-01	0.974	pan	Severe COVID-19
15343_337	Kininogen, HMW, Two Chain	Kininogen, HMW, Two Chain	P01042	KNG1	NA	Inverse variance weighted	4	-0.111	0.249	6.540E-01	0.974	pan	Severe COVID-19
7841_84	ESAM	Endothelial cell-selective adhesion molecule	Q96AP7	ESAM	NA	Inverse variance weighted	6	0.026	0.059	6.558E-01	0.974	pan	Severe COVID-19
3481_87	XPNPEP1	Xaa-Pro aminopeptidase 1	Q9NQW7	XPNPEP1	NA	Wald ratio	1	0.140	0.315	6.560E-01	0.974	pan	Severe COVID-19
5242_37	MP2K4	Dual specificity mitogen-activated protein kinase kinase 4	P45985	MAP2K4	NA	Inverse variance weighted	6	0.076	0.171	6.567E-01	0.974	pan	Severe COVID-19
9175_48	DSCAM	Down syndrome cell adhesion molecule	O60469	DSCAM	NA	Inverse variance weighted	2	-0.045	0.101	6.576E-01	0.974	pan	Severe COVID-19
11313_100	PHS	Pterin-4-alpha-carbinolamine dehydratase	P61457	PCBD1	NA	Inverse variance weighted	6	0.034	0.078	6.578E-01	0.974	pan	Severe COVID-19
17400_71	ACOT8	Acyl-coenzyme A thioesterase 8	O14734	ACOT8	NA	Wald ratio	1	0.229	0.517	6.578E-01	0.974	pan	Severe COVID-19
4342_10	sICAM-1	Intercellular adhesion molecule 1	P05362	ICAM1	NA	Inverse variance weighted	13	0.010	0.023	6.578E-01	0.974	pan	Severe COVID-19
9513_9	FGF22	Fibroblast growth factor 22	Q9HCT0	FGF22	NA	Wald ratio	1	0.158	0.357	6.581E-01	0.974	pan	Severe COVID-19
11681_8	NUPL	Arf-GAP domain and FG repeat-containing protein 1	P52594	AGFG1	NA	Wald ratio	1	-0.106	0.240	6.582E-01	0.974	pan	Severe COVID-19
12758_47	GRID2	Glutamate receptor ionotropic, delta-2	O43424	GRID2	NA	Inverse variance weighted	9	0.057	0.130	6.588E-01	0.974	pan	Severe COVID-19
16292_288	GIP	Gastric inhibitory polypeptide	P09681	GIP	NA	Wald ratio	1	-0.183	0.419	6.616E-01	0.974	pan	Severe COVID-19
19335_2	HN1	Hematological and neurological expressed 1 protein	Q9UK76	JPT1	NA	Wald ratio	1	0.149	0.340	6.619E-01	0.974	pan	Severe COVID-19
9357_4	CREG1	Protein CREG1	O75629	CREG1	NA	Inverse variance weighted	10	-0.013	0.031	6.623E-01	0.974	pan	Severe COVID-19
6221_1	ADPGK	ADP-dependent glucokinase	Q9BRR6	ADPGK	NA	Wald ratio	1	-0.233	0.535	6.629E-01	0.974	pan	Severe COVID-19
7140_1	ELA2A	Chymotrypsin-like elastase family member 2A	P08217	CELA2A	NA	Inverse variance weighted	4	0.050	0.115	6.635E-01	0.974	pan	Severe COVID-19
12376_85	p19-INK4d	Cyclin-dependent kinase 4 inhibitor D	P55273	CDKN2D	NA	Wald ratio	1	-0.210	0.484	6.639E-01	0.974	pan	Severe COVID-19
3313_21	FCN2	Ficolin-2	Q15485	FCN2	NA	Inverse variance weighted	3	-0.044	0.101	6.643E-01	0.974	pan	Severe COVID-19
19129_15	MTHFSD	Methenyltetrahydrofolate synthase domain-containing protein	Q2M296	MTHFSD	NA	Inverse variance weighted	5	-0.028	0.064	6.643E-01	0.974	pan	Severe COVID-19
4160_49	MMP-2	72 kDa type IV collagenase	P08253	MMP2	NA	Inverse variance weighted	2	-0.084	0.194	6.645E-01	0.974	pan	Severe COVID-19
5275_28	VAV	Proto-oncogene vav	P15498	VAV1	NA	Wald ratio	1	0.121	0.279	6.652E-01	0.974	pan	Severe COVID-19
15566_10	Calponin-1	Calponin-1	P51911	CNN1	NA	Inverse variance weighted	10	0.032	0.074	6.656E-01	0.974	pan	Severe COVID-19
8945_7	F175B	BRISC complex subunit Abro1	Q15018	ABRAXAS2	NA	Wald ratio	1	-0.105	0.244	6.664E-01	0.974	pan	Severe COVID-19
8942_2	RM21	39S ribosomal protein L21, mitochondrial	Q7Z2W9	MRPL21	NA	Wald ratio	1	-0.166	0.388	6.682E-01	0.974	pan	Severe COVID-19
2730_58	MICA	MHC class I polypeptide-related sequence A	Q29983	MICA	NA	Inverse variance weighted	13	-0.021	0.050	6.684E-01	0.974	pan	Severe COVID-19
12641_3	I5P2	Type II inositol 1,4,5-trisphosphate 5-phosphatase	P32019	INPP5B	NA	Inverse variance weighted	2	0.046	0.107	6.692E-01	0.974	pan	Severe COVID-19
3213_65	Nidogen	Nidogen-1	P14543	NID1	NA	Inverse variance weighted	2	0.105	0.245	6.692E-01	0.974	pan	Severe COVID-19
11278_4	COL11A2	Collagen alpha-2(XI) chain	P13942	COL11A2	NA	Inverse variance weighted	7	0.024	0.056	6.699E-01	0.974	pan	Severe COVID-19
15476_6	REG3G	Regenerating islet-derived protein 3-gamma	Q6UW15	REG3G	NA	Inverse variance weighted	17	0.015	0.035	6.712E-01	0.974	pan	Severe COVID-19
3853_56	MDHC	Malate dehydrogenase, cytoplasmic	P40925	MDH1	NA	Wald ratio	1	-0.096	0.226	6.713E-01	0.974	pan	Severe COVID-19
6574_11	FAIM3	Fas apoptotic inhibitory molecule 3	O60667	FCMR	NA	Inverse variance weighted	3	-0.075	0.177	6.720E-01	0.974	pan	Severe COVID-19
6895_1	TR:CD	Transferrin receptor protein 1:Cytoplasmic domain	P02786	TFRC	NA	Inverse variance weighted	3	0.054	0.128	6.728E-01	0.974	pan	Severe COVID-19
6990_44	SDF2L	Stromal cell-derived factor 2-like protein 1	Q9HCN8	SDF2L1	NA	Wald ratio	1	0.112	0.267	6.732E-01	0.974	pan	Severe COVID-19
18942_11	PSB9	Proteasome subunit beta type-9	P28065	PSMB9	NA	Wald ratio	1	0.140	0.331	6.733E-01	0.974	pan	Severe COVID-19
15336_7	SELM	Selenoprotein M	Q8WWX9	SELENOM	NA	Wald ratio	1	0.149	0.353	6.734E-01	0.974	pan	Severe COVID-19
18315_38	RTP4	Receptor-transporting protein 4	Q96DX8	RTP4	NA	Inverse variance weighted	4	0.048	0.113	6.734E-01	0.974	pan	Severe COVID-19
11130_158	CACB4	Voltage-dependent L-type calcium channel subunit beta-4	O00305	CACNB4	NA	Wald ratio	1	0.055	0.130	6.738E-01	0.974	pan	Severe COVID-19
12605_1	Exosome component 3	Exosome complex component RRP40	Q9NQT5	EXOSC3	NA	Wald ratio	1	-0.122	0.291	6.741E-01	0.974	pan	Severe COVID-19
16610_13	LRP10	Low-density lipoprotein receptor-related protein 10	Q7Z4F1	LRP10	NA	Inverse variance weighted	3	-0.056	0.133	6.742E-01	0.974	pan	Severe COVID-19
16055_3	complement factor H-related 5	Complement factor H-related protein 5	Q9BXR6	CFHR5	NA	Inverse variance weighted	6	-0.037	0.087	6.746E-01	0.974	pan	Severe COVID-19
11120_49	NAT14	N-acetyltransferase 14	Q8WUY8	NAT14	NA	Wald ratio	1	-0.068	0.161	6.746E-01	0.974	pan	Severe COVID-19
13930_3	ABC3G	DNA dC->dU-editing enzyme APOBEC-3G	Q9HC16	APOBEC3G	NA	Inverse variance weighted	2	0.048	0.113	6.749E-01	0.974	pan	Severe COVID-19
5954_62	PTH	Parathyroid hormone	P01270	PTH	NA	Inverse variance weighted	2	-0.056	0.134	6.755E-01	0.974	pan	Severe COVID-19
16825_20	ATX3	Ataxin-3	P54252	ATXN3	NA	Inverse variance weighted	4	-0.024	0.057	6.756E-01	0.974	pan	Severe COVID-19
4324_33	CYTT	Cystatin-SA	P09228	CST2	NA	Inverse variance weighted	9	0.040	0.095	6.760E-01	0.974	pan	Severe COVID-19
9867_23	F16P2	Fructose-1,6-bisphosphatase isozyme 2	O00757	FBP2	NA	Wald ratio	1	0.091	0.219	6.761E-01	0.974	pan	Severe COVID-19
4563_61	PLCG1	1-phosphatidylinositol 4,5-bisphosphate phosphodiesterase gamma-1	P19174	PLCG1	NA	Wald ratio	1	0.110	0.262	6.762E-01	0.974	pan	Severe COVID-19
4840_73	G-CSF	Granulocyte colony-stimulating factor	P09919	CSF3	NA	Wald ratio	1	0.213	0.509	6.762E-01	0.974	pan	Severe COVID-19
16853_5	NRK1	Nicotinamide riboside kinase 1	Q9NWW6	NMRK1	NA	Inverse variance weighted	2	0.051	0.122	6.767E-01	0.974	pan	Severe COVID-19
5615_62	F172A	Protein FAM172A	Q8WUF8	FAM172A	NA	Wald ratio	1	-0.087	0.209	6.778E-01	0.974	pan	Severe COVID-19
3004_67	PD-L2	Programmed cell death 1 ligand 2	Q9BQ51	PDCD1LG2	NA	Inverse variance weighted	6	-0.017	0.041	6.802E-01	0.974	pan	Severe COVID-19
2774_10	IL-16	Interleukin-16	Q14005	IL16	NA	Inverse variance weighted	6	-0.015	0.036	6.809E-01	0.974	pan	Severe COVID-19
3499_77	IL-17B	Interleukin-17B	Q9UHF5	IL17B	NA	Wald ratio	1	-0.086	0.209	6.814E-01	0.974	pan	Severe COVID-19
3485_28	b2-Microglobulin	Beta-2-microglobulin	P61769	B2M	NA	Inverse variance weighted	2	0.084	0.205	6.814E-01	0.974	pan	Severe COVID-19
4706_17	41	Protein 4.1	P11171	EPB41	NA	Wald ratio	1	-0.119	0.290	6.818E-01	0.974	pan	Severe COVID-19
15384_15	KLOTHO	Klotho	Q9UEF7	KL	NA	Inverse variance weighted	14	-0.029	0.071	6.823E-01	0.974	pan	Severe COVID-19
5637_81	NTNG1	Netrin-G1	Q9Y2I2	NTNG1	NA	Inverse variance weighted	15	0.013	0.032	6.825E-01	0.974	pan	Severe COVID-19

11117_2	SPT20	Spermatogenesis-associated protein 20	Q8TB22	SPATA20	NA	Inverse variance weighted	4	-0.025	0.060	6.830E-01	0.974	pan	Severe COVID-19
6123_69	p53	Cellular tumor antigen p53	P04637	TP53	NA	Inverse variance weighted	2	0.084	0.206	6.831E-01	0.974	pan	Severe COVID-19
2950_57	IGFBP-4	Insulin-like growth factor-binding protein 4	P22692	IGFBP4	NA	Inverse variance weighted	2	0.162	0.398	6.833E-01	0.974	pan	Severe COVID-19
3336_50	TFPI	Tissue factor pathway inhibitor	P10646	TFPI	NA	Wald ratio	1	-0.073	0.178	6.837E-01	0.974	pan	Severe COVID-19
19377_14	NOE2	Noelin-2	O95897	OLFM2	NA	Inverse variance weighted	8	0.010	0.025	6.851E-01	0.974	pan	Severe COVID-19
17210_2	TCL1A	T-cell leukemia/lymphoma protein 1A	P56279	TCL1A	NA	Inverse variance weighted	5	-0.056	0.138	6.854E-01	0.974	pan	Severe COVID-19
6485_59	IGLL1	Immunoglobulin lambda-like polypeptide 1	P15814	IGLL1	NA	Inverse variance weighted	26	0.015	0.036	6.860E-01	0.974	pan	Severe COVID-19
2652_15	suPAR	Urokinase plasminogen activator surface receptor	Q03405	PLAUR	NA	Wald ratio	1	0.188	0.464	6.860E-01	0.974	pan	Severe COVID-19
15363_32	Apo A-V	Apolipoprotein A-V	Q6Q788	APOA5	NA	Inverse variance weighted	11	0.022	0.053	6.868E-01	0.974	pan	Severe COVID-19
17410_5	CETN3	Centrin-3	O15182	CETN3	NA	Inverse variance weighted	3	-0.056	0.140	6.871E-01	0.974	pan	Severe COVID-19
10880_38	F163B	Protein FAM163B	P0C2L3	FAM163B	NA	Inverse variance weighted	3	-0.123	0.305	6.871E-01	0.974	pan	Severe COVID-19
14009_65	TNFAIP3	Tumor necrosis factor alpha-induced protein 3	P21580	TNFAIP3	NA	Wald ratio	1	-0.038	0.095	6.873E-01	0.974	pan	Severe COVID-19
10754_113	Prokineticin-2	Prokineticin-2	Q9HC23	PROK2	NA	Inverse variance weighted	9	-0.018	0.045	6.874E-01	0.974	pan	Severe COVID-19
14208_3	RET7	Retinoid-binding protein 7	Q96R05	RBP7	NA	Inverse variance weighted	2	-0.054	0.135	6.877E-01	0.974	pan	Severe COVID-19
17342_13	AGR3	Anterior gradient protein 3	Q8TD06	AGR3	NA	Wald ratio	1	0.132	0.328	6.887E-01	0.974	pan	Severe COVID-19
3329_14	PGRP-S	Peptidoglycan recognition protein 1	O75594	PGLYRP1	NA	Inverse variance weighted	4	0.039	0.097	6.892E-01	0.974	pan	Severe COVID-19
7970_315	NAR3	Ecto-ADP-ribosyltransferase 3	Q13508	ART3	NA	Inverse variance weighted	9	-0.025	0.063	6.899E-01	0.974	pan	Severe COVID-19
10815_2	HABP4	Intracellular hyaluronan-binding protein 4	Q5JVS0	HABP4	NA	Inverse variance weighted	5	0.037	0.093	6.904E-01	0.974	pan	Severe COVID-19
4984_83	Esterase D	S-formylglutathione hydrolase	P10768	ESD	NA	Inverse variance weighted	4	-0.031	0.079	6.908E-01	0.974	pan	Severe COVID-19
12422_143	LX15B	Arachidonate 15-lipoxygenase B	O15296	ALOX15B	NA	Wald ratio	1	0.083	0.208	6.916E-01	0.974	pan	Severe COVID-19
13416_8	T132D	Transmembrane protein 132D	Q14C87	TMEM132D	NA	Inverse variance weighted	5	-0.036	0.092	6.917E-01	0.974	pan	Severe COVID-19
19194_9	Histidyl-tRNA synthetase-related	D-tyrosyl-tRNA(Tyr) deacylase 1	Q8TEA8	DTD1	NA	Wald ratio	1	0.030	0.077	6.917E-01	0.974	pan	Severe COVID-19
8840_61	C1s	Complement C1s subcomponent	P09871	C1S	NA	Inverse variance weighted	3	0.063	0.159	6.919E-01	0.974	pan	Severe COVID-19
5689_1	DB112	Beta-defensin 112	Q30KQ8	DEFB112	NA	Wald ratio	1	0.068	0.172	6.923E-01	0.974	pan	Severe COVID-19
2190_55	Coagulation Factor XI	Coagulation Factor XI	P03951	F11	NA	Inverse variance weighted	5	-0.039	0.099	6.924E-01	0.974	pan	Severe COVID-19
6493_9	CBPZ	Carboxypeptidase Z	Q66K79	CPZ	NA	Inverse variance weighted	2	0.089	0.227	6.932E-01	0.974	pan	Severe COVID-19
11514_196	CD59	CD59 glycoprotein	P13987	CD59	NA	Inverse variance weighted	3	-0.058	0.148	6.940E-01	0.974	pan	Severe COVID-19
12020_39	PMGE	Bisphosphoglycerate mutase	P07738	BPGM	NA	Wald ratio	1	-0.190	0.484	6.945E-01	0.974	pan	Severe COVID-19
6627_25	LIPR1	Inactive pancreatic lipase-related protein 1	P54315	PNLIPRP1	NA	Inverse variance weighted	13	-0.027	0.070	6.947E-01	0.974	pan	Severe COVID-19
9816_37	ISOC1	Isochorismatase domain-containing protein 1	Q96CN7	ISOC1	NA	Inverse variance weighted	4	0.041	0.103	6.948E-01	0.974	pan	Severe COVID-19
7886_26	SPTC1	Serine palmitoyltransferase 1	O15269	SPTLC1	NA	Inverse variance weighted	2	0.072	0.184	6.949E-01	0.974	pan	Severe COVID-19
15635_4	SMOC2	SPARC-related modular calcium-binding protein 2	Q9H3U7	SMOC2	NA	Inverse variance weighted	5	-0.019	0.047	6.952E-01	0.974	pan	Severe COVID-19
14634_13	LSHR	Lutropin-choriogonadotropic hormone receptor	P22888	LHCGR	NA	Inverse variance weighted	5	-0.057	0.145	6.955E-01	0.974	pan	Severe COVID-19
15530_33	EphB4	Ephrin type-B receptor 4	P54760	EPHB4	NA	Inverse variance weighted	4	-0.064	0.164	6.957E-01	0.974	pan	Severe COVID-19
3421_54	CD30 Ligand	Tumor necrosis factor ligand superfamily member 8	P32971	TNFSF8	NA	Inverse variance weighted	2	-0.074	0.190	6.958E-01	0.974	pan	Severe COVID-19
12563_2	TFIP8	Tumor necrosis factor alpha-induced protein 8	O95379	TNFAIP8	NA	Inverse variance weighted	2	0.049	0.125	6.961E-01	0.974	pan	Severe COVID-19
7926_13	SPIT3	Kunitz-type protease inhibitor 3	P49223	SPINT3	NA	Inverse variance weighted	4	0.039	0.101	6.968E-01	0.974	pan	Severe COVID-19
8429_16	AXIN2	Axin-2	Q9Y2T1	AXIN2	NA	Inverse variance weighted	4	0.061	0.158	6.972E-01	0.974	pan	Severe COVID-19
13059_33	RIFK	Riboflavin kinase	Q969G6	RFK	NA	Inverse variance weighted	2	-0.059	0.151	6.982E-01	0.974	pan	Severe COVID-19
13959_7	LAP	Cytosol aminopeptidase	P28838	LAP3	NA	Wald ratio	1	-0.180	0.465	6.984E-01	0.974	pan	Severe COVID-19
6444_15	PSG3	Pregnancy-specific beta-1-glycoprotein 3	Q16557	PSG3	NA	Inverse variance weighted	6	-0.047	0.122	6.985E-01	0.974	pan	Severe COVID-19
3367_8	FETUB	Fetuin-B	Q9UGM5	FETUB	NA	Inverse variance weighted	7	-0.033	0.085	6.990E-01	0.974	pan	Severe COVID-19
19341_36	ACBD6	Acyl-CoA-binding domain-containing protein 6	Q9BR61	ACBD6	NA	Inverse variance weighted	5	-0.041	0.107	6.995E-01	0.974	pan	Severe COVID-19
15394_79	UNC5B	Netrin receptor UNC5B	Q8IZJ1	UNC5B	NA	Inverse variance weighted	4	0.046	0.118	7.002E-01	0.974	pan	Severe COVID-19
12530_14	CKS-1	Cyclin-dependent kinases regulatory subunit 1	P61024	CKS1B	NA	Inverse variance weighted	3	-0.066	0.171	7.004E-01	0.974	pan	Severe COVID-19
12386_11	AMPB	Aminopeptidase B	Q9H4A4	RNPEP	NA	Inverse variance weighted	5	0.033	0.085	7.010E-01	0.974	pan	Severe COVID-19
9076_25	PENK	Proenkephalin-A	P01210	PENK	NA	Inverse variance weighted	13	-0.011	0.028	7.013E-01	0.974	pan	Severe COVID-19
6367_66	fibromodulin	fibromodulin	Q06828	FMOD	NA	Inverse variance weighted	5	-0.040	0.105	7.021E-01	0.974	pan	Severe COVID-19
5704_74	Granzyme M	Granzyme M	P51124	GZMM	NA	Inverse variance weighted	2	-0.087	0.227	7.026E-01	0.974	pan	Severe COVID-19
3024_18	a2-Antiplasmin	Alpha-2-antiplasmin	P08697	SERPINF2	NA	Inverse variance weighted	6	0.033	0.085	7.027E-01	0.974	pan	Severe COVID-19
14685_17	PKB beta	RAC-beta serine/threonine-protein kinase	P31751	AKT2	NA	Inverse variance weighted	5	-0.071	0.186	7.030E-01	0.974	pan	Severe COVID-19
8017_23	FXRD1	FAD-dependent oxidoreductase domain-containing protein 1	Q96CU9	FOXRED1	NA	Wald ratio	1	-0.131	0.345	7.033E-01	0.974	pan	Severe COVID-19
4474_19	Ubiquitin	Ubiquitin	P62979	RPS27A	NA	Wald ratio	1	-0.154	0.404	7.036E-01	0.974	pan	Severe COVID-19
4991_12	GPC5	Glypican-5	P78333	GPC5	NA	Inverse variance weighted	11	-0.016	0.042	7.043E-01	0.974	pan	Severe COVID-19
15329_167	MAPIP	Ragulator complex protein LAMTOR2	Q9Y2Q5	LAMTOR2	NA	Wald ratio	1	0.156	0.412	7.047E-01	0.974	pan	Severe COVID-19
7753_21	HTAI2	Oxidoreductase HTATIP2	Q9BUP3	HTATIP2	NA	Wald ratio	1	0.154	0.407	7.052E-01	0.974	pan	Severe COVID-19
7141_21	MGT4B	Alpha-1,3-mannosyl-glycoprotein 4-beta-N-acetylglucosaminyltransferase B	Q9UQ53	MGAT4B	NA	Inverse variance weighted	4	-0.016	0.042	7.053E-01	0.974	pan	Severe COVID-19
7863_50	PGRC1	Membrane-associated progesterone receptor component 1	O00264	PGRMC1	NA	Wald ratio	1	0.144	0.382	7.057E-01	0.974	pan	Severe COVID-19
19267_14	GLO2	Hydroxyacylglutathione hydrolase, mitochondrial	Q16775	HAGH	NA	Wald ratio	1	0.038	0.101	7.062E-01	0.974	pan	Severe COVID-19
17755_5	UGDH	UDP-glucose 6-dehydrogenase	O60701	UGDH	NA	Inverse variance weighted	3	-0.026	0.069	7.065E-01	0.974	pan	Severe COVID-19
4467_49	SPARCL1	SPARC-like protein 1	Q14515	SPARCL1	NA	Inverse variance weighted	10	0.017	0.045	7.065E-01	0.974	pan	Severe COVID-19
8309_12	HYAL1	Hyaluronidase-1	Q12794	HYAL1	NA	Wald ratio	1	0.118	0.314	7.067E-01	0.974	pan	Severe COVID-19
3858_5	PPAC	Low molecular weight phosphotyrosine protein phosphatase	P24666	ACP1	NA	Inverse variance weighted	9	-0.012	0.031	7.067E-01	0.974	pan	Severe COVID-19
7696_3	NBR1	Next to BRCA1 gene 1 protein	Q14596	NBR1	NA	Wald ratio	1	-0.206	0.547	7.068E-01	0.974	pan	Severe COVID-19
2974_61	contactin-1	Contactin-1	Q12860	CNTN1	NA	Inverse variance weighted	7	-0.019	0.051	7.071E-01	0.974	pan	Severe COVID-19
11480_1	Aldehyde dehydrogenase, class 3	Aldehyde dehydrogenase, dimeric NADP-preferring	P30838	ALDH3A1	NA	Wald ratio	1	-0.047	0.125	7.085E-01	0.974	pan	Severe COVID-19
9049_2	MOT4	Monocarboxylate transporter 4	O15427	SLC16A3	NA	Wald ratio	1	0.172	0.459	7.086E-01	0.974	pan	Severe COVID-19
7933_75	ADA22	Disintegrin and metalloproteinase domain-containing protein 22	Q9P0K1	ADAM22	NA	Inverse variance weighted	5	0.017	0.047	7.092E-01	0.974	pan	Severe COVID-19
7115_5	Semenogelin	Semenogelin-1	P04279	SEMG1	NA	Inverse variance weighted	16	-0.022	0.059	7.094E-01	0.974	pan	Severe COVID-19
14614_41	CA130	Noncompact myelin-associated protein	Q5T1S8	NCMAP	NA	Inverse variance weighted	12	0.016	0.044	7.096E-01	0.974	pan	Severe COVID-19
4246_40	NCAM-L1	Neural cell adhesion molecule L1	P32004	L1CAM	NA	Inverse variance weighted	5	-0.052	0.141	7.102E-01	0.974	pan	Severe COVID-19
5632_6	CRAC1	Cartilage acidic protein 1	Q9NQ79	CRTAC1	NA	Inverse variance weighted	10	-0.013	0.036	7.108E-01	0.974	pan	Severe COVID-19
9253_52	BGAT	Histo-blood group ABO system transferase	P16442	ABO	NA	Inverse variance weighted	13	0.024	0.064	7.112E-01	0.974	pan	Severe COVID-19
4496_60	MMP-12	Macrophage metalloelastase	P39900	MMP12	NA	Inverse variance weighted	13	-0.011	0.029	7.115E-01	0.974	pan	Severe COVID-19
12408_333	RB22A	Ras-related protein Rab-22A	Q9UL26	RAB22A	NA	Inverse variance weighted	2	0.132	0.358	7.126E-01	0.974	pan	Severe COVID-19
9969_8	S22AG	Solute carrier family 22 member 16	Q86VW1	SLC22A16	NA	Inverse variance weighted	3	-0.090	0.244	7.127E-01	0.974	pan	Severe COVID-19
2961_1	Protein C	Vitamin K-dependent protein C	P04070	PROC	NA	Inverse variance weighted	8	-0.034	0.092	7.131E-01	0.974	pan	Severe COVID-19
9282_12	CRIS2	Cysteine-rich secretory protein 2	P16562	CRISP2	NA	Inverse variance weighted	33	-0.008	0.022	7.139E-01	0.974	pan	Severe COVID-19

6965_19	CNTP2	Contactin-associated protein-like 2	Q9UHC6	CNTNAP2	NA	Inverse variance weighted	6	0.023	0.063	7.142E-01	0.974	pan	Severe COVID-19
3309_2	FCG2A	Low affinity immunoglobulin gamma Fc region receptor II-a	P12318	FCGR2A	NA	Inverse variance weighted	8	-0.031	0.083	7.144E-01	0.974	pan	Severe COVID-19
7218_87	AT1B2	Sodium/potassium-transporting ATPase subunit beta-2	P14415	ATP1B2	NA	Inverse variance weighted	4	-0.035	0.095	7.149E-01	0.974	pan	Severe COVID-19
13587_10	RGAP1	Rac GTPase-activating protein 1	Q9H0H5	RACGAP1	NA	Inverse variance weighted	11	0.025	0.068	7.152E-01	0.974	pan	Severe COVID-19
17138_8	GST A1-1	Glutathione S-transferase A1	P08263	GSTA1	NA	Inverse variance weighted	4	-0.040	0.110	7.155E-01	0.974	pan	Severe COVID-19
17737_7	IVD	Isovaleryl-CoA dehydrogenase, mitochondrial	P26440	IVD	NA	Inverse variance weighted	3	0.051	0.139	7.159E-01	0.974	pan	Severe COVID-19
17345_12	ZADH2	Prostaglandin reductase 3	Q8N4Q0	PTGR3	NA	Wald ratio	1	-0.055	0.151	7.169E-01	0.974	pan	Severe COVID-19
14151_4	UCRP	Ubiquitin-like protein ISG15	P05161	ISG15	NA	Inverse variance weighted	4	-0.075	0.206	7.169E-01	0.974	pan	Severe COVID-19
12764_3	ELMO1	Engulfment and cell motility protein 1	Q92556	ELMO1	NA	Inverse variance weighted	3	0.047	0.129	7.176E-01	0.974	pan	Severe COVID-19
17746_77	FIS1	Mitochondrial fission 1 protein	Q9Y3D6	FIS1	NA	Inverse variance weighted	2	0.152	0.420	7.179E-01	0.974	pan	Severe COVID-19
13242_134	SHAN3	SH3 and multiple ankyrin repeat domains protein 3	Q9BYB0	SHANK3	NA	Inverse variance weighted	3	0.062	0.173	7.180E-01	0.974	pan	Severe COVID-19
6557_50	LRC15	Leucine-rich repeat-containing protein 15	Q8TF66	LRRC15	NA	Inverse variance weighted	4	0.050	0.140	7.184E-01	0.974	pan	Severe COVID-19
11540_37	FOXO3A	Forkhead box protein O3	O43524	FOXO3	NA	Wald ratio	1	0.106	0.294	7.185E-01	0.974	pan	Severe COVID-19
14271_23	RAB6B	Ras-related protein Rab-6B	Q9NRW1	RAB6B	NA	Wald ratio	1	0.044	0.122	7.190E-01	0.974	pan	Severe COVID-19
11606_22	DNJB6	DnaJ homolog subfamily B member 6	O75190	DNAJB6	NA	Wald ratio	1	0.115	0.322	7.197E-01	0.974	pan	Severe COVID-19
3179_51	Cathepsin A	Lysosomal protective protein	P10619	CTSA	NA	Inverse variance weighted	2	-0.242	0.676	7.207E-01	0.974	pan	Severe COVID-19
14143_8	H2B2E	Histone H2B type 2-E	Q16778	H2BC21	NA	Wald ratio	1	0.107	0.298	7.207E-01	0.974	pan	Severe COVID-19
5005_4	MK12	Mitogen-activated protein kinase 12	P53778	MAPK12	NA	Inverse variance weighted	2	0.037	0.103	7.210E-01	0.974	pan	Severe COVID-19
15559_5	ANTR2	Anthrax toxin receptor 2	P58335	ANTXR2	NA	Inverse variance weighted	5	0.040	0.113	7.223E-01	0.974	pan	Severe COVID-19
4137_57	IL-17E	Interleukin-25	Q9H293	IL25	NA	Inverse variance weighted	3	-0.078	0.220	7.227E-01	0.974	pan	Severe COVID-19
5350_14	GPC6	Glypican-6	Q9Y625	GPC6	NA	Inverse variance weighted	2	-0.077	0.217	7.229E-01	0.974	pan	Severe COVID-19
19637_9	CRH	Corticoliberin	P06850	CRH	NA	Wald ratio	1	0.087	0.245	7.230E-01	0.974	pan	Severe COVID-19
7849_3	Glutaminyl cyclase	Glutaminyl-peptide cyclotransferase	Q16769	QPCT	NA	Inverse variance weighted	7	-0.024	0.069	7.236E-01	0.974	pan	Severe COVID-19
10499_1	T106A	Transmembrane protein 106A	Q96A25	TMEM106A	NA	Inverse variance weighted	2	0.072	0.204	7.251E-01	0.974	pan	Severe COVID-19
2637_77	Macrophage mannose receptor	Macrophage mannose receptor 1	P22897	MRC1	NA	Inverse variance weighted	15	-0.015	0.042	7.254E-01	0.974	pan	Severe COVID-19
9906_21	TICN3	Testican-3	Q9BQ16	SPOCK3	NA	Inverse variance weighted	12	0.013	0.038	7.258E-01	0.974	pan	Severe COVID-19
4960_72	annexin I	Annexin A1	P04083	ANXA1	NA	Wald ratio	1	-0.040	0.115	7.258E-01	0.974	pan	Severe COVID-19
18172_71	ASF1A	Histone chaperone ASF1A	Q9Y294	ASF1A	NA	Inverse variance weighted	2	0.043	0.122	7.261E-01	0.974	pan	Severe COVID-19
6472_40	GP100	Melanocyte protein PMEL	P40967	PMEL	NA	Inverse variance weighted	14	0.020	0.057	7.269E-01	0.974	pan	Severe COVID-19
12556_7	UBE2C	Ubiquitin-conjugating enzyme E2 C	O00762	UBE2C	NA	Inverse variance weighted	2	-0.048	0.137	7.272E-01	0.974	pan	Severe COVID-19
19169_88	FIBP	Acidic fibroblast growth factor intracellular-binding protein	O43427	FIBP	NA	Wald ratio	1	-0.067	0.193	7.275E-01	0.974	pan	Severe COVID-19
12333_87	RPIA	Ribose-5-phosphate isomerase	P49247	RPIA	NA	Inverse variance weighted	2	-0.087	0.250	7.276E-01	0.974	pan	Severe COVID-19
4128_27	Eotaxin-2	C-C motif chemokine 24	O00175	CCL24	NA	Wald ratio	1	-0.113	0.325	7.278E-01	0.974	pan	Severe COVID-19
4990_87	GP1BA	Platelet glycoprotein Ib alpha chain	P07359	GP1BA	NA	Inverse variance weighted	9	-0.033	0.095	7.284E-01	0.974	pan	Severe COVID-19
13983_27	QOR	Quinone oxidoreductase	Q08257	CRYZ	NA	Inverse variance weighted	15	0.010	0.027	7.284E-01	0.974	pan	Severe COVID-19
4374_45	MIC-1	Growth/differentiation factor 15	Q99988	GDF15	NA	Inverse variance weighted	9	-0.017	0.048	7.290E-01	0.974	pan	Severe COVID-19
15444_45	SCCA2	Serpin B4	P48594	SERPINB4	NA	Inverse variance weighted	5	-0.047	0.135	7.292E-01	0.974	pan	Severe COVID-19
6433_57	FA20A	Pseudokinase FAM20A	Q96MK3	FAM20A	NA	Inverse variance weighted	3	0.060	0.174	7.297E-01	0.974	pan	Severe COVID-19
3795_6	ADAM 9	Disintegrin and metalloproteinase domain-containing protein 9	Q13443	ADAM9	NA	Wald ratio	1	0.126	0.367	7.305E-01	0.974	pan	Severe COVID-19
6375_75	XXLT1	Xyloside xylosyltransferase 1	Q8NBI6	XXYLT1	NA	Inverse variance weighted	2	0.023	0.068	7.306E-01	0.974	pan	Severe COVID-19
14332_3	RALA	Ras-related protein Ral-A	P11233	RALA	NA	Inverse variance weighted	2	-0.083	0.240	7.310E-01	0.974	pan	Severe COVID-19
17813_21	BPHL	Valacyclovir hydrolase	Q86WA6	BPHL	NA	Inverse variance weighted	2	-0.110	0.319	7.310E-01	0.974	pan	Severe COVID-19
2851_63	C5a	C5a anaphylatoxin	P01031	C5	NA	Inverse variance weighted	4	-0.039	0.115	7.315E-01	0.974	pan	Severe COVID-19
7083_74	MATN4	Matrilin-4	O95460	MATN4	NA	Inverse variance weighted	3	0.033	0.096	7.317E-01	0.974	pan	Severe COVID-19
12968_2	CSRP2	Cysteine and glycine-rich protein 2	Q16527	CSRP2	NA	Wald ratio	1	0.098	0.287	7.321E-01	0.974	pan	Severe COVID-19
9964_10	MYZAP	Myocardial zonula adherens protein	P0CAP1	MYZAP	NA	Inverse variance weighted	2	-0.065	0.190	7.325E-01	0.974	pan	Severe COVID-19
8859_51	CAH11	Carbonic anhydrase-related protein 11	O75493	CA11	NA	Inverse variance weighted	2	0.130	0.382	7.339E-01	0.974	pan	Severe COVID-19
3852_19	HSP 40	DnaJ homolog subfamily B member 1	P25685	DNAJB1	NA	Inverse variance weighted	3	0.116	0.342	7.340E-01	0.974	pan	Severe COVID-19
16607_78	Gelsolin	Gelsolin	P06396	GSN	NA	Inverse variance weighted	9	-0.034	0.102	7.343E-01	0.974	pan	Severe COVID-19
11128_29	T132C:CD	Transmembrane protein 132C:Cytoplasmic domain	Q8N3T6	TMEM132C	NA	Wald ratio	1	-0.114	0.336	7.352E-01	0.974	pan	Severe COVID-19
2381_52	C5	Complement C5	P01031	C5	NA	Inverse variance weighted	2	0.041	0.123	7.370E-01	0.974	pan	Severe COVID-19
7145_1	ITIH3	Inter-alpha-trypsin inhibitor heavy chain H3	Q06033	ITIH3	NA	Inverse variance weighted	11	0.023	0.068	7.371E-01	0.974	pan	Severe COVID-19
9256_78	NPTX1	Neuronal pentraxin-1	Q15818	NPTX1	NA	Inverse variance weighted	10	-0.015	0.046	7.372E-01	0.974	pan	Severe COVID-19
2590_69	ROR1	Inactive tyrosine-protein kinase transmembrane receptor ROR1	Q01973	ROR1	NA	Inverse variance weighted	4	0.022	0.065	7.383E-01	0.974	pan	Severe COVID-19
17490_4	SH3BGRL3-like protein	SH3 domain-binding glutamic acid-rich-like protein 3	Q9H299	SH3BGRL3	NA	Inverse variance weighted	4	0.036	0.107	7.384E-01	0.974	pan	Severe COVID-19
7761_125	CHKB	Choline/ethanolamine kinase	Q9Y259	CHKB	NA	Inverse variance weighted	3	-0.041	0.122	7.396E-01	0.974	pan	Severe COVID-19
16561_9	Alpha-1B-glycoprotein	Alpha-1B-glycoprotein	P04217	A1BG	NA	Inverse variance weighted	8	0.011	0.034	7.407E-01	0.974	pan	Severe COVID-19
17435_43	ETFA	Electron transfer flavoprotein subunit alpha, mitochondrial	P13804	ETFA	NA	Inverse variance weighted	6	0.043	0.131	7.408E-01	0.974	pan	Severe COVID-19
2789_26	MMP-7	Matrilysin	P09237	MMP7	NA	Inverse variance weighted	7	-0.022	0.067	7.409E-01	0.974	pan	Severe COVID-19
9578_263	MANS4	MANSC domain-containing protein 4	A6NHS7	MANSC4	NA	Inverse variance weighted	10	0.010	0.032	7.412E-01	0.974	pan	Severe COVID-19
12459_13	PKHA1	Pleckstrin homology domain-containing family A member 1	Q9HB21	PLEKHA1	NA	Inverse variance weighted	3	0.016	0.050	7.415E-01	0.974	pan	Severe COVID-19
6947_4	SIA10	Type 2 lactosamine alpha-2,3-sialyltransferase	Q9Y274	ST3GAL6	NA	Inverse variance weighted	30	-0.007	0.022	7.420E-01	0.974	pan	Severe COVID-19
3184_25	Coagulation Factor VII	Coagulation factor VII	P08709	F7	NA	Inverse variance weighted	10	0.011	0.034	7.425E-01	0.974	pan	Severe COVID-19
12384_92	CSN7B	COP9 signalosome complex subunit 7b	Q9H9Q2	COPS7B	NA	Inverse variance weighted	4	0.065	0.198	7.425E-01	0.974	pan	Severe COVID-19
9394_19	Aminopeptidase	Carboxypeptidase Q	Q9Y646	CPQ	NA	Inverse variance weighted	12	0.014	0.042	7.435E-01	0.974	pan	Severe COVID-19
19251_56	Platelet proteoglycan	Serglycin	P10124	SRGN	NA	Inverse variance weighted	7	0.020	0.061	7.442E-01	0.974	pan	Severe COVID-19
8402_22	CYTL1	Cytokine-like protein 1	Q9NRR1	CYTL1	NA	Inverse variance weighted	3	-0.031	0.094	7.444E-01	0.974	pan	Severe COVID-19
3796_79	ANGL4	Angiopoietin-related protein 4	Q9BY76	ANGPTL4	NA	Wald ratio	1	-0.083	0.253	7.446E-01	0.974	pan	Severe COVID-19
3028_36	Ck-b-8-1	Ck-beta-8-1	P55773	CCL23	NA	Inverse variance weighted	4	-0.049	0.151	7.447E-01	0.974	pan	Severe COVID-19
3054_3	Haptoglobin, Mixed Type	Haptoglobin	P00738	HP	NA	Inverse variance weighted	10	-0.031	0.097	7.452E-01	0.974	pan	Severe COVID-19
15440_57	NEC2	Neuroendocrine convertase 2	P16519	PCSK2	NA	Wald ratio	1	-0.058	0.178	7.456E-01	0.974	pan	Severe COVID-19
16890_37	ATL1	ADAMTS-like protein 1	Q8N6G6	ADAMTSL1	NA	Inverse variance weighted	8	0.017	0.053	7.457E-01	0.974	pan	Severe COVID-19
5737_61	SEM4D	Semaphorin-4D	Q92854	SEMA4D	NA	Inverse variance weighted	8	-0.016	0.048	7.462E-01	0.974	pan	Severe COVID-19
2436_49	CXCL16, soluble	C-X-C motif chemokine 16	Q9H2A7	CXCL16	NA	Inverse variance weighted	11	0.030	0.093	7.469E-01	0.974	pan	Severe COVID-19
19563_3	SEZ6L	Seizure 6-like protein	Q9BYH1	SEZ6L	NA	Inverse variance weighted	3	0.043	0.133	7.469E-01	0.974	pan	Severe COVID-19
5256_86	PDE5A	cGMP-specific 3',5'-cyclic phosphodiesterase	O76074	PDE5A	NA	Inverse variance weighted	5	-0.041	0.128	7.473E-01	0.974	pan	Severe COVID-19
4162_54	Transferrin	Serotransferrin	P02787	TF	NA	Inverse variance weighted	2	-0.070	0.216	7.476E-01	0.974	pan	Severe COVID-19
7157_22	PCDA4	Protocadherin alpha-4	Q9UN74	PCDHA4	NA	Inverse variance weighted	12	0.037	0.114	7.479E-01	0.974	pan	Severe COVID-19

7986_98	NALD2	N-acetylated-alpha-linked acidic dipeptidase 2	Q9Y3Q0	NAALAD2	NA	Inverse variance weighted	10	0.013	0.042	7.480E-01	0.974	pan	Severe COVID-19
19135_5	Cytosolic 5'-nucleotidase III-like protein	7-methylguanosine phosphate-specific 5'-nucleotidase	Q969T7	NT5C3B	NA	Inverse variance weighted	13	-0.016	0.048	7.482E-01	0.974	pan	Severe COVID-19
6378_2	CEI	Protein CEI	Q86SI9	C5orf38	NA	Wald ratio	1	-0.078	0.242	7.483E-01	0.974	pan	Severe COVID-19
13720_95	Proteinase-3	Myeloblastin	P24158	PRTN3	NA	Inverse variance weighted	9	0.012	0.036	7.487E-01	0.974	pan	Severe COVID-19
10569_28	MFAP2	Microfibrillar-associated protein 2	P55001	MFAP2	NA	Inverse variance weighted	3	0.064	0.201	7.491E-01	0.974	pan	Severe COVID-19
7251_64	C1QT3	Complement C1q tumor necrosis factor-related protein 3	Q9BXJ4	C1QTNF3	NA	Inverse variance weighted	3	0.032	0.102	7.498E-01	0.974	pan	Severe COVID-19
15556_49	Alpha-amylase 2B	Alpha-amylase 2B	P19961	AMY2B	NA	Inverse variance weighted	4	0.045	0.141	7.512E-01	0.974	pan	Severe COVID-19
10565_19	SLIK3	SLIT and NTRK-like protein 3	O94933	SLITRK3	NA	Inverse variance weighted	15	0.016	0.050	7.515E-01	0.974	pan	Severe COVID-19
16070_7	WIF-1	Wnt inhibitory factor 1	Q9Y5W5	WIF1	NA	Wald ratio	1	-0.079	0.250	7.518E-01	0.974	pan	Severe COVID-19
9278_9	SDF-1	Stromal cell-derived factor 1	P48061	CXCL12	NA	Inverse variance weighted	7	-0.042	0.132	7.522E-01	0.974	pan	Severe COVID-19
3196_6	HPLN1	Hyaluronan and proteoglycan link protein 1	P10915	HAPLN1	NA	Inverse variance weighted	3	0.038	0.121	7.523E-01	0.974	pan	Severe COVID-19
8304_50	OPG	Tumor necrosis factor receptor superfamily member 11B	O00300	TNFRSF11B	NA	Inverse variance weighted	3	-0.026	0.084	7.524E-01	0.974	pan	Severe COVID-19
13427_66	MA1C1	Mannosyl-oligosaccharide 1,2-alpha-mannosidase IC	Q9NR34	MAN1C1	NA	Inverse variance weighted	4	-0.082	0.259	7.525E-01	0.974	pan	Severe COVID-19
13998_26	PURA1	Adenylosuccinate synthetase isozyme 1	Q8N142	ADSS1	NA	Inverse variance weighted	3	-0.026	0.084	7.528E-01	0.974	pan	Severe COVID-19
12812_25	ACYP2	Acylphosphatase-2	P14621	ACYP2	NA	Inverse variance weighted	2	0.021	0.066	7.528E-01	0.974	pan	Severe COVID-19
14309_8	HNRH1	Heterogeneous nuclear ribonucleoprotein H	P31943	HNRNPH1	NA	Wald ratio	1	0.102	0.324	7.539E-01	0.974	pan	Severe COVID-19
13102_1	FAM3D	Protein FAM3D	Q96BQ1	FAM3D	NA	Inverse variance weighted	9	-0.016	0.052	7.543E-01	0.974	pan	Severe COVID-19
8459_10	BMP-6	Bone morphogenetic protein 6	P22004	BMP6	NA	Inverse variance weighted	3	-0.049	0.158	7.544E-01	0.974	pan	Severe COVID-19
13597_20	RAB31	Ras-related protein Rab-31	Q13636	RAB31	NA	Inverse variance weighted	3	0.098	0.312	7.545E-01	0.974	pan	Severe COVID-19
7038_45	SIA8D	CMP-N-acetylneuraminate-poly-alpha-2,8-sialyltransferase	Q92187	ST8SIA4	NA	Wald ratio	1	0.134	0.429	7.546E-01	0.974	pan	Severe COVID-19
7638_30	Lectin, mannose-binding 2	Vesicular integral-membrane protein VIP36	Q12907	LMAN2	NA	Inverse variance weighted	2	0.087	0.278	7.547E-01	0.974	pan	Severe COVID-19
15364_101	Apo C-I	Apolipoprotein C-I	P02654	APOC1	NA	Inverse variance weighted	2	-0.122	0.391	7.553E-01	0.974	pan	Severe COVID-19
16309_30	Soggy-1	Dickkopf-like protein 1	Q9UK85	DKKL1	NA	Wald ratio	1	0.119	0.381	7.554E-01	0.974	pan	Severe COVID-19
9715_15	IGSF3	Immunoglobulin superfamily member 3	O75054	IGSF3	NA	Inverse variance weighted	5	0.055	0.176	7.556E-01	0.974	pan	Severe COVID-19
11264_33	XDH	Xanthine dehydrogenase/oxidase	P47989	XDH	NA	Wald ratio	1	0.100	0.322	7.557E-01	0.974	pan	Severe COVID-19
14047_78	BDNF	Brain-derived neurotrophic factor	P23560	BDNF	NA	Inverse variance weighted	2	0.091	0.293	7.560E-01	0.974	pan	Severe COVID-19
15526_33	GSHB	Glutathione synthetase	P48637	GSS	NA	Inverse variance weighted	4	0.040	0.130	7.570E-01	0.975	pan	Severe COVID-19
10514_5	PGD2 synthase	Prostaglandin-H2 D-isomerase	P41222	PTGDS	NA	Inverse variance weighted	2	-0.114	0.370	7.579E-01	0.975	pan	Severe COVID-19
3186_2	C2	Complement C2	P06681	C2	NA	Inverse variance weighted	9	0.024	0.079	7.580E-01	0.975	pan	Severe COVID-19
4886_3	MCP-3	C-C motif chemokine 7	P80098	CCL7	NA	Inverse variance weighted	5	-0.019	0.062	7.593E-01	0.976	pan	Severe COVID-19
6391_52	LIRA3	Leukocyte immunoglobulin-like receptor subfamily A member 3	Q8N6C8	LILRA3	NA	Inverse variance weighted	8	0.018	0.061	7.602E-01	0.976	pan	Severe COVID-19
9713_67	PGFRL	Platelet-derived growth factor receptor-like protein	Q15198	PDGFRL	NA	Inverse variance weighted	6	-0.013	0.043	7.610E-01	0.976	pan	Severe COVID-19
9322_15	RCN1	Reticulocalbin-1	Q15293	RCN1	NA	Inverse variance weighted	2	-0.067	0.221	7.617E-01	0.976	pan	Severe COVID-19
15446_25	SMDF	Neuregulin-1, sensory and motor neuron-derived factor isoform	Q02297	NRG1	NA	Inverse variance weighted	2	-0.048	0.159	7.631E-01	0.976	pan	Severe COVID-19
19261_12	SDCB2	Syntenin-2	Q9H190	SDCBP2	NA	Wald ratio	1	0.082	0.274	7.641E-01	0.976	pan	Severe COVID-19
12571_14	ARL3	ADP-ribosylation factor-like protein 3	P36405	ARL3	NA	Inverse variance weighted	2	-0.075	0.250	7.643E-01	0.976	pan	Severe COVID-19
6448_36	Sema E	Semaphorin-3C	Q99985	SEMA3C	NA	Inverse variance weighted	2	0.040	0.133	7.644E-01	0.976	pan	Severe COVID-19
14655_1	DJC17	DnaJ homolog subfamily C member 17	Q9NVM6	DNAJC17	NA	Inverse variance weighted	4	-0.033	0.109	7.649E-01	0.976	pan	Severe COVID-19
2944_66	DAN	Neuroblastoma suppressor of tumorigenicity 1	P41271	NBL1	NA	Wald ratio	1	-0.110	0.369	7.659E-01	0.976	pan	Severe COVID-19
6580_29	Pregnancy zone protein	Pregnancy zone protein	P20742	PZP	NA	Inverse variance weighted	5	-0.041	0.137	7.669E-01	0.976	pan	Severe COVID-19
13399_33	RELL1	RELT-like protein 1	Q8IUW5	RELL1	NA	Inverse variance weighted	2	0.037	0.126	7.682E-01	0.976	pan	Severe COVID-19
18876_77	CHST4	Carbohydrate sulfotransferase 4	Q8NCG5	CHST4	NA	Wald ratio	1	0.120	0.410	7.700E-01	0.976	pan	Severe COVID-19
19631_13	Kininostatin	Kininostatin	P01042	KNG1	NA	Inverse variance weighted	12	-0.033	0.112	7.703E-01	0.976	pan	Severe COVID-19
18189_12	VPS25	Vacuolar protein-sorting-associated protein 25	Q9BRG1	VPS25	NA	Inverse variance weighted	2	0.058	0.200	7.703E-01	0.976	pan	Severe COVID-19
12671_35	ST1B1	Sulfotransferase family cytosolic 1B member 1	O43704	SULT1B1	NA	Inverse variance weighted	6	-0.031	0.106	7.712E-01	0.976	pan	Severe COVID-19
17456_53	GOLM1	Golgi membrane protein 1	Q8NBJ4	GOLM1	NA	Inverse variance weighted	6	0.028	0.096	7.715E-01	0.976	pan	Severe COVID-19
9876_20	aldolase C	Fructose-bisphosphate aldolase C	P09972	ALDOC	NA	Inverse variance weighted	3	-0.077	0.264	7.717E-01	0.976	pan	Severe COVID-19
15610_72	LAP	Cytosol aminopeptidase	P28838	LAP3	NA	Inverse variance weighted	2	0.074	0.254	7.720E-01	0.976	pan	Severe COVID-19
11872_9	PCDGD	Protocadherin gamma-B1	Q9Y5G3	PCDHGB1	NA	Inverse variance weighted	2	0.087	0.299	7.720E-01	0.976	pan	Severe COVID-19
5066_134	CLM6	CMRF35-like molecule 6	Q08708	CD300C	NA	Inverse variance weighted	13	-0.023	0.078	7.720E-01	0.976	pan	Severe COVID-19
12635_9	TRDMT	tRNA (cytosine(38)-C(5))-methyltransferase	O14717	TRDMT1	NA	Inverse variance weighted	4	-0.022	0.076	7.726E-01	0.976	pan	Severe COVID-19
12662_82	ECH1	Delta(3,5)-Delta(2,4)-dienoyl-CoA isomerase, mitochondrial	Q13011	ECH1	NA	Inverse variance weighted	2	0.035	0.120	7.734E-01	0.976	pan	Severe COVID-19
6918_183	CCKN	Cholecystokinin	P06307	CCK	NA	Inverse variance weighted	2	-0.152	0.528	7.734E-01	0.976	pan	Severe COVID-19
11910_27	DLX4	Homeobox protein DLX-4	Q92988	DLX4	NA	Inverse variance weighted	2	0.054	0.188	7.738E-01	0.976	pan	Severe COVID-19
6625_31	ATAD1	ATPase family AAA domain-containing protein 1	Q8NBU5	ATAD1	NA	Wald ratio	1	0.033	0.113	7.740E-01	0.976	pan	Severe COVID-19
9986_14	Neuropeptide W	Neuropeptide W	Q8N729	NPW	NA	Inverse variance weighted	5	-0.022	0.075	7.745E-01	0.976	pan	Severe COVID-19
10053_5	ILK1	Integrin-linked protein kinase	Q13418	ILK	NA	Wald ratio	1	0.077	0.269	7.749E-01	0.976	pan	Severe COVID-19
18326_50	MAGA8	Melanoma-associated antigen 8	P43361	MAGEA8	NA	Wald ratio	1	0.100	0.349	7.751E-01	0.976	pan	Severe COVID-19
9183_7	IFN-a/b R1	Interferon alpha/beta receptor 1	P17181	IFNAR1	NA	Inverse variance weighted	9	0.010	0.037	7.755E-01	0.976	pan	Severe COVID-19
3311_27	FCG3B	Low affinity immunoglobulin gamma Fc region receptor III-B	O75015	FCGR3B	NA	Inverse variance weighted	8	0.035	0.125	7.758E-01	0.976	pan	Severe COVID-19
15523_9	HEM2	Delta-aminolevulinic acid dehydratase	P13716	ALAD	NA	Inverse variance weighted	8	-0.016	0.056	7.768E-01	0.976	pan	Severe COVID-19
13982_33	RGS18	Regulator of G-protein signaling 18	Q9NS28	RGS18	NA	Wald ratio	1	-0.071	0.252	7.770E-01	0.976	pan	Severe COVID-19
11428_31	PDLI1	PDZ and LIM domain protein 1	O00151	PDLIM1	NA	Wald ratio	1	0.059	0.209	7.784E-01	0.976	pan	Severe COVID-19
9848_22	Cyclin H	Cyclin-H	P51946	CCNH	NA	Wald ratio	1	0.050	0.178	7.786E-01	0.976	pan	Severe COVID-19
18180_58	RT4I1	Reticulon-4-interacting protein 1, mitochondrial	Q8WWV3	RTN4IP1	NA	Wald ratio	1	0.069	0.245	7.787E-01	0.976	pan	Severe COVID-19
12594_5	GRAN	Grancalcin	P28676	GCA	NA	Inverse variance weighted	2	0.017	0.061	7.793E-01	0.976	pan	Severe COVID-19
15312_14	AGO1	Protein argonaute-1	Q9UL18	AGO1	NA	Wald ratio	1	0.077	0.278	7.807E-01	0.976	pan	Severe COVID-19
19159_9	DNA polymerase subunit delta 4	DNA polymerase delta subunit 4	Q9HCU8	POLD4	NA	Wald ratio	1	0.095	0.341	7.811E-01	0.976	pan	Severe COVID-19
12366_16	CRGD	Gamma-crystallin D	P07320	CRYGD	NA	Wald ratio	1	-0.039	0.141	7.813E-01	0.976	pan	Severe COVID-19
7897_75	DLG3	Disks large homolog 3	Q92796	DLG3	NA	Inverse variance weighted	5	0.056	0.201	7.816E-01	0.976	pan	Severe COVID-19
10056_5	FOXM1	Forkhead box protein M1	Q08050	FOXM1	NA	Wald ratio	1	0.051	0.185	7.820E-01	0.976	pan	Severe COVID-19
10088_37	APT	Adenine phosphoribosyltransferase	P07741	APRT	NA	Inverse variance weighted	7	-0.019	0.069	7.824E-01	0.976	pan	Severe COVID-19
6453_70	LIRB4	Leukocyte immunoglobulin-like receptor subfamily B member 4	Q8NHJ6	LILRB4	NA	Inverse variance weighted	2	0.056	0.203	7.830E-01	0.976	pan	Severe COVID-19
19145_4	MREG	Melanoregulin	Q8N565	MREG	NA	Inverse variance weighted	3	0.043	0.155	7.833E-01	0.976	pan	Severe COVID-19
7628_40	CREL1	Cysteine-rich with EGF-like domain protein 1	Q96HD1	CRELD1	NA	Inverse variance weighted	13	0.011	0.039	7.838E-01	0.976	pan	Severe COVID-19
15522_2	GAPR1	Golgi-associated plant pathogenesis-related protein 1	Q9H4G4	GLIPR2	NA	Inverse variance weighted	6	0.026	0.095	7.841E-01	0.976	pan	Severe COVID-19
12620_3	Septin-11	Septin-11	Q9NVA2	SEPTIN11	NA	Inverse variance weighted	2	0.052	0.189	7.846E-01	0.976	pan	Severe COVID-19
6457_50	PCDG1	Protocadherin gamma-A1	Q9Y5H4	PCDHGA1	NA	Inverse variance weighted	12	-0.022	0.079	7.852E-01	0.976	pan	Severe COVID-19

3583_54	Arylsulfatase A	Arylsulfatase A	P15289	ARSA	NA	Wald ratio	1	-0.050	0.183	7.856E-01	0.976	pan	Severe COVID-19
12450_42	PMVK	Phosphomevalonate kinase	Q15126	PMVK	NA	Inverse variance weighted	2	0.173	0.639	7.860E-01	0.976	pan	Severe COVID-19
18434_141	TBPL1	TATA box-binding protein-like protein 1	P62380	TBPL1	NA	Inverse variance weighted	3	-0.046	0.168	7.861E-01	0.976	pan	Severe COVID-19
15495_9	FOLR3	Folate receptor gamma	P41439	FOLR3	NA	Inverse variance weighted	15	-0.012	0.045	7.862E-01	0.976	pan	Severe COVID-19
8962_48	KS6B1	Ribosomal protein S6 kinase beta-1	P23443	RPS6KB1	NA	Inverse variance weighted	4	0.068	0.250	7.863E-01	0.976	pan	Severe COVID-19
16785_45	HD-5	Defensin-5	Q01523	DEFA5	NA	Inverse variance weighted	17	-0.015	0.056	7.865E-01	0.976	pan	Severe COVID-19
17748_21	QORX	Quinone oxidoreductase PIG3	Q53FA7	TP53I3	NA	Inverse variance weighted	10	0.015	0.056	7.871E-01	0.976	pan	Severe COVID-19
13118_5	SMOC1	SPARC-related modular calcium-binding protein 1	Q9H4F8	SMOC1	NA	Inverse variance weighted	3	-0.019	0.072	7.876E-01	0.976	pan	Severe COVID-19
18319_7	ODPX	Pyruvate dehydrogenase protein X component, mitochondrial	O00330	PDHX	NA	Wald ratio	1	-0.054	0.200	7.883E-01	0.976	pan	Severe COVID-19
4232_19	IGF-I sR	Insulin-like growth factor 1 receptor	P08069	IGF1R	NA	Inverse variance weighted	2	0.092	0.344	7.883E-01	0.976	pan	Severe COVID-19
9451_20	Uromodulin	Uromodulin	P07911	UMOD	NA	Wald ratio	1	0.103	0.385	7.884E-01	0.976	pan	Severe COVID-19
18387_7	suppression of tumorigenicity 13	Hsc70-interacting protein	P50502	ST13	NA	Wald ratio	1	-0.075	0.280	7.891E-01	0.976	pan	Severe COVID-19
4440_15	FCRL3	Fc receptor-like protein 3	Q96P31	FCRL3	NA	Inverse variance weighted	5	-0.028	0.103	7.891E-01	0.976	pan	Severe COVID-19
10974_20	ISK7	Serine protease inhibitor Kazal-type 7	P58062	SPINK7	NA	Inverse variance weighted	5	-0.030	0.112	7.894E-01	0.976	pan	Severe COVID-19
6470_19	fibulin 1	Fibulin-1	P23142	FBLN1	NA	Inverse variance weighted	4	0.030	0.114	7.895E-01	0.976	pan	Severe COVID-19
5939_42	TWEAK	Tumor necrosis factor ligand superfamily member 12	O43508	TNFSF12	NA	Inverse variance weighted	3	-0.062	0.233	7.896E-01	0.976	pan	Severe COVID-19
8866_53	QPCTL	Glutaminyl-peptide cyclotransferase-like protein	Q9NXS2	QPCTL	NA	Inverse variance weighted	7	-0.015	0.058	7.906E-01	0.976	pan	Severe COVID-19
9545_156	Granzyme K	Granzyme K	P49863	GZMK	NA	Inverse variance weighted	3	-0.033	0.123	7.906E-01	0.976	pan	Severe COVID-19
12934_1	HERC5	E3 ISG15--protein ligase HERC5	Q9UII4	HERC5	NA	Wald ratio	1	0.108	0.406	7.909E-01	0.976	pan	Severe COVID-19
17514_48	RAB21	Ras-related protein Rab-21	Q9UL25	RAB21	NA	Wald ratio	1	-0.042	0.160	7.911E-01	0.976	pan	Severe COVID-19
11187_11	CL12A	C-type lectin domain family 12 member A	Q5QGZ9	CLEC12A	NA	Inverse variance weighted	7	0.019	0.072	7.915E-01	0.976	pan	Severe COVID-19
11214_40	DNJB9	DnaJ homolog subfamily B member 9	Q9UBS3	DNAJB9	NA	Wald ratio	1	-0.077	0.290	7.916E-01	0.976	pan	Severe COVID-19
9211_19	PEDF	Pigment epithelium-derived factor	P36955	SERPINF1	NA	Inverse variance weighted	6	0.015	0.056	7.917E-01	0.976	pan	Severe COVID-19
5604_30	HPSE	Heparanase	Q9Y251	HPSE	NA	Inverse variance weighted	2	0.046	0.176	7.923E-01	0.977	pan	Severe COVID-19
14107_1	MTHFS	5-formyltetrahydrofolate cyclo-ligase	P49914	MTHFS	NA	Inverse variance weighted	6	-0.013	0.051	7.935E-01	0.977	pan	Severe COVID-19
4959_2	AGR2	Anterior gradient protein 2 homolog	O95994	AGR2	NA	Wald ratio	1	0.066	0.254	7.937E-01	0.977	pan	Severe COVID-19
9191_8	Trefoil factor 2	Trefoil factor 2	Q03403	TFF2	NA	Inverse variance weighted	4	-0.030	0.114	7.949E-01	0.977	pan	Severe COVID-19
15486_126	ABP1	Amiloride-sensitive amine oxidase [copper-containing]	P19801	AOC1	NA	Inverse variance weighted	12	-0.007	0.027	7.951E-01	0.977	pan	Severe COVID-19
2834_54	kallikrein 8	Kallikrein-8	O60259	KLK8	NA	Inverse variance weighted	2	-0.054	0.207	7.958E-01	0.977	pan	Severe COVID-19
4155_3	Tenascin	Tenascin	P24821	TNC	NA	Inverse variance weighted	12	-0.015	0.058	7.965E-01	0.977	pan	Severe COVID-19
13088_397	BTC	Betacellulin	P35070	BTC	NA	Inverse variance weighted	2	-0.023	0.089	7.966E-01	0.977	pan	Severe COVID-19
9197_4	LEG9	Galectin-9	O00182	LGALS9	NA	Wald ratio	1	-0.035	0.136	7.970E-01	0.977	pan	Severe COVID-19
3325_2	MATN2	Matrilin-2	O00339	MATN2	NA	Inverse variance weighted	4	0.027	0.107	7.978E-01	0.977	pan	Severe COVID-19
8428_102	NTRI	Neurotrimin	Q9P121	NTM	NA	Inverse variance weighted	4	-0.032	0.126	7.982E-01	0.977	pan	Severe COVID-19
17712_7	IDI1	Isopentenyl-diphosphate Delta-isomerase 1	Q13907	IDI1	NA	Wald ratio	1	0.055	0.214	7.983E-01	0.977	pan	Severe COVID-19
5061_27	B7-H2	ICOS ligand	O75144	ICOSLG	NA	Inverse variance weighted	2	0.094	0.368	7.990E-01	0.977	pan	Severe COVID-19
5599_88	PCYXL	Prenylcysteine oxidase-like	Q8NBM8	PCYOX1L	NA	Wald ratio	1	-0.054	0.213	7.992E-01	0.977	pan	Severe COVID-19
4971_1	CATZ	Cathepsin Z	Q9UBR2	CTSZ	NA	Inverse variance weighted	6	-0.012	0.047	7.993E-01	0.977	pan	Severe COVID-19
18244_1	ANXA7	Annexin A7	P20073	ANXA7	NA	Wald ratio	1	0.089	0.352	8.005E-01	0.978	pan	Severe COVID-19
8258_22	UXS1	UDP-glucuronic acid decarboxylase 1	Q8NBZ7	UXS1	NA	Inverse variance weighted	2	0.075	0.298	8.009E-01	0.978	pan	Severe COVID-19
8039_41	F177A	Protein FAM177A1	Q8N128	FAM177A1	NA	Inverse variance weighted	7	0.010	0.040	8.024E-01	0.979	pan	Severe COVID-19
4549_78	FUT5	Alpha-(1,3)-fucosyltransferase 5	Q11128	FUT5	NA	Inverse variance weighted	10	0.012	0.049	8.025E-01	0.979	pan	Severe COVID-19
9599_6	PIANP	PILR alpha-associated neural protein	Q8IYJ0	PIANP	NA	Wald ratio	1	-0.082	0.328	8.031E-01	0.979	pan	Severe COVID-19
10615_18	MYP0	Myelin protein P0	P25189	MPZ	NA	Inverse variance weighted	14	0.012	0.048	8.031E-01	0.979	pan	Severe COVID-19
4127_75	C6	Complement component C6	P13671	C6	NA	Inverse variance weighted	2	0.030	0.120	8.039E-01	0.979	pan	Severe COVID-19
19557_3	KLOTB	Beta-klotho	Q86Z14	KLB	NA	Inverse variance weighted	11	0.010	0.039	8.044E-01	0.979	pan	Severe COVID-19
6402_8	PILRA isoform FDF03-deltaTM	Paired immunoglobulin-like type 2 receptor alpha isoform FDF03-deltaTM	Q9UKJ1	PILRA	NA	Inverse variance weighted	14	-0.025	0.102	8.049E-01	0.979	pan	Severe COVID-19
5493_17	WNK3	Serine/threonine-protein kinase WNK3	Q9BYP7	WNK3	NA	Wald ratio	1	0.073	0.298	8.066E-01	0.980	pan	Severe COVID-19
2823_7	COMMD7	COMM domain-containing protein 7	Q86VX2	COMMD7	NA	Wald ratio	1	-0.048	0.197	8.066E-01	0.980	pan	Severe COVID-19
3825_18	MK08	Mitogen-activated protein kinase 8	P45983	MAPK8	NA	Inverse variance weighted	3	-0.032	0.131	8.068E-01	0.980	pan	Severe COVID-19
19197_95	THIL	Acetyl-CoA acetyltransferase, mitochondrial	P24752	ACAT1	NA	Inverse variance weighted	3	0.047	0.191	8.073E-01	0.980	pan	Severe COVID-19
13472_35	HDHD2	Haloacid dehalogenase-like hydrolase domain-containing protein 2	Q9H0R4	HDHD2	NA	Inverse variance weighted	3	0.020	0.083	8.082E-01	0.980	pan	Severe COVID-19
12334_25	cSHMT	Serine hydroxymethyltransferase, cytosolic	P34896	SHMT1	NA	Inverse variance weighted	12	0.008	0.034	8.102E-01	0.980	pan	Severe COVID-19
13114_50	Lumican	Lumican	P51884	LUM	NA	Inverse variance weighted	9	-0.011	0.046	8.119E-01	0.980	pan	Severe COVID-19
12517_52	TFAR19	Programmed cell death protein 5	O14737	PDCD5	NA	Inverse variance weighted	5	-0.026	0.112	8.136E-01	0.980	pan	Severe COVID-19
16307_22	UNC5H4	Netrin receptor UNC5D	Q6UXZ4	UNC5D	NA	Inverse variance weighted	3	-0.037	0.155	8.137E-01	0.980	pan	Severe COVID-19
9018_38	PCD10:ECD	Protocadherin-10:Extracellular domain	Q9P2E7	PCDH10	NA	Inverse variance weighted	3	0.020	0.084	8.140E-01	0.980	pan	Severe COVID-19
8780_2	PCD10:CD	Protocadherin-10:Cytoplasmic domain	Q9P2E7	PCDH10	NA	Inverse variance weighted	6	0.021	0.088	8.146E-01	0.980	pan	Severe COVID-19
12657_2	FCL	GDP-L-fucose synthase	Q13630	GFUS	NA	Wald ratio	1	-0.083	0.353	8.147E-01	0.980	pan	Severe COVID-19
9201_13	Transgelin-2	Transgelin-2	P37802	TAGLN2	NA	Wald ratio	1	0.077	0.330	8.154E-01	0.980	pan	Severe COVID-19
8024_64	TPST2	Protein-tyrosine sulfotransferase 2	O60704	TPST2	NA	Inverse variance weighted	2	0.030	0.129	8.159E-01	0.980	pan	Severe COVID-19
9940_35	DUS28	Dual specificity phosphatase 28	Q4G0W2	DUSP28	NA	Inverse variance weighted	3	0.056	0.242	8.160E-01	0.980	pan	Severe COVID-19
3903_49	Sorting nexin 4	Sorting nexin-4	O95219	SNX4	NA	Inverse variance weighted	2	-0.038	0.165	8.161E-01	0.980	pan	Severe COVID-19
5740_17	ROBO1	Roundabout homolog 1	Q9Y6N7	ROBO1	NA	Inverse variance weighted	6	0.019	0.080	8.163E-01	0.980	pan	Severe COVID-19
6390_18	NPS	Neuropeptide S	P0C0P6	NPS	NA	Inverse variance weighted	2	0.077	0.330	8.163E-01	0.980	pan	Severe COVID-19
5628_21	SEM3G	Semaphorin-3G	Q9NS98	SEMA3G	NA	Inverse variance weighted	6	-0.039	0.168	8.167E-01	0.980	pan	Severe COVID-19
15615_8	LIRB3	Leukocyte immunoglobulin-like receptor subfamily B member 3	O75022	LILRB3	NA	Inverse variance weighted	4	0.015	0.064	8.169E-01	0.980	pan	Severe COVID-19
10800_15	Collagen-binding protein	Serpin H1	P50454	SERPINH1	NA	Inverse variance weighted	4	0.026	0.114	8.172E-01	0.980	pan	Severe COVID-19
8221_19	MIF	Macrophage migration inhibitory factor	P14174	MIF	NA	Wald ratio	1	0.096	0.419	8.182E-01	0.980	pan	Severe COVID-19
18236_3	LEGL	Galectin-related protein	Q3ZCW2	LGALSL	NA	Wald ratio	1	0.045	0.194	8.184E-01	0.980	pan	Severe COVID-19
5363_51	Semaphorin 3E	Semaphorin-3E	O15041	SEMA3E	NA	Inverse variance weighted	24	-0.008	0.033	8.186E-01	0.980	pan	Severe COVID-19
7050_5	NEGR1	Neuronal growth regulator 1	Q7Z3B1	NEGR1	NA	Wald ratio	1	0.078	0.341	8.188E-01	0.980	pan	Severe COVID-19
6107_3	ELA1	Chymotrypsin-like elastase family member 1	Q9UNI1	CELA1	NA	Inverse variance weighted	13	-0.012	0.052	8.189E-01	0.980	pan	Severe COVID-19
18175_65	VAT1	Synaptic vesicle membrane protein VAT-1 homolog	Q99536	VAT1	NA	Inverse variance weighted	2	-0.050	0.218	8.190E-01	0.980	pan	Severe COVID-19
16763_11	LECT2	Leukocyte cell-derived chemotaxin-2	O14960	LECT2	NA	Inverse variance weighted	10	-0.011	0.048	8.193E-01	0.980	pan	Severe COVID-19
11369_23	ADHX	Alcohol dehydrogenase class-3	P11766	ADH5	NA	Inverse variance weighted	4	-0.050	0.218	8.199E-01	0.980	pan	Severe COVID-19
9880_33	T23O	Tryptophan 2,3-dioxygenase	P48775	TDO2	NA	Inverse variance weighted	7	-0.025	0.110	8.200E-01	0.980	pan	Severe COVID-19
8007_19	Cathepsin B	Cathepsin B	P07858	CTSB	NA	Inverse variance weighted	10	-0.012	0.054	8.216E-01	0.980	pan	Severe COVID-19

10714_7	ACE	Angiotensin-converting enzyme	P12821	ACE	NA	Inverse variance weighted	13	-0.006	0.025	8.229E-01	0.980	pan	Severe COVID-19
7208_60	MGT4C	Alpha-1,3-mannosyl-glycoprotein 4-beta-N-acetylglucosaminyltransferase C	Q9UBM8	MGAT4C	NA	Wald ratio	1	0.055	0.247	8.235E-01	0.980	pan	Severe COVID-19
19338_3	LZIC	Protein LZIC	Q8WZA0	LZIC	NA	Wald ratio	1	-0.098	0.442	8.242E-01	0.980	pan	Severe COVID-19
5586_66	MINP1	Multiple inositol polyphosphate phosphatase 1	Q9UNW1	MINPP1	NA	Inverse variance weighted	4	0.016	0.075	8.249E-01	0.980	pan	Severe COVID-19
13671_40	Elastase	Neutrophil elastase	P08246	ELANE	NA	Inverse variance weighted	12	0.025	0.116	8.257E-01	0.980	pan	Severe COVID-19
8019_73	STMN3	Stathmin-3	Q9NZ72	STMN3	NA	Wald ratio	1	-0.079	0.357	8.258E-01	0.980	pan	Severe COVID-19
8932_1	ENTP6	Ectonucleoside triphosphate diphosphohydrolase 6	O75354	ENTPD6	NA	Wald ratio	1	0.064	0.290	8.258E-01	0.980	pan	Severe COVID-19
17702_53	UGT 1A1	UDP-glucuronosyltransferase 1-1	P22309	UGT1A1	NA	Inverse variance weighted	3	-0.035	0.161	8.258E-01	0.980	pan	Severe COVID-19
12381_26	CBR1	Carbonyl reductase [NADPH] 1	P16152	CBR1	NA	Inverse variance weighted	5	-0.016	0.075	8.259E-01	0.980	pan	Severe COVID-19
3488_64	Catalase	Catalase	P04040	CAT	NA	Inverse variance weighted	4	-0.061	0.277	8.270E-01	0.980	pan	Severe COVID-19
9468_8	Lectin, mannose-binding 2	Vesicular integral-membrane protein VIP36	Q12907	LMAN2	NA	Wald ratio	1	0.039	0.176	8.271E-01	0.980	pan	Severe COVID-19
3855_56	Peroxiredoxin-1	Peroxiredoxin-1	Q06830	PRDX1	NA	Inverse variance weighted	7	0.022	0.100	8.275E-01	0.980	pan	Severe COVID-19
8960_3	LRAP	Endoplasmic reticulum aminopeptidase 2	Q6P179	ERAP2	NA	Inverse variance weighted	27	0.006	0.030	8.279E-01	0.980	pan	Severe COVID-19
6393_63	Endoplasmin	Endoplasmin	P14625	HSP90B1	NA	Inverse variance weighted	10	0.019	0.087	8.287E-01	0.980	pan	Severe COVID-19
3640_14	RAP	alpha-2-macroglobulin receptor-associated protein	P30533	LRPAP1	NA	Inverse variance weighted	10	0.011	0.051	8.290E-01	0.980	pan	Severe COVID-19
4930_21	Stanniocalcin-1	Stanniocalcin-1	P52823	STC1	NA	Inverse variance weighted	3	0.025	0.116	8.292E-01	0.980	pan	Severe COVID-19
18311_44	DCTN6	Dynactin subunit 6	O00399	DCTN6	NA	Inverse variance weighted	2	-0.037	0.171	8.300E-01	0.980	pan	Severe COVID-19
6431_68	PCYOX	Prenylcysteine oxidase 1	Q9UHG3	PCYOX1	NA	Inverse variance weighted	12	0.019	0.087	8.304E-01	0.980	pan	Severe COVID-19
12867_40	DYLT3	Dynein light chain Tctex-type 3	P51808	DYNLT3	NA	Wald ratio	1	-0.077	0.358	8.304E-01	0.980	pan	Severe COVID-19
18225_13	HEBP1	Heme-binding protein 1	Q9NRV9	HEBP1	NA	Inverse variance weighted	10	-0.006	0.027	8.308E-01	0.980	pan	Severe COVID-19
6570_1	CODA1	Collagen alpha-1(XIII) chain	Q5TAT6	COL13A1	NA	Inverse variance weighted	3	0.039	0.182	8.311E-01	0.980	pan	Severe COVID-19
14076_74	Cystatin-S	Cystatin-S	P01036	CST4	NA	Inverse variance weighted	10	0.014	0.064	8.323E-01	0.980	pan	Severe COVID-19
14100_63	C1QC	Complement C1q subcomponent subunit C	P02747	C1QC	NA	Inverse variance weighted	15	-0.011	0.054	8.325E-01	0.980	pan	Severe COVID-19
9118_7	PSD2	PH and SEC7 domain-containing protein 2	Q9BQI7	PSD2	NA	Inverse variance weighted	4	-0.040	0.193	8.345E-01	0.980	pan	Severe COVID-19
19236_24	TCP4	Activated RNA polymerase II transcriptional coactivator p15	P53999	SUB1	NA	Wald ratio	1	0.067	0.321	8.349E-01	0.980	pan	Severe COVID-19
13621_31	AP2A2	AP-2 complex subunit alpha-2	O94973	AP2A2	NA	Wald ratio	1	-0.050	0.241	8.355E-01	0.980	pan	Severe COVID-19
10075_75	ACBD6	Acyl-CoA-binding domain-containing protein 6	Q9BR61	ACBD6	NA	Wald ratio	1	0.073	0.349	8.357E-01	0.980	pan	Severe COVID-19
8957_72	XTP3B	Endoplasmic reticulum lectin 1	Q96DZ1	ERLEC1	NA	Wald ratio	1	0.045	0.221	8.372E-01	0.980	pan	Severe COVID-19
8334_65	TCP11	T-complex protein 11 homolog	Q8WWU5	TCP11	NA	Wald ratio	1	0.060	0.294	8.378E-01	0.980	pan	Severe COVID-19
15431_31	OTCase	Ornithine carbamoyltransferase, mitochondrial	P00480	OTC	NA	Inverse variance weighted	3	0.034	0.167	8.384E-01	0.980	pan	Severe COVID-19
6368_9	CST1L	Cystatin-like 1	Q9H114	CSTL1	NA	Wald ratio	1	-0.070	0.344	8.385E-01	0.980	pan	Severe COVID-19
5727_35	B3GLT	Beta-1,3-glucosyltransferase	Q6Y288	B3GLCT	NA	Inverse variance weighted	2	-0.018	0.089	8.386E-01	0.980	pan	Severe COVID-19
18213_30	UBE2F	NEDD8-conjugating enzyme UBE2F	Q969M7	UBE2F	NA	Inverse variance weighted	2	0.035	0.172	8.387E-01	0.980	pan	Severe COVID-19
18218_48	CB032	CB1 cannabinoid receptor-interacting protein 1	Q96F85	CNRIP1	NA	Inverse variance weighted	10	0.009	0.046	8.398E-01	0.980	pan	Severe COVID-19
4148_49	PAPP-A	Pappalysin-1	Q13219	PAPPA	NA	Inverse variance weighted	4	0.028	0.142	8.413E-01	0.980	pan	Severe COVID-19
18871_24	AIF1L	Allograft inflammatory factor 1-like	Q9BQI0	AIF1L	NA	Inverse variance weighted	7	-0.007	0.037	8.417E-01	0.980	pan	Severe COVID-19
12575_30	C1TC	C-1-tetrahydrofolate synthase, cytoplasmic	P11586	MTHFD1	NA	Inverse variance weighted	5	0.022	0.112	8.418E-01	0.980	pan	Severe COVID-19
6496_60	DLK1:ECD	Protein delta homolog 1:Extracellular domain	P80370	DLK1	NA	Inverse variance weighted	2	0.104	0.521	8.420E-01	0.980	pan	Severe COVID-19
5437_63	FABP	Fatty acid-binding protein, heart	P05413	FABP3	NA	Inverse variance weighted	2	0.047	0.235	8.429E-01	0.980	pan	Severe COVID-19
10037_98	SIG12:Ig-like V-type 2	Sialic acid-binding Ig-like lectin 12:Ig-like V-type 2 domain, Isoform long	Q96PQ1	SIGLEC12	NA	Inverse variance weighted	9	0.017	0.084	8.429E-01	0.980	pan	Severe COVID-19
13552_7	SWP70	Switch-associated protein 70	Q9UH65	SWAP70	NA	Inverse variance weighted	8	-0.007	0.033	8.429E-01	0.980	pan	Severe COVID-19
9511_61	NXPH2	Neurexophilin-2	O95156	NXPH2	NA	Inverse variance weighted	3	-0.074	0.375	8.429E-01	0.980	pan	Severe COVID-19
17683_2	COMD9	COMM domain-containing protein 9	Q9P000	COMMD9	NA	Wald ratio	1	-0.030	0.151	8.432E-01	0.980	pan	Severe COVID-19
3122_6	SMAC	Diablo homolog, mitochondrial	Q9NR28	DIABLO	NA	Wald ratio	1	-0.061	0.310	8.436E-01	0.980	pan	Severe COVID-19
15633_6	RBP	Retinol-binding protein 4	P02753	RBP4	NA	Wald ratio	1	0.053	0.272	8.445E-01	0.980	pan	Severe COVID-19
12348_46	SYSM	Serine--tRNA ligase, mitochondrial	Q9NP81	SARS2	NA	Wald ratio	1	0.041	0.210	8.451E-01	0.980	pan	Severe COVID-19
2665_26	BCMA	Tumor necrosis factor receptor superfamily member 17	Q02223	TNFRSF17	NA	Inverse variance weighted	11	-0.015	0.076	8.455E-01	0.980	pan	Severe COVID-19
12332_7	EF2K	Eukaryotic elongation factor 2 kinase	O00418	EEF2K	NA	Inverse variance weighted	2	0.098	0.507	8.470E-01	0.980	pan	Severe COVID-19
3419_49	CAMK2D	Calcium/calmodulin-dependent protein kinase type II subunit delta	Q13557	CAMK2D	NA	Wald ratio	1	0.064	0.334	8.484E-01	0.980	pan	Severe COVID-19
12714_38	AP1G2	AP-1 complex subunit gamma-like 2	O75843	AP1G2	NA	Inverse variance weighted	3	0.016	0.085	8.486E-01	0.980	pan	Severe COVID-19
17453_34	Ceruloplasmin	Ceruloplasmin	P00450	CP	NA	Inverse variance weighted	3	0.020	0.107	8.487E-01	0.980	pan	Severe COVID-19
15542_19	KCRU	Creatine kinase U-type, mitochondrial	P12532	CKMT1A	NA	Inverse variance weighted	9	-0.017	0.091	8.487E-01	0.980	pan	Severe COVID-19
5060_62	B7-H1	Programmed cell death 1 ligand 1	Q9NZQ7	CD274	NA	Inverse variance weighted	2	0.012	0.065	8.493E-01	0.980	pan	Severe COVID-19
18422_41	ERG19	Diphosphomevalonate decarboxylase	P53602	MVD	NA	Wald ratio	1	0.044	0.230	8.495E-01	0.980	pan	Severe COVID-19
5036_50	TSG-6	Tumor necrosis factor-inducible gene 6 protein	P98066	TNFAIP6	NA	Inverse variance weighted	22	-0.008	0.040	8.503E-01	0.980	pan	Severe COVID-19
4912_17	GOT1	Aspartate aminotransferase, cytoplasmic	P17174	GOT1	NA	Wald ratio	1	0.074	0.394	8.504E-01	0.980	pan	Severe COVID-19
19161_1	UBP15	Ubiquitin carboxyl-terminal hydrolase 15	Q9Y4E8	USP15	NA	Inverse variance weighted	2	-0.019	0.099	8.507E-01	0.980	pan	Severe COVID-19
13392_13	AT1B1	Sodium/potassium-transporting ATPase subunit beta-1	P05026	ATP1B1	NA	Inverse variance weighted	5	-0.014	0.074	8.514E-01	0.980	pan	Severe COVID-19
3364_76	Cathepsin V	Cathepsin L2	O60911	CTSV	NA	Inverse variance weighted	12	0.012	0.064	8.517E-01	0.980	pan	Severe COVID-19
15300_66	CC134	Coiled-coil domain-containing protein 134	Q9H6E4	CCDC134	NA	Wald ratio	1	0.026	0.139	8.519E-01	0.980	pan	Severe COVID-19
11207_3	Macrophage scavenger receptor:CD	Macrophage scavenger receptor types I and II:Cytoplasmic domain	P21757	MSR1	NA	Wald ratio	1	-0.121	0.650	8.525E-01	0.980	pan	Severe COVID-19
8069_85	CD3E	T-cell surface glycoprotein CD3 epsilon chain	P07766	CD3E	NA	Inverse variance weighted	3	-0.028	0.149	8.528E-01	0.980	pan	Severe COVID-19
5728_60	FCRL1	Fc receptor-like protein 1	Q96LA6	FCRL1	NA	Inverse variance weighted	10	0.017	0.090	8.537E-01	0.980	pan	Severe COVID-19
11266_8	SELPL:ECD	P-selectin glycoprotein ligand 1:Extracellular domain	Q14242	SELPLG	NA	Inverse variance weighted	12	0.008	0.041	8.543E-01	0.980	pan	Severe COVID-19
10852_114	HNRDL	Heterogeneous nuclear ribonucleoprotein D-like	O14979	HNRNPDL	NA	Wald ratio	1	0.051	0.278	8.546E-01	0.980	pan	Severe COVID-19
13116_25	CD177	CD177 antigen	Q8N6Q3	CD177	NA	Inverse variance weighted	10	-0.006	0.033	8.546E-01	0.980	pan	Severe COVID-19
19392_6	DDAH1	N(G),N(G)-dimethylarginine dimethylaminohydrolase 1	O94760	DDAH1	NA	Inverse variance weighted	12	-0.010	0.053	8.547E-01	0.980	pan	Severe COVID-19
2558_51	b-Endorphin	Beta-endorphin	P01189	POMC	NA	Wald ratio	1	0.055	0.302	8.547E-01	0.980	pan	Severe COVID-19
10558_26	PCDH9	Protocadherin-9	Q9HC56	PCDH9	NA	Inverse variance weighted	8	0.010	0.054	8.550E-01	0.980	pan	Severe COVID-19
12378_71	TPSN	Tapasin	O15533	TAPBP	NA	Inverse variance weighted	7	-0.011	0.058	8.551E-01	0.980	pan	Severe COVID-19
18930_28	SLIT2	Slit homolog 2 protein	O94813	SLIT2	NA	Inverse variance weighted	2	-0.024	0.129	8.552E-01	0.980	pan	Severe COVID-19
5034_79	Trypsin 2	Trypsin-2	P07478	PRSS2	NA	Inverse variance weighted	13	-0.013	0.074	8.559E-01	0.980	pan	Severe COVID-19
5404_53	DR6	Tumor necrosis factor receptor superfamily member 21	O75509	TNFRSF21	NA	Inverse variance weighted	2	-0.031	0.170	8.564E-01	0.980	pan	Severe COVID-19
19357_11	VAMP7	Vesicle-associated membrane protein 7	P51809	VAMP7	NA	Wald ratio	1	0.052	0.286	8.565E-01	0.980	pan	Severe COVID-19
2687_2	MIA	Melanoma-derived growth regulatory protein	Q16674	MIA	NA	Inverse variance weighted	7	-0.009	0.049	8.570E-01	0.980	pan	Severe COVID-19
14711_27	Cystatin M	Cystatin-M	Q15828	CST6	NA	Inverse variance weighted	7	0.017	0.092	8.570E-01	0.980	pan	Severe COVID-19
18899_82	HDGR3	Hepatoma-derived growth factor-related protein 3	Q9Y3E1	HDGFL3	NA	Inverse variance weighted	3	0.056	0.314	8.572E-01	0.980	pan	Severe COVID-19
17843_30	PPCS	Phosphopantothenate--cysteine ligase	Q9HAB8	PPCS	NA	Inverse variance weighted	2	0.027	0.152	8.573E-01	0.980	pan	Severe COVID-19

12709_63	H1X	Histone H1x	Q92522	H1-10	NA	Wald ratio	1	-0.078	0.434	8.575E-01	0.980	pan	Severe COVID-19
11218_84	TPMT	Thiopurine S-methyltransferase	P51580	TPMT	NA	Wald ratio	1	-0.116	0.650	8.583E-01	0.980	pan	Severe COVID-19
15453_3	a1-Microglobulin	Alpha-1-microglobulin	P02760	AMBP	NA	Wald ratio	1	0.046	0.259	8.589E-01	0.980	pan	Severe COVID-19
8274_64	Syntaxin-7	Syntaxin-7	O15400	STX7	NA	Wald ratio	1	-0.022	0.126	8.591E-01	0.980	pan	Severe COVID-19
4867_15	VEGF121	Vascular endothelial growth factor A, isoform 121	P15692	VEGFA	NA	Inverse variance weighted	8	-0.022	0.124	8.592E-01	0.980	pan	Severe COVID-19
4535_50	BST1	ADP-ribosyl cyclase/cyclic ADP-ribose hydrolase 2	Q10588	BST1	NA	Inverse variance weighted	17	-0.004	0.021	8.594E-01	0.980	pan	Severe COVID-19
2705_5	TECK	C-C motif chemokine 25	O15444	CCL25	NA	Inverse variance weighted	11	0.009	0.051	8.595E-01	0.980	pan	Severe COVID-19
19606_28	ihh	Indian hedgehog protein	Q14623	IHH	NA	Wald ratio	1	0.049	0.277	8.595E-01	0.980	pan	Severe COVID-19
2952_75	IGF-I	Insulin-like growth factor I	P05019	IGF1	NA	Inverse variance weighted	2	0.102	0.577	8.599E-01	0.980	pan	Severe COVID-19
5129_12	SREC-I	Scavenger receptor class F member 1	Q14162	SCARF1	NA	Inverse variance weighted	5	0.010	0.058	8.602E-01	0.980	pan	Severe COVID-19
3074_6	LBP	Lipopolysaccharide-binding protein	P18428	LBP	NA	Inverse variance weighted	10	0.013	0.076	8.613E-01	0.981	pan	Severe COVID-19
19206_20	8ODP	7,8-dihydro-8-oxoguanine triphosphatase	P36639	NUDT1	NA	Wald ratio	1	0.025	0.143	8.613E-01	0.981	pan	Severe COVID-19
5671_1	Chymotrypsin	Chymotrypsinogen B	P17538	CTRB1	NA	Inverse variance weighted	8	-0.013	0.074	8.620E-01	0.981	pan	Severe COVID-19
2828_82	HAI-1	Kunitz-type protease inhibitor 1	O43278	SPINT1	NA	Inverse variance weighted	10	-0.008	0.046	8.624E-01	0.981	pan	Severe COVID-19
9329_28	TRML1:ECD	Trem-like transcript 1 protein:Extracellular domain, Ig-like V-type domain	Q86YW5	TREML1	NA	Inverse variance weighted	2	-0.048	0.278	8.645E-01	0.983	pan	Severe COVID-19
13622_16	2A5A	Serine/threonine-protein phosphatase 2A 56 kDa regulatory subunit alpha isoform	Q15172	PPP2R5A	NA	Wald ratio	1	-0.061	0.359	8.659E-01	0.984	pan	Severe COVID-19
11516_7	FABPL	Fatty acid-binding protein, liver	P07148	FABP1	NA	Wald ratio	1	-0.023	0.135	8.669E-01	0.984	pan	Severe COVID-19
10647_18	ASCC1	Activating signal cointegrator 1 complex subunit 1	Q8N9N2	ASCC1	NA	Wald ratio	1	-0.054	0.322	8.680E-01	0.984	pan	Severe COVID-19
7266_4	SPA9	Serpin A9	Q86WD7	SERPINA9	NA	Wald ratio	1	0.033	0.202	8.693E-01	0.984	pan	Severe COVID-19
2474_54	SAP	Serum amyloid P-component	P02743	APCS	NA	Inverse variance weighted	7	-0.011	0.067	8.702E-01	0.984	pan	Severe COVID-19
18231_147	PRND	Prion-like protein doppel	Q9UKY0	PRND	NA	Wald ratio	1	0.047	0.290	8.703E-01	0.984	pan	Severe COVID-19
12411_60	MAX	Protein max	P61244	MAX	NA	Wald ratio	1	-0.014	0.085	8.707E-01	0.984	pan	Severe COVID-19
15322_35	CRADD	Death domain-containing protein CRADD	P78560	CRADD	NA	Inverse variance weighted	3	0.060	0.368	8.713E-01	0.984	pan	Severe COVID-19
9337_43	TKN1	Protachykinin-1	P20366	TAC1	NA	Inverse variance weighted	6	0.012	0.076	8.720E-01	0.984	pan	Severe COVID-19
3212_30	ASAH2	Neutral ceramidase	Q9NR71	ASAH2	NA	Inverse variance weighted	10	-0.008	0.052	8.722E-01	0.984	pan	Severe COVID-19
15483_377	Agrin	Agrin	O00468	AGRN	NA	Inverse variance weighted	12	-0.010	0.062	8.727E-01	0.984	pan	Severe COVID-19
8034_6	DRGX	Dorsal root ganglia homeobox protein	A6NNA5	DRGX	NA	Inverse variance weighted	7	0.008	0.048	8.734E-01	0.984	pan	Severe COVID-19
11102_22	REG4	Regenerating islet-derived protein 4	Q9BYZ8	REG4	NA	Inverse variance weighted	2	0.047	0.298	8.747E-01	0.984	pan	Severe COVID-19
13095_51	PSP	Lithostathine-1-alpha	P05451	REG1A	NA	Inverse variance weighted	7	-0.019	0.119	8.749E-01	0.984	pan	Severe COVID-19
4562_1	NXPH1	Neurexophilin-1	P58417	NXPH1	NA	Wald ratio	1	-0.074	0.472	8.753E-01	0.984	pan	Severe COVID-19
12855_16	CT032	Cas scaffolding protein family member 4	Q9NQ75	CASS4	NA	Wald ratio	1	0.045	0.285	8.757E-01	0.984	pan	Severe COVID-19
6649_51	NET1	Netrin-1	O95631	NTN1	NA	Inverse variance weighted	7	0.010	0.065	8.759E-01	0.984	pan	Severe COVID-19
4332_6	CLC1B	C-type lectin domain family 1 member B	Q9P126	CLEC1B	NA	Wald ratio	1	-0.033	0.211	8.763E-01	0.984	pan	Severe COVID-19
13565_2	p130	Retinoblastoma-like protein 2	Q08999	RBL2	NA	Inverse variance weighted	6	0.016	0.100	8.764E-01	0.984	pan	Severe COVID-19
9251_28	MA2B2	Epididymis-specific alpha-mannosidase	Q9Y2E5	MAN2B2	NA	Inverse variance weighted	10	-0.005	0.033	8.766E-01	0.984	pan	Severe COVID-19
3220_40	RET	Proto-oncogene tyrosine-protein kinase receptor Ret	P07949	RET	NA	Inverse variance weighted	5	0.008	0.054	8.771E-01	0.984	pan	Severe COVID-19
4220_39	FER	Tyrosine-protein kinase Fer	P16591	FER	NA	Inverse variance weighted	2	-0.026	0.166	8.772E-01	0.984	pan	Severe COVID-19
15376_134	CATE	Cathepsin E	P14091	CTSE	NA	Inverse variance weighted	5	0.008	0.053	8.773E-01	0.984	pan	Severe COVID-19
13534_20	MYOM2	Myomesin-2	P54296	MYOM2	NA	Inverse variance weighted	3	0.033	0.212	8.774E-01	0.984	pan	Severe COVID-19
3799_11	Carbonic anhydrase III	Carbonic anhydrase 3	P07451	CA3	NA	Inverse variance weighted	5	-0.013	0.085	8.778E-01	0.984	pan	Severe COVID-19
19558_10	LRP4	Low-density lipoprotein receptor-related protein 4	O75096	LRP4	NA	Inverse variance weighted	10	-0.008	0.053	8.781E-01	0.984	pan	Severe COVID-19
3007_7	Siglec-9	Sialic acid-binding Ig-like lectin 9	Q9Y336	SIGLEC9	NA	Inverse variance weighted	7	0.009	0.060	8.787E-01	0.984	pan	Severe COVID-19
6439_59	CN093	Uncharacterized protein C14orf93	Q9H972	C14orf93	NA	Wald ratio	1	-0.047	0.312	8.801E-01	0.984	pan	Severe COVID-19
7085_81	CDSN	Corneodesmosin	Q15517	CDSN	NA	Inverse variance weighted	7	0.017	0.112	8.805E-01	0.984	pan	Severe COVID-19
18313_4	Asparaginase-like protein 1	Isoaspartyl peptidase/L-asparaginase	Q7L266	ASRGL1	NA	Inverse variance weighted	4	0.009	0.063	8.809E-01	0.984	pan	Severe COVID-19
3435_53	FN1.4	Fibronectin Fragment 4	P02751	FN1	NA	Inverse variance weighted	2	0.057	0.387	8.825E-01	0.984	pan	Severe COVID-19
8397_147	QSOX2	Sulfhydryl oxidase 2	Q6ZRP7	QSOX2	NA	Inverse variance weighted	16	0.005	0.033	8.832E-01	0.984	pan	Severe COVID-19
2879_9	a1-Antichymotrypsin	Alpha-1-antichymotrypsin	P01011	SERPINA3	NA	Wald ratio	1	0.049	0.333	8.841E-01	0.984	pan	Severe COVID-19
6385_63	VWA1	von Willebrand factor A domain-containing protein 1	Q6PCB0	VWA1	NA	Inverse variance weighted	7	0.007	0.048	8.852E-01	0.984	pan	Severe COVID-19
4471_50	TGM3	Protein-glutamine gamma-glutamyltransferase E	Q08188	TGM3	NA	Inverse variance weighted	5	-0.006	0.039	8.866E-01	0.984	pan	Severe COVID-19
19154_41	Protease nexin I	Glia-derived nexin	P07093	SERPINE2	NA	Inverse variance weighted	13	-0.006	0.041	8.874E-01	0.984	pan	Severe COVID-19
7827_20	ADA32	Disintegrin and metalloproteinase domain-containing protein 32	Q8TC27	ADAM32	NA	Wald ratio	1	0.051	0.364	8.881E-01	0.984	pan	Severe COVID-19
16614_27	RSPO1	R-spondin-1	Q2MKA7	RSPO1	NA	Inverse variance weighted	3	0.010	0.074	8.886E-01	0.984	pan	Severe COVID-19
4297_62	Spondin-1	Spondin-1	Q9HCB6	SPON1	NA	Inverse variance weighted	4	-0.007	0.053	8.888E-01	0.984	pan	Severe COVID-19
3831_21	pTEN	Phosphatidylinositol 3,4,5-trisphosphate 3-phosphatase and dual-specificity protein phosphatase PTEN	P60484	PTEN	NA	Wald ratio	1	-0.051	0.367	8.889E-01	0.984	pan	Severe COVID-19
8697_38	Glypican 1	Glypican-1	P35052	GPC1	NA	Inverse variance weighted	14	-0.005	0.034	8.895E-01	0.984	pan	Severe COVID-19
6920_1	GFRAL	GDNF family receptor alpha-like	Q6UXV0	GFRAL	NA	Inverse variance weighted	6	0.010	0.069	8.900E-01	0.984	pan	Severe COVID-19
10490_3	RPN1:CD	Dolichyl-diphosphooligosaccharide--protein glycosyltransferase subunit 1:Cytoplasmic domain	P04843	RPN1	NA	Wald ratio	1	0.015	0.113	8.917E-01	0.984	pan	Severe COVID-19
2890_59	CCL28	C-C motif chemokine 28	Q9NRJ3	CCL28	NA	Inverse variance weighted	8	0.013	0.093	8.920E-01	0.984	pan	Severe COVID-19
9383_24	CH3L2	Chitinase-3-like protein 2	Q15782	CHI3L2	NA	Wald ratio	1	0.034	0.254	8.924E-01	0.984	pan	Severe COVID-19
16770_3	REG1B	Lithostathine-1-beta	P48304	REG1B	NA	Inverse variance weighted	4	-0.025	0.183	8.928E-01	0.984	pan	Severe COVID-19
4133_54	Granzyme B	Granzyme B	P10144	GZMB	NA	Wald ratio	1	-0.022	0.162	8.931E-01	0.984	pan	Severe COVID-19
3040_59	MIP-1a	C-C motif chemokine 3	P10147	CCL3	NA	Wald ratio	1	-0.041	0.304	8.931E-01	0.984	pan	Severe COVID-19
7144_234	KAZD1	Kazal-type serine protease inhibitor domain-containing protein 1	Q96I82	KAZALD1	NA	Wald ratio	1	0.036	0.267	8.939E-01	0.984	pan	Severe COVID-19
15481_45	LL-37	Antibacterial protein LL-37	P49913	CAMP	NA	Inverse variance weighted	10	0.012	0.093	8.946E-01	0.984	pan	Severe COVID-19
5134_52	TIMD3	Hepatitis A virus cellular receptor 2	Q8TDQ0	HAVCR2	NA	Inverse variance weighted	9	0.006	0.047	8.947E-01	0.984	pan	Severe COVID-19
8969_49	CD14	Monocyte differentiation antigen CD14	P08571	CD14	NA	Wald ratio	1	0.062	0.469	8.954E-01	0.984	pan	Severe COVID-19
13388_57	NEC1	Neuroendocrine convertase 1	P29120	PCSK1	NA	Inverse variance weighted	11	-0.007	0.051	8.957E-01	0.984	pan	Severe COVID-19
3033_57	Galectin-2	Galectin-2	P05162	LGALS2	NA	Inverse variance weighted	3	0.011	0.087	8.961E-01	0.984	pan	Severe COVID-19
8469_41	IGFBP-2	Insulin-like growth factor-binding protein 2	P18065	IGFBP2	NA	Inverse variance weighted	4	0.021	0.161	8.964E-01	0.984	pan	Severe COVID-19
10462_14	INSL5	Insulin-like peptide INSL5	Q9Y5Q6	INSL5	NA	Wald ratio	1	-0.034	0.263	8.969E-01	0.984	pan	Severe COVID-19
5131_15	TAJ	Tumor necrosis factor receptor superfamily member 19	Q9NS68	TNFRSF19	NA	Wald ratio	1	-0.020	0.155	8.972E-01	0.984	pan	Severe COVID-19
11441_11	PYGL	Glycogen phosphorylase, liver form	P06737	PYGL	NA	Inverse variance weighted	7	0.012	0.095	8.981E-01	0.984	pan	Severe COVID-19
6247_9	SIRB1	Signal-regulatory protein beta-1	O00241	SIRPB1	NA	Inverse variance weighted	9	-0.003	0.020	8.982E-01	0.984	pan	Severe COVID-19
10781_19	CLC4G	C-type lectin domain family 4 member G	Q6UXB4	CLEC4G	NA	Wald ratio	1	-0.047	0.371	8.982E-01	0.984	pan	Severe COVID-19
10366_11	PDGFRA	Platelet-derived growth factor receptor alpha	P16234	PDGFRA	NA	Inverse variance weighted	10	0.009	0.072	8.985E-01	0.984	pan	Severe COVID-19
6086_15	CRDL2	Chordin-like protein 2	Q6WN34	CHRDL2	NA	Inverse variance weighted	6	-0.015	0.114	8.985E-01	0.984	pan	Severe COVID-19
10584_7	NDUS4	NADH dehydrogenase [ubiquinone] iron-sulfur protein 4, mitochondrial	O43181	NDUFS4	NA	Inverse variance weighted	20	-0.010	0.076	8.988E-01	0.984	pan	Severe COVID-19

13634_209	PIR	Pirin	O00625	PIR	NA	Wald ratio	1	-0.038	0.297	8.993E-01	0.984	pan	Severe COVID-19
4834_61	Epithelial cell kinase	Ephrin type-A receptor 2	P29317	EPHA2	NA	Inverse variance weighted	5	0.010	0.081	8.996E-01	0.984	pan	Severe COVID-19
12697_30	PI42A	Phosphatidylinositol 5-phosphate 4-kinase type-2 alpha	P48426	PIP4K2A	NA	Wald ratio	1	0.039	0.311	8.999E-01	0.984	pan	Severe COVID-19
15487_164	carboxylesterase, liver	Liver carboxylesterase 1	P23141	CES1	NA	Wald ratio	1	0.039	0.311	8.999E-01	0.984	pan	Severe COVID-19
9449_150	C4b-binding protein alpha chain	C4b-binding protein alpha chain	P04003	C4BPA	NA	Inverse variance weighted	5	-0.018	0.144	9.004E-01	0.984	pan	Severe COVID-19
3210_1	METAP1	Methionine aminopeptidase 1	P53582	METAP1	NA	Wald ratio	1	-0.050	0.402	9.005E-01	0.984	pan	Severe COVID-19
5346_24	CPNE1:C2, 1 and 2	Copine-1:Ca2+-dependent membrane-targeting module domains 1 and 2	Q99829	CPNE1	NA	Inverse variance weighted	12	-0.006	0.051	9.008E-01	0.984	pan	Severe COVID-19
17794_6	Phosphomannomutase 2	Phosphomannomutase 2	O15305	PMM2	NA	Inverse variance weighted	5	0.013	0.108	9.012E-01	0.984	pan	Severe COVID-19
13553_4	DCNL3	DCN1-like protein 3	Q8IWE4	DCUN1D3	NA	Wald ratio	1	-0.043	0.351	9.014E-01	0.984	pan	Severe COVID-19
7866_11	DJC30	DnaJ homolog subfamily C member 30	Q96LL9	DNAJC30	NA	Inverse variance weighted	2	-0.012	0.097	9.016E-01	0.984	pan	Severe COVID-19
6584_1	SRCH	Sarcoplasmic reticulum histidine-rich calcium-binding protein	P23327	HRC	NA	Wald ratio	1	-0.038	0.306	9.016E-01	0.984	pan	Severe COVID-19
15539_15	SLIK1	SLIT and NTRK-like protein 1	Q96PX8	SLITRK1	NA	Wald ratio	1	-0.028	0.225	9.018E-01	0.984	pan	Severe COVID-19
11573_3	SRSF6	Serine/arginine-rich splicing factor 6	Q13247	SRSF6	NA	Wald ratio	1	-0.036	0.289	9.019E-01	0.984	pan	Severe COVID-19
17836_17	S100A16	Protein S100-A16	Q96FQ6	S100A16	NA	Wald ratio	1	-0.030	0.244	9.023E-01	0.984	pan	Severe COVID-19
16594_44	FAIM1	Fas apoptotic inhibitory molecule 1	Q9NVQ4	FAIM	NA	Inverse variance weighted	10	-0.005	0.045	9.027E-01	0.984	pan	Severe COVID-19
13744_37	IL-3 Ra	Interleukin-3 receptor subunit alpha	P26951	IL3RA	NA	Inverse variance weighted	3	0.021	0.168	9.027E-01	0.984	pan	Severe COVID-19
17761_2	NUDT5	ADP-sugar pyrophosphatase	Q9UKK9	NUDT5	NA	Inverse variance weighted	2	-0.023	0.190	9.030E-01	0.984	pan	Severe COVID-19
17765_3	SPN1	Snurportin-1	O95149	SNUPN	NA	Wald ratio	1	0.014	0.113	9.031E-01	0.984	pan	Severe COVID-19
15521_4	Alcadein alpha-1	Calsyntenin-1	O94985	CLSTN1	NA	Inverse variance weighted	7	-0.003	0.022	9.039E-01	0.985	pan	Severe COVID-19
5980_55	BOLA3	BolA-like protein 3	Q53S33	BOLA3	NA	Wald ratio	1	0.015	0.126	9.058E-01	0.985	pan	Severe COVID-19
17819_30	FAHD1	Acylpyruvase FAHD1, mitochondrial	Q6P587	FAHD1	NA	Inverse variance weighted	4	-0.016	0.136	9.062E-01	0.985	pan	Severe COVID-19
11257_1	DHPR	Dihydropteridine reductase	P09417	QDPR	NA	Inverse variance weighted	6	-0.005	0.043	9.062E-01	0.985	pan	Severe COVID-19
15644_1	Biotinidase	Biotinidase	P43251	BTD	NA	Inverse variance weighted	9	0.004	0.032	9.066E-01	0.985	pan	Severe COVID-19
17764_108	RHOC	Rho-related GTP-binding protein RhoC	P08134	RHOC	NA	Inverse variance weighted	2	-0.015	0.131	9.072E-01	0.985	pan	Severe COVID-19
4775_34	Gelsolin	Gelsolin	P06396	GSN	NA	Inverse variance weighted	3	0.013	0.115	9.076E-01	0.985	pan	Severe COVID-19
9470_15	MET24	Methyltransferase-like protein 24	Q5JXM2	METTL24	NA	Inverse variance weighted	5	0.012	0.107	9.081E-01	0.985	pan	Severe COVID-19
17675_17	ACO13	Acyl-coenzyme A thioesterase 13	Q9NPJ3	ACOT13	NA	Wald ratio	1	-0.034	0.298	9.081E-01	0.985	pan	Severe COVID-19
7096_30	RMD1	Regulator of microtubule dynamics protein 1	Q96DB5	RMDN1	NA	Inverse variance weighted	5	-0.009	0.078	9.081E-01	0.985	pan	Severe COVID-19
5139_32	UNC5H3	Netrin receptor UNC5C	O95185	UNC5C	NA	Inverse variance weighted	3	0.022	0.188	9.087E-01	0.985	pan	Severe COVID-19
5687_5	GLU2B	Glucosidase 2 subunit beta	P14314	PRKCSH	NA	Inverse variance weighted	3	-0.020	0.173	9.092E-01	0.985	pan	Severe COVID-19
16593_3	FADD	FAS-associated death domain protein	Q13158	FADD	NA	Inverse variance weighted	2	-0.034	0.295	9.094E-01	0.985	pan	Severe COVID-19
4276_10	prostatic binding protein	Phosphatidylethanolamine-binding protein 1	P30086	PEBP1	NA	Inverse variance weighted	10	-0.005	0.042	9.103E-01	0.985	pan	Severe COVID-19
8465_52	Cathepsin H	Cathepsin H	P09668	CTSH	NA	Inverse variance weighted	17	-0.003	0.029	9.108E-01	0.985	pan	Severe COVID-19
13476_16	KIN17	DNA/RNA-binding protein KIN17	O60870	KIN	NA	Wald ratio	1	0.032	0.291	9.136E-01	0.985	pan	Severe COVID-19
5708_1	LEAP2	Liver-expressed antimicrobial peptide 2	Q969E1	LEAP2	NA	Inverse variance weighted	5	-0.006	0.054	9.137E-01	0.985	pan	Severe COVID-19
9267_2	CBPA4	Carboxypeptidase A4	Q9UI42	CPA4	NA	Inverse variance weighted	21	-0.002	0.017	9.144E-01	0.985	pan	Severe COVID-19
18222_34	SHLB2	Endophilin-B2	Q9NR46	SH3GLB2	NA	Inverse variance weighted	3	-0.006	0.054	9.146E-01	0.985	pan	Severe COVID-19
13536_56	POLI	DNA polymerase iota	Q9UNA4	POLI	NA	Wald ratio	1	0.021	0.198	9.152E-01	0.985	pan	Severe COVID-19
10832_24	B4GT6	Beta-1,4-galactosyltransferase 6	Q9UBX8	B4GALT6	NA	Inverse variance weighted	12	-0.003	0.032	9.153E-01	0.985	pan	Severe COVID-19
14684_17	CAN2	Calpain-2 catalytic subunit	P17655	CAPN2	NA	Inverse variance weighted	2	0.024	0.229	9.161E-01	0.985	pan	Severe COVID-19
16872_248	MAAI	Maleylacetoacetate isomerase	O43708	GSTZ1	NA	Inverse variance weighted	12	-0.003	0.025	9.163E-01	0.985	pan	Severe COVID-19
9719_145	MMP-16	Matrix metalloproteinase-16	P51512	MMP16	NA	Inverse variance weighted	2	0.048	0.462	9.173E-01	0.985	pan	Severe COVID-19
4548_4	Fucosyltransferase 3	Galactoside 3(4)-L-fucosyltransferase	P21217	FUT3	NA	Inverse variance weighted	7	0.008	0.082	9.183E-01	0.985	pan	Severe COVID-19
19158_1	PAF	PCNA-associated factor	Q15004	PCLAF	NA	Inverse variance weighted	5	0.014	0.134	9.189E-01	0.985	pan	Severe COVID-19
15387_44	Neuropilin-2	Neuropilin-2	O60462	NRP2	NA	Inverse variance weighted	6	-0.011	0.112	9.199E-01	0.985	pan	Severe COVID-19
14150_7	IL-1F6	Interleukin-36 alpha	Q9UHA7	IL36A	NA	Inverse variance weighted	2	-0.017	0.167	9.208E-01	0.985	pan	Severe COVID-19
18432_32	RALB	Ras-related protein Ral-B	P11234	RALB	NA	Wald ratio	1	-0.025	0.255	9.210E-01	0.985	pan	Severe COVID-19
13934_3	MCF2L	Guanine nucleotide exchange factor DBS	O15068	MCF2L	NA	Inverse variance weighted	2	0.007	0.069	9.211E-01	0.985	pan	Severe COVID-19
2985_35	Gro-a	Growth-regulated alpha protein	P09341	CXCL1	NA	Inverse variance weighted	13	-0.004	0.044	9.213E-01	0.985	pan	Severe COVID-19
8971_9	NRX1A	Neurexin-1	Q9ULB1	NRXN1	NA	Inverse variance weighted	6	0.014	0.138	9.213E-01	0.985	pan	Severe COVID-19
12847_27	MCE1	mRNA-capping enzyme	O60942	RNGTT	NA	Wald ratio	1	-0.051	0.519	9.219E-01	0.985	pan	Severe COVID-19
5076_53	EPHAA	Ephrin type-A receptor 10	Q5JZY3	EPHA10	NA	Wald ratio	1	0.020	0.209	9.226E-01	0.985	pan	Severe COVID-19
2816_50	BCAM	Basal Cell Adhesion Molecule	P50895	BCAM	NA	Inverse variance weighted	4	0.012	0.122	9.228E-01	0.985	pan	Severe COVID-19
9478_69	KPRA	Phosphoribosyl pyrophosphate synthase-associated protein 1	Q14558	PRPSAP1	NA	Wald ratio	1	-0.027	0.278	9.234E-01	0.985	pan	Severe COVID-19
18215_5	THG1	Probable tRNA(His) guanylyltransferase	Q9NWX6	THG1L	NA	Inverse variance weighted	8	-0.004	0.039	9.243E-01	0.985	pan	Severe COVID-19
8973_23	FCRL4:ECD	Fc receptor-like protein 4:Extracellular domain	Q96PJ5	FCRL4	NA	Inverse variance weighted	19	-0.004	0.042	9.246E-01	0.985	pan	Severe COVID-19
11215_6	CAD15:CD	Cadherin-15:Cytoplasmic domain	P55291	CDH15	NA	Inverse variance weighted	2	0.021	0.223	9.246E-01	0.985	pan	Severe COVID-19
18162_167	IRAK4	Interleukin-1 receptor-associated kinase 4	Q9NWZ3	IRAK4	NA	Wald ratio	1	-0.018	0.190	9.249E-01	0.985	pan	Severe COVID-19
10907_116	NTRI	Neurotrimin	Q9P121	NTM	NA	Inverse variance weighted	3	-0.018	0.189	9.254E-01	0.985	pan	Severe COVID-19
9842_2	b-Catenin	Catenin beta-1	P35222	CTNNB1	NA	Inverse variance weighted	10	-0.003	0.037	9.254E-01	0.985	pan	Severe COVID-19
9061_3	ARHGA	Rho guanine nucleotide exchange factor 10	O15013	ARHGEF10	NA	Wald ratio	1	-0.020	0.210	9.255E-01	0.985	pan	Severe COVID-19
3617_80	HGFA	Hepatocyte growth factor activator	Q04756	HGFAC	NA	Inverse variance weighted	14	-0.003	0.037	9.264E-01	0.985	pan	Severe COVID-19
19241_31	RBP-III	Retinol-binding protein 5	P82980	RBP5	NA	Wald ratio	1	-0.028	0.303	9.264E-01	0.985	pan	Severe COVID-19
13544_9	HMHA1	Rho GTPase-activating protein 45	Q92619	ARHGAP45	NA	Wald ratio	1	0.015	0.160	9.271E-01	0.985	pan	Severe COVID-19
14708_59	CO8G	Complement component C8 gamma chain	P07360	C8G	NA	Inverse variance weighted	10	-0.008	0.094	9.279E-01	0.985	pan	Severe COVID-19
15414_316	LDHA	L-lactate dehydrogenase A chain	P00338	LDHA	NA	Wald ratio	1	0.024	0.271	9.282E-01	0.985	pan	Severe COVID-19
9313_27	CBLN1	Cerebellin-1	P23435	CBLN1	NA	Inverse variance weighted	30	-0.003	0.035	9.293E-01	0.985	pan	Severe COVID-19
3322_52	LRIG3	Leucine-rich repeats and immunoglobulin-like domains protein 3	Q6UXM1	LRIG3	NA	Inverse variance weighted	9	-0.004	0.041	9.300E-01	0.985	pan	Severe COVID-19
12646_2	RPE	Ribulose-phosphate 3-epimerase	Q96AT9	RPE	NA	Wald ratio	1	-0.016	0.185	9.301E-01	0.985	pan	Severe COVID-19
8080_24	PSMP	Prostate-associated microseminoprotein	Q1L6U9	MSMP	NA	Inverse variance weighted	15	-0.006	0.066	9.304E-01	0.985	pan	Severe COVID-19
9241_40	SIRPG	Signal-regulatory protein gamma	Q9P1W8	SIRPG	NA	Wald ratio	1	-0.021	0.241	9.305E-01	0.985	pan	Severe COVID-19
16609_106	KIRR2	Kin of IRRE-like protein 2	Q6UWL6	KIRREL2	NA	Inverse variance weighted	9	-0.004	0.040	9.311E-01	0.985	pan	Severe COVID-19
2278_61	TIMP-2	Metalloproteinase inhibitor 2	P16035	TIMP2	NA	Wald ratio	1	0.019	0.214	9.311E-01	0.985	pan	Severe COVID-19
14254_27	PTN4	Tyrosine-protein phosphatase non-receptor type 4	P29074	PTPN4	NA	Inverse variance weighted	4	0.015	0.172	9.312E-01	0.985	pan	Severe COVID-19
10612_18	PLOD3	Procollagen-lysine,2-oxoglutarate 5-dioxygenase 3	O60568	PLOD3	NA	Inverse variance weighted	7	0.009	0.106	9.312E-01	0.985	pan	Severe COVID-19
12357_41	SNP29	Synaptosomal-associated protein 29	O95721	SNAP29	NA	Wald ratio	1	0.028	0.322	9.314E-01	0.985	pan	Severe COVID-19
10521_10	MXRA8:ECD	Matrix-remodeling-associated protein 8:Extracellular domain	Q9BRK3	MXRA8	NA	Inverse variance weighted	3	0.011	0.129	9.318E-01	0.985	pan	Severe COVID-19
3326_58	Nectin-like protein 2	Cell adhesion molecule 1	Q9BY67	CADM1	NA	Wald ratio	1	-0.009	0.110	9.320E-01	0.985	pan	Severe COVID-19

3340_53	TSP4	Thrombospondin-4	P35443	THBS4	NA	Inverse variance weighted	4	-0.008	0.099	9.322E-01	0.985	pan	Severe COVID-19
2771_35	IGFBP-1	Insulin-like growth factor-binding protein 1	P08833	IGFBP1	NA	Inverse variance weighted	2	0.014	0.171	9.325E-01	0.985	pan	Severe COVID-19
17726_3	SAR1A	GTP-binding protein SAR1a	Q9NR31	SAR1A	NA	Inverse variance weighted	2	-0.011	0.133	9.336E-01	0.985	pan	Severe COVID-19
18396_10	AES	Amino-terminal enhancer of split	Q08117	TLE5	NA	Wald ratio	1	0.025	0.302	9.342E-01	0.985	pan	Severe COVID-19
12593_33	PDRG1	p53 and DNA damage-regulated protein 1	Q9NUG6	PDRG1	NA	Wald ratio	1	0.023	0.279	9.344E-01	0.985	pan	Severe COVID-19
8268_98	HS3SA	Heparan sulfate glucosamine 3-O-sulfotransferase 3A1	Q9Y663	HS3ST3A1	NA	Inverse variance weighted	2	0.027	0.331	9.346E-01	0.985	pan	Severe COVID-19
10445_20	ApoM	Apolipoprotein M	O95445	APOM	NA	Inverse variance weighted	2	0.030	0.367	9.347E-01	0.985	pan	Severe COVID-19
10372_18	STAT6	Signal transducer and activator of transcription 6	P42226	STAT6	NA	Wald ratio	1	0.018	0.220	9.350E-01	0.985	pan	Severe COVID-19
9288_7	FKBP7	Peptidyl-prolyl cis-trans isomerase FKBP7	Q9Y680	FKBP7	NA	Inverse variance weighted	7	-0.004	0.046	9.350E-01	0.985	pan	Severe COVID-19
8275_31	PEAR1:ECD	Platelet endothelial aggregation receptor 1:Extracellular domain	Q5VY43	PEAR1	NA	Inverse variance weighted	3	0.007	0.089	9.368E-01	0.985	pan	Severe COVID-19
8245_27	sICAM-5	Intercellular adhesion molecule 5	Q9UMF0	ICAM5	NA	Inverse variance weighted	6	-0.010	0.133	9.374E-01	0.985	pan	Severe COVID-19
8398_277	RAR-responsive protein TIG1	Retinoic acid receptor responder protein 1	P49788	RARRES1	NA	Inverse variance weighted	16	-0.001	0.016	9.376E-01	0.985	pan	Severe COVID-19
11178_21	SVEP1:EGF-like domains 4-6	Sushi, von Willebrand factor type A, EGF and pentraxin domain-containing protein 1:EGF-like domains 4-6	Q4LDE5	SVEP1	NA	Inverse variance weighted	5	-0.011	0.145	9.381E-01	0.985	pan	Severe COVID-19
15626_223	Perlecan	Basement membrane-specific heparan sulfate proteoglycan core protein	P98160	HSPG2	NA	Inverse variance weighted	6	0.005	0.060	9.381E-01	0.985	pan	Severe COVID-19
13133_73	LTBP4	Latent-transforming growth factor beta-binding protein 4	Q8N2S1	LTBP4	NA	Inverse variance weighted	2	0.027	0.358	9.395E-01	0.985	pan	Severe COVID-19
17706_4	PPR1A	Protein phosphatase 1 regulatory subunit 1A	Q13522	PPP1R1A	NA	Wald ratio	1	-0.022	0.287	9.400E-01	0.985	pan	Severe COVID-19
13704_5	HMCS2	Hydroxymethylglutaryl-CoA synthase, mitochondrial	P54868	HMGCS2	NA	Inverse variance weighted	2	-0.024	0.316	9.406E-01	0.985	pan	Severe COVID-19
17808_37	NIT2	Omega-amidase NIT2	Q9NQR4	NIT2	NA	Inverse variance weighted	7	0.014	0.190	9.425E-01	0.985	pan	Severe COVID-19
16324_38	TLR1:ECD	Toll-like receptor 1:Extracellular domain	Q15399	TLR1	NA	Inverse variance weighted	2	0.025	0.345	9.430E-01	0.985	pan	Severe COVID-19
9235_3	PXDC1	Plexin domain-containing protein 1	Q8IUK5	PLXDC1	NA	Inverse variance weighted	6	0.006	0.078	9.433E-01	0.985	pan	Severe COVID-19
9759_13	INDO	Indoleamine 2,3-dioxygenase 1	P14902	IDO1	NA	Wald ratio	1	0.018	0.254	9.433E-01	0.985	pan	Severe COVID-19
8974_172	COFA1	Collagen alpha-1(XV) chain	P39059	COL15A1	NA	Inverse variance weighted	7	0.005	0.067	9.448E-01	0.985	pan	Severe COVID-19
15570_99	Complement receptor type 2	Complement receptor type 2	P20023	CR2	NA	Inverse variance weighted	16	-0.004	0.060	9.448E-01	0.985	pan	Severe COVID-19
3484_60	Angiotensinogen	Angiotensinogen	P01019	AGT	NA	Inverse variance weighted	3	0.005	0.078	9.449E-01	0.985	pan	Severe COVID-19
17231_1	L-plastin	Plastin-2	P13796	LCP1	NA	Wald ratio	1	0.018	0.266	9.452E-01	0.985	pan	Severe COVID-19
16057_6	IGF-II receptor	Cation-independent mannose-6-phosphate receptor	P11717	IGF2R	NA	Inverse variance weighted	8	-0.003	0.042	9.458E-01	0.985	pan	Severe COVID-19
4413_3	SLPI	Antileukoproteinase	P03973	SLPI	NA	Wald ratio	1	-0.022	0.319	9.459E-01	0.985	pan	Severe COVID-19
10902_53	APRV1	Retroviral-like aspartic protease 1	Q53RT3	ASPRV1	NA	Inverse variance weighted	4	-0.010	0.153	9.460E-01	0.985	pan	Severe COVID-19
13748_4	MCP-2	C-C motif chemokine 8	P80075	CCL8	NA	Inverse variance weighted	10	0.006	0.088	9.461E-01	0.985	pan	Severe COVID-19
8660_5	OLFL3	Olfactomedin-like protein 3	Q9NRN5	OLFML3	NA	Inverse variance weighted	5	-0.008	0.117	9.464E-01	0.985	pan	Severe COVID-19
11377_19	ADH7	Alcohol dehydrogenase class 4 mu/sigma chain	P40394	ADH7	NA	Inverse variance weighted	3	-0.004	0.052	9.468E-01	0.985	pan	Severe COVID-19
3554_24	Adiponectin	Adiponectin	Q15848	ADIPOQ	NA	Inverse variance weighted	15	0.004	0.063	9.471E-01	0.985	pan	Severe COVID-19
6379_62	ATL2	ADAMTS-like protein 2	Q86TH1	ADAMTSL2	NA	Inverse variance weighted	2	-0.023	0.354	9.472E-01	0.985	pan	Severe COVID-19
19370_30	HS3S4	Heparan sulfate glucosamine 3-O-sulfotransferase 4	Q9Y661	HS3ST4	NA	Wald ratio	1	0.015	0.232	9.477E-01	0.985	pan	Severe COVID-19
5508_62	Cathepsin D	Cathepsin D	P07339	CTSD	NA	Inverse variance weighted	6	0.006	0.093	9.494E-01	0.985	pan	Severe COVID-19
8356_88	NEU1	Oxytocin-neurophysin 1	P01178	OXT	NA	Inverse variance weighted	4	-0.005	0.085	9.495E-01	0.985	pan	Severe COVID-19
4913_78	HCC-4	C-C motif chemokine 16	O15467	CCL16	NA	Inverse variance weighted	8	-0.006	0.087	9.497E-01	0.985	pan	Severe COVID-19
5688_65	CBLN4	Cerebellin-4	Q9NTU7	CBLN4	NA	Inverse variance weighted	6	-0.006	0.089	9.498E-01	0.985	pan	Severe COVID-19
10428_1	KI2S2	Killer cell immunoglobulin-like receptor 2DS2	P43631	KIR2DS2	NA	Inverse variance weighted	9	0.005	0.083	9.499E-01	0.985	pan	Severe COVID-19
19141_22	DAP1	Death-associated protein 1	P51397	DAP	NA	Inverse variance weighted	7	0.006	0.099	9.500E-01	0.985	pan	Severe COVID-19
16823_75	APOL3	Apolipoprotein L3	O95236	APOL3	NA	Inverse variance weighted	8	-0.002	0.033	9.503E-01	0.985	pan	Severe COVID-19
8958_51	CHL1	Neural cell adhesion molecule L1-like protein	O00533	CHL1	NA	Inverse variance weighted	8	0.005	0.085	9.507E-01	0.985	pan	Severe COVID-19
12689_56	ARC1B	Actin-related protein 2/3 complex subunit 1B	O15143	ARPC1B	NA	Wald ratio	1	0.011	0.177	9.511E-01	0.985	pan	Severe COVID-19
9995_6	DUT	Deoxyuridine 5'-triphosphate nucleotidohydrolase, mitochondrial	P33316	DUT	NA	Wald ratio	1	0.014	0.239	9.527E-01	0.985	pan	Severe COVID-19
17404_5	ARL5B	ADP-ribosylation factor-like protein 5B	Q96KC2	ARL5B	NA	Wald ratio	1	0.025	0.422	9.529E-01	0.985	pan	Severe COVID-19
18275_5	CRIP1	Cysteine-rich protein 1	P50238	CRIP1	NA	Inverse variance weighted	11	-0.004	0.075	9.529E-01	0.985	pan	Severe COVID-19
13123_3	FLRT3:ECD	Leucine-rich repeat transmembrane protein FLRT3:Extracellular domain	Q9NZU0	FLRT3	NA	Inverse variance weighted	9	0.002	0.027	9.544E-01	0.985	pan	Severe COVID-19
2580_83	Myeloperoxidase	Myeloperoxidase	P05164	MPO	NA	Inverse variance weighted	10	0.007	0.128	9.546E-01	0.985	pan	Severe COVID-19
19229_92	HOME1	Homer protein homolog 1	Q86YM7	HOMER1	NA	Inverse variance weighted	4	0.008	0.148	9.553E-01	0.985	pan	Severe COVID-19
12510_3	STAP1	Signal-transducing adaptor protein 1	Q9ULZ2	STAP1	NA	Wald ratio	1	-0.013	0.226	9.558E-01	0.985	pan	Severe COVID-19
10419_1	SCAR5	Scavenger receptor class A member 5	Q6ZMJ2	SCARA5	NA	Inverse variance weighted	10	0.005	0.086	9.563E-01	0.985	pan	Severe COVID-19
5483_1	RGMA	Repulsive guidance molecule A	Q96B86	RGMA	NA	Inverse variance weighted	8	0.007	0.129	9.566E-01	0.985	pan	Severe COVID-19
19124_9	UBCP1	Ubiquitin-like domain-containing CTD phosphatase 1	Q8WVY7	UBLCP1	NA	Inverse variance weighted	5	0.006	0.104	9.576E-01	0.985	pan	Severe COVID-19
5085_18	IL-20 Ra	Interleukin-20 receptor subunit alpha	Q9UHF4	IL20RA	NA	Wald ratio	1	-0.020	0.379	9.579E-01	0.985	pan	Severe COVID-19
13112_179	FSTL1	Follistatin-related protein 1	Q12841	FSTL1	NA	Inverse variance weighted	4	0.009	0.180	9.580E-01	0.985	pan	Severe COVID-19
3298_52	Contactin-4	Contactin-4	Q8IWV2	CNTN4	NA	Inverse variance weighted	8	0.002	0.046	9.581E-01	0.985	pan	Severe COVID-19
3440_7	granzyme A	Granzyme A	P12544	GZMA	NA	Inverse variance weighted	8	-0.003	0.053	9.592E-01	0.985	pan	Severe COVID-19
7999_23	CD39	Ectonucleoside triphosphate diphosphohydrolase 1	P49961	ENTPD1	NA	Inverse variance weighted	3	-0.008	0.159	9.592E-01	0.985	pan	Severe COVID-19
12551_3	ITCH	E3 ubiquitin-protein ligase Itchy homolog	Q96J02	ITCH	NA	Inverse variance weighted	2	-0.022	0.435	9.602E-01	0.985	pan	Severe COVID-19
13464_8	NRAC	Nutritionally-regulated adipose and cardiac enriched protein homolog	Q8N912	NRAC	NA	Inverse variance weighted	3	-0.009	0.187	9.613E-01	0.985	pan	Severe COVID-19
16926_44	Alkaline phosphatase, liver	Alkaline phosphatase, tissue-nonspecific isozyme	P05186	ALPL	NA	Inverse variance weighted	3	0.009	0.193	9.620E-01	0.985	pan	Severe COVID-19
4866_59	TrkB	BDNF/NT-3 growth factors receptor	Q16620	NTRK2	NA	Inverse variance weighted	2	-0.015	0.325	9.628E-01	0.985	pan	Severe COVID-19
5609_92	F19A5	Protein FAM19A5	Q7Z5A7	TAFA5	NA	Wald ratio	1	-0.015	0.326	9.631E-01	0.985	pan	Severe COVID-19
6471_53	FHR4	Complement factor H-related protein 4	Q92496	CFHR4	NA	Inverse variance weighted	19	0.002	0.045	9.634E-01	0.985	pan	Severe COVID-19
6257_56	CGRE1	Cell growth regulator with EF hand domain protein 1	Q99674	CGREF1	NA	Inverse variance weighted	4	-0.008	0.172	9.636E-01	0.985	pan	Severe COVID-19
5658_64	coagulation factor XIII B	Coagulation factor XIII B chain	P05160	F13B	NA	Inverse variance weighted	3	-0.013	0.282	9.636E-01	0.985	pan	Severe COVID-19
2819_23	Cadherin-5	Cadherin-5	P33151	CDH5	NA	Inverse variance weighted	5	-0.005	0.106	9.641E-01	0.985	pan	Severe COVID-19
12831_21	TESC	Calcineurin B homologous protein 3	Q96BS2	TESC	NA	Inverse variance weighted	11	-0.002	0.048	9.649E-01	0.985	pan	Severe COVID-19
8364_74	UST	Uronyl 2-sulfotransferase	Q9Y2C2	UST	NA	Inverse variance weighted	5	-0.005	0.110	9.659E-01	0.985	pan	Severe COVID-19
6060_2	PIP	Prolactin-inducible protein	P12273	PIP	NA	Inverse variance weighted	8	-0.003	0.082	9.663E-01	0.985	pan	Severe COVID-19
13686_2	IL-5 Ra	Interleukin-5 receptor subunit alpha	Q01344	IL5RA	NA	Inverse variance weighted	15	-0.002	0.047	9.671E-01	0.985	pan	Severe COVID-19
17722_5	FKBP52 protein	Peptidyl-prolyl cis-trans isomerase FKBP4	Q02790	FKBP4	NA	Inverse variance weighted	2	0.010	0.237	9.673E-01	0.985	pan	Severe COVID-19
19136_22	MMSA	Methylmalonate-semialdehyde dehydrogenase [acylating], mitochondrial	Q02252	ALDH6A1	NA	Inverse variance weighted	2	-0.011	0.265	9.675E-01	0.985	pan	Severe COVID-19
18197_97	KCRS	Creatine kinase S-type, mitochondrial	P17540	CKMT2	NA	Inverse variance weighted	2	0.008	0.211	9.682E-01	0.985	pan	Severe COVID-19
18343_10	DECR2	Peroxisomal 2,4-dienoyl-CoA reductase	Q9NUI1	DECR2	NA	Inverse variance weighted	8	-0.003	0.072	9.687E-01	0.985	pan	Severe COVID-19
7945_10	Semaphorin-6A	Semaphorin-6A	Q9H2E6	SEMA6A	NA	Inverse variance weighted	5	0.004	0.102	9.693E-01	0.985	pan	Severe COVID-19
10953_14	CLC2A	C-type lectin domain family 2 member A	Q6UVW9	CLEC2A	NA	Inverse variance weighted	4	0.004	0.102	9.694E-01	0.985	pan	Severe COVID-19
2632_5	IL-12 Rb1	Interleukin-12 receptor subunit beta-1	P42701	IL12RB1	NA	Wald ratio	1	0.007	0.193	9.698E-01	0.985	pan	Severe COVID-19

12329_21	KS6A1	Ribosomal protein S6 kinase alpha-1	Q15418	RPS6KA1	NA	Inverse variance weighted	10	-0.005	0.130	9.703E-01	0.985	pan	Severe COVID-19
9839_148	Tirap	Toll/interleukin-1 receptor domain-containing adapter protein	P58753	TIRAP	NA	Inverse variance weighted	3	-0.006	0.158	9.710E-01	0.985	pan	Severe COVID-19
5028_59	sCD163	Scavenger receptor cysteine-rich type 1 protein M130	Q86VB7	CD163	NA	Inverse variance weighted	11	0.003	0.081	9.712E-01	0.985	pan	Severe COVID-19
8028_22	SPINK5	Serine protease inhibitor Kazal-type 5	Q9NQ38	SPINK5	NA	Inverse variance weighted	2	0.008	0.230	9.717E-01	0.985	pan	Severe COVID-19
6081_52	PCOC2	Procollagen C-endopeptidase enhancer 2	Q9UKZ9	PCOLCE2	NA	Inverse variance weighted	15	-0.001	0.042	9.722E-01	0.985	pan	Severe COVID-19
19213_1	ISK4	Serine protease inhibitor Kazal-type 4	O60575	SPINK4	NA	Wald ratio	1	0.002	0.073	9.727E-01	0.985	pan	Severe COVID-19
14294_61	MBD1	Methyl-CpG-binding domain protein 1	Q9UIS9	MBD1	NA	Inverse variance weighted	3	-0.006	0.180	9.728E-01	0.985	pan	Severe COVID-19
15395_15	GST M1-1	Glutathione S-transferase Mu 1	P09488	GSTM1	NA	Inverse variance weighted	7	0.003	0.103	9.733E-01	0.985	pan	Severe COVID-19
13397_88	HBD-2	Beta-defensin 4A	O15263	DEFB4A	NA	Inverse variance weighted	4	0.003	0.088	9.742E-01	0.985	pan	Severe COVID-19
3172_28	ARSB	Arylsulfatase B	P15848	ARSB	NA	Inverse variance weighted	2	0.003	0.104	9.750E-01	0.985	pan	Severe COVID-19
5353_89	IL-1Ra	Interleukin-1 receptor antagonist protein	P18510	IL1RN	NA	Inverse variance weighted	4	0.007	0.208	9.750E-01	0.985	pan	Severe COVID-19
2212_69	tPA	Tissue-type plasminogen activator	P00750	PLAT	NA	Inverse variance weighted	9	-0.004	0.118	9.753E-01	0.985	pan	Severe COVID-19
10627_87	APLP2	Amyloid-like protein 2	Q06481	APLP2	NA	Inverse variance weighted	2	-0.005	0.167	9.760E-01	0.985	pan	Severe COVID-19
10900_272	STMN2	Stathmin-2	Q93045	STMN2	NA	Wald ratio	1	0.010	0.327	9.761E-01	0.985	pan	Severe COVID-19
5108_72	Notch-3	Neurogenic locus notch homolog protein 3	Q9UM47	NOTCH3	NA	Inverse variance weighted	2	0.005	0.159	9.766E-01	0.985	pan	Severe COVID-19
5618_50	FAM3B	Protein FAM3B	P58499	FAM3B	NA	Inverse variance weighted	2	0.012	0.411	9.771E-01	0.985	pan	Severe COVID-19
9264_11	CATO	Cathepsin O	P43234	CTSO	NA	Inverse variance weighted	3	0.003	0.094	9.772E-01	0.985	pan	Severe COVID-19
12338_27	EGFLA	Pikachurin	Q63HQ2	EGFLAM	NA	Inverse variance weighted	3	0.002	0.064	9.779E-01	0.985	pan	Severe COVID-19
19143_38	NB5R2	NADH-cytochrome b5 reductase 2	Q6BCY4	CYB5R2	NA	Inverse variance weighted	7	0.002	0.062	9.789E-01	0.985	pan	Severe COVID-19
5630_48	CMR35H	CMRF35-like molecule 8	Q9UGN4	CD300A	NA	Inverse variance weighted	13	-0.001	0.029	9.793E-01	0.985	pan	Severe COVID-19
9340_17	FKB14	Peptidyl-prolyl cis-trans isomerase FKBP14	Q9NWM8	FKBP14	NA	Wald ratio	1	0.008	0.311	9.795E-01	0.985	pan	Severe COVID-19
4469_78	ST4S6	Carbohydrate sulfotransferase 15	Q7LFX5	CHST15	NA	Inverse variance weighted	3	-0.003	0.116	9.796E-01	0.985	pan	Severe COVID-19
12663_1	THTR	Thiosulfate sulfurtransferase	Q16762	TST	NA	Inverse variance weighted	15	-0.001	0.045	9.797E-01	0.985	pan	Severe COVID-19
15565_102	CA125	Mucin-16	Q8WXI7	MUC16	NA	Inverse variance weighted	8	0.002	0.075	9.798E-01	0.985	pan	Severe COVID-19
19614_8	Holo-TC I	Transcobalamin-1	P20061	TCN1	NA	Inverse variance weighted	12	0.001	0.038	9.808E-01	0.985	pan	Severe COVID-19
2994_71	IL-1Rrp2	Interleukin-1 receptor-like 2	Q9HB29	IL1RL2	NA	Wald ratio	1	-0.003	0.106	9.810E-01	0.985	pan	Severe COVID-19
2617_56	ERBB3	Receptor tyrosine-protein kinase erbB-3	P21860	ERBB3	NA	Inverse variance weighted	2	0.008	0.369	9.816E-01	0.985	pan	Severe COVID-19
5116_62	ROBO2	Roundabout homolog 2	Q9HCK4	ROBO2	NA	Inverse variance weighted	2	0.004	0.173	9.817E-01	0.985	pan	Severe COVID-19
8243_55	TATI	Serine protease inhibitor Kazal-type 1	P00995	SPINK1	NA	Inverse variance weighted	2	-0.004	0.162	9.818E-01	0.985	pan	Severe COVID-19
5638_23	GT251	Procollagen galactosyltransferase 1	Q8NBJ5	COLGALT1	NA	Inverse variance weighted	9	-0.002	0.079	9.823E-01	0.985	pan	Severe COVID-19
8664_36	PKDCC	Extracellular tyrosine-protein kinase PKDCC	Q504Y2	PKDCC	NA	Inverse variance weighted	2	0.012	0.558	9.825E-01	0.985	pan	Severe COVID-19
18243_9	Cytidylate kinase	UMP-CMP kinase	P30085	CMPK1	NA	Wald ratio	1	0.004	0.195	9.834E-01	0.985	pan	Severe COVID-19
2602_2	Angiopoietin-2	Angiopoietin-2	O15123	ANGPT2	NA	Inverse variance weighted	3	0.002	0.101	9.837E-01	0.985	pan	Severe COVID-19
13657_2	PNKP	Bifunctional polynucleotide phosphatase/kinase	Q96T60	PNKP	NA	Wald ratio	1	0.003	0.147	9.839E-01	0.985	pan	Severe COVID-19
3806_55	EphA5	Ephrin type-A receptor 5	P54756	EPHA5	NA	Inverse variance weighted	3	-0.002	0.122	9.840E-01	0.985	pan	Severe COVID-19
5030_52	SIRT2	NAD-dependent protein deacetylase sirtuin-2	Q8IXJ6	SIRT2	NA	Inverse variance weighted	6	0.004	0.198	9.847E-01	0.985	pan	Severe COVID-19
7210_25	Amyloid-like protein 1	Amyloid-like protein 1	P51693	APLP1	NA	Wald ratio	1	0.008	0.438	9.851E-01	0.985	pan	Severe COVID-19
12033_3	BUB1	Mitotic checkpoint serine/threonine-protein kinase BUB1	O43683	BUB1	NA	Wald ratio	1	-0.004	0.231	9.855E-01	0.985	pan	Severe COVID-19
5465_32	H6ST1	Heparan-sulfate 6-O-sulfotransferase 1	O60243	HS6ST1	NA	Inverse variance weighted	2	-0.002	0.091	9.860E-01	0.985	pan	Severe COVID-19
5736_1	TRML2	Trem-like transcript 2 protein	Q5T2D2	TREML2	NA	Inverse variance weighted	5	-0.001	0.040	9.866E-01	0.985	pan	Severe COVID-19
15368_3	BMPER	BMP-binding endothelial regulator protein	Q8N8U9	BMPER	NA	Inverse variance weighted	3	0.002	0.105	9.867E-01	0.985	pan	Severe COVID-19
14012_17	LGP2	Probable ATP-dependent RNA helicase DHX58	Q96C10	DHX58	NA	Inverse variance weighted	3	-0.002	0.133	9.877E-01	0.985	pan	Severe COVID-19
10948_14	PLD3	Phospholipase D3	Q8IV08	PLD3	NA	Wald ratio	1	0.004	0.265	9.883E-01	0.985	pan	Severe COVID-19
6550_4	ICAM4	Intercellular adhesion molecule 4	Q14773	ICAM4	NA	Inverse variance weighted	13	-0.001	0.051	9.894E-01	0.985	pan	Severe COVID-19
19130_81	SPB8	Serpin B8	P50452	SERPINB8	NA	Inverse variance weighted	3	-0.001	0.104	9.899E-01	0.985	pan	Severe COVID-19
5703_26	NOE1	Noelin	Q99784	OLFM1	NA	Inverse variance weighted	3	-0.002	0.120	9.900E-01	0.985	pan	Severe COVID-19
15525_294	ADH1G	Alcohol dehydrogenase 1C	P00326	ADH1C	NA	Wald ratio	1	-0.001	0.116	9.901E-01	0.985	pan	Severe COVID-19
6520_87	MGP	Matrix Gla protein	P08493	MGP	NA	Inverse variance weighted	2	-0.002	0.215	9.909E-01	0.985	pan	Severe COVID-19
7787_25	LIRA5	Leukocyte immunoglobulin-like receptor subfamily A member 5	A6NI73	LILRA5	NA	Wald ratio	1	-0.003	0.304	9.909E-01	0.985	pan	Severe COVID-19
5676_54	ASIP	Agouti-signaling protein	P42127	ASIP	NA	Inverse variance weighted	10	0.001	0.093	9.911E-01	0.985	pan	Severe COVID-19
19113_66	SH3L2	SH3 domain-binding glutamic acid-rich-like protein 2	Q9UJC5	SH3BGRL2	NA	Wald ratio	1	-0.002	0.144	9.913E-01	0.985	pan	Severe COVID-19
3434_34	FN1.3	Fibronectin Fragment 3	P02751	FN1	NA	Wald ratio	1	0.002	0.222	9.918E-01	0.985	pan	Severe COVID-19
18864_7	TRY3	Trypsin-3	P35030	PRSS3	NA	Inverse variance weighted	29	0.000	0.030	9.923E-01	0.985	pan	Severe COVID-19
13130_150	HXK2	Hexokinase-2	P52789	HK2	NA	Inverse variance weighted	2	-0.002	0.186	9.925E-01	0.985	pan	Severe COVID-19
18241_18	HEM6	Oxygen-dependent coproporphyrinogen-III oxidase, mitochondrial	P36551	CPOX	NA	Inverse variance weighted	10	-0.001	0.083	9.926E-01	0.985	pan	Severe COVID-19
3232_28	TrATPase	Tartrate-resistant acid phosphatase type 5	P13686	ACP5	NA	Inverse variance weighted	6	-0.001	0.068	9.928E-01	0.985	pan	Severe COVID-19
4187_49	6-Phosphogluconate dehydrogenase	6-phosphogluconate dehydrogenase, decarboxylating	P52209	PGD	NA	Wald ratio	1	0.002	0.198	9.936E-01	0.985	pan	Severe COVID-19
14134_49	AMGO2	Amphoterin-induced protein 2	Q86SJ2	AMIGO2	NA	Inverse variance weighted	3	0.001	0.139	9.940E-01	0.985	pan	Severe COVID-19
9525_1	PTK7	Inactive tyrosine-protein kinase 7	Q13308	PTK7	NA	Inverse variance weighted	4	0.001	0.240	9.951E-01	0.985	pan	Severe COVID-19
9234_8	TWSG1	Twisted gastrulation protein homolog 1	Q9GZX9	TWSG1	NA	Inverse variance weighted	2	-0.002	0.283	9.955E-01	0.985	pan	Severe COVID-19
17343_6	SNPH	Syntaphilin	O15079	SNPH	NA	Inverse variance weighted	2	-0.001	0.174	9.959E-01	0.985	pan	Severe COVID-19
12703_6	NEK7	Serine/threonine-protein kinase Nek7	Q8TDX7	NEK7	NA	Wald ratio	1	-0.001	0.126	9.964E-01	0.985	pan	Severe COVID-19
8351_17	PRS57	Serine protease 57	Q6UWY2	PRSS57	NA	Inverse variance weighted	7	0.000	0.060	9.967E-01	0.985	pan	Severe COVID-19
16805_5	PDE5A	cGMP-specific 3',5'-cyclic phosphodiesterase	O76074	PDE5A	NA	Wald ratio	1	0.002	0.385	9.967E-01	0.985	pan	Severe COVID-19
9297_12	B3GN8	UDP-GlcNAc:betaGal beta-1,3-N-acetylglucosaminyltransferase 8	Q7Z7M8	B3GNT8	NA	Inverse variance weighted	11	0.000	0.069	9.968E-01	0.985	pan	Severe COVID-19
17677_47	BY55	CD160 antigen	O95971	CD160	NA	Wald ratio	1	0.000	0.137	9.972E-01	0.985	pan	Severe COVID-19
3290_50	CD109	CD109 antigen	Q6YHK3	CD109	NA	Inverse variance weighted	17	0.000	0.037	9.985E-01	0.986	pan	Severe COVID-19
2615_60	Ephrin-A5	Ephrin-A5	P52803	EFNA5	NA	Inverse variance weighted	3	0.000	0.154	1.000E+00	0.987	pan	Severe COVID-19
13992_12	NSF	Vesicle-fusing ATPase	P46459	NSF	NA	Wald ratio	1	-0.614	0.146	2.642E-05	0.046	pan	Hospitalized COVID-19
17769_28	PCNP	PEST proteolytic signal-containing nuclear protein	Q8WW12	PCNP	NA	Wald ratio	1	-0.479	0.117	4.237E-05	0.046	pan	Hospitalized COVID-19
12534_10	CACO2	Calcium-binding and coiled-coil domain-containing protein 2	Q13137	CALCOCO2	NA	Wald ratio	1	-0.451	0.116	1.041E-04	0.061	pan	Hospitalized COVID-19
10608_9	HIS1	Histatin-1	P15515	HTN1	NA	Inverse variance weighted	2	0.272	0.071	1.340E-04	0.061	pan	Hospitalized COVID-19
10346_5	STAT3	Signal transducer and activator of transcription 3	P40763	STAT3	NA	Inverse variance weighted	2	-0.303	0.080	1.497E-04	0.061	pan	Hospitalized COVID-19
8255_34	MRVI1	Protein MRVI1	Q9Y6F6	IRAG1	NA	Inverse variance weighted	2	-0.319	0.085	1.678E-04	0.061	pan	Hospitalized COVID-19
6359_50	AGO61	Protein O-linked-mannose beta-1,4-N-acetylglucosaminyltransferase 2	Q8NAT1	POMGNT2	NA	Inverse variance weighted	12	-0.103	0.028	2.295E-04	0.071	pan	Hospitalized COVID-19
12387_7	PDLI4	PDZ and LIM domain protein 4	P50479	PDLIM4	NA	Wald ratio	1	-0.276	0.078	3.729E-04	0.101	pan	Hospitalized COVID-19
12656_1	KLC1	Kinesin light chain 1	Q07866	KLC1	NA	Wald ratio	1	0.714	0.206	5.394E-04	0.130	pan	Hospitalized COVID-19
13130_150	HXK2	Hexokinase-2	P52789	HK2	NA	Inverse variance weighted	2	-0.434	0.128	7.207E-04	0.157	pan	Hospitalized COVID-19

9638_2	TIGIT	T-cell immunoreceptor with Ig and ITIM domains	Q495A1	TIGIT	NA	Wald ratio	1	-0.432	0.133	1.173E-03	0.232	pan	Hospitalized COVID-19
2731_29	NADPH-P450 Oxidoreductase	NADPH--cytochrome P450 reductase	P16435	POR	NA	Inverse variance weighted	4	-0.238	0.074	1.288E-03	0.234	pan	Hospitalized COVID-19
4541_49	CDON	Cell adhesion molecule-related/down-regulated by oncogenes	Q4KMG0	CDON	NA	Inverse variance weighted	5	0.083	0.026	1.610E-03	0.270	pan	Hospitalized COVID-19
9017_58	LPH	Lactase-phlorizin hydrolase	P09848	LCT	NA	Inverse variance weighted	14	-0.054	0.017	1.782E-03	0.277	pan	Hospitalized COVID-19
3348_49	BMP-1	Bone morphogenetic protein 1	P13497	BMP1	NA	Wald ratio	1	0.484	0.159	2.366E-03	0.343	pan	Hospitalized COVID-19
18884_22	DNJB4	DnaJ homolog subfamily B member 4	Q9UDY4	DNAJB4	NA	Inverse variance weighted	3	0.177	0.060	2.864E-03	0.370	pan	Hospitalized COVID-19
3415_61	BSP	Bone sialoprotein 2	P21815	IBSP	NA	Inverse variance weighted	2	-0.285	0.096	3.010E-03	0.370	pan	Hospitalized COVID-19
11543_84	LIMA1	LIM domain and actin-binding protein 1	Q9UHB6	LIMA1	NA	Wald ratio	1	0.476	0.161	3.057E-03	0.370	pan	Hospitalized COVID-19
6955_68	SNX1	Sorting nexin-1	Q13596	SNX1	NA	Wald ratio	1	-0.228	0.078	3.486E-03	0.399	pan	Hospitalized COVID-19
8235_48	SCG1	Secretogranin-1	P05060	CHGB	NA	Inverse variance weighted	7	0.209	0.074	4.598E-03	0.500	pan	Hospitalized COVID-19
17806_6	Syntenin 1	Syntenin-1	O00560	SDCBP	NA	Wald ratio	1	0.623	0.221	4.851E-03	0.503	pan	Hospitalized COVID-19
17447_52	SFRP4	Secreted frizzled-related protein 4	Q6FHJ7	SFRP4	NA	Inverse variance weighted	4	-0.122	0.044	5.139E-03	0.509	pan	Hospitalized COVID-19
18917_53	Pancreatic alpha-amylase	Pancreatic alpha-amylase	P04746	AMY2A	NA	Inverse variance weighted	7	-0.191	0.070	6.436E-03	0.571	pan	Hospitalized COVID-19
2871_73	RAD51	DNA repair protein RAD51 homolog 1	Q06609	RAD51	NA	Wald ratio	1	-0.586	0.216	6.683E-03	0.571	pan	Hospitalized COVID-19
10554_23	BGAL	Beta-galactosidase	P16278	GLB1	NA	Wald ratio	1	0.617	0.228	6.815E-03	0.571	pan	Hospitalized COVID-19
15583_18	FCRLB	Fc receptor-like B	Q6BAA4	FCRLB	NA	Inverse variance weighted	4	0.152	0.056	6.816E-03	0.571	pan	Hospitalized COVID-19
10758_2	KERA	Keratocan	O60938	KERA	NA	Inverse variance weighted	2	-0.330	0.125	8.537E-03	0.688	pan	Hospitalized COVID-19
11557_3	SMUF1	E3 ubiquitin-protein ligase SMURF1	Q9HCE7	SMURF1	NA	Wald ratio	1	0.222	0.085	9.031E-03	0.699	pan	Hospitalized COVID-19
2864_2	MEK1	Dual specificity mitogen-activated protein kinase kinase 1	Q02750	MAP2K1	NA	Wald ratio	1	0.451	0.176	1.028E-02	0.699	pan	Hospitalized COVID-19
6620_82	LIGO1	Leucine-rich repeat and immunoglobulin-like domain-containing nogo receptor-interacting protein 1	Q96FE5	LINGO1	NA	Inverse variance weighted	3	0.255	0.100	1.089E-02	0.699	pan	Hospitalized COVID-19
10940_25	SRCA	Sarcalumenin	Q86TD4	SRL	NA	Inverse variance weighted	3	0.120	0.047	1.098E-02	0.699	pan	Hospitalized COVID-19
8048_9	FTMT	Ferritin, mitochondrial	Q8N4E7	FTMT	NA	Inverse variance weighted	8	-0.141	0.055	1.120E-02	0.699	pan	Hospitalized COVID-19
4188_1	Aflatoxin B1 aldehyde reductase	Aflatoxin B1 aldehyde reductase member 2	O43488	AKR7A2	NA	Wald ratio	1	0.170	0.067	1.146E-02	0.699	pan	Hospitalized COVID-19
9754_33	Quinone reductase 2	Ribosyldihydronicotinamide dehydrogenase [quinone]	P16083	NQO2	NA	Inverse variance weighted	9	0.044	0.018	1.205E-02	0.699	pan	Hospitalized COVID-19
19622_7	Activin A	Activin A	P08476	INHBA	NA	Inverse variance weighted	2	0.351	0.141	1.250E-02	0.699	pan	Hospitalized COVID-19
5810_25	Cripto	Teratocarcinoma-derived growth factor 1	P13385	TDGF1	NA	Inverse variance weighted	20	-0.080	0.032	1.257E-02	0.699	pan	Hospitalized COVID-19
3708_62	a2-Macroglobulin	Alpha-2-macroglobulin	P01023	A2M	NA	Inverse variance weighted	2	-0.299	0.120	1.266E-02	0.699	pan	Hospitalized COVID-19
17460_51	Mx1	Interferon-induced GTP-binding protein Mx1	P20591	MX1	NA	Inverse variance weighted	9	-0.084	0.034	1.275E-02	0.699	pan	Hospitalized COVID-19
6342_10	Nephronectin	Nephronectin	Q6UXI9	NPNT	NA	Inverse variance weighted	4	0.237	0.095	1.275E-02	0.699	pan	Hospitalized COVID-19
19504_22	KTHY	Thymidylate kinase	P23919	DTYMK	NA	Inverse variance weighted	3	0.331	0.133	1.284E-02	0.699	pan	Hospitalized COVID-19
7257_18	TIP39	Tuberoinfundibular peptide of 39 residues	Q96A98	PTH2	NA	Wald ratio	1	0.679	0.277	1.416E-02	0.752	pan	Hospitalized COVID-19
12801_33	IRF2	Interferon regulatory factor 2	P14316	IRF2	NA	Wald ratio	1	-0.488	0.200	1.464E-02	0.759	pan	Hospitalized COVID-19
8353_15	SCN2B	Sodium channel subunit beta-2	O60939	SCN2B	NA	Inverse variance weighted	6	-0.210	0.087	1.552E-02	0.772	pan	Hospitalized COVID-19
12813_18	EHBP1	EH domain-binding protein 1	Q8NDI1	EHBP1	NA	Wald ratio	1	-0.497	0.206	1.559E-02	0.772	pan	Hospitalized COVID-19
16613_3	CAD17	Cadherin-17	Q12864	CDH17	NA	Inverse variance weighted	16	0.067	0.028	1.758E-02	0.817	pan	Hospitalized COVID-19
5229_90	IMDH1	Inosine-5'-monophosphate dehydrogenase 1	P20839	IMPDH1	NA	Inverse variance weighted	9	-0.144	0.061	1.806E-02	0.817	pan	Hospitalized COVID-19
18376_19	Myosin light chain 1	Myosin light chain 3	P08590	MYL3	NA	Inverse variance weighted	6	-0.203	0.086	1.809E-02	0.817	pan	Hospitalized COVID-19
18216_22	IL-11 RA	Interleukin-11 receptor subunit alpha	Q14626	IL11RA	NA	Inverse variance weighted	7	-0.146	0.062	1.834E-02	0.817	pan	Hospitalized COVID-19
3009_3	TGF-b R III	Transforming growth factor beta receptor type 3	Q03167	TGFBR3	NA	Inverse variance weighted	6	-0.124	0.053	1.840E-02	0.817	pan	Hospitalized COVID-19
13090_17	S100A6	Protein S100-A6	P06703	S100A6	NA	Inverse variance weighted	3	0.307	0.131	1.898E-02	0.817	pan	Hospitalized COVID-19
3175_51	ATS13	A disintegrin and metalloproteinase with thrombospondin motifs 13	Q76LX8	ADAMTS13	NA	Inverse variance weighted	13	-0.064	0.027	1.913E-02	0.817	pan	Hospitalized COVID-19
19383_131	CINP	Cyclin-dependent kinase 2-interacting protein	Q9BW66	CINP	NA	Wald ratio	1	0.436	0.189	2.101E-02	0.860	pan	Hospitalized COVID-19
5611_56	D108B	Beta-defensin 108B	Q8NET1	DEFB108B	NA	Wald ratio	1	-0.593	0.257	2.106E-02	0.860	pan	Hospitalized COVID-19
5478_50	PSMA	Glutamate carboxypeptidase 2	Q04609	FOLH1	NA	Inverse variance weighted	4	-0.126	0.055	2.143E-02	0.860	pan	Hospitalized COVID-19
12956_40	KBP	KIF1-binding protein	Q96EK5	KIFBP	NA	Wald ratio	1	0.559	0.244	2.203E-02	0.860	pan	Hospitalized COVID-19
5491_12	Testican-2	Testican-2	Q92563	SPOCK2	NA	Inverse variance weighted	13	-0.106	0.046	2.211E-02	0.860	pan	Hospitalized COVID-19
12583_77	ARAF	Serine/threonine-protein kinase A-Raf	P10398	ARAF	NA	Wald ratio	1	-0.445	0.196	2.315E-02	0.869	pan	Hospitalized COVID-19
19196_73	HOP	Homeodomain-only protein	Q9BPY8	HOPX	NA	Wald ratio	1	-0.261	0.115	2.329E-02	0.869	pan	Hospitalized COVID-19
4125_52	sRAGE	Advanced glycosylation end product-specific receptor, soluble	Q15109	AGER	NA	Inverse variance weighted	46	-0.072	0.032	2.355E-02	0.869	pan	Hospitalized COVID-19
13940_19	IP16:HIN 1	Gamma-interferon-inducible protein 16:Isoform 2, Hematopoietic expression, interferon-inducible nature, and nuclear localization 1	Q16666	IFI16	NA	Wald ratio	1	0.349	0.155	2.399E-02	0.870	pan	Hospitalized COVID-19
11103_24	HSP 27	Heat shock protein beta-1	P04792	HSPB1	NA	Inverse variance weighted	8	-0.062	0.028	2.555E-02	0.898	pan	Hospitalized COVID-19
18188_12	GATM	Glycine amidinotransferase, mitochondrial	P50440	GATM	NA	Wald ratio	1	-0.132	0.059	2.621E-02	0.898	pan	Hospitalized COVID-19
18382_109	Catechol O-methyltransferase	Catechol O-methyltransferase	P21964	COMT	NA	Wald ratio	1	-0.256	0.115	2.653E-02	0.898	pan	Hospitalized COVID-19
5008_51	Mn SOD	Superoxide dismutase [Mn], mitochondrial	P04179	SOD2	NA	Inverse variance weighted	4	0.232	0.105	2.711E-02	0.898	pan	Hospitalized COVID-19
4801_13	PERL	Lactoperoxidase	P22079	LPO	NA	Inverse variance weighted	9	0.105	0.048	2.733E-02	0.898	pan	Hospitalized COVID-19
15678_71	DKK2	Dickkopf-related protein 2	Q9UBU2	DKK2	NA	Inverse variance weighted	2	-0.432	0.196	2.742E-02	0.898	pan	Hospitalized COVID-19
6617_12	FCRL6	Fc receptor-like protein 6	Q6DN72	FCRL6	NA	Wald ratio	1	-0.183	0.084	2.922E-02	0.898	pan	Hospitalized COVID-19
13447_42	SPRN	Shadow of prion protein	Q5BIV9	SPRN	NA	Inverse variance weighted	2	-0.374	0.172	2.968E-02	0.898	pan	Hospitalized COVID-19
3181_50	Cathepsin S	Cathepsin S	P25774	CTSS	NA	Inverse variance weighted	11	-0.085	0.039	2.973E-02	0.898	pan	Hospitalized COVID-19
10630_5	HTAI2	Oxidoreductase HTATIP2	Q9BUP3	HTATIP2	NA	Inverse variance weighted	9	-0.058	0.027	2.973E-02	0.898	pan	Hospitalized COVID-19
8337_65	PTPRU	Receptor-type tyrosine-protein phosphatase U	Q92729	PTPRU	NA	Inverse variance weighted	5	-0.131	0.060	3.048E-02	0.898	pan	Hospitalized COVID-19
17513_11	ANX11	Annexin A11	P50995	ANXA11	NA	Inverse variance weighted	3	0.185	0.086	3.101E-02	0.898	pan	Hospitalized COVID-19
12634_79	BCAR3:Ras-GEF	Breast cancer anti-estrogen resistance protein 3:Guanine Nucleotide Exchange Factor Domain	O75815	BCAR3	NA	Inverse variance weighted	3	-0.163	0.076	3.112E-02	0.898	pan	Hospitalized COVID-19
3038_9	I-TAC	C-X-C motif chemokine 11	O14625	CXCL11	NA	Inverse variance weighted	8	-0.101	0.047	3.117E-02	0.898	pan	Hospitalized COVID-19
12831_21	TESC	Calcineurin B homologous protein 3	Q96BS2	TESC	NA	Inverse variance weighted	11	0.075	0.035	3.121E-02	0.898	pan	Hospitalized COVID-19
19207_119	ADK	Adenosine kinase	P55263	ADK	NA	Wald ratio	1	0.416	0.194	3.175E-02	0.898	pan	Hospitalized COVID-19
18931_40	SLIT3	Slit homolog 3 protein	O75094	SLIT3	NA	Inverse variance weighted	3	-0.141	0.066	3.244E-02	0.898	pan	Hospitalized COVID-19
5825_49	IFN-g R1	Interferon gamma receptor 1	P15260	IFNGR1	NA	Inverse variance weighted	3	-0.242	0.114	3.325E-02	0.898	pan	Hospitalized COVID-19
5337_64	B7-2	T-lymphocyte activation antigen CD86	P42081	CD86	NA	Wald ratio	1	-0.374	0.176	3.374E-02	0.898	pan	Hospitalized COVID-19
4500_50	SCGF-alpha	Stem cell growth factor-alpha	Q9Y240	CLEC11A	NA	Inverse variance weighted	8	-0.055	0.026	3.374E-02	0.898	pan	Hospitalized COVID-19
18830_1	Omentin	Intelectin-1	Q8WWA0	ITLN1	NA	Inverse variance weighted	4	0.202	0.095	3.376E-02	0.898	pan	Hospitalized COVID-19
10756_34	UCN3	Urocortin-3	Q969E3	UCN3	NA	Wald ratio	1	-0.335	0.159	3.519E-02	0.898	pan	Hospitalized COVID-19
8766_29	LIRA5	Leukocyte immunoglobulin-like receptor subfamily A member 5	A6NI73	LILRA5	NA	Inverse variance weighted	3	-0.311	0.148	3.528E-02	0.898	pan	Hospitalized COVID-19
15339_32	COF2	Cofilin-2	Q9Y281	CFL2	NA	Wald ratio	1	0.181	0.086	3.577E-02	0.898	pan	Hospitalized COVID-19
7624_19	ANK2	Ankyrin-2	Q01484	ANK2	NA	Inverse variance weighted	5	0.138	0.066	3.723E-02	0.898	pan	Hospitalized COVID-19
11212_7	TXND5	Thioredoxin domain-containing protein 5	Q8NBS9	TXNDC5	NA	Inverse variance weighted	5	0.121	0.058	3.834E-02	0.898	pan	Hospitalized COVID-19
19127_1	HSPB6	Heat shock protein beta-6	O14558	HSPB6	NA	Wald ratio	1	0.513	0.248	3.841E-02	0.898	pan	Hospitalized COVID-19
10440_26	ACAM:ECD	CXADR-like membrane protein:Extracellular domain	Q9H6B4	CLMP	NA	Inverse variance weighted	3	0.096	0.047	3.901E-02	0.898	pan	Hospitalized COVID-19

9779_63	HERP2	Homocysteine-responsive endoplasmic reticulum-resident ubiquitin-like domain member 2 protein	Q9BSE4	HERPUD2	NA	Wald ratio	1	0.462	0.224	3.906E-02	0.898	pan	Hospitalized COVID-19
16599_38	GPN1	GPN-loop GTPase 1	Q9HCN4	GPN1	NA	Inverse variance weighted	6	-0.111	0.054	3.986E-02	0.898	pan	Hospitalized COVID-19
4911_49	Glutathione S-transferase Pi	Glutathione S-transferase P	P09211	GSTP1	NA	Wald ratio	1	-0.154	0.075	4.025E-02	0.898	pan	Hospitalized COVID-19
12975_11	Keratin 20	Keratin, type I cytoskeletal 20	P35900	KRT20	NA	Inverse variance weighted	3	-0.259	0.127	4.102E-02	0.898	pan	Hospitalized COVID-19
11126_102	TRIO	Triple functional domain protein	O75962	TRIO	NA	Wald ratio	1	-0.471	0.231	4.132E-02	0.898	pan	Hospitalized COVID-19
6257_56	CGRE1	Cell growth regulator with EF hand domain protein 1	Q99674	CGREF1	NA	Inverse variance weighted	4	0.240	0.118	4.157E-02	0.898	pan	Hospitalized COVID-19
17792_158	SSDH	Succinate-semialdehyde dehydrogenase, mitochondrial	P51649	ALDH5A1	NA	Inverse variance weighted	17	-0.081	0.040	4.160E-02	0.898	pan	Hospitalized COVID-19
9173_21	PGM1	Phosphoglucomutase-1	P36871	PGM1	NA	Wald ratio	1	0.255	0.125	4.170E-02	0.898	pan	Hospitalized COVID-19
6528_95	EXTL2	Exostosin-like 2	Q9UBQ6	EXTL2	NA	Wald ratio	1	0.440	0.217	4.219E-02	0.898	pan	Hospitalized COVID-19
9599_6	PIANP	PILR alpha-associated neural protein	Q8IYJ0	PIANP	NA	Wald ratio	1	-0.462	0.228	4.267E-02	0.898	pan	Hospitalized COVID-19
6404_20	C1QRF	C1q-related factor	O75973	C1QL1	NA	Inverse variance weighted	7	-0.088	0.044	4.271E-02	0.898	pan	Hospitalized COVID-19
13384_110	FUMH	Fumarate hydratase, mitochondrial	P07954	FH	NA	Inverse variance weighted	2	0.278	0.138	4.356E-02	0.898	pan	Hospitalized COVID-19
8464_31	RSPO4	R-spondin-4	Q2I0M5	RSPO4	NA	Inverse variance weighted	3	0.216	0.107	4.435E-02	0.898	pan	Hospitalized COVID-19
14205_6	HEXI2	Protein HEXIM2	Q96MH2	HEXIM2	NA	Wald ratio	1	0.448	0.223	4.463E-02	0.898	pan	Hospitalized COVID-19
13697_51	GPDA	Glycerol-3-phosphate dehydrogenase [NAD(+)], cytoplasmic	P21695	GPD1	NA	Wald ratio	1	0.485	0.243	4.529E-02	0.898	pan	Hospitalized COVID-19
9049_2	MOT4	Monocarboxylate transporter 4	O15427	SLC16A3	NA	Wald ratio	1	0.517	0.259	4.597E-02	0.898	pan	Hospitalized COVID-19
8352_26	SIG12:Ig-like C2-type 2	Sialic acid-binding Ig-like lectin 12:Ig-like C2-type 2 domain, Isoform short	Q96PQ1	SIGLEC12	NA	Wald ratio	1	0.485	0.243	4.618E-02	0.898	pan	Hospitalized COVID-19
15467_10	CTHR1	Collagen triple helix repeat-containing protein 1	Q96CG8	CTHRC1	NA	Inverse variance weighted	2	0.591	0.297	4.622E-02	0.898	pan	Hospitalized COVID-19
6506_54	TMEDA	Transmembrane emp24 domain-containing protein 10	P49755	TMED10	NA	Inverse variance weighted	6	0.171	0.086	4.636E-02	0.898	pan	Hospitalized COVID-19
15388_24	FcRIIIa	Low affinity immunoglobulin gamma Fc region receptor III-A	P08637	FCGR3A	NA	Inverse variance weighted	11	-0.069	0.035	4.665E-02	0.898	pan	Hospitalized COVID-19
4254_6	NUDC3	NudC domain-containing protein 3	Q8IVD9	NUDCD3	NA	Wald ratio	1	0.304	0.153	4.713E-02	0.898	pan	Hospitalized COVID-19
11237_49	PCOC1	Procollagen C-endopeptidase enhancer 1	Q15113	PCOLCE	NA	Inverse variance weighted	4	0.231	0.116	4.719E-02	0.898	pan	Hospitalized COVID-19
19197_95	THIL	Acetyl-CoA acetyltransferase, mitochondrial	P24752	ACAT1	NA	Inverse variance weighted	3	0.238	0.120	4.760E-02	0.898	pan	Hospitalized COVID-19
18231_147	PRND	Prion-like protein doppel	Q9UKY0	PRND	NA	Wald ratio	1	0.396	0.200	4.773E-02	0.898	pan	Hospitalized COVID-19
8340_9	DB110	Beta-defensin 110	Q30KQ9	DEFB110	NA	Wald ratio	1	-0.407	0.206	4.810E-02	0.898	pan	Hospitalized COVID-19
13988_67	NMRL1	NmrA-like family domain-containing protein 1	Q9HBL8	NMRAL1	NA	Inverse variance weighted	12	-0.057	0.029	4.826E-02	0.898	pan	Hospitalized COVID-19
18183_3	ARH	Low density lipoprotein receptor adapter protein 1	Q5SW96	LDLRAP1	NA	Wald ratio	1	-0.251	0.127	4.849E-02	0.898	pan	Hospitalized COVID-19
9183_7	IFN-a/b R1	Interferon alpha/beta receptor 1	P17181	IFNAR1	NA	Inverse variance weighted	9	0.038	0.019	4.857E-02	0.898	pan	Hospitalized COVID-19
15333_11	SDF2	Stromal cell-derived factor 2	Q99470	SDF2	NA	Inverse variance weighted	8	0.126	0.064	4.877E-02	0.898	pan	Hospitalized COVID-19
13438_115	CHRD	Chordin	Q9H2X0	CHRD	NA	Wald ratio	1	-0.278	0.142	4.946E-02	0.898	pan	Hospitalized COVID-19
13476_16	KIN17	DNA/RNA-binding protein KIN17	O60870	KIN	NA	Wald ratio	1	-0.391	0.199	4.953E-02	0.898	pan	Hospitalized COVID-19
3030_3	DC-SIGNR	C-type lectin domain family 4 member M	Q9H2X3	CLEC4M	NA	Wald ratio	1	0.483	0.246	4.971E-02	0.898	pan	Hospitalized COVID-19
8768_4	Bcl-10	B-cell lymphoma/leukemia 10	O95999	BCL10	NA	Wald ratio	1	0.438	0.224	4.999E-02	0.898	pan	Hospitalized COVID-19
19560_23	PLXA4	Plexin-A4	Q9HCM2	PLXNA4	NA	Wald ratio	1	-0.252	0.129	5.033E-02	0.898	pan	Hospitalized COVID-19
9377_25	SCF	Kit ligand	P21583	KITLG	NA	Inverse variance weighted	3	0.226	0.117	5.255E-02	0.904	pan	Hospitalized COVID-19
9971_5	CSMD2	CUB and sushi domain-containing protein 2	Q7Z408	CSMD2	NA	Wald ratio	1	0.405	0.209	5.261E-02	0.904	pan	Hospitalized COVID-19
15486_126	ABP1	Amiloride-sensitive amine oxidase [copper-containing]	P19801	AOC1	NA	Inverse variance weighted	12	-0.034	0.017	5.288E-02	0.904	pan	Hospitalized COVID-19
9790_28	BR serine/threonine kinase 2	Serine/threonine-protein kinase BRSK2	Q8IWQ3	BRSK2	NA	Wald ratio	1	-0.128	0.066	5.355E-02	0.904	pan	Hospitalized COVID-19
14088_38	IGFBP-6	Insulin-like growth factor-binding protein 6	P24592	IGFBP6	NA	Inverse variance weighted	2	-0.223	0.116	5.395E-02	0.904	pan	Hospitalized COVID-19
13105_7	SNP25	Synaptosomal-associated protein 25	P60880	SNAP25	NA	Wald ratio	1	0.423	0.221	5.509E-02	0.904	pan	Hospitalized COVID-19
15322_35	CRADD	Death domain-containing protein CRADD	P78560	CRADD	NA	Inverse variance weighted	3	0.268	0.140	5.546E-02	0.904	pan	Hospitalized COVID-19
19614_8	Holo-TC I	Transcobalamin-1	P20061	TCN1	NA	Inverse variance weighted	12	-0.048	0.025	5.547E-02	0.904	pan	Hospitalized COVID-19
17785_11	RCL	2'-deoxynucleoside 5'-phosphate N-hydrolase 1	O43598	DNPH1	NA	Wald ratio	1	-0.271	0.142	5.620E-02	0.904	pan	Hospitalized COVID-19
19267_14	GLO2	Hydroxyacylglutathione hydrolase, mitochondrial	Q16775	HAGH	NA	Wald ratio	1	0.132	0.069	5.629E-02	0.904	pan	Hospitalized COVID-19
7228_2	SIA7F	Alpha-N-acetylgalactosaminide alpha-2,6-sialyltransferase 6	Q969X2	ST6GALNAC6	NA	Inverse variance weighted	3	-0.210	0.110	5.733E-02	0.904	pan	Hospitalized COVID-19
8053_16	DJB14:CD	DnaJ homolog subfamily B member 14:Cytoplasmic domain	Q8TBM8	DNAJB14	NA	Wald ratio	1	0.328	0.173	5.758E-02	0.904	pan	Hospitalized COVID-19
6372_7	YBOX2	Y-box-binding protein 2	Q9Y2T7	YBX2	NA	Inverse variance weighted	2	0.313	0.165	5.768E-02	0.904	pan	Hospitalized COVID-19
13580_2	Sperm-associated antigen 2	UDP-N-acetylhexosamine pyrophosphorylase	Q16222	UAP1	NA	Wald ratio	1	0.496	0.262	5.835E-02	0.904	pan	Hospitalized COVID-19
5638_23	GT251	Procollagen galactosyltransferase 1	Q8NBJ5	COLGALT1	NA	Inverse variance weighted	9	0.102	0.054	5.892E-02	0.904	pan	Hospitalized COVID-19
9590_10	COX7R	Cytochrome c oxidase subunit 7A-related protein, mitochondrial	O14548	COX7A2L	NA	Wald ratio	1	0.460	0.244	5.913E-02	0.904	pan	Hospitalized COVID-19
13126_52	DSC2	Desmocollin-2	Q02487	DSC2	NA	Inverse variance weighted	3	-0.104	0.055	6.006E-02	0.904	pan	Hospitalized COVID-19
7124_18	IL-21	Interleukin-21	Q9HBE4	IL21	NA	Wald ratio	1	0.292	0.155	6.026E-02	0.904	pan	Hospitalized COVID-19
5111_15	NRX3B	Neurexin-3-beta	Q9HDB5	NRXN3	NA	Inverse variance weighted	3	0.253	0.135	6.100E-02	0.904	pan	Hospitalized COVID-19
11608_5	MLP3B	Microtubule-associated proteins 1A/1B light chain 3B	Q9GZQ8	MAP1LC3B	NA	Wald ratio	1	-0.504	0.269	6.121E-02	0.904	pan	Hospitalized COVID-19
6462_12	TIMP-4	Metalloproteinase inhibitor 4	Q99727	TIMP4	NA	Inverse variance weighted	8	-0.063	0.033	6.158E-02	0.904	pan	Hospitalized COVID-19
3798_71	Carbonic anhydrase 9	Carbonic anhydrase 9	Q16790	CA9	NA	Wald ratio	1	0.596	0.319	6.185E-02	0.904	pan	Hospitalized COVID-19
11547_84	MUSK	Muscle, skeletal receptor tyrosine-protein kinase	O15146	MUSK	NA	Inverse variance weighted	2	0.251	0.134	6.188E-02	0.904	pan	Hospitalized COVID-19
7738_299	STX2	Syntaxin-2	P32856	STX2	NA	Inverse variance weighted	3	0.246	0.132	6.196E-02	0.904	pan	Hospitalized COVID-19
14309_8	HNRH1	Heterogeneous nuclear ribonucleoprotein H	P31943	HNRNPH1	NA	Wald ratio	1	0.416	0.223	6.215E-02	0.904	pan	Hospitalized COVID-19
19206_20	8ODP	7,8-dihydro-8-oxoguanine triphosphatase	P36639	NUDT1	NA	Wald ratio	1	0.182	0.098	6.296E-02	0.904	pan	Hospitalized COVID-19
7849_3	Glutaminyl cyclase	Glutaminyl-peptide cyclotransferase	Q16769	QPCT	NA	Inverse variance weighted	7	-0.081	0.044	6.315E-02	0.904	pan	Hospitalized COVID-19
15545_13	Calcineurin B a	Calcineurin subunit B type 1	P63098	PPP3R1	NA	Wald ratio	1	-0.315	0.170	6.418E-02	0.904	pan	Hospitalized COVID-19
12707_26	DPYL3	Dihydropyrimidinase-related protein 3	Q14195	DPYSL3	NA	Inverse variance weighted	2	0.191	0.103	6.501E-02	0.904	pan	Hospitalized COVID-19
18244_1	ANXA7	Annexin A7	P20073	ANXA7	NA	Wald ratio	1	0.446	0.243	6.602E-02	0.904	pan	Hospitalized COVID-19
5238_26	PPIE	Peptidyl-prolyl cis-trans isomerase E	Q9UNP9	PPIE	NA	Inverse variance weighted	3	0.066	0.036	6.628E-02	0.904	pan	Hospitalized COVID-19
11493_169	DYL2	Dynein light chain 2, cytoplasmic	Q96FJ2	DYNLL2	NA	Wald ratio	1	-0.368	0.201	6.668E-02	0.904	pan	Hospitalized COVID-19
11568_2	FKB1B	Peptidyl-prolyl cis-trans isomerase FKBP1B	P68106	FKBP1B	NA	Wald ratio	1	0.165	0.090	6.773E-02	0.904	pan	Hospitalized COVID-19
8221_19	MIF	Macrophage migration inhibitory factor	P14174	MIF	NA	Wald ratio	1	0.433	0.238	6.886E-02	0.904	pan	Hospitalized COVID-19
19279_42	CRBP	Retinol-binding protein 1	P09455	RBP1	NA	Wald ratio	1	0.283	0.156	6.890E-02	0.904	pan	Hospitalized COVID-19
7926_13	SPIT3	Kunitz-type protease inhibitor 3	P49223	SPINT3	NA	Inverse variance weighted	4	0.123	0.067	6.917E-02	0.904	pan	Hospitalized COVID-19
9002_36	SPA11	Serpin A11	Q86U17	SERPINA11	NA	Inverse variance weighted	7	-0.154	0.085	6.975E-02	0.904	pan	Hospitalized COVID-19
2823_7	COMMD7	COMM domain-containing protein 7	Q86VX2	COMMD7	NA	Wald ratio	1	-0.203	0.112	6.999E-02	0.904	pan	Hospitalized COVID-19
4140_3	IL-7	Interleukin-7	P13232	IL7	NA	Wald ratio	1	0.412	0.228	7.002E-02	0.904	pan	Hospitalized COVID-19
4156_74	TGF-b2	Transforming growth factor beta-2	P61812	TGFB2	NA	Wald ratio	1	-0.405	0.224	7.038E-02	0.904	pan	Hospitalized COVID-19
5091_28	ILT-4	Leukocyte immunoglobulin-like receptor subfamily B member 2	Q8N423	LILRB2	NA	Inverse variance weighted	3	-0.270	0.150	7.095E-02	0.904	pan	Hospitalized COVID-19
2211_9	TIMP-1	Metalloproteinase inhibitor 1	P01033	TIMP1	NA	Wald ratio	1	0.324	0.180	7.196E-02	0.904	pan	Hospitalized COVID-19
5735_54	C1GLC	C1GALT1-specific chaperone 1	Q96EU7	C1GALT1C1	NA	Inverse variance weighted	3	0.099	0.055	7.235E-02	0.904	pan	Hospitalized COVID-19
6641_60	PolyUbiquitin K48	PolyUbiquitin K48-linked	P0CG47	UBB	NA	Wald ratio	1	-0.361	0.201	7.248E-02	0.904	pan	Hospitalized COVID-19

15398_2	HERV1	FAD-linked sulfhydryl oxidase ALR	P55789	GFER	NA	Wald ratio	1	-0.323	0.180	7.329E-02	0.904	pan	Hospitalized COVID-19
6414_8	OAF	Out at first protein homolog	Q86UD1	OAF	NA	Inverse variance weighted	6	0.058	0.033	7.396E-02	0.904	pan	Hospitalized COVID-19
8346_9	DPP2	Dipeptidyl peptidase 2	Q9UHL4	DPP7	NA	Inverse variance weighted	7	-0.063	0.035	7.429E-02	0.904	pan	Hospitalized COVID-19
3535_84	DKK1	Dickkopf-related protein 1	O94907	DKK1	NA	Wald ratio	1	0.384	0.216	7.517E-02	0.904	pan	Hospitalized COVID-19
10008_43	GUC1A	Guanylyl cyclase-activating protein 1	P43080	GUCA1A	NA	Inverse variance weighted	2	0.371	0.210	7.628E-02	0.904	pan	Hospitalized COVID-19
5358_3	OMD	Osteomodulin	Q99983	OMD	NA	Inverse variance weighted	3	0.202	0.114	7.693E-02	0.904	pan	Hospitalized COVID-19
12940_35	AL3B1	Aldehyde dehydrogenase family 3 member B1	P43353	ALDH3B1	NA	Wald ratio	1	0.161	0.091	7.719E-02	0.904	pan	Hospitalized COVID-19
9484_75	Desmoglein-2	Desmoglein-2	Q14126	DSG2	NA	Inverse variance weighted	10	0.078	0.044	7.730E-02	0.904	pan	Hospitalized COVID-19
5605_77	MFNG	Beta-1,3-N-acetylglucosaminyltransferase manic fringe	O00587	MFNG	NA	Inverse variance weighted	2	0.231	0.131	7.733E-02	0.904	pan	Hospitalized COVID-19
8005_1	MXRA7	Matrix-remodeling-associated protein 7	P84157	MXRA7	NA	Inverse variance weighted	5	-0.055	0.031	7.745E-02	0.904	pan	Hospitalized COVID-19
18381_16	ALDH-E2	Aldehyde dehydrogenase, mitochondrial	P05091	ALDH2	NA	Inverse variance weighted	4	0.132	0.075	7.747E-02	0.904	pan	Hospitalized COVID-19
6930_95	SIA8F	Alpha-2,8-sialyltransferase 8F	P61647	ST8SIA6	NA	Wald ratio	1	-0.365	0.207	7.750E-02	0.904	pan	Hospitalized COVID-19
9713_67	PGFRL	Platelet-derived growth factor receptor-like protein	Q15198	PDGFRL	NA	Inverse variance weighted	6	-0.064	0.036	7.755E-02	0.904	pan	Hospitalized COVID-19
10702_1	COSA1	Collagen alpha-1(XXVIII) chain	Q2UY09	COL28A1	NA	Inverse variance weighted	2	0.274	0.156	7.783E-02	0.904	pan	Hospitalized COVID-19
3077_66	Coagulation Factor Xa	Coagulation factor Xa	P00742	F10	NA	Inverse variance weighted	4	-0.220	0.125	7.828E-02	0.904	pan	Hospitalized COVID-19
17828_3	S100A14	Protein S100-A14	Q9HCY8	S100A14	NA	Wald ratio	1	0.379	0.216	7.934E-02	0.904	pan	Hospitalized COVID-19
4831_4	sL-Selectin	L-Selectin	P14151	SELL	NA	Inverse variance weighted	8	-0.063	0.036	7.989E-02	0.904	pan	Hospitalized COVID-19
16317_20	Desmoglein-3	Desmoglein-3	P32926	DSG3	NA	Inverse variance weighted	2	-0.282	0.161	7.990E-02	0.904	pan	Hospitalized COVID-19
15321_8	CPLX2	Complexin-2	Q6PUV4	CPLX2	NA	Wald ratio	1	0.394	0.225	8.015E-02	0.904	pan	Hospitalized COVID-19
7923_41	SEM4C	Semaphorin-4C	Q9C0C4	SEMA4C	NA	Inverse variance weighted	10	0.122	0.070	8.043E-02	0.904	pan	Hospitalized COVID-19
9359_9	EGFL9	Protein delta homolog 2	Q6UY11	DLK2	NA	Wald ratio	1	-0.103	0.059	8.067E-02	0.904	pan	Hospitalized COVID-19
7866_11	DJC30	DnaJ homolog subfamily C member 30	Q96LL9	DNAJC30	NA	Inverse variance weighted	2	-0.115	0.066	8.274E-02	0.904	pan	Hospitalized COVID-19
13733_5	IL-12 p40	Interleukin-12 subunit beta	P29460	IL12B	NA	Inverse variance weighted	16	-0.068	0.039	8.316E-02	0.904	pan	Hospitalized COVID-19
10647_18	ASCC1	Activating signal cointegrator 1 complex subunit 1	Q8N9N2	ASCC1	NA	Wald ratio	1	-0.323	0.187	8.322E-02	0.904	pan	Hospitalized COVID-19
15544_25	kallikrein 14	Kallikrein-14	Q9P0G3	KLK14	NA	Inverse variance weighted	5	-0.162	0.094	8.353E-02	0.904	pan	Hospitalized COVID-19
6416_8	GKN2	Gastrokine-2	Q86XP6	GKN2	NA	Inverse variance weighted	10	-0.080	0.046	8.367E-02	0.904	pan	Hospitalized COVID-19
12437_18	ULK3	Serine/threonine-protein kinase ULK3	Q6PHR2	ULK3	NA	Wald ratio	1	0.361	0.209	8.371E-02	0.904	pan	Hospitalized COVID-19
4982_54	Elafin	Elafin	P19957	PI3	NA	Inverse variance weighted	7	0.087	0.050	8.379E-02	0.904	pan	Hospitalized COVID-19
16304_6	LGR4	Leucine-rich repeat-containing G-protein coupled receptor 4	Q9BXB1	LGR4	NA	Inverse variance weighted	3	0.164	0.095	8.384E-02	0.904	pan	Hospitalized COVID-19
12641_3	I5P2	Type II inositol 1,4,5-trisphosphate 5-phosphatase	P32019	INPP5B	NA	Inverse variance weighted	2	0.081	0.047	8.391E-02	0.904	pan	Hospitalized COVID-19
2611_72	Dtk	Tyrosine-protein kinase receptor TYRO3	Q06418	TYRO3	NA	Inverse variance weighted	2	0.176	0.102	8.404E-02	0.904	pan	Hospitalized COVID-19
8035_6	CA198	Uncharacterized protein C1orf198	Q9H425	C1orf198	NA	Wald ratio	1	0.154	0.089	8.419E-02	0.904	pan	Hospitalized COVID-19
12428_2	LYPL1	Lysophospholipase-like protein 1	Q5VWZ2	LYPLAL1	NA	Wald ratio	1	0.172	0.100	8.432E-02	0.904	pan	Hospitalized COVID-19
3600_2	Chitotriosidase-1	Chitotriosidase-1	Q13231	CHIT1	NA	Inverse variance weighted	8	0.033	0.019	8.441E-02	0.904	pan	Hospitalized COVID-19
15305_7	Secretagogin	Secretagogin	O76038	SCGN	NA	Inverse variance weighted	2	0.196	0.114	8.463E-02	0.904	pan	Hospitalized COVID-19
6715_63	PPBN	Alkaline phosphatase, placental-like	P10696	ALPG	NA	Inverse variance weighted	3	0.153	0.089	8.668E-02	0.904	pan	Hospitalized COVID-19
18295_102	GRHPR	Glyoxylate reductase/hydroxypyruvate reductase	Q9UBQ7	GRHPR	NA	Inverse variance weighted	3	-0.063	0.037	8.669E-02	0.904	pan	Hospitalized COVID-19
13961_18	KIF3A	Kinesin-like protein KIF3A	Q9Y496	KIF3A	NA	Wald ratio	1	-0.342	0.200	8.724E-02	0.904	pan	Hospitalized COVID-19
13724_27	FGF-19	Fibroblast growth factor 19	O95750	FGF19	NA	Inverse variance weighted	2	0.201	0.117	8.726E-02	0.904	pan	Hospitalized COVID-19
3168_8	ADAMTS-5	A disintegrin and metalloproteinase with thrombospondin motifs 5	Q9UNA0	ADAMTS5	NA	Inverse variance weighted	4	0.049	0.029	8.732E-02	0.904	pan	Hospitalized COVID-19
7638_30	Lectin, mannose-binding 2	Vesicular integral-membrane protein VIP36	Q12907	LMAN2	NA	Inverse variance weighted	2	0.266	0.156	8.759E-02	0.904	pan	Hospitalized COVID-19
9960_2	TBCD5	TBC1 domain family member 5	Q92609	TBC1D5	NA	Wald ratio	1	-0.339	0.198	8.764E-02	0.904	pan	Hospitalized COVID-19
13597_20	RAB31	Ras-related protein Rab-31	Q13636	RAB31	NA	Inverse variance weighted	3	0.243	0.142	8.790E-02	0.904	pan	Hospitalized COVID-19
11431_235	RECQ1	ATP-dependent DNA helicase Q1	P46063	RECQL	NA	Inverse variance weighted	10	0.035	0.020	8.846E-02	0.904	pan	Hospitalized COVID-19
3331_8	RGMB	RGM domain family member B	Q6NW40	RGMB	NA	Wald ratio	1	-0.371	0.218	8.868E-02	0.904	pan	Hospitalized COVID-19
11207_3	Macrophage scavenger receptor:CD	Macrophage scavenger receptor types I and II:Cytoplasmic domain	P21757	MSR1	NA	Wald ratio	1	0.646	0.380	8.913E-02	0.904	pan	Hospitalized COVID-19
5742_14	PPA6	Lysophosphatidic acid phosphatase type 6	Q9NPH0	ACP6	NA	Inverse variance weighted	22	-0.028	0.016	8.934E-02	0.904	pan	Hospitalized COVID-19
6367_66	fibromodulin	fibromodulin	Q06828	FMOD	NA	Inverse variance weighted	5	0.102	0.060	8.946E-02	0.904	pan	Hospitalized COVID-19
14636_25	Ribonuclease UK114	Ribonuclease UK114	P52758	RIDA	NA	Inverse variance weighted	4	-0.072	0.042	8.948E-02	0.904	pan	Hospitalized COVID-19
4430_44	Collectin Kidney 1	Collectin-11	Q9BWP8	COLEC11	NA	Inverse variance weighted	11	0.088	0.052	9.004E-02	0.904	pan	Hospitalized COVID-19
11542_11	TM230	Transmembrane protein 230	Q96A57	TMEM230	NA	Wald ratio	1	-0.261	0.154	9.006E-02	0.904	pan	Hospitalized COVID-19
10015_119	KCAB2	Voltage-gated potassium channel subunit beta-2	Q13303	KCNAB2	NA	Inverse variance weighted	2	-0.226	0.134	9.063E-02	0.905	pan	Hospitalized COVID-19
4568_17	SLIK5	SLIT and NTRK-like protein 5	O94991	SLITRK5	NA	Inverse variance weighted	4	0.107	0.063	9.100E-02	0.905	pan	Hospitalized COVID-19
8989_40	SCUB1	Signal peptide, CUB and EGF-like domain-containing protein 1	Q8IWY4	SCUBE1	NA	Inverse variance weighted	6	-0.092	0.054	9.148E-02	0.905	pan	Hospitalized COVID-19
17377_1	Aldose reductase-like C3	Aldo-keto reductase family 1 member C3	P42330	AKR1C3	NA	Inverse variance weighted	9	-0.074	0.044	9.462E-02	0.917	pan	Hospitalized COVID-19
11145_72	K154L	UPF0606 protein KIAA1549L	Q6ZVL6	KIAA1549L	NA	Inverse variance weighted	7	-0.050	0.030	9.500E-02	0.917	pan	Hospitalized COVID-19
5879_51	Dynactin subunit 2	Dynactin subunit 2	Q13561	DCTN2	NA	Wald ratio	1	-0.322	0.193	9.573E-02	0.917	pan	Hospitalized COVID-19
19173_5	ZFAN1	AN1-type zinc finger protein 1	Q8TCF1	ZFAND1	NA	Inverse variance weighted	4	0.095	0.057	9.656E-02	0.917	pan	Hospitalized COVID-19
3366_51	ECM1	Extracellular matrix protein 1	Q16610	ECM1	NA	Inverse variance weighted	14	-0.039	0.024	9.667E-02	0.917	pan	Hospitalized COVID-19
19617_5	LTB4DH	Prostaglandin reductase 1	Q14914	PTGR1	NA	Inverse variance weighted	12	-0.035	0.021	9.677E-02	0.917	pan	Hospitalized COVID-19
12449_16	PPIH	Peptidyl-prolyl cis-trans isomerase H	O43447	PPIH	NA	Wald ratio	1	-0.247	0.149	9.697E-02	0.917	pan	Hospitalized COVID-19
15560_52	Apo-TC II	Transcobalamin-2	P20062	TCN2	NA	Inverse variance weighted	10	0.037	0.023	9.722E-02	0.917	pan	Hospitalized COVID-19
15427_35	LOXL3	Lysyl oxidase homolog 3	P58215	LOXL3	NA	Inverse variance weighted	11	-0.043	0.026	9.730E-02	0.917	pan	Hospitalized COVID-19
6904_14	LRRT2	Leucine-rich repeat transmembrane neuronal protein 2	O43300	LRRTM2	NA	Inverse variance weighted	3	0.135	0.082	9.812E-02	0.917	pan	Hospitalized COVID-19
8310_6	U773	Zymogen granule protein 16 homolog B	Q96DA0	ZG16B	NA	Wald ratio	1	0.107	0.065	9.836E-02	0.917	pan	Hospitalized COVID-19
19223_6	RAB1A	Ras-related protein Rab-1A	P62820	RAB1A	NA	Inverse variance weighted	2	-0.256	0.155	9.876E-02	0.917	pan	Hospitalized COVID-19
10621_26	PRR16	Protein Largen	Q569H4	PRR16	NA	Inverse variance weighted	4	0.181	0.110	9.913E-02	0.917	pan	Hospitalized COVID-19
12630_8	ARFP2	Arfaptin-2	P53365	ARFIP2	NA	Inverse variance weighted	5	-0.143	0.087	9.937E-02	0.917	pan	Hospitalized COVID-19
3457_57	Periostin	Periostin	Q15063	POSTN	NA	Inverse variance weighted	4	0.072	0.044	9.989E-02	0.917	pan	Hospitalized COVID-19
10772_21	CGAT2	Chondroitin sulfate N-acetylgalactosaminyltransferase 2	Q8N6G5	CSGALNACT2	NA	Wald ratio	1	0.177	0.107	9.996E-02	0.917	pan	Hospitalized COVID-19
8275_31	PEAR1:ECD	Platelet endothelial aggregation receptor 1:Extracellular domain	Q5VY43	PEAR1	NA	Inverse variance weighted	2	-0.154	0.094	1.000E-01	0.917	pan	Hospitalized COVID-19
19482_11	HDHD3	Haloacid dehalogenase-like hydrolase domain-containing protein 3	Q9BSH5	HDHD3	NA	Wald ratio	1	0.091	0.055	1.003E-01	0.917	pan	Hospitalized COVID-19
6925_26	SNX8	Sorting nexin-8	Q9Y5X2	SNX8	NA	Inverse variance weighted	2	0.097	0.059	1.008E-01	0.917	pan	Hospitalized COVID-19
5129_12	SREC-I	Scavenger receptor class F member 1	Q14162	SCARF1	NA	Inverse variance weighted	5	0.043	0.026	1.014E-01	0.917	pan	Hospitalized COVID-19
19129_15	MTHFSD	Methenyltetrahydrofolate synthase domain-containing protein	Q2M296	MTHFSD	NA	Inverse variance weighted	5	-0.051	0.031	1.018E-01	0.917	pan	Hospitalized COVID-19
11428_31	PDLI1	PDZ and LIM domain protein 1	O00151	PDLIM1	NA	Wald ratio	1	-0.242	0.148	1.020E-01	0.917	pan	Hospitalized COVID-19
12494_99	GBRL2	Gamma-aminobutyric acid receptor-associated protein-like 2	P60520	GABARAPL2	NA	Inverse variance weighted	2	0.453	0.278	1.024E-01	0.917	pan	Hospitalized COVID-19
16916_19	SLIK6	SLIT and NTRK-like protein 6	Q9H5Y7	SLITRK6	NA	Inverse variance weighted	6	-0.079	0.049	1.033E-01	0.917	pan	Hospitalized COVID-19

15364_101	Apo C-I	Apolipoprotein C-I	P02654	APOC1	NA	Inverse variance weighted	2	-0.364	0.223	1.035E-01	0.917	pan	Hospitalized COVID-19
14294_61	MBD1	Methyl-CpG-binding domain protein 1	Q9UIS9	MBD1	NA	Inverse variance weighted	3	0.201	0.123	1.037E-01	0.917	pan	Hospitalized COVID-19
12616_45	NRBP	Nuclear receptor-binding protein	Q9UHY1	NRBP1	NA	Wald ratio	1	0.135	0.083	1.041E-01	0.917	pan	Hospitalized COVID-19
6039_24	CRHBP	Corticotropin-releasing factor-binding protein	P24387	CRHBP	NA	Inverse variance weighted	8	0.037	0.023	1.057E-01	0.927	pan	Hospitalized COVID-19
3024_18	a2-Antiplasmin	Alpha-2-antiplasmin	P08697	SERPINF2	NA	Inverse variance weighted	6	0.107	0.066	1.062E-01	0.928	pan	Hospitalized COVID-19
17419_17	TES	Testin	Q9UGI8	TES	NA	Wald ratio	1	-0.219	0.135	1.066E-01	0.928	pan	Hospitalized COVID-19
13422_66	ENOX2	Ecto-NOX disulfide-thiol exchanger 2	Q16206	ENOX2	NA	Wald ratio	1	0.392	0.244	1.077E-01	0.934	pan	Hospitalized COVID-19
12522_6	RD23B	UV excision repair protein RAD23 homolog B	P54727	RAD23B	NA	Wald ratio	1	0.294	0.183	1.082E-01	0.934	pan	Hospitalized COVID-19
7161_25	G6PE	GDH/6PGL endoplasmic bifunctional protein	O95479	H6PD	NA	Inverse variance weighted	19	-0.034	0.021	1.091E-01	0.934	pan	Hospitalized COVID-19
11352_42	TITIN	Titin	Q8WZ42	TTN	NA	Wald ratio	1	0.373	0.233	1.093E-01	0.934	pan	Hospitalized COVID-19
2742_68	Siglec-7	Sialic acid-binding Ig-like lectin 7	Q9Y286	SIGLEC7	NA	Inverse variance weighted	3	0.117	0.074	1.108E-01	0.934	pan	Hospitalized COVID-19
12396_19	HIBCH	3-hydroxyisobutyryl-CoA hydrolase, mitochondrial	Q6NVY1	HIBCH	NA	Inverse variance weighted	6	0.045	0.028	1.114E-01	0.934	pan	Hospitalized COVID-19
10521_10	MXRA8:ECD	Matrix-remodeling-associated protein 8:Extracellular domain	Q9BRK3	MXRA8	NA	Inverse variance weighted	3	-0.140	0.088	1.120E-01	0.934	pan	Hospitalized COVID-19
17512_2	NMI	N-myc-interactor	Q13287	NMI	NA	Wald ratio	1	0.440	0.277	1.123E-01	0.934	pan	Hospitalized COVID-19
11656_110	EVL	Ena/VASP-like protein	Q9UI08	EVL	NA	Inverse variance weighted	3	0.181	0.114	1.126E-01	0.934	pan	Hospitalized COVID-19
17756_69	DCTD	Deoxycytidylate deaminase	P32321	DCTD	NA	Inverse variance weighted	2	0.184	0.116	1.127E-01	0.934	pan	Hospitalized COVID-19
7779_86	CHSTB	Carbohydrate sulfotransferase 11	Q9NPF2	CHST11	NA	Inverse variance weighted	5	0.057	0.036	1.127E-01	0.934	pan	Hospitalized COVID-19
8994_65	SLAMF8	SLAM family member 8	Q9P0V8	SLAMF8	NA	Inverse variance weighted	4	0.143	0.090	1.132E-01	0.934	pan	Hospitalized COVID-19
12016_60	CBL	E3 ubiquitin-protein ligase CBL	P22681	CBL	NA	Wald ratio	1	0.113	0.072	1.143E-01	0.934	pan	Hospitalized COVID-19
4924_32	MMP-1	Interstitial collagenase	P03956	MMP1	NA	Inverse variance weighted	12	0.045	0.028	1.148E-01	0.934	pan	Hospitalized COVID-19
8982_65	TSP3	Thrombospondin-3	P49746	THBS3	NA	Inverse variance weighted	4	-0.171	0.109	1.153E-01	0.934	pan	Hospitalized COVID-19
4157_2	Thrombin	Thrombin	P00734	F2	NA	Inverse variance weighted	5	0.096	0.061	1.160E-01	0.934	pan	Hospitalized COVID-19
17672_184	Gastric intrinsic factor	Gastric intrinsic factor	P27352	CBLIF	NA	Inverse variance weighted	13	-0.045	0.028	1.164E-01	0.934	pan	Hospitalized COVID-19
17821_20	NMT2	Glycylpeptide N-tetradecanoyltransferase 2	O60551	NMT2	NA	Wald ratio	1	0.253	0.161	1.165E-01	0.934	pan	Hospitalized COVID-19
4978_54	DBNL	Drebrin-like protein	Q9UJU6	DBNL	NA	Wald ratio	1	0.262	0.167	1.168E-01	0.934	pan	Hospitalized COVID-19
10754_113	Prokineticin-2	Prokineticin-2	Q9HC23	PROK2	NA	Inverse variance weighted	9	-0.048	0.031	1.178E-01	0.934	pan	Hospitalized COVID-19
13460_4	CHAD	Chondroadherin	O15335	CHAD	NA	Inverse variance weighted	2	-0.197	0.126	1.181E-01	0.934	pan	Hospitalized COVID-19
16919_1	ACBP	Acyl-CoA-binding protein	P07108	DBI	NA	Inverse variance weighted	4	0.179	0.115	1.198E-01	0.934	pan	Hospitalized COVID-19
18841_1	SPB13	Serpin B13	Q9UIV8	SERPINB13	NA	Inverse variance weighted	4	0.081	0.052	1.202E-01	0.934	pan	Hospitalized COVID-19
9557_5	MANS1	MANSC domain-containing protein 1	Q9H8J5	MANSC1	NA	Inverse variance weighted	5	0.076	0.049	1.208E-01	0.934	pan	Hospitalized COVID-19
9212_22	CATF	Cathepsin F	Q9UBX1	CTSF	NA	Inverse variance weighted	3	0.209	0.135	1.210E-01	0.934	pan	Hospitalized COVID-19
4459_68	PCSK7	Proprotein convertase subtilisin/kexin type 7	Q16549	PCSK7	NA	Inverse variance weighted	14	-0.033	0.022	1.213E-01	0.934	pan	Hospitalized COVID-19
4455_89	MFGM	Lactadherin	Q08431	MFGE8	NA	Inverse variance weighted	4	-0.012	0.008	1.215E-01	0.934	pan	Hospitalized COVID-19
5599_88	PCYXL	Prenylcysteine oxidase-like	Q8NBM8	PCYOX1L	NA	Wald ratio	1	-0.224	0.145	1.216E-01	0.934	pan	Hospitalized COVID-19
16792_4	Siglec-5	Sialic acid-binding Ig-like lectin 5	O15389	SIGLEC5	NA	Inverse variance weighted	7	0.046	0.029	1.217E-01	0.934	pan	Hospitalized COVID-19
18401_18	ALKB3	Alpha-ketoglutarate-dependent dioxygenase alkB homolog 3	Q96Q83	ALKBH3	NA	Inverse variance weighted	6	-0.064	0.041	1.219E-01	0.934	pan	Hospitalized COVID-19
16828_8	Collagen a1(VI)	Collagen alpha-1(VI) chain	P12109	COL6A1	NA	Inverse variance weighted	14	-0.033	0.021	1.220E-01	0.934	pan	Hospitalized COVID-19
9126_171	NT5D3	5'-nucleotidase domain-containing protein 3	Q86UY8	NT5DC3	NA	Inverse variance weighted	3	0.069	0.044	1.223E-01	0.934	pan	Hospitalized COVID-19
12968_2	CSRP2	Cysteine and glycine-rich protein 2	Q16527	CSRP2	NA	Wald ratio	1	0.303	0.197	1.234E-01	0.934	pan	Hospitalized COVID-19
9314_9	PSG5	Pregnancy-specific beta-1-glycoprotein 5	Q15238	PSG5	NA	Inverse variance weighted	4	0.153	0.099	1.236E-01	0.934	pan	Hospitalized COVID-19
8903_1	COX6C	Cytochrome c oxidase subunit 6C	P09669	COX6C	NA	Inverse variance weighted	4	0.151	0.098	1.245E-01	0.934	pan	Hospitalized COVID-19
19334_62	TXD12	Thioredoxin domain-containing protein 12	O95881	TXNDC12	NA	Wald ratio	1	0.191	0.124	1.246E-01	0.934	pan	Hospitalized COVID-19
5481_16	RASA1	Ras GTPase-activating protein 1	P20936	RASA1	NA	Inverse variance weighted	2	-0.247	0.161	1.259E-01	0.934	pan	Hospitalized COVID-19
13610_9	MAGE-10	Melanoma-associated antigen 10	P43363	MAGEA10	NA	Inverse variance weighted	2	-0.238	0.156	1.270E-01	0.934	pan	Hospitalized COVID-19
6373_54	DLK1	Protein delta homolog 1	P80370	DLK1	NA	Inverse variance weighted	3	-0.228	0.150	1.276E-01	0.934	pan	Hospitalized COVID-19
19237_17	D-dopachrome decarboxylase	D-dopachrome decarboxylase	P30046	DDT	NA	Inverse variance weighted	9	-0.099	0.065	1.277E-01	0.934	pan	Hospitalized COVID-19
4249_64	NDP kinase B	Nucleoside diphosphate kinase B	P22392	NME2	NA	Wald ratio	1	0.115	0.075	1.278E-01	0.934	pan	Hospitalized COVID-19
8074_32	TMM70	Transmembrane protein 70, mitochondrial	Q9BUB7	TMEM70	NA	Inverse variance weighted	3	0.179	0.118	1.284E-01	0.934	pan	Hospitalized COVID-19
19293_6	VP26A	Vacuolar protein sorting-associated protein 26A	O75436	VPS26A	NA	Inverse variance weighted	3	-0.102	0.067	1.286E-01	0.934	pan	Hospitalized COVID-19
11540_37	FOXO3A	Forkhead box protein O3	O43524	FOXO3	NA	Wald ratio	1	-0.308	0.203	1.287E-01	0.934	pan	Hospitalized COVID-19
8840_61	C1s	Complement C1s subcomponent	P09871	C1S	NA	Inverse variance weighted	3	0.168	0.111	1.294E-01	0.934	pan	Hospitalized COVID-19
13388_57	NEC1	Neuroendocrine convertase 1	P29120	PCSK1	NA	Inverse variance weighted	11	0.046	0.030	1.295E-01	0.934	pan	Hospitalized COVID-19
12820_1	GRAP	GRB2-related adapter protein	Q13588	GRAP	NA	Inverse variance weighted	2	-0.220	0.145	1.302E-01	0.934	pan	Hospitalized COVID-19
2567_5	Factor I	Complement factor I	P05156	CFI	NA	Inverse variance weighted	2	0.097	0.064	1.303E-01	0.934	pan	Hospitalized COVID-19
9288_7	FKBP7	Peptidyl-prolyl cis-trans isomerase FKBP7	Q9Y680	FKBP7	NA	Inverse variance weighted	7	0.047	0.031	1.310E-01	0.934	pan	Hospitalized COVID-19
12713_365	RGS19	Regulator of G-protein signaling 19	P49795	RGS19	NA	Wald ratio	1	0.312	0.207	1.316E-01	0.934	pan	Hospitalized COVID-19
5090_49	ILT-2	Leukocyte immunoglobulin-like receptor subfamily B member 1	Q8NHL6	LILRB1	NA	Inverse variance weighted	10	-0.048	0.032	1.317E-01	0.934	pan	Hospitalized COVID-19
17398_55	HO-1	Heme oxygenase 1	P09601	HMOX1	NA	Inverse variance weighted	9	-0.078	0.052	1.330E-01	0.934	pan	Hospitalized COVID-19
5900_11	HINT1	Histidine triad nucleotide-binding protein 1	P49773	HINT1	NA	Wald ratio	1	0.216	0.144	1.331E-01	0.934	pan	Hospitalized COVID-19
5400_52	sLeptin R	Leptin receptor, soluble	P48357	LEPR	NA	Inverse variance weighted	18	0.029	0.019	1.334E-01	0.934	pan	Hospitalized COVID-19
2860_19	Karyopherin-a2	Importin subunit alpha-1	P52292	KPNA2	NA	Inverse variance weighted	10	-0.097	0.065	1.342E-01	0.934	pan	Hospitalized COVID-19
9478_69	KPRA	Phosphoribosyl pyrophosphate synthase-associated protein 1	Q14558	PRPSAP1	NA	Wald ratio	1	-0.232	0.155	1.344E-01	0.934	pan	Hospitalized COVID-19
9834_62	ADH1B	Alcohol dehydrogenase 1B	P00325	ADH1B	NA	Wald ratio	1	0.136	0.091	1.349E-01	0.934	pan	Hospitalized COVID-19
8971_9	NRX1A	Neurexin-1	Q9ULB1	NRXN1	NA	Inverse variance weighted	6	-0.126	0.084	1.351E-01	0.934	pan	Hospitalized COVID-19
10490_3	RPN1:CD	Dolichyl-diphosphooligosaccharide--protein glycosyltransferase subunit 1:Cytoplasmic domain	P04843	RPN1	NA	Wald ratio	1	-0.115	0.077	1.355E-01	0.934	pan	Hospitalized COVID-19
9341_1	PDGFD	Platelet-derived growth factor D	Q9GZP0	PDGFD	NA	Inverse variance weighted	3	-0.166	0.112	1.368E-01	0.934	pan	Hospitalized COVID-19
13113_7	Osteopontin	Osteopontin	P10451	SPP1	NA	Inverse variance weighted	5	0.083	0.056	1.369E-01	0.934	pan	Hospitalized COVID-19
3028_36	Ck-b-8-1	Ck-beta-8-1	P55773	CCL23	NA	Inverse variance weighted	3	-0.153	0.103	1.371E-01	0.934	pan	Hospitalized COVID-19
15607_56	KPYR	Pyruvate kinase PKLR	P30613	PKLR	NA	Inverse variance weighted	3	-0.198	0.134	1.375E-01	0.934	pan	Hospitalized COVID-19
19187_21	STABP	STAM-binding protein	O95630	STAMBP	NA	Inverse variance weighted	2	0.211	0.142	1.380E-01	0.934	pan	Hospitalized COVID-19
3035_80	IL-19	Interleukin-19	Q9UHD0	IL19	NA	Inverse variance weighted	5	-0.104	0.070	1.380E-01	0.934	pan	Hospitalized COVID-19
17820_170	MLF1	Myeloid leukemia factor 1	P58340	MLF1	NA	Inverse variance weighted	4	-0.185	0.125	1.389E-01	0.934	pan	Hospitalized COVID-19
3323_37	LRP8	Low-density lipoprotein receptor-related protein 8	Q14114	LRP8	NA	Inverse variance weighted	4	0.088	0.060	1.389E-01	0.934	pan	Hospitalized COVID-19
11825_27	PRGC1	Peroxisome proliferator-activated receptor gamma coactivator 1-alpha	Q9UBK2	PPARGC1A	NA	Wald ratio	1	0.323	0.218	1.390E-01	0.934	pan	Hospitalized COVID-19
3206_4	LYVE1	Lymphatic vessel endothelial hyaluronic acid receptor 1	Q9Y5Y7	LYVE1	NA	Inverse variance weighted	21	0.058	0.039	1.391E-01	0.934	pan	Hospitalized COVID-19
9867_23	F16P2	Fructose-1,6-bisphosphatase isozyme 2	O00757	FBP2	NA	Wald ratio	1	0.177	0.120	1.392E-01	0.934	pan	Hospitalized COVID-19
18337_4	GMDS	GDP-mannose 4,6 dehydratase	O60547	GMDS	NA	Wald ratio	1	-0.292	0.198	1.393E-01	0.934	pan	Hospitalized COVID-19
2750_3	Apo A-I	Apolipoprotein A-I	P02647	APOA1	NA	Inverse variance weighted	8	-0.065	0.044	1.393E-01	0.934	pan	Hospitalized COVID-19

14007_22	PAPS1	Bifunctional 3'-phosphoadenosine 5'-phosphosulfate synthase 1	O43252	PAPSS1	NA	Inverse variance weighted	2	-0.097	0.065	1.394E-01	0.934	pan	Hospitalized COVID-19
15363_32	Apo A-V	Apolipoprotein A-V	Q6Q788	APOA5	NA	Inverse variance weighted	11	0.052	0.035	1.395E-01	0.934	pan	Hospitalized COVID-19
17675_17	ACO13	Acyl-coenzyme A thioesterase 13	Q9NPJ3	ACOT13	NA	Wald ratio	1	0.312	0.211	1.399E-01	0.934	pan	Hospitalized COVID-19
12558_3	UBS3B	Ubiquitin-associated and SH3 domain-containing protein B	Q8TF42	UBASH3B	NA	Inverse variance weighted	2	-0.110	0.075	1.406E-01	0.934	pan	Hospitalized COVID-19
3045_72	PTN	Pleiotrophin	P21246	PTN	NA	Inverse variance weighted	4	-0.072	0.049	1.410E-01	0.934	pan	Hospitalized COVID-19
15511_37	NPTXR	Neuronal pentraxin receptor	O95502	NPTXR	NA	Inverse variance weighted	10	-0.032	0.022	1.415E-01	0.934	pan	Hospitalized COVID-19
3391_10	PK3CG	Phosphatidylinositol 4,5-bisphosphate 3-kinase catalytic subunit gamma isoform	P48736	PIK3CG	NA	Wald ratio	1	-0.354	0.241	1.415E-01	0.934	pan	Hospitalized COVID-19
7178_59	DEPP	Protein DEPP	Q9NTK1	DEPP1	NA	Inverse variance weighted	6	-0.085	0.058	1.415E-01	0.934	pan	Hospitalized COVID-19
10053_5	ILK1	Integrin-linked protein kinase	Q13418	ILK	NA	Wald ratio	1	0.269	0.183	1.428E-01	0.936	pan	Hospitalized COVID-19
12498_12	TX1B3	Tax1-binding protein 3	O14907	TAX1BP3	NA	Wald ratio	1	0.169	0.116	1.429E-01	0.936	pan	Hospitalized COVID-19
7921_65	FJX1	Four-jointed box protein 1	Q86VR8	FJX1	NA	Inverse variance weighted	4	0.060	0.041	1.432E-01	0.936	pan	Hospitalized COVID-19
5636_10	MFAP4	Microfibril-associated glycoprotein 4	P55083	MFAP4	NA	Inverse variance weighted	11	0.067	0.046	1.441E-01	0.937	pan	Hospitalized COVID-19
17148_7	BLVRB	Flavin reductase (NADPH)	P30043	BLVRB	NA	Inverse variance weighted	2	-0.132	0.090	1.442E-01	0.937	pan	Hospitalized COVID-19
15470_11	Hexosaminidase B	Beta-hexosaminidase subunit beta	P07686	HEXB	NA	Inverse variance weighted	12	0.051	0.035	1.448E-01	0.937	pan	Hospitalized COVID-19
15394_79	UNC5B	Netrin receptor UNC5B	Q8IZJ1	UNC5B	NA	Inverse variance weighted	4	0.104	0.071	1.456E-01	0.937	pan	Hospitalized COVID-19
11601_26	DHX8	ATP-dependent RNA helicase DHX8	Q14562	DHX8	NA	Inverse variance weighted	4	0.117	0.080	1.459E-01	0.937	pan	Hospitalized COVID-19
8447_11	ghrelin	Appetite-regulating hormone	Q9UBU3	GHRL	NA	Inverse variance weighted	2	-0.217	0.149	1.460E-01	0.937	pan	Hospitalized COVID-19
7211_2	RNase 1	Ribonuclease pancreatic	P07998	RNASE1	NA	Inverse variance weighted	6	-0.100	0.069	1.467E-01	0.937	pan	Hospitalized COVID-19
11277_23	ATF6A	Cyclic AMP-dependent transcription factor ATF-6 alpha	P18850	ATF6	NA	Inverse variance weighted	10	-0.081	0.056	1.470E-01	0.937	pan	Hospitalized COVID-19
16558_2	MYOC	Myocilin	Q99972	MYOC	NA	Inverse variance weighted	9	0.041	0.028	1.481E-01	0.937	pan	Hospitalized COVID-19
9451_20	Uromodulin	Uromodulin	P07911	UMOD	NA	Wald ratio	1	-0.387	0.268	1.488E-01	0.937	pan	Hospitalized COVID-19
8993_151	RIPK2	Receptor-interacting serine/threonine-protein kinase 2	O43353	RIPK2	NA	Wald ratio	1	-0.246	0.171	1.492E-01	0.937	pan	Hospitalized COVID-19
19601_15	ASB9	Ankyrin repeat and SOCS box protein 9	Q96DX5	ASB9	NA	Wald ratio	1	-0.086	0.060	1.493E-01	0.937	pan	Hospitalized COVID-19
3340_53	TSP4	Thrombospondin-4	P35443	THBS4	NA	Inverse variance weighted	4	-0.065	0.045	1.494E-01	0.937	pan	Hospitalized COVID-19
3807_1	FGF23	Fibroblast growth factor 23	Q9GZV9	FGF23	NA	Wald ratio	1	-0.286	0.198	1.494E-01	0.937	pan	Hospitalized COVID-19
6629_3	HBD-1	Beta-defensin 1	P60022	DEFB1	NA	Inverse variance weighted	10	-0.040	0.028	1.502E-01	0.939	pan	Hospitalized COVID-19
7769_29	3BP2	SH3 domain-binding protein 2	P78314	SH3BP2	NA	Inverse variance weighted	2	0.097	0.068	1.513E-01	0.941	pan	Hospitalized COVID-19
5601_2	PGRP-L	N-acetylmuramoyl-L-alanine amidase	Q96PD5	PGLYRP2	NA	Inverse variance weighted	6	-0.074	0.051	1.521E-01	0.941	pan	Hospitalized COVID-19
16593_3	FADD	FAS-associated death domain protein	Q13158	FADD	NA	Inverse variance weighted	2	-0.260	0.181	1.521E-01	0.941	pan	Hospitalized COVID-19
6538_90	K2013	Uncharacterized protein KIAA2013	Q8IYS2	KIAA2013	NA	Inverse variance weighted	2	0.215	0.150	1.522E-01	0.941	pan	Hospitalized COVID-19
5078_82	EphB6	Ephrin type-B receptor 6	O15197	EPHB6	NA	Inverse variance weighted	4	0.066	0.046	1.527E-01	0.941	pan	Hospitalized COVID-19
8269_327	ARSK	Arylsulfatase K	Q6UWY0	ARSK	NA	Inverse variance weighted	2	0.166	0.116	1.530E-01	0.941	pan	Hospitalized COVID-19
4913_78	HCC-4	C-C motif chemokine 16	O15467	CCL16	NA	Inverse variance weighted	8	0.093	0.066	1.548E-01	0.945	pan	Hospitalized COVID-19
13393_46	DERL1	Derlin-1	Q9BUN8	DERL1	NA	Wald ratio	1	0.316	0.222	1.548E-01	0.945	pan	Hospitalized COVID-19
13954_9	GNA1	Glucosamine 6-phosphate N-acetyltransferase	Q96EK6	GNPNAT1	NA	Wald ratio	1	-0.255	0.180	1.556E-01	0.945	pan	Hospitalized COVID-19
2700_56	Protein S	Vitamin K-dependent protein S	P07225	PROS1	NA	Inverse variance weighted	2	-0.208	0.147	1.566E-01	0.945	pan	Hospitalized COVID-19
15635_4	SMOC2	SPARC-related modular calcium-binding protein 2	Q9H3U7	SMOC2	NA	Inverse variance weighted	6	-0.046	0.033	1.568E-01	0.945	pan	Hospitalized COVID-19
13107_9	LYPD3	Ly6/PLAUR domain-containing protein 3	O95274	LYPD3	NA	Inverse variance weighted	3	-0.143	0.101	1.568E-01	0.945	pan	Hospitalized COVID-19
17329_2	BDH2	3-hydroxybutyrate dehydrogenase type 2	Q9BUT1	BDH2	NA	Inverse variance weighted	2	0.088	0.062	1.572E-01	0.945	pan	Hospitalized COVID-19
15298_199	NETO1	Neuropilin and tolloid-like protein 1	Q8TDF5	NETO1	NA	Inverse variance weighted	2	-0.210	0.148	1.573E-01	0.945	pan	Hospitalized COVID-19
4775_34	Gelsolin	Gelsolin	P06396	GSN	NA	Inverse variance weighted	3	-0.146	0.103	1.575E-01	0.945	pan	Hospitalized COVID-19
9026_40	BTNL8	Butyrophilin-like protein 8	Q6UX41	BTNL8	NA	Inverse variance weighted	2	-0.090	0.064	1.586E-01	0.945	pan	Hospitalized COVID-19
6895_1	TR:CD	Transferrin receptor protein 1:Cytoplasmic domain	P02786	TFRC	NA	Inverse variance weighted	3	0.120	0.085	1.586E-01	0.945	pan	Hospitalized COVID-19
2752_62	BMP-14	Growth/differentiation factor 5	P43026	GDF5	NA	Inverse variance weighted	2	0.155	0.110	1.592E-01	0.945	pan	Hospitalized COVID-19
17156_72	DCAK1	Serine/threonine-protein kinase DCLK1	O15075	DCLK1	NA	Inverse variance weighted	6	0.062	0.044	1.593E-01	0.945	pan	Hospitalized COVID-19
17735_130	GBRAP	Gamma-aminobutyric acid receptor-associated protein	O95166	GABARAP	NA	Wald ratio	1	0.292	0.208	1.605E-01	0.950	pan	Hospitalized COVID-19
18285_6	SVIP	Small VCP/p97-interacting protein	Q8NHG7	SVIP	NA	Inverse variance weighted	2	0.279	0.199	1.616E-01	0.950	pan	Hospitalized COVID-19
2991_9	IL-1 sRI	Interleukin-1 receptor type 1	P14778	IL1R1	NA	Inverse variance weighted	8	-0.074	0.053	1.618E-01	0.950	pan	Hospitalized COVID-19
2631_50	IL-10 Rb	Interleukin-10 receptor subunit beta	Q08334	IL10RB	NA	Wald ratio	1	0.232	0.166	1.619E-01	0.950	pan	Hospitalized COVID-19
8759_29	a1,4-Galactosyltransferase	Lactosylceramide 4-alpha-galactosyltransferase	Q9NPC4	A4GALT	NA	Inverse variance weighted	16	0.033	0.024	1.628E-01	0.953	pan	Hospitalized COVID-19
4989_7	Fibrinogen g-chain dimer	Fibrinogen gamma chain	P02679	FGG	NA	Wald ratio	1	0.238	0.171	1.634E-01	0.953	pan	Hospitalized COVID-19
5722_78	Prolylcarboxypeptidase	Lysosomal Pro-X carboxypeptidase	P42785	PRCP	NA	Inverse variance weighted	8	0.072	0.052	1.638E-01	0.953	pan	Hospitalized COVID-19
13682_47	M-CSF R	Macrophage colony-stimulating factor 1 receptor	P07333	CSF1R	NA	Inverse variance weighted	5	-0.128	0.092	1.653E-01	0.955	pan	Hospitalized COVID-19
17682_1	CD46	Membrane cofactor protein	P15529	CD46	NA	Inverse variance weighted	3	0.076	0.055	1.654E-01	0.955	pan	Hospitalized COVID-19
13101_60	SOST	Sclerostin	Q9BQB4	SOST	NA	Inverse variance weighted	3	0.129	0.094	1.677E-01	0.955	pan	Hospitalized COVID-19
9215_117	UBP25	Ubiquitin carboxyl-terminal hydrolase 25	Q9UHP3	USP25	NA	Wald ratio	1	-0.471	0.342	1.683E-01	0.955	pan	Hospitalized COVID-19
2974_61	contactin-1	Contactin-1	Q12860	CNTN1	NA	Inverse variance weighted	7	-0.050	0.036	1.686E-01	0.955	pan	Hospitalized COVID-19
17364_8	RU2B	U2 small nuclear ribonucleoprotein B''	P08579	SNRPB2	NA	Wald ratio	1	-0.377	0.274	1.690E-01	0.955	pan	Hospitalized COVID-19
10396_6	Mcl-1	Induced myeloid leukemia cell differentiation protein Mcl-1	Q07820	MCL1	NA	Inverse variance weighted	2	-0.218	0.159	1.695E-01	0.955	pan	Hospitalized COVID-19
13590_1	ORN	Oligoribonuclease, mitochondrial	Q9Y3B8	REXO2	NA	Inverse variance weighted	2	-0.157	0.114	1.696E-01	0.955	pan	Hospitalized COVID-19
18301_10	NAIF1	Nuclear apoptosis-inducing factor 1	Q69YI7	NAIF1	NA	Inverse variance weighted	3	-0.139	0.101	1.697E-01	0.955	pan	Hospitalized COVID-19
14150_7	IL-1F6	Interleukin-36 alpha	Q9UHA7	IL36A	NA	Inverse variance weighted	2	-0.188	0.137	1.705E-01	0.955	pan	Hospitalized COVID-19
11360_39	RRM1	Ribonucleoside-diphosphate reductase large subunit	P23921	RRM1	NA	Inverse variance weighted	16	-0.053	0.039	1.706E-01	0.955	pan	Hospitalized COVID-19
4906_35	Coagulation Factor V	Coagulation Factor V	P12259	F5	NA	Inverse variance weighted	3	-0.167	0.122	1.707E-01	0.955	pan	Hospitalized COVID-19
9995_6	DUT	Deoxyuridine 5'-triphosphate nucleotidohydrolase, mitochondrial	P33316	DUT	NA	Wald ratio	1	0.224	0.164	1.707E-01	0.955	pan	Hospitalized COVID-19
5404_53	DR6	Tumor necrosis factor receptor superfamily member 21	O75509	TNFRSF21	NA	Inverse variance weighted	2	-0.137	0.100	1.717E-01	0.955	pan	Hospitalized COVID-19
8459_10	BMP-6	Bone morphogenetic protein 6	P22004	BMP6	NA	Inverse variance weighted	3	-0.133	0.098	1.725E-01	0.955	pan	Hospitalized COVID-19
17514_48	RAB21	Ras-related protein Rab-21	Q9UL25	RAB21	NA	Wald ratio	1	-0.155	0.114	1.729E-01	0.955	pan	Hospitalized COVID-19
12687_2	DECR	2,4-dienoyl-CoA reductase, mitochondrial	Q16698	DECR1	NA	Inverse variance weighted	6	-0.116	0.085	1.733E-01	0.955	pan	Hospitalized COVID-19
5202_4	PPID	Peptidyl-prolyl cis-trans isomerase D	Q08752	PPID	NA	Inverse variance weighted	5	-0.051	0.037	1.737E-01	0.955	pan	Hospitalized COVID-19
17739_1	HCDH	Hydroxyacyl-coenzyme A dehydrogenase, mitochondrial	Q16836	HADH	NA	Wald ratio	1	-0.200	0.147	1.737E-01	0.955	pan	Hospitalized COVID-19
4588_1	PH	Pancreatic hormone	P01298	PPY	NA	Inverse variance weighted	9	-0.066	0.048	1.742E-01	0.955	pan	Hospitalized COVID-19
5728_60	FCRL1	Fc receptor-like protein 1	Q96LA6	FCRL1	NA	Inverse variance weighted	10	-0.067	0.049	1.745E-01	0.955	pan	Hospitalized COVID-19
17832_12	IDI2	Isopentenyl-diphosphate delta-isomerase 2	Q9BXS1	IDI2	NA	Inverse variance weighted	2	0.089	0.066	1.747E-01	0.955	pan	Hospitalized COVID-19
15383_200	Endothelin 3	Endothelin-3	P14138	EDN3	NA	Wald ratio	1	-0.335	0.247	1.751E-01	0.955	pan	Hospitalized COVID-19
8014_359	MANEA	Glycoprotein endo-alpha-1,2-mannosidase	Q5SRI9	MANEA	NA	Inverse variance weighted	19	-0.021	0.015	1.752E-01	0.955	pan	Hospitalized COVID-19
3169_70	IDUA	Alpha-L-iduronidase	P35475	IDUA	NA	Inverse variance weighted	6	0.039	0.029	1.755E-01	0.955	pan	Hospitalized COVID-19
4324_33	CYTT	Cystatin-SA	P09228	CST2	NA	Inverse variance weighted	9	0.074	0.055	1.759E-01	0.955	pan	Hospitalized COVID-19

2994_71	IL-1Rrp2	Interleukin-1 receptor-like 2	Q9HB29	IL1RL2	NA	Wald ratio	1	-0.098	0.072	1.767E-01	0.955	pan	Hospitalized COVID-19
11481_25	TIMD3	Hepatitis A virus cellular receptor 2	Q8TDQ0	HAVCR2	NA	Wald ratio	1	0.323	0.239	1.768E-01	0.955	pan	Hospitalized COVID-19
5107_7	Notch 1	Neurogenic locus notch homolog protein 1	P46531	NOTCH1	NA	Inverse variance weighted	3	0.123	0.091	1.770E-01	0.955	pan	Hospitalized COVID-19
18162_167	IRAK4	Interleukin-1 receptor-associated kinase 4	Q9NWZ3	IRAK4	NA	Wald ratio	1	-0.173	0.129	1.781E-01	0.955	pan	Hospitalized COVID-19
4141_79	IP-10	C-X-C motif chemokine 10	P02778	CXCL10	NA	Inverse variance weighted	2	0.218	0.162	1.781E-01	0.955	pan	Hospitalized COVID-19
14090_23	DEFI6	Differentially expressed in FDCP 6 homolog	Q9H4E7	DEF6	NA	Inverse variance weighted	2	-0.145	0.108	1.783E-01	0.955	pan	Hospitalized COVID-19
17698_15	WBP2	WW domain-binding protein 2	Q969T9	WBP2	NA	Wald ratio	1	0.358	0.266	1.785E-01	0.955	pan	Hospitalized COVID-19
3316_58	Heparin cofactor II	Heparin cofactor 2	P05546	SERPIND1	NA	Wald ratio	1	0.280	0.208	1.795E-01	0.956	pan	Hospitalized COVID-19
6415_90	CPN2	Carboxypeptidase N subunit 2	P22792	CPN2	NA	Inverse variance weighted	6	0.088	0.066	1.804E-01	0.956	pan	Hospitalized COVID-19
19117_3	PP14A	Protein phosphatase 1 regulatory subunit 14A	Q96A00	PPP1R14A	NA	Inverse variance weighted	2	0.189	0.141	1.810E-01	0.956	pan	Hospitalized COVID-19
15533_97	Macrophage scavenger receptor:ECD	Macrophage scavenger receptor types I and II:Extracellular domain	P21757	MSR1	NA	Inverse variance weighted	7	-0.076	0.057	1.811E-01	0.956	pan	Hospitalized COVID-19
15558_63	AMPE	Glutamyl aminopeptidase	Q07075	ENPEP	NA	Inverse variance weighted	8	0.037	0.028	1.826E-01	0.956	pan	Hospitalized COVID-19
10833_64	HHIP	Hedgehog-interacting protein	Q96QV1	HHIP	NA	Inverse variance weighted	3	-0.078	0.059	1.828E-01	0.956	pan	Hospitalized COVID-19
12556_7	UBE2C	Ubiquitin-conjugating enzyme E2 C	O00762	UBE2C	NA	Inverse variance weighted	2	0.123	0.093	1.829E-01	0.956	pan	Hospitalized COVID-19
8100_15	ADM2	ADM2	Q7Z4H4	ADM2	NA	Wald ratio	1	0.288	0.216	1.829E-01	0.956	pan	Hospitalized COVID-19
5628_21	SEM3G	Semaphorin-3G	Q9NS98	SEMA3G	NA	Inverse variance weighted	7	-0.104	0.078	1.832E-01	0.956	pan	Hospitalized COVID-19
6998_106	HAAH	Aspartyl/asparaginyl beta-hydroxylase	Q12797	ASPH	NA	Inverse variance weighted	3	-0.117	0.088	1.838E-01	0.956	pan	Hospitalized COVID-19
2968_61	TNFSF15	Tumor necrosis factor ligand superfamily member 15	O95150	TNFSF15	NA	Wald ratio	1	-0.251	0.189	1.845E-01	0.956	pan	Hospitalized COVID-19
12575_30	C1TC	C-1-tetrahydrofolate synthase, cytoplasmic	P11586	MTHFD1	NA	Inverse variance weighted	5	0.103	0.078	1.846E-01	0.956	pan	Hospitalized COVID-19
11205_10	Integrin beta-7	Integrin beta-7	P26010	ITGB7	NA	Wald ratio	1	0.295	0.222	1.848E-01	0.956	pan	Hospitalized COVID-19
5593_11	PDIA5	Protein disulfide-isomerase A5	Q14554	PDIA5	NA	Inverse variance weighted	11	-0.042	0.032	1.850E-01	0.956	pan	Hospitalized COVID-19
2977_7	EDAR	Tumor necrosis factor receptor superfamily member EDAR	Q9UNE0	EDAR	NA	Inverse variance weighted	15	-0.028	0.021	1.855E-01	0.956	pan	Hospitalized COVID-19
7957_2	SCG3	Secretogranin-3	Q8WXD2	SCG3	NA	Inverse variance weighted	10	-0.038	0.029	1.876E-01	0.956	pan	Hospitalized COVID-19
9094_5	CLC4C	C-type lectin domain family 4 member C	Q8WTT0	CLEC4C	NA	Inverse variance weighted	19	-0.067	0.051	1.877E-01	0.956	pan	Hospitalized COVID-19
4152_58	Prekallikrein	Plasma kallikrein	P03952	KLKB1	NA	Inverse variance weighted	4	0.127	0.096	1.888E-01	0.956	pan	Hospitalized COVID-19
13416_8	T132D	Transmembrane protein 132D	Q14C87	TMEM132D	NA	Inverse variance weighted	5	0.082	0.062	1.890E-01	0.956	pan	Hospitalized COVID-19
15515_2	SAA	Serum amyloid A-1 protein	P0DJI8	SAA1	NA	Inverse variance weighted	5	-0.102	0.078	1.890E-01	0.956	pan	Hospitalized COVID-19
17748_21	QORX	Quinone oxidoreductase PIG3	Q53FA7	TP53I3	NA	Inverse variance weighted	10	-0.050	0.038	1.891E-01	0.956	pan	Hospitalized COVID-19
6626_81	CHSTC	Carbohydrate sulfotransferase 12	Q9NRB3	CHST12	NA	Inverse variance weighted	2	-0.073	0.056	1.898E-01	0.956	pan	Hospitalized COVID-19
9312_8	AZGP1	Zinc-alpha-2-glycoprotein	P25311	AZGP1	NA	Inverse variance weighted	4	-0.190	0.145	1.903E-01	0.956	pan	Hospitalized COVID-19
10902_53	APRV1	Retroviral-like aspartic protease 1	Q53RT3	ASPRV1	NA	Inverse variance weighted	3	-0.142	0.109	1.905E-01	0.956	pan	Hospitalized COVID-19
9765_4	NDE1	Nuclear distribution protein nudE homolog 1	Q9NXR1	NDE1	NA	Wald ratio	1	0.191	0.146	1.909E-01	0.956	pan	Hospitalized COVID-19
15653_9	COAA1	Collagen alpha-1(X) chain	Q03692	COL10A1	NA	Inverse variance weighted	4	-0.068	0.052	1.921E-01	0.956	pan	Hospitalized COVID-19
7128_9	VWA2	von Willebrand factor A domain-containing protein 2	Q5GFL6	VWA2	NA	Inverse variance weighted	7	0.069	0.053	1.922E-01	0.956	pan	Hospitalized COVID-19
9326_33	ITI heavy chain H2	Inter-alpha-trypsin inhibitor heavy chain H2	P19823	ITIH2	NA	Inverse variance weighted	10	0.066	0.050	1.927E-01	0.956	pan	Hospitalized COVID-19
12817_1	GEM	GTP-binding protein GEM	P55040	GEM	NA	Wald ratio	1	-0.373	0.287	1.938E-01	0.956	pan	Hospitalized COVID-19
6064_4	TXNDC4	Endoplasmic reticulum resident protein 44	Q9BS26	ERP44	NA	Inverse variance weighted	3	0.169	0.130	1.939E-01	0.956	pan	Hospitalized COVID-19
11120_49	NAT14	N-acetyltransferase 14	Q8WUY8	NAT14	NA	Wald ratio	1	0.122	0.094	1.944E-01	0.956	pan	Hospitalized COVID-19
11510_31	Apo L1	Apolipoprotein L1	O14791	APOL1	NA	Inverse variance weighted	8	-0.036	0.028	1.947E-01	0.956	pan	Hospitalized COVID-19
9962_1	MUCDL	Cadherin-related family member 5	Q9HBB8	CDHR5	NA	Wald ratio	1	-0.236	0.182	1.950E-01	0.956	pan	Hospitalized COVID-19
19176_27	FA49B	Protein FAM49B	Q9NUQ9	CYRIB	NA	Wald ratio	1	0.282	0.217	1.951E-01	0.956	pan	Hospitalized COVID-19
9884_8	PPIL1	Peptidyl-prolyl cis-trans isomerase-like 1	Q9Y3C6	PPIL1	NA	Inverse variance weighted	9	0.038	0.029	1.960E-01	0.956	pan	Hospitalized COVID-19
6551_94	Vaspin	Serpin A12	Q8IW75	SERPINA12	NA	Inverse variance weighted	6	-0.035	0.027	1.972E-01	0.956	pan	Hospitalized COVID-19
5087_5	IL-22BP	Interleukin-22 receptor subunit alpha-2	Q969J5	IL22RA2	NA	Inverse variance weighted	19	0.052	0.040	1.973E-01	0.956	pan	Hospitalized COVID-19
9482_110	NUDT9	ADP-ribose pyrophosphatase, mitochondrial	Q9BW91	NUDT9	NA	Wald ratio	1	-0.222	0.172	1.977E-01	0.956	pan	Hospitalized COVID-19
8427_118	RSPO3	R-spondin-3	Q9BXY4	RSPO3	NA	Inverse variance weighted	4	-0.165	0.128	1.978E-01	0.956	pan	Hospitalized COVID-19
7989_5	CA226	Uncharacterized protein C1orf226	A1L170	C1orf226	NA	Wald ratio	1	0.220	0.171	1.980E-01	0.956	pan	Hospitalized COVID-19
4874_3	Angiogenin	Angiogenin	P03950	ANG	NA	Inverse variance weighted	6	0.073	0.057	1.982E-01	0.956	pan	Hospitalized COVID-19
17495_141	SIR3	NAD-dependent protein deacetylase sirtuin-3, mitochondrial	Q9NTG7	SIRT3	NA	Inverse variance weighted	3	-0.115	0.089	1.984E-01	0.956	pan	Hospitalized COVID-19
18909_11	EXOS8	Exosome complex component RRP43	Q96B26	EXOSC8	NA	Wald ratio	1	-0.197	0.153	1.989E-01	0.956	pan	Hospitalized COVID-19
17726_3	SAR1A	GTP-binding protein SAR1a	Q9NR31	SAR1A	NA	Inverse variance weighted	2	0.132	0.103	1.989E-01	0.956	pan	Hospitalized COVID-19
9256_78	NPTX1	Neuronal pentraxin-1	Q15818	NPTX1	NA	Inverse variance weighted	9	-0.041	0.032	1.992E-01	0.956	pan	Hospitalized COVID-19
17332_3	ARHL2	Poly(ADP-ribose) glycohydrolase ARH3	Q9NX46	ADPRS	NA	Inverse variance weighted	5	-0.069	0.054	1.996E-01	0.956	pan	Hospitalized COVID-19
3184_25	Coagulation Factor VII	Coagulation factor VII	P08709	F7	NA	Inverse variance weighted	10	0.029	0.023	2.006E-01	0.956	pan	Hospitalized COVID-19
19602_36	jun-D	Transcription factor jun-D	P17535	JUND	NA	Wald ratio	1	-0.266	0.208	2.011E-01	0.956	pan	Hospitalized COVID-19
15316_262	TXN4B	Thioredoxin-like protein 4B	Q9NX01	TXNL4B	NA	Inverse variance weighted	3	0.150	0.117	2.012E-01	0.956	pan	Hospitalized COVID-19
6049_64	PTPRS	Receptor-type tyrosine-protein phosphatase S	Q13332	PTPRS	NA	Inverse variance weighted	4	-0.110	0.087	2.018E-01	0.956	pan	Hospitalized COVID-19
9038_12	JTB	Protein JTB	O76095	JTB	NA	Wald ratio	1	-0.232	0.182	2.019E-01	0.956	pan	Hospitalized COVID-19
8299_66	LIRA4	Leukocyte immunoglobulin-like receptor subfamily A member 4	P59901	LILRA4	NA	Inverse variance weighted	8	0.103	0.081	2.019E-01	0.956	pan	Hospitalized COVID-19
14012_17	LGP2	Probable ATP-dependent RNA helicase DHX58	Q96C10	DHX58	NA	Inverse variance weighted	3	-0.097	0.076	2.030E-01	0.956	pan	Hospitalized COVID-19
14684_17	CAN2	Calpain-2 catalytic subunit	P17655	CAPN2	NA	Inverse variance weighted	2	-0.166	0.130	2.034E-01	0.956	pan	Hospitalized COVID-19
9197_4	LEG9	Galectin-9	O00182	LGALS9	NA	Wald ratio	1	-0.118	0.093	2.035E-01	0.956	pan	Hospitalized COVID-19
7857_22	NEUT	Neurotensin/neuromedin N	P30990	NTS	NA	Inverse variance weighted	5	0.081	0.064	2.037E-01	0.956	pan	Hospitalized COVID-19
18310_26	SELW	Selenoprotein W	P63302	SELENOW	NA	Inverse variance weighted	8	0.050	0.040	2.048E-01	0.956	pan	Hospitalized COVID-19
9249_17	TMEM9:ECD	Transmembrane protein 9:Extracellular domain	Q9P0T7	TMEM9	NA	Inverse variance weighted	2	-0.149	0.118	2.048E-01	0.956	pan	Hospitalized COVID-19
15529_33	Cysteine-rich protein 1	Cysteine and glycine-rich protein 1	P21291	CSRP1	NA	Inverse variance weighted	2	-0.181	0.143	2.056E-01	0.956	pan	Hospitalized COVID-19
18921_30	PHEX	Phosphate-regulating neutral endopeptidase	P78562	PHEX	NA	Wald ratio	1	-0.167	0.132	2.061E-01	0.956	pan	Hospitalized COVID-19
5621_64	THSD1	Thrombospondin type-1 domain-containing protein 1	Q9NS62	THSD1	NA	Inverse variance weighted	13	0.028	0.023	2.062E-01	0.956	pan	Hospitalized COVID-19
11241_8	ARLY	Argininosuccinate lyase	P04424	ASL	NA	Inverse variance weighted	7	-0.098	0.078	2.067E-01	0.956	pan	Hospitalized COVID-19
5716_49	CSAG1	Putative chondrosarcoma-associated gene 1 protein	Q6PB30	CSAG1	NA	Inverse variance weighted	3	0.130	0.103	2.072E-01	0.956	pan	Hospitalized COVID-19
6392_7	WISP-2	WNT1-inducible-signaling pathway protein 2	O76076	CCN5	NA	Inverse variance weighted	8	0.037	0.030	2.072E-01	0.956	pan	Hospitalized COVID-19
5124_69	sICAM-5	Intercellular adhesion molecule 5	Q9UMF0	ICAM5	NA	Inverse variance weighted	7	-0.088	0.070	2.075E-01	0.956	pan	Hospitalized COVID-19
11369_23	ADHX	Alcohol dehydrogenase class-3	P11766	ADH5	NA	Inverse variance weighted	4	-0.162	0.129	2.077E-01	0.956	pan	Hospitalized COVID-19
3004_67	PD-L2	Programmed cell death 1 ligand 2	Q9BQ51	PDCD1LG2	NA	Inverse variance weighted	6	-0.035	0.028	2.084E-01	0.956	pan	Hospitalized COVID-19
5963_9	Dermokine	Dermokine	Q6E0U4	DMKN	NA	Wald ratio	1	-0.241	0.192	2.093E-01	0.956	pan	Hospitalized COVID-19
8795_48	TR:ECD	Transferrin receptor protein 1:Extracellular domain	P02786	TFRC	NA	Inverse variance weighted	5	0.080	0.064	2.094E-01	0.956	pan	Hospitalized COVID-19
18312_68	NDRG3	Protein NDRG3	Q9UGV2	NDRG3	NA	Inverse variance weighted	3	-0.132	0.106	2.103E-01	0.956	pan	Hospitalized COVID-19
17671_58	ING4	Inhibitor of growth protein 4	Q9UNL4	ING4	NA	Inverse variance weighted	4	0.152	0.122	2.107E-01	0.956	pan	Hospitalized COVID-19

15641_20	TEFF1	Tomoregulin-1	Q8IYR6	TMEFF1	NA	Wald ratio	1	-0.224	0.179	2.113E-01	0.956	pan	Hospitalized COVID-19
14156_33	14-3-3 protein beta/alpha	14-3-3 protein beta/alpha	P31946	YWHAB	NA	Wald ratio	1	0.229	0.184	2.122E-01	0.956	pan	Hospitalized COVID-19
12382_2	DDX58	Probable ATP-dependent RNA helicase DDX58	O95786	DDX58	NA	Inverse variance weighted	3	0.071	0.057	2.123E-01	0.956	pan	Hospitalized COVID-19
12605_1	Exosome component 3	Exosome complex component RRP40	Q9NQT5	EXOSC3	NA	Wald ratio	1	0.255	0.205	2.127E-01	0.956	pan	Hospitalized COVID-19
9901_28	EGLN1	Egl nine homolog 1	Q9GZT9	EGLN1	NA	Inverse variance weighted	7	-0.064	0.052	2.133E-01	0.956	pan	Hospitalized COVID-19
7009_8	CD72	B-cell differentiation antigen CD72	P21854	CD72	NA	Inverse variance weighted	3	0.316	0.255	2.141E-01	0.956	pan	Hospitalized COVID-19
15513_108	Prostasin	Prostasin	Q16651	PRSS8	NA	Wald ratio	1	0.269	0.217	2.144E-01	0.956	pan	Hospitalized COVID-19
14094_29	HB-EGF	Heparin-binding EGF-like growth factor	Q99075	HBEGF	NA	Inverse variance weighted	8	-0.106	0.085	2.145E-01	0.956	pan	Hospitalized COVID-19
10056_5	FOXM1	Forkhead box protein M1	Q08050	FOXM1	NA	Wald ratio	1	0.158	0.127	2.153E-01	0.956	pan	Hospitalized COVID-19
8397_147	QSOX2	Sulfhydryl oxidase 2	Q6ZRP7	QSOX2	NA	Inverse variance weighted	16	0.027	0.022	2.173E-01	0.956	pan	Hospitalized COVID-19
3827_22	PAK6	Serine/threonine-protein kinase PAK 6	Q9NQU5	PAK6	NA	Wald ratio	1	-0.076	0.061	2.175E-01	0.956	pan	Hospitalized COVID-19
15487_164	carboxylesterase, liver	Liver carboxylesterase 1	P23141	CES1	NA	Wald ratio	1	0.269	0.218	2.175E-01	0.956	pan	Hospitalized COVID-19
5749_53	COL	Colipase	P04118	CLPS	NA	Inverse variance weighted	8	-0.032	0.026	2.184E-01	0.956	pan	Hospitalized COVID-19
2730_58	MICA	MHC class I polypeptide-related sequence A	Q29983	MICA	NA	Inverse variance weighted	13	-0.051	0.041	2.188E-01	0.956	pan	Hospitalized COVID-19
18380_78	Albumin	Serum albumin	P02768	ALB	NA	Inverse variance weighted	2	0.204	0.166	2.193E-01	0.956	pan	Hospitalized COVID-19
15449_33	TIM-4	T-cell immunoglobulin and mucin domain-containing protein 4	Q96H15	TIMD4	NA	Inverse variance weighted	19	-0.051	0.042	2.194E-01	0.956	pan	Hospitalized COVID-19
3194_36	GPVI	Platelet glycoprotein VI	Q9HCN6	GP6	NA	Inverse variance weighted	2	-0.135	0.110	2.198E-01	0.956	pan	Hospitalized COVID-19
16785_45	HD-5	Defensin-5	Q01523	DEFA5	NA	Inverse variance weighted	17	-0.053	0.044	2.204E-01	0.956	pan	Hospitalized COVID-19
13545_97	EIF1A	Probable RNA-binding protein EIF1AD	Q8N9N8	EIF1AD	NA	Inverse variance weighted	3	0.135	0.110	2.204E-01	0.956	pan	Hospitalized COVID-19
4155_3	Tenascin	Tenascin	P24821	TNC	NA	Inverse variance weighted	12	-0.040	0.032	2.210E-01	0.956	pan	Hospitalized COVID-19
2652_15	suPAR	Urokinase plasminogen activator surface receptor	Q03405	PLAUR	NA	Wald ratio	1	-0.321	0.262	2.214E-01	0.956	pan	Hospitalized COVID-19
2972_57	BMP-7	Bone morphogenetic protein 7	P18075	BMP7	NA	Wald ratio	1	0.224	0.184	2.214E-01	0.956	pan	Hospitalized COVID-19
16596_25	GLRX3	Glutaredoxin-3	O76003	GLRX3	NA	Wald ratio	1	-0.156	0.127	2.215E-01	0.956	pan	Hospitalized COVID-19
2665_26	BCMA	Tumor necrosis factor receptor superfamily member 17	Q02223	TNFRSF17	NA	Inverse variance weighted	11	0.060	0.049	2.225E-01	0.956	pan	Hospitalized COVID-19
10600_24	CLGN	Calmegin	O14967	CLGN	NA	Inverse variance weighted	3	-0.137	0.112	2.227E-01	0.956	pan	Hospitalized COVID-19
4996_66	HRG	Histidine-rich glycoprotein	P04196	HRG	NA	Inverse variance weighted	8	0.069	0.056	2.227E-01	0.956	pan	Hospitalized COVID-19
12395_86	SYDM	Aspartate--tRNA ligase, mitochondrial	Q6PI48	DARS2	NA	Wald ratio	1	0.262	0.215	2.229E-01	0.956	pan	Hospitalized COVID-19
9744_139	DNJA4	DnaJ homolog subfamily A member 4	Q8WW22	DNAJA4	NA	Wald ratio	1	0.211	0.173	2.233E-01	0.956	pan	Hospitalized COVID-19
19446_1	GMPR2	GMP reductase 2	Q9P2T1	GMPR2	NA	Inverse variance weighted	2	0.057	0.047	2.236E-01	0.956	pan	Hospitalized COVID-19
10722_13	KSYK:Protein Kinase	Tyrosine-protein kinase SYK:Protein kinase domain	P43405	SYK	NA	Wald ratio	1	0.254	0.209	2.238E-01	0.956	pan	Hospitalized COVID-19
8817_29	CENPV	Centromere protein V	Q7Z7K6	CENPV	NA	Wald ratio	1	-0.262	0.216	2.239E-01	0.956	pan	Hospitalized COVID-19
9848_22	Cyclin H	Cyclin-H	P51946	CCNH	NA	Wald ratio	1	-0.148	0.122	2.239E-01	0.956	pan	Hospitalized COVID-19
2741_22	Siglec-6	Sialic acid-binding Ig-like lectin 6	O43699	SIGLEC6	NA	Inverse variance weighted	7	-0.057	0.047	2.267E-01	0.964	pan	Hospitalized COVID-19
14618_26	ECOP	Vesicular, overexpressed in cancer, prosurvival protein 1	Q96AW1	VOPP1	NA	Inverse variance weighted	10	-0.055	0.046	2.267E-01	0.964	pan	Hospitalized COVID-19
9815_5	RHG01:CRAL-TRIO	Rho GTPase-activating protein 1:Cellular retinaldehyde-TRIO domain	Q07960	ARHGAP1	NA	Wald ratio	1	-0.301	0.250	2.274E-01	0.964	pan	Hospitalized COVID-19
8296_117	KDEL2	KDEL motif-containing protein 2	Q7Z4H8	POGLUT3	NA	Inverse variance weighted	8	0.038	0.031	2.284E-01	0.964	pan	Hospitalized COVID-19
12432_23	CYBP	Calcyclin-binding protein	Q9HB71	CACYBP	NA	Wald ratio	1	-0.175	0.145	2.294E-01	0.964	pan	Hospitalized COVID-19
10781_19	CLC4G	C-type lectin domain family 4 member G	Q6UXB4	CLEC4G	NA	Wald ratio	1	-0.250	0.208	2.295E-01	0.964	pan	Hospitalized COVID-19
15381_45	Discoidin domain receptor 2	Discoidin domain-containing receptor 2	Q16832	DDR2	NA	Wald ratio	1	0.244	0.204	2.297E-01	0.964	pan	Hospitalized COVID-19
17805_35	SPF30	Survival of motor neuron-related-splicing factor 30	O75940	SMNDC1	NA	Wald ratio	1	-0.272	0.227	2.297E-01	0.964	pan	Hospitalized COVID-19
2834_54	kallikrein 8	Kallikrein-8	O60259	KLK8	NA	Inverse variance weighted	3	0.077	0.064	2.298E-01	0.964	pan	Hospitalized COVID-19
3210_1	METAP1	Methionine aminopeptidase 1	P53582	METAP1	NA	Wald ratio	1	0.267	0.222	2.302E-01	0.964	pan	Hospitalized COVID-19
13435_31	IL-20 Rb	Interleukin-20 receptor subunit beta	Q6UXL0	IL20RB	NA	Wald ratio	1	0.242	0.203	2.327E-01	0.964	pan	Hospitalized COVID-19
3326_58	Nectin-like protein 2	Cell adhesion molecule 1	Q9BY67	CADM1	NA	Wald ratio	1	0.088	0.074	2.337E-01	0.964	pan	Hospitalized COVID-19
7776_20	UNC5B	Netrin receptor UNC5B	Q8IZJ1	UNC5B	NA	Inverse variance weighted	3	-0.141	0.119	2.338E-01	0.964	pan	Hospitalized COVID-19
5508_62	Cathepsin D	Cathepsin D	P07339	CTSD	NA	Inverse variance weighted	6	0.066	0.055	2.339E-01	0.964	pan	Hospitalized COVID-19
14204_55	FOXJ2	Forkhead box protein J2	Q9P0K8	FOXJ2	NA	Inverse variance weighted	2	-0.166	0.140	2.341E-01	0.964	pan	Hospitalized COVID-19
14121_24	TRAIL R4	Tumor necrosis factor receptor superfamily member 10D	Q9UBN6	TNFRSF10D	NA	Wald ratio	1	0.303	0.255	2.342E-01	0.964	pan	Hospitalized COVID-19
13700_10	annexin II	Annexin A2	P07355	ANXA2	NA	Inverse variance weighted	8	-0.074	0.062	2.343E-01	0.964	pan	Hospitalized COVID-19
5095_21	KI2L4	Killer cell immunoglobulin-like receptor 2DL4	Q99706	KIR2DL4	NA	Wald ratio	1	-0.194	0.163	2.347E-01	0.964	pan	Hospitalized COVID-19
3518_54	TAFI	Carboxypeptidase B2	Q96IY4	CPB2	NA	Inverse variance weighted	8	0.073	0.062	2.351E-01	0.964	pan	Hospitalized COVID-19
13659_36	AT131	Manganese-transporting ATPase 13A1	Q9HD20	ATP13A1	NA	Inverse variance weighted	2	0.182	0.153	2.352E-01	0.964	pan	Hospitalized COVID-19
3220_40	RET	Proto-oncogene tyrosine-protein kinase receptor Ret	P07949	RET	NA	Inverse variance weighted	5	0.040	0.033	2.357E-01	0.964	pan	Hospitalized COVID-19
12366_16	CRGD	Gamma-crystallin D	P07320	CRYGD	NA	Wald ratio	1	0.113	0.096	2.366E-01	0.964	pan	Hospitalized COVID-19
6965_19	CNTP2	Contactin-associated protein-like 2	Q9UHC6	CNTNAP2	NA	Inverse variance weighted	6	0.039	0.033	2.368E-01	0.964	pan	Hospitalized COVID-19
18878_15	GREM1	Gremlin-1	O60565	GREM1	NA	Inverse variance weighted	10	-0.027	0.023	2.369E-01	0.964	pan	Hospitalized COVID-19
16302_11	SPLC2	BPI fold-containing family A member 2	Q96DR5	BPIFA2	NA	Wald ratio	1	-0.189	0.160	2.373E-01	0.964	pan	Hospitalized COVID-19
14153_8	Ephrin-A3	Ephrin-A3	P52797	EFNA3	NA	Wald ratio	1	0.211	0.179	2.374E-01	0.964	pan	Hospitalized COVID-19
2962_50	PTHrP	Parathyroid hormone-related protein	P12272	PTHLH	NA	Inverse variance weighted	3	0.089	0.075	2.388E-01	0.965	pan	Hospitalized COVID-19
8877_22	F176C:ECD	Protein eva-1 homolog C:Extracellular domain	P58658	EVA1C	NA	Wald ratio	1	-0.192	0.163	2.392E-01	0.965	pan	Hospitalized COVID-19
19169_88	FIBP	Acidic fibroblast growth factor intracellular-binding protein	O43427	FIBP	NA	Wald ratio	1	-0.157	0.134	2.400E-01	0.965	pan	Hospitalized COVID-19
17325_10	KGUA	Guanylate kinase	Q16774	GUK1	NA	Inverse variance weighted	6	-0.051	0.043	2.406E-01	0.965	pan	Hospitalized COVID-19
11208_15	NAGPA	N-acetylglucosamine-1-phosphodiester alpha-N-acetylglucosaminidase	Q9UK23	NAGPA	NA	Inverse variance weighted	7	0.030	0.026	2.413E-01	0.965	pan	Hospitalized COVID-19
3396_54	Renin	Renin	P00797	REN	NA	Inverse variance weighted	7	-0.083	0.071	2.413E-01	0.965	pan	Hospitalized COVID-19
3042_7	Myoglobin	Myoglobin	P02144	MB	NA	Inverse variance weighted	2	0.343	0.293	2.415E-01	0.965	pan	Hospitalized COVID-19
15453_3	a1-Microglobulin	Alpha-1-microglobulin	P02760	AMBP	NA	Wald ratio	1	0.208	0.178	2.417E-01	0.965	pan	Hospitalized COVID-19
13943_38	DPY30	Protein dpy-30 homolog	Q9C005	DPY30	NA	Inverse variance weighted	2	-0.171	0.146	2.418E-01	0.965	pan	Hospitalized COVID-19
6525_17	DUSP13	Dual specificity protein phosphatase 13 isoform A	Q6B8I1	DUSP13	NA	Inverse variance weighted	3	0.044	0.037	2.423E-01	0.965	pan	Hospitalized COVID-19
7185_29	GPV	Platelet glycoprotein V	P40197	GP5	NA	Inverse variance weighted	2	-0.121	0.104	2.424E-01	0.965	pan	Hospitalized COVID-19
7955_195	ITI heavy chain H1	Inter-alpha-trypsin inhibitor heavy chain H1	P19827	ITIH1	NA	Inverse variance weighted	3	0.291	0.250	2.431E-01	0.965	pan	Hospitalized COVID-19
15347_12	Hemopexin	Hemopexin	P02790	HPX	NA	Wald ratio	1	-0.278	0.238	2.439E-01	0.965	pan	Hospitalized COVID-19
13472_35	HDHD2	Haloacid dehalogenase-like hydrolase domain-containing protein 2	Q9H0R4	HDHD2	NA	Inverse variance weighted	3	0.056	0.048	2.445E-01	0.965	pan	Hospitalized COVID-19
17781_191	MLP3A	Microtubule-associated proteins 1A/1B light chain 3A	Q9H492	MAP1LC3A	NA	Inverse variance weighted	8	0.086	0.074	2.449E-01	0.965	pan	Hospitalized COVID-19
15304_1	PAP1	Regenerating islet-derived protein 3-alpha	Q06141	REG3A	NA	Inverse variance weighted	15	0.062	0.053	2.450E-01	0.965	pan	Hospitalized COVID-19
5631_83	MOTI	Promotilin	P12872	MLN	NA	Inverse variance weighted	26	-0.030	0.025	2.453E-01	0.965	pan	Hospitalized COVID-19
4234_8	IL-1 R4	Interleukin-1 receptor-like 1	Q01638	IL1RL1	NA	Inverse variance weighted	24	-0.027	0.024	2.463E-01	0.965	pan	Hospitalized COVID-19
11872_9	PCDGD	Protocadherin gamma-B1	Q9Y5G3	PCDHGB1	NA	Inverse variance weighted	2	0.220	0.190	2.465E-01	0.965	pan	Hospitalized COVID-19
14208_3	RET7	Retinoid-binding protein 7	Q96R05	RBP7	NA	Inverse variance weighted	2	-0.103	0.089	2.467E-01	0.965	pan	Hospitalized COVID-19

5121_3	SEM6B	Semaphorin-6B	Q9H3T3	SEMA6B	NA	Inverse variance weighted	2	0.152	0.131	2.479E-01	0.965	pan	Hospitalized COVID-19
4920_10	Lysozyme	Lysozyme C	P61626	LYZ	NA	Inverse variance weighted	10	-0.037	0.032	2.489E-01	0.965	pan	Hospitalized COVID-19
2939_10	Artemin	Artemin	Q5T4W7	ARTN	NA	Wald ratio	1	0.263	0.228	2.492E-01	0.965	pan	Hospitalized COVID-19
17454_15	EGFL6	Epidermal growth factor-like protein 6	Q8IUX8	EGFL6	NA	Inverse variance weighted	5	0.121	0.105	2.495E-01	0.965	pan	Hospitalized COVID-19
19365_11	BCAT2	Branched-chain-amino-acid aminotransferase, mitochondrial	O15382	BCAT2	NA	Inverse variance weighted	2	0.087	0.076	2.497E-01	0.965	pan	Hospitalized COVID-19
7648_9	MYPC1	Myosin-binding protein C, slow-type	Q00872	MYBPC1	NA	Inverse variance weighted	2	-0.187	0.163	2.503E-01	0.965	pan	Hospitalized COVID-19
18832_65	SAA2	Serum amyloid A-2 protein	P0DJI9	SAA2	NA	Inverse variance weighted	3	-0.320	0.279	2.512E-01	0.965	pan	Hospitalized COVID-19
3049_61	Trypsin	Trypsin-1	P07477	PRSS1	NA	Inverse variance weighted	4	0.212	0.185	2.513E-01	0.965	pan	Hospitalized COVID-19
9265_10	GLIP1	Glioma pathogenesis-related protein 1	P48060	GLIPR1	NA	Wald ratio	1	-0.131	0.114	2.514E-01	0.965	pan	Hospitalized COVID-19
8080_24	PSMP	Prostate-associated microseminoprotein	Q1L6U9	MSMP	NA	Inverse variance weighted	15	-0.051	0.044	2.515E-01	0.965	pan	Hospitalized COVID-19
8484_24	Leptin	Leptin	P41159	LEP	NA	Inverse variance weighted	4	0.120	0.104	2.518E-01	0.965	pan	Hospitalized COVID-19
3431_54	EphA1	Ephrin type-A receptor 1	P21709	EPHA1	NA	Inverse variance weighted	10	-0.021	0.018	2.519E-01	0.965	pan	Hospitalized COVID-19
3773_15	sTie-2	Angiopoietin-1 receptor, soluble	Q02763	TEK	NA	Inverse variance weighted	5	0.053	0.046	2.523E-01	0.965	pan	Hospitalized COVID-19
6081_52	PCOC2	Procollagen C-endopeptidase enhancer 2	Q9UKZ9	PCOLCE2	NA	Inverse variance weighted	15	-0.024	0.021	2.535E-01	0.966	pan	Hospitalized COVID-19
12703_6	NEK7	Serine/threonine-protein kinase Nek7	Q8TDX7	NEK7	NA	Wald ratio	1	-0.097	0.085	2.537E-01	0.966	pan	Hospitalized COVID-19
5480_49	RANTES	C-C motif chemokine 5	P13501	CCL5	NA	Inverse variance weighted	2	0.241	0.211	2.539E-01	0.966	pan	Hospitalized COVID-19
8476_11	CgA	Chromogranin-A	P10645	CHGA	NA	Inverse variance weighted	6	0.085	0.075	2.546E-01	0.966	pan	Hospitalized COVID-19
11302_237	TENR	Tenascin-R	Q92752	TNR	NA	Inverse variance weighted	3	0.100	0.087	2.548E-01	0.966	pan	Hospitalized COVID-19
5349_69	DLL1	Delta-like protein 1	O00548	DLL1	NA	Inverse variance weighted	3	0.091	0.080	2.551E-01	0.966	pan	Hospitalized COVID-19
2637_77	Macrophage mannose receptor	Macrophage mannose receptor 1	P22897	MRC1	NA	Inverse variance weighted	15	0.037	0.033	2.564E-01	0.969	pan	Hospitalized COVID-19
11117_2	SPT20	Spermatogenesis-associated protein 20	Q8TB22	SPATA20	NA	Inverse variance weighted	4	-0.044	0.039	2.585E-01	0.972	pan	Hospitalized COVID-19
14623_26	SUMO3	Small ubiquitin-related modifier 3	P55854	SUMO3	NA	Wald ratio	1	-0.222	0.197	2.585E-01	0.972	pan	Hospitalized COVID-19
8403_18	Fatty acid synthase	Fatty acid synthase	P49327	FASN	NA	Wald ratio	1	0.082	0.073	2.592E-01	0.972	pan	Hospitalized COVID-19
15580_2	EPHA7	Ephrin type-A receptor 7	Q15375	EPHA7	NA	Wald ratio	1	-0.041	0.037	2.597E-01	0.972	pan	Hospitalized COVID-19
11248_43	HEM4	Uroporphyrinogen-III synthase	P10746	UROS	NA	Inverse variance weighted	4	0.056	0.049	2.598E-01	0.972	pan	Hospitalized COVID-19
19193_18	PFD3	Prefoldin subunit 3	P61758	VBP1	NA	Inverse variance weighted	12	-0.098	0.087	2.601E-01	0.972	pan	Hospitalized COVID-19
11109_56	SVEP1:Sushi 15-18	Sushi, von Willebrand factor type A, EGF and pentraxin domain-containing protein 1:Sushi 15-18	Q4LDE5	SVEP1	NA	Inverse variance weighted	8	0.089	0.079	2.602E-01	0.972	pan	Hospitalized COVID-19
19557_3	KLOTB	Beta-klotho	Q86Z14	KLB	NA	Inverse variance weighted	11	-0.027	0.024	2.615E-01	0.973	pan	Hospitalized COVID-19
2992_59	IL-17 sR	Interleukin-17 receptor A	Q96F46	IL17RA	NA	Inverse variance weighted	7	-0.014	0.012	2.618E-01	0.973	pan	Hospitalized COVID-19
3825_18	MK08	Mitogen-activated protein kinase 8	P45983	MAPK8	NA	Inverse variance weighted	3	-0.091	0.081	2.620E-01	0.973	pan	Hospitalized COVID-19
2890_59	CCL28	C-C motif chemokine 28	Q9NRJ3	CCL28	NA	Inverse variance weighted	8	-0.054	0.048	2.625E-01	0.973	pan	Hospitalized COVID-19
16300_4	TREM2	Triggering receptor expressed on myeloid cells 2	Q9NZC2	TREM2	NA	Inverse variance weighted	20	0.033	0.029	2.634E-01	0.975	pan	Hospitalized COVID-19
16915_153	SEM4A	Semaphorin-4A	Q9H3S1	SEMA4A	NA	Inverse variance weighted	8	0.056	0.050	2.651E-01	0.976	pan	Hospitalized COVID-19
2737_22	NovH	Protein NOV homolog	P48745	CCN3	NA	Wald ratio	1	0.264	0.237	2.652E-01	0.976	pan	Hospitalized COVID-19
9297_12	B3GN8	UDP-GlcNAc:betaGal beta-1,3-N-acetylglucosaminyltransferase 8	Q7Z7M8	B3GNT8	NA	Inverse variance weighted	13	-0.046	0.042	2.655E-01	0.976	pan	Hospitalized COVID-19
5487_7	SLAF7	SLAM family member 7	Q9NQ25	SLAMF7	NA	Inverse variance weighted	14	0.035	0.032	2.659E-01	0.976	pan	Hospitalized COVID-19
15312_14	AGO1	Protein argonaute-1	Q9UL18	AGO1	NA	Wald ratio	1	-0.212	0.190	2.664E-01	0.976	pan	Hospitalized COVID-19
2743_5	Sonic Hedgehog	Sonic hedgehog protein	Q15465	SHH	NA	Inverse variance weighted	2	-0.134	0.120	2.667E-01	0.976	pan	Hospitalized COVID-19
16070_7	WIF-1	Wnt inhibitory factor 1	Q9Y5W5	WIF1	NA	Wald ratio	1	-0.191	0.172	2.678E-01	0.976	pan	Hospitalized COVID-19
3495_15	GCP-2	C-X-C motif chemokine 6	P80162	CXCL6	NA	Inverse variance weighted	13	-0.036	0.033	2.686E-01	0.976	pan	Hospitalized COVID-19
3302_58	CYTF	Cystatin-F	O76096	CST7	NA	Inverse variance weighted	13	0.020	0.019	2.695E-01	0.976	pan	Hospitalized COVID-19
12686_15	THTM	3-mercaptopyruvate sulfurtransferase	P25325	MPST	NA	Inverse variance weighted	2	-0.120	0.109	2.696E-01	0.976	pan	Hospitalized COVID-19
4437_56	ENTP5	Ectonucleoside triphosphate diphosphohydrolase 5	O75356	ENTPD5	NA	Inverse variance weighted	9	-0.025	0.022	2.697E-01	0.976	pan	Hospitalized COVID-19
16620_26	LY75	Lymphocyte antigen 75	O60449	LY75	NA	Inverse variance weighted	19	-0.030	0.027	2.699E-01	0.976	pan	Hospitalized COVID-19
4842_62	Glypican 3	Glypican-3	P51654	GPC3	NA	Inverse variance weighted	4	-0.142	0.129	2.701E-01	0.976	pan	Hospitalized COVID-19
7950_142	BTNL9	Butyrophilin-like protein 9	Q6UXG8	BTNL9	NA	Wald ratio	1	0.167	0.151	2.704E-01	0.976	pan	Hospitalized COVID-19
15542_19	KCRU	Creatine kinase U-type, mitochondrial	P12532	CKMT1A	NA	Inverse variance weighted	9	0.068	0.062	2.705E-01	0.976	pan	Hospitalized COVID-19
15503_20	Lefty-A	Left-right determination factor 2	O00292	LEFTY2	NA	Inverse variance weighted	12	0.026	0.023	2.711E-01	0.977	pan	Hospitalized COVID-19
12954_71	PPARa	Peroxisome proliferator-activated receptor alpha	Q07869	PPARA	NA	Wald ratio	1	0.169	0.154	2.721E-01	0.978	pan	Hospitalized COVID-19
12685_57	HOME2	Homer protein homolog 2	Q9NSB8	HOMER2	NA	Inverse variance weighted	2	-0.188	0.171	2.724E-01	0.978	pan	Hospitalized COVID-19
9296_15	PTPRD	Receptor-type tyrosine-protein phosphatase delta	P23468	PTPRD	NA	Wald ratio	1	0.246	0.225	2.726E-01	0.978	pan	Hospitalized COVID-19
9171_11	CSRP3	Cysteine and glycine-rich protein 3	P50461	CSRP3	NA	Inverse variance weighted	2	0.185	0.169	2.739E-01	0.980	pan	Hospitalized COVID-19
2681_23	HGF	Hepatocyte growth factor	P14210	HGF	NA	Wald ratio	1	-0.114	0.104	2.741E-01	0.980	pan	Hospitalized COVID-19
9747_48	MARK3	MAP/microtubule affinity-regulating kinase 3	P27448	MARK3	NA	Wald ratio	1	0.292	0.268	2.752E-01	0.980	pan	Hospitalized COVID-19
5350_14	GPC6	Glypican-6	Q9Y625	GPC6	NA	Inverse variance weighted	2	0.163	0.149	2.756E-01	0.980	pan	Hospitalized COVID-19
5691_2	CRLD2	Cysteine-rich secretory protein LCCL domain-containing 2	Q9H0B8	CRISPLD2	NA	Inverse variance weighted	10	0.042	0.039	2.763E-01	0.980	pan	Hospitalized COVID-19
12348_46	SYSM	Serine--tRNA ligase, mitochondrial	Q9NP81	SARS2	NA	Wald ratio	1	0.125	0.115	2.764E-01	0.980	pan	Hospitalized COVID-19
7038_45	SIA8D	CMP-N-acetylneuraminate-poly-alpha-2,8-sialyltransferase	Q92187	ST8SIA4	NA	Wald ratio	1	-0.268	0.247	2.770E-01	0.980	pan	Hospitalized COVID-19
2911_27	Midkine	Midkine	P21741	MDK	NA	Inverse variance weighted	3	0.165	0.151	2.773E-01	0.980	pan	Hospitalized COVID-19
5861_78	3HAO	3-hydroxyanthranilate 3,4-dioxygenase	P46952	HAAO	NA	Inverse variance weighted	6	-0.153	0.141	2.781E-01	0.980	pan	Hospitalized COVID-19
7015_8	LIRB5	Leukocyte immunoglobulin-like receptor subfamily B member 5	O75023	LILRB5	NA	Inverse variance weighted	7	0.040	0.037	2.791E-01	0.980	pan	Hospitalized COVID-19
9221_6	IPIL1	Inositol 1,4,5-trisphosphate receptor-interacting protein-like 1	Q6GPH6	ITPRIPL1	NA	Inverse variance weighted	5	-0.046	0.042	2.796E-01	0.980	pan	Hospitalized COVID-19
18831_6	LRIG1	Leucine-rich repeats and immunoglobulin-like domains protein 1	Q96JA1	LRIG1	NA	Inverse variance weighted	8	-0.035	0.032	2.796E-01	0.980	pan	Hospitalized COVID-19
5649_83	PSG4	Pregnancy-specific beta-1-glycoprotein 4	Q00888	PSG4	NA	Inverse variance weighted	4	-0.117	0.109	2.797E-01	0.980	pan	Hospitalized COVID-19
19229_92	HOME1	Homer protein homolog 1	Q86YM7	HOMER1	NA	Inverse variance weighted	4	0.109	0.101	2.798E-01	0.980	pan	Hospitalized COVID-19
10938_13	sLFA-3	Lymphocyte function-associated antigen 3	P19256	CD58	NA	Inverse variance weighted	2	-0.071	0.066	2.803E-01	0.980	pan	Hospitalized COVID-19
5128_53	SLAF6	SLAM family member 6	Q96DU3	SLAMF6	NA	Wald ratio	1	-0.062	0.057	2.814E-01	0.980	pan	Hospitalized COVID-19
15466_30	CO9A1	Collagen alpha-1(IX) chain	P20849	COL9A1	NA	Inverse variance weighted	3	0.103	0.096	2.818E-01	0.980	pan	Hospitalized COVID-19
18174_79	PDC6I	Programmed cell death 6-interacting protein	Q8WUM4	PDCD6IP	NA	Inverse variance weighted	2	-0.099	0.092	2.820E-01	0.980	pan	Hospitalized COVID-19
13524_25	H6ST2	Heparan-sulfate 6-O-sulfotransferase 2	Q96MM7	HS6ST2	NA	Wald ratio	1	-0.192	0.179	2.842E-01	0.980	pan	Hospitalized COVID-19
15472_16	LRP11	Low-density lipoprotein receptor-related protein 11	Q86VZ4	LRP11	NA	Inverse variance weighted	8	-0.039	0.036	2.842E-01	0.980	pan	Hospitalized COVID-19
15559_5	ANTR2	Anthrax toxin receptor 2	P58335	ANTXR2	NA	Inverse variance weighted	5	0.071	0.067	2.848E-01	0.980	pan	Hospitalized COVID-19
10080_9	EI2BA	Translation initiation factor eIF-2B subunit alpha	Q14232	EIF2B1	NA	Wald ratio	1	0.193	0.181	2.864E-01	0.980	pan	Hospitalized COVID-19
13124_20	ISLR2	Immunoglobulin superfamily containing leucine-rich repeat protein 2	Q6UXK2	ISLR2	NA	Inverse variance weighted	4	-0.055	0.052	2.864E-01	0.980	pan	Hospitalized COVID-19
14107_1	MTHFS	5-formyltetrahydrofolate cyclo-ligase	P49914	MTHFS	NA	Inverse variance weighted	6	0.033	0.031	2.866E-01	0.980	pan	Hospitalized COVID-19
16818_200	CDCP1	CUB domain-containing protein 1	Q9H5V8	CDCP1	NA	Inverse variance weighted	5	-0.068	0.063	2.869E-01	0.980	pan	Hospitalized COVID-19
12347_29	CCM2	Cerebral cavernous malformations 2 protein	Q9BSQ5	CCM2	NA	Wald ratio	1	-0.177	0.166	2.875E-01	0.980	pan	Hospitalized COVID-19
19488_1	PDCD6	Programmed cell death protein 6	O75340	PDCD6	NA	Inverse variance weighted	3	0.074	0.069	2.875E-01	0.980	pan	Hospitalized COVID-19

15603_20	Integrin alpha-2	Integrin alpha-2	P17301	ITGA2	NA	Inverse variance weighted	2	0.135	0.127	2.877E-01	0.980	pan	Hospitalized COVID-19
17161_1	OST48	Dolichyl-diphosphooligosaccharide--protein glycosyltransferase 48 kDa subunit	P39656	DDOST	NA	Inverse variance weighted	2	-0.127	0.119	2.879E-01	0.980	pan	Hospitalized COVID-19
9076_25	PENK	Proenkephalin-A	P01210	PENK	NA	Inverse variance weighted	13	0.020	0.019	2.886E-01	0.980	pan	Hospitalized COVID-19
7059_14	LIRA6	Leukocyte immunoglobulin-like receptor subfamily A member 6	Q6PI73	LILRA6	NA	Inverse variance weighted	7	0.047	0.044	2.893E-01	0.980	pan	Hospitalized COVID-19
3519_3	TARC	C-C motif chemokine 17	Q92583	CCL17	NA	Inverse variance weighted	18	-0.028	0.026	2.905E-01	0.980	pan	Hospitalized COVID-19
11910_27	DLX4	Homeobox protein DLX-4	Q92988	DLX4	NA	Inverse variance weighted	2	-0.100	0.095	2.906E-01	0.980	pan	Hospitalized COVID-19
17210_2	TCL1A	T-cell leukemia/lymphoma protein 1A	P56279	TCL1A	NA	Inverse variance weighted	5	-0.062	0.059	2.912E-01	0.980	pan	Hospitalized COVID-19
12386_11	AMPB	Aminopeptidase B	Q9H4A4	RNPEP	NA	Inverse variance weighted	5	0.041	0.039	2.922E-01	0.980	pan	Hospitalized COVID-19
15522_2	GAPR1	Golgi-associated plant pathogenesis-related protein 1	Q9H4G4	GLIPR2	NA	Inverse variance weighted	6	-0.056	0.053	2.923E-01	0.980	pan	Hospitalized COVID-19
16307_22	UNC5H4	Netrin receptor UNC5D	Q6UXZ4	UNC5D	NA	Inverse variance weighted	3	0.078	0.074	2.931E-01	0.980	pan	Hospitalized COVID-19
12530_14	CKS-1	Cyclin-dependent kinases regulatory subunit 1	P61024	CKS1B	NA	Inverse variance weighted	3	-0.095	0.090	2.932E-01	0.980	pan	Hospitalized COVID-19
16851_50	SCO2	Protein SCO2 homolog, mitochondrial	O43819	SCO2	NA	Wald ratio	1	-0.113	0.107	2.933E-01	0.980	pan	Hospitalized COVID-19
3894_15	NAGK	N-acetyl-D-glucosamine kinase	Q9UJ70	NAGK	NA	Inverse variance weighted	5	-0.058	0.056	2.934E-01	0.980	pan	Hospitalized COVID-19
11681_8	NUPL	Arf-GAP domain and FG repeat-containing protein 1	P52594	AGFG1	NA	Wald ratio	1	-0.139	0.133	2.940E-01	0.980	pan	Hospitalized COVID-19
8786_6	F171B:ECD	Protein FAM171B:Extracellular domain	Q6P995	FAM171B	NA	Wald ratio	1	0.069	0.066	2.941E-01	0.980	pan	Hospitalized COVID-19
9213_24	FTCD	Formimidoyltransferase-cyclodeaminase	O95954	FTCD	NA	Inverse variance weighted	3	-0.082	0.079	2.947E-01	0.980	pan	Hospitalized COVID-19
18873_8	CEAM8	Carcinoembryonic antigen-related cell adhesion molecule 8	P31997	CEACAM8	NA	Inverse variance weighted	12	0.055	0.053	2.952E-01	0.980	pan	Hospitalized COVID-19
18408_26	ARF4	ADP-ribosylation factor 4	P18085	ARF4	NA	Wald ratio	1	-0.245	0.235	2.957E-01	0.980	pan	Hospitalized COVID-19
12717_65	TOX3	TOX high mobility group box family member 3	O15405	TOX3	NA	Wald ratio	1	-0.219	0.210	2.957E-01	0.980	pan	Hospitalized COVID-19
14254_27	PTN4	Tyrosine-protein phosphatase non-receptor type 4	P29074	PTPN4	NA	Inverse variance weighted	4	-0.119	0.114	2.958E-01	0.980	pan	Hospitalized COVID-19
8932_1	ENTP6	Ectonucleoside triphosphate diphosphohydrolase 6	O75354	ENTPD6	NA	Wald ratio	1	0.214	0.205	2.967E-01	0.980	pan	Hospitalized COVID-19
7861_9	ROR2	Tyrosine-protein kinase transmembrane receptor ROR2	Q01974	ROR2	NA	Inverse variance weighted	3	-0.063	0.061	2.968E-01	0.980	pan	Hospitalized COVID-19
4559_64	KYNU	Kynureninase	Q16719	KYNU	NA	Inverse variance weighted	6	-0.036	0.034	2.970E-01	0.980	pan	Hospitalized COVID-19
12637_7	CACP	Carnitine O-acetyltransferase	P43155	CRAT	NA	Wald ratio	1	0.119	0.114	2.970E-01	0.980	pan	Hospitalized COVID-19
13642_90	CIG49	Interferon-induced protein with tetratricopeptide repeats 3	O14879	IFIT3	NA	Inverse variance weighted	2	0.113	0.109	2.990E-01	0.980	pan	Hospitalized COVID-19
12646_2	RPE	Ribulose-phosphate 3-epimerase	Q96AT9	RPE	NA	Wald ratio	1	0.132	0.127	2.994E-01	0.980	pan	Hospitalized COVID-19
16616_137	ENOB	Beta-enolase	P13929	ENO3	NA	Inverse variance weighted	5	0.125	0.121	2.995E-01	0.980	pan	Hospitalized COVID-19
13405_61	ISK2	Serine protease inhibitor Kazal-type 2	P20155	SPINK2	NA	Inverse variance weighted	6	-0.027	0.026	3.003E-01	0.980	pan	Hospitalized COVID-19
9849_13	APEX1	DNA-(apurinic or apyrimidinic site) lyase	P27695	APEX1	NA	Wald ratio	1	-0.241	0.233	3.006E-01	0.980	pan	Hospitalized COVID-19
4160_49	MMP-2	72 kDa type IV collagenase	P08253	MMP2	NA	Inverse variance weighted	2	0.138	0.133	3.008E-01	0.980	pan	Hospitalized COVID-19
15375_49	Carboxypeptidase B1	Carboxypeptidase B	P15086	CPB1	NA	Inverse variance weighted	9	0.044	0.042	3.009E-01	0.980	pan	Hospitalized COVID-19
18242_8	Sperm protein 17	Sperm surface protein Sp17	Q15506	SPA17	NA	Wald ratio	1	-0.250	0.242	3.011E-01	0.980	pan	Hospitalized COVID-19
7628_40	CREL1	Cysteine-rich with EGF-like domain protein 1	Q96HD1	CRELD1	NA	Inverse variance weighted	13	0.024	0.023	3.011E-01	0.980	pan	Hospitalized COVID-19
6227_1	kallikrein 10	Kallikrein-10	O43240	KLK10	NA	Inverse variance weighted	8	0.045	0.043	3.015E-01	0.980	pan	Hospitalized COVID-19
8357_43	ST4A1	Sulfotransferase 4A1	Q9BR01	SULT4A1	NA	Wald ratio	1	0.192	0.187	3.029E-01	0.980	pan	Hospitalized COVID-19
12411_60	MAX	Protein max	P61244	MAX	NA	Wald ratio	1	0.059	0.057	3.031E-01	0.980	pan	Hospitalized COVID-19
4908_6	Endoglin	Endoglin	P17813	ENG	NA	Inverse variance weighted	7	-0.035	0.034	3.031E-01	0.980	pan	Hospitalized COVID-19
7808_5	GLCE	D-glucuronyl C5-epimerase	O94923	GLCE	NA	Inverse variance weighted	9	-0.025	0.024	3.039E-01	0.980	pan	Hospitalized COVID-19
3172_28	ARSB	Arylsulfatase B	P15848	ARSB	NA	Inverse variance weighted	2	0.096	0.094	3.040E-01	0.980	pan	Hospitalized COVID-19
3029_52	DC-SIGN	CD209 antigen	Q9NNX6	CD209	NA	Inverse variance weighted	9	0.045	0.043	3.040E-01	0.980	pan	Hospitalized COVID-19
13378_80	YD286	Glutaredoxin-like protein C5orf63	A6NC05	C5orf63	NA	Wald ratio	1	-0.204	0.198	3.043E-01	0.980	pan	Hospitalized COVID-19
5012_67	Myokinase, human	Adenylate kinase isoenzyme 1	P00568	AK1	NA	Wald ratio	1	-0.248	0.242	3.046E-01	0.980	pan	Hospitalized COVID-19
2585_2	PRL	Prolactin	P01236	PRL	NA	Wald ratio	1	0.157	0.153	3.047E-01	0.980	pan	Hospitalized COVID-19
9077_10	MA1A2	Mannosyl-oligosaccharide 1,2-alpha-mannosidase IB	O60476	MAN1A2	NA	Inverse variance weighted	5	-0.050	0.049	3.059E-01	0.982	pan	Hospitalized COVID-19
15417_3	Maspin	Serpin B5	P36952	SERPINB5	NA	Wald ratio	1	-0.083	0.081	3.063E-01	0.982	pan	Hospitalized COVID-19
9282_12	CRIS2	Cysteine-rich secretory protein 2	P16562	CRISP2	NA	Inverse variance weighted	33	-0.015	0.015	3.066E-01	0.982	pan	Hospitalized COVID-19
19296_51	MLRA	Myosin regulatory light chain 2, atrial isoform	Q01449	MYL7	NA	Inverse variance weighted	6	-0.077	0.076	3.074E-01	0.983	pan	Hospitalized COVID-19
7251_64	C1QT3	Complement C1q tumor necrosis factor-related protein 3	Q9BXJ4	C1QTNF3	NA	Inverse variance weighted	3	-0.070	0.069	3.081E-01	0.983	pan	Hospitalized COVID-19
8900_28	NEO1	Neogenin	Q92859	NEO1	NA	Inverse variance weighted	4	-0.048	0.047	3.088E-01	0.983	pan	Hospitalized COVID-19
9769_48	DNER:ECD	Delta and Notch-like epidermal growth factor-related receptor:Extracellular domain	Q8NFT8	DNER	NA	Inverse variance weighted	5	0.060	0.059	3.091E-01	0.983	pan	Hospitalized COVID-19
13125_45	Vitronectin	Vitronectin	P04004	VTN	NA	Inverse variance weighted	12	-0.060	0.060	3.103E-01	0.983	pan	Hospitalized COVID-19
8908_14	KCE1L:CD	Potassium voltage-gated channel subfamily E regulatory beta subunit 5:Cytoplasmic domain	Q9UJ90	KCNE5	NA	Inverse variance weighted	3	-0.136	0.134	3.112E-01	0.983	pan	Hospitalized COVID-19
17783_9	MMAB	Cob(I)yrinic acid a,c-diamide adenosyltransferase, mitochondrial	Q96EY8	MMAB	NA	Inverse variance weighted	6	0.031	0.031	3.113E-01	0.983	pan	Hospitalized COVID-19
18875_125	Chondrocalcin	Chondrocalcin	P02458	COL2A1	NA	Inverse variance weighted	18	-0.020	0.020	3.120E-01	0.983	pan	Hospitalized COVID-19
3805_16	Endocan	Endothelial cell-specific molecule 1	Q9NQ30	ESM1	NA	Inverse variance weighted	5	-0.068	0.067	3.126E-01	0.983	pan	Hospitalized COVID-19
6391_52	LIRA3	Leukocyte immunoglobulin-like receptor subfamily A member 3	Q8N6C8	LILRA3	NA	Inverse variance weighted	8	0.047	0.047	3.130E-01	0.983	pan	Hospitalized COVID-19
3324_51	LY9	T-lymphocyte surface antigen Ly-9	Q9HBG7	LY9	NA	Inverse variance weighted	6	0.057	0.057	3.130E-01	0.983	pan	Hospitalized COVID-19
7206_20	F16P1	Fructose-1,6-bisphosphatase 1	P09467	FBP1	NA	Inverse variance weighted	2	0.163	0.162	3.134E-01	0.983	pan	Hospitalized COVID-19
17776_15	PECR	Peroxisomal trans-2-enoyl-CoA reductase	Q9BY49	PECR	NA	Wald ratio	1	0.108	0.108	3.146E-01	0.983	pan	Hospitalized COVID-19
7753_21	HTAI2	Oxidoreductase HTATIP2	Q9BUP3	HTATIP2	NA	Wald ratio	1	0.228	0.227	3.149E-01	0.983	pan	Hospitalized COVID-19
6597_24	TM157	Membrane protein FAM174A	Q8TBP5	FAM174A	NA	Inverse variance weighted	8	-0.029	0.029	3.163E-01	0.983	pan	Hospitalized COVID-19
5867_60	ARGI1	Arginase-1	P05089	ARG1	NA	Wald ratio	1	0.132	0.132	3.164E-01	0.983	pan	Hospitalized COVID-19
13423_94	CJ058	Redox-regulatory protein FAM213A	Q9BRX8	PRXL2A	NA	Wald ratio	1	0.211	0.210	3.164E-01	0.983	pan	Hospitalized COVID-19
18342_2	SERC	Phosphoserine aminotransferase	Q9Y617	PSAT1	NA	Inverse variance weighted	2	-0.116	0.116	3.166E-01	0.983	pan	Hospitalized COVID-19
18185_118	ALDOB	Fructose-bisphosphate aldolase B	P05062	ALDOB	NA	Inverse variance weighted	3	0.062	0.062	3.167E-01	0.983	pan	Hospitalized COVID-19
18899_82	HDGR3	Hepatoma-derived growth factor-related protein 3	Q9Y3E1	HDGFL3	NA	Inverse variance weighted	3	-0.139	0.139	3.170E-01	0.983	pan	Hospitalized COVID-19
4128_27	Eotaxin-2	C-C motif chemokine 24	O00175	CCL24	NA	Wald ratio	1	-0.224	0.224	3.170E-01	0.983	pan	Hospitalized COVID-19
15367_38	LPLC1	BPI fold-containing family B member 1	Q8TDL5	BPIFB1	NA	Inverse variance weighted	15	-0.030	0.030	3.170E-01	0.983	pan	Hospitalized COVID-19
15636_49	SORC1	VPS10 domain-containing receptor SorCS1	Q8WY21	SORCS1	NA	Wald ratio	1	-0.159	0.159	3.173E-01	0.983	pan	Hospitalized COVID-19
8334_65	TCP11	T-complex protein 11 homolog	Q8WWU5	TCP11	NA	Wald ratio	1	0.161	0.162	3.191E-01	0.983	pan	Hospitalized COVID-19
5015_15	PAFAH	Platelet-activating factor acetylhydrolase	Q13093	PLA2G7	NA	Inverse variance weighted	10	-0.051	0.051	3.192E-01	0.983	pan	Hospitalized COVID-19
5236_2	NR1D1	Nuclear receptor subfamily 1 group D member 1	P20393	NR1D1	NA	Wald ratio	1	-0.238	0.240	3.196E-01	0.983	pan	Hospitalized COVID-19
8289_8	GPNMB:ECD	Transmembrane glycoprotein NMB:Extracellular domain	Q14956	GPNMB	NA	Inverse variance weighted	8	-0.051	0.051	3.196E-01	0.983	pan	Hospitalized COVID-19
7806_33	B4GT7	Beta-1,4-galactosyltransferase 7	Q9UBV7	B4GALT7	NA	Inverse variance weighted	8	-0.037	0.037	3.199E-01	0.983	pan	Hospitalized COVID-19
12460_18	PSA7	Proteasome subunit alpha type-7	O14818	PSMA7	NA	Inverse variance weighted	3	-0.062	0.062	3.204E-01	0.983	pan	Hospitalized COVID-19
12378_71	TPSN	Tapasin	O15533	TAPBP	NA	Inverse variance weighted	7	0.033	0.034	3.214E-01	0.983	pan	Hospitalized COVID-19
4992_49	GRN	Granulins	P28799	GRN	NA	Inverse variance weighted	9	-0.072	0.072	3.215E-01	0.983	pan	Hospitalized COVID-19
15596_7	HEXI1	Protein HEXIM1	O94992	HEXIM1	NA	Wald ratio	1	0.194	0.196	3.220E-01	0.983	pan	Hospitalized COVID-19

17357_33	SPCS	O-phosphoseryl-tRNA(Sec) selenium transferase	Q9HD40	SEPSECS	NA	Wald ratio	1	-0.145	0.147	3.228E-01	0.983	pan	Hospitalized COVID-19
8252_2	NOTUM	Palmitoleoyl-protein carboxylesterase NOTUM	Q6P988	NOTUM	NA	Inverse variance weighted	4	0.084	0.085	3.231E-01	0.983	pan	Hospitalized COVID-19
13118_5	SMOC1	SPARC-related modular calcium-binding protein 1	Q9H4F8	SMOC1	NA	Inverse variance weighted	3	0.050	0.050	3.242E-01	0.983	pan	Hospitalized COVID-19
15476_6	REG3G	Regenerating islet-derived protein 3-gamma	Q6UW15	REG3G	NA	Inverse variance weighted	17	0.024	0.024	3.244E-01	0.983	pan	Hospitalized COVID-19
19175_18	MARCKSL1	MARCKS-related protein	P49006	MARCKSL1	NA	Inverse variance weighted	7	0.096	0.098	3.261E-01	0.983	pan	Hospitalized COVID-19
2754_50	C3	Complement C3	P01024	C3	NA	Wald ratio	1	-0.228	0.232	3.261E-01	0.983	pan	Hospitalized COVID-19
8248_222	SIG14	Sialic acid-binding Ig-like lectin 14	Q08ET2	SIGLEC14	NA	Inverse variance weighted	4	0.093	0.095	3.272E-01	0.983	pan	Hospitalized COVID-19
8773_172	EMIL3:region 1	EMILIN-3:region 1	Q9NT22	EMILIN3	NA	Inverse variance weighted	7	-0.053	0.054	3.276E-01	0.983	pan	Hospitalized COVID-19
6461_54	Apo C-III	Apolipoprotein C-III	P02656	APOC3	NA	Inverse variance weighted	6	-0.114	0.117	3.278E-01	0.983	pan	Hospitalized COVID-19
13998_26	PURA1	Adenylosuccinate synthetase isozyme 1	Q8N142	ADSS1	NA	Inverse variance weighted	3	0.064	0.066	3.279E-01	0.983	pan	Hospitalized COVID-19
7193_98	PCDC1	Protocadherin alpha-C1	Q9H158	PCDHAC1	NA	Wald ratio	1	-0.149	0.153	3.286E-01	0.983	pan	Hospitalized COVID-19
4145_58	Neurotrophin-3	Neurotrophin-3	P20783	NTF3	NA	Inverse variance weighted	5	0.074	0.076	3.286E-01	0.983	pan	Hospitalized COVID-19
16753_46	CO6A2	Collagen alpha-2(VI) chain	P12110	COL6A2	NA	Inverse variance weighted	5	-0.073	0.074	3.287E-01	0.983	pan	Hospitalized COVID-19
12689_56	ARC1B	Actin-related protein 2/3 complex subunit 1B	O15143	ARPC1B	NA	Wald ratio	1	0.115	0.118	3.292E-01	0.983	pan	Hospitalized COVID-19
7139_14	SLIK4	SLIT and NTRK-like protein 4	Q8IW52	SLITRK4	NA	Wald ratio	1	0.185	0.190	3.300E-01	0.983	pan	Hospitalized COVID-19
19254_125	GMPR1	GMP reductase 1	P36959	GMPR	NA	Inverse variance weighted	15	-0.021	0.022	3.312E-01	0.983	pan	Hospitalized COVID-19
3000_66	MBL	Mannose-binding protein C	P11226	MBL2	NA	Inverse variance weighted	20	-0.017	0.017	3.314E-01	0.983	pan	Hospitalized COVID-19
6986_17	HS3SB	Heparan sulfate glucosamine 3-O-sulfotransferase 3B1	Q9Y662	HS3ST3B1	NA	Wald ratio	1	-0.248	0.256	3.325E-01	0.983	pan	Hospitalized COVID-19
5132_71	TCCR	Interleukin-27 receptor subunit alpha	Q6UWB1	IL27RA	NA	Inverse variance weighted	9	-0.022	0.022	3.330E-01	0.983	pan	Hospitalized COVID-19
8606_39	GPNMB:CD	Transmembrane glycoprotein NMB:Cytoplasmic domain	Q14956	GPNMB	NA	Inverse variance weighted	9	0.031	0.032	3.340E-01	0.983	pan	Hospitalized COVID-19
3344_60	Antithrombin III	Antithrombin-III	P01008	SERPINC1	NA	Wald ratio	1	0.167	0.173	3.343E-01	0.983	pan	Hospitalized COVID-19
2620_4	gp130, soluble	Interleukin-6 receptor subunit beta	P40189	IL6ST	NA	Inverse variance weighted	10	0.030	0.031	3.343E-01	0.983	pan	Hospitalized COVID-19
7173_141	T132C:ECD	Transmembrane protein 132C:Extracellular domain	Q8N3T6	TMEM132C	NA	Inverse variance weighted	10	0.030	0.031	3.351E-01	0.983	pan	Hospitalized COVID-19
3171_57	amyloid precursor protein	Amyloid beta A4 protein	P05067	APP	NA	Inverse variance weighted	2	0.117	0.121	3.355E-01	0.983	pan	Hospitalized COVID-19
17761_2	NUDT5	ADP-sugar pyrophosphatase	Q9UKK9	NUDT5	NA	Inverse variance weighted	2	-0.122	0.127	3.356E-01	0.983	pan	Hospitalized COVID-19
19170_25	DPOE3	DNA polymerase epsilon subunit 3	Q9NRF9	POLE3	NA	Wald ratio	1	0.112	0.116	3.362E-01	0.983	pan	Hospitalized COVID-19
17383_4	STAR5	StAR-related lipid transfer protein 5	Q9NSY2	STARD5	NA	Inverse variance weighted	6	0.059	0.061	3.364E-01	0.983	pan	Hospitalized COVID-19
12333_87	RPIA	Ribose-5-phosphate isomerase	P49247	RPIA	NA	Inverse variance weighted	2	-0.098	0.102	3.368E-01	0.983	pan	Hospitalized COVID-19
12501_10	TBCA	Tubulin-specific chaperone A	O75347	TBCA	NA	Inverse variance weighted	4	0.144	0.150	3.369E-01	0.983	pan	Hospitalized COVID-19
15614_168	LIRA2	Leukocyte immunoglobulin-like receptor subfamily A member 2	Q8N149	LILRA2	NA	Inverse variance weighted	5	-0.032	0.033	3.372E-01	0.983	pan	Hospitalized COVID-19
19277_4	KAT	Thiosulfate sulfurtransferase/rhodanese-like domain-containing protein 1	Q8NFU3	TSTD1	NA	Inverse variance weighted	3	0.061	0.063	3.373E-01	0.983	pan	Hospitalized COVID-19
2474_54	SAP	Serum amyloid P-component	P02743	APCS	NA	Inverse variance weighted	7	0.044	0.045	3.373E-01	0.983	pan	Hospitalized COVID-19
8376_25	LSHB	Lutropin subunit beta	P01229	LHB	NA	Inverse variance weighted	4	0.049	0.051	3.378E-01	0.983	pan	Hospitalized COVID-19
6518_85	ghrelin	Appetite-regulating hormone	Q9UBU3	GHRL	NA	Inverse variance weighted	3	-0.079	0.082	3.386E-01	0.983	pan	Hospitalized COVID-19
13993_20	E41L1	Band 4.1-like protein 1	Q9H4G0	EPB41L1	NA	Inverse variance weighted	2	0.142	0.148	3.390E-01	0.983	pan	Hospitalized COVID-19
2837_3	Met	Hepatocyte growth factor receptor	P08581	MET	NA	Inverse variance weighted	7	0.052	0.055	3.392E-01	0.983	pan	Hospitalized COVID-19
8325_37	ADH4	Alcohol dehydrogenase 4	P08319	ADH4	NA	Wald ratio	1	0.131	0.138	3.397E-01	0.983	pan	Hospitalized COVID-19
7761_125	CHKB	Choline/ethanolamine kinase	Q9Y259	CHKB	NA	Inverse variance weighted	3	0.084	0.088	3.413E-01	0.983	pan	Hospitalized COVID-19
4886_3	MCP-3	C-C motif chemokine 7	P80098	CCL7	NA	Inverse variance weighted	5	-0.071	0.075	3.415E-01	0.983	pan	Hospitalized COVID-19
7019_13	Semaphorin-7A	Semaphorin-7A	O75326	SEMA7A	NA	Inverse variance weighted	2	0.226	0.237	3.418E-01	0.983	pan	Hospitalized COVID-19
15468_14	FHR1	Complement factor H-related protein 1	Q03591	CFHR1	NA	Inverse variance weighted	10	0.057	0.060	3.422E-01	0.983	pan	Hospitalized COVID-19
3855_56	Peroxiredoxin-1	Peroxiredoxin-1	Q06830	PRDX1	NA	Inverse variance weighted	7	-0.048	0.050	3.424E-01	0.983	pan	Hospitalized COVID-19
13969_24	IMA7	Importin subunit alpha-7	O60684	KPNA6	NA	Wald ratio	1	0.166	0.175	3.434E-01	0.983	pan	Hospitalized COVID-19
15387_44	Neuropilin-2	Neuropilin-2	O60462	NRP2	NA	Inverse variance weighted	6	0.060	0.064	3.435E-01	0.983	pan	Hospitalized COVID-19
13973_62	TTL	Tubulin--tyrosine ligase	Q8NG68	TTL	NA	Wald ratio	1	0.173	0.183	3.441E-01	0.983	pan	Hospitalized COVID-19
5131_15	TAJ	Tumor necrosis factor receptor superfamily member 19	Q9NS68	TNFRSF19	NA	Wald ratio	1	-0.101	0.106	3.446E-01	0.983	pan	Hospitalized COVID-19
7898_29	MDM1	Nuclear protein MDM1	Q8TC05	MDM1	NA	Inverse variance weighted	2	-0.111	0.118	3.447E-01	0.983	pan	Hospitalized COVID-19
13947_371	NUD12	Peroxisomal NADH pyrophosphatase NUDT12	Q9BQG2	NUDT12	NA	Inverse variance weighted	5	0.027	0.029	3.450E-01	0.983	pan	Hospitalized COVID-19
18173_11	ARK73	Aflatoxin B1 aldehyde reductase member 3	O95154	AKR7A3	NA	Inverse variance weighted	6	0.034	0.036	3.453E-01	0.983	pan	Hospitalized COVID-19
14271_23	RAB6B	Ras-related protein Rab-6B	Q9NRW1	RAB6B	NA	Wald ratio	1	0.065	0.069	3.455E-01	0.983	pan	Hospitalized COVID-19
19194_9	Histidyl-tRNA synthetase-related	D-tyrosyl-tRNA(Tyr) deacylase 1	Q8TEA8	DTD1	NA	Wald ratio	1	0.049	0.052	3.455E-01	0.983	pan	Hospitalized COVID-19
3727_35	PYY	Peptide YY	P10082	PYY	NA	Inverse variance weighted	4	-0.096	0.102	3.457E-01	0.983	pan	Hospitalized COVID-19
13518_5	ASAP2	Arf-GAP with SH3 domain, ANK repeat and PH domain-containing protein 2	O43150	ASAP2	NA	Wald ratio	1	0.124	0.131	3.464E-01	0.983	pan	Hospitalized COVID-19
6485_59	IGLL1	Immunoglobulin lambda-like polypeptide 1	P15814	IGLL1	NA	Inverse variance weighted	27	-0.022	0.023	3.465E-01	0.983	pan	Hospitalized COVID-19
9416_77	CBPM	Carboxypeptidase M	P14384	CPM	NA	Inverse variance weighted	5	0.079	0.084	3.467E-01	0.983	pan	Hospitalized COVID-19
13986_6	LANC1	LanC-like protein 1	O43813	LANCL1	NA	Inverse variance weighted	2	-0.105	0.112	3.468E-01	0.983	pan	Hospitalized COVID-19
17786_5	GGPPS	Geranylgeranyl pyrophosphate synthase	O95749	GGPS1	NA	Wald ratio	1	-0.107	0.114	3.479E-01	0.983	pan	Hospitalized COVID-19
14260_112	ARHG8	Neuroepithelial cell-transforming gene 1 protein	Q7Z628	NET1	NA	Inverse variance weighted	3	0.117	0.124	3.481E-01	0.983	pan	Hospitalized COVID-19
10892_8	OSMR	Oncostatin-M-specific receptor subunit beta	Q99650	OSMR	NA	Inverse variance weighted	2	0.127	0.135	3.483E-01	0.983	pan	Hospitalized COVID-19
9896_21	FOXGB	Forkhead box protein G1	P55316	FOXG1	NA	Inverse variance weighted	2	0.158	0.169	3.497E-01	0.983	pan	Hospitalized COVID-19
13122_19	FLRT2	Leucine-rich repeat transmembrane protein FLRT2	O43155	FLRT2	NA	Inverse variance weighted	7	0.030	0.032	3.498E-01	0.983	pan	Hospitalized COVID-19
2966_65	SCGF-beta	Stem cell growth factor-beta	Q9Y240	CLEC11A	NA	Inverse variance weighted	2	0.170	0.182	3.504E-01	0.983	pan	Hospitalized COVID-19
3376_49	IL-17 RD	Interleukin-17 receptor D	Q8NFM7	IL17RD	NA	Inverse variance weighted	6	0.035	0.037	3.505E-01	0.983	pan	Hospitalized COVID-19
6586_19	ADA11	Disintegrin and metalloproteinase domain-containing protein 11	O75078	ADAM11	NA	Wald ratio	1	0.106	0.114	3.507E-01	0.983	pan	Hospitalized COVID-19
5301_7	Eotaxin	Eotaxin	P51671	CCL11	NA	Inverse variance weighted	9	0.057	0.061	3.515E-01	0.983	pan	Hospitalized COVID-19
13453_2	RM33	39S ribosomal protein L33, mitochondrial	O75394	MRPL33	NA	Inverse variance weighted	4	-0.091	0.098	3.523E-01	0.983	pan	Hospitalized COVID-19
6507_16	NCAM2	Neural cell adhesion molecule 2	O15394	NCAM2	NA	Inverse variance weighted	7	0.026	0.028	3.531E-01	0.983	pan	Hospitalized COVID-19
15539_15	SLIK1	SLIT and NTRK-like protein 1	Q96PX8	SLITRK1	NA	Wald ratio	1	-0.142	0.153	3.536E-01	0.983	pan	Hospitalized COVID-19
13477_65	LT4R1	Leukotriene B4 receptor 1	Q15722	LTB4R	NA	Inverse variance weighted	2	0.105	0.113	3.537E-01	0.983	pan	Hospitalized COVID-19
5947_90	Tpo	Thrombopoietin	P40225	THPO	NA	Inverse variance weighted	2	0.210	0.227	3.550E-01	0.983	pan	Hospitalized COVID-19
18321_38	MTDC	Bifunctional methylenetetrahydrofolate dehydrogenase/cyclohydrolase, mitochondrial	P13995	MTHFD2	NA	Inverse variance weighted	4	0.068	0.074	3.552E-01	0.983	pan	Hospitalized COVID-19
12682_5	KAT3	Kynurenine--oxoglutarate transaminase 3	Q6YP21	KYAT3	NA	Inverse variance weighted	7	-0.031	0.034	3.557E-01	0.983	pan	Hospitalized COVID-19
5223_59	GCKR	Glucokinase regulatory protein	Q14397	GCKR	NA	Wald ratio	1	0.151	0.163	3.562E-01	0.983	pan	Hospitalized COVID-19
12475_48	CLIC5	Chloride intracellular channel protein 5	Q9NZA1	CLIC5	NA	Inverse variance weighted	9	0.031	0.034	3.562E-01	0.983	pan	Hospitalized COVID-19
3285_23	C1r	Complement C1r subcomponent	P00736	C1R	NA	Inverse variance weighted	2	-0.212	0.231	3.570E-01	0.983	pan	Hospitalized COVID-19
13093_6	SECTM1	Secreted and transmembrane protein 1	Q8WVN6	SECTM1	NA	Inverse variance weighted	2	-0.078	0.085	3.576E-01	0.983	pan	Hospitalized COVID-19
12033_3	BUB1	Mitotic checkpoint serine/threonine-protein kinase BUB1	O43683	BUB1	NA	Wald ratio	1	0.143	0.156	3.590E-01	0.983	pan	Hospitalized COVID-19
19370_30	HS3S4	Heparan sulfate glucosamine 3-O-sulfotransferase 4	Q9Y661	HS3ST4	NA	Wald ratio	1	0.145	0.158	3.591E-01	0.983	pan	Hospitalized COVID-19

9175_48	DSCAM	Down syndrome cell adhesion molecule	O60469	DSCAM	NA	Inverse variance weighted	2	-0.066	0.072	3.592E-01	0.983	pan	Hospitalized COVID-19
17702_53	UGT 1A1	UDP-glucuronosyltransferase 1-1	P22309	UGT1A1	NA	Inverse variance weighted	3	-0.091	0.099	3.595E-01	0.983	pan	Hospitalized COVID-19
17799_9	6PGL	6-phosphogluconolactonase	O95336	PGLS	NA	Inverse variance weighted	4	-0.082	0.090	3.602E-01	0.983	pan	Hospitalized COVID-19
9241_40	SIRPG	Signal-regulatory protein gamma	Q9P1W8	SIRPG	NA	Wald ratio	1	-0.122	0.133	3.603E-01	0.983	pan	Hospitalized COVID-19
11378_37	KSYK:SH2, 1 and 2	Tyrosine-protein kinase SYK:Src Homology domain	P43405	SYK	NA	Wald ratio	1	0.202	0.221	3.604E-01	0.983	pan	Hospitalized COVID-19
15521_4	Alcadein alpha-1	Calsyntenin-1	O94985	CLSTN1	NA	Inverse variance weighted	7	0.014	0.015	3.604E-01	0.983	pan	Hospitalized COVID-19
17153_46	KI2L3	Killer cell immunoglobulin-like receptor 2DL3	P43628	KIR2DL3	NA	Inverse variance weighted	10	0.053	0.058	3.604E-01	0.983	pan	Hospitalized COVID-19
9459_7	Fas, soluble	Tumor necrosis factor receptor superfamily member 6	P25445	FAS	NA	Inverse variance weighted	2	-0.160	0.175	3.604E-01	0.983	pan	Hospitalized COVID-19
11134_30	RF1ML	Peptide chain release factor 1-like, mitochondrial	Q9UGC7	MTRF1L	NA	Wald ratio	1	0.059	0.064	3.607E-01	0.983	pan	Hospitalized COVID-19
6077_63	CECR1	Adenosine deaminase CECR1	Q9NZK5	ADA2	NA	Inverse variance weighted	4	-0.058	0.064	3.614E-01	0.983	pan	Hospitalized COVID-19
4474_19	Ubiquitin	Ubiquitin	P62979	RPS27A	NA	Wald ratio	1	-0.251	0.276	3.620E-01	0.984	pan	Hospitalized COVID-19
8945_7	F175B	BRISC complex subunit Abro1	Q15018	ABRAXAS2	NA	Wald ratio	1	0.124	0.137	3.632E-01	0.984	pan	Hospitalized COVID-19
7157_22	PCDA4	Protocadherin alpha-4	Q9UN74	PCDHA4	NA	Inverse variance weighted	12	0.058	0.064	3.632E-01	0.984	pan	Hospitalized COVID-19
11239_49	TMPS6	Transmembrane protease serine 6	Q8IU80	TMPRSS6	NA	Wald ratio	1	-0.180	0.199	3.648E-01	0.984	pan	Hospitalized COVID-19
3041_55	MRC2	C-type mannose receptor 2	Q9UBG0	MRC2	NA	Inverse variance weighted	6	-0.052	0.057	3.651E-01	0.984	pan	Hospitalized COVID-19
8383_20	CK094	Uncharacterized protein C11orf94	C9JXX5	C11orf94	NA	Wald ratio	1	0.086	0.096	3.670E-01	0.984	pan	Hospitalized COVID-19
14067_6	PKP2	Plakophilin-2	Q99959	PKP2	NA	Inverse variance weighted	2	0.182	0.202	3.672E-01	0.984	pan	Hospitalized COVID-19
4276_10	prostatic binding protein	Phosphatidylethanolamine-binding protein 1	P30086	PEBP1	NA	Inverse variance weighted	10	-0.026	0.029	3.677E-01	0.984	pan	Hospitalized COVID-19
9578_263	MANS4	MANSC domain-containing protein 4	A6NHS7	MANSC4	NA	Inverse variance weighted	9	-0.017	0.019	3.679E-01	0.984	pan	Hospitalized COVID-19
13552_7	SWP70	Switch-associated protein 70	Q9UH65	SWAP70	NA	Inverse variance weighted	8	-0.020	0.022	3.681E-01	0.984	pan	Hospitalized COVID-19
10959_125	BET1L	BET1-like protein	Q9NYM9	BET1L	NA	Wald ratio	1	0.147	0.163	3.681E-01	0.984	pan	Hospitalized COVID-19
5598_3	GREM2	Gremlin-2	Q9H772	GREM2	NA	Wald ratio	1	-0.155	0.172	3.684E-01	0.984	pan	Hospitalized COVID-19
7928_183	TPST1	Protein-tyrosine sulfotransferase 1	O60507	TPST1	NA	Inverse variance weighted	11	0.026	0.029	3.701E-01	0.984	pan	Hospitalized COVID-19
8368_102	TNF sR-II	Tumor necrosis factor receptor superfamily member 1B	P20333	TNFRSF1B	NA	Wald ratio	1	-0.057	0.063	3.702E-01	0.984	pan	Hospitalized COVID-19
9266_1	sTREM-1	Triggering receptor expressed on myeloid cells 1	Q9NP99	TREM1	NA	Inverse variance weighted	7	0.073	0.082	3.705E-01	0.984	pan	Hospitalized COVID-19
2879_9	a1-Antichymotrypsin	Alpha-1-antichymotrypsin	P01011	SERPINA3	NA	Wald ratio	1	-0.205	0.228	3.707E-01	0.984	pan	Hospitalized COVID-19
5227_60	PDK1	[Pyruvate dehydrogenase (acetyl-transferring)] kinase isozyme 1, mitochondrial	Q15118	PDK1	NA	Inverse variance weighted	3	0.057	0.063	3.711E-01	0.984	pan	Hospitalized COVID-19
15308_108	VWC2	Brorin	Q2TAL6	VWC2	NA	Inverse variance weighted	9	0.027	0.030	3.713E-01	0.984	pan	Hospitalized COVID-19
9595_11	B4GT2	Beta-1,4-galactosyltransferase 2	O60909	B4GALT2	NA	Inverse variance weighted	4	-0.047	0.053	3.717E-01	0.984	pan	Hospitalized COVID-19
16318_12	ALK-1	Serine/threonine-protein kinase receptor R3	P37023	ACVRL1	NA	Inverse variance weighted	3	0.046	0.051	3.727E-01	0.984	pan	Hospitalized COVID-19
9267_2	CBPA4	Carboxypeptidase A4	Q9UI42	CPA4	NA	Inverse variance weighted	22	-0.011	0.012	3.734E-01	0.984	pan	Hospitalized COVID-19
4192_10	AK1A1	Alcohol dehydrogenase [NADP(+)]	P14550	AKR1A1	NA	Inverse variance weighted	10	0.022	0.025	3.735E-01	0.984	pan	Hospitalized COVID-19
8069_85	CD3E	T-cell surface glycoprotein CD3 epsilon chain	P07766	CD3E	NA	Inverse variance weighted	3	-0.092	0.103	3.737E-01	0.984	pan	Hospitalized COVID-19
6471_53	FHR4	Complement factor H-related protein 4	Q92496	CFHR4	NA	Inverse variance weighted	19	0.026	0.030	3.742E-01	0.984	pan	Hospitalized COVID-19
19376_74	NNMT	Nicotinamide N-methyltransferase	P40261	NNMT	NA	Wald ratio	1	0.152	0.171	3.745E-01	0.984	pan	Hospitalized COVID-19
8244_16	FUT8	Alpha-(1,6)-fucosyltransferase	Q9BYC5	FUT8	NA	Inverse variance weighted	19	-0.028	0.032	3.757E-01	0.984	pan	Hospitalized COVID-19
3059_50	BAFF	Tumor necrosis factor ligand superfamily member 13B	Q9Y275	TNFSF13B	NA	Wald ratio	1	0.164	0.186	3.757E-01	0.984	pan	Hospitalized COVID-19
17722_5	FKBP52 protein	Peptidyl-prolyl cis-trans isomerase FKBP4	Q02790	FKBP4	NA	Inverse variance weighted	2	0.130	0.147	3.768E-01	0.984	pan	Hospitalized COVID-19
19379_154	RAB3D	Ras-related protein Rab-3D	O95716	RAB3D	NA	Wald ratio	1	0.160	0.181	3.771E-01	0.984	pan	Hospitalized COVID-19
13563_259	ACBD7	Acyl-CoA-binding domain-containing protein 7	Q8N6N7	ACBD7	NA	Wald ratio	1	0.232	0.265	3.798E-01	0.984	pan	Hospitalized COVID-19
6472_40	GP100	Melanocyte protein PMEL	P40967	PMEL	NA	Inverse variance weighted	13	0.040	0.045	3.810E-01	0.984	pan	Hospitalized COVID-19
11649_3	SMAP1	Stromal membrane-associated protein 1	Q8IYB5	SMAP1	NA	Inverse variance weighted	6	-0.038	0.043	3.811E-01	0.984	pan	Hospitalized COVID-19
6927_7	NDST1	Bifunctional heparan sulfate N-deacetylase/N-sulfotransferase 1	P52848	NDST1	NA	Inverse variance weighted	3	0.058	0.066	3.813E-01	0.984	pan	Hospitalized COVID-19
5092_51	JAG1:ECD	Protein jagged-1:Extracellular domain	P78504	JAG1	NA	Wald ratio	1	-0.143	0.164	3.814E-01	0.984	pan	Hospitalized COVID-19
19563_3	SEZ6L	Seizure 6-like protein	Q9BYH1	SEZ6L	NA	Inverse variance weighted	3	0.077	0.088	3.819E-01	0.984	pan	Hospitalized COVID-19
12593_33	PDRG1	p53 and DNA damage-regulated protein 1	Q9NUG6	PDRG1	NA	Wald ratio	1	-0.164	0.188	3.825E-01	0.984	pan	Hospitalized COVID-19
8664_36	PKDCC	Extracellular tyrosine-protein kinase PKDCC	Q504Y2	PKDCC	NA	Inverse variance weighted	2	-0.240	0.275	3.830E-01	0.984	pan	Hospitalized COVID-19
11142_11	ANGL1:C-term	Angiopoietin-related protein 1:C-Term, Fibrinogen domian	O95841	ANGPTL1	NA	Inverse variance weighted	7	0.027	0.031	3.833E-01	0.984	pan	Hospitalized COVID-19
9837_60	NAD(P)H dehydrogenase	NAD(P)H dehydrogenase [quinone] 1	P15559	NQO1	NA	Inverse variance weighted	12	-0.020	0.022	3.835E-01	0.984	pan	Hospitalized COVID-19
4314_12	XTP3A	dCTP pyrophosphatase 1	Q9H773	DCTPP1	NA	Wald ratio	1	0.116	0.133	3.839E-01	0.984	pan	Hospitalized COVID-19
5060_62	B7-H1	Programmed cell death 1 ligand 1	Q9NZQ7	CD274	NA	Inverse variance weighted	2	0.038	0.044	3.845E-01	0.984	pan	Hospitalized COVID-19
10391_1	ANGL3	Angiopoietin-related protein 3	Q9Y5C1	ANGPTL3	NA	Inverse variance weighted	9	-0.031	0.036	3.846E-01	0.984	pan	Hospitalized COVID-19
18396_10	AES	Amino-terminal enhancer of split	Q08117	TLE5	NA	Wald ratio	1	-0.185	0.213	3.855E-01	0.984	pan	Hospitalized COVID-19
10041_3	HNF4A	Hepatocyte nuclear factor 4-alpha	P41235	HNF4A	NA	Wald ratio	1	-0.183	0.211	3.861E-01	0.984	pan	Hospitalized COVID-19
11449_22	CAZA1	F-actin-capping protein subunit alpha-1	P52907	CAPZA1	NA	Wald ratio	1	-0.247	0.285	3.863E-01	0.984	pan	Hospitalized COVID-19
6572_10	LRRT4:ECD	Leucine-rich repeat transmembrane neuronal protein 4:Isoform 2, Extracellular domain	Q86VH4	LRRTM4	NA	Wald ratio	1	-0.188	0.217	3.865E-01	0.984	pan	Hospitalized COVID-19
17752_24	Arginase	Arginase-2, mitochondrial	P78540	ARG2	NA	Inverse variance weighted	2	0.095	0.109	3.866E-01	0.984	pan	Hospitalized COVID-19
6022_57	NELL2	Protein kinase C-binding protein NELL2	Q99435	NELL2	NA	Wald ratio	1	-0.115	0.133	3.871E-01	0.984	pan	Hospitalized COVID-19
4153_11	alpha-1-antichymotrypsin complex	Alpha-1-antichymotrypsin complex	P07288 | P01011	KLK3 | SERPINA3	NA	Inverse variance weighted	3	-0.087	0.101	3.871E-01	0.984	pan	Hospitalized COVID-19
5957_30	Somatostatin-28	Somatostatin-28	P61278	SST	NA	Wald ratio	1	0.175	0.202	3.878E-01	0.984	pan	Hospitalized COVID-19
12635_9	TRDMT	tRNA (cytosine(38)-C(5))-methyltransferase	O14717	TRDMT1	NA	Inverse variance weighted	4	-0.045	0.052	3.891E-01	0.984	pan	Hospitalized COVID-19
3054_3	Haptoglobin, Mixed Type	Haptoglobin	P00738	HP	NA	Inverse variance weighted	10	0.055	0.064	3.895E-01	0.984	pan	Hospitalized COVID-19
12709_63	H1X	Histone H1x	Q92522	H1-10	NA	Wald ratio	1	-0.205	0.238	3.897E-01	0.984	pan	Hospitalized COVID-19
8345_27	GPX7	Glutathione peroxidase 7	Q96SL4	GPX7	NA	Inverse variance weighted	8	0.023	0.027	3.911E-01	0.984	pan	Hospitalized COVID-19
17683_2	COMD9	COMM domain-containing protein 9	Q9P000	COMMD9	NA	Wald ratio	1	-0.088	0.103	3.913E-01	0.984	pan	Hospitalized COVID-19
16823_75	APOL3	Apolipoprotein L3	O95236	APOL3	NA	Inverse variance weighted	8	0.024	0.028	3.914E-01	0.984	pan	Hospitalized COVID-19
5596_75	CD5:ECD	T-cell surface glycoprotein CD5:Extracellular domain	P06127	CD5	NA	Inverse variance weighted	17	-0.044	0.051	3.915E-01	0.984	pan	Hospitalized COVID-19
13463_1	PXDN	Peroxidasin homolog	Q92626	PXDN	NA	Inverse variance weighted	4	0.059	0.069	3.916E-01	0.984	pan	Hospitalized COVID-19
15329_167	MAPIP	Ragulator complex protein LAMTOR2	Q9Y2Q5	LAMTOR2	NA	Wald ratio	1	0.196	0.229	3.920E-01	0.984	pan	Hospitalized COVID-19
11709_29	CPT1B	Carnitine O-palmitoyltransferase 1, muscle isoform	Q92523	CPT1B	NA	Wald ratio	1	0.147	0.173	3.943E-01	0.984	pan	Hospitalized COVID-19
9172_69	MMP-8	Neutrophil collagenase	P22894	MMP8	NA	Inverse variance weighted	8	-0.053	0.062	3.958E-01	0.984	pan	Hospitalized COVID-19
3622_33	LGMN	Legumain	Q99538	LGMN	NA	Inverse variance weighted	4	0.047	0.055	3.962E-01	0.984	pan	Hospitalized COVID-19
13132_14	SEM5A	Semaphorin-5A	Q13591	SEMA5A	NA	Inverse variance weighted	9	-0.010	0.012	3.970E-01	0.984	pan	Hospitalized COVID-19
6457_50	PCDG1	Protocadherin gamma-A1	Q9Y5H4	PCDHGA1	NA	Inverse variance weighted	12	0.042	0.049	3.973E-01	0.984	pan	Hospitalized COVID-19
7948_129	GLTD2	Glycolipid transfer protein domain-containing protein 2	A6NH11	GLTPD2	NA	Inverse variance weighted	3	0.041	0.048	3.983E-01	0.984	pan	Hospitalized COVID-19
13397_88	HBD-2	Beta-defensin 4A	O15263	DEFB4A	NA	Inverse variance weighted	4	0.055	0.065	3.984E-01	0.984	pan	Hospitalized COVID-19
11926_23	P34	Alpha- and gamma-adaptin-binding protein p34	Q6PD74	AAGAB	NA	Inverse variance weighted	3	0.188	0.222	3.984E-01	0.984	pan	Hospitalized COVID-19
14747_9	CRLF1	Cytokine receptor-like factor 1	O75462	CRLF1	NA	Inverse variance weighted	2	-0.047	0.055	3.985E-01	0.984	pan	Hospitalized COVID-19

15556_49	Alpha-amylase 2B	Alpha-amylase 2B	P19961	AMY2B	NA	Inverse variance weighted	4	0.081	0.096	3.991E-01	0.984	pan	Hospitalized COVID-19
17170_15	CALCB	Calcitonin gene-related peptide 2	P10092	CALCB	NA	Inverse variance weighted	2	-0.039	0.047	4.014E-01	0.984	pan	Hospitalized COVID-19
7194_36	NPTN	Neuroplastin	Q9Y639	NPTN	NA	Inverse variance weighted	2	0.123	0.147	4.026E-01	0.984	pan	Hospitalized COVID-19
11643_73	DTX3L	E3 ubiquitin-protein ligase DTX3L	Q8TDB6	DTX3L	NA	Wald ratio	1	-0.160	0.191	4.028E-01	0.984	pan	Hospitalized COVID-19
8000_17	KI2LA	Killer cell immunoglobulin-like receptor 2DL5A	Q8N109	KIR2DL5A	NA	Inverse variance weighted	8	0.027	0.033	4.039E-01	0.984	pan	Hospitalized COVID-19
13068_139	CCS	Copper chaperone for superoxide dismutase	O14618	CCS	NA	Wald ratio	1	0.090	0.108	4.047E-01	0.984	pan	Hospitalized COVID-19
5459_33	CYTN	Cystatin-SN	P01037	CST1	NA	Inverse variance weighted	6	0.068	0.082	4.049E-01	0.984	pan	Hospitalized COVID-19
6467_65	KTEL1	Protein O-glucosyltransferase 1	Q8NBL1	POGLUT1	NA	Inverse variance weighted	2	-0.038	0.046	4.054E-01	0.984	pan	Hospitalized COVID-19
6984_6	IGSF8	Immunoglobulin superfamily member 8	Q969P0	IGSF8	NA	Inverse variance weighted	2	0.056	0.068	4.054E-01	0.984	pan	Hospitalized COVID-19
14634_13	LSHR	Lutropin-choriogonadotropic hormone receptor	P22888	LHCGR	NA	Inverse variance weighted	5	-0.124	0.150	4.068E-01	0.984	pan	Hospitalized COVID-19
3052_8	Fas ligand, soluble	Tumor necrosis factor ligand superfamily member 6, soluble form	P48023	FASLG	NA	Inverse variance weighted	4	-0.095	0.114	4.074E-01	0.984	pan	Hospitalized COVID-19
7916_10	S100A7	Protein S100-A7	P31151	S100A7	NA	Inverse variance weighted	16	0.016	0.019	4.075E-01	0.984	pan	Hospitalized COVID-19
2982_82	Galectin-4	Galectin-4	P56470	LGALS4	NA	Wald ratio	1	-0.152	0.183	4.076E-01	0.984	pan	Hospitalized COVID-19
13578_98	ABLM3	Actin-binding LIM protein 3	O94929	ABLIM3	NA	Wald ratio	1	-0.082	0.098	4.077E-01	0.984	pan	Hospitalized COVID-19
7810_20	C1QTNF5	Complement C1q tumor necrosis factor-related protein 5	Q9BXJ0	C1QTNF5	NA	Inverse variance weighted	10	0.042	0.050	4.077E-01	0.984	pan	Hospitalized COVID-19
8923_94	GLTL1	Polypeptide N-acetylgalactosaminyltransferase 16	Q8N428	GALNT16	NA	Inverse variance weighted	4	-0.054	0.065	4.079E-01	0.984	pan	Hospitalized COVID-19
18916_25	Inosine triphosphatase	Inosine triphosphate pyrophosphatase	Q9BY32	ITPA	NA	Inverse variance weighted	11	0.017	0.020	4.079E-01	0.984	pan	Hospitalized COVID-19
17814_8	BPNT1	3'(2'),5'-bisphosphate nucleotidase 1	O95861	BPNT1	NA	Wald ratio	1	0.152	0.184	4.081E-01	0.984	pan	Hospitalized COVID-19
8839_4	Metaxin-2	Metaxin-2	O75431	MTX2	NA	Wald ratio	1	-0.177	0.213	4.084E-01	0.984	pan	Hospitalized COVID-19
2475_1	SCF sR	Mast/stem cell growth factor receptor Kit	P10721	KIT	NA	Inverse variance weighted	9	0.049	0.059	4.086E-01	0.984	pan	Hospitalized COVID-19
10445_20	ApoM	Apolipoprotein M	O95445	APOM	NA	Inverse variance weighted	2	-0.101	0.123	4.089E-01	0.984	pan	Hospitalized COVID-19
6527_1	TRIL:ECD	TLR4 interactor with leucine rich repeats:Extracellular domain	Q7L0X0	TRIL	NA	Wald ratio	1	0.109	0.133	4.100E-01	0.984	pan	Hospitalized COVID-19
11441_11	PYGL	Glycogen phosphorylase, liver form	P06737	PYGL	NA	Inverse variance weighted	7	-0.052	0.064	4.106E-01	0.984	pan	Hospitalized COVID-19
7891_45	UGT 1A6	UDP-glucuronosyltransferase 1-6	P19224	UGT1A6	NA	Inverse variance weighted	4	-0.067	0.082	4.109E-01	0.984	pan	Hospitalized COVID-19
13119_26	protein Z inhibitor	Protein Z-dependent protease inhibitor	Q9UK55	SERPINA10	NA	Inverse variance weighted	16	0.027	0.033	4.115E-01	0.984	pan	Hospitalized COVID-19
17677_47	BY55	CD160 antigen	O95971	CD160	NA	Wald ratio	1	-0.080	0.097	4.117E-01	0.984	pan	Hospitalized COVID-19
3079_62	TIG2	Retinoic acid receptor responder protein 2	Q99969	RARRES2	NA	Inverse variance weighted	2	0.052	0.064	4.126E-01	0.984	pan	Hospitalized COVID-19
17813_21	BPHL	Valacyclovir hydrolase	Q86WA6	BPHL	NA	Inverse variance weighted	2	0.098	0.120	4.128E-01	0.984	pan	Hospitalized COVID-19
16809_1	NDKM	Nucleoside diphosphate kinase, mitochondrial	O00746	NME4	NA	Wald ratio	1	0.116	0.142	4.134E-01	0.984	pan	Hospitalized COVID-19
6380_23	ICT1	Peptidyl-tRNA hydrolase ICT1, mitochondrial	Q14197	MRPL58	NA	Wald ratio	1	-0.117	0.143	4.140E-01	0.984	pan	Hospitalized COVID-19
6612_90	CP079	BRICHOS domain-containing protein 5	Q6PL45	BRICD5	NA	Inverse variance weighted	2	-0.082	0.100	4.144E-01	0.984	pan	Hospitalized COVID-19
15613_16	LIPP	Pancreatic triacylglycerol lipase	P16233	PNLIP	NA	Inverse variance weighted	5	0.079	0.097	4.150E-01	0.984	pan	Hospitalized COVID-19
6555_58	Stomatin-like protein 2	Stomatin-like protein 2, mitochondrial	Q9UJZ1	STOML2	NA	Wald ratio	1	0.163	0.200	4.152E-01	0.984	pan	Hospitalized COVID-19
7905_30	HPT	Haptoglobin isoform 2	P00738	HP	NA	Wald ratio	1	-0.133	0.163	4.156E-01	0.984	pan	Hospitalized COVID-19
7551_33	LRC32	Leucine-rich repeat-containing protein 32	Q14392	LRRC32	NA	Wald ratio	1	0.142	0.175	4.159E-01	0.984	pan	Hospitalized COVID-19
2836_68	Lipocalin 2	Neutrophil gelatinase-associated lipocalin	P80188	LCN2	NA	Inverse variance weighted	7	-0.057	0.070	4.160E-01	0.984	pan	Hospitalized COVID-19
10534_40	PARP:BRCT domain	Poly [ADP-ribose] polymerase 1:BRCA1 C-terminal:BRCA1 C-terminus domian	P09874	PARP1	NA	Wald ratio	1	-0.179	0.220	4.160E-01	0.984	pan	Hospitalized COVID-19
3389_7	PCI	Plasma serine protease inhibitor	P05154	SERPINA5	NA	Inverse variance weighted	2	-0.114	0.141	4.167E-01	0.984	pan	Hospitalized COVID-19
9574_11	BIN1	Myc box-dependent-interacting protein 1	O00499	BIN1	NA	Inverse variance weighted	2	0.058	0.072	4.167E-01	0.984	pan	Hospitalized COVID-19
3151_6	IL-2 sRa	Interleukin-2 receptor subunit alpha	P01589	IL2RA	NA	Wald ratio	1	0.093	0.114	4.168E-01	0.984	pan	Hospitalized COVID-19
18308_30	STXB6	Syntaxin-binding protein 6	Q8NFX7	STXBP6	NA	Wald ratio	1	-0.171	0.211	4.176E-01	0.984	pan	Hospitalized COVID-19
3858_5	PPAC	Low molecular weight phosphotyrosine protein phosphatase	P24666	ACP1	NA	Inverse variance weighted	9	-0.016	0.020	4.192E-01	0.984	pan	Hospitalized COVID-19
4912_17	GOT1	Aspartate aminotransferase, cytoplasmic	P17174	GOT1	NA	Wald ratio	1	0.169	0.209	4.195E-01	0.984	pan	Hospitalized COVID-19
12554_10	RA51D	DNA repair protein RAD51 homolog 4	O75771	RAD51D	NA	Inverse variance weighted	3	-0.130	0.161	4.205E-01	0.984	pan	Hospitalized COVID-19
8268_98	HS3SA	Heparan sulfate glucosamine 3-O-sulfotransferase 3A1	Q9Y663	HS3ST3A1	NA	Inverse variance weighted	2	-0.104	0.129	4.207E-01	0.984	pan	Hospitalized COVID-19
6990_44	SDF2L	Stromal cell-derived factor 2-like protein 1	Q9HCN8	SDF2L1	NA	Wald ratio	1	0.147	0.183	4.207E-01	0.984	pan	Hospitalized COVID-19
2658_27	TrkC	NT-3 growth factor receptor	Q16288	NTRK3	NA	Inverse variance weighted	6	-0.041	0.051	4.225E-01	0.984	pan	Hospitalized COVID-19
13614_6	CREB-binding protein	CREB-binding protein	Q92793	CREBBP	NA	Inverse variance weighted	3	0.163	0.203	4.226E-01	0.984	pan	Hospitalized COVID-19
4878_3	Coagulation Factor X	Coagulation Factor X	P00742	F10	NA	Wald ratio	1	0.187	0.233	4.226E-01	0.984	pan	Hospitalized COVID-19
7822_11	HRSL2	HRAS-like suppressor 2	Q9NWW9	PLAAT2	NA	Wald ratio	1	-0.178	0.222	4.228E-01	0.984	pan	Hospitalized COVID-19
6221_1	ADPGK	ADP-dependent glucokinase	Q9BRR6	ADPGK	NA	Wald ratio	1	0.229	0.285	4.234E-01	0.984	pan	Hospitalized COVID-19
17766_5	NCF-1	Neutrophil cytosol factor 1	P14598	NCF1	NA	Inverse variance weighted	8	0.031	0.039	4.234E-01	0.984	pan	Hospitalized COVID-19
4129_72	Factor B	Complement factor B	P00751	CFB	NA	Inverse variance weighted	3	-0.075	0.094	4.241E-01	0.984	pan	Hospitalized COVID-19
13634_209	PIR	Pirin	O00625	PIR	NA	Wald ratio	1	-0.160	0.201	4.249E-01	0.984	pan	Hospitalized COVID-19
4148_49	PAPP-A	Pappalysin-1	Q13219	PAPPA	NA	Inverse variance weighted	4	0.071	0.089	4.256E-01	0.984	pan	Hospitalized COVID-19
6448_36	Sema E	Semaphorin-3C	Q99985	SEMA3C	NA	Inverse variance weighted	2	-0.067	0.085	4.259E-01	0.984	pan	Hospitalized COVID-19
16049_43	OLR1	Oxidized low-density lipoprotein receptor 1	P78380	OLR1	NA	Wald ratio	1	0.105	0.132	4.262E-01	0.984	pan	Hospitalized COVID-19
12851_5	DPOLM	DNA-directed DNA/RNA polymerase mu	Q9NP87	POLM	NA	Inverse variance weighted	3	0.054	0.068	4.264E-01	0.984	pan	Hospitalized COVID-19
15326_64	GBP1	Guanylate-binding protein 1	P32455	GBP1	NA	Inverse variance weighted	9	-0.033	0.042	4.266E-01	0.984	pan	Hospitalized COVID-19
4154_57	P-Selectin	P-selectin	P16109	SELP	NA	Inverse variance weighted	4	-0.047	0.060	4.268E-01	0.984	pan	Hospitalized COVID-19
3831_21	pTEN	Phosphatidylinositol 3,4,5-trisphosphate 3-phosphatase and dual-specificity protein phosphatase PTEN	P60484	PTEN	NA	Wald ratio	1	0.199	0.250	4.269E-01	0.984	pan	Hospitalized COVID-19
6574_11	FAIM3	Fas apoptotic inhibitory molecule 3	O60667	FCMR	NA	Inverse variance weighted	3	-0.097	0.122	4.275E-01	0.984	pan	Hospitalized COVID-19
5462_62	Ficolin-3	Ficolin-3	O75636	FCN3	NA	Inverse variance weighted	12	-0.019	0.024	4.278E-01	0.984	pan	Hospitalized COVID-19
6086_15	CRDL2	Chordin-like protein 2	Q6WN34	CHRDL2	NA	Inverse variance weighted	6	0.058	0.073	4.280E-01	0.984	pan	Hospitalized COVID-19
16856_79	MARE2	Microtubule-associated protein RP/EB family member 2	Q15555	MAPRE2	NA	Inverse variance weighted	2	0.104	0.132	4.289E-01	0.984	pan	Hospitalized COVID-19
13516_46	TMM85	ER membrane protein complex subunit 4	Q5J8M3	EMC4	NA	Inverse variance weighted	7	-0.015	0.019	4.290E-01	0.984	pan	Hospitalized COVID-19
13740_51	sFRP-3	Secreted frizzled-related protein 3	Q92765	FRZB	NA	Inverse variance weighted	10	-0.019	0.024	4.301E-01	0.984	pan	Hospitalized COVID-19
12676_1	PACN1	Protein kinase C and casein kinase substrate in neurons protein 1	Q9BY11	PACSIN1	NA	Inverse variance weighted	2	0.060	0.076	4.314E-01	0.984	pan	Hospitalized COVID-19
19259_176	IF2A	Eukaryotic translation initiation factor 2 subunit 1	P05198	EIF2S1	NA	Wald ratio	1	0.141	0.179	4.315E-01	0.984	pan	Hospitalized COVID-19
3903_49	Sorting nexin 4	Sorting nexin-4	O95219	SNX4	NA	Inverse variance weighted	2	-0.075	0.095	4.321E-01	0.984	pan	Hospitalized COVID-19
19338_3	LZIC	Protein LZIC	Q8WZA0	LZIC	NA	Wald ratio	1	-0.210	0.267	4.327E-01	0.984	pan	Hospitalized COVID-19
16035_8	VEGF sR3	Vascular endothelial growth factor receptor 3	P35916	FLT4	NA	Inverse variance weighted	11	-0.040	0.051	4.330E-01	0.984	pan	Hospitalized COVID-19
2687_2	MIA	Melanoma-derived growth regulatory protein	Q16674	MIA	NA	Inverse variance weighted	7	-0.024	0.031	4.333E-01	0.984	pan	Hospitalized COVID-19
12338_27	EGFLA	Pikachurin	Q63HQ2	EGFLAM	NA	Inverse variance weighted	3	0.037	0.048	4.337E-01	0.984	pan	Hospitalized COVID-19
17755_5	UGDH	UDP-glucose 6-dehydrogenase	O60701	UGDH	NA	Inverse variance weighted	3	-0.037	0.047	4.345E-01	0.984	pan	Hospitalized COVID-19
5736_1	TRML2	Trem-like transcript 2 protein	Q5T2D2	TREML2	NA	Inverse variance weighted	5	-0.026	0.033	4.348E-01	0.984	pan	Hospitalized COVID-19
5456_59	CNDP1	Beta-Ala-His dipeptidase	Q96KN2	CNDP1	NA	Inverse variance weighted	12	-0.021	0.027	4.361E-01	0.984	pan	Hospitalized COVID-19
8983_7	GOLM1	Golgi membrane protein 1	Q8NBJ4	GOLM1	NA	Inverse variance weighted	5	0.090	0.116	4.367E-01	0.984	pan	Hospitalized COVID-19

10085_25	STAR	Steroidogenic acute regulatory protein, mitochondrial	P49675	STAR	NA	Wald ratio	1	-0.184	0.237	4.368E-01	0.984	pan	Hospitalized COVID-19
8660_5	OLFL3	Olfactomedin-like protein 3	Q9NRN5	OLFML3	NA	Inverse variance weighted	5	0.061	0.078	4.368E-01	0.984	pan	Hospitalized COVID-19
6408_2	INHBC	Inhibin beta C chain	P55103	INHBC	NA	Inverse variance weighted	4	0.042	0.054	4.369E-01	0.984	pan	Hospitalized COVID-19
6550_4	ICAM4	Intercellular adhesion molecule 4	Q14773	ICAM4	NA	Inverse variance weighted	13	-0.024	0.031	4.370E-01	0.984	pan	Hospitalized COVID-19
11387_3	ATF6B	Cyclic AMP-dependent transcription factor ATF-6 beta	Q99941	ATF6B	NA	Inverse variance weighted	4	-0.070	0.091	4.370E-01	0.984	pan	Hospitalized COVID-19
9409_11	TRYB1	Tryptase beta-1	Q15661	TPSAB1	NA	Inverse variance weighted	18	0.016	0.021	4.370E-01	0.984	pan	Hospitalized COVID-19
19261_12	SDCB2	Syntenin-2	Q9H190	SDCBP2	NA	Wald ratio	1	0.122	0.157	4.370E-01	0.984	pan	Hospitalized COVID-19
15336_7	SELM	Selenoprotein M	Q8WWX9	SELENOM	NA	Wald ratio	1	0.153	0.198	4.387E-01	0.984	pan	Hospitalized COVID-19
16621_77	AIBP	NAD(P)H-hydrate epimerase	Q8NCW5	NAXE	NA	Wald ratio	1	0.043	0.055	4.388E-01	0.984	pan	Hospitalized COVID-19
7192_37	CRF2-12	Interferon lambda receptor 1	Q8IU57	IFNLR1	NA	Inverse variance weighted	2	-0.080	0.104	4.399E-01	0.984	pan	Hospitalized COVID-19
17691_1	TPP1	Tripeptidyl-peptidase 1	O14773	TPP1	NA	Inverse variance weighted	6	-0.044	0.057	4.400E-01	0.984	pan	Hospitalized COVID-19
2973_15	CD36 ANTIGEN	Platelet glycoprotein 4	P16671	CD36	NA	Inverse variance weighted	8	0.034	0.044	4.403E-01	0.984	pan	Hospitalized COVID-19
5688_65	CBLN4	Cerebellin-4	Q9NTU7	CBLN4	NA	Inverse variance weighted	6	-0.033	0.043	4.414E-01	0.984	pan	Hospitalized COVID-19
15435_4	PNP	Purine nucleoside phosphorylase	P00491	PNP	NA	Inverse variance weighted	2	-0.051	0.066	4.417E-01	0.984	pan	Hospitalized COVID-19
13526_5	LA:RRM	Lupus La protein:RNA recognition motif	P05455	SSB	NA	Wald ratio	1	-0.116	0.151	4.419E-01	0.984	pan	Hospitalized COVID-19
8297_8	DJC10	DnaJ homolog subfamily C member 10	Q8IXB1	DNAJC10	NA	Inverse variance weighted	6	-0.052	0.067	4.419E-01	0.984	pan	Hospitalized COVID-19
9594_30	ZNT5	Zinc transporter 5	Q8TAD4	SLC30A5	NA	Wald ratio	1	-0.088	0.114	4.422E-01	0.984	pan	Hospitalized COVID-19
16309_30	Soggy-1	Dickkopf-like protein 1	Q9UK85	DKKL1	NA	Wald ratio	1	0.224	0.291	4.422E-01	0.984	pan	Hospitalized COVID-19
5813_58	Epo	Erythropoietin	P01588	EPO	NA	Inverse variance weighted	5	0.090	0.117	4.424E-01	0.984	pan	Hospitalized COVID-19
15506_34	LRP12	Low-density lipoprotein receptor-related protein 12	Q9Y561	LRP12	NA	Wald ratio	1	0.156	0.203	4.427E-01	0.984	pan	Hospitalized COVID-19
16606_85	Aldose reductase	Aldose reductase	P15121	AKR1B1	NA	Inverse variance weighted	2	0.128	0.167	4.428E-01	0.984	pan	Hospitalized COVID-19
13744_37	IL-3 Ra	Interleukin-3 receptor subunit alpha	P26951	IL3RA	NA	Inverse variance weighted	3	0.085	0.111	4.434E-01	0.984	pan	Hospitalized COVID-19
7227_75	COCH	Cochlin	O43405	COCH	NA	Inverse variance weighted	17	0.024	0.031	4.436E-01	0.984	pan	Hospitalized COVID-19
9385_4	GAA	Lysosomal alpha-glucosidase	P10253	GAA	NA	Inverse variance weighted	6	-0.027	0.035	4.448E-01	0.984	pan	Hospitalized COVID-19
3007_7	Siglec-9	Sialic acid-binding Ig-like lectin 9	Q9Y336	SIGLEC9	NA	Inverse variance weighted	7	-0.032	0.042	4.450E-01	0.984	pan	Hospitalized COVID-19
6611_8	KDEL1	KDEL motif-containing protein 1	Q6UW63	POGLUT2	NA	Wald ratio	1	0.179	0.234	4.453E-01	0.984	pan	Hospitalized COVID-19
18206_18	ADH6	Alcohol dehydrogenase 6	P28332	ADH6	NA	Inverse variance weighted	2	-0.154	0.202	4.453E-01	0.984	pan	Hospitalized COVID-19
10511_10	Collagen alpha-3(VI):isoform 3	Collagen alpha-3(VI) chain:isoform 3	P12111	COL6A3	NA	Inverse variance weighted	2	0.096	0.126	4.462E-01	0.984	pan	Hospitalized COVID-19
7083_74	MATN4	Matrilin-4	O95460	MATN4	NA	Inverse variance weighted	3	0.046	0.061	4.468E-01	0.984	pan	Hospitalized COVID-19
9021_1	TIM-1	Hepatitis A virus cellular receptor 1	Q96D42	HAVCR1	NA	Inverse variance weighted	7	-0.024	0.032	4.469E-01	0.984	pan	Hospitalized COVID-19
5509_7	EGF:ECD	Epidermal growth factor:Extracellular domain	P01133	EGF	NA	Inverse variance weighted	4	-0.085	0.113	4.485E-01	0.984	pan	Hospitalized COVID-19
6606_61	KISS1	Metastasis-suppressor KiSS-1	Q15726	KISS1	NA	Inverse variance weighted	14	0.021	0.028	4.490E-01	0.984	pan	Hospitalized COVID-19
15483_377	Agrin	Agrin	O00468	AGRN	NA	Inverse variance weighted	12	-0.033	0.044	4.490E-01	0.984	pan	Hospitalized COVID-19
4271_75	PFD5	Prefoldin subunit 5	Q99471	PFDN5	NA	Wald ratio	1	0.324	0.428	4.491E-01	0.984	pan	Hospitalized COVID-19
6442_6	BAGE3	B melanoma antigen 3	Q86Y29	BAGE3	NA	Wald ratio	1	-0.185	0.244	4.494E-01	0.984	pan	Hospitalized COVID-19
15667_39	BMP-4	Bone morphogenetic protein 4	P12644	BMP4	NA	Inverse variance weighted	3	-0.074	0.098	4.494E-01	0.984	pan	Hospitalized COVID-19
17396_23	ADH1A	Alcohol dehydrogenase 1A	P07327	ADH1A	NA	Inverse variance weighted	3	-0.058	0.076	4.494E-01	0.984	pan	Hospitalized COVID-19
4187_49	6-Phosphogluconate dehydrogenase	6-phosphogluconate dehydrogenase, decarboxylating	P52209	PGD	NA	Wald ratio	1	0.102	0.136	4.514E-01	0.984	pan	Hospitalized COVID-19
11098_1	PDXK	Pyridoxal kinase	O00764	PDXK	NA	Inverse variance weighted	6	-0.048	0.064	4.520E-01	0.984	pan	Hospitalized COVID-19
18215_5	THG1	Probable tRNA(His) guanylyltransferase	Q9NWX6	THG1L	NA	Inverse variance weighted	8	-0.018	0.024	4.521E-01	0.984	pan	Hospitalized COVID-19
10531_18	RASN	GTPase NRas	P01111	NRAS	NA	Wald ratio	1	-0.207	0.275	4.529E-01	0.984	pan	Hospitalized COVID-19
8970_9	RIPK2	Receptor-interacting serine/threonine-protein kinase 2	O43353	RIPK2	NA	Wald ratio	1	-0.135	0.180	4.529E-01	0.984	pan	Hospitalized COVID-19
8973_23	FCRL4:ECD	Fc receptor-like protein 4:Extracellular domain	Q96PJ5	FCRL4	NA	Inverse variance weighted	19	0.023	0.031	4.530E-01	0.984	pan	Hospitalized COVID-19
18311_44	DCTN6	Dynactin subunit 6	O00399	DCTN6	NA	Inverse variance weighted	2	-0.106	0.141	4.533E-01	0.984	pan	Hospitalized COVID-19
12571_14	ARL3	ADP-ribosylation factor-like protein 3	P36405	ARL3	NA	Inverse variance weighted	2	0.083	0.110	4.533E-01	0.984	pan	Hospitalized COVID-19
5116_62	ROBO2	Roundabout homolog 2	Q9HCK4	ROBO2	NA	Inverse variance weighted	2	0.088	0.118	4.554E-01	0.984	pan	Hospitalized COVID-19
17765_3	SPN1	Snurportin-1	O95149	SNUPN	NA	Wald ratio	1	0.057	0.077	4.559E-01	0.984	pan	Hospitalized COVID-19
11333_82	RHG25	Rho GTPase-activating protein 25	P42331	ARHGAP25	NA	Wald ratio	1	0.053	0.071	4.566E-01	0.984	pan	Hospitalized COVID-19
7933_75	ADA22	Disintegrin and metalloproteinase domain-containing protein 22	Q9P0K1	ADAM22	NA	Inverse variance weighted	5	-0.031	0.041	4.569E-01	0.984	pan	Hospitalized COVID-19
9398_30	GALP	Galanin-like peptide	Q9UBC7	GALP	NA	Wald ratio	1	-0.124	0.167	4.570E-01	0.984	pan	Hospitalized COVID-19
7897_75	DLG3	Disks large homolog 3	Q92796	DLG3	NA	Inverse variance weighted	5	-0.078	0.104	4.574E-01	0.984	pan	Hospitalized COVID-19
13622_16	2A5A	Serine/threonine-protein phosphatase 2A 56 kDa regulatory subunit alpha isoform	Q15172	PPP2R5A	NA	Wald ratio	1	-0.155	0.208	4.578E-01	0.984	pan	Hospitalized COVID-19
14134_49	AMGO2	Amphoterin-induced protein 2	Q86SJ2	AMIGO2	NA	Inverse variance weighted	3	-0.063	0.085	4.583E-01	0.984	pan	Hospitalized COVID-19
3413_50	BFL1	Bcl-2-related protein A1	Q16548	BCL2A1	NA	Wald ratio	1	-0.169	0.228	4.584E-01	0.984	pan	Hospitalized COVID-19
8231_122	VEGF sR1	Vascular endothelial growth factor receptor 1	P17948	FLT1	NA	Inverse variance weighted	4	0.075	0.102	4.590E-01	0.984	pan	Hospitalized COVID-19
5464_52	GRB2 adapter protein	Growth factor receptor-bound protein 2	P62993	GRB2	NA	Wald ratio	1	0.210	0.284	4.593E-01	0.984	pan	Hospitalized COVID-19
6252_62	Secretoglobin family 3A member 1	Secretoglobin family 3A member 1	Q96QR1	SCGB3A1	NA	Inverse variance weighted	28	0.040	0.054	4.594E-01	0.984	pan	Hospitalized COVID-19
7127_3	Apo A-II	Apolipoprotein A-II	P02652	APOA2	NA	Inverse variance weighted	11	-0.036	0.048	4.595E-01	0.984	pan	Hospitalized COVID-19
11192_168	TINAL	Tubulointerstitial nephritis antigen-like	Q9GZM7	TINAGL1	NA	Inverse variance weighted	8	-0.038	0.052	4.599E-01	0.984	pan	Hospitalized COVID-19
8088_56	CA210	Type III endosome membrane protein TEMP	Q8IVY1	C1orf210	NA	Wald ratio	1	-0.186	0.252	4.604E-01	0.984	pan	Hospitalized COVID-19
9906_21	TICN3	Testican-3	Q9BQ16	SPOCK3	NA	Inverse variance weighted	12	0.017	0.023	4.606E-01	0.984	pan	Hospitalized COVID-19
13565_2	p130	Retinoblastoma-like protein 2	Q08999	RBL2	NA	Inverse variance weighted	6	0.051	0.069	4.610E-01	0.984	pan	Hospitalized COVID-19
4534_10	BSSP4	Brain-specific serine protease 4	Q9GZN4	PRSS22	NA	Inverse variance weighted	4	-0.032	0.043	4.610E-01	0.984	pan	Hospitalized COVID-19
17685_9	Apo A-IV	Apolipoprotein A-IV	P06727	APOA4	NA	Wald ratio	1	-0.164	0.222	4.612E-01	0.984	pan	Hospitalized COVID-19
16605_2	C1T9A	Complement C1q and tumor necrosis factor-related protein 9A	P0C862	C1QTNF9	NA	Wald ratio	1	0.041	0.056	4.618E-01	0.984	pan	Hospitalized COVID-19
15573_110	CSPG3	Neurocan core protein	O14594	NCAN	NA	Inverse variance weighted	11	-0.046	0.062	4.620E-01	0.984	pan	Hospitalized COVID-19
8894_80	hnRNP A/B	Heterogeneous nuclear ribonucleoprotein A/B	Q99729	HNRNPAB	NA	Inverse variance weighted	2	-0.090	0.122	4.621E-01	0.984	pan	Hospitalized COVID-19
17692_2	BT3A3	Butyrophilin subfamily 3 member A3	O00478	BTN3A3	NA	Inverse variance weighted	10	-0.016	0.022	4.625E-01	0.984	pan	Hospitalized COVID-19
13657_2	PNKP	Bifunctional polynucleotide phosphatase/kinase	Q96T60	PNKP	NA	Wald ratio	1	0.075	0.102	4.631E-01	0.984	pan	Hospitalized COVID-19
12895_28	DGKB	Diacylglycerol kinase beta	Q9Y6T7	DGKB	NA	Wald ratio	1	0.131	0.178	4.636E-01	0.984	pan	Hospitalized COVID-19
3580_25	a1-Antitrypsin	Alpha-1-antitrypsin	P01009	SERPINA1	NA	Inverse variance weighted	6	0.042	0.058	4.639E-01	0.984	pan	Hospitalized COVID-19
17843_30	PPCS	Phosphopantothenate--cysteine ligase	Q9HAB8	PPCS	NA	Inverse variance weighted	2	0.063	0.086	4.641E-01	0.984	pan	Hospitalized COVID-19
19372_7	MDGA2	MAM domain-containing glycosylphosphatidylinositol anchor protein 2	Q7Z553	MDGA2	NA	Inverse variance weighted	19	-0.011	0.015	4.648E-01	0.984	pan	Hospitalized COVID-19
19233_75	ATOX1	Copper transport protein ATOX1	O00244	ATOX1	NA	Wald ratio	1	0.144	0.198	4.665E-01	0.984	pan	Hospitalized COVID-19
7218_87	AT1B2	Sodium/potassium-transporting ATPase subunit beta-2	P14415	ATP1B2	NA	Inverse variance weighted	4	-0.054	0.075	4.673E-01	0.984	pan	Hospitalized COVID-19
13268_45	WNT5A	Protein Wnt-5a	P41221	WNT5A	NA	Inverse variance weighted	5	0.087	0.120	4.675E-01	0.984	pan	Hospitalized COVID-19
3025_50	bFGF	Fibroblast growth factor 2	P09038	FGF2	NA	Inverse variance weighted	7	-0.021	0.028	4.676E-01	0.984	pan	Hospitalized COVID-19
5108_72	Notch-3	Neurogenic locus notch homolog protein 3	Q9UM47	NOTCH3	NA	Inverse variance weighted	2	-0.075	0.104	4.678E-01	0.984	pan	Hospitalized COVID-19

9928_125	LECT1	Leukocyte cell-derived chemotaxin 1	O75829	CNMD	NA	Wald ratio	1	-0.126	0.174	4.684E-01	0.984	pan	Hospitalized COVID-19
4479_14	C1-Esterase Inhibitor	Plasma protease C1 inhibitor	P05155	SERPING1	NA	Inverse variance weighted	14	-0.019	0.026	4.691E-01	0.984	pan	Hospitalized COVID-19
19113_66	SH3L2	SH3 domain-binding glutamic acid-rich-like protein 2	Q9UJC5	SH3BGRL2	NA	Wald ratio	1	-0.072	0.100	4.692E-01	0.984	pan	Hospitalized COVID-19
8364_74	UST	Uronyl 2-sulfotransferase	Q9Y2C2	UST	NA	Inverse variance weighted	5	-0.043	0.060	4.694E-01	0.984	pan	Hospitalized COVID-19
3040_59	MIP-1a	C-C motif chemokine 3	P10147	CCL3	NA	Wald ratio	1	0.150	0.208	4.696E-01	0.984	pan	Hospitalized COVID-19
15566_10	Calponin-1	Calponin-1	P51911	CNN1	NA	Inverse variance weighted	10	0.044	0.062	4.704E-01	0.984	pan	Hospitalized COVID-19
14708_59	CO8G	Complement component C8 gamma chain	P07360	C8G	NA	Inverse variance weighted	11	-0.032	0.045	4.704E-01	0.984	pan	Hospitalized COVID-19
5493_17	WNK3	Serine/threonine-protein kinase WNK3	Q9BYP7	WNK3	NA	Wald ratio	1	0.146	0.203	4.710E-01	0.984	pan	Hospitalized COVID-19
12855_16	CT032	Cas scaffolding protein family member 4	Q9NQ75	CASS4	NA	Wald ratio	1	-0.141	0.196	4.711E-01	0.984	pan	Hospitalized COVID-19
16057_6	IGF-II receptor	Cation-independent mannose-6-phosphate receptor	P11717	IGF2R	NA	Inverse variance weighted	8	-0.022	0.031	4.712E-01	0.984	pan	Hospitalized COVID-19
12758_47	GRID2	Glutamate receptor ionotropic, delta-2	O43424	GRID2	NA	Inverse variance weighted	10	0.044	0.061	4.713E-01	0.984	pan	Hospitalized COVID-19
11152_46	kallikrein 13	Kallikrein-13	Q9UKR3	KLK13	NA	Inverse variance weighted	4	-0.086	0.119	4.716E-01	0.984	pan	Hospitalized COVID-19
16323_8	NRX3A	Neurexin-3	Q9Y4C0	NRXN3	NA	Inverse variance weighted	3	0.089	0.123	4.726E-01	0.984	pan	Hospitalized COVID-19
5542_22	NRP1	Neuropilin-1	O14786	NRP1	NA	Inverse variance weighted	8	-0.025	0.035	4.727E-01	0.984	pan	Hospitalized COVID-19
9348_1	C1RL1	Complement C1r subcomponent-like protein	Q9NZP8	C1RL	NA	Inverse variance weighted	2	0.142	0.198	4.729E-01	0.984	pan	Hospitalized COVID-19
3026_5	Calpastatin	Calpastatin	P20810	CAST	NA	Inverse variance weighted	4	-0.122	0.171	4.735E-01	0.984	pan	Hospitalized COVID-19
3050_7	vWF	von Willebrand factor	P04275	VWF	NA	Inverse variance weighted	3	-0.093	0.130	4.738E-01	0.984	pan	Hospitalized COVID-19
18235_16	PGP	Glycerol-3-phosphate phosphatase	A6NDG6	PGP	NA	Inverse variance weighted	2	0.064	0.090	4.739E-01	0.984	pan	Hospitalized COVID-19
11260_47	SPA4L	SUN domain-containing protein 5	Q8TC36	SUN5	NA	Inverse variance weighted	4	0.080	0.112	4.740E-01	0.984	pan	Hospitalized COVID-19
3449_58	Kallistatin	Kallistatin	P29622	SERPINA4	NA	Inverse variance weighted	8	0.055	0.077	4.745E-01	0.984	pan	Hospitalized COVID-19
17341_89	THIC	Acetyl-CoA acetyltransferase, cytosolic	Q9BWD1	ACAT2	NA	Inverse variance weighted	3	0.025	0.036	4.746E-01	0.984	pan	Hospitalized COVID-19
3314_74	GFRa-1	GDNF family receptor alpha-1	P56159	GFRA1	NA	Inverse variance weighted	3	0.039	0.054	4.746E-01	0.984	pan	Hospitalized COVID-19
15391_114	GAS-6	Growth arrest-specific protein 6	Q14393	GAS6	NA	Inverse variance weighted	5	-0.024	0.034	4.753E-01	0.984	pan	Hospitalized COVID-19
9986_14	Neuropeptide W	Neuropeptide W	Q8N729	NPW	NA	Inverse variance weighted	5	-0.029	0.040	4.757E-01	0.984	pan	Hospitalized COVID-19
9039_47	TOIP1:Nuclear	Torsin-1A-interacting protein 1:Nuclear domain	Q5JTV8	TOR1AIP1	NA	Inverse variance weighted	2	-0.106	0.149	4.772E-01	0.984	pan	Hospitalized COVID-19
6383_90	TLL1	Tolloid-like protein 1	O43897	TLL1	NA	Inverse variance weighted	4	0.113	0.158	4.774E-01	0.984	pan	Hospitalized COVID-19
8303_102	MYG1	UPF0160 protein MYG1, mitochondrial	Q9HB07	MYG1	NA	Wald ratio	1	0.118	0.167	4.774E-01	0.984	pan	Hospitalized COVID-19
2635_61	Layilin	Layilin	Q6UX15	LAYN	NA	Wald ratio	1	-0.132	0.186	4.776E-01	0.984	pan	Hospitalized COVID-19
19141_22	DAP1	Death-associated protein 1	P51397	DAP	NA	Inverse variance weighted	7	0.051	0.072	4.785E-01	0.984	pan	Hospitalized COVID-19
12433_8	ARL11	ADP-ribosylation factor-like protein 11	Q969Q4	ARL11	NA	Inverse variance weighted	2	-0.112	0.159	4.798E-01	0.984	pan	Hospitalized COVID-19
8034_6	DRGX	Dorsal root ganglia homeobox protein	A6NNA5	DRGX	NA	Inverse variance weighted	7	0.021	0.029	4.798E-01	0.984	pan	Hospitalized COVID-19
12008_3	CD7	T-cell antigen CD7	P09564	CD7	NA	Inverse variance weighted	4	0.082	0.116	4.798E-01	0.984	pan	Hospitalized COVID-19
13473_55	IP3KA	Inositol-trisphosphate 3-kinase A	P23677	ITPKA	NA	Wald ratio	1	0.104	0.147	4.805E-01	0.984	pan	Hospitalized COVID-19
17787_1	Enoyl-CoA hydratase	Enoyl-CoA hydratase, mitochondrial	P30084	ECHS1	NA	Wald ratio	1	0.135	0.193	4.829E-01	0.984	pan	Hospitalized COVID-19
10714_7	ACE	Angiotensin-converting enzyme	P12821	ACE	NA	Inverse variance weighted	13	-0.012	0.017	4.830E-01	0.984	pan	Hospitalized COVID-19
9251_28	MA2B2	Epididymis-specific alpha-mannosidase	Q9Y2E5	MAN2B2	NA	Inverse variance weighted	10	0.016	0.023	4.831E-01	0.984	pan	Hospitalized COVID-19
5451_1	ALCAM	CD166 antigen	Q13740	ALCAM	NA	Inverse variance weighted	5	0.052	0.074	4.833E-01	0.984	pan	Hospitalized COVID-19
18934_50	Tissue transglutaminase	Protein-glutamine gamma-glutamyltransferase 2	P21980	TGM2	NA	Wald ratio	1	0.038	0.055	4.833E-01	0.984	pan	Hospitalized COVID-19
12633_3	PTN9	Tyrosine-protein phosphatase non-receptor type 9	P43378	PTPN9	NA	Wald ratio	1	-0.125	0.178	4.840E-01	0.984	pan	Hospitalized COVID-19
9841_197	Multifunctional protein ADE2	Multifunctional protein ADE2	P22234	PAICS	NA	Wald ratio	1	0.091	0.131	4.842E-01	0.984	pan	Hospitalized COVID-19
9253_52	BGAT	Histo-blood group ABO system transferase	P16442	ABO	NA	Inverse variance weighted	13	0.031	0.044	4.850E-01	0.984	pan	Hospitalized COVID-19
17746_77	FIS1	Mitochondrial fission 1 protein	Q9Y3D6	FIS1	NA	Inverse variance weighted	2	0.106	0.152	4.856E-01	0.984	pan	Hospitalized COVID-19
4220_39	FER	Tyrosine-protein kinase Fer	P16591	FER	NA	Inverse variance weighted	2	0.073	0.104	4.858E-01	0.984	pan	Hospitalized COVID-19
5708_1	LEAP2	Liver-expressed antimicrobial peptide 2	Q969E1	LEAP2	NA	Inverse variance weighted	6	-0.032	0.045	4.860E-01	0.984	pan	Hospitalized COVID-19
5069_9	DAF	Complement decay-accelerating factor	P08174	CD55	NA	Inverse variance weighted	5	0.036	0.051	4.864E-01	0.984	pan	Hospitalized COVID-19
17777_31	SDSL	Serine dehydratase-like	Q96GA7	SDSL	NA	Inverse variance weighted	7	0.024	0.035	4.867E-01	0.984	pan	Hospitalized COVID-19
13669_6	FGFR-3:ECD	Fibroblast growth factor receptor 3:Extracellular domain	P22607	FGFR3	NA	Inverse variance weighted	3	-0.059	0.085	4.870E-01	0.984	pan	Hospitalized COVID-19
9940_35	DUS28	Dual specificity phosphatase 28	Q4G0W2	DUSP28	NA	Inverse variance weighted	3	-0.043	0.063	4.872E-01	0.984	pan	Hospitalized COVID-19
19153_53	MPPD2	Metallophosphoesterase MPPED2	Q15777	MPPED2	NA	Inverse variance weighted	2	0.068	0.099	4.873E-01	0.984	pan	Hospitalized COVID-19
16890_37	ATL1	ADAMTS-like protein 1	Q8N6G6	ADAMTSL1	NA	Inverse variance weighted	8	0.027	0.040	4.883E-01	0.984	pan	Hospitalized COVID-19
11516_7	FABPL	Fatty acid-binding protein, liver	P07148	FABP1	NA	Wald ratio	1	-0.063	0.091	4.889E-01	0.984	pan	Hospitalized COVID-19
3173_49	ASAHL	N-acylethanolamine-hydrolyzing acid amidase	Q02083	NAAA	NA	Inverse variance weighted	24	-0.019	0.028	4.889E-01	0.984	pan	Hospitalized COVID-19
3799_11	Carbonic anhydrase III	Carbonic anhydrase 3	P07451	CA3	NA	Inverse variance weighted	5	0.048	0.070	4.893E-01	0.984	pan	Hospitalized COVID-19
2480_58	TIMP-3	Metalloproteinase inhibitor 3	P35625	TIMP3	NA	Inverse variance weighted	10	0.015	0.022	4.901E-01	0.984	pan	Hospitalized COVID-19
10832_24	B4GT6	Beta-1,4-galactosyltransferase 6	Q9UBX8	B4GALT6	NA	Inverse variance weighted	12	-0.015	0.022	4.902E-01	0.984	pan	Hospitalized COVID-19
9844_138	ACTN2	Alpha-actinin-2	P35609	ACTN2	NA	Inverse variance weighted	5	-0.087	0.127	4.905E-01	0.984	pan	Hospitalized COVID-19
12486_8	GLRX2	Glutaredoxin-2, mitochondrial	Q9NS18	GLRX2	NA	Inverse variance weighted	3	0.035	0.051	4.909E-01	0.984	pan	Hospitalized COVID-19
10042_8	SGK3	Serine/threonine-protein kinase Sgk3	Q96BR1	SGK3	NA	Wald ratio	1	-0.141	0.205	4.912E-01	0.984	pan	Hospitalized COVID-19
13434_172	PARVA	Alpha-parvin	Q9NVD7	PARVA	NA	Wald ratio	1	-0.081	0.118	4.913E-01	0.984	pan	Hospitalized COVID-19
19361_78	MATN3	Matrilin-3	O15232	MATN3	NA	Inverse variance weighted	14	0.016	0.023	4.914E-01	0.984	pan	Hospitalized COVID-19
5687_5	GLU2B	Glucosidase 2 subunit beta	P14314	PRKCSH	NA	Inverse variance weighted	3	-0.089	0.130	4.919E-01	0.984	pan	Hospitalized COVID-19
19496_1	NPL	N-acetylneuraminate lyase	Q9BXD5	NPL	NA	Inverse variance weighted	4	-0.039	0.056	4.922E-01	0.984	pan	Hospitalized COVID-19
9796_4	CEL	Bile salt-activated lipase	P19835	CEL	NA	Inverse variance weighted	7	0.023	0.034	4.924E-01	0.984	pan	Hospitalized COVID-19
9468_8	Lectin, mannose-binding 2	Vesicular integral-membrane protein VIP36	Q12907	LMAN2	NA	Wald ratio	1	0.082	0.120	4.924E-01	0.984	pan	Hospitalized COVID-19
3066_12	Galectin-3	Galectin-3	P17931	LGALS3	NA	Inverse variance weighted	6	-0.020	0.030	4.924E-01	0.984	pan	Hospitalized COVID-19
3845_51	DLRB1	Dynein light chain roadblock-type 1	Q9NP97	DYNLRB1	NA	Inverse variance weighted	8	0.039	0.057	4.926E-01	0.984	pan	Hospitalized COVID-19
13242_134	SHAN3	SH3 and multiple ankyrin repeat domains protein 3	Q9BYB0	SHANK3	NA	Inverse variance weighted	3	0.094	0.138	4.944E-01	0.985	pan	Hospitalized COVID-19
3074_6	LBP	Lipopolysaccharide-binding protein	P18428	LBP	NA	Inverse variance weighted	10	0.034	0.049	4.945E-01	0.985	pan	Hospitalized COVID-19
9886_28	XRCC4	DNA repair protein XRCC4	Q13426	XRCC4	NA	Inverse variance weighted	2	0.055	0.081	4.953E-01	0.985	pan	Hospitalized COVID-19
19289_29	DCUP	Uroporphyrinogen decarboxylase	P06132	UROD	NA	Inverse variance weighted	5	0.026	0.039	4.957E-01	0.985	pan	Hospitalized COVID-19
4240_31	M2-PK	Pyruvate kinase PKM	P14618	PKM	NA	Inverse variance weighted	3	-0.088	0.130	4.959E-01	0.985	pan	Hospitalized COVID-19
7208_60	MGT4C	Alpha-1,3-mannosyl-glycoprotein 4-beta-N-acetylglucosaminyltransferase C	Q9UBM8	MGAT4C	NA	Wald ratio	1	0.114	0.167	4.961E-01	0.985	pan	Hospitalized COVID-19
3481_87	XPNPEP1	Xaa-Pro aminopeptidase 1	Q9NQW7	XPNPEP1	NA	Wald ratio	1	0.146	0.215	4.968E-01	0.985	pan	Hospitalized COVID-19
5006_71	MK13	Mitogen-activated protein kinase 13	O15264	MAPK13	NA	Wald ratio	1	-0.077	0.114	4.970E-01	0.985	pan	Hospitalized COVID-19
18942_11	PSB9	Proteasome subunit beta type-9	P28065	PSMB9	NA	Wald ratio	1	0.152	0.225	4.991E-01	0.986	pan	Hospitalized COVID-19
9321_400	NMB	Neuromedin-B	P08949	NMB	NA	Inverse variance weighted	2	-0.043	0.064	5.000E-01	0.986	pan	Hospitalized COVID-19
8780_2	PCD10:CD	Protocadherin-10:Cytoplasmic domain	Q9P2E7	PCDH10	NA	Inverse variance weighted	6	0.040	0.059	5.016E-01	0.986	pan	Hospitalized COVID-19
3292_75	CD48	CD48 antigen	P09326	CD48	NA	Inverse variance weighted	8	0.105	0.156	5.017E-01	0.986	pan	Hospitalized COVID-19

15589_1	Gc-Globulin, Mixed Type	Vitamin D-binding protein	P02774	GC	NA	Inverse variance weighted	4	0.044	0.066	5.031E-01	0.986	pan	Hospitalized COVID-19
9313_27	CBLN1	Cerebellin-1	P23435	CBLN1	NA	Inverse variance weighted	31	-0.016	0.024	5.035E-01	0.986	pan	Hospitalized COVID-19
8992_1	TMEM2	Transmembrane protein 2	Q9UHN6	CEMIP2	NA	Inverse variance weighted	4	0.060	0.090	5.038E-01	0.986	pan	Hospitalized COVID-19
19553_14	STX1a	Syntaxin-1A	Q16623	STX1A	NA	Inverse variance weighted	4	0.056	0.084	5.041E-01	0.986	pan	Hospitalized COVID-19
3459_49	PDGF Rb	Platelet-derived growth factor receptor beta	P09619	PDGFRB	NA	Inverse variance weighted	12	-0.008	0.011	5.050E-01	0.986	pan	Hospitalized COVID-19
7655_11	N-terminal pro-BNP	N-terminal pro-BNP	P16860	NPPB	NA	Inverse variance weighted	4	-0.047	0.071	5.051E-01	0.986	pan	Hospitalized COVID-19
5021_13	PPase	Inorganic pyrophosphatase	Q15181	PPA1	NA	Inverse variance weighted	5	-0.039	0.058	5.053E-01	0.986	pan	Hospitalized COVID-19
4991_12	GPC5	Glypican-5	P78333	GPC5	NA	Inverse variance weighted	11	-0.024	0.035	5.053E-01	0.986	pan	Hospitalized COVID-19
13948_50	GABR2:ECD	Gamma-aminobutyric acid type B receptor subunit 2:Extracellular domain	O75899	GABBR2	NA	Wald ratio	1	0.124	0.186	5.055E-01	0.986	pan	Hospitalized COVID-19
5437_63	FABP	Fatty acid-binding protein, heart	P05413	FABP3	NA	Inverse variance weighted	2	-0.105	0.157	5.059E-01	0.986	pan	Hospitalized COVID-19
3905_62	UBE2N	Ubiquitin-conjugating enzyme E2 N	P61088	UBE2N	NA	Wald ratio	1	-0.150	0.226	5.062E-01	0.986	pan	Hospitalized COVID-19
13955_33	DAPK1	Death-associated protein kinase 1	P53355	DAPK1	NA	Inverse variance weighted	3	0.089	0.134	5.062E-01	0.986	pan	Hospitalized COVID-19
3364_76	Cathepsin V	Cathepsin L2	O60911	CTSV	NA	Inverse variance weighted	12	-0.028	0.043	5.067E-01	0.986	pan	Hospitalized COVID-19
11356_19	DGC14	Protein DGCR14	Q96DF8	ESS2	NA	Wald ratio	1	0.065	0.098	5.072E-01	0.986	pan	Hospitalized COVID-19
17794_6	Phosphomannomutase 2	Phosphomannomutase 2	O15305	PMM2	NA	Inverse variance weighted	5	0.064	0.097	5.109E-01	0.986	pan	Hospitalized COVID-19
15523_9	HEM2	Delta-aminolevulinic acid dehydratase	P13716	ALAD	NA	Inverse variance weighted	8	0.033	0.051	5.111E-01	0.986	pan	Hospitalized COVID-19
13686_2	IL-5 Ra	Interleukin-5 receptor subunit alpha	Q01344	IL5RA	NA	Inverse variance weighted	15	0.022	0.034	5.114E-01	0.986	pan	Hospitalized COVID-19
4126_22	BPI	Bactericidal permeability-increasing protein	P17213	BPI	NA	Inverse variance weighted	13	-0.024	0.037	5.116E-01	0.986	pan	Hospitalized COVID-19
7980_72	B3GN2	N-acetyllactosaminide beta-1,3-N-acetylglucosaminyltransferase 2	Q9NY97	B3GNT2	NA	Inverse variance weighted	5	0.062	0.095	5.121E-01	0.986	pan	Hospitalized COVID-19
10708_3	GON2	Progonadoliberin-2	O43555	GNRH2	NA	Wald ratio	1	-0.065	0.099	5.122E-01	0.986	pan	Hospitalized COVID-19
4467_49	SPARCL1	SPARC-like protein 1	Q14515	SPARCL1	NA	Inverse variance weighted	10	0.015	0.024	5.125E-01	0.986	pan	Hospitalized COVID-19
17758_79	DCXR	L-xylulose reductase	Q7Z4W1	DCXR	NA	Inverse variance weighted	2	0.070	0.106	5.127E-01	0.986	pan	Hospitalized COVID-19
13381_49	B4GT1	Beta-1,4-galactosyltransferase 1	P15291	B4GALT1	NA	Inverse variance weighted	6	0.043	0.066	5.135E-01	0.986	pan	Hospitalized COVID-19
14106_46	GDP/GTP exchange protein	Rap1 GTPase-GDP dissociation stimulator 1	P52306	RAP1GDS1	NA	Wald ratio	1	-0.132	0.202	5.136E-01	0.986	pan	Hospitalized COVID-19
6570_1	CODA1	Collagen alpha-1(XIII) chain	Q5TAT6	COL13A1	NA	Inverse variance weighted	3	-0.094	0.144	5.139E-01	0.986	pan	Hospitalized COVID-19
16055_3	complement factor H-related 5	Complement factor H-related protein 5	Q9BXR6	CFHR5	NA	Inverse variance weighted	6	-0.053	0.081	5.141E-01	0.986	pan	Hospitalized COVID-19
2811_27	Angiopoietin-1	Angiopoietin-1	Q15389	ANGPT1	NA	Inverse variance weighted	2	0.079	0.122	5.152E-01	0.986	pan	Hospitalized COVID-19
17697_2	OVCA2	Esterase OVCA2	Q8WZ82	OVCA2	NA	Wald ratio	1	-0.073	0.113	5.160E-01	0.986	pan	Hospitalized COVID-19
15579_26	ENPP6	Ectonucleotide pyrophosphatase/phosphodiesterase family member 6	Q6UWR7	ENPP6	NA	Inverse variance weighted	2	0.086	0.132	5.160E-01	0.986	pan	Hospitalized COVID-19
5584_21	Holo-TC II	Holo-Transcobalamin-2	P20062	TCN2	NA	Inverse variance weighted	12	0.025	0.039	5.165E-01	0.986	pan	Hospitalized COVID-19
6207_10	prosaposin	Prosaposin	P07602	PSAP	NA	Inverse variance weighted	5	0.076	0.117	5.166E-01	0.986	pan	Hospitalized COVID-19
5457_5	COLEC12	Collectin-12	Q5KU26	COLEC12	NA	Inverse variance weighted	12	0.047	0.073	5.170E-01	0.986	pan	Hospitalized COVID-19
3403_1	TPSB2	Tryptase beta-2	P20231	TPSB2	NA	Inverse variance weighted	10	0.025	0.039	5.170E-01	0.986	pan	Hospitalized COVID-19
18432_32	RALB	Ras-related protein Ral-B	P11234	RALB	NA	Wald ratio	1	0.112	0.173	5.174E-01	0.986	pan	Hospitalized COVID-19
18326_50	MAGA8	Melanoma-associated antigen 8	P43361	MAGEA8	NA	Wald ratio	1	0.156	0.241	5.188E-01	0.986	pan	Hospitalized COVID-19
13671_40	Elastase	Neutrophil elastase	P08246	ELANE	NA	Inverse variance weighted	12	-0.033	0.051	5.204E-01	0.986	pan	Hospitalized COVID-19
5658_64	coagulation factor XIII B	Coagulation factor XIII B chain	P05160	F13B	NA	Inverse variance weighted	3	-0.091	0.142	5.207E-01	0.986	pan	Hospitalized COVID-19
11347_9	Transaldolase	Transaldolase	P37837	TALDO1	NA	Inverse variance weighted	2	-0.064	0.099	5.215E-01	0.986	pan	Hospitalized COVID-19
4133_54	Granzyme B	Granzyme B	P10144	GZMB	NA	Wald ratio	1	0.071	0.110	5.221E-01	0.986	pan	Hospitalized COVID-19
9092_33	ANGL1:N-term	Angiopoietin-related protein 1:N-term	O95841	ANGPTL1	NA	Wald ratio	1	0.125	0.195	5.225E-01	0.986	pan	Hospitalized COVID-19
13450_49	UBP8	Ubiquitin carboxyl-terminal hydrolase 8	P40818	USP8	NA	Wald ratio	1	0.080	0.125	5.226E-01	0.986	pan	Hospitalized COVID-19
19143_38	NB5R2	NADH-cytochrome b5 reductase 2	Q6BCY4	CYB5R2	NA	Inverse variance weighted	7	-0.034	0.053	5.238E-01	0.986	pan	Hospitalized COVID-19
10512_13	IL3RB:ECD	Cytokine receptor common subunit beta:Extracellular domain	P32927	CSF2RB	NA	Inverse variance weighted	6	-0.011	0.017	5.243E-01	0.986	pan	Hospitalized COVID-19
15540_6	Vimentin	Vimentin	P08670	VIM	NA	Inverse variance weighted	2	0.079	0.125	5.245E-01	0.986	pan	Hospitalized COVID-19
3310_62	FCG2B	Low affinity immunoglobulin gamma Fc region receptor II-b	P31994	FCGR2B	NA	Inverse variance weighted	12	0.030	0.048	5.247E-01	0.986	pan	Hospitalized COVID-19
9950_229	LAG-3	Lymphocyte activation gene 3 protein	P18627	LAG3	NA	Inverse variance weighted	4	-0.085	0.133	5.252E-01	0.986	pan	Hospitalized COVID-19
15620_4	NLGN1	Neuroligin-1	Q8N2Q7	NLGN1	NA	Inverse variance weighted	3	0.097	0.153	5.253E-01	0.986	pan	Hospitalized COVID-19
12334_25	cSHMT	Serine hydroxymethyltransferase, cytosolic	P34896	SHMT1	NA	Inverse variance weighted	12	-0.014	0.022	5.254E-01	0.986	pan	Hospitalized COVID-19
13632_10	Zyxin	Zyxin	Q15942	ZYX	NA	Wald ratio	1	-0.143	0.225	5.257E-01	0.986	pan	Hospitalized COVID-19
10852_114	HNRDL	Heterogeneous nuclear ribonucleoprotein D-like	O14979	HNRNPDL	NA	Wald ratio	1	-0.120	0.190	5.258E-01	0.986	pan	Hospitalized COVID-19
19115_13	CMBL	Carboxymethylenebutenolidase homolog	Q96DG6	CMBL	NA	Wald ratio	1	0.042	0.067	5.258E-01	0.986	pan	Hospitalized COVID-19
10880_38	F163B	Protein FAM163B	P0C2L3	FAM163B	NA	Inverse variance weighted	3	-0.090	0.141	5.265E-01	0.986	pan	Hospitalized COVID-19
15610_72	LAP	Cytosol aminopeptidase	P28838	LAP3	NA	Inverse variance weighted	2	0.091	0.144	5.266E-01	0.986	pan	Hospitalized COVID-19
5355_69	LIGHT	Tumor necrosis factor ligand superfamily member 14	O43557	TNFSF14	NA	Wald ratio	1	-0.064	0.100	5.273E-01	0.986	pan	Hospitalized COVID-19
4332_6	CLC1B	C-type lectin domain family 1 member B	Q9P126	CLEC1B	NA	Wald ratio	1	-0.091	0.143	5.274E-01	0.986	pan	Hospitalized COVID-19
19561_216	PLXD1	Plexin-D1	Q9Y4D7	PLXND1	NA	Inverse variance weighted	6	0.028	0.044	5.274E-01	0.986	pan	Hospitalized COVID-19
13931_22	PSMD9	26S proteasome non-ATPase regulatory subunit 9	O00233	PSMD9	NA	Wald ratio	1	0.052	0.082	5.281E-01	0.986	pan	Hospitalized COVID-19
16322_10	PACAP	Marginal zone B- and B1-cell-specific protein	Q8WU39	MZB1	NA	Inverse variance weighted	10	0.045	0.071	5.283E-01	0.986	pan	Hospitalized COVID-19
5703_26	NOE1	Noelin	Q99784	OLFM1	NA	Inverse variance weighted	3	0.052	0.082	5.284E-01	0.986	pan	Hospitalized COVID-19
7970_315	NAR3	Ecto-ADP-ribosyltransferase 3	Q13508	ART3	NA	Inverse variance weighted	9	0.024	0.038	5.292E-01	0.986	pan	Hospitalized COVID-19
6321_65	PCDGA	Protocadherin gamma-A10	Q9Y5H3	PCDHGA10	NA	Inverse variance weighted	3	0.074	0.118	5.292E-01	0.986	pan	Hospitalized COVID-19
19558_10	LRP4	Low-density lipoprotein receptor-related protein 4	O75096	LRP4	NA	Inverse variance weighted	10	0.026	0.042	5.298E-01	0.986	pan	Hospitalized COVID-19
17686_27	TBCB	Tubulin-folding cofactor B	Q99426	TBCB	NA	Wald ratio	1	0.104	0.166	5.309E-01	0.986	pan	Hospitalized COVID-19
14158_17	Annexin V	Annexin A5	P08758	ANXA5	NA	Wald ratio	1	0.047	0.075	5.315E-01	0.986	pan	Hospitalized COVID-19
11196_31	Collagen alpha-3(VI):BPTI/Kunitz inhibitor	Collagen alpha-3(VI) chain:Bovine pancreatic trypsin inhibitor/Kunitz inhibitor domain, isoform 1	P12111	COL6A3	NA	Inverse variance weighted	2	0.116	0.185	5.316E-01	0.986	pan	Hospitalized COVID-19
12585_39	ERCC1	DNA excision repair protein ERCC-1	P07992	ERCC1	NA	Wald ratio	1	0.125	0.201	5.318E-01	0.986	pan	Hospitalized COVID-19
3803_10	CYTD	Cystatin-D	P28325	CST5	NA	Inverse variance weighted	17	0.022	0.036	5.318E-01	0.986	pan	Hospitalized COVID-19
3196_6	HPLN1	Hyaluronan and proteoglycan link protein 1	P10915	HAPLN1	NA	Inverse variance weighted	3	0.049	0.079	5.336E-01	0.986	pan	Hospitalized COVID-19
11265_8	Retinal dehydrogenase 1	Retinal dehydrogenase 1	P00352	ALDH1A1	NA	Wald ratio	1	-0.073	0.118	5.338E-01	0.986	pan	Hospitalized COVID-19
4771_10	ASM3A	Acid sphingomyelinase-like phosphodiesterase 3a	Q92484	SMPDL3A	NA	Inverse variance weighted	3	0.028	0.045	5.341E-01	0.986	pan	Hospitalized COVID-19
19228_11	BAG-2	BAG family molecular chaperone regulator 2	O95816	BAG2	NA	Inverse variance weighted	2	-0.269	0.434	5.345E-01	0.986	pan	Hospitalized COVID-19
3298_52	Contactin-4	Contactin-4	Q8IWV2	CNTN4	NA	Inverse variance weighted	8	0.018	0.029	5.346E-01	0.986	pan	Hospitalized COVID-19
6899_37	PHS2	Pterin-4-alpha-carbinolamine dehydratase 2	Q9H0N5	PCBD2	NA	Inverse variance weighted	4	0.106	0.172	5.352E-01	0.986	pan	Hospitalized COVID-19
12684_5	ADSV	Adseverin	Q9Y6U3	SCIN	NA	Wald ratio	1	-0.080	0.130	5.357E-01	0.986	pan	Hospitalized COVID-19
5103_30	MO2R1:ECD	Cell surface glycoprotein CD200 receptor 1:Isoform 4, Extracellular Domain	Q8TD46	CD200R1	NA	Wald ratio	1	0.057	0.092	5.363E-01	0.986	pan	Hospitalized COVID-19
2831_29	Kallikrein 11	Kallikrein-11	Q9UBX7	KLK11	NA	Inverse variance weighted	8	0.028	0.046	5.368E-01	0.986	pan	Hospitalized COVID-19
13748_4	MCP-2	C-C motif chemokine 8	P80075	CCL8	NA	Inverse variance weighted	10	0.035	0.057	5.370E-01	0.986	pan	Hospitalized COVID-19
9322_15	RCN1	Reticulocalbin-1	Q15293	RCN1	NA	Inverse variance weighted	2	0.089	0.144	5.373E-01	0.986	pan	Hospitalized COVID-19

2979_8	ENA-78	C-X-C motif chemokine 5	P42830	CXCL5	NA	Wald ratio	1	-0.176	0.286	5.376E-01	0.986	pan	Hospitalized COVID-19
5465_32	H6ST1	Heparan-sulfate 6-O-sulfotransferase 1	O60243	HS6ST1	NA	Inverse variance weighted	2	0.039	0.063	5.383E-01	0.986	pan	Hospitalized COVID-19
4471_50	TGM3	Protein-glutamine gamma-glutamyltransferase E	Q08188	TGM3	NA	Inverse variance weighted	5	0.016	0.027	5.384E-01	0.986	pan	Hospitalized COVID-19
16918_198	TLR3	Toll-like receptor 3	O15455	TLR3	NA	Inverse variance weighted	9	0.015	0.025	5.385E-01	0.986	pan	Hospitalized COVID-19
18189_12	VPS25	Vacuolar protein-sorting-associated protein 25	Q9BRG1	VPS25	NA	Inverse variance weighted	2	0.107	0.173	5.386E-01	0.986	pan	Hospitalized COVID-19
5731_1	ISK6	Serine protease inhibitor Kazal-type 6	Q6UWN8	SPINK6	NA	Inverse variance weighted	13	-0.015	0.024	5.402E-01	0.986	pan	Hospitalized COVID-19
19159_9	DNA polymerase subunit delta 4	DNA polymerase delta subunit 4	Q9HCU8	POLD4	NA	Wald ratio	1	0.145	0.236	5.403E-01	0.986	pan	Hospitalized COVID-19
18435_40	UBX2B	UBX domain-containing protein 2B	Q14CS0	UBXN2B	NA	Wald ratio	1	-0.106	0.174	5.404E-01	0.986	pan	Hospitalized COVID-19
7918_114	Amylase, alpha 1A	Alpha-amylase 1	P04745	AMY1A	NA	Inverse variance weighted	17	0.026	0.043	5.412E-01	0.986	pan	Hospitalized COVID-19
3421_54	CD30 Ligand	Tumor necrosis factor ligand superfamily member 8	P32971	TNFSF8	NA	Inverse variance weighted	2	-0.079	0.130	5.415E-01	0.986	pan	Hospitalized COVID-19
2278_61	TIMP-2	Metalloproteinase inhibitor 2	P16035	TIMP2	NA	Wald ratio	1	-0.089	0.147	5.433E-01	0.986	pan	Hospitalized COVID-19
11293_14	LRRN1:CD	Leucine-rich repeat neuronal protein 1:Cytoplasmic domain	Q6UXK5	LRRN1	NA	Inverse variance weighted	7	-0.056	0.092	5.439E-01	0.986	pan	Hospitalized COVID-19
2948_58	Growth hormone receptor	Growth hormone receptor	P10912	GHR	NA	Inverse variance weighted	4	0.061	0.101	5.442E-01	0.986	pan	Hospitalized COVID-19
15462_28	CD8A	T-cell surface glycoprotein CD8 alpha chain	P01732	CD8A	NA	Inverse variance weighted	8	-0.028	0.047	5.446E-01	0.986	pan	Hospitalized COVID-19
15526_33	GSHB	Glutathione synthetase	P48637	GSS	NA	Inverse variance weighted	4	-0.056	0.092	5.447E-01	0.986	pan	Hospitalized COVID-19
11161_5	SPG20	Spartin	Q8N0X7	SPART	NA	Wald ratio	1	0.132	0.219	5.467E-01	0.986	pan	Hospitalized COVID-19
2828_82	HAI-1	Kunitz-type protease inhibitor 1	O43278	SPINT1	NA	Inverse variance weighted	10	-0.018	0.029	5.467E-01	0.986	pan	Hospitalized COVID-19
8326_63	SIGIRR	Single Ig IL-1-related receptor	Q6IA17	SIGIRR	NA	Inverse variance weighted	15	0.028	0.047	5.472E-01	0.986	pan	Hospitalized COVID-19
2944_66	DAN	Neuroblastoma suppressor of tumorigenicity 1	P41271	NBL1	NA	Wald ratio	1	0.128	0.212	5.482E-01	0.986	pan	Hospitalized COVID-19
5713_9	IFN-lambda 3	Interferon lambda-3	Q8IZI9	IFNL3	NA	Inverse variance weighted	2	0.105	0.176	5.484E-01	0.986	pan	Hospitalized COVID-19
15480_2	VNN2	Vascular non-inflammatory molecule 2	O95498	VNN2	NA	Inverse variance weighted	16	0.010	0.017	5.494E-01	0.986	pan	Hospitalized COVID-19
6388_21	CC126	Coiled-coil domain-containing protein 126	Q96EE4	CCDC126	NA	Inverse variance weighted	6	-0.025	0.042	5.495E-01	0.986	pan	Hospitalized COVID-19
13692_154	WISP-1	WNT1-inducible-signaling pathway protein 1	O95388	CCN4	NA	Inverse variance weighted	12	-0.012	0.021	5.502E-01	0.986	pan	Hospitalized COVID-19
5646_20	RNAS6	Ribonuclease K6	Q93091	RNASE6	NA	Inverse variance weighted	6	0.013	0.021	5.506E-01	0.986	pan	Hospitalized COVID-19
9580_5	Laminin gamma-2	Laminin subunit gamma-2	Q13753	LAMC2	NA	Inverse variance weighted	8	-0.019	0.032	5.506E-01	0.986	pan	Hospitalized COVID-19
13944_3	SULT 1A3	Sulfotransferase 1A3	P0DMM9	SULT1A3	NA	Inverse variance weighted	7	0.053	0.089	5.523E-01	0.986	pan	Hospitalized COVID-19
11219_95	FGFP3	Fibroblast growth factor-binding protein 3	Q8TAT2	FGFBP3	NA	Inverse variance weighted	3	0.025	0.041	5.535E-01	0.986	pan	Hospitalized COVID-19
5463_22	GAS1	Growth arrest-specific protein 1	P54826	GAS1	NA	Inverse variance weighted	2	0.054	0.091	5.549E-01	0.986	pan	Hospitalized COVID-19
5112_73	OX2G	OX-2 membrane glycoprotein	P41217	CD200	NA	Wald ratio	1	-0.076	0.129	5.554E-01	0.986	pan	Hospitalized COVID-19
12867_40	DYLT3	Dynein light chain Tctex-type 3	P51808	DYNLT3	NA	Wald ratio	1	-0.147	0.249	5.555E-01	0.986	pan	Hospitalized COVID-19
9719_145	MMP-16	Matrix metalloproteinase-16	P51512	MMP16	NA	Inverse variance weighted	2	0.095	0.162	5.557E-01	0.986	pan	Hospitalized COVID-19
14100_63	C1QC	Complement C1q subcomponent subunit C	P02747	C1QC	NA	Inverse variance weighted	15	0.015	0.026	5.560E-01	0.986	pan	Hospitalized COVID-19
12422_143	LX15B	Arachidonate 15-lipoxygenase B	O15296	ALOX15B	NA	Wald ratio	1	0.083	0.140	5.560E-01	0.986	pan	Hospitalized COVID-19
11377_19	ADH7	Alcohol dehydrogenase class 4 mu/sigma chain	P40394	ADH7	NA	Inverse variance weighted	3	-0.021	0.035	5.568E-01	0.986	pan	Hospitalized COVID-19
2843_13	SPINT2	Kunitz-type protease inhibitor 2	O43291	SPINT2	NA	Inverse variance weighted	15	0.014	0.024	5.576E-01	0.986	pan	Hospitalized COVID-19
11421_10	EHD4	EH domain-containing protein 4	Q9H223	EHD4	NA	Wald ratio	1	0.060	0.103	5.582E-01	0.986	pan	Hospitalized COVID-19
8309_12	HYAL1	Hyaluronidase-1	Q12794	HYAL1	NA	Wald ratio	1	0.113	0.193	5.584E-01	0.986	pan	Hospitalized COVID-19
5671_1	Chymotrypsin	Chymotrypsinogen B	P17538	CTRB1	NA	Inverse variance weighted	8	-0.032	0.054	5.589E-01	0.986	pan	Hospitalized COVID-19
6580_29	Pregnancy zone protein	Pregnancy zone protein	P20742	PZP	NA	Inverse variance weighted	5	-0.038	0.065	5.594E-01	0.986	pan	Hospitalized COVID-19
3808_76	FGFR-2	Fibroblast growth factor receptor 2	P21802	FGFR2	NA	Wald ratio	1	-0.092	0.158	5.594E-01	0.986	pan	Hospitalized COVID-19
14151_4	UCRP	Ubiquitin-like protein ISG15	P05161	ISG15	NA	Inverse variance weighted	4	-0.076	0.131	5.597E-01	0.986	pan	Hospitalized COVID-19
3305_6	DLL4	Delta-like protein 4	Q9NR61	DLL4	NA	Wald ratio	1	0.114	0.195	5.609E-01	0.986	pan	Hospitalized COVID-19
5036_50	TSG-6	Tumor necrosis factor-inducible gene 6 protein	P98066	TNFAIP6	NA	Inverse variance weighted	22	-0.012	0.021	5.616E-01	0.986	pan	Hospitalized COVID-19
3293_2	CD5L	CD5 antigen-like	O43866	CD5L	NA	Inverse variance weighted	8	-0.029	0.051	5.628E-01	0.986	pan	Hospitalized COVID-19
3710_49	Angiostatin	Angiostatin	P00747	PLG	NA	Inverse variance weighted	8	-0.035	0.060	5.636E-01	0.986	pan	Hospitalized COVID-19
17764_108	RHOC	Rho-related GTP-binding protein RhoC	P08134	RHOC	NA	Inverse variance weighted	2	0.051	0.088	5.645E-01	0.986	pan	Hospitalized COVID-19
6649_51	NET1	Netrin-1	O95631	NTN1	NA	Inverse variance weighted	7	0.020	0.035	5.646E-01	0.986	pan	Hospitalized COVID-19
19158_1	PAF	PCNA-associated factor	Q15004	PCLAF	NA	Inverse variance weighted	5	-0.073	0.127	5.648E-01	0.986	pan	Hospitalized COVID-19
9018_38	PCD10:ECD	Protocadherin-10:Extracellular domain	Q9P2E7	PCDH10	NA	Inverse variance weighted	3	0.033	0.057	5.648E-01	0.986	pan	Hospitalized COVID-19
17370_186	ABHDA	Mycophenolic acid acyl-glucuronide esterase, mitochondrial	Q9NUJ1	ABHD10	NA	Wald ratio	1	-0.114	0.198	5.649E-01	0.986	pan	Hospitalized COVID-19
17750_8	RIR2	Ribonucleoside-diphosphate reductase subunit M2	P31350	RRM2	NA	Inverse variance weighted	2	0.067	0.116	5.651E-01	0.986	pan	Hospitalized COVID-19
7997_118	DOC2B	Double C2-like domain-containing protein beta	Q14184	DOC2B	NA	Wald ratio	1	0.139	0.241	5.655E-01	0.986	pan	Hospitalized COVID-19
4294_16	Sphingosine kinase 1	Sphingosine kinase 1	Q9NYA1	SPHK1	NA	Inverse variance weighted	3	-0.040	0.070	5.657E-01	0.986	pan	Hospitalized COVID-19
12524_18	SAT2	Diamine acetyltransferase 2	Q96F10	SAT2	NA	Inverse variance weighted	3	-0.038	0.067	5.661E-01	0.986	pan	Hospitalized COVID-19
12620_3	Septin-11	Septin-11	Q9NVA2	SEPTIN11	NA	Inverse variance weighted	2	-0.083	0.146	5.664E-01	0.986	pan	Hospitalized COVID-19
14076_74	Cystatin-S	Cystatin-S	P01036	CST4	NA	Inverse variance weighted	10	0.025	0.044	5.665E-01	0.986	pan	Hospitalized COVID-19
7154_92	CF058	Protein LEG1 homolog	Q6P5S2	LEG1	NA	Inverse variance weighted	5	0.050	0.088	5.681E-01	0.986	pan	Hospitalized COVID-19
19136_22	MMSA	Methylmalonate-semialdehyde dehydrogenase [acylating], mitochondrial	Q02252	ALDH6A1	NA	Inverse variance weighted	2	0.074	0.130	5.683E-01	0.986	pan	Hospitalized COVID-19
3365_7	Dkk-4	Dickkopf-related protein 4	Q9UBT3	DKK4	NA	Inverse variance weighted	4	0.065	0.114	5.687E-01	0.986	pan	Hospitalized COVID-19
18398_1	AK1D1	3-oxo-5-beta-steroid 4-dehydrogenase	P51857	AKR1D1	NA	Wald ratio	1	-0.120	0.211	5.690E-01	0.986	pan	Hospitalized COVID-19
14112_40	RELT	Tumor necrosis factor receptor superfamily member 19L	Q969Z4	RELT	NA	Wald ratio	1	0.116	0.204	5.690E-01	0.986	pan	Hospitalized COVID-19
13098_93	VEGF-D	Vascular endothelial growth factor D	O43915	VEGFD	NA	Wald ratio	1	0.113	0.199	5.704E-01	0.986	pan	Hospitalized COVID-19
7050_5	NEGR1	Neuronal growth regulator 1	Q7Z3B1	NEGR1	NA	Wald ratio	1	0.130	0.230	5.709E-01	0.986	pan	Hospitalized COVID-19
3325_2	MATN2	Matrilin-2	O00339	MATN2	NA	Inverse variance weighted	4	-0.052	0.093	5.711E-01	0.986	pan	Hospitalized COVID-19
14175_78	SCP2D	SCP2 sterol-binding domain-containing protein 1	Q9UJQ7	SCP2D1	NA	Inverse variance weighted	7	-0.020	0.035	5.711E-01	0.986	pan	Hospitalized COVID-19
10948_14	PLD3	Phospholipase D3	Q8IV08	PLD3	NA	Wald ratio	1	-0.106	0.187	5.717E-01	0.986	pan	Hospitalized COVID-19
13544_9	HMHA1	Rho GTPase-activating protein 45	Q92619	ARHGAP45	NA	Wald ratio	1	-0.051	0.091	5.718E-01	0.986	pan	Hospitalized COVID-19
12408_333	RB22A	Ras-related protein Rab-22A	Q9UL26	RAB22A	NA	Inverse variance weighted	2	0.080	0.142	5.728E-01	0.986	pan	Hospitalized COVID-19
10419_1	SCAR5	Scavenger receptor class A member 5	Q6ZMJ2	SCARA5	NA	Inverse variance weighted	10	0.033	0.058	5.730E-01	0.986	pan	Hospitalized COVID-19
15509_2	NAG	Alpha-N-acetylglucosaminidase	P54802	NAGLU	NA	Inverse variance weighted	28	0.010	0.018	5.743E-01	0.986	pan	Hospitalized COVID-19
10451_11	NUCB1	Nucleobindin-1	Q02818	NUCB1	NA	Wald ratio	1	0.058	0.102	5.743E-01	0.986	pan	Hospitalized COVID-19
6557_50	LRC15	Leucine-rich repeat-containing protein 15	Q8TF66	LRRC15	NA	Inverse variance weighted	4	-0.044	0.078	5.764E-01	0.986	pan	Hospitalized COVID-19
9793_145	IGDC4	Immunoglobulin superfamily DCC subclass member 4	Q8TDY8	IGDCC4	NA	Inverse variance weighted	9	0.016	0.029	5.764E-01	0.986	pan	Hospitalized COVID-19
6947_4	SIA10	Type 2 lactosamine alpha-2,3-sialyltransferase	Q9Y274	ST3GAL6	NA	Inverse variance weighted	31	0.009	0.016	5.768E-01	0.986	pan	Hospitalized COVID-19
5727_35	B3GLT	Beta-1,3-glucosyltransferase	Q6Y288	B3GLCT	NA	Inverse variance weighted	2	0.029	0.053	5.768E-01	0.986	pan	Hospitalized COVID-19
17819_30	FAHD1	Acylpyruvase FAHD1, mitochondrial	Q6P587	FAHD1	NA	Inverse variance weighted	4	-0.049	0.088	5.769E-01	0.986	pan	Hospitalized COVID-19
13123_3	FLRT3:ECD	Leucine-rich repeat transmembrane protein FLRT3:Extracellular domain	Q9NZU0	FLRT3	NA	Inverse variance weighted	9	-0.014	0.025	5.776E-01	0.986	pan	Hospitalized COVID-19
6544_33	NELL1	Protein kinase C-binding protein NELL1	Q92832	NELL1	NA	Inverse variance weighted	21	-0.018	0.033	5.785E-01	0.986	pan	Hospitalized COVID-19

17342_13	AGR3	Anterior gradient protein 3	Q8TD06	AGR3	NA	Wald ratio	1	0.125	0.226	5.793E-01	0.986	pan	Hospitalized COVID-19
18871_24	AIF1L	Allograft inflammatory factor 1-like	Q9BQI0	AIF1L	NA	Inverse variance weighted	7	-0.016	0.028	5.802E-01	0.986	pan	Hospitalized COVID-19
9244_27	PPT1	Palmitoyl-protein thioesterase 1	P50897	PPT1	NA	Inverse variance weighted	2	0.034	0.062	5.809E-01	0.986	pan	Hospitalized COVID-19
6107_3	ELA1	Chymotrypsin-like elastase family member 1	Q9UNI1	CELA1	NA	Inverse variance weighted	13	0.019	0.035	5.813E-01	0.986	pan	Hospitalized COVID-19
18819_21	PPIC	Peptidyl-prolyl cis-trans isomerase C	P45877	PPIC	NA	Inverse variance weighted	10	-0.015	0.026	5.815E-01	0.986	pan	Hospitalized COVID-19
8909_77	GNPI1	Glucosamine-6-phosphate isomerase 1	P46926	GNPDA1	NA	Wald ratio	1	0.045	0.081	5.816E-01	0.986	pan	Hospitalized COVID-19
4961_17	annexin II	Annexin A2	P07355	ANXA2	NA	Inverse variance weighted	3	0.066	0.120	5.816E-01	0.986	pan	Hospitalized COVID-19
15444_45	SCCA2	Serpin B4	P48594	SERPINB4	NA	Inverse variance weighted	5	0.047	0.085	5.818E-01	0.986	pan	Hospitalized COVID-19
6909_40	MGAT2	Alpha-1,6-mannosyl-glycoprotein 2-beta-N-acetylglucosaminyltransferase	Q10469	MGAT2	NA	Inverse variance weighted	2	-0.065	0.119	5.822E-01	0.986	pan	Hospitalized COVID-19
18880_81	Collagen Type III	Collagen Type III	P02461	COL3A1	NA	Inverse variance weighted	9	-0.036	0.066	5.823E-01	0.986	pan	Hospitalized COVID-19
9823_2	DYR	Dihydrofolate reductase	P00374	DHFR	NA	Inverse variance weighted	5	-0.022	0.040	5.826E-01	0.986	pan	Hospitalized COVID-19
6496_60	DLK1:ECD	Protein delta homolog 1:Extracellular domain	P80370	DLK1	NA	Inverse variance weighted	2	0.148	0.269	5.827E-01	0.986	pan	Hospitalized COVID-19
3235_50	WFKN2	WAP, Kazal, immunoglobulin, Kunitz and NTR domain-containing protein 2	Q8TEU8	WFIKKN2	NA	Inverse variance weighted	8	-0.015	0.027	5.828E-01	0.986	pan	Hospitalized COVID-19
8890_9	T132B	Transmembrane protein 132B	Q14DG7	TMEM132B	NA	Inverse variance weighted	3	-0.020	0.036	5.829E-01	0.986	pan	Hospitalized COVID-19
3044_3	PARC	C-C motif chemokine 18	P55774	CCL18	NA	Inverse variance weighted	9	-0.031	0.056	5.834E-01	0.986	pan	Hospitalized COVID-19
5615_62	F172A	Protein FAM172A	Q8WUF8	FAM172A	NA	Wald ratio	1	-0.079	0.144	5.836E-01	0.986	pan	Hospitalized COVID-19
8369_102	DAG1	Dystroglycan	Q14118	DAG1	NA	Wald ratio	1	-0.096	0.176	5.841E-01	0.986	pan	Hospitalized COVID-19
5475_10	PKC-B-II	Protein kinase C beta type (splice variant beta-II)	P05771	PRKCB	NA	Wald ratio	1	-0.074	0.135	5.846E-01	0.986	pan	Hospitalized COVID-19
11480_1	Aldehyde dehydrogenase, class 3	Aldehyde dehydrogenase, dimeric NADP-preferring	P30838	ALDH3A1	NA	Wald ratio	1	-0.047	0.085	5.852E-01	0.986	pan	Hospitalized COVID-19
4922_13	MIP-3b	C-C motif chemokine 19	Q99731	CCL19	NA	Inverse variance weighted	2	0.144	0.265	5.852E-01	0.986	pan	Hospitalized COVID-19
4158_54	uPA	Urokinase-type plasminogen activator	P00749	PLAU	NA	Inverse variance weighted	11	0.026	0.049	5.861E-01	0.986	pan	Hospitalized COVID-19
3719_2	p27Kip1	Cyclin-dependent kinase inhibitor 1B	P46527	CDKN1B	NA	Inverse variance weighted	2	-0.060	0.110	5.862E-01	0.986	pan	Hospitalized COVID-19
12731_12	PKHA7	Pleckstrin homology domain-containing family A member 7	Q6IQ23	PLEKHA7	NA	Inverse variance weighted	8	0.014	0.026	5.863E-01	0.986	pan	Hospitalized COVID-19
15368_3	BMPER	BMP-binding endothelial regulator protein	Q8N8U9	BMPER	NA	Inverse variance weighted	3	-0.037	0.068	5.869E-01	0.986	pan	Hospitalized COVID-19
18222_34	SHLB2	Endophilin-B2	Q9NR46	SH3GLB2	NA	Inverse variance weighted	3	0.017	0.031	5.870E-01	0.986	pan	Hospitalized COVID-19
3890_8	LDH-H 1	L-lactate dehydrogenase B chain	P07195	LDHB	NA	Wald ratio	1	0.153	0.282	5.874E-01	0.986	pan	Hospitalized COVID-19
11214_40	DNJB9	DnaJ homolog subfamily B member 9	Q9UBS3	DNAJB9	NA	Wald ratio	1	0.119	0.219	5.878E-01	0.986	pan	Hospitalized COVID-19
5644_60	RNAS4	Ribonuclease 4	P34096	RNASE4	NA	Inverse variance weighted	5	-0.024	0.045	5.879E-01	0.986	pan	Hospitalized COVID-19
12546_1	SMTN	Smoothelin	P53814	SMTN	NA	Wald ratio	1	-0.120	0.221	5.881E-01	0.986	pan	Hospitalized COVID-19
10075_75	ACBD6	Acyl-CoA-binding domain-containing protein 6	Q9BR61	ACBD6	NA	Wald ratio	1	0.109	0.201	5.884E-01	0.986	pan	Hospitalized COVID-19
8065_245	PATE4	Prostate and testis expressed protein 4	P0C8F1	PATE4	NA	Wald ratio	1	-0.044	0.082	5.887E-01	0.986	pan	Hospitalized COVID-19
2212_69	tPA	Tissue-type plasminogen activator	P00750	PLAT	NA	Inverse variance weighted	9	0.040	0.075	5.889E-01	0.986	pan	Hospitalized COVID-19
15516_12	SAA-4	Serum amyloid A-4 protein	P35542	SAA4	NA	Inverse variance weighted	7	0.021	0.039	5.897E-01	0.986	pan	Hospitalized COVID-19
6123_69	p53	Cellular tumor antigen p53	P04637	TP53	NA	Inverse variance weighted	2	0.112	0.208	5.899E-01	0.986	pan	Hospitalized COVID-19
18422_41	ERG19	Diphosphomevalonate decarboxylase	P53602	MVD	NA	Wald ratio	1	0.085	0.157	5.904E-01	0.986	pan	Hospitalized COVID-19
10366_11	PDGFRA	Platelet-derived growth factor receptor alpha	P16234	PDGFRA	NA	Inverse variance weighted	10	-0.025	0.046	5.913E-01	0.986	pan	Hospitalized COVID-19
9880_33	T23O	Tryptophan 2,3-dioxygenase	P48775	TDO2	NA	Inverse variance weighted	7	-0.053	0.098	5.914E-01	0.986	pan	Hospitalized COVID-19
4246_40	NCAM-L1	Neural cell adhesion molecule L1	P32004	L1CAM	NA	Inverse variance weighted	5	-0.048	0.090	5.919E-01	0.986	pan	Hospitalized COVID-19
12800_5	TPPP2	Tubulin polymerization-promoting protein family member 2	P59282	TPPP2	NA	Inverse variance weighted	4	0.076	0.143	5.924E-01	0.986	pan	Hospitalized COVID-19
16610_13	LRP10	Low-density lipoprotein receptor-related protein 10	Q7Z4F1	LRP10	NA	Inverse variance weighted	3	0.061	0.115	5.943E-01	0.986	pan	Hospitalized COVID-19
5701_81	Tetranectin	Tetranectin	P05452	CLEC3B	NA	Inverse variance weighted	3	0.052	0.098	5.944E-01	0.986	pan	Hospitalized COVID-19
16296_43	LGR5	Leucine-rich repeat-containing G-protein coupled receptor 5	O75473	LGR5	NA	Wald ratio	1	-0.097	0.183	5.945E-01	0.986	pan	Hospitalized COVID-19
9826_135	Fragile histidine triad protein	Bis(5'-adenosyl)-triphosphatase	P49789	FHIT	NA	Inverse variance weighted	3	0.049	0.092	5.949E-01	0.986	pan	Hospitalized COVID-19
14133_93	IL-1 sRII	Interleukin-1 receptor type 2	P27930	IL1R2	NA	Inverse variance weighted	12	-0.023	0.044	5.963E-01	0.986	pan	Hospitalized COVID-19
18434_141	TBPL1	TATA box-binding protein-like protein 1	P62380	TBPL1	NA	Inverse variance weighted	3	-0.078	0.148	5.965E-01	0.986	pan	Hospitalized COVID-19
9343_16	IL-2 sRb	Interleukin-2 receptor subunit beta	P14784	IL2RB	NA	Inverse variance weighted	5	-0.054	0.103	5.980E-01	0.986	pan	Hospitalized COVID-19
6713_4	LRP11	Low-density lipoprotein receptor-related protein 11	Q86VZ4	LRP11	NA	Inverse variance weighted	12	0.014	0.027	5.982E-01	0.986	pan	Hospitalized COVID-19
15562_24	BGLR	Beta-glucuronidase	P08236	GUSB	NA	Inverse variance weighted	13	-0.018	0.034	5.986E-01	0.986	pan	Hospitalized COVID-19
15686_49	INHBC	Inhibin beta C chain	P55103	INHBC	NA	Inverse variance weighted	17	-0.011	0.022	5.988E-01	0.986	pan	Hospitalized COVID-19
19437_61	L-VEGF165	Isoform L-VEGF165	P15692	VEGFA	NA	Inverse variance weighted	6	-0.041	0.078	5.989E-01	0.986	pan	Hospitalized COVID-19
18839_24	Thyroglobulin	Thyroglobulin	P01266	TG	NA	Inverse variance weighted	6	-0.038	0.073	6.000E-01	0.986	pan	Hospitalized COVID-19
5353_89	IL-1Ra	Interleukin-1 receptor antagonist protein	P18510	IL1RN	NA	Inverse variance weighted	4	0.057	0.108	6.003E-01	0.986	pan	Hospitalized COVID-19
19341_36	ACBD6	Acyl-CoA-binding domain-containing protein 6	Q9BR61	ACBD6	NA	Inverse variance weighted	5	0.043	0.082	6.004E-01	0.986	pan	Hospitalized COVID-19
3213_65	Nidogen	Nidogen-1	P14543	NID1	NA	Inverse variance weighted	2	0.085	0.163	6.019E-01	0.986	pan	Hospitalized COVID-19
8687_26	T106B	Transmembrane protein 106B	Q9NUM4	TMEM106B	NA	Inverse variance weighted	3	-0.038	0.072	6.019E-01	0.986	pan	Hospitalized COVID-19
15299_102	MESD2	LDLR chaperone MESD	Q14696	MESD	NA	Wald ratio	1	0.117	0.225	6.039E-01	0.986	pan	Hospitalized COVID-19
18343_10	DECR2	Peroxisomal 2,4-dienoyl-CoA reductase	Q9NUI1	DECR2	NA	Inverse variance weighted	8	0.025	0.048	6.040E-01	0.986	pan	Hospitalized COVID-19
11130_158	CACB4	Voltage-dependent L-type calcium channel subunit beta-4	O00305	CACNB4	NA	Wald ratio	1	0.046	0.089	6.041E-01	0.986	pan	Hospitalized COVID-19
11140_56	CO1A1:C-term propeptide	Collagen alpha-1(I) chain:C-term propeptide	P02452	COL1A1	NA	Inverse variance weighted	8	-0.040	0.076	6.042E-01	0.986	pan	Hospitalized COVID-19
3221_54	SARP-2	Secreted frizzled-related protein 1	Q8N474	SFRP1	NA	Inverse variance weighted	6	0.024	0.046	6.047E-01	0.986	pan	Hospitalized COVID-19
8804_39	COKA1	Collagen alpha-1(XX) chain	Q9P218	COL20A1	NA	Wald ratio	1	0.077	0.149	6.056E-01	0.986	pan	Hospitalized COVID-19
6234_74	VITRN	Vitrin	Q6UXI7	VIT	NA	Inverse variance weighted	9	-0.012	0.023	6.068E-01	0.986	pan	Hospitalized COVID-19
11264_33	XDH	Xanthine dehydrogenase/oxidase	P47989	XDH	NA	Wald ratio	1	0.114	0.222	6.069E-01	0.986	pan	Hospitalized COVID-19
16872_248	MAAI	Maleylacetoacetate isomerase	O43708	GSTZ1	NA	Inverse variance weighted	12	0.007	0.014	6.070E-01	0.986	pan	Hospitalized COVID-19
13930_3	ABC3G	DNA dC->dU-editing enzyme APOBEC-3G	Q9HC16	APOBEC3G	NA	Inverse variance weighted	2	0.040	0.077	6.071E-01	0.986	pan	Hospitalized COVID-19
8866_53	QPCTL	Glutaminyl-peptide cyclotransferase-like protein	Q9NXS2	QPCTL	NA	Inverse variance weighted	6	0.018	0.035	6.074E-01	0.986	pan	Hospitalized COVID-19
10606_34	TOIP1:Perinuclear	Torsin-1A-interacting protein 1:Perinuclear domain	Q5JTV8	TOR1AIP1	NA	Wald ratio	1	0.123	0.240	6.078E-01	0.986	pan	Hospitalized COVID-19
15525_294	ADH1G	Alcohol dehydrogenase 1C	P00326	ADH1C	NA	Wald ratio	1	0.041	0.079	6.083E-01	0.986	pan	Hospitalized COVID-19
7115_5	Semenogelin	Semenogelin-1	P04279	SEMG1	NA	Inverse variance weighted	16	-0.020	0.039	6.091E-01	0.986	pan	Hospitalized COVID-19
14006_36	GNMT	Glycine N-methyltransferase	Q14749	GNMT	NA	Inverse variance weighted	3	0.042	0.082	6.095E-01	0.986	pan	Hospitalized COVID-19
11319_106	MRE11	Double-strand break repair protein MRE11	P49959	MRE11	NA	Wald ratio	1	-0.101	0.198	6.106E-01	0.986	pan	Hospitalized COVID-19
5682_13	VASN	Vasorin	Q6EMK4	VASN	NA	Inverse variance weighted	2	-0.080	0.158	6.123E-01	0.986	pan	Hospitalized COVID-19
12431_13	PELO	Protein pelota homolog	Q9BRX2	PELO	NA	Wald ratio	1	-0.111	0.219	6.124E-01	0.986	pan	Hospitalized COVID-19
16609_106	KIRR2	Kin of IRRE-like protein 2	Q6UWL6	KIRREL2	NA	Inverse variance weighted	9	0.014	0.027	6.124E-01	0.986	pan	Hospitalized COVID-19
4496_60	MMP-12	Macrophage metalloelastase	P39900	MMP12	NA	Inverse variance weighted	13	-0.010	0.019	6.125E-01	0.986	pan	Hospitalized COVID-19
10737_96	Serpin B1	Leukocyte elastase inhibitor	P30740	SERPINB1	NA	Wald ratio	1	0.092	0.182	6.130E-01	0.986	pan	Hospitalized COVID-19
8814_33	SAPL1	Proactivator polypeptide-like 1	Q6NUJ1	PSAPL1	NA	Inverse variance weighted	10	-0.014	0.027	6.131E-01	0.986	pan	Hospitalized COVID-19
3583_54	Arylsulfatase A	Arylsulfatase A	P15289	ARSA	NA	Wald ratio	1	0.063	0.125	6.132E-01	0.986	pan	Hospitalized COVID-19

19523_215	PARK7	Protein DJ-1	Q99497	PARK7	NA	Wald ratio	1	-0.056	0.110	6.141E-01	0.986	pan	Hospitalized COVID-19
15615_8	LIRB3	Leukocyte immunoglobulin-like receptor subfamily B member 3	O75022	LILRB3	NA	Inverse variance weighted	5	0.020	0.039	6.145E-01	0.986	pan	Hospitalized COVID-19
15446_25	SMDF	Neuregulin-1, sensory and motor neuron-derived factor isoform	Q02297	NRG1	NA	Inverse variance weighted	2	-0.046	0.091	6.147E-01	0.986	pan	Hospitalized COVID-19
10815_2	HABP4	Intracellular hyaluronan-binding protein 4	Q5JVS0	HABP4	NA	Inverse variance weighted	5	-0.030	0.059	6.155E-01	0.986	pan	Hospitalized COVID-19
17680_12	EPHB1	Ephrin type-B receptor 1	P54762	EPHB1	NA	Inverse variance weighted	3	0.027	0.055	6.156E-01	0.986	pan	Hospitalized COVID-19
3461_58	PGCB	Brevican core protein	Q96GW7	BCAN	NA	Inverse variance weighted	3	-0.042	0.083	6.158E-01	0.986	pan	Hospitalized COVID-19
17400_71	ACOT8	Acyl-coenzyme A thioesterase 8	O14734	ACOT8	NA	Wald ratio	1	-0.141	0.281	6.165E-01	0.986	pan	Hospitalized COVID-19
9201_13	Transgelin-2	Transgelin-2	P37802	TAGLN2	NA	Wald ratio	1	0.113	0.227	6.169E-01	0.986	pan	Hospitalized COVID-19
18319_7	ODPX	Pyruvate dehydrogenase protein X component, mitochondrial	O00330	PDHX	NA	Wald ratio	1	0.069	0.137	6.170E-01	0.986	pan	Hospitalized COVID-19
8007_19	Cathepsin B	Cathepsin B	P07858	CTSB	NA	Inverse variance weighted	11	0.018	0.036	6.175E-01	0.986	pan	Hospitalized COVID-19
9829_91	SULT 2A1	Bile salt sulfotransferase	Q06520	SULT2A1	NA	Wald ratio	1	-0.058	0.116	6.176E-01	0.986	pan	Hospitalized COVID-19
18315_38	RTP4	Receptor-transporting protein 4	Q96DX8	RTP4	NA	Inverse variance weighted	4	0.044	0.089	6.177E-01	0.986	pan	Hospitalized COVID-19
17773_26	SNAG	Gamma-soluble NSF attachment protein	Q99747	NAPG	NA	Wald ratio	1	0.073	0.146	6.183E-01	0.986	pan	Hospitalized COVID-19
3290_50	CD109	CD109 antigen	Q6YHK3	CD109	NA	Inverse variance weighted	17	0.010	0.019	6.183E-01	0.986	pan	Hospitalized COVID-19
2900_53	HCC-1	C-C motif chemokine 14	Q16627	CCL14	NA	Inverse variance weighted	7	-0.024	0.049	6.186E-01	0.986	pan	Hospitalized COVID-19
9916_146	LRC4B:ECD	Leucine-rich repeat-containing protein 4B:Extracellular domain	Q9NT99	LRRC4B	NA	Inverse variance weighted	2	-0.101	0.203	6.191E-01	0.986	pan	Hospitalized COVID-19
8954_30	M4K1	Mitogen-activated protein kinase kinase kinase kinase 1	Q92918	MAP4K1	NA	Inverse variance weighted	6	-0.018	0.036	6.197E-01	0.986	pan	Hospitalized COVID-19
4763_31	Afamin	Afamin	P43652	AFM	NA	Inverse variance weighted	8	0.019	0.038	6.198E-01	0.986	pan	Hospitalized COVID-19
7096_30	RMD1	Regulator of microtubule dynamics protein 1	Q96DB5	RMDN1	NA	Inverse variance weighted	5	0.022	0.044	6.202E-01	0.986	pan	Hospitalized COVID-19
5660_51	SOD3	Extracellular superoxide dismutase [Cu-Zn]	P08294	SOD3	NA	Inverse variance weighted	15	0.012	0.024	6.206E-01	0.986	pan	Hospitalized COVID-19
3810_50	FGR	Tyrosine-protein kinase Fgr	P09769	FGR	NA	Inverse variance weighted	2	0.074	0.150	6.212E-01	0.986	pan	Hospitalized COVID-19
9100_32	CE046	Uncharacterized protein C5orf46	Q6UWT4	C5orf46	NA	Wald ratio	1	-0.070	0.143	6.213E-01	0.986	pan	Hospitalized COVID-19
3508_78	MDC	C-C motif chemokine 22	O00626	CCL22	NA	Inverse variance weighted	9	-0.025	0.051	6.218E-01	0.986	pan	Hospitalized COVID-19
5440_26	Troponin I, skeletal, fast twitch	Troponin I, fast skeletal muscle	P48788	TNNI2	NA	Wald ratio	1	-0.104	0.212	6.222E-01	0.986	pan	Hospitalized COVID-19
5231_79	PCSK9	Proprotein convertase subtilisin/kexin type 9	Q8NBP7	PCSK9	NA	Inverse variance weighted	5	0.022	0.045	6.223E-01	0.986	pan	Hospitalized COVID-19
8402_22	CYTL1	Cytokine-like protein 1	Q9NRR1	CYTL1	NA	Inverse variance weighted	3	0.033	0.067	6.227E-01	0.986	pan	Hospitalized COVID-19
15584_9	FHR2	Complement factor H-related protein 2	P36980	CFHR2	NA	Inverse variance weighted	16	-0.016	0.033	6.241E-01	0.986	pan	Hospitalized COVID-19
5061_27	B7-H2	ICOS ligand	O75144	ICOSLG	NA	Inverse variance weighted	3	-0.056	0.114	6.242E-01	0.986	pan	Hospitalized COVID-19
13534_20	MYOM2	Myomesin-2	P54296	MYOM2	NA	Inverse variance weighted	3	-0.066	0.135	6.246E-01	0.986	pan	Hospitalized COVID-19
5139_32	UNC5H3	Netrin receptor UNC5C	O95185	UNC5C	NA	Inverse variance weighted	3	0.063	0.128	6.249E-01	0.986	pan	Hospitalized COVID-19
11531_24	GI24:CD	V-type immunoglobulin domain-containing suppressor of T-cell activation:Cytoplasmic domain	Q9H7M9	VSIR	NA	Wald ratio	1	0.081	0.166	6.259E-01	0.986	pan	Hospitalized COVID-19
13621_31	AP2A2	AP-2 complex subunit alpha-2	O94973	AP2A2	NA	Wald ratio	1	0.079	0.163	6.269E-01	0.986	pan	Hospitalized COVID-19
3593_72	Caspase-3	Caspase-3	P42574	CASP3	NA	Wald ratio	1	0.042	0.087	6.279E-01	0.986	pan	Hospitalized COVID-19
8962_48	KS6B1	Ribosomal protein S6 kinase beta-1	P23443	RPS6KB1	NA	Inverse variance weighted	4	-0.054	0.112	6.280E-01	0.986	pan	Hospitalized COVID-19
7932_23	SYT8	Synaptotagmin-8	Q8NBV8	SYT8	NA	Wald ratio	1	0.112	0.232	6.280E-01	0.986	pan	Hospitalized COVID-19
9526_3	COLL1	Colipase-like protein 1	A2RUU4	CLPSL1	NA	Inverse variance weighted	6	0.034	0.071	6.285E-01	0.986	pan	Hospitalized COVID-19
18241_18	HEM6	Oxygen-dependent coproporphyrinogen-III oxidase, mitochondrial	P36551	CPOX	NA	Inverse variance weighted	10	0.023	0.048	6.287E-01	0.986	pan	Hospitalized COVID-19
2602_2	Angiopoietin-2	Angiopoietin-2	O15123	ANGPT2	NA	Inverse variance weighted	3	0.033	0.069	6.296E-01	0.986	pan	Hospitalized COVID-19
9216_100	PLXB2	Plexin-B2	O15031	PLXNB2	NA	Inverse variance weighted	9	-0.015	0.032	6.298E-01	0.986	pan	Hospitalized COVID-19
6603_18	KALM	Anosmin-1	P23352	ANOS1	NA	Inverse variance weighted	3	0.066	0.137	6.298E-01	0.986	pan	Hospitalized COVID-19
8841_65	CILP2	Cartilage intermediate layer protein 2	Q8IUL8	CILP2	NA	Inverse variance weighted	2	0.096	0.200	6.304E-01	0.986	pan	Hospitalized COVID-19
13374_4	DB113	Beta-defensin 113	Q30KQ7	DEFB113	NA	Wald ratio	1	-0.106	0.221	6.308E-01	0.986	pan	Hospitalized COVID-19
10903_50	STX8	Syntaxin-8	Q9UNK0	STX8	NA	Wald ratio	1	0.078	0.163	6.320E-01	0.986	pan	Hospitalized COVID-19
9231_23	IMPA3	Inositol monophosphatase 3	Q9NX62	BPNT2	NA	Inverse variance weighted	12	0.012	0.025	6.323E-01	0.986	pan	Hospitalized COVID-19
10603_1	HIS3	Histatin-3	P15516	HTN3	NA	Inverse variance weighted	12	0.022	0.047	6.323E-01	0.986	pan	Hospitalized COVID-19
14146_92	H31	Histone H3.1	P68431	H3C1	NA	Wald ratio	1	0.081	0.170	6.327E-01	0.986	pan	Hospitalized COVID-19
13481_24	TCEA2	Transcription elongation factor A protein 2	Q15560	TCEA2	NA	Wald ratio	1	-0.054	0.112	6.332E-01	0.986	pan	Hospitalized COVID-19
9522_3	AIF	Apoptosis-inducing factor 1, mitochondrial	O95831	AIFM1	NA	Wald ratio	1	0.086	0.179	6.333E-01	0.986	pan	Hospitalized COVID-19
4498_62	NCAM-120	Neural cell adhesion molecule 1, 120 kDa isoform	P13591	NCAM1	NA	Inverse variance weighted	12	-0.016	0.034	6.344E-01	0.986	pan	Hospitalized COVID-19
2952_75	IGF-I	Insulin-like growth factor I	P05019	IGF1	NA	Inverse variance weighted	2	-0.084	0.176	6.345E-01	0.986	pan	Hospitalized COVID-19
10514_5	PGD2 synthase	Prostaglandin-H2 D-isomerase	P41222	PTGDS	NA	Inverse variance weighted	3	-0.063	0.133	6.349E-01	0.986	pan	Hospitalized COVID-19
11606_22	DNJB6	DnaJ homolog subfamily B member 6	O75190	DNAJB6	NA	Wald ratio	1	-0.105	0.222	6.358E-01	0.986	pan	Hospitalized COVID-19
2780_35	Lactoferrin	Lactotransferrin	P02788	LTF	NA	Inverse variance weighted	2	0.057	0.121	6.358E-01	0.986	pan	Hospitalized COVID-19
14048_7	IL-1 R AcP	Interleukin-1 Receptor accessory protein	Q9NPH3	IL1RAP	NA	Inverse variance weighted	6	-0.008	0.017	6.362E-01	0.986	pan	Hospitalized COVID-19
7959_34	CADH7	Cadherin-7	Q9ULB5	CDH7	NA	Inverse variance weighted	6	0.039	0.083	6.364E-01	0.986	pan	Hospitalized COVID-19
6366_38	TXD15	Thioredoxin domain-containing protein 15	Q96J42	TXNDC15	NA	Inverse variance weighted	12	0.012	0.026	6.369E-01	0.986	pan	Hospitalized COVID-19
5392_73	Fas, soluble	Tumor necrosis factor receptor superfamily member 6	P25445	FAS	NA	Inverse variance weighted	3	0.050	0.106	6.370E-01	0.986	pan	Hospitalized COVID-19
19556_12	Complement receptor type 1	Complement receptor type 1	P17927	CR1	NA	Inverse variance weighted	11	0.017	0.036	6.374E-01	0.986	pan	Hospitalized COVID-19
9005_16	PLXA1	Plexin-A1	Q9UIW2	PLXNA1	NA	Inverse variance weighted	4	0.019	0.041	6.385E-01	0.986	pan	Hospitalized COVID-19
6600_70	RELL2	RELT-like protein 2	Q8NC24	RELL2	NA	Wald ratio	1	-0.081	0.173	6.388E-01	0.986	pan	Hospitalized COVID-19
13936_24	PGK2	Phosphoglycerate kinase 2	P07205	PGK2	NA	Wald ratio	1	0.062	0.131	6.392E-01	0.986	pan	Hospitalized COVID-19
9869_28	NF-kappa-B p105	Nuclear factor NF-kappa-B p105 subunit	P19838	NFKB1	NA	Wald ratio	1	-0.127	0.272	6.393E-01	0.986	pan	Hospitalized COVID-19
2436_49	CXCL16, soluble	C-X-C motif chemokine 16	Q9H2A7	CXCL16	NA	Inverse variance weighted	11	0.019	0.042	6.403E-01	0.986	pan	Hospitalized COVID-19
5654_70	TMX3	Protein disulfide-isomerase TMX3	Q96JJ7	TMX3	NA	Inverse variance weighted	10	-0.021	0.045	6.409E-01	0.986	pan	Hospitalized COVID-19
8360_169	NKp46	Natural cytotoxicity triggering receptor 1	O76036	NCR1	NA	Wald ratio	1	0.144	0.309	6.410E-01	0.986	pan	Hospitalized COVID-19
2827_23	Fractalkine/CX3CL-1	Fractalkine	P78423	CX3CL1	NA	Wald ratio	1	-0.040	0.086	6.422E-01	0.986	pan	Hospitalized COVID-19
13983_27	QOR	Quinone oxidoreductase	Q08257	CRYZ	NA	Inverse variance weighted	15	0.009	0.019	6.423E-01	0.986	pan	Hospitalized COVID-19
12628_31	LANC2	LanC-like protein 2	Q9NS86	LANCL2	NA	Wald ratio	1	-0.106	0.229	6.439E-01	0.986	pan	Hospitalized COVID-19
6444_15	PSG3	Pregnancy-specific beta-1-glycoprotein 3	Q16557	PSG3	NA	Inverse variance weighted	6	0.025	0.055	6.440E-01	0.986	pan	Hospitalized COVID-19
6520_87	MGP	Matrix Gla protein	P08493	MGP	NA	Inverse variance weighted	2	-0.066	0.144	6.441E-01	0.986	pan	Hospitalized COVID-19
18895_54	GSTM4	Glutathione S-transferase Mu 4	Q03013	GSTM4	NA	Inverse variance weighted	11	0.028	0.060	6.446E-01	0.986	pan	Hospitalized COVID-19
8957_72	XTP3B	Endoplasmic reticulum lectin 1	Q96DZ1	ERLEC1	NA	Wald ratio	1	-0.070	0.152	6.448E-01	0.986	pan	Hospitalized COVID-19
13088_397	BTC	Betacellulin	P35070	BTC	NA	Inverse variance weighted	2	0.028	0.061	6.448E-01	0.986	pan	Hospitalized COVID-19
8429_16	AXIN2	Axin-2	Q9Y2T1	AXIN2	NA	Inverse variance weighted	4	-0.082	0.177	6.456E-01	0.986	pan	Hospitalized COVID-19
11440_58	SOCS-3	Suppressor of cytokine signaling 3	O14543	SOCS3	NA	Inverse variance weighted	9	0.013	0.028	6.469E-01	0.986	pan	Hospitalized COVID-19
2851_63	C5a	C5a anaphylatoxin	P01031	C5	NA	Inverse variance weighted	4	0.033	0.073	6.469E-01	0.986	pan	Hospitalized COVID-19
17724_3	WWOX	WW domain-containing oxidoreductase	Q9NZC7	WWOX	NA	Wald ratio	1	0.063	0.138	6.477E-01	0.986	pan	Hospitalized COVID-19
15384_15	KLOTHO	Klotho	Q9UEF7	KL	NA	Inverse variance weighted	14	-0.017	0.038	6.481E-01	0.986	pan	Hospitalized COVID-19

4990_87	GP1BA	Platelet glycoprotein Ib alpha chain	P07359	GP1BA	NA	Inverse variance weighted	9	-0.031	0.068	6.496E-01	0.986	pan	Hospitalized COVID-19
3378_49	Kallikrein 7	Kallikrein-7	P49862	KLK7	NA	Inverse variance weighted	2	-0.054	0.119	6.511E-01	0.986	pan	Hospitalized COVID-19
13536_56	POLI	DNA polymerase iota	Q9UNA4	POLI	NA	Wald ratio	1	-0.052	0.116	6.514E-01	0.986	pan	Hospitalized COVID-19
7179_69	NFASC	Neurofascin	O94856	NFASC	NA	Inverse variance weighted	4	-0.013	0.028	6.516E-01	0.986	pan	Hospitalized COVID-19
10070_22	PLCG2	1-phosphatidylinositol 4,5-bisphosphate phosphodiesterase gamma-2	P16885	PLCG2	NA	Inverse variance weighted	2	0.079	0.175	6.525E-01	0.986	pan	Hospitalized COVID-19
15385_116	FABP2	Fatty acid-binding protein, intestinal	P12104	FABP2	NA	Inverse variance weighted	10	0.015	0.033	6.539E-01	0.986	pan	Hospitalized COVID-19
15324_58	Ferritin light chain	Ferritin light chain	P02792	FTL	NA	Inverse variance weighted	4	0.047	0.106	6.546E-01	0.986	pan	Hospitalized COVID-19
19323_1	RCAS1	Receptor-binding cancer antigen expressed on SiSo cells	O00559	EBAG9	NA	Inverse variance weighted	3	-0.058	0.129	6.548E-01	0.986	pan	Hospitalized COVID-19
19238_12	GLNA	Glutamine synthetase	P15104	GLUL	NA	Inverse variance weighted	2	-0.073	0.164	6.548E-01	0.986	pan	Hospitalized COVID-19
4962_52	ARMEL	Cerebral dopamine neurotrophic factor	Q49AH0	CDNF	NA	Inverse variance weighted	5	0.024	0.055	6.564E-01	0.986	pan	Hospitalized COVID-19
18930_28	SLIT2	Slit homolog 2 protein	O94813	SLIT2	NA	Inverse variance weighted	2	0.039	0.088	6.568E-01	0.986	pan	Hospitalized COVID-19
7827_20	ADA32	Disintegrin and metalloproteinase domain-containing protein 32	Q8TC27	ADAM32	NA	Wald ratio	1	0.114	0.257	6.572E-01	0.986	pan	Hospitalized COVID-19
7945_10	Semaphorin-6A	Semaphorin-6A	Q9H2E6	SEMA6A	NA	Inverse variance weighted	5	-0.030	0.067	6.579E-01	0.986	pan	Hospitalized COVID-19
5630_48	CM35H	CMRF35-like molecule 8	Q9UGN4	CD300A	NA	Inverse variance weighted	13	0.010	0.022	6.581E-01	0.986	pan	Hospitalized COVID-19
16914_104	sCD14	Monocyte differentiation antigen CD14, soluble	P08571	CD14	NA	Inverse variance weighted	5	-0.036	0.082	6.583E-01	0.986	pan	Hospitalized COVID-19
2190_55	Coagulation Factor XI	Coagulation Factor XI	P03951	F11	NA	Inverse variance weighted	5	-0.021	0.047	6.583E-01	0.986	pan	Hospitalized COVID-19
10499_1	T106A	Transmembrane protein 106A	Q96A25	TMEM106A	NA	Inverse variance weighted	2	-0.061	0.139	6.593E-01	0.986	pan	Hospitalized COVID-19
18313_4	Asparaginase-like protein 1	Isoaspartyl peptidase/L-asparaginase	Q7L266	ASRGL1	NA	Inverse variance weighted	4	-0.026	0.060	6.606E-01	0.986	pan	Hospitalized COVID-19
12934_1	HERC5	E3 ISG15--protein ligase HERC5	Q9UII4	HERC5	NA	Wald ratio	1	-0.103	0.235	6.616E-01	0.986	pan	Hospitalized COVID-19
12659_13	GTPB9	Obg-like ATPase 1	Q9NTK5	OLA1	NA	Inverse variance weighted	2	-0.032	0.073	6.621E-01	0.986	pan	Hospitalized COVID-19
2774_10	IL-16	Interleukin-16	Q14005	IL16	NA	Inverse variance weighted	6	-0.013	0.031	6.622E-01	0.986	pan	Hospitalized COVID-19
9898_161	ARI1A	AT-rich interactive domain-containing protein 1A	O14497	ARID1A	NA	Wald ratio	1	0.109	0.249	6.624E-01	0.986	pan	Hospitalized COVID-19
4337_49	CRP	C-reactive protein	P02741	CRP	NA	Inverse variance weighted	7	0.034	0.079	6.632E-01	0.986	pan	Hospitalized COVID-19
5740_17	ROBO1	Roundabout homolog 1	Q9Y6N7	ROBO1	NA	Inverse variance weighted	6	-0.024	0.056	6.643E-01	0.986	pan	Hospitalized COVID-19
6997_32	RAB26	Ras-related protein Rab-26	Q9ULW5	RAB26	NA	Inverse variance weighted	3	-0.051	0.118	6.647E-01	0.986	pan	Hospitalized COVID-19
11287_14	Cytochrome b5	Cytochrome b5	P00167	CYB5A	NA	Wald ratio	1	0.113	0.262	6.653E-01	0.986	pan	Hospitalized COVID-19
8229_1	GXLT1	Glucoside xylosyltransferase 1	Q4G148	GXYLT1	NA	Inverse variance weighted	11	-0.013	0.031	6.654E-01	0.986	pan	Hospitalized COVID-19
17345_12	ZADH2	Prostaglandin reductase 3	Q8N4Q0	PTGR3	NA	Wald ratio	1	-0.044	0.103	6.668E-01	0.986	pan	Hospitalized COVID-19
7140_1	ELA2A	Chymotrypsin-like elastase family member 2A	P08217	CELA2A	NA	Inverse variance weighted	4	0.042	0.098	6.675E-01	0.986	pan	Hospitalized COVID-19
9470_15	MET24	Methyltransferase-like protein 24	Q5JXM2	METTL24	NA	Inverse variance weighted	5	-0.031	0.073	6.688E-01	0.986	pan	Hospitalized COVID-19
9805_51	SEM4B	Semaphorin-4B	Q9NPR2	SEMA4B	NA	Wald ratio	1	0.079	0.184	6.689E-01	0.986	pan	Hospitalized COVID-19
14009_65	TNFAIP3	Tumor necrosis factor alpha-induced protein 3	P21580	TNFAIP3	NA	Wald ratio	1	-0.028	0.065	6.696E-01	0.986	pan	Hospitalized COVID-19
18275_5	CRIP1	Cysteine-rich protein 1	P50238	CRIP1	NA	Inverse variance weighted	11	0.022	0.051	6.697E-01	0.986	pan	Hospitalized COVID-19
19392_6	DDAH1	N(G),N(G)-dimethylarginine dimethylaminohydrolase 1	O94760	DDAH1	NA	Inverse variance weighted	12	0.012	0.028	6.699E-01	0.986	pan	Hospitalized COVID-19
4984_83	Esterase D	S-formylglutathione hydrolase	P10768	ESD	NA	Inverse variance weighted	4	-0.018	0.041	6.711E-01	0.986	pan	Hospitalized COVID-19
3191_50	WFKN1	WAP, kazal, immunoglobulin, kunitz and NTR domain-containing protein 1	Q96NZ8	WFIKKN1	NA	Inverse variance weighted	2	-0.039	0.092	6.711E-01	0.986	pan	Hospitalized COVID-19
14082_56	TLN2	Talin-2	Q9Y4G6	TLN2	NA	Wald ratio	1	0.089	0.211	6.718E-01	0.986	pan	Hospitalized COVID-19
3864_5	RS7	40S ribosomal protein S7	P62081	RPS7	NA	Wald ratio	1	0.087	0.206	6.729E-01	0.986	pan	Hospitalized COVID-19
15440_57	NEC2	Neuroendocrine convertase 2	P16519	PCSK2	NA	Inverse variance weighted	2	-0.047	0.112	6.733E-01	0.986	pan	Hospitalized COVID-19
18896_23	H6ST3	Heparan-sulfate 6-O-sulfotransferase 3	Q8IZP7	HS6ST3	NA	Inverse variance weighted	2	0.041	0.098	6.735E-01	0.986	pan	Hospitalized COVID-19
13044_5	TS101	Tumor susceptibility gene 101 protein	Q99816	TSG101	NA	Inverse variance weighted	2	-0.045	0.106	6.740E-01	0.986	pan	Hospitalized COVID-19
13116_25	CD177	CD177 antigen	Q8N6Q3	CD177	NA	Inverse variance weighted	10	0.011	0.025	6.747E-01	0.986	pan	Hospitalized COVID-19
8893_29	PARP:region 1	Poly [ADP-ribose] polymerase 1:region 1	P09874	PARP1	NA	Wald ratio	1	-0.085	0.204	6.759E-01	0.986	pan	Hospitalized COVID-19
14131_37	EFNB2:ECD	Ephrin-B2:Extracellular domain	P52799	EFNB2	NA	Inverse variance weighted	2	-0.062	0.148	6.760E-01	0.986	pan	Hospitalized COVID-19
8885_6	CA2D3	Voltage-dependent calcium channel subunit alpha-2/delta-3	Q8IZS8	CACNA2D3	NA	Inverse variance weighted	9	-0.015	0.035	6.760E-01	0.986	pan	Hospitalized COVID-19
7841_84	ESAM	Endothelial cell-selective adhesion molecule	Q96AP7	ESAM	NA	Inverse variance weighted	6	0.017	0.040	6.765E-01	0.986	pan	Hospitalized COVID-19
14332_3	RALA	Ras-related protein Ral-A	P11233	RALA	NA	Inverse variance weighted	2	-0.069	0.165	6.765E-01	0.986	pan	Hospitalized COVID-19
15548_35	SERA	D-3-phosphoglycerate dehydrogenase	O43175	PHGDH	NA	Inverse variance weighted	2	0.068	0.162	6.766E-01	0.986	pan	Hospitalized COVID-19
11211_7	TBCE	Tubulin-specific chaperone E	Q15813	TBCE	NA	Inverse variance weighted	2	-0.066	0.158	6.766E-01	0.986	pan	Hospitalized COVID-19
3327_27	NET4	Netrin-4	Q9HB63	NTN4	NA	Inverse variance weighted	5	0.031	0.074	6.767E-01	0.986	pan	Hospitalized COVID-19
18918_86	PDE4A	cAMP-specific 3',5'-cyclic phosphodiesterase 4A	P27815	PDE4A	NA	Inverse variance weighted	4	-0.041	0.099	6.769E-01	0.986	pan	Hospitalized COVID-19
9738_7	FAF2	FAS-associated factor 2	Q96CS3	FAF2	NA	Wald ratio	1	-0.125	0.300	6.777E-01	0.986	pan	Hospitalized COVID-19
4923_79	MIS	Muellerian-inhibiting factor	P03971	AMH	NA	Inverse variance weighted	5	0.040	0.097	6.785E-01	0.986	pan	Hospitalized COVID-19
11104_13	YKL-40	Chitinase-3-like protein 1	P36222	CHI3L1	NA	Inverse variance weighted	11	0.007	0.018	6.785E-01	0.986	pan	Hospitalized COVID-19
9264_11	CATO	Cathepsin O	P43234	CTSO	NA	Inverse variance weighted	3	-0.027	0.065	6.786E-01	0.986	pan	Hospitalized COVID-19
19335_2	HN1	Hematological and neurological expressed 1 protein	Q9UK76	JPT1	NA	Wald ratio	1	0.097	0.234	6.795E-01	0.986	pan	Hospitalized COVID-19
5648_28	CTRB2	Chymotrypsinogen B2	Q6GPI1	CTRB2	NA	Inverse variance weighted	11	0.009	0.023	6.805E-01	0.986	pan	Hospitalized COVID-19
4971_1	CATZ	Cathepsin Z	Q9UBR2	CTSZ	NA	Inverse variance weighted	6	-0.014	0.033	6.807E-01	0.986	pan	Hospitalized COVID-19
2961_1	Protein C	Vitamin K-dependent protein C	P04070	PROC	NA	Inverse variance weighted	8	0.026	0.062	6.809E-01	0.986	pan	Hospitalized COVID-19
5346_24	CPNE1:C2, 1 and 2	Copine-1:Ca2+-dependent membrane-targeting module domains 1 and 2	Q99829	CPNE1	NA	Inverse variance weighted	12	-0.010	0.025	6.813E-01	0.986	pan	Hospitalized COVID-19
7809_22	SPAT9	Spermatogenesis-associated protein 9	Q9BWV2	SPATA9	NA	Wald ratio	1	-0.072	0.176	6.818E-01	0.986	pan	Hospitalized COVID-19
12551_3	ITCH	E3 ubiquitin-protein ligase Itchy homolog	Q96J02	ITCH	NA	Inverse variance weighted	2	-0.128	0.312	6.818E-01	0.986	pan	Hospitalized COVID-19
9751_72	NSEP1	Nuclease-sensitive element-binding protein 1	P67809	YBX1	NA	Inverse variance weighted	5	-0.038	0.092	6.826E-01	0.986	pan	Hospitalized COVID-19
19272_9	PEF1	Peflin	Q9UBV8	PEF1	NA	Inverse variance weighted	10	0.013	0.032	6.827E-01	0.986	pan	Hospitalized COVID-19
8043_153	COMP	Cartilage oligomeric matrix protein	P49747	COMP	NA	Inverse variance weighted	8	0.023	0.058	6.840E-01	0.986	pan	Hospitalized COVID-19
8327_26	DPEP2	Dipeptidase 2	Q9H4A9	DPEP2	NA	Inverse variance weighted	6	-0.019	0.048	6.843E-01	0.986	pan	Hospitalized COVID-19
15594_47	HTRA1	Serine protease HTRA1	Q92743	HTRA1	NA	Inverse variance weighted	2	-0.103	0.255	6.848E-01	0.986	pan	Hospitalized COVID-19
7953_20	SLAF1	Signaling lymphocytic activation molecule	Q13291	SLAMF1	NA	Wald ratio	1	-0.074	0.184	6.855E-01	0.986	pan	Hospitalized COVID-19
18340_2	Proteasome beta chain	Proteasome subunit beta type-4	P28070	PSMB4	NA	Inverse variance weighted	4	-0.010	0.025	6.859E-01	0.986	pan	Hospitalized COVID-19
12357_41	SNP29	Synaptosomal-associated protein 29	O95721	SNAP29	NA	Wald ratio	1	0.067	0.166	6.868E-01	0.986	pan	Hospitalized COVID-19
18458_4	PGES2	Prostaglandin E synthase 2	Q9H7Z7	PTGES2	NA	Wald ratio	1	-0.055	0.137	6.872E-01	0.986	pan	Hospitalized COVID-19
6920_1	GFRAL	GDNF family receptor alpha-like	Q6UXV0	GFRAL	NA	Inverse variance weighted	6	0.019	0.047	6.875E-01	0.986	pan	Hospitalized COVID-19
8874_53	CLN5:LD	Ceroid-lipofuscinosis neuronal protein 5:Lumenal domain	O75503	CLN5	NA	Inverse variance weighted	2	-0.038	0.095	6.886E-01	0.986	pan	Hospitalized COVID-19
4969_2	Carbonic anhydrase I	Carbonic anhydrase 1	P00915	CA1	NA	Inverse variance weighted	6	0.022	0.055	6.888E-01	0.986	pan	Hospitalized COVID-19
18218_48	CB032	CB1 cannabinoid receptor-interacting protein 1	Q96F85	CNRIP1	NA	Inverse variance weighted	10	0.011	0.028	6.889E-01	0.986	pan	Hospitalized COVID-19
3607_71	DKK3	Dickkopf-related protein 3	Q9UBP4	DKK3	NA	Inverse variance weighted	10	-0.013	0.032	6.890E-01	0.986	pan	Hospitalized COVID-19
10924_258	NEUFC	Neuferricin	Q8WUJ1	CYB5D2	NA	Inverse variance weighted	3	-0.023	0.057	6.893E-01	0.986	pan	Hospitalized COVID-19
3339_33	TSP2	Thrombospondin-2	P35442	THBS2	NA	Inverse variance weighted	8	0.010	0.025	6.907E-01	0.986	pan	Hospitalized COVID-19

3212_30	ASAH2	Neutral ceramidase	Q9NR71	ASAH2	NA	Inverse variance weighted	10	0.015	0.038	6.910E-01	0.986	pan	Hospitalized COVID-19
15565_102	CA125	Mucin-16	Q8WXI7	MUC16	NA	Inverse variance weighted	8	0.016	0.040	6.913E-01	0.986	pan	Hospitalized COVID-19
12743_18	BAG5	BAG family molecular chaperone regulator 5	Q9UL15	BAG5	NA	Wald ratio	1	-0.089	0.226	6.926E-01	0.986	pan	Hospitalized COVID-19
7935_26	LARGE	Glycosyltransferase-like protein LARGE1	O95461	LARGE1	NA	Inverse variance weighted	7	-0.024	0.062	6.926E-01	0.986	pan	Hospitalized COVID-19
14318_1	VPS29	Vacuolar protein sorting-associated protein 29	Q9UBQ0	VPS29	NA	Inverse variance weighted	2	0.049	0.125	6.930E-01	0.986	pan	Hospitalized COVID-19
6402_8	PILRA isoform FDF03-deltaTM	Paired immunoglobulin-like type 2 receptor alpha isoform FDF03-deltaTM	Q9UKJ1	PILRA	NA	Inverse variance weighted	14	0.028	0.072	6.942E-01	0.986	pan	Hospitalized COVID-19
16773_29	SCUB3	Signal peptide, CUB and EGF-like domain-containing protein 3	Q8IX30	SCUBE3	NA	Inverse variance weighted	4	0.018	0.047	6.953E-01	0.986	pan	Hospitalized COVID-19
19241_31	RBP-III	Retinol-binding protein 5	P82980	RBP5	NA	Wald ratio	1	-0.080	0.205	6.961E-01	0.986	pan	Hospitalized COVID-19
9759_13	INDO	Indoleamine 2,3-dioxygenase 1	P14902	IDO1	NA	Wald ratio	1	-0.068	0.173	6.963E-01	0.986	pan	Hospitalized COVID-19
6923_1	PLOD2	Procollagen-lysine,2-oxoglutarate 5-dioxygenase 2	O00469	PLOD2	NA	Inverse variance weighted	3	-0.037	0.096	6.973E-01	0.986	pan	Hospitalized COVID-19
5586_66	MINP1	Multiple inositol polyphosphate phosphatase 1	Q9UNW1	MINPP1	NA	Inverse variance weighted	4	0.034	0.087	6.980E-01	0.986	pan	Hospitalized COVID-19
15585_304	fibulin 5	Fibulin-5	Q9UBX5	FBLN5	NA	Wald ratio	1	0.049	0.126	6.980E-01	0.986	pan	Hospitalized COVID-19
12549_33	PTGD2	Hematopoietic prostaglandin D synthase	O60760	HPGDS	NA	Inverse variance weighted	12	-0.008	0.021	6.983E-01	0.986	pan	Hospitalized COVID-19
15370_5	BOLA1	BolA-like protein 1	Q9Y3E2	BOLA1	NA	Inverse variance weighted	13	-0.013	0.033	6.989E-01	0.986	pan	Hospitalized COVID-19
3448_13	IR	Insulin receptor	P06213	INSR	NA	Inverse variance weighted	14	-0.028	0.071	6.995E-01	0.986	pan	Hospitalized COVID-19
2647_66	Rab GDP dissociation inhibitor beta	Rab GDP dissociation inhibitor beta	P50395	GDI2	NA	Wald ratio	1	-0.040	0.104	6.996E-01	0.986	pan	Hospitalized COVID-19
18882_7	CSTN2	Calsyntenin-2	Q9H4D0	CLSTN2	NA	Inverse variance weighted	10	-0.016	0.043	6.998E-01	0.986	pan	Hospitalized COVID-19
5109_24	Nr-CAM	Neuronal cell adhesion molecule	Q92823	NRCAM	NA	Wald ratio	1	0.070	0.180	6.998E-01	0.986	pan	Hospitalized COVID-19
15535_3	Marapsin	Serine protease 27	Q9BQR3	PRSS27	NA	Inverse variance weighted	11	0.021	0.055	6.999E-01	0.986	pan	Hospitalized COVID-19
12450_42	PMVK	Phosphomevalonate kinase	Q15126	PMVK	NA	Inverse variance weighted	2	0.206	0.536	7.003E-01	0.986	pan	Hospitalized COVID-19
12332_7	EF2K	Eukaryotic elongation factor 2 kinase	O00418	EEF2K	NA	Inverse variance weighted	2	0.094	0.245	7.004E-01	0.986	pan	Hospitalized COVID-19
16305_10	Cadherin-11:ECD	Cadherin-11:Extracellular domain	P55287	CDH11	NA	Inverse variance weighted	11	0.017	0.044	7.006E-01	0.986	pan	Hospitalized COVID-19
12376_85	p19-INK4d	Cyclin-dependent kinase 4 inhibitor D	P55273	CDKN2D	NA	Wald ratio	1	-0.098	0.256	7.015E-01	0.986	pan	Hospitalized COVID-19
7215_18	NADH-cytochrome b5 reductase	NADH-cytochrome b5 reductase 3	P00387	CYB5R3	NA	Inverse variance weighted	2	-0.057	0.149	7.018E-01	0.986	pan	Hospitalized COVID-19
7952_2	VTI1A	Vesicle transport through interaction with t-SNAREs homolog 1A	Q96AJ9	VTI1A	NA	Wald ratio	1	-0.089	0.232	7.019E-01	0.986	pan	Hospitalized COVID-19
16561_9	Alpha-1B-glycoprotein	Alpha-1B-glycoprotein	P04217	A1BG	NA	Inverse variance weighted	8	0.009	0.024	7.020E-01	0.986	pan	Hospitalized COVID-19
16770_3	REG1B	Lithostathine-1-beta	P48304	REG1B	NA	Inverse variance weighted	4	0.061	0.159	7.022E-01	0.986	pan	Hospitalized COVID-19
5837_49	LIF sR	Leukemia inhibitory factor receptor	P42702	LIFR	NA	Inverse variance weighted	9	-0.020	0.052	7.024E-01	0.986	pan	Hospitalized COVID-19
10047_12	NCF-2	Neutrophil cytosol factor 2	P19878	NCF2	NA	Inverse variance weighted	4	-0.035	0.091	7.027E-01	0.986	pan	Hospitalized COVID-19
9340_17	FKB14	Peptidyl-prolyl cis-trans isomerase FKBP14	Q9NWM8	FKBP14	NA	Wald ratio	1	0.082	0.214	7.029E-01	0.986	pan	Hospitalized COVID-19
9191_8	Trefoil factor 2	Trefoil factor 2	Q03403	TFF2	NA	Inverse variance weighted	4	0.028	0.072	7.034E-01	0.986	pan	Hospitalized COVID-19
19161_1	UBP15	Ubiquitin carboxyl-terminal hydrolase 15	Q9Y4E8	USP15	NA	Inverse variance weighted	2	-0.027	0.070	7.037E-01	0.986	pan	Hospitalized COVID-19
4546_27	EMR2	Adhesion G protein-coupled receptor E2	Q9UHX3	ADGRE2	NA	Inverse variance weighted	6	-0.016	0.041	7.039E-01	0.986	pan	Hospitalized COVID-19
3352_80	Carbonic anhydrase 6	Carbonic anhydrase 6	P23280	CA6	NA	Inverse variance weighted	17	-0.010	0.027	7.045E-01	0.986	pan	Hospitalized COVID-19
15633_6	RBP	Retinol-binding protein 4	P02753	RBP4	NA	Wald ratio	1	-0.057	0.150	7.048E-01	0.986	pan	Hospitalized COVID-19
6625_31	ATAD1	ATPase family AAA domain-containing protein 1	Q8NBU5	ATAD1	NA	Wald ratio	1	-0.029	0.077	7.049E-01	0.986	pan	Hospitalized COVID-19
2201_17	Endostatin	Endostatin	P39060	COL18A1	NA	Inverse variance weighted	4	-0.022	0.058	7.050E-01	0.986	pan	Hospitalized COVID-19
17455_42	FOLR1	Folate receptor alpha	P15328	FOLR1	NA	Wald ratio	1	0.034	0.090	7.053E-01	0.986	pan	Hospitalized COVID-19
14091_42	Carbonyl reductase 3	Carbonyl reductase [NADPH] 3	O75828	CBR3	NA	Inverse variance weighted	5	0.011	0.029	7.055E-01	0.986	pan	Hospitalized COVID-19
4131_72	Fibronectin	Fibronectin	P02751	FN1	NA	Inverse variance weighted	2	0.117	0.311	7.063E-01	0.986	pan	Hospitalized COVID-19
2692_74	NPS-PLA2	Phospholipase A2, membrane associated	P14555	PLA2G2A	NA	Inverse variance weighted	7	-0.007	0.019	7.063E-01	0.986	pan	Hospitalized COVID-19
3299_29	Contactin-5	Contactin-5	O94779	CNTN5	NA	Inverse variance weighted	4	0.016	0.043	7.070E-01	0.986	pan	Hospitalized COVID-19
18289_16	MIP-5	C-C motif chemokine 15	Q16663	CCL15	NA	Inverse variance weighted	9	0.018	0.048	7.074E-01	0.986	pan	Hospitalized COVID-19
7784_1	Kininogen, HMW	Kininogen-1	P01042	KNG1	NA	Inverse variance weighted	2	0.052	0.140	7.080E-01	0.986	pan	Hospitalized COVID-19
19631_13	Kininostatin	Kininostatin	P01042	KNG1	NA	Inverse variance weighted	12	0.024	0.064	7.082E-01	0.986	pan	Hospitalized COVID-19
13934_3	MCF2L	Guanine nucleotide exchange factor DBS	O15068	MCF2L	NA	Inverse variance weighted	2	-0.018	0.048	7.090E-01	0.986	pan	Hospitalized COVID-19
18338_26	IDH	Isocitrate dehydrogenase [NADP] cytoplasmic	O75874	IDH1	NA	Inverse variance weighted	3	-0.018	0.048	7.098E-01	0.986	pan	Hospitalized COVID-19
8245_27	sICAM-5	Intercellular adhesion molecule 5	Q9UMF0	ICAM5	NA	Inverse variance weighted	6	-0.029	0.078	7.100E-01	0.986	pan	Hospitalized COVID-19
5657_28	SIA4A	CMP-N-acetylneuraminate-beta-galactosamide-alpha-2,3-sialyltransferase 1	Q11201	ST3GAL1	NA	Inverse variance weighted	3	-0.036	0.096	7.101E-01	0.986	pan	Hospitalized COVID-19
10442_1	TM190	Transmembrane protein 190	Q8WZ59	TMEM190	NA	Inverse variance weighted	8	-0.008	0.021	7.107E-01	0.986	pan	Hospitalized COVID-19
9386_42	TTC9B	Tetratricopeptide repeat protein 9B	Q8N6N2	TTC9B	NA	Inverse variance weighted	2	-0.031	0.085	7.115E-01	0.986	pan	Hospitalized COVID-19
15491_20	CD248	Endosialin	Q9HCU0	CD248	NA	Wald ratio	1	-0.070	0.189	7.117E-01	0.986	pan	Hospitalized COVID-19
14101_2	CNTFR alpha	Ciliary neurotrophic factor receptor subunit alpha	P26992	CNTFR	NA	Inverse variance weighted	5	-0.025	0.068	7.125E-01	0.986	pan	Hospitalized COVID-19
7244_16	TM149	IGF-like family receptor 1	Q9H665	IGFLR1	NA	Inverse variance weighted	7	0.009	0.024	7.125E-01	0.986	pan	Hospitalized COVID-19
10569_28	MFAP2	Microfibrillar-associated protein 2	P55001	MFAP2	NA	Inverse variance weighted	3	-0.050	0.137	7.126E-01	0.986	pan	Hospitalized COVID-19
14123_34	GI24:ECD	V-type immunoglobulin domain-containing suppressor of T-cell activation:Extracellular domain	Q9H7M9	VSIR	NA	Inverse variance weighted	6	-0.029	0.079	7.126E-01	0.986	pan	Hospitalized COVID-19
11616_9	HSF1	Heat shock factor protein 1	Q00613	HSF1	NA	Wald ratio	1	0.069	0.188	7.136E-01	0.986	pan	Hospitalized COVID-19
4563_61	PLCG1	1-phosphatidylinositol 4,5-bisphosphate phosphodiesterase gamma-1	P19174	PLCG1	NA	Wald ratio	1	-0.066	0.179	7.139E-01	0.986	pan	Hospitalized COVID-19
2617_56	ERBB3	Receptor tyrosine-protein kinase erbB-3	P21860	ERBB3	NA	Inverse variance weighted	2	-0.075	0.204	7.142E-01	0.986	pan	Hospitalized COVID-19
15376_134	CATE	Cathepsin E	P14091	CTSE	NA	Inverse variance weighted	5	0.018	0.050	7.144E-01	0.986	pan	Hospitalized COVID-19
11177_16	K2C5	Keratin, type II cytoskeletal 5	P13647	KRT5	NA	Inverse variance weighted	7	-0.029	0.078	7.144E-01	0.986	pan	Hospitalized COVID-19
11278_4	COL11A2	Collagen alpha-2(XI) chain	P13942	COL11A2	NA	Inverse variance weighted	7	-0.016	0.045	7.148E-01	0.986	pan	Hospitalized COVID-19
5005_4	MK12	Mitogen-activated protein kinase 12	P53778	MAPK12	NA	Inverse variance weighted	2	0.030	0.083	7.148E-01	0.986	pan	Hospitalized COVID-19
3470_1	sE-Selectin	E-selectin	P16581	SELE	NA	Inverse variance weighted	8	0.023	0.062	7.157E-01	0.986	pan	Hospitalized COVID-19
6453_70	LIRB4	Leukocyte immunoglobulin-like receptor subfamily B member 4	Q8NHJ6	LILRB4	NA	Inverse variance weighted	2	-0.049	0.135	7.158E-01	0.986	pan	Hospitalized COVID-19
10082_251	NFL	Neurofilament light polypeptide	P07196	NEFL	NA	Inverse variance weighted	3	-0.090	0.248	7.159E-01	0.986	pan	Hospitalized COVID-19
8099_42	SPON2	Spondin-2	Q9BUD6	SPON2	NA	Inverse variance weighted	4	-0.016	0.044	7.175E-01	0.986	pan	Hospitalized COVID-19
5029_3	SEPR	Prolyl endopeptidase FAP	Q12884	FAP	NA	Inverse variance weighted	6	0.021	0.057	7.178E-01	0.986	pan	Hospitalized COVID-19
10372_18	STAT6	Signal transducer and activator of transcription 6	P42226	STAT6	NA	Wald ratio	1	-0.054	0.151	7.181E-01	0.986	pan	Hospitalized COVID-19
2813_11	ART	Agouti-related protein	O00253	AGRP	NA	Inverse variance weighted	5	0.042	0.117	7.181E-01	0.986	pan	Hospitalized COVID-19
12350_86	ETS2	Protein C-ets-2	P15036	ETS2	NA	Inverse variance weighted	6	0.032	0.088	7.185E-01	0.986	pan	Hospitalized COVID-19
6439_59	CN093	Uncharacterized protein C14orf93	Q9H972	C14orf93	NA	Wald ratio	1	0.078	0.215	7.185E-01	0.986	pan	Hospitalized COVID-19
18213_30	UBE2F	NEDD8-conjugating enzyme UBE2F	Q969M7	UBE2F	NA	Inverse variance weighted	2	-0.039	0.109	7.197E-01	0.986	pan	Hospitalized COVID-19
2999_6	LSAMP	Limbic system-associated membrane protein	Q13449	LSAMP	NA	Inverse variance weighted	5	0.021	0.059	7.198E-01	0.986	pan	Hospitalized COVID-19
17410_5	CETN3	Centrin-3	O15182	CETN3	NA	Inverse variance weighted	3	-0.032	0.090	7.206E-01	0.986	pan	Hospitalized COVID-19
10977_55	UCMA	Unique cartilage matrix-associated protein	Q8WVF2	UCMA	NA	Inverse variance weighted	9	0.010	0.029	7.213E-01	0.986	pan	Hospitalized COVID-19
13095_51	PSP	Lithostathine-1-alpha	P05451	REG1A	NA	Inverse variance weighted	7	0.028	0.078	7.214E-01	0.986	pan	Hospitalized COVID-19
12459_13	PKHA1	Pleckstrin homology domain-containing family A member 1	Q9HB21	PLEKHA1	NA	Inverse variance weighted	3	0.012	0.034	7.214E-01	0.986	pan	Hospitalized COVID-19

18864_7	TRY3	Trypsin-3	P35030	PRSS3	NA	Inverse variance weighted	29	0.008	0.022	7.215E-01	0.986	pan	Hospitalized COVID-19
3504_58	LEAP-1	Hepcidin	P81172	HAMP	NA	Wald ratio	1	0.072	0.202	7.222E-01	0.986	pan	Hospitalized COVID-19
10574_10	b2-Microglobulin	Beta-2-microglobulin	P61769	B2M	NA	Inverse variance weighted	8	-0.016	0.046	7.227E-01	0.986	pan	Hospitalized COVID-19
7145_1	ITIH3	Inter-alpha-trypsin inhibitor heavy chain H3	Q06033	ITIH3	NA	Inverse variance weighted	11	-0.016	0.045	7.227E-01	0.986	pan	Hospitalized COVID-19
16781_2	ENASE	Cytosolic endo-beta-N-acetylglucosaminidase	Q8NFI3	ENGASE	NA	Inverse variance weighted	11	0.008	0.022	7.233E-01	0.986	pan	Hospitalized COVID-19
14054_17	IL-15 Ra	Interleukin-15 receptor subunit alpha	Q13261	IL15RA	NA	Inverse variance weighted	8	0.012	0.034	7.236E-01	0.986	pan	Hospitalized COVID-19
12643_4	ARRB1	Beta-arrestin-1	P49407	ARRB1	NA	Wald ratio	1	-0.047	0.132	7.239E-01	0.986	pan	Hospitalized COVID-19
10710_23	ZPBP2	Zona pellucida-binding protein 2	Q6X784	ZPBP2	NA	Wald ratio	1	0.076	0.214	7.239E-01	0.986	pan	Hospitalized COVID-19
3284_75	BGN	Biglycan	P21810	BGN	NA	Inverse variance weighted	14	-0.013	0.037	7.241E-01	0.986	pan	Hospitalized COVID-19
12612_37	PSB1	Proteasome subunit beta type-1	P20618	PSMB1	NA	Inverse variance weighted	3	0.017	0.048	7.242E-01	0.986	pan	Hospitalized COVID-19
13114_50	Lumican	Lumican	P51884	LUM	NA	Inverse variance weighted	9	-0.011	0.032	7.246E-01	0.986	pan	Hospitalized COVID-19
9235_3	PXDC1	Plexin domain-containing protein 1	Q8IUK5	PLXDC1	NA	Inverse variance weighted	6	0.019	0.055	7.247E-01	0.986	pan	Hospitalized COVID-19
12560_9	NT5C	5'(3')-deoxyribonucleotidase, cytosolic type	Q8TCD5	NT5C	NA	Inverse variance weighted	5	-0.009	0.027	7.248E-01	0.986	pan	Hospitalized COVID-19
15475_4	PLTP	Phospholipid transfer protein	P55058	PLTP	NA	Inverse variance weighted	11	0.017	0.049	7.251E-01	0.986	pan	Hospitalized COVID-19
18236_3	LEGL	Galectin-related protein	Q3ZCW2	LGALSL	NA	Wald ratio	1	-0.047	0.133	7.253E-01	0.986	pan	Hospitalized COVID-19
16015_19	ALT	Alanine aminotransferase 1	P24298	GPT	NA	Wald ratio	1	-0.067	0.192	7.262E-01	0.986	pan	Hospitalized COVID-19
4549_78	FUT5	Alpha-(1,3)-fucosyltransferase 5	Q11128	FUT5	NA	Inverse variance weighted	11	-0.008	0.022	7.266E-01	0.986	pan	Hospitalized COVID-19
3283_21	BGH3	Transforming growth factor-beta-induced protein ig-h3	Q15582	TGFBI	NA	Inverse variance weighted	11	-0.011	0.032	7.268E-01	0.986	pan	Hospitalized COVID-19
10818_36	ASM	Sphingomyelin phosphodiesterase	P17405	SMPD1	NA	Inverse variance weighted	11	0.009	0.026	7.268E-01	0.986	pan	Hospitalized COVID-19
2816_50	BCAM	Basal Cell Adhesion Molecule	P50895	BCAM	NA	Inverse variance weighted	3	-0.031	0.088	7.270E-01	0.986	pan	Hospitalized COVID-19
3581_53	a2-HS-Glycoprotein	Alpha-2-HS-glycoprotein	P02765	AHSG	NA	Inverse variance weighted	4	0.032	0.092	7.274E-01	0.986	pan	Hospitalized COVID-19
8382_47	MAPK5	MAP kinase-activated protein kinase 5	Q8IW41	MAPKAPK5	NA	Inverse variance weighted	12	-0.018	0.052	7.279E-01	0.986	pan	Hospitalized COVID-19
18823_52	H2B3B	Histone H2B type 3-B	Q8N257	H2BU1	NA	Inverse variance weighted	2	0.061	0.175	7.280E-01	0.986	pan	Hospitalized COVID-19
8465_52	Cathepsin H	Cathepsin H	P09668	CTSH	NA	Inverse variance weighted	17	-0.007	0.020	7.283E-01	0.986	pan	Hospitalized COVID-19
9511_61	NXPH2	Neurexophilin-2	O95156	NXPH2	NA	Inverse variance weighted	3	-0.054	0.156	7.285E-01	0.986	pan	Hospitalized COVID-19
17320_19	ADPPT	L-aminoadipate-semialdehyde dehydrogenase-phosphopantetheinyl transferase	Q9NRN7	AASDHPPT	NA	Wald ratio	1	0.082	0.237	7.286E-01	0.986	pan	Hospitalized COVID-19
3186_2	C2	Complement C2	P06681	C2	NA	Inverse variance weighted	9	0.019	0.054	7.287E-01	0.986	pan	Hospitalized COVID-19
8358_30	Peroxiredoxin-3	Thioredoxin-dependent peroxide reductase, mitochondrial	P30048	PRDX3	NA	Wald ratio	1	0.075	0.217	7.291E-01	0.986	pan	Hospitalized COVID-19
4960_72	annexin I	Annexin A1	P04083	ANXA1	NA	Wald ratio	1	-0.029	0.084	7.309E-01	0.986	pan	Hospitalized COVID-19
15604_18	JNK2	Mitogen-activated protein kinase 9	P45984	MAPK9	NA	Inverse variance weighted	2	-0.036	0.106	7.310E-01	0.986	pan	Hospitalized COVID-19
9842_2	b-Catenin	Catenin beta-1	P35222	CTNNB1	NA	Inverse variance weighted	10	-0.010	0.029	7.312E-01	0.986	pan	Hospitalized COVID-19
6385_63	VWA1	von Willebrand factor A domain-containing protein 1	Q6PCB0	VWA1	NA	Inverse variance weighted	7	-0.014	0.043	7.338E-01	0.986	pan	Hospitalized COVID-19
5980_55	BOLA3	BolA-like protein 3	Q53S33	BOLA3	NA	Wald ratio	1	0.029	0.086	7.342E-01	0.986	pan	Hospitalized COVID-19
8859_51	CAH11	Carbonic anhydrase-related protein 11	O75493	CA11	NA	Inverse variance weighted	2	-0.047	0.137	7.344E-01	0.986	pan	Hospitalized COVID-19
9316_67	WFDC1	WAP four-disulfide core domain protein 1	Q9HC57	WFDC1	NA	Inverse variance weighted	5	0.013	0.039	7.345E-01	0.986	pan	Hospitalized COVID-19
12714_38	AP1G2	AP-1 complex subunit gamma-like 2	O75843	AP1G2	NA	Inverse variance weighted	3	0.019	0.057	7.345E-01	0.986	pan	Hospitalized COVID-19
11266_8	SELPL:ECD	P-selectin glycoprotein ligand 1:Extracellular domain	Q14242	SELPLG	NA	Inverse variance weighted	13	-0.009	0.027	7.346E-01	0.986	pan	Hospitalized COVID-19
5620_13	AMD	Peptidyl-glycine alpha-amidating monooxygenase	P19021	PAM	NA	Inverse variance weighted	19	0.007	0.022	7.348E-01	0.986	pan	Hospitalized COVID-19
12764_3	ELMO1	Engulfment and cell motility protein 1	Q92556	ELMO1	NA	Inverse variance weighted	3	-0.030	0.088	7.348E-01	0.986	pan	Hospitalized COVID-19
4721_54	TFF3	Trefoil factor 3	Q07654	TFF3	NA	Inverse variance weighted	4	0.034	0.101	7.351E-01	0.986	pan	Hospitalized COVID-19
12356_65	Sorcin	Sorcin	P30626	SRI	NA	Wald ratio	1	0.030	0.088	7.356E-01	0.986	pan	Hospitalized COVID-19
3852_19	HSP 40	DnaJ homolog subfamily B member 1	P25685	DNAJB1	NA	Inverse variance weighted	3	-0.037	0.109	7.357E-01	0.986	pan	Hospitalized COVID-19
9876_20	aldolase C	Fructose-bisphosphate aldolase C	P09972	ALDOC	NA	Inverse variance weighted	3	-0.036	0.105	7.357E-01	0.986	pan	Hospitalized COVID-19
13056_18	S6A16	Orphan sodium- and chloride-dependent neurotransmitter transporter NTT5	Q9GZN6	SLC6A16	NA	Wald ratio	1	0.080	0.239	7.362E-01	0.986	pan	Hospitalized COVID-19
9329_28	TRML1:ECD	Trem-like transcript 1 protein:Extracellular domain, Ig-like V-type domain	Q86YW5	TREML1	NA	Inverse variance weighted	2	0.085	0.253	7.363E-01	0.986	pan	Hospitalized COVID-19
10627_87	APLP2	Amyloid-like protein 2	Q06481	APLP2	NA	Inverse variance weighted	2	0.049	0.145	7.366E-01	0.986	pan	Hospitalized COVID-19
19606_28	ihh	Indian hedgehog protein	Q14623	IHH	NA	Wald ratio	1	0.064	0.192	7.369E-01	0.986	pan	Hospitalized COVID-19
8948_13	ADA19	Disintegrin and metalloproteinase domain-containing protein 19	Q9H013	ADAM19	NA	Wald ratio	1	-0.113	0.336	7.370E-01	0.986	pan	Hospitalized COVID-19
13666_222	Carbonic Anhydrase X	Carbonic anhydrase-related protein 10	Q9NS85	CA10	NA	Inverse variance weighted	8	-0.025	0.074	7.372E-01	0.986	pan	Hospitalized COVID-19
13059_33	RIFK	Riboflavin kinase	Q969G6	RFK	NA	Inverse variance weighted	2	-0.034	0.102	7.374E-01	0.986	pan	Hospitalized COVID-19
6455_52	HPLN4	Hyaluronan and proteoglycan link protein 4	Q86UW8	HAPLN4	NA	Wald ratio	1	0.075	0.225	7.378E-01	0.986	pan	Hospitalized COVID-19
8258_22	UXS1	UDP-glucuronic acid decarboxylase 1	Q8NBZ7	UXS1	NA	Inverse variance weighted	2	0.123	0.368	7.378E-01	0.986	pan	Hospitalized COVID-19
9832_33	HGD	Homogentisate 1,2-dioxygenase	Q93099	HGD	NA	Inverse variance weighted	2	-0.055	0.166	7.380E-01	0.986	pan	Hospitalized COVID-19
2590_69	ROR1	Inactive tyrosine-protein kinase transmembrane receptor ROR1	Q01973	ROR1	NA	Inverse variance weighted	4	-0.013	0.040	7.384E-01	0.986	pan	Hospitalized COVID-19
17350_13	CHM2B	Charged multivesicular body protein 2b	Q9UQN3	CHMP2B	NA	Inverse variance weighted	3	-0.018	0.053	7.385E-01	0.986	pan	Hospitalized COVID-19
2515_14	GFRa-2	GDNF family receptor alpha-2	O00451	GFRA2	NA	Inverse variance weighted	7	0.009	0.027	7.386E-01	0.986	pan	Hospitalized COVID-19
17453_34	Ceruloplasmin	Ceruloplasmin	P00450	CP	NA	Inverse variance weighted	3	-0.029	0.086	7.391E-01	0.986	pan	Hospitalized COVID-19
13982_33	RGS18	Regulator of G-protein signaling 18	Q9NS28	RGS18	NA	Wald ratio	1	-0.057	0.173	7.394E-01	0.986	pan	Hospitalized COVID-19
3132_1	VEGF-C	Vascular endothelial growth factor C	P49767	VEGFC	NA	Wald ratio	1	-0.042	0.126	7.397E-01	0.986	pan	Hospitalized COVID-19
10953_14	CLC2A	C-type lectin domain family 2 member A	Q6UVW9	CLEC2A	NA	Inverse variance weighted	4	0.031	0.095	7.399E-01	0.986	pan	Hospitalized COVID-19
2706_69	Thyroxine-Binding Globulin	Thyroxine-binding globulin	P05543	SERPINA7	NA	Inverse variance weighted	2	0.067	0.201	7.406E-01	0.986	pan	Hospitalized COVID-19
4407_10	MSP	Hepatocyte growth factor-like protein	P26927	MST1	NA	Inverse variance weighted	20	0.010	0.029	7.411E-01	0.986	pan	Hospitalized COVID-19
3651_50	VEGF sR2	Vascular endothelial growth factor receptor 2	P35968	KDR	NA	Inverse variance weighted	9	0.008	0.025	7.417E-01	0.986	pan	Hospitalized COVID-19
2516_57	6Ckine	C-C motif chemokine 21	O00585	CCL21	NA	Inverse variance weighted	17	0.014	0.042	7.418E-01	0.986	pan	Hospitalized COVID-19
12020_39	PMGE	Bisphosphoglycerate mutase	P07738	BPGM	NA	Wald ratio	1	0.089	0.271	7.423E-01	0.986	pan	Hospitalized COVID-19
19188_21	NAP2L	nucleosome assembly protein 1-like 4	Q99733	NAP1L4	NA	Inverse variance weighted	3	0.060	0.182	7.425E-01	0.986	pan	Hospitalized COVID-19
19110_6	MEMO1	Protein MEMO1	Q9Y316	MEMO1	NA	Inverse variance weighted	2	0.056	0.172	7.426E-01	0.986	pan	Hospitalized COVID-19
9275_2	Siglec-5	Sialic acid-binding Ig-like lectin 5	O15389	SIGLEC5	NA	Inverse variance weighted	4	-0.066	0.202	7.427E-01	0.986	pan	Hospitalized COVID-19
11351_233	NHEJ1	Non-homologous end-joining factor 1	Q9H9Q4	NHEJ1	NA	Inverse variance weighted	3	0.029	0.088	7.427E-01	0.986	pan	Hospitalized COVID-19
16760_2	IL-26	Interleukin-26	Q9NPH9	IL26	NA	Inverse variance weighted	4	-0.040	0.121	7.431E-01	0.986	pan	Hospitalized COVID-19
3835_11	TLR2	Toll-like receptor 2	O60603	TLR2	NA	Wald ratio	1	0.105	0.322	7.433E-01	0.986	pan	Hospitalized COVID-19
12786_61	GDE5	Glycerophosphocholine phosphodiesterase GPCPD1	Q9NPB8	GPCPD1	NA	Inverse variance weighted	2	-0.037	0.112	7.436E-01	0.986	pan	Hospitalized COVID-19
14079_14	IL-18 Ra	Interleukin-18 receptor 1	Q13478	IL18R1	NA	Inverse variance weighted	10	-0.015	0.045	7.457E-01	0.987	pan	Hospitalized COVID-19
17515_6	STCH	Heat shock 70 kDa protein 13	P48723	HSPA13	NA	Inverse variance weighted	2	0.050	0.154	7.458E-01	0.987	pan	Hospitalized COVID-19
19135_5	Cytosolic 5'-nucleotidase III-like protein	7-methylguanosine phosphate-specific 5'-nucleotidase	Q969T7	NT5C3B	NA	Inverse variance weighted	13	-0.008	0.026	7.463E-01	0.987	pan	Hospitalized COVID-19
17837_5	CLIC2	Chloride intracellular channel protein 2	O15247	CLIC2	NA	Wald ratio	1	0.069	0.215	7.464E-01	0.987	pan	Hospitalized COVID-19
8888_33	CX068	Small integral membrane protein 9	A6NGZ8	SMIM9	NA	Inverse variance weighted	2	0.019	0.059	7.476E-01	0.988	pan	Hospitalized COVID-19
18840_205	SH21B	SH2 domain-containing protein 1B	O14796	SH2D1B	NA	Wald ratio	1	-0.079	0.248	7.486E-01	0.988	pan	Hospitalized COVID-19

13094_75	RSPO3	R-spondin-3	Q9BXY4	RSPO3	NA	Inverse variance weighted	2	0.048	0.151	7.487E-01	0.988	pan	Hospitalized COVID-19
6584_1	SRCH	Sarcoplasmic reticulum histidine-rich calcium-binding protein	P23327	HRC	NA	Wald ratio	1	-0.068	0.212	7.488E-01	0.988	pan	Hospitalized COVID-19
15587_20	FOLR2	Folate receptor beta	P14207	FOLR2	NA	Inverse variance weighted	3	0.053	0.166	7.500E-01	0.988	pan	Hospitalized COVID-19
2797_56	Apo B	Apolipoprotein B	P04114	APOB	NA	Wald ratio	1	-0.066	0.208	7.502E-01	0.988	pan	Hospitalized COVID-19
12662_82	ECH1	Delta(3,5)-Delta(2,4)-dienoyl-CoA isomerase, mitochondrial	Q13011	ECH1	NA	Inverse variance weighted	2	0.022	0.069	7.507E-01	0.988	pan	Hospitalized COVID-19
10908_2	GLT13	Polypeptide N-acetylgalactosaminyltransferase 13	Q8IUC8	GALNT13	NA	Wald ratio	1	0.061	0.191	7.509E-01	0.988	pan	Hospitalized COVID-19
4929_55	SHBG	Sex hormone-binding globulin	P04278	SHBG	NA	Inverse variance weighted	5	0.026	0.082	7.514E-01	0.988	pan	Hospitalized COVID-19
17175_5	MP2K6	Dual specificity mitogen-activated protein kinase kinase 6	P52564	MAP2K6	NA	Inverse variance weighted	2	0.049	0.155	7.523E-01	0.988	pan	Hospitalized COVID-19
8842_16	GRM1C	GRAM domain-containing protein 1C	Q8IYS0	GRAMD1C	NA	Inverse variance weighted	9	0.017	0.053	7.526E-01	0.988	pan	Hospitalized COVID-19
19615_213	5NT3	Cytosolic 5'-nucleotidase 3A	Q9H0P0	NT5C3A	NA	Wald ratio	1	0.048	0.153	7.537E-01	0.988	pan	Hospitalized COVID-19
4127_75	C6	Complement component C6	P13671	C6	NA	Inverse variance weighted	2	-0.025	0.078	7.542E-01	0.988	pan	Hospitalized COVID-19
5939_42	TWEAK	Tumor necrosis factor ligand superfamily member 12	O43508	TNFSF12	NA	Inverse variance weighted	3	-0.027	0.088	7.543E-01	0.988	pan	Hospitalized COVID-19
11530_37	HEM3	Porphobilinogen deaminase	P08397	HMBS	NA	Wald ratio	1	-0.062	0.197	7.545E-01	0.988	pan	Hospitalized COVID-19
9772_153	NLGN2:ECD	Neuroligin-2:Extracellular domain	Q8NFZ4	NLGN2	NA	Inverse variance weighted	2	0.026	0.083	7.546E-01	0.988	pan	Hospitalized COVID-19
19377_14	NOE2	Noelin-2	O95897	OLFM2	NA	Inverse variance weighted	8	0.007	0.023	7.592E-01	0.994	pan	Hospitalized COVID-19
18413_24	ARL4D	ADP-ribosylation factor-like protein 4D	P49703	ARL4D	NA	Wald ratio	1	0.058	0.189	7.600E-01	0.994	pan	Hospitalized COVID-19
18897_31	HDAC2	Histone deacetylase 2	Q92769	HDAC2	NA	Wald ratio	1	-0.088	0.288	7.610E-01	0.995	pan	Hospitalized COVID-19
5744_12	CA056	Protein MENT	Q9BUN1	MENT	NA	Inverse variance weighted	4	-0.039	0.127	7.613E-01	0.995	pan	Hospitalized COVID-19
6255_74	CPXM1	Probable carboxypeptidase X1	Q96SM3	CPXM1	NA	Inverse variance weighted	5	-0.012	0.038	7.628E-01	0.996	pan	Hospitalized COVID-19
9383_24	CH3L2	Chitinase-3-like protein 2	Q15782	CHI3L2	NA	Wald ratio	1	0.052	0.172	7.635E-01	0.996	pan	Hospitalized COVID-19
10557_6	TEX29	Testis-expressed sequence 29 protein	Q8N6K0	TEX29	NA	Inverse variance weighted	4	-0.026	0.088	7.645E-01	0.997	pan	Hospitalized COVID-19
15494_11	FGFP1	Fibroblast growth factor-binding protein 1	Q14512	FGFBP1	NA	Inverse variance weighted	2	-0.043	0.144	7.663E-01	0.997	pan	Hospitalized COVID-19
8958_51	CHL1	Neural cell adhesion molecule L1-like protein	O00533	CHL1	NA	Inverse variance weighted	8	0.016	0.055	7.663E-01	0.997	pan	Hospitalized COVID-19
6289_78	RHG36	Rho GTPase-activating protein 36	Q6ZRI8	ARHGAP36	NA	Wald ratio	1	-0.057	0.193	7.672E-01	0.997	pan	Hospitalized COVID-19
12594_5	GRAN	Grancalcin	P28676	GCA	NA	Inverse variance weighted	2	-0.010	0.035	7.678E-01	0.997	pan	Hospitalized COVID-19
17751_68	CRBB1	Beta-crystallin B1	P53674	CRYBB1	NA	Inverse variance weighted	3	0.015	0.052	7.682E-01	0.997	pan	Hospitalized COVID-19
5680_54	OBP2B	Odorant-binding protein 2b	Q9NPH6	OBP2B	NA	Inverse variance weighted	6	0.009	0.030	7.707E-01	0.997	pan	Hospitalized COVID-19
9226_6	TMUB2	Transmembrane and ubiquitin-like domain-containing protein 2	Q71RG4	TMUB2	NA	Wald ratio	1	-0.070	0.241	7.709E-01	0.997	pan	Hospitalized COVID-19
5076_53	EPHAA	Ephrin type-A receptor 10	Q5JZY3	EPHA10	NA	Wald ratio	1	-0.042	0.145	7.711E-01	0.997	pan	Hospitalized COVID-19
8243_55	TATI	Serine protease inhibitor Kazal-type 1	P00995	SPINK1	NA	Inverse variance weighted	2	0.032	0.111	7.714E-01	0.997	pan	Hospitalized COVID-19
8017_23	FXRD1	FAD-dependent oxidoreductase domain-containing protein 1	Q96CU9	FOXRED1	NA	Wald ratio	1	-0.069	0.238	7.715E-01	0.997	pan	Hospitalized COVID-19
11215_6	CAD15:CD	Cadherin-15:Cytoplasmic domain	P55291	CDH15	NA	Inverse variance weighted	2	0.046	0.159	7.716E-01	0.997	pan	Hospitalized COVID-19
8969_49	CD14	Monocyte differentiation antigen CD14	P08571	CD14	NA	Wald ratio	1	-0.077	0.265	7.721E-01	0.997	pan	Hospitalized COVID-19
7141_21	MGT4B	Alpha-1,3-mannosyl-glycoprotein 4-beta-N-acetylglucosaminyltransferase B	Q9UQ53	MGAT4B	NA	Inverse variance weighted	4	-0.010	0.033	7.724E-01	0.997	pan	Hospitalized COVID-19
7266_4	SPA9	Serpin A9	Q86WD7	SERPINA9	NA	Wald ratio	1	0.040	0.139	7.727E-01	0.997	pan	Hospitalized COVID-19
4469_78	ST4S6	Carbohydrate sulfotransferase 15	Q7LFX5	CHST15	NA	Inverse variance weighted	3	0.023	0.080	7.733E-01	0.997	pan	Hospitalized COVID-19
17408_2	PMM1	Phosphomannomutase 1	Q92871	PMM1	NA	Wald ratio	1	0.057	0.197	7.745E-01	0.997	pan	Hospitalized COVID-19
10045_47	CUL3	Cullin-3	Q13618	CUL3	NA	Wald ratio	1	-0.034	0.119	7.747E-01	0.997	pan	Hospitalized COVID-19
16583_8	AP4A	Bis(5'-nucleosyl)-tetraphosphatase [asymmetrical]	P50583	NUDT2	NA	Inverse variance weighted	8	0.016	0.055	7.751E-01	0.997	pan	Hospitalized COVID-19
3367_8	FETUB	Fetuin-B	Q9UGM5	FETUB	NA	Inverse variance weighted	7	-0.015	0.053	7.759E-01	0.997	pan	Hospitalized COVID-19
11288_26	5NTC	Cytosolic purine 5'-nucleotidase	P49902	NT5C2	NA	Inverse variance weighted	3	0.027	0.095	7.760E-01	0.997	pan	Hospitalized COVID-19
12657_2	FCL	GDP-L-fucose synthase	Q13630	GFUS	NA	Wald ratio	1	0.069	0.243	7.761E-01	0.997	pan	Hospitalized COVID-19
18299_13	CPNS1	Calpain small subunit 1	P04632	CAPNS1	NA	Inverse variance weighted	4	-0.019	0.066	7.766E-01	0.997	pan	Hospitalized COVID-19
6918_183	CCKN	Cholecystokinin	P06307	CCK	NA	Inverse variance weighted	2	0.050	0.177	7.768E-01	0.997	pan	Hospitalized COVID-19
4209_60	DRG-1	Vacuolar protein sorting-associated protein VTA1 homolog	Q9NP79	VTA1	NA	Wald ratio	1	0.054	0.192	7.773E-01	0.997	pan	Hospitalized COVID-19
3322_52	LRIG3	Leucine-rich repeats and immunoglobulin-like domains protein 3	Q6UXM1	LRIG3	NA	Inverse variance weighted	9	-0.009	0.033	7.776E-01	0.997	pan	Hospitalized COVID-19
2844_53	sTie-1	Tyrosine-protein kinase receptor Tie-1, soluble	P35590	TIE1	NA	Inverse variance weighted	16	0.009	0.033	7.779E-01	0.997	pan	Hospitalized COVID-19
19130_81	SPB8	Serpin B8	P50452	SERPINB8	NA	Inverse variance weighted	3	-0.013	0.047	7.787E-01	0.997	pan	Hospitalized COVID-19
9061_3	ARHGA	Rho guanine nucleotide exchange factor 10	O15013	ARHGEF10	NA	Wald ratio	1	0.039	0.141	7.796E-01	0.997	pan	Hospitalized COVID-19
10462_14	INSL5	Insulin-like peptide INSL5	Q9Y5Q6	INSL5	NA	Wald ratio	1	-0.051	0.181	7.797E-01	0.997	pan	Hospitalized COVID-19
16825_20	ATX3	Ataxin-3	P54252	ATXN3	NA	Inverse variance weighted	4	0.017	0.063	7.806E-01	0.997	pan	Hospitalized COVID-19
11615_16	DAPP1	Dual adapter for phosphotyrosine and 3-phosphotyrosine and 3-phosphoinositide	Q9UN19	DAPP1	NA	Inverse variance weighted	2	-0.043	0.154	7.814E-01	0.997	pan	Hospitalized COVID-19
3485_28	b2-Microglobulin	Beta-2-microglobulin	P61769	B2M	NA	Inverse variance weighted	2	0.034	0.122	7.830E-01	0.997	pan	Hospitalized COVID-19
5242_37	MP2K4	Dual specificity mitogen-activated protein kinase kinase 4	P45985	MAP2K4	NA	Inverse variance weighted	6	-0.042	0.152	7.836E-01	0.997	pan	Hospitalized COVID-19
5763_67	HBD-4	Beta-defensin 104	Q8WTQ1	DEFB104A	NA	Wald ratio	1	-0.076	0.278	7.837E-01	0.997	pan	Hospitalized COVID-19
13720_95	Proteinase-3	Myeloblastin	P24158	PRTN3	NA	Inverse variance weighted	10	-0.007	0.025	7.840E-01	0.997	pan	Hospitalized COVID-19
7999_23	CD39	Ectonucleoside triphosphate diphosphohydrolase 1	P49961	ENTPD1	NA	Inverse variance weighted	3	0.023	0.085	7.854E-01	0.997	pan	Hospitalized COVID-19
7049_2	ADAM 23	Disintegrin and metalloproteinase domain-containing protein 23	O75077	ADAM23	NA	Inverse variance weighted	13	-0.006	0.021	7.855E-01	0.997	pan	Hospitalized COVID-19
5852_6	S100A12	Protein S100-A12	P80511	S100A12	NA	Inverse variance weighted	2	-0.023	0.085	7.855E-01	0.997	pan	Hospitalized COVID-19
7099_33	FSTL5	Follistatin-related protein 5	Q8N475	FSTL5	NA	Wald ratio	1	0.028	0.105	7.862E-01	0.997	pan	Hospitalized COVID-19
15582_25	FCN1	Ficolin-1	O00602	FCN1	NA	Inverse variance weighted	4	0.021	0.077	7.866E-01	0.997	pan	Hospitalized COVID-19
9369_174	NGL1	Leucine-rich repeat-containing protein 4C	Q9HCJ2	LRRC4C	NA	Inverse variance weighted	4	-0.020	0.075	7.875E-01	0.997	pan	Hospitalized COVID-19
3554_24	Adiponectin	Adiponectin	Q15848	ADIPOQ	NA	Inverse variance weighted	15	-0.011	0.042	7.879E-01	0.997	pan	Hospitalized COVID-19
11229_16	ERN1	Serine/threonine-protein kinase/endoribonuclease IRE1	O75460	ERN1	NA	Wald ratio	1	0.077	0.287	7.883E-01	0.997	pan	Hospitalized COVID-19
13704_5	HMCS2	Hydroxymethylglutaryl-CoA synthase, mitochondrial	P54868	HMGCS2	NA	Inverse variance weighted	2	0.038	0.142	7.886E-01	0.997	pan	Hospitalized COVID-19
10565_19	SLIK3	SLIT and NTRK-like protein 3	O94933	SLITRK3	NA	Inverse variance weighted	15	0.008	0.028	7.888E-01	0.997	pan	Hospitalized COVID-19
2381_52	C5	Complement C5	P01031	C5	NA	Inverse variance weighted	2	0.041	0.154	7.907E-01	0.997	pan	Hospitalized COVID-19
2677_1	ERBB1	Epidermal growth factor receptor	P00533	EGFR	NA	Inverse variance weighted	5	0.017	0.063	7.908E-01	0.997	pan	Hospitalized COVID-19
9294_45	MFAP2	Microfibrillar-associated protein 2	P55001	MFAP2	NA	Inverse variance weighted	2	-0.045	0.169	7.909E-01	0.997	pan	Hospitalized COVID-19
18280_29	DTD2	Putative D-tyrosyl-tRNA(Tyr) deacylase 2	Q96FN9	DTD2	NA	Inverse variance weighted	2	-0.023	0.088	7.916E-01	0.997	pan	Hospitalized COVID-19
9969_8	S22AG	Solute carrier family 22 member 16	Q86VW1	SLC22A16	NA	Inverse variance weighted	3	0.036	0.135	7.921E-01	0.997	pan	Hospitalized COVID-19
17712_7	IDI1	Isopentenyl-diphosphate Delta-isomerase 1	Q13907	IDI1	NA	Wald ratio	1	-0.039	0.147	7.921E-01	0.997	pan	Hospitalized COVID-19
5737_61	SEM4D	Semaphorin-4D	Q92854	SEMA4D	NA	Inverse variance weighted	8	-0.009	0.036	7.925E-01	0.997	pan	Hospitalized COVID-19
3313_21	FCN2	Ficolin-2	Q15485	FCN2	NA	Inverse variance weighted	3	0.018	0.068	7.928E-01	0.997	pan	Hospitalized COVID-19
19639_53	IAPP	Islet amyloid polypeptide	P10997	IAPP	NA	Inverse variance weighted	9	0.015	0.057	7.928E-01	0.997	pan	Hospitalized COVID-19
6247_9	SIRB1	Signal-regulatory protein beta-1	O00241	SIRPB1	NA	Inverse variance weighted	9	0.004	0.014	7.940E-01	0.997	pan	Hospitalized COVID-19
4355_13	DAPK2	Death-associated protein kinase 2	Q9UIK4	DAPK2	NA	Inverse variance weighted	5	0.025	0.095	7.940E-01	0.997	pan	Hospitalized COVID-19
6382_17	MANBA	Beta-mannosidase	O00462	MANBA	NA	Inverse variance weighted	8	0.008	0.031	7.940E-01	0.997	pan	Hospitalized COVID-19

9118_7	PSD2	PH and SEC7 domain-containing protein 2	Q9BQI7	PSD2	NA	Inverse variance weighted	4	-0.031	0.121	7.958E-01	0.997	pan	Hospitalized COVID-19
15514_26	Pseudocholinesterase	Cholinesterase	P06276	BCHE	NA	Inverse variance weighted	15	0.012	0.048	7.958E-01	0.997	pan	Hospitalized COVID-19
3043_49	ON	SPARC	P09486	SPARC	NA	Inverse variance weighted	2	0.034	0.130	7.962E-01	0.997	pan	Hospitalized COVID-19
7886_26	SPTC1	Serine palmitoyltransferase 1	O15269	SPTLC1	NA	Inverse variance weighted	2	-0.033	0.126	7.962E-01	0.997	pan	Hospitalized COVID-19
3046_31	resistin	Resistin	Q9HD89	RETN	NA	Inverse variance weighted	16	0.008	0.032	7.965E-01	0.997	pan	Hospitalized COVID-19
17137_160	Beta-globin	Hemoglobin subunit beta	P68871	HBB	NA	Inverse variance weighted	2	0.065	0.251	7.972E-01	0.997	pan	Hospitalized COVID-19
2888_49	C7	Complement component C7	P10643	C7	NA	Inverse variance weighted	14	-0.005	0.018	7.976E-01	0.997	pan	Hospitalized COVID-19
19213_1	ISK4	Serine protease inhibitor Kazal-type 4	O60575	SPINK4	NA	Wald ratio	1	-0.013	0.050	7.979E-01	0.997	pan	Hospitalized COVID-19
13575_40	DVL2	Segment polarity protein dishevelled homolog DVL-2	O14641	DVL2	NA	Wald ratio	1	0.064	0.249	7.981E-01	0.997	pan	Hospitalized COVID-19
18925_24	Proteasome subunit alpha type 5	Proteasome subunit alpha type-5	P28066	PSMA5	NA	Wald ratio	1	-0.065	0.254	7.989E-01	0.997	pan	Hospitalized COVID-19
11178_21	SVEP1:EGF-like domains 4-6	Sushi, von Willebrand factor type A, EGF and pentraxin domain-containing protein 1:EGF-like domains 4-6	Q4LDE5	SVEP1	NA	Inverse variance weighted	5	0.032	0.126	7.990E-01	0.997	pan	Hospitalized COVID-19
7986_98	NALD2	N-acetylated-alpha-linked acidic dipeptidase 2	Q9Y3Q0	NAALAD2	NA	Inverse variance weighted	10	0.007	0.029	7.993E-01	0.997	pan	Hospitalized COVID-19
8794_13	DPEP1	Dipeptidase 1	P16444	DPEP1	NA	Inverse variance weighted	7	0.009	0.034	8.005E-01	0.997	pan	Hospitalized COVID-19
10800_15	Collagen-binding protein	Serpin H1	P50454	SERPINH1	NA	Inverse variance weighted	4	-0.018	0.071	8.005E-01	0.997	pan	Hospitalized COVID-19
3796_79	ANGL4	Angiopoietin-related protein 4	Q9BY76	ANGPTL4	NA	Wald ratio	1	0.046	0.181	8.009E-01	0.997	pan	Hospitalized COVID-19
2654_19	TNF sR-I	Tumor necrosis factor receptor superfamily member 1A	P19438	TNFRSF1A	NA	Wald ratio	1	0.023	0.090	8.013E-01	0.997	pan	Hospitalized COVID-19
16060_99	NID2	Nidogen-2	Q14112	NID2	NA	Inverse variance weighted	9	-0.007	0.027	8.014E-01	0.997	pan	Hospitalized COVID-19
11102_22	REG4	Regenerating islet-derived protein 4	Q9BYZ8	REG4	NA	Inverse variance weighted	2	0.034	0.134	8.016E-01	0.997	pan	Hospitalized COVID-19
2615_60	Ephrin-A5	Ephrin-A5	P52803	EFNA5	NA	Inverse variance weighted	3	0.027	0.106	8.018E-01	0.997	pan	Hospitalized COVID-19
5034_79	Trypsin 2	Trypsin-2	P07478	PRSS2	NA	Inverse variance weighted	13	-0.012	0.050	8.042E-01	0.997	pan	Hospitalized COVID-19
9263_57	SIAE	Sialate O-acetylesterase	Q9HAT2	SIAE	NA	Inverse variance weighted	3	0.020	0.080	8.044E-01	0.997	pan	Hospitalized COVID-19
2855_49	ERK-1	Mitogen-activated protein kinase 3	P27361	MAPK3	NA	Inverse variance weighted	2	0.015	0.060	8.044E-01	0.997	pan	Hospitalized COVID-19
4866_59	TrkB	BDNF/NT-3 growth factors receptor	Q16620	NTRK2	NA	Inverse variance weighted	2	0.080	0.324	8.047E-01	0.997	pan	Hospitalized COVID-19
15395_15	GST M1-1	Glutathione S-transferase Mu 1	P09488	GSTM1	NA	Inverse variance weighted	7	-0.017	0.069	8.061E-01	0.997	pan	Hospitalized COVID-19
18387_7	suppression of tumorigenicity 13	Hsc70-interacting protein	P50502	ST13	NA	Wald ratio	1	-0.047	0.194	8.071E-01	0.997	pan	Hospitalized COVID-19
5532_53	bFGF-R	Fibroblast growth factor receptor 1	P11362	FGFR1	NA	Inverse variance weighted	5	0.027	0.111	8.071E-01	0.997	pan	Hospitalized COVID-19
9449_150	C4b-binding protein alpha chain	C4b-binding protein alpha chain	P04003	C4BPA	NA	Inverse variance weighted	5	-0.017	0.071	8.071E-01	0.997	pan	Hospitalized COVID-19
19230_12	GSTT1	Glutathione S-transferase theta-1	P30711	GSTT1	NA	Inverse variance weighted	11	0.008	0.034	8.074E-01	0.997	pan	Hospitalized COVID-19
10835_25	A4GCT	Alpha-1,4-N-acetylglucosaminyltransferase	Q9UNA3	A4GNT	NA	Wald ratio	1	-0.038	0.157	8.076E-01	0.997	pan	Hospitalized COVID-19
10816_150	PILRA isoform FDF03-M14	Paired immunoglobulin-like type 2 receptor alpha isoform FDF03-M14	Q9UKJ1	PILRA	NA	Inverse variance weighted	9	-0.018	0.073	8.077E-01	0.997	pan	Hospitalized COVID-19
17403_14	acyl-Coenzyme A dehydrogenase	Short/branched chain specific acyl-CoA dehydrogenase, mitochondrial	P45954	ACADSB	NA	Inverse variance weighted	4	0.033	0.134	8.078E-01	0.997	pan	Hospitalized COVID-19
11573_3	SRSF6	Serine/arginine-rich splicing factor 6	Q13247	SRSF6	NA	Wald ratio	1	0.048	0.197	8.078E-01	0.997	pan	Hospitalized COVID-19
7871_16	T132A	Transmembrane protein 132A	Q24JP5	TMEM132A	NA	Inverse variance weighted	9	0.006	0.024	8.083E-01	0.997	pan	Hospitalized COVID-19
13427_66	MA1C1	Mannosyl-oligosaccharide 1,2-alpha-mannosidase IC	Q9NR34	MAN1C1	NA	Inverse variance weighted	4	-0.022	0.090	8.090E-01	0.997	pan	Hospitalized COVID-19
4562_1	NXPH1	Neurexophilin-1	P58417	NXPH1	NA	Wald ratio	1	0.060	0.251	8.100E-01	0.997	pan	Hospitalized COVID-19
4964_67	ARTS1	Endoplasmic reticulum aminopeptidase 1	Q9NZ08	ERAP1	NA	Inverse variance weighted	21	-0.006	0.024	8.102E-01	0.997	pan	Hospitalized COVID-19
2946_52	Factor D	Complement factor D	P00746	CFD	NA	Inverse variance weighted	5	-0.020	0.082	8.108E-01	0.997	pan	Hospitalized COVID-19
9360_33	EDIL3	EGF-like repeat and discoidin I-like domain-containing protein 3	O43854	EDIL3	NA	Inverse variance weighted	3	0.026	0.108	8.110E-01	0.997	pan	Hospitalized COVID-19
16926_44	Alkaline phosphatase, liver	Alkaline phosphatase, tissue-nonspecific isozyme	P05186	ALPL	NA	Inverse variance weighted	3	-0.031	0.131	8.124E-01	0.997	pan	Hospitalized COVID-19
9380_2	sPLA(2)-XIII	Group XIIB secretory phospholipase A2-like protein	Q9BX93	PLA2G12B	NA	Inverse variance weighted	2	-0.030	0.126	8.126E-01	0.997	pan	Hospitalized COVID-19
12573_80	TRIM3	Tripartite motif-containing protein 3	O75382	TRIM3	NA	Wald ratio	1	0.039	0.166	8.131E-01	0.997	pan	Hospitalized COVID-19
15499_11	Attractin	Attractin	O75882	ATRN	NA	Inverse variance weighted	5	-0.014	0.061	8.167E-01	0.997	pan	Hospitalized COVID-19
2632_5	IL-12 Rb1	Interleukin-12 receptor subunit beta-1	P42701	IL12RB1	NA	Wald ratio	1	-0.030	0.130	8.169E-01	0.997	pan	Hospitalized COVID-19
18225_13	HEBP1	Heme-binding protein 1	Q9NRV9	HEBP1	NA	Inverse variance weighted	10	0.004	0.018	8.180E-01	0.997	pan	Hospitalized COVID-19
19251_56	Platelet proteoglycan	Serglycin	P10124	SRGN	NA	Inverse variance weighted	7	0.009	0.041	8.182E-01	0.997	pan	Hospitalized COVID-19
5803_24	C3d	Complement C3d fragment	P01024	C3	NA	Wald ratio	1	0.041	0.178	8.182E-01	0.997	pan	Hospitalized COVID-19
13392_13	AT1B1	Sodium/potassium-transporting ATPase subunit beta-1	P05026	ATP1B1	NA	Inverse variance weighted	5	-0.012	0.053	8.186E-01	0.997	pan	Hospitalized COVID-19
10613_33	CASC4	Protein CASC4	Q6P4E1	GOLM2	NA	Inverse variance weighted	6	0.020	0.087	8.194E-01	0.997	pan	Hospitalized COVID-19
8479_4	MMP-10	Stromelysin-2	P09238	MMP10	NA	Inverse variance weighted	9	-0.010	0.042	8.195E-01	0.997	pan	Hospitalized COVID-19
3195_50	Granulysin	Granulysin	P22749	GNLY	NA	Inverse variance weighted	17	0.006	0.026	8.196E-01	0.997	pan	Hospitalized COVID-19
9177_6	FAM3B	Protein FAM3B	P58499	FAM3B	NA	Inverse variance weighted	7	0.009	0.040	8.199E-01	0.997	pan	Hospitalized COVID-19
9237_54	Lysosomal acid phosphatase	Lysosomal acid phosphatase	P11117	ACP2	NA	Inverse variance weighted	3	-0.032	0.143	8.207E-01	0.997	pan	Hospitalized COVID-19
5030_52	SIRT2	NAD-dependent protein deacetylase sirtuin-2	Q8IXJ6	SIRT2	NA	Inverse variance weighted	6	0.018	0.080	8.212E-01	0.997	pan	Hospitalized COVID-19
18214_2	GSH0	Glutamate--cysteine ligase regulatory subunit	P48507	GCLM	NA	Inverse variance weighted	2	0.027	0.121	8.231E-01	0.997	pan	Hospitalized COVID-19
9394_19	Aminopeptidase	Carboxypeptidase Q	Q9Y646	CPQ	NA	Inverse variance weighted	12	0.006	0.026	8.234E-01	0.997	pan	Hospitalized COVID-19
10612_18	PLOD3	Procollagen-lysine,2-oxoglutarate 5-dioxygenase 3	O60568	PLOD3	NA	Inverse variance weighted	7	-0.016	0.071	8.234E-01	0.997	pan	Hospitalized COVID-19
8013_9	LMA2L	VIP36-like protein	Q9H0V9	LMAN2L	NA	Inverse variance weighted	2	-0.034	0.152	8.236E-01	0.997	pan	Hospitalized COVID-19
16857_2	RAB2A	Ras-related protein Rab-2A	P61019	RAB2A	NA	Wald ratio	1	0.027	0.120	8.238E-01	0.997	pan	Hospitalized COVID-19
13109_82	NEGR1	Neuronal growth regulator 1	Q7Z3B1	NEGR1	NA	Inverse variance weighted	2	0.041	0.185	8.238E-01	0.997	pan	Hospitalized COVID-19
9883_29	Glyoxalase I	Lactoylglutathione lyase	Q04760	GLO1	NA	Inverse variance weighted	2	-0.020	0.088	8.239E-01	0.997	pan	Hospitalized COVID-19
3628_3	MP2K2	Dual specificity mitogen-activated protein kinase kinase 2	P36507	MAP2K2	NA	Inverse variance weighted	6	-0.017	0.079	8.253E-01	0.997	pan	Hospitalized COVID-19
9839_148	Tirap	Toll/interleukin-1 receptor domain-containing adapter protein	P58753	TIRAP	NA	Inverse variance weighted	3	0.033	0.150	8.256E-01	0.997	pan	Hospitalized COVID-19
9525_1	PTK7	Inactive tyrosine-protein kinase 7	Q13308	PTK7	NA	Inverse variance weighted	4	0.037	0.171	8.265E-01	0.997	pan	Hospitalized COVID-19
19581_15	IGFBP-5	Insulin-like growth factor-binding protein 5	P24593	IGFBP5	NA	Wald ratio	1	-0.044	0.201	8.265E-01	0.997	pan	Hospitalized COVID-19
8356_88	NEU1	Oxytocin-neurophysin 1	P01178	OXT	NA	Inverse variance weighted	4	-0.014	0.064	8.266E-01	0.997	pan	Hospitalized COVID-19
9233_71	TFPI -2	Tissue factor pathway inhibitor 2	P48307	TFPI2	NA	Inverse variance weighted	2	-0.048	0.218	8.277E-01	0.997	pan	Hospitalized COVID-19
4968_50	CAPG	Macrophage-capping protein	P40121	CAPG	NA	Inverse variance weighted	8	0.007	0.032	8.278E-01	0.997	pan	Hospitalized COVID-19
7787_25	LIRA5	Leukocyte immunoglobulin-like receptor subfamily A member 5	A6NI73	LILRA5	NA	Wald ratio	1	0.044	0.205	8.288E-01	0.997	pan	Hospitalized COVID-19
3440_7	granzyme A	Granzyme A	P12544	GZMA	NA	Inverse variance weighted	8	0.008	0.040	8.306E-01	0.997	pan	Hospitalized COVID-19
5089_11	IL-7 Ra	Interleukin-7 receptor subunit alpha	P16871	IL7R	NA	Wald ratio	1	-0.026	0.122	8.306E-01	0.997	pan	Hospitalized COVID-19
8250_2	PTPRJ	Receptor-type tyrosine-protein phosphatase eta	Q12913	PTPRJ	NA	Inverse variance weighted	8	-0.014	0.068	8.310E-01	0.997	pan	Hospitalized COVID-19
5483_1	RGMA	Repulsive guidance molecule A	Q96B86	RGMA	NA	Inverse variance weighted	8	-0.016	0.074	8.321E-01	0.997	pan	Hospitalized COVID-19
3122_6	SMAC	Diablo homolog, mitochondrial	Q9NR28	DIABLO	NA	Wald ratio	1	0.045	0.210	8.323E-01	0.997	pan	Hospitalized COVID-19
6368_9	CST1L	Cystatin-like 1	Q9H114	CSTL1	NA	Wald ratio	1	-0.050	0.239	8.339E-01	0.997	pan	Hospitalized COVID-19
5363_51	Semaphorin 3E	Semaphorin-3E	O15041	SEMA3E	NA	Inverse variance weighted	24	-0.004	0.019	8.339E-01	0.997	pan	Hospitalized COVID-19
15343_337	Kininogen, HMW, Two Chain	Kininogen, HMW, Two Chain	P01042	KNG1	NA	Inverse variance weighted	4	-0.030	0.145	8.342E-01	0.997	pan	Hospitalized COVID-19
8916_32	STIM1:CD	Stromal interaction molecule 1:Cytoplasmic domain	Q13586	STIM1	NA	Wald ratio	1	-0.023	0.110	8.346E-01	0.997	pan	Hospitalized COVID-19
9504_19	RB27A	Ras-related protein Rab-27A	P51159	RAB27A	NA	Wald ratio	1	-0.022	0.106	8.348E-01	0.997	pan	Hospitalized COVID-19

9211_19	PEDF	Pigment epithelium-derived factor	P36955	SERPINF1	NA	Inverse variance weighted	6	0.009	0.045	8.350E-01	0.997	pan	Hospitalized COVID-19
3179_51	Cathepsin A	Lysosomal protective protein	P10619	CTSA	NA	Inverse variance weighted	2	-0.126	0.606	8.350E-01	0.997	pan	Hospitalized COVID-19
11390_24	Carbonic Anhydrase VIII	Carbonic anhydrase-related protein	P35219	CA8	NA	Inverse variance weighted	7	-0.006	0.027	8.352E-01	0.997	pan	Hospitalized COVID-19
17738_7	CRNN	Cornulin	Q9UBG3	CRNN	NA	Wald ratio	1	-0.024	0.114	8.358E-01	0.997	pan	Hospitalized COVID-19
9204_33	Corticotropin-lipotropin	Pro-opiomelanocortin	P01189	POMC	NA	Inverse variance weighted	11	-0.011	0.051	8.359E-01	0.997	pan	Hospitalized COVID-19
4374_45	MIC-1	Growth/differentiation factor 15	Q99988	GDF15	NA	Inverse variance weighted	9	-0.006	0.028	8.381E-01	0.997	pan	Hospitalized COVID-19
17694_32	PSME2	Proteasome activator complex subunit 2	Q9UL46	PSME2	NA	Inverse variance weighted	3	0.012	0.057	8.383E-01	0.997	pan	Hospitalized COVID-19
8398_277	RAR-responsive protein TIG1	Retinoic acid receptor responder protein 1	P49788	RARRES1	NA	Inverse variance weighted	16	-0.002	0.012	8.384E-01	0.997	pan	Hospitalized COVID-19
3216_2	PIGR	Polymeric immunoglobulin receptor	P01833	PIGR	NA	Inverse variance weighted	4	-0.033	0.162	8.386E-01	0.997	pan	Hospitalized COVID-19
17856_23	NT5M	5'(3')-deoxyribonucleotidase, mitochondrial	Q9NPB1	NT5M	NA	Inverse variance weighted	2	-0.020	0.097	8.390E-01	0.997	pan	Hospitalized COVID-19
8697_38	Glypican 1	Glypican-1	P35052	GPC1	NA	Inverse variance weighted	16	-0.005	0.022	8.390E-01	0.997	pan	Hospitalized COVID-19
3592_4	CAMK1	Calcium/calmodulin-dependent protein kinase type 1	Q14012	CAMK1	NA	Inverse variance weighted	11	-0.011	0.056	8.394E-01	0.997	pan	Hospitalized COVID-19
17327_3	CNPY3	Protein canopy homolog 3	Q9BT09	CNPY3	NA	Inverse variance weighted	2	0.031	0.152	8.399E-01	0.997	pan	Hospitalized COVID-19
2985_35	Gro-a	Growth-regulated alpha protein	P09341	CXCL1	NA	Inverse variance weighted	12	0.006	0.029	8.406E-01	0.997	pan	Hospitalized COVID-19
4564_2	PLXC1	Plexin-C1	O60486	PLXNC1	NA	Inverse variance weighted	11	-0.003	0.015	8.408E-01	0.997	pan	Hospitalized COVID-19
9964_10	MYZAP	Myocardial zonula adherens protein	P0CAP1	MYZAP	NA	Inverse variance weighted	2	-0.026	0.129	8.413E-01	0.997	pan	Hospitalized COVID-19
3499_77	IL-17B	Interleukin-17B	Q9UHF5	IL17B	NA	Wald ratio	1	0.028	0.141	8.421E-01	0.997	pan	Hospitalized COVID-19
5798_3	BID	BH3-interacting domain death agonist	P55957	BID	NA	Wald ratio	1	0.027	0.138	8.423E-01	0.997	pan	Hospitalized COVID-19
7210_25	Amyloid-like protein 1	Amyloid-like protein 1	P51693	APLP1	NA	Wald ratio	1	0.060	0.300	8.423E-01	0.997	pan	Hospitalized COVID-19
13553_4	DCNL3	DCN1-like protein 3	Q8IWE4	DCUN1D3	NA	Wald ratio	1	0.048	0.244	8.426E-01	0.997	pan	Hospitalized COVID-19
12381_26	CBR1	Carbonyl reductase [NADPH] 1	P16152	CBR1	NA	Inverse variance weighted	5	0.007	0.033	8.433E-01	0.997	pan	Hospitalized COVID-19
6451_64	ASPN	Asporin	Q9BXN1	ASPN	NA	Inverse variance weighted	8	0.018	0.090	8.435E-01	0.997	pan	Hospitalized COVID-19
11273_176	GSTT2	Glutathione S-transferase theta-2B	P0CG30	GSTT2B	NA	Inverse variance weighted	4	-0.019	0.099	8.439E-01	0.997	pan	Hospitalized COVID-19
8042_88	ISK9	Serine protease inhibitor Kazal-type 9	Q5DT21	SPINK9	NA	Inverse variance weighted	5	-0.014	0.071	8.445E-01	0.997	pan	Hospitalized COVID-19
12663_1	THTR	Thiosulfate sulfurtransferase	Q16762	TST	NA	Inverse variance weighted	15	-0.006	0.031	8.445E-01	0.997	pan	Hospitalized COVID-19
19367_34	D3D2	Enoyl-CoA delta isomerase 1, mitochondrial	P42126	ECI1	NA	Inverse variance weighted	2	-0.045	0.230	8.446E-01	0.997	pan	Hospitalized COVID-19
9981_18	K1467:C-term	Protein FAM234B:C-term	A2RU67	FAM234B	NA	Inverse variance weighted	4	-0.012	0.063	8.448E-01	0.997	pan	Hospitalized COVID-19
11094_104	LPPL	Galectin-10	Q05315	CLC	NA	Wald ratio	1	0.034	0.174	8.453E-01	0.997	pan	Hospitalized COVID-19
5085_18	IL-20 Ra	Interleukin-20 receptor subunit alpha	Q9UHF4	IL20RA	NA	Wald ratio	1	0.041	0.209	8.461E-01	0.997	pan	Hospitalized COVID-19
3474_19	Thrombospondin-1	Thrombospondin-1	P07996	THBS1	NA	Wald ratio	1	0.042	0.215	8.463E-01	0.997	pan	Hospitalized COVID-19
15471_29	LIPR2	Pancreatic lipase-related protein 2	P54317	PNLIPRP2	NA	Inverse variance weighted	14	0.003	0.015	8.469E-01	0.997	pan	Hospitalized COVID-19
10088_37	APT	Adenine phosphoribosyltransferase	P07741	APRT	NA	Inverse variance weighted	7	-0.009	0.046	8.470E-01	0.997	pan	Hospitalized COVID-19
11514_196	CD59	CD59 glycoprotein	P13987	CD59	NA	Inverse variance weighted	3	-0.012	0.062	8.470E-01	0.997	pan	Hospitalized COVID-19
15495_9	FOLR3	Folate receptor gamma	P41439	FOLR3	NA	Inverse variance weighted	15	0.005	0.028	8.479E-01	0.997	pan	Hospitalized COVID-19
19364_163	PCNA	Proliferating cell nuclear antigen	P12004	PCNA	NA	Inverse variance weighted	2	-0.026	0.136	8.481E-01	0.997	pan	Hospitalized COVID-19
3309_2	FCG2A	Low affinity immunoglobulin gamma Fc region receptor II-a	P12318	FCGR2A	NA	Inverse variance weighted	8	0.008	0.044	8.484E-01	0.997	pan	Hospitalized COVID-19
17404_5	ARL5B	ADP-ribosylation factor-like protein 5B	Q96KC2	ARL5B	NA	Wald ratio	1	-0.045	0.233	8.485E-01	0.997	pan	Hospitalized COVID-19
2967_8	VCAM-1	Vascular cell adhesion protein 1	P19320	VCAM1	NA	Inverse variance weighted	3	0.023	0.123	8.485E-01	0.997	pan	Hospitalized COVID-19
4548_4	Fucosyltransferase 3	Galactoside 3(4)-L-fucosyltransferase	P21217	FUT3	NA	Inverse variance weighted	7	-0.010	0.052	8.487E-01	0.997	pan	Hospitalized COVID-19
18243_9	Cytidylate kinase	UMP-CMP kinase	P30085	CMPK1	NA	Wald ratio	1	0.025	0.131	8.490E-01	0.997	pan	Hospitalized COVID-19
3311_27	FCG3B	Low affinity immunoglobulin gamma Fc region receptor III-B	O75015	FCGR3B	NA	Inverse variance weighted	8	0.014	0.075	8.492E-01	0.997	pan	Hospitalized COVID-19
16900_29	MDGA1	MAM domain-containing glycosylphosphatidylinositol anchor protein 1	Q8NFP4	MDGA1	NA	Inverse variance weighted	12	0.005	0.024	8.497E-01	0.997	pan	Hospitalized COVID-19
16892_23	ENPP2	Ectonucleotide pyrophosphatase/phosphodiesterase family member 2	Q13822	ENPP2	NA	Inverse variance weighted	6	-0.012	0.066	8.503E-01	0.997	pan	Hospitalized COVID-19
5066_134	CLM6	CMRF35-like molecule 6	Q08708	CD300C	NA	Inverse variance weighted	13	-0.011	0.060	8.503E-01	0.997	pan	Hospitalized COVID-19
2950_57	IGFBP-4	Insulin-like growth factor-binding protein 4	P22692	IGFBP4	NA	Inverse variance weighted	2	-0.047	0.249	8.506E-01	0.997	pan	Hospitalized COVID-19
6605_17	IGFALS	Insulin-like growth factor-binding protein complex acid labile subunit	P35858	IGFALS	NA	Inverse variance weighted	3	-0.021	0.114	8.507E-01	0.997	pan	Hospitalized COVID-19
2778_10	IL-22	Interleukin-22	Q9GZX6	IL22	NA	Wald ratio	1	0.033	0.177	8.510E-01	0.997	pan	Hospitalized COVID-19
17706_4	PPR1A	Protein phosphatase 1 regulatory subunit 1A	Q13522	PPP1R1A	NA	Wald ratio	1	-0.037	0.196	8.514E-01	0.997	pan	Hospitalized COVID-19
5632_6	CRAC1	Cartilage acidic protein 1	Q9NQ79	CRTAC1	NA	Inverse variance weighted	10	-0.005	0.026	8.523E-01	0.997	pan	Hospitalized COVID-19
9357_4	CREG1	Protein CREG1	O75629	CREG1	NA	Inverse variance weighted	10	-0.004	0.020	8.531E-01	0.997	pan	Hospitalized COVID-19
5704_74	Granzyme M	Granzyme M	P51124	GZMM	NA	Inverse variance weighted	2	0.035	0.191	8.533E-01	0.997	pan	Hospitalized COVID-19
3336_50	TFPI	Tissue factor pathway inhibitor	P10646	TFPI	NA	Wald ratio	1	-0.023	0.126	8.534E-01	0.997	pan	Hospitalized COVID-19
12517_52	TFAR19	Programmed cell death protein 5	O14737	PDCD5	NA	Inverse variance weighted	5	0.011	0.061	8.540E-01	0.997	pan	Hospitalized COVID-19
8006_12	DJB12	DnaJ homolog subfamily B member 12	Q9NXW2	DNAJB12	NA	Inverse variance weighted	2	0.015	0.084	8.541E-01	0.997	pan	Hospitalized COVID-19
10620_21	PSP-94	Beta-microseminoprotein	P08118	MSMB	NA	Inverse variance weighted	19	-0.003	0.015	8.545E-01	0.997	pan	Hospitalized COVID-19
9350_3	FSTL4	Follistatin-related protein 4	Q6MZW2	FSTL4	NA	Inverse variance weighted	2	-0.024	0.129	8.548E-01	0.997	pan	Hospitalized COVID-19
10916_44	PLA2R	Secretory phospholipase A2 receptor	Q13018	PLA2R1	NA	Inverse variance weighted	19	0.005	0.025	8.569E-01	0.997	pan	Hospitalized COVID-19
16607_78	Gelsolin	Gelsolin	P06396	GSN	NA	Inverse variance weighted	9	-0.012	0.068	8.569E-01	0.997	pan	Hospitalized COVID-19
7863_50	PGRC1	Membrane-associated progesterone receptor component 1	O00264	PGRMC1	NA	Wald ratio	1	0.039	0.218	8.571E-01	0.997	pan	Hospitalized COVID-19
16594_44	FAIM1	Fas apoptotic inhibitory molecule 1	Q9NVQ4	FAIM	NA	Inverse variance weighted	10	0.007	0.042	8.591E-01	0.997	pan	Hospitalized COVID-19
5663_18	PF4V	Platelet factor 4 variant	P10720	PF4V1	NA	Inverse variance weighted	2	0.046	0.258	8.597E-01	0.997	pan	Hospitalized COVID-19
15452_5	5'-Nucleotidase	5'-Nucleotidase	P21589	NT5E	NA	Inverse variance weighted	7	-0.006	0.035	8.601E-01	0.997	pan	Hospitalized COVID-19
3605_77	MASP3:Light	Mannan-binding lectin serine protease 1:Mannan-binding lectin serine protease 1 light chain	P48740	MASP1	NA	Wald ratio	1	0.035	0.197	8.606E-01	0.997	pan	Hospitalized COVID-19
2571_12	IGFBP-3	Insulin-like growth factor-binding protein 3	P17936	IGFBP3	NA	Inverse variance weighted	8	-0.006	0.036	8.611E-01	0.997	pan	Hospitalized COVID-19
3898_5	PSD7	26S proteasome non-ATPase regulatory subunit 7	P51665	PSMD7	NA	Wald ratio	1	0.036	0.206	8.612E-01	0.997	pan	Hospitalized COVID-19
2580_83	Myeloperoxidase	Myeloperoxidase	P05164	MPO	NA	Inverse variance weighted	11	-0.013	0.072	8.615E-01	0.997	pan	Hospitalized COVID-19
16908_5	OMGP	Oligodendrocyte-myelin glycoprotein	P23515	OMG	NA	Inverse variance weighted	5	-0.014	0.083	8.620E-01	0.997	pan	Hospitalized COVID-19
16079_2	TEC	Tyrosine-protein kinase Tec	P42680	TEC	NA	Inverse variance weighted	2	0.033	0.192	8.620E-01	0.997	pan	Hospitalized COVID-19
8039_41	F177A	Protein FAM177A1	Q8N128	FAM177A1	NA	Inverse variance weighted	7	0.005	0.029	8.621E-01	0.997	pan	Hospitalized COVID-19
6393_63	Endoplasmin	Endoplasmin	P14625	HSP90B1	NA	Inverse variance weighted	10	0.005	0.031	8.624E-01	0.997	pan	Hospitalized COVID-19
5452_71	ASGR1	Asialoglycoprotein receptor 1	P07306	ASGR1	NA	Wald ratio	1	0.033	0.192	8.626E-01	0.997	pan	Hospitalized COVID-19
15570_99	Complement receptor type 2	Complement receptor type 2	P20023	CR2	NA	Inverse variance weighted	16	-0.006	0.037	8.632E-01	0.997	pan	Hospitalized COVID-19
13717_15	FCN2	Ficolin-2	Q15485	FCN2	NA	Inverse variance weighted	11	0.007	0.041	8.638E-01	0.997	pan	Hospitalized COVID-19
8597_1	TMEM9:CD	Transmembrane protein 9:Cytoplasmic domain	Q9P0T7	TMEM9	NA	Inverse variance weighted	7	-0.007	0.038	8.646E-01	0.997	pan	Hospitalized COVID-19
5604_30	HPSE	Heparanase	Q9Y251	HPSE	NA	Inverse variance weighted	2	0.019	0.114	8.649E-01	0.997	pan	Hospitalized COVID-19
6478_2	IGLO5	IgLON family member 5	A6NGN9	IGLON5	NA	Wald ratio	1	-0.041	0.243	8.657E-01	0.997	pan	Hospitalized COVID-19
2579_17	MMP-9	Matrix metalloproteinase-9	P14780	MMP9	NA	Inverse variance weighted	6	-0.013	0.080	8.662E-01	0.997	pan	Hospitalized COVID-19
4342_10	sICAM-1	Intercellular adhesion molecule 1	P05362	ICAM1	NA	Inverse variance weighted	13	-0.003	0.015	8.664E-01	0.997	pan	Hospitalized COVID-19

9506_10	Apo L1	Apolipoprotein L1	O14791	APOL1	NA	Inverse variance weighted	15	-0.009	0.051	8.672E-01	0.997	pan	Hospitalized COVID-19
4891_50	Glucagon	Glucagon	P01275	GCG	NA	Wald ratio	1	0.035	0.209	8.674E-01	0.997	pan	Hospitalized COVID-19
15431_31	OTCase	Ornithine carbamoyltransferase, mitochondrial	P00480	OTC	NA	Inverse variance weighted	3	0.041	0.244	8.675E-01	0.997	pan	Hospitalized COVID-19
6407_63	LAD1	Ladinin-1	O00515	LAD1	NA	Wald ratio	1	0.054	0.321	8.677E-01	0.997	pan	Hospitalized COVID-19
13465_5	CCP1	Calcipressin-1	P53805	RCAN1	NA	Wald ratio	1	0.028	0.171	8.683E-01	0.997	pan	Hospitalized COVID-19
4834_61	Epithelial cell kinase	Ephrin type-A receptor 2	P29317	EPHA2	NA	Inverse variance weighted	5	-0.009	0.057	8.687E-01	0.997	pan	Hospitalized COVID-19
2819_23	Cadherin-5	Cadherin-5	P33151	CDH5	NA	Inverse variance weighted	5	-0.012	0.070	8.690E-01	0.997	pan	Hospitalized COVID-19
11218_84	TPMT	Thiopurine S-methyltransferase	P51580	TPMT	NA	Wald ratio	1	-0.061	0.370	8.698E-01	0.997	pan	Hospitalized COVID-19
12384_92	CSN7B	COP9 signalosome complex subunit 7b	Q9H9Q2	COPS7B	NA	Inverse variance weighted	4	0.013	0.080	8.706E-01	0.997	pan	Hospitalized COVID-19
8469_41	IGFBP-2	Insulin-like growth factor-binding protein 2	P18065	IGFBP2	NA	Inverse variance weighted	5	-0.017	0.106	8.725E-01	0.997	pan	Hospitalized COVID-19
5689_1	DB112	Beta-defensin 112	Q30KQ8	DEFB112	NA	Wald ratio	1	0.020	0.123	8.729E-01	0.997	pan	Hospitalized COVID-19
8304_50	OPG	Tumor necrosis factor receptor superfamily member 11B	O00300	TNFRSF11B	NA	Inverse variance weighted	3	0.009	0.057	8.731E-01	0.997	pan	Hospitalized COVID-19
4151_6	Plasminogen	Plasminogen	P00747	PLG	NA	Inverse variance weighted	5	0.017	0.109	8.742E-01	0.997	pan	Hospitalized COVID-19
16853_5	NRK1	Nicotinamide riboside kinase 1	Q9NWW6	NMRK1	NA	Inverse variance weighted	2	0.010	0.062	8.743E-01	0.997	pan	Hospitalized COVID-19
3419_49	CAMK2D	Calcium/calmodulin-dependent protein kinase type II subunit delta	Q13557	CAMK2D	NA	Wald ratio	1	0.036	0.231	8.747E-01	0.997	pan	Hospitalized COVID-19
11708_2	LCN1	Lipocalin-1	P31025	LCN1	NA	Wald ratio	1	0.038	0.245	8.750E-01	0.997	pan	Hospitalized COVID-19
2558_51	b-Endorphin	Beta-endorphin	P01189	POMC	NA	Wald ratio	1	0.032	0.204	8.751E-01	0.997	pan	Hospitalized COVID-19
17782_23	THIK	3-ketoacyl-CoA thiolase, peroxisomal	P09110	ACAA1	NA	Inverse variance weighted	8	-0.007	0.047	8.752E-01	0.997	pan	Hospitalized COVID-19
17808_37	NIT2	Omega-amidase NIT2	Q9NQR4	NIT2	NA	Inverse variance weighted	7	-0.012	0.079	8.755E-01	0.997	pan	Hospitalized COVID-19
5581_28	FGL1	Fibrinogen-like protein 1	Q08830	FGL1	NA	Inverse variance weighted	10	-0.004	0.026	8.765E-01	0.997	pan	Hospitalized COVID-19
2705_5	TECK	C-C motif chemokine 25	O15444	CCL25	NA	Inverse variance weighted	11	-0.004	0.023	8.766E-01	0.997	pan	Hospitalized COVID-19
4867_15	VEGF121	Vascular endothelial growth factor A, isoform 121	P15692	VEGFA	NA	Inverse variance weighted	8	0.012	0.080	8.767E-01	0.997	pan	Hospitalized COVID-19
13464_8	NRAC	Nutritionally-regulated adipose and cardiac enriched protein homolog	Q8N912	NRAC	NA	Inverse variance weighted	3	-0.020	0.129	8.771E-01	0.997	pan	Hospitalized COVID-19
12993_21	NPL4	Nuclear protein localization protein 4 homolog	Q8TAT6	NPLOC4	NA	Wald ratio	1	0.051	0.333	8.773E-01	0.997	pan	Hospitalized COVID-19
13399_33	RELL1	RELT-like protein 1	Q8IUW5	RELL1	NA	Inverse variance weighted	2	-0.013	0.086	8.785E-01	0.997	pan	Hospitalized COVID-19
15602_43	IL-6 sRa	Interleukin-6 receptor subunit alpha	P08887	IL6R	NA	Inverse variance weighted	15	-0.004	0.024	8.791E-01	0.997	pan	Hospitalized COVID-19
15325_14	NEUM	Neuromodulin	P17677	GAP43	NA	Wald ratio	1	-0.022	0.149	8.799E-01	0.997	pan	Hospitalized COVID-19
16805_5	PDE5A	cGMP-specific 3',5'-cyclic phosphodiesterase	O76074	PDE5A	NA	Wald ratio	1	-0.034	0.224	8.801E-01	0.997	pan	Hospitalized COVID-19
8233_2	ITIH5	Inter-alpha-trypsin inhibitor heavy chain H5	Q86UX2	ITIH5	NA	Inverse variance weighted	10	-0.005	0.033	8.807E-01	0.997	pan	Hospitalized COVID-19
16324_38	TLR1:ECD	Toll-like receptor 1:Extracellular domain	Q15399	TLR1	NA	Inverse variance weighted	2	0.028	0.185	8.808E-01	0.997	pan	Hospitalized COVID-19
6060_2	PIP	Prolactin-inducible protein	P12273	PIP	NA	Inverse variance weighted	8	0.009	0.060	8.812E-01	0.997	pan	Hospitalized COVID-19
9234_8	TWSG1	Twisted gastrulation protein homolog 1	Q9GZX9	TWSG1	NA	Inverse variance weighted	2	-0.038	0.259	8.824E-01	0.997	pan	Hospitalized COVID-19
15300_66	CC134	Coiled-coil domain-containing protein 134	Q9H6E4	CCDC134	NA	Wald ratio	1	0.014	0.095	8.833E-01	0.997	pan	Hospitalized COVID-19
7223_60	S100A13	Protein S100-A13	Q99584	S100A13	NA	Inverse variance weighted	6	-0.011	0.076	8.834E-01	0.997	pan	Hospitalized COVID-19
13102_1	FAM3D	Protein FAM3D	Q96BQ1	FAM3D	NA	Inverse variance weighted	9	0.004	0.030	8.835E-01	0.997	pan	Hospitalized COVID-19
12812_25	ACYP2	Acylphosphatase-2	P14621	ACYP2	NA	Inverse variance weighted	2	0.007	0.046	8.840E-01	0.997	pan	Hospitalized COVID-19
11257_1	DHPR	Dihydropteridine reductase	P09417	QDPR	NA	Inverse variance weighted	6	-0.004	0.031	8.865E-01	0.997	pan	Hospitalized COVID-19
3320_49	IGFBP-7	Insulin-like growth factor-binding protein 7	Q16270	IGFBP7	NA	Inverse variance weighted	9	0.007	0.048	8.875E-01	0.997	pan	Hospitalized COVID-19
11187_11	CL12A	C-type lectin domain family 12 member A	Q5QGZ9	CLEC12A	NA	Inverse variance weighted	7	0.005	0.036	8.876E-01	0.997	pan	Hospitalized COVID-19
15447_45	Sorbitol dehydrogenase	Sorbitol dehydrogenase	Q00796	SORD	NA	Inverse variance weighted	5	0.006	0.045	8.877E-01	0.997	pan	Hospitalized COVID-19
12697_30	PI42A	Phosphatidylinositol 5-phosphate 4-kinase type-2 alpha	P48426	PIP4K2A	NA	Wald ratio	1	-0.030	0.211	8.879E-01	0.997	pan	Hospitalized COVID-19
10672_75	SP-B	Pulmonary surfactant-associated protein B	P07988	SFTPB	NA	Inverse variance weighted	6	-0.010	0.073	8.890E-01	0.997	pan	Hospitalized COVID-19
19492_5	KAAG1	Kidney-associated antigen 1	Q9UBP8	KAAG1	NA	Inverse variance weighted	14	-0.005	0.036	8.894E-01	0.997	pan	Hospitalized COVID-19
5698_60	Tenascin-X	Tenascin-X	P22105	TNXB	NA	Inverse variance weighted	13	-0.003	0.025	8.895E-01	0.997	pan	Hospitalized COVID-19
19227_18	BATF	Basic leucine zipper transcriptional factor ATF-like	Q16520	BATF	NA	Wald ratio	1	0.031	0.223	8.897E-01	0.997	pan	Hospitalized COVID-19
8098_37	THAS	Thromboxane-A synthase	P24557	TBXAS1	NA	Wald ratio	1	-0.022	0.157	8.902E-01	0.997	pan	Hospitalized COVID-19
3617_80	HGFA	Hepatocyte growth factor activator	Q04756	HGFAC	NA	Inverse variance weighted	14	-0.002	0.018	8.909E-01	0.997	pan	Hospitalized COVID-19
10435_2	ASPG	N(4)-(beta-N-acetylglucosaminyl)-L-asparaginase	P20933	AGA	NA	Inverse variance weighted	2	-0.027	0.198	8.910E-01	0.997	pan	Hospitalized COVID-19
14614_41	CA130	Noncompact myelin-associated protein	Q5T1S8	NCMAP	NA	Inverse variance weighted	12	-0.004	0.030	8.913E-01	0.997	pan	Hospitalized COVID-19
17490_4	SH3BGRL3-like protein	SH3 domain-binding glutamic acid-rich-like protein 3	Q9H299	SH3BGRL3	NA	Inverse variance weighted	4	-0.010	0.070	8.916E-01	0.997	pan	Hospitalized COVID-19
3234_23	URB	Coiled-coil domain-containing protein 80	Q76M96	CCDC80	NA	Inverse variance weighted	4	0.011	0.084	8.923E-01	0.997	pan	Hospitalized COVID-19
17812_2	TP4A1	Protein tyrosine phosphatase type IVA 1	Q93096	PTP4A1	NA	Wald ratio	1	0.028	0.208	8.925E-01	0.997	pan	Hospitalized COVID-19
8458_111	a-Synuclein	Alpha-synuclein	P37840	SNCA	NA	Wald ratio	1	-0.032	0.234	8.925E-01	0.997	pan	Hospitalized COVID-19
12671_35	ST1B1	Sulfotransferase family cytosolic 1B member 1	O43704	SULT1B1	NA	Inverse variance weighted	6	0.010	0.073	8.937E-01	0.997	pan	Hospitalized COVID-19
9545_156	Granzyme K	Granzyme K	P49863	GZMK	NA	Inverse variance weighted	3	0.008	0.064	8.946E-01	0.997	pan	Hospitalized COVID-19
9863_1	Tropomyosin 4	Tropomyosin alpha-4 chain	P67936	TPM4	NA	Wald ratio	1	0.018	0.137	8.956E-01	0.997	pan	Hospitalized COVID-19
13929_27	OCTC	Peroxisomal carnitine O-octanoyltransferase	Q9UKG9	CROT	NA	Inverse variance weighted	9	-0.005	0.040	8.958E-01	0.997	pan	Hospitalized COVID-19
6390_18	NPS	Neuropeptide S	P0C0P6	NPS	NA	Inverse variance weighted	2	0.052	0.399	8.962E-01	0.997	pan	Hospitalized COVID-19
15530_33	EphB4	Ephrin type-B receptor 4	P54760	EPHB4	NA	Inverse variance weighted	4	-0.011	0.083	8.975E-01	0.997	pan	Hospitalized COVID-19
19273_3	Glutathione reductase	Glutathione reductase, mitochondrial	P00390	GSR	NA	Inverse variance weighted	3	0.008	0.061	8.983E-01	0.997	pan	Hospitalized COVID-19
13231_90	IgG4, Kappa	Ig gamma-4, Kappa	P01861	IGHG4	NA	Inverse variance weighted	2	0.018	0.145	8.984E-01	0.997	pan	Hospitalized COVID-19
7875_86	PLEK	Pleckstrin	P08567	PLEK	NA	Inverse variance weighted	2	0.010	0.077	8.984E-01	0.997	pan	Hospitalized COVID-19
19236_24	TCP4	Activated RNA polymerase II transcriptional coactivator p15	P53999	SUB1	NA	Wald ratio	1	-0.028	0.221	8.989E-01	0.997	pan	Hospitalized COVID-19
9102_28	ZFY27:CD region 2	Protrudin:Cytoplasmic domain, region 2, isoform 5	Q5T4F4	ZFYVE27	NA	Wald ratio	1	0.022	0.174	8.998E-01	0.997	pan	Hospitalized COVID-19
6576_1	ART4	Ecto-ADP-ribosyltransferase 4	Q93070	ART4	NA	Inverse variance weighted	10	0.002	0.018	8.998E-01	0.997	pan	Hospitalized COVID-19
10558_26	PCDH9	Protocadherin-9	Q9HC56	PCDH9	NA	Inverse variance weighted	8	-0.004	0.036	9.006E-01	0.997	pan	Hospitalized COVID-19
13566_2	SSU72	RNA polymerase II subunit A C-terminal domain phosphatase SSU72	Q9NP77	SSU72	NA	Inverse variance weighted	6	0.005	0.042	9.011E-01	0.997	pan	Hospitalized COVID-19
6433_57	FA20A	Pseudokinase FAM20A	Q96MK3	FAM20A	NA	Inverse variance weighted	3	-0.014	0.113	9.016E-01	0.997	pan	Hospitalized COVID-19
4535_50	BST1	ADP-ribosyl cyclase/cyclic ADP-ribose hydrolase 2	Q10588	BST1	NA	Inverse variance weighted	17	-0.002	0.015	9.018E-01	0.997	pan	Hospitalized COVID-19
3291_30	CD23	Low affinity immunoglobulin epsilon Fc receptor	P06734	FCER2	NA	Inverse variance weighted	10	0.006	0.047	9.020E-01	0.997	pan	Hospitalized COVID-19
8956_96	SREC-II:ECD	Scavenger receptor class F member 2:Extracellular domain	Q96GP6	SCARF2	NA	Inverse variance weighted	2	-0.019	0.156	9.024E-01	0.997	pan	Hospitalized COVID-19
5610_32	CJ038	Protein FAM171A1	Q5VUB5	FAM171A1	NA	Inverse variance weighted	3	-0.020	0.162	9.025E-01	0.997	pan	Hospitalized COVID-19
9715_15	IGSF3	Immunoglobulin superfamily member 3	O75054	IGSF3	NA	Inverse variance weighted	5	0.010	0.085	9.031E-01	0.997	pan	Hospitalized COVID-19
9513_9	FGF22	Fibroblast growth factor 22	Q9HCT0	FGF22	NA	Wald ratio	1	-0.031	0.251	9.031E-01	0.997	pan	Hospitalized COVID-19
13488_3	ARFP1	Arfaptin-1	P53367	ARFIP1	NA	Inverse variance weighted	2	0.009	0.072	9.039E-01	0.997	pan	Hospitalized COVID-19
15626_223	Perlecan	Basement membrane-specific heparan sulfate proteoglycan core protein	P98160	HSPG2	NA	Inverse variance weighted	6	0.009	0.077	9.047E-01	0.997	pan	Hospitalized COVID-19
11313_100	PHS	Pterin-4-alpha-carbinolamine dehydratase	P61457	PCBD1	NA	Inverse variance weighted	6	-0.006	0.054	9.060E-01	0.997	pan	Hospitalized COVID-19
4840_73	G-CSF	Granulocyte colony-stimulating factor	P09919	CSF3	NA	Wald ratio	1	-0.042	0.362	9.079E-01	0.997	pan	Hospitalized COVID-19

2913_1	MPIF-1	C-C motif chemokine 23	P55773	CCL23	NA	Inverse variance weighted	4	-0.011	0.095	9.081E-01	0.997	pan	Hospitalized COVID-19
16614_27	RSPO1	R-spondin-1	Q2MKA7	RSPO1	NA	Inverse variance weighted	3	-0.005	0.044	9.088E-01	0.997	pan	Hospitalized COVID-19
16858_384	RCN3	Reticulocalbin-3	Q96D15	RCN3	NA	Inverse variance weighted	2	0.020	0.173	9.092E-01	0.997	pan	Hospitalized COVID-19
3795_6	ADAM 9	Disintegrin and metalloproteinase domain-containing protein 9	Q13443	ADAM9	NA	Wald ratio	1	0.029	0.256	9.093E-01	0.997	pan	Hospitalized COVID-19
18220_141	SRA1	Steroid receptor RNA activator 1	Q9HD15	SRA1	NA	Inverse variance weighted	2	-0.018	0.159	9.097E-01	0.997	pan	Hospitalized COVID-19
15441_6	SAP3	Ganglioside GM2 activator	P17900	GM2A	NA	Inverse variance weighted	6	-0.006	0.054	9.098E-01	0.997	pan	Hospitalized COVID-19
3820_68	MAPK2	MAP kinase-activated protein kinase 2	P49137	MAPKAPK2	NA	Wald ratio	1	0.011	0.098	9.099E-01	0.997	pan	Hospitalized COVID-19
18175_65	VAT1	Synaptic vesicle membrane protein VAT-1 homolog	Q99536	VAT1	NA	Inverse variance weighted	2	0.040	0.358	9.101E-01	0.997	pan	Hospitalized COVID-19
7156_2	FUT10	Alpha-(1,3)-fucosyltransferase 10	Q6P4F1	FUT10	NA	Inverse variance weighted	16	0.002	0.021	9.105E-01	0.997	pan	Hospitalized COVID-19
8028_22	SPINK5	Serine protease inhibitor Kazal-type 5	Q9NQ38	SPINK5	NA	Inverse variance weighted	2	-0.013	0.117	9.106E-01	0.997	pan	Hospitalized COVID-19
14143_8	H2B2E	Histone H2B type 2-E	Q16778	H2BC21	NA	Wald ratio	1	-0.023	0.202	9.107E-01	0.997	pan	Hospitalized COVID-19
12847_27	MCE1	mRNA-capping enzyme	O60942	RNGTT	NA	Wald ratio	1	-0.033	0.293	9.113E-01	0.997	pan	Hospitalized COVID-19
7696_3	NBR1	Next to BRCA1 gene 1 protein	Q14596	NBR1	NA	Wald ratio	1	-0.038	0.343	9.119E-01	0.997	pan	Hospitalized COVID-19
8019_73	STMN3	Stathmin-3	Q9NZ72	STMN3	NA	Wald ratio	1	0.027	0.248	9.121E-01	0.997	pan	Hospitalized COVID-19
10900_272	STMN2	Stathmin-2	Q93045	STMN2	NA	Wald ratio	1	0.025	0.228	9.121E-01	0.997	pan	Hospitalized COVID-19
4328_2	BOC	Brother of CDO	Q9BWV1	BOC	NA	Inverse variance weighted	4	0.005	0.044	9.124E-01	0.997	pan	Hospitalized COVID-19
5656_53	PP11	Poly(U)-specific endoribonuclease	P21128	ENDOU	NA	Wald ratio	1	-0.018	0.167	9.128E-01	0.997	pan	Hospitalized COVID-19
18891_98	GBP2	Guanylate-binding protein 2	P32456	GBP2	NA	Wald ratio	1	-0.015	0.137	9.134E-01	0.997	pan	Hospitalized COVID-19
14685_17	PKB beta	RAC-beta serine/threonine-protein kinase	P31751	AKT2	NA	Inverse variance weighted	5	-0.009	0.086	9.135E-01	0.997	pan	Hospitalized COVID-19
8394_56	RNase 2	Non-secretory ribonuclease	P10153	RNASE2	NA	Wald ratio	1	-0.015	0.140	9.143E-01	0.997	pan	Hospitalized COVID-19
17331_138	KREM1	Kremen protein 1	Q96MU8	KREMEN1	NA	Inverse variance weighted	4	0.006	0.059	9.154E-01	0.997	pan	Hospitalized COVID-19
4440_15	FCRL3	Fc receptor-like protein 3	Q96P31	FCRL3	NA	Inverse variance weighted	5	0.007	0.067	9.168E-01	0.997	pan	Hospitalized COVID-19
9870_17	SYWC	Tryptophan--tRNA ligase, cytoplasmic	P23381	WARS1	NA	Inverse variance weighted	2	-0.006	0.058	9.178E-01	0.997	pan	Hospitalized COVID-19
4959_2	AGR2	Anterior gradient protein 2 homolog	O95994	AGR2	NA	Wald ratio	1	-0.018	0.177	9.184E-01	0.997	pan	Hospitalized COVID-19
14066_49	MAGI2	Membrane-associated guanylate kinase, WW and PDZ domain-containing protein 2	Q86UL8	MAGI2	NA	Inverse variance weighted	10	0.002	0.022	9.193E-01	0.997	pan	Hospitalized COVID-19
2771_35	IGFBP-1	Insulin-like growth factor-binding protein 1	P08833	IGFBP1	NA	Inverse variance weighted	2	0.012	0.117	9.193E-01	0.997	pan	Hospitalized COVID-19
19145_4	MREG	Melanoregulin	Q8N565	MREG	NA	Inverse variance weighted	3	0.011	0.113	9.211E-01	0.997	pan	Hospitalized COVID-19
9384_17	Cathelicidin peptide	Cathelicidin antimicrobial peptide	P49913	CAMP	NA	Inverse variance weighted	8	0.007	0.073	9.211E-01	0.997	pan	Hospitalized COVID-19
5765_53	DB121	Beta-defensin 121	Q5J5C9	DEFB121	NA	Wald ratio	1	0.027	0.278	9.212E-01	0.997	pan	Hospitalized COVID-19
15414_316	LDHA	L-lactate dehydrogenase A chain	P00338	LDHA	NA	Wald ratio	1	-0.018	0.182	9.222E-01	0.997	pan	Hospitalized COVID-19
7198_197	FA20B	Glycosaminoglycan xylosylkinase	O75063	FAM20B	NA	Inverse variance weighted	2	0.010	0.106	9.227E-01	0.997	pan	Hospitalized COVID-19
3640_14	RAP	alpha-2-macroglobulin receptor-associated protein	P30533	LRPAP1	NA	Inverse variance weighted	10	0.004	0.041	9.234E-01	0.997	pan	Hospitalized COVID-19
17140_57	PDGFD	Platelet-derived growth factor D	Q9GZP0	PDGFD	NA	Inverse variance weighted	5	0.010	0.103	9.237E-01	0.997	pan	Hospitalized COVID-19
7856_51	F151A	Protein FAM151A	Q8WW52	FAM151A	NA	Inverse variance weighted	11	0.003	0.034	9.241E-01	0.997	pan	Hospitalized COVID-19
17151_84	IRF-3	Interferon regulatory factor 3	Q14653	IRF3	NA	Wald ratio	1	0.013	0.137	9.250E-01	0.997	pan	Hospitalized COVID-19
9391_60	PCSK1	ProSAAS	Q9UHG2	PCSK1N	NA	Inverse variance weighted	14	-0.002	0.023	9.258E-01	0.997	pan	Hospitalized COVID-19
16763_11	LECT2	Leukocyte cell-derived chemotaxin-2	O14960	LECT2	NA	Inverse variance weighted	10	0.003	0.028	9.261E-01	0.997	pan	Hospitalized COVID-19
10666_7	GNPTG	N-acetylglucosamine-1-phosphotransferase subunit gamma	Q9UJJ9	GNPTG	NA	Inverse variance weighted	4	-0.005	0.052	9.264E-01	0.997	pan	Hospitalized COVID-19
8033_1	DJC18	DnaJ homolog subfamily C member 18	Q9H819	DNAJC18	NA	Wald ratio	1	-0.017	0.189	9.266E-01	0.997	pan	Hospitalized COVID-19
9015_1	PRG3	Proteoglycan 3	Q9Y2Y8	PRG3	NA	Inverse variance weighted	10	-0.007	0.072	9.270E-01	0.997	pan	Hospitalized COVID-19
8925_25	RIR2B	Ribonucleoside-diphosphate reductase subunit M2 B	Q7LG56	RRM2B	NA	Inverse variance weighted	2	0.006	0.069	9.275E-01	0.997	pan	Hospitalized COVID-19
7788_1	CF6	ATP synthase-coupling factor 6, mitochondrial	P18859	ATP5PF	NA	Wald ratio	1	0.013	0.149	9.280E-01	0.997	pan	Hospitalized COVID-19
15481_45	LL-37	Antibacterial protein LL-37	P49913	CAMP	NA	Inverse variance weighted	10	0.007	0.078	9.282E-01	0.997	pan	Hospitalized COVID-19
5634_39	OFUT1	GDP-fucose protein O-fucosyltransferase 1	Q9H488	POFUT1	NA	Inverse variance weighted	6	-0.003	0.036	9.283E-01	0.997	pan	Hospitalized COVID-19
3853_56	MDHC	Malate dehydrogenase, cytoplasmic	P40925	MDH1	NA	Wald ratio	1	-0.014	0.154	9.287E-01	0.997	pan	Hospitalized COVID-19
10560_1	ITM2C:N-term	Integral membrane protein 2C:N-term	Q9NQX7	ITM2C	NA	Wald ratio	1	0.020	0.222	9.288E-01	0.997	pan	Hospitalized COVID-19
4435_66	ENPP7	Ectonucleotide pyrophosphatase/phosphodiesterase family member 7	Q6UWV6	ENPP7	NA	Inverse variance weighted	10	0.003	0.030	9.293E-01	0.997	pan	Hospitalized COVID-19
11128_29	T132C:CD	Transmembrane protein 132C:Cytoplasmic domain	Q8N3T6	TMEM132C	NA	Wald ratio	1	0.021	0.240	9.296E-01	0.997	pan	Hospitalized COVID-19
3438_10	FSTL3	Follistatin-related protein 3	O95633	FSTL3	NA	Inverse variance weighted	2	0.022	0.253	9.297E-01	0.997	pan	Hospitalized COVID-19
3488_64	Catalase	Catalase	P04040	CAT	NA	Inverse variance weighted	4	-0.015	0.171	9.299E-01	0.997	pan	Hospitalized COVID-19
3484_60	Angiotensinogen	Angiotensinogen	P01019	AGT	NA	Inverse variance weighted	3	-0.008	0.087	9.301E-01	0.997	pan	Hospitalized COVID-19
17138_8	GST A1-1	Glutathione S-transferase A1	P08263	GSTA1	NA	Inverse variance weighted	4	-0.010	0.114	9.306E-01	0.997	pan	Hospitalized COVID-19
16882_27	PHP14	14 kDa phosphohistidine phosphatase	Q9NRX4	PHPT1	NA	Inverse variance weighted	2	0.014	0.161	9.308E-01	0.997	pan	Hospitalized COVID-19
10037_98	SIG12:Ig-like V-type 2	Sialic acid-binding Ig-like lectin 12:Ig-like V-type 2 domain, Isoform long	Q96PQ1	SIGLEC12	NA	Inverse variance weighted	9	0.006	0.065	9.310E-01	0.997	pan	Hospitalized COVID-19
9339_204	FKBP2	Peptidyl-prolyl cis-trans isomerase FKBP2	P26885	FKBP2	NA	Wald ratio	1	0.019	0.216	9.311E-01	0.997	pan	Hospitalized COVID-19
12727_7	FPRP	Prostaglandin F2 receptor negative regulator	Q9P2B2	PTGFRN	NA	Inverse variance weighted	14	0.002	0.025	9.314E-01	0.997	pan	Hospitalized COVID-19
17696_1	TIRP	TIR domain-containing adapter molecule 2	Q86XR7	TICAM2	NA	Wald ratio	1	-0.029	0.334	9.318E-01	0.997	pan	Hospitalized COVID-19
13133_73	LTBP4	Latent-transforming growth factor beta-binding protein 4	Q8N2S1	LTBP4	NA	Inverse variance weighted	2	0.019	0.225	9.319E-01	0.997	pan	Hospitalized COVID-19
5018_68	Peroxiredoxin-6	Peroxiredoxin-6	P30041	PRDX6	NA	Wald ratio	1	-0.014	0.170	9.322E-01	0.997	pan	Hospitalized COVID-19
5954_62	PTH	Parathyroid hormone	P01270	PTH	NA	Inverse variance weighted	2	-0.007	0.080	9.326E-01	0.997	pan	Hospitalized COVID-19
10974_20	ISK7	Serine protease inhibitor Kazal-type 7	P58062	SPINK7	NA	Inverse variance weighted	5	-0.006	0.074	9.327E-01	0.997	pan	Hospitalized COVID-19
5000_52	LG3BP	Galectin-3-binding protein	Q08380	LGALS3BP	NA	Inverse variance weighted	10	-0.004	0.049	9.338E-01	0.997	pan	Hospitalized COVID-19
13624_17	NADK	NAD kinase	O95544	NADK	NA	Wald ratio	1	0.007	0.085	9.339E-01	0.997	pan	Hospitalized COVID-19
14273_19	PPCE	Prolyl endopeptidase	P48147	PREP	NA	Inverse variance weighted	3	0.005	0.059	9.340E-01	0.997	pan	Hospitalized COVID-19
17391_10	SPF45	Splicing factor 45	Q96I25	RBM17	NA	Inverse variance weighted	2	-0.004	0.048	9.341E-01	0.997	pan	Hospitalized COVID-19
4925_54	MMP-13	Collagenase 3	P45452	MMP13	NA	Inverse variance weighted	2	0.011	0.133	9.343E-01	0.997	pan	Hospitalized COVID-19
19124_9	UBCP1	Ubiquitin-like domain-containing CTD phosphatase 1	Q8WVY7	UBLCP1	NA	Inverse variance weighted	5	-0.006	0.073	9.343E-01	0.997	pan	Hospitalized COVID-19
14662_6	MZF1	Myeloid zinc finger 1	P28698	MZF1	NA	Wald ratio	1	-0.011	0.138	9.344E-01	0.997	pan	Hospitalized COVID-19
6375_75	XXLT1	Xyloside xylosyltransferase 1	Q8NBI6	XXYLT1	NA	Inverse variance weighted	2	-0.004	0.046	9.346E-01	0.997	pan	Hospitalized COVID-19
9565_6	PAWR	PRKC apoptosis WT1 regulator protein	Q96IZ0	PAWR	NA	Wald ratio	1	0.019	0.229	9.352E-01	0.997	pan	Hospitalized COVID-19
18397_5	AK1C4	Aldo-keto reductase family 1 member C4	P17516	AKR1C4	NA	Inverse variance weighted	11	0.003	0.034	9.352E-01	0.997	pan	Hospitalized COVID-19
4232_19	IGF-I sR	Insulin-like growth factor 1 receptor	P08069	IGF1R	NA	Inverse variance weighted	2	0.008	0.097	9.354E-01	0.997	pan	Hospitalized COVID-19
13959_7	LAP	Cytosol aminopeptidase	P28838	LAP3	NA	Wald ratio	1	0.020	0.250	9.358E-01	0.997	pan	Hospitalized COVID-19
9185_15	TFF1	Trefoil factor 1	P04155	TFF1	NA	Inverse variance weighted	8	0.004	0.047	9.358E-01	0.997	pan	Hospitalized COVID-19
12399_194	CCD50	Coiled-coil domain-containing protein 50	Q8IVM0	CCDC50	NA	Inverse variance weighted	2	0.012	0.153	9.359E-01	0.997	pan	Hospitalized COVID-19
8480_29	FBLN3	EGF-containing fibulin-like extracellular matrix protein 1	Q12805	EFEMP1	NA	Inverse variance weighted	3	-0.005	0.058	9.374E-01	0.997	pan	Hospitalized COVID-19
12563_2	TFIP8	Tumor necrosis factor alpha-induced protein 8	O95379	TNFAIP8	NA	Inverse variance weighted	2	-0.005	0.066	9.374E-01	0.997	pan	Hospitalized COVID-19
17456_53	GOLM1	Golgi membrane protein 1	Q8NBJ4	GOLM1	NA	Inverse variance weighted	6	-0.005	0.065	9.381E-01	0.997	pan	Hospitalized COVID-19

4706_17	41	Protein 4.1	P11171	EPB41	NA	Wald ratio	1	0.015	0.198	9.384E-01	0.997	pan	Hospitalized COVID-19
8942_2	RM21	39S ribosomal protein L21, mitochondrial	Q7Z2W9	MRPL21	NA	Wald ratio	1	-0.021	0.268	9.387E-01	0.997	pan	Hospitalized COVID-19
6493_9	CBPZ	Carboxypeptidase Z	Q66K79	CPZ	NA	Inverse variance weighted	2	-0.011	0.145	9.387E-01	0.997	pan	Hospitalized COVID-19
12370_30	Apo F	Apolipoprotein F	Q13790	APOF	NA	Inverse variance weighted	2	-0.007	0.087	9.396E-01	0.997	pan	Hospitalized COVID-19
6521_35	NPTX2	Neuronal pentraxin-2	P47972	NPTX2	NA	Inverse variance weighted	3	-0.007	0.088	9.408E-01	0.997	pan	Hospitalized COVID-19
5028_59	sCD163	Scavenger receptor cysteine-rich type 1 protein M130	Q86VB7	CD163	NA	Inverse variance weighted	12	0.004	0.051	9.408E-01	0.997	pan	Hospitalized COVID-19
11311_79	RAG-1	V(D)J recombination-activating protein 1	P15918	RAG1	NA	Wald ratio	1	0.014	0.189	9.412E-01	0.997	pan	Hospitalized COVID-19
19448_104	UPP1	Uridine phosphorylase 1	Q16831	UPP1	NA	Wald ratio	1	-0.009	0.119	9.414E-01	0.997	pan	Hospitalized COVID-19
18180_58	RT4I1	Reticulon-4-interacting protein 1, mitochondrial	Q8WWV3	RTN4IP1	NA	Wald ratio	1	0.012	0.168	9.419E-01	0.997	pan	Hospitalized COVID-19
19637_9	CRH	Corticoliberin	P06850	CRH	NA	Wald ratio	1	-0.012	0.168	9.421E-01	0.997	pan	Hospitalized COVID-19
8960_3	LRAP	Endoplasmic reticulum aminopeptidase 2	Q6P179	ERAP2	NA	Inverse variance weighted	27	-0.002	0.025	9.424E-01	0.997	pan	Hospitalized COVID-19
8040_9	LEMD1	LEM domain-containing protein 1	Q68G75	LEMD1	NA	Wald ratio	1	-0.010	0.136	9.434E-01	0.997	pan	Hospitalized COVID-19
4258_15	PA2G4	Proliferation-associated protein 2G4	Q9UQ80	PA2G4	NA	Wald ratio	1	-0.012	0.175	9.436E-01	0.997	pan	Hospitalized COVID-19
11388_75	HE4	WAP four-disulfide core domain protein 2	Q14508	WFDC2	NA	Wald ratio	1	0.013	0.191	9.438E-01	0.997	pan	Hospitalized COVID-19
8428_102	NTRI	Neurotrimin	Q9P121	NTM	NA	Inverse variance weighted	4	0.006	0.090	9.438E-01	0.997	pan	Hospitalized COVID-19
17802_4	SIAS	Sialic acid synthase	Q9NR45	NANS	NA	Wald ratio	1	0.008	0.119	9.445E-01	0.997	pan	Hospitalized COVID-19
10907_116	NTRI	Neurotrimin	Q9P121	NTM	NA	Inverse variance weighted	3	0.010	0.148	9.447E-01	0.997	pan	Hospitalized COVID-19
2644_11	PKC-A	Protein kinase C alpha type	P17252	PRKCA	NA	Wald ratio	1	0.015	0.221	9.449E-01	0.997	pan	Hospitalized COVID-19
5256_86	PDE5A	cGMP-specific 3',5'-cyclic phosphodiesterase	O76074	PDE5A	NA	Inverse variance weighted	5	0.006	0.091	9.455E-01	0.997	pan	Hospitalized COVID-19
11308_8	CR3L4	Cyclic AMP-responsive element-binding protein 3-like protein 4	Q8TEY5	CREB3L4	NA	Inverse variance weighted	8	0.004	0.053	9.461E-01	0.997	pan	Hospitalized COVID-19
17224_12	MIME	Mimecan	P20774	OGN	NA	Inverse variance weighted	6	-0.007	0.110	9.462E-01	0.997	pan	Hospitalized COVID-19
13112_179	FSTL1	Follistatin-related protein 1	Q12841	FSTL1	NA	Inverse variance weighted	4	-0.008	0.126	9.470E-01	0.997	pan	Hospitalized COVID-19
16288_17	EPHA4	Ephrin type-A receptor 4	P54764	EPHA4	NA	Inverse variance weighted	5	0.004	0.060	9.472E-01	0.997	pan	Hospitalized COVID-19
12510_3	STAP1	Signal-transducing adaptor protein 1	Q9ULZ2	STAP1	NA	Wald ratio	1	0.010	0.153	9.474E-01	0.997	pan	Hospitalized COVID-19
9838_4	SMAD1	Mothers against decapentaplegic homolog 1	Q15797	SMAD1	NA	Wald ratio	1	0.014	0.219	9.474E-01	0.997	pan	Hospitalized COVID-19
8097_77	LIPN	Lipase member N	Q5VXI9	LIPN	NA	Inverse variance weighted	14	-0.003	0.046	9.484E-01	0.997	pan	Hospitalized COVID-19
12329_21	KS6A1	Ribosomal protein S6 kinase alpha-1	Q15418	RPS6KA1	NA	Inverse variance weighted	10	-0.004	0.066	9.492E-01	0.997	pan	Hospitalized COVID-19
7956_11	MOS1	MICOS complex subunit MIC10	Q5TGZ0	MICOS10	NA	Inverse variance weighted	12	0.000	0.007	9.495E-01	0.997	pan	Hospitalized COVID-19
18197_97	KCRS	Creatine kinase S-type, mitochondrial	P17540	CKMT2	NA	Inverse variance weighted	2	0.015	0.240	9.501E-01	0.997	pan	Hospitalized COVID-19
4979_34	DERM	Dermatopontin	Q07507	DPT	NA	Inverse variance weighted	7	-0.002	0.035	9.504E-01	0.997	pan	Hospitalized COVID-19
5609_92	F19A5	Protein FAM19A5	Q7Z5A7	TAFA5	NA	Wald ratio	1	-0.014	0.222	9.506E-01	0.997	pan	Hospitalized COVID-19
6919_3	HBAZ	Hemoglobin subunit zeta	P02008	HBZ	NA	Inverse variance weighted	8	-0.002	0.028	9.506E-01	0.997	pan	Hospitalized COVID-19
6470_19	fibulin 1	Fibulin-1	P23142	FBLN1	NA	Inverse variance weighted	4	0.003	0.055	9.509E-01	0.997	pan	Hospitalized COVID-19
8274_64	Syntaxin-7	Syntaxin-7	O15400	STX7	NA	Wald ratio	1	0.005	0.087	9.510E-01	0.997	pan	Hospitalized COVID-19
4162_54	Transferrin	Serotransferrin	P02787	TF	NA	Inverse variance weighted	2	0.008	0.130	9.514E-01	0.997	pan	Hospitalized COVID-19
14011_17	S100A11	Protein S100-A11	P31949	S100A11	NA	Inverse variance weighted	3	0.003	0.057	9.519E-01	0.997	pan	Hospitalized COVID-19
4297_62	Spondin-1	Spondin-1	Q9HCB6	SPON1	NA	Inverse variance weighted	4	0.002	0.035	9.534E-01	0.997	pan	Hospitalized COVID-19
18330_7	PGM2	Phosphoglucomutase-2	Q96G03	PGM2	NA	Inverse variance weighted	6	-0.003	0.048	9.536E-01	0.997	pan	Hospitalized COVID-19
17164_15	annexin IV	Annexin A4	P09525	ANXA4	NA	Inverse variance weighted	2	0.006	0.103	9.537E-01	0.997	pan	Hospitalized COVID-19
8024_64	TPST2	Protein-tyrosine sulfotransferase 2	O60704	TPST2	NA	Inverse variance weighted	2	0.007	0.117	9.537E-01	0.997	pan	Hospitalized COVID-19
3033_57	Galectin-2	Galectin-2	P05162	LGALS2	NA	Inverse variance weighted	3	-0.003	0.050	9.547E-01	0.997	pan	Hospitalized COVID-19
14008_22	DCP1A	mRNA-decapping enzyme 1A	Q9NPI6	DCP1A	NA	Inverse variance weighted	3	0.003	0.055	9.549E-01	0.997	pan	Hospitalized COVID-19
3728_52	Secretin	Secretin	P09683	SCT	NA	Wald ratio	1	0.008	0.138	9.551E-01	0.997	pan	Hospitalized COVID-19
6223_5	GUC2B	Guanylate cyclase activator 2B	Q16661	GUCA2B	NA	Inverse variance weighted	5	-0.007	0.125	9.552E-01	0.997	pan	Hospitalized COVID-19
3806_55	EphA5	Ephrin type-A receptor 5	P54756	EPHA5	NA	Inverse variance weighted	3	-0.005	0.083	9.553E-01	0.997	pan	Hospitalized COVID-19
15644_1	Biotinidase	Biotinidase	P43251	BTD	NA	Inverse variance weighted	9	0.001	0.020	9.558E-01	0.997	pan	Hospitalized COVID-19
11696_7	RABP2	Cellular retinoic acid-binding protein 2	P29373	CRABP2	NA	Wald ratio	1	-0.004	0.066	9.560E-01	0.997	pan	Hospitalized COVID-19
12457_10	MTND	1,2-dihydroxy-3-keto-5-methylthiopentene dioxygenase	Q9BV57	ADI1	NA	Wald ratio	1	-0.008	0.152	9.570E-01	0.997	pan	Hospitalized COVID-19
4137_57	IL-17E	Interleukin-25	Q9H293	IL25	NA	Inverse variance weighted	2	-0.009	0.164	9.580E-01	0.997	pan	Hospitalized COVID-19
12990_39	MAP3K3	Mitogen-activated protein kinase kinase kinase 3	Q99759	MAP3K3	NA	Wald ratio	1	-0.005	0.093	9.588E-01	0.997	pan	Hospitalized COVID-19
6431_68	PCYOX	Prenylcysteine oxidase 1	Q9UHG3	PCYOX1	NA	Inverse variance weighted	12	-0.003	0.050	9.592E-01	0.997	pan	Hospitalized COVID-19
6425_87	MMP19	Matrix metalloproteinase-19	Q99542	MMP19	NA	Inverse variance weighted	4	-0.005	0.093	9.614E-01	0.997	pan	Hospitalized COVID-19
6379_62	ATL2	ADAMTS-like protein 2	Q86TH1	ADAMTSL2	NA	Inverse variance weighted	2	0.006	0.135	9.616E-01	0.997	pan	Hospitalized COVID-19
18881_7	CD97	CD97 antigen	P48960	ADGRE5	NA	Inverse variance weighted	7	-0.003	0.063	9.617E-01	0.997	pan	Hospitalized COVID-19
3166_92	Siglec-3	Myeloid cell surface antigen CD33	P20138	CD33	NA	Inverse variance weighted	6	0.001	0.022	9.621E-01	0.997	pan	Hospitalized COVID-19
18172_71	ASF1A	Histone chaperone ASF1A	Q9Y294	ASF1A	NA	Inverse variance weighted	2	0.004	0.084	9.621E-01	0.997	pan	Hospitalized COVID-19
17231_1	L-plastin	Plastin-2	P13796	LCP1	NA	Wald ratio	1	-0.008	0.174	9.625E-01	0.997	pan	Hospitalized COVID-19
12436_84	GST omega-1	Glutathione S-transferase omega-1	P78417	GSTO1	NA	Inverse variance weighted	17	-0.001	0.025	9.627E-01	0.997	pan	Hospitalized COVID-19
10428_1	KI2S2	Killer cell immunoglobulin-like receptor 2DS2	P43631	KIR2DS2	NA	Inverse variance weighted	9	0.003	0.057	9.630E-01	0.997	pan	Hospitalized COVID-19
7968_15	CRTAM	Cytotoxic and regulatory T-cell molecule	O95727	CRTAM	NA	Inverse variance weighted	7	-0.003	0.055	9.630E-01	0.997	pan	Hospitalized COVID-19
8778_3	Noggin	Noggin	Q13253	NOG	NA	Inverse variance weighted	11	-0.002	0.040	9.630E-01	0.997	pan	Hospitalized COVID-19
18876_77	CHST4	Carbohydrate sulfotransferase 4	Q8NCG5	CHST4	NA	Wald ratio	1	-0.010	0.235	9.651E-01	0.997	pan	Hospitalized COVID-19
18307_71	PPase 2	Inorganic pyrophosphatase 2, mitochondrial	Q9H2U2	PPA2	NA	Wald ratio	1	0.006	0.129	9.652E-01	0.997	pan	Hospitalized COVID-19
18935_14	TLR5	Toll-like receptor 5	O60602	TLR5	NA	Inverse variance weighted	11	0.001	0.033	9.655E-01	0.997	pan	Hospitalized COVID-19
5637_81	NTNG1	Netrin-G1	Q9Y2I2	NTNG1	NA	Inverse variance weighted	15	0.001	0.022	9.658E-01	0.997	pan	Hospitalized COVID-19
17836_17	S100A16	Protein S100-A16	Q96FQ6	S100A16	NA	Wald ratio	1	0.006	0.145	9.664E-01	0.997	pan	Hospitalized COVID-19
7994_41	ERO1B	ERO1-like protein beta	Q86YB8	ERO1B	NA	Inverse variance weighted	2	0.002	0.056	9.666E-01	0.997	pan	Hospitalized COVID-19
19357_11	VAMP7	Vesicle-associated membrane protein 7	P51809	VAMP7	NA	Wald ratio	1	-0.008	0.197	9.666E-01	0.997	pan	Hospitalized COVID-19
3343_1	Aminoacylase-1	Aminoacylase-1	Q03154	ACY1	NA	Inverse variance weighted	3	-0.004	0.105	9.670E-01	0.997	pan	Hospitalized COVID-19
3198_4	IDS	Iduronate 2-sulfatase	P22304	IDS	NA	Wald ratio	1	-0.006	0.158	9.680E-01	0.997	pan	Hospitalized COVID-19
2789_26	MMP-7	Matrilysin	P09237	MMP7	NA	Inverse variance weighted	7	0.002	0.044	9.692E-01	0.997	pan	Hospitalized COVID-19
17435_43	ETFA	Electron transfer flavoprotein subunit alpha, mitochondrial	P13804	ETFA	NA	Inverse variance weighted	6	-0.003	0.074	9.693E-01	0.997	pan	Hospitalized COVID-19
13676_46	Inhibin bB chain	Inhibin beta B chain	P09529	INHBB	NA	Inverse variance weighted	6	0.002	0.043	9.694E-01	0.997	pan	Hospitalized COVID-19
6440_31	MFAP5	Microfibrillar-associated protein 5	Q13361	MFAP5	NA	Inverse variance weighted	3	0.007	0.179	9.699E-01	0.997	pan	Hospitalized COVID-19
4413_3	SLPI	Antileukoproteinase	P03973	SLPI	NA	Wald ratio	1	-0.008	0.221	9.699E-01	0.997	pan	Hospitalized COVID-19
6378_2	CEI	Protein CEI	Q86SI9	C5orf38	NA	Wald ratio	1	0.006	0.160	9.703E-01	0.997	pan	Hospitalized COVID-19
15576_158	ECP	Eosinophil cationic protein	P12724	RNASE3	NA	Inverse variance weighted	8	0.001	0.035	9.705E-01	0.997	pan	Hospitalized COVID-19
5134_52	TIMD3	Hepatitis A virus cellular receptor 2	Q8TDQ0	HAVCR2	NA	Inverse variance weighted	9	-0.001	0.031	9.707E-01	0.997	pan	Hospitalized COVID-19

10851_77	IL27B	Interleukin-27 subunit beta	Q14213	EBI3	NA	Inverse variance weighted	10	-0.001	0.020	9.728E-01	0.997	pan	Hospitalized COVID-19
18877_15	CNN2	Calponin-2	Q99439	CNN2	NA	Inverse variance weighted	2	-0.006	0.172	9.735E-01	0.997	pan	Hospitalized COVID-19
19590_46	SP-D	Pulmonary surfactant-associated protein D	P35247	SFTPD	NA	Inverse variance weighted	21	-0.001	0.032	9.738E-01	0.997	pan	Hospitalized COVID-19
12859_33	PECI	Enoyl-CoA delta isomerase 2, mitochondrial	O75521	ECI2	NA	Inverse variance weighted	4	0.002	0.055	9.740E-01	0.997	pan	Hospitalized COVID-19
14711_27	Cystatin M	Cystatin-M	Q15828	CST6	NA	Inverse variance weighted	7	-0.002	0.057	9.742E-01	0.997	pan	Hospitalized COVID-19
7947_19	AP4AT	AP-4 complex accessory subunit tepsin	Q96N21	TEPSIN	NA	Wald ratio	1	0.006	0.190	9.744E-01	0.997	pan	Hospitalized COVID-19
5275_28	VAV	Proto-oncogene vav	P15498	VAV1	NA	Wald ratio	1	-0.006	0.185	9.748E-01	0.997	pan	Hospitalized COVID-19
3420_21	Carbonic anhydrase XIII	Carbonic anhydrase 13	Q8N1Q1	CA13	NA	Inverse variance weighted	5	-0.001	0.034	9.754E-01	0.997	pan	Hospitalized COVID-19
10584_7	NDUS4	NADH dehydrogenase [ubiquinone] iron-sulfur protein 4, mitochondrial	O43181	NDUFS4	NA	Inverse variance weighted	20	-0.002	0.055	9.755E-01	0.997	pan	Hospitalized COVID-19
3516_60	SDF-1	Stromal cell-derived factor 1	P48061	CXCL12	NA	Inverse variance weighted	2	-0.002	0.066	9.760E-01	0.997	pan	Hospitalized COVID-19
9931_20	Keratin-1	Keratin, type II cytoskeletal 1	P04264	KRT1	NA	Inverse variance weighted	2	-0.007	0.237	9.770E-01	0.997	pan	Hospitalized COVID-19
17384_110	K6PF	ATP-dependent 6-phosphofructokinase, muscle type	P08237	PFKM	NA	Inverse variance weighted	3	-0.002	0.084	9.775E-01	0.997	pan	Hospitalized COVID-19
16292_288	GIP	Gastric inhibitory polypeptide	P09681	GIP	NA	Wald ratio	1	-0.006	0.237	9.789E-01	0.997	pan	Hospitalized COVID-19
12401_3	STALP	AMSH-like protease	Q96FJ0	STAMBPL1	NA	Wald ratio	1	-0.003	0.110	9.790E-01	0.997	pan	Hospitalized COVID-19
18922_27	CD68	Macrosialin	P34810	CD68	NA	Inverse variance weighted	4	-0.002	0.095	9.791E-01	0.997	pan	Hospitalized COVID-19
17343_6	SNPH	Syntaphilin	O15079	SNPH	NA	Inverse variance weighted	2	-0.003	0.118	9.797E-01	0.997	pan	Hospitalized COVID-19
5430_66	SHPS1	Tyrosine-protein phosphatase non-receptor type substrate 1	P78324	SIRPA	NA	Inverse variance weighted	9	-0.001	0.026	9.805E-01	0.997	pan	Hospitalized COVID-19
13499_30	Coagulation Factor VIII	Coagulation Factor VIII	P00451	F8	NA	Inverse variance weighted	6	-0.002	0.093	9.806E-01	0.997	pan	Hospitalized COVID-19
13587_10	RGAP1	Rac GTPase-activating protein 1	Q9H0H5	RACGAP1	NA	Inverse variance weighted	11	-0.001	0.049	9.810E-01	0.997	pan	Hospitalized COVID-19
6556_5	ENPP5	Ectonucleotide pyrophosphatase/phosphodiesterase family member 5	Q9UJA9	ENPP5	NA	Inverse variance weighted	15	0.000	0.014	9.816E-01	0.997	pan	Hospitalized COVID-19
7085_81	CDSN	Corneodesmosin	Q15517	CDSN	NA	Inverse variance weighted	7	-0.001	0.051	9.817E-01	0.997	pan	Hospitalized COVID-19
5102_55	MICB	MHC class I polypeptide-related sequence B	Q29980	MICB	NA	Inverse variance weighted	14	-0.001	0.040	9.817E-01	0.997	pan	Hospitalized COVID-19
11424_4	FAAA	Fumarylacetoacetase	P16930	FAH	NA	Inverse variance weighted	7	0.001	0.037	9.817E-01	0.997	pan	Hospitalized COVID-19
5618_50	FAM3B	Protein FAM3B	P58499	FAM3B	NA	Inverse variance weighted	2	0.005	0.240	9.818E-01	0.997	pan	Hospitalized COVID-19
14655_1	DJC17	DnaJ homolog subfamily C member 17	Q9NVM6	DNAJC17	NA	Inverse variance weighted	4	-0.002	0.075	9.824E-01	0.997	pan	Hospitalized COVID-19
3232_28	TrATPase	Tartrate-resistant acid phosphatase type 5	P13686	ACP5	NA	Inverse variance weighted	6	-0.001	0.041	9.826E-01	0.997	pan	Hospitalized COVID-19
10615_18	MYP0	Myelin protein P0	P25189	MPZ	NA	Inverse variance weighted	14	-0.001	0.031	9.828E-01	0.997	pan	Hospitalized COVID-19
14047_78	BDNF	Brain-derived neurotrophic factor	P23560	BDNF	NA	Inverse variance weighted	2	-0.004	0.185	9.830E-01	0.997	pan	Hospitalized COVID-19
9370_69	GGH	Gamma-glutamyl hydrolase	Q92820	GGH	NA	Inverse variance weighted	13	-0.001	0.027	9.830E-01	0.997	pan	Hospitalized COVID-19
6364_7	TPSNR	Tapasin-related protein	Q9BX59	TAPBPL	NA	Inverse variance weighted	14	0.000	0.022	9.835E-01	0.997	pan	Hospitalized COVID-19
8974_172	COFA1	Collagen alpha-1(XV) chain	P39059	COL15A1	NA	Inverse variance weighted	7	0.001	0.042	9.836E-01	0.997	pan	Hospitalized COVID-19
9748_31	GSTM3-3	Glutathione S-transferase Mu 3	P21266	GSTM3	NA	Inverse variance weighted	7	0.001	0.035	9.837E-01	0.997	pan	Hospitalized COVID-19
8300_82	PEX14:N-term	Peroxisomal membrane protein PEX14:N-term	O75381	PEX14	NA	Inverse variance weighted	2	-0.006	0.280	9.842E-01	0.997	pan	Hospitalized COVID-19
5105_2	Nogo Receptor	Reticulon-4 receptor	Q9BZR6	RTN4R	NA	Inverse variance weighted	5	-0.001	0.031	9.847E-01	0.997	pan	Hospitalized COVID-19
3280_49	Aggrecan	Aggrecan core protein	P16112	ACAN	NA	Inverse variance weighted	3	0.001	0.072	9.851E-01	0.997	pan	Hospitalized COVID-19
3296_92	CNTN2	Contactin-2	Q02246	CNTN2	NA	Inverse variance weighted	9	0.000	0.020	9.852E-01	0.997	pan	Hospitalized COVID-19
6627_25	LIPR1	Inactive pancreatic lipase-related protein 1	P54315	PNLIPRP1	NA	Inverse variance weighted	13	-0.001	0.063	9.856E-01	0.997	pan	Hospitalized COVID-19
4159_130	Factor H	Complement factor H	P08603	CFH	NA	Wald ratio	1	0.003	0.160	9.860E-01	0.997	pan	Hospitalized COVID-19
4930_21	Stanniocalcin-1	Stanniocalcin-1	P52823	STC1	NA	Inverse variance weighted	3	-0.002	0.134	9.866E-01	0.997	pan	Hospitalized COVID-19
10643_16	NOE3:C-term	Noelin-3:Isoform 2, C-term	Q96PB7	OLFM3	NA	Wald ratio	1	0.003	0.207	9.873E-01	0.997	pan	Hospitalized COVID-19
8288_27	b2-Glycoprotein I	Beta-2-glycoprotein 1	P02749	APOH	NA	Inverse variance weighted	6	0.000	0.033	9.884E-01	0.997	pan	Hospitalized COVID-19
10605_22	APMAP	Adipocyte plasma membrane-associated protein	Q9HDC9	APMAP	NA	Wald ratio	1	0.003	0.179	9.885E-01	0.997	pan	Hospitalized COVID-19
8225_86	EPHB2	Ephrin type-B receptor 2	P29323	EPHB2	NA	Inverse variance weighted	3	-0.001	0.057	9.888E-01	0.997	pan	Hospitalized COVID-19
5676_54	ASIP	Agouti-signaling protein	P42127	ASIP	NA	Inverse variance weighted	10	-0.001	0.058	9.888E-01	0.997	pan	Hospitalized COVID-19
17737_7	IVD	Isovaleryl-CoA dehydrogenase, mitochondrial	P26440	IVD	NA	Inverse variance weighted	3	-0.001	0.071	9.893E-01	0.997	pan	Hospitalized COVID-19
16913_8	RNT2	Ribonuclease T2	O00584	RNASET2	NA	Inverse variance weighted	6	0.000	0.023	9.915E-01	0.997	pan	Hospitalized COVID-19
6409_57	GP116	Adhesion G protein-coupled receptor F5	Q8IZF2	ADGRF5	NA	Inverse variance weighted	13	0.000	0.016	9.916E-01	0.997	pan	Hospitalized COVID-19
4499_21	PDGF-AA	Platelet-derived growth factor subunit A	P04085	PDGFA	NA	Inverse variance weighted	3	-0.001	0.126	9.919E-01	0.997	pan	Hospitalized COVID-19
3060_43	C9	Complement component C9	P02748	C9	NA	Inverse variance weighted	6	0.001	0.062	9.925E-01	0.997	pan	Hospitalized COVID-19
19154_41	Protease nexin I	Glia-derived nexin	P07093	SERPINE2	NA	Inverse variance weighted	13	0.000	0.028	9.930E-01	0.997	pan	Hospitalized COVID-19
8351_17	PRS57	Serine protease 57	Q6UWY2	PRSS57	NA	Inverse variance weighted	7	0.000	0.038	9.933E-01	0.997	pan	Hospitalized COVID-19
3329_14	PGRP-S	Peptidoglycan recognition protein 1	O75594	PGLYRP1	NA	Inverse variance weighted	4	-0.001	0.066	9.933E-01	0.997	pan	Hospitalized COVID-19
3342_76	ABL2	Abelson tyrosine-protein kinase 2	P42684	ABL2	NA	Wald ratio	1	0.002	0.219	9.942E-01	0.998	pan	Hospitalized COVID-19
3434_34	FN1.3	Fibronectin Fragment 3	P02751	FN1	NA	Wald ratio	1	0.001	0.153	9.948E-01	0.998	pan	Hospitalized COVID-19
3435_53	FN1.4	Fibronectin Fragment 4	P02751	FN1	NA	Inverse variance weighted	2	-0.001	0.178	9.952E-01	0.998	pan	Hospitalized COVID-19
7144_234	KAZD1	Kazal-type serine protease inhibitor domain-containing protein 1	Q96I82	KAZALD1	NA	Wald ratio	1	-0.001	0.181	9.958E-01	0.998	pan	Hospitalized COVID-19
9816_37	ISOC1	Isochorismatase domain-containing protein 1	Q96CN7	ISOC1	NA	Inverse variance weighted	4	0.000	0.051	9.968E-01	0.999	pan	Hospitalized COVID-19
8374_5	NGF R	Tumor necrosis factor receptor superfamily member 16	P08138	NGFR	NA	Inverse variance weighted	6	0.000	0.054	9.972E-01	0.999	pan	Hospitalized COVID-19
11646_4	Carbohydrate sulfotransferase 9	Carbohydrate sulfotransferase 9	Q7L1S5	CHST9	NA	Inverse variance weighted	3	0.000	0.065	9.982E-01	0.999	pan	Hospitalized COVID-19
9278_9	SDF-1	Stromal cell-derived factor 1	P48061	CXCL12	NA	Inverse variance weighted	7	0.000	0.079	9.988E-01	0.999	pan	Hospitalized COVID-19
9337_43	TKN1	Protachykinin-1	P20366	TAC1	NA	Inverse variance weighted	6	0.000	0.046	9.990E-01	0.999	pan	Hospitalized COVID-19
17769_28	PCNP	PEST proteolytic signal-containing nuclear protein	Q8WW12	PCNP	NA	Wald ratio	1	-0.381	0.056	1.221E-11	0.000	pan	SARS-CoV-2 infection
6359_50	AGO61	Protein O-linked-mannose beta-1,4-N-acetylglucosaminyltransferase 2	Q8NAT1	POMGNT2	NA	Inverse variance weighted	12	-0.044	0.013	5.067E-04	0.323	pan	SARS-CoV-2 infection
12616_45	NRBP	Nuclear receptor-binding protein	Q9UHY1	NRBP1	NA	Wald ratio	1	0.128	0.038	7.708E-04	0.323	pan	SARS-CoV-2 infection
19272_9	PEF1	Peflin	Q9UBV8	PEF1	NA	Inverse variance weighted	11	0.046	0.014	7.949E-04	0.323	pan	SARS-CoV-2 infection
19127_1	HSPB6	Heat shock protein beta-6	O14558	HSPB6	NA	Wald ratio	1	0.377	0.115	1.021E-03	0.323	pan	SARS-CoV-2 infection
11212_7	TXND5	Thioredoxin domain-containing protein 5	Q8NBS9	TXNDC5	NA	Inverse variance weighted	5	0.090	0.028	1.231E-03	0.323	pan	SARS-CoV-2 infection
17697_2	OVCA2	Esterase OVCA2	Q8WZ82	OVCA2	NA	Wald ratio	1	0.148	0.046	1.344E-03	0.323	pan	SARS-CoV-2 infection
11646_4	Carbohydrate sulfotransferase 9	Carbohydrate sulfotransferase 9	Q7L1S5	CHST9	NA	Inverse variance weighted	3	-0.094	0.029	1.449E-03	0.323	pan	SARS-CoV-2 infection
11205_10	Integrin beta-7	Integrin beta-7	P26010	ITGB7	NA	Wald ratio	1	0.318	0.101	1.559E-03	0.323	pan	SARS-CoV-2 infection
17777_31	SDSL	Serine dehydratase-like	Q96GA7	SDSL	NA	Inverse variance weighted	7	0.052	0.016	1.621E-03	0.323	pan	SARS-CoV-2 infection
15305_7	Secretagogin	Secretagogin	O76038	SCGN	NA	Inverse variance weighted	2	0.169	0.054	1.701E-03	0.323	pan	SARS-CoV-2 infection
3181_50	Cathepsin S	Cathepsin S	P25774	CTSS	NA	Inverse variance weighted	11	-0.055	0.018	1.771E-03	0.323	pan	SARS-CoV-2 infection
6444_15	PSG3	Pregnancy-specific beta-1-glycoprotein 3	Q16557	PSG3	NA	Inverse variance weighted	7	0.065	0.022	2.371E-03	0.399	pan	SARS-CoV-2 infection
16918_198	TLR3	Toll-like receptor 3	O15455	TLR3	NA	Inverse variance weighted	9	0.033	0.011	3.159E-03	0.493	pan	SARS-CoV-2 infection
12656_1	KLC1	Kinesin light chain 1	Q07866	KLC1	NA	Wald ratio	1	0.249	0.085	3.398E-03	0.495	pan	SARS-CoV-2 infection
9916_146	LRC4B:ECD	Leucine-rich repeat-containing protein 4B:Extracellular domain	Q9NT99	LRRC4B	NA	Inverse variance weighted	2	-0.192	0.067	4.040E-03	0.550	pan	SARS-CoV-2 infection
4240_31	M2-PK	Pyruvate kinase PKM	P14618	PKM	NA	Inverse variance weighted	3	-0.181	0.063	4.275E-03	0.550	pan	SARS-CoV-2 infection

10015_119	KCAB2	Voltage-gated potassium channel subunit beta-2	Q13303	KCNAB2	NA	Inverse variance weighted	2	-0.181	0.064	4.683E-03	0.569	pan	SARS-CoV-2 infection
19206_20	8ODP	7,8-dihydro-8-oxoguanine triphosphatase	P36639	NUDT1	NA	Wald ratio	1	0.131	0.048	6.217E-03	0.576	pan	SARS-CoV-2 infection
4479_14	C1-Esterase Inhibitor	Plasma protease C1 inhibitor	P05155	SERPING1	NA	Inverse variance weighted	14	-0.028	0.010	6.569E-03	0.576	pan	SARS-CoV-2 infection
4923_79	MIS	Muellerian-inhibiting factor	P03971	AMH	NA	Inverse variance weighted	5	0.088	0.032	6.573E-03	0.576	pan	SARS-CoV-2 infection
10451_11	NUCB1	Nucleobindin-1	Q02818	NUCB1	NA	Wald ratio	1	0.132	0.049	6.821E-03	0.576	pan	SARS-CoV-2 infection
10647_18	ASCC1	Activating signal cointegrator 1 complex subunit 1	Q8N9N2	ASCC1	NA	Wald ratio	1	-0.224	0.083	7.222E-03	0.576	pan	SARS-CoV-2 infection
7257_18	TIP39	Tuberoinfundibular peptide of 39 residues	Q96A98	PTH2	NA	Wald ratio	1	0.377	0.141	7.306E-03	0.576	pan	SARS-CoV-2 infection
9017_58	LPH	Lactase-phlorizin hydrolase	P09848	LCT	NA	Inverse variance weighted	14	-0.021	0.008	7.540E-03	0.576	pan	SARS-CoV-2 infection
6457_50	PCDG1	Protocadherin gamma-A1	Q9Y5H4	PCDHGA1	NA	Inverse variance weighted	11	0.059	0.022	7.572E-03	0.576	pan	SARS-CoV-2 infection
16915_153	SEM4A	Semaphorin-4A	Q9H3S1	SEMA4A	NA	Inverse variance weighted	8	-0.058	0.022	7.675E-03	0.576	pan	SARS-CoV-2 infection
9092_33	ANGL1:N-term	Angiopoietin-related protein 1:N-term	O95841	ANGPTL1	NA	Wald ratio	1	0.249	0.094	8.065E-03	0.576	pan	SARS-CoV-2 infection
5735_54	C1GLC	C1GALT1-specific chaperone 1	Q96EU7	C1GALT1C1	NA	Inverse variance weighted	3	0.067	0.025	8.273E-03	0.576	pan	SARS-CoV-2 infection
10608_9	HIS1	Histatin-1	P15515	HTN1	NA	Inverse variance weighted	2	0.089	0.034	8.295E-03	0.576	pan	SARS-CoV-2 infection
8814_33	SAPL1	Proactivator polypeptide-like 1	Q6NUJ1	PSAPL1	NA	Inverse variance weighted	10	0.032	0.012	8.504E-03	0.576	pan	SARS-CoV-2 infection
17447_52	SFRP4	Secreted frizzled-related protein 4	Q6FHJ7	SFRP4	NA	Inverse variance weighted	4	-0.062	0.024	8.732E-03	0.576	pan	SARS-CoV-2 infection
10346_5	STAT3	Signal transducer and activator of transcription 3	P40763	STAT3	NA	Inverse variance weighted	2	-0.099	0.038	8.908E-03	0.576	pan	SARS-CoV-2 infection
4498_62	NCAM-120	Neural cell adhesion molecule 1, 120 kDa isoform	P13591	NCAM1	NA	Inverse variance weighted	12	-0.033	0.013	9.107E-03	0.576	pan	SARS-CoV-2 infection
9960_2	TBCD5	TBC1 domain family member 5	Q92609	TBC1D5	NA	Wald ratio	1	-0.242	0.093	9.479E-03	0.576	pan	SARS-CoV-2 infection
9234_8	TWSG1	Twisted gastrulation protein homolog 1	Q9GZX9	TWSG1	NA	Inverse variance weighted	2	0.126	0.049	9.648E-03	0.576	pan	SARS-CoV-2 infection
9377_25	SCF	Kit ligand	P21583	KITLG	NA	Inverse variance weighted	3	0.145	0.056	1.013E-02	0.576	pan	SARS-CoV-2 infection
17332_3	ARHL2	Poly(ADP-ribose) glycohydrolase ARH3	Q9NX46	ADPRS	NA	Inverse variance weighted	5	-0.071	0.028	1.048E-02	0.576	pan	SARS-CoV-2 infection
10521_10	MXRA8:ECD	Matrix-remodeling-associated protein 8:Extracellular domain	Q9BRK3	MXRA8	NA	Inverse variance weighted	3	-0.107	0.042	1.068E-02	0.576	pan	SARS-CoV-2 infection
9638_2	TIGIT	T-cell immunoreceptor with Ig and ITIM domains	Q495A1	TIGIT	NA	Wald ratio	1	-0.161	0.063	1.088E-02	0.576	pan	SARS-CoV-2 infection
13961_18	KIF3A	Kinesin-like protein KIF3A	Q9Y496	KIF3A	NA	Wald ratio	1	-0.231	0.091	1.103E-02	0.576	pan	SARS-CoV-2 infection
7788_1	CF6	ATP synthase-coupling factor 6, mitochondrial	P18859	ATP5PF	NA	Wald ratio	1	-0.161	0.063	1.107E-02	0.576	pan	SARS-CoV-2 infection
10428_1	KI2S2	Killer cell immunoglobulin-like receptor 2DS2	P43631	KIR2DS2	NA	Inverse variance weighted	9	-0.069	0.027	1.184E-02	0.602	pan	SARS-CoV-2 infection
5593_11	PDIA5	Protein disulfide-isomerase A5	Q14554	PDIA5	NA	Inverse variance weighted	11	-0.032	0.013	1.277E-02	0.610	pan	SARS-CoV-2 infection
11480_1	Aldehyde dehydrogenase, class 3	Aldehyde dehydrogenase, dimeric NADP-preferring	P30838	ALDH3A1	NA	Wald ratio	1	-0.097	0.039	1.297E-02	0.610	pan	SARS-CoV-2 infection
19376_74	NNMT	Nicotinamide N-methyltransferase	P40261	NNMT	NA	Wald ratio	1	0.199	0.080	1.303E-02	0.610	pan	SARS-CoV-2 infection
3348_49	BMP-1	Bone morphogenetic protein 1	P13497	BMP1	NA	Wald ratio	1	0.183	0.074	1.311E-02	0.610	pan	SARS-CoV-2 infection
4430_44	Collectin Kidney 1	Collectin-11	Q9BWP8	COLEC11	NA	Inverse variance weighted	11	0.069	0.028	1.445E-02	0.628	pan	SARS-CoV-2 infection
16818_200	CDCP1	CUB domain-containing protein 1	Q9H5V8	CDCP1	NA	Inverse variance weighted	5	-0.093	0.038	1.451E-02	0.628	pan	SARS-CoV-2 infection
3600_2	Chitotriosidase-1	Chitotriosidase-1	Q13231	CHIT1	NA	Inverse variance weighted	8	0.022	0.009	1.494E-02	0.628	pan	SARS-CoV-2 infection
9848_22	Cyclin H	Cyclin-H	P51946	CCNH	NA	Wald ratio	1	-0.141	0.058	1.513E-02	0.628	pan	SARS-CoV-2 infection
18891_98	GBP2	Guanylate-binding protein 2	P32456	GBP2	NA	Wald ratio	1	-0.163	0.067	1.520E-02	0.628	pan	SARS-CoV-2 infection
6372_7	YBOX2	Y-box-binding protein 2	Q9Y2T7	YBX2	NA	Inverse variance weighted	2	0.189	0.078	1.543E-02	0.628	pan	SARS-CoV-2 infection
5716_49	CSAG1	Putative chondrosarcoma-associated gene 1 protein	Q6PB30	CSAG1	NA	Inverse variance weighted	3	0.114	0.047	1.551E-02	0.628	pan	SARS-CoV-2 infection
19207_119	ADK	Adenosine kinase	P55263	ADK	NA	Inverse variance weighted	2	0.123	0.051	1.696E-02	0.667	pan	SARS-CoV-2 infection
6574_11	FAIM3	Fas apoptotic inhibitory molecule 3	O60667	FCMR	NA	Inverse variance weighted	3	-0.133	0.056	1.759E-02	0.667	pan	SARS-CoV-2 infection
3617_80	HGFA	Hepatocyte growth factor activator	Q04756	HGFAC	NA	Inverse variance weighted	12	0.020	0.009	1.767E-02	0.667	pan	SARS-CoV-2 infection
13545_97	EIF1A	Probable RNA-binding protein EIF1AD	Q8N9N8	EIF1AD	NA	Inverse variance weighted	3	0.112	0.047	1.847E-02	0.667	pan	SARS-CoV-2 infection
10534_40	PARP:BRCT domain	Poly [ADP-ribose] polymerase 1:BRCA1 C-terminal:BRCA1 C-terminus domian	P09874	PARP1	NA	Wald ratio	1	-0.241	0.102	1.852E-02	0.667	pan	SARS-CoV-2 infection
5810_25	Cripto	Teratocarcinoma-derived growth factor 1	P13385	TDGF1	NA	Inverse variance weighted	20	-0.037	0.016	1.863E-02	0.667	pan	SARS-CoV-2 infection
4140_3	IL-7	Interleukin-7	P13232	IL7	NA	Wald ratio	1	0.239	0.102	1.877E-02	0.667	pan	SARS-CoV-2 infection
9002_36	SPA11	Serpin A11	Q86U17	SERPINA11	NA	Inverse variance weighted	7	-0.063	0.027	1.913E-02	0.667	pan	SARS-CoV-2 infection
9754_33	Quinone reductase 2	Ribosyldihydronicotinamide dehydrogenase [quinone]	P16083	NQO2	NA	Inverse variance weighted	9	0.019	0.008	1.923E-02	0.667	pan	SARS-CoV-2 infection
3773_15	sTie-2	Angiopoietin-1 receptor, soluble	Q02763	TEK	NA	Inverse variance weighted	5	0.049	0.021	1.966E-02	0.672	pan	SARS-CoV-2 infection
15503_20	Lefty-A	Left-right determination factor 2	O00292	LEFTY2	NA	Inverse variance weighted	12	0.025	0.011	2.113E-02	0.705	pan	SARS-CoV-2 infection
6462_12	TIMP-4	Metalloproteinase inhibitor 4	Q99727	TIMP4	NA	Inverse variance weighted	8	-0.031	0.014	2.159E-02	0.705	pan	SARS-CoV-2 infection
18832_65	SAA2	Serum amyloid A-2 protein	P0DJI9	SAA2	NA	Inverse variance weighted	3	-0.123	0.054	2.160E-02	0.705	pan	SARS-CoV-2 infection
7769_29	3BP2	SH3 domain-binding protein 2	P78314	SH3BP2	NA	Inverse variance weighted	2	0.070	0.030	2.198E-02	0.707	pan	SARS-CoV-2 infection
7849_3	Glutaminyl cyclase	Glutaminyl-peptide cyclotransferase	Q16769	QPCT	NA	Inverse variance weighted	8	-0.042	0.018	2.238E-02	0.709	pan	SARS-CoV-2 infection
12356_65	Sorcin	Sorcin	P30626	SRI	NA	Wald ratio	1	-0.096	0.042	2.315E-02	0.721	pan	SARS-CoV-2 infection
16857_2	RAB2A	Ras-related protein Rab-2A	P61019	RAB2A	NA	Wald ratio	1	0.118	0.052	2.377E-02	0.721	pan	SARS-CoV-2 infection
4834_61	Epithelial cell kinase	Ephrin type-A receptor 2	P29317	EPHA2	NA	Inverse variance weighted	5	-0.060	0.027	2.380E-02	0.721	pan	SARS-CoV-2 infection
5128_53	SLAF6	SLAM family member 6	Q96DU3	SLAMF6	NA	Wald ratio	1	-0.061	0.027	2.407E-02	0.721	pan	SARS-CoV-2 infection
17680_12	EPHB1	Ephrin type-B receptor 1	P54762	EPHB1	NA	Inverse variance weighted	3	0.031	0.014	2.473E-02	0.731	pan	SARS-CoV-2 infection
11514_196	CD59	CD59 glycoprotein	P13987	CD59	NA	Inverse variance weighted	3	0.055	0.025	2.545E-02	0.733	pan	SARS-CoV-2 infection
8464_31	RSPO4	R-spondin-4	Q2I0M5	RSPO4	NA	Inverse variance weighted	3	0.098	0.044	2.549E-02	0.733	pan	SARS-CoV-2 infection
11369_23	ADHX	Alcohol dehydrogenase class-3	P11766	ADH5	NA	Inverse variance weighted	4	-0.125	0.057	2.712E-02	0.737	pan	SARS-CoV-2 infection
11542_11	TM230	Transmembrane protein 230	Q96A57	TMEM230	NA	Wald ratio	1	-0.155	0.070	2.721E-02	0.737	pan	SARS-CoV-2 infection
12437_18	ULK3	Serine/threonine-protein kinase ULK3	Q6PHR2	ULK3	NA	Wald ratio	1	0.232	0.105	2.727E-02	0.737	pan	SARS-CoV-2 infection
9468_8	Lectin, mannose-binding 2	Vesicular integral-membrane protein VIP36	Q12907	LMAN2	NA	Wald ratio	1	0.128	0.058	2.779E-02	0.737	pan	SARS-CoV-2 infection
12703_6	NEK7	Serine/threonine-protein kinase Nek7	Q8TDX7	NEK7	NA	Wald ratio	1	-0.082	0.037	2.780E-02	0.737	pan	SARS-CoV-2 infection
6625_31	ATAD1	ATPase family AAA domain-containing protein 1	Q8NBU5	ATAD1	NA	Wald ratio	1	-0.080	0.037	2.782E-02	0.737	pan	SARS-CoV-2 infection
9715_15	IGSF3	Immunoglobulin superfamily member 3	O75054	IGSF3	NA	Inverse variance weighted	5	0.084	0.038	2.806E-02	0.737	pan	SARS-CoV-2 infection
5400_52	sLeptin R	Leptin receptor, soluble	P48357	LEPR	NA	Inverse variance weighted	18	0.022	0.010	2.831E-02	0.737	pan	SARS-CoV-2 infection
19448_104	UPP1	Uridine phosphorylase 1	Q16831	UPP1	NA	Wald ratio	1	0.115	0.053	2.906E-02	0.742	pan	SARS-CoV-2 infection
3339_33	TSP2	Thrombospondin-2	P35442	THBS2	NA	Inverse variance weighted	8	-0.025	0.012	2.952E-02	0.742	pan	SARS-CoV-2 infection
3593_72	Caspase-3	Caspase-3	P42574	CASP3	NA	Wald ratio	1	0.089	0.041	2.953E-02	0.742	pan	SARS-CoV-2 infection
2974_61	contactin-1	Contactin-1	Q12860	CNTN1	NA	Inverse variance weighted	8	0.037	0.017	3.169E-02	0.787	pan	SARS-CoV-2 infection
8033_1	DJC18	DnaJ homolog subfamily C member 18	Q9H819	DNAJC18	NA	Wald ratio	1	0.191	0.089	3.218E-02	0.787	pan	SARS-CoV-2 infection
12558_3	UBS3B	Ubiquitin-associated and SH3 domain-containing protein B	Q8TF42	UBASH3B	NA	Inverse variance weighted	2	-0.097	0.046	3.259E-02	0.787	pan	SARS-CoV-2 infection
9962_1	MUCDL	Cadherin-related family member 5	Q9HBB8	CDHR5	NA	Wald ratio	1	-0.184	0.086	3.286E-02	0.787	pan	SARS-CoV-2 infection
19188_21	NAP2L	nucleosome assembly protein 1-like 4	Q99733	NAP1L4	NA	Inverse variance weighted	3	0.105	0.049	3.349E-02	0.787	pan	SARS-CoV-2 infection
10600_24	CLGN	Calmegin	O14967	CLGN	NA	Inverse variance weighted	3	-0.107	0.050	3.384E-02	0.787	pan	SARS-CoV-2 infection
9478_69	KPRA	Phosphoribosyl pyrophosphate synthase-associated protein 1	Q14558	PRPSAP1	NA	Wald ratio	1	-0.135	0.064	3.385E-02	0.787	pan	SARS-CoV-2 infection
8480_29	FBLN3	EGF-containing fibulin-like extracellular matrix protein 1	Q12805	EFEMP1	NA	Inverse variance weighted	3	-0.068	0.033	3.723E-02	0.815	pan	SARS-CoV-2 infection

15449_33	TIM-4	T-cell immunoglobulin and mucin domain-containing protein 4	Q96H15	TIMD4	NA	Inverse variance weighted	19	-0.044	0.021	3.730E-02	0.815	pan	SARS-CoV-2 infection
9385_4	GAA	Lysosomal alpha-glucosidase	P10253	GAA	NA	Inverse variance weighted	6	-0.035	0.017	3.861E-02	0.815	pan	SARS-CoV-2 infection
2952_75	IGF-I	Insulin-like growth factor I	P05019	IGF1	NA	Inverse variance weighted	2	0.155	0.075	3.970E-02	0.815	pan	SARS-CoV-2 infection
11211_7	TBCE	Tubulin-specific chaperone E	Q15813	TBCE	NA	Inverse variance weighted	2	-0.085	0.041	4.065E-02	0.815	pan	SARS-CoV-2 infection
13959_7	LAP	Cytosol aminopeptidase	P28838	LAP3	NA	Wald ratio	1	0.211	0.103	4.066E-02	0.815	pan	SARS-CoV-2 infection
4562_1	NXPH1	Neurexophilin-1	P58417	NXPH1	NA	Wald ratio	1	0.198	0.097	4.078E-02	0.815	pan	SARS-CoV-2 infection
8369_102	DAG1	Dystroglycan	Q14118	DAG1	NA	Wald ratio	1	0.168	0.083	4.219E-02	0.815	pan	SARS-CoV-2 infection
8074_32	TMM70	Transmembrane protein 70, mitochondrial	Q9BUB7	TMEM70	NA	Inverse variance weighted	3	-0.127	0.063	4.221E-02	0.815	pan	SARS-CoV-2 infection
16302_11	SPLC2	BPI fold-containing family A member 2	Q96DR5	BPIFA2	NA	Wald ratio	1	-0.149	0.073	4.240E-02	0.815	pan	SARS-CoV-2 infection
11196_31	Collagen alpha-3(VI):BPTI/Kunitz inhibitor	Collagen alpha-3(VI) chain:Bovine pancreatic trypsin inhibitor/Kunitz inhibitor domain, isoform 1	P12111	COL6A3	NA	Inverse variance weighted	2	0.164	0.081	4.256E-02	0.815	pan	SARS-CoV-2 infection
12501_10	TBCA	Tubulin-specific chaperone A	O75347	TBCA	NA	Inverse variance weighted	4	0.082	0.040	4.286E-02	0.815	pan	SARS-CoV-2 infection
3210_1	METAP1	Methionine aminopeptidase 1	P53582	METAP1	NA	Wald ratio	1	0.206	0.102	4.339E-02	0.815	pan	SARS-CoV-2 infection
9876_20	aldolase C	Fructose-bisphosphate aldolase C	P09972	ALDOC	NA	Inverse variance weighted	3	-0.098	0.049	4.368E-02	0.815	pan	SARS-CoV-2 infection
13948_50	GABR2:ECD	Gamma-aminobutyric acid type B receptor subunit 2:Extracellular domain	O75899	GABBR2	NA	Wald ratio	1	0.181	0.090	4.386E-02	0.815	pan	SARS-CoV-2 infection
5301_7	Eotaxin	Eotaxin	P51671	CCL11	NA	Inverse variance weighted	9	0.052	0.026	4.396E-02	0.815	pan	SARS-CoV-2 infection
6022_57	NELL2	Protein kinase C-binding protein NELL2	Q99435	NELL2	NA	Wald ratio	1	-0.126	0.062	4.407E-02	0.815	pan	SARS-CoV-2 infection
9594_30	ZNT5	Zinc transporter 5	Q8TAD4	SLC30A5	NA	Wald ratio	1	-0.099	0.049	4.470E-02	0.815	pan	SARS-CoV-2 infection
7198_197	FA20B	Glycosaminoglycan xylosylkinase	O75063	FAM20B	NA	Inverse variance weighted	2	-0.095	0.047	4.481E-02	0.815	pan	SARS-CoV-2 infection
17761_2	NUDT5	ADP-sugar pyrophosphatase	Q9UKK9	NUDT5	NA	Inverse variance weighted	2	-0.161	0.080	4.514E-02	0.815	pan	SARS-CoV-2 infection
17514_48	RAB21	Ras-related protein Rab-21	Q9UL25	RAB21	NA	Wald ratio	1	-0.126	0.063	4.549E-02	0.815	pan	SARS-CoV-2 infection
11356_19	DGC14	Protein DGCR14	Q96DF8	ESS2	NA	Wald ratio	1	-0.089	0.045	4.590E-02	0.815	pan	SARS-CoV-2 infection
12817_1	GEM	GTP-binding protein GEM	P55040	GEM	NA	Wald ratio	1	-0.213	0.107	4.596E-02	0.815	pan	SARS-CoV-2 infection
7956_11	MOS1	MICOS complex subunit MIC10	Q5TGZ0	MICOS10	NA	Inverse variance weighted	12	0.005	0.003	4.647E-02	0.815	pan	SARS-CoV-2 infection
19601_15	ASB9	Ankyrin repeat and SOCS box protein 9	Q96DX5	ASB9	NA	Wald ratio	1	-0.057	0.028	4.669E-02	0.815	pan	SARS-CoV-2 infection
12475_48	CLIC5	Chloride intracellular channel protein 5	Q9NZA1	CLIC5	NA	Inverse variance weighted	9	0.034	0.017	4.694E-02	0.815	pan	SARS-CoV-2 infection
4127_75	C6	Complement component C6	P13671	C6	NA	Inverse variance weighted	2	-0.071	0.036	4.844E-02	0.815	pan	SARS-CoV-2 infection
9100_32	CE046	Uncharacterized protein C5orf46	Q6UWT4	C5orf46	NA	Wald ratio	1	-0.137	0.069	4.858E-02	0.815	pan	SARS-CoV-2 infection
3035_80	IL-19	Interleukin-19	Q9UHD0	IL19	NA	Inverse variance weighted	5	0.065	0.033	4.872E-02	0.815	pan	SARS-CoV-2 infection
18188_12	GATM	Glycine amidinotransferase, mitochondrial	P50440	GATM	NA	Wald ratio	1	-0.056	0.028	4.905E-02	0.815	pan	SARS-CoV-2 infection
12387_7	PDLI4	PDZ and LIM domain protein 4	P50479	PDLIM4	NA	Wald ratio	1	-0.072	0.037	4.990E-02	0.815	pan	SARS-CoV-2 infection
6947_4	SIA10	Type 2 lactosamine alpha-2,3-sialyltransferase	Q9Y274	ST3GAL6	NA	Inverse variance weighted	31	-0.017	0.009	5.056E-02	0.815	pan	SARS-CoV-2 infection
12376_85	p19-INK4d	Cyclin-dependent kinase 4 inhibitor D	P55273	CDKN2D	NA	Wald ratio	1	-0.212	0.108	5.096E-02	0.815	pan	SARS-CoV-2 infection
9173_21	PGM1	Phosphoglucomutase-1	P36871	PGM1	NA	Wald ratio	1	0.116	0.060	5.106E-02	0.815	pan	SARS-CoV-2 infection
19129_15	MTHFSD	Methenyltetrahydrofolate synthase domain-containing protein	Q2M296	MTHFSD	NA	Inverse variance weighted	5	-0.036	0.019	5.106E-02	0.815	pan	SARS-CoV-2 infection
15641_20	TEFF1	Tomoregulin-1	Q8IYR6	TMEFF1	NA	Wald ratio	1	-0.168	0.087	5.157E-02	0.815	pan	SARS-CoV-2 infection
7210_25	Amyloid-like protein 1	Amyloid-like protein 1	P51693	APLP1	NA	Wald ratio	1	0.296	0.152	5.164E-02	0.815	pan	SARS-CoV-2 infection
4546_27	EMR2	Adhesion G protein-coupled receptor E2	Q9UHX3	ADGRE2	NA	Inverse variance weighted	6	0.026	0.013	5.175E-02	0.815	pan	SARS-CoV-2 infection
15613_16	LIPP	Pancreatic triacylglycerol lipase	P16233	PNLIP	NA	Inverse variance weighted	5	0.075	0.038	5.205E-02	0.815	pan	SARS-CoV-2 infection
18921_30	PHEX	Phosphate-regulating neutral endopeptidase	P78562	PHEX	NA	Wald ratio	1	-0.111	0.057	5.217E-02	0.815	pan	SARS-CoV-2 infection
12831_21	TESC	Calcineurin B homologous protein 3	Q96BS2	TESC	NA	Inverse variance weighted	11	0.030	0.016	5.253E-02	0.815	pan	SARS-CoV-2 infection
3890_8	LDH-H 1	L-lactate dehydrogenase B chain	P07195	LDHB	NA	Wald ratio	1	0.226	0.117	5.295E-02	0.815	pan	SARS-CoV-2 infection
4455_89	MFGM	Lactadherin	Q08431	MFGE8	NA	Inverse variance weighted	4	-0.006	0.003	5.305E-02	0.815	pan	SARS-CoV-2 infection
19267_14	GLO2	Hydroxyacylglutathione hydrolase, mitochondrial	Q16775	HAGH	NA	Wald ratio	1	0.064	0.033	5.313E-02	0.815	pan	SARS-CoV-2 infection
11547_84	MUSK	Muscle, skeletal receptor tyrosine-protein kinase	O15146	MUSK	NA	Inverse variance weighted	2	0.110	0.057	5.322E-02	0.815	pan	SARS-CoV-2 infection
8945_7	F175B	BRISC complex subunit Abro1	Q15018	ABRAXAS2	NA	Wald ratio	1	0.114	0.059	5.379E-02	0.815	pan	SARS-CoV-2 infection
8903_1	COX6C	Cytochrome c oxidase subunit 6C	P09669	COX6C	NA	Inverse variance weighted	4	0.098	0.051	5.437E-02	0.815	pan	SARS-CoV-2 infection
8969_49	CD14	Monocyte differentiation antigen CD14	P08571	CD14	NA	Wald ratio	1	-0.240	0.125	5.475E-02	0.815	pan	SARS-CoV-2 infection
4964_67	ARTS1	Endoplasmic reticulum aminopeptidase 1	Q9NZ08	ERAP1	NA	Inverse variance weighted	22	-0.018	0.009	5.494E-02	0.815	pan	SARS-CoV-2 infection
3186_2	C2	Complement C2	P06681	C2	NA	Inverse variance weighted	9	-0.049	0.026	5.613E-02	0.815	pan	SARS-CoV-2 infection
14094_29	HB-EGF	Heparin-binding EGF-like growth factor	Q99075	HBEGF	NA	Inverse variance weighted	8	-0.088	0.046	5.622E-02	0.815	pan	SARS-CoV-2 infection
6342_10	Nephronectin	Nephronectin	Q6UXI9	NPNT	NA	Inverse variance weighted	4	0.046	0.024	5.639E-02	0.815	pan	SARS-CoV-2 infection
17726_3	SAR1A	GTP-binding protein SAR1a	Q9NR31	SAR1A	NA	Inverse variance weighted	2	0.099	0.052	5.647E-02	0.815	pan	SARS-CoV-2 infection
18321_38	MTDC	Bifunctional methylenetetrahydrofolate dehydrogenase/cyclohydrolase, mitochondrial	P13995	MTHFD2	NA	Inverse variance weighted	4	0.060	0.032	5.689E-02	0.815	pan	SARS-CoV-2 infection
18295_102	GRHPR	Glyoxylate reductase/hydroxypyruvate reductase	Q9UBQ7	GRHPR	NA	Inverse variance weighted	3	-0.033	0.017	5.713E-02	0.815	pan	SARS-CoV-2 infection
7898_29	MDM1	Nuclear protein MDM1	Q8TC05	MDM1	NA	Inverse variance weighted	2	-0.103	0.054	5.749E-02	0.815	pan	SARS-CoV-2 infection
19372_7	MDGA2	MAM domain-containing glycosylphosphatidylinositol anchor protein 2	Q7Z553	MDGA2	NA	Inverse variance weighted	20	-0.014	0.007	5.761E-02	0.815	pan	SARS-CoV-2 infection
10606_34	TOIP1:Perinuclear	Torsin-1A-interacting protein 1:Perinuclear domain	Q5JTV8	TOR1AIP1	NA	Wald ratio	1	0.229	0.121	5.816E-02	0.815	pan	SARS-CoV-2 infection
14012_17	LGP2	Probable ATP-dependent RNA helicase DHX58	Q96C10	DHX58	NA	Inverse variance weighted	3	-0.065	0.034	5.839E-02	0.815	pan	SARS-CoV-2 infection
9201_13	Transgelin-2	Transgelin-2	P37802	TAGLN2	NA	Wald ratio	1	-0.194	0.103	5.866E-02	0.815	pan	SARS-CoV-2 infection
6641_60	PolyUbiquitin K48	PolyUbiquitin K48-linked	P0CG47	UBB	NA	Wald ratio	1	-0.184	0.098	5.875E-02	0.815	pan	SARS-CoV-2 infection
6955_68	SNX1	Sorting nexin-1	Q13596	SNX1	NA	Wald ratio	1	-0.072	0.038	5.890E-02	0.815	pan	SARS-CoV-2 infection
15678_71	DKK2	Dickkopf-related protein 2	Q9UBU2	DKK2	NA	Inverse variance weighted	2	-0.166	0.088	5.918E-02	0.815	pan	SARS-CoV-2 infection
2991_9	IL-1 sRI	Interleukin-1 receptor type 1	P14778	IL1R1	NA	Inverse variance weighted	8	-0.047	0.025	5.948E-02	0.815	pan	SARS-CoV-2 infection
4968_50	CAPG	Macrophage-capping protein	P40121	CAPG	NA	Inverse variance weighted	7	0.027	0.015	5.959E-02	0.815	pan	SARS-CoV-2 infection
4568_17	SLIK5	SLIT and NTRK-like protein 5	O94991	SLITRK5	NA	Inverse variance weighted	4	0.063	0.033	6.012E-02	0.815	pan	SARS-CoV-2 infection
17682_1	CD46	Membrane cofactor protein	P15529	CD46	NA	Inverse variance weighted	3	0.049	0.026	6.015E-02	0.815	pan	SARS-CoV-2 infection
2860_19	Karyopherin-a2	Importin subunit alpha-1	P52292	KPNA2	NA	Inverse variance weighted	10	-0.061	0.032	6.049E-02	0.815	pan	SARS-CoV-2 infection
6493_9	CBPZ	Carboxypeptidase Z	Q66K79	CPZ	NA	Inverse variance weighted	2	-0.125	0.067	6.092E-02	0.815	pan	SARS-CoV-2 infection
8340_9	DB110	Beta-defensin 110	Q30KQ9	DEFB110	NA	Wald ratio	1	-0.178	0.095	6.119E-02	0.815	pan	SARS-CoV-2 infection
12637_7	CACP	Carnitine O-acetyltransferase	P43155	CRAT	NA	Wald ratio	1	0.103	0.055	6.207E-02	0.815	pan	SARS-CoV-2 infection
10815_2	HABP4	Intracellular hyaluronan-binding protein 4	Q5JVS0	HABP4	NA	Inverse variance weighted	5	-0.058	0.031	6.210E-02	0.815	pan	SARS-CoV-2 infection
8288_27	b2-Glycoprotein I	Beta-2-glycoprotein 1	P02749	APOH	NA	Inverse variance weighted	6	0.030	0.016	6.258E-02	0.815	pan	SARS-CoV-2 infection
5636_10	MFAP4	Microfibril-associated glycoprotein 4	P55083	MFAP4	NA	Inverse variance weighted	11	0.045	0.024	6.280E-02	0.815	pan	SARS-CoV-2 infection
16809_1	NDKM	Nucleoside diphosphate kinase, mitochondrial	O00746	NME4	NA	Wald ratio	1	0.128	0.069	6.319E-02	0.815	pan	SARS-CoV-2 infection
5900_11	HINT1	Histidine triad nucleotide-binding protein 1	P49773	HINT1	NA	Wald ratio	1	0.126	0.068	6.335E-02	0.815	pan	SARS-CoV-2 infection
2831_29	Kallikrein 11	Kallikrein-11	Q9UBX7	KLK11	NA	Inverse variance weighted	8	0.040	0.022	6.454E-02	0.825	pan	SARS-CoV-2 infection
7059_14	LIRA6	Leukocyte immunoglobulin-like receptor subfamily A member 6	Q6PI73	LILRA6	NA	Inverse variance weighted	7	0.047	0.025	6.522E-02	0.825	pan	SARS-CoV-2 infection
6551_94	Vaspin	Serpin A12	Q8IW75	SERPINA12	NA	Inverse variance weighted	6	-0.024	0.013	6.545E-02	0.825	pan	SARS-CoV-2 infection

16606_85	Aldose reductase	Aldose reductase	P15121	AKR1B1	NA	Inverse variance weighted	2	0.080	0.044	6.568E-02	0.825	pan	SARS-CoV-2 infection
14205_6	HEXI2	Protein HEXIM2	Q96MH2	HEXIM2	NA	Wald ratio	1	0.203	0.111	6.643E-02	0.825	pan	SARS-CoV-2 infection
15487_164	carboxylesterase, liver	Liver carboxylesterase 1	P23141	CES1	NA	Wald ratio	1	-0.198	0.108	6.645E-02	0.825	pan	SARS-CoV-2 infection
13133_73	LTBP4	Latent-transforming growth factor beta-binding protein 4	Q8N2S1	LTBP4	NA	Inverse variance weighted	2	-0.127	0.070	6.842E-02	0.845	pan	SARS-CoV-2 infection
14131_37	EFNB2:ECD	Ephrin-B2:Extracellular domain	P52799	EFNB2	NA	Inverse variance weighted	2	0.116	0.064	6.976E-02	0.850	pan	SARS-CoV-2 infection
18231_147	PRND	Prion-like protein doppel	Q9UKY0	PRND	NA	Wald ratio	1	0.165	0.091	7.041E-02	0.850	pan	SARS-CoV-2 infection
5957_30	Somatostatin-28	Somatostatin-28	P61278	SST	NA	Wald ratio	1	0.181	0.100	7.052E-02	0.850	pan	SARS-CoV-2 infection
2939_10	Artemin	Artemin	Q5T4W7	ARTN	NA	Wald ratio	1	0.195	0.108	7.055E-02	0.850	pan	SARS-CoV-2 infection
8841_65	CILP2	Cartilage intermediate layer protein 2	Q8IUL8	CILP2	NA	Inverse variance weighted	2	0.101	0.056	7.076E-02	0.850	pan	SARS-CoV-2 infection
15486_126	ABP1	Amiloride-sensitive amine oxidase [copper-containing]	P19801	AOC1	NA	Inverse variance weighted	12	-0.015	0.008	7.176E-02	0.857	pan	SARS-CoV-2 infection
2742_68	Siglec-7	Sialic acid-binding Ig-like lectin 7	Q9Y286	SIGLEC7	NA	Inverse variance weighted	3	0.060	0.034	7.357E-02	0.865	pan	SARS-CoV-2 infection
6899_37	PHS2	Pterin-4-alpha-carbinolamine dehydratase 2	Q9H0N5	PCBD2	NA	Inverse variance weighted	4	0.088	0.049	7.412E-02	0.865	pan	SARS-CoV-2 infection
14618_26	ECOP	Vesicular, overexpressed in cancer, prosurvival protein 1	Q96AW1	VOPP1	NA	Inverse variance weighted	11	-0.038	0.021	7.419E-02	0.865	pan	SARS-CoV-2 infection
15333_11	SDF2	Stromal cell-derived factor 2	Q99470	SDF2	NA	Inverse variance weighted	8	0.064	0.036	7.442E-02	0.865	pan	SARS-CoV-2 infection
5765_53	DB121	Beta-defensin 121	Q5J5C9	DEFB121	NA	Wald ratio	1	0.203	0.114	7.481E-02	0.865	pan	SARS-CoV-2 infection
5236_2	NR1D1	Nuclear receptor subfamily 1 group D member 1	P20393	NR1D1	NA	Wald ratio	1	-0.210	0.118	7.516E-02	0.865	pan	SARS-CoV-2 infection
6528_95	EXTL2	Exostosin-like 2	Q9UBQ6	EXTL2	NA	Wald ratio	1	0.184	0.103	7.586E-02	0.865	pan	SARS-CoV-2 infection
5116_62	ROBO2	Roundabout homolog 2	Q9HCK4	ROBO2	NA	Inverse variance weighted	2	0.095	0.053	7.638E-02	0.865	pan	SARS-CoV-2 infection
7124_18	IL-21	Interleukin-21	Q9HBE4	IL21	NA	Wald ratio	1	0.121	0.068	7.687E-02	0.865	pan	SARS-CoV-2 infection
13566_2	SSU72	RNA polymerase II subunit A C-terminal domain phosphatase SSU72	Q9NP77	SSU72	NA	Inverse variance weighted	6	-0.041	0.023	7.712E-02	0.865	pan	SARS-CoV-2 infection
3212_30	ASAH2	Neutral ceramidase	Q9NR71	ASAH2	NA	Inverse variance weighted	10	-0.023	0.013	7.762E-02	0.865	pan	SARS-CoV-2 infection
14158_17	Annexin V	Annexin A5	P08758	ANXA5	NA	Wald ratio	1	0.063	0.036	7.792E-02	0.865	pan	SARS-CoV-2 infection
3314_74	GFRa-1	GDNF family receptor alpha-1	P56159	GFRA1	NA	Inverse variance weighted	3	0.043	0.025	7.799E-02	0.865	pan	SARS-CoV-2 infection
15367_38	LPLC1	BPI fold-containing family B member 1	Q8TDL5	BPIFB1	NA	Inverse variance weighted	15	-0.023	0.013	7.837E-02	0.865	pan	SARS-CoV-2 infection
10605_22	APMAP	Adipocyte plasma membrane-associated protein	Q9HDC9	APMAP	NA	Wald ratio	1	0.147	0.084	7.838E-02	0.865	pan	SARS-CoV-2 infection
5124_69	sICAM-5	Intercellular adhesion molecule 5	Q9UMF0	ICAM5	NA	Inverse variance weighted	7	-0.058	0.033	7.910E-02	0.869	pan	SARS-CoV-2 infection
8888_33	CX068	Small integral membrane protein 9	A6NGZ8	SMIM9	NA	Inverse variance weighted	2	-0.042	0.024	7.967E-02	0.869	pan	SARS-CoV-2 infection
4971_1	CATZ	Cathepsin Z	Q9UBR2	CTSZ	NA	Inverse variance weighted	6	-0.032	0.018	8.011E-02	0.869	pan	SARS-CoV-2 infection
7139_14	SLIK4	SLIT and NTRK-like protein 4	Q8IW52	SLITRK4	NA	Wald ratio	1	0.151	0.087	8.070E-02	0.869	pan	SARS-CoV-2 infection
2999_6	LSAMP	Limbic system-associated membrane protein	Q13449	LSAMP	NA	Inverse variance weighted	5	-0.047	0.027	8.092E-02	0.869	pan	SARS-CoV-2 infection
2743_5	Sonic Hedgehog	Sonic hedgehog protein	Q15465	SHH	NA	Inverse variance weighted	2	-0.073	0.042	8.112E-02	0.869	pan	SARS-CoV-2 infection
9094_5	CLC4C	C-type lectin domain family 4 member C	Q8WTT0	CLEC4C	NA	Inverse variance weighted	19	-0.045	0.026	8.241E-02	0.879	pan	SARS-CoV-2 infection
7950_142	BTNL9	Butyrophilin-like protein 9	Q6UXG8	BTNL9	NA	Wald ratio	1	-0.130	0.075	8.452E-02	0.892	pan	SARS-CoV-2 infection
3858_5	PPAC	Low molecular weight phosphotyrosine protein phosphatase	P24666	ACP1	NA	Inverse variance weighted	9	-0.016	0.009	8.509E-02	0.892	pan	SARS-CoV-2 infection
5202_4	PPID	Peptidyl-prolyl cis-trans isomerase D	Q08752	PPID	NA	Inverse variance weighted	5	-0.030	0.018	8.558E-02	0.892	pan	SARS-CoV-2 infection
7761_125	CHKB	Choline/ethanolamine kinase	Q9Y259	CHKB	NA	Inverse variance weighted	3	0.076	0.044	8.576E-02	0.892	pan	SARS-CoV-2 infection
5691_2	CRLD2	Cysteine-rich secretory protein LCCL domain-containing 2	Q9H0B8	CRISPLD2	NA	Inverse variance weighted	11	0.033	0.020	8.611E-02	0.892	pan	SARS-CoV-2 infection
17738_7	CRNN	Cornulin	Q9UBG3	CRNN	NA	Wald ratio	1	-0.095	0.055	8.613E-02	0.892	pan	SARS-CoV-2 infection
6923_1	PLOD2	Procollagen-lysine,2-oxoglutarate 5-dioxygenase 2	O00469	PLOD2	NA	Inverse variance weighted	3	0.077	0.045	8.675E-02	0.894	pan	SARS-CoV-2 infection
13936_24	PGK2	Phosphoglycerate kinase 2	P07205	PGK2	NA	Wald ratio	1	0.106	0.062	8.755E-02	0.894	pan	SARS-CoV-2 infection
18873_8	CEAM8	Carcinoembryonic antigen-related cell adhesion molecule 8	P31997	CEACAM8	NA	Inverse variance weighted	12	0.050	0.029	8.787E-02	0.894	pan	SARS-CoV-2 infection
5078_82	EphB6	Ephrin type-B receptor 6	O15197	EPHB6	NA	Inverse variance weighted	4	-0.039	0.023	8.802E-02	0.894	pan	SARS-CoV-2 infection
13481_24	TCEA2	Transcription elongation factor A protein 2	Q15560	TCEA2	NA	Wald ratio	1	0.088	0.051	8.836E-02	0.894	pan	SARS-CoV-2 infection
13597_20	RAB31	Ras-related protein Rab-31	Q13636	RAB31	NA	Inverse variance weighted	3	0.136	0.080	8.924E-02	0.895	pan	SARS-CoV-2 infection
8352_26	SIG12:Ig-like C2-type 2	Sialic acid-binding Ig-like lectin 12:Ig-like C2-type 2 domain, Isoform short	Q96PQ1	SIGLEC12	NA	Wald ratio	1	0.186	0.110	9.089E-02	0.895	pan	SARS-CoV-2 infection
8397_147	QSOX2	Sulfhydryl oxidase 2	Q6ZRP7	QSOX2	NA	Inverse variance weighted	16	0.018	0.011	9.100E-02	0.895	pan	SARS-CoV-2 infection
7948_129	GLTD2	Glycolipid transfer protein domain-containing protein 2	A6NH11	GLTPD2	NA	Inverse variance weighted	3	0.035	0.021	9.114E-02	0.895	pan	SARS-CoV-2 infection
6252_62	Secretoglobin family 3A member 1	Secretoglobin family 3A member 1	Q96QR1	SCGB3A1	NA	Inverse variance weighted	28	0.034	0.020	9.119E-02	0.895	pan	SARS-CoV-2 infection
2201_17	Endostatin	Endostatin	P39060	COL18A1	NA	Inverse variance weighted	4	-0.045	0.027	9.151E-02	0.895	pan	SARS-CoV-2 infection
17408_2	PMM1	Phosphomannomutase 1	Q92871	PMM1	NA	Wald ratio	1	0.159	0.095	9.193E-02	0.895	pan	SARS-CoV-2 infection
14153_8	Ephrin-A3	Ephrin-A3	P52797	EFNA3	NA	Wald ratio	1	0.147	0.088	9.234E-02	0.895	pan	SARS-CoV-2 infection
13473_55	IP3KA	Inositol-trisphosphate 3-kinase A	P23677	ITPKA	NA	Wald ratio	1	0.114	0.068	9.277E-02	0.895	pan	SARS-CoV-2 infection
14208_3	RET7	Retinoid-binding protein 7	Q96R05	RBP7	NA	Inverse variance weighted	2	0.066	0.039	9.348E-02	0.895	pan	SARS-CoV-2 infection
19504_22	KTHY	Thymidylate kinase	P23919	DTYMK	NA	Inverse variance weighted	3	0.102	0.061	9.356E-02	0.895	pan	SARS-CoV-2 infection
5634_39	OFUT1	GDP-fucose protein O-fucosyltransferase 1	Q9H488	POFUT1	NA	Inverse variance weighted	7	0.025	0.015	9.369E-02	0.895	pan	SARS-CoV-2 infection
13130_150	HXK2	Hexokinase-2	P52789	HK2	NA	Inverse variance weighted	2	-0.101	0.060	9.392E-02	0.895	pan	SARS-CoV-2 infection
17694_32	PSME2	Proteasome activator complex subunit 2	Q9UL46	PSME2	NA	Inverse variance weighted	3	-0.041	0.024	9.413E-02	0.895	pan	SARS-CoV-2 infection
14150_7	IL-1F6	Interleukin-36 alpha	Q9UHA7	IL36A	NA	Inverse variance weighted	2	-0.082	0.049	9.561E-02	0.905	pan	SARS-CoV-2 infection
17748_21	QORX	Quinone oxidoreductase PIG3	Q53FA7	TP53I3	NA	Inverse variance weighted	10	-0.029	0.018	9.669E-02	0.908	pan	SARS-CoV-2 infection
4157_2	Thrombin	Thrombin	P00734	F2	NA	Inverse variance weighted	7	0.055	0.033	9.702E-02	0.908	pan	SARS-CoV-2 infection
6965_19	CNTP2	Contactin-associated protein-like 2	Q9UHC6	CNTNAP2	NA	Inverse variance weighted	6	0.020	0.012	9.739E-02	0.908	pan	SARS-CoV-2 infection
12593_33	PDRG1	p53 and DNA damage-regulated protein 1	Q9NUG6	PDRG1	NA	Wald ratio	1	-0.148	0.089	9.758E-02	0.908	pan	SARS-CoV-2 infection
5437_63	FABP	Fatty acid-binding protein, heart	P05413	FABP3	NA	Inverse variance weighted	2	-0.115	0.070	9.976E-02	0.913	pan	SARS-CoV-2 infection
19279_42	CRBP	Retinol-binding protein 1	P09455	RBP1	NA	Wald ratio	1	0.118	0.072	9.982E-02	0.913	pan	SARS-CoV-2 infection
11130_158	CACB4	Voltage-dependent L-type calcium channel subunit beta-4	O00305	CACNB4	NA	Wald ratio	1	0.069	0.042	1.007E-01	0.913	pan	SARS-CoV-2 infection
14121_24	TRAIL R4	Tumor necrosis factor receptor superfamily member 10D	Q9UBN6	TNFRSF10D	NA	Wald ratio	1	0.188	0.114	1.011E-01	0.913	pan	SARS-CoV-2 infection
13044_5	TS101	Tumor susceptibility gene 101 protein	Q99816	TSG101	NA	Inverse variance weighted	2	-0.079	0.048	1.014E-01	0.913	pan	SARS-CoV-2 infection
6904_14	LRRT2	Leucine-rich repeat transmembrane neuronal protein 2	O43300	LRRTM2	NA	Inverse variance weighted	3	0.092	0.056	1.017E-01	0.913	pan	SARS-CoV-2 infection
3508_78	MDC	C-C motif chemokine 22	O00626	CCL22	NA	Inverse variance weighted	9	-0.034	0.021	1.018E-01	0.913	pan	SARS-CoV-2 infection
2966_65	SCGF-beta	Stem cell growth factor-beta	Q9Y240	CLEC11A	NA	Inverse variance weighted	2	0.139	0.085	1.020E-01	0.913	pan	SARS-CoV-2 infection
7161_25	G6PE	GDH/6PGL endoplasmic bifunctional protein	O95479	H6PD	NA	Inverse variance weighted	19	-0.018	0.011	1.021E-01	0.913	pan	SARS-CoV-2 infection
18173_11	ARK73	Aflatoxin B1 aldehyde reductase member 3	O95154	AKR7A3	NA	Inverse variance weighted	6	0.031	0.019	1.023E-01	0.913	pan	SARS-CoV-2 infection
15316_262	TXN4B	Thioredoxin-like protein 4B	Q9NX01	TXNL4B	NA	Inverse variance weighted	3	0.088	0.054	1.035E-01	0.916	pan	SARS-CoV-2 infection
8334_65	TCP11	T-complex protein 11 homolog	Q8WWU5	TCP11	NA	Wald ratio	1	0.109	0.067	1.035E-01	0.916	pan	SARS-CoV-2 infection
3280_49	Aggrecan	Aggrecan core protein	P16112	ACAN	NA	Inverse variance weighted	3	0.056	0.034	1.042E-01	0.919	pan	SARS-CoV-2 infection
8840_61	C1s	Complement C1s subcomponent	P09871	C1S	NA	Inverse variance weighted	3	0.094	0.058	1.048E-01	0.920	pan	SARS-CoV-2 infection
6461_54	Apo C-III	Apolipoprotein C-III	P02656	APOC3	NA	Inverse variance weighted	6	-0.061	0.038	1.071E-01	0.936	pan	SARS-CoV-2 infection
4254_6	NUDC3	NudC domain-containing protein 3	Q8IVD9	NUDCD3	NA	Wald ratio	1	0.116	0.072	1.080E-01	0.937	pan	SARS-CoV-2 infection

4126_22	BPI	Bactericidal permeability-increasing protein	P17213	BPI	NA	Inverse variance weighted	13	0.026	0.016	1.087E-01	0.937	pan	SARS-CoV-2 infection
17156_72	DCAK1	Serine/threonine-protein kinase DCLK1	O15075	DCLK1	NA	Inverse variance weighted	6	0.033	0.021	1.093E-01	0.937	pan	SARS-CoV-2 infection
6221_1	ADPGK	ADP-dependent glucokinase	Q9BRR6	ADPGK	NA	Wald ratio	1	0.221	0.138	1.101E-01	0.937	pan	SARS-CoV-2 infection
4775_34	Gelsolin	Gelsolin	P06396	GSN	NA	Inverse variance weighted	3	-0.061	0.038	1.105E-01	0.937	pan	SARS-CoV-2 infection
3343_1	Aminoacylase-1	Aminoacylase-1	Q03154	ACY1	NA	Inverse variance weighted	3	0.076	0.047	1.115E-01	0.937	pan	SARS-CoV-2 infection
12366_16	CRGD	Gamma-crystallin D	P07320	CRYGD	NA	Wald ratio	1	0.069	0.043	1.117E-01	0.937	pan	SARS-CoV-2 infection
7145_1	ITIH3	Inter-alpha-trypsin inhibitor heavy chain H3	Q06033	ITIH3	NA	Inverse variance weighted	11	-0.041	0.026	1.118E-01	0.937	pan	SARS-CoV-2 infection
13093_6	SECTM1	Secreted and transmembrane protein 1	Q8WVN6	SECTM1	NA	Inverse variance weighted	2	-0.040	0.025	1.120E-01	0.937	pan	SARS-CoV-2 infection
9288_7	FKBP7	Peptidyl-prolyl cis-trans isomerase FKBP7	Q9Y680	FKBP7	NA	Inverse variance weighted	7	0.023	0.015	1.122E-01	0.937	pan	SARS-CoV-2 infection
3403_1	TPSB2	Tryptase beta-2	P20231	TPSB2	NA	Inverse variance weighted	10	0.016	0.010	1.127E-01	0.937	pan	SARS-CoV-2 infection
5109_24	Nr-CAM	Neuronal cell adhesion molecule	Q92823	NRCAM	NA	Wald ratio	1	0.126	0.079	1.127E-01	0.937	pan	SARS-CoV-2 infection
17419_17	TES	Testin	Q9UGI8	TES	NA	Wald ratio	1	-0.105	0.066	1.127E-01	0.937	pan	SARS-CoV-2 infection
19617_5	LTB4DH	Prostaglandin reductase 1	Q14914	PTGR1	NA	Inverse variance weighted	12	-0.016	0.010	1.135E-01	0.937	pan	SARS-CoV-2 infection
8992_1	TMEM2	Transmembrane protein 2	Q9UHN6	CEMIP2	NA	Inverse variance weighted	4	-0.044	0.028	1.138E-01	0.937	pan	SARS-CoV-2 infection
4867_15	VEGF121	Vascular endothelial growth factor A, isoform 121	P15692	VEGFA	NA	Inverse variance weighted	8	0.081	0.051	1.152E-01	0.937	pan	SARS-CoV-2 infection
3310_62	FCG2B	Low affinity immunoglobulin gamma Fc region receptor II-b	P31994	FCGR2B	NA	Inverse variance weighted	12	-0.028	0.018	1.152E-01	0.937	pan	SARS-CoV-2 infection
8768_4	Bcl-10	B-cell lymphoma/leukemia 10	O95999	BCL10	NA	Wald ratio	1	-0.143	0.091	1.154E-01	0.937	pan	SARS-CoV-2 infection
17327_3	CNPY3	Protein canopy homolog 3	Q9BT09	CNPY3	NA	Inverse variance weighted	2	0.072	0.046	1.155E-01	0.937	pan	SARS-CoV-2 infection
9256_78	NPTX1	Neuronal pentraxin-1	Q15818	NPTX1	NA	Inverse variance weighted	10	-0.023	0.015	1.157E-01	0.937	pan	SARS-CoV-2 infection
13090_17	S100A6	Protein S100-A6	P06703	S100A6	NA	Inverse variance weighted	3	0.097	0.062	1.163E-01	0.938	pan	SARS-CoV-2 infection
18881_7	CD97	CD97 antigen	P48960	ADGRE5	NA	Inverse variance weighted	7	-0.044	0.028	1.172E-01	0.940	pan	SARS-CoV-2 infection
11308_8	CR3L4	Cyclic AMP-responsive element-binding protein 3-like protein 4	Q8TEY5	CREB3L4	NA	Inverse variance weighted	8	0.039	0.025	1.177E-01	0.940	pan	SARS-CoV-2 infection
6629_3	HBD-1	Beta-defensin 1	P60022	DEFB1	NA	Inverse variance weighted	10	-0.019	0.012	1.178E-01	0.940	pan	SARS-CoV-2 infection
4407_10	MSP	Hepatocyte growth factor-like protein	P26927	MST1	NA	Inverse variance weighted	20	0.018	0.012	1.183E-01	0.940	pan	SARS-CoV-2 infection
18222_34	SHLB2	Endophilin-B2	Q9NR46	SH3GLB2	NA	Inverse variance weighted	3	-0.033	0.021	1.187E-01	0.940	pan	SARS-CoV-2 infection
17686_27	TBCB	Tubulin-folding cofactor B	Q99426	TBCB	NA	Wald ratio	1	0.118	0.076	1.195E-01	0.940	pan	SARS-CoV-2 infection
3029_52	DC-SIGN	CD209 antigen	Q9NNX6	CD209	NA	Inverse variance weighted	9	0.035	0.022	1.196E-01	0.940	pan	SARS-CoV-2 infection
12954_71	PPARa	Peroxisome proliferator-activated receptor alpha	Q07869	PPARA	NA	Wald ratio	1	0.114	0.074	1.211E-01	0.940	pan	SARS-CoV-2 infection
3322_52	LRIG3	Leucine-rich repeats and immunoglobulin-like domains protein 3	Q6UXM1	LRIG3	NA	Inverse variance weighted	9	-0.020	0.013	1.212E-01	0.940	pan	SARS-CoV-2 infection
19238_12	GLNA	Glutamine synthetase	P15104	GLUL	NA	Inverse variance weighted	2	-0.116	0.075	1.218E-01	0.940	pan	SARS-CoV-2 infection
17765_3	SPN1	Snurportin-1	O95149	SNUPN	NA	Wald ratio	1	0.059	0.038	1.228E-01	0.940	pan	SARS-CoV-2 infection
3026_5	Calpastatin	Calpastatin	P20810	CAST	NA	Inverse variance weighted	4	-0.097	0.063	1.237E-01	0.940	pan	SARS-CoV-2 infection
8766_29	LIRA5	Leukocyte immunoglobulin-like receptor subfamily A member 5	A6NI73	LILRA5	NA	Inverse variance weighted	3	-0.098	0.064	1.249E-01	0.940	pan	SARS-CoV-2 infection
3198_4	IDS	Iduronate 2-sulfatase	P22304	IDS	NA	Wald ratio	1	0.115	0.075	1.250E-01	0.940	pan	SARS-CoV-2 infection
3151_6	IL-2 sRa	Interleukin-2 receptor subunit alpha	P01589	IL2RA	NA	Wald ratio	1	-0.082	0.054	1.256E-01	0.940	pan	SARS-CoV-2 infection
12332_7	EF2K	Eukaryotic elongation factor 2 kinase	O00418	EEF2K	NA	Inverse variance weighted	2	0.144	0.094	1.264E-01	0.940	pan	SARS-CoV-2 infection
9884_8	PPIL1	Peptidyl-prolyl cis-trans isomerase-like 1	Q9Y3C6	PPIL1	NA	Inverse variance weighted	9	0.020	0.013	1.266E-01	0.940	pan	SARS-CoV-2 infection
5491_12	Testican-2	Testican-2	Q92563	SPOCK2	NA	Inverse variance weighted	13	-0.029	0.019	1.273E-01	0.940	pan	SARS-CoV-2 infection
13381_49	B4GT1	Beta-1,4-galactosyltransferase 1	P15291	B4GALT1	NA	Inverse variance weighted	6	-0.046	0.030	1.273E-01	0.940	pan	SARS-CoV-2 infection
16605_2	C1T9A	Complement C1q and tumor necrosis factor-related protein 9A	P0C862	C1QTNF9	NA	Wald ratio	1	-0.037	0.025	1.280E-01	0.940	pan	SARS-CoV-2 infection
17828_3	S100A14	Protein S100-A14	Q9HCY8	S100A14	NA	Wald ratio	1	-0.134	0.088	1.281E-01	0.940	pan	SARS-CoV-2 infection
5358_3	OMD	Osteomodulin	Q99983	OMD	NA	Inverse variance weighted	3	0.082	0.054	1.282E-01	0.940	pan	SARS-CoV-2 infection
15585_304	fibulin 5	Fibulin-5	Q9UBX5	FBLN5	NA	Wald ratio	1	0.094	0.062	1.292E-01	0.940	pan	SARS-CoV-2 infection
13114_50	Lumican	Lumican	P51884	LUM	NA	Inverse variance weighted	9	-0.023	0.015	1.293E-01	0.940	pan	SARS-CoV-2 infection
5628_21	SEM3G	Semaphorin-3G	Q9NS98	SEMA3G	NA	Inverse variance weighted	7	-0.047	0.031	1.294E-01	0.940	pan	SARS-CoV-2 infection
15480_2	VNN2	Vascular non-inflammatory molecule 2	O95498	VNN2	NA	Inverse variance weighted	16	0.012	0.008	1.295E-01	0.940	pan	SARS-CoV-2 infection
12697_30	PI42A	Phosphatidylinositol 5-phosphate 4-kinase type-2 alpha	P48426	PIP4K2A	NA	Wald ratio	1	0.151	0.100	1.311E-01	0.940	pan	SARS-CoV-2 infection
4159_130	Factor H	Complement factor H	P08603	CFH	NA	Wald ratio	1	-0.117	0.078	1.323E-01	0.940	pan	SARS-CoV-2 infection
9901_28	EGLN1	Egl nine homolog 1	Q9GZT9	EGLN1	NA	Inverse variance weighted	7	0.039	0.026	1.327E-01	0.940	pan	SARS-CoV-2 infection
2844_53	sTie-1	Tyrosine-protein kinase receptor Tie-1, soluble	P35590	TIE1	NA	Inverse variance weighted	16	0.023	0.016	1.330E-01	0.940	pan	SARS-CoV-2 infection
12707_26	DPYL3	Dihydropyrimidinase-related protein 3	Q14195	DPYSL3	NA	Inverse variance weighted	2	0.070	0.047	1.331E-01	0.940	pan	SARS-CoV-2 infection
5457_5	COLEC12	Collectin-12	Q5KU26	COLEC12	NA	Inverse variance weighted	12	0.034	0.023	1.340E-01	0.940	pan	SARS-CoV-2 infection
13580_2	Sperm-associated antigen 2	UDP-N-acetylhexosamine pyrophosphorylase	Q16222	UAP1	NA	Wald ratio	1	0.157	0.105	1.342E-01	0.940	pan	SARS-CoV-2 infection
18917_53	Pancreatic alpha-amylase	Pancreatic alpha-amylase	P04746	AMY2A	NA	Inverse variance weighted	7	-0.047	0.032	1.344E-01	0.940	pan	SARS-CoV-2 infection
18340_2	Proteasome beta chain	Proteasome subunit beta type-4	P28070	PSMB4	NA	Inverse variance weighted	4	-0.014	0.009	1.347E-01	0.940	pan	SARS-CoV-2 infection
12498_12	TX1B3	Tax1-binding protein 3	O14907	TAX1BP3	NA	Wald ratio	1	-0.079	0.053	1.356E-01	0.940	pan	SARS-CoV-2 infection
6895_1	TR:CD	Transferrin receptor protein 1:Cytoplasmic domain	P02786	TFRC	NA	Inverse variance weighted	3	0.060	0.040	1.365E-01	0.940	pan	SARS-CoV-2 infection
13472_35	HDHD2	Haloacid dehalogenase-like hydrolase domain-containing protein 2	Q9H0R4	HDHD2	NA	Inverse variance weighted	3	0.030	0.020	1.366E-01	0.940	pan	SARS-CoV-2 infection
3293_2	CD5L	CD5 antigen-like	O43866	CD5L	NA	Inverse variance weighted	8	0.036	0.024	1.368E-01	0.940	pan	SARS-CoV-2 infection
2730_58	MICA	MHC class I polypeptide-related sequence A	Q29983	MICA	NA	Inverse variance weighted	14	-0.023	0.015	1.373E-01	0.940	pan	SARS-CoV-2 infection
8983_7	GOLM1	Golgi membrane protein 1	Q8NBJ4	GOLM1	NA	Inverse variance weighted	5	0.080	0.054	1.377E-01	0.940	pan	SARS-CoV-2 infection
16616_137	ENOB	Beta-enolase	P13929	ENO3	NA	Inverse variance weighted	5	0.056	0.038	1.382E-01	0.940	pan	SARS-CoV-2 infection
17343_6	SNPH	Syntaphilin	O15079	SNPH	NA	Inverse variance weighted	2	0.082	0.056	1.383E-01	0.940	pan	SARS-CoV-2 infection
9312_8	AZGP1	Zinc-alpha-2-glycoprotein	P25311	AZGP1	NA	Inverse variance weighted	4	-0.091	0.061	1.385E-01	0.940	pan	SARS-CoV-2 infection
5660_51	SOD3	Extracellular superoxide dismutase [Cu-Zn]	P08294	SOD3	NA	Inverse variance weighted	16	-0.015	0.010	1.385E-01	0.940	pan	SARS-CoV-2 infection
4549_78	FUT5	Alpha-(1,3)-fucosyltransferase 5	Q11128	FUT5	NA	Inverse variance weighted	11	-0.016	0.011	1.394E-01	0.940	pan	SARS-CoV-2 infection
5740_17	ROBO1	Roundabout homolog 1	Q9Y6N7	ROBO1	NA	Inverse variance weighted	6	0.029	0.020	1.400E-01	0.940	pan	SARS-CoV-2 infection
3066_12	Galectin-3	Galectin-3	P17931	LGALS3	NA	Inverse variance weighted	6	-0.021	0.015	1.410E-01	0.940	pan	SARS-CoV-2 infection
13621_31	AP2A2	AP-2 complex subunit alpha-2	O94973	AP2A2	NA	Wald ratio	1	0.114	0.077	1.421E-01	0.940	pan	SARS-CoV-2 infection
9744_139	DNJA4	DnaJ homolog subfamily A member 4	Q8WW22	DNAJA4	NA	Wald ratio	1	-0.104	0.071	1.423E-01	0.940	pan	SARS-CoV-2 infection
11229_16	ERN1	Serine/threonine-protein kinase/endoribonuclease IRE1	O75460	ERN1	NA	Wald ratio	1	-0.194	0.132	1.424E-01	0.940	pan	SARS-CoV-2 infection
13125_45	Vitronectin	Vitronectin	P04004	VTN	NA	Inverse variance weighted	12	-0.035	0.024	1.425E-01	0.940	pan	SARS-CoV-2 infection
5654_70	TMX3	Protein disulfide-isomerase TMX3	Q96JJ7	TMX3	NA	Inverse variance weighted	10	-0.030	0.020	1.428E-01	0.940	pan	SARS-CoV-2 infection
3622_33	LGMN	Legumain	Q99538	LGMN	NA	Inverse variance weighted	4	0.048	0.033	1.430E-01	0.940	pan	SARS-CoV-2 infection
4763_31	Afamin	Afamin	P43652	AFM	NA	Inverse variance weighted	8	0.033	0.022	1.430E-01	0.940	pan	SARS-CoV-2 infection
12847_27	MCE1	mRNA-capping enzyme	O60942	RNGTT	NA	Wald ratio	1	0.200	0.137	1.436E-01	0.940	pan	SARS-CoV-2 infection
8325_37	ADH4	Alcohol dehydrogenase 4	P08319	ADH4	NA	Wald ratio	1	0.094	0.065	1.443E-01	0.940	pan	SARS-CoV-2 infection
4959_2	AGR2	Anterior gradient protein 2 homolog	O95994	AGR2	NA	Wald ratio	1	0.123	0.084	1.447E-01	0.940	pan	SARS-CoV-2 infection

5618_50	FAM3B	Protein FAM3B	P58499	FAM3B	NA	Inverse variance weighted	2	0.108	0.074	1.457E-01	0.940	pan	SARS-CoV-2 infection
9253_52	BGAT	Histo-blood group ABO system transferase	P16442	ABO	NA	Inverse variance weighted	13	0.029	0.020	1.458E-01	0.940	pan	SARS-CoV-2 infection
6506_54	TMEDA	Transmembrane emp24 domain-containing protein 10	P49755	TMED10	NA	Inverse variance weighted	6	0.046	0.032	1.459E-01	0.940	pan	SARS-CoV-2 infection
12457_10	MTND	1,2-dihydroxy-3-keto-5-methylthiopentene dioxygenase	Q9BV57	ADI1	NA	Wald ratio	1	-0.091	0.062	1.463E-01	0.940	pan	SARS-CoV-2 infection
15388_24	FcRIIIa	Low affinity immunoglobulin gamma Fc region receptor III-A	P08637	FCGR3A	NA	Inverse variance weighted	11	-0.024	0.017	1.472E-01	0.940	pan	SARS-CoV-2 infection
3043_49	ON	SPARC	P09486	SPARC	NA	Inverse variance weighted	2	0.087	0.060	1.479E-01	0.940	pan	SARS-CoV-2 infection
5947_90	Tpo	Thrombopoietin	P40225	THPO	NA	Inverse variance weighted	2	0.110	0.076	1.481E-01	0.940	pan	SARS-CoV-2 infection
3415_61	BSP	Bone sialoprotein 2	P21815	IBSP	NA	Inverse variance weighted	2	-0.103	0.071	1.484E-01	0.940	pan	SARS-CoV-2 infection
4906_35	Coagulation Factor V	Coagulation Factor V	P12259	F5	NA	Inverse variance weighted	3	-0.090	0.062	1.496E-01	0.940	pan	SARS-CoV-2 infection
6998_106	HAAH	Aspartyl/asparaginyl beta-hydroxylase	Q12797	ASPH	NA	Inverse variance weighted	3	-0.059	0.041	1.496E-01	0.940	pan	SARS-CoV-2 infection
2968_61	TNFSF15	Tumor necrosis factor ligand superfamily member 15	O95150	TNFSF15	NA	Wald ratio	1	-0.131	0.091	1.498E-01	0.940	pan	SARS-CoV-2 infection
4878_3	Coagulation Factor X	Coagulation Factor X	P00742	F10	NA	Wald ratio	1	0.154	0.107	1.499E-01	0.940	pan	SARS-CoV-2 infection
5615_62	F172A	Protein FAM172A	Q8WUF8	FAM172A	NA	Wald ratio	1	-0.102	0.071	1.501E-01	0.940	pan	SARS-CoV-2 infection
9557_5	MANS1	MANSC domain-containing protein 1	Q9H8J5	MANSC1	NA	Inverse variance weighted	6	0.043	0.030	1.506E-01	0.940	pan	SARS-CoV-2 infection
3651_50	VEGF sR2	Vascular endothelial growth factor receptor 2	P35968	KDR	NA	Inverse variance weighted	9	0.021	0.015	1.516E-01	0.940	pan	SARS-CoV-2 infection
3852_19	HSP 40	DnaJ homolog subfamily B member 1	P25685	DNAJB1	NA	Inverse variance weighted	3	-0.070	0.049	1.532E-01	0.940	pan	SARS-CoV-2 infection
19606_28	ihh	Indian hedgehog protein	Q14623	IHH	NA	Wald ratio	1	-0.130	0.091	1.533E-01	0.940	pan	SARS-CoV-2 infection
5036_50	TSG-6	Tumor necrosis factor-inducible gene 6 protein	P98066	TNFAIP6	NA	Inverse variance weighted	22	-0.014	0.010	1.543E-01	0.940	pan	SARS-CoV-2 infection
7738_299	STX2	Syntaxin-2	P32856	STX2	NA	Inverse variance weighted	3	0.087	0.061	1.545E-01	0.940	pan	SARS-CoV-2 infection
3470_1	sE-Selectin	E-selectin	P16581	SELE	NA	Inverse variance weighted	8	-0.042	0.029	1.546E-01	0.940	pan	SARS-CoV-2 infection
8024_64	TPST2	Protein-tyrosine sulfotransferase 2	O60704	TPST2	NA	Inverse variance weighted	2	0.043	0.030	1.552E-01	0.940	pan	SARS-CoV-2 infection
15603_20	Integrin alpha-2	Integrin alpha-2	P17301	ITGA2	NA	Inverse variance weighted	2	0.116	0.082	1.561E-01	0.940	pan	SARS-CoV-2 infection
9049_2	MOT4	Monocarboxylate transporter 4	O15427	SLC16A3	NA	Wald ratio	1	0.149	0.105	1.564E-01	0.940	pan	SARS-CoV-2 infection
8034_6	DRGX	Dorsal root ganglia homeobox protein	A6NNA5	DRGX	NA	Inverse variance weighted	7	-0.019	0.013	1.571E-01	0.940	pan	SARS-CoV-2 infection
6086_15	CRDL2	Chordin-like protein 2	Q6WN34	CHRDL2	NA	Inverse variance weighted	6	0.047	0.033	1.573E-01	0.940	pan	SARS-CoV-2 infection
2979_8	ENA-78	C-X-C motif chemokine 5	P42830	CXCL5	NA	Wald ratio	1	0.181	0.128	1.583E-01	0.940	pan	SARS-CoV-2 infection
6620_82	LIGO1	Leucine-rich repeat and immunoglobulin-like domain-containing nogo receptor-interacting protein 1	Q96FE5	LINGO1	NA	Inverse variance weighted	3	0.061	0.044	1.596E-01	0.940	pan	SARS-CoV-2 infection
7244_16	TM149	IGF-like family receptor 1	Q9H665	IGFLR1	NA	Inverse variance weighted	7	0.014	0.010	1.598E-01	0.940	pan	SARS-CoV-2 infection
10977_55	UCMA	Unique cartilage matrix-associated protein	Q8WVF2	UCMA	NA	Inverse variance weighted	9	0.023	0.016	1.608E-01	0.940	pan	SARS-CoV-2 infection
12940_35	AL3B1	Aldehyde dehydrogenase family 3 member B1	P43353	ALDH3B1	NA	Wald ratio	1	0.057	0.041	1.619E-01	0.940	pan	SARS-CoV-2 infection
12657_2	FCL	GDP-L-fucose synthase	Q13630	GFUS	NA	Wald ratio	1	-0.157	0.112	1.620E-01	0.940	pan	SARS-CoV-2 infection
6485_59	IGLL1	Immunoglobulin lambda-like polypeptide 1	P15814	IGLL1	NA	Inverse variance weighted	28	-0.015	0.011	1.620E-01	0.940	pan	SARS-CoV-2 infection
5803_24	C3d	Complement C3d fragment	P01024	C3	NA	Wald ratio	1	0.117	0.084	1.620E-01	0.940	pan	SARS-CoV-2 infection
14107_1	MTHFS	5-formyltetrahydrofolate cyclo-ligase	P49914	MTHFS	NA	Inverse variance weighted	6	0.018	0.013	1.621E-01	0.940	pan	SARS-CoV-2 infection
7038_45	SIA8D	CMP-N-acetylneuraminate-poly-alpha-2,8-sialyltransferase	Q92187	ST8SIA4	NA	Wald ratio	1	-0.155	0.111	1.621E-01	0.940	pan	SARS-CoV-2 infection
18382_109	Catechol O-methyltransferase	Catechol O-methyltransferase	P21964	COMT	NA	Wald ratio	1	-0.078	0.056	1.621E-01	0.940	pan	SARS-CoV-2 infection
19334_62	TXD12	Thioredoxin domain-containing protein 12	O95881	TXNDC12	NA	Wald ratio	1	0.081	0.058	1.644E-01	0.940	pan	SARS-CoV-2 infection
16613_3	CAD17	Cadherin-17	Q12864	CDH17	NA	Inverse variance weighted	16	0.018	0.013	1.647E-01	0.940	pan	SARS-CoV-2 infection
5005_4	MK12	Mitogen-activated protein kinase 12	P53778	MAPK12	NA	Wald ratio	1	-0.104	0.075	1.671E-01	0.940	pan	SARS-CoV-2 infection
4913_78	HCC-4	C-C motif chemokine 16	O15467	CCL16	NA	Inverse variance weighted	8	-0.044	0.032	1.673E-01	0.940	pan	SARS-CoV-2 infection
17370_186	ABHDA	Mycophenolic acid acyl-glucuronide esterase, mitochondrial	Q9NUJ1	ABHD10	NA	Wald ratio	1	-0.132	0.096	1.676E-01	0.940	pan	SARS-CoV-2 infection
11218_84	TPMT	Thiopurine S-methyltransferase	P51580	TPMT	NA	Wald ratio	1	-0.216	0.157	1.676E-01	0.940	pan	SARS-CoV-2 infection
6617_12	FCRL6	Fc receptor-like protein 6	Q6DN72	FCRL6	NA	Wald ratio	1	-0.056	0.041	1.677E-01	0.940	pan	SARS-CoV-2 infection
9837_60	NAD(P)H dehydrogenase	NAD(P)H dehydrogenase [quinone] 1	P15559	NQO1	NA	Inverse variance weighted	13	-0.014	0.010	1.677E-01	0.940	pan	SARS-CoV-2 infection
17812_2	TP4A1	Protein tyrosine phosphatase type IVA 1	Q93096	PTP4A1	NA	Wald ratio	1	-0.129	0.094	1.681E-01	0.940	pan	SARS-CoV-2 infection
11656_110	EVL	Ena/VASP-like protein	Q9UI08	EVL	NA	Inverse variance weighted	4	0.055	0.040	1.684E-01	0.940	pan	SARS-CoV-2 infection
15548_35	SERA	D-3-phosphoglycerate dehydrogenase	O43175	PHGDH	NA	Inverse variance weighted	2	0.088	0.064	1.703E-01	0.940	pan	SARS-CoV-2 infection
6713_4	LRP11	Low-density lipoprotein receptor-related protein 11	Q86VZ4	LRP11	NA	Inverse variance weighted	12	-0.017	0.012	1.710E-01	0.940	pan	SARS-CoV-2 infection
5825_49	IFN-g R1	Interferon gamma receptor 1	P15260	IFNGR1	NA	Inverse variance weighted	3	-0.070	0.051	1.719E-01	0.940	pan	SARS-CoV-2 infection
9869_28	NF-kappa-B p105	Nuclear factor NF-kappa-B p105 subunit	P19838	NFKB1	NA	Wald ratio	1	-0.165	0.121	1.719E-01	0.940	pan	SARS-CoV-2 infection
12370_30	Apo F	Apolipoprotein F	Q13790	APOF	NA	Inverse variance weighted	2	0.052	0.038	1.720E-01	0.940	pan	SARS-CoV-2 infection
13563_259	ACBD7	Acyl-CoA-binding domain-containing protein 7	Q8N6N7	ACBD7	NA	Wald ratio	1	0.174	0.127	1.721E-01	0.940	pan	SARS-CoV-2 infection
14711_27	Cystatin M	Cystatin-M	Q15828	CST6	NA	Inverse variance weighted	7	-0.038	0.028	1.726E-01	0.940	pan	SARS-CoV-2 infection
14708_59	CO8G	Complement component C8 gamma chain	P07360	C8G	NA	Inverse variance weighted	11	-0.028	0.021	1.729E-01	0.940	pan	SARS-CoV-2 infection
8664_36	PKDCC	Extracellular tyrosine-protein kinase PKDCC	Q504Y2	PKDCC	NA	Inverse variance weighted	2	-0.174	0.128	1.732E-01	0.940	pan	SARS-CoV-2 infection
12347_29	CCM2	Cerebral cavernous malformations 2 protein	Q9BSQ5	CCM2	NA	Wald ratio	1	-0.106	0.078	1.733E-01	0.940	pan	SARS-CoV-2 infection
5008_51	Mn SOD	Superoxide dismutase [Mn], mitochondrial	P04179	SOD2	NA	Inverse variance weighted	4	0.065	0.048	1.735E-01	0.940	pan	SARS-CoV-2 infection
3421_54	CD30 Ligand	Tumor necrosis factor ligand superfamily member 8	P32971	TNFSF8	NA	Inverse variance weighted	2	0.081	0.060	1.744E-01	0.940	pan	SARS-CoV-2 infection
13720_95	Proteinase-3	Myeloblastin	P24158	PRTN3	NA	Inverse variance weighted	10	0.016	0.012	1.747E-01	0.940	pan	SARS-CoV-2 infection
16300_4	TREM2	Triggering receptor expressed on myeloid cells 2	Q9NZC2	TREM2	NA	Inverse variance weighted	21	0.014	0.011	1.749E-01	0.940	pan	SARS-CoV-2 infection
11311_79	RAG-1	V(D)J recombination-activating protein 1	P15918	RAG1	NA	Wald ratio	1	0.123	0.091	1.750E-01	0.940	pan	SARS-CoV-2 infection
18398_1	AK1D1	3-oxo-5-beta-steroid 4-dehydrogenase	P51857	AKR1D1	NA	Wald ratio	1	0.136	0.100	1.755E-01	0.940	pan	SARS-CoV-2 infection
6378_2	CEI	Protein CEI	Q86SI9	C5orf38	NA	Wald ratio	1	0.101	0.075	1.758E-01	0.940	pan	SARS-CoV-2 infection
8994_65	SLAMF8	SLAM family member 8	Q9P0V8	SLAMF8	NA	Inverse variance weighted	5	-0.054	0.040	1.762E-01	0.940	pan	SARS-CoV-2 infection
5238_26	PPIE	Peptidyl-prolyl cis-trans isomerase E	Q9UNP9	PPIE	NA	Inverse variance weighted	3	0.022	0.016	1.764E-01	0.940	pan	SARS-CoV-2 infection
7989_5	CA226	Uncharacterized protein C1orf226	A1L170	C1orf226	NA	Wald ratio	1	0.110	0.082	1.767E-01	0.940	pan	SARS-CoV-2 infection
18884_22	DNJB4	DnaJ homolog subfamily B member 4	Q9UDY4	DNAJB4	NA	Inverse variance weighted	3	0.038	0.028	1.769E-01	0.940	pan	SARS-CoV-2 infection
15440_57	NEC2	Neuroendocrine convertase 2	P16519	PCSK2	NA	Inverse variance weighted	2	-0.068	0.050	1.770E-01	0.940	pan	SARS-CoV-2 infection
5542_22	NRP1	Neuropilin-1	O14786	NRP1	NA	Inverse variance weighted	8	-0.024	0.018	1.774E-01	0.940	pan	SARS-CoV-2 infection
13056_18	S6A16	Orphan sodium- and chloride-dependent neurotransmitter transporter NTT5	Q9GZN6	SLC6A16	NA	Wald ratio	1	0.148	0.110	1.776E-01	0.940	pan	SARS-CoV-2 infection
3519_3	TARC	C-C motif chemokine 17	Q92583	CCL17	NA	Inverse variance weighted	18	0.017	0.013	1.780E-01	0.940	pan	SARS-CoV-2 infection
9898_161	ARI1A	AT-rich interactive domain-containing protein 1A	O14497	ARID1A	NA	Wald ratio	1	0.147	0.109	1.780E-01	0.940	pan	SARS-CoV-2 infection
17786_5	GGPPS	Geranylgeranyl pyrophosphate synthase	O95749	GGPS1	NA	Wald ratio	1	0.074	0.055	1.781E-01	0.940	pan	SARS-CoV-2 infection
10085_25	STAR	Steroidogenic acute regulatory protein, mitochondrial	P49675	STAR	NA	Wald ratio	1	-0.149	0.111	1.786E-01	0.940	pan	SARS-CoV-2 infection
9805_51	SEM4B	Semaphorin-4B	Q9NPR2	SEMA4B	NA	Wald ratio	1	0.119	0.088	1.790E-01	0.940	pan	SARS-CoV-2 infection
10612_18	PLOD3	Procollagen-lysine,2-oxoglutarate 5-dioxygenase 3	O60568	PLOD3	NA	Inverse variance weighted	7	-0.043	0.032	1.794E-01	0.940	pan	SARS-CoV-2 infection
15515_2	SAA	Serum amyloid A-1 protein	P0DJI8	SAA1	NA	Inverse variance weighted	5	0.056	0.041	1.795E-01	0.940	pan	SARS-CoV-2 infection
3605_77	MASP3:Light	Mannan-binding lectin serine protease 1:Mannan-binding lectin serine protease 1 light chain	P48740	MASP1	NA	Wald ratio	1	-0.120	0.090	1.795E-01	0.940	pan	SARS-CoV-2 infection

11573_3	SRSF6	Serine/arginine-rich splicing factor 6	Q13247	SRSF6	NA	Wald ratio	1	0.120	0.090	1.797E-01	0.940	pan	SARS-CoV-2 infection
11510_31	Apo L1	Apolipoprotein L1	O14791	APOL1	NA	Inverse variance weighted	9	-0.021	0.016	1.803E-01	0.940	pan	SARS-CoV-2 infection
13423_94	CJ058	Redox-regulatory protein FAM213A	Q9BRX8	PRXL2A	NA	Wald ratio	1	0.135	0.101	1.814E-01	0.940	pan	SARS-CoV-2 infection
9525_1	PTK7	Inactive tyrosine-protein kinase 7	Q13308	PTK7	NA	Inverse variance weighted	4	-0.045	0.034	1.822E-01	0.940	pan	SARS-CoV-2 infection
12422_143	LX15B	Arachidonate 15-lipoxygenase B	O15296	ALOX15B	NA	Wald ratio	1	0.088	0.066	1.822E-01	0.940	pan	SARS-CoV-2 infection
19523_215	PARK7	Protein DJ-1	Q99497	PARK7	NA	Wald ratio	1	-0.071	0.053	1.823E-01	0.940	pan	SARS-CoV-2 infection
12381_26	CBR1	Carbonyl reductase [NADPH] 1	P16152	CBR1	NA	Inverse variance weighted	5	0.022	0.016	1.826E-01	0.940	pan	SARS-CoV-2 infection
19213_1	ISK4	Serine protease inhibitor Kazal-type 4	O60575	SPINK4	NA	Wald ratio	1	-0.031	0.023	1.826E-01	0.940	pan	SARS-CoV-2 infection
11142_11	ANGL1:C-term	Angiopoietin-related protein 1:C-Term, Fibrinogen domian	O95841	ANGPTL1	NA	Inverse variance weighted	7	0.026	0.019	1.832E-01	0.940	pan	SARS-CoV-2 infection
11449_22	CAZA1	F-actin-capping protein subunit alpha-1	P52907	CAPZA1	NA	Wald ratio	1	-0.182	0.137	1.833E-01	0.940	pan	SARS-CoV-2 infection
5727_35	B3GLT	Beta-1,3-glucosyltransferase	Q6Y288	B3GLCT	NA	Inverse variance weighted	2	0.031	0.023	1.833E-01	0.940	pan	SARS-CoV-2 infection
16307_22	UNC5H4	Netrin receptor UNC5D	Q6UXZ4	UNC5D	NA	Inverse variance weighted	3	0.049	0.037	1.843E-01	0.940	pan	SARS-CoV-2 infection
10056_5	FOXM1	Forkhead box protein M1	Q08050	FOXM1	NA	Wald ratio	1	0.083	0.062	1.844E-01	0.940	pan	SARS-CoV-2 infection
11237_49	PCOC1	Procollagen C-endopeptidase enhancer 1	Q15113	PCOLCE	NA	Inverse variance weighted	4	0.072	0.054	1.846E-01	0.940	pan	SARS-CoV-2 infection
10953_14	CLC2A	C-type lectin domain family 2 member A	Q6UVW9	CLEC2A	NA	Inverse variance weighted	4	-0.054	0.041	1.849E-01	0.940	pan	SARS-CoV-2 infection
15620_4	NLGN1	Neuroligin-1	Q8N2Q7	NLGN1	NA	Inverse variance weighted	3	0.048	0.036	1.849E-01	0.940	pan	SARS-CoV-2 infection
3316_58	Heparin cofactor II	Heparin cofactor 2	P05546	SERPIND1	NA	Wald ratio	1	0.136	0.103	1.853E-01	0.940	pan	SARS-CoV-2 infection
9772_153	NLGN2:ECD	Neuroligin-2:Extracellular domain	Q8NFZ4	NLGN2	NA	Inverse variance weighted	2	0.071	0.054	1.858E-01	0.940	pan	SARS-CoV-2 infection
7787_25	LIRA5	Leukocyte immunoglobulin-like receptor subfamily A member 5	A6NI73	LILRA5	NA	Wald ratio	1	-0.130	0.099	1.859E-01	0.940	pan	SARS-CoV-2 infection
19492_5	KAAG1	Kidney-associated antigen 1	Q9UBP8	KAAG1	NA	Inverse variance weighted	14	0.020	0.015	1.860E-01	0.940	pan	SARS-CoV-2 infection
16070_7	WIF-1	Wnt inhibitory factor 1	Q9Y5W5	WIF1	NA	Wald ratio	1	-0.105	0.079	1.867E-01	0.940	pan	SARS-CoV-2 infection
14271_23	RAB6B	Ras-related protein Rab-6B	Q9NRW1	RAB6B	NA	Wald ratio	1	0.039	0.029	1.868E-01	0.940	pan	SARS-CoV-2 infection
16773_29	SCUB3	Signal peptide, CUB and EGF-like domain-containing protein 3	Q8IX30	SCUBE3	NA	Inverse variance weighted	4	-0.030	0.023	1.871E-01	0.940	pan	SARS-CoV-2 infection
8597_1	TMEM9:CD	Transmembrane protein 9:Cytoplasmic domain	Q9P0T7	TMEM9	NA	Inverse variance weighted	7	-0.022	0.017	1.876E-01	0.940	pan	SARS-CoV-2 infection
18432_32	RALB	Ras-related protein Ral-B	P11234	RALB	NA	Wald ratio	1	0.104	0.079	1.884E-01	0.940	pan	SARS-CoV-2 infection
6207_10	prosaposin	Prosaposin	P07602	PSAP	NA	Inverse variance weighted	5	0.072	0.055	1.887E-01	0.940	pan	SARS-CoV-2 infection
3378_49	Kallikrein 7	Kallikrein-7	P49862	KLK7	NA	Inverse variance weighted	2	0.043	0.033	1.899E-01	0.940	pan	SARS-CoV-2 infection
18942_11	PSB9	Proteasome subunit beta type-9	P28065	PSMB9	NA	Wald ratio	1	0.139	0.106	1.899E-01	0.940	pan	SARS-CoV-2 infection
4920_10	Lysozyme	Lysozyme C	P61626	LYZ	NA	Inverse variance weighted	10	-0.015	0.011	1.904E-01	0.940	pan	SARS-CoV-2 infection
9713_67	PGFRL	Platelet-derived growth factor receptor-like protein	Q15198	PDGFRL	NA	Inverse variance weighted	6	-0.017	0.013	1.905E-01	0.940	pan	SARS-CoV-2 infection
14101_2	CNTFR alpha	Ciliary neurotrophic factor receptor subunit alpha	P26992	CNTFR	NA	Inverse variance weighted	6	-0.039	0.030	1.905E-01	0.940	pan	SARS-CoV-2 infection
17819_30	FAHD1	Acylpyruvase FAHD1, mitochondrial	Q6P587	FAHD1	NA	Inverse variance weighted	4	-0.053	0.040	1.907E-01	0.940	pan	SARS-CoV-2 infection
14112_40	RELT	Tumor necrosis factor receptor superfamily member 19L	Q969Z4	RELT	NA	Wald ratio	1	-0.125	0.096	1.916E-01	0.940	pan	SARS-CoV-2 infection
19631_13	Kininostatin	Kininostatin	P01042	KNG1	NA	Inverse variance weighted	12	-0.040	0.031	1.925E-01	0.940	pan	SARS-CoV-2 infection
14076_74	Cystatin-S	Cystatin-S	P01036	CST4	NA	Inverse variance weighted	12	-0.027	0.021	1.926E-01	0.940	pan	SARS-CoV-2 infection
17342_13	AGR3	Anterior gradient protein 3	Q8TD06	AGR3	NA	Wald ratio	1	0.135	0.104	1.928E-01	0.940	pan	SARS-CoV-2 infection
13098_93	VEGF-D	Vascular endothelial growth factor D	O43915	VEGFD	NA	Wald ratio	1	0.112	0.086	1.934E-01	0.940	pan	SARS-CoV-2 infection
10903_50	STX8	Syntaxin-8	Q9UNK0	STX8	NA	Wald ratio	1	-0.101	0.078	1.948E-01	0.940	pan	SARS-CoV-2 infection
13102_1	FAM3D	Protein FAM3D	Q96BQ1	FAM3D	NA	Inverse variance weighted	9	0.018	0.014	1.948E-01	0.940	pan	SARS-CoV-2 infection
7083_74	MATN4	Matrilin-4	O95460	MATN4	NA	Inverse variance weighted	3	0.036	0.027	1.950E-01	0.940	pan	SARS-CoV-2 infection
15511_37	NPTXR	Neuronal pentraxin receptor	O95502	NPTXR	NA	Inverse variance weighted	10	-0.015	0.012	1.953E-01	0.940	pan	SARS-CoV-2 infection
3905_62	UBE2N	Ubiquitin-conjugating enzyme E2 N	P61088	UBE2N	NA	Wald ratio	1	-0.131	0.101	1.955E-01	0.940	pan	SARS-CoV-2 infection
8043_153	COMP	Cartilage oligomeric matrix protein	P49747	COMP	NA	Inverse variance weighted	8	-0.030	0.023	1.956E-01	0.940	pan	SARS-CoV-2 infection
4866_59	TrkB	BDNF/NT-3 growth factors receptor	Q16620	NTRK2	NA	Inverse variance weighted	2	0.135	0.104	1.961E-01	0.940	pan	SARS-CoV-2 infection
13399_33	RELL1	RELT-like protein 1	Q8IUW5	RELL1	NA	Inverse variance weighted	2	-0.075	0.058	1.966E-01	0.940	pan	SARS-CoV-2 infection
17806_6	Syntenin 1	Syntenin-1	O00560	SDCBP	NA	Wald ratio	1	0.135	0.105	1.977E-01	0.940	pan	SARS-CoV-2 infection
5663_18	PF4V	Platelet factor 4 variant	P10720	PF4V1	NA	Inverse variance weighted	2	0.098	0.076	1.977E-01	0.940	pan	SARS-CoV-2 infection
8484_24	Leptin	Leptin	P41159	LEP	NA	Inverse variance weighted	4	0.063	0.049	1.981E-01	0.940	pan	SARS-CoV-2 infection
19296_51	MLRA	Myosin regulatory light chain 2, atrial isoform	Q01449	MYL7	NA	Inverse variance weighted	6	0.045	0.035	1.983E-01	0.940	pan	SARS-CoV-2 infection
15475_4	PLTP	Phospholipid transfer protein	P55058	PLTP	NA	Inverse variance weighted	11	0.026	0.020	1.990E-01	0.940	pan	SARS-CoV-2 infection
2611_72	Dtk	Tyrosine-protein kinase receptor TYRO3	Q06418	TYRO3	NA	Inverse variance weighted	2	0.058	0.045	2.006E-01	0.940	pan	SARS-CoV-2 infection
10754_113	Prokineticin-2	Prokineticin-2	Q9HC23	PROK2	NA	Inverse variance weighted	9	-0.017	0.013	2.011E-01	0.940	pan	SARS-CoV-2 infection
6404_20	C1QRF	C1q-related factor	O75973	C1QL1	NA	Inverse variance weighted	7	-0.030	0.023	2.015E-01	0.940	pan	SARS-CoV-2 infection
4535_50	BST1	ADP-ribosyl cyclase/cyclic ADP-ribose hydrolase 2	Q10588	BST1	NA	Inverse variance weighted	17	-0.007	0.006	2.018E-01	0.940	pan	SARS-CoV-2 infection
12530_14	CKS-1	Cyclin-dependent kinases regulatory subunit 1	P61024	CKS1B	NA	Inverse variance weighted	4	0.074	0.058	2.022E-01	0.940	pan	SARS-CoV-2 infection
9394_19	Aminopeptidase	Carboxypeptidase Q	Q9Y646	CPQ	NA	Inverse variance weighted	12	0.014	0.011	2.025E-01	0.940	pan	SARS-CoV-2 infection
10560_1	ITM2C:N-term	Integral membrane protein 2C:N-term	Q9NQX7	ITM2C	NA	Wald ratio	1	0.123	0.096	2.031E-01	0.940	pan	SARS-CoV-2 infection
17329_2	BDH2	3-hydroxybutyrate dehydrogenase type 2	Q9BUT1	BDH2	NA	Inverse variance weighted	2	0.036	0.029	2.036E-01	0.940	pan	SARS-CoV-2 infection
13518_5	ASAP2	Arf-GAP with SH3 domain, ANK repeat and PH domain-containing protein 2	O43150	ASAP2	NA	Wald ratio	1	0.065	0.051	2.036E-01	0.940	pan	SARS-CoV-2 infection
6107_3	ELA1	Chymotrypsin-like elastase family member 1	Q9UNI1	CELA1	NA	Inverse variance weighted	13	0.020	0.016	2.039E-01	0.940	pan	SARS-CoV-2 infection
5722_78	Prolylcarboxypeptidase	Lysosomal Pro-X carboxypeptidase	P42785	PRCP	NA	Inverse variance weighted	8	0.031	0.024	2.041E-01	0.940	pan	SARS-CoV-2 infection
3296_92	CNTN2	Contactin-2	Q02246	CNTN2	NA	Inverse variance weighted	9	0.016	0.012	2.041E-01	0.940	pan	SARS-CoV-2 infection
18206_18	ADH6	Alcohol dehydrogenase 6	P28332	ADH6	NA	Inverse variance weighted	2	-0.093	0.073	2.044E-01	0.940	pan	SARS-CoV-2 infection
19227_18	BATF	Basic leucine zipper transcriptional factor ATF-like	Q16520	BATF	NA	Wald ratio	1	0.133	0.105	2.057E-01	0.940	pan	SARS-CoV-2 infection
2871_73	RAD51	DNA repair protein RAD51 homolog 1	Q06609	RAD51	NA	Wald ratio	1	-0.130	0.103	2.058E-01	0.940	pan	SARS-CoV-2 infection
7806_33	B4GT7	Beta-1,4-galactosyltransferase 7	Q9UBV7	B4GALT7	NA	Inverse variance weighted	8	-0.021	0.016	2.059E-01	0.940	pan	SARS-CoV-2 infection
17724_3	WWOX	WW domain-containing oxidoreductase	Q9NZC7	WWOX	NA	Wald ratio	1	-0.084	0.067	2.060E-01	0.940	pan	SARS-CoV-2 infection
11825_27	PRGC1	Peroxisome proliferator-activated receptor gamma coactivator 1-alpha	Q9UBK2	PPARGC1A	NA	Wald ratio	1	0.128	0.102	2.069E-01	0.940	pan	SARS-CoV-2 infection
17331_138	KREM1	Kremen protein 1	Q96MU8	KREMEN1	NA	Inverse variance weighted	4	-0.036	0.028	2.070E-01	0.940	pan	SARS-CoV-2 infection
19223_6	RAB1A	Ras-related protein Rab-1A	P62820	RAB1A	NA	Inverse variance weighted	2	-0.121	0.096	2.071E-01	0.940	pan	SARS-CoV-2 infection
18841_1	SPB13	Serpin B13	Q9UIV8	SERPINB13	NA	Inverse variance weighted	4	0.031	0.025	2.072E-01	0.940	pan	SARS-CoV-2 infection
8304_50	OPG	Tumor necrosis factor receptor superfamily member 11B	O00300	TNFRSF11B	NA	Inverse variance weighted	3	0.034	0.027	2.075E-01	0.940	pan	SARS-CoV-2 infection
2436_49	CXCL16, soluble	C-X-C motif chemokine 16	Q9H2A7	CXCL16	NA	Inverse variance weighted	11	0.022	0.018	2.077E-01	0.940	pan	SARS-CoV-2 infection
2992_59	IL-17 sR	Interleukin-17 receptor A	Q96F46	IL17RA	NA	Inverse variance weighted	8	-0.007	0.006	2.078E-01	0.940	pan	SARS-CoV-2 infection
7127_3	Apo A-II	Apolipoprotein A-II	P02652	APOA2	NA	Inverse variance weighted	11	-0.035	0.028	2.086E-01	0.940	pan	SARS-CoV-2 infection
19365_11	BCAT2	Branched-chain-amino-acid aminotransferase, mitochondrial	O15382	BCAT2	NA	Inverse variance weighted	2	0.066	0.053	2.092E-01	0.940	pan	SARS-CoV-2 infection
6227_1	kallikrein 10	Kallikrein-10	O43240	KLK10	NA	Inverse variance weighted	8	0.030	0.024	2.096E-01	0.940	pan	SARS-CoV-2 infection
3554_24	Adiponectin	Adiponectin	Q15848	ADIPOQ	NA	Inverse variance weighted	15	-0.025	0.020	2.116E-01	0.940	pan	SARS-CoV-2 infection

6550_4	ICAM4	Intercellular adhesion molecule 4	Q14773	ICAM4	NA	Inverse variance weighted	13	0.016	0.013	2.119E-01	0.940	pan	SARS-CoV-2 infection
11302_237	TENR	Tenascin-R	Q92752	TNR	NA	Inverse variance weighted	3	0.058	0.047	2.121E-01	0.940	pan	SARS-CoV-2 infection
7193_98	PCDC1	Protocadherin alpha-C1	Q9H158	PCDHAC1	NA	Wald ratio	1	-0.086	0.069	2.121E-01	0.940	pan	SARS-CoV-2 infection
11094_104	LPPL	Galectin-10	Q05315	CLC	NA	Wald ratio	1	-0.123	0.098	2.123E-01	0.940	pan	SARS-CoV-2 infection
13669_6	FGFR-3:ECD	Fibroblast growth factor receptor 3:Extracellular domain	P22607	FGFR3	NA	Inverse variance weighted	3	-0.035	0.028	2.123E-01	0.940	pan	SARS-CoV-2 infection
6408_2	INHBC	Inhibin beta C chain	P55103	INHBC	NA	Inverse variance weighted	5	0.030	0.024	2.124E-01	0.940	pan	SARS-CoV-2 infection
18376_19	Myosin light chain 1	Myosin light chain 3	P08590	MYL3	NA	Inverse variance weighted	6	-0.043	0.035	2.129E-01	0.940	pan	SARS-CoV-2 infection
11208_15	NAGPA	N-acetylglucosamine-1-phosphodiester alpha-N-acetylglucosaminidase	Q9UK23	NAGPA	NA	Inverse variance weighted	7	0.015	0.012	2.130E-01	0.940	pan	SARS-CoV-2 infection
14067_6	PKP2	Plakophilin-2	Q99959	PKP2	NA	Inverse variance weighted	2	0.071	0.057	2.132E-01	0.940	pan	SARS-CoV-2 infection
13934_3	MCF2L	Guanine nucleotide exchange factor DBS	O15068	MCF2L	NA	Inverse variance weighted	2	-0.028	0.022	2.143E-01	0.940	pan	SARS-CoV-2 infection
5111_15	NRX3B	Neurexin-3-beta	Q9HDB5	NRXN3	NA	Inverse variance weighted	3	0.076	0.061	2.150E-01	0.940	pan	SARS-CoV-2 infection
13132_14	SEM5A	Semaphorin-5A	Q13591	SEMA5A	NA	Inverse variance weighted	9	-0.007	0.005	2.151E-01	0.940	pan	SARS-CoV-2 infection
2843_13	SPINT2	Kunitz-type protease inhibitor 2	O43291	SPINT2	NA	Inverse variance weighted	15	0.014	0.011	2.154E-01	0.940	pan	SARS-CoV-2 infection
12583_77	ARAF	Serine/threonine-protein kinase A-Raf	P10398	ARAF	NA	Wald ratio	1	-0.112	0.090	2.160E-01	0.940	pan	SARS-CoV-2 infection
16318_12	ALK-1	Serine/threonine-protein kinase receptor R3	P37023	ACVRL1	NA	Inverse variance weighted	3	-0.030	0.024	2.163E-01	0.940	pan	SARS-CoV-2 infection
2579_17	MMP-9	Matrix metalloproteinase-9	P14780	MMP9	NA	Inverse variance weighted	6	0.056	0.045	2.177E-01	0.940	pan	SARS-CoV-2 infection
12585_39	ERCC1	DNA excision repair protein ERCC-1	P07992	ERCC1	NA	Wald ratio	1	0.122	0.099	2.178E-01	0.940	pan	SARS-CoV-2 infection
13969_24	IMA7	Importin subunit alpha-7	O60684	KPNA6	NA	Wald ratio	1	0.099	0.080	2.181E-01	0.940	pan	SARS-CoV-2 infection
3803_10	CYTD	Cystatin-D	P28325	CST5	NA	Inverse variance weighted	17	0.021	0.017	2.182E-01	0.940	pan	SARS-CoV-2 infection
6409_57	GP116	Adhesion G protein-coupled receptor F5	Q8IZF2	ADGRF5	NA	Inverse variance weighted	13	-0.009	0.008	2.183E-01	0.940	pan	SARS-CoV-2 infection
17341_89	THIC	Acetyl-CoA acetyltransferase, cytosolic	Q9BWD1	ACAT2	NA	Inverse variance weighted	3	0.021	0.017	2.185E-01	0.940	pan	SARS-CoV-2 infection
10816_150	PILRA isoform FDF03-M14	Paired immunoglobulin-like type 2 receptor alpha isoform FDF03-M14	Q9UKJ1	PILRA	NA	Inverse variance weighted	9	-0.041	0.033	2.195E-01	0.940	pan	SARS-CoV-2 infection
15383_200	Endothelin 3	Endothelin-3	P14138	EDN3	NA	Wald ratio	1	-0.124	0.101	2.198E-01	0.940	pan	SARS-CoV-2 infection
12643_4	ARRB1	Beta-arrestin-1	P49407	ARRB1	NA	Wald ratio	1	-0.078	0.064	2.206E-01	0.940	pan	SARS-CoV-2 infection
3009_3	TGF-b R III	Transforming growth factor beta receptor type 3	Q03167	TGFBR3	NA	Inverse variance weighted	6	-0.030	0.024	2.209E-01	0.940	pan	SARS-CoV-2 infection
11192_168	TINAL	Tubulointerstitial nephritis antigen-like	Q9GZM7	TINAGL1	NA	Inverse variance weighted	8	-0.026	0.021	2.210E-01	0.940	pan	SARS-CoV-2 infection
8383_20	CK094	Uncharacterized protein C11orf94	C9JXX5	C11orf94	NA	Wald ratio	1	-0.054	0.044	2.212E-01	0.940	pan	SARS-CoV-2 infection
18922_27	CD68	Macrosialin	P34810	CD68	NA	Inverse variance weighted	4	-0.053	0.043	2.215E-01	0.940	pan	SARS-CoV-2 infection
13068_139	CCS	Copper chaperone for superoxide dismutase	O14618	CCS	NA	Wald ratio	1	-0.060	0.049	2.221E-01	0.940	pan	SARS-CoV-2 infection
4355_13	DAPK2	Death-associated protein kinase 2	Q9UIK4	DAPK2	NA	Inverse variance weighted	5	0.057	0.047	2.222E-01	0.940	pan	SARS-CoV-2 infection
8890_9	T132B	Transmembrane protein 132B	Q14DG7	TMEM132B	NA	Inverse variance weighted	3	-0.021	0.017	2.229E-01	0.940	pan	SARS-CoV-2 infection
13463_1	PXDN	Peroxidasin homolog	Q92626	PXDN	NA	Inverse variance weighted	4	0.047	0.039	2.235E-01	0.940	pan	SARS-CoV-2 infection
10435_2	ASPG	N(4)-(beta-N-acetylglucosaminyl)-L-asparaginase	P20933	AGA	NA	Inverse variance weighted	2	-0.073	0.060	2.244E-01	0.940	pan	SARS-CoV-2 infection
3184_25	Coagulation Factor VII	Coagulation factor VII	P08709	F7	NA	Inverse variance weighted	10	0.013	0.010	2.244E-01	0.940	pan	SARS-CoV-2 infection
12813_18	EHBP1	EH domain-binding protein 1	Q8NDI1	EHBP1	NA	Wald ratio	1	0.122	0.100	2.246E-01	0.940	pan	SARS-CoV-2 infection
13940_19	IP16:HIN 1	Gamma-interferon-inducible protein 16:Isoform 2, Hematopoietic expression, interferon-inducible nature, and nuclear localization 1	Q16666	IFI16	NA	Wald ratio	1	0.088	0.073	2.248E-01	0.940	pan	SARS-CoV-2 infection
12717_65	TOX3	TOX high mobility group box family member 3	O15405	TOX3	NA	Wald ratio	1	-0.116	0.096	2.251E-01	0.940	pan	SARS-CoV-2 infection
13930_3	ABC3G	DNA dC->dU-editing enzyme APOBEC-3G	Q9HC16	APOBEC3G	NA	Inverse variance weighted	2	0.056	0.046	2.255E-01	0.940	pan	SARS-CoV-2 infection
7638_30	Lectin, mannose-binding 2	Vesicular integral-membrane protein VIP36	Q12907	LMAN2	NA	Inverse variance weighted	2	0.115	0.095	2.266E-01	0.940	pan	SARS-CoV-2 infection
5632_6	CRAC1	Cartilage acidic protein 1	Q9NQ79	CRTAC1	NA	Inverse variance weighted	10	-0.017	0.014	2.271E-01	0.940	pan	SARS-CoV-2 infection
3195_50	Granulysin	Granulysin	P22749	GNLY	NA	Inverse variance weighted	17	-0.015	0.012	2.274E-01	0.940	pan	SARS-CoV-2 infection
15522_2	GAPR1	Golgi-associated plant pathogenesis-related protein 1	Q9H4G4	GLIPR2	NA	Inverse variance weighted	6	-0.030	0.025	2.275E-01	0.940	pan	SARS-CoV-2 infection
3810_50	FGR	Tyrosine-protein kinase Fgr	P09769	FGR	NA	Inverse variance weighted	2	0.044	0.037	2.279E-01	0.940	pan	SARS-CoV-2 infection
11248_43	HEM4	Uroporphyrinogen-III synthase	P10746	UROS	NA	Inverse variance weighted	4	-0.028	0.023	2.279E-01	0.940	pan	SARS-CoV-2 infection
3132_1	VEGF-C	Vascular endothelial growth factor C	P49767	VEGFC	NA	Inverse variance weighted	2	-0.056	0.047	2.280E-01	0.940	pan	SARS-CoV-2 infection
19117_3	PP14A	Protein phosphatase 1 regulatory subunit 14A	Q96A00	PPP1R14A	NA	Inverse variance weighted	2	0.077	0.064	2.315E-01	0.944	pan	SARS-CoV-2 infection
9241_40	SIRPG	Signal-regulatory protein gamma	Q9P1W8	SIRPG	NA	Wald ratio	1	-0.069	0.057	2.318E-01	0.944	pan	SARS-CoV-2 infection
17513_11	ANX11	Annexin A11	P50995	ANXA11	NA	Inverse variance weighted	3	0.054	0.045	2.319E-01	0.944	pan	SARS-CoV-2 infection
11140_56	CO1A1:C-term propeptide	Collagen alpha-1(I) chain:C-term propeptide	P02452	COL1A1	NA	Inverse variance weighted	8	-0.027	0.023	2.320E-01	0.944	pan	SARS-CoV-2 infection
3028_36	Ck-b-8-1	Ck-beta-8-1	P55773	CCL23	NA	Inverse variance weighted	4	-0.055	0.046	2.321E-01	0.944	pan	SARS-CoV-2 infection
6556_5	ENPP5	Ectonucleotide pyrophosphatase/phosphodiesterase family member 5	Q9UJA9	ENPP5	NA	Inverse variance weighted	15	0.009	0.008	2.323E-01	0.944	pan	SARS-CoV-2 infection
9779_63	HERP2	Homocysteine-responsive endoplasmic reticulum-resident ubiquitin-like domain member 2 protein	Q9BSE4	HERPUD2	NA	Wald ratio	1	0.124	0.103	2.323E-01	0.944	pan	SARS-CoV-2 infection
18819_21	PPIC	Peptidyl-prolyl cis-trans isomerase C	P45877	PPIC	NA	Inverse variance weighted	11	-0.014	0.012	2.327E-01	0.944	pan	SARS-CoV-2 infection
19176_27	FA49B	Protein FAM49B	Q9NUQ9	CYRIB	NA	Wald ratio	1	0.117	0.098	2.329E-01	0.944	pan	SARS-CoV-2 infection
5132_71	TCCR	Interleukin-27 receptor subunit alpha	Q6UWB1	IL27RA	NA	Inverse variance weighted	9	-0.010	0.009	2.346E-01	0.944	pan	SARS-CoV-2 infection
15472_16	LRP11	Low-density lipoprotein receptor-related protein 11	Q86VZ4	LRP11	NA	Inverse variance weighted	8	-0.022	0.019	2.347E-01	0.944	pan	SARS-CoV-2 infection
6367_66	fibromodulin	fibromodulin	Q06828	FMOD	NA	Inverse variance weighted	5	0.030	0.025	2.350E-01	0.944	pan	SARS-CoV-2 infection
11134_30	RF1ML	Peptide chain release factor 1-like, mitochondrial	Q9UGC7	MTRF1L	NA	Wald ratio	1	-0.036	0.030	2.368E-01	0.944	pan	SARS-CoV-2 infection
2985_35	Gro-a	Growth-regulated alpha protein	P09341	CXCL1	NA	Inverse variance weighted	12	0.016	0.013	2.369E-01	0.944	pan	SARS-CoV-2 infection
5227_60	PDK1	[Pyruvate dehydrogenase (acetyl-transferring)] kinase isozyme 1, mitochondrial	Q15118	PDK1	NA	Inverse variance weighted	3	0.030	0.026	2.376E-01	0.944	pan	SARS-CoV-2 infection
19110_6	MEMO1	Protein MEMO1	Q9Y316	MEMO1	NA	Inverse variance weighted	2	0.052	0.044	2.387E-01	0.944	pan	SARS-CoV-2 infection
11104_13	YKL-40	Chitinase-3-like protein 1	P36222	CHI3L1	NA	Inverse variance weighted	12	-0.011	0.009	2.388E-01	0.944	pan	SARS-CoV-2 infection
6984_6	IGSF8	Immunoglobulin superfamily member 8	Q969P0	IGSF8	NA	Inverse variance weighted	3	0.043	0.036	2.391E-01	0.944	pan	SARS-CoV-2 infection
3580_25	a1-Antitrypsin	Alpha-1-antitrypsin	P01009	SERPINA1	NA	Inverse variance weighted	6	-0.030	0.026	2.393E-01	0.944	pan	SARS-CoV-2 infection
13947_371	NUD12	Peroxisomal NADH pyrophosphatase NUDT12	Q9BQG2	NUDT12	NA	Inverse variance weighted	5	-0.016	0.013	2.393E-01	0.944	pan	SARS-CoV-2 infection
7809_22	SPAT9	Spermatogenesis-associated protein 9	Q9BWV2	SPATA9	NA	Wald ratio	1	-0.095	0.081	2.393E-01	0.944	pan	SARS-CoV-2 infection
8014_359	MANEA	Glycoprotein endo-alpha-1,2-mannosidase	Q5SRI9	MANEA	NA	Inverse variance weighted	19	-0.008	0.007	2.396E-01	0.944	pan	SARS-CoV-2 infection
18880_81	Collagen Type III	Collagen Type III	P02461	COL3A1	NA	Inverse variance weighted	9	-0.042	0.036	2.401E-01	0.944	pan	SARS-CoV-2 infection
2677_1	ERBB1	Epidermal growth factor receptor	P00533	EGFR	NA	Inverse variance weighted	5	0.035	0.030	2.403E-01	0.944	pan	SARS-CoV-2 infection
9316_67	WFDC1	WAP four-disulfide core domain protein 1	Q9HC57	WFDC1	NA	Inverse variance weighted	5	0.021	0.018	2.403E-01	0.944	pan	SARS-CoV-2 infection
9313_27	CBLN1	Cerebellin-1	P23435	CBLN1	NA	Inverse variance weighted	31	-0.014	0.012	2.413E-01	0.944	pan	SARS-CoV-2 infection
16035_8	VEGF sR3	Vascular endothelial growth factor receptor 3	P35916	FLT4	NA	Inverse variance weighted	11	-0.028	0.024	2.413E-01	0.944	pan	SARS-CoV-2 infection
8299_66	LIRA4	Leukocyte immunoglobulin-like receptor subfamily A member 4	P59901	LILRA4	NA	Inverse variance weighted	9	0.034	0.029	2.413E-01	0.944	pan	SARS-CoV-2 infection
13499_30	Coagulation Factor VIII	Coagulation Factor VIII	P00451	F8	NA	Inverse variance weighted	6	0.044	0.037	2.423E-01	0.944	pan	SARS-CoV-2 infection
13692_154	WISP-1	WNT1-inducible-signaling pathway protein 1	O95388	CCN4	NA	Inverse variance weighted	12	-0.011	0.010	2.424E-01	0.944	pan	SARS-CoV-2 infection
9409_11	TRYB1	Tryptase beta-1	Q15661	TPSAB1	NA	Inverse variance weighted	18	0.012	0.010	2.426E-01	0.944	pan	SARS-CoV-2 infection
12801_33	IRF2	Interferon regulatory factor 2	P14316	IRF2	NA	Wald ratio	1	-0.116	0.099	2.430E-01	0.944	pan	SARS-CoV-2 infection
9578_263	MANS4	MANSC domain-containing protein 4	A6NHS7	MANSC4	NA	Inverse variance weighted	10	-0.011	0.009	2.434E-01	0.944	pan	SARS-CoV-2 infection

6649_51	NET1	Netrin-1	O95631	NTN1	NA	Inverse variance weighted	7	0.014	0.012	2.445E-01	0.944	pan	SARS-CoV-2 infection
2590_69	ROR1	Inactive tyrosine-protein kinase transmembrane receptor ROR1	Q01973	ROR1	NA	Inverse variance weighted	4	-0.022	0.019	2.449E-01	0.944	pan	SARS-CoV-2 infection
9391_60	PCSK1	ProSAAS	Q9UHG2	PCSK1N	NA	Inverse variance weighted	14	-0.015	0.013	2.451E-01	0.944	pan	SARS-CoV-2 infection
4564_2	PLXC1	Plexin-C1	O60486	PLXNC1	NA	Inverse variance weighted	11	0.008	0.007	2.451E-01	0.944	pan	SARS-CoV-2 infection
17805_35	SPF30	Survival of motor neuron-related-splicing factor 30	O75940	SMNDC1	NA	Wald ratio	1	-0.119	0.102	2.456E-01	0.944	pan	SARS-CoV-2 infection
13998_26	PURA1	Adenylosuccinate synthetase isozyme 1	Q8N142	ADSS1	NA	Inverse variance weighted	3	0.029	0.025	2.457E-01	0.944	pan	SARS-CoV-2 infection
12401_3	STALP	AMSH-like protease	Q96FJ0	STAMBPL1	NA	Wald ratio	1	0.059	0.051	2.472E-01	0.948	pan	SARS-CoV-2 infection
12549_33	PTGD2	Hematopoietic prostaglandin D synthase	O60760	HPGDS	NA	Inverse variance weighted	12	0.011	0.010	2.478E-01	0.948	pan	SARS-CoV-2 infection
19370_30	HS3S4	Heparan sulfate glucosamine 3-O-sulfotransferase 4	Q9Y661	HS3ST4	NA	Wald ratio	1	-0.088	0.076	2.480E-01	0.948	pan	SARS-CoV-2 infection
3488_64	Catalase	Catalase	P04040	CAT	NA	Inverse variance weighted	5	-0.057	0.050	2.484E-01	0.948	pan	SARS-CoV-2 infection
11264_33	XDH	Xanthine dehydrogenase/oxidase	P47989	XDH	NA	Wald ratio	1	0.118	0.102	2.499E-01	0.950	pan	SARS-CoV-2 infection
11601_26	DHX8	ATP-dependent RNA helicase DHX8	Q14562	DHX8	NA	Inverse variance weighted	4	0.042	0.037	2.502E-01	0.950	pan	SARS-CoV-2 infection
9015_1	PRG3	Proteoglycan 3	Q9Y2Y8	PRG3	NA	Inverse variance weighted	10	0.033	0.029	2.503E-01	0.950	pan	SARS-CoV-2 infection
18839_24	Thyroglobulin	Thyroglobulin	P01266	TG	NA	Inverse variance weighted	6	0.030	0.026	2.512E-01	0.951	pan	SARS-CoV-2 infection
9793_145	IGDC4	Immunoglobulin superfamily DCC subclass member 4	Q8TDY8	IGDCC4	NA	Inverse variance weighted	10	-0.012	0.011	2.522E-01	0.951	pan	SARS-CoV-2 infection
2731_29	NADPH-P450 Oxidoreductase	NADPH--cytochrome P450 reductase	P16435	POR	NA	Inverse variance weighted	4	-0.040	0.035	2.523E-01	0.951	pan	SARS-CoV-2 infection
4982_54	Elafin	Elafin	P19957	PI3	NA	Inverse variance weighted	8	0.027	0.024	2.525E-01	0.951	pan	SARS-CoV-2 infection
16599_38	GPN1	GPN-loop GTPase 1	Q9HCN4	GPN1	NA	Inverse variance weighted	6	-0.029	0.025	2.528E-01	0.951	pan	SARS-CoV-2 infection
2516_57	6Ckine	C-C motif chemokine 21	O00585	CCL21	NA	Inverse variance weighted	17	0.017	0.015	2.539E-01	0.954	pan	SARS-CoV-2 infection
12594_5	GRAN	Grancalcin	P28676	GCA	NA	Inverse variance weighted	2	0.019	0.017	2.555E-01	0.958	pan	SARS-CoV-2 infection
18387_7	suppression of tumorigenicity 13	Hsc70-interacting protein	P50502	ST13	NA	Wald ratio	1	-0.102	0.089	2.559E-01	0.958	pan	SARS-CoV-2 infection
2211_9	TIMP-1	Metalloproteinase inhibitor 1	P01033	TIMP1	NA	Wald ratio	1	0.091	0.080	2.575E-01	0.959	pan	SARS-CoV-2 infection
13453_2	RM33	39S ribosomal protein L33, mitochondrial	O75394	MRPL33	NA	Inverse variance weighted	4	-0.051	0.045	2.580E-01	0.959	pan	SARS-CoV-2 infection
2774_10	IL-16	Interleukin-16	Q14005	IL16	NA	Inverse variance weighted	7	-0.016	0.014	2.587E-01	0.959	pan	SARS-CoV-2 infection
15394_79	UNC5B	Netrin receptor UNC5B	Q8IZJ1	UNC5B	NA	Inverse variance weighted	4	-0.046	0.041	2.587E-01	0.959	pan	SARS-CoV-2 infection
15444_45	SCCA2	Serpin B4	P48594	SERPINB4	NA	Inverse variance weighted	5	0.041	0.036	2.593E-01	0.959	pan	SARS-CoV-2 infection
11649_3	SMAP1	Stromal membrane-associated protein 1	Q8IYB5	SMAP1	NA	Inverse variance weighted	6	-0.017	0.015	2.595E-01	0.959	pan	SARS-CoV-2 infection
18214_2	GSH0	Glutamate--cysteine ligase regulatory subunit	P48507	GCLM	NA	Inverse variance weighted	2	0.050	0.045	2.596E-01	0.959	pan	SARS-CoV-2 infection
19143_38	NB5R2	NADH-cytochrome b5 reductase 2	Q6BCY4	CYB5R2	NA	Inverse variance weighted	7	-0.031	0.028	2.602E-01	0.959	pan	SARS-CoV-2 infection
8297_8	DJC10	DnaJ homolog subfamily C member 10	Q8IXB1	DNAJC10	NA	Inverse variance weighted	6	-0.035	0.031	2.604E-01	0.959	pan	SARS-CoV-2 infection
6455_52	HPLN4	Hyaluronan and proteoglycan link protein 4	Q86UW8	HAPLN4	NA	Wald ratio	1	0.132	0.117	2.605E-01	0.959	pan	SARS-CoV-2 infection
17712_7	IDI1	Isopentenyl-diphosphate Delta-isomerase 1	Q13907	IDI1	NA	Wald ratio	1	-0.083	0.074	2.618E-01	0.959	pan	SARS-CoV-2 infection
12573_80	TRIM3	Tripartite motif-containing protein 3	O75382	TRIM3	NA	Wald ratio	1	-0.090	0.080	2.618E-01	0.959	pan	SARS-CoV-2 infection
12686_15	THTM	3-mercaptopyruvate sulfurtransferase	P25325	MPST	NA	Inverse variance weighted	2	-0.057	0.051	2.620E-01	0.959	pan	SARS-CoV-2 infection
5076_53	EPHAA	Ephrin type-A receptor 10	Q5JZY3	EPHA10	NA	Wald ratio	1	0.074	0.066	2.623E-01	0.959	pan	SARS-CoV-2 infection
13536_56	POLI	DNA polymerase iota	Q9UNA4	POLI	NA	Wald ratio	1	-0.060	0.054	2.646E-01	0.964	pan	SARS-CoV-2 infection
12386_11	AMPB	Aminopeptidase B	Q9H4A4	RNPEP	NA	Inverse variance weighted	5	-0.023	0.021	2.647E-01	0.964	pan	SARS-CoV-2 infection
5676_54	ASIP	Agouti-signaling protein	P42127	ASIP	NA	Inverse variance weighted	11	-0.030	0.027	2.664E-01	0.969	pan	SARS-CoV-2 infection
15509_2	NAG	Alpha-N-acetylglucosaminidase	P54802	NAGLU	NA	Inverse variance weighted	28	0.009	0.008	2.675E-01	0.971	pan	SARS-CoV-2 infection
3831_21	pTEN	Phosphatidylinositol 3,4,5-trisphosphate 3-phosphatase and dual-specificity protein phosphatase PTEN	P60484	PTEN	NA	Wald ratio	1	-0.131	0.118	2.686E-01	0.971	pan	SARS-CoV-2 infection
8269_327	ARSK	Arylsulfatase K	Q6UWY0	ARSK	NA	Inverse variance weighted	2	0.075	0.068	2.693E-01	0.971	pan	SARS-CoV-2 infection
4153_11	alpha-1-antichymotrypsin complex	Alpha-1-antichymotrypsin complex	P07288 | P01011	KLK3 | SERPINA3	NA	Inverse variance weighted	3	0.054	0.049	2.698E-01	0.971	pan	SARS-CoV-2 infection
5459_33	CYTN	Cystatin-SN	P01037	CST1	NA	Inverse variance weighted	6	0.051	0.047	2.709E-01	0.971	pan	SARS-CoV-2 infection
17325_10	KGUA	Guanylate kinase	Q16774	GUK1	NA	Inverse variance weighted	6	-0.021	0.019	2.717E-01	0.971	pan	SARS-CoV-2 infection
2480_58	TIMP-3	Metalloproteinase inhibitor 3	P35625	TIMP3	NA	Inverse variance weighted	10	-0.010	0.009	2.718E-01	0.971	pan	SARS-CoV-2 infection
19229_92	HOME1	Homer protein homolog 1	Q86YM7	HOMER1	NA	Inverse variance weighted	4	0.077	0.070	2.719E-01	0.971	pan	SARS-CoV-2 infection
19115_13	CMBL	Carboxymethylenebutenolidase homolog	Q96DG6	CMBL	NA	Wald ratio	1	0.035	0.032	2.727E-01	0.971	pan	SARS-CoV-2 infection
13552_7	SWP70	Switch-associated protein 70	Q9UH65	SWAP70	NA	Inverse variance weighted	8	-0.012	0.011	2.727E-01	0.971	pan	SARS-CoV-2 infection
14047_78	BDNF	Brain-derived neurotrophic factor	P23560	BDNF	NA	Inverse variance weighted	2	-0.089	0.081	2.730E-01	0.971	pan	SARS-CoV-2 infection
9126_171	NT5D3	5'-nucleotidase domain-containing protein 3	Q86UY8	NT5DC3	NA	Inverse variance weighted	3	0.023	0.021	2.734E-01	0.971	pan	SARS-CoV-2 infection
16900_29	MDGA1	MAM domain-containing glycosylphosphatidylinositol anchor protein 1	Q8NFP4	MDGA1	NA	Inverse variance weighted	13	-0.011	0.010	2.736E-01	0.971	pan	SARS-CoV-2 infection
4969_2	Carbonic anhydrase I	Carbonic anhydrase 1	P00915	CA1	NA	Inverse variance weighted	6	-0.038	0.034	2.741E-01	0.971	pan	SARS-CoV-2 infection
4324_33	CYTT	Cystatin-SA	P09228	CST2	NA	Inverse variance weighted	9	0.037	0.034	2.743E-01	0.971	pan	SARS-CoV-2 infection
2700_56	Protein S	Vitamin K-dependent protein S	P07225	PROS1	NA	Inverse variance weighted	2	-0.074	0.068	2.744E-01	0.971	pan	SARS-CoV-2 infection
14100_63	C1QC	Complement C1q subcomponent subunit C	P02747	C1QC	NA	Inverse variance weighted	14	0.016	0.015	2.754E-01	0.971	pan	SARS-CoV-2 infection
15391_114	GAS-6	Growth arrest-specific protein 6	Q14393	GAS6	NA	Inverse variance weighted	5	-0.018	0.016	2.759E-01	0.971	pan	SARS-CoV-2 infection
14123_34	GI24:ECD	V-type immunoglobulin domain-containing suppressor of T-cell activation:Extracellular domain	Q9H7M9	VSIR	NA	Inverse variance weighted	6	0.041	0.037	2.762E-01	0.971	pan	SARS-CoV-2 infection
19289_29	DCUP	Uroporphyrinogen decarboxylase	P06132	UROD	NA	Inverse variance weighted	5	0.020	0.018	2.764E-01	0.971	pan	SARS-CoV-2 infection
3607_71	DKK3	Dickkopf-related protein 3	Q9UBP4	DKK3	NA	Inverse variance weighted	10	-0.017	0.016	2.764E-01	0.971	pan	SARS-CoV-2 infection
5609_92	F19A5	Protein FAM19A5	Q7Z5A7	TAFA5	NA	Wald ratio	1	0.110	0.101	2.767E-01	0.971	pan	SARS-CoV-2 infection
18326_50	MAGA8	Melanoma-associated antigen 8	P43361	MAGEA8	NA	Wald ratio	1	-0.119	0.110	2.778E-01	0.972	pan	SARS-CoV-2 infection
10756_34	UCN3	Urocortin-3	Q969E3	UCN3	NA	Wald ratio	1	-0.083	0.077	2.784E-01	0.972	pan	SARS-CoV-2 infection
8337_65	PTPRU	Receptor-type tyrosine-protein phosphatase U	Q92729	PTPRU	NA	Inverse variance weighted	5	-0.035	0.033	2.786E-01	0.972	pan	SARS-CoV-2 infection
18241_18	HEM6	Oxygen-dependent coproporphyrinogen-III oxidase, mitochondrial	P36551	CPOX	NA	Inverse variance weighted	10	-0.021	0.019	2.787E-01	0.972	pan	SARS-CoV-2 infection
16892_23	ENPP2	Ectonucleotide pyrophosphatase/phosphodiesterase family member 2	Q13822	ENPP2	NA	Inverse variance weighted	6	-0.029	0.027	2.802E-01	0.973	pan	SARS-CoV-2 infection
6364_7	TPSNR	Tapasin-related protein	Q9BX59	TAPBPL	NA	Inverse variance weighted	14	-0.009	0.008	2.803E-01	0.973	pan	SARS-CoV-2 infection
17137_160	Beta-globin	Hemoglobin subunit beta	P68871	HBB	NA	Inverse variance weighted	2	-0.068	0.063	2.806E-01	0.973	pan	SARS-CoV-2 infection
4962_52	ARMEL	Cerebral dopamine neurotrophic factor	Q49AH0	CDNF	NA	Inverse variance weighted	5	0.027	0.025	2.809E-01	0.973	pan	SARS-CoV-2 infection
11273_176	GSTT2	Glutathione S-transferase theta-2B	P0CG30	GSTT2B	NA	Inverse variance weighted	4	0.040	0.038	2.815E-01	0.973	pan	SARS-CoV-2 infection
3042_7	Myoglobin	Myoglobin	P02144	MB	NA	Inverse variance weighted	2	0.143	0.133	2.817E-01	0.973	pan	SARS-CoV-2 infection
4559_64	KYNU	Kynureninase	Q16719	KYNU	NA	Inverse variance weighted	6	-0.016	0.015	2.822E-01	0.973	pan	SARS-CoV-2 infection
14063_17	OSM	Oncostatin-M	P13725	OSM	NA	Wald ratio	1	-0.084	0.079	2.835E-01	0.975	pan	SARS-CoV-2 infection
10440_26	ACAM:ECD	CXADR-like membrane protein:Extracellular domain	Q9H6B4	CLMP	NA	Inverse variance weighted	3	0.035	0.032	2.836E-01	0.975	pan	SARS-CoV-2 infection
4209_60	DRG-1	Vacuolar protein sorting-associated protein VTA1 homolog	Q9NP79	VTA1	NA	Wald ratio	1	-0.098	0.092	2.848E-01	0.977	pan	SARS-CoV-2 infection
8916_32	STIM1:CD	Stromal interaction molecule 1:Cytoplasmic domain	Q13586	STIM1	NA	Wald ratio	1	-0.055	0.052	2.853E-01	0.978	pan	SARS-CoV-2 infection
9264_11	CATO	Cathepsin O	P43234	CTSO	NA	Inverse variance weighted	3	0.032	0.030	2.862E-01	0.979	pan	SARS-CoV-2 infection
17403_14	acyl-Coenzyme A dehydrogenase	Short/branched chain specific acyl-CoA dehydrogenase, mitochondrial	P45954	ACADSB	NA	Inverse variance weighted	4	0.036	0.034	2.877E-01	0.981	pan	SARS-CoV-2 infection
2972_57	BMP-7	Bone morphogenetic protein 7	P18075	BMP7	NA	Wald ratio	1	0.081	0.076	2.878E-01	0.981	pan	SARS-CoV-2 infection

10075_75	ACBD6	Acyl-CoA-binding domain-containing protein 6	Q9BR61	ACBD6	NA	Wald ratio	1	-0.093	0.088	2.888E-01	0.982	pan	SARS-CoV-2 infection
18189_12	VPS25	Vacuolar protein-sorting-associated protein 25	Q9BRG1	VPS25	NA	Inverse variance weighted	2	0.069	0.065	2.891E-01	0.982	pan	SARS-CoV-2 infection
14091_42	Carbonyl reductase 3	Carbonyl reductase [NADPH] 3	O75828	CBR3	NA	Inverse variance weighted	5	0.017	0.016	2.892E-01	0.982	pan	SARS-CoV-2 infection
9175_48	DSCAM	Down syndrome cell adhesion molecule	O60469	DSCAM	NA	Inverse variance weighted	2	-0.035	0.033	2.901E-01	0.983	pan	SARS-CoV-2 infection
16913_8	RNT2	Ribonuclease T2	O00584	RNASET2	NA	Inverse variance weighted	7	0.012	0.011	2.910E-01	0.984	pan	SARS-CoV-2 infection
3169_70	IDUA	Alpha-L-iduronidase	P35475	IDUA	NA	Inverse variance weighted	6	0.016	0.015	2.918E-01	0.984	pan	SARS-CoV-2 infection
5601_2	PGRP-L	N-acetylmuramoyl-L-alanine amidase	Q96PD5	PGLYRP2	NA	Inverse variance weighted	6	-0.030	0.028	2.919E-01	0.984	pan	SARS-CoV-2 infection
10880_38	F163B	Protein FAM163B	P0C2L3	FAM163B	NA	Inverse variance weighted	3	-0.070	0.067	2.934E-01	0.984	pan	SARS-CoV-2 infection
3438_10	FSTL3	Follistatin-related protein 3	O95633	FSTL3	NA	Inverse variance weighted	2	-0.092	0.087	2.935E-01	0.984	pan	SARS-CoV-2 infection
18338_26	IDH	Isocitrate dehydrogenase [NADP] cytoplasmic	O75874	IDH1	NA	Inverse variance weighted	3	-0.018	0.017	2.940E-01	0.984	pan	SARS-CoV-2 infection
7997_118	DOC2B	Double C2-like domain-containing protein beta	Q14184	DOC2B	NA	Wald ratio	1	-0.116	0.111	2.957E-01	0.984	pan	SARS-CoV-2 infection
15560_52	Apo-TC II	Transcobalamin-2	P20062	TCN2	NA	Inverse variance weighted	10	0.012	0.011	2.964E-01	0.984	pan	SARS-CoV-2 infection
5487_7	SLAF7	SLAM family member 7	Q9NQ25	SLAMF7	NA	Inverse variance weighted	14	-0.010	0.010	2.978E-01	0.984	pan	SARS-CoV-2 infection
9459_7	Fas, soluble	Tumor necrosis factor receptor superfamily member 6	P25445	FAS	NA	Inverse variance weighted	2	-0.085	0.082	2.985E-01	0.984	pan	SARS-CoV-2 infection
17783_9	MMAB	Cob(I)yrinic acid a,c-diamide adenosyltransferase, mitochondrial	Q96EY8	MMAB	NA	Inverse variance weighted	6	0.014	0.013	2.993E-01	0.984	pan	SARS-CoV-2 infection
12634_79	BCAR3:Ras-GEF	Breast cancer anti-estrogen resistance protein 3:Guanine Nucleotide Exchange Factor Domain	O75815	BCAR3	NA	Inverse variance weighted	3	-0.037	0.036	3.009E-01	0.984	pan	SARS-CoV-2 infection
4148_49	PAPP-A	Pappalysin-1	Q13219	PAPPA	NA	Inverse variance weighted	5	0.052	0.050	3.011E-01	0.984	pan	SARS-CoV-2 infection
5657_28	SIA4A	CMP-N-acetylneuraminate-beta-galactosamide-alpha-2,3-sialyltransferase 1	Q11201	ST3GAL1	NA	Inverse variance weighted	3	0.029	0.028	3.013E-01	0.984	pan	SARS-CoV-2 infection
8795_48	TR:ECD	Transferrin receptor protein 1:Extracellular domain	P02786	TFRC	NA	Inverse variance weighted	5	0.049	0.047	3.015E-01	0.984	pan	SARS-CoV-2 infection
12743_18	BAG5	BAG family molecular chaperone regulator 5	Q9UL15	BAG5	NA	Wald ratio	1	-0.101	0.098	3.020E-01	0.984	pan	SARS-CoV-2 infection
5092_51	JAG1:ECD	Protein jagged-1:Extracellular domain	P78504	JAG1	NA	Wald ratio	1	-0.073	0.071	3.023E-01	0.984	pan	SARS-CoV-2 infection
16609_106	KIRR2	Kin of IRRE-like protein 2	Q6UWL6	KIRREL2	NA	Inverse variance weighted	9	0.013	0.013	3.024E-01	0.984	pan	SARS-CoV-2 infection
16621_77	AIBP	NAD(P)H-hydrate epimerase	Q8NCW5	NAXE	NA	Wald ratio	1	0.029	0.028	3.026E-01	0.984	pan	SARS-CoV-2 infection
8989_40	SCUB1	Signal peptide, CUB and EGF-like domain-containing protein 1	Q8IWY4	SCUBE1	NA	Inverse variance weighted	6	0.027	0.027	3.026E-01	0.984	pan	SARS-CoV-2 infection
7935_26	LARGE	Glycosyltransferase-like protein LARGE1	O95461	LARGE1	NA	Inverse variance weighted	7	0.027	0.026	3.029E-01	0.984	pan	SARS-CoV-2 infection
17698_15	WBP2	WW domain-binding protein 2	Q969T9	WBP2	NA	Wald ratio	1	0.115	0.112	3.033E-01	0.984	pan	SARS-CoV-2 infection
18185_118	ALDOB	Fructose-bisphosphate aldolase B	P05062	ALDOB	NA	Inverse variance weighted	3	0.015	0.015	3.047E-01	0.984	pan	SARS-CoV-2 infection
10672_75	SP-B	Pulmonary surfactant-associated protein B	P07988	SFTPB	NA	Inverse variance weighted	6	-0.056	0.054	3.057E-01	0.984	pan	SARS-CoV-2 infection
14156_33	14-3-3 protein beta/alpha	14-3-3 protein beta/alpha	P31946	YWHAB	NA	Wald ratio	1	0.085	0.083	3.058E-01	0.984	pan	SARS-CoV-2 infection
5599_88	PCYXL	Prenylcysteine oxidase-like	Q8NBM8	PCYOX1L	NA	Wald ratio	1	-0.071	0.070	3.066E-01	0.984	pan	SARS-CoV-2 infection
16792_4	Siglec-5	Sialic acid-binding Ig-like lectin 5	O15389	SIGLEC5	NA	Inverse variance weighted	7	-0.014	0.014	3.090E-01	0.984	pan	SARS-CoV-2 infection
13405_61	ISK2	Serine protease inhibitor Kazal-type 2	P20155	SPINK2	NA	Inverse variance weighted	6	-0.012	0.012	3.094E-01	0.984	pan	SARS-CoV-2 infection
9545_156	Granzyme K	Granzyme K	P49863	GZMK	NA	Inverse variance weighted	3	-0.030	0.030	3.097E-01	0.984	pan	SARS-CoV-2 infection
9832_33	HGD	Homogentisate 1,2-dioxygenase	Q93099	HGD	NA	Inverse variance weighted	2	-0.042	0.042	3.097E-01	0.984	pan	SARS-CoV-2 infection
9384_17	Cathelicidin peptide	Cathelicidin antimicrobial peptide	P49913	CAMP	NA	Inverse variance weighted	8	-0.034	0.034	3.102E-01	0.984	pan	SARS-CoV-2 infection
9329_28	TRML1:ECD	Trem-like transcript 1 protein:Extracellular domain, Ig-like V-type domain	Q86YW5	TREML1	NA	Inverse variance weighted	2	-0.071	0.070	3.111E-01	0.984	pan	SARS-CoV-2 infection
9386_42	TTC9B	Tetratricopeptide repeat protein 9B	Q8N6N2	TTC9B	NA	Inverse variance weighted	2	0.040	0.039	3.113E-01	0.984	pan	SARS-CoV-2 infection
15610_72	LAP	Cytosol aminopeptidase	P28838	LAP3	NA	Inverse variance weighted	2	0.078	0.077	3.115E-01	0.984	pan	SARS-CoV-2 infection
11388_75	HE4	WAP four-disulfide core domain protein 2	Q14508	WFDC2	NA	Wald ratio	1	0.088	0.087	3.122E-01	0.984	pan	SARS-CoV-2 infection
15594_47	HTRA1	Serine protease HTRA1	Q92743	HTRA1	NA	Inverse variance weighted	2	-0.063	0.062	3.124E-01	0.984	pan	SARS-CoV-2 infection
9580_5	Laminin gamma-2	Laminin subunit gamma-2	Q13753	LAMC2	NA	Inverse variance weighted	9	-0.013	0.013	3.125E-01	0.984	pan	SARS-CoV-2 infection
12033_3	BUB1	Mitotic checkpoint serine/threonine-protein kinase BUB1	O43683	BUB1	NA	Wald ratio	1	-0.074	0.073	3.125E-01	0.984	pan	SARS-CoV-2 infection
12956_40	KBP	KIF1-binding protein	Q96EK5	KIFBP	NA	Wald ratio	1	0.113	0.112	3.129E-01	0.984	pan	SARS-CoV-2 infection
18343_10	DECR2	Peroxisomal 2,4-dienoyl-CoA reductase	Q9NUI1	DECR2	NA	Inverse variance weighted	8	0.027	0.027	3.134E-01	0.984	pan	SARS-CoV-2 infection
19560_23	PLXA4	Plexin-A4	Q9HCM2	PLXNA4	NA	Wald ratio	1	-0.058	0.058	3.134E-01	0.984	pan	SARS-CoV-2 infection
17456_53	GOLM1	Golgi membrane protein 1	Q8NBJ4	GOLM1	NA	Inverse variance weighted	6	-0.030	0.029	3.137E-01	0.984	pan	SARS-CoV-2 infection
15483_377	Agrin	Agrin	O00468	AGRN	NA	Inverse variance weighted	13	-0.020	0.020	3.142E-01	0.984	pan	SARS-CoV-2 infection
3495_15	GCP-2	C-X-C motif chemokine 6	P80162	CXCL6	NA	Inverse variance weighted	13	-0.015	0.014	3.143E-01	0.984	pan	SARS-CoV-2 infection
15604_18	JNK2	Mitogen-activated protein kinase 9	P45984	MAPK9	NA	Inverse variance weighted	2	-0.052	0.051	3.145E-01	0.984	pan	SARS-CoV-2 infection
17821_20	NMT2	Glycylpeptide N-tetradecanoyltransferase 2	O60551	NMT2	NA	Wald ratio	1	0.076	0.076	3.145E-01	0.984	pan	SARS-CoV-2 infection
10710_23	ZPBP2	Zona pellucida-binding protein 2	Q6X784	ZPBP2	NA	Wald ratio	1	-0.103	0.102	3.148E-01	0.984	pan	SARS-CoV-2 infection
18315_38	RTP4	Receptor-transporting protein 4	Q96DX8	RTP4	NA	Inverse variance weighted	4	0.024	0.023	3.148E-01	0.984	pan	SARS-CoV-2 infection
10037_98	SIG12:Ig-like V-type 2	Sialic acid-binding Ig-like lectin 12:Ig-like V-type 2 domain, Isoform long	Q96PQ1	SIGLEC12	NA	Inverse variance weighted	9	0.028	0.028	3.149E-01	0.984	pan	SARS-CoV-2 infection
14294_61	MBD1	Methyl-CpG-binding domain protein 1	Q9UIS9	MBD1	NA	Inverse variance weighted	3	0.058	0.058	3.151E-01	0.984	pan	SARS-CoV-2 infection
10557_6	TEX29	Testis-expressed sequence 29 protein	Q8N6K0	TEX29	NA	Inverse variance weighted	4	0.043	0.043	3.153E-01	0.984	pan	SARS-CoV-2 infection
19130_81	SPB8	Serpin B8	P50452	SERPINB8	NA	Inverse variance weighted	3	-0.021	0.021	3.158E-01	0.984	pan	SARS-CoV-2 infection
5687_5	GLU2B	Glucosidase 2 subunit beta	P14314	PRKCSH	NA	Inverse variance weighted	3	-0.060	0.060	3.158E-01	0.984	pan	SARS-CoV-2 infection
15363_32	Apo A-V	Apolipoprotein A-V	Q6Q788	APOA5	NA	Inverse variance weighted	11	0.015	0.014	3.158E-01	0.984	pan	SARS-CoV-2 infection
9235_3	PXDC1	Plexin domain-containing protein 1	Q8IUK5	PLXDC1	NA	Inverse variance weighted	6	-0.028	0.028	3.160E-01	0.984	pan	SARS-CoV-2 infection
16558_2	MYOC	Myocilin	Q99972	MYOC	NA	Inverse variance weighted	9	0.023	0.023	3.164E-01	0.984	pan	SARS-CoV-2 infection
10558_26	PCDH9	Protocadherin-9	Q9HC56	PCDH9	NA	Inverse variance weighted	8	-0.016	0.016	3.165E-01	0.984	pan	SARS-CoV-2 infection
15565_102	CA125	Mucin-16	Q8WXI7	MUC16	NA	Inverse variance weighted	8	-0.022	0.022	3.182E-01	0.984	pan	SARS-CoV-2 infection
7980_72	B3GN2	N-acetyllactosaminide beta-1,3-N-acetylglucosaminyltransferase 2	Q9NY97	B3GNT2	NA	Inverse variance weighted	5	0.025	0.025	3.183E-01	0.984	pan	SARS-CoV-2 infection
5349_69	DLL1	Delta-like protein 1	O00548	DLL1	NA	Inverse variance weighted	3	0.056	0.056	3.191E-01	0.984	pan	SARS-CoV-2 infection
10511_10	Collagen alpha-3(VI):isoform 3	Collagen alpha-3(VI) chain:isoform 3	P12111	COL6A3	NA	Inverse variance weighted	2	-0.060	0.061	3.193E-01	0.984	pan	SARS-CoV-2 infection
5813_58	Epo	Erythropoietin	P01588	EPO	NA	Inverse variance weighted	5	0.031	0.032	3.193E-01	0.984	pan	SARS-CoV-2 infection
3435_53	FN1.4	Fibronectin Fragment 4	P02751	FN1	NA	Inverse variance weighted	2	0.102	0.102	3.195E-01	0.984	pan	SARS-CoV-2 infection
18180_58	RT4I1	Reticulon-4-interacting protein 1, mitochondrial	Q8WWV3	RTN4IP1	NA	Wald ratio	1	-0.076	0.076	3.197E-01	0.984	pan	SARS-CoV-2 infection
15513_108	Prostasin	Prostasin	Q16651	PRSS8	NA	Wald ratio	1	0.103	0.103	3.206E-01	0.984	pan	SARS-CoV-2 infection
15347_12	Hemopexin	Hemopexin	P02790	HPX	NA	Wald ratio	1	-0.118	0.119	3.214E-01	0.984	pan	SARS-CoV-2 infection
5006_71	MK13	Mitogen-activated protein kinase 13	O15264	MAPK13	NA	Wald ratio	1	-0.049	0.050	3.221E-01	0.984	pan	SARS-CoV-2 infection
2658_27	TrkC	NT-3 growth factor receptor	Q16288	NTRK3	NA	Inverse variance weighted	6	0.024	0.024	3.228E-01	0.984	pan	SARS-CoV-2 infection
10758_2	KERA	Keratocan	O60938	KERA	NA	Inverse variance weighted	2	-0.055	0.056	3.235E-01	0.984	pan	SARS-CoV-2 infection
5061_27	B7-H2	ICOS ligand	O75144	ICOSLG	NA	Inverse variance weighted	3	-0.048	0.049	3.239E-01	0.984	pan	SARS-CoV-2 infection
5611_56	D108B	Beta-defensin 108B	Q8NET1	DEFB108B	NA	Wald ratio	1	-0.119	0.120	3.245E-01	0.984	pan	SARS-CoV-2 infection
6521_35	NPTX2	Neuronal pentraxin-2	P47972	NPTX2	NA	Inverse variance weighted	3	-0.040	0.041	3.253E-01	0.984	pan	SARS-CoV-2 infection
2644_11	PKC-A	Protein kinase C alpha type	P17252	PRKCA	NA	Wald ratio	1	-0.096	0.097	3.253E-01	0.984	pan	SARS-CoV-2 infection
11277_23	ATF6A	Cyclic AMP-dependent transcription factor ATF-6 alpha	P18850	ATF6	NA	Inverse variance weighted	10	-0.026	0.027	3.254E-01	0.984	pan	SARS-CoV-2 infection

17756_69	DCTD	Deoxycytidylate deaminase	P32321	DCTD	NA	Inverse variance weighted	2	0.047	0.048	3.257E-01	0.984	pan	SARS-CoV-2 infection
11431_235	RECQ1	ATP-dependent DNA helicase Q1	P46063	RECQL	NA	Inverse variance weighted	10	0.011	0.012	3.259E-01	0.984	pan	SARS-CoV-2 infection
9314_9	PSG5	Pregnancy-specific beta-1-glycoprotein 5	Q15238	PSG5	NA	Inverse variance weighted	4	0.046	0.047	3.261E-01	0.984	pan	SARS-CoV-2 infection
15494_11	FGFP1	Fibroblast growth factor-binding protein 1	Q14512	FGFBP1	NA	Inverse variance weighted	2	-0.049	0.050	3.265E-01	0.984	pan	SARS-CoV-2 infection
19135_5	Cytosolic 5'-nucleotidase III-like protein	7-methylguanosine phosphate-specific 5'-nucleotidase	Q969T7	NT5C3B	NA	Inverse variance weighted	13	-0.012	0.012	3.266E-01	0.984	pan	SARS-CoV-2 infection
9221_6	IPIL1	Inositol 1,4,5-trisphosphate receptor-interacting protein-like 1	Q6GPH6	ITPRIPL1	NA	Inverse variance weighted	5	-0.019	0.020	3.267E-01	0.984	pan	SARS-CoV-2 infection
6380_23	ICT1	Peptidyl-tRNA hydrolase ICT1, mitochondrial	Q14197	MRPL58	NA	Wald ratio	1	-0.066	0.067	3.274E-01	0.984	pan	SARS-CoV-2 infection
15441_6	SAP3	Ganglioside GM2 activator	P17900	GM2A	NA	Inverse variance weighted	6	-0.022	0.022	3.288E-01	0.984	pan	SARS-CoV-2 infection
10490_3	RPN1:CD	Dolichyl-diphosphooligosaccharide--protein glycosyltransferase subunit 1:Cytoplasmic domain	P04843	RPN1	NA	Wald ratio	1	-0.036	0.036	3.293E-01	0.984	pan	SARS-CoV-2 infection
13059_33	RIFK	Riboflavin kinase	Q969G6	RFK	NA	Inverse variance weighted	2	0.038	0.039	3.301E-01	0.984	pan	SARS-CoV-2 infection
11441_11	PYGL	Glycogen phosphorylase, liver form	P06737	PYGL	NA	Inverse variance weighted	7	-0.028	0.029	3.303E-01	0.984	pan	SARS-CoV-2 infection
7947_19	AP4AT	AP-4 complex accessory subunit tepsin	Q96N21	TEPSIN	NA	Wald ratio	1	0.084	0.086	3.310E-01	0.984	pan	SARS-CoV-2 infection
3213_65	Nidogen	Nidogen-1	P14543	NID1	NA	Inverse variance weighted	2	0.074	0.077	3.311E-01	0.984	pan	SARS-CoV-2 infection
3835_11	TLR2	Toll-like receptor 2	O60603	TLR2	NA	Wald ratio	1	0.123	0.127	3.317E-01	0.984	pan	SARS-CoV-2 infection
8982_65	TSP3	Thrombospondin-3	P49746	THBS3	NA	Inverse variance weighted	4	-0.065	0.067	3.317E-01	0.984	pan	SARS-CoV-2 infection
3299_29	Contactin-5	Contactin-5	O94779	CNTN5	NA	Inverse variance weighted	4	-0.018	0.019	3.320E-01	0.984	pan	SARS-CoV-2 infection
15521_4	Alcadein alpha-1	Calsyntenin-1	O94985	CLSTN1	NA	Inverse variance weighted	7	0.009	0.009	3.321E-01	0.984	pan	SARS-CoV-2 infection
8956_96	SREC-II:ECD	Scavenger receptor class F member 2:Extracellular domain	Q96GP6	SCARF2	NA	Inverse variance weighted	2	-0.069	0.071	3.322E-01	0.984	pan	SARS-CoV-2 infection
6393_63	Endoplasmin	Endoplasmin	P14625	HSP90B1	NA	Inverse variance weighted	10	0.014	0.015	3.324E-01	0.984	pan	SARS-CoV-2 infection
6520_87	MGP	Matrix Gla protein	P08493	MGP	NA	Inverse variance weighted	2	0.062	0.064	3.329E-01	0.984	pan	SARS-CoV-2 infection
5000_52	LG3BP	Galectin-3-binding protein	Q08380	LGALS3BP	NA	Inverse variance weighted	10	-0.021	0.022	3.330E-01	0.984	pan	SARS-CoV-2 infection
18831_6	LRIG1	Leucine-rich repeats and immunoglobulin-like domains protein 1	Q96JA1	LRIG1	NA	Inverse variance weighted	8	-0.012	0.013	3.345E-01	0.985	pan	SARS-CoV-2 infection
18934_50	Tissue transglutaminase	Protein-glutamine gamma-glutamyltransferase 2	P21980	TGM2	NA	Wald ratio	1	0.025	0.026	3.355E-01	0.985	pan	SARS-CoV-2 infection
7096_30	RMD1	Regulator of microtubule dynamics protein 1	Q96DB5	RMDN1	NA	Inverse variance weighted	5	-0.017	0.017	3.355E-01	0.985	pan	SARS-CoV-2 infection
3628_3	MP2K2	Dual specificity mitogen-activated protein kinase kinase 2	P36507	MAP2K2	NA	Inverse variance weighted	6	0.036	0.037	3.377E-01	0.985	pan	SARS-CoV-2 infection
3060_43	C9	Complement component C9	P02748	C9	NA	Inverse variance weighted	7	-0.013	0.013	3.395E-01	0.985	pan	SARS-CoV-2 infection
14175_78	SCP2D	SCP2 sterol-binding domain-containing protein 1	Q9UJQ7	SCP2D1	NA	Inverse variance weighted	7	-0.012	0.012	3.395E-01	0.985	pan	SARS-CoV-2 infection
15583_18	FCRLB	Fc receptor-like B	Q6BAA4	FCRLB	NA	Inverse variance weighted	4	0.028	0.029	3.398E-01	0.985	pan	SARS-CoV-2 infection
15539_15	SLIK1	SLIT and NTRK-like protein 1	Q96PX8	SLITRK1	NA	Wald ratio	1	-0.070	0.074	3.399E-01	0.985	pan	SARS-CoV-2 infection
3331_8	RGMB	RGM domain family member B	Q6NW40	RGMB	NA	Wald ratio	1	0.099	0.103	3.401E-01	0.985	pan	SARS-CoV-2 infection
12934_1	HERC5	E3 ISG15--protein ligase HERC5	Q9UII4	HERC5	NA	Wald ratio	1	-0.099	0.104	3.410E-01	0.985	pan	SARS-CoV-2 infection
14133_93	IL-1 sRII	Interleukin-1 receptor type 2	P27930	IL1R2	NA	Inverse variance weighted	12	0.017	0.017	3.426E-01	0.985	pan	SARS-CoV-2 infection
11257_1	DHPR	Dihydropteridine reductase	P09417	QDPR	NA	Inverse variance weighted	6	0.016	0.017	3.431E-01	0.985	pan	SARS-CoV-2 infection
7970_315	NAR3	Ecto-ADP-ribosyltransferase 3	Q13508	ART3	NA	Inverse variance weighted	9	0.016	0.017	3.432E-01	0.985	pan	SARS-CoV-2 infection
12968_2	CSRP2	Cysteine and glycine-rich protein 2	Q16527	CSRP2	NA	Wald ratio	1	-0.090	0.095	3.433E-01	0.985	pan	SARS-CoV-2 infection
2750_3	Apo A-I	Apolipoprotein A-I	P02647	APOA1	NA	Inverse variance weighted	8	-0.024	0.025	3.435E-01	0.985	pan	SARS-CoV-2 infection
11109_56	SVEP1:Sushi 15-18	Sushi, von Willebrand factor type A, EGF and pentraxin domain-containing protein 1:Sushi 15-18	Q4LDE5	SVEP1	NA	Inverse variance weighted	8	0.031	0.033	3.439E-01	0.985	pan	SARS-CoV-2 infection
5478_50	PSMA	Glutamate carboxypeptidase 2	Q04609	FOLH1	NA	Inverse variance weighted	4	-0.036	0.038	3.440E-01	0.985	pan	SARS-CoV-2 infection
19158_1	PAF	PCNA-associated factor	Q15004	PCLAF	NA	Inverse variance weighted	5	-0.040	0.042	3.444E-01	0.985	pan	SARS-CoV-2 infection
3481_87	XPNPEP1	Xaa-Pro aminopeptidase 1	Q9NQW7	XPNPEP1	NA	Wald ratio	1	-0.093	0.099	3.453E-01	0.985	pan	SARS-CoV-2 infection
12687_2	DECR	2,4-dienoyl-CoA reductase, mitochondrial	Q16698	DECR1	NA	Inverse variance weighted	6	-0.044	0.047	3.463E-01	0.985	pan	SARS-CoV-2 infection
11219_95	FGFP3	Fibroblast growth factor-binding protein 3	Q8TAT2	FGFBP3	NA	Inverse variance weighted	3	0.015	0.016	3.468E-01	0.985	pan	SARS-CoV-2 infection
2837_3	Met	Hepatocyte growth factor receptor	P08581	MET	NA	Inverse variance weighted	7	0.030	0.032	3.469E-01	0.985	pan	SARS-CoV-2 infection
16596_25	GLRX3	Glutaredoxin-3	O76003	GLRX3	NA	Wald ratio	1	-0.055	0.059	3.470E-01	0.985	pan	SARS-CoV-2 infection
9321_400	NMB	Neuromedin-B	P08949	NMB	NA	Inverse variance weighted	2	0.029	0.031	3.473E-01	0.985	pan	SARS-CoV-2 infection
9183_7	IFN-a/b R1	Interferon alpha/beta receptor 1	P17181	IFNAR1	NA	Inverse variance weighted	9	0.008	0.008	3.487E-01	0.985	pan	SARS-CoV-2 infection
3708_62	a2-Macroglobulin	Alpha-2-macroglobulin	P01023	A2M	NA	Inverse variance weighted	2	-0.052	0.056	3.489E-01	0.985	pan	SARS-CoV-2 infection
19175_18	MARCKSL1	MARCKS-related protein	P49006	MARCKSL1	NA	Inverse variance weighted	7	-0.032	0.034	3.505E-01	0.985	pan	SARS-CoV-2 infection
19637_9	CRH	Corticoliberin	P06850	CRH	NA	Wald ratio	1	0.076	0.081	3.510E-01	0.985	pan	SARS-CoV-2 infection
6234_74	VITRN	Vitrin	Q6UXI7	VIT	NA	Inverse variance weighted	9	-0.014	0.015	3.517E-01	0.985	pan	SARS-CoV-2 infection
8357_43	ST4A1	Sulfotransferase 4A1	Q9BR01	SULT4A1	NA	Wald ratio	1	0.083	0.090	3.527E-01	0.985	pan	SARS-CoV-2 infection
5737_61	SEM4D	Semaphorin-4D	Q92854	SEMA4D	NA	Inverse variance weighted	8	0.009	0.010	3.529E-01	0.985	pan	SARS-CoV-2 infection
18823_52	H2B3B	Histone H2B type 3-B	Q8N257	H2BU1	NA	Inverse variance weighted	2	-0.074	0.080	3.534E-01	0.985	pan	SARS-CoV-2 infection
19194_9	Histidyl-tRNA synthetase-related	D-tyrosyl-tRNA(Tyr) deacylase 1	Q8TEA8	DTD1	NA	Wald ratio	1	0.026	0.028	3.545E-01	0.985	pan	SARS-CoV-2 infection
16926_44	Alkaline phosphatase, liver	Alkaline phosphatase, tissue-nonspecific isozyme	P05186	ALPL	NA	Inverse variance weighted	3	0.051	0.055	3.549E-01	0.985	pan	SARS-CoV-2 infection
12571_14	ARL3	ADP-ribosylation factor-like protein 3	P36405	ARL3	NA	Inverse variance weighted	2	-0.032	0.035	3.550E-01	0.985	pan	SARS-CoV-2 infection
16882_27	PHP14	14 kDa phosphohistidine phosphatase	Q9NRX4	PHPT1	NA	Inverse variance weighted	2	0.069	0.075	3.554E-01	0.985	pan	SARS-CoV-2 infection
6077_63	CECR1	Adenosine deaminase CECR1	Q9NZK5	ADA2	NA	Inverse variance weighted	4	-0.023	0.025	3.555E-01	0.985	pan	SARS-CoV-2 infection
6715_63	PPBN	Alkaline phosphatase, placental-like	P10696	ALPG	NA	Inverse variance weighted	3	0.027	0.029	3.558E-01	0.985	pan	SARS-CoV-2 infection
15398_2	HERV1	FAD-linked sulfhydryl oxidase ALR	P55789	GFER	NA	Wald ratio	1	-0.079	0.086	3.561E-01	0.985	pan	SARS-CoV-2 infection
18931_40	SLIT3	Slit homolog 3 protein	O75094	SLIT3	NA	Inverse variance weighted	4	-0.029	0.031	3.563E-01	0.985	pan	SARS-CoV-2 infection
17460_51	Mx1	Interferon-induced GTP-binding protein Mx1	P20591	MX1	NA	Inverse variance weighted	9	-0.018	0.020	3.564E-01	0.985	pan	SARS-CoV-2 infection
2752_62	BMP-14	Growth/differentiation factor 5	P43026	GDF5	NA	Inverse variance weighted	2	0.047	0.051	3.570E-01	0.985	pan	SARS-CoV-2 infection
7959_34	CADH7	Cadherin-7	Q9ULB5	CDH7	NA	Inverse variance weighted	6	0.035	0.038	3.570E-01	0.985	pan	SARS-CoV-2 infection
10938_13	sLFA-3	Lymphocyte function-associated antigen 3	P19256	CD58	NA	Inverse variance weighted	2	-0.025	0.027	3.571E-01	0.985	pan	SARS-CoV-2 infection
18877_15	CNN2	Calponin-2	Q99439	CNN2	NA	Inverse variance weighted	2	0.037	0.041	3.580E-01	0.985	pan	SARS-CoV-2 infection
16288_17	EPHA4	Ephrin type-A receptor 4	P54764	EPHA4	NA	Inverse variance weighted	5	0.022	0.024	3.581E-01	0.985	pan	SARS-CoV-2 infection
9249_17	TMEM9:ECD	Transmembrane protein 9:Extracellular domain	Q9P0T7	TMEM9	NA	Inverse variance weighted	2	-0.042	0.045	3.581E-01	0.985	pan	SARS-CoV-2 infection
5229_90	IMDH1	Inosine-5'-monophosphate dehydrogenase 1	P20839	IMPDH1	NA	Inverse variance weighted	9	-0.025	0.028	3.586E-01	0.985	pan	SARS-CoV-2 infection
19277_4	KAT	Thiosulfate sulfurtransferase/rhodanese-like domain-containing protein 1	Q8NFU3	TSTD1	NA	Inverse variance weighted	3	0.028	0.031	3.588E-01	0.985	pan	SARS-CoV-2 infection
5493_17	WNK3	Serine/threonine-protein kinase WNK3	Q9BYP7	WNK3	NA	Wald ratio	1	-0.090	0.098	3.589E-01	0.985	pan	SARS-CoV-2 infection
5392_73	Fas, soluble	Tumor necrosis factor receptor superfamily member 6	P25445	FAS	NA	Inverse variance weighted	3	-0.046	0.050	3.591E-01	0.985	pan	SARS-CoV-2 infection
5223_59	GCKR	Glucokinase regulatory protein	Q14397	GCKR	NA	Wald ratio	1	0.072	0.078	3.600E-01	0.985	pan	SARS-CoV-2 infection
5867_60	ARGI1	Arginase-1	P05089	ARG1	NA	Wald ratio	1	0.058	0.063	3.600E-01	0.985	pan	SARS-CoV-2 infection
13438_115	CHRD	Chordin	Q9H2X0	CHRD	NA	Wald ratio	1	-0.063	0.069	3.600E-01	0.985	pan	SARS-CoV-2 infection
4548_4	Fucosyltransferase 3	Galactoside 3(4)-L-fucosyltransferase	P21217	FUT3	NA	Inverse variance weighted	7	-0.016	0.018	3.611E-01	0.985	pan	SARS-CoV-2 infection
4874_3	Angiogenin	Angiogenin	P03950	ANG	NA	Inverse variance weighted	6	0.029	0.031	3.629E-01	0.985	pan	SARS-CoV-2 infection
5610_32	CJ038	Protein FAM171A1	Q5VUB5	FAM171A1	NA	Inverse variance weighted	3	-0.056	0.061	3.634E-01	0.985	pan	SARS-CoV-2 infection

12605_1	Exosome component 3	Exosome complex component RRP40	Q9NQT5	EXOSC3	NA	Wald ratio	1	0.094	0.103	3.638E-01	0.985	pan	SARS-CoV-2 infection
15580_2	EPHA7	Ephrin type-A receptor 7	Q15375	EPHA7	NA	Wald ratio	1	-0.017	0.019	3.657E-01	0.985	pan	SARS-CoV-2 infection
7891_45	UGT 1A6	UDP-glucuronosyltransferase 1-6	P19224	UGT1A6	NA	Inverse variance weighted	4	-0.023	0.025	3.664E-01	0.985	pan	SARS-CoV-2 infection
12338_27	EGFLA	Pikachurin	Q63HQ2	EGFLAM	NA	Inverse variance weighted	3	0.016	0.017	3.669E-01	0.985	pan	SARS-CoV-2 infection
17455_42	FOLR1	Folate receptor alpha	P15328	FOLR1	NA	Wald ratio	1	-0.039	0.043	3.670E-01	0.985	pan	SARS-CoV-2 infection
19563_3	SEZ6L	Seizure 6-like protein	Q9BYH1	SEZ6L	NA	Inverse variance weighted	3	0.041	0.046	3.673E-01	0.985	pan	SARS-CoV-2 infection
2911_27	Midkine	Midkine	P21741	MDK	NA	Inverse variance weighted	3	0.066	0.073	3.674E-01	0.985	pan	SARS-CoV-2 infection
8957_72	XTP3B	Endoplasmic reticulum lectin 1	Q96DZ1	ERLEC1	NA	Wald ratio	1	-0.065	0.073	3.694E-01	0.985	pan	SARS-CoV-2 infection
3329_14	PGRP-S	Peptidoglycan recognition protein 1	O75594	PGLYRP1	NA	Inverse variance weighted	4	-0.039	0.044	3.701E-01	0.985	pan	SARS-CoV-2 infection
3326_58	Nectin-like protein 2	Cell adhesion molecule 1	Q9BY67	CADM1	NA	Wald ratio	1	0.031	0.035	3.707E-01	0.985	pan	SARS-CoV-2 infection
9574_11	BIN1	Myc box-dependent-interacting protein 1	O00499	BIN1	NA	Inverse variance weighted	2	0.031	0.034	3.719E-01	0.985	pan	SARS-CoV-2 infection
12820_1	GRAP	GRB2-related adapter protein	Q13588	GRAP	NA	Inverse variance weighted	2	-0.131	0.147	3.720E-01	0.985	pan	SARS-CoV-2 infection
11288_26	5NTC	Cytosolic purine 5'-nucleotidase	P49902	NT5C2	NA	Inverse variance weighted	3	-0.039	0.043	3.722E-01	0.985	pan	SARS-CoV-2 infection
7933_75	ADA22	Disintegrin and metalloproteinase domain-containing protein 22	Q9P0K1	ADAM22	NA	Inverse variance weighted	6	-0.015	0.017	3.729E-01	0.985	pan	SARS-CoV-2 infection
9599_6	PIANP	PILR alpha-associated neural protein	Q8IYJ0	PIANP	NA	Wald ratio	1	-0.093	0.105	3.730E-01	0.985	pan	SARS-CoV-2 infection
5465_32	H6ST1	Heparan-sulfate 6-O-sulfotransferase 1	O60243	HS6ST1	NA	Inverse variance weighted	2	-0.026	0.029	3.734E-01	0.985	pan	SARS-CoV-2 infection
16872_248	MAAI	Maleylacetoacetate isomerase	O43708	GSTZ1	NA	Inverse variance weighted	12	0.006	0.007	3.737E-01	0.985	pan	SARS-CoV-2 infection
9337_43	TKN1	Protachykinin-1	P20366	TAC1	NA	Inverse variance weighted	7	-0.019	0.021	3.746E-01	0.985	pan	SARS-CoV-2 infection
12449_16	PPIH	Peptidyl-prolyl cis-trans isomerase H	O43447	PPIH	NA	Inverse variance weighted	2	0.036	0.040	3.752E-01	0.985	pan	SARS-CoV-2 infection
9341_1	PDGFD	Platelet-derived growth factor D	Q9GZP0	PDGFD	NA	Inverse variance weighted	3	0.052	0.059	3.761E-01	0.985	pan	SARS-CoV-2 infection
8893_29	PARP:region 1	Poly [ADP-ribose] polymerase 1:region 1	P09874	PARP1	NA	Wald ratio	1	-0.082	0.093	3.762E-01	0.985	pan	SARS-CoV-2 infection
13676_46	Inhibin bB chain	Inhibin beta B chain	P09529	INHBB	NA	Inverse variance weighted	6	-0.018	0.020	3.766E-01	0.985	pan	SARS-CoV-2 infection
15384_15	KLOTHO	Klotho	Q9UEF7	KL	NA	Inverse variance weighted	15	-0.018	0.021	3.769E-01	0.985	pan	SARS-CoV-2 infection
9834_62	ADH1B	Alcohol dehydrogenase 1B	P00325	ADH1B	NA	Wald ratio	1	0.044	0.050	3.770E-01	0.985	pan	SARS-CoV-2 infection
4499_21	PDGF-AA	Platelet-derived growth factor subunit A	P04085	PDGFA	NA	Inverse variance weighted	3	-0.049	0.055	3.771E-01	0.985	pan	SARS-CoV-2 infection
4831_4	sL-Selectin	L-Selectin	P14151	SELL	NA	Inverse variance weighted	8	0.015	0.016	3.779E-01	0.985	pan	SARS-CoV-2 infection
5451_1	ALCAM	CD166 antigen	Q13740	ALCAM	NA	Inverse variance weighted	5	0.030	0.034	3.784E-01	0.985	pan	SARS-CoV-2 infection
13944_3	SULT 1A3	Sulfotransferase 1A3	P0DMM9	SULT1A3	NA	Inverse variance weighted	7	0.041	0.046	3.793E-01	0.985	pan	SARS-CoV-2 infection
6986_17	HS3SB	Heparan sulfate glucosamine 3-O-sulfotransferase 3B1	Q9Y662	HS3ST3B1	NA	Wald ratio	1	-0.105	0.120	3.800E-01	0.985	pan	SARS-CoV-2 infection
9816_37	ISOC1	Isochorismatase domain-containing protein 1	Q96CN7	ISOC1	NA	Inverse variance weighted	5	0.021	0.024	3.802E-01	0.985	pan	SARS-CoV-2 infection
17333_20	ACADM	Medium-chain specific acyl-CoA dehydrogenase, mitochondrial	P11310	ACADM	NA	Wald ratio	1	0.038	0.043	3.813E-01	0.985	pan	SARS-CoV-2 infection
18909_11	EXOS8	Exosome complex component RRP43	Q96B26	EXOSC8	NA	Wald ratio	1	0.064	0.073	3.814E-01	0.985	pan	SARS-CoV-2 infection
15558_63	AMPE	Glutamyl aminopeptidase	Q07075	ENPEP	NA	Inverse variance weighted	9	0.011	0.012	3.828E-01	0.985	pan	SARS-CoV-2 infection
8794_13	DPEP1	Dipeptidase 1	P16444	DPEP1	NA	Inverse variance weighted	7	0.014	0.016	3.834E-01	0.985	pan	SARS-CoV-2 infection
17400_71	ACOT8	Acyl-coenzyme A thioesterase 8	O14734	ACOT8	NA	Wald ratio	1	0.107	0.122	3.838E-01	0.985	pan	SARS-CoV-2 infection
4328_2	BOC	Brother of CDO	Q9BWV1	BOC	NA	Inverse variance weighted	4	0.034	0.039	3.839E-01	0.985	pan	SARS-CoV-2 infection
5350_14	GPC6	Glypican-6	Q9Y625	GPC6	NA	Inverse variance weighted	2	0.085	0.097	3.844E-01	0.985	pan	SARS-CoV-2 infection
15614_168	LIRA2	Leukocyte immunoglobulin-like receptor subfamily A member 2	Q8N149	LILRA2	NA	Inverse variance weighted	5	-0.016	0.018	3.845E-01	0.985	pan	SARS-CoV-2 infection
15589_1	Gc-Globulin, Mixed Type	Vitamin D-binding protein	P02774	GC	NA	Inverse variance weighted	4	-0.028	0.032	3.857E-01	0.985	pan	SARS-CoV-2 infection
18213_30	UBE2F	NEDD8-conjugating enzyme UBE2F	Q969M7	UBE2F	NA	Inverse variance weighted	2	-0.042	0.049	3.860E-01	0.985	pan	SARS-CoV-2 infection
11260_47	SPA4L	SUN domain-containing protein 5	Q8TC36	SUN5	NA	Inverse variance weighted	4	0.028	0.033	3.860E-01	0.985	pan	SARS-CoV-2 infection
15570_99	Complement receptor type 2	Complement receptor type 2	P20023	CR2	NA	Inverse variance weighted	16	-0.015	0.017	3.861E-01	0.985	pan	SARS-CoV-2 infection
5464_52	GRB2 adapter protein	Growth factor receptor-bound protein 2	P62993	GRB2	NA	Wald ratio	1	0.118	0.137	3.868E-01	0.985	pan	SARS-CoV-2 infection
6584_1	SRCH	Sarcoplasmic reticulum histidine-rich calcium-binding protein	P23327	HRC	NA	Wald ratio	1	-0.089	0.103	3.873E-01	0.985	pan	SARS-CoV-2 infection
8255_34	MRVI1	Protein MRVI1	Q9Y6F6	IRAG1	NA	Inverse variance weighted	2	-0.038	0.044	3.875E-01	0.985	pan	SARS-CoV-2 infection
11568_2	FKB1B	Peptidyl-prolyl cis-trans isomerase FKBP1B	P68106	FKBP1B	NA	Wald ratio	1	-0.037	0.043	3.875E-01	0.985	pan	SARS-CoV-2 infection
4413_3	SLPI	Antileukoproteinase	P03973	SLPI	NA	Wald ratio	1	-0.082	0.095	3.899E-01	0.985	pan	SARS-CoV-2 infection
4563_61	PLCG1	1-phosphatidylinositol 4,5-bisphosphate phosphodiesterase gamma-1	P19174	PLCG1	NA	Wald ratio	1	-0.074	0.086	3.901E-01	0.985	pan	SARS-CoV-2 infection
4960_72	annexin I	Annexin A1	P04083	ANXA1	NA	Wald ratio	1	0.033	0.038	3.911E-01	0.985	pan	SARS-CoV-2 infection
9844_138	ACTN2	Alpha-actinin-2	P35609	ACTN2	NA	Inverse variance weighted	5	0.043	0.050	3.911E-01	0.985	pan	SARS-CoV-2 infection
8019_73	STMN3	Stathmin-3	Q9NZ72	STMN3	NA	Wald ratio	1	-0.105	0.122	3.917E-01	0.985	pan	SARS-CoV-2 infection
13983_27	QOR	Quinone oxidoreductase	Q08257	CRYZ	NA	Inverse variance weighted	15	0.008	0.009	3.924E-01	0.985	pan	SARS-CoV-2 infection
7185_29	GPV	Platelet glycoprotein V	P40197	GP5	NA	Inverse variance weighted	2	-0.047	0.055	3.935E-01	0.985	pan	SARS-CoV-2 infection
4925_54	MMP-13	Collagenase 3	P45452	MMP13	NA	Inverse variance weighted	2	-0.046	0.054	3.938E-01	0.985	pan	SARS-CoV-2 infection
11557_3	SMUF1	E3 ubiquitin-protein ligase SMURF1	Q9HCE7	SMURF1	NA	Wald ratio	1	0.035	0.041	3.943E-01	0.985	pan	SARS-CoV-2 infection
3323_37	LRP8	Low-density lipoprotein receptor-related protein 8	Q14114	LRP8	NA	Inverse variance weighted	4	0.024	0.028	3.948E-01	0.985	pan	SARS-CoV-2 infection
5742_14	PPA6	Lysophosphatidic acid phosphatase type 6	Q9NPH0	ACP6	NA	Inverse variance weighted	21	-0.007	0.008	3.949E-01	0.985	pan	SARS-CoV-2 infection
9077_10	MA1A2	Mannosyl-oligosaccharide 1,2-alpha-mannosidase IB	O60476	MAN1A2	NA	Inverse variance weighted	5	-0.026	0.031	3.951E-01	0.985	pan	SARS-CoV-2 infection
16770_3	REG1B	Lithostathine-1-beta	P48304	REG1B	NA	Inverse variance weighted	4	0.071	0.084	3.953E-01	0.985	pan	SARS-CoV-2 infection
8364_74	UST	Uronyl 2-sulfotransferase	Q9Y2C2	UST	NA	Inverse variance weighted	5	0.023	0.027	3.955E-01	0.985	pan	SARS-CoV-2 infection
5139_32	UNC5H3	Netrin receptor UNC5C	O95185	UNC5C	NA	Inverse variance weighted	3	-0.050	0.059	3.956E-01	0.985	pan	SARS-CoV-2 infection
18342_2	SERC	Phosphoserine aminotransferase	Q9Y617	PSAT1	NA	Inverse variance weighted	2	-0.031	0.036	3.964E-01	0.985	pan	SARS-CoV-2 infection
18435_40	UBX2B	UBX domain-containing protein 2B	Q14CS0	UBXN2B	NA	Wald ratio	1	-0.068	0.080	3.972E-01	0.985	pan	SARS-CoV-2 infection
9759_13	INDO	Indoleamine 2,3-dioxygenase 1	P14902	IDO1	NA	Wald ratio	1	0.070	0.083	3.979E-01	0.985	pan	SARS-CoV-2 infection
12329_21	KS6A1	Ribosomal protein S6 kinase alpha-1	Q15418	RPS6KA1	NA	Inverse variance weighted	10	-0.029	0.034	3.982E-01	0.985	pan	SARS-CoV-2 infection
9449_150	C4b-binding protein alpha chain	C4b-binding protein alpha chain	P04003	C4BPA	NA	Inverse variance weighted	5	-0.026	0.031	3.994E-01	0.985	pan	SARS-CoV-2 infection
10512_13	IL3RB:ECD	Cytokine receptor common subunit beta:Extracellular domain	P32927	CSF2RB	NA	Inverse variance weighted	6	-0.013	0.015	3.998E-01	0.985	pan	SARS-CoV-2 infection
16785_45	HD-5	Defensin-5	Q01523	DEFA5	NA	Inverse variance weighted	17	-0.013	0.015	3.999E-01	0.985	pan	SARS-CoV-2 infection
11161_5	SPG20	Spartin	Q8N0X7	SPART	NA	Wald ratio	1	0.083	0.099	4.004E-01	0.985	pan	SARS-CoV-2 infection
3074_6	LBP	Lipopolysaccharide-binding protein	P18428	LBP	NA	Inverse variance weighted	10	0.020	0.023	4.010E-01	0.985	pan	SARS-CoV-2 infection
9076_25	PENK	Proenkephalin-A	P01210	PENK	NA	Inverse variance weighted	13	-0.008	0.009	4.012E-01	0.985	pan	SARS-CoV-2 infection
17755_5	UGDH	UDP-glucose 6-dehydrogenase	O60701	UGDH	NA	Inverse variance weighted	3	0.018	0.022	4.019E-01	0.985	pan	SARS-CoV-2 infection
5353_89	IL-1Ra	Interleukin-1 receptor antagonist protein	P18510	IL1RN	NA	Inverse variance weighted	4	-0.052	0.062	4.020E-01	0.985	pan	SARS-CoV-2 infection
2950_57	IGFBP-4	Insulin-like growth factor-binding protein 4	P22692	IGFBP4	NA	Inverse variance weighted	2	-0.054	0.065	4.027E-01	0.985	pan	SARS-CoV-2 infection
2977_7	EDAR	Tumor necrosis factor receptor superfamily member EDAR	Q9UNE0	EDAR	NA	Inverse variance weighted	15	-0.011	0.013	4.032E-01	0.985	pan	SARS-CoV-2 infection
7968_15	CRTAM	Cytotoxic and regulatory T-cell molecule	O95727	CRTAM	NA	Inverse variance weighted	7	-0.028	0.033	4.033E-01	0.985	pan	SARS-CoV-2 infection
13242_134	SHAN3	SH3 and multiple ankyrin repeat domains protein 3	Q9BYB0	SHANK3	NA	Inverse variance weighted	4	-0.037	0.045	4.039E-01	0.985	pan	SARS-CoV-2 infection
9931_20	Keratin-1	Keratin, type II cytoskeletal 1	P04264	KRT1	NA	Inverse variance weighted	2	-0.060	0.072	4.039E-01	0.985	pan	SARS-CoV-2 infection

4886_3	MCP-3	C-C motif chemokine 7	P80098	CCL7	NA	Inverse variance weighted	5	-0.017	0.021	4.040E-01	0.985	pan	SARS-CoV-2 infection
13427_66	MA1C1	Mannosyl-oligosaccharide 1,2-alpha-mannosidase IC	Q9NR34	MAN1C1	NA	Inverse variance weighted	4	0.027	0.032	4.042E-01	0.985	pan	SARS-CoV-2 infection
3234_23	URB	Coiled-coil domain-containing protein 80	Q76M96	CCDC80	NA	Inverse variance weighted	4	-0.039	0.047	4.042E-01	0.985	pan	SARS-CoV-2 infection
4912_17	GOT1	Aspartate aminotransferase, cytoplasmic	P17174	GOT1	NA	Wald ratio	1	0.071	0.085	4.043E-01	0.985	pan	SARS-CoV-2 infection
9266_1	sTREM-1	Triggering receptor expressed on myeloid cells 1	Q9NP99	TREM1	NA	Inverse variance weighted	7	0.019	0.023	4.049E-01	0.985	pan	SARS-CoV-2 infection
18401_18	ALKB3	Alpha-ketoglutarate-dependent dioxygenase alkB homolog 3	Q96Q83	ALKBH3	NA	Inverse variance weighted	6	-0.016	0.019	4.054E-01	0.985	pan	SARS-CoV-2 infection
3367_8	FETUB	Fetuin-B	Q9UGM5	FETUB	NA	Inverse variance weighted	7	-0.021	0.025	4.059E-01	0.985	pan	SARS-CoV-2 infection
18875_125	Chondrocalcin	Chondrocalcin	P02458	COL2A1	NA	Inverse variance weighted	19	-0.008	0.010	4.062E-01	0.985	pan	SARS-CoV-2 infection
9823_2	DYR	Dihydrofolate reductase	P00374	DHFR	NA	Inverse variance weighted	5	0.015	0.018	4.064E-01	0.985	pan	SARS-CoV-2 infection
11207_3	Macrophage scavenger receptor:CD	Macrophage scavenger receptor types I and II:Cytoplasmic domain	P21757	MSR1	NA	Wald ratio	1	-0.128	0.154	4.065E-01	0.985	pan	SARS-CoV-2 infection
18312_68	NDRG3	Protein NDRG3	Q9UGV2	NDRG3	NA	Inverse variance weighted	3	-0.049	0.059	4.066E-01	0.985	pan	SARS-CoV-2 infection
19196_73	HOP	Homeodomain-only protein	Q9BPY8	HOPX	NA	Wald ratio	1	-0.046	0.055	4.066E-01	0.985	pan	SARS-CoV-2 infection
9340_17	FKB14	Peptidyl-prolyl cis-trans isomerase FKBP14	Q9NWM8	FKBP14	NA	Wald ratio	1	0.083	0.099	4.068E-01	0.985	pan	SARS-CoV-2 infection
3344_60	Antithrombin III	Antithrombin-III	P01008	SERPINC1	NA	Wald ratio	1	0.069	0.083	4.069E-01	0.985	pan	SARS-CoV-2 infection
18289_16	MIP-5	C-C motif chemokine 15	Q16663	CCL15	NA	Inverse variance weighted	9	0.018	0.021	4.070E-01	0.985	pan	SARS-CoV-2 infection
3413_50	BFL1	Bcl-2-related protein A1	Q16548	BCL2A1	NA	Wald ratio	1	-0.088	0.107	4.071E-01	0.985	pan	SARS-CoV-2 infection
16323_8	NRX3A	Neurexin-3	Q9Y4C0	NRXN3	NA	Inverse variance weighted	3	0.047	0.057	4.074E-01	0.985	pan	SARS-CoV-2 infection
11481_25	TIMD3	Hepatitis A virus cellular receptor 2	Q8TDQ0	HAVCR2	NA	Wald ratio	1	0.091	0.110	4.074E-01	0.985	pan	SARS-CoV-2 infection
5861_78	3HAO	3-hydroxyanthranilate 3,4-dioxygenase	P46952	HAAO	NA	Inverse variance weighted	6	-0.056	0.068	4.081E-01	0.985	pan	SARS-CoV-2 infection
8804_39	COKA1	Collagen alpha-1(XX) chain	Q9P218	COL20A1	NA	Wald ratio	1	0.054	0.066	4.087E-01	0.985	pan	SARS-CoV-2 infection
5598_3	GREM2	Gremlin-2	Q9H772	GREM2	NA	Wald ratio	1	-0.069	0.084	4.090E-01	0.985	pan	SARS-CoV-2 infection
6470_19	fibulin 1	Fibulin-1	P23142	FBLN1	NA	Inverse variance weighted	4	-0.022	0.026	4.091E-01	0.985	pan	SARS-CoV-2 infection
3024_18	a2-Antiplasmin	Alpha-2-antiplasmin	P08697	SERPINF2	NA	Inverse variance weighted	6	-0.022	0.027	4.101E-01	0.985	pan	SARS-CoV-2 infection
6918_183	CCKN	Cholecystokinin	P06307	CCK	NA	Inverse variance weighted	2	0.052	0.063	4.105E-01	0.985	pan	SARS-CoV-2 infection
17802_4	SIAS	Sialic acid synthase	Q9NR45	NANS	NA	Wald ratio	1	-0.046	0.056	4.106E-01	0.985	pan	SARS-CoV-2 infection
3806_55	EphA5	Ephrin type-A receptor 5	P54756	EPHA5	NA	Inverse variance weighted	3	0.034	0.041	4.109E-01	0.985	pan	SARS-CoV-2 infection
8932_1	ENTP6	Ectonucleoside triphosphate diphosphohydrolase 6	O75354	ENTPD6	NA	Wald ratio	1	-0.078	0.095	4.116E-01	0.985	pan	SARS-CoV-2 infection
8028_22	SPINK5	Serine protease inhibitor Kazal-type 5	Q9NQ38	SPINK5	NA	Inverse variance weighted	2	-0.028	0.035	4.118E-01	0.985	pan	SARS-CoV-2 infection
2558_51	b-Endorphin	Beta-endorphin	P01189	POMC	NA	Wald ratio	1	-0.078	0.095	4.120E-01	0.985	pan	SARS-CoV-2 infection
4129_72	Factor B	Complement factor B	P00751	CFB	NA	Inverse variance weighted	3	0.036	0.044	4.121E-01	0.985	pan	SARS-CoV-2 infection
13931_22	PSMD9	26S proteasome non-ATPase regulatory subunit 9	O00233	PSMD9	NA	Wald ratio	1	0.032	0.039	4.124E-01	0.985	pan	SARS-CoV-2 infection
16853_5	NRK1	Nicotinamide riboside kinase 1	Q9NWW6	NMRK1	NA	Inverse variance weighted	2	0.024	0.030	4.129E-01	0.985	pan	SARS-CoV-2 infection
2190_55	Coagulation Factor XI	Coagulation Factor XI	P03951	F11	NA	Inverse variance weighted	5	-0.017	0.021	4.130E-01	0.985	pan	SARS-CoV-2 infection
6402_8	PILRA isoform FDF03-deltaTM	Paired immunoglobulin-like type 2 receptor alpha isoform FDF03-deltaTM	Q9UKJ1	PILRA	NA	Inverse variance weighted	14	-0.026	0.031	4.132E-01	0.985	pan	SARS-CoV-2 infection
12432_23	CYBP	Calcyclin-binding protein	Q9HB71	CACYBP	NA	Wald ratio	1	-0.058	0.071	4.133E-01	0.985	pan	SARS-CoV-2 infection
5085_18	IL-20 Ra	Interleukin-20 receptor subunit alpha	Q9UHF4	IL20RA	NA	Wald ratio	1	0.069	0.085	4.134E-01	0.985	pan	SARS-CoV-2 infection
4314_12	XTP3A	dCTP pyrophosphatase 1	Q9H773	DCTPP1	NA	Wald ratio	1	-0.059	0.072	4.137E-01	0.985	pan	SARS-CoV-2 infection
3807_1	FGF23	Fibroblast growth factor 23	Q9GZV9	FGF23	NA	Wald ratio	1	-0.077	0.094	4.141E-01	0.985	pan	SARS-CoV-2 infection
11530_37	HEM3	Porphobilinogen deaminase	P08397	HMBS	NA	Wald ratio	1	-0.077	0.094	4.156E-01	0.985	pan	SARS-CoV-2 infection
9511_61	NXPH2	Neurexophilin-2	O95156	NXPH2	NA	Inverse variance weighted	3	-0.043	0.053	4.156E-01	0.985	pan	SARS-CoV-2 infection
10948_14	PLD3	Phospholipase D3	Q8IV08	PLD3	NA	Wald ratio	1	-0.077	0.094	4.164E-01	0.985	pan	SARS-CoV-2 infection
6064_4	TXNDC4	Endoplasmic reticulum resident protein 44	Q9BS26	ERP44	NA	Inverse variance weighted	3	-0.049	0.060	4.165E-01	0.985	pan	SARS-CoV-2 infection
5680_54	OBP2B	Odorant-binding protein 2b	Q9NPH6	OBP2B	NA	Inverse variance weighted	6	0.008	0.010	4.165E-01	0.985	pan	SARS-CoV-2 infection
3196_6	HPLN1	Hyaluronan and proteoglycan link protein 1	P10915	HAPLN1	NA	Inverse variance weighted	3	0.031	0.039	4.171E-01	0.985	pan	SARS-CoV-2 infection
7179_69	NFASC	Neurofascin	O94856	NFASC	NA	Inverse variance weighted	4	-0.011	0.013	4.173E-01	0.985	pan	SARS-CoV-2 infection
12431_13	PELO	Protein pelota homolog	Q9BRX2	PELO	NA	Wald ratio	1	0.085	0.105	4.180E-01	0.985	pan	SARS-CoV-2 infection
5090_49	ILT-2	Leukocyte immunoglobulin-like receptor subfamily B member 1	Q8NHL6	LILRB1	NA	Inverse variance weighted	10	-0.012	0.014	4.188E-01	0.985	pan	SARS-CoV-2 infection
2647_66	Rab GDP dissociation inhibitor beta	Rab GDP dissociation inhibitor beta	P50395	GDI2	NA	Wald ratio	1	-0.040	0.050	4.189E-01	0.985	pan	SARS-CoV-2 infection
9841_197	Multifunctional protein ADE2	Multifunctional protein ADE2	P22234	PAICS	NA	Wald ratio	1	0.049	0.061	4.200E-01	0.985	pan	SARS-CoV-2 infection
12662_82	ECH1	Delta(3,5)-Delta(2,4)-dienoyl-CoA isomerase, mitochondrial	Q13011	ECH1	NA	Inverse variance weighted	2	-0.042	0.052	4.201E-01	0.985	pan	SARS-CoV-2 infection
2816_50	BCAM	Basal Cell Adhesion Molecule	P50895	BCAM	NA	Inverse variance weighted	4	-0.030	0.037	4.202E-01	0.985	pan	SARS-CoV-2 infection
12659_13	GTPB9	Obg-like ATPase 1	Q9NTK5	OLA1	NA	Inverse variance weighted	2	0.039	0.049	4.211E-01	0.985	pan	SARS-CoV-2 infection
6289_78	RHG36	Rho GTPase-activating protein 36	Q6ZRI8	ARHGAP36	NA	Wald ratio	1	0.072	0.090	4.213E-01	0.985	pan	SARS-CoV-2 infection
11643_73	DTX3L	E3 ubiquitin-protein ligase DTX3L	Q8TDB6	DTX3L	NA	Wald ratio	1	-0.059	0.073	4.214E-01	0.985	pan	SARS-CoV-2 infection
4984_83	Esterase D	S-formylglutathione hydrolase	P10768	ESD	NA	Inverse variance weighted	4	0.016	0.020	4.214E-01	0.985	pan	SARS-CoV-2 infection
7015_8	LIRB5	Leukocyte immunoglobulin-like receptor subfamily B member 5	O75023	LILRB5	NA	Inverse variance weighted	7	0.013	0.017	4.215E-01	0.985	pan	SARS-CoV-2 infection
13516_46	TMM85	ER membrane protein complex subunit 4	Q5J8M3	EMC4	NA	Inverse variance weighted	7	-0.012	0.014	4.221E-01	0.985	pan	SARS-CoV-2 infection
13988_67	NMRL1	NmrA-like family domain-containing protein 1	Q9HBL8	NMRAL1	NA	Inverse variance weighted	13	0.010	0.012	4.222E-01	0.985	pan	SARS-CoV-2 infection
5462_62	Ficolin-3	Ficolin-3	O75636	FCN3	NA	Inverse variance weighted	12	-0.011	0.014	4.229E-01	0.985	pan	SARS-CoV-2 infection
4840_73	G-CSF	Granulocyte colony-stimulating factor	P09919	CSF3	NA	Wald ratio	1	-0.141	0.176	4.234E-01	0.985	pan	SARS-CoV-2 infection
8017_23	FXRD1	FAD-dependent oxidoreductase domain-containing protein 1	Q96CU9	FOXRED1	NA	Wald ratio	1	0.086	0.107	4.241E-01	0.985	pan	SARS-CoV-2 infection
9370_69	GGH	Gamma-glutamyl hydrolase	Q92820	GGH	NA	Inverse variance weighted	13	0.009	0.011	4.241E-01	0.985	pan	SARS-CoV-2 infection
8310_6	U773	Zymogen granule protein 16 homolog B	Q96DA0	ZG16B	NA	Wald ratio	1	-0.025	0.031	4.247E-01	0.985	pan	SARS-CoV-2 infection
9863_1	Tropomyosin 4	Tropomyosin alpha-4 chain	P67936	TPM4	NA	Wald ratio	1	0.054	0.068	4.247E-01	0.985	pan	SARS-CoV-2 infection
10737_96	Serpin B1	Leukocyte elastase inhibitor	P30740	SERPINB1	NA	Wald ratio	1	0.060	0.075	4.256E-01	0.985	pan	SARS-CoV-2 infection
7905_30	HPT	Haptoglobin isoform 2	P00738	HP	NA	Wald ratio	1	-0.063	0.079	4.256E-01	0.985	pan	SARS-CoV-2 infection
15544_25	kallikrein 14	Kallikrein-14	Q9P0G3	KLK14	NA	Inverse variance weighted	5	-0.034	0.043	4.257E-01	0.985	pan	SARS-CoV-2 infection
8042_88	ISK9	Serine protease inhibitor Kazal-type 9	Q5DT21	SPINK9	NA	Inverse variance weighted	5	-0.032	0.041	4.262E-01	0.985	pan	SARS-CoV-2 infection
7857_22	NEUT	Neurotensin/neuromedin N	P30990	NTS	NA	Inverse variance weighted	5	0.023	0.029	4.266E-01	0.985	pan	SARS-CoV-2 infection
15516_12	SAA-4	Serum amyloid A-4 protein	P35542	SAA4	NA	Inverse variance weighted	8	-0.012	0.016	4.275E-01	0.985	pan	SARS-CoV-2 infection
19136_22	MMSA	Methylmalonate-semialdehyde dehydrogenase [acylating], mitochondrial	Q02252	ALDH6A1	NA	Inverse variance weighted	2	-0.046	0.058	4.278E-01	0.985	pan	SARS-CoV-2 infection
8360_169	NKp46	Natural cytotoxicity triggering receptor 1	O76036	NCR1	NA	Wald ratio	1	0.089	0.112	4.280E-01	0.985	pan	SARS-CoV-2 infection
4160_49	MMP-2	72 kDa type IV collagenase	P08253	MMP2	NA	Inverse variance weighted	3	0.041	0.052	4.294E-01	0.985	pan	SARS-CoV-2 infection
7154_92	CF058	Protein LEG1 homolog	Q6P5S2	LEG1	NA	Inverse variance weighted	5	-0.033	0.041	4.295E-01	0.985	pan	SARS-CoV-2 infection
17383_4	STAR5	StAR-related lipid transfer protein 5	Q9NSY2	STARD5	NA	Inverse variance weighted	6	0.025	0.031	4.298E-01	0.985	pan	SARS-CoV-2 infection
15368_3	BMPER	BMP-binding endothelial regulator protein	Q8N8U9	BMPER	NA	Inverse variance weighted	3	0.025	0.032	4.305E-01	0.985	pan	SARS-CoV-2 infection
13565_2	p130	Retinoblastoma-like protein 2	Q08999	RBL2	NA	Inverse variance weighted	6	-0.025	0.032	4.306E-01	0.985	pan	SARS-CoV-2 infection
16805_5	PDE5A	cGMP-specific 3',5'-cyclic phosphodiesterase	O76074	PDE5A	NA	Wald ratio	1	0.080	0.101	4.306E-01	0.985	pan	SARS-CoV-2 infection

11319_106	MRE11	Double-strand break repair protein MRE11	P49959	MRE11	NA	Wald ratio	1	0.075	0.095	4.317E-01	0.985	pan	SARS-CoV-2 infection
14254_27	PTN4	Tyrosine-protein phosphatase non-receptor type 4	P29074	PTPN4	NA	Inverse variance weighted	4	0.053	0.067	4.325E-01	0.985	pan	SARS-CoV-2 infection
10499_1	T106A	Transmembrane protein 106A	Q96A25	TMEM106A	NA	Inverse variance weighted	2	-0.052	0.066	4.325E-01	0.985	pan	SARS-CoV-2 infection
2967_8	VCAM-1	Vascular cell adhesion protein 1	P19320	VCAM1	NA	Inverse variance weighted	3	-0.044	0.056	4.326E-01	0.985	pan	SARS-CoV-2 infection
3352_80	Carbonic anhydrase 6	Carbonic anhydrase 6	P23280	CA6	NA	Inverse variance weighted	17	-0.008	0.010	4.328E-01	0.985	pan	SARS-CoV-2 infection
8958_51	CHL1	Neural cell adhesion molecule L1-like protein	O00533	CHL1	NA	Inverse variance weighted	8	0.013	0.017	4.330E-01	0.985	pan	SARS-CoV-2 infection
7266_4	SPA9	Serpin A9	Q86WD7	SERPINA9	NA	Wald ratio	1	0.052	0.067	4.330E-01	0.985	pan	SARS-CoV-2 infection
6392_7	WISP-2	WNT1-inducible-signaling pathway protein 2	O76076	CCN5	NA	Inverse variance weighted	10	0.010	0.012	4.345E-01	0.985	pan	SARS-CoV-2 infection
6525_17	DUSP13	Dual specificity protein phosphatase 13 isoform A	Q6B8I1	DUSP13	NA	Inverse variance weighted	3	0.014	0.018	4.349E-01	0.985	pan	SARS-CoV-2 infection
18925_24	Proteasome subunit alpha type 5	Proteasome subunit alpha type-5	P28066	PSMA5	NA	Wald ratio	1	0.082	0.105	4.354E-01	0.985	pan	SARS-CoV-2 infection
7551_33	LRC32	Leucine-rich repeat-containing protein 32	Q14392	LRRC32	NA	Wald ratio	1	-0.064	0.082	4.356E-01	0.985	pan	SARS-CoV-2 infection
11241_8	ARLY	Argininosuccinate lyase	P04424	ASL	NA	Inverse variance weighted	7	-0.034	0.044	4.365E-01	0.985	pan	SARS-CoV-2 infection
18876_77	CHST4	Carbohydrate sulfotransferase 4	Q8NCG5	CHST4	NA	Wald ratio	1	0.082	0.105	4.368E-01	0.985	pan	SARS-CoV-2 infection
7945_10	Semaphorin-6A	Semaphorin-6A	Q9H2E6	SEMA6A	NA	Inverse variance weighted	5	-0.032	0.041	4.376E-01	0.985	pan	SARS-CoV-2 infection
9265_10	GLIP1	Glioma pathogenesis-related protein 1	P48060	GLIPR1	NA	Wald ratio	1	0.042	0.055	4.378E-01	0.985	pan	SARS-CoV-2 infection
5939_42	TWEAK	Tumor necrosis factor ligand superfamily member 12	O43508	TNFSF12	NA	Inverse variance weighted	3	0.032	0.041	4.380E-01	0.985	pan	SARS-CoV-2 infection
6416_8	GKN2	Gastrokine-2	Q86XP6	GKN2	NA	Inverse variance weighted	10	0.017	0.022	4.380E-01	0.985	pan	SARS-CoV-2 infection
9294_45	MFAP2	Microfibrillar-associated protein 2	P55001	MFAP2	NA	Inverse variance weighted	2	0.096	0.124	4.384E-01	0.985	pan	SARS-CoV-2 infection
14011_17	S100A11	Protein S100-A11	P31949	S100A11	NA	Inverse variance weighted	3	-0.021	0.027	4.384E-01	0.985	pan	SARS-CoV-2 infection
13700_10	annexin II	Annexin A2	P07355	ANXA2	NA	Inverse variance weighted	8	-0.022	0.029	4.402E-01	0.985	pan	SARS-CoV-2 infection
2705_5	TECK	C-C motif chemokine 25	O15444	CCL25	NA	Inverse variance weighted	12	0.007	0.010	4.414E-01	0.985	pan	SARS-CoV-2 infection
3194_36	GPVI	Platelet glycoprotein VI	Q9HCN6	GP6	NA	Inverse variance weighted	2	-0.020	0.027	4.416E-01	0.985	pan	SARS-CoV-2 infection
5508_62	Cathepsin D	Cathepsin D	P07339	CTSD	NA	Inverse variance weighted	6	-0.020	0.026	4.421E-01	0.985	pan	SARS-CoV-2 infection
13477_65	LT4R1	Leukotriene B4 receptor 1	Q15722	LTB4R	NA	Inverse variance weighted	2	0.040	0.051	4.422E-01	0.985	pan	SARS-CoV-2 infection
5980_55	BOLA3	BolA-like protein 3	Q53S33	BOLA3	NA	Wald ratio	1	0.032	0.041	4.430E-01	0.985	pan	SARS-CoV-2 infection
6415_90	CPN2	Carboxypeptidase N subunit 2	P22792	CPN2	NA	Inverse variance weighted	6	0.023	0.031	4.433E-01	0.985	pan	SARS-CoV-2 infection
11177_16	K2C5	Keratin, type II cytoskeletal 5	P13647	KRT5	NA	Inverse variance weighted	7	0.028	0.036	4.434E-01	0.985	pan	SARS-CoV-2 infection
3504_58	LEAP-1	Hepcidin	P81172	HAMP	NA	Wald ratio	1	-0.074	0.097	4.440E-01	0.985	pan	SARS-CoV-2 infection
2982_82	Galectin-4	Galectin-4	P56470	LGALS4	NA	Wald ratio	1	-0.067	0.088	4.443E-01	0.985	pan	SARS-CoV-2 infection
13116_25	CD177	CD177 antigen	Q8N6Q3	CD177	NA	Inverse variance weighted	10	-0.009	0.012	4.450E-01	0.985	pan	SARS-CoV-2 infection
5532_53	bFGF-R	Fibroblast growth factor receptor 1	P11362	FGFR1	NA	Inverse variance weighted	5	0.027	0.036	4.457E-01	0.985	pan	SARS-CoV-2 infection
8005_1	MXRA7	Matrix-remodeling-associated protein 7	P84157	MXRA7	NA	Inverse variance weighted	5	-0.015	0.020	4.471E-01	0.985	pan	SARS-CoV-2 infection
5107_7	Notch 1	Neurogenic locus notch homolog protein 1	P46531	NOTCH1	NA	Inverse variance weighted	3	0.031	0.040	4.471E-01	0.985	pan	SARS-CoV-2 infection
7863_50	PGRC1	Membrane-associated progesterone receptor component 1	O00264	PGRMC1	NA	Wald ratio	1	0.075	0.099	4.474E-01	0.985	pan	SARS-CoV-2 infection
9484_75	Desmoglein-2	Desmoglein-2	Q14126	DSG2	NA	Inverse variance weighted	10	0.015	0.020	4.477E-01	0.985	pan	SARS-CoV-2 infection
12551_3	ITCH	E3 ubiquitin-protein ligase Itchy homolog	Q96J02	ITCH	NA	Wald ratio	1	0.083	0.110	4.480E-01	0.985	pan	SARS-CoV-2 infection
4234_8	IL-1 R4	Interleukin-1 receptor-like 1	Q01638	IL1RL1	NA	Inverse variance weighted	23	0.008	0.010	4.492E-01	0.985	pan	SARS-CoV-2 infection
2681_23	HGF	Hepatocyte growth factor	P14210	HGF	NA	Wald ratio	1	-0.036	0.047	4.494E-01	0.985	pan	SARS-CoV-2 infection
15414_316	LDHA	L-lactate dehydrogenase A chain	P00338	LDHA	NA	Wald ratio	1	-0.065	0.086	4.495E-01	0.985	pan	SARS-CoV-2 infection
5954_62	PTH	Parathyroid hormone	P01270	PTH	NA	Inverse variance weighted	2	0.031	0.041	4.501E-01	0.985	pan	SARS-CoV-2 infection
11287_14	Cytochrome b5	Cytochrome b5	P00167	CYB5A	NA	Wald ratio	1	-0.087	0.116	4.507E-01	0.985	pan	SARS-CoV-2 infection
15596_7	HEXI1	Protein HEXIM1	O94992	HEXIM1	NA	Wald ratio	1	-0.070	0.093	4.507E-01	0.985	pan	SARS-CoV-2 infection
8971_9	NRX1A	Neurexin-1	Q9ULB1	NRXN1	NA	Inverse variance weighted	6	-0.030	0.040	4.511E-01	0.985	pan	SARS-CoV-2 infection
8778_3	Noggin	Noggin	Q13253	NOG	NA	Inverse variance weighted	11	-0.014	0.019	4.519E-01	0.985	pan	SARS-CoV-2 infection
7861_9	ROR2	Tyrosine-protein kinase transmembrane receptor ROR2	Q01974	ROR2	NA	Inverse variance weighted	3	-0.022	0.029	4.520E-01	0.985	pan	SARS-CoV-2 infection
12510_3	STAP1	Signal-transducing adaptor protein 1	Q9ULZ2	STAP1	NA	Wald ratio	1	0.055	0.073	4.520E-01	0.985	pan	SARS-CoV-2 infection
18235_16	PGP	Glycerol-3-phosphate phosphatase	A6NDG6	PGP	NA	Inverse variance weighted	2	0.028	0.037	4.523E-01	0.985	pan	SARS-CoV-2 infection
13724_27	FGF-19	Fibroblast growth factor 19	O95750	FGF19	NA	Inverse variance weighted	2	0.041	0.055	4.524E-01	0.985	pan	SARS-CoV-2 infection
13105_7	SNP25	Synaptosomal-associated protein 25	P60880	SNAP25	NA	Wald ratio	1	0.075	0.100	4.532E-01	0.985	pan	SARS-CoV-2 infection
6439_59	CN093	Uncharacterized protein C14orf93	Q9H972	C14orf93	NA	Wald ratio	1	-0.074	0.099	4.533E-01	0.985	pan	SARS-CoV-2 infection
12395_86	SYDM	Aspartate--tRNA ligase, mitochondrial	Q6PI48	DARS2	NA	Wald ratio	1	0.076	0.101	4.535E-01	0.985	pan	SARS-CoV-2 infection
18458_4	PGES2	Prostaglandin E synthase 2	Q9H7Z7	PTGES2	NA	Wald ratio	1	0.048	0.064	4.545E-01	0.985	pan	SARS-CoV-2 infection
2632_5	IL-12 Rb1	Interleukin-12 receptor subunit beta-1	P42701	IL12RB1	NA	Wald ratio	1	-0.046	0.062	4.563E-01	0.985	pan	SARS-CoV-2 infection
8817_29	CENPV	Centromere protein V	Q7Z7K6	CENPV	NA	Wald ratio	1	-0.066	0.088	4.566E-01	0.985	pan	SARS-CoV-2 infection
7223_60	S100A13	Protein S100-A13	Q99584	S100A13	NA	Inverse variance weighted	6	0.021	0.028	4.570E-01	0.985	pan	SARS-CoV-2 infection
19228_11	BAG-2	BAG family molecular chaperone regulator 2	O95816	BAG2	NA	Inverse variance weighted	2	-0.069	0.093	4.575E-01	0.985	pan	SARS-CoV-2 infection
2834_54	kallikrein 8	Kallikrein-8	O60259	KLK8	NA	Inverse variance weighted	3	0.023	0.031	4.579E-01	0.985	pan	SARS-CoV-2 infection
7932_23	SYT8	Synaptotagmin-8	Q8NBV8	SYT8	NA	Wald ratio	1	-0.082	0.111	4.592E-01	0.985	pan	SARS-CoV-2 infection
17357_33	SPCS	O-phosphoseryl-tRNA(Sec) selenium transferase	Q9HD40	SEPSECS	NA	Wald ratio	1	-0.052	0.071	4.592E-01	0.985	pan	SARS-CoV-2 infection
12460_18	PSA7	Proteasome subunit alpha type-7	O14818	PSMA7	NA	Inverse variance weighted	3	0.023	0.031	4.593E-01	0.985	pan	SARS-CoV-2 infection
3305_6	DLL4	Delta-like protein 4	Q9NR61	DLL4	NA	Wald ratio	1	0.064	0.087	4.595E-01	0.985	pan	SARS-CoV-2 infection
7918_114	Amylase, alpha 1A	Alpha-amylase 1	P04745	AMY1A	NA	Inverse variance weighted	17	0.012	0.016	4.598E-01	0.985	pan	SARS-CoV-2 infection
15653_9	COAA1	Collagen alpha-1(X) chain	Q03692	COL10A1	NA	Inverse variance weighted	4	0.015	0.021	4.599E-01	0.985	pan	SARS-CoV-2 infection
12859_33	PECI	Enoyl-CoA delta isomerase 2, mitochondrial	O75521	ECI2	NA	Inverse variance weighted	4	0.013	0.018	4.600E-01	0.985	pan	SARS-CoV-2 infection
5604_30	HPSE	Heparanase	Q9Y251	HPSE	NA	Inverse variance weighted	2	-0.053	0.071	4.617E-01	0.985	pan	SARS-CoV-2 infection
10832_24	B4GT6	Beta-1,4-galactosyltransferase 6	Q9UBX8	B4GALT6	NA	Inverse variance weighted	12	-0.008	0.010	4.619E-01	0.985	pan	SARS-CoV-2 infection
16594_44	FAIM1	Fas apoptotic inhibitory molecule 1	Q9NVQ4	FAIM	NA	Inverse variance weighted	10	0.010	0.014	4.620E-01	0.985	pan	SARS-CoV-2 infection
13526_5	LA:RRM	Lupus La protein:RNA recognition motif	P05455	SSB	NA	Wald ratio	1	-0.059	0.080	4.622E-01	0.985	pan	SARS-CoV-2 infection
13123_3	FLRT3:ECD	Leucine-rich repeat transmembrane protein FLRT3:Extracellular domain	Q9NZU0	FLRT3	NA	Inverse variance weighted	9	0.006	0.009	4.622E-01	0.985	pan	SARS-CoV-2 infection
3122_6	SMAC	Diablo homolog, mitochondrial	Q9NR28	DIABLO	NA	Wald ratio	1	-0.074	0.100	4.623E-01	0.985	pan	SARS-CoV-2 infection
2946_52	Factor D	Complement factor D	P00746	CFD	NA	Inverse variance weighted	5	-0.030	0.041	4.627E-01	0.985	pan	SARS-CoV-2 infection
15491_20	CD248	Endosialin	Q9HCU0	CD248	NA	Wald ratio	1	0.067	0.091	4.627E-01	0.985	pan	SARS-CoV-2 infection
10852_114	HNRDL	Heterogeneous nuclear ribonucleoprotein D-like	O14979	HNRNPDL	NA	Wald ratio	1	-0.063	0.086	4.630E-01	0.985	pan	SARS-CoV-2 infection
8065_245	PATE4	Prostate and testis expressed protein 4	P0C8F1	PATE4	NA	Wald ratio	1	-0.029	0.039	4.640E-01	0.985	pan	SARS-CoV-2 infection
10531_18	RASN	GTPase NRas	P01111	NRAS	NA	Wald ratio	1	-0.100	0.137	4.641E-01	0.985	pan	SARS-CoV-2 infection
12689_56	ARC1B	Actin-related protein 2/3 complex subunit 1B	O15143	ARPC1B	NA	Wald ratio	1	0.040	0.055	4.645E-01	0.985	pan	SARS-CoV-2 infection
9506_10	Apo L1	Apolipoprotein L1	O14791	APOL1	NA	Inverse variance weighted	15	0.016	0.022	4.649E-01	0.985	pan	SARS-CoV-2 infection
16919_1	ACBP	Acyl-CoA-binding protein	P07108	DBI	NA	Inverse variance weighted	4	0.047	0.065	4.657E-01	0.985	pan	SARS-CoV-2 infection

4541_49	CDON	Cell adhesion molecule-related/down-regulated by oncogenes	Q4KMG0	CDON	NA	Inverse variance weighted	5	0.011	0.016	4.661E-01	0.985	pan	SARS-CoV-2 infection
4911_49	Glutathione S-transferase Pi	Glutathione S-transferase P	P09211	GSTP1	NA	Wald ratio	1	-0.026	0.036	4.663E-01	0.985	pan	SARS-CoV-2 infection
2637_77	Macrophage mannose receptor	Macrophage mannose receptor 1	P22897	MRC1	NA	Inverse variance weighted	13	-0.012	0.017	4.668E-01	0.985	pan	SARS-CoV-2 infection
18916_25	Inosine triphosphatase	Inosine triphosphate pyrophosphatase	Q9BY32	ITPA	NA	Inverse variance weighted	11	0.007	0.010	4.669E-01	0.985	pan	SARS-CoV-2 infection
8327_26	DPEP2	Dipeptidase 2	Q9H4A9	DPEP2	NA	Inverse variance weighted	6	0.017	0.024	4.670E-01	0.985	pan	SARS-CoV-2 infection
2631_50	IL-10 Rb	Interleukin-10 receptor subunit beta	Q08334	IL10RB	NA	Wald ratio	1	0.050	0.068	4.678E-01	0.986	pan	SARS-CoV-2 infection
7173_141	T132C:ECD	Transmembrane protein 132C:Extracellular domain	Q8N3T6	TMEM132C	NA	Inverse variance weighted	10	0.009	0.012	4.680E-01	0.986	pan	SARS-CoV-2 infection
3805_16	Endocan	Endothelial cell-specific molecule 1	Q9NQ30	ESM1	NA	Inverse variance weighted	5	-0.027	0.038	4.700E-01	0.989	pan	SARS-CoV-2 infection
6580_29	Pregnancy zone protein	Pregnancy zone protein	P20742	PZP	NA	Inverse variance weighted	5	-0.021	0.030	4.718E-01	0.990	pan	SARS-CoV-2 infection
2778_10	IL-22	Interleukin-22	Q9GZX6	IL22	NA	Wald ratio	1	-0.062	0.087	4.720E-01	0.990	pan	SARS-CoV-2 infection
19364_163	PCNA	Proliferating cell nuclear antigen	P12004	PCNA	NA	Inverse variance weighted	2	-0.051	0.072	4.729E-01	0.990	pan	SARS-CoV-2 infection
3320_49	IGFBP-7	Insulin-like growth factor-binding protein 7	Q16270	IGFBP7	NA	Inverse variance weighted	9	-0.014	0.019	4.742E-01	0.990	pan	SARS-CoV-2 infection
15322_35	CRADD	Death domain-containing protein CRADD	P78560	CRADD	NA	Inverse variance weighted	3	0.054	0.075	4.743E-01	0.990	pan	SARS-CoV-2 infection
15525_294	ADH1G	Alcohol dehydrogenase 1C	P00326	ADH1C	NA	Wald ratio	1	0.026	0.036	4.746E-01	0.990	pan	SARS-CoV-2 infection
17175_5	MP2K6	Dual specificity mitogen-activated protein kinase kinase 6	P52564	MAP2K6	NA	Inverse variance weighted	2	0.051	0.072	4.761E-01	0.990	pan	SARS-CoV-2 infection
18878_15	GREM1	Gremlin-1	O60565	GREM1	NA	Inverse variance weighted	10	0.008	0.011	4.764E-01	0.990	pan	SARS-CoV-2 infection
3309_2	FCG2A	Low affinity immunoglobulin gamma Fc region receptor II-a	P12318	FCGR2A	NA	Inverse variance weighted	8	-0.014	0.020	4.770E-01	0.990	pan	SARS-CoV-2 infection
4842_62	Glypican 3	Glypican-3	P51654	GPC3	NA	Inverse variance weighted	4	-0.041	0.058	4.774E-01	0.990	pan	SARS-CoV-2 infection
2654_19	TNF sR-I	Tumor necrosis factor receptor superfamily member 1A	P19438	TNFRSF1A	NA	Wald ratio	1	0.031	0.043	4.781E-01	0.990	pan	SARS-CoV-2 infection
15467_10	CTHR1	Collagen triple helix repeat-containing protein 1	Q96CG8	CTHRC1	NA	Inverse variance weighted	2	0.090	0.127	4.782E-01	0.990	pan	SARS-CoV-2 infection
4137_57	IL-17E	Interleukin-25	Q9H293	IL25	NA	Wald ratio	1	-0.057	0.080	4.786E-01	0.990	pan	SARS-CoV-2 infection
17843_30	PPCS	Phosphopantothenate--cysteine ligase	Q9HAB8	PPCS	NA	Inverse variance weighted	2	0.029	0.041	4.787E-01	0.990	pan	SARS-CoV-2 infection
4258_15	PA2G4	Proliferation-associated protein 2G4	Q9UQ80	PA2G4	NA	Wald ratio	1	0.059	0.083	4.792E-01	0.990	pan	SARS-CoV-2 infection
11387_3	ATF6B	Cyclic AMP-dependent transcription factor ATF-6 beta	Q99941	ATF6B	NA	Inverse variance weighted	4	-0.031	0.043	4.793E-01	0.990	pan	SARS-CoV-2 infection
2615_60	Ephrin-A5	Ephrin-A5	P52803	EFNA5	NA	Inverse variance weighted	3	0.034	0.049	4.799E-01	0.990	pan	SARS-CoV-2 infection
15530_33	EphB4	Ephrin type-B receptor 4	P54760	EPHB4	NA	Inverse variance weighted	4	0.027	0.038	4.811E-01	0.990	pan	SARS-CoV-2 infection
8243_55	TATI	Serine protease inhibitor Kazal-type 1	P00995	SPINK1	NA	Inverse variance weighted	2	-0.044	0.062	4.814E-01	0.990	pan	SARS-CoV-2 infection
14260_112	ARHG8	Neuroepithelial cell-transforming gene 1 protein	Q7Z628	NET1	NA	Inverse variance weighted	3	-0.040	0.057	4.815E-01	0.990	pan	SARS-CoV-2 infection
4990_87	GP1BA	Platelet glycoprotein Ib alpha chain	P07359	GP1BA	NA	Inverse variance weighted	9	-0.020	0.028	4.816E-01	0.990	pan	SARS-CoV-2 infection
13534_20	MYOM2	Myomesin-2	P54296	MYOM2	NA	Inverse variance weighted	3	0.043	0.061	4.824E-01	0.990	pan	SARS-CoV-2 infection
8309_12	HYAL1	Hyaluronidase-1	Q12794	HYAL1	NA	Wald ratio	1	-0.064	0.091	4.833E-01	0.990	pan	SARS-CoV-2 infection
8954_30	M4K1	Mitogen-activated protein kinase kinase kinase kinase 1	Q92918	MAP4K1	NA	Inverse variance weighted	6	-0.012	0.017	4.839E-01	0.990	pan	SARS-CoV-2 infection
7952_2	VTI1A	Vesicle transport through interaction with t-SNAREs homolog 1A	Q96AJ9	VTI1A	NA	Wald ratio	1	0.085	0.122	4.846E-01	0.990	pan	SARS-CoV-2 infection
6379_62	ATL2	ADAMTS-like protein 2	Q86TH1	ADAMTSL2	NA	Inverse variance weighted	2	-0.080	0.115	4.847E-01	0.990	pan	SARS-CoV-2 infection
17398_55	HO-1	Heme oxygenase 1	P09601	HMOX1	NA	Inverse variance weighted	10	-0.014	0.021	4.855E-01	0.990	pan	SARS-CoV-2 infection
14634_13	LSHR	Lutropin-choriogonadotropic hormone receptor	P22888	LHCGR	NA	Inverse variance weighted	5	-0.042	0.060	4.855E-01	0.990	pan	SARS-CoV-2 infection
4979_34	DERM	Dermatopontin	Q07507	DPT	NA	Inverse variance weighted	7	-0.009	0.013	4.856E-01	0.990	pan	SARS-CoV-2 infection
10080_9	EI2BA	Translation initiation factor eIF-2B subunit alpha	Q14232	EIF2B1	NA	Wald ratio	1	0.063	0.090	4.856E-01	0.990	pan	SARS-CoV-2 infection
7227_75	COCH	Cochlin	O43405	COCH	NA	Inverse variance weighted	17	0.010	0.014	4.857E-01	0.990	pan	SARS-CoV-2 infection
19488_1	PDCD6	Programmed cell death protein 6	O75340	PDCD6	NA	Inverse variance weighted	3	-0.024	0.034	4.858E-01	0.990	pan	SARS-CoV-2 infection
9171_11	CSRP3	Cysteine and glycine-rich protein 3	P50461	CSRP3	NA	Inverse variance weighted	2	0.043	0.062	4.863E-01	0.990	pan	SARS-CoV-2 infection
19241_31	RBP-III	Retinol-binding protein 5	P82980	RBP5	NA	Wald ratio	1	-0.067	0.096	4.864E-01	0.990	pan	SARS-CoV-2 infection
8007_19	Cathepsin B	Cathepsin B	P07858	CTSB	NA	Inverse variance weighted	11	0.010	0.014	4.867E-01	0.990	pan	SARS-CoV-2 infection
10554_23	BGAL	Beta-galactosidase	P16278	GLB1	NA	Wald ratio	1	0.075	0.108	4.870E-01	0.990	pan	SARS-CoV-2 infection
2687_2	MIA	Melanoma-derived growth regulatory protein	Q16674	MIA	NA	Inverse variance weighted	8	-0.007	0.010	4.885E-01	0.992	pan	SARS-CoV-2 infection
5034_79	Trypsin 2	Trypsin-2	P07478	PRSS2	NA	Inverse variance weighted	13	0.014	0.020	4.890E-01	0.992	pan	SARS-CoV-2 infection
15324_58	Ferritin light chain	Ferritin light chain	P02792	FTL	NA	Inverse variance weighted	4	-0.048	0.069	4.892E-01	0.992	pan	SARS-CoV-2 infection
13393_46	DERL1	Derlin-1	Q9BUN8	DERL1	NA	Wald ratio	1	-0.070	0.102	4.909E-01	0.995	pan	SARS-CoV-2 infection
4924_32	MMP-1	Interstitial collagenase	P03956	MMP1	NA	Inverse variance weighted	12	-0.008	0.012	4.920E-01	0.995	pan	SARS-CoV-2 infection
5763_67	HBD-4	Beta-defensin 104	Q8WTQ1	DEFB104A	NA	Wald ratio	1	-0.106	0.154	4.924E-01	0.995	pan	SARS-CoV-2 infection
12855_16	CT032	Cas scaffolding protein family member 4	Q9NQ75	CASS4	NA	Wald ratio	1	-0.061	0.089	4.940E-01	0.995	pan	SARS-CoV-2 infection
3484_60	Angiotensinogen	Angiotensinogen	P01019	AGT	NA	Inverse variance weighted	3	-0.027	0.039	4.941E-01	0.995	pan	SARS-CoV-2 infection
9969_8	S22AG	Solute carrier family 22 member 16	Q86VW1	SLC22A16	NA	Inverse variance weighted	3	0.043	0.063	4.945E-01	0.995	pan	SARS-CoV-2 infection
6586_19	ADA11	Disintegrin and metalloproteinase domain-containing protein 11	O75078	ADAM11	NA	Wald ratio	1	0.037	0.054	4.948E-01	0.995	pan	SARS-CoV-2 infection
5018_68	Peroxiredoxin-6	Peroxiredoxin-6	P30041	PRDX6	NA	Wald ratio	1	-0.054	0.080	4.955E-01	0.995	pan	SARS-CoV-2 infection
13642_90	CIG49	Interferon-induced protein with tetratricopeptide repeats 3	O14879	IFIT3	NA	Inverse variance weighted	2	0.034	0.050	4.968E-01	0.995	pan	SARS-CoV-2 infection
4437_56	ENTP5	Ectonucleoside triphosphate diphosphohydrolase 5	O75356	ENTPD5	NA	Inverse variance weighted	9	-0.008	0.012	4.969E-01	0.995	pan	SARS-CoV-2 infection
12534_10	CACO2	Calcium-binding and coiled-coil domain-containing protein 2	Q13137	CALCOCO2	NA	Wald ratio	1	-0.037	0.055	4.971E-01	0.995	pan	SARS-CoV-2 infection
9360_33	EDIL3	EGF-like repeat and discoidin I-like domain-containing protein 3	O43854	EDIL3	NA	Inverse variance weighted	3	0.034	0.051	4.976E-01	0.995	pan	SARS-CoV-2 infection
5744_12	CA056	Protein MENT	Q9BUN1	MENT	NA	Inverse variance weighted	4	-0.058	0.085	4.980E-01	0.995	pan	SARS-CoV-2 infection
19173_5	ZFAN1	AN1-type zinc finger protein 1	Q8TCF1	ZFAND1	NA	Inverse variance weighted	4	0.018	0.026	4.986E-01	0.995	pan	SARS-CoV-2 infection
5089_11	IL-7 Ra	Interleukin-7 receptor subunit alpha	P16871	IL7R	NA	Wald ratio	1	0.040	0.059	4.988E-01	0.995	pan	SARS-CoV-2 infection
3191_50	WFKN1	WAP, kazal, immunoglobulin, kunitz and NTR domain-containing protein 1	Q96NZ8	WFIKKN1	NA	Inverse variance weighted	2	0.028	0.042	4.991E-01	0.995	pan	SARS-CoV-2 infection
7251_64	C1QT3	Complement C1q tumor necrosis factor-related protein 3	Q9BXJ4	C1QTNF3	NA	Inverse variance weighted	3	-0.022	0.032	4.992E-01	0.995	pan	SARS-CoV-2 infection
9326_33	ITI heavy chain H2	Inter-alpha-trypsin inhibitor heavy chain H2	P19823	ITIH2	NA	Inverse variance weighted	10	0.012	0.018	5.000E-01	0.995	pan	SARS-CoV-2 infection
15526_33	GSHB	Glutathione synthetase	P48637	GSS	NA	Inverse variance weighted	5	-0.042	0.062	5.001E-01	0.995	pan	SARS-CoV-2 infection
10442_1	TM190	Transmembrane protein 190	Q8WZ59	TMEM190	NA	Inverse variance weighted	8	0.010	0.015	5.008E-01	0.995	pan	SARS-CoV-2 infection
9883_29	Glyoxalase I	Lactoylglutathione lyase	Q04760	GLO1	NA	Inverse variance weighted	2	-0.028	0.042	5.012E-01	0.995	pan	SARS-CoV-2 infection
3808_76	FGFR-2	Fibroblast growth factor receptor 2	P21802	FGFR2	NA	Wald ratio	1	-0.049	0.073	5.032E-01	0.995	pan	SARS-CoV-2 infection
2780_35	Lactoferrin	Lactotransferrin	P02788	LTF	NA	Inverse variance weighted	2	-0.069	0.103	5.035E-01	0.995	pan	SARS-CoV-2 infection
10818_36	ASM	Sphingomyelin phosphodiesterase	P17405	SMPD1	NA	Inverse variance weighted	11	0.008	0.012	5.039E-01	0.995	pan	SARS-CoV-2 infection
6605_17	IGFALS	Insulin-like growth factor-binding protein complex acid labile subunit	P35858	IGFALS	NA	Inverse variance weighted	3	-0.034	0.050	5.041E-01	0.995	pan	SARS-CoV-2 infection
3220_40	RET	Proto-oncogene tyrosine-protein kinase receptor Ret	P07949	RET	NA	Inverse variance weighted	5	-0.010	0.014	5.047E-01	0.995	pan	SARS-CoV-2 infection
6919_3	HBAZ	Hemoglobin subunit zeta	P02008	HBZ	NA	Inverse variance weighted	9	0.009	0.013	5.053E-01	0.995	pan	SARS-CoV-2 infection
18174_79	PDC6I	Programmed cell death 6-interacting protein	Q8WUM4	PDCD6IP	NA	Inverse variance weighted	2	-0.029	0.044	5.054E-01	0.995	pan	SARS-CoV-2 infection
7696_3	NBR1	Next to BRCA1 gene 1 protein	Q14596	NBR1	NA	Wald ratio	1	0.091	0.137	5.055E-01	0.995	pan	SARS-CoV-2 infection
19169_88	FIBP	Acidic fibroblast growth factor intracellular-binding protein	O43427	FIBP	NA	Wald ratio	1	0.041	0.062	5.061E-01	0.995	pan	SARS-CoV-2 infection
6425_87	MMP19	Matrix metalloproteinase-19	Q99542	MMP19	NA	Inverse variance weighted	4	0.028	0.042	5.081E-01	0.995	pan	SARS-CoV-2 infection

15542_19	KCRU	Creatine kinase U-type, mitochondrial	P12532	CKMT1A	NA	Inverse variance weighted	10	0.017	0.026	5.082E-01	0.995	pan	SARS-CoV-2 infection
10042_8	SGK3	Serine/threonine-protein kinase Sgk3	Q96BR1	SGK3	NA	Wald ratio	1	-0.061	0.093	5.084E-01	0.995	pan	SARS-CoV-2 infection
2737_22	NovH	Protein NOV homolog	P48745	CCN3	NA	Wald ratio	1	-0.075	0.113	5.098E-01	0.995	pan	SARS-CoV-2 infection
12459_13	PKHA1	Pleckstrin homology domain-containing family A member 1	Q9HB21	PLEKHA1	NA	Inverse variance weighted	3	0.011	0.017	5.104E-01	0.995	pan	SARS-CoV-2 infection
17512_2	NMI	N-myc-interactor	Q13287	NMI	NA	Wald ratio	1	0.084	0.128	5.104E-01	0.995	pan	SARS-CoV-2 infection
6930_95	SIA8F	Alpha-2,8-sialyltransferase 8F	P61647	ST8SIA6	NA	Wald ratio	1	-0.064	0.098	5.113E-01	0.995	pan	SARS-CoV-2 infection
18307_71	PPase 2	Inorganic pyrophosphatase 2, mitochondrial	Q9H2U2	PPA2	NA	Wald ratio	1	-0.036	0.055	5.120E-01	0.995	pan	SARS-CoV-2 infection
3796_79	ANGL4	Angiopoietin-related protein 4	Q9BY76	ANGPTL4	NA	Wald ratio	1	-0.054	0.083	5.127E-01	0.995	pan	SARS-CoV-2 infection
18299_13	CPNS1	Calpain small subunit 1	P04632	CAPNS1	NA	Inverse variance weighted	4	-0.021	0.032	5.138E-01	0.995	pan	SARS-CoV-2 infection
11347_9	Transaldolase	Transaldolase	P37837	TALDO1	NA	Inverse variance weighted	2	-0.032	0.049	5.141E-01	0.995	pan	SARS-CoV-2 infection
6373_54	DLK1	Protein delta homolog 1	P80370	DLK1	NA	Inverse variance weighted	3	-0.052	0.080	5.143E-01	0.995	pan	SARS-CoV-2 infection
14054_17	IL-15 Ra	Interleukin-15 receptor subunit alpha	Q13261	IL15RA	NA	Inverse variance weighted	8	0.009	0.013	5.144E-01	0.995	pan	SARS-CoV-2 infection
9981_18	K1467:C-term	Protein FAM234B:C-term	A2RU67	FAM234B	NA	Inverse variance weighted	4	-0.019	0.029	5.160E-01	0.995	pan	SARS-CoV-2 infection
11265_8	Retinal dehydrogenase 1	Retinal dehydrogenase 1	P00352	ALDH1A1	NA	Wald ratio	1	0.033	0.051	5.185E-01	0.995	pan	SARS-CoV-2 infection
5231_79	PCSK9	Proprotein convertase subtilisin/kexin type 9	Q8NBP7	PCSK9	NA	Inverse variance weighted	5	0.019	0.030	5.188E-01	0.995	pan	SARS-CoV-2 infection
8874_53	CLN5:LD	Ceroid-lipofuscinosis neuronal protein 5:Lumenal domain	O75503	CLN5	NA	Inverse variance weighted	2	0.029	0.045	5.189E-01	0.995	pan	SARS-CoV-2 infection
13955_33	DAPK1	Death-associated protein kinase 1	P53355	DAPK1	NA	Inverse variance weighted	3	0.062	0.097	5.190E-01	0.995	pan	SARS-CoV-2 infection
13388_57	NEC1	Neuroendocrine convertase 1	P29120	PCSK1	NA	Inverse variance weighted	12	-0.010	0.015	5.192E-01	0.995	pan	SARS-CoV-2 infection
5698_60	Tenascin-X	Tenascin-X	P22105	TNXB	NA	Inverse variance weighted	13	-0.007	0.011	5.197E-01	0.995	pan	SARS-CoV-2 infection
15636_49	SORC1	VPS10 domain-containing receptor SorCS1	Q8WY21	SORCS1	NA	Wald ratio	1	0.047	0.073	5.211E-01	0.995	pan	SARS-CoV-2 infection
5105_2	Nogo Receptor	Reticulon-4 receptor	Q9BZR6	RTN4R	NA	Inverse variance weighted	5	-0.009	0.014	5.213E-01	0.995	pan	SARS-CoV-2 infection
4188_1	Aflatoxin B1 aldehyde reductase	Aflatoxin B1 aldehyde reductase member 2	O43488	AKR7A2	NA	Wald ratio	1	0.021	0.032	5.213E-01	0.995	pan	SARS-CoV-2 infection
2851_63	C5a	C5a anaphylatoxin	P01031	C5	NA	Inverse variance weighted	4	0.041	0.064	5.213E-01	0.995	pan	SARS-CoV-2 infection
15468_14	FHR1	Complement factor H-related protein 1	Q03591	CFHR1	NA	Inverse variance weighted	11	0.017	0.027	5.215E-01	0.995	pan	SARS-CoV-2 infection
13544_9	HMHA1	Rho GTPase-activating protein 45	Q92619	ARHGAP45	NA	Wald ratio	1	0.025	0.040	5.221E-01	0.995	pan	SARS-CoV-2 infection
7926_13	SPIT3	Kunitz-type protease inhibitor 3	P49223	SPINT3	NA	Inverse variance weighted	4	0.027	0.042	5.227E-01	0.995	pan	SARS-CoV-2 infection
17814_8	BPNT1	3'(2'),5'-bisphosphate nucleotidase 1	O95861	BPNT1	NA	Wald ratio	1	0.054	0.085	5.242E-01	0.995	pan	SARS-CoV-2 infection
15387_44	Neuropilin-2	Neuropilin-2	O60462	NRP2	NA	Inverse variance weighted	6	0.014	0.023	5.256E-01	0.995	pan	SARS-CoV-2 infection
4922_13	MIP-3b	C-C motif chemokine 19	Q99731	CCL19	NA	Inverse variance weighted	2	-0.054	0.085	5.265E-01	0.995	pan	SARS-CoV-2 infection
10708_3	GON2	Progonadoliberin-2	O43555	GNRH2	NA	Wald ratio	1	-0.029	0.047	5.266E-01	0.995	pan	SARS-CoV-2 infection
8469_41	IGFBP-2	Insulin-like growth factor-binding protein 2	P18065	IGFBP2	NA	Inverse variance weighted	5	-0.031	0.048	5.268E-01	0.995	pan	SARS-CoV-2 infection
14134_49	AMGO2	Amphoterin-induced protein 2	Q86SJ2	AMIGO2	NA	Inverse variance weighted	3	0.024	0.039	5.273E-01	0.995	pan	SARS-CoV-2 infection
3285_23	C1r	Complement C1r subcomponent	P00736	C1R	NA	Inverse variance weighted	2	-0.057	0.090	5.285E-01	0.995	pan	SARS-CoV-2 infection
9267_2	CBPA4	Carboxypeptidase A4	Q9UI42	CPA4	NA	Inverse variance weighted	22	-0.003	0.005	5.287E-01	0.995	pan	SARS-CoV-2 infection
13109_82	NEGR1	Neuronal growth regulator 1	Q7Z3B1	NEGR1	NA	Inverse variance weighted	2	0.039	0.062	5.287E-01	0.995	pan	SARS-CoV-2 infection
6383_90	TLL1	Tolloid-like protein 1	O43897	TLL1	NA	Inverse variance weighted	4	0.029	0.046	5.289E-01	0.995	pan	SARS-CoV-2 infection
8100_15	ADM2	ADM2	Q7Z4H4	ADM2	NA	Wald ratio	1	0.055	0.088	5.301E-01	0.995	pan	SARS-CoV-2 infection
8248_222	SIG14	Sialic acid-binding Ig-like lectin 14	Q08ET2	SIGLEC14	NA	Inverse variance weighted	4	0.052	0.083	5.303E-01	0.995	pan	SARS-CoV-2 infection
9061_3	ARHGA	Rho guanine nucleotide exchange factor 10	O15013	ARHGEF10	NA	Wald ratio	1	0.042	0.067	5.313E-01	0.995	pan	SARS-CoV-2 infection
7144_234	KAZD1	Kazal-type serine protease inhibitor domain-containing protein 1	Q96I82	KAZALD1	NA	Wald ratio	1	-0.055	0.087	5.315E-01	0.995	pan	SARS-CoV-2 infection
17377_1	Aldose reductase-like C3	Aldo-keto reductase family 1 member C3	P42330	AKR1C3	NA	Inverse variance weighted	9	0.014	0.023	5.335E-01	0.995	pan	SARS-CoV-2 infection
11608_5	MLP3B	Microtubule-associated proteins 1A/1B light chain 3B	Q9GZQ8	MAP1LC3B	NA	Wald ratio	1	-0.077	0.124	5.337E-01	0.995	pan	SARS-CoV-2 infection
4996_66	HRG	Histidine-rich glycoprotein	P04196	HRG	NA	Inverse variance weighted	8	0.016	0.026	5.339E-01	0.995	pan	SARS-CoV-2 infection
4276_10	prostatic binding protein	Phosphatidylethanolamine-binding protein 1	P30086	PEBP1	NA	Inverse variance weighted	10	-0.009	0.014	5.343E-01	0.995	pan	SARS-CoV-2 infection
8773_172	EMIL3:region 1	EMILIN-3:region 1	Q9NT22	EMILIN3	NA	Inverse variance weighted	7	-0.009	0.015	5.344E-01	0.995	pan	SARS-CoV-2 infection
18285_6	SVIP	Small VCP/p97-interacting protein	Q8NHG7	SVIP	NA	Inverse variance weighted	2	0.088	0.141	5.352E-01	0.995	pan	SARS-CoV-2 infection
12556_7	UBE2C	Ubiquitin-conjugating enzyme E2 C	O00762	UBE2C	NA	Inverse variance weighted	2	0.027	0.044	5.355E-01	0.995	pan	SARS-CoV-2 infection
2475_1	SCF sR	Mast/stem cell growth factor receptor Kit	P10721	KIT	NA	Inverse variance weighted	10	-0.017	0.027	5.355E-01	0.995	pan	SARS-CoV-2 infection
17490_4	SH3BGRL3-like protein	SH3 domain-binding glutamic acid-rich-like protein 3	Q9H299	SH3BGRL3	NA	Inverse variance weighted	4	-0.021	0.034	5.355E-01	0.995	pan	SARS-CoV-2 infection
17751_68	CRBB1	Beta-crystallin B1	P53674	CRYBB1	NA	Inverse variance weighted	3	-0.015	0.025	5.362E-01	0.995	pan	SARS-CoV-2 infection
5069_9	DAF	Complement decay-accelerating factor	P08174	CD55	NA	Inverse variance weighted	5	-0.015	0.025	5.363E-01	0.995	pan	SARS-CoV-2 infection
10908_2	GLT13	Polypeptide N-acetylgalactosaminyltransferase 13	Q8IUC8	GALNT13	NA	Wald ratio	1	-0.052	0.084	5.373E-01	0.995	pan	SARS-CoV-2 infection
15321_8	CPLX2	Complexin-2	Q6PUV4	CPLX2	NA	Wald ratio	1	0.066	0.108	5.397E-01	0.995	pan	SARS-CoV-2 infection
9751_72	NSEP1	Nuclease-sensitive element-binding protein 1	P67809	YBX1	NA	Inverse variance weighted	5	0.026	0.042	5.402E-01	0.995	pan	SARS-CoV-2 infection
17696_1	TIRP	TIR domain-containing adapter molecule 2	Q86XR7	TICAM2	NA	Wald ratio	1	0.095	0.155	5.403E-01	0.995	pan	SARS-CoV-2 infection
4374_45	MIC-1	Growth/differentiation factor 15	Q99988	GDF15	NA	Inverse variance weighted	9	0.008	0.012	5.407E-01	0.995	pan	SARS-CoV-2 infection
8479_4	MMP-10	Stromelysin-2	P09238	MMP10	NA	Inverse variance weighted	9	0.011	0.018	5.410E-01	0.995	pan	SARS-CoV-2 infection
4249_64	NDP kinase B	Nucleoside diphosphate kinase B	P22392	NME2	NA	Wald ratio	1	0.020	0.033	5.415E-01	0.995	pan	SARS-CoV-2 infection
19261_12	SDCB2	Syntenin-2	Q9H190	SDCBP2	NA	Wald ratio	1	0.042	0.068	5.421E-01	0.995	pan	SARS-CoV-2 infection
12450_42	PMVK	Phosphomevalonate kinase	Q15126	PMVK	NA	Inverse variance weighted	2	0.094	0.154	5.422E-01	0.995	pan	SARS-CoV-2 infection
17758_79	DCXR	L-xylulose reductase	Q7Z4W1	DCXR	NA	Inverse variance weighted	2	-0.040	0.066	5.425E-01	0.995	pan	SARS-CoV-2 infection
5131_15	TAJ	Tumor necrosis factor receptor superfamily member 19	Q9NS68	TNFRSF19	NA	Wald ratio	1	-0.029	0.048	5.426E-01	0.995	pan	SARS-CoV-2 infection
17199_43	HIF1N	Hypoxia-inducible factor 1-alpha inhibitor	Q9NWT6	HIF1AN	NA	Wald ratio	1	-0.101	0.166	5.430E-01	0.995	pan	SARS-CoV-2 infection
3366_51	ECM1	Extracellular matrix protein 1	Q16610	ECM1	NA	Inverse variance weighted	15	-0.007	0.011	5.436E-01	0.995	pan	SARS-CoV-2 infection
3232_28	TrATPase	Tartrate-resistant acid phosphatase type 5	P13686	ACP5	NA	Inverse variance weighted	7	0.012	0.020	5.439E-01	0.995	pan	SARS-CoV-2 infection
17350_13	CHM2B	Charged multivesicular body protein 2b	Q9UQN3	CHMP2B	NA	Inverse variance weighted	3	-0.018	0.029	5.440E-01	0.995	pan	SARS-CoV-2 infection
3038_9	I-TAC	C-X-C motif chemokine 11	O14625	CXCL11	NA	Inverse variance weighted	8	-0.022	0.037	5.447E-01	0.995	pan	SARS-CoV-2 infection
16610_13	LRP10	Low-density lipoprotein receptor-related protein 10	Q7Z4F1	LRP10	NA	Inverse variance weighted	3	0.047	0.077	5.453E-01	0.995	pan	SARS-CoV-2 infection
13126_52	DSC2	Desmocollin-2	Q02487	DSC2	NA	Inverse variance weighted	3	-0.018	0.029	5.455E-01	0.995	pan	SARS-CoV-2 infection
8402_22	CYTL1	Cytokine-like protein 1	Q9NRR1	CYTL1	NA	Inverse variance weighted	4	-0.009	0.014	5.456E-01	0.995	pan	SARS-CoV-2 infection
9482_110	NUDT9	ADP-ribose pyrophosphatase, mitochondrial	Q9BW91	NUDT9	NA	Wald ratio	1	-0.048	0.080	5.460E-01	0.995	pan	SARS-CoV-2 infection
6538_90	K2013	Uncharacterized protein KIAA2013	Q8IYS2	KIAA2013	NA	Inverse variance weighted	2	0.060	0.099	5.461E-01	0.995	pan	SARS-CoV-2 infection
17677_47	BY55	CD160 antigen	O95971	CD160	NA	Wald ratio	1	-0.029	0.048	5.462E-01	0.995	pan	SARS-CoV-2 infection
11696_7	RABP2	Cellular retinoic acid-binding protein 2	P29373	CRABP2	NA	Wald ratio	1	0.019	0.031	5.468E-01	0.995	pan	SARS-CoV-2 infection
4332_6	CLC1B	C-type lectin domain family 1 member B	Q9P126	CLEC1B	NA	Wald ratio	1	-0.041	0.068	5.483E-01	0.995	pan	SARS-CoV-2 infection
15447_45	Sorbitol dehydrogenase	Sorbitol dehydrogenase	Q00796	SORD	NA	Inverse variance weighted	6	0.012	0.020	5.485E-01	0.995	pan	SARS-CoV-2 infection
3044_3	PARC	C-C motif chemokine 18	P55774	CCL18	NA	Inverse variance weighted	9	-0.017	0.029	5.489E-01	0.995	pan	SARS-CoV-2 infection
5102_55	MICB	MHC class I polypeptide-related sequence B	Q29980	MICB	NA	Inverse variance weighted	14	-0.010	0.016	5.495E-01	0.995	pan	SARS-CoV-2 infection

6920_1	GFRAL	GDNF family receptor alpha-like	Q6UXV0	GFRAL	NA	Inverse variance weighted	6	0.013	0.022	5.498E-01	0.995	pan	SARS-CoV-2 infection
2652_15	suPAR	Urokinase plasminogen activator surface receptor	Q03405	PLAUR	NA	Wald ratio	1	-0.064	0.107	5.499E-01	0.995	pan	SARS-CoV-2 infection
5456_59	CNDP1	Beta-Ala-His dipeptidase	Q96KN2	CNDP1	NA	Inverse variance weighted	12	-0.007	0.011	5.502E-01	0.995	pan	SARS-CoV-2 infection
19615_213	5NT3	Cytosolic 5'-nucleotidase 3A	Q9H0P0	NT5C3A	NA	Wald ratio	1	0.042	0.070	5.504E-01	0.995	pan	SARS-CoV-2 infection
2879_9	a1-Antichymotrypsin	Alpha-1-antichymotrypsin	P01011	SERPINA3	NA	Wald ratio	1	0.065	0.109	5.504E-01	0.995	pan	SARS-CoV-2 infection
9251_28	MA2B2	Epididymis-specific alpha-mannosidase	Q9Y2E5	MAN2B2	NA	Inverse variance weighted	10	0.006	0.010	5.510E-01	0.995	pan	SARS-CoV-2 infection
6467_65	KTEL1	Protein O-glucosyltransferase 1	Q8NBL1	POGLUT1	NA	Inverse variance weighted	2	0.013	0.022	5.511E-01	0.995	pan	SARS-CoV-2 infection
5509_7	EGF:ECD	Epidermal growth factor:Extracellular domain	P01133	EGF	NA	Inverse variance weighted	4	-0.029	0.049	5.515E-01	0.995	pan	SARS-CoV-2 infection
14614_41	CA130	Noncompact myelin-associated protein	Q5T1S8	NCMAP	NA	Inverse variance weighted	13	-0.011	0.019	5.523E-01	0.995	pan	SARS-CoV-2 infection
6557_50	LRC15	Leucine-rich repeat-containing protein 15	Q8TF66	LRRC15	NA	Inverse variance weighted	4	-0.038	0.063	5.523E-01	0.995	pan	SARS-CoV-2 infection
7923_41	SEM4C	Semaphorin-4C	Q9C0C4	SEMA4C	NA	Inverse variance weighted	10	0.019	0.032	5.528E-01	0.995	pan	SARS-CoV-2 infection
4930_21	Stanniocalcin-1	Stanniocalcin-1	P52823	STC1	NA	Inverse variance weighted	3	-0.027	0.045	5.529E-01	0.995	pan	SARS-CoV-2 infection
13624_17	NADK	NAD kinase	O95544	NADK	NA	Inverse variance weighted	2	-0.035	0.060	5.533E-01	0.995	pan	SARS-CoV-2 infection
3046_31	resistin	Resistin	Q9HD89	RETN	NA	Inverse variance weighted	16	0.009	0.015	5.537E-01	0.995	pan	SARS-CoV-2 infection
8235_48	SCG1	Secretogranin-1	P05060	CHGB	NA	Inverse variance weighted	7	0.018	0.030	5.539E-01	0.995	pan	SARS-CoV-2 infection
8458_111	a-Synuclein	Alpha-synuclein	P37840	SNCA	NA	Wald ratio	1	-0.061	0.103	5.540E-01	0.995	pan	SARS-CoV-2 infection
18935_14	TLR5	Toll-like receptor 5	O60602	TLR5	NA	Inverse variance weighted	11	-0.010	0.018	5.543E-01	0.995	pan	SARS-CoV-2 infection
11178_21	SVEP1:EGF-like domains 4-6	Sushi, von Willebrand factor type A, EGF and pentraxin domain-containing protein 1:EGF-like domains 4-6	Q4LDE5	SVEP1	NA	Inverse variance weighted	5	0.029	0.050	5.549E-01	0.995	pan	SARS-CoV-2 infection
16060_99	NID2	Nidogen-2	Q14112	NID2	NA	Inverse variance weighted	9	0.007	0.012	5.558E-01	0.995	pan	SARS-CoV-2 infection
18319_7	ODPX	Pyruvate dehydrogenase protein X component, mitochondrial	O00330	PDHX	NA	Wald ratio	1	0.037	0.063	5.558E-01	0.995	pan	SARS-CoV-2 infection
3168_8	ADAMTS-5	A disintegrin and metalloproteinase with thrombospondin motifs 5	Q9UNA0	ADAMTS5	NA	Inverse variance weighted	4	-0.008	0.014	5.565E-01	0.995	pan	SARS-CoV-2 infection
11616_9	HSF1	Heat shock factor protein 1	Q00613	HSF1	NA	Wald ratio	1	0.045	0.076	5.571E-01	0.995	pan	SARS-CoV-2 infection
3049_61	Trypsin	Trypsin-1	P07477	PRSS1	NA	Inverse variance weighted	4	0.032	0.054	5.576E-01	0.995	pan	SARS-CoV-2 infection
8973_23	FCRL4:ECD	Fc receptor-like protein 4:Extracellular domain	Q96PJ5	FCRL4	NA	Inverse variance weighted	20	-0.008	0.013	5.585E-01	0.995	pan	SARS-CoV-2 infection
15325_14	NEUM	Neuromodulin	P17677	GAP43	NA	Wald ratio	1	0.040	0.069	5.587E-01	0.995	pan	SARS-CoV-2 infection
12008_3	CD7	T-cell antigen CD7	P09564	CD7	NA	Inverse variance weighted	4	0.030	0.051	5.591E-01	0.995	pan	SARS-CoV-2 infection
17794_6	Phosphomannomutase 2	Phosphomannomutase 2	O15305	PMM2	NA	Inverse variance weighted	5	0.015	0.026	5.594E-01	0.995	pan	SARS-CoV-2 infection
19377_14	NOE2	Noelin-2	O95897	OLFM2	NA	Inverse variance weighted	8	0.006	0.010	5.607E-01	0.995	pan	SARS-CoV-2 infection
16322_10	PACAP	Marginal zone B- and B1-cell-specific protein	Q8WU39	MZB1	NA	Inverse variance weighted	10	0.019	0.033	5.608E-01	0.995	pan	SARS-CoV-2 infection
12676_1	PACN1	Protein kinase C and casein kinase substrate in neurons protein 1	Q9BY11	PACSIN1	NA	Inverse variance weighted	2	0.021	0.036	5.611E-01	0.995	pan	SARS-CoV-2 infection
3045_72	PTN	Pleiotrophin	P21246	PTN	NA	Inverse variance weighted	4	-0.013	0.022	5.629E-01	0.995	pan	SARS-CoV-2 infection
7953_20	SLAF1	Signaling lymphocytic activation molecule	Q13291	SLAMF1	NA	Wald ratio	1	0.049	0.085	5.634E-01	0.995	pan	SARS-CoV-2 infection
12486_8	GLRX2	Glutaredoxin-2, mitochondrial	Q9NS18	GLRX2	NA	Inverse variance weighted	3	-0.013	0.022	5.634E-01	0.995	pan	SARS-CoV-2 infection
2212_69	tPA	Tissue-type plasminogen activator	P00750	PLAT	NA	Inverse variance weighted	9	-0.016	0.028	5.639E-01	0.995	pan	SARS-CoV-2 infection
13107_9	LYPD3	Ly6/PLAUR domain-containing protein 3	O95274	LYPD3	NA	Inverse variance weighted	3	-0.048	0.083	5.648E-01	0.995	pan	SARS-CoV-2 infection
13622_16	2A5A	Serine/threonine-protein phosphatase 2A 56 kDa regulatory subunit alpha isoform	Q15172	PPP2R5A	NA	Wald ratio	1	0.058	0.100	5.651E-01	0.995	pan	SARS-CoV-2 infection
13435_31	IL-20 Rb	Interleukin-20 receptor subunit beta	Q6UXL0	IL20RB	NA	Wald ratio	1	0.056	0.097	5.654E-01	0.995	pan	SARS-CoV-2 infection
16914_104	sCD14	Monocyte differentiation antigen CD14, soluble	P08571	CD14	NA	Inverse variance weighted	5	-0.022	0.039	5.664E-01	0.995	pan	SARS-CoV-2 infection
5355_69	LIGHT	Tumor necrosis factor ligand superfamily member 14	O43557	TNFSF14	NA	Wald ratio	1	-0.028	0.048	5.664E-01	0.995	pan	SARS-CoV-2 infection
18183_3	ARH	Low density lipoprotein receptor adapter protein 1	Q5SW96	LDLRAP1	NA	Wald ratio	1	-0.034	0.059	5.668E-01	0.995	pan	SARS-CoV-2 infection
4801_13	PERL	Lactoperoxidase	P22079	LPO	NA	Inverse variance weighted	9	0.016	0.028	5.686E-01	0.995	pan	SARS-CoV-2 infection
13578_98	ABLM3	Actin-binding LIM protein 3	O94929	ABLIM3	NA	Wald ratio	1	-0.027	0.047	5.691E-01	0.995	pan	SARS-CoV-2 infection
12975_11	Keratin 20	Keratin, type I cytoskeletal 20	P35900	KRT20	NA	Inverse variance weighted	3	-0.033	0.057	5.695E-01	0.995	pan	SARS-CoV-2 infection
2827_23	Fractalkine/CX3CL-1	Fractalkine	P78423	CX3CL1	NA	Wald ratio	1	-0.019	0.033	5.702E-01	0.995	pan	SARS-CoV-2 infection
8942_2	RM21	39S ribosomal protein L21, mitochondrial	Q7Z2W9	MRPL21	NA	Wald ratio	1	-0.069	0.121	5.702E-01	0.995	pan	SARS-CoV-2 infection
5630_48	CM35H	CMRF35-like molecule 8	Q9UGN4	CD300A	NA	Inverse variance weighted	13	-0.007	0.012	5.708E-01	0.995	pan	SARS-CoV-2 infection
2474_54	SAP	Serum amyloid P-component	P02743	APCS	NA	Inverse variance weighted	7	0.012	0.021	5.712E-01	0.995	pan	SARS-CoV-2 infection
3431_54	EphA1	Ephrin type-A receptor 1	P21709	EPHA1	NA	Inverse variance weighted	10	0.005	0.008	5.719E-01	0.995	pan	SARS-CoV-2 infection
10781_19	CLC4G	C-type lectin domain family 4 member G	Q6UXB4	CLEC4G	NA	Wald ratio	1	-0.051	0.091	5.720E-01	0.995	pan	SARS-CoV-2 infection
15375_49	Carboxypeptidase B1	Carboxypeptidase B	P15086	CPB1	NA	Inverse variance weighted	9	-0.014	0.025	5.726E-01	0.995	pan	SARS-CoV-2 infection
9038_12	JTB	Protein JTB	O76095	JTB	NA	Wald ratio	1	-0.047	0.084	5.727E-01	0.995	pan	SARS-CoV-2 infection
10924_258	NEUFC	Neuferricin	Q8WUJ1	CYB5D2	NA	Inverse variance weighted	3	-0.013	0.022	5.730E-01	0.995	pan	SARS-CoV-2 infection
7141_21	MGT4B	Alpha-1,3-mannosyl-glycoprotein 4-beta-N-acetylglucosaminyltransferase B	Q9UQ53	MGAT4B	NA	Inverse variance weighted	4	-0.013	0.022	5.735E-01	0.995	pan	SARS-CoV-2 infection
12408_333	RB22A	Ras-related protein Rab-22A	Q9UL26	RAB22A	NA	Inverse variance weighted	2	0.037	0.066	5.737E-01	0.995	pan	SARS-CoV-2 infection
6049_64	PTPRS	Receptor-type tyrosine-protein phosphatase S	Q13332	PTPRS	NA	Inverse variance weighted	4	-0.021	0.038	5.749E-01	0.995	pan	SARS-CoV-2 infection
11126_102	TRIO	Triple functional domain protein	O75962	TRIO	NA	Wald ratio	1	-0.059	0.106	5.756E-01	0.995	pan	SARS-CoV-2 infection
14655_1	DJC17	DnaJ homolog subfamily C member 17	Q9NVM6	DNAJC17	NA	Inverse variance weighted	4	-0.024	0.043	5.757E-01	0.995	pan	SARS-CoV-2 infection
18897_31	HDAC2	Histone deacetylase 2	Q92769	HDAC2	NA	Wald ratio	1	-0.076	0.135	5.758E-01	0.995	pan	SARS-CoV-2 infection
11351_233	NHEJ1	Non-homologous end-joining factor 1	Q9H9Q4	NHEJ1	NA	Inverse variance weighted	3	-0.031	0.055	5.760E-01	0.995	pan	SARS-CoV-2 infection
13124_20	ISLR2	Immunoglobulin superfamily containing leucine-rich repeat protein 2	Q6UXK2	ISLR2	NA	Inverse variance weighted	4	-0.025	0.045	5.761E-01	0.995	pan	SARS-CoV-2 infection
7009_8	CD72	B-cell differentiation antigen CD72	P21854	CD72	NA	Inverse variance weighted	3	0.051	0.090	5.767E-01	0.995	pan	SARS-CoV-2 infection
6366_38	TXD15	Thioredoxin domain-containing protein 15	Q96J42	TXNDC15	NA	Inverse variance weighted	12	0.007	0.012	5.768E-01	0.995	pan	SARS-CoV-2 infection
15514_26	Pseudocholinesterase	Cholinesterase	P06276	BCHE	NA	Inverse variance weighted	17	-0.012	0.021	5.774E-01	0.995	pan	SARS-CoV-2 infection
6453_70	LIRB4	Leukocyte immunoglobulin-like receptor subfamily B member 4	Q8NHJ6	LILRB4	NA	Inverse variance weighted	2	-0.034	0.062	5.774E-01	0.995	pan	SARS-CoV-2 infection
9513_9	FGF22	Fibroblast growth factor 22	Q9HCT0	FGF22	NA	Wald ratio	1	-0.066	0.119	5.775E-01	0.995	pan	SARS-CoV-2 infection
5656_53	PP11	Poly(U)-specific endoribonuclease	P21128	ENDOU	NA	Wald ratio	1	-0.045	0.080	5.780E-01	0.995	pan	SARS-CoV-2 infection
9416_77	CBPM	Carboxypeptidase M	P14384	CPM	NA	Inverse variance weighted	5	-0.027	0.048	5.786E-01	0.995	pan	SARS-CoV-2 infection
8099_42	SPON2	Spondin-2	Q9BUD6	SPON2	NA	Inverse variance weighted	5	-0.011	0.020	5.786E-01	0.995	pan	SARS-CoV-2 infection
10666_7	GNPTG	N-acetylglucosamine-1-phosphotransferase subunit gamma	Q9UJJ9	GNPTG	NA	Inverse variance weighted	4	0.017	0.031	5.791E-01	0.995	pan	SARS-CoV-2 infection
12671_35	ST1B1	Sulfotransferase family cytosolic 1B member 1	O43704	SULT1B1	NA	Inverse variance weighted	6	0.020	0.035	5.798E-01	0.995	pan	SARS-CoV-2 infection
19187_21	STABP	STAM-binding protein	O95630	STAMBP	NA	Inverse variance weighted	2	0.039	0.071	5.799E-01	0.995	pan	SARS-CoV-2 infection
9282_12	CRIS2	Cysteine-rich secretory protein 2	P16562	CRISP2	NA	Inverse variance weighted	33	0.004	0.007	5.800E-01	0.995	pan	SARS-CoV-2 infection
8244_16	FUT8	Alpha-(1,6)-fucosyltransferase	Q9BYC5	FUT8	NA	Inverse variance weighted	19	0.010	0.019	5.803E-01	0.995	pan	SARS-CoV-2 infection
8429_16	AXIN2	Axin-2	Q9Y2T1	AXIN2	NA	Inverse variance weighted	4	0.028	0.050	5.810E-01	0.995	pan	SARS-CoV-2 infection
12812_25	ACYP2	Acylphosphatase-2	P14621	ACYP2	NA	Inverse variance weighted	2	0.012	0.021	5.811E-01	0.995	pan	SARS-CoV-2 infection
8326_63	SIGIRR	Single Ig IL-1-related receptor	Q6IA17	SIGIRR	NA	Inverse variance weighted	15	0.012	0.022	5.812E-01	0.995	pan	SARS-CoV-2 infection
3313_21	FCN2	Ficolin-2	Q15485	FCN2	NA	Inverse variance weighted	4	0.024	0.043	5.824E-01	0.995	pan	SARS-CoV-2 infection
5095_21	KI2L4	Killer cell immunoglobulin-like receptor 2DL4	Q99706	KIR2DL4	NA	Inverse variance weighted	2	-0.019	0.035	5.831E-01	0.995	pan	SARS-CoV-2 infection

10565_19	SLIK3	SLIT and NTRK-like protein 3	O94933	SLITRK3	NA	Inverse variance weighted	16	-0.007	0.012	5.833E-01	0.995	pan	SARS-CoV-2 infection
12560_9	NT5C	5'(3')-deoxyribonucleotidase, cytosolic type	Q8TCD5	NT5C	NA	Inverse variance weighted	5	-0.009	0.017	5.833E-01	0.995	pan	SARS-CoV-2 infection
9964_10	MYZAP	Myocardial zonula adherens protein	P0CAP1	MYZAP	NA	Inverse variance weighted	2	0.033	0.060	5.835E-01	0.995	pan	SARS-CoV-2 infection
10702_1	COSA1	Collagen alpha-1(XXVIII) chain	Q2UY09	COL28A1	NA	Inverse variance weighted	2	0.078	0.142	5.836E-01	0.995	pan	SARS-CoV-2 infection
8697_38	Glypican 1	Glypican-1	P35052	GPC1	NA	Inverse variance weighted	16	0.006	0.011	5.840E-01	0.995	pan	SARS-CoV-2 infection
9026_40	BTNL8	Butyrophilin-like protein 8	Q6UX41	BTNL8	NA	Inverse variance weighted	2	0.016	0.029	5.841E-01	0.995	pan	SARS-CoV-2 infection
13113_7	Osteopontin	Osteopontin	P10451	SPP1	NA	Inverse variance weighted	5	0.014	0.026	5.848E-01	0.995	pan	SARS-CoV-2 infection
17722_5	FKBP52 protein	Peptidyl-prolyl cis-trans isomerase FKBP4	Q02790	FKBP4	NA	Inverse variance weighted	2	0.035	0.064	5.852E-01	0.995	pan	SARS-CoV-2 infection
7776_20	UNC5B	Netrin receptor UNC5B	Q8IZJ1	UNC5B	NA	Inverse variance weighted	3	-0.029	0.054	5.861E-01	0.995	pan	SARS-CoV-2 infection
4297_62	Spondin-1	Spondin-1	Q9HCB6	SPON1	NA	Inverse variance weighted	4	0.009	0.016	5.872E-01	0.995	pan	SARS-CoV-2 infection
13524_25	H6ST2	Heparan-sulfate 6-O-sulfotransferase 2	Q96MM7	HS6ST2	NA	Wald ratio	1	-0.049	0.090	5.872E-01	0.995	pan	SARS-CoV-2 infection
15566_10	Calponin-1	Calponin-1	P51911	CNN1	NA	Inverse variance weighted	10	0.012	0.022	5.902E-01	0.995	pan	SARS-CoV-2 infection
4294_16	Sphingosine kinase 1	Sphingosine kinase 1	Q9NYA1	SPHK1	NA	Inverse variance weighted	4	0.015	0.028	5.903E-01	0.995	pan	SARS-CoV-2 infection
6385_63	VWA1	von Willebrand factor A domain-containing protein 1	Q6PCB0	VWA1	NA	Inverse variance weighted	6	0.008	0.014	5.904E-01	0.995	pan	SARS-CoV-2 infection
14048_7	IL-1 R AcP	Interleukin-1 Receptor accessory protein	Q9NPH3	IL1RAP	NA	Inverse variance weighted	6	-0.004	0.007	5.918E-01	0.995	pan	SARS-CoV-2 infection
2864_2	MEK1	Dual specificity mitogen-activated protein kinase kinase 1	Q02750	MAP2K1	NA	Wald ratio	1	0.041	0.077	5.922E-01	0.995	pan	SARS-CoV-2 infection
7810_20	C1QTNF5	Complement C1q tumor necrosis factor-related protein 5	Q9BXJ0	C1QTNF5	NA	Inverse variance weighted	10	0.013	0.024	5.923E-01	0.995	pan	SARS-CoV-2 infection
3342_76	ABL2	Abelson tyrosine-protein kinase 2	P42684	ABL2	NA	Wald ratio	1	0.054	0.101	5.927E-01	0.995	pan	SARS-CoV-2 infection
8894_80	hnRNP A/B	Heterogeneous nuclear ribonucleoprotein A/B	Q99729	HNRNPAB	NA	Inverse variance weighted	2	0.054	0.100	5.933E-01	0.995	pan	SARS-CoV-2 infection
19557_3	KLOTB	Beta-klotho	Q86Z14	KLB	NA	Inverse variance weighted	11	-0.006	0.011	5.936E-01	0.995	pan	SARS-CoV-2 infection
9216_100	PLXB2	Plexin-B2	O15031	PLXNB2	NA	Inverse variance weighted	9	-0.008	0.014	5.936E-01	0.995	pan	SARS-CoV-2 infection
19273_3	Glutathione reductase	Glutathione reductase, mitochondrial	P00390	GSR	NA	Inverse variance weighted	3	-0.017	0.031	5.941E-01	0.995	pan	SARS-CoV-2 infection
13094_75	RSPO3	R-spondin-3	Q9BXY4	RSPO3	NA	Inverse variance weighted	2	-0.036	0.068	5.942E-01	0.995	pan	SARS-CoV-2 infection
9748_31	GSTM3-3	Glutathione S-transferase Mu 3	P21266	GSTM3	NA	Inverse variance weighted	7	-0.008	0.016	5.943E-01	0.995	pan	SARS-CoV-2 infection
5256_86	PDE5A	cGMP-specific 3',5'-cyclic phosphodiesterase	O76074	PDE5A	NA	Inverse variance weighted	5	-0.022	0.042	5.948E-01	0.995	pan	SARS-CoV-2 infection
18216_22	IL-11 RA	Interleukin-11 receptor subunit alpha	Q14626	IL11RA	NA	Inverse variance weighted	7	-0.012	0.022	5.953E-01	0.995	pan	SARS-CoV-2 infection
5713_9	IFN-lambda 3	Interferon lambda-3	Q8IZI9	IFNL3	NA	Inverse variance weighted	2	-0.053	0.099	5.954E-01	0.995	pan	SARS-CoV-2 infection
5091_28	ILT-4	Leukocyte immunoglobulin-like receptor subfamily B member 2	Q8N423	LILRB2	NA	Inverse variance weighted	4	-0.020	0.038	5.956E-01	0.995	pan	SARS-CoV-2 infection
2771_35	IGFBP-1	Insulin-like growth factor-binding protein 1	P08833	IGFBP1	NA	Inverse variance weighted	2	-0.029	0.054	5.956E-01	0.995	pan	SARS-CoV-2 infection
9204_33	Corticotropin-lipotropin	Pro-opiomelanocortin	P01189	POMC	NA	Inverse variance weighted	11	-0.014	0.026	5.958E-01	0.995	pan	SARS-CoV-2 infection
11531_24	GI24:CD	V-type immunoglobulin domain-containing suppressor of T-cell activation:Cytoplasmic domain	Q9H7M9	VSIR	NA	Wald ratio	1	-0.043	0.080	5.959E-01	0.995	pan	SARS-CoV-2 infection
5701_81	Tetranectin	Tetranectin	P05452	CLEC3B	NA	Inverse variance weighted	3	0.018	0.034	5.970E-01	0.995	pan	SARS-CoV-2 infection
17750_8	RIR2	Ribonucleoside-diphosphate reductase subunit M2	P31350	RRM2	NA	Inverse variance weighted	3	0.003	0.006	5.977E-01	0.995	pan	SARS-CoV-2 infection
3795_6	ADAM 9	Disintegrin and metalloproteinase domain-containing protein 9	Q13443	ADAM9	NA	Wald ratio	1	0.066	0.126	5.993E-01	0.995	pan	SARS-CoV-2 infection
5404_53	DR6	Tumor necrosis factor receptor superfamily member 21	O75509	TNFRSF21	NA	Inverse variance weighted	2	-0.023	0.043	5.993E-01	0.995	pan	SARS-CoV-2 infection
4496_60	MMP-12	Macrophage metalloelastase	P39900	MMP12	NA	Inverse variance weighted	13	0.005	0.010	5.998E-01	0.995	pan	SARS-CoV-2 infection
15300_66	CC134	Coiled-coil domain-containing protein 134	Q9H6E4	CCDC134	NA	Inverse variance weighted	2	0.018	0.035	6.000E-01	0.995	pan	SARS-CoV-2 infection
5644_60	RNAS4	Ribonuclease 4	P34096	RNASE4	NA	Inverse variance weighted	5	-0.010	0.019	6.007E-01	0.995	pan	SARS-CoV-2 infection
2741_22	Siglec-6	Sialic acid-binding Ig-like lectin 6	O43699	SIGLEC6	NA	Inverse variance weighted	7	-0.008	0.015	6.013E-01	0.995	pan	SARS-CoV-2 infection
3864_5	RS7	40S ribosomal protein S7	P62081	RPS7	NA	Wald ratio	1	-0.052	0.099	6.022E-01	0.995	pan	SARS-CoV-2 infection
7886_26	SPTC1	Serine palmitoyltransferase 1	O15269	SPTLC1	NA	Inverse variance weighted	2	-0.030	0.057	6.028E-01	0.995	pan	SARS-CoV-2 infection
6496_60	DLK1:ECD	Protein delta homolog 1:Extracellular domain	P80370	DLK1	NA	Inverse variance weighted	2	0.035	0.066	6.033E-01	0.995	pan	SARS-CoV-2 infection
13450_49	UBP8	Ubiquitin carboxyl-terminal hydrolase 8	P40818	USP8	NA	Wald ratio	1	-0.033	0.064	6.033E-01	0.995	pan	SARS-CoV-2 infection
8877_22	F176C:ECD	Protein eva-1 homolog C:Extracellular domain	P58658	EVA1C	NA	Wald ratio	1	-0.040	0.077	6.034E-01	0.995	pan	SARS-CoV-2 infection
15329_167	MAPIP	Ragulator complex protein LAMTOR2	Q9Y2Q5	LAMTOR2	NA	Wald ratio	1	0.057	0.110	6.051E-01	0.995	pan	SARS-CoV-2 infection
3034_1	GFAP	Glial fibrillary acidic protein	P14136	GFAP	NA	Wald ratio	1	0.037	0.071	6.052E-01	0.995	pan	SARS-CoV-2 infection
6255_74	CPXM1	Probable carboxypeptidase X1	Q96SM3	CPXM1	NA	Inverse variance weighted	6	-0.008	0.016	6.059E-01	0.995	pan	SARS-CoV-2 infection
19233_75	ATOX1	Copper transport protein ATOX1	O00244	ATOX1	NA	Wald ratio	1	-0.047	0.091	6.059E-01	0.995	pan	SARS-CoV-2 infection
3798_71	Carbonic anhydrase 9	Carbonic anhydrase 9	Q16790	CA9	NA	Wald ratio	1	0.094	0.183	6.065E-01	0.995	pan	SARS-CoV-2 infection
19193_18	PFD3	Prefoldin subunit 3	P61758	VBP1	NA	Inverse variance weighted	12	-0.013	0.026	6.070E-01	0.995	pan	SARS-CoV-2 infection
3853_56	MDHC	Malate dehydrogenase, cytoplasmic	P40925	MDH1	NA	Wald ratio	1	-0.038	0.074	6.073E-01	0.995	pan	SARS-CoV-2 infection
7115_5	Semenogelin	Semenogelin-1	P04279	SEMG1	NA	Inverse variance weighted	16	0.010	0.019	6.074E-01	0.995	pan	SARS-CoV-2 infection
4459_68	PCSK7	Proprotein convertase subtilisin/kexin type 7	Q16549	PCSK7	NA	Inverse variance weighted	14	-0.005	0.010	6.076E-01	0.995	pan	SARS-CoV-2 infection
10800_15	Collagen-binding protein	Serpin H1	P50454	SERPINH1	NA	Inverse variance weighted	4	0.016	0.031	6.080E-01	0.995	pan	SARS-CoV-2 infection
7211_2	RNase 1	Ribonuclease pancreatic	P07998	RNASE1	NA	Inverse variance weighted	6	-0.022	0.043	6.081E-01	0.995	pan	SARS-CoV-2 infection
3336_50	TFPI	Tissue factor pathway inhibitor	P10646	TFPI	NA	Wald ratio	1	-0.032	0.062	6.087E-01	0.995	pan	SARS-CoV-2 infection
5596_75	CD5:ECD	T-cell surface glycoprotein CD5:Extracellular domain	P06127	CD5	NA	Inverse variance weighted	17	0.017	0.034	6.098E-01	0.995	pan	SARS-CoV-2 infection
3004_67	PD-L2	Programmed cell death 1 ligand 2	Q9BQ51	PDCD1LG2	NA	Inverse variance weighted	6	-0.009	0.017	6.100E-01	0.995	pan	SARS-CoV-2 infection
13632_10	Zyxin	Zyxin	Q15942	ZYX	NA	Wald ratio	1	-0.051	0.099	6.101E-01	0.995	pan	SARS-CoV-2 infection
8403_18	Fatty acid synthase	Fatty acid synthase	P49327	FASN	NA	Wald ratio	1	-0.017	0.034	6.102E-01	0.995	pan	SARS-CoV-2 infection
15633_6	RBP	Retinol-binding protein 4	P02753	RBP4	NA	Wald ratio	1	0.031	0.060	6.104E-01	0.995	pan	SARS-CoV-2 infection
15471_29	LIPR2	Pancreatic lipase-related protein 2	P54317	PNLIPRP2	NA	Inverse variance weighted	14	0.004	0.008	6.115E-01	0.995	pan	SARS-CoV-2 infection
12800_5	TPPP2	Tubulin polymerization-promoting protein family member 2	P59282	TPPP2	NA	Inverse variance weighted	4	-0.016	0.032	6.115E-01	0.995	pan	SARS-CoV-2 infection
4337_49	CRP	C-reactive protein	P02741	CRP	NA	Inverse variance weighted	7	0.020	0.040	6.121E-01	0.995	pan	SARS-CoV-2 infection
8250_2	PTPRJ	Receptor-type tyrosine-protein phosphatase eta	Q12913	PTPRJ	NA	Inverse variance weighted	8	0.015	0.030	6.122E-01	0.995	pan	SARS-CoV-2 infection
7856_51	F151A	Protein FAM151A	Q8WW52	FAM151A	NA	Inverse variance weighted	11	-0.010	0.020	6.127E-01	0.995	pan	SARS-CoV-2 infection
17153_46	KI2L3	Killer cell immunoglobulin-like receptor 2DL3	P43628	KIR2DL3	NA	Inverse variance weighted	10	-0.012	0.024	6.127E-01	0.995	pan	SARS-CoV-2 infection
12016_60	CBL	E3 ubiquitin-protein ligase CBL	P22681	CBL	NA	Wald ratio	1	0.018	0.036	6.130E-01	0.995	pan	SARS-CoV-2 infection
11424_4	FAAA	Fumarylacetoacetase	P16930	FAH	NA	Inverse variance weighted	8	0.007	0.015	6.131E-01	0.995	pan	SARS-CoV-2 infection
13671_40	Elastase	Neutrophil elastase	P08246	ELANE	NA	Inverse variance weighted	12	0.011	0.021	6.133E-01	0.995	pan	SARS-CoV-2 infection
12333_87	RPIA	Ribose-5-phosphate isomerase	P49247	RPIA	NA	Inverse variance weighted	2	-0.023	0.046	6.135E-01	0.995	pan	SARS-CoV-2 infection
17515_6	STCH	Heat shock 70 kDa protein 13	P48723	HSPA13	NA	Inverse variance weighted	2	0.023	0.045	6.140E-01	0.995	pan	SARS-CoV-2 infection
5648_28	CTRB2	Chymotrypsinogen B2	Q6GPI1	CTRB2	NA	Inverse variance weighted	12	0.004	0.009	6.144E-01	0.995	pan	SARS-CoV-2 infection
5837_49	LIF sR	Leukemia inhibitory factor receptor	P42702	LIFR	NA	Inverse variance weighted	9	-0.012	0.024	6.145E-01	0.995	pan	SARS-CoV-2 infection
3311_27	FCG3B	Low affinity immunoglobulin gamma Fc region receptor III-B	O75015	FCGR3B	NA	Inverse variance weighted	8	-0.015	0.030	6.145E-01	0.995	pan	SARS-CoV-2 infection
6576_1	ART4	Ecto-ADP-ribosyltransferase 4	Q93070	ART4	NA	Inverse variance weighted	10	-0.004	0.009	6.147E-01	0.995	pan	SARS-CoV-2 infection
13101_60	SOST	Sclerostin	Q9BQB4	SOST	NA	Inverse variance weighted	3	-0.022	0.045	6.150E-01	0.995	pan	SARS-CoV-2 infection
17671_58	ING4	Inhibitor of growth protein 4	Q9UNL4	ING4	NA	Inverse variance weighted	4	-0.022	0.043	6.150E-01	0.995	pan	SARS-CoV-2 infection

19124_9	UBCP1	Ubiquitin-like domain-containing CTD phosphatase 1	Q8WVY7	UBLCP1	NA	Inverse variance weighted	5	-0.018	0.035	6.154E-01	0.995	pan	SARS-CoV-2 infection
15446_25	SMDF	Neuregulin-1, sensory and motor neuron-derived factor isoform	Q02297	NRG1	NA	Inverse variance weighted	2	-0.022	0.043	6.156E-01	0.995	pan	SARS-CoV-2 infection
5452_71	ASGR1	Asialoglycoprotein receptor 1	P07306	ASGR1	NA	Wald ratio	1	0.042	0.083	6.158E-01	0.995	pan	SARS-CoV-2 infection
13112_179	FSTL1	Follistatin-related protein 1	Q12841	FSTL1	NA	Inverse variance weighted	4	0.013	0.025	6.162E-01	0.995	pan	SARS-CoV-2 infection
6611_8	KDEL1	KDEL motif-containing protein 1	Q6UW63	POGLUT2	NA	Wald ratio	1	-0.053	0.106	6.166E-01	0.995	pan	SARS-CoV-2 infection
12554_10	RA51D	DNA repair protein RAD51 homolog 4	O75771	RAD51D	NA	Inverse variance weighted	3	-0.049	0.098	6.169E-01	0.995	pan	SARS-CoV-2 infection
6997_32	RAB26	Ras-related protein Rab-26	Q9ULW5	RAB26	NA	Inverse variance weighted	3	-0.032	0.063	6.172E-01	0.995	pan	SARS-CoV-2 infection
16620_26	LY75	Lymphocyte antigen 75	O60449	LY75	NA	Inverse variance weighted	19	-0.005	0.010	6.176E-01	0.995	pan	SARS-CoV-2 infection
9796_4	CEL	Bile salt-activated lipase	P19835	CEL	NA	Inverse variance weighted	8	-0.008	0.016	6.182E-01	0.995	pan	SARS-CoV-2 infection
17495_141	SIR3	NAD-dependent protein deacetylase sirtuin-3, mitochondrial	Q9NTG7	SIRT3	NA	Inverse variance weighted	3	-0.021	0.042	6.198E-01	0.995	pan	SARS-CoV-2 infection
3448_13	IR	Insulin receptor	P06213	INSR	NA	Inverse variance weighted	15	0.012	0.024	6.204E-01	0.995	pan	SARS-CoV-2 infection
3324_51	LY9	T-lymphocyte surface antigen Ly-9	Q9HBG7	LY9	NA	Inverse variance weighted	6	-0.013	0.026	6.208E-01	0.995	pan	SARS-CoV-2 infection
8459_10	BMP-6	Bone morphogenetic protein 6	P22004	BMP6	NA	Inverse variance weighted	3	0.022	0.044	6.210E-01	0.995	pan	SARS-CoV-2 infection
13717_15	FCN2	Ficolin-2	Q15485	FCN2	NA	Inverse variance weighted	11	0.009	0.019	6.214E-01	0.995	pan	SARS-CoV-2 infection
5121_3	SEM6B	Semaphorin-6B	Q9H3T3	SEMA6B	NA	Inverse variance weighted	2	0.042	0.085	6.215E-01	0.995	pan	SARS-CoV-2 infection
6390_18	NPS	Neuropeptide S	P0C0P6	NPS	NA	Inverse variance weighted	2	0.060	0.121	6.218E-01	0.995	pan	SARS-CoV-2 infection
15607_56	KPYR	Pyruvate kinase PKLR	P30613	PKLR	NA	Inverse variance weighted	3	-0.036	0.074	6.222E-01	0.995	pan	SARS-CoV-2 infection
3535_84	DKK1	Dickkopf-related protein 1	O94907	DKK1	NA	Wald ratio	1	-0.051	0.104	6.228E-01	0.995	pan	SARS-CoV-2 infection
3291_30	CD23	Low affinity immunoglobulin epsilon Fc receptor	P06734	FCER2	NA	Inverse variance weighted	11	0.007	0.014	6.241E-01	0.995	pan	SARS-CoV-2 infection
3206_4	LYVE1	Lymphatic vessel endothelial hyaluronic acid receptor 1	Q9Y5Y7	LYVE1	NA	Inverse variance weighted	21	-0.007	0.015	6.246E-01	0.995	pan	SARS-CoV-2 infection
15431_31	OTCase	Ornithine carbamoyltransferase, mitochondrial	P00480	OTC	NA	Inverse variance weighted	3	-0.035	0.072	6.246E-01	0.995	pan	SARS-CoV-2 infection
2900_53	HCC-1	C-C motif chemokine 14	Q16627	CCL14	NA	Inverse variance weighted	7	-0.011	0.023	6.256E-01	0.995	pan	SARS-CoV-2 infection
8974_172	COFA1	Collagen alpha-1(XV) chain	P39059	COL15A1	NA	Inverse variance weighted	7	0.009	0.019	6.257E-01	0.995	pan	SARS-CoV-2 infection
9838_4	SMAD1	Mothers against decapentaplegic homolog 1	Q15797	SMAD1	NA	Wald ratio	1	-0.047	0.096	6.264E-01	0.995	pan	SARS-CoV-2 infection
3474_19	Thrombospondin-1	Thrombospondin-1	P07996	THBS1	NA	Wald ratio	1	0.048	0.098	6.266E-01	0.995	pan	SARS-CoV-2 infection
15495_9	FOLR3	Folate receptor gamma	P41439	FOLR3	NA	Inverse variance weighted	15	0.006	0.013	6.267E-01	0.995	pan	SARS-CoV-2 infection
10462_14	INSL5	Insulin-like peptide INSL5	Q9Y5Q6	INSL5	NA	Wald ratio	1	-0.040	0.083	6.273E-01	0.995	pan	SARS-CoV-2 infection
18408_26	ARF4	ADP-ribosylation factor 4	P18085	ARF4	NA	Wald ratio	1	-0.051	0.104	6.278E-01	0.995	pan	SARS-CoV-2 infection
2515_14	GFRa-2	GDNF family receptor alpha-2	O00451	GFRA2	NA	Inverse variance weighted	7	-0.007	0.014	6.279E-01	0.995	pan	SARS-CoV-2 infection
4978_54	DBNL	Drebrin-like protein	Q9UJU6	DBNL	NA	Wald ratio	1	0.040	0.083	6.288E-01	0.995	pan	SARS-CoV-2 infection
8859_51	CAH11	Carbonic anhydrase-related protein 11	O75493	CA11	NA	Inverse variance weighted	2	0.028	0.059	6.296E-01	0.995	pan	SARS-CoV-2 infection
2836_68	Lipocalin 2	Neutrophil gelatinase-associated lipocalin	P80188	LCN2	NA	Inverse variance weighted	7	-0.015	0.032	6.302E-01	0.995	pan	SARS-CoV-2 infection
12348_46	SYSM	Serine--tRNA ligase, mitochondrial	Q9NP81	SARS2	NA	Wald ratio	1	-0.022	0.046	6.310E-01	0.995	pan	SARS-CoV-2 infection
3376_49	IL-17 RD	Interleukin-17 receptor D	Q8NFM7	IL17RD	NA	Inverse variance weighted	6	-0.007	0.015	6.319E-01	0.995	pan	SARS-CoV-2 infection
19581_15	IGFBP-5	Insulin-like growth factor-binding protein 5	P24593	IGFBP5	NA	Wald ratio	1	-0.044	0.093	6.326E-01	0.995	pan	SARS-CoV-2 infection
18244_1	ANXA7	Annexin A7	P20073	ANXA7	NA	Wald ratio	1	0.056	0.117	6.330E-01	0.995	pan	SARS-CoV-2 infection
10088_37	APT	Adenine phosphoribosyltransferase	P07741	APRT	NA	Inverse variance weighted	7	-0.010	0.022	6.336E-01	0.995	pan	SARS-CoV-2 infection
11102_22	REG4	Regenerating islet-derived protein 4	Q9BYZ8	REG4	NA	Inverse variance weighted	2	0.017	0.035	6.337E-01	0.995	pan	SARS-CoV-2 infection
15462_28	CD8A	T-cell surface glycoprotein CD8 alpha chain	P01732	CD8A	NA	Inverse variance weighted	8	-0.008	0.017	6.355E-01	0.995	pan	SARS-CoV-2 infection
3171_57	amyloid precursor protein	Amyloid beta A4 protein	P05067	APP	NA	Inverse variance weighted	2	0.026	0.055	6.358E-01	0.995	pan	SARS-CoV-2 infection
6442_6	BAGE3	B melanoma antigen 3	Q86Y29	BAGE3	NA	Wald ratio	1	0.052	0.109	6.359E-01	0.995	pan	SARS-CoV-2 infection
9826_135	Fragile histidine triad protein	Bis(5'-adenosyl)-triphosphatase	P49789	FHIT	NA	Inverse variance weighted	3	-0.034	0.072	6.360E-01	0.995	pan	SARS-CoV-2 infection
5631_83	MOTI	Promotilin	P12872	MLN	NA	Inverse variance weighted	26	-0.005	0.011	6.366E-01	0.995	pan	SARS-CoV-2 infection
13986_6	LANC1	LanC-like protein 1	O43813	LANCL1	NA	Inverse variance weighted	2	-0.021	0.045	6.374E-01	0.995	pan	SARS-CoV-2 infection
17320_19	ADPPT	L-aminoadipate-semialdehyde dehydrogenase-phosphopantetheinyl transferase	Q9NRN7	AASDHPPT	NA	Wald ratio	1	0.051	0.109	6.393E-01	0.995	pan	SARS-CoV-2 infection
12630_8	ARFP2	Arfaptin-2	P53365	ARFIP2	NA	Inverse variance weighted	5	0.019	0.040	6.395E-01	0.995	pan	SARS-CoV-2 infection
14662_6	MZF1	Myeloid zinc finger 1	P28698	MZF1	NA	Wald ratio	1	0.030	0.064	6.403E-01	0.995	pan	SARS-CoV-2 infection
7986_98	NALD2	N-acetylated-alpha-linked acidic dipeptidase 2	Q9Y3Q0	NAALAD2	NA	Inverse variance weighted	10	0.007	0.015	6.409E-01	0.995	pan	SARS-CoV-2 infection
12713_365	RGS19	Regulator of G-protein signaling 19	P49795	RGS19	NA	Wald ratio	1	0.046	0.100	6.410E-01	0.995	pan	SARS-CoV-2 infection
4471_50	TGM3	Protein-glutamine gamma-glutamyltransferase E	Q08188	TGM3	NA	Inverse variance weighted	5	0.006	0.013	6.410E-01	0.995	pan	SARS-CoV-2 infection
12361_102	RRAS2	Ras-related protein R-Ras2	P62070	RRAS2	NA	Wald ratio	1	-0.018	0.039	6.419E-01	0.995	pan	SARS-CoV-2 infection
3827_22	PAK6	Serine/threonine-protein kinase PAK 6	Q9NQU5	PAK6	NA	Wald ratio	1	-0.014	0.030	6.430E-01	0.995	pan	SARS-CoV-2 infection
4435_66	ENPP7	Ectonucleotide pyrophosphatase/phosphodiesterase family member 7	Q6UWV6	ENPP7	NA	Inverse variance weighted	10	0.007	0.015	6.430E-01	0.995	pan	SARS-CoV-2 infection
16057_6	IGF-II receptor	Cation-independent mannose-6-phosphate receptor	P11717	IGF2R	NA	Inverse variance weighted	8	0.005	0.010	6.430E-01	0.995	pan	SARS-CoV-2 infection
12628_31	LANC2	LanC-like protein 2	Q9NS86	LANCL2	NA	Wald ratio	1	0.054	0.117	6.432E-01	0.995	pan	SARS-CoV-2 infection
10772_21	CGAT2	Chondroitin sulfate N-acetylgalactosaminyltransferase 2	Q8N6G5	CSGALNACT2	NA	Wald ratio	1	-0.023	0.051	6.433E-01	0.995	pan	SARS-CoV-2 infection
9867_23	F16P2	Fructose-1,6-bisphosphatase isozyme 2	O00757	FBP2	NA	Wald ratio	1	-0.023	0.049	6.434E-01	0.995	pan	SARS-CoV-2 infection
15385_116	FABP2	Fatty acid-binding protein, intestinal	P12104	FABP2	NA	Inverse variance weighted	10	0.006	0.013	6.439E-01	0.995	pan	SARS-CoV-2 infection
5012_67	Myokinase, human	Adenylate kinase isoenzyme 1	P00568	AK1	NA	Wald ratio	1	-0.052	0.113	6.448E-01	0.995	pan	SARS-CoV-2 infection
15427_35	LOXL3	Lysyl oxidase homolog 3	P58215	LOXL3	NA	Inverse variance weighted	11	-0.008	0.018	6.449E-01	0.995	pan	SARS-CoV-2 infection
3221_54	SARP-2	Secreted frizzled-related protein 1	Q8N474	SFRP1	NA	Inverse variance weighted	6	0.009	0.020	6.451E-01	0.995	pan	SARS-CoV-2 infection
2811_27	Angiopoietin-1	Angiopoietin-1	Q15389	ANGPT1	NA	Inverse variance weighted	2	0.028	0.061	6.455E-01	0.995	pan	SARS-CoV-2 infection
5638_23	GT251	Procollagen galactosyltransferase 1	Q8NBJ5	COLGALT1	NA	Inverse variance weighted	9	0.012	0.025	6.457E-01	0.995	pan	SARS-CoV-2 infection
6927_7	NDST1	Bifunctional heparan sulfate N-deacetylase/N-sulfotransferase 1	P52848	NDST1	NA	Inverse variance weighted	3	-0.017	0.037	6.462E-01	0.995	pan	SARS-CoV-2 infection
5021_13	PPase	Inorganic pyrophosphatase	Q15181	PPA1	NA	Inverse variance weighted	5	0.012	0.026	6.462E-01	0.995	pan	SARS-CoV-2 infection
16851_50	SCO2	Protein SCO2 homolog, mitochondrial	O43819	SCO2	NA	Wald ratio	1	0.023	0.050	6.463E-01	0.995	pan	SARS-CoV-2 infection
12620_3	Septin-11	Septin-11	Q9NVA2	SEPTIN11	NA	Inverse variance weighted	2	-0.028	0.061	6.467E-01	0.995	pan	SARS-CoV-2 infection
17391_10	SPF45	Splicing factor 45	Q96I25	RBM17	NA	Inverse variance weighted	2	0.011	0.023	6.473E-01	0.995	pan	SARS-CoV-2 infection
3000_66	MBL	Mannose-binding protein C	P11226	MBL2	NA	Inverse variance weighted	21	0.003	0.008	6.478E-01	0.995	pan	SARS-CoV-2 infection
8428_102	NTRI	Neurotrimin	Q9P121	NTM	NA	Inverse variance weighted	4	-0.019	0.042	6.483E-01	0.995	pan	SARS-CoV-2 infection
13733_5	IL-12 p40	Interleukin-12 subunit beta	P29460	IL12B	NA	Inverse variance weighted	16	-0.007	0.015	6.484E-01	0.995	pan	SARS-CoV-2 infection
16305_10	Cadherin-11:ECD	Cadherin-11:Extracellular domain	P55287	CDH11	NA	Inverse variance weighted	10	-0.013	0.028	6.484E-01	0.995	pan	SARS-CoV-2 infection
8225_86	EPHB2	Ephrin type-B receptor 2	P29323	EPHB2	NA	Inverse variance weighted	3	0.013	0.029	6.491E-01	0.995	pan	SARS-CoV-2 infection
16317_20	Desmoglein-3	Desmoglein-3	P32926	DSG3	NA	Inverse variance weighted	2	-0.033	0.073	6.496E-01	0.995	pan	SARS-CoV-2 infection
9595_11	B4GT2	Beta-1,4-galactosyltransferase 2	O60909	B4GALT2	NA	Inverse variance weighted	4	0.011	0.025	6.500E-01	0.995	pan	SARS-CoV-2 infection
6626_81	CHSTC	Carbohydrate sulfotransferase 12	Q9NRB3	CHST12	NA	Inverse variance weighted	2	-0.012	0.027	6.503E-01	0.995	pan	SARS-CoV-2 infection
6572_10	LRRT4:ECD	Leucine-rich repeat transmembrane neuronal protein 4:Isoform 2, Extracellular domain	Q86VH4	LRRTM4	NA	Wald ratio	1	0.046	0.101	6.508E-01	0.995	pan	SARS-CoV-2 infection
4271_75	PFD5	Prefoldin subunit 5	Q99471	PFDN5	NA	Wald ratio	1	0.090	0.200	6.510E-01	0.995	pan	SARS-CoV-2 infection

4155_3	Tenascin	Tenascin	P24821	TNC	NA	Inverse variance weighted	12	-0.006	0.014	6.523E-01	0.995	pan	SARS-CoV-2 infection
11117_2	SPT20	Spermatogenesis-associated protein 20	Q8TB22	SPATA20	NA	Inverse variance weighted	4	-0.014	0.031	6.544E-01	0.995	pan	SARS-CoV-2 infection
13587_10	RGAP1	Rac GTPase-activating protein 1	Q9H0H5	RACGAP1	NA	Inverse variance weighted	12	-0.009	0.020	6.546E-01	0.995	pan	SARS-CoV-2 infection
17210_2	TCL1A	T-cell leukemia/lymphoma protein 1A	P56279	TCL1A	NA	Inverse variance weighted	5	-0.010	0.023	6.548E-01	0.995	pan	SARS-CoV-2 infection
17776_15	PECR	Peroxisomal trans-2-enoyl-CoA reductase	Q9BY49	PECR	NA	Wald ratio	1	0.023	0.052	6.553E-01	0.995	pan	SARS-CoV-2 infection
6478_2	IGLO5	IgLON family member 5	A6NGN9	IGLON5	NA	Wald ratio	1	0.055	0.123	6.555E-01	0.995	pan	SARS-CoV-2 infection
16049_43	OLR1	Oxidized low-density lipoprotein receptor 1	P78380	OLR1	NA	Wald ratio	1	0.026	0.059	6.561E-01	0.995	pan	SARS-CoV-2 infection
8006_12	DJB12	DnaJ homolog subfamily B member 12	Q9NXW2	DNAJB12	NA	Inverse variance weighted	2	-0.018	0.041	6.569E-01	0.995	pan	SARS-CoV-2 infection
13575_40	DVL2	Segment polarity protein dishevelled homolog DVL-2	O14641	DVL2	NA	Wald ratio	1	-0.045	0.102	6.571E-01	0.995	pan	SARS-CoV-2 infection
15343_337	Kininogen, HMW, Two Chain	Kininogen, HMW, Two Chain	P01042	KNG1	NA	Inverse variance weighted	4	-0.041	0.092	6.574E-01	0.995	pan	SARS-CoV-2 infection
18311_44	DCTN6	Dynactin subunit 6	O00399	DCTN6	NA	Inverse variance weighted	2	-0.025	0.055	6.578E-01	0.995	pan	SARS-CoV-2 infection
12350_86	ETS2	Protein C-ets-2	P15036	ETS2	NA	Inverse variance weighted	6	0.025	0.057	6.584E-01	0.995	pan	SARS-CoV-2 infection
16309_30	Soggy-1	Dickkopf-like protein 1	Q9UK85	DKKL1	NA	Wald ratio	1	0.047	0.106	6.585E-01	0.995	pan	SARS-CoV-2 infection
16324_38	TLR1:ECD	Toll-like receptor 1:Extracellular domain	Q15399	TLR1	NA	Inverse variance weighted	2	0.021	0.047	6.596E-01	0.995	pan	SARS-CoV-2 infection
7178_59	DEPP	Protein DEPP	Q9NTK1	DEPP1	NA	Inverse variance weighted	6	0.020	0.045	6.605E-01	0.995	pan	SARS-CoV-2 infection
12786_61	GDE5	Glycerophosphocholine phosphodiesterase GPCPD1	Q9NPB8	GPCPD1	NA	Inverse variance weighted	2	-0.027	0.061	6.609E-01	0.995	pan	SARS-CoV-2 infection
18197_97	KCRS	Creatine kinase S-type, mitochondrial	P17540	CKMT2	NA	Inverse variance weighted	2	0.042	0.095	6.611E-01	0.995	pan	SARS-CoV-2 infection
5852_6	S100A12	Protein S100-A12	P80511	S100A12	NA	Inverse variance weighted	2	0.017	0.040	6.620E-01	0.995	pan	SARS-CoV-2 infection
8000_17	KI2LA	Killer cell immunoglobulin-like receptor 2DL5A	Q8N109	KIR2DL5A	NA	Inverse variance weighted	8	-0.006	0.014	6.625E-01	0.995	pan	SARS-CoV-2 infection
15364_101	Apo C-I	Apolipoprotein C-I	P02654	APOC1	NA	Inverse variance weighted	2	-0.073	0.168	6.626E-01	0.995	pan	SARS-CoV-2 infection
15470_11	Hexosaminidase B	Beta-hexosaminidase subunit beta	P07686	HEXB	NA	Inverse variance weighted	12	0.007	0.016	6.637E-01	0.995	pan	SARS-CoV-2 infection
18308_30	STXB6	Syntaxin-binding protein 6	Q8NFX7	STXBP6	NA	Wald ratio	1	-0.044	0.101	6.639E-01	0.995	pan	SARS-CoV-2 infection
17735_130	GBRAP	Gamma-aminobutyric acid receptor-associated protein	O95166	GABARAP	NA	Wald ratio	1	0.041	0.095	6.645E-01	0.995	pan	SARS-CoV-2 infection
5649_83	PSG4	Pregnancy-specific beta-1-glycoprotein 4	Q00888	PSG4	NA	Inverse variance weighted	4	-0.023	0.052	6.648E-01	0.995	pan	SARS-CoV-2 infection
12895_28	DGKB	Diacylglycerol kinase beta	Q9Y6T7	DGKB	NA	Wald ratio	1	0.037	0.085	6.671E-01	0.995	pan	SARS-CoV-2 infection
9839_148	Tirap	Toll/interleukin-1 receptor domain-containing adapter protein	P58753	TIRAP	NA	Inverse variance weighted	3	0.028	0.064	6.675E-01	0.995	pan	SARS-CoV-2 infection
17364_8	RU2B	U2 small nuclear ribonucleoprotein B''	P08579	SNRPB2	NA	Wald ratio	1	0.048	0.111	6.680E-01	0.995	pan	SARS-CoV-2 infection
9226_6	TMUB2	Transmembrane and ubiquitin-like domain-containing protein 2	Q71RG4	TMUB2	NA	Wald ratio	1	0.049	0.113	6.681E-01	0.995	pan	SARS-CoV-2 infection
4467_49	SPARCL1	SPARC-like protein 1	Q14515	SPARCL1	NA	Inverse variance weighted	10	-0.006	0.015	6.688E-01	0.995	pan	SARS-CoV-2 infection
18895_54	GSTM4	Glutathione S-transferase Mu 4	Q03013	GSTM4	NA	Inverse variance weighted	11	-0.010	0.025	6.695E-01	0.995	pan	SARS-CoV-2 infection
8465_52	Cathepsin H	Cathepsin H	P09668	CTSH	NA	Inverse variance weighted	17	-0.003	0.008	6.703E-01	0.995	pan	SARS-CoV-2 infection
19197_95	THIL	Acetyl-CoA acetyltransferase, mitochondrial	P24752	ACAT1	NA	Inverse variance weighted	3	0.021	0.050	6.713E-01	0.995	pan	SARS-CoV-2 infection
9213_24	FTCD	Formimidoyltransferase-cyclodeaminase	O95954	FTCD	NA	Inverse variance weighted	3	-0.020	0.047	6.716E-01	0.995	pan	SARS-CoV-2 infection
17739_1	HCDH	Hydroxyacyl-coenzyme A dehydrogenase, mitochondrial	Q16836	HADH	NA	Wald ratio	1	-0.030	0.071	6.721E-01	0.995	pan	SARS-CoV-2 infection
10584_7	NDUS4	NADH dehydrogenase [ubiquinone] iron-sulfur protein 4, mitochondrial	O43181	NDUFS4	NA	Inverse variance weighted	19	0.012	0.029	6.723E-01	0.995	pan	SARS-CoV-2 infection
12764_3	ELMO1	Engulfment and cell motility protein 1	Q92556	ELMO1	NA	Inverse variance weighted	3	-0.024	0.057	6.740E-01	0.995	pan	SARS-CoV-2 infection
12851_5	DPOLM	DNA-directed DNA/RNA polymerase mu	Q9NP87	POLM	NA	Inverse variance weighted	3	0.012	0.028	6.747E-01	0.995	pan	SARS-CoV-2 infection
7784_1	Kininogen, HMW	Kininogen-1	P01042	KNG1	NA	Inverse variance weighted	2	-0.029	0.069	6.753E-01	0.995	pan	SARS-CoV-2 infection
3391_10	PK3CG	Phosphatidylinositol 4,5-bisphosphate 3-kinase catalytic subunit gamma isoform	P48736	PIK3CG	NA	Wald ratio	1	-0.042	0.101	6.755E-01	0.995	pan	SARS-CoV-2 infection
9233_71	TFPI -2	Tissue factor pathway inhibitor 2	P48307	TFPI2	NA	Inverse variance weighted	2	0.027	0.066	6.761E-01	0.995	pan	SARS-CoV-2 infection
18301_10	NAIF1	Nuclear apoptosis-inducing factor 1	Q69YI7	NAIF1	NA	Inverse variance weighted	3	-0.019	0.047	6.763E-01	0.995	pan	SARS-CoV-2 infection
5798_3	BID	BH3-interacting domain death agonist	P55957	BID	NA	Wald ratio	1	0.027	0.065	6.770E-01	0.995	pan	SARS-CoV-2 infection
19558_10	LRP4	Low-density lipoprotein receptor-related protein 4	O75096	LRP4	NA	Inverse variance weighted	11	-0.009	0.023	6.771E-01	0.995	pan	SARS-CoV-2 infection
9380_2	sPLA(2)-XIII	Group XIIB secretory phospholipase A2-like protein	Q9BX93	PLA2G12B	NA	Inverse variance weighted	2	-0.027	0.064	6.778E-01	0.995	pan	SARS-CoV-2 infection
9940_35	DUS28	Dual specificity phosphatase 28	Q4G0W2	DUSP28	NA	Inverse variance weighted	3	-0.012	0.028	6.781E-01	0.995	pan	SARS-CoV-2 infection
15529_33	Cysteine-rich protein 1	Cysteine and glycine-rich protein 1	P21291	CSRP1	NA	Inverse variance weighted	2	-0.026	0.064	6.783E-01	0.995	pan	SARS-CoV-2 infection
19159_9	DNA polymerase subunit delta 4	DNA polymerase delta subunit 4	Q9HCU8	POLD4	NA	Wald ratio	1	-0.045	0.108	6.809E-01	0.995	pan	SARS-CoV-2 infection
9504_19	RB27A	Ras-related protein Rab-27A	P51159	RAB27A	NA	Wald ratio	1	-0.020	0.050	6.815E-01	0.995	pan	SARS-CoV-2 infection
13384_110	FUMH	Fumarate hydratase, mitochondrial	P07954	FH	NA	Inverse variance weighted	2	0.026	0.063	6.817E-01	0.995	pan	SARS-CoV-2 infection
12575_30	C1TC	C-1-tetrahydrofolate synthase, cytoplasmic	P11586	MTHFD1	NA	Inverse variance weighted	5	0.018	0.044	6.823E-01	0.995	pan	SARS-CoV-2 infection
13993_20	E41L1	Band 4.1-like protein 1	Q9H4G0	EPB41L1	NA	Inverse variance weighted	2	0.061	0.148	6.829E-01	0.995	pan	SARS-CoV-2 infection
19379_154	RAB3D	Ras-related protein Rab-3D	O95716	RAB3D	NA	Wald ratio	1	-0.034	0.083	6.829E-01	0.995	pan	SARS-CoV-2 infection
7871_16	T132A	Transmembrane protein 132A	Q24JP5	TMEM132A	NA	Inverse variance weighted	9	0.005	0.011	6.837E-01	0.995	pan	SARS-CoV-2 infection
2813_11	ART	Agouti-related protein	O00253	AGRP	NA	Inverse variance weighted	5	-0.018	0.045	6.838E-01	0.995	pan	SARS-CoV-2 infection
18380_78	Albumin	Serum albumin	P02768	ALB	NA	Inverse variance weighted	2	0.032	0.078	6.839E-01	0.995	pan	SARS-CoV-2 infection
6518_85	ghrelin	Appetite-regulating hormone	Q9UBU3	GHRL	NA	Inverse variance weighted	4	-0.017	0.042	6.841E-01	0.995	pan	SARS-CoV-2 infection
8382_47	MAPK5	MAP kinase-activated protein kinase 5	Q8IW41	MAPKAPK5	NA	Inverse variance weighted	12	-0.008	0.020	6.844E-01	0.995	pan	SARS-CoV-2 infection
17675_17	ACO13	Acyl-coenzyme A thioesterase 13	Q9NPJ3	ACOT13	NA	Wald ratio	1	-0.039	0.096	6.847E-01	0.995	pan	SARS-CoV-2 infection
7192_37	CRF2-12	Interferon lambda receptor 1	Q8IU57	IFNLR1	NA	Inverse variance weighted	2	0.017	0.043	6.851E-01	0.995	pan	SARS-CoV-2 infection
18313_4	Asparaginase-like protein 1	Isoaspartyl peptidase/L-asparaginase	Q7L266	ASRGL1	NA	Inverse variance weighted	4	-0.008	0.020	6.859E-01	0.995	pan	SARS-CoV-2 infection
8398_277	RAR-responsive protein TIG1	Retinoic acid receptor responder protein 1	P49788	RARRES1	NA	Inverse variance weighted	16	0.003	0.008	6.859E-01	0.995	pan	SARS-CoV-2 infection
3179_51	Cathepsin A	Lysosomal protective protein	P10619	CTSA	NA	Inverse variance weighted	2	-0.077	0.191	6.864E-01	0.995	pan	SARS-CoV-2 infection
3302_58	CYTF	Cystatin-F	O76096	CST7	NA	Inverse variance weighted	13	0.004	0.009	6.866E-01	0.995	pan	SARS-CoV-2 infection
15506_34	LRP12	Low-density lipoprotein receptor-related protein 12	Q9Y561	LRP12	NA	Wald ratio	1	0.039	0.098	6.875E-01	0.995	pan	SARS-CoV-2 infection
14747_9	CRLF1	Cytokine receptor-like factor 1	O75462	CRLF1	NA	Inverse variance weighted	2	0.013	0.031	6.880E-01	0.995	pan	SARS-CoV-2 infection
9237_54	Lysosomal acid phosphatase	Lysosomal acid phosphatase	P11117	ACP2	NA	Inverse variance weighted	3	-0.019	0.047	6.882E-01	0.995	pan	SARS-CoV-2 infection
18181_2	TSSC4	Protein TSSC4	Q9Y5U2	TSSC4	NA	Wald ratio	1	0.049	0.123	6.892E-01	0.995	pan	SARS-CoV-2 infection
8268_98	HS3SA	Heparan sulfate glucosamine 3-O-sulfotransferase 3A1	Q9Y663	HS3ST3A1	NA	Inverse variance weighted	2	-0.017	0.043	6.901E-01	0.995	pan	SARS-CoV-2 infection
2692_74	NPS-PLA2	Phospholipase A2, membrane associated	P14555	PLA2G2A	NA	Inverse variance weighted	7	0.003	0.008	6.903E-01	0.995	pan	SARS-CoV-2 infection
18896_23	H6ST3	Heparan-sulfate 6-O-sulfotransferase 3	Q8IZP7	HS6ST3	NA	Inverse variance weighted	2	0.023	0.059	6.907E-01	0.995	pan	SARS-CoV-2 infection
8786_6	F171B:ECD	Protein FAM171B:Extracellular domain	Q6P995	FAM171B	NA	Wald ratio	1	-0.013	0.032	6.913E-01	0.995	pan	SARS-CoV-2 infection
18413_24	ARL4D	ADP-ribosylation factor-like protein 4D	P49703	ARL4D	NA	Wald ratio	1	-0.035	0.089	6.915E-01	0.995	pan	SARS-CoV-2 infection
4992_49	GRN	Granulins	P28799	GRN	NA	Inverse variance weighted	9	-0.011	0.028	6.916E-01	0.995	pan	SARS-CoV-2 infection
15453_3	a1-Microglobulin	Alpha-1-microglobulin	P02760	AMBP	NA	Wald ratio	1	0.034	0.087	6.917E-01	0.995	pan	SARS-CoV-2 infection
6440_31	MFAP5	Microfibrillar-associated protein 5	Q13361	MFAP5	NA	Inverse variance weighted	3	-0.025	0.062	6.924E-01	0.995	pan	SARS-CoV-2 infection
12394_53	CP054	Transmembrane protein C16orf54	Q6UWD8	C16orf54	NA	Wald ratio	1	0.067	0.171	6.926E-01	0.995	pan	SARS-CoV-2 infection
14106_46	GDP/GTP exchange protein	Rap1 GTPase-GDP dissociation stimulator 1	P52306	RAP1GDS1	NA	Wald ratio	1	-0.038	0.097	6.927E-01	0.995	pan	SARS-CoV-2 infection
12641_3	I5P2	Type II inositol 1,4,5-trisphosphate 5-phosphatase	P32019	INPP5B	NA	Inverse variance weighted	2	0.012	0.031	6.927E-01	0.995	pan	SARS-CoV-2 infection

3030_3	DC-SIGNR	C-type lectin domain family 4 member M	Q9H2X3	CLEC4M	NA	Wald ratio	1	-0.041	0.103	6.930E-01	0.995	pan	SARS-CoV-2 infection
13476_16	KIN17	DNA/RNA-binding protein KIN17	O60870	KIN	NA	Wald ratio	1	-0.037	0.094	6.930E-01	0.995	pan	SARS-CoV-2 infection
2890_59	CCL28	C-C motif chemokine 28	Q9NRJ3	CCL28	NA	Inverse variance weighted	8	-0.011	0.027	6.931E-01	0.995	pan	SARS-CoV-2 infection
15615_8	LIRB3	Leukocyte immunoglobulin-like receptor subfamily B member 3	O75022	LILRB3	NA	Inverse variance weighted	5	0.008	0.020	6.932E-01	0.995	pan	SARS-CoV-2 infection
3040_59	MIP-1a	C-C motif chemokine 3	P10147	CCL3	NA	Wald ratio	1	0.039	0.100	6.938E-01	0.995	pan	SARS-CoV-2 infection
8048_9	FTMT	Ferritin, mitochondrial	Q8N4E7	FTMT	NA	Inverse variance weighted	8	0.010	0.027	6.942E-01	0.995	pan	SARS-CoV-2 infection
11278_4	COL11A2	Collagen alpha-2(XI) chain	P13942	COL11A2	NA	Inverse variance weighted	7	-0.007	0.018	6.952E-01	0.995	pan	SARS-CoV-2 infection
9172_69	MMP-8	Neutrophil collagenase	P22894	MMP8	NA	Inverse variance weighted	8	0.009	0.023	6.953E-01	0.995	pan	SARS-CoV-2 infection
7628_40	CREL1	Cysteine-rich with EGF-like domain protein 1	Q96HD1	CRELD1	NA	Inverse variance weighted	13	0.004	0.009	6.954E-01	0.995	pan	SARS-CoV-2 infection
12546_1	SMTN	Smoothelin	P53814	SMTN	NA	Wald ratio	1	0.038	0.098	6.963E-01	0.995	pan	SARS-CoV-2 infection
7128_9	VWA2	von Willebrand factor A domain-containing protein 2	Q5GFL6	VWA2	NA	Inverse variance weighted	7	0.008	0.020	6.965E-01	0.995	pan	SARS-CoV-2 infection
2571_12	IGFBP-3	Insulin-like growth factor-binding protein 3	P17936	IGFBP3	NA	Inverse variance weighted	8	-0.006	0.016	6.968E-01	0.995	pan	SARS-CoV-2 infection
8097_77	LIPN	Lipase member N	Q5VXI9	LIPN	NA	Inverse variance weighted	15	-0.005	0.013	6.969E-01	0.995	pan	SARS-CoV-2 infection
15576_158	ECP	Eosinophil cationic protein	P12724	RNASE3	NA	Inverse variance weighted	9	-0.005	0.013	6.972E-01	0.995	pan	SARS-CoV-2 infection
16614_27	RSPO1	R-spondin-1	Q2MKA7	RSPO1	NA	Inverse variance weighted	3	0.008	0.021	6.973E-01	0.995	pan	SARS-CoV-2 infection
8885_6	CA2D3	Voltage-dependent calcium channel subunit alpha-2/delta-3	Q8IZS8	CACNA2D3	NA	Inverse variance weighted	9	-0.011	0.029	6.978E-01	0.995	pan	SARS-CoV-2 infection
13268_45	WNT5A	Protein Wnt-5a	P41221	WNT5A	NA	Inverse variance weighted	5	-0.015	0.039	6.978E-01	0.995	pan	SARS-CoV-2 infection
11214_40	DNJB9	DnaJ homolog subfamily B member 9	Q9UBS3	DNAJB9	NA	Wald ratio	1	-0.042	0.109	6.981E-01	0.995	pan	SARS-CoV-2 infection
13748_4	MCP-2	C-C motif chemokine 8	P80075	CCL8	NA	Inverse variance weighted	10	0.008	0.020	6.992E-01	0.995	pan	SARS-CoV-2 infection
9185_15	TFF1	Trefoil factor 1	P04155	TFF1	NA	Inverse variance weighted	8	-0.006	0.015	7.001E-01	0.995	pan	SARS-CoV-2 infection
10900_272	STMN2	Stathmin-2	Q93045	STMN2	NA	Wald ratio	1	-0.041	0.107	7.013E-01	0.995	pan	SARS-CoV-2 infection
15686_49	INHBC	Inhibin beta C chain	P55103	INHBC	NA	Inverse variance weighted	17	-0.004	0.011	7.018E-01	0.995	pan	SARS-CoV-2 infection
15339_32	COF2	Cofilin-2	Q9Y281	CFL2	NA	Wald ratio	1	-0.016	0.042	7.021E-01	0.995	pan	SARS-CoV-2 infection
17764_108	RHOC	Rho-related GTP-binding protein RhoC	P08134	RHOC	NA	Inverse variance weighted	2	0.022	0.058	7.021E-01	0.995	pan	SARS-CoV-2 infection
11360_39	RRM1	Ribonucleoside-diphosphate reductase large subunit	P23921	RRM1	NA	Inverse variance weighted	17	-0.006	0.016	7.027E-01	0.995	pan	SARS-CoV-2 infection
14008_22	DCP1A	mRNA-decapping enzyme 1A	Q9NPI6	DCP1A	NA	Inverse variance weighted	3	0.009	0.024	7.029E-01	0.995	pan	SARS-CoV-2 infection
14007_22	PAPS1	Bifunctional 3'-phosphoadenosine 5'-phosphosulfate synthase 1	O43252	PAPSS1	NA	Inverse variance weighted	2	0.021	0.054	7.029E-01	0.995	pan	SARS-CoV-2 infection
8303_102	MYG1	UPF0160 protein MYG1, mitochondrial	Q9HB07	MYG1	NA	Wald ratio	1	-0.025	0.066	7.030E-01	0.995	pan	SARS-CoV-2 infection
6039_24	CRHBP	Corticotropin-releasing factor-binding protein	P24387	CRHBP	NA	Inverse variance weighted	8	-0.005	0.013	7.031E-01	0.995	pan	SARS-CoV-2 infection
3166_92	Siglec-3	Myeloid cell surface antigen CD33	P20138	CD33	NA	Inverse variance weighted	6	-0.004	0.011	7.032E-01	0.995	pan	SARS-CoV-2 infection
7999_23	CD39	Ectonucleoside triphosphate diphosphohydrolase 1	P49961	ENTPD1	NA	Inverse variance weighted	3	-0.011	0.028	7.038E-01	0.995	pan	SARS-CoV-2 infection
19141_22	DAP1	Death-associated protein 1	P51397	DAP	NA	Inverse variance weighted	7	-0.011	0.029	7.041E-01	0.995	pan	SARS-CoV-2 infection
18899_82	HDGR3	Hepatoma-derived growth factor-related protein 3	Q9Y3E1	HDGFL3	NA	Inverse variance weighted	3	-0.015	0.040	7.054E-01	0.995	pan	SARS-CoV-2 infection
2819_23	Cadherin-5	Cadherin-5	P33151	CDH5	NA	Inverse variance weighted	5	0.012	0.032	7.054E-01	0.995	pan	SARS-CoV-2 infection
13943_38	DPY30	Protein dpy-30 homolog	Q9C005	DPY30	NA	Inverse variance weighted	2	-0.031	0.083	7.059E-01	0.995	pan	SARS-CoV-2 infection
16593_3	FADD	FAS-associated death domain protein	Q13158	FADD	NA	Inverse variance weighted	2	0.032	0.086	7.059E-01	0.995	pan	SARS-CoV-2 infection
6990_44	SDF2L	Stromal cell-derived factor 2-like protein 1	Q9HCN8	SDF2L1	NA	Wald ratio	1	-0.035	0.094	7.060E-01	0.995	pan	SARS-CoV-2 infection
19553_14	STX1a	Syntaxin-1A	Q16623	STX1A	NA	Inverse variance weighted	4	-0.019	0.051	7.063E-01	0.995	pan	SARS-CoV-2 infection
8960_3	LRAP	Endoplasmic reticulum aminopeptidase 2	Q6P179	ERAP2	NA	Inverse variance weighted	27	-0.004	0.010	7.069E-01	0.995	pan	SARS-CoV-2 infection
5658_64	coagulation factor XIII B	Coagulation factor XIII B chain	P05160	F13B	NA	Inverse variance weighted	3	-0.023	0.063	7.091E-01	0.995	pan	SARS-CoV-2 infection
3389_7	PCI	Plasma serine protease inhibitor	P05154	SERPINA5	NA	Inverse variance weighted	2	-0.025	0.066	7.093E-01	0.995	pan	SARS-CoV-2 infection
14332_3	RALA	Ras-related protein Ral-A	P11233	RALA	NA	Inverse variance weighted	2	-0.029	0.079	7.095E-01	0.995	pan	SARS-CoV-2 infection
10445_20	ApoM	Apolipoprotein M	O95445	APOM	NA	Inverse variance weighted	2	-0.021	0.056	7.098E-01	0.995	pan	SARS-CoV-2 infection
10643_16	NOE3:C-term	Noelin-3:Isoform 2, C-term	Q96PB7	OLFM3	NA	Wald ratio	1	-0.035	0.095	7.104E-01	0.995	pan	SARS-CoV-2 infection
7194_36	NPTN	Neuroplastin	Q9Y639	NPTN	NA	Inverse variance weighted	2	-0.026	0.069	7.114E-01	0.995	pan	SARS-CoV-2 infection
10892_8	OSMR	Oncostatin-M-specific receptor subunit beta	Q99650	OSMR	NA	Inverse variance weighted	2	0.036	0.098	7.126E-01	0.995	pan	SARS-CoV-2 infection
6603_18	KALM	Anosmin-1	P23352	ANOS1	NA	Inverse variance weighted	3	0.017	0.046	7.129E-01	0.995	pan	SARS-CoV-2 infection
2855_49	ERK-1	Mitogen-activated protein kinase 3	P27361	MAPK3	NA	Inverse variance weighted	2	-0.036	0.097	7.129E-01	0.995	pan	SARS-CoV-2 infection
5621_64	THSD1	Thrombospondin type-1 domain-containing protein 1	Q9NS62	THSD1	NA	Inverse variance weighted	13	0.005	0.015	7.136E-01	0.995	pan	SARS-CoV-2 infection
13119_26	protein Z inhibitor	Protein Z-dependent protease inhibitor	Q9UK55	SERPINA10	NA	Inverse variance weighted	16	-0.007	0.019	7.143E-01	0.995	pan	SARS-CoV-2 infection
11708_2	LCN1	Lipocalin-1	P31025	LCN1	NA	Wald ratio	1	0.039	0.106	7.159E-01	0.995	pan	SARS-CoV-2 infection
17691_1	TPP1	Tripeptidyl-peptidase 1	O14773	TPP1	NA	Inverse variance weighted	7	-0.008	0.022	7.164E-01	0.995	pan	SARS-CoV-2 infection
9398_30	GALP	Galanin-like peptide	Q9UBC7	GALP	NA	Wald ratio	1	-0.030	0.083	7.166E-01	0.995	pan	SARS-CoV-2 infection
13434_172	PARVA	Alpha-parvin	Q9NVD7	PARVA	NA	Wald ratio	1	-0.022	0.060	7.167E-01	0.995	pan	SARS-CoV-2 infection
13614_6	CREB-binding protein	CREB-binding protein	Q92793	CREBBP	NA	Inverse variance weighted	3	0.032	0.087	7.167E-01	0.995	pan	SARS-CoV-2 infection
8759_29	a1,4-Galactosyltransferase	Lactosylceramide 4-alpha-galactosyltransferase	Q9NPC4	A4GALT	NA	Inverse variance weighted	16	0.004	0.011	7.169E-01	0.995	pan	SARS-CoV-2 infection
4141_79	IP-10	C-X-C motif chemokine 10	P02778	CXCL10	NA	Inverse variance weighted	2	0.017	0.047	7.174E-01	0.995	pan	SARS-CoV-2 infection
14309_8	HNRH1	Heterogeneous nuclear ribonucleoprotein H	P31943	HNRNPH1	NA	Wald ratio	1	-0.037	0.102	7.180E-01	0.995	pan	SARS-CoV-2 infection
12612_37	PSB1	Proteasome subunit beta type-1	P20618	PSMB1	NA	Inverse variance weighted	3	0.005	0.015	7.191E-01	0.995	pan	SARS-CoV-2 infection
8909_77	GNPI1	Glucosamine-6-phosphate isomerase 1	P46926	GNPDA1	NA	Wald ratio	1	0.013	0.037	7.195E-01	0.995	pan	SARS-CoV-2 infection
10514_5	PGD2 synthase	Prostaglandin-H2 D-isomerase	P41222	PTGDS	NA	Inverse variance weighted	3	0.021	0.059	7.195E-01	0.995	pan	SARS-CoV-2 infection
14685_17	PKB beta	RAC-beta serine/threonine-protein kinase	P31751	AKT2	NA	Inverse variance weighted	5	-0.022	0.062	7.200E-01	0.995	pan	SARS-CoV-2 infection
9322_15	RCN1	Reticulocalbin-1	Q15293	RCN1	NA	Inverse variance weighted	2	0.025	0.069	7.207E-01	0.995	pan	SARS-CoV-2 infection
3499_77	IL-17B	Interleukin-17B	Q9UHF5	IL17B	NA	Wald ratio	1	-0.025	0.070	7.207E-01	0.995	pan	SARS-CoV-2 infection
17808_37	NIT2	Omega-amidase NIT2	Q9NQR4	NIT2	NA	Inverse variance weighted	7	-0.013	0.036	7.213E-01	0.995	pan	SARS-CoV-2 infection
19338_3	LZIC	Protein LZIC	Q8WZA0	LZIC	NA	Wald ratio	1	-0.038	0.106	7.216E-01	0.995	pan	SARS-CoV-2 infection
8358_30	Peroxiredoxin-3	Thioredoxin-dependent peroxide reductase, mitochondrial	P30048	PRDX3	NA	Wald ratio	1	-0.037	0.103	7.217E-01	0.995	pan	SARS-CoV-2 infection
2567_5	Factor I	Complement factor I	P05156	CFI	NA	Inverse variance weighted	2	0.013	0.036	7.225E-01	0.995	pan	SARS-CoV-2 infection
8447_11	ghrelin	Appetite-regulating hormone	Q9UBU3	GHRL	NA	Inverse variance weighted	2	-0.025	0.071	7.225E-01	0.995	pan	SARS-CoV-2 infection
11516_7	FABPL	Fatty acid-binding protein, liver	P07148	FABP1	NA	Wald ratio	1	-0.016	0.044	7.231E-01	0.995	pan	SARS-CoV-2 infection
15626_223	Perlecan	Basement membrane-specific heparan sulfate proteoglycan core protein	P98160	HSPG2	NA	Inverse variance weighted	6	-0.013	0.035	7.232E-01	0.995	pan	SARS-CoV-2 infection
12758_47	GRID2	Glutamate receptor ionotropic, delta-2	O43424	GRID2	NA	Inverse variance weighted	10	0.011	0.032	7.233E-01	0.995	pan	SARS-CoV-2 infection
10041_3	HNF4A	Hepatocyte nuclear factor 4-alpha	P41235	HNF4A	NA	Wald ratio	1	-0.036	0.101	7.235E-01	0.995	pan	SARS-CoV-2 infection
2962_50	PTHrP	Parathyroid hormone-related protein	P12272	PTHLH	NA	Inverse variance weighted	3	0.012	0.035	7.243E-01	0.995	pan	SARS-CoV-2 infection
10053_5	ILK1	Integrin-linked protein kinase	Q13418	ILK	NA	Wald ratio	1	0.031	0.088	7.249E-01	0.995	pan	SARS-CoV-2 infection
8970_9	RIPK2	Receptor-interacting serine/threonine-protein kinase 2	O43353	RIPK2	NA	Wald ratio	1	0.029	0.083	7.251E-01	0.995	pan	SARS-CoV-2 infection
13744_37	IL-3 Ra	Interleukin-3 receptor subunit alpha	P26951	IL3RA	NA	Inverse variance weighted	3	0.018	0.050	7.252E-01	0.995	pan	SARS-CoV-2 infection
16828_8	Collagen a1(VI)	Collagen alpha-1(VI) chain	P12109	COL6A1	NA	Inverse variance weighted	15	-0.003	0.008	7.270E-01	0.995	pan	SARS-CoV-2 infection

2944_66	DAN	Neuroblastoma suppressor of tumorigenicity 1	P41271	NBL1	NA	Wald ratio	1	0.032	0.092	7.272E-01	0.995	pan	SARS-CoV-2 infection
3298_52	Contactin-4	Contactin-4	Q8IWV2	CNTN4	NA	Inverse variance weighted	8	-0.005	0.013	7.273E-01	0.995	pan	SARS-CoV-2 infection
18236_3	LEGL	Galectin-related protein	Q3ZCW2	LGALSL	NA	Wald ratio	1	-0.022	0.064	7.291E-01	0.995	pan	SARS-CoV-2 infection
8289_8	GPNMB:ECD	Transmembrane glycoprotein NMB:Extracellular domain	Q14956	GPNMB	NA	Inverse variance weighted	8	0.008	0.024	7.292E-01	0.995	pan	SARS-CoV-2 infection
16858_384	RCN3	Reticulocalbin-3	Q96D15	RCN3	NA	Inverse variance weighted	2	-0.028	0.080	7.298E-01	0.995	pan	SARS-CoV-2 infection
15644_1	Biotinidase	Biotinidase	P43251	BTD	NA	Inverse variance weighted	9	-0.003	0.010	7.300E-01	0.995	pan	SARS-CoV-2 infection
16763_11	LECT2	Leukocyte cell-derived chemotaxin-2	O14960	LECT2	NA	Inverse variance weighted	11	-0.004	0.012	7.302E-01	0.995	pan	SARS-CoV-2 infection
9738_7	FAF2	FAS-associated factor 2	Q96CS3	FAF2	NA	Wald ratio	1	-0.044	0.128	7.305E-01	0.995	pan	SARS-CoV-2 infection
3050_7	vWF	von Willebrand factor	P04275	VWF	NA	Inverse variance weighted	3	-0.019	0.056	7.312E-01	0.995	pan	SARS-CoV-2 infection
15370_5	BOLA1	BolA-like protein 1	Q9Y3E2	BOLA1	NA	Inverse variance weighted	14	-0.004	0.013	7.314E-01	0.995	pan	SARS-CoV-2 infection
13118_5	SMOC1	SPARC-related modular calcium-binding protein 1	Q9H4F8	SMOC1	NA	Inverse variance weighted	4	0.017	0.049	7.326E-01	0.995	pan	SARS-CoV-2 infection
17384_110	K6PF	ATP-dependent 6-phosphofructokinase, muscle type	P08237	PFKM	NA	Inverse variance weighted	3	-0.014	0.040	7.327E-01	0.995	pan	SARS-CoV-2 infection
17836_17	S100A16	Protein S100-A16	Q96FQ6	S100A16	NA	Wald ratio	1	-0.021	0.061	7.331E-01	0.995	pan	SARS-CoV-2 infection
4891_50	Glucagon	Glucagon	P01275	GCG	NA	Wald ratio	1	-0.034	0.101	7.335E-01	0.995	pan	SARS-CoV-2 infection
19153_53	MPPD2	Metallophosphoesterase MPPED2	Q15777	MPPED2	NA	Inverse variance weighted	2	0.033	0.096	7.340E-01	0.995	pan	SARS-CoV-2 infection
3283_21	BGH3	Transforming growth factor-beta-induced protein ig-h3	Q15582	TGFBI	NA	Inverse variance weighted	11	0.005	0.015	7.340E-01	0.995	pan	SARS-CoV-2 infection
3459_49	PDGF Rb	Platelet-derived growth factor receptor beta	P09619	PDGFRB	NA	Inverse variance weighted	13	-0.002	0.005	7.342E-01	0.995	pan	SARS-CoV-2 infection
4474_19	Ubiquitin	Ubiquitin	P62979	RPS27A	NA	Wald ratio	1	-0.042	0.125	7.353E-01	0.995	pan	SARS-CoV-2 infection
6606_61	KISS1	Metastasis-suppressor KiSS-1	Q15726	KISS1	NA	Inverse variance weighted	14	-0.004	0.013	7.353E-01	0.995	pan	SARS-CoV-2 infection
3077_66	Coagulation Factor Xa	Coagulation factor Xa	P00742	F10	NA	Inverse variance weighted	4	-0.017	0.050	7.368E-01	0.995	pan	SARS-CoV-2 infection
6471_53	FHR4	Complement factor H-related protein 4	Q92496	CFHR4	NA	Inverse variance weighted	19	-0.006	0.017	7.372E-01	0.995	pan	SARS-CoV-2 infection
12685_57	HOME2	Homer protein homolog 2	Q9NSB8	HOMER2	NA	Inverse variance weighted	2	-0.025	0.075	7.382E-01	0.995	pan	SARS-CoV-2 infection
8346_9	DPP2	Dipeptidyl peptidase 2	Q9UHL4	DPP7	NA	Inverse variance weighted	7	0.005	0.016	7.383E-01	0.995	pan	SARS-CoV-2 infection
7157_22	PCDA4	Protocadherin alpha-4	Q9UN74	PCDHA4	NA	Inverse variance weighted	12	-0.009	0.027	7.389E-01	0.995	pan	SARS-CoV-2 infection
6612_90	CP079	BRICHOS domain-containing protein 5	Q6PL45	BRICD5	NA	Inverse variance weighted	2	0.015	0.045	7.397E-01	0.995	pan	SARS-CoV-2 infection
2994_71	IL-1Rrp2	Interleukin-1 receptor-like 2	Q9HB29	IL1RL2	NA	Wald ratio	1	-0.012	0.035	7.400E-01	0.995	pan	SARS-CoV-2 infection
18337_4	GMDS	GDP-mannose 4,6 dehydratase	O60547	GMDS	NA	Wald ratio	1	-0.030	0.091	7.409E-01	0.995	pan	SARS-CoV-2 infection
17685_9	Apo A-IV	Apolipoprotein A-IV	P06727	APOA4	NA	Wald ratio	1	-0.033	0.099	7.413E-01	0.995	pan	SARS-CoV-2 infection
3365_7	Dkk-4	Dickkopf-related protein 4	Q9UBT3	DKK4	NA	Inverse variance weighted	4	0.031	0.094	7.413E-01	0.995	pan	SARS-CoV-2 infection
15556_49	Alpha-amylase 2B	Alpha-amylase 2B	P19961	AMY2B	NA	Inverse variance weighted	4	0.015	0.046	7.416E-01	0.995	pan	SARS-CoV-2 infection
11098_1	PDXK	Pyridoxal kinase	O00764	PDXK	NA	Inverse variance weighted	7	-0.008	0.024	7.428E-01	0.995	pan	SARS-CoV-2 infection
4961_17	annexin II	Annexin A2	P07355	ANXA2	NA	Inverse variance weighted	3	0.017	0.052	7.430E-01	0.995	pan	SARS-CoV-2 infection
14066_49	MAGI2	Membrane-associated guanylate kinase, WW and PDZ domain-containing protein 2	Q86UL8	MAGI2	NA	Inverse variance weighted	10	-0.005	0.015	7.432E-01	0.995	pan	SARS-CoV-2 infection
9177_6	FAM3B	Protein FAM3B	P58499	FAM3B	NA	Inverse variance weighted	7	-0.009	0.026	7.435E-01	0.995	pan	SARS-CoV-2 infection
4500_50	SCGF-alpha	Stem cell growth factor-alpha	Q9Y240	CLEC11A	NA	Inverse variance weighted	8	-0.005	0.014	7.440E-01	0.995	pan	SARS-CoV-2 infection
13465_5	CCP1	Calcipressin-1	P53805	RCAN1	NA	Wald ratio	1	0.026	0.079	7.440E-01	0.995	pan	SARS-CoV-2 infection
12663_1	THTR	Thiosulfate sulfurtransferase	Q16762	TST	NA	Inverse variance weighted	15	0.006	0.017	7.443E-01	0.995	pan	SARS-CoV-2 infection
12635_9	TRDMT	tRNA (cytosine(38)-C(5))-methyltransferase	O14717	TRDMT1	NA	Inverse variance weighted	4	-0.011	0.033	7.445E-01	0.995	pan	SARS-CoV-2 infection
9296_15	PTPRD	Receptor-type tyrosine-protein phosphatase delta	P23468	PTPRD	NA	Wald ratio	1	0.033	0.101	7.447E-01	0.995	pan	SARS-CoV-2 infection
8356_88	NEU1	Oxytocin-neurophysin 1	P01178	OXT	NA	Inverse variance weighted	4	0.009	0.027	7.448E-01	0.995	pan	SARS-CoV-2 infection
7228_2	SIA7F	Alpha-N-acetylgalactosaminide alpha-2,6-sialyltransferase 6	Q969X2	ST6GALNAC6	NA	Inverse variance weighted	3	-0.020	0.063	7.451E-01	0.995	pan	SARS-CoV-2 infection
17231_1	L-plastin	Plastin-2	P13796	LCP1	NA	Wald ratio	1	-0.027	0.084	7.464E-01	0.995	pan	SARS-CoV-2 infection
17224_12	MIME	Mimecan	P20774	OGN	NA	Inverse variance weighted	6	-0.010	0.032	7.467E-01	0.995	pan	SARS-CoV-2 infection
15476_6	REG3G	Regenerating islet-derived protein 3-gamma	Q6UW15	REG3G	NA	Inverse variance weighted	17	0.004	0.011	7.469E-01	0.995	pan	SARS-CoV-2 infection
15298_199	NETO1	Neuropilin and tolloid-like protein 1	Q8TDF5	NETO1	NA	Inverse variance weighted	2	0.022	0.068	7.471E-01	0.995	pan	SARS-CoV-2 infection
3292_75	CD48	CD48 antigen	P09326	CD48	NA	Inverse variance weighted	9	-0.013	0.041	7.481E-01	0.995	pan	SARS-CoV-2 infection
9590_10	COX7R	Cytochrome c oxidase subunit 7A-related protein, mitochondrial	O14548	COX7A2L	NA	Wald ratio	1	0.034	0.107	7.482E-01	0.995	pan	SARS-CoV-2 infection
3172_28	ARSB	Arylsulfatase B	P15848	ARSB	NA	Inverse variance weighted	2	-0.011	0.033	7.494E-01	0.995	pan	SARS-CoV-2 infection
5584_21	Holo-TC II	Holo-Transcobalamin-2	P20062	TCN2	NA	Inverse variance weighted	12	0.007	0.020	7.495E-01	0.995	pan	SARS-CoV-2 infection
12563_2	TFIP8	Tumor necrosis factor alpha-induced protein 8	O95379	TNFAIP8	NA	Inverse variance weighted	2	0.009	0.030	7.496E-01	0.995	pan	SARS-CoV-2 infection
18172_71	ASF1A	Histone chaperone ASF1A	Q9Y294	ASF1A	NA	Inverse variance weighted	2	0.013	0.040	7.503E-01	0.995	pan	SARS-CoV-2 infection
12993_21	NPL4	Nuclear protein localization protein 4 homolog	Q8TAT6	NPLOC4	NA	Wald ratio	1	-0.050	0.157	7.505E-01	0.995	pan	SARS-CoV-2 infection
12731_12	PKHA7	Pleckstrin homology domain-containing family A member 7	Q6IQ23	PLEKHA7	NA	Inverse variance weighted	8	-0.005	0.015	7.513E-01	0.995	pan	SARS-CoV-2 infection
4989_7	Fibrinogen g-chain dimer	Fibrinogen gamma chain	P02679	FGG	NA	Wald ratio	1	0.022	0.069	7.520E-01	0.995	pan	SARS-CoV-2 infection
5066_134	CLM6	CMRF35-like molecule 6	Q08708	CD300C	NA	Inverse variance weighted	13	-0.009	0.028	7.523E-01	0.995	pan	SARS-CoV-2 infection
13392_13	AT1B1	Sodium/potassium-transporting ATPase subunit beta-1	P05026	ATP1B1	NA	Inverse variance weighted	5	-0.009	0.028	7.524E-01	0.995	pan	SARS-CoV-2 infection
13634_209	PIR	Pirin	O00625	PIR	NA	Wald ratio	1	0.030	0.096	7.526E-01	0.995	pan	SARS-CoV-2 infection
19392_6	DDAH1	N(G),N(G)-dimethylarginine dimethylaminohydrolase 1	O94760	DDAH1	NA	Inverse variance weighted	12	0.005	0.015	7.533E-01	0.995	pan	SARS-CoV-2 infection
9359_9	EGFL9	Protein delta homolog 2	Q6UY11	DLK2	NA	Wald ratio	1	-0.009	0.029	7.539E-01	0.995	pan	SARS-CoV-2 infection
8229_1	GXLT1	Glucoside xylosyltransferase 1	Q4G148	GXYLT1	NA	Inverse variance weighted	11	-0.004	0.012	7.549E-01	0.995	pan	SARS-CoV-2 infection
18162_167	IRAK4	Interleukin-1 receptor-associated kinase 4	Q9NWZ3	IRAK4	NA	Wald ratio	1	-0.020	0.063	7.557E-01	0.995	pan	SARS-CoV-2 infection
7049_2	ADAM 23	Disintegrin and metalloproteinase domain-containing protein 23	O75077	ADAM23	NA	Inverse variance weighted	14	-0.003	0.010	7.561E-01	0.995	pan	SARS-CoV-2 infection
12396_19	HIBCH	3-hydroxyisobutyryl-CoA hydrolase, mitochondrial	Q6NVY1	HIBCH	NA	Inverse variance weighted	6	-0.004	0.014	7.565E-01	0.995	pan	SARS-CoV-2 infection
7994_41	ERO1B	ERO1-like protein beta	Q86YB8	ERO1B	NA	Inverse variance weighted	2	-0.010	0.031	7.567E-01	0.995	pan	SARS-CoV-2 infection
18218_48	CB032	CB1 cannabinoid receptor-interacting protein 1	Q96F85	CNRIP1	NA	Inverse variance weighted	10	0.004	0.014	7.573E-01	0.995	pan	SARS-CoV-2 infection
2635_61	Layilin	Layilin	Q6UX15	LAYN	NA	Wald ratio	1	-0.028	0.090	7.573E-01	0.995	pan	SARS-CoV-2 infection
7206_20	F16P1	Fructose-1,6-bisphosphatase 1	P09467	FBP1	NA	Inverse variance weighted	2	0.024	0.077	7.578E-01	0.995	pan	SARS-CoV-2 infection
19154_41	Protease nexin I	Glia-derived nexin	P07093	SERPINE2	NA	Inverse variance weighted	13	-0.004	0.013	7.580E-01	0.995	pan	SARS-CoV-2 infection
8351_17	PRS57	Serine protease 57	Q6UWY2	PRSS57	NA	Inverse variance weighted	7	-0.006	0.019	7.589E-01	0.995	pan	SARS-CoV-2 infection
17737_7	IVD	Isovaleryl-CoA dehydrogenase, mitochondrial	P26440	IVD	NA	Inverse variance weighted	3	-0.015	0.049	7.591E-01	0.995	pan	SARS-CoV-2 infection
19259_176	IF2A	Eukaryotic translation initiation factor 2 subunit 1	P05198	EIF2S1	NA	Wald ratio	1	0.023	0.077	7.601E-01	0.995	pan	SARS-CoV-2 infection
5605_77	MFNG	Beta-1,3-N-acetylglucosaminyltransferase manic fringe	O00587	MFNG	NA	Inverse variance weighted	2	0.030	0.099	7.602E-01	0.995	pan	SARS-CoV-2 infection
3396_54	Renin	Renin	P00797	REN	NA	Inverse variance weighted	7	-0.011	0.036	7.605E-01	0.995	pan	SARS-CoV-2 infection
7928_183	TPST1	Protein-tyrosine sulfotransferase 1	O60507	TPST1	NA	Inverse variance weighted	11	-0.004	0.014	7.619E-01	0.995	pan	SARS-CoV-2 infection
5103_30	MO2R1:ECD	Cell surface glycoprotein CD200 receptor 1:Isoform 4, Extracellular Domain	Q8TD46	CD200R1	NA	Wald ratio	1	0.013	0.044	7.620E-01	0.995	pan	SARS-CoV-2 infection
5736_1	TRML2	Trem-like transcript 2 protein	Q5T2D2	TREML2	NA	Inverse variance weighted	5	0.004	0.013	7.628E-01	0.995	pan	SARS-CoV-2 infection
4232_19	IGF-I sR	Insulin-like growth factor 1 receptor	P08069	IGF1R	NA	Inverse variance weighted	2	0.013	0.042	7.631E-01	0.995	pan	SARS-CoV-2 infection
8221_19	MIF	Macrophage migration inhibitory factor	P14174	MIF	NA	Wald ratio	1	0.031	0.103	7.633E-01	0.995	pan	SARS-CoV-2 infection

9383_24	CH3L2	Chitinase-3-like protein 2	Q15782	CHI3L2	NA	Wald ratio	1	-0.025	0.082	7.633E-01	0.995	pan	SARS-CoV-2 infection
16055_3	complement factor H-related 5	Complement factor H-related protein 5	Q9BXR6	CFHR5	NA	Inverse variance weighted	6	-0.012	0.040	7.641E-01	0.995	pan	SARS-CoV-2 infection
16908_5	OMGP	Oligodendrocyte-myelin glycoprotein	P23515	OMG	NA	Inverse variance weighted	5	0.019	0.063	7.641E-01	0.995	pan	SARS-CoV-2 infection
8993_151	RIPK2	Receptor-interacting serine/threonine-protein kinase 2	O43353	RIPK2	NA	Wald ratio	1	0.020	0.067	7.646E-01	0.995	pan	SARS-CoV-2 infection
18275_5	CRIP1	Cysteine-rich protein 1	P50238	CRIP1	NA	Inverse variance weighted	11	-0.008	0.026	7.646E-01	0.995	pan	SARS-CoV-2 infection
11377_19	ADH7	Alcohol dehydrogenase class 4 mu/sigma chain	P40394	ADH7	NA	Inverse variance weighted	3	-0.005	0.017	7.657E-01	0.995	pan	SARS-CoV-2 infection
2278_61	TIMP-2	Metalloproteinase inhibitor 2	P16035	TIMP2	NA	Wald ratio	1	0.021	0.071	7.660E-01	0.995	pan	SARS-CoV-2 infection
13122_19	FLRT2	Leucine-rich repeat transmembrane protein FLRT2	O43155	FLRT2	NA	Inverse variance weighted	7	0.004	0.015	7.661E-01	0.995	pan	SARS-CoV-2 infection
11313_100	PHS	Pterin-4-alpha-carbinolamine dehydratase	P61457	PCBD1	NA	Inverse variance weighted	6	-0.012	0.040	7.664E-01	0.995	pan	SARS-CoV-2 infection
17683_2	COMD9	COMM domain-containing protein 9	Q9P000	COMMD9	NA	Wald ratio	1	-0.015	0.049	7.669E-01	0.995	pan	SARS-CoV-2 infection
15559_5	ANTR2	Anthrax toxin receptor 2	P58335	ANTXR2	NA	Inverse variance weighted	5	-0.009	0.032	7.671E-01	0.995	pan	SARS-CoV-2 infection
9470_15	MET24	Methyltransferase-like protein 24	Q5JXM2	METTL24	NA	Inverse variance weighted	5	-0.012	0.040	7.686E-01	0.995	pan	SARS-CoV-2 infection
16304_6	LGR4	Leucine-rich repeat-containing G-protein coupled receptor 4	Q9BXB1	LGR4	NA	Inverse variance weighted	3	0.013	0.044	7.689E-01	0.995	pan	SARS-CoV-2 infection
17781_191	MLP3A	Microtubule-associated proteins 1A/1B light chain 3A	Q9H492	MAP1LC3A	NA	Inverse variance weighted	8	-0.014	0.046	7.691E-01	0.995	pan	SARS-CoV-2 infection
8394_56	RNase 2	Non-secretory ribonuclease	P10153	RNASE2	NA	Wald ratio	1	-0.018	0.060	7.691E-01	0.995	pan	SARS-CoV-2 infection
15435_4	PNP	Purine nucleoside phosphorylase	P00491	PNP	NA	Inverse variance weighted	2	-0.009	0.032	7.700E-01	0.995	pan	SARS-CoV-2 infection
9102_28	ZFY27:CD region 2	Protrudin:Cytoplasmic domain, region 2, isoform 5	Q5T4F4	ZFYVE27	NA	Wald ratio	1	0.024	0.082	7.704E-01	0.995	pan	SARS-CoV-2 infection
3325_2	MATN2	Matrilin-2	O00339	MATN2	NA	Inverse variance weighted	4	0.009	0.032	7.712E-01	0.995	pan	SARS-CoV-2 infection
3033_57	Galectin-2	Galectin-2	P05162	LGALS2	NA	Inverse variance weighted	3	0.007	0.025	7.716E-01	0.995	pan	SARS-CoV-2 infection
6388_21	CC126	Coiled-coil domain-containing protein 126	Q96EE4	CCDC126	NA	Inverse variance weighted	6	0.006	0.022	7.719E-01	0.995	pan	SARS-CoV-2 infection
5346_24	CPNE1:C2, 1 and 2	Copine-1:Ca2+-dependent membrane-targeting module domains 1 and 2	Q99829	CPNE1	NA	Inverse variance weighted	12	0.003	0.011	7.720E-01	0.995	pan	SARS-CoV-2 infection
11493_169	DYL2	Dynein light chain 2, cytoplasmic	Q96FJ2	DYNLL2	NA	Wald ratio	1	-0.027	0.092	7.725E-01	0.995	pan	SARS-CoV-2 infection
5242_37	MP2K4	Dual specificity mitogen-activated protein kinase kinase 4	P45985	MAP2K4	NA	Inverse variance weighted	6	-0.011	0.037	7.729E-01	0.995	pan	SARS-CoV-2 infection
18396_10	AES	Amino-terminal enhancer of split	Q08117	TLE5	NA	Wald ratio	1	-0.028	0.097	7.733E-01	0.995	pan	SARS-CoV-2 infection
13740_51	sFRP-3	Secreted frizzled-related protein 3	Q92765	FRZB	NA	Inverse variance weighted	10	-0.003	0.009	7.735E-01	0.995	pan	SARS-CoV-2 infection
17164_15	annexin IV	Annexin A4	P09525	ANXA4	NA	Inverse variance weighted	2	0.025	0.088	7.737E-01	0.995	pan	SARS-CoV-2 infection
18397_5	AK1C4	Aldo-keto reductase family 1 member C4	P17516	AKR1C4	NA	Inverse variance weighted	11	0.004	0.014	7.738E-01	0.995	pan	SARS-CoV-2 infection
13610_9	MAGE-10	Melanoma-associated antigen 10	P43363	MAGEA10	NA	Inverse variance weighted	2	-0.021	0.074	7.742E-01	0.995	pan	SARS-CoV-2 infection
13659_36	AT131	Manganese-transporting ATPase 13A1	Q9HD20	ATP13A1	NA	Inverse variance weighted	2	0.021	0.072	7.747E-01	0.995	pan	SARS-CoV-2 infection
19323_1	RCAS1	Receptor-binding cancer antigen expressed on SiSo cells	O00559	EBAG9	NA	Inverse variance weighted	3	0.020	0.069	7.750E-01	0.995	pan	SARS-CoV-2 infection
14273_19	PPCE	Prolyl endopeptidase	P48147	PREP	NA	Inverse variance weighted	3	-0.009	0.030	7.763E-01	0.995	pan	SARS-CoV-2 infection
19446_1	GMPR2	GMP reductase 2	Q9P2T1	GMPR2	NA	Inverse variance weighted	3	0.003	0.010	7.764E-01	0.995	pan	SARS-CoV-2 infection
7218_87	AT1B2	Sodium/potassium-transporting ATPase subunit beta-2	P14415	ATP1B2	NA	Inverse variance weighted	4	0.010	0.034	7.766E-01	0.995	pan	SARS-CoV-2 infection
11910_27	DLX4	Homeobox protein DLX-4	Q92988	DLX4	NA	Inverse variance weighted	2	-0.013	0.047	7.775E-01	0.995	pan	SARS-CoV-2 infection
18864_7	TRY3	Trypsin-3	P35030	PRSS3	NA	Inverse variance weighted	29	0.003	0.009	7.776E-01	0.995	pan	SARS-CoV-2 infection
11421_10	EHD4	EH domain-containing protein 4	Q9H223	EHD4	NA	Wald ratio	1	0.013	0.048	7.783E-01	0.995	pan	SARS-CoV-2 infection
11606_22	DNJB6	DnaJ homolog subfamily B member 6	O75190	DNAJB6	NA	Wald ratio	1	0.029	0.103	7.790E-01	0.995	pan	SARS-CoV-2 infection
18330_7	PGM2	Phosphoglucomutase-2	Q96G03	PGM2	NA	Inverse variance weighted	7	-0.008	0.028	7.800E-01	0.995	pan	SARS-CoV-2 infection
15573_110	CSPG3	Neurocan core protein	O14594	NCAN	NA	Inverse variance weighted	11	0.007	0.024	7.801E-01	0.995	pan	SARS-CoV-2 infection
9765_4	NDE1	Nuclear distribution protein nudE homolog 1	Q9NXR1	NDE1	NA	Wald ratio	1	0.019	0.069	7.803E-01	0.995	pan	SARS-CoV-2 infection
12684_5	ADSV	Adseverin	Q9Y6U3	SCIN	NA	Wald ratio	1	0.019	0.067	7.808E-01	0.995	pan	SARS-CoV-2 infection
10627_87	APLP2	Amyloid-like protein 2	Q06481	APLP2	NA	Inverse variance weighted	2	-0.009	0.034	7.808E-01	0.995	pan	SARS-CoV-2 infection
9369_174	NGL1	Leucine-rich repeat-containing protein 4C	Q9HCJ2	LRRC4C	NA	Inverse variance weighted	4	0.007	0.027	7.808E-01	0.995	pan	SARS-CoV-2 infection
10045_47	CUL3	Cullin-3	Q13618	CUL3	NA	Wald ratio	1	0.016	0.057	7.814E-01	0.995	pan	SARS-CoV-2 infection
14636_25	Ribonuclease UK114	Ribonuclease UK114	P52758	RIDA	NA	Inverse variance weighted	4	-0.005	0.017	7.819E-01	0.995	pan	SARS-CoV-2 infection
6321_65	PCDGA	Protocadherin gamma-A10	Q9Y5H3	PCDHGA10	NA	Inverse variance weighted	3	0.014	0.051	7.826E-01	0.995	pan	SARS-CoV-2 infection
15395_15	GST M1-1	Glutathione S-transferase Mu 1	P09488	GSTM1	NA	Inverse variance weighted	9	-0.008	0.030	7.830E-01	0.995	pan	SARS-CoV-2 infection
5704_74	Granzyme M	Granzyme M	P51124	GZMM	NA	Inverse variance weighted	2	0.027	0.099	7.832E-01	0.995	pan	SARS-CoV-2 infection
10902_53	APRV1	Retroviral-like aspartic protease 1	Q53RT3	ASPRV1	NA	Inverse variance weighted	4	0.014	0.052	7.832E-01	0.995	pan	SARS-CoV-2 infection
8296_117	KDEL2	KDEL motif-containing protein 2	Q7Z4H8	POGLUT3	NA	Inverse variance weighted	8	0.004	0.015	7.835E-01	0.995	pan	SARS-CoV-2 infection
9906_21	TICN3	Testican-3	Q9BQ16	SPOCK3	NA	Inverse variance weighted	12	-0.003	0.010	7.836E-01	0.995	pan	SARS-CoV-2 infection
13464_8	NRAC	Nutritionally-regulated adipose and cardiac enriched protein homolog	Q8N912	NRAC	NA	Inverse variance weighted	3	-0.017	0.061	7.836E-01	0.995	pan	SARS-CoV-2 infection
17785_11	RCL	2'-deoxynucleoside 5'-phosphate N-hydrolase 1	O43598	DNPH1	NA	Wald ratio	1	-0.016	0.060	7.836E-01	0.995	pan	SARS-CoV-2 infection
12020_39	PMGE	Bisphosphoglycerate mutase	P07738	BPGM	NA	Wald ratio	1	0.034	0.125	7.837E-01	0.995	pan	SARS-CoV-2 infection
12867_40	DYLT3	Dynein light chain Tctex-type 3	P51808	DYNLT3	NA	Wald ratio	1	-0.031	0.114	7.840E-01	0.995	pan	SARS-CoV-2 infection
2948_58	Growth hormone receptor	Growth hormone receptor	P10912	GHR	NA	Inverse variance weighted	4	-0.008	0.028	7.844E-01	0.995	pan	SARS-CoV-2 infection
4588_1	PH	Pancreatic hormone	P01298	PPY	NA	Inverse variance weighted	9	-0.006	0.023	7.847E-01	0.995	pan	SARS-CoV-2 infection
9018_38	PCD10:ECD	Protocadherin-10:Extracellular domain	Q9P2E7	PCDH10	NA	Inverse variance weighted	3	-0.016	0.058	7.854E-01	0.995	pan	SARS-CoV-2 infection
19145_4	MREG	Melanoregulin	Q8N565	MREG	NA	Inverse variance weighted	3	-0.012	0.046	7.855E-01	0.995	pan	SARS-CoV-2 infection
13704_5	HMCS2	Hydroxymethylglutaryl-CoA synthase, mitochondrial	P54868	HMGCS2	NA	Inverse variance weighted	2	-0.017	0.062	7.862E-01	0.995	pan	SARS-CoV-2 infection
18882_7	CSTN2	Calsyntenin-2	Q9H4D0	CLSTN2	NA	Inverse variance weighted	10	-0.004	0.017	7.865E-01	0.995	pan	SARS-CoV-2 infection
4156_74	TGF-b2	Transforming growth factor beta-2	P61812	TGFB2	NA	Wald ratio	1	0.030	0.112	7.872E-01	0.995	pan	SARS-CoV-2 infection
10569_28	MFAP2	Microfibrillar-associated protein 2	P55001	MFAP2	NA	Inverse variance weighted	4	0.025	0.094	7.888E-01	0.995	pan	SARS-CoV-2 infection
9526_3	COLL1	Colipase-like protein 1	A2RUU4	CLPSL1	NA	Inverse variance weighted	6	-0.009	0.033	7.909E-01	0.995	pan	SARS-CoV-2 infection
16583_8	AP4A	Bis(5'-nucleosyl)-tetraphosphatase [asymmetrical]	P50583	NUDT2	NA	Inverse variance weighted	8	0.007	0.025	7.910E-01	0.995	pan	SARS-CoV-2 infection
5363_51	Semaphorin 3E	Semaphorin-3E	O15041	SEMA3E	NA	Inverse variance weighted	24	0.002	0.008	7.910E-01	0.995	pan	SARS-CoV-2 infection
5134_52	TIMD3	Hepatitis A virus cellular receptor 2	Q8TDQ0	HAVCR2	NA	Inverse variance weighted	9	0.004	0.014	7.910E-01	0.995	pan	SARS-CoV-2 infection
3719_2	p27Kip1	Cyclin-dependent kinase inhibitor 1B	P46527	CDKN1B	NA	Inverse variance weighted	2	-0.014	0.051	7.913E-01	0.995	pan	SARS-CoV-2 infection
8275_31	PEAR1:ECD	Platelet endothelial aggregation receptor 1:Extracellular domain	Q5VY43	PEAR1	NA	Inverse variance weighted	3	-0.010	0.040	7.914E-01	0.995	pan	SARS-CoV-2 infection
2797_56	Apo B	Apolipoprotein B	P04114	APOB	NA	Wald ratio	1	0.027	0.101	7.921E-01	0.995	pan	SARS-CoV-2 infection
18422_41	ERG19	Diphosphomevalonate decarboxylase	P53602	MVD	NA	Wald ratio	1	-0.020	0.076	7.921E-01	0.995	pan	SARS-CoV-2 infection
5275_28	VAV	Proto-oncogene vav	P15498	VAV1	NA	Wald ratio	1	-0.022	0.084	7.924E-01	0.995	pan	SARS-CoV-2 infection
19556_12	Complement receptor type 1	Complement receptor type 1	P17927	CR1	NA	Inverse variance weighted	11	-0.005	0.018	7.940E-01	0.995	pan	SARS-CoV-2 infection
16856_79	MARE2	Microtubule-associated protein RP/EB family member 2	Q15555	MAPRE2	NA	Inverse variance weighted	2	0.015	0.059	7.941E-01	0.995	pan	SARS-CoV-2 infection
3041_55	MRC2	C-type mannose receptor 2	Q9UBG0	MRC2	NA	Inverse variance weighted	6	-0.011	0.041	7.944E-01	0.995	pan	SARS-CoV-2 infection
9191_8	Trefoil factor 2	Trefoil factor 2	Q03403	TFF2	NA	Inverse variance weighted	4	-0.012	0.045	7.945E-01	0.995	pan	SARS-CoV-2 infection
3284_75	BGN	Biglycan	P21810	BGN	NA	Inverse variance weighted	14	0.004	0.015	7.946E-01	0.995	pan	SARS-CoV-2 infection
12522_6	RD23B	UV excision repair protein RAD23 homolog B	P54727	RAD23B	NA	Wald ratio	1	-0.023	0.087	7.954E-01	0.995	pan	SARS-CoV-2 infection

8427_118	RSPO3	R-spondin-3	Q9BXY4	RSPO3	NA	Inverse variance weighted	4	0.014	0.055	7.959E-01	0.995	pan	SARS-CoV-2 infection
13488_3	ARFP1	Arfaptin-1	P53367	ARFIP1	NA	Inverse variance weighted	3	-0.011	0.042	7.962E-01	0.995	pan	SARS-CoV-2 infection
6472_40	GP100	Melanocyte protein PMEL	P40967	PMEL	NA	Inverse variance weighted	13	-0.005	0.018	7.963E-01	0.995	pan	SARS-CoV-2 infection
3727_35	PYY	Peptide YY	P10082	PYY	NA	Inverse variance weighted	4	-0.016	0.062	7.964E-01	0.995	pan	SARS-CoV-2 infection
19161_1	UBP15	Ubiquitin carboxyl-terminal hydrolase 15	Q9Y4E8	USP15	NA	Inverse variance weighted	2	0.009	0.034	7.965E-01	0.995	pan	SARS-CoV-2 infection
13447_42	SPRN	Shadow of prion protein	Q5BIV9	SPRN	NA	Inverse variance weighted	2	-0.052	0.203	7.966E-01	0.995	pan	SARS-CoV-2 infection
19236_24	TCP4	Activated RNA polymerase II transcriptional coactivator p15	P53999	SUB1	NA	Wald ratio	1	-0.026	0.100	7.968E-01	0.995	pan	SARS-CoV-2 infection
5703_26	NOE1	Noelin	Q99784	OLFM1	NA	Inverse variance weighted	3	0.010	0.039	7.973E-01	0.995	pan	SARS-CoV-2 infection
11128_29	T132C:CD	Transmembrane protein 132C:Cytoplasmic domain	Q8N3T6	TMEM132C	NA	Wald ratio	1	0.029	0.112	7.976E-01	0.995	pan	SARS-CoV-2 infection
7050_5	NEGR1	Neuronal growth regulator 1	Q7Z3B1	NEGR1	NA	Wald ratio	1	-0.027	0.107	7.985E-01	0.995	pan	SARS-CoV-2 infection
13657_2	PNKP	Bifunctional polynucleotide phosphatase/kinase	Q96T60	PNKP	NA	Wald ratio	1	0.012	0.047	7.992E-01	0.995	pan	SARS-CoV-2 infection
10366_11	PDGFRA	Platelet-derived growth factor receptor alpha	P16234	PDGFRA	NA	Inverse variance weighted	10	0.007	0.028	7.992E-01	0.995	pan	SARS-CoV-2 infection
17782_23	THIK	3-ketoacyl-CoA thiolase, peroxisomal	P09110	ACAA1	NA	Inverse variance weighted	8	-0.005	0.021	8.003E-01	0.995	pan	SARS-CoV-2 infection
3581_53	a2-HS-Glycoprotein	Alpha-2-HS-glycoprotein	P02765	AHSG	NA	Inverse variance weighted	4	-0.011	0.042	8.007E-01	0.995	pan	SARS-CoV-2 infection
13929_27	OCTC	Peroxisomal carnitine O-octanoyltransferase	Q9UKG9	CROT	NA	Inverse variance weighted	9	0.004	0.015	8.013E-01	0.995	pan	SARS-CoV-2 infection
10396_6	Mcl-1	Induced myeloid leukemia cell differentiation protein Mcl-1	Q07820	MCL1	NA	Inverse variance weighted	2	-0.018	0.073	8.030E-01	0.995	pan	SARS-CoV-2 infection
15499_11	Attractin	Attractin	O75882	ATRN	NA	Inverse variance weighted	5	0.007	0.027	8.041E-01	0.995	pan	SARS-CoV-2 infection
5430_66	SHPS1	Tyrosine-protein phosphatase non-receptor type substrate 1	P78324	SIRPA	NA	Inverse variance weighted	9	0.003	0.012	8.041E-01	0.995	pan	SARS-CoV-2 infection
16781_2	ENASE	Cytosolic endo-beta-N-acetylglucosaminidase	Q8NFI3	ENGASE	NA	Inverse variance weighted	11	-0.002	0.009	8.041E-01	0.995	pan	SARS-CoV-2 infection
3235_50	WFKN2	WAP, Kazal, immunoglobulin, Kunitz and NTR domain-containing protein 2	Q8TEU8	WFIKKN2	NA	Inverse variance weighted	8	0.003	0.012	8.044E-01	0.995	pan	SARS-CoV-2 infection
9357_4	CREG1	Protein CREG1	O75629	CREG1	NA	Inverse variance weighted	10	0.002	0.010	8.050E-01	0.995	pan	SARS-CoV-2 infection
5028_59	sCD163	Scavenger receptor cysteine-rich type 1 protein M130	Q86VB7	CD163	NA	Inverse variance weighted	13	-0.007	0.028	8.052E-01	0.995	pan	SARS-CoV-2 infection
15579_26	ENPP6	Ectonucleotide pyrophosphatase/phosphodiesterase family member 6	Q6UWR7	ENPP6	NA	Inverse variance weighted	2	0.016	0.063	8.054E-01	0.995	pan	SARS-CoV-2 infection
15523_9	HEM2	Delta-aminolevulinic acid dehydratase	P13716	ALAD	NA	Inverse variance weighted	8	-0.006	0.023	8.058E-01	0.995	pan	SARS-CoV-2 infection
15417_3	Maspin	Serpin B5	P36952	SERPINB5	NA	Wald ratio	1	-0.009	0.036	8.058E-01	0.995	pan	SARS-CoV-2 infection
6257_56	CGRE1	Cell growth regulator with EF hand domain protein 1	Q99674	CGREF1	NA	Inverse variance weighted	4	0.013	0.055	8.059E-01	0.995	pan	SARS-CoV-2 infection
18434_141	TBPL1	TATA box-binding protein-like protein 1	P62380	TBPL1	NA	Inverse variance weighted	3	0.014	0.057	8.060E-01	0.995	pan	SARS-CoV-2 infection
19335_2	HN1	Hematological and neurological expressed 1 protein	Q9UK76	JPT1	NA	Wald ratio	1	-0.026	0.107	8.066E-01	0.995	pan	SARS-CoV-2 infection
17140_57	PDGFD	Platelet-derived growth factor D	Q9GZP0	PDGFD	NA	Inverse variance weighted	5	-0.011	0.044	8.067E-01	0.995	pan	SARS-CoV-2 infection
11120_49	NAT14	N-acetyltransferase 14	Q8WUY8	NAT14	NA	Wald ratio	1	0.011	0.046	8.070E-01	0.995	pan	SARS-CoV-2 infection
7019_13	Semaphorin-7A	Semaphorin-7A	O75326	SEMA7A	NA	Inverse variance weighted	2	-0.013	0.052	8.077E-01	0.995	pan	SARS-CoV-2 infection
3054_3	Haptoglobin, Mixed Type	Haptoglobin	P00738	HP	NA	Inverse variance weighted	10	0.010	0.041	8.080E-01	0.995	pan	SARS-CoV-2 infection
4154_57	P-Selectin	P-selectin	P16109	SELP	NA	Inverse variance weighted	4	-0.007	0.027	8.081E-01	0.995	pan	SARS-CoV-2 infection
17837_5	CLIC2	Chloride intracellular channel protein 2	O15247	CLIC2	NA	Wald ratio	1	-0.024	0.098	8.082E-01	0.995	pan	SARS-CoV-2 infection
16823_75	APOL3	Apolipoprotein L3	O95236	APOL3	NA	Inverse variance weighted	8	-0.003	0.011	8.084E-01	0.995	pan	SARS-CoV-2 infection
2706_69	Thyroxine-Binding Globulin	Thyroxine-binding globulin	P05543	SERPINA7	NA	Inverse variance weighted	2	0.017	0.071	8.086E-01	0.995	pan	SARS-CoV-2 infection
5060_62	B7-H1	Programmed cell death 1 ligand 1	Q9NZQ7	CD274	NA	Inverse variance weighted	2	0.005	0.021	8.092E-01	0.995	pan	SARS-CoV-2 infection
10082_251	NFL	Neurofilament light polypeptide	P07196	NEFL	NA	Inverse variance weighted	3	-0.016	0.066	8.100E-01	0.995	pan	SARS-CoV-2 infection
15667_39	BMP-4	Bone morphogenetic protein 4	P12644	BMP4	NA	Inverse variance weighted	3	0.011	0.048	8.102E-01	0.995	pan	SARS-CoV-2 infection
9896_21	FOXGB	Forkhead box protein G1	P55316	FOXG1	NA	Inverse variance weighted	2	-0.031	0.129	8.110E-01	0.995	pan	SARS-CoV-2 infection
8839_4	Metaxin-2	Metaxin-2	O75431	MTX2	NA	Wald ratio	1	-0.025	0.105	8.116E-01	0.995	pan	SARS-CoV-2 infection
18381_16	ALDH-E2	Aldehyde dehydrogenase, mitochondrial	P05091	ALDH2	NA	Inverse variance weighted	4	0.009	0.037	8.117E-01	0.995	pan	SARS-CoV-2 infection
17791_25	HPCL1	Hippocalcin-like protein 1	P37235	HPCAL1	NA	Wald ratio	1	-0.015	0.064	8.119E-01	0.995	pan	SARS-CoV-2 infection
4187_49	6-Phosphogluconate dehydrogenase	6-phosphogluconate dehydrogenase, decarboxylating	P52209	PGD	NA	Wald ratio	1	0.015	0.065	8.124E-01	0.995	pan	SARS-CoV-2 infection
18242_8	Sperm protein 17	Sperm surface protein Sp17	Q15506	SPA17	NA	Wald ratio	1	-0.023	0.098	8.125E-01	0.995	pan	SARS-CoV-2 infection
3898_5	PSD7	26S proteasome non-ATPase regulatory subunit 7	P51665	PSMD7	NA	Wald ratio	1	0.023	0.098	8.128E-01	0.995	pan	SARS-CoV-2 infection
5728_60	FCRL1	Fc receptor-like protein 1	Q96LA6	FCRL1	NA	Inverse variance weighted	10	-0.006	0.024	8.134E-01	0.995	pan	SARS-CoV-2 infection
8231_122	VEGF sR1	Vascular endothelial growth factor receptor 1	P17948	FLT1	NA	Inverse variance weighted	4	-0.011	0.047	8.140E-01	0.995	pan	SARS-CoV-2 infection
12727_7	FPRP	Prostaglandin F2 receptor negative regulator	Q9P2B2	PTGFRN	NA	Inverse variance weighted	14	-0.003	0.013	8.143E-01	0.995	pan	SARS-CoV-2 infection
15545_13	Calcineurin B a	Calcineurin subunit B type 1	P63098	PPP3R1	NA	Wald ratio	1	-0.019	0.081	8.151E-01	0.995	pan	SARS-CoV-2 infection
5708_1	LEAP2	Liver-expressed antimicrobial peptide 2	Q969E1	LEAP2	NA	Inverse variance weighted	6	-0.004	0.017	8.158E-01	0.995	pan	SARS-CoV-2 infection
12384_92	CSN7B	COP9 signalosome complex subunit 7b	Q9H9Q2	COPS7B	NA	Inverse variance weighted	4	0.015	0.064	8.162E-01	0.995	pan	SARS-CoV-2 infection
19237_17	D-dopachrome decarboxylase	D-dopachrome decarboxylase	P30046	DDT	NA	Inverse variance weighted	8	0.007	0.031	8.169E-01	0.995	pan	SARS-CoV-2 infection
18215_5	THG1	Probable tRNA(His) guanylyltransferase	Q9NWX6	THG1L	NA	Inverse variance weighted	8	-0.003	0.011	8.174E-01	0.995	pan	SARS-CoV-2 infection
8925_25	RIR2B	Ribonucleoside-diphosphate reductase subunit M2 B	Q7LG56	RRM2B	NA	Inverse variance weighted	2	-0.015	0.065	8.174E-01	0.995	pan	SARS-CoV-2 infection
17799_9	6PGL	6-phosphogluconolactonase	O95336	PGLS	NA	Inverse variance weighted	4	0.009	0.038	8.177E-01	0.995	pan	SARS-CoV-2 infection
4908_6	Endoglin	Endoglin	P17813	ENG	NA	Inverse variance weighted	8	-0.002	0.011	8.177E-01	0.995	pan	SARS-CoV-2 infection
19113_66	SH3L2	SH3 domain-binding glutamic acid-rich-like protein 2	Q9UJC5	SH3BGRL2	NA	Wald ratio	1	0.011	0.046	8.178E-01	0.995	pan	SARS-CoV-2 infection
19383_131	CINP	Cyclin-dependent kinase 2-interacting protein	Q9BW66	CINP	NA	Wald ratio	1	0.021	0.091	8.193E-01	0.995	pan	SARS-CoV-2 infection
11390_24	Carbonic Anhydrase VIII	Carbonic anhydrase-related protein	P35219	CA8	NA	Inverse variance weighted	7	0.003	0.011	8.194E-01	0.995	pan	SARS-CoV-2 infection
6507_16	NCAM2	Neural cell adhesion molecule 2	O15394	NCAM2	NA	Inverse variance weighted	7	0.003	0.012	8.200E-01	0.995	pan	SARS-CoV-2 infection
7099_33	FSTL5	Follistatin-related protein 5	Q8N475	FSTL5	NA	Wald ratio	1	-0.013	0.055	8.200E-01	0.995	pan	SARS-CoV-2 infection
15326_64	GBP1	Guanylate-binding protein 1	P32455	GBP1	NA	Inverse variance weighted	10	-0.005	0.020	8.206E-01	0.995	pan	SARS-CoV-2 infection
15336_7	SELM	Selenoprotein M	Q8WWX9	SELENOM	NA	Wald ratio	1	-0.020	0.087	8.207E-01	0.995	pan	SARS-CoV-2 infection
2789_26	MMP-7	Matrilysin	P09237	MMP7	NA	Inverse variance weighted	7	-0.006	0.027	8.208E-01	0.995	pan	SARS-CoV-2 infection
3825_18	MK08	Mitogen-activated protein kinase 8	P45983	MAPK8	NA	Inverse variance weighted	3	0.008	0.037	8.212E-01	0.995	pan	SARS-CoV-2 infection
6597_24	TM157	Membrane protein FAM174A	Q8TBP5	FAM174A	NA	Inverse variance weighted	8	0.003	0.014	8.213E-01	0.995	pan	SARS-CoV-2 infection
3845_51	DLRB1	Dynein light chain roadblock-type 1	Q9NP97	DYNLRB1	NA	Inverse variance weighted	8	0.005	0.021	8.214E-01	0.995	pan	SARS-CoV-2 infection
11543_84	LIMA1	LIM domain and actin-binding protein 1	Q9UHB6	LIMA1	NA	Wald ratio	1	-0.017	0.077	8.217E-01	0.995	pan	SARS-CoV-2 infection
16015_19	ALT	Alanine aminotransferase 1	P24298	GPT	NA	Wald ratio	1	0.021	0.092	8.224E-01	0.995	pan	SARS-CoV-2 infection
2617_56	ERBB3	Receptor tyrosine-protein kinase erbB-3	P21860	ERBB3	NA	Inverse variance weighted	2	0.016	0.070	8.228E-01	0.995	pan	SARS-CoV-2 infection
12411_60	MAX	Protein max	P61244	MAX	NA	Wald ratio	1	-0.006	0.027	8.229E-01	0.995	pan	SARS-CoV-2 infection
17672_184	Gastric intrinsic factor	Gastric intrinsic factor	P27352	CBLIF	NA	Inverse variance weighted	13	0.003	0.013	8.233E-01	0.995	pan	SARS-CoV-2 infection
6375_75	XXLT1	Xyloside xylosyltransferase 1	Q8NBI6	XXYLT1	NA	Inverse variance weighted	2	-0.005	0.022	8.241E-01	0.995	pan	SARS-CoV-2 infection
15381_45	Discoidin domain receptor 2	Discoidin domain-containing receptor 2	Q16832	DDR2	NA	Wald ratio	1	0.020	0.092	8.242E-01	0.995	pan	SARS-CoV-2 infection
8252_2	NOTUM	Palmitoleoyl-protein carboxylesterase NOTUM	Q6P988	NOTUM	NA	Inverse variance weighted	4	0.011	0.052	8.256E-01	0.995	pan	SARS-CoV-2 infection
3461_58	PGCB	Brevican core protein	Q96GW7	BCAN	NA	Inverse variance weighted	3	0.013	0.060	8.269E-01	0.995	pan	SARS-CoV-2 infection
15466_30	CO9A1	Collagen alpha-1(IX) chain	P20849	COL9A1	NA	Inverse variance weighted	3	0.010	0.044	8.276E-01	0.995	pan	SARS-CoV-2 infection

12633_3	PTN9	Tyrosine-protein phosphatase non-receptor type 9	P43378	PTPN9	NA	Wald ratio	1	0.019	0.086	8.276E-01	0.995	pan	SARS-CoV-2 infection
14623_26	SUMO3	Small ubiquitin-related modifier 3	P55854	SUMO3	NA	Wald ratio	1	-0.019	0.086	8.282E-01	0.995	pan	SARS-CoV-2 infection
19639_53	IAPP	Islet amyloid polypeptide	P10997	IAPP	NA	Inverse variance weighted	9	0.006	0.028	8.284E-01	0.995	pan	SARS-CoV-2 infection
18930_28	SLIT2	Slit homolog 2 protein	O94813	SLIT2	NA	Inverse variance weighted	2	-0.013	0.062	8.287E-01	0.995	pan	SARS-CoV-2 infection
11152_46	kallikrein 13	Kallikrein-13	Q9UKR3	KLK13	NA	Inverse variance weighted	4	0.008	0.039	8.292E-01	0.995	pan	SARS-CoV-2 infection
4131_72	Fibronectin	Fibronectin	P02751	FN1	NA	Inverse variance weighted	2	0.024	0.114	8.308E-01	0.995	pan	SARS-CoV-2 infection
4192_10	AK1A1	Alcohol dehydrogenase [NADP(+)]	P14550	AKR1A1	NA	Inverse variance weighted	10	-0.002	0.010	8.315E-01	0.995	pan	SARS-CoV-2 infection
17345_12	ZADH2	Prostaglandin reductase 3	Q8N4Q0	PTGR3	NA	Wald ratio	1	-0.010	0.049	8.319E-01	0.995	pan	SARS-CoV-2 infection
7827_20	ADA32	Disintegrin and metalloproteinase domain-containing protein 32	Q8TC27	ADAM32	NA	Wald ratio	1	0.025	0.116	8.322E-01	0.995	pan	SARS-CoV-2 infection
14204_55	FOXJ2	Forkhead box protein J2	Q9P0K8	FOXJ2	NA	Inverse variance weighted	3	-0.014	0.065	8.327E-01	0.995	pan	SARS-CoV-2 infection
6081_52	PCOC2	Procollagen C-endopeptidase enhancer 2	Q9UKZ9	PCOLCE2	NA	Inverse variance weighted	15	-0.002	0.009	8.328E-01	0.995	pan	SARS-CoV-2 infection
9339_204	FKBP2	Peptidyl-prolyl cis-trans isomerase FKBP2	P26885	FKBP2	NA	Wald ratio	1	0.022	0.104	8.339E-01	0.995	pan	SARS-CoV-2 infection
13397_88	HBD-2	Beta-defensin 4A	O15263	DEFB4A	NA	Inverse variance weighted	4	0.007	0.034	8.349E-01	0.995	pan	SARS-CoV-2 infection
17410_5	CETN3	Centrin-3	O15182	CETN3	NA	Inverse variance weighted	3	-0.009	0.043	8.351E-01	0.995	pan	SARS-CoV-2 infection
17856_23	NT5M	5'(3')-deoxyribonucleotidase, mitochondrial	Q9NPB1	NT5M	NA	Inverse variance weighted	2	0.016	0.077	8.352E-01	0.995	pan	SARS-CoV-2 infection
17396_23	ADH1A	Alcohol dehydrogenase 1A	P07327	ADH1A	NA	Inverse variance weighted	3	0.010	0.047	8.354E-01	0.995	pan	SARS-CoV-2 infection
17820_170	MLF1	Myeloid leukemia factor 1	P58340	MLF1	NA	Inverse variance weighted	4	-0.010	0.049	8.355E-01	0.995	pan	SARS-CoV-2 infection
6382_17	MANBA	Beta-mannosidase	O00462	MANBA	NA	Inverse variance weighted	8	-0.004	0.021	8.356E-01	0.995	pan	SARS-CoV-2 infection
7140_1	ELA2A	Chymotrypsin-like elastase family member 2A	P08217	CELA2A	NA	Inverse variance weighted	4	0.012	0.058	8.362E-01	0.995	pan	SARS-CoV-2 infection
4128_27	Eotaxin-2	C-C motif chemokine 24	O00175	CCL24	NA	Wald ratio	1	-0.022	0.107	8.366E-01	0.995	pan	SARS-CoV-2 infection
2665_26	BCMA	Tumor necrosis factor receptor superfamily member 17	Q02223	TNFRSF17	NA	Inverse variance weighted	11	0.004	0.022	8.371E-01	0.995	pan	SARS-CoV-2 infection
3820_68	MAPK2	MAP kinase-activated protein kinase 2	P49137	MAPKAPK2	NA	Wald ratio	1	-0.009	0.044	8.377E-01	0.995	pan	SARS-CoV-2 infection
18225_13	HEBP1	Heme-binding protein 1	Q9NRV9	HEBP1	NA	Inverse variance weighted	10	-0.002	0.009	8.377E-01	0.995	pan	SARS-CoV-2 infection
8908_14	KCE1L:CD	Potassium voltage-gated channel subfamily E regulatory beta subunit 5:Cytoplasmic domain	Q9UJ90	KCNE5	NA	Inverse variance weighted	3	-0.011	0.056	8.379E-01	0.995	pan	SARS-CoV-2 infection
10008_43	GUC1A	Guanylyl cyclase-activating protein 1	P43080	GUCA1A	NA	Inverse variance weighted	2	0.019	0.094	8.381E-01	0.995	pan	SARS-CoV-2 infection
17813_21	BPHL	Valacyclovir hydrolase	Q86WA6	BPHL	NA	Inverse variance weighted	2	0.013	0.062	8.384E-01	0.995	pan	SARS-CoV-2 infection
11145_72	K154L	UPF0606 protein KIAA1549L	Q6ZVL6	KIAA1549L	NA	Inverse variance weighted	7	0.004	0.018	8.386E-01	0.995	pan	SARS-CoV-2 infection
2585_2	PRL	Prolactin	P01236	PRL	NA	Wald ratio	1	-0.015	0.073	8.392E-01	0.995	pan	SARS-CoV-2 infection
18840_205	SH21B	SH2 domain-containing protein 1B	O14796	SH2D1B	NA	Wald ratio	1	-0.023	0.112	8.402E-01	0.995	pan	SARS-CoV-2 infection
3894_15	NAGK	N-acetyl-D-glucosamine kinase	Q9UJ70	NAGK	NA	Inverse variance weighted	6	-0.007	0.034	8.406E-01	0.995	pan	SARS-CoV-2 infection
16916_19	SLIK6	SLIT and NTRK-like protein 6	Q9H5Y7	SLITRK6	NA	Inverse variance weighted	6	-0.004	0.022	8.406E-01	0.995	pan	SARS-CoV-2 infection
5029_3	SEPR	Prolyl endopeptidase FAP	Q12884	FAP	NA	Inverse variance weighted	6	0.004	0.021	8.407E-01	0.995	pan	SARS-CoV-2 infection
16292_288	GIP	Gastric inhibitory polypeptide	P09681	GIP	NA	Wald ratio	1	-0.020	0.102	8.409E-01	0.995	pan	SARS-CoV-2 infection
9747_48	MARK3	MAP/microtubule affinity-regulating kinase 3	P27448	MARK3	NA	Wald ratio	1	-0.023	0.116	8.412E-01	0.995	pan	SARS-CoV-2 infection
18175_65	VAT1	Synaptic vesicle membrane protein VAT-1 homolog	Q99536	VAT1	NA	Inverse variance weighted	2	-0.013	0.065	8.413E-01	0.995	pan	SARS-CoV-2 infection
5688_65	CBLN4	Cerebellin-4	Q9NTU7	CBLN4	NA	Inverse variance weighted	6	-0.004	0.020	8.433E-01	0.995	pan	SARS-CoV-2 infection
19496_1	NPL	N-acetylneuraminate lyase	Q9BXD5	NPL	NA	Inverse variance weighted	4	0.009	0.045	8.437E-01	0.995	pan	SARS-CoV-2 infection
3052_8	Fas ligand, soluble	Tumor necrosis factor ligand superfamily member 6, soluble form	P48023	FASLG	NA	Inverse variance weighted	4	0.010	0.052	8.438E-01	0.995	pan	SARS-CoV-2 infection
6247_9	SIRB1	Signal-regulatory protein beta-1	O00241	SIRPB1	NA	Inverse variance weighted	9	-0.001	0.005	8.442E-01	0.995	pan	SARS-CoV-2 infection
5483_1	RGMA	Repulsive guidance molecule A	Q96B86	RGMA	NA	Inverse variance weighted	8	0.006	0.030	8.454E-01	0.995	pan	SARS-CoV-2 infection
9297_12	B3GN8	UDP-GlcNAc:betaGal beta-1,3-N-acetylglucosaminyltransferase 8	Q7Z7M8	B3GNT8	NA	Inverse variance weighted	13	0.004	0.018	8.455E-01	0.995	pan	SARS-CoV-2 infection
3079_62	TIG2	Retinoic acid receptor responder protein 2	Q99969	RARRES2	NA	Inverse variance weighted	2	-0.005	0.026	8.460E-01	0.995	pan	SARS-CoV-2 infection
17138_8	GST A1-1	Glutathione S-transferase A1	P08263	GSTA1	NA	Inverse variance weighted	4	0.007	0.037	8.461E-01	0.995	pan	SARS-CoV-2 infection
4152_58	Prekallikrein	Plasma kallikrein	P03952	KLKB1	NA	Inverse variance weighted	4	0.010	0.050	8.463E-01	0.995	pan	SARS-CoV-2 infection
10047_12	NCF-2	Neutrophil cytosol factor 2	P19878	NCF2	NA	Inverse variance weighted	4	-0.008	0.044	8.463E-01	0.995	pan	SARS-CoV-2 infection
14146_92	H31	Histone H3.1	P68431	H3C1	NA	Wald ratio	1	-0.016	0.082	8.471E-01	0.995	pan	SARS-CoV-2 infection
9039_47	TOIP1:Nuclear	Torsin-1A-interacting protein 1:Nuclear domain	Q5JTV8	TOR1AIP1	NA	Inverse variance weighted	2	-0.013	0.069	8.472E-01	0.995	pan	SARS-CoV-2 infection
5481_16	RASA1	Ras GTPase-activating protein 1	P20936	RASA1	NA	Inverse variance weighted	2	-0.014	0.073	8.480E-01	0.995	pan	SARS-CoV-2 infection
19590_46	SP-D	Pulmonary surfactant-associated protein D	P35247	SFTPD	NA	Inverse variance weighted	21	0.003	0.014	8.480E-01	0.995	pan	SARS-CoV-2 infection
15299_102	MESD2	LDLR chaperone MESD	Q14696	MESD	NA	Wald ratio	1	0.020	0.103	8.491E-01	0.995	pan	SARS-CoV-2 infection
16825_20	ATX3	Ataxin-3	P54252	ATXN3	NA	Inverse variance weighted	4	0.004	0.019	8.496E-01	0.995	pan	SARS-CoV-2 infection
6060_2	PIP	Prolactin-inducible protein	P12273	PIP	NA	Inverse variance weighted	8	0.006	0.030	8.498E-01	0.995	pan	SARS-CoV-2 infection
10907_116	NTRI	Neurotrimin	Q9P121	NTM	NA	Inverse variance weighted	3	-0.009	0.049	8.499E-01	0.995	pan	SARS-CoV-2 infection
7208_60	MGT4C	Alpha-1,3-mannosyl-glycoprotein 4-beta-N-acetylglucosaminyltransferase C	Q9UBM8	MGAT4C	NA	Wald ratio	1	0.015	0.082	8.516E-01	0.995	pan	SARS-CoV-2 infection
10974_20	ISK7	Serine protease inhibitor Kazal-type 7	P58062	SPINK7	NA	Inverse variance weighted	5	0.010	0.055	8.548E-01	0.995	pan	SARS-CoV-2 infection
17792_158	SSDH	Succinate-semialdehyde dehydrogenase, mitochondrial	P51649	ALDH5A1	NA	Inverse variance weighted	18	-0.003	0.018	8.550E-01	0.995	pan	SARS-CoV-2 infection
12517_52	TFAR19	Programmed cell death protein 5	O14737	PDCD5	NA	Inverse variance weighted	5	0.004	0.022	8.558E-01	0.995	pan	SARS-CoV-2 infection
8353_15	SCN2B	Sodium channel subunit beta-2	O60939	SCN2B	NA	Inverse variance weighted	6	-0.007	0.039	8.559E-01	0.995	pan	SARS-CoV-2 infection
10419_1	SCAR5	Scavenger receptor class A member 5	Q6ZMJ2	SCARA5	NA	Inverse variance weighted	10	-0.005	0.030	8.561E-01	0.995	pan	SARS-CoV-2 infection
4706_17	41	Protein 4.1	P11171	EPB41	NA	Wald ratio	1	0.017	0.094	8.565E-01	0.995	pan	SARS-CoV-2 infection
13982_33	RGS18	Regulator of G-protein signaling 18	Q9NS28	RGS18	NA	Wald ratio	1	-0.015	0.083	8.578E-01	0.995	pan	SARS-CoV-2 infection
9829_91	SULT 2A1	Bile salt sulfotransferase	Q06520	SULT2A1	NA	Wald ratio	1	-0.009	0.053	8.581E-01	0.995	pan	SARS-CoV-2 infection
8900_28	NEO1	Neogenin	Q92859	NEO1	NA	Inverse variance weighted	5	0.004	0.021	8.591E-01	0.995	pan	SARS-CoV-2 infection
12682_5	KAT3	Kynurenine--oxoglutarate transaminase 3	Q6YP21	KYAT3	NA	Inverse variance weighted	7	-0.003	0.016	8.593E-01	0.995	pan	SARS-CoV-2 infection
13088_397	BTC	Betacellulin	P35070	BTC	NA	Inverse variance weighted	2	-0.007	0.037	8.596E-01	0.995	pan	SARS-CoV-2 infection
12436_84	GST omega-1	Glutathione S-transferase omega-1	P78417	GSTO1	NA	Inverse variance weighted	16	-0.002	0.011	8.598E-01	0.995	pan	SARS-CoV-2 infection
17773_26	SNAG	Gamma-soluble NSF attachment protein	Q99747	NAPG	NA	Wald ratio	1	-0.012	0.066	8.601E-01	0.995	pan	SARS-CoV-2 infection
19361_78	MATN3	Matrilin-3	O15232	MATN3	NA	Inverse variance weighted	14	0.002	0.009	8.603E-01	0.995	pan	SARS-CoV-2 infection
8476_11	CgA	Chromogranin-A	P10645	CHGA	NA	Inverse variance weighted	6	-0.006	0.033	8.614E-01	0.995	pan	SARS-CoV-2 infection
9451_20	Uromodulin	Uromodulin	P07911	UMOD	NA	Wald ratio	1	0.022	0.126	8.623E-01	0.995	pan	SARS-CoV-2 infection
12714_38	AP1G2	AP-1 complex subunit gamma-like 2	O75843	AP1G2	NA	Inverse variance weighted	3	0.005	0.028	8.634E-01	0.995	pan	SARS-CoV-2 infection
9565_6	PAWR	PRKC apoptosis WT1 regulator protein	Q96IZ0	PAWR	NA	Wald ratio	1	-0.018	0.104	8.649E-01	0.995	pan	SARS-CoV-2 infection
14090_23	DEFI6	Differentially expressed in FDCP 6 homolog	Q9H4E7	DEF6	NA	Inverse variance weighted	2	0.013	0.078	8.649E-01	0.995	pan	SARS-CoV-2 infection
9719_145	MMP-16	Matrix metalloproteinase-16	P51512	MMP16	NA	Inverse variance weighted	2	0.012	0.073	8.651E-01	0.995	pan	SARS-CoV-2 infection
8687_26	T106B	Transmembrane protein 106B	Q9NUM4	TMEM106B	NA	Inverse variance weighted	3	0.009	0.055	8.652E-01	0.995	pan	SARS-CoV-2 infection
9021_1	TIM-1	Hepatitis A virus cellular receptor 1	Q96D42	HAVCR1	NA	Inverse variance weighted	7	0.003	0.016	8.652E-01	0.995	pan	SARS-CoV-2 infection
10620_21	PSP-94	Beta-microseminoprotein	P08118	MSMB	NA	Inverse variance weighted	19	0.001	0.007	8.653E-01	0.995	pan	SARS-CoV-2 infection
8080_24	PSMP	Prostate-associated microseminoprotein	Q1L6U9	MSMP	NA	Inverse variance weighted	15	0.003	0.018	8.655E-01	0.995	pan	SARS-CoV-2 infection

8035_6	CA198	Uncharacterized protein C1orf198	Q9H425	C1orf198	NA	Wald ratio	1	0.007	0.043	8.658E-01	0.995	pan	SARS-CoV-2 infection
12357_41	SNP29	Synaptosomal-associated protein 29	O95721	SNAP29	NA	Wald ratio	1	0.012	0.073	8.658E-01	0.995	pan	SARS-CoV-2 infection
17746_77	FIS1	Mitochondrial fission 1 protein	Q9Y3D6	FIS1	NA	Inverse variance weighted	2	-0.011	0.068	8.659E-01	0.995	pan	SARS-CoV-2 infection
8368_102	TNF sR-II	Tumor necrosis factor receptor superfamily member 1B	P20333	TNFRSF1B	NA	Wald ratio	1	0.005	0.031	8.659E-01	0.995	pan	SARS-CoV-2 infection
6414_8	OAF	Out at first protein homolog	Q86UD1	OAF	NA	Inverse variance weighted	6	0.003	0.019	8.673E-01	0.995	pan	SARS-CoV-2 infection
5030_52	SIRT2	NAD-dependent protein deacetylase sirtuin-2	Q8IXJ6	SIRT2	NA	Inverse variance weighted	6	0.012	0.071	8.675E-01	0.995	pan	SARS-CoV-2 infection
10833_64	HHIP	Hedgehog-interacting protein	Q96QV1	HHIP	NA	Inverse variance weighted	3	-0.005	0.029	8.675E-01	0.995	pan	SARS-CoV-2 infection
4158_54	uPA	Urokinase-type plasminogen activator	P00749	PLAU	NA	Inverse variance weighted	11	-0.004	0.022	8.680E-01	0.995	pan	SARS-CoV-2 infection
9971_5	CSMD2	CUB and sushi domain-containing protein 2	Q7Z408	CSMD2	NA	Wald ratio	1	0.016	0.095	8.686E-01	0.995	pan	SARS-CoV-2 infection
15533_97	Macrophage scavenger receptor:ECD	Macrophage scavenger receptor types I and II:Extracellular domain	P21757	MSR1	NA	Inverse variance weighted	7	-0.004	0.025	8.687E-01	0.995	pan	SARS-CoV-2 infection
19561_216	PLXD1	Plexin-D1	Q9Y4D7	PLXND1	NA	Inverse variance weighted	6	-0.003	0.020	8.688E-01	0.995	pan	SARS-CoV-2 infection
2381_52	C5	Complement C5	P01031	C5	NA	Inverse variance weighted	2	-0.008	0.052	8.697E-01	0.995	pan	SARS-CoV-2 infection
9928_125	LECT1	Leukocyte cell-derived chemotaxin 1	O75829	CNMD	NA	Wald ratio	1	-0.014	0.085	8.700E-01	0.995	pan	SARS-CoV-2 infection
15481_45	LL-37	Antibacterial protein LL-37	P49913	CAMP	NA	Inverse variance weighted	10	-0.005	0.030	8.703E-01	0.995	pan	SARS-CoV-2 infection
8345_27	GPX7	Glutathione peroxidase 7	Q96SL4	GPX7	NA	Inverse variance weighted	8	0.002	0.011	8.713E-01	0.995	pan	SARS-CoV-2 infection
13697_51	GPDA	Glycerol-3-phosphate dehydrogenase [NAD(+)], cytoplasmic	P21695	GPD1	NA	Wald ratio	1	-0.020	0.125	8.715E-01	0.995	pan	SARS-CoV-2 infection
13553_4	DCNL3	DCN1-like protein 3	Q8IWE4	DCUN1D3	NA	Wald ratio	1	0.018	0.111	8.716E-01	0.995	pan	SARS-CoV-2 infection
6223_5	GUC2B	Guanylate cyclase activator 2B	Q16661	GUCA2B	NA	Inverse variance weighted	5	0.005	0.034	8.727E-01	0.995	pan	SARS-CoV-2 infection
4151_6	Plasminogen	Plasminogen	P00747	PLG	NA	Inverse variance weighted	5	0.008	0.048	8.729E-01	0.995	pan	SARS-CoV-2 infection
5682_13	VASN	Vasorin	Q6EMK4	VASN	NA	Inverse variance weighted	2	-0.009	0.054	8.734E-01	0.995	pan	SARS-CoV-2 infection
3327_27	NET4	Netrin-4	Q9HB63	NTN4	NA	Inverse variance weighted	5	0.004	0.023	8.748E-01	0.995	pan	SARS-CoV-2 infection
11215_6	CAD15:CD	Cadherin-15:Cytoplasmic domain	P55291	CDH15	NA	Inverse variance weighted	2	0.021	0.137	8.754E-01	0.995	pan	SARS-CoV-2 infection
17161_1	OST48	Dolichyl-diphosphooligosaccharide--protein glycosyltransferase 48 kDa subunit	P39656	DDOST	NA	Inverse variance weighted	3	-0.002	0.015	8.762E-01	0.995	pan	SARS-CoV-2 infection
5475_10	PKC-B-II	Protein kinase C beta type (splice variant beta-II)	P05771	PRKCB	NA	Wald ratio	1	0.010	0.065	8.763E-01	0.995	pan	SARS-CoV-2 infection
2888_49	C7	Complement component C7	P10643	C7	NA	Inverse variance weighted	14	-0.001	0.008	8.766E-01	0.995	pan	SARS-CoV-2 infection
12378_71	TPSN	Tapasin	O15533	TAPBP	NA	Inverse variance weighted	7	0.002	0.012	8.776E-01	0.995	pan	SARS-CoV-2 infection
9212_22	CATF	Cathepsin F	Q9UBX1	CTSF	NA	Inverse variance weighted	3	0.012	0.077	8.782E-01	0.995	pan	SARS-CoV-2 infection
4771_10	ASM3A	Acid sphingomyelinase-like phosphodiesterase 3a	Q92484	SMPDL3A	NA	Inverse variance weighted	3	-0.003	0.022	8.786E-01	0.995	pan	SARS-CoV-2 infection
9211_19	PEDF	Pigment epithelium-derived factor	P36955	SERPINF1	NA	Inverse variance weighted	6	0.003	0.018	8.794E-01	0.995	pan	SARS-CoV-2 infection
14079_14	IL-18 Ra	Interleukin-18 receptor 1	Q13478	IL18R1	NA	Inverse variance weighted	10	-0.003	0.020	8.795E-01	0.995	pan	SARS-CoV-2 infection
9231_23	IMPA3	Inositol monophosphatase 3	Q9NX62	BPNT2	NA	Inverse variance weighted	12	0.002	0.012	8.798E-01	0.995	pan	SARS-CoV-2 infection
16079_2	TEC	Tyrosine-protein kinase Tec	P42680	TEC	NA	Inverse variance weighted	2	-0.009	0.060	8.800E-01	0.995	pan	SARS-CoV-2 infection
15540_6	Vimentin	Vimentin	P08670	VIM	NA	Inverse variance weighted	2	0.009	0.061	8.804E-01	0.995	pan	SARS-CoV-2 infection
8088_56	CA210	Type III endosome membrane protein TEMP	Q8IVY1	C1orf210	NA	Wald ratio	1	0.017	0.111	8.805E-01	0.995	pan	SARS-CoV-2 infection
17752_24	Arginase	Arginase-2, mitochondrial	P78540	ARG2	NA	Inverse variance weighted	2	-0.009	0.058	8.810E-01	0.995	pan	SARS-CoV-2 infection
9769_48	DNER:ECD	Delta and Notch-like epidermal growth factor-related receptor:Extracellular domain	Q8NFT8	DNER	NA	Inverse variance weighted	5	-0.003	0.019	8.813E-01	0.995	pan	SARS-CoV-2 infection
8258_22	UXS1	UDP-glucuronic acid decarboxylase 1	Q8NBZ7	UXS1	NA	Inverse variance weighted	2	-0.010	0.065	8.822E-01	0.995	pan	SARS-CoV-2 infection
13374_4	DB113	Beta-defensin 113	Q30KQ7	DEFB113	NA	Wald ratio	1	-0.015	0.104	8.824E-01	0.995	pan	SARS-CoV-2 infection
8040_9	LEMD1	LEM domain-containing protein 1	Q68G75	LEMD1	NA	Wald ratio	1	-0.011	0.075	8.834E-01	0.995	pan	SARS-CoV-2 infection
4220_39	FER	Tyrosine-protein kinase Fer	P16591	FER	NA	Inverse variance weighted	2	0.010	0.065	8.837E-01	0.995	pan	SARS-CoV-2 infection
8374_5	NGF R	Tumor necrosis factor receptor superfamily member 16	P08138	NGFR	NA	Inverse variance weighted	6	0.005	0.036	8.856E-01	0.995	pan	SARS-CoV-2 infection
3516_60	SDF-1	Stromal cell-derived factor 1	P48061	CXCL12	NA	Inverse variance weighted	2	0.005	0.033	8.856E-01	0.995	pan	SARS-CoV-2 infection
3449_58	Kallistatin	Kallistatin	P29622	SERPINA4	NA	Inverse variance weighted	8	0.005	0.032	8.864E-01	0.995	pan	SARS-CoV-2 infection
6407_63	LAD1	Ladinin-1	O00515	LAD1	NA	Wald ratio	1	-0.022	0.155	8.877E-01	0.995	pan	SARS-CoV-2 infection
11872_9	PCDGD	Protocadherin gamma-B1	Q9Y5G3	PCDHGB1	NA	Inverse variance weighted	2	0.012	0.085	8.884E-01	0.995	pan	SARS-CoV-2 infection
5646_20	RNAS6	Ribonuclease K6	Q93091	RNASE6	NA	Inverse variance weighted	6	0.001	0.009	8.886E-01	0.995	pan	SARS-CoV-2 infection
15582_25	FCN1	Ficolin-1	O00602	FCN1	NA	Inverse variance weighted	4	-0.005	0.035	8.895E-01	0.995	pan	SARS-CoV-2 infection
3855_56	Peroxiredoxin-1	Peroxiredoxin-1	Q06830	PRDX1	NA	Inverse variance weighted	7	-0.005	0.035	8.897E-01	0.995	pan	SARS-CoV-2 infection
13460_4	CHAD	Chondroadherin	O15335	CHAD	NA	Inverse variance weighted	2	-0.013	0.092	8.904E-01	0.995	pan	SARS-CoV-2 infection
13992_12	NSF	Vesicle-fusing ATPase	P46459	NSF	NA	Wald ratio	1	-0.008	0.059	8.908E-01	0.995	pan	SARS-CoV-2 infection
7957_2	SCG3	Secretogranin-3	Q8WXD2	SCG3	NA	Inverse variance weighted	10	-0.002	0.012	8.917E-01	0.995	pan	SARS-CoV-2 infection
7808_5	GLCE	D-glucuronyl C5-epimerase	O94923	GLCE	NA	Inverse variance weighted	9	0.002	0.011	8.919E-01	0.995	pan	SARS-CoV-2 infection
3364_76	Cathepsin V	Cathepsin L2	O60911	CTSV	NA	Inverse variance weighted	12	0.003	0.021	8.929E-01	0.995	pan	SARS-CoV-2 infection
7648_9	MYPC1	Myosin-binding protein C, slow-type	Q00872	MYBPC1	NA	Inverse variance weighted	2	-0.010	0.073	8.930E-01	0.995	pan	SARS-CoV-2 infection
9197_4	LEG9	Galectin-9	O00182	LGALS9	NA	Wald ratio	1	-0.006	0.044	8.930E-01	0.995	pan	SARS-CoV-2 infection
7875_86	PLEK	Pleckstrin	P08567	PLEK	NA	Inverse variance weighted	2	-0.008	0.057	8.937E-01	0.995	pan	SARS-CoV-2 infection
3175_51	ATS13	A disintegrin and metalloproteinase with thrombospondin motifs 13	Q76LX8	ADAMTS13	NA	Inverse variance weighted	13	0.002	0.013	8.940E-01	0.995	pan	SARS-CoV-2 infection
17454_15	EGFL6	Epidermal growth factor-like protein 6	Q8IUX8	EGFL6	NA	Inverse variance weighted	5	-0.005	0.041	8.942E-01	0.995	pan	SARS-CoV-2 infection
8660_5	OLFL3	Olfactomedin-like protein 3	Q9NRN5	OLFML3	NA	Inverse variance weighted	5	0.005	0.037	8.951E-01	0.995	pan	SARS-CoV-2 infection
10615_18	MYP0	Myelin protein P0	P25189	MPZ	NA	Inverse variance weighted	14	0.002	0.018	8.956E-01	0.995	pan	SARS-CoV-2 infection
5689_1	DB112	Beta-defensin 112	Q30KQ8	DEFB112	NA	Wald ratio	1	0.009	0.066	8.966E-01	0.995	pan	SARS-CoV-2 infection
14082_56	TLN2	Talin-2	Q9Y4G6	TLN2	NA	Wald ratio	1	0.013	0.100	8.971E-01	0.995	pan	SARS-CoV-2 infection
19367_34	D3D2	Enoyl-CoA delta isomerase 1, mitochondrial	P42126	ECI1	NA	Inverse variance weighted	2	0.008	0.059	8.972E-01	0.995	pan	SARS-CoV-2 infection
9849_13	APEX1	DNA-(apurinic or apyrimidinic site) lyase	P27695	APEX1	NA	Wald ratio	1	0.014	0.107	8.973E-01	0.995	pan	SARS-CoV-2 infection
3420_21	Carbonic anhydrase XIII	Carbonic anhydrase 13	Q8N1Q1	CA13	NA	Inverse variance weighted	5	-0.002	0.018	8.993E-01	0.995	pan	SARS-CoV-2 infection
14006_36	GNMT	Glycine N-methyltransferase	Q14749	GNMT	NA	Inverse variance weighted	3	-0.005	0.038	8.995E-01	0.995	pan	SARS-CoV-2 infection
9995_6	DUT	Deoxyuridine 5'-triphosphate nucleotidohydrolase, mitochondrial	P33316	DUT	NA	Wald ratio	1	0.010	0.080	8.997E-01	0.995	pan	SARS-CoV-2 infection
[truncated: 66,813 more chars]
